# Predicting Chemical Toxicity Effects Based on Chemical-Chemical Interactions

**DOI:** 10.1371/journal.pone.0056517

**Published:** 2013-02-15

**Authors:** Lei Chen, Jing Lu, Jian Zhang, Kai-Rui Feng, Ming-Yue Zheng, Yu-Dong Cai

**Affiliations:** 1 Institute of Systems Biology, Shanghai University, Shanghai, China; 2 College of Information Engineering, Shanghai Maritime University, Shanghai, China; 3 Drug Discovery and Design Center (DDDC), Shanghai Institute of Materia Medica, Shanghai, China; 4 Department of Ophthalmology, Shanghai First People’s Hospital Affiliated to Shanghai Jiaotong University, Shanghai, China; 5 Simcyp Limited, Blades Enterprise Centre, Sheffield, United Kingdom; CSIR-Institute of Microbial Technology, India

## Abstract

Toxicity is a major contributor to high attrition rates of new chemical entities in drug discoveries. In this study, an order-classifier was built to predict a series of toxic effects based on data concerning chemical-chemical interactions under the assumption that interactive compounds are more likely to share similar toxicity profiles. According to their interaction confidence scores, the order from the most likely toxicity to the least was obtained for each compound. Ten test groups, each of them containing one training dataset and one test dataset, were constructed from a benchmark dataset consisting of 17,233 compounds. By a Jackknife test on each of these test groups, the 1^st^ order prediction accuracies of the training dataset and the test dataset were all approximately 79.50%, substantially higher than the rate of 25.43% achieved by random guesses. Encouraged by the promising results, we expect that our method will become a useful tool in screening out drugs with high toxicity.

## Introduction

Toxicity is a key cause of late-stage failures in drug discovery. Even some approved drugs such as Phenacetin [1] and Troglitazone [2] have been withdrawn from the market because of unexpected toxicities that were not detected during Phase III clinical trials. Thus, early toxicology data on compounds are needed to reduce R&D costs. Evaluating toxicity and assessing risks of diverse chemicals require comprehensive experimental testing against a broad spectrum of toxicity end points. These tests can cost millions of dollars, involving several thousand animals, and take many years to complete. As a result, very few chemicals have undergone the degree of testing needed to support accurate health risk assessments or meet regulatory requirements for drug approval. In recent years, the number of synthetic compounds has surged with the advance of combinatorial chemistry, and accordingly large quantities of toxicity data are urgently demanded.

Recently, particular interest has been raised to apply fast and cost-effective *in silico* toxicological models to supplement those *in vitro* and *in vivo* testing. These models require high quality toxicity data for a large set of structurally diverse drug candidates. Accelrys Toxicity is a database of toxicity information compiled from the open scientific literature [3] and containing toxicological data for approximately 0.17 million chemicals. This database is of great value for investigating the pharmacokinetic properties, metabolism and potential toxicities of compounds. Six types of toxicity data are collected in the database: (1) Acute Toxicity; (2) Mutagenicity; (3) Tumorigenicity; (4) Skin and Eye Irritation; (5) Reproductive Effects; and (6) Multiple Dose Effects. It should be noted that these categories have multiple and overlapping mechanisms of toxic action and each category represents only specific types of experiments. The combination of these experimental results may help define the overall safety profile of a compound. However, this kind of databases only provides toxicological information for recorded compounds, not for new ones. It would be valuable to accurately predict toxicities of a new compound based on the information available for recorded compounds. In order to meet the demand, there is a drive to develop quick, reliable, and non-animal-involved prediction methods, *e.g.* using structure-activity relationships (SARs) to predict drugs toxicities.

Currently, most toxicological SAR models belong to binary classifiers, which only predict compounds to be toxic or non-toxic within a single toxicity class [4], [5]. It is desired to modify the strategy to predict a series of toxicity effects. In this study, we chose to build a multiclass model [6], [7] to predict six categories of toxicity using the Accelrys Toxicity database instead of only one or two toxicity endpoints. However, the quadratic optimization problem in multiclass models is difficult to solve. Thus, many previous multiclass approaches tended to decompose a multiclass problem into multiple independent binary classifications. Investigators built a set of binary classifiers, such as the model of Dietterich et al [7], each classifier distinguishing only one of the classes from the others. Although this greatly simplifies the problem, such an approach cannot provide order prediction information for the query compounds. That is, it can only predict whether the query compound has some toxicity end points, but cannot determine which is the most likely toxicity, or even the order of toxicity end points by toxicity likelihoods.

In recent years, the assessment of protein-protein interactions has been widely used to predict many attributes of proteins [8], [9], [10], [11]. Furthermore, multiclass predictions of protein attributes have become more common [12], [13], [14]. These methods and their results show that interactive proteins tend to share the same functions with higher probability than do non-interactive ones. Likewise, it is reasonable to expect that interactive compounds are also more likely to share common functions as indicated by some pioneer studies [15], [16]. Thus, toxicity, as part of the biological functions of compounds, should follow the same rule. Moreover, based on a previous work on the Anatomical Therapeutic Chemical (ATC) classification of drugs [16], compared to the SAR models based on physicochemical descriptors or structural alerts, a model based on chemical-chemical interactions can rank the order of the predictions more easily and yield better prediction results. In our study, we attempt to quantify chemical-chemical interactions for each pair of interactive compounds, and obtain the confidence scores of the interactions by which the toxicity end points were ordered. Briefly, compounds of seven categories including six categories of toxicity plus non-toxicity were collected. The interactive compounds of each query compound were identified utilizing STITCH (Search tool for interactions of chemicals) [17], [18]. Then, the score of each class of the query compound was obtained from the confidence scores of interactions between the query compound and its interactive compounds using the toxicity profile of the interactive compounds. Finally, the prediction quality of the model was evaluated using the Jackknife test through ten test groups. Each of these was constructed from the benchmark dataset and contained one training dataset and one external test dataset. Details are described in the following sections.

## Materials and Methods

### Benchmark Dataset

We obtained a total of 171,266 compounds from the Accelrys Toxicity Database 2011.4 [19], which had at least one toxicity effect belonging to the following six categories: (1) Acute Toxicity; (2) Mutagenicity; (3) Tumorigenicity; (4) Skin and Eye Irritation; (5) Reproductive Effects; (6) Multiple Dose Effects. Based on compound toxicity, these compounds are allocated to the 6 categories, allowing multiple assignments. In addition, 2,871 “non-toxic” compounds including FDA-approved drugs from DrugBank [20] and endogenic metabolites from the Human Metabolome database (HMDB) [21] were collected and labeled as a negative class. For convenience, the ‘non-toxic set’ is regarded as the 7^th^ category of compound toxicity. Due to lack of chemical-chemical interaction information in STITCH [17], [18], some compounds cannot be investigated by this approach. After excluding these compounds, a benchmark dataset 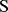 consisting of 17,233 compounds was retrieved, of which 16,587 were toxic and 646 were non-toxic. These compounds are classified into 7 categories of compound toxicity. Shown in **Table 1** is the distribution of compounds in each category. The codes of 17,233 compounds and their toxicity information can be found in **Table S1**.

**Table 1:** Distribution of compounds in each category of compound toxicity.

Tag	Toxicity	Total
*T*_1_	Acute Toxicity	12,633
*T*_2_	Mutagenicity	6,110
*T*_3_	Tumorigenicity	2,293
*T*_4_	Skin and Eye Irritation	2,353
*T*_5_	Reproductive Effects	2,501
*T*_6_	Multiple Dose Effects	4,198
*T*_7_	Non-toxicity	646
Total	–	30,734

**Figure 1:**
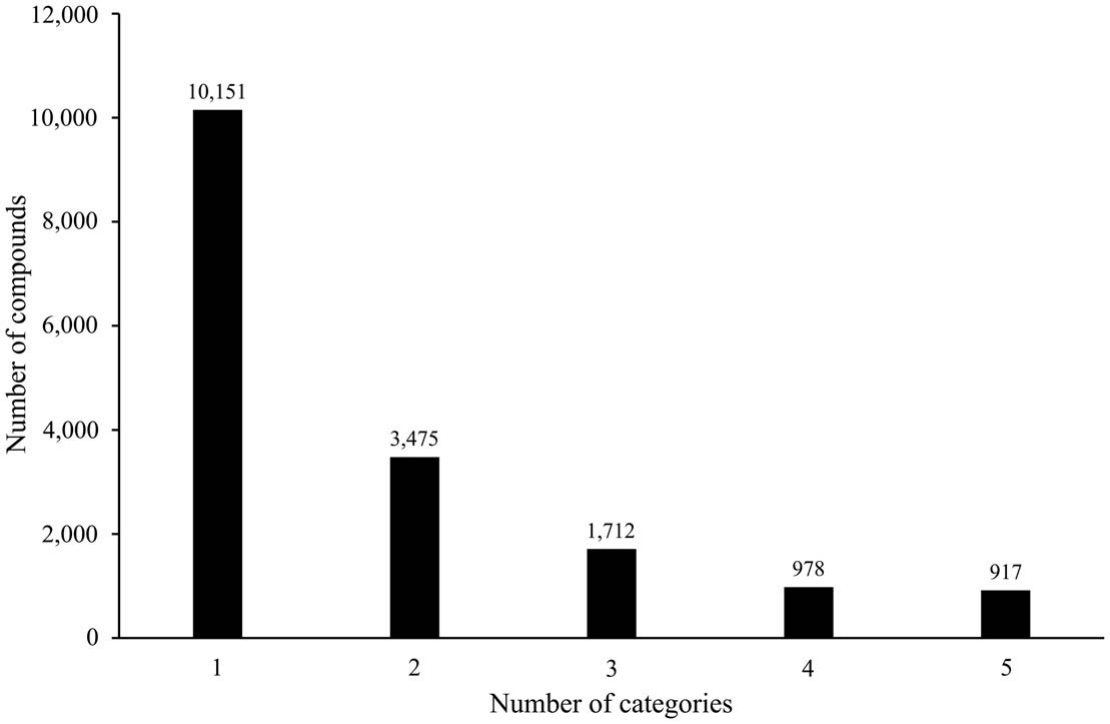
The number of compounds plotted against the number of categories in the benchmark dataset.

It is observed from **Table 1** that the sum of the number of compounds in all the 7 categories is much larger than the number of compounds, indicating that some compounds are allocated to more than one category of toxicity. Of the 17,233 compounds in the benchmark dataset, 10,151 compounds belong to only one category of toxicity, 3,475 compounds belong to two categories of toxicity, while others belong to 3–5 categories of toxicity and no compounds belong to more than five categories of toxicity - refer to **Figure 1** for a plot of the number of compounds against the number of categories of toxicity. Thus, prediction of compound toxicity is a multi-label classification problem. Like the case of processing proteins or compounds with multiple attributes [15], [16], [22], the proposed method would provide a series of candidate toxicities, ranging from the most to the least likely, instead of presenting only the most likely one.

To sufficiently evaluate the prediction method described in the following section, we constructed 10 test groups, denoted by 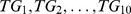, respectively. In each test group 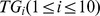, there is one training dataset 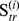 and one test dataset 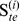, *i.e.*, 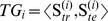, where the test dataset consisted of 1,723 compounds which were randomly selected from 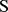, while the training dataset contained the remaining 15,510 samples in 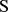, *i.e.*, 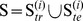 for each 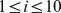. It is necessary to point out that, in each test group, the portion of the data in each class of the test dataset is roughly the same as that of the training dataset. Shown in **Table 2** is the distribution of compounds in training and test datasets of each test group.

**Table 2:** Distribution of compounds in training and test datasets of each test group.

	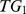		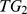		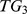		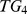		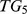	
Tag										
*T*_1_	11,382	1,251	11,387	1,246	11,351	1,282	11,364	1,269	11,385	1,248
*T*_2_	5,475	635	5,476	634	5,529	581	5,492	618	5,491	619
*T*_3_	2,065	228	2,065	228	2,063	230	2,063	230	2,056	237
*T*_4_	2,102	251	2,102	251	2,115	238	2,112	241	2,093	260
*T*_5_	2,235	266	2,235	266	2,260	241	2,255	246	2,235	266
*T*_6_	3,747	451	3,749	449	3,777	421	3,784	414	3,799	399
*T*_7_	582	64	577	69	586	60	582	64	583	63
Total	27,588	3,146	27,591	3,143	27,681	3,053	27,652	3,082	27,642	3,092
										
	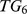		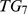		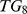		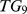		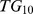	
**Tag**										
*T*_1_	11,367	1,266	11,395	1,238	11,369	1,264	11,374	1,259	11,353	1,280
*T*_2_	5,489	621	5,500	610	5,492	618	5,497	613	5,506	604
*T*_3_	2,075	218	2,067	226	2,070	223	2,043	250	2,070	223
*T*_4_	2,123	230	2,125	228	2,135	218	2,102	251	2,133	220
*T*_5_	2,244	257	2,243	258	2,236	265	2,258	243	2,234	267
*T*_6_	3,762	436	3,750	448	3,772	426	3,777	421	3,755	443
*T*_7_	583	63	587	59	579	67	569	77	584	62
Total	27,643	3,091	27,667	3,067	27,653	3,081	27,620	3,114	27,635	3,099

### Chemical-chemical Interactions

It is known that two proteins that can interact with each other are more likely to share common biological functions than non-interactive ones [8], [9], [10], [11]. Likewise, two interactive compounds are also more likely to share similar biological functions [15], [16]. Since toxicity is one of a compound’s properties and functions, utilizing chemical-chemical interactions to identify compound toxicity is deemed to be feasible.

The data for chemical-chemical interactions were retrieved from STITCH (chemical_chemical.links.detailed.v3.0.tsv.gz, http://stitch.embl.de/cgi/show_download_page.) [17], a well-known database including known and predicted interactions of chemicals and proteins collected from experiments, literature or other reliable sources. In the obtained file, the interaction unit contains two compounds and five kinds of scores with titles “Similarity”, “Experimental”, “Database”, “Textmining” and “Combined_score”. The last kind of score was used here to indicate the interactivity of two compounds, *i.e.*, two compounds with “Combined_score” greater than zero were deemed interactive compounds, because the last kind of score integrates the information of the other kinds of scores. Thus, the considered interactive compounds in this study contain the following three categories: (1) those participating in the same reactions; (2) those sharing similar structures or activities and (3) those with literature associations [17]. It is known that these categories correspond to the following three facts: (I) compounds involved in the same reactions occupy the same biological pathways; (II) compounds with similar structures or activities are likely to share similar functions, thereby occupying the same pathways with high probability; (III) the co-occurrence of two compounds, as noted in many studies, indicates some direct or indirect relationships, suggesting that they have the potential to share the same pathways. On the other hand, compounds in the same biological pathways always induce similar side effects, thereby having similar toxicity effects. Accordingly, it is reasonable to suppose that interactive compounds tend to have similar toxicity effects.

The value of the “Combined_score” of two interactive compounds indicates the likelihood that they can interact, *i.e.*, two interactive compounds with high “Combined_score” can interact with high probability. Thus, this score is also termed a confidence score in this study. For two compounds *c*_1_ and *c*_2_, let us denote the confidence score of an interaction between them by **Q**(*c*_1_,*c*_2_). Specifically, if there is no interaction information between *c*_1_ and *c*_2_ based on the current records in STITCH, their interaction confidence score is assigned zero, *i.e.*, **Q**(*c*_1_,*c*_2_) = 0. In this study, 323,432 interaction units, *i.e.*, 323,432 pairs of compounds with confidence scores greater than 0, were used to predict compound toxicity. The detailed information on these interaction units can be found in **Table S2**.

### Prediction Method

As is mentioned in the above section, interactive compounds are more likely to have common toxicity. Accordingly, the toxicities of a query compound can be identified according to its interactive compounds.

For convenience, let *T*_1_, *T*_2_, …, *T*_7_ denote the seven categories of toxicity, where *T*_1_ denotes “Acute Toxicity”, *T*_2_ “Mutagenicity”, and so forth (see column 1 and 2 of **Table 1**). Suppose that there are *n* compounds in the training dataset, that is *c*_1_, *c*_2_, …, *c_n_*, the toxicity of a compound *c_i_* in the training dataset is formulated as
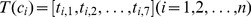
(1)where



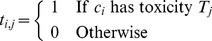
(2)Given a query compound *c_q_*, its toxicity is predicted not only by its interactive compounds but also by the confidence scores of their interactions. The score indicating that the query compound *c_q_* has toxicity *T_j_* is calculated by
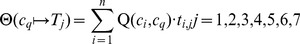
(3)


The high score 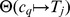 means that there are many interactive compounds of *c_q_* in the training dataset that have toxicity *T_j_* or some interactions between *c_q_* and its interactive compounds having toxicity *T_j_* are labeled by high confidence scores. In view of this, the greater the score 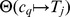, the more likely that the compound *c_q_* has toxicity *T_j_*. In particular, if 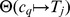 for some *j*, it is indicated that the probability that the query *c_q_* having the *j*-th category of toxicity is zero because there are no interactive compounds of *c_q_* in the training dataset that have toxicity *T_j_*.

Since this is a multi-label classification problem, *i.e.*, some compounds have more than one category of toxicity. A prediction method only providing the most likely toxicity is not an optimal choice. Thus, our method is valuable in that it can provide a series of candidate toxicities for a query compound, ranging from the most likely to the least likely. For example, if the results obtained from **Eq. 3** are
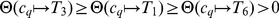
(4)it can be interpreted to mean that there are three candidate toxicities for the query compound *c_q_*, and the most likely toxicity for *c_q_* is *T*_3_ (“Tumorigenicity”, cf. **Table 1**), followed by *T*_1_ (“Acute Toxicity”) and *T*_6_ (“Multiple Dose Effects”). In addition, *T*_3_ is called the 1^st^ order prediction, *T*_1_ the 2^nd^ order prediction, and so forth.

### Jackknife Test

The Jackknife test [16] is often used to examine the performance of various predictors, because it can always provide a unique prediction result for a given dataset. It has been widely used by investigators to evaluate their predictors [23], [24], [25], [26], [27], [28], [29], [30], [31], [32], [33]. During the test, each sample in the training dataset is singled out one-by-one and tested by the predictor trained by the other samples. Thus, each sample is tested exactly once.

### Accuracy Measurement

The *j*-th order prediction accuracy is calculated by the following formula [15], [16]:
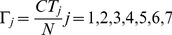
(5)where *CT_j_* denotes the number of compounds whose *j*-th order prediction is one of its true toxicities, and *N* denotes the total number of compounds in the dataset. If a prediction method can obtain high 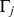 with small *j* and low 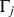 with large *j*, it implies that the method arranges the candidate toxicities well. Among them, the 1^st^ order prediction accuracy is the most important indicator of good or bad performance.

Although the seven prediction accuracies can be obtained by **Eq. 5**, none of them provides the overall prediction accuracy. In view of this, we employ another measurement that calculates the proportion of true toxicities of the first *m* predictions. It can be calculated as follows [16]:
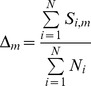
(6)where *S_i,m_* represents the number of the correct predictions of the *i-*th compound among its first *m* predictions, and *N_i_* represents the number of toxicities that the *i-*th compound has. Since different compounds may have different numbers of toxicities, the parameter *m* in **Eq. 6** is usually taken as the smallest integer no less than the average number of toxicities in the dataset, which can be computed by
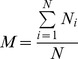
(7)where 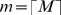. Obviously, a larger 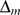 implies better prediction performance by the method for the identification of compound toxicity.

## Results

As described in the Section “Benchmark dataset”, 10 test groups were constructed to evaluate the method described in Section “Prediction method”. In each test group, there were one training dataset consisting of 15,510 compounds and one test dataset containing 1,723 compounds. The predicted results for each test group obtained by the proposed method are as follows.

### Performance of the Method on the Training Dataset

For the 15,510 compounds in each training dataset 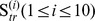, we conducted the prediction and evaluated its performance by the Jackknife test. Listed in the column with title 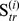 of **Table 3** are seven prediction accuracies, calculated by **Eq. 5**, for training dataset 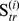, from which we can see that the 1^st^ order prediction accuracies were all around 79.50%, where the maximum was 79.57%, while the minimum was 79.23%; the 2^nd^ order ones were all around 37.30%. It is indicated that the proposed method is very stable. It is also observed from the corresponding columns of **Table 3** that the accuracies followed a descending trend when increasing the order number, indicating that the method sorted the candidate toxicities quite well for the compounds in each training dataset 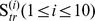. The average numbers of toxicities for compounds in each training dataset 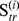 were about 1.78 according to **Eq. 7**, *i.e.*, *M* = 1.78. It is noteworthy that if one predicts compound toxicity by random guesses, the average success rate would be only 25.43% (1.78/7), which is much lower than each of the 1^st^ order prediction accuracies by our method. To evaluate the prediction accuracy by the method more thoroughly, **Eq. 6** was calculated by taking *m* = 2, *i.e.*, we considered the first two predictions for each compound in 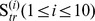 to see the proportions of true toxicities covered by these predictions. These proportions are shown in column 2 of **Table 4**, from which we can see that they were all about 65.50%, where the maximum was 65.61% while the minimum was 65.32%. Thus, it is indicated once again that our method is reliable.

**Table 3:** Prediction accuracies obtained by the method as applied to training and test datasets of each test group.

	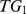		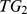		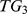		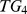		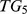	
Prediction Order										
1	79.40%	79.69%	79.45%	79.28%	79.23%	80.62%	79.28%	79.45%	79.30%	79.34%
2	37.16%	38.42%	37.14%	38.24%	37.54%	37.20%	37.17%	38.31%	37.40%	36.16%
3	22.18%	23.16%	22.20%	22.87%	22.32%	21.65%	22.29%	22.63%	22.53%	22.87%
4	15.45%	16.66%	15.49%	16.77%	16.35%	14.86%	15.46%	16.13%	15.41%	15.55%
5	11.06%	11.61%	11.04%	11.49%	11.00%	10.85%	10.88%	10.16%	10.95%	11.20%
6	6.92%	7.25%	6.84%	7.89%	7.23%	5.86%	6.99%	6.56%	6.85%	7.84%
7	1.21%	1.33%	1.22%	1.04%	1.27%	1.51%	1.39%	1.45%	1.26%	1.68%
										
	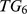		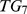		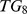		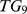		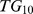	
**Prediction Order**	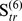	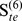	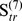	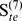	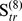	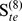	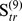	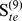	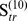	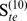
1	79.57%	80.15%	79.36%	79.98%	79.45%	79.05%	79.52%	79.80%	79.46%	79.34%
2	37.11%	37.72%	37.57%	36.10%	37.21%	38.65%	37.32%	35.98%	37.44%	37.20%
3	22.57%	22.29%	22.30%	23.39%	22.23%	24.03%	22.46%	23.33%	22.42%	22.93%
4	15.31%	15.90%	15.36%	15.55%	15.52%	14.74%	15.40%	16.25%	15.36%	16.37%
5	10.93%	10.45%	10.95%	11.55%	11.08%	10.10%	10.74%	11.55%	10.87%	10.74%
6	7.00%	6.56%	7.00%	6.62%	7.16%	5.86%	6.76%	7.78%	6.97%	7.25%
7	1.25%	1.57%	1.32%	0.99%	1.32%	1.45%	1.27%	1.57%	1.30%	1.33%

**Table 4:** Proportions of true toxicities covered by the first two predictions for training and test datasets of each test group.

Test group	Training dataset	Test dataset
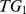	65.52%	64.69%
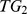	65.54%	64.52%
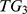	65.43%	66.49%
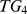	65.32%	65.83%
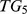	65.48%	64.36%
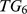	65.46%	65.71%
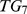	65.55%	65.21%
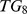	65.43%	65.82%
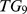	65.61%	64.07%
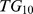	65.61%	64.79%

### Performance of the Method on the Test Dataset

For the 1,723 compounds in each test dataset 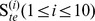, the toxicities of these compounds were predicted by the proposed method described in Section “Prediction method” based on the compounds in the training dataset 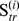. After processing by **Eq. 5**, seven prediction accuracies for each test dataset 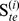 were obtained and were listed in the column with title 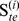 of **Table 3**. It is observed that the 1^st^ order prediction accuracies were all about 79.50%. Similar to the seven prediction accuracies for each training dataset 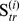, those of test dataset 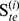 also followed a descending trend with the increase of the order number, implying that our method also arranged the candidate toxicities of samples in each test dataset quite well. According to **Eq. 7**, the average numbers of toxicities for the compounds in each test dataset were about 1.80. Thus, we still considered the first two predictions of each sample in 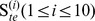 to calculate the proportions of true toxicities covered by these predictions, *i.e.*, computing **Eq. 6** by taking *m* = 2. Listed in column 3 of **Table 4** are ten proportions for ten test datasets, each yielding a probability of approximately 65%.

## Discussion

### Understanding of the Toxicity Prediction Results

It is observed from **Table 3** that the performance of the method on ten test groups is similar. Thus, the first test group (*i.e.*, 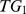) is used as an example to show how to interpret the toxicity predicting results in detail.

**Figure 2:**
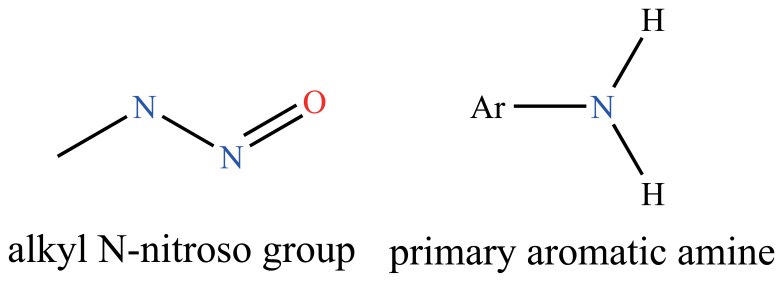
The structures of the alkyl N-nitroso group and the primary aromatic amine group.

Our multiclass model achieved a quite promising performance using the chemical-chemical interactions data on test group 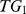 (see **Table 3** for details). For example, the compound 4-(N-methyl-N-nitrosamino)-1-(3-pyridyl)-1-butanone (CID000047289, NNK) shows positive results for five toxicity endpoints: *T*_1_, *T*_2_, *T*_3_, *T*_5_, and *T*_6_. Our model accurately predicted these five kinds of endpoints, and provided the order predictions as *T*_3_> *T*_2_> *T*_1_>*T*_6_> *T*_5_> *T*_4_>*T*_7_. The 7^th^ label representing ‘non-toxic’ was ranked as the last, suggesting that this compound is very likely to have toxic effects. As stated in the Section “Chemical-chemical interactions”, the interactive compounds derived from STITCH tend to have the same toxicity categories. 4-(Methylnitrosamino)-1-(3-pyridyl)-1-butanol (CID000104856, NNAL), an interactive compound of NNK, has toxicities *T*_2_ and *T*_3_, which are also shared by NNK. The alkyl N-nitroso group (see **Figure 2**) of these two compounds associates with the formation of DNA adducts, and induces lung cancer in laboratory animals [34], [35], [36]. Another example is trimethoprim (CID000005578), which is positive for five toxicity endpoints: *T*_1_, *T*_2_, *T*_4_, *T*_5_, and *T*_6_. The prediction order of our model was *T*_1_> *T*_6_> *T*_2_>*T*_5_> *T*_4_> *T*_3_>*T*_7_. This compound was considered to be a carcinogen according to chemical-chemical interactions, but the Accelrys Toxicity database [19] labeled this compound only as a mutagen. However, it is reasonable to assume this compound as a carcinogen because it has a genotoxic toxicophore-aromatic amine (see **Figure 2**) [5], [37], [38]. Typically, mutation is one of the first steps in the development of cancer [39].

Tasosartan (CID000060919) is an angiotensin II (AngII) receptor blocker [40], which is labeled as a relatively “non-toxic” compound in the dataset. Using our model, the order prediction of this compound was *T*_7_> *T*_1_> *T*_6_> *T*_2_. The 1^st^ order prediction is “non-toxic”, consistent with the experimental data available. Among seven interactive compounds in the training dataset retrieved from STITCH (see **Table 5**), the top five interactive compounds are “non-toxic”, and their confidence scores are relatively high. However, the latter two interactive compounds are toxic, so tasosartan is predicted to have some toxicity effects in our model. However, the possibility of its possessing these toxicities is less than that of its not possessing toxicity (*i.e.*, “non-toxic”).

**Table 5:** Details of Tasosartan’s interactive compounds in the training dataset.

Compound ID	Tag of toxicity class	Its interactive compound ID	Tag of toxicity class	Confidence score
CID000060919	*T*_7_	CID000003749	*T*_7_	679
CID000060919	*T*_7_	CID000002541	*T*_7_	670
CID000060919	*T*_7_	CID000060921	*T*_7_	669
CID000060919	*T*_7_	CID000003961	*T*_7_	667
CID000060919	*T*_7_	CID000060846	*T*_7_	658
CID000060919	*T*_7_	CID000065999	*T*_1_, *T*_6_	643
CID000060919	*T*_7_	CID000054738	*T*_1_, *T*_2_	172

The predictions for NNK, trimethoprim, and tasosartan and the prediction accuracies of the method indicate that interactive compounds can share common toxicity with high probability, which assessment conforms to the results of predicting other attributes of compounds [15], [16]. The confidence scores of chemical-chemical interactions contribute significantly to the prediction of compound toxicity. As shown in **Table 5**, the interactive compounds of tasosartan with high confidence scores dominantly have the same toxicity as tasosartan. On the other hand, the predicted results for NNK, trimethoprim, and tasosartan reflect a limitation of our model: the judgment of “toxic” or “non-toxic” is based on a collective set of compounds with interactive information. However, some compounds with low confidence scores exist and they may contribute to the input of promiscuous interaction information to the final classification model. To address this issue, a future endeavor should introduce a threshold to the interaction confidence score and exclude “noisy” information to obtain a more accurate prediction.

Moreover, many more compounds are without chemical-chemical interactions in the original Accelrys Toxicity database. It is expected that the problem of predicting compound toxicity can be solved more favorably by the method as increasing amounts of chemical-chemical interaction information become available.

### Analysis of the Relationship between Different Chemical Toxicity Effects

In the Accelrys Toxicity Database, there are 3,607 compounds with more than two types of toxicity effects and 3,475 compounds with exact two effects (refer to **Figure 1**). We analyzed the number of common compounds belonging to two categories, and the ratio of the number of common compounds to the number of non-overlapping compounds of the two categories (see **Table 6**). It can be found that the intersection of *T*_5_ (“Reproductive Effects”, cf. **Table 1**) and *T*_6_ (“Multiple Dose Effects”) is the largest, sharing 26.6% of common compounds. The overlapping compounds suggest that there may be a causal relationship between the two categories. Specifically, the reproductive effects may cause multiple dose effects, *i.e.*, reproductive toxicities may be cumulative, and hence be regarded as showing multiple dose effects in the meantime. The followed instances of correspondence between two categories are *T*_2_ (“Mutagenicity”) vs. *T*_3_ (“Tumorigenicity”) and *T*_1_ (“Acute Toxicity”) vs. *T*_6_ (“Multiple Dose Effects”). Since, in many cases, mutation is one of the first steps in the development of cancer [39], we took *T*_2_ (“Mutagenicity”) vs. *T*_3_ (“Tumorigenicity”) as an example to study the relationship between the two toxic categories.

**Table 6:** The details of common compounds belonging to two categories.

Tag of toxicity class	*T*_1_	*T*_2_	*T*_3_	*T*_4_	*T*_5_	*T*_6_
*T*_1_	12,633^a^	3,483(22.8%)^b^	1,485(11.0%)	2,027(15.6%)	2,075(15.9%)	3,446(25.7%)
*T*_2_		6110	1,720(25.7%)	1,213(16.7%)	1,336(18.4%)	1,723(20.1%)
*T*_3_			2293	570(14.0%)	753(18.6%)	781(13.7%)
*T*_4_				2353	731(17.7%)	897(15.9%)
*T*_5_					2501	1,409(26.6%)
*T*_6_						4,198

a The number of common compounds belonging to two categories.

b The number in parenthesis means the ratio of the number of common compounds to the number of non-overlapping compounds of the two categories.

From the viewpoint of mechanism of action, carcinogens can be classified into genotoxic or epigenetic carcinogens. Genotoxic carcinogens can bind covalently to DNA, and many known mutagens belong to this category. In the dataset, there are 1,720 common compounds with simultaneous toxicity *T*_2_ (“Mutagenicity”) and *T*_3_ (“Tumorigenicity”). The Structural alerts (SAs) provided by Benigni [37], which are molecular functional groups associated with a specific toxicity end point [38], were used here to gain insights into the correspondence of the two toxic effects. As summarized in **Table S3**, we illustrated a few examples for each of the matched SAs.

As previously mentioned, not all of the mutagens are carcinogens. For example, α,β-unsaturated carbonyl compounds can interact with DNA by Michael addition, then lead to mutagenic and carcinogenic responses [37], *e.g.* acrylamide (CID000006579) and 2-butenal (CID000447466). However, if an α,β-unsaturated carbonyl compound has conformational constraints or alkyl groups at the site of nucleophilic attack, the compound would be prone to reaction via Schiff base formation [41]. This change may only generate the DNA-adducts, but not undergo the following carcinogenic process [37]. This means that this kind of compound has no carcinogenicity, *e.g.* (E)-2-methyl-2-butenal (CID005321950) and 2-propylacrolein (CID000070609).

**Figure 3:**
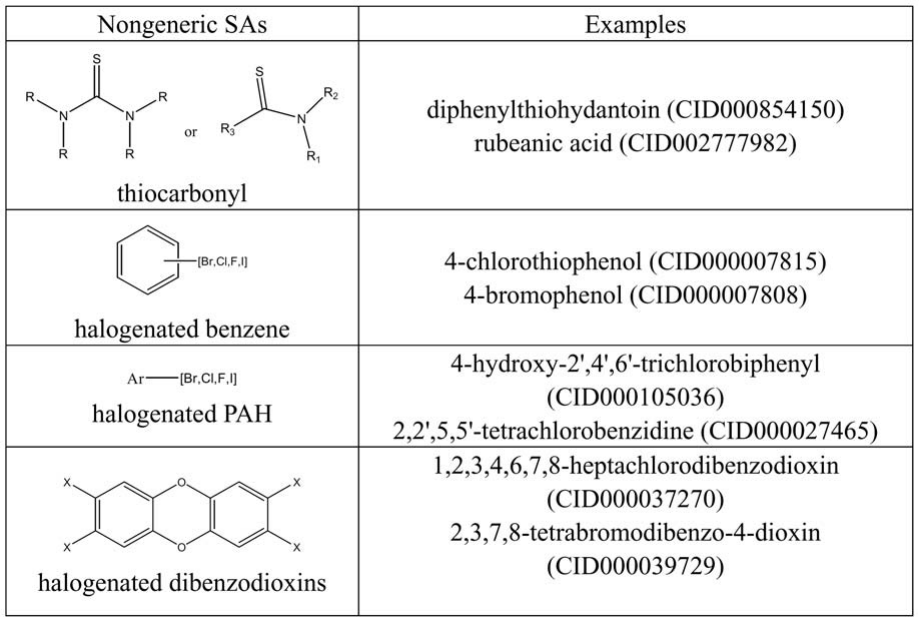
Nongeneric SAs (Benigni) and some carcinogens matching these SAs.

Epigenetic carcinogens do not usually bind directly to DNA, but have a large variety of different and specific mechanisms, and behave negatively in the standard mutagenicity assay [42]. Thus, some compounds that can match nongeneric SAs [37] are only carcinogens, not mutagens (see **Figure 3**).

### Conclusions

In this study, a multi-classifier for six toxicity effects was built based on 17,233 compounds with their experimental toxicity information available and 323,432 pairs of mapped chemical-chemical interaction information extracted from the STITCH database. A new chemical entity can have multiple toxicity effects, so a multiclass toxicity prediction tool may prove to be practically more valuable to chemists than a traditional binary classification model. It can provide a better toxicity profile for a compound rather than merely indicating whether the compound has a specific toxic action or potential. The outstanding performance of our approach suggests that the multi-classification scheme is feasible and effective for *in silico* chemical toxicity prediction.

## Supporting Information

Table S1 **List of 17,233 compounds investigated in this study and their toxicity information.**
(PDF)

Table S2 **List of 323,432 interaction units used to predict compound toxicity in this study.**
(PDF)

Table S3 **List of SAs (Benigni) and examples matching SAs in our dataset.**
(PDF)
