# Supplementary material for: Predicting Chemical Toxicity Effects Based on Chemical-Chemical Interactions
Source: PLoS One. 2013 Feb 15;8(2):e56517. doi: 10.1371/journal.pone.0056517 (PMC3574107; doi:10.1371/journal.pone.0056517)
Supplement: Table S1 — List of 17,233 compounds investigated in this study and their toxicity information. (PDF) [file pone.0056517.s001.pdf]

Table S1: 17,233 compounds investigated in this study and their toxicity information. We use "A××××" to represent each compound due to the copyright of the data.

Compound	Toxicity
A00001	Acute Toxicity
A00002	Acute Toxicity, Mutagenicity, Skin and Eye Irritation, Reproductive Effects
A00003	Acute Toxicity, Mutagenicity, Tumorigenicity, Skin and Eye Irritation, Multiple Dose Effects
A00004	Acute Toxicity, Mutagenicity, Tumorigenicity, Skin and Eye Irritation,
A00005	Acute Toxicity, Mutagenicity, Tumorigenicity, Skin and Eye Irritation,
A00006	Acute Toxicity, Multiple Dose Effects
A00007	Acute Toxicity, Mutagenicity, Tumorigenicity, Multiple Dose Effects
A00008	Acute Toxicity, Mutagenicity, Tumorigenicity, Skin and Eye Irritation,
A00009	Non-toxicity
A00010	Acute Toxicity, Multiple Dose Effects
A00011	Non-toxicity
A00012	Mutagenicity
A00013	Acute Toxicity, Mutagenicity, Multiple Dose Effects
A00014	Non-toxicity
A00015	Acute Toxicity, Mutagenicity, Tumorigenicity, Multiple Dose Effects
A00016	Non-toxicity
A00017	Acute Toxicity, Multiple Dose Effects
A00018	Mutagenicity, Tumorigenicity
A00019	Mutagenicity, Tumorigenicity
A00020	Acute Toxicity
A00021	Non-toxicity
A00022	Mutagenicity
A00023	Mutagenicity
A00024	Skin and Eye Irritation, Multiple Dose Effects
A00025	Non-toxicity
A00026	Acute Toxicity, Mutagenicity
A00027	Non-toxicity
A00028	Non-toxicity
A00029	Acute Toxicity, Tumorigenicity, Multiple Dose Effects
A00030	Acute Toxicity, Mutagenicity, Skin and Eye Irritation, Multiple Dose Effects
A00031	Acute Toxicity, Mutagenicity, Skin and Eye Irritation
A00032	Acute Toxicity
A00033	Acute Toxicity, Skin and Eye Irritation
A00034	Acute Toxicity, Mutagenicity, Skin and Eye Irritation, Multiple Dose Effects
A00035	Acute Toxicity, Mutagenicity, Skin and Eye Irritation
A00036	Non-toxicity
A00037	Acute Toxicity, Reproductive Effects, Multiple Dose Effects
A00038	Mutagenicity
A00039	Non-toxicity
A00040	Acute Toxicity, Mutagenicity, Tumorigenicity, Skin and Eye Irritation,

A00041	Non-toxicity
A00042	Acute Toxicity, Mutagenicity, Tumorigenicity, Skin and Eye Irritation,
A00043	Acute Toxicity, Mutagenicity, Tumorigenicity, Reproductive Effects
A00044	Acute Toxicity, Mutagenicity, Skin and Eye Irritation, Reproductive Effects, Multiple Dose Effects
A00045	Acute Toxicity, Mutagenicity, Skin and Eye Irritation, Reproductive Effects, Multiple Dose Effects
A00046	Non-toxicity
A00047	Acute Toxicity
A00048	Acute Toxicity, Mutagenicity, Skin and Eye Irritation, Multiple Dose Effects
A00049	Non-toxicity
A00050	Tumorigenicity, Skin and Eye Irritation
A00051	Acute Toxicity
A00052	Acute Toxicity, Mutagenicity, Tumorigenicity, Skin and Eye Irritation, Multiple Dose Effects
A00053	Acute Toxicity, Mutagenicity, Tumorigenicity, Reproductive Effects
A00054	Acute Toxicity, Mutagenicity, Reproductive Effects
A00055	Acute Toxicity, Mutagenicity, Tumorigenicity, Reproductive Effects,
A00056	Acute Toxicity, Mutagenicity, Tumorigenicity, Skin and Eye Irritation, Multiple Dose Effects
A00057	Acute Toxicity, Mutagenicity, Tumorigenicity, Skin and Eye Irritation,
A00058	Acute Toxicity, Multiple Dose Effects
A00059	Non-toxicity
A00060	Acute Toxicity
A00061	Acute Toxicity, Mutagenicity, Skin and Eye Irritation, Multiple Dose Effects
A00062	Acute Toxicity, Mutagenicity, Skin and Eye Irritation
A00063	Acute Toxicity, Mutagenicity, Skin and Eye Irritation, Reproductive Effects, Multiple Dose Effects
A00064	Acute Toxicity, Mutagenicity, Skin and Eye Irritation, Multiple Dose Effects
A00065	Acute Toxicity, Mutagenicity
A00066	Non-toxicity
A00067	Acute Toxicity, Reproductive Effects, Multiple Dose Effects
A00068	Acute Toxicity, Mutagenicity, Reproductive Effects, Multiple Dose Effects
A00069	Acute Toxicity, Mutagenicity, Tumorigenicity, Skin and Eye Irritation,
A00070	Acute Toxicity, Multiple Dose Effects
A00071	Acute Toxicity, Mutagenicity, Tumorigenicity, Multiple Dose Effects
A00072	Acute Toxicity, Reproductive Effects
A00073	Acute Toxicity, Mutagenicity, Tumorigenicity, Multiple Dose Effects
A00074	Acute Toxicity, Mutagenicity, Tumorigenicity, Skin and Eye Irritation, Multiple Dose Effects
A00075	Non-toxicity
A00076	Acute Toxicity, Mutagenicity, Tumorigenicity, Skin and Eye Irritation,
A00077	Acute Toxicity, Skin and Eye Irritation
A00078	Acute Toxicity, Skin and Eye Irritation
A00079	Mutagenicity, Skin and Eye Irritation, Reproductive Effects
A00080	Acute Toxicity, Mutagenicity, Tumorigenicity, Reproductive Effects,

A00081	Acute Toxicity, Mutagenicity, Skin and Eye Irritation, Reproductive Effects, Multiple Dose Effects
A00082	Non-toxicity
A00083	Acute Toxicity
A00084	Acute Toxicity
A00085	Acute Toxicity, Mutagenicity, Tumorigenicity, Reproductive Effects,
A00086	Acute Toxicity, Multiple Dose Effects
A00087	Acute Toxicity, Mutagenicity, Tumorigenicity, Skin and Eye Irritation,
A00088	Multiple Dose Effects
A00089	Acute Toxicity, Mutagenicity, Tumorigenicity, Skin and Eye Irritation,
A00090	Acute Toxicity, Mutagenicity, Skin and Eye Irritation, Reproductive Effects
A00091	Acute Toxicity
A00092	Acute Toxicity, Mutagenicity, Tumorigenicity, Skin and Eye Irritation,
A00093	Acute Toxicity
A00094	Acute Toxicity, Skin and Eye Irritation, Reproductive Effects, Multiple Dose
A00095	Acute Toxicity, Mutagenicity, Skin and Eye Irritation, Reproductive Effects, Multiple Dose Effects
A00096	Acute Toxicity
A00097	Mutagenicity
A00098	Acute Toxicity, Multiple Dose Effects
A00099	Acute Toxicity, Multiple Dose Effects
A00100	Acute Toxicity, Mutagenicity, Tumorigenicity, Skin and Eye Irritation,
A00101	Non-toxicity
A00102	Non-toxicity
A00103	Non-toxicity
A00104	Non-toxicity
A00105	Acute Toxicity, Multiple Dose Effects
A00106	Acute Toxicity, Mutagenicity
A00107	Non-toxicity
A00108	Acute Toxicity
A00109	Acute Toxicity, Mutagenicity, Skin and Eye Irritation
A00110	Acute Toxicity, Mutagenicity
A00111	Acute Toxicity, Mutagenicity, Skin and Eye Irritation, Multiple Dose Effects
A00112	Acute Toxicity
A00113	Acute Toxicity
A00114	Acute Toxicity
A00115	Non-toxicity
A00116	Non-toxicity
A00117	Non-toxicity
A00118	Acute Toxicity, Multiple Dose Effects
A00119	Non-toxicity
A00120	Non-toxicity
A00121	Non-toxicity
A00122	Acute Toxicity, Mutagenicity, Skin and Eye Irritation, Multiple Dose Effects
A00123	Acute Toxicity, Mutagenicity, Skin and Eye Irritation, Multiple Dose Effects
A00124	Acute Toxicity, Mutagenicity, Multiple Dose Effects

A00125	Acute Toxicity, Mutagenicity
A00126	Acute Toxicity
A00127	Mutagenicity
A00128	Non-toxicity
A00129	Non-toxicity
A00130	Acute Toxicity, Tumorigenicity, Skin and Eye Irritation, Reproductive Effects, Multiple Dose Effects
A00131	Acute Toxicity, Mutagenicity
A00132	Non-toxicity
A00133	Mutagenicity, Multiple Dose Effects
A00134	Mutagenicity
A00135	Acute Toxicity
A00136	Mutagenicity, Multiple Dose Effects
A00137	Acute Toxicity
A00138	Acute Toxicity, Mutagenicity, Skin and Eye Irritation, Reproductive Effects, Multiple Dose Effects
A00139	Mutagenicity
A00140	Mutagenicity, Tumorigenicity
A00141	Acute Toxicity, Mutagenicity, Skin and Eye Irritation, Multiple Dose Effects
A00142	Acute Toxicity
A00143	Mutagenicity
A00144	Acute Toxicity, Mutagenicity, Tumorigenicity, Skin and Eye Irritation, Multiple Dose Effects
A00145	Acute Toxicity, Mutagenicity, Tumorigenicity, Skin and Eye Irritation
A00146	Acute Toxicity
A00147	Acute Toxicity, Mutagenicity, Skin and Eye Irritation, Multiple Dose Effects
A00148	Acute Toxicity
A00149	Acute Toxicity, Mutagenicity, Tumorigenicity, Skin and Eye Irritation,
A00150	Acute Toxicity, Mutagenicity, Reproductive Effects, Multiple Dose Effects
A00151	Acute Toxicity, Mutagenicity, Skin and Eye Irritation, Reproductive Effects, Multiple Dose Effects
A00152	Acute Toxicity, Mutagenicity, Tumorigenicity, Skin and Eye Irritation,
A00153	Acute Toxicity, Mutagenicity, Tumorigenicity, Skin and Eye Irritation,
A00154	Acute Toxicity, Mutagenicity, Skin and Eye Irritation, Reproductive Effects, Multiple Dose Effects
A00155	Acute Toxicity, Mutagenicity, Tumorigenicity, Reproductive Effects,
A00156	Acute Toxicity, Skin and Eye Irritation, Reproductive Effects, Multiple Dose
A00157	Acute Toxicity, Mutagenicity, Tumorigenicity, Skin and Eye Irritation,
A00158	Acute Toxicity, Mutagenicity
A00159	Acute Toxicity, Mutagenicity, Tumorigenicity, Skin and Eye Irritation,
A00160	Acute Toxicity, Mutagenicity
A00161	Acute Toxicity, Skin and Eye Irritation, Reproductive Effects, Multiple Dose
A00162	Acute Toxicity, Mutagenicity
A00163	Acute Toxicity, Multiple Dose Effects
A00164	Non-toxicity
A00165	Acute Toxicity, Mutagenicity

A00166	Mutagenicity, Multiple Dose Effects
A00167	Acute Toxicity, Mutagenicity, Tumorigenicity, Skin and Eye Irritation, Multiple Dose Effects
A00168	Acute Toxicity, Mutagenicity, Tumorigenicity, Skin and Eye Irritation,
A00169	Acute Toxicity, Mutagenicity
A00170	Non-toxicity
A00171	Acute Toxicity, Mutagenicity, Tumorigenicity, Skin and Eye Irritation,
A00172	Non-toxicity
A00173	Acute Toxicity, Mutagenicity, Tumorigenicity, Skin and Eye Irritation, Multiple Dose Effects
A00174	Tumorigenicity
A00175	Acute Toxicity, Mutagenicity
A00176	Acute Toxicity, Mutagenicity, Tumorigenicity, Skin and Eye Irritation,
A00177	Acute Toxicity, Mutagenicity, Tumorigenicity
A00178	Acute Toxicity, Mutagenicity
A00179	Acute Toxicity, Reproductive Effects
A00180	Acute Toxicity, Mutagenicity
A00181	Acute Toxicity, Mutagenicity, Reproductive Effects, Multiple Dose Effects
A00182	Acute Toxicity
A00183	Acute Toxicity, Reproductive Effects, Multiple Dose Effects
A00184	Mutagenicity
A00185	Acute Toxicity, Mutagenicity
A00186	Non-toxicity
A00187	Acute Toxicity, Mutagenicity, Tumorigenicity, Reproductive Effects,
A00188	Acute Toxicity, Mutagenicity, Multiple Dose Effects
A00189	Acute Toxicity, Mutagenicity, Tumorigenicity, Skin and Eye Irritation, Multiple Dose Effects
A00190	Acute Toxicity, Mutagenicity, Tumorigenicity, Skin and Eye Irritation,
A00191	Acute Toxicity, Mutagenicity, Tumorigenicity, Reproductive Effects,
A00192	Non-toxicity
A00193	Acute Toxicity, Mutagenicity, Skin and Eye Irritation
A00194	Acute Toxicity
A00195	Non-toxicity
A00196	Acute Toxicity, Mutagenicity, Tumorigenicity, Skin and Eye Irritation,
A00197	Mutagenicity, Tumorigenicity
A00198	Acute Toxicity, Mutagenicity, Tumorigenicity, Skin and Eye Irritation, Multiple Dose Effects
A00199	Acute Toxicity
A00200	Acute Toxicity
A00201	Acute Toxicity, Mutagenicity, Tumorigenicity, Reproductive Effects,
A00202	Acute Toxicity, Mutagenicity, Tumorigenicity, Skin and Eye Irritation,
A00203	Acute Toxicity
A00204	Acute Toxicity, Mutagenicity, Skin and Eye Irritation, Reproductive Effects, Multiple Dose Effects
A00205	Non-toxicity
A00206	Acute Toxicity, Mutagenicity, Tumorigenicity, Multiple Dose Effects

A00207	Acute Toxicity
A00208	Acute Toxicity, Mutagenicity, Skin and Eye Irritation, Reproductive Effects, Multiple Dose Effects
A00209	Acute Toxicity, Mutagenicity, Reproductive Effects, Multiple Dose Effects
A00210	Acute Toxicity, Mutagenicity, Skin and Eye Irritation, Reproductive Effects, Multiple Dose Effects
A00211	Tumorigenicity, Multiple Dose Effects
A00212	Acute Toxicity, Mutagenicity, Tumorigenicity, Skin and Eye Irritation, Multiple Dose Effects
A00213	Acute Toxicity
A00214	Acute Toxicity, Mutagenicity, Tumorigenicity, Skin and Eye Irritation
A00215	Acute Toxicity, Mutagenicity, Tumorigenicity, Skin and Eye Irritation,
A00216	Acute Toxicity, Mutagenicity, Tumorigenicity, Skin and Eye Irritation,
A00217	Mutagenicity
A00218	Acute Toxicity, Mutagenicity, Tumorigenicity, Multiple Dose Effects
A00219	Acute Toxicity, Mutagenicity, Tumorigenicity, Skin and Eye Irritation,
A00220	Acute Toxicity, Skin and Eye Irritation, Multiple Dose Effects
A00221	Acute Toxicity, Skin and Eye Irritation, Reproductive Effects
A00222	Acute Toxicity
A00223	Acute Toxicity
A00224	Non-toxicity
A00225	Non-toxicity
A00226	Acute Toxicity
A00227	Acute Toxicity, Mutagenicity, Reproductive Effects, Multiple Dose Effects
A00228	Acute Toxicity, Mutagenicity, Reproductive Effects, Multiple Dose Effects
A00229	Non-toxicity
A00230	Acute Toxicity
A00231	Non-toxicity
A00232	Acute Toxicity, Mutagenicity, Tumorigenicity, Skin and Eye Irritation,
A00233	Acute Toxicity, Mutagenicity, Tumorigenicity, Skin and Eye Irritation,
A00234	Acute Toxicity, Mutagenicity, Skin and Eye Irritation, Multiple Dose Effects
A00235	Non-toxicity
A00236	Acute Toxicity, Mutagenicity, Tumorigenicity, Reproductive Effects
A00237	Acute Toxicity, Mutagenicity, Tumorigenicity, Skin and Eye Irritation,
A00238	Acute Toxicity, Reproductive Effects, Multiple Dose Effects
A00239	Acute Toxicity
A00240	Acute Toxicity, Mutagenicity, Skin and Eye Irritation
A00241	Non-toxicity
A00242	Acute Toxicity, Skin and Eye Irritation
A00243	Acute Toxicity, Mutagenicity, Skin and Eye Irritation, Reproductive Effects, Multiple Dose Effects
A00244	Acute Toxicity
A00245	Acute Toxicity, Mutagenicity, Tumorigenicity, Skin and Eye Irritation,
A00246	Acute Toxicity
A00247	Acute Toxicity, Skin and Eye Irritation, Reproductive Effects, Multiple Dose
A00248	Acute Toxicity, Skin and Eye Irritation, Multiple Dose Effects

A00249	Non-toxicity
A00250	Acute Toxicity, Mutagenicity
A00251	Mutagenicity, Tumorigenicity, Reproductive Effects, Multiple Dose Effects
A00252	Acute Toxicity, Mutagenicity, Multiple Dose Effects
A00253	Acute Toxicity, Mutagenicity, Multiple Dose Effects
A00254	Acute Toxicity, Mutagenicity, Skin and Eye Irritation, Multiple Dose Effects
A00255	Acute Toxicity
A00256	Acute Toxicity, Mutagenicity, Tumorigenicity, Skin and Eye Irritation,
A00257	Acute Toxicity, Mutagenicity, Reproductive Effects, Multiple Dose Effects
A00258	Non-toxicity
A00259	Acute Toxicity
A00260	Acute Toxicity
A00261	Non-toxicity
A00262	Acute Toxicity, Mutagenicity, Skin and Eye Irritation, Multiple Dose Effects
A00263	Acute Toxicity, Mutagenicity, Tumorigenicity
A00264	Acute Toxicity, Mutagenicity, Tumorigenicity, Skin and Eye Irritation,
A00265	Acute Toxicity, Mutagenicity, Reproductive Effects, Multiple Dose Effects
A00266	Non-toxicity
A00267	Acute Toxicity, Mutagenicity, Tumorigenicity
A00268	Acute Toxicity, Mutagenicity
A00269	Acute Toxicity, Mutagenicity, Tumorigenicity, Skin and Eye Irritation,
A00270	Mutagenicity, Reproductive Effects, Multiple Dose Effects
A00271	Acute Toxicity, Mutagenicity, Tumorigenicity, Skin and Eye Irritation,
A00272	Non-toxicity
A00273	Mutagenicity
A00274	Acute Toxicity, Mutagenicity, Tumorigenicity, Skin and Eye Irritation,
A00275	Non-toxicity
A00276	Non-toxicity
A00277	Acute Toxicity
A00278	Acute Toxicity
A00279	Acute Toxicity, Reproductive Effects, Multiple Dose Effects
A00280	Acute Toxicity, Multiple Dose Effects
A00281	Acute Toxicity
A00282	Acute Toxicity
A00283	Acute Toxicity, Multiple Dose Effects
A00284	Acute Toxicity
A00285	Acute Toxicity
A00286	Multiple Dose Effects
A00287	Acute Toxicity, Reproductive Effects
A00288	Acute Toxicity, Reproductive Effects, Multiple Dose Effects
A00289	Acute Toxicity
A00290	Acute Toxicity, Mutagenicity, Tumorigenicity, Skin and Eye Irritation, Multiple Dose Effects
A00291	Mutagenicity
A00292	Acute Toxicity, Skin and Eye Irritation
A00293	Acute Toxicity

A00294	Acute Toxicity, Mutagenicity, Tumorigenicity, Reproductive Effects
A00295	Acute Toxicity, Mutagenicity, Tumorigenicity, Reproductive Effects,
A00296	Acute Toxicity
A00297	Multiple Dose Effects
A00298	Acute Toxicity, Mutagenicity
A00299	Acute Toxicity, Multiple Dose Effects
A00300	Acute Toxicity
A00301	Acute Toxicity, Multiple Dose Effects
A00302	Acute Toxicity
A00303	Mutagenicity
A00304	Acute Toxicity, Mutagenicity, Reproductive Effects, Multiple Dose Effects
A00305	Acute Toxicity
A00306	Mutagenicity, Multiple Dose Effects
A00307	Acute Toxicity
A00308	Acute Toxicity
A00309	Acute Toxicity, Mutagenicity, Tumorigenicity, Reproductive Effects
A00310	Acute Toxicity, Mutagenicity, Tumorigenicity, Reproductive Effects,
A00311	Acute Toxicity, Mutagenicity, Skin and Eye Irritation, Reproductive Effects, Multiple Dose Effects
A00312	Acute Toxicity, Mutagenicity, Tumorigenicity, Skin and Eye Irritation,
A00313	Mutagenicity, Reproductive Effects
A00314	Acute Toxicity, Mutagenicity, Tumorigenicity, Reproductive Effects,
A00315	Acute Toxicity, Reproductive Effects
A00316	Acute Toxicity, Mutagenicity, Tumorigenicity, Skin and Eye Irritation,
A00317	Acute Toxicity
A00318	Acute Toxicity, Mutagenicity, Tumorigenicity, Reproductive Effects,
A00319	Acute Toxicity
A00320	Acute Toxicity
A00321	Acute Toxicity, Mutagenicity
A00322	Acute Toxicity, Mutagenicity, Tumorigenicity
A00323	Acute Toxicity
A00324	Acute Toxicity, Mutagenicity, Skin and Eye Irritation, Multiple Dose Effects
A00325	Acute Toxicity
A00326	Acute Toxicity, Mutagenicity, Multiple Dose Effects
A00327	Acute Toxicity, Mutagenicity
A00328	Acute Toxicity, Mutagenicity, Reproductive Effects, Multiple Dose Effects
A00329	Acute Toxicity
A00330	Acute Toxicity, Mutagenicity, Tumorigenicity
A00331	Acute Toxicity, Mutagenicity, Reproductive Effects, Multiple Dose Effects
A00332	Acute Toxicity
A00333	Acute Toxicity
A00334	Acute Toxicity, Mutagenicity
A00335	Acute Toxicity
A00336	Acute Toxicity, Mutagenicity
A00337	Acute Toxicity
A00338	Acute Toxicity, Mutagenicity, Tumorigenicity, Reproductive Effects,

A00339	Acute Toxicity
A00340	Acute Toxicity, Mutagenicity, Tumorigenicity, Multiple Dose Effects
A00341	Acute Toxicity, Skin and Eye Irritation
A00342	Acute Toxicity, Mutagenicity, Multiple Dose Effects
A00343	Acute Toxicity, Mutagenicity, Multiple Dose Effects
A00344	Mutagenicity
A00345	Acute Toxicity, Multiple Dose Effects
A00346	Acute Toxicity
A00347	Reproductive Effects
A00348	Acute Toxicity
A00349	Acute Toxicity, Mutagenicity, Skin and Eye Irritation, Reproductive Effects
A00350	Acute Toxicity, Mutagenicity, Skin and Eye Irritation, Multiple Dose Effects
A00351	Acute Toxicity, Mutagenicity, Tumorigenicity, Reproductive Effects,
A00352	Acute Toxicity, Mutagenicity, Tumorigenicity, Reproductive Effects,
A00353	Acute Toxicity
A00354	Acute Toxicity, Skin and Eye Irritation
A00355	Acute Toxicity
A00356	Acute Toxicity, Mutagenicity, Tumorigenicity
A00357	Acute Toxicity
A00358	Acute Toxicity, Mutagenicity, Reproductive Effects, Multiple Dose Effects
A00359	Mutagenicity, Tumorigenicity
A00360	Acute Toxicity, Reproductive Effects
A00361	Mutagenicity, Reproductive Effects
A00362	Multiple Dose Effects
A00363	Mutagenicity
A00364	Acute Toxicity
A00365	Acute Toxicity, Reproductive Effects
A00366	Acute Toxicity, Mutagenicity, Multiple Dose Effects
A00367	Acute Toxicity
A00368	Acute Toxicity
A00369	Acute Toxicity
A00370	Acute Toxicity, Mutagenicity, Tumorigenicity, Skin and Eye Irritation, Multiple Dose Effects
A00371	Acute Toxicity, Mutagenicity, Tumorigenicity, Multiple Dose Effects
A00372	Tumorigenicity, Multiple Dose Effects
A00373	Acute Toxicity
A00374	Acute Toxicity, Mutagenicity, Reproductive Effects, Multiple Dose Effects
A00375	Acute Toxicity, Reproductive Effects, Multiple Dose Effects
A00376	Acute Toxicity, Mutagenicity, Tumorigenicity, Skin and Eye Irritation,
A00377	Acute Toxicity, Mutagenicity, Tumorigenicity, Skin and Eye Irritation,
A00378	Acute Toxicity, Tumorigenicity, Reproductive Effects, Multiple Dose Effects
A00379	Acute Toxicity, Mutagenicity, Tumorigenicity, Skin and Eye Irritation,
A00380	Acute Toxicity, Mutagenicity, Tumorigenicity, Reproductive Effects
A00381	Acute Toxicity, Mutagenicity, Reproductive Effects
A00382	Acute Toxicity, Multiple Dose Effects
A00383	Acute Toxicity, Multiple Dose Effects

A00384	Skin and Eye Irritation
A00385	Mutagenicity
A00386	Mutagenicity
A00387	Acute Toxicity, Mutagenicity, Tumorigenicity, Reproductive Effects,
A00388	Non-toxicity
A00389	Acute Toxicity
A00390	Acute Toxicity, Mutagenicity
A00391	Acute Toxicity, Multiple Dose Effects
A00392	Acute Toxicity
A00393	Multiple Dose Effects
A00394	Acute Toxicity
A00395	Acute Toxicity, Mutagenicity, Tumorigenicity, Skin and Eye Irritation, Multiple Dose Effects
A00396	Non-toxicity
A00397	Acute Toxicity, Tumorigenicity, Reproductive Effects, Multiple Dose Effects
A00398	Acute Toxicity, Skin and Eye Irritation, Reproductive Effects, Multiple Dose
A00399	Acute Toxicity, Multiple Dose Effects
A00400	Non-toxicity
A00401	Acute Toxicity, Reproductive Effects, Multiple Dose Effects
A00402	Acute Toxicity
A00403	Acute Toxicity
A00404	Acute Toxicity
A00405	Acute Toxicity, Reproductive Effects, Multiple Dose Effects
A00406	Acute Toxicity, Multiple Dose Effects
A00407	Acute Toxicity
A00408	Acute Toxicity, Mutagenicity, Tumorigenicity, Reproductive Effects,
A00409	Acute Toxicity, Multiple Dose Effects
A00410	Mutagenicity, Reproductive Effects, Multiple Dose Effects
A00411	Non-toxicity
A00412	Non-toxicity
A00413	Mutagenicity, Multiple Dose Effects
A00414	Acute Toxicity
A00415	Acute Toxicity
A00416	Acute Toxicity, Mutagenicity, Reproductive Effects, Multiple Dose Effects
A00417	Acute Toxicity, Tumorigenicity, Reproductive Effects, Multiple Dose Effects
A00418	Non-toxicity
A00419	Acute Toxicity
A00420	Acute Toxicity, Mutagenicity
A00421	Non-toxicity
A00422	Acute Toxicity, Mutagenicity, Multiple Dose Effects
A00423	Acute Toxicity, Reproductive Effects, Multiple Dose Effects
A00424	Acute Toxicity, Mutagenicity, Skin and Eye Irritation, Multiple Dose Effects
A00425	Acute Toxicity, Multiple Dose Effects
A00426	Acute Toxicity, Mutagenicity, Multiple Dose Effects
A00427	Acute Toxicity, Multiple Dose Effects
A00428	Acute Toxicity, Reproductive Effects, Multiple Dose Effects

A00429	Acute Toxicity, Mutagenicity, Tumorigenicity, Reproductive Effects,
A00430	Acute Toxicity
A00431	Reproductive Effects, Multiple Dose Effects
A00432	Acute Toxicity, Tumorigenicity, Multiple Dose Effects
A00433	Acute Toxicity, Reproductive Effects, Multiple Dose Effects
A00434	Acute Toxicity, Multiple Dose Effects
A00435	Acute Toxicity, Skin and Eye Irritation
A00436	Acute Toxicity, Mutagenicity
A00437	Acute Toxicity, Mutagenicity, Tumorigenicity, Skin and Eye Irritation
A00438	Acute Toxicity, Mutagenicity, Tumorigenicity
A00439	Acute Toxicity
A00440	Acute Toxicity, Mutagenicity, Multiple Dose Effects
A00441	Non-toxicity
A00442	Acute Toxicity
A00443	Acute Toxicity, Mutagenicity, Reproductive Effects, Multiple Dose Effects
A00444	Acute Toxicity, Mutagenicity, Tumorigenicity, Multiple Dose Effects
A00445	Acute Toxicity, Mutagenicity, Tumorigenicity, Multiple Dose Effects
A00446	Acute Toxicity, Mutagenicity, Tumorigenicity, Skin and Eye Irritation,
A00447	Acute Toxicity, Mutagenicity, Tumorigenicity, Reproductive Effects,
A00448	Acute Toxicity, Mutagenicity, Skin and Eye Irritation, Reproductive Effects, Multiple Dose Effects
A00449	Acute Toxicity, Mutagenicity, Tumorigenicity, Skin and Eye Irritation,
A00450	Non-toxicity
A00451	Acute Toxicity, Skin and Eye Irritation
A00452	Acute Toxicity
A00453	Acute Toxicity, Mutagenicity, Tumorigenicity, Skin and Eye Irritation,
A00454	Acute Toxicity, Tumorigenicity, Multiple Dose Effects
A00455	Acute Toxicity, Mutagenicity, Tumorigenicity, Skin and Eye Irritation, Multiple Dose Effects
A00456	Acute Toxicity
A00457	Acute Toxicity, Mutagenicity, Tumorigenicity
A00458	Acute Toxicity
A00459	Acute Toxicity, Skin and Eye Irritation
A00460	Acute Toxicity
A00461	Acute Toxicity, Skin and Eye Irritation, Multiple Dose Effects
A00462	Acute Toxicity, Mutagenicity, Tumorigenicity, Multiple Dose Effects
A00463	Acute Toxicity, Mutagenicity, Multiple Dose Effects
A00464	Acute Toxicity, Multiple Dose Effects
A00465	Acute Toxicity, Tumorigenicity, Skin and Eye Irritation
A00466	Acute Toxicity, Reproductive Effects
A00467	Reproductive Effects, Multiple Dose Effects
A00468	Acute Toxicity, Mutagenicity, Tumorigenicity, Reproductive Effects,
A00469	Acute Toxicity, Mutagenicity
A00470	Mutagenicity
A00471	Acute Toxicity, Reproductive Effects, Multiple Dose Effects
A00472	Acute Toxicity, Mutagenicity, Tumorigenicity, Skin and Eye Irritation,

A00473	Acute Toxicity, Skin and Eye Irritation
A00474	Acute Toxicity
A00475	Non-toxicity
A00476	Acute Toxicity, Skin and Eye Irritation, Multiple Dose Effects
A00477	Acute Toxicity, Mutagenicity, Tumorigenicity, Skin and Eye Irritation, Multiple Dose Effects
A00478	Acute Toxicity, Mutagenicity, Tumorigenicity, Skin and Eye Irritation,
A00479	Acute Toxicity
A00480	Non-toxicity
A00481	Acute Toxicity, Mutagenicity
A00482	Acute Toxicity
A00483	Acute Toxicity, Mutagenicity, Skin and Eye Irritation, Reproductive Effects, Multiple Dose Effects
A00484	Mutagenicity
A00485	Acute Toxicity, Mutagenicity, Tumorigenicity, Reproductive Effects,
A00486	Acute Toxicity, Mutagenicity, Tumorigenicity
A00487	Acute Toxicity, Multiple Dose Effects
A00488	Acute Toxicity, Skin and Eye Irritation
A00489	Acute Toxicity
A00490	Acute Toxicity, Mutagenicity
A00491	Acute Toxicity, Tumorigenicity, Multiple Dose Effects
A00492	Acute Toxicity, Tumorigenicity, Reproductive Effects, Multiple Dose Effects
A00493	Acute Toxicity, Skin and Eye Irritation, Reproductive Effects, Multiple Dose
A00494	Non-toxicity
A00495	Acute Toxicity, Tumorigenicity, Multiple Dose Effects
A00496	Acute Toxicity
A00497	Acute Toxicity, Reproductive Effects, Multiple Dose Effects
A00498	Acute Toxicity, Mutagenicity, Tumorigenicity, Skin and Eye Irritation
A00499	Acute Toxicity
A00500	Non-toxicity
A00501	Acute Toxicity, Multiple Dose Effects
A00502	Acute Toxicity, Mutagenicity, Skin and Eye Irritation, Reproductive Effects, Multiple Dose Effects
A00503	Skin and Eye Irritation
A00504	Skin and Eye Irritation
A00505	Acute Toxicity, Reproductive Effects
A00506	Acute Toxicity, Skin and Eye Irritation
A00507	Acute Toxicity, Reproductive Effects
A00508	Acute Toxicity, Tumorigenicity, Reproductive Effects, Multiple Dose Effects
A00509	Acute Toxicity, Skin and Eye Irritation, Reproductive Effects, Multiple Dose
A00510	Acute Toxicity, Tumorigenicity, Reproductive Effects, Multiple Dose Effects
A00511	Acute Toxicity, Mutagenicity, Multiple Dose Effects
A00512	Acute Toxicity
A00513	Acute Toxicity, Mutagenicity, Reproductive Effects, Multiple Dose Effects
A00514	Acute Toxicity, Reproductive Effects, Multiple Dose Effects

A00515	Acute Toxicity, Mutagenicity, Skin and Eye Irritation, Reproductive Effects, Multiple Dose Effects
A00516	Acute Toxicity, Mutagenicity
A00517	Acute Toxicity, Multiple Dose Effects
A00518	Acute Toxicity
A00519	Acute Toxicity, Skin and Eye Irritation, Reproductive Effects
A00520	Acute Toxicity
A00521	Acute Toxicity, Multiple Dose Effects
A00522	Acute Toxicity, Mutagenicity, Tumorigenicity, Reproductive Effects,
A00523	Acute Toxicity
A00524	Acute Toxicity, Skin and Eye Irritation
A00525	Acute Toxicity
A00526	Acute Toxicity, Mutagenicity, Skin and Eye Irritation
A00527	Skin and Eye Irritation
A00528	Acute Toxicity, Reproductive Effects
A00529	Acute Toxicity
A00530	Acute Toxicity, Reproductive Effects, Multiple Dose Effects
A00531	Acute Toxicity, Mutagenicity, Tumorigenicity, Skin and Eye Irritation,
A00532	Acute Toxicity, Tumorigenicity, Reproductive Effects, Multiple Dose Effects
A00533	Non-toxicity
A00534	Acute Toxicity
A00535	Non-toxicity
A00536	Acute Toxicity, Mutagenicity, Reproductive Effects, Multiple Dose Effects
A00537	Acute Toxicity, Mutagenicity, Skin and Eye Irritation, Reproductive Effects, Multiple Dose Effects
A00538	Acute Toxicity, Mutagenicity, Skin and Eye Irritation, Reproductive Effects
A00539	Acute Toxicity
A00540	Acute Toxicity
A00541	Acute Toxicity, Mutagenicity, Tumorigenicity, Skin and Eye Irritation,
A00542	Acute Toxicity, Mutagenicity, Multiple Dose Effects
A00543	Acute Toxicity
A00544	Acute Toxicity, Mutagenicity, Reproductive Effects, Multiple Dose Effects
A00545	Acute Toxicity, Mutagenicity, Reproductive Effects, Multiple Dose Effects
A00546	Acute Toxicity, Mutagenicity, Tumorigenicity, Reproductive Effects,
A00547	Acute Toxicity
A00548	Acute Toxicity, Mutagenicity, Tumorigenicity, Skin and Eye Irritation,
A00549	Acute Toxicity
A00550	Acute Toxicity, Skin and Eye Irritation
A00551	Acute Toxicity, Reproductive Effects, Multiple Dose Effects
A00552	Acute Toxicity
A00553	Acute Toxicity, Tumorigenicity, Multiple Dose Effects
A00554	Mutagenicity, Tumorigenicity, Skin and Eye Irritation, Multiple Dose Effects
A00555	Acute Toxicity, Multiple Dose Effects
A00556	Acute Toxicity
A00557	Acute Toxicity, Skin and Eye Irritation, Multiple Dose Effects
A00558	Acute Toxicity, Skin and Eye Irritation

A00559	Acute Toxicity
A00560	Acute Toxicity
A00561	Acute Toxicity, Mutagenicity, Tumorigenicity, Reproductive Effects,
A00562	Acute Toxicity, Mutagenicity, Tumorigenicity, Skin and Eye Irritation,
A00563	Mutagenicity, Reproductive Effects
A00564	Acute Toxicity, Multiple Dose Effects
A00565	Acute Toxicity, Mutagenicity, Skin and Eye Irritation, Reproductive Effects, Multiple Dose Effects
A00566	Non-toxicity
A00567	Acute Toxicity, Mutagenicity
A00568	Acute Toxicity, Mutagenicity, Skin and Eye Irritation, Reproductive Effects, Multiple Dose Effects
A00569	Acute Toxicity, Reproductive Effects, Multiple Dose Effects
A00570	Acute Toxicity, Mutagenicity, Skin and Eye Irritation, Multiple Dose Effects
A00571	Acute Toxicity, Mutagenicity, Tumorigenicity, Skin and Eye Irritation,
A00572	Acute Toxicity, Mutagenicity, Tumorigenicity, Skin and Eye Irritation,
A00573	Acute Toxicity, Mutagenicity, Tumorigenicity, Reproductive Effects,
A00574	Acute Toxicity, Mutagenicity, Tumorigenicity, Skin and Eye Irritation,
A00575	Acute Toxicity, Reproductive Effects, Multiple Dose Effects
A00576	Acute Toxicity, Multiple Dose Effects
A00577	Acute Toxicity
A00578	Acute Toxicity
A00579	Acute Toxicity, Mutagenicity
A00580	Multiple Dose Effects
A00581	Mutagenicity
A00582	Reproductive Effects, Multiple Dose Effects
A00583	Acute Toxicity, Reproductive Effects, Multiple Dose Effects
A00584	Multiple Dose Effects
A00585	Non-toxicity
A00586	Acute Toxicity, Mutagenicity, Reproductive Effects, Multiple Dose Effects
A00587	Acute Toxicity, Mutagenicity, Tumorigenicity, Reproductive Effects,
A00588	Acute Toxicity, Mutagenicity, Skin and Eye Irritation, Reproductive Effects, Multiple Dose Effects
A00589	Acute Toxicity
A00590	Acute Toxicity, Multiple Dose Effects
A00591	Mutagenicity, Tumorigenicity, Multiple Dose Effects
A00592	Multiple Dose Effects
A00593	Acute Toxicity
A00594	Skin and Eye Irritation
A00595	Acute Toxicity, Reproductive Effects, Multiple Dose Effects
A00596	Acute Toxicity
A00597	Acute Toxicity, Mutagenicity, Skin and Eye Irritation, Reproductive Effects, Multiple Dose Effects
A00598	Acute Toxicity, Mutagenicity, Skin and Eye Irritation, Reproductive Effects, Multiple Dose Effects
A00599	Acute Toxicity

A00600	Acute Toxicity, Mutagenicity, Tumorigenicity, Reproductive Effects,
A00601	Acute Toxicity, Mutagenicity, Tumorigenicity, Multiple Dose Effects
A00602	Acute Toxicity
A00603	Acute Toxicity, Reproductive Effects, Multiple Dose Effects
A00604	Acute Toxicity, Mutagenicity, Reproductive Effects, Multiple Dose Effects
A00605	Acute Toxicity, Mutagenicity, Skin and Eye Irritation, Reproductive Effects, Multiple Dose Effects
A00606	Acute Toxicity
A00607	Multiple Dose Effects
A00608	Acute Toxicity, Reproductive Effects, Multiple Dose Effects
A00609	Acute Toxicity, Tumorigenicity, Skin and Eye Irritation, Reproductive Effects, Multiple Dose Effects
A00610	Acute Toxicity, Reproductive Effects
A00611	Acute Toxicity, Mutagenicity, Skin and Eye Irritation
A00612	Acute Toxicity
A00613	Non-toxicity
A00614	Acute Toxicity, Mutagenicity, Tumorigenicity, Reproductive Effects,
A00615	Acute Toxicity, Multiple Dose Effects
A00616	Acute Toxicity, Mutagenicity, Tumorigenicity, Reproductive Effects,
A00617	Skin and Eye Irritation
A00618	Non-toxicity
A00619	Acute Toxicity, Multiple Dose Effects
A00620	Acute Toxicity, Reproductive Effects, Multiple Dose Effects
A00621	Acute Toxicity, Mutagenicity, Multiple Dose Effects
A00622	Acute Toxicity, Skin and Eye Irritation, Multiple Dose Effects
A00623	Skin and Eye Irritation
A00624	Acute Toxicity
A00625	Acute Toxicity, Mutagenicity, Tumorigenicity, Reproductive Effects,
A00626	Non-toxicity
A00627	Non-toxicity
A00628	Acute Toxicity, Mutagenicity, Tumorigenicity, Skin and Eye Irritation,
A00629	Acute Toxicity, Mutagenicity, Reproductive Effects
A00630	Acute Toxicity, Mutagenicity
A00631	Reproductive Effects, Multiple Dose Effects
A00632	Acute Toxicity, Mutagenicity
A00633	Acute Toxicity, Tumorigenicity, Reproductive Effects, Multiple Dose Effects
A00634	Non-toxicity
A00635	Mutagenicity
A00636	Acute Toxicity, Mutagenicity, Tumorigenicity, Skin and Eye Irritation, Multiple Dose Effects
A00637	Mutagenicity
A00638	Acute Toxicity, Mutagenicity, Tumorigenicity, Reproductive Effects,
A00639	Acute Toxicity
A00640	Acute Toxicity
A00641	Acute Toxicity, Mutagenicity, Skin and Eye Irritation, Multiple Dose Effects
A00642	Acute Toxicity, Mutagenicity, Multiple Dose Effects

A00643	Acute Toxicity, Mutagenicity, Multiple Dose Effects
A00644	Acute Toxicity
A00645	Acute Toxicity
A00646	Acute Toxicity
A00647	Skin and Eye Irritation
A00648	Acute Toxicity, Mutagenicity, Tumorigenicity, Reproductive Effects,
A00649	Acute Toxicity, Mutagenicity, Reproductive Effects
A00650	Acute Toxicity
A00651	Acute Toxicity, Mutagenicity, Reproductive Effects, Multiple Dose Effects
A00652	Acute Toxicity, Mutagenicity, Tumorigenicity, Reproductive Effects,
A00653	Acute Toxicity, Mutagenicity, Tumorigenicity, Skin and Eye Irritation,
A00654	Acute Toxicity, Mutagenicity, Tumorigenicity, Skin and Eye Irritation,
A00655	Non-toxicity
A00656	Acute Toxicity, Mutagenicity, Multiple Dose Effects
A00657	Acute Toxicity, Mutagenicity, Multiple Dose Effects
A00658	Acute Toxicity, Skin and Eye Irritation
A00659	Acute Toxicity, Mutagenicity, Skin and Eye Irritation, Reproductive Effects, Multiple Dose Effects
A00660	Acute Toxicity, Mutagenicity, Reproductive Effects, Multiple Dose Effects
A00661	Acute Toxicity, Mutagenicity, Tumorigenicity, Multiple Dose Effects
A00662	Acute Toxicity, Mutagenicity, Tumorigenicity, Reproductive Effects,
A00663	Acute Toxicity, Mutagenicity, Tumorigenicity, Skin and Eye Irritation,
A00664	Acute Toxicity, Mutagenicity, Skin and Eye Irritation
A00665	Acute Toxicity, Reproductive Effects
A00666	Acute Toxicity, Mutagenicity, Tumorigenicity, Reproductive Effects,
A00667	Acute Toxicity, Multiple Dose Effects
A00668	Acute Toxicity, Mutagenicity, Multiple Dose Effects
A00669	Acute Toxicity, Mutagenicity
A00670	Acute Toxicity, Mutagenicity, Reproductive Effects
A00671	Acute Toxicity
A00672	Non-toxicity
A00673	Acute Toxicity
A00674	Acute Toxicity, Mutagenicity, Reproductive Effects, Multiple Dose Effects
A00675	Mutagenicity, Tumorigenicity, Multiple Dose Effects
A00676	Acute Toxicity, Multiple Dose Effects
A00677	Acute Toxicity
A00678	Acute Toxicity
A00679	Acute Toxicity, Mutagenicity, Tumorigenicity, Reproductive Effects,
A00680	Acute Toxicity, Tumorigenicity, Reproductive Effects
A00681	Acute Toxicity, Mutagenicity, Tumorigenicity, Skin and Eye Irritation,
A00682	Acute Toxicity, Multiple Dose Effects
A00683	Acute Toxicity, Reproductive Effects
A00684	Mutagenicity, Tumorigenicity
A00685	Acute Toxicity
A00686	Acute Toxicity, Mutagenicity
A00687	Acute Toxicity, Mutagenicity, Tumorigenicity, Skin and Eye Irritation,

A00688	Acute Toxicity, Mutagenicity, Tumorigenicity, Skin and Eye Irritation,
A00689	Acute Toxicity, Skin and Eye Irritation
A00690	Acute Toxicity, Skin and Eye Irritation
A00691	Acute Toxicity, Mutagenicity, Multiple Dose Effects
A00692	Skin and Eye Irritation, Reproductive Effects
A00693	Acute Toxicity, Reproductive Effects, Multiple Dose Effects
A00694	Acute Toxicity, Mutagenicity, Tumorigenicity, Skin and Eye Irritation,
A00695	Acute Toxicity, Mutagenicity, Multiple Dose Effects
A00696	Acute Toxicity, Mutagenicity, Tumorigenicity, Reproductive Effects,
A00697	Acute Toxicity, Mutagenicity, Skin and Eye Irritation, Reproductive Effects, Multiple Dose Effects
A00698	Acute Toxicity
A00699	Acute Toxicity
A00700	Acute Toxicity, Multiple Dose Effects
A00701	Acute Toxicity, Multiple Dose Effects
A00702	Acute Toxicity
A00703	Acute Toxicity, Multiple Dose Effects
A00704	Mutagenicity, Tumorigenicity
A00705	Acute Toxicity, Mutagenicity, Reproductive Effects, Multiple Dose Effects
A00706	Acute Toxicity, Mutagenicity, Multiple Dose Effects
A00707	Acute Toxicity, Reproductive Effects
A00708	Acute Toxicity
A00709	Acute Toxicity
A00710	Acute Toxicity
A00711	Acute Toxicity, Mutagenicity, Reproductive Effects, Multiple Dose Effects
A00712	Acute Toxicity, Mutagenicity
A00713	Acute Toxicity, Multiple Dose Effects
A00714	Reproductive Effects, Multiple Dose Effects
A00715	Acute Toxicity
A00716	Acute Toxicity, Mutagenicity
A00717	Acute Toxicity, Reproductive Effects, Multiple Dose Effects
A00718	Acute Toxicity, Mutagenicity, Skin and Eye Irritation
A00719	Acute Toxicity, Mutagenicity, Tumorigenicity
A00720	Acute Toxicity, Reproductive Effects, Multiple Dose Effects
A00721	Non-toxicity
A00722	Acute Toxicity, Mutagenicity, Tumorigenicity, Reproductive Effects,
A00723	Acute Toxicity, Mutagenicity, Reproductive Effects, Multiple Dose Effects
A00724	Acute Toxicity, Mutagenicity, Tumorigenicity, Skin and Eye Irritation,
A00725	Acute Toxicity, Mutagenicity, Reproductive Effects, Multiple Dose Effects
A00726	Multiple Dose Effects
A00727	Non-toxicity
A00728	Acute Toxicity, Multiple Dose Effects
A00729	Acute Toxicity
A00730	Acute Toxicity, Skin and Eye Irritation, Multiple Dose Effects
A00731	Acute Toxicity, Mutagenicity, Tumorigenicity, Reproductive Effects,
A00732	Acute Toxicity, Mutagenicity

A00733	Acute Toxicity, Mutagenicity, Multiple Dose Effects
A00734	Acute Toxicity
A00735	Acute Toxicity, Mutagenicity, Tumorigenicity
A00736	Acute Toxicity, Mutagenicity, Skin and Eye Irritation, Multiple Dose Effects
A00737	Acute Toxicity, Reproductive Effects
A00738	Acute Toxicity, Mutagenicity, Multiple Dose Effects
A00739	Acute Toxicity, Multiple Dose Effects
A00740	Acute Toxicity
A00741	Acute Toxicity, Mutagenicity, Reproductive Effects, Multiple Dose Effects
A00742	Acute Toxicity, Reproductive Effects
A00743	Acute Toxicity, Mutagenicity, Tumorigenicity, Skin and Eye Irritation,
A00744	Reproductive Effects
A00745	Acute Toxicity, Reproductive Effects, Multiple Dose Effects
A00746	Acute Toxicity
A00747	Acute Toxicity, Mutagenicity, Tumorigenicity, Skin and Eye Irritation,
A00748	Acute Toxicity, Reproductive Effects, Multiple Dose Effects
A00749	Acute Toxicity, Reproductive Effects
A00750	Acute Toxicity, Reproductive Effects, Multiple Dose Effects
A00751	Acute Toxicity, Mutagenicity, Tumorigenicity, Reproductive Effects,
A00752	Acute Toxicity, Mutagenicity, Tumorigenicity, Skin and Eye Irritation, Multiple Dose Effects
A00753	Acute Toxicity
A00754	Multiple Dose Effects
A00755	Non-toxicity
A00756	Acute Toxicity
A00757	Acute Toxicity
A00758	Acute Toxicity, Multiple Dose Effects
A00759	Acute Toxicity, Reproductive Effects, Multiple Dose Effects
A00760	Acute Toxicity, Reproductive Effects, Multiple Dose Effects
A00761	Acute Toxicity, Reproductive Effects, Multiple Dose Effects
A00762	Acute Toxicity, Multiple Dose Effects
A00763	Acute Toxicity, Reproductive Effects
A00764	Acute Toxicity, Reproductive Effects, Multiple Dose Effects
A00765	Acute Toxicity, Mutagenicity, Tumorigenicity, Reproductive Effects,
A00766	Acute Toxicity, Skin and Eye Irritation, Multiple Dose Effects
A00767	Acute Toxicity, Reproductive Effects
A00768	Acute Toxicity
A00769	Acute Toxicity, Multiple Dose Effects
A00770	Acute Toxicity, Mutagenicity, Tumorigenicity, Skin and Eye Irritation,
A00771	Acute Toxicity, Mutagenicity, Tumorigenicity, Reproductive Effects,
A00772	Acute Toxicity
A00773	Acute Toxicity, Reproductive Effects, Multiple Dose Effects
A00774	Acute Toxicity
A00775	Acute Toxicity, Mutagenicity, Tumorigenicity, Reproductive Effects,
A00776	Acute Toxicity, Reproductive Effects, Multiple Dose Effects
A00777	Acute Toxicity, Multiple Dose Effects

A00778	Acute Toxicity, Mutagenicity, Tumorigenicity, Skin and Eye Irritation,
A00779	Non-toxicity
A00780	Acute Toxicity, Multiple Dose Effects
A00781	Acute Toxicity, Mutagenicity, Skin and Eye Irritation, Reproductive Effects, Multiple Dose Effects
A00782	Acute Toxicity, Reproductive Effects, Multiple Dose Effects
A00783	Acute Toxicity, Reproductive Effects, Multiple Dose Effects
A00784	Mutagenicity, Tumorigenicity, Multiple Dose Effects
A00785	Acute Toxicity, Mutagenicity, Reproductive Effects, Multiple Dose Effects
A00786	Acute Toxicity, Mutagenicity, Tumorigenicity, Skin and Eye Irritation,
A00787	Acute Toxicity, Reproductive Effects, Multiple Dose Effects
A00788	Acute Toxicity, Mutagenicity, Reproductive Effects, Multiple Dose Effects
A00789	Acute Toxicity, Mutagenicity, Multiple Dose Effects
A00790	Acute Toxicity, Reproductive Effects, Multiple Dose Effects
A00791	Acute Toxicity, Mutagenicity, Tumorigenicity, Skin and Eye Irritation,
A00792	Acute Toxicity, Reproductive Effects, Multiple Dose Effects
A00793	Acute Toxicity, Multiple Dose Effects
A00794	Acute Toxicity, Mutagenicity, Tumorigenicity, Reproductive Effects,
A00795	Acute Toxicity, Reproductive Effects, Multiple Dose Effects
A00796	Acute Toxicity, Reproductive Effects, Multiple Dose Effects
A00797	Acute Toxicity
A00798	Non-toxicity
A00799	Acute Toxicity, Multiple Dose Effects
A00800	Non-toxicity
A00801	Acute Toxicity, Multiple Dose Effects
A00802	Mutagenicity
A00803	Non-toxicity
A00804	Acute Toxicity
A00805	Acute Toxicity, Mutagenicity, Tumorigenicity, Skin and Eye Irritation,
A00806	Acute Toxicity, Mutagenicity, Reproductive Effects
A00807	Acute Toxicity, Multiple Dose Effects
A00808	Acute Toxicity
A00809	Acute Toxicity, Mutagenicity, Tumorigenicity, Reproductive Effects,
A00810	Acute Toxicity
A00811	Acute Toxicity
A00812	Non-toxicity
A00813	Acute Toxicity, Tumorigenicity, Reproductive Effects, Multiple Dose Effects
A00814	Non-toxicity
A00815	Acute Toxicity, Mutagenicity, Reproductive Effects
A00816	Acute Toxicity, Multiple Dose Effects
A00817	Acute Toxicity, Reproductive Effects, Multiple Dose Effects
A00818	Non-toxicity
A00819	Acute Toxicity, Mutagenicity, Multiple Dose Effects
A00820	Acute Toxicity, Mutagenicity, Tumorigenicity, Skin and Eye Irritation,
A00821	Acute Toxicity, Mutagenicity, Reproductive Effects, Multiple Dose Effects
A00822	Acute Toxicity, Mutagenicity, Reproductive Effects, Multiple Dose Effects

A00823	Acute Toxicity, Reproductive Effects
A00824	Non-toxicity
A00825	Acute Toxicity, Mutagenicity, Tumorigenicity, Reproductive Effects,
A00826	Acute Toxicity, Mutagenicity, Reproductive Effects, Multiple Dose Effects
A00827	Non-toxicity
A00828	Acute Toxicity
A00829	Acute Toxicity, Multiple Dose Effects
A00830	Non-toxicity
A00831	Non-toxicity
A00832	Acute Toxicity
A00833	Acute Toxicity
A00834	Acute Toxicity
A00835	Acute Toxicity
A00836	Acute Toxicity
A00837	Acute Toxicity, Multiple Dose Effects
A00838	Acute Toxicity, Mutagenicity, Multiple Dose Effects
A00839	Acute Toxicity, Mutagenicity, Tumorigenicity, Reproductive Effects,
A00840	Acute Toxicity, Mutagenicity, Skin and Eye Irritation
A00841	Acute Toxicity, Mutagenicity, Skin and Eye Irritation, Reproductive Effects, Multiple Dose Effects
A00842	Acute Toxicity, Reproductive Effects
A00843	Acute Toxicity
A00844	Acute Toxicity
A00845	Acute Toxicity
A00846	Acute Toxicity, Mutagenicity, Tumorigenicity, Skin and Eye Irritation,
A00847	Acute Toxicity, Multiple Dose Effects
A00848	Acute Toxicity
A00849	Acute Toxicity, Mutagenicity, Multiple Dose Effects
A00850	Acute Toxicity, Mutagenicity, Tumorigenicity, Skin and Eye Irritation,
A00851	Mutagenicity
A00852	Acute Toxicity, Tumorigenicity
A00853	Mutagenicity
A00854	Acute Toxicity, Multiple Dose Effects
A00855	Acute Toxicity, Mutagenicity, Tumorigenicity, Reproductive Effects
A00856	Acute Toxicity, Mutagenicity, Tumorigenicity, Skin and Eye Irritation,
A00857	Acute Toxicity
A00858	Acute Toxicity, Mutagenicity, Tumorigenicity, Skin and Eye Irritation,
A00859	Acute Toxicity
A00860	Acute Toxicity, Multiple Dose Effects
A00861	Acute Toxicity
A00862	Acute Toxicity, Multiple Dose Effects
A00863	Non-toxicity
A00864	Acute Toxicity, Mutagenicity, Tumorigenicity, Skin and Eye Irritation,
A00865	Acute Toxicity, Reproductive Effects
A00866	Acute Toxicity
A00867	Acute Toxicity, Reproductive Effects, Multiple Dose Effects

A00868	Acute Toxicity, Mutagenicity, Tumorigenicity, Skin and Eye Irritation,
A00869	Acute Toxicity, Skin and Eye Irritation, Reproductive Effects, Multiple Dose
A00870	Acute Toxicity, Mutagenicity, Reproductive Effects, Multiple Dose Effects
A00871	Acute Toxicity, Mutagenicity, Skin and Eye Irritation, Reproductive Effects, Multiple Dose Effects
A00872	Acute Toxicity
A00873	Acute Toxicity
A00874	Acute Toxicity
A00875	Acute Toxicity, Skin and Eye Irritation, Multiple Dose Effects
A00876	Acute Toxicity
A00877	Acute Toxicity, Mutagenicity, Tumorigenicity, Reproductive Effects,
A00878	Acute Toxicity, Mutagenicity, Reproductive Effects, Multiple Dose Effects
A00879	Acute Toxicity, Mutagenicity, Reproductive Effects, Multiple Dose Effects
A00880	Acute Toxicity, Reproductive Effects, Multiple Dose Effects
A00881	Acute Toxicity, Reproductive Effects, Multiple Dose Effects
A00882	Acute Toxicity, Mutagenicity, Tumorigenicity, Reproductive Effects,
A00883	Acute Toxicity, Mutagenicity, Tumorigenicity, Skin and Eye Irritation,
A00884	Acute Toxicity, Multiple Dose Effects
A00885	Acute Toxicity
A00886	Acute Toxicity, Mutagenicity, Multiple Dose Effects
A00887	Acute Toxicity, Mutagenicity, Tumorigenicity, Multiple Dose Effects
A00888	Acute Toxicity, Mutagenicity
A00889	Acute Toxicity, Multiple Dose Effects
A00890	Acute Toxicity, Multiple Dose Effects
A00891	Acute Toxicity, Mutagenicity, Reproductive Effects, Multiple Dose Effects
A00892	Acute Toxicity, Multiple Dose Effects
A00893	Acute Toxicity
A00894	Acute Toxicity
A00895	Acute Toxicity
A00896	Acute Toxicity
A00897	Acute Toxicity, Reproductive Effects, Multiple Dose Effects
A00898	Acute Toxicity, Reproductive Effects, Multiple Dose Effects
A00899	Acute Toxicity, Mutagenicity, Multiple Dose Effects
A00900	Acute Toxicity, Mutagenicity
A00901	Acute Toxicity, Mutagenicity
A00902	Acute Toxicity, Mutagenicity, Tumorigenicity, Reproductive Effects,
A00903	Non-toxicity
A00904	Acute Toxicity
A00905	Acute Toxicity, Mutagenicity, Tumorigenicity
A00906	Acute Toxicity, Mutagenicity, Reproductive Effects, Multiple Dose Effects
A00907	Acute Toxicity, Skin and Eye Irritation
A00908	Multiple Dose Effects
A00909	Acute Toxicity, Mutagenicity, Tumorigenicity, Reproductive Effects,
A00910	Acute Toxicity
A00911	Acute Toxicity, Mutagenicity, Tumorigenicity, Skin and Eye Irritation,
A00912	Acute Toxicity

A00913	Acute Toxicity
A00914	Acute Toxicity, Mutagenicity, Tumorigenicity, Skin and Eye Irritation,
A00915	Acute Toxicity, Mutagenicity
A00916	Acute Toxicity, Mutagenicity, Tumorigenicity, Skin and Eye Irritation,
A00917	Acute Toxicity, Skin and Eye Irritation
A00918	Acute Toxicity, Skin and Eye Irritation, Reproductive Effects
A00919	Acute Toxicity, Multiple Dose Effects
A00920	Acute Toxicity, Skin and Eye Irritation, Reproductive Effects, Multiple Dose
A00921	Acute Toxicity, Mutagenicity, Tumorigenicity, Skin and Eye Irritation
A00922	Acute Toxicity, Reproductive Effects, Multiple Dose Effects
A00923	Acute Toxicity
A00924	Non-toxicity
A00925	Acute Toxicity, Multiple Dose Effects
A00926	Acute Toxicity, Mutagenicity, Tumorigenicity, Skin and Eye Irritation,
A00927	Acute Toxicity, Reproductive Effects, Multiple Dose Effects
A00928	Acute Toxicity, Multiple Dose Effects
A00929	Acute Toxicity, Mutagenicity, Reproductive Effects
A00930	Non-toxicity
A00931	Acute Toxicity, Mutagenicity, Tumorigenicity, Multiple Dose Effects
A00932	Non-toxicity
A00933	Acute Toxicity
A00934	Acute Toxicity, Multiple Dose Effects
A00935	Acute Toxicity, Reproductive Effects, Multiple Dose Effects
A00936	Acute Toxicity, Mutagenicity, Skin and Eye Irritation, Reproductive Effects, Multiple Dose Effects
A00937	Mutagenicity, Multiple Dose Effects
A00938	Acute Toxicity
A00939	Acute Toxicity, Mutagenicity, Tumorigenicity, Skin and Eye Irritation, Multiple Dose Effects
A00940	Acute Toxicity
A00941	Acute Toxicity, Reproductive Effects, Multiple Dose Effects
A00942	Tumorigenicity, Reproductive Effects, Multiple Dose Effects
A00943	Acute Toxicity
A00944	Acute Toxicity
A00945	Acute Toxicity
A00946	Acute Toxicity, Multiple Dose Effects
A00947	Acute Toxicity
A00948	Acute Toxicity, Reproductive Effects, Multiple Dose Effects
A00949	Acute Toxicity
A00950	Acute Toxicity, Mutagenicity, Skin and Eye Irritation, Reproductive Effects, Multiple Dose Effects
A00951	Acute Toxicity, Mutagenicity, Tumorigenicity, Reproductive Effects,
A00952	Acute Toxicity, Reproductive Effects, Multiple Dose Effects
A00953	Acute Toxicity, Reproductive Effects, Multiple Dose Effects
A00954	Non-toxicity
A00955	Acute Toxicity, Mutagenicity, Reproductive Effects, Multiple Dose Effects

A00956	Multiple Dose Effects
A00957	Acute Toxicity, Multiple Dose Effects
A00958	Acute Toxicity
A00959	Acute Toxicity
A00960	Mutagenicity
A00961	Skin and Eye Irritation
A00962	Acute Toxicity, Mutagenicity, Tumorigenicity, Skin and Eye Irritation,
A00963	Acute Toxicity, Reproductive Effects, Multiple Dose Effects
A00964	Multiple Dose Effects
A00965	Non-toxicity
A00966	Acute Toxicity, Mutagenicity, Reproductive Effects, Multiple Dose Effects
A00967	Acute Toxicity, Mutagenicity, Tumorigenicity, Skin and Eye Irritation,
A00968	Non-toxicity
A00969	Acute Toxicity, Multiple Dose Effects
A00970	Non-toxicity
A00971	Acute Toxicity, Mutagenicity, Tumorigenicity, Skin and Eye Irritation,
A00972	Acute Toxicity, Reproductive Effects, Multiple Dose Effects
A00973	Acute Toxicity, Multiple Dose Effects
A00974	Acute Toxicity, Mutagenicity, Reproductive Effects, Multiple Dose Effects
A00975	Acute Toxicity, Skin and Eye Irritation, Reproductive Effects, Multiple Dose
A00976	Acute Toxicity
A00977	Non-toxicity
A00978	Acute Toxicity, Multiple Dose Effects
A00979	Acute Toxicity, Mutagenicity, Tumorigenicity, Reproductive Effects
A00980	Non-toxicity
A00981	Acute Toxicity, Mutagenicity, Tumorigenicity, Skin and Eye Irritation,
A00982	Non-toxicity
A00983	Acute Toxicity, Reproductive Effects, Multiple Dose Effects
A00984	Acute Toxicity
A00985	Acute Toxicity, Mutagenicity, Reproductive Effects
A00986	Acute Toxicity, Skin and Eye Irritation, Reproductive Effects
A00987	Acute Toxicity, Mutagenicity, Skin and Eye Irritation, Reproductive Effects, Multiple Dose Effects
A00988	Acute Toxicity
A00989	Acute Toxicity, Mutagenicity, Multiple Dose Effects
A00990	Acute Toxicity, Reproductive Effects
A00991	Acute Toxicity, Multiple Dose Effects
A00992	Acute Toxicity, Mutagenicity, Skin and Eye Irritation, Reproductive Effects
A00993	Acute Toxicity, Mutagenicity, Skin and Eye Irritation, Multiple Dose Effects
A00994	Acute Toxicity, Mutagenicity, Reproductive Effects, Multiple Dose Effects
A00995	Acute Toxicity, Mutagenicity, Reproductive Effects, Multiple Dose Effects
A00996	Non-toxicity
A00997	Acute Toxicity, Mutagenicity, Multiple Dose Effects
A00998	Acute Toxicity
A00999	Acute Toxicity, Mutagenicity, Tumorigenicity, Skin and Eye Irritation, Multiple Dose Effects

A01000	Acute Toxicity
A01001	Acute Toxicity, Multiple Dose Effects
A01002	Acute Toxicity
A01003	Acute Toxicity, Mutagenicity
A01004	Acute Toxicity, Mutagenicity, Tumorigenicity, Skin and Eye Irritation,
A01005	Acute Toxicity, Mutagenicity, Tumorigenicity, Reproductive Effects,
A01006	Acute Toxicity, Mutagenicity, Skin and Eye Irritation, Reproductive Effects, Multiple Dose Effects
A01007	Acute Toxicity, Mutagenicity, Multiple Dose Effects
A01008	Acute Toxicity, Mutagenicity, Tumorigenicity, Skin and Eye Irritation,
A01009	Acute Toxicity, Mutagenicity, Skin and Eye Irritation, Reproductive Effects, Multiple Dose Effects
A01010	Acute Toxicity
A01011	Acute Toxicity, Mutagenicity, Tumorigenicity, Reproductive Effects,
A01012	Acute Toxicity, Mutagenicity, Reproductive Effects, Multiple Dose Effects
A01013	Acute Toxicity, Multiple Dose Effects
A01014	Acute Toxicity, Reproductive Effects, Multiple Dose Effects
A01015	Acute Toxicity, Reproductive Effects, Multiple Dose Effects
A01016	Acute Toxicity
A01017	Acute Toxicity, Mutagenicity, Reproductive Effects, Multiple Dose Effects
A01018	Acute Toxicity, Mutagenicity, Skin and Eye Irritation, Reproductive Effects, Multiple Dose Effects
A01019	Acute Toxicity, Reproductive Effects, Multiple Dose Effects
A01020	Acute Toxicity, Mutagenicity, Skin and Eye Irritation, Reproductive Effects, Multiple Dose Effects
A01021	Acute Toxicity, Mutagenicity, Tumorigenicity, Skin and Eye Irritation,
A01022	Acute Toxicity, Mutagenicity, Reproductive Effects, Multiple Dose Effects
A01023	Acute Toxicity
A01024	Acute Toxicity
A01025	Acute Toxicity, Reproductive Effects, Multiple Dose Effects
A01026	Acute Toxicity, Mutagenicity, Tumorigenicity, Skin and Eye Irritation,
A01027	Acute Toxicity, Mutagenicity, Reproductive Effects, Multiple Dose Effects
A01028	Non-toxicity
A01029	Acute Toxicity, Mutagenicity, Multiple Dose Effects
A01030	Acute Toxicity
A01031	Acute Toxicity
A01032	Acute Toxicity, Skin and Eye Irritation, Reproductive Effects, Multiple Dose
A01033	Acute Toxicity
A01034	Acute Toxicity, Multiple Dose Effects
A01035	Acute Toxicity, Mutagenicity, Tumorigenicity, Reproductive Effects,
A01036	Acute Toxicity, Mutagenicity, Multiple Dose Effects
A01037	Acute Toxicity, Multiple Dose Effects
A01038	Acute Toxicity, Multiple Dose Effects
A01039	Acute Toxicity, Mutagenicity, Reproductive Effects, Multiple Dose Effects
A01040	Acute Toxicity, Multiple Dose Effects
A01041	Multiple Dose Effects

A01042	Acute Toxicity, Tumorigenicity
A01043	Acute Toxicity, Mutagenicity, Tumorigenicity, Multiple Dose Effects
A01044	Multiple Dose Effects
A01045	Acute Toxicity, Multiple Dose Effects
A01046	Acute Toxicity, Mutagenicity, Tumorigenicity, Skin and Eye Irritation,
A01047	Acute Toxicity
A01048	Mutagenicity
A01049	Acute Toxicity
A01050	Acute Toxicity
A01051	Mutagenicity
A01052	Acute Toxicity, Mutagenicity
A01053	Acute Toxicity
A01054	Acute Toxicity, Reproductive Effects, Multiple Dose Effects
A01055	Acute Toxicity
A01056	Acute Toxicity, Reproductive Effects, Multiple Dose Effects
A01057	Acute Toxicity, Multiple Dose Effects
A01058	Acute Toxicity, Mutagenicity, Tumorigenicity, Reproductive Effects,
A01059	Mutagenicity
A01060	Mutagenicity, Tumorigenicity, Reproductive Effects
A01061	Acute Toxicity
A01062	Acute Toxicity, Reproductive Effects, Multiple Dose Effects
A01063	Acute Toxicity, Mutagenicity, Skin and Eye Irritation, Multiple Dose Effects
A01064	Acute Toxicity, Mutagenicity, Tumorigenicity, Reproductive Effects,
A01065	Acute Toxicity
A01066	Acute Toxicity
A01067	Acute Toxicity, Multiple Dose Effects
A01068	Acute Toxicity
A01069	Acute Toxicity, Tumorigenicity, Multiple Dose Effects
A01070	Non-toxicity
A01071	Acute Toxicity, Reproductive Effects, Multiple Dose Effects
A01072	Acute Toxicity, Mutagenicity, Tumorigenicity, Reproductive Effects,
A01073	Acute Toxicity, Multiple Dose Effects
A01074	Acute Toxicity, Mutagenicity, Reproductive Effects, Multiple Dose Effects
A01075	Acute Toxicity, Mutagenicity, Skin and Eye Irritation, Reproductive Effects, Multiple Dose Effects
A01076	Acute Toxicity
A01077	Acute Toxicity, Mutagenicity, Skin and Eye Irritation, Reproductive Effects
A01078	Acute Toxicity, Reproductive Effects
A01079	Acute Toxicity, Reproductive Effects, Multiple Dose Effects
A01080	Acute Toxicity, Mutagenicity, Tumorigenicity, Skin and Eye Irritation,
A01081	Acute Toxicity, Mutagenicity, Reproductive Effects
A01082	Acute Toxicity, Mutagenicity, Reproductive Effects, Multiple Dose Effects
A01083	Acute Toxicity
A01084	Acute Toxicity, Multiple Dose Effects
A01085	Acute Toxicity
A01086	Acute Toxicity, Mutagenicity, Reproductive Effects, Multiple Dose Effects

A01087	Acute Toxicity, Reproductive Effects, Multiple Dose Effects
A01088	Acute Toxicity, Mutagenicity, Tumorigenicity, Skin and Eye Irritation,
A01089	Acute Toxicity, Skin and Eye Irritation
A01090	Acute Toxicity, Mutagenicity, Reproductive Effects, Multiple Dose Effects
A01091	Acute Toxicity, Mutagenicity, Tumorigenicity, Skin and Eye Irritation, Multiple Dose Effects
A01092	Acute Toxicity, Multiple Dose Effects
A01093	Acute Toxicity, Mutagenicity, Skin and Eye Irritation, Reproductive Effects, Multiple Dose Effects
A01094	Acute Toxicity
A01095	Acute Toxicity
A01096	Acute Toxicity
A01097	Acute Toxicity, Mutagenicity, Tumorigenicity, Multiple Dose Effects
A01098	Acute Toxicity
A01099	Acute Toxicity, Mutagenicity, Skin and Eye Irritation, Reproductive Effects, Multiple Dose Effects
A01100	Non-toxicity
A01101	Acute Toxicity, Multiple Dose Effects
A01102	Acute Toxicity, Multiple Dose Effects
A01103	Acute Toxicity
A01104	Acute Toxicity, Skin and Eye Irritation, Multiple Dose Effects
A01105	Acute Toxicity, Mutagenicity, Multiple Dose Effects
A01106	Acute Toxicity, Reproductive Effects, Multiple Dose Effects
A01107	Acute Toxicity, Reproductive Effects, Multiple Dose Effects
A01108	Acute Toxicity, Mutagenicity, Tumorigenicity, Reproductive Effects,
A01109	Acute Toxicity, Mutagenicity, Reproductive Effects, Multiple Dose Effects
A01110	Acute Toxicity
A01111	Acute Toxicity, Mutagenicity
A01112	Acute Toxicity, Mutagenicity, Tumorigenicity, Reproductive Effects
A01113	Acute Toxicity, Mutagenicity, Reproductive Effects, Multiple Dose Effects
A01114	Acute Toxicity, Mutagenicity, Tumorigenicity, Multiple Dose Effects
A01115	Acute Toxicity, Multiple Dose Effects
A01116	Acute Toxicity, Mutagenicity
A01117	Acute Toxicity, Mutagenicity, Skin and Eye Irritation, Reproductive Effects, Multiple Dose Effects
A01118	Acute Toxicity, Skin and Eye Irritation
A01119	Acute Toxicity, Multiple Dose Effects
A01120	Acute Toxicity, Reproductive Effects
A01121	Acute Toxicity, Multiple Dose Effects
A01122	Acute Toxicity, Mutagenicity, Skin and Eye Irritation, Reproductive Effects
A01123	Non-toxicity
A01124	Acute Toxicity, Mutagenicity, Skin and Eye Irritation, Multiple Dose Effects
A01125	Acute Toxicity, Mutagenicity, Tumorigenicity
A01126	Acute Toxicity
A01127	Acute Toxicity, Reproductive Effects, Multiple Dose Effects
A01128	Acute Toxicity, Mutagenicity

A01129	Acute Toxicity
A01130	Acute Toxicity
A01131	Acute Toxicity, Mutagenicity
A01132	Acute Toxicity
A01133	Acute Toxicity
A01134	Acute Toxicity
A01135	Non-toxicity
A01136	Acute Toxicity, Mutagenicity, Skin and Eye Irritation
A01137	Acute Toxicity, Mutagenicity, Tumorigenicity, Skin and Eye Irritation,
A01138	Acute Toxicity, Mutagenicity, Tumorigenicity, Skin and Eye Irritation,
A01139	Mutagenicity, Reproductive Effects
A01140	Acute Toxicity, Tumorigenicity, Reproductive Effects, Multiple Dose Effects
A01141	Acute Toxicity
A01142	Acute Toxicity, Mutagenicity, Tumorigenicity, Reproductive Effects,
A01143	Acute Toxicity, Mutagenicity
A01144	Acute Toxicity, Reproductive Effects, Multiple Dose Effects
A01145	Acute Toxicity, Mutagenicity, Multiple Dose Effects
A01146	Non-toxicity
A01147	Acute Toxicity
A01148	Acute Toxicity, Mutagenicity
A01149	Acute Toxicity, Mutagenicity, Multiple Dose Effects
A01150	Acute Toxicity, Tumorigenicity, Reproductive Effects
A01151	Acute Toxicity, Mutagenicity, Tumorigenicity, Reproductive Effects,
A01152	Acute Toxicity, Mutagenicity
A01153	Acute Toxicity, Multiple Dose Effects
A01154	Acute Toxicity, Mutagenicity, Skin and Eye Irritation, Reproductive Effects, Multiple Dose Effects
A01155	Acute Toxicity, Reproductive Effects
A01156	Acute Toxicity, Mutagenicity, Tumorigenicity, Skin and Eye Irritation,
A01157	Acute Toxicity, Mutagenicity, Tumorigenicity
A01158	Acute Toxicity, Multiple Dose Effects
A01159	Acute Toxicity, Skin and Eye Irritation
A01160	Acute Toxicity, Multiple Dose Effects
A01161	Acute Toxicity, Mutagenicity, Tumorigenicity, Skin and Eye Irritation,
A01162	Acute Toxicity, Mutagenicity, Tumorigenicity, Reproductive Effects,
A01163	Acute Toxicity, Skin and Eye Irritation
A01164	Acute Toxicity, Mutagenicity, Skin and Eye Irritation
A01165	Acute Toxicity, Mutagenicity, Multiple Dose Effects
A01166	Acute Toxicity, Mutagenicity, Tumorigenicity, Reproductive Effects
A01167	Acute Toxicity
A01168	Acute Toxicity, Mutagenicity, Multiple Dose Effects
A01169	Acute Toxicity, Mutagenicity, Tumorigenicity, Skin and Eye Irritation,
A01170	Acute Toxicity
A01171	Acute Toxicity
A01172	Acute Toxicity
A01173	Mutagenicity

A01174	Acute Toxicity, Mutagenicity, Skin and Eye Irritation, Reproductive Effects
A01175	Acute Toxicity, Mutagenicity, Reproductive Effects, Multiple Dose Effects
A01176	Acute Toxicity
A01177	Acute Toxicity, Skin and Eye Irritation, Multiple Dose Effects
A01178	Acute Toxicity, Multiple Dose Effects
A01179	Acute Toxicity, Multiple Dose Effects
A01180	Acute Toxicity
A01181	Acute Toxicity
A01182	Acute Toxicity
A01183	Mutagenicity
A01184	Acute Toxicity, Mutagenicity, Skin and Eye Irritation, Reproductive Effects, Multiple Dose Effects
A01185	Acute Toxicity, Tumorigenicity, Multiple Dose Effects
A01186	Acute Toxicity, Mutagenicity, Reproductive Effects
A01187	Acute Toxicity, Mutagenicity, Skin and Eye Irritation, Reproductive Effects, Multiple Dose Effects
A01188	Acute Toxicity
A01189	Acute Toxicity, Mutagenicity, Tumorigenicity, Skin and Eye Irritation, Multiple Dose Effects
A01190	Acute Toxicity
A01191	Acute Toxicity, Mutagenicity, Reproductive Effects, Multiple Dose Effects
A01192	Acute Toxicity, Reproductive Effects, Multiple Dose Effects
A01193	Acute Toxicity
A01194	Acute Toxicity
A01195	Acute Toxicity, Reproductive Effects, Multiple Dose Effects
A01196	Acute Toxicity, Multiple Dose Effects
A01197	Acute Toxicity, Multiple Dose Effects
A01198	Acute Toxicity, Skin and Eye Irritation
A01199	Non-toxicity
A01200	Acute Toxicity, Mutagenicity, Multiple Dose Effects
A01201	Acute Toxicity, Mutagenicity, Skin and Eye Irritation, Reproductive Effects, Multiple Dose Effects
A01202	Acute Toxicity, Skin and Eye Irritation
A01203	Acute Toxicity, Reproductive Effects, Multiple Dose Effects
A01204	Acute Toxicity, Tumorigenicity, Reproductive Effects, Multiple Dose Effects
A01205	Acute Toxicity, Mutagenicity, Tumorigenicity, Multiple Dose Effects
A01206	Acute Toxicity, Reproductive Effects, Multiple Dose Effects
A01207	Acute Toxicity
A01208	Acute Toxicity, Mutagenicity, Skin and Eye Irritation
A01209	Acute Toxicity, Mutagenicity, Multiple Dose Effects
A01210	Acute Toxicity, Mutagenicity, Tumorigenicity, Skin and Eye Irritation,
A01211	Acute Toxicity, Tumorigenicity, Reproductive Effects, Multiple Dose Effects
A01212	Acute Toxicity, Mutagenicity, Tumorigenicity, Multiple Dose Effects
A01213	Acute Toxicity, Tumorigenicity, Multiple Dose Effects
A01214	Acute Toxicity, Mutagenicity, Multiple Dose Effects
A01215	Acute Toxicity, Mutagenicity, Skin and Eye Irritation

A01216	Acute Toxicity, Mutagenicity, Tumorigenicity, Reproductive Effects
A01217	Acute Toxicity, Skin and Eye Irritation, Reproductive Effects, Multiple Dose
A01218	Acute Toxicity
A01219	Acute Toxicity, Reproductive Effects, Multiple Dose Effects
A01220	Acute Toxicity, Mutagenicity, Skin and Eye Irritation, Reproductive Effects, Multiple Dose Effects
A01221	Acute Toxicity, Mutagenicity, Skin and Eye Irritation, Reproductive Effects
A01222	Acute Toxicity, Mutagenicity, Skin and Eye Irritation, Multiple Dose Effects
A01223	Acute Toxicity, Mutagenicity, Skin and Eye Irritation, Reproductive Effects, Multiple Dose Effects
A01224	Acute Toxicity, Mutagenicity, Tumorigenicity
A01225	Acute Toxicity, Mutagenicity, Multiple Dose Effects
A01226	Acute Toxicity, Mutagenicity, Multiple Dose Effects
A01227	Acute Toxicity, Mutagenicity, Multiple Dose Effects
A01228	Acute Toxicity, Skin and Eye Irritation
A01229	Acute Toxicity
A01230	Acute Toxicity, Mutagenicity, Tumorigenicity, Reproductive Effects,
A01231	Acute Toxicity, Multiple Dose Effects
A01232	Acute Toxicity, Mutagenicity, Reproductive Effects, Multiple Dose Effects
A01233	Acute Toxicity, Mutagenicity, Tumorigenicity, Reproductive Effects,
A01234	Acute Toxicity, Mutagenicity, Tumorigenicity, Skin and Eye Irritation,
A01235	Acute Toxicity, Mutagenicity, Tumorigenicity, Skin and Eye Irritation,
A01236	Acute Toxicity
A01237	Acute Toxicity
A01238	Acute Toxicity, Multiple Dose Effects
A01239	Acute Toxicity
A01240	Acute Toxicity
A01241	Acute Toxicity, Multiple Dose Effects
A01242	Acute Toxicity, Reproductive Effects, Multiple Dose Effects
A01243	Acute Toxicity, Mutagenicity, Skin and Eye Irritation, Reproductive Effects
A01244	Acute Toxicity, Mutagenicity, Tumorigenicity, Reproductive Effects,
A01245	Acute Toxicity
A01246	Acute Toxicity, Reproductive Effects, Multiple Dose Effects
A01247	Acute Toxicity, Multiple Dose Effects
A01248	Acute Toxicity, Mutagenicity
A01249	Acute Toxicity
A01250	Acute Toxicity
A01251	Acute Toxicity
A01252	Mutagenicity, Tumorigenicity, Multiple Dose Effects
A01253	Acute Toxicity
A01254	Acute Toxicity, Mutagenicity, Tumorigenicity, Reproductive Effects
A01255	Acute Toxicity, Reproductive Effects
A01256	Acute Toxicity, Mutagenicity, Multiple Dose Effects
A01257	Acute Toxicity, Mutagenicity, Tumorigenicity, Skin and Eye Irritation, Multiple Dose Effects
A01258	Acute Toxicity, Skin and Eye Irritation

A01259	Acute Toxicity
A01260	Acute Toxicity
A01261	Acute Toxicity, Multiple Dose Effects
A01262	Non-toxicity
A01263	Acute Toxicity, Reproductive Effects, Multiple Dose Effects
A01264	Acute Toxicity, Multiple Dose Effects
A01265	Acute Toxicity
A01266	Acute Toxicity, Multiple Dose Effects
A01267	Acute Toxicity, Tumorigenicity, Multiple Dose Effects
A01268	Acute Toxicity, Reproductive Effects, Multiple Dose Effects
A01269	Acute Toxicity, Mutagenicity
A01270	Acute Toxicity, Multiple Dose Effects
A01271	Acute Toxicity, Multiple Dose Effects
A01272	Acute Toxicity, Mutagenicity, Skin and Eye Irritation, Reproductive Effects, Multiple Dose Effects
A01273	Acute Toxicity
A01274	Acute Toxicity
A01275	Acute Toxicity, Mutagenicity, Tumorigenicity, Skin and Eye Irritation,
A01276	Acute Toxicity, Mutagenicity, Tumorigenicity, Skin and Eye Irritation,
A01277	Skin and Eye Irritation, Reproductive Effects
A01278	Acute Toxicity, Mutagenicity, Skin and Eye Irritation, Reproductive Effects, Multiple Dose Effects
A01279	Acute Toxicity, Skin and Eye Irritation, Multiple Dose Effects
A01280	Acute Toxicity, Mutagenicity
A01281	Acute Toxicity, Reproductive Effects, Multiple Dose Effects
A01282	Acute Toxicity
A01283	Tumorigenicity
A01284	Acute Toxicity
A01285	Acute Toxicity
A01286	Acute Toxicity, Mutagenicity, Skin and Eye Irritation, Multiple Dose Effects
A01287	Acute Toxicity
A01288	Acute Toxicity, Reproductive Effects, Multiple Dose Effects
A01289	Acute Toxicity, Mutagenicity, Tumorigenicity, Reproductive Effects,
A01290	Acute Toxicity, Mutagenicity, Tumorigenicity, Skin and Eye Irritation
A01291	Acute Toxicity, Mutagenicity, Reproductive Effects
A01292	Acute Toxicity
A01293	Acute Toxicity, Mutagenicity, Reproductive Effects, Multiple Dose Effects
A01294	Acute Toxicity, Multiple Dose Effects
A01295	Non-toxicity
A01296	Acute Toxicity, Skin and Eye Irritation, Reproductive Effects, Multiple Dose
A01297	Acute Toxicity, Mutagenicity, Tumorigenicity, Skin and Eye Irritation,
A01298	Acute Toxicity, Multiple Dose Effects
A01299	Acute Toxicity, Mutagenicity
A01300	Acute Toxicity
A01301	Acute Toxicity, Reproductive Effects
A01302	Acute Toxicity, Multiple Dose Effects

A01303	Non-toxicity
A01304	Acute Toxicity, Mutagenicity, Reproductive Effects, Multiple Dose Effects
A01305	Mutagenicity
A01306	Acute Toxicity, Reproductive Effects
A01307	Acute Toxicity, Mutagenicity, Tumorigenicity, Skin and Eye Irritation, Multiple Dose Effects
A01308	Acute Toxicity, Mutagenicity, Multiple Dose Effects
A01309	Reproductive Effects, Multiple Dose Effects
A01310	Mutagenicity
A01311	Acute Toxicity
A01312	Acute Toxicity
A01313	Skin and Eye Irritation
A01314	Acute Toxicity
A01315	Acute Toxicity, Mutagenicity, Reproductive Effects, Multiple Dose Effects
A01316	Non-toxicity
A01317	Acute Toxicity, Tumorigenicity, Reproductive Effects, Multiple Dose Effects
A01318	Acute Toxicity, Reproductive Effects, Multiple Dose Effects
A01319	Acute Toxicity
A01320	Acute Toxicity, Mutagenicity, Tumorigenicity, Skin and Eye Irritation,
A01321	Acute Toxicity, Mutagenicity, Tumorigenicity, Skin and Eye Irritation
A01322	Acute Toxicity, Mutagenicity, Tumorigenicity, Skin and Eye Irritation, Multiple Dose Effects
A01323	Acute Toxicity, Reproductive Effects
A01324	Acute Toxicity, Reproductive Effects
A01325	Acute Toxicity, Mutagenicity, Tumorigenicity, Skin and Eye Irritation,
A01326	Acute Toxicity, Mutagenicity
A01327	Acute Toxicity, Mutagenicity, Tumorigenicity, Skin and Eye Irritation,
A01328	Acute Toxicity, Mutagenicity, Tumorigenicity, Skin and Eye Irritation,
A01329	Acute Toxicity, Mutagenicity, Multiple Dose Effects
A01330	Acute Toxicity, Reproductive Effects
A01331	Acute Toxicity, Mutagenicity, Tumorigenicity, Reproductive Effects,
A01332	Acute Toxicity, Mutagenicity, Tumorigenicity, Multiple Dose Effects
A01333	Non-toxicity
A01334	Acute Toxicity, Multiple Dose Effects
A01335	Acute Toxicity, Tumorigenicity, Reproductive Effects, Multiple Dose Effects
A01336	Acute Toxicity
A01337	Acute Toxicity, Reproductive Effects
A01338	Acute Toxicity, Tumorigenicity, Multiple Dose Effects
A01339	Acute Toxicity, Skin and Eye Irritation, Reproductive Effects, Multiple Dose
A01340	Acute Toxicity, Reproductive Effects, Multiple Dose Effects
A01341	Acute Toxicity, Multiple Dose Effects
A01342	Acute Toxicity, Skin and Eye Irritation, Multiple Dose Effects
A01343	Acute Toxicity, Mutagenicity, Tumorigenicity, Multiple Dose Effects
A01344	Acute Toxicity, Multiple Dose Effects
A01345	Acute Toxicity, Mutagenicity, Tumorigenicity, Reproductive Effects,
A01346	Acute Toxicity, Tumorigenicity, Multiple Dose Effects

A01347	Acute Toxicity, Mutagenicity, Skin and Eye Irritation, Reproductive Effects, Multiple Dose Effects
A01348	Acute Toxicity, Mutagenicity, Multiple Dose Effects
A01349	Acute Toxicity
A01350	Non-toxicity
A01351	Acute Toxicity, Reproductive Effects, Multiple Dose Effects
A01352	Acute Toxicity, Mutagenicity, Reproductive Effects, Multiple Dose Effects
A01353	Acute Toxicity, Mutagenicity, Skin and Eye Irritation, Multiple Dose Effects
A01354	Acute Toxicity
A01355	Acute Toxicity
A01356	Acute Toxicity
A01357	Mutagenicity
A01358	Acute Toxicity, Mutagenicity, Tumorigenicity, Reproductive Effects,
A01359	Acute Toxicity, Mutagenicity, Tumorigenicity, Reproductive Effects,
A01360	Acute Toxicity, Mutagenicity, Tumorigenicity, Skin and Eye Irritation,
A01361	Acute Toxicity
A01362	Acute Toxicity
A01363	Acute Toxicity, Skin and Eye Irritation
A01364	Acute Toxicity, Mutagenicity, Skin and Eye Irritation, Reproductive Effects, Multiple Dose Effects
A01365	Acute Toxicity, Mutagenicity, Skin and Eye Irritation, Reproductive Effects, Multiple Dose Effects
A01366	Acute Toxicity, Mutagenicity, Tumorigenicity, Skin and Eye Irritation,
A01367	Acute Toxicity, Mutagenicity, Tumorigenicity, Skin and Eye Irritation,
A01368	Acute Toxicity, Reproductive Effects
A01369	Acute Toxicity, Reproductive Effects
A01370	Acute Toxicity, Mutagenicity, Skin and Eye Irritation, Reproductive Effects, Multiple Dose Effects
A01371	Acute Toxicity
A01372	Acute Toxicity, Skin and Eye Irritation, Multiple Dose Effects
A01373	Acute Toxicity, Mutagenicity
A01374	Non-toxicity
A01375	Acute Toxicity, Mutagenicity, Skin and Eye Irritation, Reproductive Effects, Multiple Dose Effects
A01376	Acute Toxicity
A01377	Acute Toxicity, Skin and Eye Irritation, Reproductive Effects, Multiple Dose
A01378	Acute Toxicity, Mutagenicity, Reproductive Effects, Multiple Dose Effects
A01379	Acute Toxicity
A01380	Acute Toxicity
A01381	Acute Toxicity, Multiple Dose Effects
A01382	Acute Toxicity, Mutagenicity, Reproductive Effects, Multiple Dose Effects
A01383	Acute Toxicity, Mutagenicity, Tumorigenicity, Multiple Dose Effects
A01384	Acute Toxicity, Mutagenicity
A01385	Acute Toxicity, Mutagenicity, Tumorigenicity, Skin and Eye Irritation,
A01386	Acute Toxicity, Mutagenicity, Skin and Eye Irritation, Reproductive Effects
A01387	Acute Toxicity

A01388	Acute Toxicity, Mutagenicity
A01389	Acute Toxicity
A01390	Acute Toxicity, Multiple Dose Effects
A01391	Acute Toxicity, Reproductive Effects, Multiple Dose Effects
A01392	Acute Toxicity, Reproductive Effects, Multiple Dose Effects
A01393	Acute Toxicity
A01394	Acute Toxicity, Multiple Dose Effects
A01395	Non-toxicity
A01396	Acute Toxicity, Mutagenicity, Skin and Eye Irritation
A01397	Acute Toxicity, Mutagenicity, Reproductive Effects, Multiple Dose Effects
A01398	Acute Toxicity, Mutagenicity, Tumorigenicity, Multiple Dose Effects
A01399	Acute Toxicity, Mutagenicity, Reproductive Effects, Multiple Dose Effects
A01400	Acute Toxicity, Mutagenicity, Tumorigenicity, Reproductive Effects,
A01401	Acute Toxicity, Mutagenicity, Multiple Dose Effects
A01402	Acute Toxicity, Mutagenicity, Tumorigenicity, Skin and Eye Irritation
A01403	Acute Toxicity, Reproductive Effects, Multiple Dose Effects
A01404	Acute Toxicity, Mutagenicity, Tumorigenicity, Skin and Eye Irritation,
A01405	Acute Toxicity, Mutagenicity, Skin and Eye Irritation, Reproductive Effects
A01406	Acute Toxicity, Mutagenicity, Tumorigenicity, Reproductive Effects,
A01407	Acute Toxicity, Reproductive Effects
A01408	Acute Toxicity, Mutagenicity, Reproductive Effects
A01409	Acute Toxicity, Mutagenicity, Tumorigenicity, Skin and Eye Irritation,
A01410	Acute Toxicity, Mutagenicity
A01411	Acute Toxicity
A01412	Acute Toxicity, Skin and Eye Irritation
A01413	Acute Toxicity, Tumorigenicity, Reproductive Effects
A01414	Non-toxicity
A01415	Acute Toxicity, Mutagenicity, Skin and Eye Irritation, Reproductive Effects, Multiple Dose Effects
A01416	Acute Toxicity, Mutagenicity, Multiple Dose Effects
A01417	Acute Toxicity, Mutagenicity, Multiple Dose Effects
A01418	Acute Toxicity, Mutagenicity, Skin and Eye Irritation, Multiple Dose Effects
A01419	Acute Toxicity, Tumorigenicity, Multiple Dose Effects
A01420	Acute Toxicity, Skin and Eye Irritation
A01421	Non-toxicity
A01422	Acute Toxicity, Reproductive Effects, Multiple Dose Effects
A01423	Acute Toxicity
A01424	Multiple Dose Effects
A01425	Acute Toxicity, Mutagenicity
A01426	Acute Toxicity
A01427	Acute Toxicity, Reproductive Effects, Multiple Dose Effects
A01428	Acute Toxicity, Skin and Eye Irritation, Multiple Dose Effects
A01429	Acute Toxicity, Reproductive Effects, Multiple Dose Effects
A01430	Acute Toxicity
A01431	Acute Toxicity, Mutagenicity, Tumorigenicity, Reproductive Effects,
A01432	Acute Toxicity, Mutagenicity, Skin and Eye Irritation, Multiple Dose Effects

A01433	Acute Toxicity, Reproductive Effects, Multiple Dose Effects
A01434	Acute Toxicity, Mutagenicity, Reproductive Effects, Multiple Dose Effects
A01435	Acute Toxicity, Reproductive Effects, Multiple Dose Effects
A01436	Acute Toxicity, Mutagenicity, Reproductive Effects, Multiple Dose Effects
A01437	Acute Toxicity, Multiple Dose Effects
A01438	Acute Toxicity, Mutagenicity, Tumorigenicity, Reproductive Effects,
A01439	Acute Toxicity, Multiple Dose Effects
A01440	Acute Toxicity
A01441	Acute Toxicity, Multiple Dose Effects
A01442	Multiple Dose Effects
A01443	Acute Toxicity, Multiple Dose Effects
A01444	Acute Toxicity, Skin and Eye Irritation, Reproductive Effects, Multiple Dose
A01445	Acute Toxicity
A01446	Acute Toxicity, Multiple Dose Effects
A01447	Acute Toxicity, Reproductive Effects, Multiple Dose Effects
A01448	Acute Toxicity, Mutagenicity, Reproductive Effects, Multiple Dose Effects
A01449	Acute Toxicity, Reproductive Effects, Multiple Dose Effects
A01450	Acute Toxicity, Mutagenicity, Skin and Eye Irritation
A01451	Acute Toxicity, Mutagenicity, Tumorigenicity, Skin and Eye Irritation,
A01452	Acute Toxicity, Mutagenicity, Skin and Eye Irritation, Reproductive Effects, Multiple Dose Effects
A01453	Acute Toxicity, Mutagenicity, Tumorigenicity, Skin and Eye Irritation,
A01454	Acute Toxicity, Mutagenicity, Tumorigenicity, Skin and Eye Irritation,
A01455	Acute Toxicity
A01456	Acute Toxicity, Multiple Dose Effects
A01457	Acute Toxicity, Mutagenicity, Tumorigenicity, Reproductive Effects,
A01458	Acute Toxicity, Mutagenicity, Tumorigenicity, Skin and Eye Irritation,
A01459	Acute Toxicity, Mutagenicity, Tumorigenicity, Skin and Eye Irritation,
A01460	Acute Toxicity, Mutagenicity, Tumorigenicity, Reproductive Effects,
A01461	Acute Toxicity, Mutagenicity, Tumorigenicity, Skin and Eye Irritation,
A01462	Acute Toxicity, Mutagenicity
A01463	Acute Toxicity, Skin and Eye Irritation, Reproductive Effects, Multiple Dose
A01464	Acute Toxicity, Mutagenicity, Reproductive Effects
A01465	Acute Toxicity
A01466	Acute Toxicity
A01467	Acute Toxicity
A01468	Acute Toxicity, Mutagenicity, Tumorigenicity, Reproductive Effects,
A01469	Acute Toxicity, Reproductive Effects, Multiple Dose Effects
A01470	Acute Toxicity, Reproductive Effects
A01471	Acute Toxicity
A01472	Acute Toxicity
A01473	Acute Toxicity, Mutagenicity, Skin and Eye Irritation
A01474	Acute Toxicity, Mutagenicity, Skin and Eye Irritation, Reproductive Effects, Multiple Dose Effects
A01475	Acute Toxicity, Mutagenicity
A01476	Acute Toxicity

A01477	Acute Toxicity
A01478	Acute Toxicity, Mutagenicity, Tumorigenicity, Skin and Eye Irritation,
A01479	Acute Toxicity, Mutagenicity, Skin and Eye Irritation, Reproductive Effects, Multiple Dose Effects
A01480	Acute Toxicity
A01481	Acute Toxicity, Mutagenicity, Tumorigenicity, Skin and Eye Irritation,
A01482	Acute Toxicity
A01483	Acute Toxicity, Mutagenicity
A01484	Acute Toxicity, Mutagenicity, Tumorigenicity, Reproductive Effects,
A01485	Acute Toxicity
A01486	Acute Toxicity, Mutagenicity, Skin and Eye Irritation, Reproductive Effects, Multiple Dose Effects
A01487	Acute Toxicity
A01488	Acute Toxicity
A01489	Mutagenicity
A01490	Acute Toxicity, Mutagenicity
A01491	Acute Toxicity
A01492	Acute Toxicity, Mutagenicity, Skin and Eye Irritation, Reproductive Effects, Multiple Dose Effects
A01493	Acute Toxicity
A01494	Acute Toxicity, Mutagenicity, Tumorigenicity, Reproductive Effects,
A01495	Acute Toxicity, Mutagenicity, Reproductive Effects
A01496	Acute Toxicity, Reproductive Effects, Multiple Dose Effects
A01497	Acute Toxicity
A01498	Acute Toxicity
A01499	Mutagenicity, Tumorigenicity, Skin and Eye Irritation, Reproductive Effects, Multiple Dose Effects
A01500	Acute Toxicity, Skin and Eye Irritation, Reproductive Effects, Multiple Dose
A01501	Acute Toxicity, Skin and Eye Irritation, Reproductive Effects
A01502	Acute Toxicity
A01503	Acute Toxicity, Mutagenicity, Reproductive Effects, Multiple Dose Effects
A01504	Acute Toxicity, Tumorigenicity, Multiple Dose Effects
A01505	Acute Toxicity, Mutagenicity, Reproductive Effects, Multiple Dose Effects
A01506	Acute Toxicity
A01507	Acute Toxicity, Mutagenicity, Tumorigenicity, Skin and Eye Irritation,
A01508	Acute Toxicity, Mutagenicity, Tumorigenicity, Skin and Eye Irritation,
A01509	Reproductive Effects, Multiple Dose Effects
A01510	Mutagenicity, Reproductive Effects, Multiple Dose Effects
A01511	Acute Toxicity, Multiple Dose Effects
A01512	Acute Toxicity, Mutagenicity, Tumorigenicity, Reproductive Effects,
A01513	Mutagenicity
A01514	Acute Toxicity, Mutagenicity, Tumorigenicity, Skin and Eye Irritation,
A01515	Acute Toxicity, Mutagenicity, Tumorigenicity, Reproductive Effects,
A01516	Non-toxicity
A01517	Skin and Eye Irritation
A01518	Acute Toxicity, Skin and Eye Irritation, Reproductive Effects, Multiple Dose

A01519	Acute Toxicity, Reproductive Effects, Multiple Dose Effects
A01520	Acute Toxicity, Mutagenicity, Reproductive Effects
A01521	Acute Toxicity
A01522	Acute Toxicity, Mutagenicity, Tumorigenicity, Reproductive Effects,
A01523	Reproductive Effects
A01524	Acute Toxicity
A01525	Tumorigenicity, Reproductive Effects
A01526	Acute Toxicity, Mutagenicity, Tumorigenicity, Multiple Dose Effects
A01527	Acute Toxicity, Mutagenicity
A01528	Mutagenicity
A01529	Mutagenicity, Tumorigenicity
A01530	Acute Toxicity, Mutagenicity, Tumorigenicity, Reproductive Effects
A01531	Acute Toxicity
A01532	Acute Toxicity
A01533	Acute Toxicity, Mutagenicity, Reproductive Effects, Multiple Dose Effects
A01534	Acute Toxicity
A01535	Acute Toxicity, Multiple Dose Effects
A01536	Acute Toxicity, Mutagenicity, Tumorigenicity, Skin and Eye Irritation, Multiple Dose Effects
A01537	Acute Toxicity, Mutagenicity, Tumorigenicity, Skin and Eye Irritation,
A01538	Acute Toxicity, Tumorigenicity
A01539	Acute Toxicity, Skin and Eye Irritation, Reproductive Effects, Multiple Dose
A01540	Acute Toxicity, Multiple Dose Effects
A01541	Acute Toxicity
A01542	Acute Toxicity, Reproductive Effects, Multiple Dose Effects
A01543	Acute Toxicity, Mutagenicity, Tumorigenicity, Reproductive Effects,
A01544	Acute Toxicity, Tumorigenicity, Skin and Eye Irritation, Multiple Dose
A01545	Acute Toxicity, Mutagenicity
A01546	Acute Toxicity, Mutagenicity
A01547	Acute Toxicity, Mutagenicity, Skin and Eye Irritation, Multiple Dose Effects
A01548	Acute Toxicity, Mutagenicity, Reproductive Effects, Multiple Dose Effects
A01549	Mutagenicity, Reproductive Effects
A01550	Acute Toxicity, Mutagenicity, Skin and Eye Irritation
A01551	Acute Toxicity, Mutagenicity, Tumorigenicity, Skin and Eye Irritation,
A01552	Acute Toxicity, Tumorigenicity, Skin and Eye Irritation
A01553	Mutagenicity
A01554	Mutagenicity
A01555	Acute Toxicity, Tumorigenicity, Reproductive Effects, Multiple Dose Effects
A01556	Acute Toxicity, Mutagenicity, Tumorigenicity, Multiple Dose Effects
A01557	Acute Toxicity, Mutagenicity, Tumorigenicity, Reproductive Effects,
A01558	Acute Toxicity, Mutagenicity, Multiple Dose Effects
A01559	Acute Toxicity, Mutagenicity, Tumorigenicity, Skin and Eye Irritation,
A01560	Acute Toxicity, Mutagenicity, Multiple Dose Effects
A01561	Acute Toxicity, Mutagenicity, Skin and Eye Irritation, Multiple Dose Effects
A01562	Mutagenicity, Reproductive Effects
A01563	Acute Toxicity, Mutagenicity, Multiple Dose Effects

A01564	Acute Toxicity
A01565	Acute Toxicity, Mutagenicity, Tumorigenicity, Skin and Eye Irritation,
A01566	Acute Toxicity, Mutagenicity, Tumorigenicity, Reproductive Effects,
A01567	Acute Toxicity, Mutagenicity, Skin and Eye Irritation, Reproductive Effects
A01568	Acute Toxicity, Mutagenicity, Tumorigenicity, Skin and Eye Irritation,
A01569	Acute Toxicity, Mutagenicity, Tumorigenicity, Reproductive Effects,
A01570	Acute Toxicity, Tumorigenicity, Multiple Dose Effects
A01571	Acute Toxicity, Skin and Eye Irritation
A01572	Acute Toxicity, Mutagenicity, Tumorigenicity, Skin and Eye Irritation,
A01573	Acute Toxicity, Mutagenicity, Tumorigenicity, Reproductive Effects,
A01574	Acute Toxicity, Mutagenicity, Tumorigenicity, Skin and Eye Irritation,
A01575	Acute Toxicity, Mutagenicity, Tumorigenicity, Skin and Eye Irritation,
A01576	Mutagenicity, Tumorigenicity, Skin and Eye Irritation, Reproductive Effects, Multiple Dose Effects
A01577	Acute Toxicity
A01578	Acute Toxicity, Mutagenicity, Tumorigenicity, Skin and Eye Irritation,
A01579	Acute Toxicity, Reproductive Effects, Multiple Dose Effects
A01580	Acute Toxicity
A01581	Acute Toxicity
A01582	Acute Toxicity, Mutagenicity, Tumorigenicity, Skin and Eye Irritation,
A01583	Acute Toxicity, Mutagenicity, Tumorigenicity, Reproductive Effects,
A01584	Mutagenicity, Reproductive Effects
A01585	Acute Toxicity, Mutagenicity, Tumorigenicity, Skin and Eye Irritation,
A01586	Acute Toxicity, Reproductive Effects, Multiple Dose Effects
A01587	Acute Toxicity, Mutagenicity, Reproductive Effects, Multiple Dose Effects
A01588	Acute Toxicity, Mutagenicity
A01589	Acute Toxicity, Mutagenicity, Skin and Eye Irritation, Reproductive Effects, Multiple Dose Effects
A01590	Acute Toxicity, Mutagenicity, Tumorigenicity, Reproductive Effects,
A01591	Acute Toxicity, Mutagenicity, Multiple Dose Effects
A01592	Acute Toxicity
A01593	Acute Toxicity
A01594	Acute Toxicity, Mutagenicity, Skin and Eye Irritation, Reproductive Effects, Multiple Dose Effects
A01595	Acute Toxicity, Mutagenicity, Tumorigenicity, Skin and Eye Irritation,
A01596	Acute Toxicity, Mutagenicity, Tumorigenicity, Reproductive Effects,
A01597	Acute Toxicity
A01598	Acute Toxicity, Tumorigenicity, Skin and Eye Irritation, Reproductive Effects
A01599	Acute Toxicity, Mutagenicity
A01600	Acute Toxicity, Mutagenicity, Tumorigenicity, Multiple Dose Effects
A01601	Acute Toxicity, Mutagenicity, Tumorigenicity, Reproductive Effects,
A01602	Acute Toxicity, Mutagenicity, Skin and Eye Irritation, Reproductive Effects, Multiple Dose Effects
A01603	Acute Toxicity, Tumorigenicity, Multiple Dose Effects
A01604	Acute Toxicity, Mutagenicity, Skin and Eye Irritation, Reproductive Effects, Multiple Dose Effects

A01605	Acute Toxicity, Mutagenicity, Reproductive Effects, Multiple Dose Effects
A01606	Acute Toxicity, Mutagenicity, Tumorigenicity, Reproductive Effects
A01607	Acute Toxicity, Mutagenicity, Tumorigenicity, Reproductive Effects,
A01608	Acute Toxicity
A01609	Acute Toxicity, Reproductive Effects
A01610	Acute Toxicity, Reproductive Effects
A01611	Acute Toxicity
A01612	Mutagenicity, Tumorigenicity
A01613	Acute Toxicity
A01614	Acute Toxicity, Reproductive Effects, Multiple Dose Effects
A01615	Acute Toxicity, Mutagenicity, Reproductive Effects
A01616	Acute Toxicity, Mutagenicity
A01617	Acute Toxicity
A01618	Acute Toxicity
A01619	Acute Toxicity
A01620	Acute Toxicity
A01621	Acute Toxicity
A01622	Acute Toxicity, Mutagenicity, Tumorigenicity, Reproductive Effects,
A01623	Acute Toxicity, Mutagenicity, Reproductive Effects
A01624	Acute Toxicity
A01625	Acute Toxicity, Mutagenicity, Skin and Eye Irritation, Multiple Dose Effects
A01626	Acute Toxicity, Skin and Eye Irritation
A01627	Acute Toxicity
A01628	Acute Toxicity, Mutagenicity, Reproductive Effects, Multiple Dose Effects
A01629	Acute Toxicity, Mutagenicity, Tumorigenicity, Skin and Eye Irritation,
A01630	Acute Toxicity, Mutagenicity, Tumorigenicity, Reproductive Effects,
A01631	Acute Toxicity
A01632	Acute Toxicity, Mutagenicity, Tumorigenicity, Reproductive Effects,
A01633	Acute Toxicity
A01634	Acute Toxicity, Tumorigenicity, Reproductive Effects, Multiple Dose Effects
A01635	Acute Toxicity, Mutagenicity, Tumorigenicity, Skin and Eye Irritation,
A01636	Acute Toxicity, Mutagenicity
A01637	Mutagenicity
A01638	Acute Toxicity, Mutagenicity
A01639	Acute Toxicity, Mutagenicity, Tumorigenicity, Reproductive Effects,
A01640	Acute Toxicity, Mutagenicity, Tumorigenicity, Reproductive Effects,
A01641	Acute Toxicity
A01642	Acute Toxicity
A01643	Acute Toxicity
A01644	Acute Toxicity
A01645	Acute Toxicity, Mutagenicity, Tumorigenicity, Skin and Eye Irritation,
A01646	Acute Toxicity, Mutagenicity, Reproductive Effects
A01647	Acute Toxicity, Mutagenicity, Skin and Eye Irritation, Reproductive Effects, Multiple Dose Effects
A01648	Acute Toxicity, Multiple Dose Effects
A01649	Acute Toxicity, Multiple Dose Effects

A01650	Acute Toxicity, Mutagenicity
A01651	Mutagenicity
A01652	Acute Toxicity
A01653	Acute Toxicity, Multiple Dose Effects
A01654	Acute Toxicity, Mutagenicity, Skin and Eye Irritation, Multiple Dose Effects
A01655	Acute Toxicity
A01656	Acute Toxicity, Mutagenicity
A01657	Acute Toxicity, Mutagenicity, Tumorigenicity
A01658	Acute Toxicity
A01659	Acute Toxicity, Mutagenicity
A01660	Acute Toxicity, Mutagenicity, Skin and Eye Irritation, Reproductive Effects, Multiple Dose Effects
A01661	Acute Toxicity, Mutagenicity, Skin and Eye Irritation, Multiple Dose Effects
A01662	Acute Toxicity
A01663	Acute Toxicity
A01664	Acute Toxicity, Mutagenicity, Tumorigenicity, Reproductive Effects,
A01665	Acute Toxicity
A01666	Acute Toxicity
A01667	Acute Toxicity, Tumorigenicity, Multiple Dose Effects
A01668	Acute Toxicity, Mutagenicity, Tumorigenicity, Skin and Eye Irritation,
A01669	Acute Toxicity, Mutagenicity, Reproductive Effects, Multiple Dose Effects
A01670	Acute Toxicity, Mutagenicity, Tumorigenicity, Reproductive Effects,
A01671	Acute Toxicity, Mutagenicity, Tumorigenicity, Skin and Eye Irritation,
A01672	Skin and Eye Irritation, Reproductive Effects, Multiple Dose Effects
A01673	Acute Toxicity, Tumorigenicity, Reproductive Effects, Multiple Dose Effects
A01674	Acute Toxicity, Mutagenicity
A01675	Mutagenicity
A01676	Acute Toxicity, Mutagenicity, Tumorigenicity, Skin and Eye Irritation,
A01677	Acute Toxicity, Mutagenicity, Tumorigenicity, Reproductive Effects,
A01678	Mutagenicity, Tumorigenicity, Reproductive Effects, Multiple Dose Effects
A01679	Acute Toxicity, Reproductive Effects, Multiple Dose Effects
A01680	Acute Toxicity, Mutagenicity, Skin and Eye Irritation, Multiple Dose Effects
A01681	Acute Toxicity, Mutagenicity, Tumorigenicity, Reproductive Effects,
A01682	Acute Toxicity
A01683	Acute Toxicity, Skin and Eye Irritation, Reproductive Effects, Multiple Dose
A01684	Acute Toxicity
A01685	Acute Toxicity, Mutagenicity, Skin and Eye Irritation
A01686	Acute Toxicity, Mutagenicity, Skin and Eye Irritation, Reproductive Effects, Multiple Dose Effects
A01687	Acute Toxicity, Mutagenicity, Skin and Eye Irritation, Reproductive Effects
A01688	Acute Toxicity
A01689	Acute Toxicity, Mutagenicity, Skin and Eye Irritation, Multiple Dose Effects
A01690	Acute Toxicity, Multiple Dose Effects
A01691	Mutagenicity
A01692	Mutagenicity
A01693	Acute Toxicity

A01694	Acute Toxicity, Mutagenicity, Tumorigenicity, Multiple Dose Effects
A01695	Acute Toxicity, Multiple Dose Effects
A01696	Acute Toxicity, Multiple Dose Effects
A01697	Multiple Dose Effects
A01698	Acute Toxicity
A01699	Acute Toxicity, Mutagenicity, Reproductive Effects, Multiple Dose Effects
A01700	Acute Toxicity
A01701	Acute Toxicity, Reproductive Effects
A01702	Non-toxicity
A01703	Mutagenicity
A01704	Acute Toxicity, Mutagenicity, Skin and Eye Irritation, Multiple Dose Effects
A01705	Acute Toxicity, Mutagenicity, Tumorigenicity, Skin and Eye Irritation,
A01706	Acute Toxicity, Mutagenicity, Tumorigenicity, Reproductive Effects,
A01707	Acute Toxicity
A01708	Acute Toxicity, Mutagenicity, Multiple Dose Effects
A01709	Acute Toxicity, Mutagenicity
A01710	Acute Toxicity, Mutagenicity, Tumorigenicity, Reproductive Effects,
A01711	Acute Toxicity, Reproductive Effects, Multiple Dose Effects
A01712	Acute Toxicity, Mutagenicity, Tumorigenicity, Skin and Eye Irritation, Multiple Dose Effects
A01713	Acute Toxicity, Mutagenicity, Tumorigenicity, Multiple Dose Effects
A01714	Acute Toxicity, Mutagenicity, Tumorigenicity, Reproductive Effects,
A01715	Acute Toxicity, Mutagenicity, Tumorigenicity, Reproductive Effects,
A01716	Acute Toxicity, Mutagenicity, Skin and Eye Irritation
A01717	Acute Toxicity, Mutagenicity, Reproductive Effects
A01718	Acute Toxicity, Mutagenicity, Tumorigenicity, Reproductive Effects,
A01719	Acute Toxicity, Mutagenicity, Tumorigenicity, Multiple Dose Effects
A01720	Acute Toxicity, Mutagenicity
A01721	Acute Toxicity
A01722	Acute Toxicity, Mutagenicity
A01723	Acute Toxicity, Mutagenicity, Tumorigenicity, Skin and Eye Irritation,
A01724	Acute Toxicity, Multiple Dose Effects
A01725	Acute Toxicity, Mutagenicity, Tumorigenicity, Reproductive Effects,
A01726	Acute Toxicity
A01727	Acute Toxicity
A01728	Acute Toxicity, Mutagenicity, Tumorigenicity, Reproductive Effects,
A01729	Acute Toxicity, Mutagenicity, Tumorigenicity, Skin and Eye Irritation,
A01730	Acute Toxicity, Mutagenicity, Skin and Eye Irritation, Multiple Dose Effects
A01731	Acute Toxicity, Mutagenicity, Tumorigenicity, Multiple Dose Effects
A01732	Acute Toxicity, Mutagenicity, Multiple Dose Effects
A01733	Acute Toxicity
A01734	Acute Toxicity, Mutagenicity, Multiple Dose Effects
A01735	Acute Toxicity, Reproductive Effects, Multiple Dose Effects
A01736	Acute Toxicity, Mutagenicity, Tumorigenicity, Skin and Eye Irritation,
A01737	Acute Toxicity, Mutagenicity, Tumorigenicity, Reproductive Effects,
A01738	Acute Toxicity, Mutagenicity, Tumorigenicity, Multiple Dose Effects

A01739	Acute Toxicity, Mutagenicity
A01740	Acute Toxicity, Mutagenicity, Skin and Eye Irritation
A01741	Acute Toxicity, Mutagenicity, Tumorigenicity, Skin and Eye Irritation,
A01742	Acute Toxicity, Skin and Eye Irritation
A01743	Acute Toxicity, Mutagenicity, Tumorigenicity, Skin and Eye Irritation,
A01744	Acute Toxicity, Reproductive Effects, Multiple Dose Effects
A01745	Acute Toxicity, Mutagenicity
A01746	Acute Toxicity, Mutagenicity, Tumorigenicity, Reproductive Effects,
A01747	Mutagenicity
A01748	Acute Toxicity, Mutagenicity
A01749	Acute Toxicity, Mutagenicity, Tumorigenicity, Skin and Eye Irritation,
A01750	Acute Toxicity, Mutagenicity, Reproductive Effects, Multiple Dose Effects
A01751	Acute Toxicity, Mutagenicity, Tumorigenicity, Reproductive Effects,
A01752	Acute Toxicity
A01753	Acute Toxicity, Mutagenicity, Multiple Dose Effects
A01754	Acute Toxicity, Tumorigenicity
A01755	Acute Toxicity, Mutagenicity, Skin and Eye Irritation
A01756	Acute Toxicity
A01757	Acute Toxicity, Mutagenicity, Tumorigenicity, Reproductive Effects,
A01758	Acute Toxicity, Mutagenicity, Tumorigenicity, Reproductive Effects,
A01759	Acute Toxicity, Mutagenicity
A01760	Acute Toxicity, Mutagenicity, Multiple Dose Effects
A01761	Acute Toxicity, Mutagenicity, Tumorigenicity, Multiple Dose Effects
A01762	Acute Toxicity, Reproductive Effects, Multiple Dose Effects
A01763	Acute Toxicity, Multiple Dose Effects
A01764	Acute Toxicity, Mutagenicity, Tumorigenicity, Reproductive Effects,
A01765	Acute Toxicity, Mutagenicity
A01766	Acute Toxicity, Mutagenicity, Tumorigenicity, Multiple Dose Effects
A01767	Acute Toxicity, Mutagenicity, Tumorigenicity, Multiple Dose Effects
A01768	Acute Toxicity, Mutagenicity, Tumorigenicity, Skin and Eye Irritation
A01769	Acute Toxicity, Mutagenicity, Tumorigenicity, Skin and Eye Irritation,
A01770	Acute Toxicity
A01771	Acute Toxicity
A01772	Acute Toxicity, Mutagenicity
A01773	Acute Toxicity
A01774	Acute Toxicity, Mutagenicity, Multiple Dose Effects
A01775	Acute Toxicity, Mutagenicity, Tumorigenicity, Skin and Eye Irritation,
A01776	Acute Toxicity, Skin and Eye Irritation, Multiple Dose Effects
A01777	Acute Toxicity, Mutagenicity, Reproductive Effects, Multiple Dose Effects
A01778	Acute Toxicity, Mutagenicity, Tumorigenicity, Skin and Eye Irritation, Multiple Dose Effects
A01779	Acute Toxicity, Mutagenicity, Tumorigenicity, Multiple Dose Effects
A01780	Acute Toxicity
A01781	Acute Toxicity, Mutagenicity, Skin and Eye Irritation, Multiple Dose Effects
A01782	Acute Toxicity, Mutagenicity
A01783	Acute Toxicity

A01784	Mutagenicity
A01785	Acute Toxicity, Skin and Eye Irritation, Multiple Dose Effects
A01786	Acute Toxicity, Mutagenicity, Multiple Dose Effects
A01787	Acute Toxicity, Mutagenicity, Multiple Dose Effects
A01788	Acute Toxicity, Tumorigenicity, Multiple Dose Effects
A01789	Acute Toxicity, Mutagenicity, Skin and Eye Irritation, Reproductive Effects, Multiple Dose Effects
A01790	Acute Toxicity, Mutagenicity, Skin and Eye Irritation, Reproductive Effects, Multiple Dose Effects
A01791	Acute Toxicity
A01792	Acute Toxicity, Tumorigenicity, Multiple Dose Effects
A01793	Acute Toxicity, Mutagenicity
A01794	Acute Toxicity, Mutagenicity, Tumorigenicity, Multiple Dose Effects
A01795	Acute Toxicity, Mutagenicity
A01796	Acute Toxicity, Mutagenicity, Tumorigenicity, Skin and Eye Irritation,
A01797	Acute Toxicity, Mutagenicity, Reproductive Effects, Multiple Dose Effects
A01798	Acute Toxicity, Mutagenicity, Tumorigenicity, Multiple Dose Effects
A01799	Acute Toxicity, Skin and Eye Irritation
A01800	Acute Toxicity, Multiple Dose Effects
A01801	Acute Toxicity, Skin and Eye Irritation, Multiple Dose Effects
A01802	Acute Toxicity, Tumorigenicity, Skin and Eye Irritation, Reproductive Effects, Multiple Dose Effects
A01803	Acute Toxicity, Mutagenicity
A01804	Acute Toxicity
A01805	Acute Toxicity, Multiple Dose Effects
A01806	Acute Toxicity, Skin and Eye Irritation, Multiple Dose Effects
A01807	Tumorigenicity
A01808	Acute Toxicity
A01809	Acute Toxicity, Mutagenicity, Tumorigenicity, Skin and Eye Irritation,
A01810	Acute Toxicity
A01811	Acute Toxicity, Mutagenicity, Reproductive Effects, Multiple Dose Effects
A01812	Mutagenicity
A01813	Acute Toxicity, Mutagenicity, Multiple Dose Effects
A01814	Acute Toxicity
A01815	Acute Toxicity
A01816	Acute Toxicity
A01817	Acute Toxicity
A01818	Acute Toxicity
A01819	Acute Toxicity, Tumorigenicity
A01820	Acute Toxicity
A01821	Acute Toxicity, Mutagenicity, Tumorigenicity, Multiple Dose Effects
A01822	Acute Toxicity, Reproductive Effects, Multiple Dose Effects
A01823	Acute Toxicity
A01824	Acute Toxicity
A01825	Acute Toxicity, Reproductive Effects
A01826	Acute Toxicity

A01827	Acute Toxicity, Reproductive Effects, Multiple Dose Effects
A01828	Acute Toxicity, Mutagenicity, Tumorigenicity
A01829	Acute Toxicity, Mutagenicity, Tumorigenicity, Skin and Eye Irritation,
A01830	Acute Toxicity, Multiple Dose Effects
A01831	Acute Toxicity
A01832	Acute Toxicity, Mutagenicity, Tumorigenicity
A01833	Acute Toxicity
A01834	Acute Toxicity, Mutagenicity
A01835	Acute Toxicity, Reproductive Effects, Multiple Dose Effects
A01836	Acute Toxicity
A01837	Acute Toxicity
A01838	Acute Toxicity, Mutagenicity, Skin and Eye Irritation
A01839	Acute Toxicity, Mutagenicity, Tumorigenicity, Skin and Eye Irritation,
A01840	Acute Toxicity, Mutagenicity, Tumorigenicity, Skin and Eye Irritation, Multiple Dose Effects
A01841	Acute Toxicity, Mutagenicity, Multiple Dose Effects
A01842	Acute Toxicity, Mutagenicity, Tumorigenicity, Multiple Dose Effects
A01843	Acute Toxicity, Mutagenicity
A01844	Acute Toxicity, Skin and Eye Irritation, Reproductive Effects, Multiple Dose
A01845	Acute Toxicity, Multiple Dose Effects
A01846	Acute Toxicity, Mutagenicity, Reproductive Effects, Multiple Dose Effects
A01847	Acute Toxicity, Multiple Dose Effects
A01848	Acute Toxicity, Multiple Dose Effects
A01849	Acute Toxicity, Multiple Dose Effects
A01850	Acute Toxicity, Reproductive Effects
A01851	Acute Toxicity, Mutagenicity, Tumorigenicity, Reproductive Effects,
A01852	Acute Toxicity
A01853	Acute Toxicity, Skin and Eye Irritation
A01854	Acute Toxicity
A01855	Acute Toxicity, Mutagenicity, Reproductive Effects, Multiple Dose Effects
A01856	Skin and Eye Irritation, Multiple Dose Effects
A01857	Acute Toxicity, Mutagenicity, Tumorigenicity, Multiple Dose Effects
A01858	Acute Toxicity, Mutagenicity, Multiple Dose Effects
A01859	Acute Toxicity, Mutagenicity, Skin and Eye Irritation, Reproductive Effects, Multiple Dose Effects
A01860	Acute Toxicity, Reproductive Effects
A01861	Acute Toxicity, Mutagenicity, Tumorigenicity, Skin and Eye Irritation, Multiple Dose Effects
A01862	Acute Toxicity, Mutagenicity, Multiple Dose Effects
A01863	Acute Toxicity
A01864	Acute Toxicity, Mutagenicity, Skin and Eye Irritation, Reproductive Effects, Multiple Dose Effects
A01865	Acute Toxicity
A01866	Acute Toxicity, Mutagenicity
A01867	Acute Toxicity, Mutagenicity, Tumorigenicity, Skin and Eye Irritation,
A01868	Acute Toxicity, Mutagenicity

A01869	Acute Toxicity, Skin and Eye Irritation
A01870	Acute Toxicity, Mutagenicity, Tumorigenicity, Skin and Eye Irritation,
A01871	Acute Toxicity, Multiple Dose Effects
A01872	Acute Toxicity, Mutagenicity
A01873	Mutagenicity, Tumorigenicity
A01874	Acute Toxicity, Mutagenicity, Tumorigenicity
A01875	Acute Toxicity, Mutagenicity, Multiple Dose Effects
A01876	Acute Toxicity, Mutagenicity, Tumorigenicity, Reproductive Effects,
A01877	Acute Toxicity, Mutagenicity
A01878	Acute Toxicity, Skin and Eye Irritation
A01879	Acute Toxicity, Mutagenicity, Tumorigenicity, Reproductive Effects,
A01880	Acute Toxicity, Mutagenicity, Tumorigenicity, Skin and Eye Irritation,
A01881	Acute Toxicity, Mutagenicity, Skin and Eye Irritation
A01882	Acute Toxicity, Tumorigenicity
A01883	Acute Toxicity, Mutagenicity, Tumorigenicity, Skin and Eye Irritation,
A01884	Acute Toxicity, Mutagenicity, Skin and Eye Irritation, Multiple Dose Effects
A01885	Acute Toxicity, Mutagenicity, Skin and Eye Irritation, Multiple Dose Effects
A01886	Acute Toxicity, Mutagenicity, Skin and Eye Irritation
A01887	Acute Toxicity, Skin and Eye Irritation, Reproductive Effects, Multiple Dose
A01888	Acute Toxicity, Mutagenicity, Skin and Eye Irritation, Reproductive Effects, Multiple Dose Effects
A01889	Acute Toxicity, Mutagenicity, Skin and Eye Irritation, Multiple Dose Effects
A01890	Acute Toxicity, Mutagenicity, Multiple Dose Effects
A01891	Acute Toxicity, Mutagenicity
A01892	Acute Toxicity, Skin and Eye Irritation
A01893	Acute Toxicity, Mutagenicity, Tumorigenicity, Skin and Eye Irritation, Multiple Dose Effects
A01894	Acute Toxicity, Mutagenicity, Tumorigenicity, Skin and Eye Irritation,
A01895	Mutagenicity
A01896	Acute Toxicity, Mutagenicity, Skin and Eye Irritation, Multiple Dose Effects
A01897	Acute Toxicity
A01898	Acute Toxicity, Mutagenicity, Tumorigenicity, Skin and Eye Irritation,
A01899	Acute Toxicity, Mutagenicity, Skin and Eye Irritation, Multiple Dose Effects
A01900	Acute Toxicity, Mutagenicity, Tumorigenicity, Skin and Eye Irritation,
A01901	Acute Toxicity, Mutagenicity, Reproductive Effects, Multiple Dose Effects
A01902	Acute Toxicity, Mutagenicity, Skin and Eye Irritation, Multiple Dose Effects
A01903	Acute Toxicity, Mutagenicity, Tumorigenicity, Skin and Eye Irritation, Multiple Dose Effects
A01904	Acute Toxicity, Multiple Dose Effects
A01905	Acute Toxicity, Mutagenicity, Multiple Dose Effects
A01906	Acute Toxicity, Mutagenicity, Tumorigenicity, Skin and Eye Irritation, Multiple Dose Effects
A01907	Mutagenicity, Multiple Dose Effects
A01908	Acute Toxicity, Skin and Eye Irritation
A01909	Acute Toxicity, Mutagenicity, Tumorigenicity, Skin and Eye Irritation,
A01910	Acute Toxicity, Multiple Dose Effects

A01911	Acute Toxicity, Mutagenicity, Tumorigenicity, Skin and Eye Irritation, Multiple Dose Effects
A01912	Acute Toxicity, Multiple Dose Effects
A01913	Acute Toxicity, Tumorigenicity, Skin and Eye Irritation, Reproductive Effects, Multiple Dose Effects
A01914	Acute Toxicity, Mutagenicity, Tumorigenicity, Skin and Eye Irritation,
A01915	Acute Toxicity, Mutagenicity, Tumorigenicity
A01916	Acute Toxicity, Reproductive Effects
A01917	Acute Toxicity
A01918	Mutagenicity, Reproductive Effects
A01919	Acute Toxicity, Reproductive Effects, Multiple Dose Effects
A01920	Acute Toxicity, Reproductive Effects, Multiple Dose Effects
A01921	Acute Toxicity
A01922	Acute Toxicity
A01923	Acute Toxicity, Mutagenicity, Skin and Eye Irritation, Reproductive Effects, Multiple Dose Effects
A01924	Acute Toxicity, Skin and Eye Irritation, Multiple Dose Effects
A01925	Acute Toxicity, Mutagenicity
A01926	Acute Toxicity, Mutagenicity, Tumorigenicity, Skin and Eye Irritation,
A01927	Acute Toxicity
A01928	Acute Toxicity, Tumorigenicity, Multiple Dose Effects
A01929	Acute Toxicity, Mutagenicity, Multiple Dose Effects
A01930	Acute Toxicity, Mutagenicity, Tumorigenicity
A01931	Acute Toxicity, Mutagenicity, Tumorigenicity, Skin and Eye Irritation, Multiple Dose Effects
A01932	Acute Toxicity, Mutagenicity, Multiple Dose Effects
A01933	Acute Toxicity, Tumorigenicity, Multiple Dose Effects
A01934	Acute Toxicity, Skin and Eye Irritation
A01935	Acute Toxicity, Mutagenicity, Tumorigenicity
A01936	Acute Toxicity, Mutagenicity, Multiple Dose Effects
A01937	Acute Toxicity, Mutagenicity, Skin and Eye Irritation, Multiple Dose Effects
A01938	Mutagenicity, Tumorigenicity
A01939	Acute Toxicity, Mutagenicity, Tumorigenicity, Skin and Eye Irritation
A01940	Acute Toxicity, Mutagenicity, Skin and Eye Irritation, Multiple Dose Effects
A01941	Acute Toxicity, Mutagenicity, Tumorigenicity, Skin and Eye Irritation,
A01942	Acute Toxicity, Mutagenicity, Skin and Eye Irritation, Multiple Dose Effects
A01943	Acute Toxicity, Mutagenicity
A01944	Acute Toxicity, Skin and Eye Irritation
A01945	Non-toxicity
A01946	Acute Toxicity
A01947	Acute Toxicity, Mutagenicity, Skin and Eye Irritation, Reproductive Effects, Multiple Dose Effects
A01948	Acute Toxicity, Mutagenicity, Tumorigenicity, Skin and Eye Irritation
A01949	Acute Toxicity, Mutagenicity
A01950	Acute Toxicity, Multiple Dose Effects
A01951	Acute Toxicity

A01952	Acute Toxicity, Mutagenicity, Tumorigenicity
A01953	Mutagenicity, Tumorigenicity, Skin and Eye Irritation, Multiple Dose Effects
A01954	Acute Toxicity, Mutagenicity, Tumorigenicity, Skin and Eye Irritation
A01955	Acute Toxicity, Mutagenicity, Skin and Eye Irritation
A01956	Acute Toxicity, Mutagenicity, Skin and Eye Irritation, Multiple Dose Effects
A01957	Acute Toxicity
A01958	Acute Toxicity
A01959	Acute Toxicity, Mutagenicity, Tumorigenicity, Reproductive Effects,
A01960	Acute Toxicity, Skin and Eye Irritation, Multiple Dose Effects
A01961	Acute Toxicity, Mutagenicity, Skin and Eye Irritation, Multiple Dose Effects
A01962	Mutagenicity, Tumorigenicity
A01963	Acute Toxicity, Mutagenicity, Skin and Eye Irritation
A01964	Acute Toxicity
A01965	Skin and Eye Irritation
A01966	Acute Toxicity, Skin and Eye Irritation, Multiple Dose Effects
A01967	Acute Toxicity, Mutagenicity, Tumorigenicity, Skin and Eye Irritation, Multiple Dose Effects
A01968	Skin and Eye Irritation
A01969	Acute Toxicity
A01970	Acute Toxicity
A01971	Acute Toxicity, Multiple Dose Effects
A01972	Acute Toxicity, Mutagenicity, Tumorigenicity, Reproductive Effects,
A01973	Mutagenicity
A01974	Acute Toxicity, Mutagenicity
A01975	Acute Toxicity, Mutagenicity
A01976	Acute Toxicity, Reproductive Effects
A01977	Reproductive Effects
A01978	Acute Toxicity, Mutagenicity, Reproductive Effects, Multiple Dose Effects
A01979	Acute Toxicity, Mutagenicity, Tumorigenicity, Skin and Eye Irritation,
A01980	Acute Toxicity, Mutagenicity, Multiple Dose Effects
A01981	Acute Toxicity
A01982	Acute Toxicity, Mutagenicity, Multiple Dose Effects
A01983	Acute Toxicity, Mutagenicity
A01984	Mutagenicity
A01985	Acute Toxicity, Mutagenicity
A01986	Mutagenicity
A01987	Acute Toxicity
A01988	Acute Toxicity, Skin and Eye Irritation, Multiple Dose Effects
A01989	Acute Toxicity
A01990	Mutagenicity
A01991	Acute Toxicity, Tumorigenicity
A01992	Acute Toxicity, Skin and Eye Irritation
A01993	Acute Toxicity, Mutagenicity, Skin and Eye Irritation, Multiple Dose Effects
A01994	Acute Toxicity
A01995	Acute Toxicity, Mutagenicity, Tumorigenicity, Skin and Eye Irritation,
A01996	Non-toxicity

A01997	Mutagenicity
A01998	Acute Toxicity
A01999	Acute Toxicity
A02000	Acute Toxicity, Mutagenicity, Tumorigenicity
A02001	Acute Toxicity, Mutagenicity, Multiple Dose Effects
A02002	Acute Toxicity, Mutagenicity, Multiple Dose Effects
A02003	Acute Toxicity, Reproductive Effects
A02004	Acute Toxicity
A02005	Mutagenicity
A02006	Acute Toxicity, Mutagenicity
A02007	Acute Toxicity, Mutagenicity
A02008	Acute Toxicity, Mutagenicity, Reproductive Effects, Multiple Dose Effects
A02009	Acute Toxicity, Multiple Dose Effects
A02010	Acute Toxicity, Mutagenicity, Tumorigenicity, Skin and Eye Irritation, Multiple Dose Effects
A02011	Acute Toxicity, Mutagenicity, Tumorigenicity, Skin and Eye Irritation,
A02012	Acute Toxicity, Mutagenicity, Reproductive Effects, Multiple Dose Effects
A02013	Acute Toxicity
A02014	Acute Toxicity, Mutagenicity, Skin and Eye Irritation, Reproductive Effects, Multiple Dose Effects
A02015	Acute Toxicity
A02016	Acute Toxicity, Skin and Eye Irritation
A02017	Acute Toxicity, Mutagenicity
A02018	Acute Toxicity, Mutagenicity
A02019	Acute Toxicity, Multiple Dose Effects
A02020	Acute Toxicity, Mutagenicity, Skin and Eye Irritation, Reproductive Effects, Multiple Dose Effects
A02021	Mutagenicity, Tumorigenicity
A02022	Acute Toxicity, Mutagenicity
A02023	Acute Toxicity
A02024	Acute Toxicity
A02025	Mutagenicity, Reproductive Effects
A02026	Acute Toxicity, Reproductive Effects
A02027	Acute Toxicity, Skin and Eye Irritation
A02028	Acute Toxicity, Mutagenicity, Tumorigenicity, Skin and Eye Irritation,
A02029	Acute Toxicity
A02030	Acute Toxicity, Mutagenicity, Reproductive Effects, Multiple Dose Effects
A02031	Acute Toxicity, Mutagenicity, Skin and Eye Irritation, Reproductive Effects
A02032	Mutagenicity
A02033	Acute Toxicity, Mutagenicity, Multiple Dose Effects
A02034	Acute Toxicity, Mutagenicity, Multiple Dose Effects
A02035	Acute Toxicity, Mutagenicity, Multiple Dose Effects
A02036	Acute Toxicity, Mutagenicity, Skin and Eye Irritation
A02037	Mutagenicity, Tumorigenicity
A02038	Acute Toxicity, Tumorigenicity

A02039	Acute Toxicity, Mutagenicity, Tumorigenicity, Skin and Eye Irritation, Multiple Dose Effects
A02040	Acute Toxicity, Mutagenicity, Reproductive Effects, Multiple Dose Effects
A02041	Acute Toxicity
A02042	Acute Toxicity
A02043	Mutagenicity
A02044	Acute Toxicity
A02045	Acute Toxicity, Mutagenicity
A02046	Acute Toxicity, Mutagenicity, Tumorigenicity
A02047	Tumorigenicity
A02048	Acute Toxicity, Mutagenicity
A02049	Acute Toxicity, Mutagenicity, Tumorigenicity, Multiple Dose Effects
A02050	Mutagenicity
A02051	Acute Toxicity
A02052	Acute Toxicity
A02053	Acute Toxicity, Tumorigenicity
A02054	Acute Toxicity, Mutagenicity, Tumorigenicity, Skin and Eye Irritation, Multiple Dose Effects
A02055	Acute Toxicity, Mutagenicity
A02056	Acute Toxicity
A02057	Acute Toxicity, Mutagenicity, Skin and Eye Irritation
A02058	Acute Toxicity, Mutagenicity, Skin and Eye Irritation
A02059	Acute Toxicity, Mutagenicity
A02060	Acute Toxicity, Skin and Eye Irritation
A02061	Acute Toxicity
A02062	Acute Toxicity, Reproductive Effects, Multiple Dose Effects
A02063	Acute Toxicity, Mutagenicity, Skin and Eye Irritation
A02064	Acute Toxicity, Mutagenicity, Skin and Eye Irritation, Reproductive Effects, Multiple Dose Effects
A02065	Acute Toxicity, Tumorigenicity
A02066	Acute Toxicity
A02067	Acute Toxicity, Skin and Eye Irritation, Multiple Dose Effects
A02068	Acute Toxicity, Multiple Dose Effects
A02069	Acute Toxicity, Mutagenicity
A02070	Acute Toxicity, Mutagenicity
A02071	Acute Toxicity, Mutagenicity, Reproductive Effects, Multiple Dose Effects
A02072	Acute Toxicity, Mutagenicity, Tumorigenicity, Multiple Dose Effects
A02073	Acute Toxicity, Mutagenicity
A02074	Acute Toxicity, Mutagenicity, Skin and Eye Irritation
A02075	Acute Toxicity, Mutagenicity, Tumorigenicity, Skin and Eye Irritation,
A02076	Acute Toxicity
A02077	Acute Toxicity, Mutagenicity, Multiple Dose Effects
A02078	Mutagenicity, Multiple Dose Effects
A02079	Acute Toxicity, Mutagenicity, Tumorigenicity, Multiple Dose Effects
A02080	Acute Toxicity, Tumorigenicity
A02081	Acute Toxicity, Mutagenicity, Skin and Eye Irritation

A02082	Acute Toxicity, Mutagenicity, Tumorigenicity, Skin and Eye Irritation, Multiple Dose Effects
A02083	Acute Toxicity, Mutagenicity, Tumorigenicity, Skin and Eye Irritation,
A02084	Acute Toxicity
A02085	Acute Toxicity, Tumorigenicity, Skin and Eye Irritation, Multiple Dose
A02086	Acute Toxicity, Tumorigenicity, Skin and Eye Irritation, Multiple Dose
A02087	Acute Toxicity
A02088	Acute Toxicity, Mutagenicity
A02089	Acute Toxicity
A02090	Acute Toxicity, Mutagenicity
A02091	Mutagenicity
A02092	Acute Toxicity, Tumorigenicity, Skin and Eye Irritation, Multiple Dose
A02093	Acute Toxicity, Mutagenicity, Tumorigenicity, Skin and Eye Irritation,
A02094	Mutagenicity
A02095	Acute Toxicity, Tumorigenicity, Reproductive Effects, Multiple Dose Effects
A02096	Acute Toxicity, Mutagenicity, Tumorigenicity, Multiple Dose Effects
A02097	Acute Toxicity, Skin and Eye Irritation, Multiple Dose Effects
A02098	Acute Toxicity
A02099	Tumorigenicity
A02100	Acute Toxicity, Mutagenicity, Skin and Eye Irritation
A02101	Mutagenicity
A02102	Acute Toxicity
A02103	Acute Toxicity
A02104	Acute Toxicity
A02105	Acute Toxicity
A02106	Acute Toxicity
A02107	Acute Toxicity, Mutagenicity
A02108	Acute Toxicity, Mutagenicity, Tumorigenicity, Reproductive Effects,
A02109	Acute Toxicity, Mutagenicity, Tumorigenicity, Reproductive Effects,
A02110	Acute Toxicity, Mutagenicity, Reproductive Effects, Multiple Dose Effects
A02111	Acute Toxicity, Mutagenicity
A02112	Acute Toxicity, Mutagenicity
A02113	Acute Toxicity, Skin and Eye Irritation
A02114	Acute Toxicity, Mutagenicity, Tumorigenicity, Skin and Eye Irritation,
A02115	Acute Toxicity
A02116	Acute Toxicity, Mutagenicity, Skin and Eye Irritation, Multiple Dose Effects
A02117	Acute Toxicity, Mutagenicity, Skin and Eye Irritation
A02118	Mutagenicity
A02119	Acute Toxicity, Mutagenicity, Tumorigenicity
A02120	Acute Toxicity, Skin and Eye Irritation
A02121	Acute Toxicity
A02122	Acute Toxicity, Skin and Eye Irritation, Multiple Dose Effects
A02123	Mutagenicity
A02124	Acute Toxicity
A02125	Acute Toxicity
A02126	Acute Toxicity

A02127	Acute Toxicity, Mutagenicity
A02128	Mutagenicity
A02129	Acute Toxicity, Mutagenicity, Skin and Eye Irritation, Reproductive Effects, Multiple Dose Effects
A02130	Acute Toxicity, Mutagenicity
A02131	Acute Toxicity, Mutagenicity
A02132	Acute Toxicity
A02133	Acute Toxicity
A02134	Acute Toxicity, Mutagenicity, Skin and Eye Irritation
A02135	Acute Toxicity, Skin and Eye Irritation, Multiple Dose Effects
A02136	Acute Toxicity, Skin and Eye Irritation
A02137	Acute Toxicity, Skin and Eye Irritation, Multiple Dose Effects
A02138	Acute Toxicity, Mutagenicity, Skin and Eye Irritation, Reproductive Effects
A02139	Acute Toxicity, Skin and Eye Irritation, Reproductive Effects
A02140	Acute Toxicity, Mutagenicity, Multiple Dose Effects
A02141	Acute Toxicity, Mutagenicity
A02142	Acute Toxicity, Mutagenicity, Tumorigenicity
A02143	Acute Toxicity, Mutagenicity, Skin and Eye Irritation, Reproductive Effects
A02144	Acute Toxicity, Mutagenicity
A02145	Acute Toxicity
A02146	Acute Toxicity, Mutagenicity, Tumorigenicity, Skin and Eye Irritation, Multiple Dose Effects
A02147	Acute Toxicity, Mutagenicity
A02148	Acute Toxicity, Mutagenicity, Skin and Eye Irritation, Reproductive Effects, Multiple Dose Effects
A02149	Skin and Eye Irritation
A02150	Acute Toxicity
A02151	Acute Toxicity
A02152	Acute Toxicity, Mutagenicity, Tumorigenicity, Skin and Eye Irritation, Multiple Dose Effects
A02153	Acute Toxicity
A02154	Acute Toxicity, Mutagenicity, Multiple Dose Effects
A02155	Acute Toxicity, Skin and Eye Irritation, Multiple Dose Effects
A02156	Acute Toxicity, Mutagenicity, Tumorigenicity, Skin and Eye Irritation,
A02157	Mutagenicity
A02158	Acute Toxicity
A02159	Acute Toxicity
A02160	Acute Toxicity
A02161	Acute Toxicity
A02162	Acute Toxicity, Skin and Eye Irritation
A02163	Mutagenicity
A02164	Acute Toxicity, Skin and Eye Irritation, Multiple Dose Effects
A02165	Acute Toxicity, Multiple Dose Effects
A02166	Acute Toxicity
A02167	Acute Toxicity, Mutagenicity, Tumorigenicity, Skin and Eye Irritation
A02168	Acute Toxicity

A02169	Acute Toxicity
A02170	Acute Toxicity, Reproductive Effects
A02171	Acute Toxicity
A02172	Acute Toxicity
A02173	Acute Toxicity, Mutagenicity, Multiple Dose Effects
A02174	Acute Toxicity, Mutagenicity, Multiple Dose Effects
A02175	Acute Toxicity, Mutagenicity, Tumorigenicity
A02176	Acute Toxicity
A02177	Acute Toxicity, Mutagenicity, Tumorigenicity, Multiple Dose Effects
A02178	Acute Toxicity, Mutagenicity
A02179	Acute Toxicity
A02180	Acute Toxicity, Mutagenicity, Tumorigenicity, Skin and Eye Irritation
A02181	Acute Toxicity, Mutagenicity, Tumorigenicity, Multiple Dose Effects
A02182	Acute Toxicity, Mutagenicity
A02183	Acute Toxicity
A02184	Mutagenicity
A02185	Acute Toxicity, Mutagenicity, Reproductive Effects, Multiple Dose Effects
A02186	Acute Toxicity, Mutagenicity, Tumorigenicity, Skin and Eye Irritation
A02187	Acute Toxicity, Mutagenicity
A02188	Acute Toxicity, Mutagenicity, Tumorigenicity
A02189	Acute Toxicity
A02190	Acute Toxicity, Mutagenicity, Tumorigenicity, Skin and Eye Irritation
A02191	Acute Toxicity, Mutagenicity, Skin and Eye Irritation
A02192	Acute Toxicity, Mutagenicity, Skin and Eye Irritation, Reproductive Effects, Multiple Dose Effects
A02193	Mutagenicity
A02194	Acute Toxicity, Mutagenicity, Tumorigenicity
A02195	Acute Toxicity, Mutagenicity, Tumorigenicity
A02196	Acute Toxicity
A02197	Acute Toxicity, Mutagenicity, Tumorigenicity
A02198	Mutagenicity, Tumorigenicity
A02199	Acute Toxicity, Mutagenicity, Tumorigenicity
A02200	Acute Toxicity
A02201	Acute Toxicity, Skin and Eye Irritation
A02202	Acute Toxicity, Mutagenicity, Multiple Dose Effects
A02203	Acute Toxicity
A02204	Acute Toxicity, Skin and Eye Irritation, Multiple Dose Effects
A02205	Mutagenicity
A02206	Acute Toxicity, Mutagenicity
A02207	Acute Toxicity, Multiple Dose Effects
A02208	Acute Toxicity, Mutagenicity
A02209	Acute Toxicity, Mutagenicity
A02210	Acute Toxicity, Mutagenicity, Multiple Dose Effects
A02211	Acute Toxicity, Mutagenicity
A02212	Acute Toxicity, Tumorigenicity, Multiple Dose Effects
A02213	Acute Toxicity

A02214	Acute Toxicity, Mutagenicity, Tumorigenicity, Skin and Eye Irritation
A02215	Acute Toxicity, Mutagenicity, Tumorigenicity, Skin and Eye Irritation, Multiple Dose Effects
A02216	Acute Toxicity, Mutagenicity, Skin and Eye Irritation
A02217	Mutagenicity, Tumorigenicity
A02218	Mutagenicity
A02219	Acute Toxicity, Mutagenicity, Skin and Eye Irritation
A02220	Mutagenicity
A02221	Acute Toxicity, Mutagenicity, Tumorigenicity, Multiple Dose Effects
A02222	Acute Toxicity, Mutagenicity, Tumorigenicity
A02223	Acute Toxicity, Mutagenicity, Multiple Dose Effects
A02224	Acute Toxicity
A02225	Acute Toxicity
A02226	Acute Toxicity
A02227	Acute Toxicity, Mutagenicity, Tumorigenicity, Reproductive Effects
A02228	Acute Toxicity, Mutagenicity, Tumorigenicity
A02229	Acute Toxicity, Mutagenicity, Skin and Eye Irritation
A02230	Acute Toxicity
A02231	Acute Toxicity, Mutagenicity, Tumorigenicity
A02232	Acute Toxicity, Mutagenicity, Multiple Dose Effects
A02233	Acute Toxicity
A02234	Acute Toxicity
A02235	Acute Toxicity, Mutagenicity, Skin and Eye Irritation
A02236	Acute Toxicity, Mutagenicity
A02237	Acute Toxicity, Mutagenicity, Skin and Eye Irritation
A02238	Acute Toxicity
A02239	Acute Toxicity, Mutagenicity
A02240	Acute Toxicity, Mutagenicity, Tumorigenicity, Skin and Eye Irritation,
A02241	Acute Toxicity, Mutagenicity, Skin and Eye Irritation
A02242	Acute Toxicity, Skin and Eye Irritation
A02243	Acute Toxicity, Mutagenicity
A02244	Acute Toxicity, Mutagenicity, Tumorigenicity, Skin and Eye Irritation
A02245	Acute Toxicity, Mutagenicity, Multiple Dose Effects
A02246	Mutagenicity
A02247	Acute Toxicity
A02248	Acute Toxicity
A02249	Acute Toxicity, Skin and Eye Irritation
A02250	Acute Toxicity, Skin and Eye Irritation, Multiple Dose Effects
A02251	Acute Toxicity, Mutagenicity, Skin and Eye Irritation, Multiple Dose Effects
A02252	Acute Toxicity
A02253	Acute Toxicity
A02254	Acute Toxicity, Mutagenicity, Reproductive Effects, Multiple Dose Effects
A02255	Acute Toxicity, Mutagenicity, Multiple Dose Effects
A02256	Acute Toxicity
A02257	Acute Toxicity, Mutagenicity, Tumorigenicity, Reproductive Effects,
A02258	Mutagenicity, Skin and Eye Irritation, Reproductive Effects

A02259	Reproductive Effects
A02260	Acute Toxicity
A02261	Acute Toxicity
A02262	Acute Toxicity
A02263	Acute Toxicity
A02264	Tumorigenicity
A02265	Acute Toxicity, Multiple Dose Effects
A02266	Acute Toxicity, Mutagenicity, Tumorigenicity, Reproductive Effects
A02267	Acute Toxicity
A02268	Acute Toxicity, Mutagenicity, Skin and Eye Irritation, Multiple Dose Effects
A02269	Acute Toxicity
A02270	Acute Toxicity
A02271	Acute Toxicity, Skin and Eye Irritation
A02272	Skin and Eye Irritation, Multiple Dose Effects
A02273	Acute Toxicity
A02274	Acute Toxicity
A02275	Acute Toxicity, Mutagenicity, Tumorigenicity, Skin and Eye Irritation, Multiple Dose Effects
A02276	Acute Toxicity, Skin and Eye Irritation
A02277	Acute Toxicity, Multiple Dose Effects
A02278	Acute Toxicity, Mutagenicity, Tumorigenicity, Skin and Eye Irritation, Multiple Dose Effects
A02279	Acute Toxicity, Skin and Eye Irritation
A02280	Acute Toxicity, Skin and Eye Irritation
A02281	Acute Toxicity
A02282	Acute Toxicity, Skin and Eye Irritation
A02283	Acute Toxicity, Mutagenicity, Tumorigenicity, Skin and Eye Irritation, Multiple Dose Effects
A02284	Acute Toxicity, Mutagenicity, Tumorigenicity, Skin and Eye Irritation,
A02285	Acute Toxicity, Mutagenicity, Tumorigenicity, Reproductive Effects,
A02286	Acute Toxicity, Mutagenicity
A02287	Acute Toxicity, Mutagenicity, Skin and Eye Irritation, Reproductive Effects, Multiple Dose Effects
A02288	Acute Toxicity
A02289	Acute Toxicity
A02290	Acute Toxicity, Mutagenicity, Tumorigenicity
A02291	Acute Toxicity, Multiple Dose Effects
A02292	Acute Toxicity, Mutagenicity, Skin and Eye Irritation, Multiple Dose Effects
A02293	Acute Toxicity
A02294	Acute Toxicity, Skin and Eye Irritation
A02295	Acute Toxicity, Skin and Eye Irritation, Multiple Dose Effects
A02296	Acute Toxicity, Mutagenicity, Tumorigenicity, Skin and Eye Irritation,
A02297	Acute Toxicity, Mutagenicity
A02298	Acute Toxicity, Mutagenicity, Skin and Eye Irritation, Reproductive Effects, Multiple Dose Effects
A02299	Acute Toxicity, Mutagenicity, Skin and Eye Irritation, Multiple Dose Effects

A02300	Acute Toxicity, Mutagenicity, Tumorigenicity, Skin and Eye Irritation,
A02301	Acute Toxicity, Mutagenicity, Multiple Dose Effects
A02302	Acute Toxicity
A02303	Acute Toxicity, Mutagenicity, Tumorigenicity, Skin and Eye Irritation,
A02304	Acute Toxicity, Mutagenicity, Tumorigenicity, Skin and Eye Irritation, Multiple Dose Effects
A02305	Acute Toxicity, Reproductive Effects, Multiple Dose Effects
A02306	Acute Toxicity, Mutagenicity, Tumorigenicity, Reproductive Effects,
A02307	Acute Toxicity, Mutagenicity, Multiple Dose Effects
A02308	Acute Toxicity, Mutagenicity
A02309	Acute Toxicity, Tumorigenicity, Multiple Dose Effects
A02310	Acute Toxicity, Mutagenicity, Multiple Dose Effects
A02311	Acute Toxicity, Mutagenicity, Tumorigenicity
A02312	Acute Toxicity, Mutagenicity, Skin and Eye Irritation, Multiple Dose Effects
A02313	Acute Toxicity, Tumorigenicity, Skin and Eye Irritation
A02314	Acute Toxicity, Multiple Dose Effects
A02315	Acute Toxicity, Mutagenicity, Tumorigenicity
A02316	Acute Toxicity, Mutagenicity, Skin and Eye Irritation, Multiple Dose Effects
A02317	Acute Toxicity, Mutagenicity
A02318	Acute Toxicity, Mutagenicity, Multiple Dose Effects
A02319	Acute Toxicity, Mutagenicity, Tumorigenicity
A02320	Acute Toxicity, Mutagenicity, Tumorigenicity, Skin and Eye Irritation,
A02321	Acute Toxicity, Mutagenicity, Multiple Dose Effects
A02322	Mutagenicity, Tumorigenicity, Skin and Eye Irritation, Multiple Dose Effects
A02323	Mutagenicity
A02324	Acute Toxicity, Mutagenicity, Skin and Eye Irritation
A02325	Acute Toxicity, Mutagenicity
A02326	Acute Toxicity, Tumorigenicity, Multiple Dose Effects
A02327	Acute Toxicity, Skin and Eye Irritation
A02328	Acute Toxicity, Mutagenicity, Multiple Dose Effects
A02329	Acute Toxicity, Mutagenicity, Multiple Dose Effects
A02330	Acute Toxicity, Mutagenicity, Tumorigenicity, Skin and Eye Irritation,
A02331	Acute Toxicity, Multiple Dose Effects
A02332	Acute Toxicity, Skin and Eye Irritation, Reproductive Effects, Multiple Dose
A02333	Acute Toxicity, Mutagenicity, Tumorigenicity, Skin and Eye Irritation,
A02334	Acute Toxicity, Mutagenicity, Reproductive Effects
A02335	Acute Toxicity, Mutagenicity, Tumorigenicity, Skin and Eye Irritation,
A02336	Acute Toxicity, Mutagenicity, Tumorigenicity, Skin and Eye Irritation, Multiple Dose Effects
A02337	Acute Toxicity, Mutagenicity, Skin and Eye Irritation
A02338	Acute Toxicity, Mutagenicity, Tumorigenicity, Skin and Eye Irritation,
A02339	Acute Toxicity, Mutagenicity
A02340	Acute Toxicity, Mutagenicity, Skin and Eye Irritation
A02341	Acute Toxicity, Mutagenicity, Tumorigenicity, Skin and Eye Irritation,
A02342	Acute Toxicity, Mutagenicity, Tumorigenicity, Skin and Eye Irritation,
A02343	Acute Toxicity, Mutagenicity, Reproductive Effects, Multiple Dose Effects

A02344	Acute Toxicity, Mutagenicity, Reproductive Effects
A02345	Acute Toxicity, Mutagenicity, Tumorigenicity, Skin and Eye Irritation,
A02346	Acute Toxicity, Mutagenicity
A02347	Acute Toxicity, Mutagenicity, Multiple Dose Effects
A02348	Acute Toxicity, Skin and Eye Irritation
A02349	Acute Toxicity, Mutagenicity, Skin and Eye Irritation
A02350	Acute Toxicity, Mutagenicity, Tumorigenicity, Skin and Eye Irritation,
A02351	Acute Toxicity, Mutagenicity, Tumorigenicity, Skin and Eye Irritation
A02352	Mutagenicity
A02353	Acute Toxicity, Mutagenicity, Multiple Dose Effects
A02354	Acute Toxicity
A02355	Acute Toxicity, Mutagenicity, Tumorigenicity, Reproductive Effects,
A02356	Acute Toxicity, Skin and Eye Irritation, Multiple Dose Effects
A02357	Acute Toxicity, Skin and Eye Irritation
A02358	Acute Toxicity, Multiple Dose Effects
A02359	Acute Toxicity, Mutagenicity
A02360	Acute Toxicity, Mutagenicity, Skin and Eye Irritation, Reproductive Effects
A02361	Acute Toxicity
A02362	Acute Toxicity, Tumorigenicity
A02363	Acute Toxicity, Mutagenicity, Multiple Dose Effects
A02364	Acute Toxicity, Mutagenicity, Skin and Eye Irritation
A02365	Acute Toxicity
A02366	Acute Toxicity, Mutagenicity, Multiple Dose Effects
A02367	Mutagenicity, Skin and Eye Irritation
A02368	Acute Toxicity, Mutagenicity
A02369	Acute Toxicity, Skin and Eye Irritation
A02370	Acute Toxicity, Skin and Eye Irritation
A02371	Acute Toxicity, Mutagenicity, Skin and Eye Irritation, Reproductive Effects, Multiple Dose Effects
A02372	Acute Toxicity, Skin and Eye Irritation
A02373	Acute Toxicity, Mutagenicity, Tumorigenicity, Skin and Eye Irritation,
A02374	Acute Toxicity, Mutagenicity, Skin and Eye Irritation, Multiple Dose Effects
A02375	Acute Toxicity, Mutagenicity, Skin and Eye Irritation, Reproductive Effects
A02376	Acute Toxicity, Mutagenicity, Skin and Eye Irritation, Reproductive Effects, Multiple Dose Effects
A02377	Acute Toxicity, Mutagenicity, Skin and Eye Irritation
A02378	Acute Toxicity, Skin and Eye Irritation
A02379	Acute Toxicity, Skin and Eye Irritation
A02380	Acute Toxicity, Mutagenicity, Skin and Eye Irritation, Reproductive Effects, Multiple Dose Effects
A02381	Acute Toxicity, Mutagenicity, Tumorigenicity, Skin and Eye Irritation, Multiple Dose Effects
A02382	Acute Toxicity, Mutagenicity, Tumorigenicity, Skin and Eye Irritation,
A02383	Acute Toxicity, Reproductive Effects, Multiple Dose Effects
A02384	Acute Toxicity, Skin and Eye Irritation
A02385	Acute Toxicity, Multiple Dose Effects

A02386	Acute Toxicity, Mutagenicity, Tumorigenicity, Multiple Dose Effects
A02387	Acute Toxicity, Mutagenicity, Multiple Dose Effects
A02388	Acute Toxicity
A02389	Acute Toxicity, Multiple Dose Effects
A02390	Acute Toxicity, Mutagenicity, Skin and Eye Irritation
A02391	Acute Toxicity, Mutagenicity
A02392	Acute Toxicity, Mutagenicity, Skin and Eye Irritation, Multiple Dose Effects
A02393	Acute Toxicity
A02394	Acute Toxicity
A02395	Acute Toxicity
A02396	Acute Toxicity
A02397	Acute Toxicity, Mutagenicity, Tumorigenicity, Skin and Eye Irritation
A02398	Mutagenicity
A02399	Acute Toxicity, Mutagenicity
A02400	Mutagenicity, Skin and Eye Irritation
A02401	Skin and Eye Irritation
A02402	Acute Toxicity, Mutagenicity, Skin and Eye Irritation, Multiple Dose Effects
A02403	Acute Toxicity, Multiple Dose Effects
A02404	Acute Toxicity, Mutagenicity
A02405	Acute Toxicity, Mutagenicity, Tumorigenicity, Skin and Eye Irritation, Multiple Dose Effects
A02406	Acute Toxicity, Mutagenicity, Multiple Dose Effects
A02407	Skin and Eye Irritation, Multiple Dose Effects
A02408	Acute Toxicity, Skin and Eye Irritation
A02409	Acute Toxicity
A02410	Acute Toxicity, Skin and Eye Irritation, Reproductive Effects, Multiple Dose
A02411	Acute Toxicity
A02412	Acute Toxicity, Mutagenicity, Skin and Eye Irritation, Multiple Dose Effects
A02413	Acute Toxicity, Mutagenicity, Skin and Eye Irritation, Multiple Dose Effects
A02414	Acute Toxicity
A02415	Acute Toxicity, Mutagenicity, Tumorigenicity, Skin and Eye Irritation, Multiple Dose Effects
A02416	Acute Toxicity, Mutagenicity, Skin and Eye Irritation, Reproductive Effects, Multiple Dose Effects
A02417	Acute Toxicity, Mutagenicity, Tumorigenicity
A02418	Acute Toxicity, Mutagenicity, Tumorigenicity, Skin and Eye Irritation
A02419	Acute Toxicity, Mutagenicity
A02420	Acute Toxicity, Mutagenicity
A02421	Acute Toxicity
A02422	Acute Toxicity, Mutagenicity, Skin and Eye Irritation
A02423	Acute Toxicity, Multiple Dose Effects
A02424	Acute Toxicity
A02425	Acute Toxicity, Reproductive Effects
A02426	Mutagenicity
A02427	Acute Toxicity, Mutagenicity, Skin and Eye Irritation, Reproductive Effects, Multiple Dose Effects

A02428	Acute Toxicity, Mutagenicity, Skin and Eye Irritation, Multiple Dose Effects
A02429	Acute Toxicity
A02430	Mutagenicity
A02431	Acute Toxicity, Tumorigenicity, Multiple Dose Effects
A02432	Acute Toxicity, Mutagenicity, Tumorigenicity, Multiple Dose Effects
A02433	Acute Toxicity
A02434	Mutagenicity
A02435	Mutagenicity, Skin and Eye Irritation
A02436	Acute Toxicity, Mutagenicity, Tumorigenicity, Skin and Eye Irritation, Multiple Dose Effects
A02437	Acute Toxicity, Mutagenicity
A02438	Mutagenicity, Tumorigenicity
A02439	Acute Toxicity, Skin and Eye Irritation
A02440	Acute Toxicity, Mutagenicity
A02441	Mutagenicity
A02442	Acute Toxicity
A02443	Acute Toxicity, Mutagenicity, Skin and Eye Irritation, Reproductive Effects, Multiple Dose Effects
A02444	Mutagenicity, Tumorigenicity
A02445	Acute Toxicity, Mutagenicity, Tumorigenicity, Multiple Dose Effects
A02446	Skin and Eye Irritation
A02447	Acute Toxicity, Mutagenicity
A02448	Acute Toxicity, Reproductive Effects, Multiple Dose Effects
A02449	Acute Toxicity, Skin and Eye Irritation, Multiple Dose Effects
A02450	Acute Toxicity, Mutagenicity, Skin and Eye Irritation, Reproductive Effects, Multiple Dose Effects
A02451	Acute Toxicity, Mutagenicity
A02452	Acute Toxicity, Mutagenicity, Tumorigenicity
A02453	Acute Toxicity, Mutagenicity, Skin and Eye Irritation
A02454	Acute Toxicity, Mutagenicity, Skin and Eye Irritation, Multiple Dose Effects
A02455	Acute Toxicity
A02456	Mutagenicity
A02457	Acute Toxicity, Skin and Eye Irritation
A02458	Acute Toxicity, Mutagenicity, Skin and Eye Irritation
A02459	Acute Toxicity, Mutagenicity, Skin and Eye Irritation, Reproductive Effects
A02460	Acute Toxicity, Mutagenicity, Skin and Eye Irritation, Multiple Dose Effects
A02461	Acute Toxicity
A02462	Acute Toxicity, Multiple Dose Effects
A02463	Acute Toxicity, Mutagenicity
A02464	Acute Toxicity, Reproductive Effects
A02465	Acute Toxicity, Reproductive Effects, Multiple Dose Effects
A02466	Acute Toxicity, Mutagenicity, Skin and Eye Irritation
A02467	Acute Toxicity, Mutagenicity, Multiple Dose Effects
A02468	Acute Toxicity, Mutagenicity, Tumorigenicity, Reproductive Effects,
A02469	Acute Toxicity, Mutagenicity, Tumorigenicity, Reproductive Effects,
A02470	Acute Toxicity, Mutagenicity, Tumorigenicity, Skin and Eye Irritation,

A02471	Acute Toxicity, Skin and Eye Irritation, Multiple Dose Effects
A02472	Acute Toxicity
A02473	Acute Toxicity, Multiple Dose Effects
A02474	Mutagenicity
A02475	Mutagenicity
A02476	Acute Toxicity, Mutagenicity, Multiple Dose Effects
A02477	Acute Toxicity, Mutagenicity, Multiple Dose Effects
A02478	Acute Toxicity, Multiple Dose Effects
A02479	Mutagenicity
A02480	Acute Toxicity, Mutagenicity, Reproductive Effects, Multiple Dose Effects
A02481	Acute Toxicity, Mutagenicity, Tumorigenicity, Skin and Eye Irritation,
A02482	Acute Toxicity, Mutagenicity
A02483	Acute Toxicity
A02484	Acute Toxicity, Mutagenicity
A02485	Acute Toxicity, Mutagenicity
A02486	Mutagenicity, Multiple Dose Effects
A02487	Acute Toxicity, Mutagenicity, Skin and Eye Irritation, Multiple Dose Effects
A02488	Acute Toxicity, Mutagenicity
A02489	Acute Toxicity, Mutagenicity, Tumorigenicity, Skin and Eye Irritation,
A02490	Acute Toxicity, Mutagenicity, Tumorigenicity, Skin and Eye Irritation,
A02491	Acute Toxicity, Mutagenicity, Tumorigenicity, Skin and Eye Irritation,
A02492	Acute Toxicity, Mutagenicity, Tumorigenicity, Skin and Eye Irritation
A02493	Acute Toxicity, Mutagenicity, Tumorigenicity, Reproductive Effects,
A02494	Acute Toxicity, Skin and Eye Irritation
A02495	Acute Toxicity, Mutagenicity, Skin and Eye Irritation, Multiple Dose Effects
A02496	Acute Toxicity
A02497	Acute Toxicity, Mutagenicity, Skin and Eye Irritation, Multiple Dose Effects
A02498	Acute Toxicity, Mutagenicity, Tumorigenicity, Skin and Eye Irritation
A02499	Acute Toxicity
A02500	Acute Toxicity
A02501	Acute Toxicity, Mutagenicity, Multiple Dose Effects
A02502	Acute Toxicity, Mutagenicity, Tumorigenicity, Reproductive Effects,
A02503	Acute Toxicity, Mutagenicity, Multiple Dose Effects
A02504	Acute Toxicity, Mutagenicity, Multiple Dose Effects
A02505	Acute Toxicity
A02506	Acute Toxicity, Mutagenicity, Tumorigenicity, Skin and Eye Irritation, Multiple Dose Effects
A02507	Acute Toxicity
A02508	Skin and Eye Irritation
A02509	Acute Toxicity, Skin and Eye Irritation
A02510	Acute Toxicity, Mutagenicity, Skin and Eye Irritation
A02511	Acute Toxicity, Mutagenicity, Tumorigenicity
A02512	Acute Toxicity, Skin and Eye Irritation
A02513	Acute Toxicity, Mutagenicity, Skin and Eye Irritation
A02514	Mutagenicity, Skin and Eye Irritation
A02515	Acute Toxicity

A02516	Acute Toxicity
A02517	Acute Toxicity
A02518	Acute Toxicity, Tumorigenicity, Skin and Eye Irritation
A02519	Acute Toxicity
A02520	Acute Toxicity, Mutagenicity, Skin and Eye Irritation, Multiple Dose Effects
A02521	Acute Toxicity, Mutagenicity, Tumorigenicity, Skin and Eye Irritation
A02522	Acute Toxicity
A02523	Acute Toxicity, Mutagenicity, Tumorigenicity, Multiple Dose Effects
A02524	Acute Toxicity, Mutagenicity, Skin and Eye Irritation, Reproductive Effects, Multiple Dose Effects
A02525	Acute Toxicity, Mutagenicity
A02526	Acute Toxicity, Multiple Dose Effects
A02527	Acute Toxicity
A02528	Acute Toxicity, Skin and Eye Irritation
A02529	Acute Toxicity, Mutagenicity, Multiple Dose Effects
A02530	Acute Toxicity, Multiple Dose Effects
A02531	Acute Toxicity, Mutagenicity, Tumorigenicity, Skin and Eye Irritation,
A02532	Acute Toxicity, Mutagenicity, Tumorigenicity
A02533	Mutagenicity
A02534	Acute Toxicity, Mutagenicity
A02535	Acute Toxicity, Mutagenicity, Skin and Eye Irritation, Reproductive Effects, Multiple Dose Effects
A02536	Acute Toxicity
A02537	Acute Toxicity, Mutagenicity, Skin and Eye Irritation, Reproductive Effects, Multiple Dose Effects
A02538	Acute Toxicity
A02539	Acute Toxicity, Mutagenicity, Tumorigenicity, Skin and Eye Irritation, Multiple Dose Effects
A02540	Mutagenicity, Tumorigenicity
A02541	Acute Toxicity, Mutagenicity, Tumorigenicity, Skin and Eye Irritation,
A02542	Acute Toxicity
A02543	Acute Toxicity
A02544	Acute Toxicity, Mutagenicity, Tumorigenicity, Skin and Eye Irritation
A02545	Acute Toxicity, Mutagenicity, Skin and Eye Irritation, Reproductive Effects, Multiple Dose Effects
A02546	Acute Toxicity, Mutagenicity, Tumorigenicity, Skin and Eye Irritation, Multiple Dose Effects
A02547	Acute Toxicity, Mutagenicity
A02548	Acute Toxicity, Tumorigenicity
A02549	Acute Toxicity, Mutagenicity, Multiple Dose Effects
A02550	Acute Toxicity
A02551	Acute Toxicity, Mutagenicity
A02552	Acute Toxicity, Tumorigenicity
A02553	Acute Toxicity
A02554	Acute Toxicity
A02555	Acute Toxicity, Mutagenicity, Skin and Eye Irritation, Multiple Dose Effects

A02556	Acute Toxicity, Mutagenicity, Skin and Eye Irritation
A02557	Acute Toxicity
A02558	Acute Toxicity
A02559	Acute Toxicity, Multiple Dose Effects
A02560	Acute Toxicity
A02561	Acute Toxicity, Mutagenicity, Multiple Dose Effects
A02562	Acute Toxicity
A02563	Acute Toxicity, Mutagenicity, Skin and Eye Irritation, Multiple Dose Effects
A02564	Acute Toxicity, Mutagenicity, Tumorigenicity, Skin and Eye Irritation, Multiple Dose Effects
A02565	Acute Toxicity, Mutagenicity, Tumorigenicity, Skin and Eye Irritation,
A02566	Acute Toxicity
A02567	Acute Toxicity, Mutagenicity, Skin and Eye Irritation, Multiple Dose Effects
A02568	Acute Toxicity, Mutagenicity, Multiple Dose Effects
A02569	Acute Toxicity, Skin and Eye Irritation
A02570	Acute Toxicity
A02571	Acute Toxicity, Mutagenicity, Tumorigenicity, Skin and Eye Irritation, Multiple Dose Effects
A02572	Acute Toxicity, Tumorigenicity, Skin and Eye Irritation
A02573	Acute Toxicity, Mutagenicity
A02574	Acute Toxicity, Mutagenicity, Tumorigenicity, Reproductive Effects,
A02575	Acute Toxicity
A02576	Acute Toxicity, Mutagenicity, Skin and Eye Irritation, Multiple Dose Effects
A02577	Acute Toxicity
A02578	Acute Toxicity, Skin and Eye Irritation, Multiple Dose Effects
A02579	Acute Toxicity, Mutagenicity, Skin and Eye Irritation
A02580	Acute Toxicity, Skin and Eye Irritation
A02581	Acute Toxicity
A02582	Acute Toxicity, Multiple Dose Effects
A02583	Acute Toxicity, Mutagenicity
A02584	Acute Toxicity, Mutagenicity
A02585	Acute Toxicity, Mutagenicity, Multiple Dose Effects
A02586	Acute Toxicity, Mutagenicity, Tumorigenicity, Reproductive Effects,
A02587	Acute Toxicity
A02588	Acute Toxicity
A02589	Acute Toxicity, Mutagenicity, Skin and Eye Irritation, Multiple Dose Effects
A02590	Acute Toxicity
A02591	Mutagenicity
A02592	Acute Toxicity
A02593	Acute Toxicity, Mutagenicity, Skin and Eye Irritation, Multiple Dose Effects
A02594	Acute Toxicity
A02595	Acute Toxicity, Mutagenicity
A02596	Acute Toxicity
A02597	Acute Toxicity, Tumorigenicity, Skin and Eye Irritation
A02598	Acute Toxicity, Mutagenicity
A02599	Acute Toxicity, Skin and Eye Irritation, Multiple Dose Effects

A02600	Acute Toxicity, Multiple Dose Effects
A02601	Acute Toxicity, Skin and Eye Irritation
A02602	Acute Toxicity, Multiple Dose Effects
A02603	Acute Toxicity, Mutagenicity
A02604	Acute Toxicity, Skin and Eye Irritation
A02605	Acute Toxicity
A02606	Acute Toxicity, Skin and Eye Irritation
A02607	Acute Toxicity, Mutagenicity
A02608	Acute Toxicity, Mutagenicity, Skin and Eye Irritation
A02609	Acute Toxicity, Mutagenicity, Skin and Eye Irritation
A02610	Acute Toxicity, Mutagenicity, Skin and Eye Irritation
A02611	Acute Toxicity, Mutagenicity, Tumorigenicity, Skin and Eye Irritation,
A02612	Acute Toxicity, Mutagenicity
A02613	Acute Toxicity, Mutagenicity, Multiple Dose Effects
A02614	Acute Toxicity, Mutagenicity, Skin and Eye Irritation
A02615	Acute Toxicity, Mutagenicity, Skin and Eye Irritation
A02616	Acute Toxicity, Mutagenicity
A02617	Acute Toxicity, Mutagenicity, Skin and Eye Irritation
A02618	Acute Toxicity, Mutagenicity
A02619	Acute Toxicity
A02620	Acute Toxicity, Skin and Eye Irritation, Multiple Dose Effects
A02621	Acute Toxicity, Mutagenicity, Multiple Dose Effects
A02622	Acute Toxicity, Multiple Dose Effects
A02623	Acute Toxicity, Multiple Dose Effects
A02624	Acute Toxicity, Mutagenicity, Skin and Eye Irritation, Multiple Dose Effects
A02625	Acute Toxicity
A02626	Non-toxicity
A02627	Acute Toxicity, Skin and Eye Irritation
A02628	Acute Toxicity
A02629	Mutagenicity, Tumorigenicity
A02630	Acute Toxicity, Skin and Eye Irritation
A02631	Acute Toxicity, Mutagenicity, Skin and Eye Irritation
A02632	Acute Toxicity, Mutagenicity, Tumorigenicity, Skin and Eye Irritation
A02633	Acute Toxicity, Mutagenicity, Tumorigenicity, Reproductive Effects
A02634	Acute Toxicity, Mutagenicity, Skin and Eye Irritation
A02635	Acute Toxicity
A02636	Acute Toxicity, Skin and Eye Irritation
A02637	Acute Toxicity, Mutagenicity, Skin and Eye Irritation
A02638	Acute Toxicity
A02639	Acute Toxicity, Skin and Eye Irritation
A02640	Acute Toxicity, Mutagenicity, Tumorigenicity, Reproductive Effects
A02641	Acute Toxicity, Mutagenicity, Skin and Eye Irritation, Reproductive Effects, Multiple Dose Effects
A02642	Acute Toxicity, Mutagenicity, Tumorigenicity, Skin and Eye Irritation,
A02643	Acute Toxicity
A02644	Acute Toxicity, Mutagenicity, Tumorigenicity, Multiple Dose Effects

A02645	Acute Toxicity
A02646	Acute Toxicity, Mutagenicity, Tumorigenicity, Skin and Eye Irritation
A02647	Acute Toxicity, Skin and Eye Irritation
A02648	Acute Toxicity, Skin and Eye Irritation
A02649	Acute Toxicity, Mutagenicity, Reproductive Effects, Multiple Dose Effects
A02650	Acute Toxicity, Skin and Eye Irritation
A02651	Mutagenicity, Tumorigenicity
A02652	Acute Toxicity, Mutagenicity, Skin and Eye Irritation
A02653	Acute Toxicity, Skin and Eye Irritation
A02654	Skin and Eye Irritation
A02655	Acute Toxicity, Mutagenicity
A02656	Acute Toxicity, Mutagenicity, Skin and Eye Irritation
A02657	Acute Toxicity, Mutagenicity, Skin and Eye Irritation
A02658	Acute Toxicity, Multiple Dose Effects
A02659	Acute Toxicity, Skin and Eye Irritation
A02660	Acute Toxicity, Mutagenicity
A02661	Acute Toxicity, Skin and Eye Irritation, Multiple Dose Effects
A02662	Acute Toxicity, Skin and Eye Irritation, Multiple Dose Effects
A02663	Acute Toxicity, Mutagenicity, Multiple Dose Effects
A02664	Acute Toxicity, Mutagenicity
A02665	Mutagenicity
A02666	Acute Toxicity, Mutagenicity
A02667	Acute Toxicity, Tumorigenicity, Reproductive Effects
A02668	Acute Toxicity, Mutagenicity, Skin and Eye Irritation, Reproductive Effects, Multiple Dose Effects
A02669	Acute Toxicity, Mutagenicity, Multiple Dose Effects
A02670	Acute Toxicity
A02671	Acute Toxicity, Mutagenicity, Tumorigenicity, Skin and Eye Irritation, Multiple Dose Effects
A02672	Acute Toxicity, Mutagenicity, Tumorigenicity, Skin and Eye Irritation, Multiple Dose Effects
A02673	Acute Toxicity, Mutagenicity, Tumorigenicity, Skin and Eye Irritation, Multiple Dose Effects
A02674	Acute Toxicity, Tumorigenicity, Skin and Eye Irritation
A02675	Acute Toxicity
A02676	Acute Toxicity, Mutagenicity
A02677	Acute Toxicity, Mutagenicity, Skin and Eye Irritation
A02678	Acute Toxicity, Mutagenicity, Skin and Eye Irritation, Multiple Dose Effects
A02679	Acute Toxicity, Skin and Eye Irritation
A02680	Acute Toxicity, Skin and Eye Irritation
A02681	Acute Toxicity
A02682	Acute Toxicity
A02683	Acute Toxicity, Skin and Eye Irritation
A02684	Acute Toxicity, Skin and Eye Irritation, Reproductive Effects, Multiple Dose
A02685	Acute Toxicity, Mutagenicity, Tumorigenicity, Skin and Eye Irritation,
A02686	Acute Toxicity, Mutagenicity, Tumorigenicity, Skin and Eye Irritation,

A02687	Acute Toxicity, Mutagenicity, Tumorigenicity, Skin and Eye Irritation,
A02688	Acute Toxicity, Mutagenicity, Skin and Eye Irritation
A02689	Acute Toxicity, Mutagenicity, Skin and Eye Irritation, Reproductive Effects, Multiple Dose Effects
A02690	Acute Toxicity, Mutagenicity, Tumorigenicity, Skin and Eye Irritation, Multiple Dose Effects
A02691	Acute Toxicity, Mutagenicity, Tumorigenicity, Skin and Eye Irritation,
A02692	Acute Toxicity, Mutagenicity, Reproductive Effects, Multiple Dose Effects
A02693	Acute Toxicity, Mutagenicity
A02694	Acute Toxicity
A02695	Acute Toxicity, Mutagenicity
A02696	Acute Toxicity, Mutagenicity
A02697	Acute Toxicity, Mutagenicity, Tumorigenicity, Reproductive Effects,
A02698	Acute Toxicity, Mutagenicity, Tumorigenicity, Skin and Eye Irritation,
A02699	Acute Toxicity, Mutagenicity, Skin and Eye Irritation
A02700	Acute Toxicity, Mutagenicity
A02701	Acute Toxicity, Mutagenicity, Tumorigenicity, Skin and Eye Irritation,
A02702	Acute Toxicity, Mutagenicity, Multiple Dose Effects
A02703	Acute Toxicity, Skin and Eye Irritation, Multiple Dose Effects
A02704	Acute Toxicity, Mutagenicity, Skin and Eye Irritation, Multiple Dose Effects
A02705	Acute Toxicity, Mutagenicity, Skin and Eye Irritation, Reproductive Effects, Multiple Dose Effects
A02706	Acute Toxicity, Mutagenicity, Tumorigenicity, Skin and Eye Irritation,
A02707	Acute Toxicity, Mutagenicity, Tumorigenicity, Skin and Eye Irritation,
A02708	Acute Toxicity, Mutagenicity, Skin and Eye Irritation
A02709	Acute Toxicity, Mutagenicity, Tumorigenicity, Skin and Eye Irritation,
A02710	Acute Toxicity, Mutagenicity, Multiple Dose Effects
A02711	Acute Toxicity, Mutagenicity, Tumorigenicity, Skin and Eye Irritation, Multiple Dose Effects
A02712	Acute Toxicity, Mutagenicity, Reproductive Effects, Multiple Dose Effects
A02713	Acute Toxicity, Mutagenicity, Tumorigenicity, Multiple Dose Effects
A02714	Acute Toxicity, Mutagenicity
A02715	Acute Toxicity
A02716	Mutagenicity
A02717	Acute Toxicity, Skin and Eye Irritation
A02718	Acute Toxicity, Mutagenicity, Multiple Dose Effects
A02719	Acute Toxicity
A02720	Acute Toxicity
A02721	Acute Toxicity
A02722	Acute Toxicity
A02723	Acute Toxicity, Skin and Eye Irritation, Reproductive Effects, Multiple Dose
A02724	Mutagenicity
A02725	Acute Toxicity, Mutagenicity, Tumorigenicity
A02726	Acute Toxicity, Mutagenicity, Skin and Eye Irritation
A02727	Acute Toxicity, Skin and Eye Irritation, Multiple Dose Effects
A02728	Acute Toxicity

A02729	Multiple Dose Effects
A02730	Acute Toxicity, Mutagenicity, Skin and Eye Irritation, Multiple Dose Effects
A02731	Acute Toxicity, Skin and Eye Irritation, Reproductive Effects, Multiple Dose
A02732	Acute Toxicity, Skin and Eye Irritation
A02733	Acute Toxicity
A02734	Acute Toxicity, Mutagenicity, Tumorigenicity
A02735	Acute Toxicity, Skin and Eye Irritation, Reproductive Effects, Multiple Dose
A02736	Acute Toxicity, Mutagenicity
A02737	Acute Toxicity, Mutagenicity, Tumorigenicity, Skin and Eye Irritation, Multiple Dose Effects
A02738	Acute Toxicity, Mutagenicity, Tumorigenicity, Skin and Eye Irritation, Multiple Dose Effects
A02739	Acute Toxicity, Mutagenicity, Tumorigenicity, Skin and Eye Irritation,
A02740	Acute Toxicity, Mutagenicity, Skin and Eye Irritation
A02741	Acute Toxicity, Mutagenicity, Tumorigenicity, Skin and Eye Irritation,
A02742	Acute Toxicity, Mutagenicity
A02743	Acute Toxicity
A02744	Acute Toxicity, Mutagenicity, Multiple Dose Effects
A02745	Acute Toxicity, Skin and Eye Irritation, Reproductive Effects, Multiple Dose
A02746	Acute Toxicity, Mutagenicity, Skin and Eye Irritation
A02747	Acute Toxicity, Mutagenicity, Skin and Eye Irritation
A02748	Acute Toxicity, Skin and Eye Irritation, Multiple Dose Effects
A02749	Acute Toxicity, Mutagenicity, Skin and Eye Irritation, Multiple Dose Effects
A02750	Acute Toxicity
A02751	Acute Toxicity, Mutagenicity, Tumorigenicity, Skin and Eye Irritation, Multiple Dose Effects
A02752	Acute Toxicity, Mutagenicity, Tumorigenicity, Skin and Eye Irritation,
A02753	Acute Toxicity, Mutagenicity, Tumorigenicity, Skin and Eye Irritation,
A02754	Acute Toxicity, Skin and Eye Irritation, Reproductive Effects, Multiple Dose
A02755	Acute Toxicity
A02756	Acute Toxicity, Mutagenicity, Skin and Eye Irritation, Reproductive Effects, Multiple Dose Effects
A02757	Acute Toxicity
A02758	Acute Toxicity, Mutagenicity, Multiple Dose Effects
A02759	Acute Toxicity, Mutagenicity, Tumorigenicity
A02760	Acute Toxicity, Mutagenicity, Skin and Eye Irritation, Multiple Dose Effects
A02761	Acute Toxicity, Mutagenicity, Tumorigenicity, Skin and Eye Irritation,
A02762	Acute Toxicity, Mutagenicity
A02763	Acute Toxicity, Mutagenicity
A02764	Acute Toxicity, Mutagenicity
A02765	Acute Toxicity
A02766	Acute Toxicity
A02767	Skin and Eye Irritation
A02768	Acute Toxicity, Mutagenicity, Skin and Eye Irritation
A02769	Acute Toxicity, Mutagenicity, Skin and Eye Irritation
A02770	Acute Toxicity, Multiple Dose Effects

A02771	Acute Toxicity, Mutagenicity, Skin and Eye Irritation, Multiple Dose Effects
A02772	Acute Toxicity, Mutagenicity, Tumorigenicity, Skin and Eye Irritation,
A02773	Acute Toxicity, Mutagenicity
A02774	Acute Toxicity, Mutagenicity, Skin and Eye Irritation, Multiple Dose Effects
A02775	Acute Toxicity
A02776	Acute Toxicity
A02777	Acute Toxicity, Tumorigenicity, Skin and Eye Irritation, Multiple Dose
A02778	Acute Toxicity, Mutagenicity, Tumorigenicity, Skin and Eye Irritation,
A02779	Acute Toxicity, Mutagenicity, Tumorigenicity, Skin and Eye Irritation, Multiple Dose Effects
A02780	Acute Toxicity, Mutagenicity, Skin and Eye Irritation, Multiple Dose Effects
A02781	Acute Toxicity, Skin and Eye Irritation
A02782	Acute Toxicity
A02783	Acute Toxicity, Skin and Eye Irritation, Multiple Dose Effects
A02784	Acute Toxicity, Mutagenicity, Skin and Eye Irritation
A02785	Acute Toxicity, Mutagenicity, Tumorigenicity, Reproductive Effects,
A02786	Acute Toxicity, Mutagenicity, Skin and Eye Irritation, Reproductive Effects, Multiple Dose Effects
A02787	Acute Toxicity, Mutagenicity, Tumorigenicity, Skin and Eye Irritation,
A02788	Acute Toxicity, Mutagenicity, Tumorigenicity, Skin and Eye Irritation,
A02789	Acute Toxicity, Mutagenicity, Skin and Eye Irritation, Reproductive Effects, Multiple Dose Effects
A02790	Acute Toxicity, Mutagenicity, Skin and Eye Irritation, Multiple Dose Effects
A02791	Acute Toxicity
A02792	Acute Toxicity, Mutagenicity, Skin and Eye Irritation, Reproductive Effects
A02793	Acute Toxicity
A02794	Acute Toxicity
A02795	Acute Toxicity, Mutagenicity
A02796	Acute Toxicity, Mutagenicity
A02797	Acute Toxicity, Mutagenicity
A02798	Acute Toxicity, Mutagenicity, Skin and Eye Irritation, Reproductive Effects, Multiple Dose Effects
A02799	Acute Toxicity, Mutagenicity, Skin and Eye Irritation
A02800	Acute Toxicity, Mutagenicity, Skin and Eye Irritation
A02801	Acute Toxicity, Skin and Eye Irritation
A02802	Acute Toxicity, Mutagenicity, Skin and Eye Irritation, Reproductive Effects, Multiple Dose Effects
A02803	Acute Toxicity, Reproductive Effects
A02804	Acute Toxicity
A02805	Acute Toxicity, Mutagenicity, Skin and Eye Irritation, Multiple Dose Effects
A02806	Acute Toxicity, Reproductive Effects, Multiple Dose Effects
A02807	Acute Toxicity, Skin and Eye Irritation, Multiple Dose Effects
A02808	Acute Toxicity, Skin and Eye Irritation
A02809	Acute Toxicity
A02810	Acute Toxicity, Mutagenicity, Multiple Dose Effects
A02811	Acute Toxicity, Mutagenicity

A02812	Acute Toxicity, Multiple Dose Effects
A02813	Acute Toxicity, Multiple Dose Effects
A02814	Acute Toxicity, Mutagenicity, Tumorigenicity, Reproductive Effects,
A02815	Acute Toxicity, Mutagenicity
A02816	Acute Toxicity, Mutagenicity, Tumorigenicity, Skin and Eye Irritation,
A02817	Acute Toxicity, Skin and Eye Irritation, Reproductive Effects
A02818	Acute Toxicity, Skin and Eye Irritation, Reproductive Effects, Multiple Dose
A02819	Acute Toxicity, Mutagenicity, Skin and Eye Irritation, Multiple Dose Effects
A02820	Acute Toxicity, Mutagenicity, Skin and Eye Irritation, Multiple Dose Effects
A02821	Acute Toxicity, Skin and Eye Irritation, Reproductive Effects, Multiple Dose
A02822	Acute Toxicity
A02823	Acute Toxicity
A02824	Acute Toxicity
A02825	Acute Toxicity, Mutagenicity, Skin and Eye Irritation
A02826	Acute Toxicity, Mutagenicity, Tumorigenicity
A02827	Acute Toxicity, Skin and Eye Irritation
A02828	Acute Toxicity, Mutagenicity, Skin and Eye Irritation, Reproductive Effects, Multiple Dose Effects
A02829	Acute Toxicity, Skin and Eye Irritation, Multiple Dose Effects
A02830	Acute Toxicity, Mutagenicity, Skin and Eye Irritation, Multiple Dose Effects
A02831	Acute Toxicity, Mutagenicity
A02832	Acute Toxicity, Mutagenicity, Skin and Eye Irritation
A02833	Acute Toxicity, Mutagenicity
A02834	Acute Toxicity, Tumorigenicity, Skin and Eye Irritation, Multiple Dose
A02835	Acute Toxicity
A02836	Acute Toxicity, Mutagenicity
A02837	Acute Toxicity, Mutagenicity, Tumorigenicity, Reproductive Effects,
A02838	Acute Toxicity, Mutagenicity, Tumorigenicity, Reproductive Effects,
A02839	Acute Toxicity, Mutagenicity, Tumorigenicity, Skin and Eye Irritation,
A02840	Acute Toxicity, Skin and Eye Irritation, Multiple Dose Effects
A02841	Acute Toxicity, Mutagenicity, Skin and Eye Irritation, Reproductive Effects, Multiple Dose Effects
A02842	Acute Toxicity, Mutagenicity, Skin and Eye Irritation, Multiple Dose Effects
A02843	Acute Toxicity, Skin and Eye Irritation
A02844	Acute Toxicity, Mutagenicity
A02845	Tumorigenicity, Skin and Eye Irritation
A02846	Acute Toxicity, Mutagenicity, Skin and Eye Irritation, Reproductive Effects, Multiple Dose Effects
A02847	Acute Toxicity, Skin and Eye Irritation
A02848	Mutagenicity, Skin and Eye Irritation
A02849	Acute Toxicity
A02850	Acute Toxicity, Skin and Eye Irritation
A02851	Skin and Eye Irritation
A02852	Acute Toxicity, Skin and Eye Irritation
A02853	Acute Toxicity, Tumorigenicity

A02854	Acute Toxicity, Mutagenicity, Skin and Eye Irritation, Reproductive Effects, Multiple Dose Effects
A02855	Acute Toxicity, Mutagenicity, Multiple Dose Effects
A02856	Acute Toxicity, Mutagenicity, Skin and Eye Irritation, Reproductive Effects
A02857	Acute Toxicity, Mutagenicity
A02858	Acute Toxicity
A02859	Acute Toxicity, Mutagenicity, Tumorigenicity, Skin and Eye Irritation,
A02860	Acute Toxicity, Mutagenicity, Skin and Eye Irritation
A02861	Acute Toxicity, Mutagenicity, Multiple Dose Effects
A02862	Acute Toxicity, Mutagenicity, Reproductive Effects
A02863	Acute Toxicity, Mutagenicity, Skin and Eye Irritation
A02864	Acute Toxicity, Mutagenicity, Multiple Dose Effects
A02865	Acute Toxicity, Skin and Eye Irritation, Reproductive Effects, Multiple Dose
A02866	Acute Toxicity
A02867	Acute Toxicity, Skin and Eye Irritation
A02868	Acute Toxicity
A02869	Acute Toxicity, Skin and Eye Irritation, Reproductive Effects, Multiple Dose
A02870	Acute Toxicity
A02871	Skin and Eye Irritation
A02872	Acute Toxicity, Mutagenicity, Skin and Eye Irritation, Reproductive Effects, Multiple Dose Effects
A02873	Acute Toxicity, Skin and Eye Irritation, Multiple Dose Effects
A02874	Acute Toxicity, Mutagenicity, Tumorigenicity, Skin and Eye Irritation,
A02875	Acute Toxicity, Mutagenicity, Multiple Dose Effects
A02876	Acute Toxicity, Mutagenicity, Reproductive Effects, Multiple Dose Effects
A02877	Acute Toxicity, Mutagenicity, Tumorigenicity, Skin and Eye Irritation,
A02878	Acute Toxicity, Mutagenicity, Tumorigenicity, Skin and Eye Irritation, Multiple Dose Effects
A02879	Acute Toxicity, Multiple Dose Effects
A02880	Acute Toxicity, Mutagenicity
A02881	Acute Toxicity, Mutagenicity, Tumorigenicity, Skin and Eye Irritation, Multiple Dose Effects
A02882	Acute Toxicity
A02883	Skin and Eye Irritation
A02884	Acute Toxicity
A02885	Acute Toxicity, Skin and Eye Irritation
A02886	Acute Toxicity, Skin and Eye Irritation, Reproductive Effects
A02887	Acute Toxicity, Mutagenicity, Reproductive Effects, Multiple Dose Effects
A02888	Acute Toxicity, Mutagenicity, Skin and Eye Irritation, Reproductive Effects, Multiple Dose Effects
A02889	Acute Toxicity, Tumorigenicity, Skin and Eye Irritation
A02890	Acute Toxicity, Skin and Eye Irritation
A02891	Acute Toxicity, Skin and Eye Irritation, Reproductive Effects
A02892	Acute Toxicity, Multiple Dose Effects
A02893	Mutagenicity
A02894	Acute Toxicity

A02895	Acute Toxicity, Skin and Eye Irritation
A02896	Acute Toxicity, Mutagenicity, Skin and Eye Irritation, Reproductive Effects, Multiple Dose Effects
A02897	Acute Toxicity, Skin and Eye Irritation
A02898	Acute Toxicity
A02899	Acute Toxicity, Skin and Eye Irritation, Multiple Dose Effects
A02900	Acute Toxicity, Skin and Eye Irritation, Reproductive Effects
A02901	Acute Toxicity, Multiple Dose Effects
A02902	Acute Toxicity, Mutagenicity, Skin and Eye Irritation, Multiple Dose Effects
A02903	Acute Toxicity, Mutagenicity, Skin and Eye Irritation, Reproductive Effects, Multiple Dose Effects
A02904	Acute Toxicity, Mutagenicity, Tumorigenicity, Skin and Eye Irritation,
A02905	Acute Toxicity
A02906	Acute Toxicity, Mutagenicity, Tumorigenicity, Skin and Eye Irritation, Multiple Dose Effects
A02907	Acute Toxicity, Skin and Eye Irritation, Multiple Dose Effects
A02908	Acute Toxicity, Mutagenicity, Tumorigenicity, Skin and Eye Irritation,
A02909	Tumorigenicity
A02910	Acute Toxicity, Mutagenicity, Skin and Eye Irritation
A02911	Acute Toxicity, Skin and Eye Irritation, Multiple Dose Effects
A02912	Acute Toxicity, Skin and Eye Irritation
A02913	Mutagenicity
A02914	Acute Toxicity, Tumorigenicity
A02915	Acute Toxicity, Mutagenicity, Skin and Eye Irritation, Reproductive Effects, Multiple Dose Effects
A02916	Acute Toxicity, Mutagenicity, Multiple Dose Effects
A02917	Acute Toxicity, Mutagenicity, Skin and Eye Irritation, Multiple Dose Effects
A02918	Acute Toxicity, Mutagenicity, Tumorigenicity, Skin and Eye Irritation,
A02919	Acute Toxicity, Skin and Eye Irritation, Reproductive Effects, Multiple Dose
A02920	Acute Toxicity, Skin and Eye Irritation
A02921	Acute Toxicity, Mutagenicity
A02922	Acute Toxicity, Mutagenicity, Skin and Eye Irritation, Multiple Dose Effects
A02923	Mutagenicity
A02924	Acute Toxicity, Skin and Eye Irritation
A02925	Acute Toxicity, Mutagenicity, Skin and Eye Irritation, Reproductive Effects, Multiple Dose Effects
A02926	Acute Toxicity, Skin and Eye Irritation, Multiple Dose Effects
A02927	Acute Toxicity, Mutagenicity, Skin and Eye Irritation, Multiple Dose Effects
A02928	Acute Toxicity, Mutagenicity, Skin and Eye Irritation, Reproductive Effects, Multiple Dose Effects
A02929	Acute Toxicity, Mutagenicity, Reproductive Effects, Multiple Dose Effects
A02930	Acute Toxicity
A02931	Acute Toxicity, Mutagenicity, Skin and Eye Irritation, Reproductive Effects, Multiple Dose Effects
A02932	Acute Toxicity, Skin and Eye Irritation
A02933	Acute Toxicity, Skin and Eye Irritation, Multiple Dose Effects

A02934	Acute Toxicity, Skin and Eye Irritation
A02935	Acute Toxicity, Reproductive Effects
A02936	Acute Toxicity, Skin and Eye Irritation
A02937	Acute Toxicity, Skin and Eye Irritation, Multiple Dose Effects
A02938	Acute Toxicity
A02939	Skin and Eye Irritation
A02940	Skin and Eye Irritation
A02941	Acute Toxicity, Skin and Eye Irritation, Multiple Dose Effects
A02942	Acute Toxicity, Mutagenicity, Tumorigenicity, Skin and Eye Irritation,
A02943	Acute Toxicity
A02944	Acute Toxicity, Mutagenicity, Tumorigenicity, Skin and Eye Irritation,
A02945	Acute Toxicity, Mutagenicity, Skin and Eye Irritation
A02946	Acute Toxicity, Skin and Eye Irritation
A02947	Acute Toxicity, Mutagenicity, Skin and Eye Irritation, Multiple Dose Effects
A02948	Acute Toxicity, Mutagenicity, Skin and Eye Irritation, Reproductive Effects, Multiple Dose Effects
A02949	Acute Toxicity, Skin and Eye Irritation, Reproductive Effects, Multiple Dose
A02950	Acute Toxicity, Skin and Eye Irritation
A02951	Acute Toxicity, Tumorigenicity, Skin and Eye Irritation, Multiple Dose
A02952	Acute Toxicity
A02953	Acute Toxicity, Mutagenicity, Skin and Eye Irritation
A02954	Acute Toxicity, Skin and Eye Irritation
A02955	Skin and Eye Irritation
A02956	Acute Toxicity, Skin and Eye Irritation
A02957	Acute Toxicity, Skin and Eye Irritation
A02958	Acute Toxicity, Mutagenicity, Reproductive Effects, Multiple Dose Effects
A02959	Acute Toxicity, Skin and Eye Irritation, Multiple Dose Effects
A02960	Mutagenicity
A02961	Acute Toxicity, Mutagenicity, Tumorigenicity, Skin and Eye Irritation
A02962	Acute Toxicity, Skin and Eye Irritation
A02963	Mutagenicity, Skin and Eye Irritation
A02964	Acute Toxicity, Mutagenicity, Multiple Dose Effects
A02965	Acute Toxicity, Mutagenicity, Skin and Eye Irritation
A02966	Acute Toxicity, Mutagenicity, Skin and Eye Irritation
A02967	Acute Toxicity, Mutagenicity, Skin and Eye Irritation, Multiple Dose Effects
A02968	Acute Toxicity, Mutagenicity, Skin and Eye Irritation, Reproductive Effects, Multiple Dose Effects
A02969	Tumorigenicity
A02970	Skin and Eye Irritation
A02971	Acute Toxicity, Mutagenicity, Skin and Eye Irritation
A02972	Acute Toxicity, Tumorigenicity, Skin and Eye Irritation
A02973	Acute Toxicity, Skin and Eye Irritation, Reproductive Effects, Multiple Dose
A02974	Acute Toxicity
A02975	Non-toxicity
A02976	Acute Toxicity, Mutagenicity, Tumorigenicity, Skin and Eye Irritation
A02977	Mutagenicity, Tumorigenicity

A02978	Acute Toxicity, Multiple Dose Effects
A02979	Acute Toxicity, Mutagenicity, Tumorigenicity, Reproductive Effects
A02980	Acute Toxicity, Reproductive Effects
A02981	Acute Toxicity, Mutagenicity, Tumorigenicity, Skin and Eye Irritation, Multiple Dose Effects
A02982	Acute Toxicity, Mutagenicity, Tumorigenicity, Multiple Dose Effects
A02983	Acute Toxicity, Reproductive Effects, Multiple Dose Effects
A02984	Acute Toxicity, Mutagenicity, Tumorigenicity, Multiple Dose Effects
A02985	Acute Toxicity
A02986	Acute Toxicity, Reproductive Effects, Multiple Dose Effects
A02987	Acute Toxicity
A02988	Acute Toxicity, Mutagenicity, Tumorigenicity, Multiple Dose Effects
A02989	Acute Toxicity
A02990	Acute Toxicity, Mutagenicity, Multiple Dose Effects
A02991	Acute Toxicity, Multiple Dose Effects
A02992	Acute Toxicity, Mutagenicity, Tumorigenicity, Reproductive Effects,
A02993	Acute Toxicity
A02994	Acute Toxicity, Reproductive Effects
A02995	Mutagenicity
A02996	Acute Toxicity
A02997	Acute Toxicity
A02998	Acute Toxicity
A02999	Acute Toxicity, Reproductive Effects
A03000	Acute Toxicity, Skin and Eye Irritation
A03001	Acute Toxicity, Mutagenicity, Multiple Dose Effects
A03002	Acute Toxicity
A03003	Acute Toxicity, Mutagenicity, Skin and Eye Irritation, Multiple Dose Effects
A03004	Acute Toxicity, Mutagenicity, Multiple Dose Effects
A03005	Acute Toxicity
A03006	Acute Toxicity, Mutagenicity, Skin and Eye Irritation, Multiple Dose Effects
A03007	Acute Toxicity, Mutagenicity, Tumorigenicity, Reproductive Effects,
A03008	Acute Toxicity, Multiple Dose Effects
A03009	Acute Toxicity, Skin and Eye Irritation
A03010	Acute Toxicity, Mutagenicity
A03011	Acute Toxicity
A03012	Acute Toxicity, Tumorigenicity, Reproductive Effects, Multiple Dose Effects
A03013	Acute Toxicity, Multiple Dose Effects
A03014	Acute Toxicity, Mutagenicity, Reproductive Effects, Multiple Dose Effects
A03015	Acute Toxicity, Mutagenicity
A03016	Acute Toxicity, Mutagenicity, Tumorigenicity, Reproductive Effects,
A03017	Acute Toxicity, Multiple Dose Effects
A03018	Acute Toxicity, Mutagenicity, Skin and Eye Irritation, Multiple Dose Effects
A03019	Acute Toxicity
A03020	Acute Toxicity, Mutagenicity
A03021	Mutagenicity
A03022	Acute Toxicity, Multiple Dose Effects

A03023	Acute Toxicity, Mutagenicity
A03024	Acute Toxicity, Mutagenicity, Skin and Eye Irritation, Reproductive Effects, Multiple Dose Effects
A03025	Mutagenicity, Skin and Eye Irritation
A03026	Acute Toxicity, Multiple Dose Effects
A03027	Acute Toxicity, Mutagenicity
A03028	Acute Toxicity
A03029	Acute Toxicity, Skin and Eye Irritation, Multiple Dose Effects
A03030	Acute Toxicity
A03031	Acute Toxicity, Multiple Dose Effects
A03032	Acute Toxicity, Mutagenicity, Tumorigenicity, Multiple Dose Effects
A03033	Acute Toxicity, Mutagenicity, Tumorigenicity, Skin and Eye Irritation, Multiple Dose Effects
A03034	Acute Toxicity, Mutagenicity, Tumorigenicity, Skin and Eye Irritation,
A03035	Acute Toxicity, Mutagenicity, Skin and Eye Irritation, Reproductive Effects, Multiple Dose Effects
A03036	Acute Toxicity, Mutagenicity, Skin and Eye Irritation, Reproductive Effects, Multiple Dose Effects
A03037	Acute Toxicity, Skin and Eye Irritation
A03038	Acute Toxicity, Mutagenicity
A03039	Reproductive Effects
A03040	Acute Toxicity
A03041	Reproductive Effects
A03042	Acute Toxicity, Mutagenicity, Skin and Eye Irritation, Multiple Dose Effects
A03043	Acute Toxicity, Mutagenicity, Skin and Eye Irritation, Reproductive Effects
A03044	Mutagenicity
A03045	Acute Toxicity, Mutagenicity, Skin and Eye Irritation
A03046	Acute Toxicity, Skin and Eye Irritation
A03047	Acute Toxicity, Skin and Eye Irritation
A03048	Acute Toxicity, Multiple Dose Effects
A03049	Acute Toxicity, Mutagenicity, Tumorigenicity, Skin and Eye Irritation, Multiple Dose Effects
A03050	Acute Toxicity, Mutagenicity, Tumorigenicity, Reproductive Effects,
A03051	Acute Toxicity, Mutagenicity, Tumorigenicity, Multiple Dose Effects
A03052	Acute Toxicity, Mutagenicity, Tumorigenicity, Multiple Dose Effects
A03053	Acute Toxicity
A03054	Acute Toxicity, Mutagenicity, Skin and Eye Irritation
A03055	Acute Toxicity
A03056	Acute Toxicity, Mutagenicity, Skin and Eye Irritation, Reproductive Effects, Multiple Dose Effects
A03057	Acute Toxicity, Skin and Eye Irritation
A03058	Acute Toxicity, Mutagenicity, Skin and Eye Irritation, Multiple Dose Effects
A03059	Acute Toxicity, Multiple Dose Effects
A03060	Acute Toxicity, Mutagenicity, Skin and Eye Irritation, Multiple Dose Effects
A03061	Acute Toxicity
A03062	Acute Toxicity, Mutagenicity

A03063	Acute Toxicity
A03064	Acute Toxicity, Mutagenicity, Tumorigenicity, Skin and Eye Irritation
A03065	Acute Toxicity, Mutagenicity
A03066	Acute Toxicity, Mutagenicity
A03067	Acute Toxicity, Mutagenicity, Skin and Eye Irritation, Reproductive Effects, Multiple Dose Effects
A03068	Acute Toxicity, Mutagenicity, Tumorigenicity, Skin and Eye Irritation, Multiple Dose Effects
A03069	Acute Toxicity, Mutagenicity, Skin and Eye Irritation
A03070	Acute Toxicity, Mutagenicity, Skin and Eye Irritation
A03071	Acute Toxicity
A03072	Mutagenicity
A03073	Acute Toxicity, Skin and Eye Irritation
A03074	Acute Toxicity
A03075	Acute Toxicity, Skin and Eye Irritation
A03076	Acute Toxicity, Mutagenicity, Tumorigenicity
A03077	Acute Toxicity, Mutagenicity, Tumorigenicity
A03078	Acute Toxicity, Skin and Eye Irritation, Multiple Dose Effects
A03079	Acute Toxicity, Reproductive Effects, Multiple Dose Effects
A03080	Acute Toxicity, Mutagenicity, Tumorigenicity, Skin and Eye Irritation, Multiple Dose Effects
A03081	Acute Toxicity, Mutagenicity, Skin and Eye Irritation
A03082	Acute Toxicity
A03083	Acute Toxicity
A03084	Acute Toxicity, Mutagenicity
A03085	Acute Toxicity
A03086	Acute Toxicity, Mutagenicity, Tumorigenicity, Skin and Eye Irritation, Multiple Dose Effects
A03087	Acute Toxicity, Mutagenicity, Tumorigenicity, Reproductive Effects,
A03088	Mutagenicity
A03089	Acute Toxicity, Mutagenicity, Tumorigenicity, Skin and Eye Irritation, Multiple Dose Effects
A03090	Acute Toxicity, Tumorigenicity, Multiple Dose Effects
A03091	Acute Toxicity, Mutagenicity, Skin and Eye Irritation, Reproductive Effects, Multiple Dose Effects
A03092	Acute Toxicity, Skin and Eye Irritation, Multiple Dose Effects
A03093	Acute Toxicity, Mutagenicity, Skin and Eye Irritation, Multiple Dose Effects
A03094	Acute Toxicity, Mutagenicity, Tumorigenicity, Skin and Eye Irritation,
A03095	Acute Toxicity, Mutagenicity, Tumorigenicity, Reproductive Effects
A03096	Acute Toxicity, Mutagenicity
A03097	Acute Toxicity, Mutagenicity, Tumorigenicity, Skin and Eye Irritation, Multiple Dose Effects
A03098	Acute Toxicity, Mutagenicity, Tumorigenicity, Skin and Eye Irritation,
A03099	Acute Toxicity, Mutagenicity, Tumorigenicity, Skin and Eye Irritation,
A03100	Acute Toxicity, Skin and Eye Irritation, Multiple Dose Effects
A03101	Acute Toxicity, Tumorigenicity, Reproductive Effects

A03102	Acute Toxicity
A03103	Acute Toxicity, Skin and Eye Irritation
A03104	Acute Toxicity, Mutagenicity, Tumorigenicity, Skin and Eye Irritation,
A03105	Acute Toxicity, Skin and Eye Irritation
A03106	Acute Toxicity
A03107	Acute Toxicity, Mutagenicity, Tumorigenicity, Skin and Eye Irritation,
A03108	Acute Toxicity, Mutagenicity, Skin and Eye Irritation, Multiple Dose Effects
A03109	Acute Toxicity, Mutagenicity, Skin and Eye Irritation, Multiple Dose Effects
A03110	Acute Toxicity, Mutagenicity
A03111	Acute Toxicity, Mutagenicity
A03112	Acute Toxicity, Mutagenicity, Skin and Eye Irritation, Multiple Dose Effects
A03113	Acute Toxicity, Mutagenicity, Tumorigenicity, Skin and Eye Irritation,
A03114	Mutagenicity, Skin and Eye Irritation
A03115	Acute Toxicity, Mutagenicity, Skin and Eye Irritation
A03116	Acute Toxicity, Mutagenicity
A03117	Mutagenicity
A03118	Acute Toxicity, Skin and Eye Irritation
A03119	Acute Toxicity, Mutagenicity, Tumorigenicity, Reproductive Effects
A03120	Acute Toxicity, Mutagenicity, Multiple Dose Effects
A03121	Acute Toxicity, Mutagenicity, Skin and Eye Irritation, Reproductive Effects, Multiple Dose Effects
A03122	Skin and Eye Irritation
A03123	Acute Toxicity, Mutagenicity
A03124	Acute Toxicity, Mutagenicity, Skin and Eye Irritation
A03125	Acute Toxicity, Mutagenicity
A03126	Acute Toxicity, Mutagenicity, Tumorigenicity, Skin and Eye Irritation, Multiple Dose Effects
A03127	Acute Toxicity, Mutagenicity, Reproductive Effects, Multiple Dose Effects
A03128	Acute Toxicity, Skin and Eye Irritation
A03129	Acute Toxicity, Skin and Eye Irritation
A03130	Mutagenicity, Multiple Dose Effects
A03131	Acute Toxicity, Mutagenicity, Reproductive Effects, Multiple Dose Effects
A03132	Acute Toxicity
A03133	Skin and Eye Irritation
A03134	Mutagenicity, Tumorigenicity, Skin and Eye Irritation, Reproductive Effects, Multiple Dose Effects
A03135	Acute Toxicity, Mutagenicity, Skin and Eye Irritation, Multiple Dose Effects
A03136	Acute Toxicity, Multiple Dose Effects
A03137	Mutagenicity
A03138	Acute Toxicity, Mutagenicity, Tumorigenicity, Skin and Eye Irritation, Multiple Dose Effects
A03139	Acute Toxicity, Multiple Dose Effects
A03140	Acute Toxicity
A03141	Acute Toxicity, Mutagenicity, Tumorigenicity, Reproductive Effects,
A03142	Acute Toxicity, Skin and Eye Irritation
A03143	Acute Toxicity, Tumorigenicity, Reproductive Effects, Multiple Dose Effects

A03144	Acute Toxicity, Mutagenicity, Skin and Eye Irritation, Reproductive Effects, Multiple Dose Effects
A03145	Mutagenicity, Skin and Eye Irritation
A03146	Acute Toxicity, Reproductive Effects, Multiple Dose Effects
A03147	Acute Toxicity, Mutagenicity, Tumorigenicity, Skin and Eye Irritation,
A03148	Acute Toxicity, Reproductive Effects, Multiple Dose Effects
A03149	Acute Toxicity, Skin and Eye Irritation
A03150	Acute Toxicity
A03151	Acute Toxicity, Skin and Eye Irritation
A03152	Mutagenicity
A03153	Acute Toxicity, Mutagenicity
A03154	Acute Toxicity, Mutagenicity, Skin and Eye Irritation, Multiple Dose Effects
A03155	Acute Toxicity, Skin and Eye Irritation, Multiple Dose Effects
A03156	Acute Toxicity, Skin and Eye Irritation, Reproductive Effects, Multiple Dose
A03157	Acute Toxicity, Mutagenicity, Tumorigenicity
A03158	Acute Toxicity, Mutagenicity
A03159	Acute Toxicity, Mutagenicity, Multiple Dose Effects
A03160	Acute Toxicity, Mutagenicity, Tumorigenicity
A03161	Acute Toxicity
A03162	Acute Toxicity, Tumorigenicity
A03163	Acute Toxicity, Multiple Dose Effects
A03164	Acute Toxicity, Mutagenicity
A03165	Acute Toxicity, Mutagenicity
A03166	Acute Toxicity
A03167	Acute Toxicity, Mutagenicity, Tumorigenicity, Skin and Eye Irritation,
A03168	Acute Toxicity, Mutagenicity, Tumorigenicity, Skin and Eye Irritation,
A03169	Acute Toxicity
A03170	Acute Toxicity
A03171	Acute Toxicity, Mutagenicity, Multiple Dose Effects
A03172	Acute Toxicity, Skin and Eye Irritation
A03173	Skin and Eye Irritation
A03174	Acute Toxicity, Mutagenicity, Tumorigenicity, Multiple Dose Effects
A03175	Acute Toxicity
A03176	Mutagenicity
A03177	Acute Toxicity, Mutagenicity, Skin and Eye Irritation, Reproductive Effects
A03178	Acute Toxicity, Mutagenicity, Tumorigenicity
A03179	Acute Toxicity
A03180	Acute Toxicity
A03181	Acute Toxicity, Mutagenicity, Skin and Eye Irritation
A03182	Acute Toxicity
A03183	Acute Toxicity
A03184	Acute Toxicity, Reproductive Effects, Multiple Dose Effects
A03185	Acute Toxicity, Mutagenicity, Skin and Eye Irritation
A03186	Acute Toxicity, Mutagenicity, Multiple Dose Effects
A03187	Acute Toxicity
A03188	Acute Toxicity, Mutagenicity, Tumorigenicity, Skin and Eye Irritation,

A03189	Acute Toxicity, Skin and Eye Irritation, Multiple Dose Effects
A03190	Acute Toxicity
A03191	Acute Toxicity, Multiple Dose Effects
A03192	Acute Toxicity, Skin and Eye Irritation
A03193	Skin and Eye Irritation
A03194	Acute Toxicity
A03195	Acute Toxicity, Mutagenicity, Skin and Eye Irritation, Reproductive Effects, Multiple Dose Effects
A03196	Acute Toxicity, Skin and Eye Irritation
A03197	Acute Toxicity, Mutagenicity
A03198	Acute Toxicity
A03199	Acute Toxicity, Mutagenicity, Skin and Eye Irritation, Reproductive Effects, Multiple Dose Effects
A03200	Acute Toxicity
A03201	Acute Toxicity, Mutagenicity
A03202	Mutagenicity, Tumorigenicity
A03203	Acute Toxicity, Mutagenicity
A03204	Acute Toxicity, Mutagenicity, Skin and Eye Irritation
A03205	Non-toxicity
A03206	Acute Toxicity, Skin and Eye Irritation
A03207	Acute Toxicity, Mutagenicity
A03208	Mutagenicity
A03209	Acute Toxicity, Mutagenicity, Tumorigenicity
A03210	Mutagenicity
A03211	Acute Toxicity, Mutagenicity, Tumorigenicity
A03212	Acute Toxicity, Skin and Eye Irritation
A03213	Acute Toxicity, Mutagenicity, Tumorigenicity, Multiple Dose Effects
A03214	Acute Toxicity
A03215	Acute Toxicity
A03216	Mutagenicity, Skin and Eye Irritation
A03217	Acute Toxicity, Mutagenicity, Tumorigenicity, Reproductive Effects
A03218	Acute Toxicity, Skin and Eye Irritation, Multiple Dose Effects
A03219	Acute Toxicity
A03220	Acute Toxicity
A03221	Acute Toxicity, Mutagenicity, Tumorigenicity
A03222	Skin and Eye Irritation
A03223	Acute Toxicity, Skin and Eye Irritation
A03224	Acute Toxicity, Mutagenicity, Tumorigenicity, Skin and Eye Irritation, Multiple Dose Effects
A03225	Acute Toxicity
A03226	Acute Toxicity, Mutagenicity
A03227	Acute Toxicity
A03228	Acute Toxicity, Mutagenicity, Skin and Eye Irritation, Multiple Dose Effects
A03229	Acute Toxicity, Mutagenicity, Skin and Eye Irritation, Reproductive Effects
A03230	Mutagenicity
A03231	Acute Toxicity

A03232	Acute Toxicity, Mutagenicity
A03233	Acute Toxicity
A03234	Acute Toxicity, Mutagenicity, Tumorigenicity, Reproductive Effects,
A03235	Acute Toxicity, Mutagenicity, Tumorigenicity, Reproductive Effects,
A03236	Acute Toxicity
A03237	Acute Toxicity, Mutagenicity, Reproductive Effects, Multiple Dose Effects
A03238	Acute Toxicity, Mutagenicity, Tumorigenicity, Skin and Eye Irritation,
A03239	Acute Toxicity, Mutagenicity, Tumorigenicity, Skin and Eye Irritation, Multiple Dose Effects
A03240	Acute Toxicity, Skin and Eye Irritation
A03241	Acute Toxicity, Mutagenicity, Tumorigenicity, Reproductive Effects
A03242	Acute Toxicity
A03243	Acute Toxicity, Mutagenicity, Tumorigenicity, Skin and Eye Irritation,
A03244	Mutagenicity
A03245	Acute Toxicity, Mutagenicity
A03246	Acute Toxicity
A03247	Acute Toxicity, Mutagenicity, Reproductive Effects
A03248	Acute Toxicity
A03249	Skin and Eye Irritation
A03250	Acute Toxicity
A03251	Acute Toxicity
A03252	Tumorigenicity
A03253	Acute Toxicity, Mutagenicity, Tumorigenicity, Skin and Eye Irritation, Multiple Dose Effects
A03254	Acute Toxicity, Mutagenicity, Reproductive Effects
A03255	Acute Toxicity
A03256	Acute Toxicity, Mutagenicity, Tumorigenicity, Skin and Eye Irritation,
A03257	Mutagenicity
A03258	Acute Toxicity, Mutagenicity, Tumorigenicity
A03259	Acute Toxicity, Mutagenicity, Skin and Eye Irritation, Multiple Dose Effects
A03260	Acute Toxicity, Skin and Eye Irritation, Multiple Dose Effects
A03261	Acute Toxicity, Mutagenicity
A03262	Acute Toxicity
A03263	Acute Toxicity, Mutagenicity, Skin and Eye Irritation
A03264	Acute Toxicity, Mutagenicity, Tumorigenicity, Skin and Eye Irritation,
A03265	Mutagenicity
A03266	Acute Toxicity, Skin and Eye Irritation, Reproductive Effects, Multiple Dose
A03267	Acute Toxicity, Skin and Eye Irritation
A03268	Acute Toxicity, Mutagenicity, Multiple Dose Effects
A03269	Acute Toxicity
A03270	Acute Toxicity
A03271	Acute Toxicity, Mutagenicity, Skin and Eye Irritation, Multiple Dose Effects
A03272	Acute Toxicity, Mutagenicity
A03273	Acute Toxicity
A03274	Mutagenicity
A03275	Acute Toxicity, Multiple Dose Effects

A03276	Acute Toxicity, Mutagenicity, Skin and Eye Irritation, Multiple Dose Effects
A03277	Acute Toxicity
A03278	Acute Toxicity, Skin and Eye Irritation
A03279	Acute Toxicity, Skin and Eye Irritation
A03280	Acute Toxicity, Skin and Eye Irritation
A03281	Acute Toxicity, Mutagenicity, Skin and Eye Irritation, Multiple Dose Effects
A03282	Acute Toxicity, Reproductive Effects, Multiple Dose Effects
A03283	Acute Toxicity
A03284	Acute Toxicity, Mutagenicity, Skin and Eye Irritation
A03285	Acute Toxicity, Skin and Eye Irritation
A03286	Acute Toxicity
A03287	Acute Toxicity, Skin and Eye Irritation, Reproductive Effects
A03288	Acute Toxicity, Mutagenicity, Reproductive Effects
A03289	Acute Toxicity
A03290	Acute Toxicity
A03291	Acute Toxicity, Skin and Eye Irritation
A03292	Acute Toxicity, Skin and Eye Irritation, Multiple Dose Effects
A03293	Acute Toxicity, Reproductive Effects
A03294	Mutagenicity, Reproductive Effects
A03295	Acute Toxicity, Multiple Dose Effects
A03296	Acute Toxicity
A03297	Acute Toxicity, Mutagenicity, Tumorigenicity, Reproductive Effects
A03298	Acute Toxicity, Reproductive Effects
A03299	Acute Toxicity
A03300	Acute Toxicity, Mutagenicity, Tumorigenicity, Skin and Eye Irritation,
A03301	Acute Toxicity
A03302	Acute Toxicity, Reproductive Effects
A03303	Acute Toxicity
A03304	Acute Toxicity, Mutagenicity, Tumorigenicity, Multiple Dose Effects
A03305	Acute Toxicity, Mutagenicity, Skin and Eye Irritation
A03306	Acute Toxicity
A03307	Acute Toxicity, Mutagenicity
A03308	Acute Toxicity, Multiple Dose Effects
A03309	Skin and Eye Irritation
A03310	Acute Toxicity, Skin and Eye Irritation, Reproductive Effects, Multiple Dose
A03311	Acute Toxicity, Skin and Eye Irritation, Multiple Dose Effects
A03312	Mutagenicity, Skin and Eye Irritation
A03313	Acute Toxicity, Mutagenicity, Tumorigenicity, Multiple Dose Effects
A03314	Acute Toxicity, Mutagenicity, Tumorigenicity, Skin and Eye Irritation,
A03315	Acute Toxicity, Mutagenicity, Skin and Eye Irritation
A03316	Acute Toxicity, Skin and Eye Irritation
A03317	Acute Toxicity
A03318	Acute Toxicity, Skin and Eye Irritation
A03319	Acute Toxicity
A03320	Acute Toxicity
A03321	Acute Toxicity, Mutagenicity, Tumorigenicity, Skin and Eye Irritation,

A03322	Acute Toxicity, Multiple Dose Effects
A03323	Acute Toxicity, Mutagenicity
A03324	Acute Toxicity, Mutagenicity, Reproductive Effects, Multiple Dose Effects
A03325	Acute Toxicity
A03326	Reproductive Effects
A03327	Acute Toxicity, Mutagenicity, Reproductive Effects, Multiple Dose Effects
A03328	Acute Toxicity
A03329	Acute Toxicity, Mutagenicity, Reproductive Effects, Multiple Dose Effects
A03330	Skin and Eye Irritation, Reproductive Effects
A03331	Acute Toxicity
A03332	Reproductive Effects
A03333	Acute Toxicity, Mutagenicity, Tumorigenicity, Skin and Eye Irritation
A03334	Acute Toxicity, Mutagenicity, Skin and Eye Irritation, Multiple Dose Effects
A03335	Acute Toxicity, Reproductive Effects, Multiple Dose Effects
A03336	Acute Toxicity, Mutagenicity, Tumorigenicity, Multiple Dose Effects
A03337	Acute Toxicity, Mutagenicity, Reproductive Effects
A03338	Acute Toxicity, Skin and Eye Irritation, Reproductive Effects
A03339	Acute Toxicity
A03340	Acute Toxicity
A03341	Acute Toxicity
A03342	Mutagenicity
A03343	Tumorigenicity
A03344	Mutagenicity, Tumorigenicity
A03345	Mutagenicity, Tumorigenicity
A03346	Mutagenicity, Tumorigenicity
A03347	Mutagenicity, Tumorigenicity
A03348	Mutagenicity, Tumorigenicity
A03349	Acute Toxicity, Mutagenicity, Tumorigenicity, Reproductive Effects,
A03350	Mutagenicity, Tumorigenicity
A03351	Mutagenicity, Tumorigenicity, Reproductive Effects
A03352	Mutagenicity
A03353	Mutagenicity, Tumorigenicity
A03354	Mutagenicity
A03355	Mutagenicity
A03356	Acute Toxicity, Mutagenicity, Tumorigenicity
A03357	Mutagenicity, Tumorigenicity
A03358	Mutagenicity, Tumorigenicity
A03359	Mutagenicity
A03360	Mutagenicity, Tumorigenicity
A03361	Mutagenicity
A03362	Mutagenicity, Tumorigenicity
A03363	Mutagenicity
A03364	Mutagenicity, Tumorigenicity
A03365	Mutagenicity, Tumorigenicity
A03366	Mutagenicity, Tumorigenicity
A03367	Mutagenicity

A03368	Mutagenicity, Tumorigenicity
A03369	Acute Toxicity, Mutagenicity, Tumorigenicity, Multiple Dose Effects
A03370	Acute Toxicity, Mutagenicity, Tumorigenicity, Multiple Dose Effects
A03371	Acute Toxicity, Mutagenicity, Tumorigenicity, Multiple Dose Effects
A03372	Tumorigenicity
A03373	Acute Toxicity, Mutagenicity, Multiple Dose Effects
A03374	Mutagenicity, Tumorigenicity
A03375	Mutagenicity, Tumorigenicity
A03376	Mutagenicity, Tumorigenicity
A03377	Tumorigenicity
A03378	Mutagenicity
A03379	Acute Toxicity, Mutagenicity, Tumorigenicity, Multiple Dose Effects
A03380	Mutagenicity
A03381	Mutagenicity
A03382	Mutagenicity
A03383	Mutagenicity, Tumorigenicity
A03384	Mutagenicity, Tumorigenicity, Reproductive Effects
A03385	Mutagenicity, Tumorigenicity
A03386	Mutagenicity, Tumorigenicity
A03387	Tumorigenicity
A03388	Mutagenicity, Tumorigenicity
A03389	Mutagenicity, Tumorigenicity
A03390	Mutagenicity, Tumorigenicity
A03391	Mutagenicity, Tumorigenicity
A03392	Mutagenicity
A03393	Acute Toxicity
A03394	Mutagenicity
A03395	Tumorigenicity, Reproductive Effects
A03396	Mutagenicity, Tumorigenicity
A03397	Mutagenicity
A03398	Mutagenicity
A03399	Acute Toxicity
A03400	Acute Toxicity, Tumorigenicity, Skin and Eye Irritation, Reproductive Effects, Multiple Dose Effects
A03401	Acute Toxicity, Mutagenicity, Tumorigenicity, Multiple Dose Effects
A03402	Acute Toxicity, Mutagenicity, Tumorigenicity
A03403	Acute Toxicity, Mutagenicity
A03404	Acute Toxicity
A03405	Acute Toxicity
A03406	Acute Toxicity
A03407	Acute Toxicity
A03408	Acute Toxicity, Mutagenicity, Tumorigenicity, Multiple Dose Effects
A03409	Acute Toxicity
A03410	Mutagenicity
A03411	Acute Toxicity
A03412	Acute Toxicity

A03413	Acute Toxicity, Multiple Dose Effects
A03414	Acute Toxicity, Skin and Eye Irritation
A03415	Acute Toxicity, Mutagenicity, Skin and Eye Irritation, Multiple Dose Effects
A03416	Mutagenicity
A03417	Acute Toxicity, Mutagenicity, Tumorigenicity
A03418	Mutagenicity
A03419	Acute Toxicity, Multiple Dose Effects
A03420	Acute Toxicity, Skin and Eye Irritation, Reproductive Effects, Multiple Dose
A03421	Acute Toxicity
A03422	Acute Toxicity, Mutagenicity
A03423	Acute Toxicity, Mutagenicity
A03424	Mutagenicity
A03425	Acute Toxicity, Skin and Eye Irritation
A03426	Acute Toxicity
A03427	Acute Toxicity, Mutagenicity, Skin and Eye Irritation, Reproductive Effects, Multiple Dose Effects
A03428	Mutagenicity, Tumorigenicity, Reproductive Effects, Multiple Dose Effects
A03429	Acute Toxicity
A03430	Acute Toxicity, Mutagenicity
A03431	Acute Toxicity, Mutagenicity, Skin and Eye Irritation
A03432	Acute Toxicity, Skin and Eye Irritation
A03433	Acute Toxicity
A03434	Acute Toxicity, Mutagenicity
A03435	Acute Toxicity
A03436	Acute Toxicity, Mutagenicity
A03437	Acute Toxicity, Reproductive Effects
A03438	Acute Toxicity, Skin and Eye Irritation, Reproductive Effects, Multiple Dose
A03439	Acute Toxicity
A03440	Multiple Dose Effects
A03441	Acute Toxicity, Mutagenicity, Reproductive Effects, Multiple Dose Effects
A03442	Acute Toxicity, Multiple Dose Effects
A03443	Acute Toxicity, Reproductive Effects, Multiple Dose Effects
A03444	Acute Toxicity
A03445	Multiple Dose Effects
A03446	Acute Toxicity
A03447	Acute Toxicity, Skin and Eye Irritation, Multiple Dose Effects
A03448	Acute Toxicity
A03449	Acute Toxicity
A03450	Acute Toxicity
A03451	Acute Toxicity, Mutagenicity, Tumorigenicity, Skin and Eye Irritation,
A03452	Acute Toxicity, Mutagenicity, Tumorigenicity, Reproductive Effects,
A03453	Acute Toxicity, Mutagenicity
A03454	Acute Toxicity
A03455	Acute Toxicity
A03456	Acute Toxicity
A03457	Acute Toxicity, Mutagenicity, Tumorigenicity

A03458	Acute Toxicity
A03459	Acute Toxicity
A03460	Reproductive Effects
A03461	Acute Toxicity
A03462	Acute Toxicity
A03463	Mutagenicity, Tumorigenicity
A03464	Acute Toxicity
A03465	Acute Toxicity
A03466	Acute Toxicity, Mutagenicity, Reproductive Effects, Multiple Dose Effects
A03467	Acute Toxicity
A03468	Acute Toxicity
A03469	Acute Toxicity, Mutagenicity
A03470	Tumorigenicity
A03471	Acute Toxicity, Mutagenicity, Tumorigenicity, Reproductive Effects,
A03472	Acute Toxicity
A03473	Acute Toxicity, Mutagenicity, Tumorigenicity, Skin and Eye Irritation,
A03474	Acute Toxicity, Multiple Dose Effects
A03475	Acute Toxicity
A03476	Acute Toxicity, Multiple Dose Effects
A03477	Acute Toxicity, Mutagenicity, Multiple Dose Effects
A03478	Acute Toxicity, Multiple Dose Effects
A03479	Acute Toxicity
A03480	Acute Toxicity, Mutagenicity, Skin and Eye Irritation, Reproductive Effects, Multiple Dose Effects
A03481	Acute Toxicity, Reproductive Effects, Multiple Dose Effects
A03482	Tumorigenicity
A03483	Mutagenicity
A03484	Acute Toxicity, Mutagenicity, Tumorigenicity, Reproductive Effects,
A03485	Acute Toxicity
A03486	Acute Toxicity
A03487	Acute Toxicity, Mutagenicity, Tumorigenicity, Reproductive Effects,
A03488	Acute Toxicity, Mutagenicity, Tumorigenicity, Reproductive Effects,
A03489	Acute Toxicity, Mutagenicity, Tumorigenicity, Skin and Eye Irritation,
A03490	Acute Toxicity
A03491	Mutagenicity, Tumorigenicity
A03492	Acute Toxicity, Mutagenicity
A03493	Acute Toxicity, Mutagenicity, Reproductive Effects
A03494	Acute Toxicity, Mutagenicity, Skin and Eye Irritation, Multiple Dose Effects
A03495	Acute Toxicity
A03496	Acute Toxicity, Mutagenicity, Tumorigenicity, Skin and Eye Irritation,
A03497	Acute Toxicity
A03498	Acute Toxicity, Mutagenicity, Multiple Dose Effects
A03499	Acute Toxicity
A03500	Acute Toxicity
A03501	Acute Toxicity, Mutagenicity
A03502	Acute Toxicity

A03503	Acute Toxicity
A03504	Mutagenicity
A03505	Acute Toxicity, Mutagenicity, Tumorigenicity
A03506	Acute Toxicity
A03507	Acute Toxicity, Mutagenicity, Skin and Eye Irritation
A03508	Acute Toxicity, Multiple Dose Effects
A03509	Acute Toxicity
A03510	Acute Toxicity
A03511	Acute Toxicity, Mutagenicity
A03512	Acute Toxicity, Mutagenicity, Reproductive Effects, Multiple Dose Effects
A03513	Acute Toxicity, Mutagenicity, Reproductive Effects, Multiple Dose Effects
A03514	Acute Toxicity
A03515	Mutagenicity
A03516	Acute Toxicity, Multiple Dose Effects
A03517	Acute Toxicity, Mutagenicity
A03518	Acute Toxicity, Mutagenicity, Reproductive Effects
A03519	Acute Toxicity
A03520	Acute Toxicity, Mutagenicity, Tumorigenicity
A03521	Acute Toxicity
A03522	Acute Toxicity
A03523	Acute Toxicity, Mutagenicity, Tumorigenicity, Reproductive Effects,
A03524	Acute Toxicity, Mutagenicity, Reproductive Effects, Multiple Dose Effects
A03525	Acute Toxicity, Mutagenicity, Skin and Eye Irritation, Reproductive Effects, Multiple Dose Effects
A03526	Acute Toxicity, Mutagenicity
A03527	Acute Toxicity
A03528	Acute Toxicity, Mutagenicity, Tumorigenicity, Reproductive Effects
A03529	Acute Toxicity
A03530	Tumorigenicity
A03531	Acute Toxicity
A03532	Multiple Dose Effects
A03533	Acute Toxicity, Mutagenicity, Reproductive Effects, Multiple Dose Effects
A03534	Acute Toxicity
A03535	Acute Toxicity
A03536	Acute Toxicity, Mutagenicity
A03537	Acute Toxicity, Mutagenicity
A03538	Acute Toxicity
A03539	Mutagenicity
A03540	Acute Toxicity
A03541	Acute Toxicity, Skin and Eye Irritation
A03542	Acute Toxicity
A03543	Acute Toxicity
A03544	Acute Toxicity
A03545	Acute Toxicity, Mutagenicity, Reproductive Effects, Multiple Dose Effects
A03546	Acute Toxicity
A03547	Acute Toxicity, Skin and Eye Irritation, Multiple Dose Effects

A03548	Acute Toxicity, Multiple Dose Effects
A03549	Acute Toxicity, Mutagenicity, Multiple Dose Effects
A03550	Acute Toxicity
A03551	Acute Toxicity
A03552	Acute Toxicity
A03553	Acute Toxicity, Multiple Dose Effects
A03554	Acute Toxicity, Multiple Dose Effects
A03555	Acute Toxicity
A03556	Acute Toxicity, Mutagenicity, Skin and Eye Irritation, Reproductive Effects, Multiple Dose Effects
A03557	Acute Toxicity
A03558	Acute Toxicity, Multiple Dose Effects
A03559	Acute Toxicity, Multiple Dose Effects
A03560	Acute Toxicity, Multiple Dose Effects
A03561	Acute Toxicity
A03562	Mutagenicity
A03563	Acute Toxicity
A03564	Acute Toxicity
A03565	Acute Toxicity, Reproductive Effects, Multiple Dose Effects
A03566	Non-toxicity
A03567	Acute Toxicity, Multiple Dose Effects
A03568	Acute Toxicity
A03569	Acute Toxicity, Mutagenicity
A03570	Acute Toxicity, Mutagenicity, Tumorigenicity, Reproductive Effects
A03571	Non-toxicity
A03572	Acute Toxicity
A03573	Mutagenicity, Tumorigenicity
A03574	Acute Toxicity, Multiple Dose Effects
A03575	Mutagenicity
A03576	Mutagenicity
A03577	Acute Toxicity
A03578	Acute Toxicity, Mutagenicity
A03579	Acute Toxicity, Mutagenicity
A03580	Skin and Eye Irritation
A03581	Mutagenicity, Reproductive Effects, Multiple Dose Effects
A03582	Acute Toxicity
A03583	Acute Toxicity, Mutagenicity, Skin and Eye Irritation
A03584	Acute Toxicity, Tumorigenicity, Reproductive Effects
A03585	Acute Toxicity
A03586	Acute Toxicity, Multiple Dose Effects
A03587	Acute Toxicity
A03588	Acute Toxicity
A03589	Acute Toxicity
A03590	Mutagenicity
A03591	Non-toxicity
A03592	Acute Toxicity

A03593	Acute Toxicity
A03594	Acute Toxicity, Multiple Dose Effects
A03595	Acute Toxicity
A03596	Acute Toxicity
A03597	Acute Toxicity, Skin and Eye Irritation, Reproductive Effects, Multiple Dose
A03598	Acute Toxicity
A03599	Mutagenicity
A03600	Reproductive Effects
A03601	Acute Toxicity, Reproductive Effects, Multiple Dose Effects
A03602	Acute Toxicity
A03603	Acute Toxicity, Skin and Eye Irritation
A03604	Acute Toxicity, Mutagenicity, Multiple Dose Effects
A03605	Tumorigenicity
A03606	Acute Toxicity
A03607	Acute Toxicity
A03608	Acute Toxicity, Mutagenicity, Reproductive Effects
A03609	Skin and Eye Irritation
A03610	Acute Toxicity, Skin and Eye Irritation
A03611	Acute Toxicity, Skin and Eye Irritation, Reproductive Effects, Multiple Dose
A03612	Acute Toxicity, Mutagenicity, Tumorigenicity, Skin and Eye Irritation
A03613	Acute Toxicity, Mutagenicity, Reproductive Effects
A03614	Acute Toxicity
A03615	Acute Toxicity
A03616	Acute Toxicity, Skin and Eye Irritation
A03617	Acute Toxicity, Skin and Eye Irritation, Multiple Dose Effects
A03618	Acute Toxicity, Mutagenicity, Tumorigenicity, Reproductive Effects,
A03619	Acute Toxicity
A03620	Acute Toxicity
A03621	Acute Toxicity, Multiple Dose Effects
A03622	Acute Toxicity
A03623	Acute Toxicity
A03624	Acute Toxicity
A03625	Acute Toxicity, Mutagenicity, Tumorigenicity, Reproductive Effects,
A03626	Reproductive Effects
A03627	Acute Toxicity
A03628	Acute Toxicity, Reproductive Effects, Multiple Dose Effects
A03629	Acute Toxicity
A03630	Acute Toxicity
A03631	Mutagenicity
A03632	Acute Toxicity, Reproductive Effects, Multiple Dose Effects
A03633	Acute Toxicity
A03634	Acute Toxicity, Skin and Eye Irritation
A03635	Acute Toxicity
A03636	Acute Toxicity, Skin and Eye Irritation
A03637	Acute Toxicity, Mutagenicity
A03638	Acute Toxicity

A03639	Mutagenicity, Tumorigenicity, Multiple Dose Effects
A03640	Acute Toxicity, Multiple Dose Effects
A03641	Acute Toxicity
A03642	Acute Toxicity, Mutagenicity, Multiple Dose Effects
A03643	Acute Toxicity
A03644	Acute Toxicity
A03645	Acute Toxicity
A03646	Acute Toxicity
A03647	Acute Toxicity
A03648	Mutagenicity, Tumorigenicity
A03649	Acute Toxicity, Skin and Eye Irritation, Multiple Dose Effects
A03650	Acute Toxicity
A03651	Mutagenicity, Reproductive Effects
A03652	Acute Toxicity, Mutagenicity, Reproductive Effects, Multiple Dose Effects
A03653	Acute Toxicity, Multiple Dose Effects
A03654	Acute Toxicity, Skin and Eye Irritation, Multiple Dose Effects
A03655	Acute Toxicity
A03656	Acute Toxicity, Mutagenicity
A03657	Acute Toxicity, Mutagenicity
A03658	Acute Toxicity, Mutagenicity
A03659	Acute Toxicity
A03660	Acute Toxicity
A03661	Acute Toxicity, Mutagenicity
A03662	Acute Toxicity, Multiple Dose Effects
A03663	Acute Toxicity, Skin and Eye Irritation
A03664	Acute Toxicity
A03665	Acute Toxicity
A03666	Acute Toxicity, Multiple Dose Effects
A03667	Acute Toxicity, Mutagenicity
A03668	Acute Toxicity
A03669	Acute Toxicity
A03670	Acute Toxicity
A03671	Acute Toxicity
A03672	Acute Toxicity, Mutagenicity
A03673	Acute Toxicity
A03674	Acute Toxicity
A03675	Acute Toxicity, Reproductive Effects
A03676	Acute Toxicity
A03677	Acute Toxicity
A03678	Acute Toxicity
A03679	Acute Toxicity
A03680	Acute Toxicity
A03681	Acute Toxicity, Skin and Eye Irritation
A03682	Acute Toxicity, Tumorigenicity, Skin and Eye Irritation, Multiple Dose
A03683	Acute Toxicity
A03684	Acute Toxicity

A03685	Acute Toxicity, Mutagenicity
A03686	Acute Toxicity, Mutagenicity, Tumorigenicity, Reproductive Effects,
A03687	Mutagenicity
A03688	Mutagenicity, Multiple Dose Effects
A03689	Acute Toxicity
A03690	Acute Toxicity, Mutagenicity
A03691	Acute Toxicity, Multiple Dose Effects
A03692	Mutagenicity
A03693	Mutagenicity
A03694	Acute Toxicity, Mutagenicity
A03695	Mutagenicity
A03696	Acute Toxicity
A03697	Acute Toxicity, Mutagenicity, Reproductive Effects
A03698	Acute Toxicity, Mutagenicity
A03699	Acute Toxicity
A03700	Acute Toxicity
A03701	Mutagenicity, Multiple Dose Effects
A03702	Acute Toxicity
A03703	Acute Toxicity, Mutagenicity, Multiple Dose Effects
A03704	Acute Toxicity, Tumorigenicity, Multiple Dose Effects
A03705	Acute Toxicity, Reproductive Effects
A03706	Acute Toxicity
A03707	Acute Toxicity
A03708	Tumorigenicity
A03709	Acute Toxicity, Mutagenicity, Skin and Eye Irritation, Multiple Dose Effects
A03710	Acute Toxicity, Mutagenicity
A03711	Mutagenicity
A03712	Acute Toxicity, Mutagenicity, Tumorigenicity
A03713	Acute Toxicity
A03714	Acute Toxicity
A03715	Acute Toxicity
A03716	Acute Toxicity
A03717	Acute Toxicity, Mutagenicity
A03718	Reproductive Effects
A03719	Acute Toxicity, Mutagenicity, Tumorigenicity, Reproductive Effects,
A03720	Acute Toxicity, Mutagenicity, Tumorigenicity, Reproductive Effects,
A03721	Mutagenicity
A03722	Mutagenicity
A03723	Acute Toxicity
A03724	Acute Toxicity, Mutagenicity, Tumorigenicity, Multiple Dose Effects
A03725	Acute Toxicity
A03726	Acute Toxicity
A03727	Acute Toxicity
A03728	Acute Toxicity, Mutagenicity, Skin and Eye Irritation
A03729	Mutagenicity
A03730	Mutagenicity

A03731	Mutagenicity
A03732	Acute Toxicity, Multiple Dose Effects
A03733	Mutagenicity
A03734	Acute Toxicity, Mutagenicity, Reproductive Effects
A03735	Acute Toxicity, Mutagenicity
A03736	Acute Toxicity, Mutagenicity
A03737	Acute Toxicity, Mutagenicity
A03738	Acute Toxicity
A03739	Acute Toxicity, Mutagenicity
A03740	Acute Toxicity, Reproductive Effects
A03741	Acute Toxicity
A03742	Acute Toxicity
A03743	Acute Toxicity
A03744	Acute Toxicity, Mutagenicity, Tumorigenicity
A03745	Acute Toxicity
A03746	Mutagenicity
A03747	Acute Toxicity
A03748	Acute Toxicity
A03749	Acute Toxicity
A03750	Acute Toxicity
A03751	Mutagenicity
A03752	Acute Toxicity, Mutagenicity, Reproductive Effects, Multiple Dose Effects
A03753	Acute Toxicity
A03754	Acute Toxicity
A03755	Acute Toxicity, Mutagenicity, Skin and Eye Irritation
A03756	Non-toxicity
A03757	Tumorigenicity, Multiple Dose Effects
A03758	Acute Toxicity
A03759	Acute Toxicity
A03760	Acute Toxicity
A03761	Acute Toxicity, Mutagenicity, Multiple Dose Effects
A03762	Mutagenicity, Tumorigenicity
A03763	Acute Toxicity
A03764	Acute Toxicity, Mutagenicity
A03765	Acute Toxicity, Mutagenicity, Tumorigenicity
A03766	Acute Toxicity, Mutagenicity, Tumorigenicity, Multiple Dose Effects
A03767	Acute Toxicity
A03768	Acute Toxicity, Tumorigenicity
A03769	Acute Toxicity, Mutagenicity, Tumorigenicity, Reproductive Effects
A03770	Acute Toxicity, Skin and Eye Irritation
A03771	Acute Toxicity, Mutagenicity
A03772	Acute Toxicity
A03773	Acute Toxicity
A03774	Mutagenicity
A03775	Acute Toxicity
A03776	Acute Toxicity

A03777	Acute Toxicity, Mutagenicity
A03778	Acute Toxicity
A03779	Acute Toxicity
A03780	Acute Toxicity, Mutagenicity, Multiple Dose Effects
A03781	Mutagenicity
A03782	Mutagenicity, Reproductive Effects
A03783	Acute Toxicity, Skin and Eye Irritation, Multiple Dose Effects
A03784	Mutagenicity
A03785	Mutagenicity
A03786	Acute Toxicity
A03787	Acute Toxicity
A03788	Acute Toxicity, Mutagenicity, Skin and Eye Irritation
A03789	Acute Toxicity
A03790	Acute Toxicity, Skin and Eye Irritation
A03791	Acute Toxicity, Multiple Dose Effects
A03792	Acute Toxicity
A03793	Acute Toxicity
A03794	Mutagenicity
A03795	Acute Toxicity
A03796	Acute Toxicity
A03797	Acute Toxicity, Mutagenicity
A03798	Acute Toxicity, Multiple Dose Effects
A03799	Multiple Dose Effects
A03800	Acute Toxicity
A03801	Acute Toxicity
A03802	Acute Toxicity
A03803	Acute Toxicity, Mutagenicity
A03804	Acute Toxicity, Skin and Eye Irritation
A03805	Acute Toxicity
A03806	Acute Toxicity
A03807	Acute Toxicity, Multiple Dose Effects
A03808	Skin and Eye Irritation
A03809	Acute Toxicity, Multiple Dose Effects
A03810	Acute Toxicity, Mutagenicity
A03811	Acute Toxicity
A03812	Acute Toxicity
A03813	Acute Toxicity, Mutagenicity, Tumorigenicity
A03814	Acute Toxicity
A03815	Multiple Dose Effects
A03816	Acute Toxicity
A03817	Acute Toxicity, Skin and Eye Irritation
A03818	Acute Toxicity, Mutagenicity, Skin and Eye Irritation, Multiple Dose Effects
A03819	Acute Toxicity, Mutagenicity
A03820	Acute Toxicity, Mutagenicity
A03821	Acute Toxicity, Mutagenicity, Skin and Eye Irritation
A03822	Acute Toxicity, Mutagenicity, Tumorigenicity

A03823	Acute Toxicity
A03824	Mutagenicity
A03825	Acute Toxicity, Mutagenicity
A03826	Acute Toxicity, Mutagenicity, Multiple Dose Effects
A03827	Acute Toxicity, Skin and Eye Irritation, Reproductive Effects, Multiple Dose
A03828	Acute Toxicity
A03829	Acute Toxicity, Reproductive Effects
A03830	Non-toxicity
A03831	Non-toxicity
A03832	Acute Toxicity, Mutagenicity, Tumorigenicity, Skin and Eye Irritation,
A03833	Acute Toxicity
A03834	Acute Toxicity
A03835	Tumorigenicity
A03836	Non-toxicity
A03837	Acute Toxicity, Multiple Dose Effects
A03838	Non-toxicity
A03839	Acute Toxicity, Mutagenicity, Tumorigenicity, Reproductive Effects
A03840	Acute Toxicity
A03841	Acute Toxicity
A03842	Acute Toxicity
A03843	Acute Toxicity, Mutagenicity
A03844	Acute Toxicity, Skin and Eye Irritation
A03845	Acute Toxicity
A03846	Acute Toxicity, Multiple Dose Effects
A03847	Acute Toxicity, Mutagenicity, Tumorigenicity
A03848	Mutagenicity, Tumorigenicity
A03849	Acute Toxicity
A03850	Acute Toxicity
A03851	Acute Toxicity, Tumorigenicity, Skin and Eye Irritation, Multiple Dose
A03852	Acute Toxicity
A03853	Skin and Eye Irritation
A03854	Acute Toxicity, Mutagenicity, Tumorigenicity, Multiple Dose Effects
A03855	Non-toxicity
A03856	Acute Toxicity
A03857	Acute Toxicity
A03858	Acute Toxicity, Mutagenicity, Tumorigenicity, Skin and Eye Irritation, Multiple Dose Effects
A03859	Acute Toxicity
A03860	Acute Toxicity
A03861	Acute Toxicity, Mutagenicity, Tumorigenicity, Reproductive Effects,
A03862	Acute Toxicity
A03863	Acute Toxicity, Multiple Dose Effects
A03864	Acute Toxicity, Mutagenicity
A03865	Mutagenicity
A03866	Acute Toxicity, Mutagenicity, Tumorigenicity, Multiple Dose Effects
A03867	Acute Toxicity, Mutagenicity

A03868	Acute Toxicity, Skin and Eye Irritation
A03869	Acute Toxicity, Skin and Eye Irritation
A03870	Mutagenicity, Tumorigenicity
A03871	Multiple Dose Effects
A03872	Acute Toxicity, Mutagenicity, Multiple Dose Effects
A03873	Acute Toxicity, Mutagenicity, Skin and Eye Irritation, Multiple Dose Effects
A03874	Acute Toxicity
A03875	Acute Toxicity
A03876	Reproductive Effects
A03877	Acute Toxicity
A03878	Acute Toxicity, Multiple Dose Effects
A03879	Acute Toxicity
A03880	Acute Toxicity, Mutagenicity, Skin and Eye Irritation, Reproductive Effects, Multiple Dose Effects
A03881	Acute Toxicity
A03882	Mutagenicity, Skin and Eye Irritation
A03883	Acute Toxicity
A03884	Acute Toxicity
A03885	Acute Toxicity, Multiple Dose Effects
A03886	Acute Toxicity
A03887	Acute Toxicity, Mutagenicity, Tumorigenicity, Multiple Dose Effects
A03888	Acute Toxicity, Mutagenicity
A03889	Acute Toxicity, Mutagenicity, Reproductive Effects, Multiple Dose Effects
A03890	Acute Toxicity, Reproductive Effects
A03891	Reproductive Effects
A03892	Acute Toxicity, Mutagenicity, Reproductive Effects, Multiple Dose Effects
A03893	Acute Toxicity, Mutagenicity
A03894	Acute Toxicity, Mutagenicity
A03895	Acute Toxicity
A03896	Acute Toxicity, Reproductive Effects, Multiple Dose Effects
A03897	Acute Toxicity
A03898	Mutagenicity
A03899	Acute Toxicity, Mutagenicity, Tumorigenicity, Multiple Dose Effects
A03900	Acute Toxicity, Mutagenicity
A03901	Acute Toxicity, Mutagenicity, Skin and Eye Irritation, Multiple Dose Effects
A03902	Acute Toxicity
A03903	Acute Toxicity
A03904	Acute Toxicity
A03905	Acute Toxicity
A03906	Acute Toxicity, Mutagenicity
A03907	Skin and Eye Irritation
A03908	Mutagenicity
A03909	Mutagenicity, Tumorigenicity
A03910	Acute Toxicity, Mutagenicity, Tumorigenicity, Multiple Dose Effects
A03911	Acute Toxicity
A03912	Acute Toxicity

A03913	Acute Toxicity, Mutagenicity
A03914	Acute Toxicity, Mutagenicity, Multiple Dose Effects
A03915	Acute Toxicity, Mutagenicity
A03916	Mutagenicity
A03917	Acute Toxicity
A03918	Acute Toxicity, Mutagenicity, Skin and Eye Irritation, Multiple Dose Effects
A03919	Acute Toxicity, Mutagenicity
A03920	Mutagenicity
A03921	Acute Toxicity
A03922	Acute Toxicity, Mutagenicity
A03923	Acute Toxicity
A03924	Mutagenicity
A03925	Acute Toxicity
A03926	Mutagenicity
A03927	Acute Toxicity
A03928	Mutagenicity
A03929	Acute Toxicity, Mutagenicity, Tumorigenicity, Reproductive Effects
A03930	Acute Toxicity
A03931	Acute Toxicity, Skin and Eye Irritation, Multiple Dose Effects
A03932	Acute Toxicity
A03933	Acute Toxicity, Mutagenicity
A03934	Mutagenicity
A03935	Acute Toxicity, Skin and Eye Irritation
A03936	Acute Toxicity
A03937	Skin and Eye Irritation
A03938	Acute Toxicity
A03939	Non-toxicity
A03940	Mutagenicity
A03941	Reproductive Effects
A03942	Acute Toxicity, Mutagenicity
A03943	Acute Toxicity
A03944	Acute Toxicity, Multiple Dose Effects
A03945	Acute Toxicity, Mutagenicity, Skin and Eye Irritation
A03946	Acute Toxicity
A03947	Mutagenicity, Tumorigenicity
A03948	Acute Toxicity, Mutagenicity, Tumorigenicity, Skin and Eye Irritation, Multiple Dose Effects
A03949	Acute Toxicity, Mutagenicity, Skin and Eye Irritation
A03950	Acute Toxicity, Multiple Dose Effects
A03951	Acute Toxicity
A03952	Acute Toxicity
A03953	Acute Toxicity
A03954	Acute Toxicity
A03955	Mutagenicity, Reproductive Effects
A03956	Acute Toxicity
A03957	Reproductive Effects

A03958	Acute Toxicity, Skin and Eye Irritation
A03959	Acute Toxicity, Multiple Dose Effects
A03960	Acute Toxicity
A03961	Acute Toxicity, Mutagenicity, Skin and Eye Irritation
A03962	Acute Toxicity, Skin and Eye Irritation, Multiple Dose Effects
A03963	Acute Toxicity, Mutagenicity
A03964	Acute Toxicity, Mutagenicity, Tumorigenicity, Skin and Eye Irritation, Multiple Dose Effects
A03965	Acute Toxicity
A03966	Acute Toxicity, Mutagenicity, Skin and Eye Irritation, Multiple Dose Effects
A03967	Acute Toxicity
A03968	Acute Toxicity, Mutagenicity, Skin and Eye Irritation, Reproductive Effects, Multiple Dose Effects
A03969	Reproductive Effects
A03970	Acute Toxicity
A03971	Acute Toxicity
A03972	Acute Toxicity
A03973	Acute Toxicity
A03974	Acute Toxicity, Mutagenicity, Tumorigenicity, Skin and Eye Irritation, Multiple Dose Effects
A03975	Acute Toxicity
A03976	Acute Toxicity
A03977	Skin and Eye Irritation
A03978	Acute Toxicity, Mutagenicity
A03979	Acute Toxicity, Skin and Eye Irritation
A03980	Acute Toxicity, Skin and Eye Irritation
A03981	Acute Toxicity, Mutagenicity, Reproductive Effects, Multiple Dose Effects
A03982	Acute Toxicity
A03983	Acute Toxicity
A03984	Acute Toxicity, Mutagenicity, Tumorigenicity, Reproductive Effects,
A03985	Tumorigenicity, Skin and Eye Irritation
A03986	Acute Toxicity
A03987	Acute Toxicity, Skin and Eye Irritation
A03988	Tumorigenicity
A03989	Acute Toxicity, Mutagenicity
A03990	Acute Toxicity, Skin and Eye Irritation, Multiple Dose Effects
A03991	Acute Toxicity, Multiple Dose Effects
A03992	Acute Toxicity, Mutagenicity
A03993	Acute Toxicity, Mutagenicity
A03994	Acute Toxicity, Skin and Eye Irritation
A03995	Acute Toxicity, Skin and Eye Irritation
A03996	Acute Toxicity
A03997	Acute Toxicity
A03998	Skin and Eye Irritation
A03999	Acute Toxicity
A04000	Acute Toxicity

A04001	Acute Toxicity, Mutagenicity
A04002	Acute Toxicity, Tumorigenicity
A04003	Acute Toxicity
A04004	Acute Toxicity, Mutagenicity, Tumorigenicity
A04005	Acute Toxicity, Multiple Dose Effects
A04006	Reproductive Effects
A04007	Acute Toxicity
A04008	Acute Toxicity
A04009	Acute Toxicity, Mutagenicity, Multiple Dose Effects
A04010	Acute Toxicity, Mutagenicity, Multiple Dose Effects
A04011	Acute Toxicity, Mutagenicity, Skin and Eye Irritation, Reproductive Effects, Multiple Dose Effects
A04012	Acute Toxicity, Multiple Dose Effects
A04013	Acute Toxicity
A04014	Acute Toxicity
A04015	Acute Toxicity
A04016	Acute Toxicity, Multiple Dose Effects
A04017	Acute Toxicity
A04018	Acute Toxicity, Skin and Eye Irritation
A04019	Acute Toxicity
A04020	Acute Toxicity, Mutagenicity, Reproductive Effects
A04021	Acute Toxicity
A04022	Acute Toxicity
A04023	Acute Toxicity, Mutagenicity, Multiple Dose Effects
A04024	Acute Toxicity
A04025	Acute Toxicity, Skin and Eye Irritation
A04026	Mutagenicity
A04027	Acute Toxicity, Multiple Dose Effects
A04028	Acute Toxicity, Mutagenicity, Tumorigenicity, Multiple Dose Effects
A04029	Acute Toxicity
A04030	Acute Toxicity, Skin and Eye Irritation, Multiple Dose Effects
A04031	Acute Toxicity, Mutagenicity, Tumorigenicity
A04032	Acute Toxicity
A04033	Acute Toxicity, Mutagenicity, Tumorigenicity, Multiple Dose Effects
A04034	Mutagenicity
A04035	Acute Toxicity, Mutagenicity, Tumorigenicity, Multiple Dose Effects
A04036	Acute Toxicity, Multiple Dose Effects
A04037	Acute Toxicity
A04038	Non-toxicity
A04039	Acute Toxicity, Skin and Eye Irritation
A04040	Acute Toxicity, Multiple Dose Effects
A04041	Acute Toxicity, Mutagenicity
A04042	Acute Toxicity
A04043	Mutagenicity
A04044	Acute Toxicity
A04045	Acute Toxicity

A04046	Acute Toxicity
A04047	Acute Toxicity, Mutagenicity, Multiple Dose Effects
A04048	Acute Toxicity, Mutagenicity, Multiple Dose Effects
A04049	Acute Toxicity, Skin and Eye Irritation
A04050	Acute Toxicity, Mutagenicity, Skin and Eye Irritation
A04051	Acute Toxicity, Tumorigenicity, Skin and Eye Irritation, Multiple Dose
A04052	Acute Toxicity
A04053	Acute Toxicity, Mutagenicity, Skin and Eye Irritation, Reproductive Effects
A04054	Acute Toxicity, Mutagenicity, Tumorigenicity, Reproductive Effects
A04055	Multiple Dose Effects
A04056	Acute Toxicity
A04057	Acute Toxicity
A04058	Acute Toxicity, Reproductive Effects
A04059	Acute Toxicity
A04060	Acute Toxicity
A04061	Acute Toxicity
A04062	Acute Toxicity, Mutagenicity
A04063	Mutagenicity, Tumorigenicity
A04064	Mutagenicity
A04065	Acute Toxicity
A04066	Acute Toxicity
A04067	Acute Toxicity
A04068	Acute Toxicity
A04069	Acute Toxicity, Multiple Dose Effects
A04070	Mutagenicity
A04071	Acute Toxicity
A04072	Acute Toxicity, Mutagenicity, Tumorigenicity, Multiple Dose Effects
A04073	Acute Toxicity, Tumorigenicity
A04074	Mutagenicity
A04075	Acute Toxicity, Mutagenicity, Skin and Eye Irritation, Multiple Dose Effects
A04076	Acute Toxicity, Multiple Dose Effects
A04077	Acute Toxicity
A04078	Acute Toxicity, Mutagenicity
A04079	Acute Toxicity, Mutagenicity
A04080	Acute Toxicity, Mutagenicity
A04081	Acute Toxicity, Mutagenicity, Multiple Dose Effects
A04082	Acute Toxicity, Tumorigenicity
A04083	Acute Toxicity
A04084	Acute Toxicity, Multiple Dose Effects
A04085	Acute Toxicity
A04086	Acute Toxicity, Mutagenicity
A04087	Acute Toxicity, Mutagenicity
A04088	Acute Toxicity, Skin and Eye Irritation
A04089	Acute Toxicity, Mutagenicity
A04090	Acute Toxicity
A04091	Acute Toxicity, Reproductive Effects

A04092	Acute Toxicity, Mutagenicity, Skin and Eye Irritation
A04093	Acute Toxicity
A04094	Mutagenicity
A04095	Acute Toxicity, Reproductive Effects
A04096	Skin and Eye Irritation
A04097	Acute Toxicity, Tumorigenicity, Skin and Eye Irritation
A04098	Acute Toxicity
A04099	Reproductive Effects
A04100	Acute Toxicity, Multiple Dose Effects
A04101	Acute Toxicity, Mutagenicity
A04102	Acute Toxicity, Mutagenicity
A04103	Acute Toxicity
A04104	Non-toxicity
A04105	Acute Toxicity, Mutagenicity, Tumorigenicity, Skin and Eye Irritation,
A04106	Acute Toxicity
A04107	Acute Toxicity, Mutagenicity
A04108	Acute Toxicity, Mutagenicity, Skin and Eye Irritation, Reproductive Effects, Multiple Dose Effects
A04109	Acute Toxicity, Multiple Dose Effects
A04110	Acute Toxicity, Skin and Eye Irritation, Reproductive Effects, Multiple Dose
A04111	Acute Toxicity, Mutagenicity
A04112	Acute Toxicity
A04113	Acute Toxicity, Skin and Eye Irritation
A04114	Acute Toxicity
A04115	Non-toxicity
A04116	Acute Toxicity
A04117	Acute Toxicity
A04118	Acute Toxicity, Mutagenicity
A04119	Mutagenicity
A04120	Acute Toxicity, Mutagenicity, Multiple Dose Effects
A04121	Acute Toxicity
A04122	Acute Toxicity
A04123	Mutagenicity
A04124	Acute Toxicity, Reproductive Effects, Multiple Dose Effects
A04125	Acute Toxicity, Mutagenicity
A04126	Acute Toxicity
A04127	Acute Toxicity
A04128	Acute Toxicity
A04129	Multiple Dose Effects
A04130	Acute Toxicity, Mutagenicity, Tumorigenicity, Skin and Eye Irritation
A04131	Acute Toxicity, Multiple Dose Effects
A04132	Mutagenicity, Skin and Eye Irritation
A04133	Acute Toxicity, Mutagenicity, Tumorigenicity, Reproductive Effects,
A04134	Mutagenicity
A04135	Acute Toxicity, Mutagenicity, Tumorigenicity, Multiple Dose Effects
A04136	Mutagenicity

A04137	Mutagenicity
A04138	Acute Toxicity, Mutagenicity, Skin and Eye Irritation, Reproductive Effects, Multiple Dose Effects
A04139	Acute Toxicity, Mutagenicity, Tumorigenicity, Skin and Eye Irritation,
A04140	Acute Toxicity, Mutagenicity
A04141	Reproductive Effects
A04142	Multiple Dose Effects
A04143	Multiple Dose Effects
A04144	Acute Toxicity, Multiple Dose Effects
A04145	Tumorigenicity, Reproductive Effects, Multiple Dose Effects
A04146	Acute Toxicity, Skin and Eye Irritation
A04147	Acute Toxicity
A04148	Acute Toxicity
A04149	Mutagenicity, Tumorigenicity
A04150	Acute Toxicity, Mutagenicity, Tumorigenicity, Reproductive Effects
A04151	Acute Toxicity, Mutagenicity, Tumorigenicity, Reproductive Effects
A04152	Acute Toxicity, Mutagenicity
A04153	Acute Toxicity
A04154	Mutagenicity
A04155	Acute Toxicity, Skin and Eye Irritation
A04156	Acute Toxicity
A04157	Acute Toxicity, Mutagenicity
A04158	Acute Toxicity
A04159	Mutagenicity
A04160	Acute Toxicity
A04161	Reproductive Effects
A04162	Acute Toxicity, Mutagenicity
A04163	Acute Toxicity, Mutagenicity, Tumorigenicity, Skin and Eye Irritation,
A04164	Acute Toxicity
A04165	Mutagenicity
A04166	Mutagenicity
A04167	Acute Toxicity, Mutagenicity
A04168	Acute Toxicity, Mutagenicity
A04169	Acute Toxicity
A04170	Acute Toxicity
A04171	Acute Toxicity, Mutagenicity
A04172	Acute Toxicity, Mutagenicity
A04173	Acute Toxicity
A04174	Mutagenicity, Reproductive Effects
A04175	Non-toxicity
A04176	Mutagenicity
A04177	Acute Toxicity
A04178	Acute Toxicity
A04179	Acute Toxicity, Mutagenicity, Skin and Eye Irritation, Reproductive Effects, Multiple Dose Effects
A04180	Acute Toxicity

A04181	Acute Toxicity
A04182	Mutagenicity
A04183	Mutagenicity
A04184	Acute Toxicity, Mutagenicity
A04185	Acute Toxicity, Mutagenicity
A04186	Mutagenicity
A04187	Mutagenicity
A04188	Acute Toxicity
A04189	Acute Toxicity, Mutagenicity
A04190	Acute Toxicity, Mutagenicity
A04191	Mutagenicity
A04192	Acute Toxicity, Mutagenicity
A04193	Acute Toxicity, Mutagenicity, Tumorigenicity
A04194	Acute Toxicity
A04195	Mutagenicity
A04196	Acute Toxicity
A04197	Acute Toxicity
A04198	Acute Toxicity
A04199	Acute Toxicity
A04200	Acute Toxicity
A04201	Acute Toxicity
A04202	Mutagenicity
A04203	Mutagenicity
A04204	Mutagenicity
A04205	Acute Toxicity
A04206	Acute Toxicity, Skin and Eye Irritation
A04207	Acute Toxicity
A04208	Acute Toxicity, Tumorigenicity
A04209	Acute Toxicity
A04210	Mutagenicity
A04211	Acute Toxicity, Mutagenicity, Multiple Dose Effects
A04212	Acute Toxicity, Mutagenicity, Tumorigenicity, Skin and Eye Irritation, Multiple Dose Effects
A04213	Acute Toxicity, Mutagenicity
A04214	Mutagenicity
A04215	Acute Toxicity
A04216	Acute Toxicity
A04217	Acute Toxicity, Skin and Eye Irritation
A04218	Acute Toxicity
A04219	Acute Toxicity
A04220	Mutagenicity
A04221	Acute Toxicity
A04222	Multiple Dose Effects
A04223	Acute Toxicity
A04224	Acute Toxicity
A04225	Acute Toxicity

A04226	Mutagenicity
A04227	Mutagenicity
A04228	Acute Toxicity, Mutagenicity
A04229	Acute Toxicity
A04230	Non-toxicity
A04231	Acute Toxicity
A04232	Reproductive Effects
A04233	Acute Toxicity
A04234	Acute Toxicity
A04235	Acute Toxicity, Reproductive Effects
A04236	Acute Toxicity, Mutagenicity
A04237	Acute Toxicity, Mutagenicity, Skin and Eye Irritation
A04238	Acute Toxicity, Skin and Eye Irritation
A04239	Acute Toxicity, Mutagenicity, Tumorigenicity, Reproductive Effects
A04240	Acute Toxicity, Multiple Dose Effects
A04241	Mutagenicity
A04242	Acute Toxicity
A04243	Acute Toxicity, Multiple Dose Effects
A04244	Acute Toxicity, Skin and Eye Irritation, Multiple Dose Effects
A04245	Acute Toxicity, Skin and Eye Irritation, Reproductive Effects, Multiple Dose
A04246	Mutagenicity, Reproductive Effects, Multiple Dose Effects
A04247	Acute Toxicity
A04248	Acute Toxicity, Mutagenicity, Skin and Eye Irritation
A04249	Acute Toxicity, Mutagenicity, Tumorigenicity
A04250	Skin and Eye Irritation
A04251	Acute Toxicity, Tumorigenicity
A04252	Acute Toxicity, Mutagenicity, Tumorigenicity
A04253	Acute Toxicity
A04254	Acute Toxicity
A04255	Acute Toxicity
A04256	Acute Toxicity, Mutagenicity, Skin and Eye Irritation, Reproductive Effects, Multiple Dose Effects
A04257	Mutagenicity
A04258	Acute Toxicity, Mutagenicity, Reproductive Effects, Multiple Dose Effects
A04259	Acute Toxicity
A04260	Acute Toxicity
A04261	Acute Toxicity, Mutagenicity, Tumorigenicity
A04262	Acute Toxicity, Multiple Dose Effects
A04263	Acute Toxicity, Mutagenicity, Skin and Eye Irritation, Reproductive Effects, Multiple Dose Effects
A04264	Acute Toxicity, Skin and Eye Irritation, Multiple Dose Effects
A04265	Acute Toxicity, Mutagenicity, Tumorigenicity
A04266	Acute Toxicity, Mutagenicity, Tumorigenicity, Reproductive Effects
A04267	Acute Toxicity, Multiple Dose Effects
A04268	Acute Toxicity
A04269	Acute Toxicity

A04270	Acute Toxicity, Skin and Eye Irritation, Multiple Dose Effects
A04271	Acute Toxicity
A04272	Acute Toxicity, Multiple Dose Effects
A04273	Mutagenicity, Multiple Dose Effects
A04274	Acute Toxicity
A04275	Acute Toxicity, Mutagenicity, Tumorigenicity, Multiple Dose Effects
A04276	Acute Toxicity
A04277	Acute Toxicity
A04278	Tumorigenicity
A04279	Acute Toxicity, Mutagenicity, Tumorigenicity, Multiple Dose Effects
A04280	Acute Toxicity, Skin and Eye Irritation
A04281	Acute Toxicity, Mutagenicity, Tumorigenicity, Multiple Dose Effects
A04282	Acute Toxicity, Mutagenicity
A04283	Acute Toxicity, Skin and Eye Irritation
A04284	Acute Toxicity
A04285	Acute Toxicity, Mutagenicity
A04286	Acute Toxicity, Skin and Eye Irritation, Multiple Dose Effects
A04287	Acute Toxicity
A04288	Non-toxicity
A04289	Mutagenicity
A04290	Acute Toxicity, Mutagenicity
A04291	Acute Toxicity, Mutagenicity
A04292	Acute Toxicity, Mutagenicity, Tumorigenicity, Reproductive Effects,
A04293	Non-toxicity
A04294	Mutagenicity, Tumorigenicity, Multiple Dose Effects
A04295	Acute Toxicity
A04296	Acute Toxicity, Mutagenicity, Tumorigenicity, Multiple Dose Effects
A04297	Non-toxicity
A04298	Acute Toxicity, Multiple Dose Effects
A04299	Acute Toxicity, Multiple Dose Effects
A04300	Acute Toxicity
A04301	Acute Toxicity, Multiple Dose Effects
A04302	Acute Toxicity
A04303	Acute Toxicity, Mutagenicity, Skin and Eye Irritation
A04304	Mutagenicity
A04305	Acute Toxicity, Mutagenicity
A04306	Acute Toxicity
A04307	Acute Toxicity, Mutagenicity, Tumorigenicity, Skin and Eye Irritation, Multiple Dose Effects
A04308	Acute Toxicity, Skin and Eye Irritation, Multiple Dose Effects
A04309	Acute Toxicity
A04310	Mutagenicity, Multiple Dose Effects
A04311	Acute Toxicity, Multiple Dose Effects
A04312	Acute Toxicity, Skin and Eye Irritation
A04313	Acute Toxicity, Skin and Eye Irritation
A04314	Acute Toxicity, Multiple Dose Effects

A04315	Acute Toxicity
A04316	Acute Toxicity, Mutagenicity
A04317	Acute Toxicity
A04318	Acute Toxicity, Mutagenicity, Multiple Dose Effects
A04319	Mutagenicity
A04320	Acute Toxicity, Mutagenicity, Skin and Eye Irritation
A04321	Acute Toxicity, Mutagenicity, Tumorigenicity
A04322	Acute Toxicity, Multiple Dose Effects
A04323	Non-toxicity
A04324	Acute Toxicity, Mutagenicity, Tumorigenicity
A04325	Acute Toxicity
A04326	Acute Toxicity, Mutagenicity, Skin and Eye Irritation
A04327	Acute Toxicity
A04328	Mutagenicity
A04329	Mutagenicity
A04330	Mutagenicity
A04331	Mutagenicity, Tumorigenicity
A04332	Acute Toxicity
A04333	Acute Toxicity, Mutagenicity
A04334	Acute Toxicity, Mutagenicity, Skin and Eye Irritation, Multiple Dose Effects
A04335	Acute Toxicity
A04336	Mutagenicity
A04337	Mutagenicity, Tumorigenicity, Multiple Dose Effects
A04338	Acute Toxicity, Tumorigenicity
A04339	Acute Toxicity
A04340	Mutagenicity
A04341	Acute Toxicity
A04342	Acute Toxicity
A04343	Acute Toxicity, Mutagenicity, Reproductive Effects
A04344	Mutagenicity
A04345	Non-toxicity
A04346	Acute Toxicity, Multiple Dose Effects
A04347	Tumorigenicity
A04348	Acute Toxicity
A04349	Acute Toxicity, Mutagenicity, Tumorigenicity, Skin and Eye Irritation, Multiple Dose Effects
A04350	Mutagenicity
A04351	Acute Toxicity, Reproductive Effects
A04352	Acute Toxicity, Mutagenicity
A04353	Mutagenicity
A04354	Mutagenicity
A04355	Acute Toxicity, Mutagenicity
A04356	Mutagenicity
A04357	Skin and Eye Irritation
A04358	Mutagenicity, Tumorigenicity
A04359	Mutagenicity

A04360	Acute Toxicity, Mutagenicity, Tumorigenicity
A04361	Acute Toxicity, Mutagenicity, Tumorigenicity, Multiple Dose Effects
A04362	Acute Toxicity, Mutagenicity, Multiple Dose Effects
A04363	Acute Toxicity
A04364	Acute Toxicity
A04365	Acute Toxicity, Multiple Dose Effects
A04366	Acute Toxicity
A04367	Acute Toxicity
A04368	Acute Toxicity, Mutagenicity
A04369	Acute Toxicity, Mutagenicity, Reproductive Effects, Multiple Dose Effects
A04370	Mutagenicity
A04371	Acute Toxicity, Mutagenicity
A04372	Acute Toxicity, Mutagenicity, Tumorigenicity, Multiple Dose Effects
A04373	Acute Toxicity
A04374	Mutagenicity
A04375	Mutagenicity
A04376	Mutagenicity
A04377	Skin and Eye Irritation
A04378	Acute Toxicity, Mutagenicity
A04379	Acute Toxicity, Multiple Dose Effects
A04380	Acute Toxicity
A04381	Mutagenicity
A04382	Mutagenicity
A04383	Mutagenicity
A04384	Acute Toxicity, Mutagenicity, Skin and Eye Irritation
A04385	Mutagenicity
A04386	Acute Toxicity, Mutagenicity, Tumorigenicity
A04387	Acute Toxicity, Mutagenicity
A04388	Acute Toxicity
A04389	Mutagenicity
A04390	Acute Toxicity, Mutagenicity, Skin and Eye Irritation
A04391	Acute Toxicity, Multiple Dose Effects
A04392	Mutagenicity, Skin and Eye Irritation
A04393	Mutagenicity
A04394	Mutagenicity, Tumorigenicity
A04395	Mutagenicity, Tumorigenicity
A04396	Acute Toxicity, Mutagenicity
A04397	Acute Toxicity
A04398	Acute Toxicity
A04399	Acute Toxicity
A04400	Acute Toxicity
A04401	Acute Toxicity, Mutagenicity, Multiple Dose Effects
A04402	Mutagenicity
A04403	Mutagenicity
A04404	Mutagenicity
A04405	Mutagenicity, Skin and Eye Irritation

A04406	Acute Toxicity, Mutagenicity, Reproductive Effects
A04407	Tumorigenicity
A04408	Mutagenicity
A04409	Mutagenicity
A04410	Acute Toxicity, Mutagenicity, Tumorigenicity, Reproductive Effects,
A04411	Mutagenicity, Tumorigenicity
A04412	Tumorigenicity
A04413	Mutagenicity, Tumorigenicity
A04414	Mutagenicity
A04415	Tumorigenicity
A04416	Acute Toxicity, Mutagenicity
A04417	Acute Toxicity, Tumorigenicity
A04418	Acute Toxicity, Mutagenicity, Tumorigenicity, Reproductive Effects
A04419	Acute Toxicity
A04420	Acute Toxicity, Mutagenicity, Tumorigenicity, Skin and Eye Irritation, Multiple Dose Effects
A04421	Acute Toxicity
A04422	Non-toxicity
A04423	Mutagenicity, Skin and Eye Irritation
A04424	Mutagenicity
A04425	Acute Toxicity, Mutagenicity, Tumorigenicity, Reproductive Effects
A04426	Acute Toxicity
A04427	Acute Toxicity
A04428	Acute Toxicity
A04429	Acute Toxicity, Mutagenicity, Reproductive Effects
A04430	Acute Toxicity
A04431	Acute Toxicity
A04432	Acute Toxicity
A04433	Acute Toxicity
A04434	Acute Toxicity
A04435	Acute Toxicity, Mutagenicity, Tumorigenicity, Reproductive Effects,
A04436	Acute Toxicity, Multiple Dose Effects
A04437	Acute Toxicity, Reproductive Effects
A04438	Mutagenicity, Skin and Eye Irritation
A04439	Acute Toxicity
A04440	Acute Toxicity, Multiple Dose Effects
A04441	Mutagenicity
A04442	Acute Toxicity, Skin and Eye Irritation
A04443	Acute Toxicity, Mutagenicity, Skin and Eye Irritation
A04444	Acute Toxicity
A04445	Acute Toxicity, Mutagenicity
A04446	Acute Toxicity, Mutagenicity, Skin and Eye Irritation, Reproductive Effects, Multiple Dose Effects
A04447	Acute Toxicity
A04448	Acute Toxicity
A04449	Acute Toxicity, Mutagenicity, Tumorigenicity, Reproductive Effects,

A04450	Acute Toxicity
A04451	Mutagenicity
A04452	Acute Toxicity
A04453	Acute Toxicity, Tumorigenicity
A04454	Acute Toxicity, Skin and Eye Irritation, Multiple Dose Effects
A04455	Acute Toxicity
A04456	Acute Toxicity
A04457	Acute Toxicity
A04458	Acute Toxicity
A04459	Acute Toxicity, Mutagenicity
A04460	Mutagenicity
A04461	Acute Toxicity, Skin and Eye Irritation
A04462	Acute Toxicity
A04463	Acute Toxicity
A04464	Acute Toxicity, Mutagenicity, Skin and Eye Irritation
A04465	Mutagenicity, Tumorigenicity
A04466	Mutagenicity
A04467	Acute Toxicity, Mutagenicity
A04468	Acute Toxicity
A04469	Acute Toxicity
A04470	Acute Toxicity
A04471	Acute Toxicity
A04472	Non-toxicity
A04473	Mutagenicity
A04474	Mutagenicity, Tumorigenicity
A04475	Acute Toxicity, Mutagenicity
A04476	Acute Toxicity, Skin and Eye Irritation
A04477	Acute Toxicity
A04478	Acute Toxicity, Mutagenicity
A04479	Acute Toxicity
A04480	Acute Toxicity
A04481	Acute Toxicity, Multiple Dose Effects
A04482	Mutagenicity
A04483	Acute Toxicity, Mutagenicity, Skin and Eye Irritation
A04484	Acute Toxicity
A04485	Acute Toxicity
A04486	Mutagenicity, Skin and Eye Irritation
A04487	Acute Toxicity, Mutagenicity, Skin and Eye Irritation, Multiple Dose Effects
A04488	Acute Toxicity
A04489	Mutagenicity
A04490	Acute Toxicity
A04491	Acute Toxicity, Mutagenicity
A04492	Acute Toxicity, Mutagenicity
A04493	Acute Toxicity, Mutagenicity, Tumorigenicity
A04494	Acute Toxicity
A04495	Acute Toxicity, Reproductive Effects

A04496	Mutagenicity
A04497	Mutagenicity
A04498	Mutagenicity
A04499	Acute Toxicity, Skin and Eye Irritation
A04500	Acute Toxicity
A04501	Acute Toxicity, Skin and Eye Irritation
A04502	Acute Toxicity, Mutagenicity, Tumorigenicity
A04503	Acute Toxicity
A04504	Acute Toxicity, Reproductive Effects
A04505	Acute Toxicity, Mutagenicity
A04506	Acute Toxicity
A04507	Acute Toxicity
A04508	Acute Toxicity
A04509	Acute Toxicity, Mutagenicity, Reproductive Effects, Multiple Dose Effects
A04510	Acute Toxicity, Mutagenicity, Tumorigenicity, Skin and Eye Irritation,
A04511	Acute Toxicity
A04512	Acute Toxicity, Skin and Eye Irritation
A04513	Mutagenicity
A04514	Mutagenicity
A04515	Acute Toxicity, Mutagenicity, Skin and Eye Irritation, Multiple Dose Effects
A04516	Acute Toxicity, Skin and Eye Irritation
A04517	Acute Toxicity
A04518	Acute Toxicity
A04519	Acute Toxicity, Skin and Eye Irritation
A04520	Acute Toxicity
A04521	Acute Toxicity, Skin and Eye Irritation
A04522	Acute Toxicity
A04523	Acute Toxicity, Mutagenicity
A04524	Acute Toxicity
A04525	Acute Toxicity
A04526	Acute Toxicity
A04527	Acute Toxicity
A04528	Acute Toxicity, Skin and Eye Irritation
A04529	Acute Toxicity, Mutagenicity
A04530	Acute Toxicity, Mutagenicity, Multiple Dose Effects
A04531	Acute Toxicity, Mutagenicity
A04532	Acute Toxicity
A04533	Acute Toxicity, Mutagenicity, Skin and Eye Irritation, Reproductive Effects, Multiple Dose Effects
A04534	Acute Toxicity, Tumorigenicity
A04535	Acute Toxicity, Mutagenicity
A04536	Acute Toxicity, Mutagenicity, Skin and Eye Irritation, Reproductive Effects, Multiple Dose Effects
A04537	Acute Toxicity
A04538	Acute Toxicity, Multiple Dose Effects
A04539	Acute Toxicity

A04540	Acute Toxicity, Mutagenicity, Reproductive Effects, Multiple Dose Effects
A04541	Acute Toxicity, Mutagenicity
A04542	Acute Toxicity
A04543	Acute Toxicity, Mutagenicity, Reproductive Effects
A04544	Acute Toxicity
A04545	Acute Toxicity, Mutagenicity
A04546	Acute Toxicity, Skin and Eye Irritation
A04547	Acute Toxicity
A04548	Acute Toxicity, Mutagenicity
A04549	Acute Toxicity, Tumorigenicity
A04550	Acute Toxicity
A04551	Acute Toxicity
A04552	Acute Toxicity
A04553	Acute Toxicity, Mutagenicity, Skin and Eye Irritation, Multiple Dose Effects
A04554	Acute Toxicity
A04555	Acute Toxicity, Multiple Dose Effects
A04556	Mutagenicity
A04557	Acute Toxicity, Tumorigenicity, Reproductive Effects
A04558	Non-toxicity
A04559	Acute Toxicity
A04560	Acute Toxicity, Mutagenicity
A04561	Acute Toxicity, Mutagenicity
A04562	Acute Toxicity
A04563	Acute Toxicity
A04564	Acute Toxicity, Skin and Eye Irritation
A04565	Acute Toxicity, Multiple Dose Effects
A04566	Acute Toxicity, Multiple Dose Effects
A04567	Acute Toxicity, Multiple Dose Effects
A04568	Acute Toxicity, Mutagenicity, Reproductive Effects, Multiple Dose Effects
A04569	Acute Toxicity, Mutagenicity, Tumorigenicity
A04570	Acute Toxicity
A04571	Acute Toxicity, Mutagenicity, Tumorigenicity, Reproductive Effects
A04572	Acute Toxicity
A04573	Mutagenicity
A04574	Acute Toxicity, Mutagenicity
A04575	Mutagenicity
A04576	Acute Toxicity, Mutagenicity
A04577	Acute Toxicity, Multiple Dose Effects
A04578	Acute Toxicity, Skin and Eye Irritation, Reproductive Effects, Multiple Dose
A04579	Mutagenicity, Tumorigenicity
A04580	Mutagenicity, Tumorigenicity
A04581	Acute Toxicity
A04582	Acute Toxicity
A04583	Mutagenicity
A04584	Acute Toxicity, Multiple Dose Effects
A04585	Acute Toxicity, Mutagenicity, Multiple Dose Effects

A04586	Tumorigenicity
A04587	Reproductive Effects, Multiple Dose Effects
A04588	Acute Toxicity
A04589	Acute Toxicity, Skin and Eye Irritation, Multiple Dose Effects
A04590	Acute Toxicity
A04591	Acute Toxicity
A04592	Mutagenicity
A04593	Acute Toxicity
A04594	Acute Toxicity, Skin and Eye Irritation
A04595	Acute Toxicity, Mutagenicity, Tumorigenicity
A04596	Acute Toxicity, Mutagenicity, Skin and Eye Irritation
A04597	Acute Toxicity
A04598	Mutagenicity
A04599	Mutagenicity
A04600	Acute Toxicity, Mutagenicity, Skin and Eye Irritation, Multiple Dose Effects
A04601	Acute Toxicity, Skin and Eye Irritation, Reproductive Effects, Multiple Dose
A04602	Acute Toxicity, Skin and Eye Irritation
A04603	Multiple Dose Effects
A04604	Acute Toxicity
A04605	Acute Toxicity
A04606	Acute Toxicity
A04607	Acute Toxicity, Skin and Eye Irritation
A04608	Acute Toxicity, Mutagenicity, Tumorigenicity, Skin and Eye Irritation, Multiple Dose Effects
A04609	Acute Toxicity, Skin and Eye Irritation
A04610	Acute Toxicity
A04611	Acute Toxicity, Skin and Eye Irritation
A04612	Acute Toxicity
A04613	Non-toxicity
A04614	Acute Toxicity
A04615	Acute Toxicity
A04616	Acute Toxicity
A04617	Acute Toxicity, Multiple Dose Effects
A04618	Tumorigenicity
A04619	Mutagenicity
A04620	Acute Toxicity, Mutagenicity, Tumorigenicity, Skin and Eye Irritation, Multiple Dose Effects
A04621	Tumorigenicity
A04622	Acute Toxicity, Multiple Dose Effects
A04623	Acute Toxicity, Mutagenicity, Tumorigenicity, Reproductive Effects,
A04624	Acute Toxicity
A04625	Acute Toxicity
A04626	Acute Toxicity, Mutagenicity, Reproductive Effects
A04627	Acute Toxicity, Mutagenicity, Reproductive Effects, Multiple Dose Effects
A04628	Acute Toxicity
A04629	Acute Toxicity, Mutagenicity, Skin and Eye Irritation, Multiple Dose Effects

A04630	Multiple Dose Effects
A04631	Mutagenicity
A04632	Non-toxicity
A04633	Acute Toxicity, Mutagenicity, Reproductive Effects, Multiple Dose Effects
A04634	Mutagenicity
A04635	Acute Toxicity, Mutagenicity, Reproductive Effects, Multiple Dose Effects
A04636	Mutagenicity
A04637	Acute Toxicity, Mutagenicity, Tumorigenicity, Multiple Dose Effects
A04638	Acute Toxicity
A04639	Non-toxicity
A04640	Acute Toxicity
A04641	Acute Toxicity, Skin and Eye Irritation, Reproductive Effects, Multiple Dose
A04642	Acute Toxicity, Reproductive Effects, Multiple Dose Effects
A04643	Acute Toxicity
A04644	Acute Toxicity
A04645	Acute Toxicity, Multiple Dose Effects
A04646	Mutagenicity
A04647	Acute Toxicity, Multiple Dose Effects
A04648	Multiple Dose Effects
A04649	Mutagenicity
A04650	Acute Toxicity
A04651	Reproductive Effects, Multiple Dose Effects
A04652	Acute Toxicity, Mutagenicity, Skin and Eye Irritation, Reproductive Effects, Multiple Dose Effects
A04653	Acute Toxicity, Multiple Dose Effects
A04654	Acute Toxicity
A04655	Acute Toxicity
A04656	Acute Toxicity
A04657	Acute Toxicity
A04658	Acute Toxicity, Skin and Eye Irritation, Multiple Dose Effects
A04659	Acute Toxicity
A04660	Acute Toxicity, Mutagenicity, Skin and Eye Irritation, Multiple Dose Effects
A04661	Acute Toxicity, Mutagenicity, Reproductive Effects, Multiple Dose Effects
A04662	Acute Toxicity
A04663	Acute Toxicity
A04664	Acute Toxicity
A04665	Tumorigenicity
A04666	Acute Toxicity, Skin and Eye Irritation, Multiple Dose Effects
A04667	Acute Toxicity, Mutagenicity, Tumorigenicity, Skin and Eye Irritation, Multiple Dose Effects
A04668	Acute Toxicity, Mutagenicity, Skin and Eye Irritation, Reproductive Effects, Multiple Dose Effects
A04669	Acute Toxicity
A04670	Mutagenicity
A04671	Mutagenicity, Tumorigenicity, Multiple Dose Effects
A04672	Acute Toxicity

A04673	Acute Toxicity, Multiple Dose Effects
A04674	Acute Toxicity
A04675	Acute Toxicity
A04676	Acute Toxicity
A04677	Acute Toxicity
A04678	Mutagenicity
A04679	Acute Toxicity, Multiple Dose Effects
A04680	Acute Toxicity
A04681	Acute Toxicity, Mutagenicity, Skin and Eye Irritation, Reproductive Effects, Multiple Dose Effects
A04682	Acute Toxicity, Skin and Eye Irritation
A04683	Mutagenicity
A04684	Acute Toxicity
A04685	Non-toxicity
A04686	Acute Toxicity, Tumorigenicity, Reproductive Effects, Multiple Dose Effects
A04687	Acute Toxicity
A04688	Acute Toxicity
A04689	Acute Toxicity
A04690	Acute Toxicity, Mutagenicity, Skin and Eye Irritation
A04691	Acute Toxicity
A04692	Acute Toxicity, Mutagenicity
A04693	Acute Toxicity, Mutagenicity, Skin and Eye Irritation
A04694	Acute Toxicity
A04695	Acute Toxicity, Reproductive Effects
A04696	Acute Toxicity
A04697	Acute Toxicity
A04698	Acute Toxicity, Mutagenicity
A04699	Non-toxicity
A04700	Acute Toxicity, Mutagenicity, Skin and Eye Irritation
A04701	Acute Toxicity
A04702	Acute Toxicity
A04703	Acute Toxicity
A04704	Acute Toxicity, Mutagenicity, Skin and Eye Irritation
A04705	Acute Toxicity
A04706	Acute Toxicity
A04707	Acute Toxicity
A04708	Acute Toxicity, Multiple Dose Effects
A04709	Acute Toxicity
A04710	Acute Toxicity, Mutagenicity, Tumorigenicity, Skin and Eye Irritation,
A04711	Mutagenicity
A04712	Acute Toxicity
A04713	Acute Toxicity
A04714	Acute Toxicity, Reproductive Effects, Multiple Dose Effects
A04715	Acute Toxicity, Mutagenicity, Tumorigenicity, Reproductive Effects,
A04716	Acute Toxicity
A04717	Acute Toxicity, Tumorigenicity

A04718	Acute Toxicity
A04719	Mutagenicity
A04720	Acute Toxicity, Mutagenicity, Tumorigenicity, Reproductive Effects
A04721	Acute Toxicity
A04722	Acute Toxicity, Reproductive Effects
A04723	Acute Toxicity
A04724	Acute Toxicity, Mutagenicity, Multiple Dose Effects
A04725	Acute Toxicity
A04726	Acute Toxicity, Skin and Eye Irritation
A04727	Acute Toxicity
A04728	Acute Toxicity, Mutagenicity, Skin and Eye Irritation, Multiple Dose Effects
A04729	Acute Toxicity, Mutagenicity, Tumorigenicity, Skin and Eye Irritation, Multiple Dose Effects
A04730	Acute Toxicity
A04731	Acute Toxicity, Skin and Eye Irritation
A04732	Acute Toxicity, Mutagenicity
A04733	Acute Toxicity, Multiple Dose Effects
A04734	Acute Toxicity
A04735	Acute Toxicity, Multiple Dose Effects
A04736	Acute Toxicity, Multiple Dose Effects
A04737	Acute Toxicity, Mutagenicity, Tumorigenicity, Reproductive Effects,
A04738	Acute Toxicity
A04739	Acute Toxicity, Mutagenicity, Multiple Dose Effects
A04740	Acute Toxicity
A04741	Acute Toxicity, Skin and Eye Irritation
A04742	Acute Toxicity, Skin and Eye Irritation, Reproductive Effects, Multiple Dose
A04743	Mutagenicity
A04744	Acute Toxicity
A04745	Acute Toxicity
A04746	Acute Toxicity
A04747	Mutagenicity, Skin and Eye Irritation
A04748	Acute Toxicity, Skin and Eye Irritation
A04749	Acute Toxicity, Reproductive Effects
A04750	Acute Toxicity, Mutagenicity
A04751	Acute Toxicity
A04752	Acute Toxicity, Multiple Dose Effects
A04753	Acute Toxicity
A04754	Acute Toxicity
A04755	Acute Toxicity, Skin and Eye Irritation
A04756	Acute Toxicity, Mutagenicity, Skin and Eye Irritation
A04757	Acute Toxicity
A04758	Acute Toxicity, Multiple Dose Effects
A04759	Acute Toxicity, Multiple Dose Effects
A04760	Acute Toxicity
A04761	Acute Toxicity, Reproductive Effects
A04762	Acute Toxicity

A04763	Acute Toxicity
A04764	Acute Toxicity, Mutagenicity
A04765	Acute Toxicity, Multiple Dose Effects
A04766	Acute Toxicity, Skin and Eye Irritation
A04767	Acute Toxicity
A04768	Acute Toxicity
A04769	Acute Toxicity
A04770	Acute Toxicity, Tumorigenicity
A04771	Acute Toxicity
A04772	Mutagenicity, Multiple Dose Effects
A04773	Acute Toxicity
A04774	Acute Toxicity
A04775	Acute Toxicity
A04776	Acute Toxicity, Mutagenicity, Multiple Dose Effects
A04777	Acute Toxicity
A04778	Acute Toxicity, Reproductive Effects, Multiple Dose Effects
A04779	Acute Toxicity, Mutagenicity, Reproductive Effects, Multiple Dose Effects
A04780	Acute Toxicity, Tumorigenicity, Multiple Dose Effects
A04781	Acute Toxicity, Tumorigenicity
A04782	Acute Toxicity, Mutagenicity, Reproductive Effects, Multiple Dose Effects
A04783	Acute Toxicity, Mutagenicity, Tumorigenicity, Skin and Eye Irritation,
A04784	Acute Toxicity, Skin and Eye Irritation, Reproductive Effects
A04785	Acute Toxicity
A04786	Acute Toxicity
A04787	Acute Toxicity, Mutagenicity, Tumorigenicity
A04788	Acute Toxicity, Mutagenicity
A04789	Acute Toxicity, Reproductive Effects, Multiple Dose Effects
A04790	Acute Toxicity, Mutagenicity, Tumorigenicity, Reproductive Effects,
A04791	Acute Toxicity, Mutagenicity, Multiple Dose Effects
A04792	Acute Toxicity, Mutagenicity, Reproductive Effects
A04793	Acute Toxicity
A04794	Acute Toxicity, Reproductive Effects, Multiple Dose Effects
A04795	Acute Toxicity
A04796	Acute Toxicity
A04797	Acute Toxicity
A04798	Acute Toxicity
A04799	Acute Toxicity
A04800	Acute Toxicity, Multiple Dose Effects
A04801	Acute Toxicity, Skin and Eye Irritation, Multiple Dose Effects
A04802	Acute Toxicity, Multiple Dose Effects
A04803	Acute Toxicity, Skin and Eye Irritation, Multiple Dose Effects
A04804	Acute Toxicity
A04805	Acute Toxicity, Mutagenicity, Tumorigenicity, Skin and Eye Irritation, Multiple Dose Effects
A04806	Acute Toxicity, Skin and Eye Irritation
A04807	Mutagenicity

A04808	Acute Toxicity, Skin and Eye Irritation
A04809	Tumorigenicity
A04810	Acute Toxicity
A04811	Acute Toxicity
A04812	Acute Toxicity
A04813	Acute Toxicity
A04814	Multiple Dose Effects
A04815	Acute Toxicity, Tumorigenicity
A04816	Acute Toxicity, Mutagenicity, Skin and Eye Irritation, Multiple Dose Effects
A04817	Acute Toxicity
A04818	Acute Toxicity
A04819	Acute Toxicity
A04820	Acute Toxicity
A04821	Acute Toxicity
A04822	Acute Toxicity
A04823	Acute Toxicity
A04824	Acute Toxicity
A04825	Acute Toxicity
A04826	Acute Toxicity, Skin and Eye Irritation
A04827	Acute Toxicity, Reproductive Effects
A04828	Mutagenicity
A04829	Acute Toxicity
A04830	Mutagenicity, Tumorigenicity
A04831	Acute Toxicity
A04832	Acute Toxicity, Mutagenicity, Tumorigenicity
A04833	Acute Toxicity
A04834	Acute Toxicity, Mutagenicity, Multiple Dose Effects
A04835	Reproductive Effects, Multiple Dose Effects
A04836	Acute Toxicity
A04837	Acute Toxicity
A04838	Acute Toxicity, Reproductive Effects, Multiple Dose Effects
A04839	Acute Toxicity, Mutagenicity, Reproductive Effects, Multiple Dose Effects
A04840	Mutagenicity, Tumorigenicity
A04841	Mutagenicity
A04842	Acute Toxicity
A04843	Acute Toxicity, Mutagenicity, Skin and Eye Irritation, Reproductive Effects, Multiple Dose Effects
A04844	Acute Toxicity, Reproductive Effects
A04845	Acute Toxicity, Mutagenicity, Multiple Dose Effects
A04846	Acute Toxicity
A04847	Acute Toxicity
A04848	Acute Toxicity, Mutagenicity, Tumorigenicity, Reproductive Effects
A04849	Acute Toxicity, Mutagenicity
A04850	Acute Toxicity
A04851	Acute Toxicity, Skin and Eye Irritation
A04852	Acute Toxicity, Skin and Eye Irritation

A04853	Acute Toxicity
A04854	Acute Toxicity, Mutagenicity, Skin and Eye Irritation
A04855	Skin and Eye Irritation
A04856	Acute Toxicity, Mutagenicity, Skin and Eye Irritation
A04857	Acute Toxicity
A04858	Acute Toxicity, Mutagenicity, Tumorigenicity, Reproductive Effects,
A04859	Acute Toxicity, Reproductive Effects
A04860	Acute Toxicity, Skin and Eye Irritation
A04861	Acute Toxicity, Mutagenicity, Skin and Eye Irritation, Reproductive Effects, Multiple Dose Effects
A04862	Acute Toxicity, Multiple Dose Effects
A04863	Acute Toxicity
A04864	Non-toxicity
A04865	Acute Toxicity, Multiple Dose Effects
A04866	Mutagenicity
A04867	Acute Toxicity, Mutagenicity, Skin and Eye Irritation, Multiple Dose Effects
A04868	Acute Toxicity
A04869	Acute Toxicity, Mutagenicity
A04870	Mutagenicity, Multiple Dose Effects
A04871	Mutagenicity
A04872	Acute Toxicity
A04873	Acute Toxicity
A04874	Mutagenicity
A04875	Acute Toxicity, Skin and Eye Irritation
A04876	Acute Toxicity
A04877	Acute Toxicity, Mutagenicity, Tumorigenicity, Multiple Dose Effects
A04878	Acute Toxicity
A04879	Acute Toxicity
A04880	Mutagenicity
A04881	Acute Toxicity, Multiple Dose Effects
A04882	Mutagenicity
A04883	Acute Toxicity, Mutagenicity, Skin and Eye Irritation, Reproductive Effects, Multiple Dose Effects
A04884	Mutagenicity, Tumorigenicity
A04885	Acute Toxicity
A04886	Acute Toxicity, Mutagenicity, Skin and Eye Irritation, Reproductive Effects, Multiple Dose Effects
A04887	Acute Toxicity
A04888	Mutagenicity
A04889	Mutagenicity, Tumorigenicity, Skin and Eye Irritation
A04890	Mutagenicity
A04891	Acute Toxicity
A04892	Mutagenicity, Tumorigenicity
A04893	Reproductive Effects
A04894	Reproductive Effects
A04895	Acute Toxicity, Reproductive Effects, Multiple Dose Effects

A04896	Acute Toxicity, Reproductive Effects
A04897	Reproductive Effects
A04898	Acute Toxicity, Multiple Dose Effects
A04899	Acute Toxicity
A04900	Acute Toxicity
A04901	Acute Toxicity
A04902	Acute Toxicity, Mutagenicity, Skin and Eye Irritation, Reproductive Effects, Multiple Dose Effects
A04903	Acute Toxicity, Mutagenicity, Tumorigenicity
A04904	Acute Toxicity, Mutagenicity, Skin and Eye Irritation
A04905	Mutagenicity, Skin and Eye Irritation
A04906	Acute Toxicity, Mutagenicity
A04907	Tumorigenicity
A04908	Acute Toxicity, Mutagenicity, Tumorigenicity, Skin and Eye Irritation,
A04909	Acute Toxicity
A04910	Acute Toxicity, Skin and Eye Irritation
A04911	Acute Toxicity, Mutagenicity, Skin and Eye Irritation, Multiple Dose Effects
A04912	Acute Toxicity, Mutagenicity
A04913	Acute Toxicity, Multiple Dose Effects
A04914	Non-toxicity
A04915	Mutagenicity
A04916	Acute Toxicity, Mutagenicity
A04917	Acute Toxicity
A04918	Multiple Dose Effects
A04919	Multiple Dose Effects
A04920	Mutagenicity, Tumorigenicity
A04921	Mutagenicity
A04922	Acute Toxicity, Multiple Dose Effects
A04923	Mutagenicity
A04924	Acute Toxicity
A04925	Mutagenicity
A04926	Mutagenicity
A04927	Acute Toxicity, Tumorigenicity, Skin and Eye Irritation, Multiple Dose
A04928	Mutagenicity, Tumorigenicity
A04929	Reproductive Effects
A04930	Acute Toxicity
A04931	Acute Toxicity
A04932	Acute Toxicity
A04933	Mutagenicity
A04934	Acute Toxicity
A04935	Acute Toxicity
A04936	Acute Toxicity
A04937	Acute Toxicity, Mutagenicity
A04938	Acute Toxicity
A04939	Acute Toxicity, Mutagenicity
A04940	Acute Toxicity, Mutagenicity, Multiple Dose Effects

A04941	Acute Toxicity
A04942	Acute Toxicity, Mutagenicity, Skin and Eye Irritation
A04943	Acute Toxicity
A04944	Acute Toxicity, Mutagenicity, Tumorigenicity, Reproductive Effects
A04945	Acute Toxicity, Mutagenicity, Tumorigenicity, Skin and Eye Irritation,
A04946	Acute Toxicity, Mutagenicity, Multiple Dose Effects
A04947	Acute Toxicity, Mutagenicity, Tumorigenicity
A04948	Mutagenicity
A04949	Acute Toxicity, Mutagenicity, Tumorigenicity, Reproductive Effects
A04950	Acute Toxicity, Skin and Eye Irritation
A04951	Acute Toxicity
A04952	Acute Toxicity, Skin and Eye Irritation
A04953	Mutagenicity
A04954	Acute Toxicity
A04955	Acute Toxicity, Mutagenicity, Tumorigenicity
A04956	Acute Toxicity
A04957	Acute Toxicity
A04958	Acute Toxicity
A04959	Acute Toxicity
A04960	Acute Toxicity, Mutagenicity, Tumorigenicity, Multiple Dose Effects
A04961	Acute Toxicity
A04962	Mutagenicity
A04963	Acute Toxicity, Mutagenicity, Skin and Eye Irritation
A04964	Acute Toxicity, Mutagenicity, Tumorigenicity, Multiple Dose Effects
A04965	Acute Toxicity, Mutagenicity, Skin and Eye Irritation, Multiple Dose Effects
A04966	Acute Toxicity
A04967	Mutagenicity
A04968	Acute Toxicity
A04969	Acute Toxicity
A04970	Acute Toxicity
A04971	Acute Toxicity, Mutagenicity, Skin and Eye Irritation
A04972	Acute Toxicity, Mutagenicity, Tumorigenicity, Reproductive Effects
A04973	Acute Toxicity
A04974	Acute Toxicity, Mutagenicity, Multiple Dose Effects
A04975	Mutagenicity, Multiple Dose Effects
A04976	Tumorigenicity
A04977	Acute Toxicity, Mutagenicity, Reproductive Effects
A04978	Acute Toxicity, Mutagenicity, Skin and Eye Irritation, Multiple Dose Effects
A04979	Acute Toxicity
A04980	Acute Toxicity
A04981	Acute Toxicity, Mutagenicity, Multiple Dose Effects
A04982	Acute Toxicity
A04983	Acute Toxicity
A04984	Mutagenicity, Tumorigenicity
A04985	Acute Toxicity, Mutagenicity, Tumorigenicity, Multiple Dose Effects
A04986	Acute Toxicity

A04987	Acute Toxicity
A04988	Acute Toxicity, Mutagenicity
A04989	Acute Toxicity
A04990	Acute Toxicity
A04991	Acute Toxicity, Skin and Eye Irritation
A04992	Acute Toxicity, Skin and Eye Irritation
A04993	Acute Toxicity
A04994	Mutagenicity, Tumorigenicity
A04995	Acute Toxicity, Mutagenicity
A04996	Acute Toxicity
A04997	Acute Toxicity
A04998	Acute Toxicity, Multiple Dose Effects
A04999	Acute Toxicity
A05000	Mutagenicity
A05001	Mutagenicity, Tumorigenicity
A05002	Acute Toxicity
A05003	Acute Toxicity, Mutagenicity
A05004	Acute Toxicity
A05005	Acute Toxicity
A05006	Acute Toxicity, Mutagenicity, Multiple Dose Effects
A05007	Acute Toxicity, Mutagenicity, Tumorigenicity, Skin and Eye Irritation, Multiple Dose Effects
A05008	Mutagenicity, Reproductive Effects
A05009	Mutagenicity, Skin and Eye Irritation
A05010	Acute Toxicity, Mutagenicity
A05011	Acute Toxicity
A05012	Mutagenicity
A05013	Acute Toxicity, Multiple Dose Effects
A05014	Acute Toxicity, Mutagenicity, Multiple Dose Effects
A05015	Acute Toxicity, Mutagenicity
A05016	Acute Toxicity, Mutagenicity
A05017	Acute Toxicity
A05018	Acute Toxicity
A05019	Acute Toxicity
A05020	Mutagenicity
A05021	Multiple Dose Effects
A05022	Mutagenicity, Tumorigenicity
A05023	Acute Toxicity, Reproductive Effects
A05024	Multiple Dose Effects
A05025	Acute Toxicity, Reproductive Effects
A05026	Acute Toxicity, Mutagenicity, Multiple Dose Effects
A05027	Acute Toxicity, Skin and Eye Irritation
A05028	Acute Toxicity, Tumorigenicity, Reproductive Effects, Multiple Dose Effects
A05029	Acute Toxicity
A05030	Acute Toxicity
A05031	Acute Toxicity, Mutagenicity, Tumorigenicity

A05032	Acute Toxicity
A05033	Mutagenicity
A05034	Acute Toxicity, Mutagenicity, Tumorigenicity, Skin and Eye Irritation, Multiple Dose Effects
A05035	Acute Toxicity, Skin and Eye Irritation
A05036	Acute Toxicity, Skin and Eye Irritation
A05037	Acute Toxicity
A05038	Acute Toxicity
A05039	Acute Toxicity, Skin and Eye Irritation
A05040	Acute Toxicity, Multiple Dose Effects
A05041	Acute Toxicity
A05042	Acute Toxicity
A05043	Acute Toxicity, Multiple Dose Effects
A05044	Acute Toxicity
A05045	Acute Toxicity, Mutagenicity, Multiple Dose Effects
A05046	Acute Toxicity, Mutagenicity
A05047	Acute Toxicity
A05048	Acute Toxicity
A05049	Acute Toxicity
A05050	Acute Toxicity, Mutagenicity, Multiple Dose Effects
A05051	Acute Toxicity, Reproductive Effects, Multiple Dose Effects
A05052	Acute Toxicity, Skin and Eye Irritation
A05053	Acute Toxicity
A05054	Mutagenicity
A05055	Acute Toxicity, Mutagenicity, Multiple Dose Effects
A05056	Acute Toxicity
A05057	Acute Toxicity
A05058	Acute Toxicity
A05059	Acute Toxicity
A05060	Acute Toxicity
A05061	Reproductive Effects
A05062	Reproductive Effects
A05063	Mutagenicity
A05064	Multiple Dose Effects
A05065	Acute Toxicity, Mutagenicity, Tumorigenicity
A05066	Skin and Eye Irritation
A05067	Acute Toxicity
A05068	Acute Toxicity, Mutagenicity, Tumorigenicity
A05069	Acute Toxicity, Skin and Eye Irritation
A05070	Acute Toxicity
A05071	Acute Toxicity, Multiple Dose Effects
A05072	Acute Toxicity, Multiple Dose Effects
A05073	Acute Toxicity, Mutagenicity
A05074	Acute Toxicity
A05075	Acute Toxicity, Mutagenicity, Tumorigenicity, Skin and Eye Irritation, Multiple Dose Effects

A05076	Mutagenicity, Tumorigenicity
A05077	Mutagenicity
A05078	Mutagenicity
A05079	Acute Toxicity, Multiple Dose Effects
A05080	Acute Toxicity, Tumorigenicity, Multiple Dose Effects
A05081	Acute Toxicity
A05082	Mutagenicity
A05083	Acute Toxicity
A05084	Acute Toxicity
A05085	Acute Toxicity
A05086	Acute Toxicity
A05087	Acute Toxicity, Mutagenicity, Tumorigenicity, Multiple Dose Effects
A05088	Acute Toxicity
A05089	Acute Toxicity
A05090	Non-toxicity
A05091	Mutagenicity
A05092	Acute Toxicity
A05093	Acute Toxicity, Multiple Dose Effects
A05094	Acute Toxicity
A05095	Acute Toxicity, Mutagenicity, Tumorigenicity
A05096	Acute Toxicity
A05097	Acute Toxicity
A05098	Acute Toxicity, Mutagenicity, Skin and Eye Irritation
A05099	Acute Toxicity
A05100	Acute Toxicity
A05101	Acute Toxicity, Skin and Eye Irritation, Multiple Dose Effects
A05102	Acute Toxicity, Mutagenicity, Skin and Eye Irritation, Multiple Dose Effects
A05103	Acute Toxicity, Mutagenicity, Tumorigenicity
A05104	Acute Toxicity
A05105	Mutagenicity
A05106	Acute Toxicity, Mutagenicity, Tumorigenicity
A05107	Acute Toxicity
A05108	Acute Toxicity, Skin and Eye Irritation
A05109	Acute Toxicity
A05110	Acute Toxicity
A05111	Acute Toxicity, Skin and Eye Irritation, Multiple Dose Effects
A05112	Acute Toxicity
A05113	Acute Toxicity, Mutagenicity, Skin and Eye Irritation
A05114	Acute Toxicity, Mutagenicity, Tumorigenicity, Skin and Eye Irritation,
A05115	Mutagenicity
A05116	Acute Toxicity, Mutagenicity
A05117	Mutagenicity, Tumorigenicity
A05118	Acute Toxicity
A05119	Tumorigenicity
A05120	Acute Toxicity, Mutagenicity
A05121	Skin and Eye Irritation

A05122	Acute Toxicity, Mutagenicity
A05123	Acute Toxicity, Mutagenicity, Multiple Dose Effects
A05124	Acute Toxicity, Mutagenicity
A05125	Acute Toxicity, Skin and Eye Irritation
A05126	Acute Toxicity, Multiple Dose Effects
A05127	Acute Toxicity, Multiple Dose Effects
A05128	Acute Toxicity, Mutagenicity, Tumorigenicity
A05129	Acute Toxicity
A05130	Acute Toxicity
A05131	Tumorigenicity
A05132	Mutagenicity
A05133	Acute Toxicity
A05134	Acute Toxicity
A05135	Mutagenicity
A05136	Acute Toxicity
A05137	Acute Toxicity
A05138	Acute Toxicity, Mutagenicity
A05139	Acute Toxicity
A05140	Acute Toxicity, Mutagenicity, Tumorigenicity, Multiple Dose Effects
A05141	Acute Toxicity
A05142	Acute Toxicity
A05143	Acute Toxicity, Multiple Dose Effects
A05144	Acute Toxicity, Reproductive Effects
A05145	Acute Toxicity, Skin and Eye Irritation, Multiple Dose Effects
A05146	Acute Toxicity
A05147	Acute Toxicity
A05148	Mutagenicity
A05149	Acute Toxicity
A05150	Acute Toxicity, Skin and Eye Irritation, Multiple Dose Effects
A05151	Mutagenicity, Tumorigenicity
A05152	Acute Toxicity
A05153	Acute Toxicity, Mutagenicity, Tumorigenicity, Skin and Eye Irritation,
A05154	Acute Toxicity
A05155	Acute Toxicity, Mutagenicity, Tumorigenicity, Reproductive Effects
A05156	Acute Toxicity
A05157	Acute Toxicity, Tumorigenicity, Skin and Eye Irritation, Multiple Dose
A05158	Acute Toxicity, Multiple Dose Effects
A05159	Acute Toxicity, Reproductive Effects
A05160	Acute Toxicity
A05161	Acute Toxicity
A05162	Acute Toxicity
A05163	Acute Toxicity
A05164	Acute Toxicity, Mutagenicity, Reproductive Effects, Multiple Dose Effects
A05165	Acute Toxicity
A05166	Acute Toxicity
A05167	Acute Toxicity, Skin and Eye Irritation

A05168	Acute Toxicity
A05169	Acute Toxicity
A05170	Acute Toxicity
A05171	Acute Toxicity
A05172	Acute Toxicity, Tumorigenicity
A05173	Acute Toxicity
A05174	Acute Toxicity, Mutagenicity, Multiple Dose Effects
A05175	Acute Toxicity
A05176	Acute Toxicity, Mutagenicity
A05177	Acute Toxicity
A05178	Acute Toxicity
A05179	Acute Toxicity
A05180	Mutagenicity
A05181	Acute Toxicity
A05182	Acute Toxicity
A05183	Acute Toxicity, Mutagenicity
A05184	Acute Toxicity, Mutagenicity, Skin and Eye Irritation
A05185	Acute Toxicity
A05186	Mutagenicity
A05187	Acute Toxicity
A05188	Acute Toxicity
A05189	Acute Toxicity, Skin and Eye Irritation
A05190	Acute Toxicity
A05191	Acute Toxicity
A05192	Mutagenicity, Tumorigenicity
A05193	Acute Toxicity
A05194	Mutagenicity
A05195	Acute Toxicity
A05196	Acute Toxicity
A05197	Acute Toxicity, Reproductive Effects
A05198	Acute Toxicity
A05199	Acute Toxicity, Mutagenicity, Reproductive Effects, Multiple Dose Effects
A05200	Reproductive Effects
A05201	Acute Toxicity, Reproductive Effects
A05202	Tumorigenicity, Multiple Dose Effects
A05203	Acute Toxicity, Mutagenicity, Reproductive Effects, Multiple Dose Effects
A05204	Reproductive Effects
A05205	Acute Toxicity
A05206	Acute Toxicity
A05207	Reproductive Effects, Multiple Dose Effects
A05208	Acute Toxicity, Mutagenicity, Multiple Dose Effects
A05209	Skin and Eye Irritation
A05210	Acute Toxicity, Mutagenicity, Skin and Eye Irritation
A05211	Acute Toxicity
A05212	Acute Toxicity, Skin and Eye Irritation
A05213	Acute Toxicity, Skin and Eye Irritation

A05214	Acute Toxicity, Skin and Eye Irritation, Multiple Dose Effects
A05215	Acute Toxicity, Mutagenicity
A05216	Acute Toxicity, Mutagenicity, Skin and Eye Irritation, Reproductive Effects, Multiple Dose Effects
A05217	Acute Toxicity, Multiple Dose Effects
A05218	Acute Toxicity, Skin and Eye Irritation, Multiple Dose Effects
A05219	Acute Toxicity, Mutagenicity
A05220	Acute Toxicity, Mutagenicity, Tumorigenicity, Multiple Dose Effects
A05221	Tumorigenicity
A05222	Acute Toxicity, Tumorigenicity, Reproductive Effects
A05223	Acute Toxicity, Multiple Dose Effects
A05224	Acute Toxicity
A05225	Mutagenicity, Reproductive Effects
A05226	Acute Toxicity
A05227	Acute Toxicity, Reproductive Effects
A05228	Acute Toxicity, Mutagenicity, Multiple Dose Effects
A05229	Acute Toxicity, Mutagenicity
A05230	Acute Toxicity, Skin and Eye Irritation
A05231	Mutagenicity, Tumorigenicity
A05232	Acute Toxicity
A05233	Skin and Eye Irritation, Reproductive Effects
A05234	Acute Toxicity, Tumorigenicity, Multiple Dose Effects
A05235	Mutagenicity
A05236	Acute Toxicity, Multiple Dose Effects
A05237	Mutagenicity
A05238	Acute Toxicity, Multiple Dose Effects
A05239	Acute Toxicity
A05240	Mutagenicity, Tumorigenicity
A05241	Acute Toxicity
A05242	Acute Toxicity
A05243	Acute Toxicity, Mutagenicity, Tumorigenicity
A05244	Acute Toxicity
A05245	Acute Toxicity, Mutagenicity, Tumorigenicity
A05246	Acute Toxicity, Skin and Eye Irritation
A05247	Acute Toxicity, Mutagenicity, Tumorigenicity
A05248	Acute Toxicity
A05249	Acute Toxicity, Mutagenicity
A05250	Acute Toxicity, Mutagenicity
A05251	Acute Toxicity
A05252	Acute Toxicity, Skin and Eye Irritation
A05253	Acute Toxicity, Skin and Eye Irritation
A05254	Acute Toxicity, Mutagenicity, Reproductive Effects
A05255	Acute Toxicity, Mutagenicity, Reproductive Effects, Multiple Dose Effects
A05256	Acute Toxicity, Mutagenicity, Skin and Eye Irritation
A05257	Acute Toxicity, Mutagenicity, Reproductive Effects
A05258	Acute Toxicity, Reproductive Effects

A05259	Mutagenicity, Tumorigenicity
A05260	Acute Toxicity, Multiple Dose Effects
A05261	Skin and Eye Irritation
A05262	Acute Toxicity, Multiple Dose Effects
A05263	Acute Toxicity, Multiple Dose Effects
A05264	Mutagenicity
A05265	Acute Toxicity
A05266	Acute Toxicity, Mutagenicity
A05267	Acute Toxicity
A05268	Mutagenicity
A05269	Acute Toxicity
A05270	Acute Toxicity
A05271	Acute Toxicity, Mutagenicity, Skin and Eye Irritation, Multiple Dose Effects
A05272	Acute Toxicity
A05273	Mutagenicity, Skin and Eye Irritation
A05274	Mutagenicity, Tumorigenicity, Skin and Eye Irritation
A05275	Acute Toxicity, Reproductive Effects, Multiple Dose Effects
A05276	Acute Toxicity, Multiple Dose Effects
A05277	Acute Toxicity, Mutagenicity, Skin and Eye Irritation, Reproductive Effects
A05278	Mutagenicity, Skin and Eye Irritation, Reproductive Effects, Multiple Dose
A05279	Acute Toxicity, Skin and Eye Irritation, Reproductive Effects, Multiple Dose
A05280	Acute Toxicity, Skin and Eye Irritation, Multiple Dose Effects
A05281	Skin and Eye Irritation
A05282	Acute Toxicity
A05283	Mutagenicity
A05284	Acute Toxicity, Skin and Eye Irritation
A05285	Acute Toxicity, Mutagenicity
A05286	Mutagenicity
A05287	Acute Toxicity
A05288	Acute Toxicity, Mutagenicity
A05289	Acute Toxicity, Mutagenicity, Tumorigenicity
A05290	Mutagenicity
A05291	Acute Toxicity
A05292	Acute Toxicity
A05293	Mutagenicity
A05294	Acute Toxicity, Mutagenicity
A05295	Acute Toxicity
A05296	Mutagenicity
A05297	Acute Toxicity
A05298	Acute Toxicity
A05299	Mutagenicity
A05300	Mutagenicity, Tumorigenicity, Reproductive Effects
A05301	Acute Toxicity
A05302	Acute Toxicity
A05303	Acute Toxicity, Multiple Dose Effects
A05304	Acute Toxicity

A05305	Acute Toxicity, Mutagenicity, Multiple Dose Effects
A05306	Acute Toxicity
A05307	Acute Toxicity, Mutagenicity, Tumorigenicity
A05308	Acute Toxicity
A05309	Acute Toxicity, Mutagenicity, Tumorigenicity, Reproductive Effects,
A05310	Acute Toxicity, Mutagenicity, Tumorigenicity, Reproductive Effects
A05311	Tumorigenicity
A05312	Acute Toxicity
A05313	Acute Toxicity
A05314	Acute Toxicity
A05315	Acute Toxicity, Tumorigenicity, Skin and Eye Irritation
A05316	Acute Toxicity
A05317	Acute Toxicity, Skin and Eye Irritation, Reproductive Effects, Multiple Dose
A05318	Acute Toxicity, Mutagenicity, Skin and Eye Irritation
A05319	Acute Toxicity, Skin and Eye Irritation, Reproductive Effects, Multiple Dose
A05320	Acute Toxicity, Skin and Eye Irritation
A05321	Acute Toxicity
A05322	Mutagenicity
A05323	Acute Toxicity
A05324	Mutagenicity, Reproductive Effects
A05325	Acute Toxicity
A05326	Acute Toxicity, Mutagenicity
A05327	Acute Toxicity
A05328	Acute Toxicity
A05329	Acute Toxicity
A05330	Acute Toxicity, Tumorigenicity, Multiple Dose Effects
A05331	Acute Toxicity, Mutagenicity, Tumorigenicity, Multiple Dose Effects
A05332	Acute Toxicity, Mutagenicity
A05333	Acute Toxicity, Multiple Dose Effects
A05334	Acute Toxicity, Skin and Eye Irritation
A05335	Acute Toxicity
A05336	Acute Toxicity
A05337	Acute Toxicity, Mutagenicity, Skin and Eye Irritation, Reproductive Effects, Multiple Dose Effects
A05338	Acute Toxicity, Reproductive Effects, Multiple Dose Effects
A05339	Acute Toxicity, Skin and Eye Irritation, Multiple Dose Effects
A05340	Mutagenicity, Tumorigenicity
A05341	Mutagenicity, Tumorigenicity
A05342	Acute Toxicity
A05343	Acute Toxicity, Multiple Dose Effects
A05344	Multiple Dose Effects
A05345	Acute Toxicity
A05346	Acute Toxicity
A05347	Acute Toxicity, Mutagenicity
A05348	Acute Toxicity, Mutagenicity, Tumorigenicity, Reproductive Effects,

A05349	Acute Toxicity, Mutagenicity, Tumorigenicity, Skin and Eye Irritation, Multiple Dose Effects
A05350	Acute Toxicity
A05351	Acute Toxicity, Skin and Eye Irritation, Multiple Dose Effects
A05352	Reproductive Effects
A05353	Acute Toxicity
A05354	Acute Toxicity
A05355	Mutagenicity, Tumorigenicity, Skin and Eye Irritation
A05356	Acute Toxicity, Mutagenicity, Tumorigenicity, Skin and Eye Irritation,
A05357	Acute Toxicity, Mutagenicity, Tumorigenicity
A05358	Acute Toxicity, Reproductive Effects, Multiple Dose Effects
A05359	Acute Toxicity
A05360	Acute Toxicity
A05361	Acute Toxicity, Mutagenicity, Multiple Dose Effects
A05362	Acute Toxicity
A05363	Acute Toxicity
A05364	Acute Toxicity, Mutagenicity, Skin and Eye Irritation, Multiple Dose Effects
A05365	Acute Toxicity
A05366	Acute Toxicity, Mutagenicity, Multiple Dose Effects
A05367	Acute Toxicity
A05368	Acute Toxicity
A05369	Acute Toxicity, Skin and Eye Irritation
A05370	Acute Toxicity
A05371	Acute Toxicity
A05372	Mutagenicity
A05373	Acute Toxicity
A05374	Acute Toxicity
A05375	Acute Toxicity
A05376	Acute Toxicity, Mutagenicity, Reproductive Effects, Multiple Dose Effects
A05377	Acute Toxicity, Mutagenicity
A05378	Mutagenicity
A05379	Mutagenicity, Tumorigenicity
A05380	Reproductive Effects
A05381	Acute Toxicity, Mutagenicity, Skin and Eye Irritation
A05382	Mutagenicity
A05383	Mutagenicity
A05384	Acute Toxicity
A05385	Acute Toxicity
A05386	Acute Toxicity
A05387	Acute Toxicity
A05388	Acute Toxicity
A05389	Acute Toxicity
A05390	Acute Toxicity, Mutagenicity, Tumorigenicity, Reproductive Effects,
A05391	Acute Toxicity
A05392	Acute Toxicity
A05393	Acute Toxicity, Mutagenicity, Tumorigenicity, Reproductive Effects,

A05394	Acute Toxicity, Mutagenicity, Reproductive Effects
A05395	Mutagenicity, Tumorigenicity
A05396	Acute Toxicity, Multiple Dose Effects
A05397	Acute Toxicity
A05398	Mutagenicity
A05399	Acute Toxicity
A05400	Non-toxicity
A05401	Acute Toxicity, Reproductive Effects
A05402	Acute Toxicity, Mutagenicity, Skin and Eye Irritation, Multiple Dose Effects
A05403	Acute Toxicity
A05404	Acute Toxicity
A05405	Acute Toxicity
A05406	Acute Toxicity, Multiple Dose Effects
A05407	Acute Toxicity, Mutagenicity, Multiple Dose Effects
A05408	Acute Toxicity
A05409	Acute Toxicity
A05410	Acute Toxicity, Skin and Eye Irritation
A05411	Acute Toxicity
A05412	Acute Toxicity, Mutagenicity
A05413	Mutagenicity, Tumorigenicity
A05414	Acute Toxicity
A05415	Skin and Eye Irritation
A05416	Acute Toxicity, Mutagenicity, Tumorigenicity, Skin and Eye Irritation,
A05417	Acute Toxicity
A05418	Acute Toxicity, Mutagenicity, Skin and Eye Irritation, Reproductive Effects, Multiple Dose Effects
A05419	Acute Toxicity
A05420	Acute Toxicity
A05421	Mutagenicity, Reproductive Effects
A05422	Mutagenicity
A05423	Acute Toxicity
A05424	Acute Toxicity, Mutagenicity, Tumorigenicity, Reproductive Effects,
A05425	Acute Toxicity, Mutagenicity, Multiple Dose Effects
A05426	Acute Toxicity
A05427	Acute Toxicity, Tumorigenicity, Skin and Eye Irritation
A05428	Mutagenicity
A05429	Acute Toxicity
A05430	Acute Toxicity, Mutagenicity, Reproductive Effects
A05431	Acute Toxicity, Mutagenicity, Multiple Dose Effects
A05432	Acute Toxicity, Mutagenicity, Reproductive Effects
A05433	Acute Toxicity, Mutagenicity
A05434	Acute Toxicity
A05435	Acute Toxicity
A05436	Acute Toxicity
A05437	Acute Toxicity, Mutagenicity, Skin and Eye Irritation
A05438	Mutagenicity, Skin and Eye Irritation

A05439	Acute Toxicity, Mutagenicity, Tumorigenicity, Skin and Eye Irritation,
A05440	Tumorigenicity
A05441	Acute Toxicity, Multiple Dose Effects
A05442	Mutagenicity
A05443	Acute Toxicity, Mutagenicity
A05444	Acute Toxicity
A05445	Acute Toxicity, Mutagenicity, Tumorigenicity, Skin and Eye Irritation
A05446	Mutagenicity
A05447	Acute Toxicity
A05448	Acute Toxicity
A05449	Acute Toxicity, Mutagenicity, Tumorigenicity, Reproductive Effects,
A05450	Acute Toxicity
A05451	Acute Toxicity, Mutagenicity, Reproductive Effects
A05452	Acute Toxicity, Mutagenicity
A05453	Acute Toxicity, Mutagenicity, Reproductive Effects
A05454	Acute Toxicity
A05455	Acute Toxicity
A05456	Mutagenicity
A05457	Acute Toxicity
A05458	Acute Toxicity
A05459	Acute Toxicity, Skin and Eye Irritation
A05460	Acute Toxicity
A05461	Acute Toxicity
A05462	Acute Toxicity, Mutagenicity, Reproductive Effects, Multiple Dose Effects
A05463	Acute Toxicity, Mutagenicity, Tumorigenicity, Multiple Dose Effects
A05464	Acute Toxicity
A05465	Acute Toxicity
A05466	Acute Toxicity
A05467	Acute Toxicity
A05468	Acute Toxicity
A05469	Acute Toxicity
A05470	Acute Toxicity
A05471	Acute Toxicity, Mutagenicity, Multiple Dose Effects
A05472	Acute Toxicity, Skin and Eye Irritation, Multiple Dose Effects
A05473	Acute Toxicity
A05474	Acute Toxicity
A05475	Acute Toxicity, Skin and Eye Irritation
A05476	Acute Toxicity
A05477	Acute Toxicity
A05478	Acute Toxicity
A05479	Acute Toxicity
A05480	Acute Toxicity
A05481	Acute Toxicity, Skin and Eye Irritation
A05482	Acute Toxicity
A05483	Acute Toxicity, Skin and Eye Irritation
A05484	Acute Toxicity

A05485	Acute Toxicity, Multiple Dose Effects
A05486	Acute Toxicity
A05487	Acute Toxicity, Mutagenicity, Multiple Dose Effects
A05488	Acute Toxicity
A05489	Acute Toxicity, Mutagenicity, Tumorigenicity, Multiple Dose Effects
A05490	Acute Toxicity, Mutagenicity, Skin and Eye Irritation
A05491	Mutagenicity
A05492	Acute Toxicity
A05493	Acute Toxicity, Multiple Dose Effects
A05494	Acute Toxicity, Mutagenicity, Reproductive Effects, Multiple Dose Effects
A05495	Acute Toxicity, Skin and Eye Irritation, Multiple Dose Effects
A05496	Mutagenicity
A05497	Acute Toxicity
A05498	Acute Toxicity, Skin and Eye Irritation
A05499	Acute Toxicity, Reproductive Effects
A05500	Acute Toxicity
A05501	Acute Toxicity, Mutagenicity, Reproductive Effects, Multiple Dose Effects
A05502	Acute Toxicity, Mutagenicity, Multiple Dose Effects
A05503	Acute Toxicity, Mutagenicity
A05504	Acute Toxicity, Mutagenicity
A05505	Acute Toxicity
A05506	Acute Toxicity, Reproductive Effects, Multiple Dose Effects
A05507	Mutagenicity, Skin and Eye Irritation
A05508	Acute Toxicity
A05509	Mutagenicity
A05510	Reproductive Effects
A05511	Acute Toxicity, Skin and Eye Irritation
A05512	Acute Toxicity
A05513	Acute Toxicity
A05514	Tumorigenicity
A05515	Acute Toxicity, Skin and Eye Irritation, Reproductive Effects, Multiple Dose
A05516	Acute Toxicity, Mutagenicity, Reproductive Effects, Multiple Dose Effects
A05517	Acute Toxicity, Mutagenicity, Skin and Eye Irritation
A05518	Mutagenicity, Tumorigenicity
A05519	Tumorigenicity
A05520	Acute Toxicity, Mutagenicity
A05521	Mutagenicity
A05522	Acute Toxicity
A05523	Mutagenicity, Tumorigenicity
A05524	Acute Toxicity
A05525	Acute Toxicity
A05526	Acute Toxicity
A05527	Acute Toxicity, Mutagenicity, Tumorigenicity, Multiple Dose Effects
A05528	Acute Toxicity
A05529	Mutagenicity
A05530	Acute Toxicity, Reproductive Effects

A05531	Skin and Eye Irritation, Reproductive Effects
A05532	Acute Toxicity, Skin and Eye Irritation
A05533	Acute Toxicity
A05534	Acute Toxicity, Skin and Eye Irritation
A05535	Acute Toxicity
A05536	Acute Toxicity, Mutagenicity
A05537	Acute Toxicity
A05538	Acute Toxicity
A05539	Acute Toxicity, Skin and Eye Irritation, Reproductive Effects, Multiple Dose
A05540	Acute Toxicity
A05541	Acute Toxicity, Mutagenicity, Reproductive Effects
A05542	Acute Toxicity, Skin and Eye Irritation
A05543	Mutagenicity
A05544	Mutagenicity
A05545	Acute Toxicity
A05546	Acute Toxicity
A05547	Acute Toxicity
A05548	Acute Toxicity, Mutagenicity, Multiple Dose Effects
A05549	Acute Toxicity, Tumorigenicity
A05550	Acute Toxicity, Mutagenicity, Tumorigenicity, Multiple Dose Effects
A05551	Acute Toxicity
A05552	Acute Toxicity
A05553	Acute Toxicity, Multiple Dose Effects
A05554	Acute Toxicity, Skin and Eye Irritation, Reproductive Effects, Multiple Dose
A05555	Acute Toxicity
A05556	Acute Toxicity, Mutagenicity, Tumorigenicity, Skin and Eye Irritation, Multiple Dose Effects
A05557	Mutagenicity
A05558	Tumorigenicity
A05559	Mutagenicity
A05560	Acute Toxicity
A05561	Acute Toxicity
A05562	Mutagenicity
A05563	Skin and Eye Irritation, Multiple Dose Effects
A05564	Acute Toxicity, Reproductive Effects, Multiple Dose Effects
A05565	Acute Toxicity, Mutagenicity
A05566	Acute Toxicity, Multiple Dose Effects
A05567	Acute Toxicity
A05568	Acute Toxicity, Mutagenicity, Skin and Eye Irritation, Reproductive Effects, Multiple Dose Effects
A05569	Acute Toxicity, Mutagenicity, Skin and Eye Irritation
A05570	Acute Toxicity
A05571	Acute Toxicity, Mutagenicity, Skin and Eye Irritation
A05572	Mutagenicity
A05573	Acute Toxicity, Mutagenicity, Reproductive Effects, Multiple Dose Effects
A05574	Mutagenicity

A05575	Acute Toxicity, Mutagenicity
A05576	Acute Toxicity
A05577	Acute Toxicity, Mutagenicity, Tumorigenicity, Skin and Eye Irritation
A05578	Mutagenicity
A05579	Acute Toxicity, Multiple Dose Effects
A05580	Acute Toxicity
A05581	Acute Toxicity
A05582	Acute Toxicity, Mutagenicity, Tumorigenicity, Skin and Eye Irritation
A05583	Acute Toxicity
A05584	Acute Toxicity, Mutagenicity, Skin and Eye Irritation
A05585	Acute Toxicity, Tumorigenicity, Reproductive Effects, Multiple Dose Effects
A05586	Acute Toxicity, Mutagenicity, Skin and Eye Irritation, Multiple Dose Effects
A05587	Mutagenicity
A05588	Acute Toxicity
A05589	Acute Toxicity, Skin and Eye Irritation
A05590	Acute Toxicity, Mutagenicity
A05591	Acute Toxicity, Mutagenicity, Tumorigenicity, Skin and Eye Irritation, Multiple Dose Effects
A05592	Acute Toxicity
A05593	Mutagenicity
A05594	Mutagenicity
A05595	Mutagenicity
A05596	Mutagenicity, Tumorigenicity
A05597	Acute Toxicity, Mutagenicity, Tumorigenicity, Skin and Eye Irritation, Multiple Dose Effects
A05598	Acute Toxicity, Mutagenicity, Skin and Eye Irritation
A05599	Acute Toxicity, Skin and Eye Irritation
A05600	Acute Toxicity, Mutagenicity
A05601	Acute Toxicity, Mutagenicity, Tumorigenicity, Multiple Dose Effects
A05602	Acute Toxicity
A05603	Acute Toxicity
A05604	Acute Toxicity
A05605	Acute Toxicity, Reproductive Effects, Multiple Dose Effects
A05606	Acute Toxicity
A05607	Acute Toxicity
A05608	Acute Toxicity, Tumorigenicity
A05609	Acute Toxicity, Mutagenicity
A05610	Acute Toxicity
A05611	Mutagenicity
A05612	Mutagenicity
A05613	Skin and Eye Irritation
A05614	Acute Toxicity
A05615	Acute Toxicity
A05616	Acute Toxicity
A05617	Acute Toxicity, Reproductive Effects
A05618	Acute Toxicity

A05619	Acute Toxicity, Mutagenicity, Multiple Dose Effects
A05620	Acute Toxicity
A05621	Acute Toxicity, Mutagenicity, Tumorigenicity, Skin and Eye Irritation, Multiple Dose Effects
A05622	Acute Toxicity
A05623	Acute Toxicity
A05624	Acute Toxicity, Mutagenicity
A05625	Mutagenicity
A05626	Acute Toxicity
A05627	Acute Toxicity, Multiple Dose Effects
A05628	Acute Toxicity
A05629	Acute Toxicity
A05630	Mutagenicity, Skin and Eye Irritation
A05631	Non-toxicity
A05632	Mutagenicity
A05633	Acute Toxicity
A05634	Acute Toxicity, Multiple Dose Effects
A05635	Acute Toxicity
A05636	Mutagenicity
A05637	Acute Toxicity
A05638	Skin and Eye Irritation, Multiple Dose Effects
A05639	Acute Toxicity
A05640	Acute Toxicity
A05641	Mutagenicity, Tumorigenicity
A05642	Mutagenicity, Tumorigenicity
A05643	Mutagenicity, Tumorigenicity
A05644	Mutagenicity, Tumorigenicity
A05645	Acute Toxicity
A05646	Non-toxicity
A05647	Acute Toxicity, Mutagenicity
A05648	Acute Toxicity, Mutagenicity
A05649	Acute Toxicity, Multiple Dose Effects
A05650	Acute Toxicity
A05651	Mutagenicity
A05652	Tumorigenicity
A05653	Acute Toxicity, Mutagenicity, Tumorigenicity, Skin and Eye Irritation, Multiple Dose Effects
A05654	Acute Toxicity
A05655	Acute Toxicity, Mutagenicity, Tumorigenicity
A05656	Acute Toxicity
A05657	Mutagenicity, Tumorigenicity
A05658	Acute Toxicity
A05659	Acute Toxicity, Mutagenicity, Tumorigenicity, Skin and Eye Irritation,
A05660	Acute Toxicity, Mutagenicity
A05661	Acute Toxicity
A05662	Acute Toxicity

A05663	Acute Toxicity
A05664	Acute Toxicity
A05665	Acute Toxicity, Mutagenicity, Tumorigenicity, Skin and Eye Irritation, Multiple Dose Effects
A05666	Acute Toxicity, Mutagenicity, Tumorigenicity, Skin and Eye Irritation
A05667	Acute Toxicity, Mutagenicity, Skin and Eye Irritation, Reproductive Effects
A05668	Acute Toxicity, Skin and Eye Irritation, Reproductive Effects
A05669	Acute Toxicity, Tumorigenicity, Multiple Dose Effects
A05670	Acute Toxicity, Mutagenicity, Multiple Dose Effects
A05671	Acute Toxicity
A05672	Acute Toxicity
A05673	Acute Toxicity, Mutagenicity, Tumorigenicity, Multiple Dose Effects
A05674	Non-toxicity
A05675	Mutagenicity
A05676	Acute Toxicity
A05677	Acute Toxicity, Mutagenicity
A05678	Multiple Dose Effects
A05679	Acute Toxicity, Mutagenicity, Skin and Eye Irritation, Multiple Dose Effects
A05680	Mutagenicity, Tumorigenicity
A05681	Acute Toxicity, Mutagenicity, Tumorigenicity
A05682	Acute Toxicity, Mutagenicity, Multiple Dose Effects
A05683	Acute Toxicity, Mutagenicity
A05684	Acute Toxicity, Multiple Dose Effects
A05685	Acute Toxicity, Skin and Eye Irritation, Multiple Dose Effects
A05686	Mutagenicity
A05687	Acute Toxicity
A05688	Acute Toxicity, Mutagenicity
A05689	Acute Toxicity, Skin and Eye Irritation
A05690	Acute Toxicity, Multiple Dose Effects
A05691	Acute Toxicity, Mutagenicity, Skin and Eye Irritation, Reproductive Effects, Multiple Dose Effects
A05692	Acute Toxicity
A05693	Skin and Eye Irritation
A05694	Acute Toxicity, Mutagenicity
A05695	Acute Toxicity, Mutagenicity
A05696	Mutagenicity
A05697	Mutagenicity
A05698	Acute Toxicity, Mutagenicity, Skin and Eye Irritation
A05699	Acute Toxicity
A05700	Acute Toxicity
A05701	Acute Toxicity, Mutagenicity, Tumorigenicity
A05702	Acute Toxicity
A05703	Acute Toxicity, Mutagenicity
A05704	Acute Toxicity, Mutagenicity, Tumorigenicity, Skin and Eye Irritation, Multiple Dose Effects
A05705	Acute Toxicity, Mutagenicity, Skin and Eye Irritation, Multiple Dose Effects

A05706	Acute Toxicity, Mutagenicity
A05707	Acute Toxicity, Mutagenicity, Multiple Dose Effects
A05708	Acute Toxicity, Multiple Dose Effects
A05709	Acute Toxicity
A05710	Mutagenicity
A05711	Mutagenicity
A05712	Acute Toxicity
A05713	Acute Toxicity
A05714	Acute Toxicity
A05715	Acute Toxicity
A05716	Mutagenicity
A05717	Mutagenicity, Tumorigenicity
A05718	Mutagenicity, Tumorigenicity
A05719	Acute Toxicity, Mutagenicity
A05720	Mutagenicity, Tumorigenicity
A05721	Mutagenicity, Tumorigenicity
A05722	Mutagenicity, Tumorigenicity
A05723	Mutagenicity
A05724	Acute Toxicity
A05725	Mutagenicity
A05726	Mutagenicity
A05727	Acute Toxicity, Mutagenicity, Multiple Dose Effects
A05728	Acute Toxicity, Mutagenicity, Multiple Dose Effects
A05729	Acute Toxicity
A05730	Acute Toxicity, Mutagenicity, Reproductive Effects, Multiple Dose Effects
A05731	Mutagenicity
A05732	Acute Toxicity
A05733	Acute Toxicity
A05734	Acute Toxicity
A05735	Acute Toxicity
A05736	Acute Toxicity, Mutagenicity, Multiple Dose Effects
A05737	Mutagenicity
A05738	Acute Toxicity, Mutagenicity, Multiple Dose Effects
A05739	Mutagenicity, Tumorigenicity
A05740	Acute Toxicity, Mutagenicity
A05741	Acute Toxicity, Skin and Eye Irritation
A05742	Acute Toxicity
A05743	Acute Toxicity
A05744	Mutagenicity, Tumorigenicity
A05745	Acute Toxicity
A05746	Mutagenicity
A05747	Acute Toxicity, Multiple Dose Effects
A05748	Acute Toxicity
A05749	Skin and Eye Irritation
A05750	Acute Toxicity
A05751	Acute Toxicity, Mutagenicity, Multiple Dose Effects

A05752	Acute Toxicity, Mutagenicity
A05753	Acute Toxicity
A05754	Acute Toxicity
A05755	Acute Toxicity
A05756	Acute Toxicity, Skin and Eye Irritation, Reproductive Effects
A05757	Acute Toxicity, Mutagenicity, Multiple Dose Effects
A05758	Acute Toxicity, Mutagenicity
A05759	Acute Toxicity, Mutagenicity, Multiple Dose Effects
A05760	Acute Toxicity, Skin and Eye Irritation
A05761	Acute Toxicity
A05762	Acute Toxicity
A05763	Acute Toxicity
A05764	Acute Toxicity
A05765	Acute Toxicity, Mutagenicity
A05766	Acute Toxicity
A05767	Acute Toxicity
A05768	Acute Toxicity, Mutagenicity
A05769	Acute Toxicity, Mutagenicity, Skin and Eye Irritation
A05770	Acute Toxicity
A05771	Acute Toxicity
A05772	Acute Toxicity, Multiple Dose Effects
A05773	Acute Toxicity, Skin and Eye Irritation
A05774	Acute Toxicity
A05775	Acute Toxicity
A05776	Mutagenicity
A05777	Acute Toxicity, Mutagenicity, Tumorigenicity, Multiple Dose Effects
A05778	Acute Toxicity
A05779	Acute Toxicity, Mutagenicity, Multiple Dose Effects
A05780	Mutagenicity
A05781	Acute Toxicity
A05782	Acute Toxicity, Mutagenicity
A05783	Acute Toxicity
A05784	Acute Toxicity
A05785	Acute Toxicity, Multiple Dose Effects
A05786	Mutagenicity
A05787	Acute Toxicity
A05788	Acute Toxicity
A05789	Acute Toxicity, Mutagenicity
A05790	Acute Toxicity
A05791	Acute Toxicity, Skin and Eye Irritation, Multiple Dose Effects
A05792	Acute Toxicity
A05793	Acute Toxicity, Mutagenicity
A05794	Acute Toxicity, Skin and Eye Irritation, Reproductive Effects, Multiple Dose
A05795	Acute Toxicity, Skin and Eye Irritation, Multiple Dose Effects
A05796	Acute Toxicity, Mutagenicity, Tumorigenicity, Skin and Eye Irritation,
A05797	Acute Toxicity, Reproductive Effects, Multiple Dose Effects

A05798	Acute Toxicity
A05799	Acute Toxicity, Skin and Eye Irritation
A05800	Acute Toxicity, Reproductive Effects
A05801	Acute Toxicity, Mutagenicity
A05802	Acute Toxicity, Skin and Eye Irritation
A05803	Acute Toxicity, Skin and Eye Irritation, Multiple Dose Effects
A05804	Acute Toxicity, Skin and Eye Irritation
A05805	Acute Toxicity
A05806	Acute Toxicity
A05807	Acute Toxicity
A05808	Acute Toxicity, Skin and Eye Irritation, Multiple Dose Effects
A05809	Acute Toxicity
A05810	Acute Toxicity, Mutagenicity, Tumorigenicity, Reproductive Effects
A05811	Acute Toxicity, Skin and Eye Irritation, Multiple Dose Effects
A05812	Acute Toxicity, Mutagenicity, Reproductive Effects
A05813	Mutagenicity, Tumorigenicity
A05814	Acute Toxicity, Mutagenicity, Tumorigenicity, Multiple Dose Effects
A05815	Acute Toxicity, Skin and Eye Irritation
A05816	Acute Toxicity, Multiple Dose Effects
A05817	Acute Toxicity, Skin and Eye Irritation, Reproductive Effects, Multiple Dose
A05818	Acute Toxicity
A05819	Acute Toxicity
A05820	Acute Toxicity, Mutagenicity, Skin and Eye Irritation
A05821	Acute Toxicity
A05822	Acute Toxicity, Mutagenicity
A05823	Acute Toxicity
A05824	Acute Toxicity
A05825	Acute Toxicity, Mutagenicity, Skin and Eye Irritation, Multiple Dose Effects
A05826	Acute Toxicity, Mutagenicity
A05827	Mutagenicity, Tumorigenicity, Multiple Dose Effects
A05828	Mutagenicity, Skin and Eye Irritation, Multiple Dose Effects
A05829	Mutagenicity, Tumorigenicity
A05830	Acute Toxicity
A05831	Acute Toxicity
A05832	Acute Toxicity, Mutagenicity, Skin and Eye Irritation, Multiple Dose Effects
A05833	Acute Toxicity
A05834	Acute Toxicity, Mutagenicity, Skin and Eye Irritation
A05835	Acute Toxicity, Mutagenicity
A05836	Acute Toxicity
A05837	Acute Toxicity
A05838	Mutagenicity, Tumorigenicity
A05839	Acute Toxicity
A05840	Acute Toxicity
A05841	Acute Toxicity
A05842	Acute Toxicity
A05843	Acute Toxicity

A05844	Acute Toxicity
A05845	Acute Toxicity
A05846	Acute Toxicity
A05847	Acute Toxicity, Multiple Dose Effects
A05848	Acute Toxicity
A05849	Acute Toxicity, Multiple Dose Effects
A05850	Acute Toxicity
A05851	Acute Toxicity, Multiple Dose Effects
A05852	Acute Toxicity
A05853	Mutagenicity
A05854	Acute Toxicity, Tumorigenicity
A05855	Acute Toxicity
A05856	Acute Toxicity
A05857	Acute Toxicity
A05858	Acute Toxicity
A05859	Acute Toxicity, Tumorigenicity
A05860	Mutagenicity
A05861	Mutagenicity, Tumorigenicity
A05862	Reproductive Effects
A05863	Acute Toxicity
A05864	Acute Toxicity
A05865	Acute Toxicity, Tumorigenicity
A05866	Acute Toxicity, Mutagenicity, Reproductive Effects, Multiple Dose Effects
A05867	Acute Toxicity, Reproductive Effects
A05868	Acute Toxicity
A05869	Mutagenicity
A05870	Acute Toxicity, Mutagenicity, Skin and Eye Irritation, Reproductive Effects, Multiple Dose Effects
A05871	Acute Toxicity, Mutagenicity, Skin and Eye Irritation
A05872	Mutagenicity
A05873	Mutagenicity
A05874	Mutagenicity
A05875	Acute Toxicity
A05876	Acute Toxicity
A05877	Acute Toxicity
A05878	Acute Toxicity
A05879	Mutagenicity
A05880	Acute Toxicity, Mutagenicity, Reproductive Effects, Multiple Dose Effects
A05881	Acute Toxicity, Mutagenicity, Skin and Eye Irritation
A05882	Reproductive Effects
A05883	Acute Toxicity, Mutagenicity, Skin and Eye Irritation, Multiple Dose Effects
A05884	Acute Toxicity
A05885	Mutagenicity, Tumorigenicity
A05886	Mutagenicity, Tumorigenicity
A05887	Mutagenicity, Tumorigenicity
A05888	Acute Toxicity

A05889	Acute Toxicity, Mutagenicity, Tumorigenicity, Skin and Eye Irritation
A05890	Mutagenicity
A05891	Acute Toxicity, Mutagenicity, Reproductive Effects
A05892	Mutagenicity, Reproductive Effects
A05893	Acute Toxicity, Reproductive Effects
A05894	Acute Toxicity, Mutagenicity, Reproductive Effects, Multiple Dose Effects
A05895	Acute Toxicity
A05896	Tumorigenicity
A05897	Mutagenicity
A05898	Mutagenicity
A05899	Acute Toxicity, Mutagenicity
A05900	Mutagenicity
A05901	Acute Toxicity
A05902	Acute Toxicity, Mutagenicity
A05903	Acute Toxicity
A05904	Acute Toxicity
A05905	Acute Toxicity
A05906	Acute Toxicity, Skin and Eye Irritation, Reproductive Effects
A05907	Acute Toxicity, Multiple Dose Effects
A05908	Acute Toxicity
A05909	Acute Toxicity
A05910	Acute Toxicity, Reproductive Effects
A05911	Acute Toxicity, Mutagenicity
A05912	Acute Toxicity
A05913	Acute Toxicity
A05914	Acute Toxicity
A05915	Acute Toxicity
A05916	Acute Toxicity
A05917	Acute Toxicity
A05918	Acute Toxicity, Mutagenicity
A05919	Acute Toxicity, Mutagenicity
A05920	Multiple Dose Effects
A05921	Mutagenicity
A05922	Acute Toxicity, Mutagenicity
A05923	Acute Toxicity, Tumorigenicity
A05924	Skin and Eye Irritation
A05925	Mutagenicity
A05926	Acute Toxicity, Mutagenicity, Reproductive Effects, Multiple Dose Effects
A05927	Acute Toxicity
A05928	Acute Toxicity, Mutagenicity
A05929	Tumorigenicity
A05930	Acute Toxicity
A05931	Mutagenicity
A05932	Mutagenicity
A05933	Acute Toxicity
A05934	Acute Toxicity

A05935	Mutagenicity
A05936	Acute Toxicity, Skin and Eye Irritation
A05937	Acute Toxicity, Mutagenicity
A05938	Acute Toxicity
A05939	Acute Toxicity
A05940	Acute Toxicity, Mutagenicity, Tumorigenicity, Reproductive Effects,
A05941	Acute Toxicity
A05942	Acute Toxicity
A05943	Mutagenicity
A05944	Acute Toxicity, Mutagenicity, Skin and Eye Irritation, Multiple Dose Effects
A05945	Acute Toxicity, Mutagenicity, Tumorigenicity, Skin and Eye Irritation,
A05946	Acute Toxicity
A05947	Acute Toxicity
A05948	Acute Toxicity
A05949	Acute Toxicity, Mutagenicity, Skin and Eye Irritation, Reproductive Effects
A05950	Acute Toxicity
A05951	Acute Toxicity, Mutagenicity, Tumorigenicity, Skin and Eye Irritation,
A05952	Mutagenicity, Tumorigenicity
A05953	Multiple Dose Effects
A05954	Mutagenicity
A05955	Acute Toxicity
A05956	Acute Toxicity, Mutagenicity, Skin and Eye Irritation, Multiple Dose Effects
A05957	Acute Toxicity, Mutagenicity
A05958	Acute Toxicity, Multiple Dose Effects
A05959	Acute Toxicity
A05960	Acute Toxicity
A05961	Acute Toxicity
A05962	Acute Toxicity, Mutagenicity, Reproductive Effects, Multiple Dose Effects
A05963	Acute Toxicity
A05964	Acute Toxicity
A05965	Acute Toxicity
A05966	Acute Toxicity, Reproductive Effects, Multiple Dose Effects
A05967	Acute Toxicity, Mutagenicity
A05968	Mutagenicity, Tumorigenicity
A05969	Mutagenicity, Tumorigenicity
A05970	Tumorigenicity
A05971	Mutagenicity
A05972	Acute Toxicity, Mutagenicity
A05973	Acute Toxicity
A05974	Acute Toxicity, Mutagenicity
A05975	Mutagenicity, Tumorigenicity
A05976	Mutagenicity, Tumorigenicity
A05977	Mutagenicity, Tumorigenicity
A05978	Mutagenicity, Tumorigenicity
A05979	Acute Toxicity, Mutagenicity
A05980	Acute Toxicity

A05981	Reproductive Effects
A05982	Acute Toxicity
A05983	Acute Toxicity, Mutagenicity
A05984	Acute Toxicity
A05985	Acute Toxicity, Mutagenicity
A05986	Acute Toxicity, Mutagenicity, Multiple Dose Effects
A05987	Acute Toxicity, Mutagenicity, Tumorigenicity
A05988	Acute Toxicity, Multiple Dose Effects
A05989	Acute Toxicity
A05990	Acute Toxicity, Mutagenicity, Reproductive Effects
A05991	Acute Toxicity
A05992	Acute Toxicity, Mutagenicity
A05993	Acute Toxicity
A05994	Acute Toxicity
A05995	Acute Toxicity
A05996	Acute Toxicity, Tumorigenicity
A05997	Mutagenicity
A05998	Acute Toxicity, Mutagenicity
A05999	Acute Toxicity
A06000	Acute Toxicity, Multiple Dose Effects
A06001	Acute Toxicity, Multiple Dose Effects
A06002	Mutagenicity
A06003	Multiple Dose Effects
A06004	Acute Toxicity, Mutagenicity, Tumorigenicity, Skin and Eye Irritation,
A06005	Acute Toxicity
A06006	Mutagenicity
A06007	Acute Toxicity, Reproductive Effects
A06008	Mutagenicity
A06009	Acute Toxicity, Mutagenicity
A06010	Acute Toxicity
A06011	Acute Toxicity, Multiple Dose Effects
A06012	Acute Toxicity
A06013	Acute Toxicity
A06014	Acute Toxicity
A06015	Acute Toxicity, Multiple Dose Effects
A06016	Acute Toxicity, Mutagenicity
A06017	Acute Toxicity
A06018	Acute Toxicity
A06019	Acute Toxicity
A06020	Acute Toxicity
A06021	Acute Toxicity, Mutagenicity, Reproductive Effects, Multiple Dose Effects
A06022	Acute Toxicity, Mutagenicity
A06023	Acute Toxicity
A06024	Acute Toxicity
A06025	Acute Toxicity
A06026	Acute Toxicity

A06027	Acute Toxicity, Multiple Dose Effects
A06028	Acute Toxicity
A06029	Acute Toxicity, Reproductive Effects
A06030	Acute Toxicity
A06031	Acute Toxicity, Mutagenicity, Skin and Eye Irritation
A06032	Acute Toxicity
A06033	Acute Toxicity, Mutagenicity
A06034	Non-toxicity
A06035	Mutagenicity
A06036	Mutagenicity, Reproductive Effects
A06037	Acute Toxicity, Mutagenicity, Skin and Eye Irritation, Multiple Dose Effects
A06038	Acute Toxicity, Mutagenicity
A06039	Mutagenicity, Tumorigenicity, Multiple Dose Effects
A06040	Acute Toxicity, Multiple Dose Effects
A06041	Acute Toxicity
A06042	Acute Toxicity
A06043	Skin and Eye Irritation
A06044	Mutagenicity
A06045	Acute Toxicity
A06046	Acute Toxicity, Skin and Eye Irritation, Multiple Dose Effects
A06047	Acute Toxicity
A06048	Acute Toxicity
A06049	Acute Toxicity
A06050	Acute Toxicity, Skin and Eye Irritation
A06051	Acute Toxicity
A06052	Acute Toxicity, Mutagenicity, Skin and Eye Irritation, Reproductive Effects, Multiple Dose Effects
A06053	Mutagenicity, Tumorigenicity, Reproductive Effects, Multiple Dose Effects
A06054	Acute Toxicity, Mutagenicity, Tumorigenicity
A06055	Non-toxicity
A06056	Acute Toxicity
A06057	Mutagenicity, Tumorigenicity
A06058	Acute Toxicity, Mutagenicity, Skin and Eye Irritation
A06059	Acute Toxicity, Reproductive Effects, Multiple Dose Effects
A06060	Acute Toxicity, Mutagenicity
A06061	Acute Toxicity, Mutagenicity, Tumorigenicity, Reproductive Effects,
A06062	Acute Toxicity
A06063	Acute Toxicity, Mutagenicity, Multiple Dose Effects
A06064	Acute Toxicity, Skin and Eye Irritation
A06065	Acute Toxicity
A06066	Acute Toxicity
A06067	Acute Toxicity, Multiple Dose Effects
A06068	Mutagenicity, Tumorigenicity
A06069	Mutagenicity, Tumorigenicity
A06070	Tumorigenicity
A06071	Mutagenicity, Tumorigenicity

A06072	Acute Toxicity
A06073	Acute Toxicity
A06074	Acute Toxicity, Mutagenicity, Skin and Eye Irritation, Reproductive Effects, Multiple Dose Effects
A06075	Acute Toxicity
A06076	Acute Toxicity
A06077	Acute Toxicity
A06078	Acute Toxicity
A06079	Mutagenicity
A06080	Acute Toxicity
A06081	Acute Toxicity
A06082	Acute Toxicity
A06083	Acute Toxicity
A06084	Acute Toxicity
A06085	Acute Toxicity, Multiple Dose Effects
A06086	Acute Toxicity
A06087	Acute Toxicity, Skin and Eye Irritation
A06088	Acute Toxicity
A06089	Acute Toxicity
A06090	Acute Toxicity, Mutagenicity, Reproductive Effects
A06091	Acute Toxicity, Mutagenicity
A06092	Acute Toxicity, Mutagenicity, Tumorigenicity, Multiple Dose Effects
A06093	Reproductive Effects
A06094	Acute Toxicity
A06095	Acute Toxicity, Mutagenicity
A06096	Tumorigenicity
A06097	Acute Toxicity
A06098	Acute Toxicity, Mutagenicity
A06099	Acute Toxicity
A06100	Acute Toxicity, Mutagenicity, Tumorigenicity, Multiple Dose Effects
A06101	Acute Toxicity
A06102	Acute Toxicity
A06103	Acute Toxicity, Reproductive Effects
A06104	Acute Toxicity
A06105	Acute Toxicity
A06106	Acute Toxicity, Mutagenicity, Tumorigenicity
A06107	Acute Toxicity, Skin and Eye Irritation
A06108	Acute Toxicity
A06109	Acute Toxicity
A06110	Acute Toxicity, Mutagenicity
A06111	Acute Toxicity, Mutagenicity
A06112	Acute Toxicity
A06113	Acute Toxicity, Skin and Eye Irritation
A06114	Acute Toxicity
A06115	Acute Toxicity
A06116	Acute Toxicity

A06117	Acute Toxicity
A06118	Acute Toxicity
A06119	Acute Toxicity
A06120	Acute Toxicity
A06121	Acute Toxicity
A06122	Multiple Dose Effects
A06123	Tumorigenicity
A06124	Acute Toxicity, Multiple Dose Effects
A06125	Acute Toxicity
A06126	Acute Toxicity
A06127	Acute Toxicity
A06128	Mutagenicity
A06129	Acute Toxicity, Mutagenicity, Reproductive Effects, Multiple Dose Effects
A06130	Acute Toxicity, Tumorigenicity
A06131	Mutagenicity
A06132	Acute Toxicity, Mutagenicity, Skin and Eye Irritation, Multiple Dose Effects
A06133	Acute Toxicity
A06134	Acute Toxicity
A06135	Acute Toxicity
A06136	Acute Toxicity
A06137	Acute Toxicity, Skin and Eye Irritation
A06138	Acute Toxicity, Mutagenicity
A06139	Acute Toxicity, Mutagenicity, Skin and Eye Irritation
A06140	Acute Toxicity, Multiple Dose Effects
A06141	Mutagenicity, Tumorigenicity
A06142	Acute Toxicity
A06143	Acute Toxicity, Mutagenicity, Multiple Dose Effects
A06144	Multiple Dose Effects
A06145	Acute Toxicity, Mutagenicity, Tumorigenicity
A06146	Acute Toxicity
A06147	Acute Toxicity
A06148	Mutagenicity, Tumorigenicity
A06149	Acute Toxicity, Multiple Dose Effects
A06150	Acute Toxicity
A06151	Acute Toxicity, Mutagenicity, Tumorigenicity
A06152	Mutagenicity
A06153	Mutagenicity
A06154	Acute Toxicity
A06155	Acute Toxicity
A06156	Acute Toxicity
A06157	Mutagenicity
A06158	Acute Toxicity, Reproductive Effects
A06159	Acute Toxicity, Multiple Dose Effects
A06160	Acute Toxicity
A06161	Acute Toxicity
A06162	Mutagenicity, Tumorigenicity

A06163	Acute Toxicity
A06164	Acute Toxicity
A06165	Acute Toxicity, Multiple Dose Effects
A06166	Acute Toxicity
A06167	Acute Toxicity, Tumorigenicity, Multiple Dose Effects
A06168	Reproductive Effects
A06169	Non-toxicity
A06170	Mutagenicity
A06171	Acute Toxicity
A06172	Mutagenicity, Reproductive Effects
A06173	Acute Toxicity
A06174	Mutagenicity
A06175	Acute Toxicity
A06176	Acute Toxicity, Mutagenicity, Reproductive Effects, Multiple Dose Effects
A06177	Acute Toxicity, Mutagenicity
A06178	Acute Toxicity
A06179	Acute Toxicity
A06180	Acute Toxicity
A06181	Acute Toxicity
A06182	Acute Toxicity
A06183	Acute Toxicity, Mutagenicity
A06184	Acute Toxicity
A06185	Mutagenicity
A06186	Acute Toxicity
A06187	Mutagenicity
A06188	Mutagenicity
A06189	Acute Toxicity
A06190	Acute Toxicity, Mutagenicity, Tumorigenicity, Multiple Dose Effects
A06191	Multiple Dose Effects
A06192	Acute Toxicity
A06193	Tumorigenicity
A06194	Acute Toxicity
A06195	Acute Toxicity, Mutagenicity, Skin and Eye Irritation, Reproductive Effects, Multiple Dose Effects
A06196	Mutagenicity
A06197	Acute Toxicity
A06198	Acute Toxicity
A06199	Acute Toxicity, Skin and Eye Irritation
A06200	Acute Toxicity, Mutagenicity
A06201	Acute Toxicity
A06202	Acute Toxicity
A06203	Acute Toxicity, Mutagenicity
A06204	Skin and Eye Irritation
A06205	Acute Toxicity, Multiple Dose Effects
A06206	Acute Toxicity
A06207	Acute Toxicity, Mutagenicity

A06208	Acute Toxicity
A06209	Mutagenicity
A06210	Acute Toxicity
A06211	Acute Toxicity, Mutagenicity
A06212	Mutagenicity, Tumorigenicity
A06213	Mutagenicity
A06214	Acute Toxicity
A06215	Acute Toxicity
A06216	Acute Toxicity
A06217	Tumorigenicity
A06218	Acute Toxicity
A06219	Tumorigenicity
A06220	Acute Toxicity, Tumorigenicity
A06221	Acute Toxicity
A06222	Acute Toxicity, Mutagenicity, Tumorigenicity
A06223	Acute Toxicity, Mutagenicity
A06224	Reproductive Effects, Multiple Dose Effects
A06225	Mutagenicity
A06226	Acute Toxicity, Mutagenicity, Tumorigenicity
A06227	Acute Toxicity, Mutagenicity, Tumorigenicity
A06228	Acute Toxicity
A06229	Acute Toxicity, Mutagenicity, Skin and Eye Irritation, Multiple Dose Effects
A06230	Acute Toxicity, Reproductive Effects
A06231	Acute Toxicity
A06232	Acute Toxicity
A06233	Acute Toxicity
A06234	Acute Toxicity
A06235	Acute Toxicity, Skin and Eye Irritation
A06236	Acute Toxicity
A06237	Acute Toxicity
A06238	Acute Toxicity, Multiple Dose Effects
A06239	Acute Toxicity
A06240	Acute Toxicity, Reproductive Effects, Multiple Dose Effects
A06241	Acute Toxicity
A06242	Acute Toxicity, Mutagenicity, Skin and Eye Irritation
A06243	Reproductive Effects
A06244	Acute Toxicity, Mutagenicity
A06245	Acute Toxicity
A06246	Acute Toxicity
A06247	Acute Toxicity
A06248	Acute Toxicity
A06249	Acute Toxicity, Skin and Eye Irritation
A06250	Skin and Eye Irritation
A06251	Acute Toxicity
A06252	Acute Toxicity
A06253	Acute Toxicity, Mutagenicity

A06254	Acute Toxicity
A06255	Mutagenicity
A06256	Non-toxicity
A06257	Acute Toxicity
A06258	Acute Toxicity
A06259	Acute Toxicity
A06260	Tumorigenicity
A06261	Acute Toxicity
A06262	Mutagenicity, Skin and Eye Irritation
A06263	Acute Toxicity, Mutagenicity, Multiple Dose Effects
A06264	Mutagenicity
A06265	Acute Toxicity, Skin and Eye Irritation
A06266	Reproductive Effects
A06267	Mutagenicity
A06268	Acute Toxicity
A06269	Acute Toxicity, Mutagenicity, Multiple Dose Effects
A06270	Acute Toxicity
A06271	Acute Toxicity, Multiple Dose Effects
A06272	Acute Toxicity, Mutagenicity, Reproductive Effects, Multiple Dose Effects
A06273	Multiple Dose Effects
A06274	Acute Toxicity
A06275	Mutagenicity
A06276	Reproductive Effects
A06277	Acute Toxicity, Mutagenicity, Reproductive Effects, Multiple Dose Effects
A06278	Acute Toxicity, Mutagenicity, Multiple Dose Effects
A06279	Acute Toxicity
A06280	Acute Toxicity
A06281	Acute Toxicity, Skin and Eye Irritation, Multiple Dose Effects
A06282	Acute Toxicity
A06283	Acute Toxicity, Mutagenicity, Multiple Dose Effects
A06284	Acute Toxicity, Mutagenicity, Tumorigenicity
A06285	Acute Toxicity, Multiple Dose Effects
A06286	Acute Toxicity, Skin and Eye Irritation
A06287	Non-toxicity
A06288	Mutagenicity
A06289	Mutagenicity
A06290	Reproductive Effects
A06291	Acute Toxicity
A06292	Acute Toxicity, Skin and Eye Irritation
A06293	Mutagenicity, Tumorigenicity
A06294	Acute Toxicity
A06295	Acute Toxicity, Mutagenicity, Tumorigenicity, Reproductive Effects,
A06296	Acute Toxicity
A06297	Skin and Eye Irritation, Multiple Dose Effects
A06298	Acute Toxicity, Mutagenicity
A06299	Acute Toxicity

A06300	Mutagenicity
A06301	Acute Toxicity
A06302	Acute Toxicity
A06303	Acute Toxicity
A06304	Acute Toxicity
A06305	Acute Toxicity
A06306	Acute Toxicity
A06307	Mutagenicity
A06308	Acute Toxicity
A06309	Acute Toxicity
A06310	Acute Toxicity, Mutagenicity
A06311	Acute Toxicity
A06312	Skin and Eye Irritation
A06313	Acute Toxicity, Mutagenicity
A06314	Acute Toxicity, Mutagenicity
A06315	Acute Toxicity, Skin and Eye Irritation
A06316	Acute Toxicity
A06317	Acute Toxicity
A06318	Acute Toxicity, Mutagenicity
A06319	Acute Toxicity, Mutagenicity, Multiple Dose Effects
A06320	Acute Toxicity
A06321	Mutagenicity, Tumorigenicity
A06322	Mutagenicity
A06323	Acute Toxicity
A06324	Acute Toxicity
A06325	Acute Toxicity, Mutagenicity, Tumorigenicity, Reproductive Effects
A06326	Acute Toxicity
A06327	Acute Toxicity
A06328	Mutagenicity
A06329	Mutagenicity, Tumorigenicity
A06330	Acute Toxicity
A06331	Acute Toxicity
A06332	Acute Toxicity
A06333	Acute Toxicity, Skin and Eye Irritation
A06334	Acute Toxicity
A06335	Acute Toxicity
A06336	Acute Toxicity, Mutagenicity, Tumorigenicity
A06337	Acute Toxicity
A06338	Acute Toxicity, Mutagenicity, Skin and Eye Irritation
A06339	Acute Toxicity, Mutagenicity, Reproductive Effects
A06340	Acute Toxicity
A06341	Acute Toxicity
A06342	Acute Toxicity
A06343	Acute Toxicity
A06344	Acute Toxicity
A06345	Acute Toxicity, Mutagenicity

A06346	Mutagenicity, Tumorigenicity, Multiple Dose Effects
A06347	Acute Toxicity, Skin and Eye Irritation, Reproductive Effects, Multiple Dose
A06348	Acute Toxicity, Mutagenicity, Skin and Eye Irritation, Reproductive Effects, Multiple Dose Effects
A06349	Acute Toxicity
A06350	Acute Toxicity
A06351	Acute Toxicity
A06352	Acute Toxicity
A06353	Acute Toxicity
A06354	Acute Toxicity
A06355	Acute Toxicity
A06356	Acute Toxicity
A06357	Acute Toxicity
A06358	Acute Toxicity, Mutagenicity, Skin and Eye Irritation, Multiple Dose Effects
A06359	Acute Toxicity, Mutagenicity, Multiple Dose Effects
A06360	Acute Toxicity
A06361	Skin and Eye Irritation, Reproductive Effects, Multiple Dose Effects
A06362	Acute Toxicity
A06363	Acute Toxicity, Skin and Eye Irritation
A06364	Acute Toxicity, Mutagenicity, Tumorigenicity
A06365	Acute Toxicity
A06366	Acute Toxicity
A06367	Acute Toxicity
A06368	Mutagenicity, Tumorigenicity, Multiple Dose Effects
A06369	Acute Toxicity, Skin and Eye Irritation
A06370	Acute Toxicity
A06371	Acute Toxicity
A06372	Acute Toxicity
A06373	Acute Toxicity
A06374	Acute Toxicity
A06375	Acute Toxicity
A06376	Acute Toxicity
A06377	Acute Toxicity
A06378	Acute Toxicity
A06379	Acute Toxicity
A06380	Acute Toxicity
A06381	Acute Toxicity
A06382	Acute Toxicity
A06383	Acute Toxicity
A06384	Acute Toxicity, Mutagenicity, Tumorigenicity
A06385	Acute Toxicity, Mutagenicity, Tumorigenicity, Multiple Dose Effects
A06386	Acute Toxicity
A06387	Mutagenicity
A06388	Acute Toxicity, Tumorigenicity, Reproductive Effects
A06389	Mutagenicity
A06390	Acute Toxicity

A06391	Acute Toxicity
A06392	Acute Toxicity
A06393	Acute Toxicity
A06394	Acute Toxicity
A06395	Acute Toxicity
A06396	Tumorigenicity
A06397	Acute Toxicity, Skin and Eye Irritation
A06398	Acute Toxicity
A06399	Acute Toxicity, Skin and Eye Irritation
A06400	Acute Toxicity
A06401	Acute Toxicity
A06402	Acute Toxicity, Reproductive Effects, Multiple Dose Effects
A06403	Acute Toxicity, Reproductive Effects, Multiple Dose Effects
A06404	Acute Toxicity, Mutagenicity
A06405	Acute Toxicity, Multiple Dose Effects
A06406	Acute Toxicity, Mutagenicity
A06407	Acute Toxicity, Multiple Dose Effects
A06408	Acute Toxicity, Skin and Eye Irritation
A06409	Acute Toxicity
A06410	Acute Toxicity
A06411	Acute Toxicity
A06412	Acute Toxicity
A06413	Acute Toxicity
A06414	Acute Toxicity, Skin and Eye Irritation
A06415	Acute Toxicity, Skin and Eye Irritation
A06416	Acute Toxicity, Mutagenicity
A06417	Acute Toxicity
A06418	Acute Toxicity
A06419	Acute Toxicity
A06420	Acute Toxicity
A06421	Acute Toxicity, Tumorigenicity
A06422	Acute Toxicity, Skin and Eye Irritation
A06423	Acute Toxicity
A06424	Mutagenicity, Tumorigenicity
A06425	Acute Toxicity
A06426	Mutagenicity
A06427	Acute Toxicity
A06428	Acute Toxicity, Mutagenicity, Tumorigenicity
A06429	Tumorigenicity
A06430	Mutagenicity, Tumorigenicity
A06431	Acute Toxicity
A06432	Acute Toxicity, Tumorigenicity
A06433	Acute Toxicity, Multiple Dose Effects
A06434	Mutagenicity
A06435	Acute Toxicity, Multiple Dose Effects
A06436	Multiple Dose Effects

A06437	Mutagenicity
A06438	Acute Toxicity, Tumorigenicity, Skin and Eye Irritation
A06439	Mutagenicity, Multiple Dose Effects
A06440	Acute Toxicity
A06441	Acute Toxicity, Skin and Eye Irritation
A06442	Acute Toxicity
A06443	Acute Toxicity
A06444	Acute Toxicity
A06445	Acute Toxicity, Mutagenicity, Tumorigenicity
A06446	Acute Toxicity
A06447	Mutagenicity
A06448	Tumorigenicity
A06449	Acute Toxicity
A06450	Acute Toxicity
A06451	Acute Toxicity
A06452	Acute Toxicity
A06453	Acute Toxicity, Mutagenicity, Skin and Eye Irritation, Reproductive Effects, Multiple Dose Effects
A06454	Acute Toxicity
A06455	Acute Toxicity
A06456	Acute Toxicity
A06457	Mutagenicity
A06458	Reproductive Effects
A06459	Mutagenicity, Tumorigenicity
A06460	Acute Toxicity
A06461	Mutagenicity
A06462	Mutagenicity
A06463	Acute Toxicity, Multiple Dose Effects
A06464	Mutagenicity
A06465	Skin and Eye Irritation
A06466	Acute Toxicity, Multiple Dose Effects
A06467	Acute Toxicity
A06468	Acute Toxicity, Mutagenicity
A06469	Acute Toxicity, Mutagenicity, Skin and Eye Irritation, Reproductive Effects, Multiple Dose Effects
A06470	Acute Toxicity, Skin and Eye Irritation, Multiple Dose Effects
A06471	Acute Toxicity, Skin and Eye Irritation
A06472	Acute Toxicity, Mutagenicity, Reproductive Effects, Multiple Dose Effects
A06473	Acute Toxicity, Mutagenicity, Reproductive Effects, Multiple Dose Effects
A06474	Acute Toxicity
A06475	Acute Toxicity
A06476	Acute Toxicity, Mutagenicity, Tumorigenicity
A06477	Acute Toxicity
A06478	Acute Toxicity
A06479	Acute Toxicity
A06480	Mutagenicity, Tumorigenicity

A06481	Acute Toxicity, Mutagenicity
A06482	Acute Toxicity
A06483	Mutagenicity
A06484	Mutagenicity
A06485	Mutagenicity
A06486	Acute Toxicity
A06487	Acute Toxicity
A06488	Acute Toxicity
A06489	Acute Toxicity
A06490	Acute Toxicity
A06491	Acute Toxicity, Mutagenicity, Skin and Eye Irritation, Multiple Dose Effects
A06492	Acute Toxicity
A06493	Acute Toxicity
A06494	Acute Toxicity, Skin and Eye Irritation
A06495	Acute Toxicity
A06496	Mutagenicity, Reproductive Effects
A06497	Acute Toxicity
A06498	Acute Toxicity
A06499	Skin and Eye Irritation
A06500	Acute Toxicity
A06501	Acute Toxicity
A06502	Acute Toxicity
A06503	Acute Toxicity
A06504	Acute Toxicity
A06505	Acute Toxicity
A06506	Acute Toxicity
A06507	Acute Toxicity, Tumorigenicity
A06508	Acute Toxicity
A06509	Mutagenicity
A06510	Acute Toxicity, Skin and Eye Irritation
A06511	Mutagenicity
A06512	Acute Toxicity
A06513	Mutagenicity
A06514	Acute Toxicity, Reproductive Effects, Multiple Dose Effects
A06515	Acute Toxicity
A06516	Mutagenicity, Tumorigenicity
A06517	Acute Toxicity, Mutagenicity, Tumorigenicity
A06518	Mutagenicity
A06519	Acute Toxicity
A06520	Acute Toxicity, Multiple Dose Effects
A06521	Acute Toxicity
A06522	Acute Toxicity
A06523	Acute Toxicity, Mutagenicity, Skin and Eye Irritation, Reproductive Effects, Multiple Dose Effects
A06524	Acute Toxicity
A06525	Acute Toxicity

A06526	Acute Toxicity
A06527	Acute Toxicity, Mutagenicity, Reproductive Effects, Multiple Dose Effects
A06528	Acute Toxicity
A06529	Acute Toxicity
A06530	Acute Toxicity, Mutagenicity, Tumorigenicity
A06531	Acute Toxicity, Reproductive Effects, Multiple Dose Effects
A06532	Mutagenicity
A06533	Mutagenicity, Tumorigenicity
A06534	Acute Toxicity, Mutagenicity, Multiple Dose Effects
A06535	Acute Toxicity, Mutagenicity, Tumorigenicity
A06536	Acute Toxicity, Mutagenicity
A06537	Non-toxicity
A06538	Acute Toxicity, Mutagenicity
A06539	Acute Toxicity
A06540	Acute Toxicity
A06541	Tumorigenicity
A06542	Acute Toxicity
A06543	Non-toxicity
A06544	Acute Toxicity
A06545	Acute Toxicity
A06546	Mutagenicity
A06547	Acute Toxicity
A06548	Acute Toxicity
A06549	Mutagenicity, Tumorigenicity
A06550	Acute Toxicity
A06551	Acute Toxicity
A06552	Mutagenicity
A06553	Acute Toxicity
A06554	Acute Toxicity, Mutagenicity
A06555	Acute Toxicity
A06556	Acute Toxicity
A06557	Acute Toxicity, Skin and Eye Irritation
A06558	Acute Toxicity, Mutagenicity, Tumorigenicity, Skin and Eye Irritation, Multiple Dose Effects
A06559	Acute Toxicity
A06560	Acute Toxicity
A06561	Acute Toxicity, Mutagenicity
A06562	Mutagenicity
A06563	Acute Toxicity
A06564	Acute Toxicity
A06565	Acute Toxicity, Mutagenicity, Tumorigenicity
A06566	Mutagenicity, Tumorigenicity
A06567	Acute Toxicity, Skin and Eye Irritation
A06568	Acute Toxicity
A06569	Acute Toxicity
A06570	Non-toxicity

A06571	Tumorigenicity
A06572	Acute Toxicity, Mutagenicity, Tumorigenicity, Reproductive Effects,
A06573	Acute Toxicity, Skin and Eye Irritation
A06574	Mutagenicity, Tumorigenicity, Multiple Dose Effects
A06575	Acute Toxicity
A06576	Acute Toxicity
A06577	Acute Toxicity
A06578	Reproductive Effects
A06579	Acute Toxicity, Mutagenicity, Tumorigenicity, Multiple Dose Effects
A06580	Mutagenicity
A06581	Acute Toxicity, Multiple Dose Effects
A06582	Acute Toxicity
A06583	Acute Toxicity
A06584	Acute Toxicity, Tumorigenicity
A06585	Acute Toxicity, Mutagenicity, Multiple Dose Effects
A06586	Acute Toxicity, Mutagenicity
A06587	Acute Toxicity
A06588	Mutagenicity, Reproductive Effects, Multiple Dose Effects
A06589	Acute Toxicity
A06590	Acute Toxicity
A06591	Acute Toxicity, Mutagenicity, Skin and Eye Irritation, Multiple Dose Effects
A06592	Acute Toxicity
A06593	Acute Toxicity, Tumorigenicity, Reproductive Effects
A06594	Acute Toxicity
A06595	Acute Toxicity, Mutagenicity
A06596	Acute Toxicity
A06597	Acute Toxicity, Mutagenicity, Tumorigenicity, Multiple Dose Effects
A06598	Mutagenicity
A06599	Acute Toxicity, Mutagenicity, Multiple Dose Effects
A06600	Acute Toxicity
A06601	Acute Toxicity, Mutagenicity, Tumorigenicity, Multiple Dose Effects
A06602	Acute Toxicity, Mutagenicity, Reproductive Effects, Multiple Dose Effects
A06603	Mutagenicity, Tumorigenicity
A06604	Acute Toxicity, Skin and Eye Irritation
A06605	Acute Toxicity
A06606	Acute Toxicity, Skin and Eye Irritation, Multiple Dose Effects
A06607	Acute Toxicity, Mutagenicity, Tumorigenicity, Reproductive Effects,
A06608	Mutagenicity
A06609	Acute Toxicity, Mutagenicity
A06610	Acute Toxicity, Mutagenicity, Skin and Eye Irritation, Multiple Dose Effects
A06611	Acute Toxicity, Reproductive Effects
A06612	Acute Toxicity
A06613	Acute Toxicity, Multiple Dose Effects
A06614	Acute Toxicity
A06615	Mutagenicity, Reproductive Effects, Multiple Dose Effects
A06616	Acute Toxicity

A06617	Acute Toxicity, Skin and Eye Irritation
A06618	Acute Toxicity
A06619	Acute Toxicity, Skin and Eye Irritation, Multiple Dose Effects
A06620	Acute Toxicity
A06621	Acute Toxicity
A06622	Acute Toxicity, Tumorigenicity, Multiple Dose Effects
A06623	Acute Toxicity, Mutagenicity, Tumorigenicity, Reproductive Effects,
A06624	Acute Toxicity, Mutagenicity, Tumorigenicity
A06625	Acute Toxicity, Skin and Eye Irritation, Multiple Dose Effects
A06626	Acute Toxicity, Mutagenicity, Tumorigenicity, Reproductive Effects
A06627	Acute Toxicity
A06628	Acute Toxicity
A06629	Acute Toxicity, Mutagenicity, Tumorigenicity, Reproductive Effects
A06630	Acute Toxicity, Reproductive Effects, Multiple Dose Effects
A06631	Acute Toxicity, Skin and Eye Irritation
A06632	Acute Toxicity
A06633	Acute Toxicity, Mutagenicity, Reproductive Effects, Multiple Dose Effects
A06634	Acute Toxicity
A06635	Acute Toxicity, Mutagenicity, Tumorigenicity, Multiple Dose Effects
A06636	Acute Toxicity, Mutagenicity, Tumorigenicity, Skin and Eye Irritation, Multiple Dose Effects
A06637	Acute Toxicity, Reproductive Effects
A06638	Acute Toxicity
A06639	Acute Toxicity
A06640	Acute Toxicity
A06641	Acute Toxicity, Mutagenicity, Reproductive Effects
A06642	Mutagenicity
A06643	Acute Toxicity, Skin and Eye Irritation
A06644	Acute Toxicity, Skin and Eye Irritation
A06645	Acute Toxicity, Mutagenicity
A06646	Acute Toxicity
A06647	Acute Toxicity
A06648	Acute Toxicity, Multiple Dose Effects
A06649	Acute Toxicity
A06650	Acute Toxicity, Mutagenicity, Tumorigenicity, Skin and Eye Irritation,
A06651	Non-toxicity
A06652	Acute Toxicity, Skin and Eye Irritation
A06653	Acute Toxicity, Mutagenicity, Tumorigenicity, Skin and Eye Irritation, Multiple Dose Effects
A06654	Acute Toxicity, Mutagenicity
A06655	Mutagenicity
A06656	Acute Toxicity
A06657	Acute Toxicity, Skin and Eye Irritation
A06658	Acute Toxicity, Skin and Eye Irritation
A06659	Mutagenicity, Reproductive Effects, Multiple Dose Effects
A06660	Mutagenicity

A06661	Acute Toxicity, Mutagenicity, Reproductive Effects, Multiple Dose Effects
A06662	Acute Toxicity
A06663	Acute Toxicity, Skin and Eye Irritation
A06664	Acute Toxicity, Reproductive Effects
A06665	Mutagenicity
A06666	Acute Toxicity
A06667	Acute Toxicity
A06668	Acute Toxicity, Reproductive Effects
A06669	Reproductive Effects
A06670	Mutagenicity
A06671	Acute Toxicity, Multiple Dose Effects
A06672	Acute Toxicity
A06673	Acute Toxicity, Multiple Dose Effects
A06674	Acute Toxicity, Skin and Eye Irritation
A06675	Acute Toxicity, Mutagenicity, Reproductive Effects, Multiple Dose Effects
A06676	Acute Toxicity
A06677	Acute Toxicity
A06678	Acute Toxicity, Mutagenicity, Skin and Eye Irritation, Reproductive Effects, Multiple Dose Effects
A06679	Acute Toxicity, Reproductive Effects, Multiple Dose Effects
A06680	Acute Toxicity, Mutagenicity, Reproductive Effects
A06681	Acute Toxicity
A06682	Acute Toxicity
A06683	Acute Toxicity
A06684	Mutagenicity
A06685	Acute Toxicity
A06686	Acute Toxicity, Mutagenicity, Tumorigenicity
A06687	Acute Toxicity, Multiple Dose Effects
A06688	Mutagenicity, Tumorigenicity, Skin and Eye Irritation, Multiple Dose Effects
A06689	Acute Toxicity, Mutagenicity, Tumorigenicity, Reproductive Effects,
A06690	Acute Toxicity
A06691	Acute Toxicity
A06692	Acute Toxicity
A06693	Acute Toxicity, Multiple Dose Effects
A06694	Acute Toxicity
A06695	Skin and Eye Irritation
A06696	Acute Toxicity, Reproductive Effects, Multiple Dose Effects
A06697	Acute Toxicity, Mutagenicity, Skin and Eye Irritation, Reproductive Effects, Multiple Dose Effects
A06698	Acute Toxicity
A06699	Acute Toxicity, Multiple Dose Effects
A06700	Acute Toxicity, Mutagenicity
A06701	Acute Toxicity, Mutagenicity, Tumorigenicity, Multiple Dose Effects
A06702	Acute Toxicity
A06703	Acute Toxicity
A06704	Acute Toxicity

A06705	Reproductive Effects
A06706	Acute Toxicity, Reproductive Effects, Multiple Dose Effects
A06707	Mutagenicity
A06708	Acute Toxicity, Mutagenicity, Tumorigenicity
A06709	Acute Toxicity
A06710	Mutagenicity
A06711	Mutagenicity, Tumorigenicity
A06712	Mutagenicity
A06713	Acute Toxicity
A06714	Acute Toxicity
A06715	Mutagenicity
A06716	Acute Toxicity
A06717	Acute Toxicity
A06718	Acute Toxicity
A06719	Acute Toxicity, Mutagenicity, Tumorigenicity, Multiple Dose Effects
A06720	Acute Toxicity, Mutagenicity, Tumorigenicity, Reproductive Effects
A06721	Acute Toxicity, Mutagenicity, Tumorigenicity
A06722	Tumorigenicity
A06723	Acute Toxicity, Reproductive Effects
A06724	Acute Toxicity, Multiple Dose Effects
A06725	Acute Toxicity, Mutagenicity
A06726	Tumorigenicity
A06727	Acute Toxicity, Skin and Eye Irritation
A06728	Acute Toxicity
A06729	Mutagenicity, Tumorigenicity
A06730	Mutagenicity
A06731	Mutagenicity, Tumorigenicity
A06732	Mutagenicity
A06733	Acute Toxicity
A06734	Acute Toxicity, Skin and Eye Irritation, Reproductive Effects, Multiple Dose
A06735	Mutagenicity
A06736	Acute Toxicity, Mutagenicity, Multiple Dose Effects
A06737	Acute Toxicity
A06738	Acute Toxicity
A06739	Acute Toxicity, Mutagenicity, Tumorigenicity, Multiple Dose Effects
A06740	Acute Toxicity
A06741	Acute Toxicity
A06742	Acute Toxicity
A06743	Acute Toxicity
A06744	Acute Toxicity, Mutagenicity
A06745	Mutagenicity
A06746	Acute Toxicity
A06747	Acute Toxicity, Skin and Eye Irritation
A06748	Acute Toxicity
A06749	Acute Toxicity, Tumorigenicity
A06750	Acute Toxicity

A06751	Non-toxicity
A06752	Acute Toxicity, Mutagenicity, Reproductive Effects, Multiple Dose Effects
A06753	Acute Toxicity, Mutagenicity, Multiple Dose Effects
A06754	Mutagenicity
A06755	Acute Toxicity, Skin and Eye Irritation
A06756	Acute Toxicity, Mutagenicity, Tumorigenicity, Skin and Eye Irritation,
A06757	Mutagenicity
A06758	Acute Toxicity, Skin and Eye Irritation, Reproductive Effects, Multiple Dose
A06759	Tumorigenicity
A06760	Acute Toxicity
A06761	Acute Toxicity
A06762	Mutagenicity
A06763	Acute Toxicity
A06764	Acute Toxicity, Mutagenicity, Multiple Dose Effects
A06765	Acute Toxicity, Mutagenicity, Tumorigenicity, Skin and Eye Irritation,
A06766	Acute Toxicity
A06767	Acute Toxicity, Skin and Eye Irritation
A06768	Acute Toxicity
A06769	Acute Toxicity, Mutagenicity
A06770	Acute Toxicity
A06771	Acute Toxicity, Multiple Dose Effects
A06772	Mutagenicity
A06773	Acute Toxicity, Skin and Eye Irritation
A06774	Acute Toxicity
A06775	Acute Toxicity
A06776	Acute Toxicity, Mutagenicity, Tumorigenicity
A06777	Acute Toxicity
A06778	Acute Toxicity, Mutagenicity, Multiple Dose Effects
A06779	Mutagenicity
A06780	Acute Toxicity
A06781	Acute Toxicity
A06782	Acute Toxicity
A06783	Acute Toxicity, Mutagenicity, Tumorigenicity
A06784	Acute Toxicity
A06785	Acute Toxicity, Multiple Dose Effects
A06786	Acute Toxicity
A06787	Acute Toxicity
A06788	Acute Toxicity
A06789	Acute Toxicity
A06790	Acute Toxicity, Mutagenicity, Tumorigenicity
A06791	Acute Toxicity
A06792	Acute Toxicity, Mutagenicity
A06793	Acute Toxicity
A06794	Acute Toxicity
A06795	Acute Toxicity
A06796	Acute Toxicity

A06797	Acute Toxicity, Skin and Eye Irritation
A06798	Acute Toxicity, Mutagenicity
A06799	Tumorigenicity
A06800	Acute Toxicity, Mutagenicity, Multiple Dose Effects
A06801	Acute Toxicity
A06802	Acute Toxicity, Mutagenicity
A06803	Acute Toxicity, Multiple Dose Effects
A06804	Acute Toxicity
A06805	Acute Toxicity
A06806	Acute Toxicity
A06807	Acute Toxicity
A06808	Acute Toxicity
A06809	Acute Toxicity
A06810	Acute Toxicity, Multiple Dose Effects
A06811	Acute Toxicity
A06812	Acute Toxicity
A06813	Acute Toxicity
A06814	Acute Toxicity, Multiple Dose Effects
A06815	Acute Toxicity
A06816	Acute Toxicity
A06817	Acute Toxicity
A06818	Acute Toxicity, Mutagenicity
A06819	Acute Toxicity, Mutagenicity, Skin and Eye Irritation, Multiple Dose Effects
A06820	Mutagenicity
A06821	Mutagenicity
A06822	Skin and Eye Irritation
A06823	Mutagenicity
A06824	Acute Toxicity
A06825	Non-toxicity
A06826	Acute Toxicity
A06827	Acute Toxicity
A06828	Mutagenicity
A06829	Acute Toxicity, Mutagenicity
A06830	Mutagenicity, Tumorigenicity
A06831	Tumorigenicity
A06832	Non-toxicity
A06833	Acute Toxicity
A06834	Acute Toxicity, Mutagenicity, Tumorigenicity
A06835	Mutagenicity
A06836	Acute Toxicity
A06837	Mutagenicity, Tumorigenicity
A06838	Acute Toxicity
A06839	Acute Toxicity
A06840	Acute Toxicity
A06841	Acute Toxicity
A06842	Acute Toxicity

A06843	Acute Toxicity
A06844	Acute Toxicity, Mutagenicity, Tumorigenicity
A06845	Acute Toxicity
A06846	Multiple Dose Effects
A06847	Acute Toxicity
A06848	Acute Toxicity
A06849	Acute Toxicity
A06850	Acute Toxicity
A06851	Acute Toxicity, Tumorigenicity, Skin and Eye Irritation, Reproductive Effects, Multiple Dose Effects
A06852	Acute Toxicity
A06853	Acute Toxicity
A06854	Acute Toxicity, Reproductive Effects, Multiple Dose Effects
A06855	Acute Toxicity, Mutagenicity, Skin and Eye Irritation
A06856	Acute Toxicity
A06857	Acute Toxicity
A06858	Acute Toxicity
A06859	Acute Toxicity
A06860	Acute Toxicity, Mutagenicity, Reproductive Effects, Multiple Dose Effects
A06861	Acute Toxicity
A06862	Acute Toxicity
A06863	Acute Toxicity, Mutagenicity, Skin and Eye Irritation, Reproductive Effects, Multiple Dose Effects
A06864	Acute Toxicity, Skin and Eye Irritation, Reproductive Effects, Multiple Dose
A06865	Acute Toxicity
A06866	Tumorigenicity
A06867	Acute Toxicity, Tumorigenicity
A06868	Acute Toxicity, Mutagenicity, Tumorigenicity
A06869	Acute Toxicity
A06870	Acute Toxicity
A06871	Acute Toxicity, Mutagenicity
A06872	Mutagenicity
A06873	Acute Toxicity
A06874	Mutagenicity
A06875	Multiple Dose Effects
A06876	Acute Toxicity, Mutagenicity
A06877	Acute Toxicity, Mutagenicity, Skin and Eye Irritation
A06878	Acute Toxicity
A06879	Mutagenicity
A06880	Acute Toxicity
A06881	Acute Toxicity, Mutagenicity, Reproductive Effects, Multiple Dose Effects
A06882	Multiple Dose Effects
A06883	Acute Toxicity
A06884	Tumorigenicity
A06885	Acute Toxicity
A06886	Skin and Eye Irritation

A06887	Mutagenicity, Tumorigenicity
A06888	Acute Toxicity
A06889	Acute Toxicity
A06890	Acute Toxicity, Reproductive Effects, Multiple Dose Effects
A06891	Mutagenicity
A06892	Acute Toxicity
A06893	Acute Toxicity, Mutagenicity, Tumorigenicity
A06894	Acute Toxicity, Mutagenicity, Tumorigenicity
A06895	Acute Toxicity, Mutagenicity, Tumorigenicity
A06896	Acute Toxicity, Mutagenicity, Tumorigenicity
A06897	Acute Toxicity
A06898	Acute Toxicity
A06899	Acute Toxicity, Skin and Eye Irritation
A06900	Acute Toxicity, Skin and Eye Irritation
A06901	Acute Toxicity
A06902	Acute Toxicity
A06903	Non-toxicity
A06904	Acute Toxicity, Mutagenicity, Skin and Eye Irritation, Multiple Dose Effects
A06905	Acute Toxicity
A06906	Acute Toxicity, Multiple Dose Effects
A06907	Acute Toxicity, Mutagenicity, Tumorigenicity, Skin and Eye Irritation,
A06908	Reproductive Effects, Multiple Dose Effects
A06909	Acute Toxicity
A06910	Acute Toxicity
A06911	Mutagenicity, Tumorigenicity
A06912	Acute Toxicity, Mutagenicity
A06913	Acute Toxicity, Skin and Eye Irritation
A06914	Acute Toxicity, Mutagenicity, Multiple Dose Effects
A06915	Acute Toxicity, Multiple Dose Effects
A06916	Acute Toxicity, Mutagenicity
A06917	Acute Toxicity, Multiple Dose Effects
A06918	Acute Toxicity
A06919	Acute Toxicity
A06920	Mutagenicity, Tumorigenicity
A06921	Mutagenicity, Tumorigenicity
A06922	Mutagenicity
A06923	Acute Toxicity
A06924	Acute Toxicity, Mutagenicity, Reproductive Effects, Multiple Dose Effects
A06925	Mutagenicity, Tumorigenicity
A06926	Mutagenicity, Tumorigenicity
A06927	Acute Toxicity
A06928	Mutagenicity
A06929	Acute Toxicity
A06930	Acute Toxicity
A06931	Acute Toxicity, Mutagenicity
A06932	Mutagenicity, Tumorigenicity

A06933	Acute Toxicity
A06934	Acute Toxicity, Mutagenicity, Multiple Dose Effects
A06935	Mutagenicity
A06936	Acute Toxicity, Multiple Dose Effects
A06937	Acute Toxicity
A06938	Acute Toxicity, Reproductive Effects, Multiple Dose Effects
A06939	Acute Toxicity
A06940	Acute Toxicity
A06941	Acute Toxicity, Mutagenicity, Tumorigenicity, Reproductive Effects,
A06942	Acute Toxicity
A06943	Mutagenicity
A06944	Acute Toxicity
A06945	Non-toxicity
A06946	Acute Toxicity, Mutagenicity, Skin and Eye Irritation
A06947	Acute Toxicity, Mutagenicity, Tumorigenicity, Multiple Dose Effects
A06948	Acute Toxicity
A06949	Acute Toxicity, Mutagenicity, Skin and Eye Irritation, Multiple Dose Effects
A06950	Acute Toxicity, Multiple Dose Effects
A06951	Acute Toxicity, Mutagenicity
A06952	Mutagenicity
A06953	Mutagenicity, Tumorigenicity
A06954	Mutagenicity, Tumorigenicity
A06955	Acute Toxicity
A06956	Tumorigenicity
A06957	Acute Toxicity
A06958	Acute Toxicity, Mutagenicity, Tumorigenicity
A06959	Acute Toxicity
A06960	Acute Toxicity
A06961	Acute Toxicity
A06962	Acute Toxicity
A06963	Acute Toxicity
A06964	Acute Toxicity
A06965	Acute Toxicity, Reproductive Effects
A06966	Acute Toxicity
A06967	Mutagenicity, Tumorigenicity
A06968	Acute Toxicity, Reproductive Effects, Multiple Dose Effects
A06969	Acute Toxicity, Mutagenicity, Tumorigenicity, Skin and Eye Irritation,
A06970	Acute Toxicity, Mutagenicity, Tumorigenicity, Skin and Eye Irritation,
A06971	Acute Toxicity, Mutagenicity, Tumorigenicity, Skin and Eye Irritation, Multiple Dose Effects
A06972	Acute Toxicity, Reproductive Effects, Multiple Dose Effects
A06973	Acute Toxicity
A06974	Acute Toxicity, Reproductive Effects, Multiple Dose Effects
A06975	Acute Toxicity, Reproductive Effects
A06976	Acute Toxicity
A06977	Acute Toxicity

A06978	Acute Toxicity, Multiple Dose Effects
A06979	Acute Toxicity, Mutagenicity, Skin and Eye Irritation, Reproductive Effects
A06980	Acute Toxicity
A06981	Mutagenicity
A06982	Non-toxicity
A06983	Acute Toxicity
A06984	Acute Toxicity
A06985	Tumorigenicity
A06986	Acute Toxicity
A06987	Acute Toxicity, Multiple Dose Effects
A06988	Acute Toxicity, Skin and Eye Irritation, Reproductive Effects, Multiple Dose
A06989	Acute Toxicity
A06990	Reproductive Effects
A06991	Acute Toxicity, Mutagenicity
A06992	Acute Toxicity
A06993	Mutagenicity, Skin and Eye Irritation
A06994	Acute Toxicity, Mutagenicity
A06995	Mutagenicity
A06996	Mutagenicity
A06997	Acute Toxicity, Mutagenicity, Tumorigenicity, Reproductive Effects
A06998	Mutagenicity
A06999	Mutagenicity
A07000	Acute Toxicity
A07001	Mutagenicity, Reproductive Effects, Multiple Dose Effects
A07002	Acute Toxicity
A07003	Acute Toxicity, Multiple Dose Effects
A07004	Acute Toxicity, Mutagenicity, Tumorigenicity, Reproductive Effects,
A07005	Acute Toxicity, Mutagenicity
A07006	Acute Toxicity
A07007	Acute Toxicity, Mutagenicity, Multiple Dose Effects
A07008	Mutagenicity
A07009	Mutagenicity
A07010	Mutagenicity, Tumorigenicity
A07011	Acute Toxicity
A07012	Acute Toxicity
A07013	Acute Toxicity
A07014	Acute Toxicity, Mutagenicity, Tumorigenicity
A07015	Mutagenicity, Tumorigenicity
A07016	Acute Toxicity, Skin and Eye Irritation
A07017	Multiple Dose Effects
A07018	Acute Toxicity
A07019	Acute Toxicity, Mutagenicity
A07020	Acute Toxicity, Mutagenicity
A07021	Mutagenicity
A07022	Skin and Eye Irritation
A07023	Acute Toxicity, Skin and Eye Irritation

A07024	Acute Toxicity
A07025	Mutagenicity
A07026	Mutagenicity
A07027	Acute Toxicity, Mutagenicity, Multiple Dose Effects
A07028	Acute Toxicity
A07029	Acute Toxicity
A07030	Mutagenicity
A07031	Mutagenicity
A07032	Acute Toxicity, Mutagenicity
A07033	Acute Toxicity
A07034	Acute Toxicity
A07035	Acute Toxicity
A07036	Mutagenicity, Tumorigenicity
A07037	Acute Toxicity, Mutagenicity, Multiple Dose Effects
A07038	Mutagenicity, Tumorigenicity
A07039	Mutagenicity
A07040	Acute Toxicity
A07041	Acute Toxicity
A07042	Acute Toxicity
A07043	Acute Toxicity
A07044	Mutagenicity
A07045	Acute Toxicity, Skin and Eye Irritation, Multiple Dose Effects
A07046	Acute Toxicity
A07047	Acute Toxicity
A07048	Acute Toxicity
A07049	Skin and Eye Irritation
A07050	Acute Toxicity, Mutagenicity, Reproductive Effects
A07051	Mutagenicity, Multiple Dose Effects
A07052	Acute Toxicity, Skin and Eye Irritation, Reproductive Effects, Multiple Dose
A07053	Acute Toxicity, Skin and Eye Irritation
A07054	Acute Toxicity, Mutagenicity, Tumorigenicity, Reproductive Effects,
A07055	Acute Toxicity
A07056	Acute Toxicity, Mutagenicity, Tumorigenicity
A07057	Acute Toxicity, Mutagenicity, Tumorigenicity
A07058	Acute Toxicity
A07059	Acute Toxicity, Mutagenicity, Reproductive Effects, Multiple Dose Effects
A07060	Acute Toxicity
A07061	Acute Toxicity
A07062	Skin and Eye Irritation
A07063	Tumorigenicity
A07064	Mutagenicity, Tumorigenicity
A07065	Tumorigenicity
A07066	Tumorigenicity
A07067	Acute Toxicity, Mutagenicity
A07068	Mutagenicity
A07069	Mutagenicity, Tumorigenicity, Multiple Dose Effects

A07070	Acute Toxicity
A07071	Acute Toxicity
A07072	Acute Toxicity
A07073	Acute Toxicity, Mutagenicity, Tumorigenicity, Reproductive Effects,
A07074	Acute Toxicity, Mutagenicity, Reproductive Effects
A07075	Acute Toxicity
A07076	Acute Toxicity, Tumorigenicity, Reproductive Effects, Multiple Dose Effects
A07077	Acute Toxicity, Skin and Eye Irritation
A07078	Acute Toxicity, Skin and Eye Irritation, Multiple Dose Effects
A07079	Acute Toxicity, Multiple Dose Effects
A07080	Acute Toxicity, Mutagenicity, Reproductive Effects, Multiple Dose Effects
A07081	Acute Toxicity, Mutagenicity, Tumorigenicity
A07082	Acute Toxicity
A07083	Acute Toxicity, Mutagenicity, Multiple Dose Effects
A07084	Reproductive Effects
A07085	Mutagenicity, Tumorigenicity
A07086	Acute Toxicity, Multiple Dose Effects
A07087	Acute Toxicity
A07088	Acute Toxicity, Mutagenicity, Tumorigenicity, Reproductive Effects,
A07089	Acute Toxicity
A07090	Acute Toxicity, Mutagenicity, Tumorigenicity, Multiple Dose Effects
A07091	Non-toxicity
A07092	Acute Toxicity
A07093	Acute Toxicity
A07094	Acute Toxicity
A07095	Acute Toxicity
A07096	Acute Toxicity
A07097	Acute Toxicity, Reproductive Effects
A07098	Mutagenicity
A07099	Acute Toxicity
A07100	Acute Toxicity
A07101	Acute Toxicity, Skin and Eye Irritation, Reproductive Effects, Multiple Dose
A07102	Acute Toxicity
A07103	Acute Toxicity
A07104	Acute Toxicity
A07105	Acute Toxicity
A07106	Acute Toxicity
A07107	Mutagenicity
A07108	Acute Toxicity
A07109	Acute Toxicity, Mutagenicity, Reproductive Effects
A07110	Tumorigenicity
A07111	Acute Toxicity, Mutagenicity, Reproductive Effects, Multiple Dose Effects
A07112	Acute Toxicity
A07113	Acute Toxicity
A07114	Acute Toxicity
A07115	Acute Toxicity

A07116	Acute Toxicity
A07117	Mutagenicity
A07118	Acute Toxicity
A07119	Mutagenicity, Tumorigenicity
A07120	Acute Toxicity
A07121	Reproductive Effects
A07122	Acute Toxicity, Mutagenicity, Reproductive Effects, Multiple Dose Effects
A07123	Acute Toxicity, Skin and Eye Irritation
A07124	Acute Toxicity, Mutagenicity, Skin and Eye Irritation, Multiple Dose Effects
A07125	Acute Toxicity, Skin and Eye Irritation, Multiple Dose Effects
A07126	Acute Toxicity, Mutagenicity, Skin and Eye Irritation
A07127	Acute Toxicity, Skin and Eye Irritation
A07128	Acute Toxicity, Skin and Eye Irritation
A07129	Acute Toxicity, Mutagenicity, Tumorigenicity, Skin and Eye Irritation,
A07130	Acute Toxicity, Mutagenicity, Skin and Eye Irritation
A07131	Acute Toxicity
A07132	Mutagenicity, Tumorigenicity
A07133	Acute Toxicity
A07134	Acute Toxicity
A07135	Acute Toxicity, Skin and Eye Irritation
A07136	Acute Toxicity, Mutagenicity, Skin and Eye Irritation
A07137	Acute Toxicity, Skin and Eye Irritation
A07138	Acute Toxicity, Mutagenicity, Skin and Eye Irritation
A07139	Acute Toxicity, Skin and Eye Irritation
A07140	Acute Toxicity, Mutagenicity, Skin and Eye Irritation, Multiple Dose Effects
A07141	Acute Toxicity, Mutagenicity, Tumorigenicity
A07142	Acute Toxicity, Mutagenicity, Skin and Eye Irritation, Multiple Dose Effects
A07143	Acute Toxicity, Mutagenicity
A07144	Acute Toxicity, Mutagenicity, Skin and Eye Irritation, Multiple Dose Effects
A07145	Acute Toxicity, Mutagenicity, Skin and Eye Irritation, Multiple Dose Effects
A07146	Acute Toxicity, Skin and Eye Irritation, Multiple Dose Effects
A07147	Acute Toxicity, Skin and Eye Irritation
A07148	Acute Toxicity, Skin and Eye Irritation
A07149	Acute Toxicity, Mutagenicity, Skin and Eye Irritation, Multiple Dose Effects
A07150	Acute Toxicity, Skin and Eye Irritation
A07151	Acute Toxicity, Mutagenicity
A07152	Acute Toxicity, Mutagenicity, Tumorigenicity, Skin and Eye Irritation,
A07153	Acute Toxicity, Mutagenicity, Skin and Eye Irritation, Reproductive Effects, Multiple Dose Effects
A07154	Acute Toxicity, Mutagenicity, Skin and Eye Irritation, Multiple Dose Effects
A07155	Acute Toxicity, Skin and Eye Irritation
A07156	Acute Toxicity, Skin and Eye Irritation
A07157	Acute Toxicity, Mutagenicity, Tumorigenicity, Skin and Eye Irritation
A07158	Acute Toxicity, Mutagenicity, Skin and Eye Irritation, Reproductive Effects, Multiple Dose Effects
A07159	Acute Toxicity, Skin and Eye Irritation

A07160	Acute Toxicity, Skin and Eye Irritation
A07161	Mutagenicity, Skin and Eye Irritation
A07162	Acute Toxicity, Mutagenicity, Tumorigenicity, Skin and Eye Irritation
A07163	Acute Toxicity, Mutagenicity, Skin and Eye Irritation, Multiple Dose Effects
A07164	Acute Toxicity, Mutagenicity, Skin and Eye Irritation, Reproductive Effects, Multiple Dose Effects
A07165	Acute Toxicity, Mutagenicity, Tumorigenicity, Skin and Eye Irritation,
A07166	Acute Toxicity, Mutagenicity
A07167	Acute Toxicity
A07168	Acute Toxicity, Skin and Eye Irritation, Reproductive Effects
A07169	Acute Toxicity, Mutagenicity, Skin and Eye Irritation, Multiple Dose Effects
A07170	Acute Toxicity
A07171	Acute Toxicity
A07172	Acute Toxicity, Skin and Eye Irritation, Multiple Dose Effects
A07173	Acute Toxicity, Mutagenicity, Skin and Eye Irritation, Multiple Dose Effects
A07174	Acute Toxicity, Skin and Eye Irritation, Multiple Dose Effects
A07175	Mutagenicity, Tumorigenicity
A07176	Acute Toxicity
A07177	Acute Toxicity, Mutagenicity, Tumorigenicity, Multiple Dose Effects
A07178	Acute Toxicity, Mutagenicity, Skin and Eye Irritation, Multiple Dose Effects
A07179	Acute Toxicity, Multiple Dose Effects
A07180	Acute Toxicity, Mutagenicity, Skin and Eye Irritation, Reproductive Effects, Multiple Dose Effects
A07181	Acute Toxicity
A07182	Acute Toxicity, Skin and Eye Irritation, Reproductive Effects
A07183	Acute Toxicity, Skin and Eye Irritation
A07184	Acute Toxicity
A07185	Acute Toxicity, Mutagenicity
A07186	Acute Toxicity, Mutagenicity, Multiple Dose Effects
A07187	Acute Toxicity, Mutagenicity, Skin and Eye Irritation, Reproductive Effects, Multiple Dose Effects
A07188	Acute Toxicity
A07189	Acute Toxicity
A07190	Acute Toxicity, Mutagenicity, Tumorigenicity, Skin and Eye Irritation,
A07191	Acute Toxicity, Mutagenicity, Tumorigenicity, Skin and Eye Irritation,
A07192	Acute Toxicity
A07193	Acute Toxicity
A07194	Acute Toxicity, Skin and Eye Irritation, Multiple Dose Effects
A07195	Acute Toxicity, Tumorigenicity
A07196	Acute Toxicity
A07197	Acute Toxicity, Mutagenicity, Tumorigenicity, Skin and Eye Irritation,
A07198	Acute Toxicity, Mutagenicity, Tumorigenicity, Skin and Eye Irritation,
A07199	Acute Toxicity, Mutagenicity, Tumorigenicity, Reproductive Effects,
A07200	Acute Toxicity, Mutagenicity, Tumorigenicity, Skin and Eye Irritation,
A07201	Acute Toxicity, Mutagenicity, Tumorigenicity, Skin and Eye Irritation,
A07202	Acute Toxicity

A07203	Acute Toxicity, Reproductive Effects
A07204	Acute Toxicity
A07205	Acute Toxicity, Multiple Dose Effects
A07206	Acute Toxicity, Tumorigenicity, Multiple Dose Effects
A07207	Acute Toxicity, Mutagenicity, Tumorigenicity, Reproductive Effects,
A07208	Acute Toxicity
A07209	Acute Toxicity, Mutagenicity, Tumorigenicity, Skin and Eye Irritation,
A07210	Acute Toxicity, Tumorigenicity, Skin and Eye Irritation, Reproductive Effects, Multiple Dose Effects
A07211	Acute Toxicity, Skin and Eye Irritation, Multiple Dose Effects
A07212	Acute Toxicity, Mutagenicity, Tumorigenicity, Multiple Dose Effects
A07213	Acute Toxicity
A07214	Acute Toxicity, Skin and Eye Irritation
A07215	Mutagenicity, Skin and Eye Irritation
A07216	Acute Toxicity, Mutagenicity, Skin and Eye Irritation, Multiple Dose Effects
A07217	Acute Toxicity
A07218	Acute Toxicity, Mutagenicity, Tumorigenicity, Skin and Eye Irritation, Multiple Dose Effects
A07219	Acute Toxicity, Mutagenicity, Tumorigenicity, Multiple Dose Effects
A07220	Acute Toxicity
A07221	Acute Toxicity, Mutagenicity, Multiple Dose Effects
A07222	Acute Toxicity
A07223	Acute Toxicity, Skin and Eye Irritation
A07224	Acute Toxicity, Mutagenicity
A07225	Acute Toxicity
A07226	Acute Toxicity, Tumorigenicity, Multiple Dose Effects
A07227	Mutagenicity
A07228	Acute Toxicity
A07229	Acute Toxicity, Mutagenicity, Multiple Dose Effects
A07230	Acute Toxicity
A07231	Tumorigenicity, Multiple Dose Effects
A07232	Acute Toxicity, Mutagenicity, Tumorigenicity, Reproductive Effects
A07233	Mutagenicity
A07234	Acute Toxicity, Mutagenicity
A07235	Mutagenicity
A07236	Acute Toxicity, Mutagenicity
A07237	Acute Toxicity
A07238	Acute Toxicity, Mutagenicity, Multiple Dose Effects
A07239	Acute Toxicity, Mutagenicity, Tumorigenicity, Multiple Dose Effects
A07240	Acute Toxicity
A07241	Acute Toxicity
A07242	Acute Toxicity, Mutagenicity, Tumorigenicity, Skin and Eye Irritation,
A07243	Acute Toxicity
A07244	Acute Toxicity, Reproductive Effects, Multiple Dose Effects
A07245	Acute Toxicity
A07246	Mutagenicity

A07247	Acute Toxicity
A07248	Acute Toxicity
A07249	Acute Toxicity
A07250	Acute Toxicity, Multiple Dose Effects
A07251	Acute Toxicity
A07252	Acute Toxicity
A07253	Acute Toxicity, Mutagenicity, Multiple Dose Effects
A07254	Acute Toxicity
A07255	Acute Toxicity, Skin and Eye Irritation
A07256	Acute Toxicity, Mutagenicity
A07257	Mutagenicity
A07258	Acute Toxicity
A07259	Acute Toxicity, Reproductive Effects
A07260	Mutagenicity
A07261	Acute Toxicity
A07262	Acute Toxicity
A07263	Acute Toxicity
A07264	Acute Toxicity, Tumorigenicity, Reproductive Effects, Multiple Dose Effects
A07265	Acute Toxicity, Reproductive Effects, Multiple Dose Effects
A07266	Acute Toxicity
A07267	Acute Toxicity
A07268	Mutagenicity, Tumorigenicity
A07269	Acute Toxicity, Mutagenicity
A07270	Mutagenicity
A07271	Acute Toxicity, Multiple Dose Effects
A07272	Acute Toxicity
A07273	Acute Toxicity, Mutagenicity, Multiple Dose Effects
A07274	Acute Toxicity, Mutagenicity
A07275	Acute Toxicity, Tumorigenicity, Multiple Dose Effects
A07276	Tumorigenicity
A07277	Mutagenicity
A07278	Acute Toxicity, Mutagenicity
A07279	Acute Toxicity, Mutagenicity
A07280	Acute Toxicity, Mutagenicity, Multiple Dose Effects
A07281	Acute Toxicity, Mutagenicity, Skin and Eye Irritation, Multiple Dose Effects
A07282	Acute Toxicity, Mutagenicity, Tumorigenicity
A07283	Acute Toxicity, Mutagenicity, Reproductive Effects
A07284	Non-toxicity
A07285	Acute Toxicity
A07286	Mutagenicity, Tumorigenicity
A07287	Acute Toxicity, Mutagenicity
A07288	Acute Toxicity, Mutagenicity, Multiple Dose Effects
A07289	Acute Toxicity, Skin and Eye Irritation
A07290	Acute Toxicity, Mutagenicity
A07291	Acute Toxicity, Multiple Dose Effects
A07292	Acute Toxicity, Mutagenicity, Reproductive Effects

A07293	Mutagenicity, Tumorigenicity
A07294	Acute Toxicity, Skin and Eye Irritation, Reproductive Effects, Multiple Dose
A07295	Acute Toxicity, Reproductive Effects, Multiple Dose Effects
A07296	Mutagenicity
A07297	Mutagenicity
A07298	Acute Toxicity
A07299	Acute Toxicity
A07300	Acute Toxicity
A07301	Mutagenicity
A07302	Reproductive Effects, Multiple Dose Effects
A07303	Acute Toxicity
A07304	Acute Toxicity, Mutagenicity, Reproductive Effects, Multiple Dose Effects
A07305	Acute Toxicity
A07306	Acute Toxicity
A07307	Acute Toxicity
A07308	Acute Toxicity, Skin and Eye Irritation, Reproductive Effects
A07309	Acute Toxicity, Multiple Dose Effects
A07310	Mutagenicity, Tumorigenicity
A07311	Acute Toxicity, Mutagenicity, Tumorigenicity, Skin and Eye Irritation
A07312	Acute Toxicity
A07313	Mutagenicity
A07314	Acute Toxicity, Mutagenicity, Skin and Eye Irritation, Reproductive Effects, Multiple Dose Effects
A07315	Acute Toxicity
A07316	Mutagenicity
A07317	Acute Toxicity
A07318	Mutagenicity
A07319	Acute Toxicity, Skin and Eye Irritation, Multiple Dose Effects
A07320	Acute Toxicity
A07321	Acute Toxicity, Mutagenicity, Skin and Eye Irritation, Multiple Dose Effects
A07322	Acute Toxicity, Reproductive Effects, Multiple Dose Effects
A07323	Acute Toxicity
A07324	Mutagenicity, Skin and Eye Irritation, Multiple Dose Effects
A07325	Acute Toxicity
A07326	Acute Toxicity
A07327	Acute Toxicity, Skin and Eye Irritation
A07328	Acute Toxicity
A07329	Acute Toxicity
A07330	Mutagenicity
A07331	Tumorigenicity
A07332	Acute Toxicity, Reproductive Effects, Multiple Dose Effects
A07333	Mutagenicity
A07334	Acute Toxicity, Skin and Eye Irritation
A07335	Acute Toxicity, Mutagenicity
A07336	Acute Toxicity, Mutagenicity, Skin and Eye Irritation
A07337	Mutagenicity, Tumorigenicity

A07338	Mutagenicity, Tumorigenicity
A07339	Mutagenicity
A07340	Mutagenicity
A07341	Acute Toxicity
A07342	Acute Toxicity, Mutagenicity, Tumorigenicity, Skin and Eye Irritation,
A07343	Acute Toxicity
A07344	Mutagenicity
A07345	Mutagenicity, Multiple Dose Effects
A07346	Acute Toxicity, Mutagenicity, Skin and Eye Irritation, Reproductive Effects, Multiple Dose Effects
A07347	Acute Toxicity, Skin and Eye Irritation
A07348	Acute Toxicity
A07349	Acute Toxicity, Reproductive Effects
A07350	Acute Toxicity, Tumorigenicity
A07351	Mutagenicity, Tumorigenicity
A07352	Acute Toxicity
A07353	Acute Toxicity
A07354	Mutagenicity
A07355	Acute Toxicity
A07356	Acute Toxicity
A07357	Acute Toxicity, Mutagenicity, Tumorigenicity
A07358	Acute Toxicity, Mutagenicity
A07359	Mutagenicity
A07360	Acute Toxicity, Skin and Eye Irritation, Multiple Dose Effects
A07361	Mutagenicity, Tumorigenicity
A07362	Acute Toxicity, Multiple Dose Effects
A07363	Acute Toxicity, Reproductive Effects
A07364	Acute Toxicity, Tumorigenicity, Multiple Dose Effects
A07365	Acute Toxicity, Skin and Eye Irritation, Multiple Dose Effects
A07366	Acute Toxicity, Skin and Eye Irritation
A07367	Acute Toxicity, Multiple Dose Effects
A07368	Mutagenicity
A07369	Acute Toxicity
A07370	Multiple Dose Effects
A07371	Acute Toxicity
A07372	Acute Toxicity, Tumorigenicity
A07373	Acute Toxicity, Skin and Eye Irritation
A07374	Acute Toxicity
A07375	Mutagenicity
A07376	Reproductive Effects
A07377	Acute Toxicity
A07378	Acute Toxicity, Skin and Eye Irritation, Reproductive Effects, Multiple Dose
A07379	Acute Toxicity
A07380	Acute Toxicity, Reproductive Effects, Multiple Dose Effects
A07381	Acute Toxicity, Mutagenicity
A07382	Acute Toxicity

A07383	Tumorigenicity
A07384	Acute Toxicity, Mutagenicity, Tumorigenicity, Reproductive Effects
A07385	Acute Toxicity
A07386	Acute Toxicity, Mutagenicity, Tumorigenicity
A07387	Mutagenicity
A07388	Mutagenicity, Tumorigenicity
A07389	Acute Toxicity, Mutagenicity, Tumorigenicity, Skin and Eye Irritation,
A07390	Acute Toxicity
A07391	Mutagenicity, Tumorigenicity
A07392	Mutagenicity
A07393	Acute Toxicity
A07394	Acute Toxicity, Mutagenicity, Skin and Eye Irritation, Reproductive Effects, Multiple Dose Effects
A07395	Acute Toxicity
A07396	Mutagenicity
A07397	Acute Toxicity
A07398	Acute Toxicity, Mutagenicity, Reproductive Effects, Multiple Dose Effects
A07399	Mutagenicity
A07400	Mutagenicity
A07401	Acute Toxicity
A07402	Acute Toxicity, Multiple Dose Effects
A07403	Acute Toxicity, Multiple Dose Effects
A07404	Acute Toxicity, Tumorigenicity
A07405	Acute Toxicity
A07406	Mutagenicity
A07407	Acute Toxicity, Mutagenicity, Tumorigenicity, Multiple Dose Effects
A07408	Acute Toxicity
A07409	Acute Toxicity, Reproductive Effects, Multiple Dose Effects
A07410	Acute Toxicity
A07411	Acute Toxicity
A07412	Acute Toxicity
A07413	Acute Toxicity, Mutagenicity, Multiple Dose Effects
A07414	Acute Toxicity, Multiple Dose Effects
A07415	Acute Toxicity
A07416	Acute Toxicity, Mutagenicity
A07417	Acute Toxicity, Mutagenicity, Multiple Dose Effects
A07418	Non-toxicity
A07419	Acute Toxicity
A07420	Acute Toxicity
A07421	Acute Toxicity, Reproductive Effects
A07422	Acute Toxicity, Mutagenicity, Tumorigenicity, Multiple Dose Effects
A07423	Tumorigenicity
A07424	Acute Toxicity, Multiple Dose Effects
A07425	Acute Toxicity, Multiple Dose Effects
A07426	Acute Toxicity
A07427	Acute Toxicity

A07428	Mutagenicity
A07429	Acute Toxicity
A07430	Acute Toxicity
A07431	Mutagenicity
A07432	Acute Toxicity, Multiple Dose Effects
A07433	Acute Toxicity, Mutagenicity, Tumorigenicity, Reproductive Effects,
A07434	Acute Toxicity, Skin and Eye Irritation, Reproductive Effects, Multiple Dose
A07435	Acute Toxicity
A07436	Acute Toxicity, Reproductive Effects, Multiple Dose Effects
A07437	Reproductive Effects
A07438	Acute Toxicity, Multiple Dose Effects
A07439	Acute Toxicity, Skin and Eye Irritation, Reproductive Effects, Multiple Dose
A07440	Acute Toxicity, Multiple Dose Effects
A07441	Mutagenicity, Tumorigenicity
A07442	Mutagenicity
A07443	Acute Toxicity
A07444	Acute Toxicity, Mutagenicity, Reproductive Effects, Multiple Dose Effects
A07445	Mutagenicity
A07446	Acute Toxicity
A07447	Acute Toxicity
A07448	Acute Toxicity
A07449	Acute Toxicity
A07450	Mutagenicity, Tumorigenicity
A07451	Acute Toxicity
A07452	Acute Toxicity, Reproductive Effects, Multiple Dose Effects
A07453	Mutagenicity
A07454	Mutagenicity
A07455	Acute Toxicity, Multiple Dose Effects
A07456	Acute Toxicity
A07457	Acute Toxicity, Mutagenicity, Multiple Dose Effects
A07458	Acute Toxicity
A07459	Acute Toxicity, Multiple Dose Effects
A07460	Acute Toxicity, Multiple Dose Effects
A07461	Acute Toxicity, Reproductive Effects, Multiple Dose Effects
A07462	Acute Toxicity, Skin and Eye Irritation, Reproductive Effects, Multiple Dose
A07463	Acute Toxicity, Mutagenicity, Multiple Dose Effects
A07464	Acute Toxicity
A07465	Mutagenicity
A07466	Acute Toxicity, Skin and Eye Irritation, Reproductive Effects, Multiple Dose
A07467	Acute Toxicity
A07468	Acute Toxicity, Mutagenicity, Reproductive Effects, Multiple Dose Effects
A07469	Acute Toxicity
A07470	Acute Toxicity
A07471	Acute Toxicity, Mutagenicity, Tumorigenicity, Reproductive Effects,
A07472	Acute Toxicity
A07473	Acute Toxicity

A07474	Acute Toxicity
A07475	Acute Toxicity, Mutagenicity, Reproductive Effects, Multiple Dose Effects
A07476	Acute Toxicity
A07477	Acute Toxicity, Mutagenicity, Reproductive Effects, Multiple Dose Effects
A07478	Acute Toxicity
A07479	Acute Toxicity, Mutagenicity, Tumorigenicity
A07480	Acute Toxicity, Mutagenicity, Skin and Eye Irritation, Reproductive Effects, Multiple Dose Effects
A07481	Acute Toxicity
A07482	Mutagenicity
A07483	Acute Toxicity, Mutagenicity, Reproductive Effects, Multiple Dose Effects
A07484	Mutagenicity
A07485	Acute Toxicity
A07486	Acute Toxicity, Mutagenicity, Skin and Eye Irritation
A07487	Acute Toxicity
A07488	Acute Toxicity
A07489	Acute Toxicity, Mutagenicity, Tumorigenicity, Multiple Dose Effects
A07490	Acute Toxicity, Mutagenicity, Multiple Dose Effects
A07491	Multiple Dose Effects
A07492	Mutagenicity
A07493	Mutagenicity
A07494	Mutagenicity
A07495	Acute Toxicity, Mutagenicity, Reproductive Effects, Multiple Dose Effects
A07496	Acute Toxicity, Reproductive Effects
A07497	Multiple Dose Effects
A07498	Acute Toxicity
A07499	Non-toxicity
A07500	Acute Toxicity
A07501	Acute Toxicity
A07502	Acute Toxicity
A07503	Acute Toxicity, Mutagenicity
A07504	Acute Toxicity
A07505	Acute Toxicity, Mutagenicity, Reproductive Effects, Multiple Dose Effects
A07506	Acute Toxicity
A07507	Acute Toxicity
A07508	Acute Toxicity, Mutagenicity
A07509	Mutagenicity, Tumorigenicity, Skin and Eye Irritation, Reproductive Effects
A07510	Acute Toxicity
A07511	Acute Toxicity, Mutagenicity, Tumorigenicity
A07512	Acute Toxicity
A07513	Mutagenicity
A07514	Mutagenicity, Tumorigenicity
A07515	Acute Toxicity, Multiple Dose Effects
A07516	Acute Toxicity
A07517	Acute Toxicity
A07518	Acute Toxicity

A07519	Acute Toxicity
A07520	Acute Toxicity, Mutagenicity, Reproductive Effects, Multiple Dose Effects
A07521	Mutagenicity
A07522	Mutagenicity
A07523	Acute Toxicity
A07524	Acute Toxicity
A07525	Acute Toxicity, Skin and Eye Irritation
A07526	Mutagenicity, Tumorigenicity
A07527	Acute Toxicity
A07528	Acute Toxicity
A07529	Mutagenicity, Tumorigenicity
A07530	Acute Toxicity, Reproductive Effects, Multiple Dose Effects
A07531	Acute Toxicity, Multiple Dose Effects
A07532	Acute Toxicity
A07533	Acute Toxicity, Multiple Dose Effects
A07534	Non-toxicity
A07535	Acute Toxicity, Reproductive Effects
A07536	Acute Toxicity
A07537	Acute Toxicity, Mutagenicity
A07538	Acute Toxicity, Multiple Dose Effects
A07539	Skin and Eye Irritation
A07540	Acute Toxicity
A07541	Acute Toxicity, Mutagenicity, Tumorigenicity, Reproductive Effects,
A07542	Acute Toxicity, Tumorigenicity
A07543	Mutagenicity, Reproductive Effects, Multiple Dose Effects
A07544	Acute Toxicity
A07545	Acute Toxicity
A07546	Acute Toxicity
A07547	Acute Toxicity
A07548	Acute Toxicity
A07549	Acute Toxicity
A07550	Acute Toxicity, Multiple Dose Effects
A07551	Acute Toxicity, Multiple Dose Effects
A07552	Acute Toxicity
A07553	Acute Toxicity, Mutagenicity, Multiple Dose Effects
A07554	Acute Toxicity, Mutagenicity, Reproductive Effects, Multiple Dose Effects
A07555	Mutagenicity
A07556	Acute Toxicity, Tumorigenicity, Reproductive Effects, Multiple Dose Effects
A07557	Acute Toxicity
A07558	Acute Toxicity, Skin and Eye Irritation, Reproductive Effects, Multiple Dose
A07559	Acute Toxicity, Mutagenicity
A07560	Acute Toxicity
A07561	Acute Toxicity, Mutagenicity, Tumorigenicity, Multiple Dose Effects
A07562	Multiple Dose Effects
A07563	Multiple Dose Effects
A07564	Acute Toxicity

A07565	Acute Toxicity, Multiple Dose Effects
A07566	Acute Toxicity
A07567	Acute Toxicity, Tumorigenicity, Multiple Dose Effects
A07568	Acute Toxicity
A07569	Acute Toxicity
A07570	Acute Toxicity
A07571	Acute Toxicity, Mutagenicity
A07572	Acute Toxicity, Reproductive Effects, Multiple Dose Effects
A07573	Reproductive Effects
A07574	Acute Toxicity, Mutagenicity
A07575	Acute Toxicity
A07576	Acute Toxicity
A07577	Mutagenicity, Tumorigenicity
A07578	Mutagenicity, Tumorigenicity
A07579	Mutagenicity
A07580	Mutagenicity, Tumorigenicity
A07581	Acute Toxicity
A07582	Acute Toxicity
A07583	Acute Toxicity, Mutagenicity
A07584	Non-toxicity
A07585	Mutagenicity
A07586	Acute Toxicity
A07587	Acute Toxicity
A07588	Acute Toxicity
A07589	Acute Toxicity, Skin and Eye Irritation
A07590	Acute Toxicity, Tumorigenicity, Reproductive Effects, Multiple Dose Effects
A07591	Acute Toxicity
A07592	Acute Toxicity, Reproductive Effects, Multiple Dose Effects
A07593	Acute Toxicity, Mutagenicity, Reproductive Effects, Multiple Dose Effects
A07594	Acute Toxicity
A07595	Mutagenicity, Reproductive Effects, Multiple Dose Effects
A07596	Acute Toxicity
A07597	Non-toxicity
A07598	Non-toxicity
A07599	Acute Toxicity
A07600	Mutagenicity
A07601	Mutagenicity
A07602	Multiple Dose Effects
A07603	Acute Toxicity, Mutagenicity, Skin and Eye Irritation, Reproductive Effects, Multiple Dose Effects
A07604	Acute Toxicity, Mutagenicity
A07605	Mutagenicity, Tumorigenicity
A07606	Mutagenicity
A07607	Acute Toxicity
A07608	Multiple Dose Effects
A07609	Acute Toxicity, Skin and Eye Irritation, Multiple Dose Effects

A07610	Acute Toxicity, Multiple Dose Effects
A07611	Acute Toxicity, Reproductive Effects
A07612	Acute Toxicity
A07613	Acute Toxicity, Reproductive Effects, Multiple Dose Effects
A07614	Acute Toxicity, Multiple Dose Effects
A07615	Acute Toxicity
A07616	Acute Toxicity, Mutagenicity
A07617	Acute Toxicity, Mutagenicity, Skin and Eye Irritation, Multiple Dose Effects
A07618	Acute Toxicity
A07619	Acute Toxicity
A07620	Mutagenicity, Tumorigenicity
A07621	Acute Toxicity, Mutagenicity
A07622	Tumorigenicity
A07623	Mutagenicity
A07624	Acute Toxicity
A07625	Acute Toxicity, Tumorigenicity
A07626	Mutagenicity, Tumorigenicity
A07627	Acute Toxicity
A07628	Mutagenicity
A07629	Acute Toxicity, Reproductive Effects, Multiple Dose Effects
A07630	Acute Toxicity, Multiple Dose Effects
A07631	Acute Toxicity
A07632	Acute Toxicity, Multiple Dose Effects
A07633	Acute Toxicity, Reproductive Effects, Multiple Dose Effects
A07634	Acute Toxicity, Reproductive Effects, Multiple Dose Effects
A07635	Acute Toxicity, Reproductive Effects, Multiple Dose Effects
A07636	Mutagenicity, Reproductive Effects, Multiple Dose Effects
A07637	Multiple Dose Effects
A07638	Multiple Dose Effects
A07639	Multiple Dose Effects
A07640	Acute Toxicity
A07641	Mutagenicity, Tumorigenicity
A07642	Acute Toxicity
A07643	Acute Toxicity, Mutagenicity, Reproductive Effects, Multiple Dose Effects
A07644	Acute Toxicity
A07645	Acute Toxicity
A07646	Mutagenicity, Tumorigenicity
A07647	Acute Toxicity, Skin and Eye Irritation, Reproductive Effects, Multiple Dose
A07648	Acute Toxicity
A07649	Acute Toxicity
A07650	Acute Toxicity
A07651	Acute Toxicity, Mutagenicity
A07652	Acute Toxicity
A07653	Multiple Dose Effects
A07654	Acute Toxicity, Tumorigenicity, Skin and Eye Irritation, Reproductive Effects, Multiple Dose Effects

A07655	Acute Toxicity, Skin and Eye Irritation
A07656	Reproductive Effects
A07657	Acute Toxicity, Multiple Dose Effects
A07658	Acute Toxicity
A07659	Acute Toxicity, Tumorigenicity
A07660	Acute Toxicity, Skin and Eye Irritation, Reproductive Effects, Multiple Dose
A07661	Acute Toxicity
A07662	Acute Toxicity
A07663	Acute Toxicity, Mutagenicity, Tumorigenicity, Multiple Dose Effects
A07664	Multiple Dose Effects
A07665	Acute Toxicity, Mutagenicity
A07666	Mutagenicity
A07667	Acute Toxicity, Tumorigenicity
A07668	Acute Toxicity
A07669	Acute Toxicity
A07670	Acute Toxicity, Mutagenicity, Multiple Dose Effects
A07671	Acute Toxicity
A07672	Acute Toxicity
A07673	Acute Toxicity, Reproductive Effects, Multiple Dose Effects
A07674	Acute Toxicity, Mutagenicity, Tumorigenicity, Multiple Dose Effects
A07675	Acute Toxicity, Reproductive Effects
A07676	Mutagenicity, Tumorigenicity
A07677	Non-toxicity
A07678	Mutagenicity, Tumorigenicity
A07679	Reproductive Effects
A07680	Acute Toxicity
A07681	Acute Toxicity
A07682	Acute Toxicity, Tumorigenicity, Multiple Dose Effects
A07683	Acute Toxicity
A07684	Acute Toxicity
A07685	Mutagenicity
A07686	Acute Toxicity
A07687	Acute Toxicity, Mutagenicity, Multiple Dose Effects
A07688	Acute Toxicity, Mutagenicity, Reproductive Effects, Multiple Dose Effects
A07689	Acute Toxicity
A07690	Acute Toxicity, Mutagenicity
A07691	Acute Toxicity
A07692	Acute Toxicity, Skin and Eye Irritation
A07693	Acute Toxicity, Multiple Dose Effects
A07694	Acute Toxicity
A07695	Acute Toxicity
A07696	Acute Toxicity, Mutagenicity, Multiple Dose Effects
A07697	Acute Toxicity, Tumorigenicity, Reproductive Effects, Multiple Dose Effects
A07698	Acute Toxicity
A07699	Mutagenicity

A07700	Acute Toxicity, Mutagenicity, Skin and Eye Irritation, Reproductive Effects, Multiple Dose Effects
A07701	Mutagenicity
A07702	Mutagenicity
A07703	Acute Toxicity
A07704	Mutagenicity, Tumorigenicity
A07705	Mutagenicity, Tumorigenicity
A07706	Acute Toxicity, Skin and Eye Irritation, Multiple Dose Effects
A07707	Acute Toxicity
A07708	Acute Toxicity, Multiple Dose Effects
A07709	Mutagenicity, Reproductive Effects
A07710	Acute Toxicity, Mutagenicity
A07711	Acute Toxicity, Mutagenicity, Tumorigenicity
A07712	Acute Toxicity
A07713	Acute Toxicity
A07714	Mutagenicity
A07715	Acute Toxicity
A07716	Mutagenicity
A07717	Acute Toxicity
A07718	Acute Toxicity, Multiple Dose Effects
A07719	Acute Toxicity
A07720	Acute Toxicity
A07721	Acute Toxicity, Mutagenicity, Reproductive Effects, Multiple Dose Effects
A07722	Mutagenicity, Tumorigenicity
A07723	Acute Toxicity, Multiple Dose Effects
A07724	Mutagenicity
A07725	Tumorigenicity, Skin and Eye Irritation
A07726	Acute Toxicity, Mutagenicity
A07727	Acute Toxicity, Skin and Eye Irritation
A07728	Acute Toxicity
A07729	Non-toxicity
A07730	Acute Toxicity
A07731	Acute Toxicity
A07732	Acute Toxicity, Mutagenicity, Tumorigenicity, Reproductive Effects,
A07733	Mutagenicity, Tumorigenicity
A07734	Mutagenicity, Tumorigenicity
A07735	Acute Toxicity, Mutagenicity, Tumorigenicity
A07736	Acute Toxicity, Mutagenicity, Tumorigenicity, Reproductive Effects,
A07737	Acute Toxicity
A07738	Acute Toxicity
A07739	Acute Toxicity
A07740	Acute Toxicity, Tumorigenicity, Reproductive Effects, Multiple Dose Effects
A07741	Acute Toxicity
A07742	Acute Toxicity
A07743	Mutagenicity
A07744	Acute Toxicity, Multiple Dose Effects

A07745	Acute Toxicity
A07746	Acute Toxicity, Mutagenicity, Reproductive Effects, Multiple Dose Effects
A07747	Acute Toxicity
A07748	Skin and Eye Irritation
A07749	Reproductive Effects
A07750	Acute Toxicity
A07751	Acute Toxicity
A07752	Mutagenicity
A07753	Acute Toxicity
A07754	Acute Toxicity, Mutagenicity, Reproductive Effects, Multiple Dose Effects
A07755	Acute Toxicity
A07756	Acute Toxicity, Mutagenicity, Multiple Dose Effects
A07757	Acute Toxicity, Tumorigenicity, Skin and Eye Irritation, Reproductive Effects, Multiple Dose Effects
A07758	Mutagenicity
A07759	Acute Toxicity
A07760	Acute Toxicity
A07761	Acute Toxicity
A07762	Acute Toxicity, Mutagenicity, Reproductive Effects
A07763	Acute Toxicity, Multiple Dose Effects
A07764	Mutagenicity, Tumorigenicity
A07765	Mutagenicity
A07766	Acute Toxicity, Mutagenicity, Tumorigenicity, Skin and Eye Irritation,
A07767	Acute Toxicity
A07768	Acute Toxicity
A07769	Acute Toxicity
A07770	Acute Toxicity
A07771	Acute Toxicity, Reproductive Effects, Multiple Dose Effects
A07772	Mutagenicity, Tumorigenicity
A07773	Acute Toxicity, Reproductive Effects, Multiple Dose Effects
A07774	Mutagenicity, Tumorigenicity
A07775	Acute Toxicity
A07776	Acute Toxicity
A07777	Acute Toxicity
A07778	Acute Toxicity
A07779	Acute Toxicity, Mutagenicity
A07780	Mutagenicity
A07781	Acute Toxicity
A07782	Mutagenicity
A07783	Mutagenicity
A07784	Mutagenicity, Tumorigenicity
A07785	Mutagenicity
A07786	Acute Toxicity
A07787	Tumorigenicity
A07788	Acute Toxicity

A07789	Acute Toxicity, Mutagenicity, Skin and Eye Irritation, Reproductive Effects, Multiple Dose Effects
A07790	Mutagenicity, Tumorigenicity
A07791	Acute Toxicity
A07792	Acute Toxicity
A07793	Acute Toxicity
A07794	Acute Toxicity, Mutagenicity, Multiple Dose Effects
A07795	Acute Toxicity
A07796	Acute Toxicity, Multiple Dose Effects
A07797	Acute Toxicity
A07798	Acute Toxicity
A07799	Acute Toxicity
A07800	Acute Toxicity, Mutagenicity, Tumorigenicity, Skin and Eye Irritation,
A07801	Mutagenicity
A07802	Acute Toxicity, Multiple Dose Effects
A07803	Acute Toxicity, Multiple Dose Effects
A07804	Mutagenicity
A07805	Acute Toxicity, Mutagenicity, Tumorigenicity
A07806	Mutagenicity
A07807	Acute Toxicity
A07808	Acute Toxicity
A07809	Acute Toxicity, Multiple Dose Effects
A07810	Mutagenicity
A07811	Acute Toxicity, Multiple Dose Effects
A07812	Acute Toxicity, Multiple Dose Effects
A07813	Acute Toxicity
A07814	Acute Toxicity
A07815	Acute Toxicity, Mutagenicity, Tumorigenicity, Skin and Eye Irritation, Multiple Dose Effects
A07816	Acute Toxicity, Mutagenicity, Multiple Dose Effects
A07817	Mutagenicity, Tumorigenicity
A07818	Acute Toxicity
A07819	Multiple Dose Effects
A07820	Reproductive Effects, Multiple Dose Effects
A07821	Acute Toxicity, Reproductive Effects
A07822	Acute Toxicity
A07823	Acute Toxicity
A07824	Acute Toxicity, Mutagenicity
A07825	Acute Toxicity, Mutagenicity
A07826	Acute Toxicity, Reproductive Effects, Multiple Dose Effects
A07827	Acute Toxicity, Multiple Dose Effects
A07828	Mutagenicity
A07829	Mutagenicity, Tumorigenicity
A07830	Acute Toxicity
A07831	Acute Toxicity
A07832	Acute Toxicity

A07833	Acute Toxicity, Mutagenicity
A07834	Acute Toxicity
A07835	Acute Toxicity
A07836	Mutagenicity, Tumorigenicity
A07837	Mutagenicity, Tumorigenicity
A07838	Mutagenicity
A07839	Acute Toxicity
A07840	Acute Toxicity
A07841	Tumorigenicity
A07842	Acute Toxicity, Mutagenicity, Tumorigenicity
A07843	Mutagenicity
A07844	Acute Toxicity, Reproductive Effects, Multiple Dose Effects
A07845	Acute Toxicity
A07846	Acute Toxicity
A07847	Acute Toxicity, Reproductive Effects, Multiple Dose Effects
A07848	Acute Toxicity, Mutagenicity, Skin and Eye Irritation
A07849	Acute Toxicity
A07850	Acute Toxicity, Multiple Dose Effects
A07851	Acute Toxicity, Reproductive Effects, Multiple Dose Effects
A07852	Mutagenicity, Tumorigenicity
A07853	Acute Toxicity
A07854	Acute Toxicity
A07855	Acute Toxicity
A07856	Acute Toxicity, Reproductive Effects
A07857	Mutagenicity, Tumorigenicity
A07858	Tumorigenicity
A07859	Mutagenicity, Tumorigenicity
A07860	Mutagenicity, Tumorigenicity
A07861	Mutagenicity, Tumorigenicity
A07862	Mutagenicity, Tumorigenicity
A07863	Mutagenicity, Tumorigenicity
A07864	Mutagenicity, Tumorigenicity
A07865	Mutagenicity, Tumorigenicity
A07866	Acute Toxicity
A07867	Reproductive Effects
A07868	Mutagenicity
A07869	Acute Toxicity
A07870	Mutagenicity
A07871	Reproductive Effects, Multiple Dose Effects
A07872	Acute Toxicity
A07873	Acute Toxicity
A07874	Acute Toxicity
A07875	Acute Toxicity
A07876	Acute Toxicity
A07877	Mutagenicity
A07878	Acute Toxicity, Mutagenicity, Skin and Eye Irritation, Multiple Dose Effects

A07879	Acute Toxicity
A07880	Acute Toxicity, Reproductive Effects, Multiple Dose Effects
A07881	Acute Toxicity, Multiple Dose Effects
A07882	Acute Toxicity
A07883	Non-toxicity
A07884	Mutagenicity
A07885	Acute Toxicity, Multiple Dose Effects
A07886	Mutagenicity
A07887	Acute Toxicity, Mutagenicity, Reproductive Effects, Multiple Dose Effects
A07888	Acute Toxicity
A07889	Acute Toxicity, Reproductive Effects, Multiple Dose Effects
A07890	Acute Toxicity
A07891	Acute Toxicity
A07892	Mutagenicity
A07893	Non-toxicity
A07894	Acute Toxicity, Reproductive Effects, Multiple Dose Effects
A07895	Acute Toxicity, Tumorigenicity
A07896	Acute Toxicity
A07897	Acute Toxicity, Mutagenicity
A07898	Acute Toxicity, Reproductive Effects, Multiple Dose Effects
A07899	Reproductive Effects
A07900	Acute Toxicity, Mutagenicity, Multiple Dose Effects
A07901	Tumorigenicity
A07902	Acute Toxicity, Reproductive Effects
A07903	Acute Toxicity, Mutagenicity, Tumorigenicity, Reproductive Effects
A07904	Mutagenicity, Tumorigenicity
A07905	Mutagenicity, Tumorigenicity
A07906	Mutagenicity, Tumorigenicity
A07907	Mutagenicity, Tumorigenicity
A07908	Acute Toxicity
A07909	Mutagenicity, Tumorigenicity
A07910	Acute Toxicity, Mutagenicity, Tumorigenicity
A07911	Acute Toxicity
A07912	Acute Toxicity, Multiple Dose Effects
A07913	Acute Toxicity, Mutagenicity, Tumorigenicity, Reproductive Effects,
A07914	Acute Toxicity, Mutagenicity, Reproductive Effects, Multiple Dose Effects
A07915	Acute Toxicity
A07916	Acute Toxicity, Multiple Dose Effects
A07917	Tumorigenicity
A07918	Acute Toxicity
A07919	Acute Toxicity
A07920	Acute Toxicity, Reproductive Effects
A07921	Acute Toxicity
A07922	Acute Toxicity, Mutagenicity
A07923	Acute Toxicity
A07924	Acute Toxicity, Reproductive Effects

A07925	Acute Toxicity, Reproductive Effects, Multiple Dose Effects
A07926	Acute Toxicity
A07927	Mutagenicity
A07928	Acute Toxicity, Mutagenicity, Tumorigenicity, Multiple Dose Effects
A07929	Acute Toxicity
A07930	Acute Toxicity
A07931	Acute Toxicity, Reproductive Effects, Multiple Dose Effects
A07932	Acute Toxicity
A07933	Acute Toxicity, Mutagenicity, Reproductive Effects, Multiple Dose Effects
A07934	Acute Toxicity, Mutagenicity, Multiple Dose Effects
A07935	Acute Toxicity
A07936	Acute Toxicity
A07937	Mutagenicity
A07938	Mutagenicity
A07939	Mutagenicity
A07940	Acute Toxicity
A07941	Acute Toxicity, Skin and Eye Irritation
A07942	Acute Toxicity, Mutagenicity, Multiple Dose Effects
A07943	Mutagenicity
A07944	Acute Toxicity, Mutagenicity, Tumorigenicity, Multiple Dose Effects
A07945	Mutagenicity, Tumorigenicity
A07946	Acute Toxicity
A07947	Acute Toxicity
A07948	Acute Toxicity
A07949	Acute Toxicity
A07950	Acute Toxicity
A07951	Acute Toxicity, Reproductive Effects, Multiple Dose Effects
A07952	Skin and Eye Irritation
A07953	Mutagenicity
A07954	Acute Toxicity, Mutagenicity, Multiple Dose Effects
A07955	Skin and Eye Irritation, Multiple Dose Effects
A07956	Acute Toxicity
A07957	Acute Toxicity
A07958	Acute Toxicity, Mutagenicity, Tumorigenicity, Reproductive Effects,
A07959	Acute Toxicity
A07960	Acute Toxicity
A07961	Acute Toxicity, Skin and Eye Irritation
A07962	Reproductive Effects
A07963	Reproductive Effects
A07964	Acute Toxicity
A07965	Non-toxicity
A07966	Acute Toxicity
A07967	Acute Toxicity, Reproductive Effects, Multiple Dose Effects
A07968	Acute Toxicity, Reproductive Effects, Multiple Dose Effects
A07969	Mutagenicity, Tumorigenicity
A07970	Acute Toxicity, Tumorigenicity, Multiple Dose Effects

A07971	Acute Toxicity
A07972	Multiple Dose Effects
A07973	Acute Toxicity, Mutagenicity, Reproductive Effects, Multiple Dose Effects
A07974	Acute Toxicity
A07975	Acute Toxicity
A07976	Acute Toxicity, Tumorigenicity, Reproductive Effects, Multiple Dose Effects
A07977	Multiple Dose Effects
A07978	Mutagenicity
A07979	Acute Toxicity, Mutagenicity, Tumorigenicity
A07980	Acute Toxicity
A07981	Mutagenicity
A07982	Mutagenicity, Tumorigenicity
A07983	Acute Toxicity
A07984	Tumorigenicity
A07985	Acute Toxicity, Mutagenicity, Tumorigenicity, Multiple Dose Effects
A07986	Acute Toxicity
A07987	Mutagenicity
A07988	Acute Toxicity, Mutagenicity
A07989	Non-toxicity
A07990	Tumorigenicity
A07991	Acute Toxicity
A07992	Acute Toxicity
A07993	Acute Toxicity
A07994	Mutagenicity, Tumorigenicity
A07995	Mutagenicity, Tumorigenicity
A07996	Acute Toxicity, Mutagenicity, Skin and Eye Irritation
A07997	Acute Toxicity, Mutagenicity, Tumorigenicity
A07998	Acute Toxicity
A07999	Acute Toxicity
A08000	Acute Toxicity, Multiple Dose Effects
A08001	Reproductive Effects
A08002	Mutagenicity, Tumorigenicity
A08003	Mutagenicity
A08004	Mutagenicity
A08005	Mutagenicity, Tumorigenicity
A08006	Mutagenicity, Tumorigenicity
A08007	Mutagenicity, Tumorigenicity
A08008	Acute Toxicity, Tumorigenicity
A08009	Mutagenicity
A08010	Acute Toxicity, Multiple Dose Effects
A08011	Acute Toxicity, Reproductive Effects, Multiple Dose Effects
A08012	Acute Toxicity
A08013	Acute Toxicity, Multiple Dose Effects
A08014	Acute Toxicity
A08015	Acute Toxicity
A08016	Acute Toxicity

A08017	Acute Toxicity
A08018	Reproductive Effects
A08019	Acute Toxicity
A08020	Acute Toxicity
A08021	Acute Toxicity
A08022	Acute Toxicity, Skin and Eye Irritation, Reproductive Effects, Multiple Dose
A08023	Mutagenicity, Tumorigenicity
A08024	Acute Toxicity, Reproductive Effects
A08025	Mutagenicity
A08026	Acute Toxicity
A08027	Acute Toxicity
A08028	Acute Toxicity
A08029	Non-toxicity
A08030	Reproductive Effects, Multiple Dose Effects
A08031	Mutagenicity
A08032	Acute Toxicity
A08033	Mutagenicity
A08034	Mutagenicity
A08035	Mutagenicity
A08036	Tumorigenicity
A08037	Tumorigenicity
A08038	Mutagenicity
A08039	Mutagenicity, Tumorigenicity
A08040	Mutagenicity, Tumorigenicity
A08041	Mutagenicity, Tumorigenicity
A08042	Mutagenicity
A08043	Mutagenicity, Tumorigenicity
A08044	Mutagenicity, Tumorigenicity
A08045	Acute Toxicity, Multiple Dose Effects
A08046	Mutagenicity
A08047	Mutagenicity
A08048	Acute Toxicity, Mutagenicity, Tumorigenicity
A08049	Acute Toxicity
A08050	Acute Toxicity
A08051	Acute Toxicity
A08052	Acute Toxicity
A08053	Acute Toxicity
A08054	Acute Toxicity
A08055	Mutagenicity
A08056	Acute Toxicity
A08057	Acute Toxicity
A08058	Acute Toxicity
A08059	Acute Toxicity
A08060	Acute Toxicity
A08061	Acute Toxicity
A08062	Acute Toxicity, Mutagenicity

A08063	Acute Toxicity
A08064	Mutagenicity
A08065	Acute Toxicity
A08066	Acute Toxicity
A08067	Acute Toxicity
A08068	Acute Toxicity
A08069	Acute Toxicity, Mutagenicity
A08070	Acute Toxicity
A08071	Acute Toxicity
A08072	Acute Toxicity
A08073	Acute Toxicity
A08074	Acute Toxicity
A08075	Acute Toxicity
A08076	Acute Toxicity
A08077	Acute Toxicity
A08078	Acute Toxicity
A08079	Acute Toxicity
A08080	Acute Toxicity
A08081	Acute Toxicity
A08082	Acute Toxicity
A08083	Acute Toxicity
A08084	Acute Toxicity
A08085	Acute Toxicity
A08086	Acute Toxicity
A08087	Acute Toxicity
A08088	Acute Toxicity
A08089	Acute Toxicity, Mutagenicity
A08090	Acute Toxicity
A08091	Multiple Dose Effects
A08092	Acute Toxicity
A08093	Acute Toxicity, Mutagenicity, Tumorigenicity, Reproductive Effects,
A08094	Acute Toxicity
A08095	Acute Toxicity, Reproductive Effects
A08096	Acute Toxicity, Reproductive Effects
A08097	Acute Toxicity, Mutagenicity, Tumorigenicity, Multiple Dose Effects
A08098	Acute Toxicity, Multiple Dose Effects
A08099	Acute Toxicity, Mutagenicity, Reproductive Effects, Multiple Dose Effects
A08100	Acute Toxicity
A08101	Acute Toxicity
A08102	Acute Toxicity
A08103	Acute Toxicity, Multiple Dose Effects
A08104	Acute Toxicity
A08105	Mutagenicity
A08106	Mutagenicity
A08107	Mutagenicity, Tumorigenicity
A08108	Acute Toxicity

A08109	Acute Toxicity, Multiple Dose Effects
A08110	Acute Toxicity
A08111	Tumorigenicity, Skin and Eye Irritation
A08112	Mutagenicity
A08113	Acute Toxicity
A08114	Acute Toxicity, Tumorigenicity, Multiple Dose Effects
A08115	Mutagenicity, Tumorigenicity
A08116	Mutagenicity
A08117	Non-toxicity
A08118	Acute Toxicity, Reproductive Effects, Multiple Dose Effects
A08119	Acute Toxicity
A08120	Acute Toxicity
A08121	Acute Toxicity
A08122	Acute Toxicity
A08123	Acute Toxicity
A08124	Acute Toxicity
A08125	Mutagenicity
A08126	Acute Toxicity, Mutagenicity, Multiple Dose Effects
A08127	Acute Toxicity, Mutagenicity, Reproductive Effects, Multiple Dose Effects
A08128	Mutagenicity
A08129	Acute Toxicity, Mutagenicity, Tumorigenicity
A08130	Acute Toxicity, Mutagenicity, Tumorigenicity
A08131	Acute Toxicity
A08132	Acute Toxicity
A08133	Acute Toxicity, Reproductive Effects, Multiple Dose Effects
A08134	Mutagenicity, Tumorigenicity
A08135	Acute Toxicity, Multiple Dose Effects
A08136	Mutagenicity
A08137	Acute Toxicity, Mutagenicity
A08138	Acute Toxicity, Mutagenicity, Reproductive Effects, Multiple Dose Effects
A08139	Acute Toxicity
A08140	Mutagenicity, Tumorigenicity
A08141	Acute Toxicity
A08142	Mutagenicity, Tumorigenicity
A08143	Mutagenicity, Tumorigenicity
A08144	Acute Toxicity, Multiple Dose Effects
A08145	Acute Toxicity
A08146	Acute Toxicity
A08147	Acute Toxicity
A08148	Non-toxicity
A08149	Acute Toxicity
A08150	Acute Toxicity
A08151	Acute Toxicity
A08152	Acute Toxicity
A08153	Mutagenicity
A08154	Multiple Dose Effects

A08155	Acute Toxicity
A08156	Acute Toxicity
A08157	Mutagenicity, Tumorigenicity
A08158	Mutagenicity, Tumorigenicity
A08159	Acute Toxicity
A08160	Acute Toxicity
A08161	Acute Toxicity
A08162	Mutagenicity, Tumorigenicity
A08163	Mutagenicity
A08164	Acute Toxicity
A08165	Multiple Dose Effects
A08166	Tumorigenicity
A08167	Acute Toxicity, Mutagenicity, Tumorigenicity
A08168	Acute Toxicity
A08169	Acute Toxicity
A08170	Mutagenicity, Tumorigenicity
A08171	Mutagenicity, Tumorigenicity
A08172	Mutagenicity, Tumorigenicity
A08173	Acute Toxicity, Reproductive Effects
A08174	Mutagenicity
A08175	Mutagenicity
A08176	Mutagenicity
A08177	Mutagenicity, Reproductive Effects, Multiple Dose Effects
A08178	Non-toxicity
A08179	Mutagenicity
A08180	Acute Toxicity
A08181	Acute Toxicity
A08182	Acute Toxicity, Reproductive Effects
A08183	Non-toxicity
A08184	Mutagenicity
A08185	Acute Toxicity, Mutagenicity
A08186	Acute Toxicity, Mutagenicity, Tumorigenicity
A08187	Mutagenicity
A08188	Acute Toxicity
A08189	Acute Toxicity
A08190	Acute Toxicity, Multiple Dose Effects
A08191	Reproductive Effects
A08192	Acute Toxicity, Mutagenicity, Multiple Dose Effects
A08193	Acute Toxicity, Skin and Eye Irritation, Reproductive Effects, Multiple Dose
A08194	Non-toxicity
A08195	Acute Toxicity
A08196	Non-toxicity
A08197	Multiple Dose Effects
A08198	Acute Toxicity
A08199	Acute Toxicity, Skin and Eye Irritation, Reproductive Effects, Multiple Dose
A08200	Acute Toxicity

A08201	Acute Toxicity
A08202	Mutagenicity, Tumorigenicity
A08203	Mutagenicity, Tumorigenicity
A08204	Acute Toxicity
A08205	Acute Toxicity
A08206	Mutagenicity
A08207	Acute Toxicity
A08208	Acute Toxicity
A08209	Skin and Eye Irritation
A08210	Acute Toxicity
A08211	Acute Toxicity
A08212	Acute Toxicity, Mutagenicity, Reproductive Effects, Multiple Dose Effects
A08213	Acute Toxicity
A08214	Acute Toxicity
A08215	Acute Toxicity
A08216	Mutagenicity
A08217	Acute Toxicity
A08218	Acute Toxicity, Reproductive Effects, Multiple Dose Effects
A08219	Acute Toxicity, Reproductive Effects, Multiple Dose Effects
A08220	Mutagenicity, Tumorigenicity
A08221	Acute Toxicity
A08222	Acute Toxicity
A08223	Mutagenicity
A08224	Mutagenicity, Tumorigenicity
A08225	Mutagenicity
A08226	Mutagenicity
A08227	Mutagenicity
A08228	Acute Toxicity, Reproductive Effects
A08229	Mutagenicity
A08230	Mutagenicity
A08231	Mutagenicity
A08232	Mutagenicity
A08233	Acute Toxicity, Mutagenicity
A08234	Acute Toxicity
A08235	Acute Toxicity
A08236	Mutagenicity, Tumorigenicity
A08237	Mutagenicity
A08238	Acute Toxicity
A08239	Mutagenicity
A08240	Mutagenicity
A08241	Mutagenicity
A08242	Acute Toxicity, Reproductive Effects, Multiple Dose Effects
A08243	Acute Toxicity
A08244	Mutagenicity, Tumorigenicity
A08245	Acute Toxicity
A08246	Acute Toxicity

A08247	Mutagenicity
A08248	Acute Toxicity
A08249	Acute Toxicity, Mutagenicity, Reproductive Effects, Multiple Dose Effects
A08250	Acute Toxicity
A08251	Acute Toxicity
A08252	Acute Toxicity, Multiple Dose Effects
A08253	Mutagenicity
A08254	Mutagenicity
A08255	Reproductive Effects, Multiple Dose Effects
A08256	Acute Toxicity, Mutagenicity, Tumorigenicity
A08257	Mutagenicity
A08258	Acute Toxicity
A08259	Multiple Dose Effects
A08260	Acute Toxicity
A08261	Acute Toxicity
A08262	Acute Toxicity, Multiple Dose Effects
A08263	Acute Toxicity
A08264	Acute Toxicity
A08265	Acute Toxicity
A08266	Mutagenicity, Tumorigenicity
A08267	Acute Toxicity
A08268	Tumorigenicity
A08269	Acute Toxicity, Multiple Dose Effects
A08270	Mutagenicity
A08271	Mutagenicity
A08272	Non-toxicity
A08273	Acute Toxicity
A08274	Acute Toxicity, Mutagenicity
A08275	Acute Toxicity
A08276	Mutagenicity
A08277	Acute Toxicity, Reproductive Effects, Multiple Dose Effects
A08278	Acute Toxicity, Skin and Eye Irritation
A08279	Acute Toxicity
A08280	Acute Toxicity, Reproductive Effects, Multiple Dose Effects
A08281	Acute Toxicity
A08282	Acute Toxicity, Mutagenicity
A08283	Acute Toxicity, Reproductive Effects
A08284	Acute Toxicity, Skin and Eye Irritation
A08285	Tumorigenicity
A08286	Mutagenicity, Tumorigenicity
A08287	Acute Toxicity, Mutagenicity, Reproductive Effects, Multiple Dose Effects
A08288	Mutagenicity
A08289	Acute Toxicity, Mutagenicity, Skin and Eye Irritation, Multiple Dose Effects
A08290	Acute Toxicity
A08291	Mutagenicity
A08292	Mutagenicity, Tumorigenicity

A08293	Acute Toxicity, Mutagenicity, Tumorigenicity, Reproductive Effects,
A08294	Mutagenicity, Tumorigenicity
A08295	Acute Toxicity
A08296	Acute Toxicity, Mutagenicity, Skin and Eye Irritation
A08297	Acute Toxicity
A08298	Mutagenicity
A08299	Mutagenicity
A08300	Multiple Dose Effects
A08301	Acute Toxicity
A08302	Mutagenicity, Tumorigenicity
A08303	Mutagenicity, Tumorigenicity
A08304	Acute Toxicity, Multiple Dose Effects
A08305	Mutagenicity
A08306	Mutagenicity, Tumorigenicity
A08307	Mutagenicity
A08308	Mutagenicity
A08309	Mutagenicity, Tumorigenicity, Reproductive Effects, Multiple Dose Effects
A08310	Mutagenicity
A08311	Mutagenicity
A08312	Acute Toxicity
A08313	Acute Toxicity, Multiple Dose Effects
A08314	Mutagenicity
A08315	Acute Toxicity
A08316	Acute Toxicity
A08317	Acute Toxicity
A08318	Acute Toxicity
A08319	Acute Toxicity, Multiple Dose Effects
A08320	Mutagenicity
A08321	Tumorigenicity
A08322	Mutagenicity
A08323	Acute Toxicity, Mutagenicity, Tumorigenicity, Multiple Dose Effects
A08324	Acute Toxicity, Multiple Dose Effects
A08325	Tumorigenicity
A08326	Acute Toxicity
A08327	Acute Toxicity, Multiple Dose Effects
A08328	Acute Toxicity, Mutagenicity, Tumorigenicity
A08329	Acute Toxicity, Tumorigenicity
A08330	Acute Toxicity, Multiple Dose Effects
A08331	Acute Toxicity
A08332	Acute Toxicity
A08333	Acute Toxicity
A08334	Acute Toxicity
A08335	Acute Toxicity
A08336	Mutagenicity
A08337	Acute Toxicity
A08338	Acute Toxicity

A08339	Acute Toxicity
A08340	Acute Toxicity
A08341	Acute Toxicity
A08342	Mutagenicity
A08343	Acute Toxicity, Reproductive Effects, Multiple Dose Effects
A08344	Acute Toxicity
A08345	Acute Toxicity
A08346	Acute Toxicity, Skin and Eye Irritation
A08347	Acute Toxicity
A08348	Tumorigenicity
A08349	Acute Toxicity
A08350	Mutagenicity
A08351	Acute Toxicity
A08352	Acute Toxicity, Mutagenicity, Multiple Dose Effects
A08353	Acute Toxicity
A08354	Acute Toxicity
A08355	Multiple Dose Effects
A08356	Acute Toxicity, Tumorigenicity, Skin and Eye Irritation, Reproductive Effects, Multiple Dose Effects
A08357	Acute Toxicity, Multiple Dose Effects
A08358	Acute Toxicity
A08359	Acute Toxicity, Multiple Dose Effects
A08360	Non-toxicity
A08361	Mutagenicity
A08362	Tumorigenicity, Multiple Dose Effects
A08363	Acute Toxicity
A08364	Acute Toxicity
A08365	Acute Toxicity, Mutagenicity
A08366	Acute Toxicity
A08367	Acute Toxicity, Multiple Dose Effects
A08368	Acute Toxicity
A08369	Acute Toxicity, Reproductive Effects, Multiple Dose Effects
A08370	Acute Toxicity, Mutagenicity, Multiple Dose Effects
A08371	Acute Toxicity
A08372	Acute Toxicity, Reproductive Effects
A08373	Mutagenicity
A08374	Acute Toxicity, Mutagenicity, Tumorigenicity
A08375	Acute Toxicity
A08376	Acute Toxicity
A08377	Acute Toxicity, Multiple Dose Effects
A08378	Acute Toxicity
A08379	Acute Toxicity, Mutagenicity, Reproductive Effects, Multiple Dose Effects
A08380	Mutagenicity
A08381	Acute Toxicity, Multiple Dose Effects
A08382	Acute Toxicity, Reproductive Effects, Multiple Dose Effects
A08383	Acute Toxicity

A08384	Mutagenicity
A08385	Acute Toxicity, Reproductive Effects, Multiple Dose Effects
A08386	Mutagenicity
A08387	Acute Toxicity, Mutagenicity, Tumorigenicity
A08388	Reproductive Effects
A08389	Mutagenicity, Tumorigenicity, Reproductive Effects
A08390	Acute Toxicity
A08391	Acute Toxicity, Mutagenicity
A08392	Mutagenicity
A08393	Mutagenicity
A08394	Acute Toxicity
A08395	Acute Toxicity
A08396	Acute Toxicity
A08397	Mutagenicity, Tumorigenicity
A08398	Acute Toxicity
A08399	Mutagenicity
A08400	Acute Toxicity, Reproductive Effects, Multiple Dose Effects
A08401	Mutagenicity
A08402	Non-toxicity
A08403	Acute Toxicity
A08404	Mutagenicity
A08405	Mutagenicity
A08406	Mutagenicity
A08407	Mutagenicity
A08408	Acute Toxicity
A08409	Acute Toxicity, Mutagenicity
A08410	Acute Toxicity, Multiple Dose Effects
A08411	Acute Toxicity
A08412	Mutagenicity, Tumorigenicity
A08413	Acute Toxicity
A08414	Acute Toxicity
A08415	Mutagenicity
A08416	Mutagenicity
A08417	Acute Toxicity
A08418	Acute Toxicity, Mutagenicity
A08419	Acute Toxicity
A08420	Acute Toxicity
A08421	Acute Toxicity
A08422	Acute Toxicity
A08423	Acute Toxicity
A08424	Acute Toxicity
A08425	Mutagenicity
A08426	Mutagenicity, Tumorigenicity, Multiple Dose Effects
A08427	Mutagenicity
A08428	Mutagenicity
A08429	Mutagenicity

A08430	Multiple Dose Effects
A08431	Acute Toxicity
A08432	Acute Toxicity
A08433	Acute Toxicity
A08434	Acute Toxicity
A08435	Acute Toxicity, Multiple Dose Effects
A08436	Mutagenicity
A08437	Acute Toxicity, Multiple Dose Effects
A08438	Acute Toxicity
A08439	Acute Toxicity, Multiple Dose Effects
A08440	Mutagenicity
A08441	Acute Toxicity
A08442	Acute Toxicity
A08443	Acute Toxicity
A08444	Acute Toxicity
A08445	Acute Toxicity
A08446	Acute Toxicity
A08447	Multiple Dose Effects
A08448	Mutagenicity, Tumorigenicity
A08449	Mutagenicity
A08450	Acute Toxicity
A08451	Mutagenicity
A08452	Acute Toxicity, Multiple Dose Effects
A08453	Mutagenicity
A08454	Mutagenicity
A08455	Mutagenicity
A08456	Acute Toxicity
A08457	Acute Toxicity, Reproductive Effects, Multiple Dose Effects
A08458	Mutagenicity
A08459	Mutagenicity
A08460	Acute Toxicity
A08461	Acute Toxicity, Tumorigenicity, Reproductive Effects, Multiple Dose Effects
A08462	Acute Toxicity
A08463	Skin and Eye Irritation, Multiple Dose Effects
A08464	Acute Toxicity, Mutagenicity
A08465	Acute Toxicity, Multiple Dose Effects
A08466	Non-toxicity
A08467	Non-toxicity
A08468	Mutagenicity
A08469	Mutagenicity
A08470	Acute Toxicity
A08471	Acute Toxicity
A08472	Acute Toxicity
A08473	Acute Toxicity
A08474	Mutagenicity
A08475	Mutagenicity

A08476	Acute Toxicity, Reproductive Effects
A08477	Acute Toxicity, Multiple Dose Effects
A08478	Acute Toxicity
A08479	Mutagenicity
A08480	Acute Toxicity
A08481	Acute Toxicity
A08482	Acute Toxicity
A08483	Acute Toxicity
A08484	Tumorigenicity, Multiple Dose Effects
A08485	Acute Toxicity
A08486	Mutagenicity
A08487	Mutagenicity
A08488	Acute Toxicity
A08489	Acute Toxicity
A08490	Multiple Dose Effects
A08491	Reproductive Effects
A08492	Acute Toxicity
A08493	Acute Toxicity
A08494	Multiple Dose Effects
A08495	Acute Toxicity
A08496	Mutagenicity, Tumorigenicity
A08497	Mutagenicity, Tumorigenicity
A08498	Acute Toxicity
A08499	Mutagenicity
A08500	Mutagenicity
A08501	Acute Toxicity, Multiple Dose Effects
A08502	Non-toxicity
A08503	Acute Toxicity, Mutagenicity, Multiple Dose Effects
A08504	Mutagenicity
A08505	Acute Toxicity, Mutagenicity, Multiple Dose Effects
A08506	Reproductive Effects, Multiple Dose Effects
A08507	Acute Toxicity, Multiple Dose Effects
A08508	Mutagenicity
A08509	Mutagenicity
A08510	Mutagenicity
A08511	Acute Toxicity
A08512	Acute Toxicity
A08513	Acute Toxicity
A08514	Acute Toxicity, Reproductive Effects
A08515	Acute Toxicity
A08516	Acute Toxicity, Reproductive Effects, Multiple Dose Effects
A08517	Acute Toxicity, Multiple Dose Effects
A08518	Acute Toxicity, Reproductive Effects, Multiple Dose Effects
A08519	Acute Toxicity, Multiple Dose Effects
A08520	Mutagenicity
A08521	Acute Toxicity, Reproductive Effects, Multiple Dose Effects

A08522	Acute Toxicity, Multiple Dose Effects
A08523	Mutagenicity
A08524	Acute Toxicity, Multiple Dose Effects
A08525	Skin and Eye Irritation, Multiple Dose Effects
A08526	Acute Toxicity, Mutagenicity
A08527	Acute Toxicity, Reproductive Effects, Multiple Dose Effects
A08528	Acute Toxicity
A08529	Acute Toxicity, Mutagenicity, Skin and Eye Irritation
A08530	Acute Toxicity
A08531	Multiple Dose Effects
A08532	Acute Toxicity, Multiple Dose Effects
A08533	Acute Toxicity
A08534	Acute Toxicity
A08535	Acute Toxicity
A08536	Acute Toxicity, Reproductive Effects, Multiple Dose Effects
A08537	Acute Toxicity
A08538	Mutagenicity
A08539	Acute Toxicity, Multiple Dose Effects
A08540	Non-toxicity
A08541	Acute Toxicity, Multiple Dose Effects
A08542	Mutagenicity
A08543	Acute Toxicity, Mutagenicity, Multiple Dose Effects
A08544	Tumorigenicity
A08545	Multiple Dose Effects
A08546	Acute Toxicity
A08547	Acute Toxicity
A08548	Acute Toxicity, Mutagenicity
A08549	Mutagenicity, Reproductive Effects, Multiple Dose Effects
A08550	Acute Toxicity, Multiple Dose Effects
A08551	Acute Toxicity, Multiple Dose Effects
A08552	Non-toxicity
A08553	Non-toxicity
A08554	Acute Toxicity
A08555	Acute Toxicity, Multiple Dose Effects
A08556	Acute Toxicity, Multiple Dose Effects
A08557	Non-toxicity
A08558	Acute Toxicity
A08559	Acute Toxicity
A08560	Multiple Dose Effects
A08561	Multiple Dose Effects
A08562	Acute Toxicity
A08563	Acute Toxicity
A08564	Non-toxicity
A08565	Non-toxicity
A08566	Mutagenicity, Multiple Dose Effects
A08567	Acute Toxicity, Multiple Dose Effects

A08568	Multiple Dose Effects
A08569	Acute Toxicity, Multiple Dose Effects
A08570	Multiple Dose Effects
A08571	Acute Toxicity, Mutagenicity, Reproductive Effects, Multiple Dose Effects
A08572	Acute Toxicity, Multiple Dose Effects
A08573	Acute Toxicity, Mutagenicity, Tumorigenicity, Reproductive Effects,
A08574	Acute Toxicity
A08575	Skin and Eye Irritation
A08576	Acute Toxicity
A08577	Acute Toxicity
A08578	Acute Toxicity, Mutagenicity, Tumorigenicity
A08579	Acute Toxicity, Multiple Dose Effects
A08580	Acute Toxicity
A08581	Acute Toxicity, Skin and Eye Irritation, Multiple Dose Effects
A08582	Acute Toxicity, Mutagenicity, Skin and Eye Irritation
A08583	Acute Toxicity
A08584	Acute Toxicity, Mutagenicity, Skin and Eye Irritation
A08585	Acute Toxicity, Reproductive Effects, Multiple Dose Effects
A08586	Acute Toxicity, Skin and Eye Irritation
A08587	Acute Toxicity, Skin and Eye Irritation, Reproductive Effects, Multiple Dose
A08588	Acute Toxicity, Mutagenicity, Reproductive Effects
A08589	Acute Toxicity
A08590	Acute Toxicity
A08591	Acute Toxicity
A08592	Skin and Eye Irritation
A08593	Acute Toxicity, Skin and Eye Irritation
A08594	Acute Toxicity
A08595	Acute Toxicity
A08596	Acute Toxicity, Multiple Dose Effects
A08597	Acute Toxicity, Skin and Eye Irritation
A08598	Acute Toxicity
A08599	Acute Toxicity
A08600	Acute Toxicity
A08601	Acute Toxicity
A08602	Acute Toxicity
A08603	Acute Toxicity, Multiple Dose Effects
A08604	Acute Toxicity, Mutagenicity, Multiple Dose Effects
A08605	Acute Toxicity
A08606	Acute Toxicity
A08607	Acute Toxicity, Mutagenicity
A08608	Acute Toxicity, Multiple Dose Effects
A08609	Acute Toxicity
A08610	Mutagenicity
A08611	Acute Toxicity
A08612	Acute Toxicity
A08613	Acute Toxicity

A08614	Acute Toxicity, Mutagenicity, Skin and Eye Irritation
A08615	Acute Toxicity, Skin and Eye Irritation
A08616	Acute Toxicity
A08617	Acute Toxicity
A08618	Skin and Eye Irritation
A08619	Mutagenicity, Tumorigenicity
A08620	Acute Toxicity, Mutagenicity, Tumorigenicity
A08621	Acute Toxicity
A08622	Acute Toxicity, Skin and Eye Irritation
A08623	Acute Toxicity
A08624	Acute Toxicity, Skin and Eye Irritation
A08625	Acute Toxicity
A08626	Acute Toxicity
A08627	Acute Toxicity, Mutagenicity, Skin and Eye Irritation, Reproductive Effects, Multiple Dose Effects
A08628	Acute Toxicity, Mutagenicity, Skin and Eye Irritation
A08629	Acute Toxicity
A08630	Acute Toxicity, Skin and Eye Irritation, Multiple Dose Effects
A08631	Skin and Eye Irritation
A08632	Acute Toxicity
A08633	Acute Toxicity
A08634	Acute Toxicity
A08635	Acute Toxicity
A08636	Acute Toxicity, Mutagenicity, Multiple Dose Effects
A08637	Acute Toxicity
A08638	Acute Toxicity, Mutagenicity, Tumorigenicity, Skin and Eye Irritation
A08639	Acute Toxicity
A08640	Acute Toxicity
A08641	Acute Toxicity, Tumorigenicity, Skin and Eye Irritation
A08642	Acute Toxicity, Skin and Eye Irritation
A08643	Acute Toxicity, Multiple Dose Effects
A08644	Acute Toxicity, Skin and Eye Irritation
A08645	Acute Toxicity
A08646	Acute Toxicity, Skin and Eye Irritation
A08647	Mutagenicity
A08648	Acute Toxicity, Skin and Eye Irritation
A08649	Tumorigenicity
A08650	Acute Toxicity, Multiple Dose Effects
A08651	Acute Toxicity, Skin and Eye Irritation
A08652	Acute Toxicity
A08653	Acute Toxicity
A08654	Skin and Eye Irritation
A08655	Acute Toxicity
A08656	Acute Toxicity
A08657	Acute Toxicity, Multiple Dose Effects
A08658	Tumorigenicity, Multiple Dose Effects

A08659	Acute Toxicity
A08660	Acute Toxicity, Skin and Eye Irritation, Reproductive Effects, Multiple Dose
A08661	Acute Toxicity, Skin and Eye Irritation
A08662	Acute Toxicity
A08663	Acute Toxicity, Reproductive Effects, Multiple Dose Effects
A08664	Acute Toxicity
A08665	Multiple Dose Effects
A08666	Acute Toxicity
A08667	Acute Toxicity, Skin and Eye Irritation, Reproductive Effects, Multiple Dose
A08668	Mutagenicity, Tumorigenicity
A08669	Acute Toxicity, Skin and Eye Irritation, Reproductive Effects, Multiple Dose
A08670	Tumorigenicity
A08671	Mutagenicity, Tumorigenicity
A08672	Acute Toxicity, Skin and Eye Irritation
A08673	Acute Toxicity
A08674	Acute Toxicity
A08675	Mutagenicity, Tumorigenicity, Multiple Dose Effects
A08676	Mutagenicity, Tumorigenicity
A08677	Acute Toxicity, Mutagenicity
A08678	Acute Toxicity, Multiple Dose Effects
A08679	Mutagenicity, Tumorigenicity
A08680	Acute Toxicity, Skin and Eye Irritation
A08681	Mutagenicity, Tumorigenicity
A08682	Acute Toxicity, Mutagenicity, Reproductive Effects
A08683	Acute Toxicity, Mutagenicity
A08684	Acute Toxicity
A08685	Mutagenicity, Tumorigenicity
A08686	Acute Toxicity, Mutagenicity, Tumorigenicity, Multiple Dose Effects
A08687	Acute Toxicity, Reproductive Effects, Multiple Dose Effects
A08688	Acute Toxicity, Mutagenicity, Skin and Eye Irritation, Multiple Dose Effects
A08689	Acute Toxicity
A08690	Acute Toxicity, Tumorigenicity, Multiple Dose Effects
A08691	Acute Toxicity, Skin and Eye Irritation
A08692	Acute Toxicity
A08693	Acute Toxicity, Mutagenicity, Tumorigenicity, Skin and Eye Irritation,
A08694	Acute Toxicity, Skin and Eye Irritation
A08695	Acute Toxicity
A08696	Acute Toxicity
A08697	Acute Toxicity, Mutagenicity, Skin and Eye Irritation, Multiple Dose Effects
A08698	Acute Toxicity, Mutagenicity, Tumorigenicity
A08699	Acute Toxicity, Mutagenicity, Multiple Dose Effects
A08700	Acute Toxicity, Multiple Dose Effects
A08701	Non-toxicity
A08702	Acute Toxicity, Skin and Eye Irritation
A08703	Acute Toxicity, Mutagenicity, Tumorigenicity, Reproductive Effects,
A08704	Acute Toxicity, Skin and Eye Irritation

A08705	Acute Toxicity, Skin and Eye Irritation
A08706	Acute Toxicity
A08707	Acute Toxicity, Mutagenicity, Multiple Dose Effects
A08708	Tumorigenicity
A08709	Acute Toxicity, Tumorigenicity, Skin and Eye Irritation, Multiple Dose
A08710	Acute Toxicity, Mutagenicity, Skin and Eye Irritation, Multiple Dose Effects
A08711	Tumorigenicity
A08712	Skin and Eye Irritation
A08713	Skin and Eye Irritation
A08714	Acute Toxicity, Skin and Eye Irritation, Reproductive Effects, Multiple Dose
A08715	Acute Toxicity, Skin and Eye Irritation
A08716	Mutagenicity
A08717	Mutagenicity, Skin and Eye Irritation
A08718	Mutagenicity
A08719	Acute Toxicity, Mutagenicity
A08720	Mutagenicity, Tumorigenicity
A08721	Acute Toxicity, Skin and Eye Irritation
A08722	Acute Toxicity
A08723	Acute Toxicity
A08724	Acute Toxicity
A08725	Acute Toxicity, Multiple Dose Effects
A08726	Acute Toxicity
A08727	Acute Toxicity
A08728	Skin and Eye Irritation
A08729	Mutagenicity
A08730	Acute Toxicity, Mutagenicity, Reproductive Effects, Multiple Dose Effects
A08731	Acute Toxicity, Multiple Dose Effects
A08732	Acute Toxicity, Reproductive Effects, Multiple Dose Effects
A08733	Acute Toxicity, Mutagenicity
A08734	Acute Toxicity
A08735	Acute Toxicity
A08736	Acute Toxicity
A08737	Mutagenicity
A08738	Acute Toxicity
A08739	Acute Toxicity, Skin and Eye Irritation
A08740	Acute Toxicity
A08741	Acute Toxicity, Mutagenicity, Multiple Dose Effects
A08742	Mutagenicity
A08743	Acute Toxicity
A08744	Mutagenicity
A08745	Acute Toxicity
A08746	Mutagenicity
A08747	Mutagenicity
A08748	Acute Toxicity
A08749	Mutagenicity
A08750	Mutagenicity, Tumorigenicity

A08751	Acute Toxicity, Multiple Dose Effects
A08752	Acute Toxicity, Mutagenicity
A08753	Mutagenicity, Tumorigenicity, Multiple Dose Effects
A08754	Acute Toxicity, Skin and Eye Irritation, Multiple Dose Effects
A08755	Acute Toxicity, Multiple Dose Effects
A08756	Acute Toxicity, Reproductive Effects, Multiple Dose Effects
A08757	Acute Toxicity, Skin and Eye Irritation
A08758	Acute Toxicity
A08759	Acute Toxicity, Skin and Eye Irritation
A08760	Acute Toxicity, Skin and Eye Irritation
A08761	Multiple Dose Effects
A08762	Acute Toxicity
A08763	Multiple Dose Effects
A08764	Tumorigenicity
A08765	Acute Toxicity, Mutagenicity, Tumorigenicity, Reproductive Effects,
A08766	Acute Toxicity, Reproductive Effects, Multiple Dose Effects
A08767	Acute Toxicity
A08768	Acute Toxicity
A08769	Non-toxicity
A08770	Skin and Eye Irritation
A08771	Acute Toxicity
A08772	Acute Toxicity, Tumorigenicity, Reproductive Effects, Multiple Dose Effects
A08773	Acute Toxicity, Reproductive Effects, Multiple Dose Effects
A08774	Acute Toxicity, Multiple Dose Effects
A08775	Acute Toxicity, Skin and Eye Irritation, Reproductive Effects, Multiple Dose
A08776	Mutagenicity, Tumorigenicity, Reproductive Effects
A08777	Acute Toxicity, Tumorigenicity, Reproductive Effects, Multiple Dose Effects
A08778	Acute Toxicity
A08779	Acute Toxicity
A08780	Acute Toxicity, Reproductive Effects, Multiple Dose Effects
A08781	Acute Toxicity, Skin and Eye Irritation
A08782	Acute Toxicity, Reproductive Effects, Multiple Dose Effects
A08783	Acute Toxicity
A08784	Acute Toxicity
A08785	Acute Toxicity, Mutagenicity, Tumorigenicity, Reproductive Effects,
A08786	Mutagenicity, Multiple Dose Effects
A08787	Acute Toxicity
A08788	Acute Toxicity
A08789	Acute Toxicity
A08790	Acute Toxicity
A08791	Acute Toxicity
A08792	Acute Toxicity
A08793	Acute Toxicity
A08794	Acute Toxicity
A08795	Acute Toxicity
A08796	Acute Toxicity

A08797	Acute Toxicity
A08798	Acute Toxicity
A08799	Acute Toxicity
A08800	Acute Toxicity
A08801	Acute Toxicity
A08802	Acute Toxicity
A08803	Acute Toxicity
A08804	Acute Toxicity
A08805	Acute Toxicity
A08806	Acute Toxicity
A08807	Acute Toxicity
A08808	Acute Toxicity
A08809	Acute Toxicity
A08810	Acute Toxicity, Reproductive Effects
A08811	Acute Toxicity
A08812	Acute Toxicity, Multiple Dose Effects
A08813	Acute Toxicity, Tumorigenicity, Multiple Dose Effects
A08814	Non-toxicity
A08815	Acute Toxicity
A08816	Acute Toxicity
A08817	Acute Toxicity, Multiple Dose Effects
A08818	Acute Toxicity
A08819	Mutagenicity
A08820	Acute Toxicity, Skin and Eye Irritation, Multiple Dose Effects
A08821	Acute Toxicity, Tumorigenicity
A08822	Acute Toxicity
A08823	Acute Toxicity
A08824	Acute Toxicity, Mutagenicity
A08825	Acute Toxicity
A08826	Acute Toxicity
A08827	Acute Toxicity
A08828	Acute Toxicity
A08829	Acute Toxicity, Multiple Dose Effects
A08830	Acute Toxicity
A08831	Acute Toxicity, Mutagenicity
A08832	Acute Toxicity
A08833	Acute Toxicity, Reproductive Effects, Multiple Dose Effects
A08834	Acute Toxicity, Tumorigenicity, Multiple Dose Effects
A08835	Acute Toxicity, Mutagenicity, Tumorigenicity, Reproductive Effects,
A08836	Acute Toxicity
A08837	Acute Toxicity
A08838	Mutagenicity
A08839	Mutagenicity
A08840	Acute Toxicity, Tumorigenicity, Multiple Dose Effects
A08841	Acute Toxicity, Multiple Dose Effects
A08842	Acute Toxicity, Multiple Dose Effects

A08843	Acute Toxicity
A08844	Acute Toxicity, Multiple Dose Effects
A08845	Acute Toxicity
A08846	Acute Toxicity
A08847	Multiple Dose Effects
A08848	Acute Toxicity
A08849	Mutagenicity
A08850	Acute Toxicity, Mutagenicity, Multiple Dose Effects
A08851	Acute Toxicity
A08852	Mutagenicity
A08853	Acute Toxicity, Mutagenicity
A08854	Mutagenicity
A08855	Mutagenicity
A08856	Acute Toxicity
A08857	Skin and Eye Irritation, Multiple Dose Effects
A08858	Mutagenicity
A08859	Acute Toxicity, Mutagenicity, Tumorigenicity, Skin and Eye Irritation,
A08860	Acute Toxicity, Multiple Dose Effects
A08861	Acute Toxicity, Mutagenicity
A08862	Non-toxicity
A08863	Tumorigenicity
A08864	Acute Toxicity
A08865	Acute Toxicity, Multiple Dose Effects
A08866	Acute Toxicity
A08867	Skin and Eye Irritation, Multiple Dose Effects
A08868	Acute Toxicity, Tumorigenicity, Multiple Dose Effects
A08869	Mutagenicity
A08870	Acute Toxicity
A08871	Acute Toxicity, Multiple Dose Effects
A08872	Non-toxicity
A08873	Mutagenicity, Skin and Eye Irritation
A08874	Skin and Eye Irritation, Reproductive Effects, Multiple Dose Effects
A08875	Mutagenicity
A08876	Acute Toxicity
A08877	Acute Toxicity
A08878	Acute Toxicity, Mutagenicity
A08879	Acute Toxicity, Mutagenicity, Skin and Eye Irritation, Multiple Dose Effects
A08880	Acute Toxicity
A08881	Mutagenicity, Tumorigenicity, Multiple Dose Effects
A08882	Acute Toxicity
A08883	Acute Toxicity, Mutagenicity
A08884	Mutagenicity
A08885	Mutagenicity
A08886	Acute Toxicity
A08887	Acute Toxicity
A08888	Acute Toxicity

A08889	Acute Toxicity, Mutagenicity, Tumorigenicity, Reproductive Effects,
A08890	Acute Toxicity
A08891	Acute Toxicity
A08892	Acute Toxicity, Mutagenicity, Tumorigenicity, Reproductive Effects,
A08893	Acute Toxicity
A08894	Acute Toxicity, Mutagenicity
A08895	Acute Toxicity
A08896	Acute Toxicity
A08897	Acute Toxicity
A08898	Acute Toxicity, Mutagenicity, Skin and Eye Irritation
A08899	Non-toxicity
A08900	Acute Toxicity, Multiple Dose Effects
A08901	Acute Toxicity
A08902	Acute Toxicity
A08903	Acute Toxicity
A08904	Acute Toxicity
A08905	Mutagenicity
A08906	Acute Toxicity
A08907	Acute Toxicity
A08908	Acute Toxicity, Skin and Eye Irritation
A08909	Multiple Dose Effects
A08910	Skin and Eye Irritation, Multiple Dose Effects
A08911	Acute Toxicity
A08912	Acute Toxicity
A08913	Acute Toxicity
A08914	Acute Toxicity
A08915	Acute Toxicity
A08916	Acute Toxicity
A08917	Mutagenicity
A08918	Acute Toxicity, Multiple Dose Effects
A08919	Acute Toxicity, Multiple Dose Effects
A08920	Acute Toxicity
A08921	Acute Toxicity
A08922	Acute Toxicity
A08923	Acute Toxicity, Multiple Dose Effects
A08924	Acute Toxicity
A08925	Acute Toxicity, Mutagenicity, Reproductive Effects, Multiple Dose Effects
A08926	Multiple Dose Effects
A08927	Acute Toxicity, Skin and Eye Irritation
A08928	Acute Toxicity
A08929	Acute Toxicity
A08930	Acute Toxicity, Mutagenicity
A08931	Acute Toxicity
A08932	Acute Toxicity
A08933	Acute Toxicity
A08934	Acute Toxicity, Mutagenicity

A08935	Acute Toxicity
A08936	Acute Toxicity
A08937	Acute Toxicity, Reproductive Effects
A08938	Acute Toxicity
A08939	Acute Toxicity
A08940	Acute Toxicity
A08941	Acute Toxicity, Tumorigenicity, Skin and Eye Irritation
A08942	Acute Toxicity, Mutagenicity, Reproductive Effects, Multiple Dose Effects
A08943	Tumorigenicity
A08944	Acute Toxicity, Mutagenicity
A08945	Acute Toxicity
A08946	Acute Toxicity, Multiple Dose Effects
A08947	Acute Toxicity, Skin and Eye Irritation
A08948	Mutagenicity
A08949	Acute Toxicity, Multiple Dose Effects
A08950	Acute Toxicity
A08951	Mutagenicity
A08952	Acute Toxicity
A08953	Acute Toxicity, Multiple Dose Effects
A08954	Acute Toxicity, Reproductive Effects
A08955	Acute Toxicity
A08956	Multiple Dose Effects
A08957	Non-toxicity
A08958	Acute Toxicity
A08959	Acute Toxicity, Reproductive Effects
A08960	Acute Toxicity
A08961	Mutagenicity, Reproductive Effects, Multiple Dose Effects
A08962	Acute Toxicity, Mutagenicity
A08963	Acute Toxicity
A08964	Acute Toxicity, Multiple Dose Effects
A08965	Acute Toxicity, Mutagenicity
A08966	Acute Toxicity
A08967	Acute Toxicity
A08968	Acute Toxicity
A08969	Acute Toxicity
A08970	Acute Toxicity
A08971	Acute Toxicity, Multiple Dose Effects
A08972	Acute Toxicity
A08973	Acute Toxicity
A08974	Acute Toxicity
A08975	Acute Toxicity, Skin and Eye Irritation, Reproductive Effects, Multiple Dose
A08976	Acute Toxicity, Multiple Dose Effects
A08977	Acute Toxicity
A08978	Acute Toxicity
A08979	Acute Toxicity
A08980	Acute Toxicity

A08981	Acute Toxicity, Mutagenicity, Multiple Dose Effects
A08982	Acute Toxicity, Reproductive Effects
A08983	Acute Toxicity
A08984	Acute Toxicity, Mutagenicity
A08985	Acute Toxicity, Multiple Dose Effects
A08986	Acute Toxicity, Mutagenicity, Reproductive Effects, Multiple Dose Effects
A08987	Acute Toxicity, Multiple Dose Effects
A08988	Acute Toxicity
A08989	Acute Toxicity
A08990	Acute Toxicity
A08991	Acute Toxicity
A08992	Acute Toxicity
A08993	Acute Toxicity, Reproductive Effects, Multiple Dose Effects
A08994	Acute Toxicity, Mutagenicity, Multiple Dose Effects
A08995	Acute Toxicity, Mutagenicity, Multiple Dose Effects
A08996	Acute Toxicity, Mutagenicity, Reproductive Effects, Multiple Dose Effects
A08997	Multiple Dose Effects
A08998	Acute Toxicity, Mutagenicity, Reproductive Effects, Multiple Dose Effects
A08999	Acute Toxicity
A09000	Acute Toxicity, Mutagenicity, Reproductive Effects, Multiple Dose Effects
A09001	Acute Toxicity, Reproductive Effects, Multiple Dose Effects
A09002	Acute Toxicity
A09003	Acute Toxicity, Multiple Dose Effects
A09004	Acute Toxicity
A09005	Acute Toxicity
A09006	Acute Toxicity
A09007	Acute Toxicity, Reproductive Effects, Multiple Dose Effects
A09008	Acute Toxicity
A09009	Acute Toxicity, Reproductive Effects
A09010	Acute Toxicity, Multiple Dose Effects
A09011	Acute Toxicity, Mutagenicity, Skin and Eye Irritation
A09012	Multiple Dose Effects
A09013	Acute Toxicity, Skin and Eye Irritation, Multiple Dose Effects
A09014	Acute Toxicity
A09015	Acute Toxicity
A09016	Acute Toxicity
A09017	Acute Toxicity
A09018	Acute Toxicity
A09019	Acute Toxicity, Skin and Eye Irritation
A09020	Acute Toxicity, Multiple Dose Effects
A09021	Acute Toxicity
A09022	Multiple Dose Effects
A09023	Acute Toxicity, Reproductive Effects, Multiple Dose Effects
A09024	Acute Toxicity
A09025	Mutagenicity
A09026	Acute Toxicity, Skin and Eye Irritation

A09027	Acute Toxicity
A09028	Tumorigenicity
A09029	Acute Toxicity
A09030	Tumorigenicity
A09031	Acute Toxicity
A09032	Acute Toxicity
A09033	Acute Toxicity
A09034	Acute Toxicity
A09035	Acute Toxicity
A09036	Multiple Dose Effects
A09037	Acute Toxicity
A09038	Mutagenicity
A09039	Acute Toxicity
A09040	Acute Toxicity
A09041	Multiple Dose Effects
A09042	Acute Toxicity
A09043	Reproductive Effects
A09044	Acute Toxicity, Multiple Dose Effects
A09045	Acute Toxicity
A09046	Tumorigenicity, Multiple Dose Effects
A09047	Mutagenicity
A09048	Mutagenicity
A09049	Acute Toxicity
A09050	Reproductive Effects
A09051	Acute Toxicity, Mutagenicity
A09052	Mutagenicity
A09053	Tumorigenicity, Reproductive Effects
A09054	Mutagenicity, Multiple Dose Effects
A09055	Reproductive Effects
A09056	Mutagenicity, Tumorigenicity, Reproductive Effects
A09057	Acute Toxicity
A09058	Mutagenicity
A09059	Acute Toxicity, Reproductive Effects, Multiple Dose Effects
A09060	Reproductive Effects
A09061	Acute Toxicity
A09062	Acute Toxicity
A09063	Multiple Dose Effects
A09064	Acute Toxicity, Multiple Dose Effects
A09065	Mutagenicity
A09066	Acute Toxicity
A09067	Acute Toxicity
A09068	Mutagenicity
A09069	Acute Toxicity
A09070	Acute Toxicity
A09071	Mutagenicity
A09072	Acute Toxicity, Mutagenicity, Multiple Dose Effects

A09073	Acute Toxicity
A09074	Mutagenicity
A09075	Mutagenicity
A09076	Acute Toxicity
A09077	Acute Toxicity
A09078	Acute Toxicity, Mutagenicity
A09079	Acute Toxicity
A09080	Mutagenicity
A09081	Mutagenicity
A09082	Acute Toxicity
A09083	Acute Toxicity
A09084	Acute Toxicity, Skin and Eye Irritation, Multiple Dose Effects
A09085	Acute Toxicity, Reproductive Effects
A09086	Acute Toxicity
A09087	Acute Toxicity
A09088	Skin and Eye Irritation
A09089	Acute Toxicity, Skin and Eye Irritation
A09090	Acute Toxicity
A09091	Acute Toxicity
A09092	Acute Toxicity
A09093	Acute Toxicity
A09094	Acute Toxicity
A09095	Acute Toxicity
A09096	Acute Toxicity
A09097	Skin and Eye Irritation
A09098	Acute Toxicity
A09099	Acute Toxicity
A09100	Acute Toxicity
A09101	Acute Toxicity, Skin and Eye Irritation
A09102	Acute Toxicity
A09103	Mutagenicity
A09104	Acute Toxicity
A09105	Acute Toxicity
A09106	Acute Toxicity
A09107	Acute Toxicity, Mutagenicity, Skin and Eye Irritation
A09108	Acute Toxicity
A09109	Acute Toxicity
A09110	Acute Toxicity
A09111	Skin and Eye Irritation
A09112	Acute Toxicity
A09113	Mutagenicity
A09114	Acute Toxicity
A09115	Acute Toxicity, Multiple Dose Effects
A09116	Acute Toxicity
A09117	Acute Toxicity, Mutagenicity, Skin and Eye Irritation
A09118	Mutagenicity

A09119	Acute Toxicity, Skin and Eye Irritation
A09120	Acute Toxicity, Skin and Eye Irritation
A09121	Acute Toxicity
A09122	Mutagenicity
A09123	Acute Toxicity, Mutagenicity, Tumorigenicity
A09124	Mutagenicity
A09125	Tumorigenicity
A09126	Mutagenicity, Tumorigenicity, Multiple Dose Effects
A09127	Acute Toxicity, Multiple Dose Effects
A09128	Acute Toxicity
A09129	Acute Toxicity
A09130	Acute Toxicity, Mutagenicity, Skin and Eye Irritation, Multiple Dose Effects
A09131	Multiple Dose Effects
A09132	Mutagenicity
A09133	Acute Toxicity
A09134	Acute Toxicity, Mutagenicity, Skin and Eye Irritation
A09135	Acute Toxicity
A09136	Acute Toxicity
A09137	Acute Toxicity
A09138	Mutagenicity
A09139	Mutagenicity
A09140	Mutagenicity, Tumorigenicity
A09141	Mutagenicity
A09142	Mutagenicity
A09143	Mutagenicity
A09144	Acute Toxicity
A09145	Acute Toxicity
A09146	Acute Toxicity
A09147	Mutagenicity
A09148	Acute Toxicity
A09149	Acute Toxicity
A09150	Acute Toxicity
A09151	Multiple Dose Effects
A09152	Reproductive Effects, Multiple Dose Effects
A09153	Acute Toxicity
A09154	Tumorigenicity
A09155	Multiple Dose Effects
A09156	Mutagenicity
A09157	Mutagenicity
A09158	Acute Toxicity
A09159	Skin and Eye Irritation
A09160	Acute Toxicity
A09161	Acute Toxicity
A09162	Acute Toxicity, Tumorigenicity, Multiple Dose Effects
A09163	Mutagenicity
A09164	Acute Toxicity, Multiple Dose Effects

A09165	Mutagenicity
A09166	Acute Toxicity
A09167	Acute Toxicity, Mutagenicity
A09168	Acute Toxicity, Tumorigenicity, Reproductive Effects
A09169	Acute Toxicity
A09170	Mutagenicity
A09171	Acute Toxicity
A09172	Acute Toxicity
A09173	Acute Toxicity
A09174	Reproductive Effects, Multiple Dose Effects
A09175	Acute Toxicity
A09176	Acute Toxicity
A09177	Mutagenicity
A09178	Acute Toxicity
A09179	Acute Toxicity
A09180	Acute Toxicity, Mutagenicity
A09181	Acute Toxicity
A09182	Non-toxicity
A09183	Acute Toxicity
A09184	Acute Toxicity, Mutagenicity
A09185	Acute Toxicity
A09186	Mutagenicity
A09187	Acute Toxicity
A09188	Acute Toxicity
A09189	Acute Toxicity
A09190	Mutagenicity
A09191	Acute Toxicity
A09192	Acute Toxicity, Multiple Dose Effects
A09193	Acute Toxicity, Mutagenicity
A09194	Acute Toxicity
A09195	Acute Toxicity, Mutagenicity, Tumorigenicity
A09196	Acute Toxicity, Mutagenicity
A09197	Acute Toxicity
A09198	Mutagenicity
A09199	Acute Toxicity, Skin and Eye Irritation
A09200	Mutagenicity
A09201	Acute Toxicity, Multiple Dose Effects
A09202	Acute Toxicity, Multiple Dose Effects
A09203	Acute Toxicity
A09204	Acute Toxicity, Multiple Dose Effects
A09205	Acute Toxicity, Mutagenicity, Tumorigenicity, Multiple Dose Effects
A09206	Mutagenicity
A09207	Acute Toxicity
A09208	Acute Toxicity
A09209	Acute Toxicity
A09210	Acute Toxicity, Mutagenicity

A09211	Acute Toxicity
A09212	Acute Toxicity
A09213	Acute Toxicity, Skin and Eye Irritation
A09214	Acute Toxicity
A09215	Tumorigenicity
A09216	Acute Toxicity, Mutagenicity
A09217	Non-toxicity
A09218	Non-toxicity
A09219	Mutagenicity
A09220	Non-toxicity
A09221	Acute Toxicity, Skin and Eye Irritation
A09222	Acute Toxicity, Multiple Dose Effects
A09223	Acute Toxicity
A09224	Acute Toxicity
A09225	Acute Toxicity
A09226	Acute Toxicity
A09227	Acute Toxicity, Multiple Dose Effects
A09228	Acute Toxicity, Multiple Dose Effects
A09229	Acute Toxicity, Mutagenicity
A09230	Mutagenicity
A09231	Mutagenicity
A09232	Acute Toxicity
A09233	Acute Toxicity
A09234	Mutagenicity
A09235	Mutagenicity
A09236	Mutagenicity
A09237	Acute Toxicity
A09238	Acute Toxicity, Mutagenicity, Multiple Dose Effects
A09239	Acute Toxicity, Mutagenicity
A09240	Mutagenicity
A09241	Mutagenicity, Skin and Eye Irritation
A09242	Mutagenicity
A09243	Acute Toxicity, Mutagenicity
A09244	Acute Toxicity, Mutagenicity
A09245	Acute Toxicity, Mutagenicity, Tumorigenicity, Skin and Eye Irritation
A09246	Multiple Dose Effects
A09247	Non-toxicity
A09248	Non-toxicity
A09249	Mutagenicity, Skin and Eye Irritation
A09250	Mutagenicity
A09251	Non-toxicity
A09252	Acute Toxicity
A09253	Non-toxicity
A09254	Skin and Eye Irritation
A09255	Tumorigenicity, Skin and Eye Irritation
A09256	Acute Toxicity, Mutagenicity

A09257	Acute Toxicity, Mutagenicity
A09258	Acute Toxicity, Tumorigenicity
A09259	Acute Toxicity
A09260	Acute Toxicity
A09261	Acute Toxicity
A09262	Acute Toxicity, Mutagenicity
A09263	Acute Toxicity
A09264	Acute Toxicity
A09265	Acute Toxicity
A09266	Mutagenicity
A09267	Mutagenicity
A09268	Skin and Eye Irritation
A09269	Mutagenicity
A09270	Mutagenicity
A09271	Mutagenicity
A09272	Reproductive Effects
A09273	Acute Toxicity
A09274	Mutagenicity
A09275	Mutagenicity
A09276	Mutagenicity
A09277	Acute Toxicity, Mutagenicity, Tumorigenicity, Multiple Dose Effects
A09278	Acute Toxicity
A09279	Reproductive Effects
A09280	Acute Toxicity
A09281	Acute Toxicity
A09282	Acute Toxicity
A09283	Acute Toxicity, Mutagenicity
A09284	Acute Toxicity
A09285	Acute Toxicity
A09286	Acute Toxicity, Reproductive Effects, Multiple Dose Effects
A09287	Acute Toxicity, Multiple Dose Effects
A09288	Non-toxicity
A09289	Acute Toxicity
A09290	Acute Toxicity, Skin and Eye Irritation
A09291	Acute Toxicity
A09292	Acute Toxicity, Mutagenicity, Multiple Dose Effects
A09293	Acute Toxicity
A09294	Acute Toxicity
A09295	Reproductive Effects
A09296	Acute Toxicity
A09297	Acute Toxicity
A09298	Acute Toxicity, Multiple Dose Effects
A09299	Mutagenicity, Tumorigenicity, Multiple Dose Effects
A09300	Acute Toxicity, Reproductive Effects, Multiple Dose Effects
A09301	Acute Toxicity
A09302	Acute Toxicity, Mutagenicity

A09303	Acute Toxicity
A09304	Acute Toxicity, Multiple Dose Effects
A09305	Acute Toxicity, Skin and Eye Irritation, Multiple Dose Effects
A09306	Acute Toxicity
A09307	Acute Toxicity
A09308	Acute Toxicity
A09309	Multiple Dose Effects
A09310	Skin and Eye Irritation
A09311	Acute Toxicity, Mutagenicity, Tumorigenicity
A09312	Acute Toxicity
A09313	Acute Toxicity
A09314	Acute Toxicity, Skin and Eye Irritation
A09315	Acute Toxicity, Reproductive Effects, Multiple Dose Effects
A09316	Acute Toxicity, Reproductive Effects, Multiple Dose Effects
A09317	Acute Toxicity, Reproductive Effects
A09318	Acute Toxicity
A09319	Acute Toxicity
A09320	Acute Toxicity
A09321	Acute Toxicity
A09322	Acute Toxicity
A09323	Acute Toxicity, Mutagenicity, Tumorigenicity, Multiple Dose Effects
A09324	Acute Toxicity, Mutagenicity
A09325	Acute Toxicity
A09326	Acute Toxicity, Mutagenicity, Reproductive Effects, Multiple Dose Effects
A09327	Acute Toxicity
A09328	Skin and Eye Irritation
A09329	Acute Toxicity, Reproductive Effects
A09330	Non-toxicity
A09331	Acute Toxicity, Skin and Eye Irritation
A09332	Acute Toxicity, Reproductive Effects, Multiple Dose Effects
A09333	Mutagenicity, Reproductive Effects
A09334	Acute Toxicity, Multiple Dose Effects
A09335	Acute Toxicity
A09336	Acute Toxicity
A09337	Acute Toxicity
A09338	Acute Toxicity
A09339	Acute Toxicity, Multiple Dose Effects
A09340	Non-toxicity
A09341	Acute Toxicity
A09342	Acute Toxicity
A09343	Acute Toxicity
A09344	Skin and Eye Irritation
A09345	Acute Toxicity
A09346	Acute Toxicity
A09347	Acute Toxicity
A09348	Acute Toxicity, Multiple Dose Effects

A09349	Acute Toxicity
A09350	Acute Toxicity, Mutagenicity
A09351	Acute Toxicity
A09352	Acute Toxicity
A09353	Acute Toxicity
A09354	Mutagenicity
A09355	Acute Toxicity
A09356	Acute Toxicity, Mutagenicity
A09357	Acute Toxicity
A09358	Acute Toxicity
A09359	Acute Toxicity, Mutagenicity
A09360	Acute Toxicity, Skin and Eye Irritation
A09361	Acute Toxicity
A09362	Acute Toxicity
A09363	Mutagenicity
A09364	Acute Toxicity, Mutagenicity
A09365	Mutagenicity
A09366	Acute Toxicity
A09367	Acute Toxicity, Multiple Dose Effects
A09368	Mutagenicity
A09369	Acute Toxicity
A09370	Acute Toxicity, Skin and Eye Irritation
A09371	Mutagenicity
A09372	Mutagenicity
A09373	Acute Toxicity
A09374	Acute Toxicity
A09375	Acute Toxicity, Mutagenicity, Multiple Dose Effects
A09376	Mutagenicity
A09377	Non-toxicity
A09378	Mutagenicity
A09379	Acute Toxicity
A09380	Mutagenicity, Tumorigenicity
A09381	Acute Toxicity
A09382	Mutagenicity
A09383	Non-toxicity
A09384	Mutagenicity
A09385	Acute Toxicity
A09386	Tumorigenicity
A09387	Acute Toxicity
A09388	Mutagenicity
A09389	Acute Toxicity
A09390	Mutagenicity, Skin and Eye Irritation
A09391	Acute Toxicity
A09392	Acute Toxicity
A09393	Acute Toxicity, Multiple Dose Effects
A09394	Acute Toxicity

A09395	Acute Toxicity
A09396	Mutagenicity
A09397	Multiple Dose Effects
A09398	Mutagenicity
A09399	Mutagenicity
A09400	Acute Toxicity
A09401	Non-toxicity
A09402	Acute Toxicity
A09403	Mutagenicity
A09404	Acute Toxicity
A09405	Acute Toxicity
A09406	Acute Toxicity
A09407	Acute Toxicity
A09408	Acute Toxicity
A09409	Acute Toxicity
A09410	Multiple Dose Effects
A09411	Non-toxicity
A09412	Acute Toxicity
A09413	Acute Toxicity
A09414	Mutagenicity
A09415	Acute Toxicity
A09416	Acute Toxicity
A09417	Acute Toxicity
A09418	Non-toxicity
A09419	Acute Toxicity, Mutagenicity, Multiple Dose Effects
A09420	Acute Toxicity
A09421	Mutagenicity
A09422	Acute Toxicity, Reproductive Effects
A09423	Non-toxicity
A09424	Acute Toxicity
A09425	Acute Toxicity
A09426	Acute Toxicity, Multiple Dose Effects
A09427	Acute Toxicity
A09428	Acute Toxicity
A09429	Acute Toxicity, Reproductive Effects
A09430	Acute Toxicity
A09431	Acute Toxicity
A09432	Acute Toxicity
A09433	Mutagenicity
A09434	Acute Toxicity
A09435	Acute Toxicity, Reproductive Effects, Multiple Dose Effects
A09436	Acute Toxicity
A09437	Acute Toxicity
A09438	Acute Toxicity, Skin and Eye Irritation, Reproductive Effects, Multiple Dose
A09439	Acute Toxicity
A09440	Mutagenicity

A09441	Mutagenicity
A09442	Mutagenicity
A09443	Acute Toxicity, Mutagenicity, Skin and Eye Irritation
A09444	Acute Toxicity
A09445	Acute Toxicity
A09446	Acute Toxicity
A09447	Acute Toxicity
A09448	Mutagenicity
A09449	Acute Toxicity
A09450	Mutagenicity
A09451	Non-toxicity
A09452	Mutagenicity
A09453	Mutagenicity
A09454	Acute Toxicity, Mutagenicity
A09455	Acute Toxicity
A09456	Mutagenicity, Tumorigenicity
A09457	Skin and Eye Irritation, Multiple Dose Effects
A09458	Acute Toxicity, Multiple Dose Effects
A09459	Acute Toxicity
A09460	Mutagenicity
A09461	Acute Toxicity
A09462	Acute Toxicity
A09463	Acute Toxicity, Skin and Eye Irritation, Multiple Dose Effects
A09464	Acute Toxicity
A09465	Acute Toxicity
A09466	Mutagenicity
A09467	Tumorigenicity
A09468	Acute Toxicity, Mutagenicity
A09469	Acute Toxicity
A09470	Mutagenicity
A09471	Acute Toxicity
A09472	Acute Toxicity
A09473	Mutagenicity
A09474	Tumorigenicity
A09475	Acute Toxicity
A09476	Mutagenicity, Tumorigenicity
A09477	Mutagenicity, Tumorigenicity
A09478	Mutagenicity
A09479	Mutagenicity
A09480	Mutagenicity
A09481	Mutagenicity
A09482	Mutagenicity
A09483	Acute Toxicity, Mutagenicity
A09484	Acute Toxicity
A09485	Acute Toxicity, Skin and Eye Irritation
A09486	Acute Toxicity

A09487	Acute Toxicity
A09488	Non-toxicity
A09489	Acute Toxicity
A09490	Mutagenicity
A09491	Non-toxicity
A09492	Mutagenicity
A09493	Acute Toxicity
A09494	Acute Toxicity
A09495	Acute Toxicity
A09496	Acute Toxicity
A09497	Acute Toxicity, Mutagenicity, Reproductive Effects
A09498	Mutagenicity
A09499	Acute Toxicity, Mutagenicity, Multiple Dose Effects
A09500	Acute Toxicity, Skin and Eye Irritation
A09501	Acute Toxicity
A09502	Acute Toxicity
A09503	Acute Toxicity
A09504	Acute Toxicity
A09505	Acute Toxicity
A09506	Acute Toxicity, Mutagenicity
A09507	Mutagenicity
A09508	Acute Toxicity
A09509	Tumorigenicity
A09510	Acute Toxicity
A09511	Non-toxicity
A09512	Non-toxicity
A09513	Tumorigenicity
A09514	Acute Toxicity
A09515	Acute Toxicity
A09516	Skin and Eye Irritation
A09517	Acute Toxicity
A09518	Mutagenicity
A09519	Acute Toxicity, Mutagenicity, Skin and Eye Irritation
A09520	Acute Toxicity
A09521	Acute Toxicity
A09522	Acute Toxicity
A09523	Acute Toxicity
A09524	Mutagenicity
A09525	Tumorigenicity, Skin and Eye Irritation
A09526	Acute Toxicity
A09527	Acute Toxicity, Mutagenicity
A09528	Acute Toxicity, Mutagenicity, Multiple Dose Effects
A09529	Acute Toxicity
A09530	Acute Toxicity
A09531	Acute Toxicity
A09532	Acute Toxicity

A09533	Mutagenicity
A09534	Mutagenicity
A09535	Acute Toxicity, Mutagenicity, Skin and Eye Irritation
A09536	Non-toxicity
A09537	Non-toxicity
A09538	Acute Toxicity, Tumorigenicity, Multiple Dose Effects
A09539	Tumorigenicity
A09540	Acute Toxicity
A09541	Acute Toxicity
A09542	Acute Toxicity
A09543	Mutagenicity
A09544	Acute Toxicity
A09545	Acute Toxicity, Reproductive Effects, Multiple Dose Effects
A09546	Acute Toxicity
A09547	Acute Toxicity
A09548	Acute Toxicity
A09549	Acute Toxicity
A09550	Mutagenicity
A09551	Non-toxicity
A09552	Mutagenicity
A09553	Acute Toxicity
A09554	Mutagenicity
A09555	Acute Toxicity
A09556	Acute Toxicity
A09557	Acute Toxicity
A09558	Acute Toxicity
A09559	Multiple Dose Effects
A09560	Acute Toxicity
A09561	Mutagenicity
A09562	Acute Toxicity
A09563	Skin and Eye Irritation
A09564	Acute Toxicity
A09565	Acute Toxicity
A09566	Acute Toxicity, Reproductive Effects
A09567	Acute Toxicity
A09568	Acute Toxicity, Multiple Dose Effects
A09569	Acute Toxicity
A09570	Non-toxicity
A09571	Mutagenicity
A09572	Non-toxicity
A09573	Mutagenicity
A09574	Acute Toxicity
A09575	Acute Toxicity
A09576	Acute Toxicity
A09577	Acute Toxicity, Multiple Dose Effects
A09578	Acute Toxicity

A09579	Acute Toxicity
A09580	Acute Toxicity
A09581	Acute Toxicity, Skin and Eye Irritation, Reproductive Effects, Multiple Dose
A09582	Acute Toxicity, Reproductive Effects, Multiple Dose Effects
A09583	Acute Toxicity
A09584	Acute Toxicity
A09585	Acute Toxicity
A09586	Acute Toxicity
A09587	Acute Toxicity
A09588	Acute Toxicity
A09589	Acute Toxicity, Reproductive Effects
A09590	Acute Toxicity, Reproductive Effects
A09591	Acute Toxicity, Mutagenicity, Tumorigenicity, Reproductive Effects,
A09592	Acute Toxicity, Multiple Dose Effects
A09593	Acute Toxicity
A09594	Acute Toxicity
A09595	Acute Toxicity
A09596	Acute Toxicity
A09597	Acute Toxicity
A09598	Acute Toxicity, Mutagenicity
A09599	Acute Toxicity
A09600	Acute Toxicity
A09601	Acute Toxicity
A09602	Acute Toxicity, Multiple Dose Effects
A09603	Acute Toxicity, Mutagenicity
A09604	Acute Toxicity
A09605	Acute Toxicity, Multiple Dose Effects
A09606	Acute Toxicity, Reproductive Effects
A09607	Acute Toxicity, Mutagenicity, Reproductive Effects, Multiple Dose Effects
A09608	Acute Toxicity
A09609	Acute Toxicity
A09610	Acute Toxicity
A09611	Acute Toxicity, Mutagenicity, Reproductive Effects, Multiple Dose Effects
A09612	Acute Toxicity, Reproductive Effects, Multiple Dose Effects
A09613	Acute Toxicity
A09614	Acute Toxicity
A09615	Acute Toxicity
A09616	Acute Toxicity
A09617	Acute Toxicity
A09618	Acute Toxicity
A09619	Non-toxicity
A09620	Mutagenicity
A09621	Acute Toxicity, Reproductive Effects
A09622	Acute Toxicity
A09623	Acute Toxicity
A09624	Acute Toxicity, Mutagenicity, Multiple Dose Effects

A09625	Acute Toxicity, Skin and Eye Irritation, Reproductive Effects, Multiple Dose
A09626	Acute Toxicity
A09627	Non-toxicity
A09628	Acute Toxicity, Skin and Eye Irritation, Reproductive Effects, Multiple Dose
A09629	Acute Toxicity
A09630	Acute Toxicity, Tumorigenicity
A09631	Acute Toxicity, Mutagenicity, Reproductive Effects, Multiple Dose Effects
A09632	Acute Toxicity, Mutagenicity, Multiple Dose Effects
A09633	Acute Toxicity, Mutagenicity, Tumorigenicity
A09634	Acute Toxicity
A09635	Acute Toxicity
A09636	Acute Toxicity
A09637	Acute Toxicity
A09638	Acute Toxicity
A09639	Acute Toxicity
A09640	Acute Toxicity
A09641	Acute Toxicity
A09642	Acute Toxicity
A09643	Acute Toxicity
A09644	Acute Toxicity, Mutagenicity
A09645	Acute Toxicity
A09646	Non-toxicity
A09647	Mutagenicity, Tumorigenicity
A09648	Acute Toxicity, Multiple Dose Effects
A09649	Acute Toxicity
A09650	Acute Toxicity, Mutagenicity
A09651	Acute Toxicity
A09652	Skin and Eye Irritation
A09653	Acute Toxicity
A09654	Acute Toxicity, Multiple Dose Effects
A09655	Acute Toxicity
A09656	Acute Toxicity
A09657	Acute Toxicity
A09658	Acute Toxicity
A09659	Multiple Dose Effects
A09660	Acute Toxicity
A09661	Acute Toxicity
A09662	Acute Toxicity
A09663	Multiple Dose Effects
A09664	Mutagenicity, Reproductive Effects
A09665	Reproductive Effects
A09666	Acute Toxicity, Mutagenicity, Reproductive Effects
A09667	Acute Toxicity
A09668	Acute Toxicity, Mutagenicity
A09669	Acute Toxicity
A09670	Acute Toxicity, Mutagenicity, Multiple Dose Effects

A09671	Acute Toxicity, Mutagenicity, Reproductive Effects
A09672	Acute Toxicity, Skin and Eye Irritation
A09673	Acute Toxicity, Mutagenicity, Reproductive Effects, Multiple Dose Effects
A09674	Multiple Dose Effects
A09675	Acute Toxicity, Reproductive Effects, Multiple Dose Effects
A09676	Acute Toxicity, Tumorigenicity
A09677	Acute Toxicity, Multiple Dose Effects
A09678	Acute Toxicity
A09679	Acute Toxicity
A09680	Mutagenicity
A09681	Acute Toxicity
A09682	Acute Toxicity
A09683	Acute Toxicity
A09684	Acute Toxicity
A09685	Acute Toxicity
A09686	Acute Toxicity
A09687	Acute Toxicity, Reproductive Effects, Multiple Dose Effects
A09688	Acute Toxicity
A09689	Acute Toxicity
A09690	Acute Toxicity
A09691	Multiple Dose Effects
A09692	Acute Toxicity
A09693	Acute Toxicity
A09694	Acute Toxicity
A09695	Acute Toxicity
A09696	Acute Toxicity
A09697	Acute Toxicity, Reproductive Effects, Multiple Dose Effects
A09698	Acute Toxicity
A09699	Acute Toxicity
A09700	Acute Toxicity
A09701	Acute Toxicity
A09702	Acute Toxicity, Reproductive Effects
A09703	Acute Toxicity, Reproductive Effects, Multiple Dose Effects
A09704	Acute Toxicity, Reproductive Effects
A09705	Acute Toxicity, Mutagenicity, Reproductive Effects, Multiple Dose Effects
A09706	Acute Toxicity, Multiple Dose Effects
A09707	Acute Toxicity
A09708	Acute Toxicity
A09709	Acute Toxicity, Tumorigenicity
A09710	Acute Toxicity
A09711	Acute Toxicity
A09712	Acute Toxicity, Multiple Dose Effects
A09713	Acute Toxicity
A09714	Mutagenicity
A09715	Acute Toxicity, Multiple Dose Effects
A09716	Acute Toxicity

A09717	Acute Toxicity
A09718	Acute Toxicity
A09719	Acute Toxicity
A09720	Acute Toxicity
A09721	Acute Toxicity, Multiple Dose Effects
A09722	Acute Toxicity
A09723	Acute Toxicity, Skin and Eye Irritation, Multiple Dose Effects
A09724	Acute Toxicity, Multiple Dose Effects
A09725	Mutagenicity
A09726	Acute Toxicity
A09727	Acute Toxicity, Mutagenicity, Reproductive Effects, Multiple Dose Effects
A09728	Multiple Dose Effects
A09729	Acute Toxicity, Mutagenicity
A09730	Acute Toxicity
A09731	Acute Toxicity
A09732	Acute Toxicity, Mutagenicity, Tumorigenicity, Multiple Dose Effects
A09733	Acute Toxicity, Mutagenicity, Tumorigenicity
A09734	Multiple Dose Effects
A09735	Acute Toxicity, Skin and Eye Irritation, Multiple Dose Effects
A09736	Mutagenicity
A09737	Mutagenicity, Tumorigenicity
A09738	Tumorigenicity
A09739	Acute Toxicity
A09740	Acute Toxicity, Mutagenicity, Multiple Dose Effects
A09741	Acute Toxicity, Multiple Dose Effects
A09742	Reproductive Effects
A09743	Skin and Eye Irritation, Multiple Dose Effects
A09744	Acute Toxicity
A09745	Acute Toxicity, Tumorigenicity, Multiple Dose Effects
A09746	Non-toxicity
A09747	Acute Toxicity
A09748	Acute Toxicity, Mutagenicity
A09749	Acute Toxicity, Mutagenicity
A09750	Acute Toxicity, Tumorigenicity, Multiple Dose Effects
A09751	Acute Toxicity
A09752	Acute Toxicity
A09753	Acute Toxicity
A09754	Acute Toxicity
A09755	Acute Toxicity
A09756	Mutagenicity
A09757	Acute Toxicity, Multiple Dose Effects
A09758	Acute Toxicity, Mutagenicity, Tumorigenicity
A09759	Acute Toxicity
A09760	Acute Toxicity
A09761	Acute Toxicity, Mutagenicity, Reproductive Effects, Multiple Dose Effects
A09762	Mutagenicity, Reproductive Effects, Multiple Dose Effects

A09763	Acute Toxicity
A09764	Acute Toxicity, Multiple Dose Effects
A09765	Acute Toxicity
A09766	Acute Toxicity
A09767	Mutagenicity
A09768	Acute Toxicity, Mutagenicity
A09769	Acute Toxicity
A09770	Acute Toxicity, Skin and Eye Irritation
A09771	Acute Toxicity, Mutagenicity
A09772	Mutagenicity
A09773	Reproductive Effects
A09774	Acute Toxicity, Reproductive Effects
A09775	Acute Toxicity
A09776	Skin and Eye Irritation
A09777	Mutagenicity
A09778	Non-toxicity
A09779	Acute Toxicity
A09780	Acute Toxicity
A09781	Mutagenicity
A09782	Mutagenicity
A09783	Acute Toxicity, Multiple Dose Effects
A09784	Acute Toxicity, Mutagenicity
A09785	Acute Toxicity
A09786	Acute Toxicity
A09787	Mutagenicity
A09788	Acute Toxicity
A09789	Acute Toxicity
A09790	Acute Toxicity
A09791	Mutagenicity
A09792	Acute Toxicity
A09793	Mutagenicity
A09794	Reproductive Effects
A09795	Acute Toxicity
A09796	Mutagenicity, Tumorigenicity
A09797	Acute Toxicity, Mutagenicity
A09798	Acute Toxicity
A09799	Acute Toxicity
A09800	Acute Toxicity, Skin and Eye Irritation
A09801	Acute Toxicity
A09802	Acute Toxicity
A09803	Mutagenicity, Tumorigenicity
A09804	Non-toxicity
A09805	Acute Toxicity
A09806	Acute Toxicity
A09807	Acute Toxicity, Mutagenicity
A09808	Acute Toxicity

A09809	Acute Toxicity
A09810	Acute Toxicity
A09811	Mutagenicity, Multiple Dose Effects
A09812	Mutagenicity
A09813	Acute Toxicity
A09814	Acute Toxicity
A09815	Reproductive Effects
A09816	Acute Toxicity
A09817	Acute Toxicity, Multiple Dose Effects
A09818	Mutagenicity
A09819	Acute Toxicity, Mutagenicity
A09820	Mutagenicity
A09821	Mutagenicity
A09822	Acute Toxicity, Mutagenicity
A09823	Acute Toxicity
A09824	Mutagenicity
A09825	Acute Toxicity, Mutagenicity, Multiple Dose Effects
A09826	Reproductive Effects
A09827	Acute Toxicity
A09828	Multiple Dose Effects
A09829	Mutagenicity, Reproductive Effects
A09830	Acute Toxicity
A09831	Skin and Eye Irritation
A09832	Acute Toxicity
A09833	Acute Toxicity
A09834	Skin and Eye Irritation
A09835	Acute Toxicity, Multiple Dose Effects
A09836	Multiple Dose Effects
A09837	Acute Toxicity, Mutagenicity, Multiple Dose Effects
A09838	Acute Toxicity, Tumorigenicity
A09839	Acute Toxicity
A09840	Mutagenicity
A09841	Acute Toxicity
A09842	Acute Toxicity
A09843	Tumorigenicity
A09844	Acute Toxicity
A09845	Acute Toxicity
A09846	Acute Toxicity, Mutagenicity, Multiple Dose Effects
A09847	Acute Toxicity, Mutagenicity, Skin and Eye Irritation
A09848	Acute Toxicity
A09849	Acute Toxicity
A09850	Acute Toxicity
A09851	Acute Toxicity
A09852	Acute Toxicity, Multiple Dose Effects
A09853	Mutagenicity
A09854	Non-toxicity

A09855	Acute Toxicity
A09856	Acute Toxicity
A09857	Acute Toxicity, Tumorigenicity
A09858	Acute Toxicity
A09859	Mutagenicity
A09860	Acute Toxicity
A09861	Acute Toxicity
A09862	Acute Toxicity, Reproductive Effects
A09863	Acute Toxicity, Multiple Dose Effects
A09864	Acute Toxicity
A09865	Acute Toxicity, Mutagenicity, Reproductive Effects
A09866	Acute Toxicity
A09867	Mutagenicity
A09868	Acute Toxicity
A09869	Mutagenicity
A09870	Acute Toxicity
A09871	Non-toxicity
A09872	Mutagenicity, Reproductive Effects
A09873	Acute Toxicity, Multiple Dose Effects
A09874	Multiple Dose Effects
A09875	Acute Toxicity, Multiple Dose Effects
A09876	Acute Toxicity, Reproductive Effects, Multiple Dose Effects
A09877	Acute Toxicity
A09878	Acute Toxicity, Reproductive Effects, Multiple Dose Effects
A09879	Acute Toxicity, Multiple Dose Effects
A09880	Acute Toxicity, Reproductive Effects, Multiple Dose Effects
A09881	Acute Toxicity, Multiple Dose Effects
A09882	Acute Toxicity
A09883	Mutagenicity
A09884	Acute Toxicity
A09885	Acute Toxicity
A09886	Tumorigenicity
A09887	Acute Toxicity
A09888	Acute Toxicity
A09889	Acute Toxicity
A09890	Mutagenicity
A09891	Multiple Dose Effects
A09892	Acute Toxicity, Mutagenicity, Multiple Dose Effects
A09893	Acute Toxicity
A09894	Acute Toxicity
A09895	Acute Toxicity, Skin and Eye Irritation, Multiple Dose Effects
A09896	Acute Toxicity
A09897	Non-toxicity
A09898	Acute Toxicity, Multiple Dose Effects
A09899	Mutagenicity
A09900	Acute Toxicity

A09901	Tumorigenicity
A09902	Acute Toxicity
A09903	Mutagenicity, Tumorigenicity
A09904	Acute Toxicity
A09905	Acute Toxicity
A09906	Tumorigenicity
A09907	Acute Toxicity
A09908	Acute Toxicity, Skin and Eye Irritation
A09909	Acute Toxicity
A09910	Skin and Eye Irritation
A09911	Mutagenicity
A09912	Acute Toxicity
A09913	Acute Toxicity
A09914	Tumorigenicity
A09915	Mutagenicity
A09916	Acute Toxicity, Multiple Dose Effects
A09917	Tumorigenicity
A09918	Acute Toxicity
A09919	Acute Toxicity, Skin and Eye Irritation, Multiple Dose Effects
A09920	Acute Toxicity, Reproductive Effects
A09921	Mutagenicity
A09922	Acute Toxicity, Reproductive Effects, Multiple Dose Effects
A09923	Acute Toxicity, Mutagenicity
A09924	Acute Toxicity
A09925	Mutagenicity
A09926	Skin and Eye Irritation
A09927	Tumorigenicity
A09928	Acute Toxicity
A09929	Acute Toxicity
A09930	Acute Toxicity
A09931	Acute Toxicity
A09932	Acute Toxicity, Reproductive Effects, Multiple Dose Effects
A09933	Acute Toxicity, Mutagenicity, Tumorigenicity
A09934	Acute Toxicity
A09935	Mutagenicity
A09936	Acute Toxicity
A09937	Non-toxicity
A09938	Mutagenicity
A09939	Acute Toxicity, Tumorigenicity
A09940	Acute Toxicity
A09941	Acute Toxicity
A09942	Acute Toxicity
A09943	Acute Toxicity, Multiple Dose Effects
A09944	Acute Toxicity
A09945	Acute Toxicity, Reproductive Effects
A09946	Mutagenicity

A09947	Acute Toxicity
A09948	Acute Toxicity
A09949	Tumorigenicity
A09950	Acute Toxicity, Mutagenicity, Tumorigenicity
A09951	Acute Toxicity
A09952	Mutagenicity, Reproductive Effects
A09953	Mutagenicity, Reproductive Effects
A09954	Acute Toxicity
A09955	Acute Toxicity, Reproductive Effects
A09956	Tumorigenicity
A09957	Acute Toxicity, Mutagenicity
A09958	Acute Toxicity
A09959	Acute Toxicity
A09960	Acute Toxicity
A09961	Skin and Eye Irritation
A09962	Mutagenicity
A09963	Acute Toxicity, Mutagenicity, Reproductive Effects
A09964	Acute Toxicity
A09965	Acute Toxicity
A09966	Acute Toxicity
A09967	Skin and Eye Irritation
A09968	Acute Toxicity
A09969	Acute Toxicity
A09970	Acute Toxicity
A09971	Skin and Eye Irritation
A09972	Acute Toxicity
A09973	Acute Toxicity
A09974	Acute Toxicity
A09975	Acute Toxicity, Mutagenicity, Skin and Eye Irritation
A09976	Acute Toxicity
A09977	Acute Toxicity
A09978	Acute Toxicity
A09979	Acute Toxicity
A09980	Mutagenicity
A09981	Acute Toxicity
A09982	Acute Toxicity
A09983	Acute Toxicity
A09984	Multiple Dose Effects
A09985	Acute Toxicity
A09986	Acute Toxicity
A09987	Acute Toxicity
A09988	Acute Toxicity, Mutagenicity, Skin and Eye Irritation
A09989	Acute Toxicity, Skin and Eye Irritation
A09990	Acute Toxicity
A09991	Acute Toxicity, Multiple Dose Effects
A09992	Mutagenicity

A09993	Acute Toxicity
A09994	Acute Toxicity
A09995	Acute Toxicity, Mutagenicity
A09996	Acute Toxicity, Mutagenicity, Multiple Dose Effects
A09997	Multiple Dose Effects
A09998	Acute Toxicity
A09999	Acute Toxicity
A10000	Non-toxicity
A10001	Multiple Dose Effects
A10002	Skin and Eye Irritation
A10003	Mutagenicity, Skin and Eye Irritation
A10004	Acute Toxicity
A10005	Acute Toxicity
A10006	Acute Toxicity
A10007	Acute Toxicity, Mutagenicity
A10008	Mutagenicity
A10009	Multiple Dose Effects
A10010	Mutagenicity, Tumorigenicity
A10011	Acute Toxicity, Multiple Dose Effects
A10012	Acute Toxicity
A10013	Mutagenicity
A10014	Non-toxicity
A10015	Acute Toxicity
A10016	Acute Toxicity
A10017	Acute Toxicity
A10018	Mutagenicity
A10019	Acute Toxicity, Mutagenicity
A10020	Acute Toxicity
A10021	Non-toxicity
A10022	Acute Toxicity, Reproductive Effects
A10023	Mutagenicity
A10024	Tumorigenicity
A10025	Acute Toxicity, Skin and Eye Irritation, Multiple Dose Effects
A10026	Acute Toxicity
A10027	Acute Toxicity, Mutagenicity
A10028	Acute Toxicity, Multiple Dose Effects
A10029	Acute Toxicity
A10030	Acute Toxicity, Skin and Eye Irritation, Multiple Dose Effects
A10031	Acute Toxicity, Multiple Dose Effects
A10032	Skin and Eye Irritation
A10033	Acute Toxicity, Skin and Eye Irritation
A10034	Acute Toxicity, Multiple Dose Effects
A10035	Acute Toxicity, Skin and Eye Irritation
A10036	Acute Toxicity
A10037	Acute Toxicity, Multiple Dose Effects
A10038	Acute Toxicity, Multiple Dose Effects

A10039	Mutagenicity
A10040	Mutagenicity
A10041	Mutagenicity
A10042	Mutagenicity
A10043	Acute Toxicity
A10044	Skin and Eye Irritation, Multiple Dose Effects
A10045	Skin and Eye Irritation, Multiple Dose Effects
A10046	Skin and Eye Irritation, Multiple Dose Effects
A10047	Acute Toxicity
A10048	Acute Toxicity
A10049	Mutagenicity
A10050	Reproductive Effects
A10051	Non-toxicity
A10052	Acute Toxicity, Multiple Dose Effects
A10053	Acute Toxicity, Skin and Eye Irritation
A10054	Acute Toxicity, Skin and Eye Irritation
A10055	Acute Toxicity
A10056	Multiple Dose Effects
A10057	Acute Toxicity
A10058	Acute Toxicity
A10059	Acute Toxicity
A10060	Acute Toxicity, Skin and Eye Irritation
A10061	Mutagenicity
A10062	Acute Toxicity, Skin and Eye Irritation
A10063	Non-toxicity
A10064	Acute Toxicity
A10065	Acute Toxicity
A10066	Acute Toxicity, Mutagenicity, Skin and Eye Irritation
A10067	Mutagenicity
A10068	Acute Toxicity
A10069	Acute Toxicity
A10070	Acute Toxicity
A10071	Acute Toxicity
A10072	Mutagenicity, Multiple Dose Effects
A10073	Acute Toxicity
A10074	Acute Toxicity
A10075	Acute Toxicity
A10076	Acute Toxicity
A10077	Acute Toxicity, Skin and Eye Irritation
A10078	Acute Toxicity, Mutagenicity
A10079	Non-toxicity
A10080	Acute Toxicity
A10081	Mutagenicity
A10082	Acute Toxicity
A10083	Acute Toxicity
A10084	Reproductive Effects

A10085	Mutagenicity
A10086	Non-toxicity
A10087	Acute Toxicity
A10088	Acute Toxicity, Mutagenicity
A10089	Mutagenicity
A10090	Acute Toxicity
A10091	Skin and Eye Irritation
A10092	Acute Toxicity
A10093	Acute Toxicity, Tumorigenicity
A10094	Acute Toxicity
A10095	Mutagenicity, Tumorigenicity
A10096	Mutagenicity, Tumorigenicity
A10097	Acute Toxicity
A10098	Acute Toxicity
A10099	Acute Toxicity
A10100	Acute Toxicity
A10101	Mutagenicity, Tumorigenicity
A10102	Reproductive Effects
A10103	Multiple Dose Effects
A10104	Acute Toxicity
A10105	Acute Toxicity
A10106	Acute Toxicity
A10107	Acute Toxicity
A10108	Acute Toxicity
A10109	Acute Toxicity
A10110	Acute Toxicity
A10111	Acute Toxicity
A10112	Acute Toxicity
A10113	Non-toxicity
A10114	Acute Toxicity
A10115	Acute Toxicity
A10116	Acute Toxicity, Mutagenicity, Multiple Dose Effects
A10117	Acute Toxicity
A10118	Acute Toxicity
A10119	Acute Toxicity
A10120	Acute Toxicity
A10121	Acute Toxicity, Multiple Dose Effects
A10122	Acute Toxicity
A10123	Acute Toxicity
A10124	Acute Toxicity, Skin and Eye Irritation
A10125	Mutagenicity
A10126	Acute Toxicity
A10127	Mutagenicity
A10128	Tumorigenicity
A10129	Acute Toxicity
A10130	Mutagenicity

A10131	Acute Toxicity
A10132	Acute Toxicity
A10133	Acute Toxicity
A10134	Acute Toxicity, Reproductive Effects, Multiple Dose Effects
A10135	Acute Toxicity
A10136	Mutagenicity
A10137	Acute Toxicity
A10138	Acute Toxicity
A10139	Mutagenicity
A10140	Acute Toxicity, Tumorigenicity
A10141	Acute Toxicity
A10142	Acute Toxicity
A10143	Acute Toxicity
A10144	Acute Toxicity
A10145	Acute Toxicity, Skin and Eye Irritation
A10146	Acute Toxicity, Skin and Eye Irritation, Reproductive Effects, Multiple Dose
A10147	Multiple Dose Effects
A10148	Acute Toxicity, Multiple Dose Effects
A10149	Acute Toxicity, Skin and Eye Irritation, Multiple Dose Effects
A10150	Acute Toxicity
A10151	Acute Toxicity, Multiple Dose Effects
A10152	Acute Toxicity
A10153	Skin and Eye Irritation
A10154	Acute Toxicity
A10155	Acute Toxicity
A10156	Acute Toxicity, Mutagenicity, Tumorigenicity
A10157	Acute Toxicity
A10158	Mutagenicity
A10159	Acute Toxicity, Mutagenicity, Tumorigenicity, Reproductive Effects,
A10160	Mutagenicity
A10161	Acute Toxicity, Mutagenicity
A10162	Acute Toxicity
A10163	Acute Toxicity
A10164	Non-toxicity
A10165	Acute Toxicity
A10166	Mutagenicity
A10167	Acute Toxicity
A10168	Acute Toxicity, Mutagenicity
A10169	Tumorigenicity
A10170	Acute Toxicity
A10171	Mutagenicity
A10172	Mutagenicity, Tumorigenicity
A10173	Mutagenicity
A10174	Acute Toxicity
A10175	Non-toxicity
A10176	Acute Toxicity, Mutagenicity

A10177	Skin and Eye Irritation
A10178	Skin and Eye Irritation
A10179	Non-toxicity
A10180	Acute Toxicity
A10181	Acute Toxicity
A10182	Acute Toxicity
A10183	Acute Toxicity, Skin and Eye Irritation
A10184	Acute Toxicity
A10185	Mutagenicity
A10186	Acute Toxicity
A10187	Acute Toxicity
A10188	Acute Toxicity
A10189	Skin and Eye Irritation
A10190	Acute Toxicity
A10191	Acute Toxicity
A10192	Acute Toxicity
A10193	Mutagenicity
A10194	Mutagenicity
A10195	Acute Toxicity
A10196	Mutagenicity
A10197	Acute Toxicity
A10198	Mutagenicity, Tumorigenicity
A10199	Reproductive Effects
A10200	Acute Toxicity, Skin and Eye Irritation
A10201	Acute Toxicity, Tumorigenicity
A10202	Multiple Dose Effects
A10203	Mutagenicity
A10204	Acute Toxicity
A10205	Non-toxicity
A10206	Acute Toxicity
A10207	Acute Toxicity
A10208	Acute Toxicity
A10209	Acute Toxicity
A10210	Acute Toxicity
A10211	Acute Toxicity, Skin and Eye Irritation, Multiple Dose Effects
A10212	Acute Toxicity
A10213	Acute Toxicity
A10214	Non-toxicity
A10215	Mutagenicity
A10216	Acute Toxicity
A10217	Non-toxicity
A10218	Acute Toxicity
A10219	Acute Toxicity
A10220	Acute Toxicity, Skin and Eye Irritation, Multiple Dose Effects
A10221	Acute Toxicity
A10222	Skin and Eye Irritation, Multiple Dose Effects

A10223	Acute Toxicity, Mutagenicity
A10224	Mutagenicity
A10225	Acute Toxicity, Tumorigenicity
A10226	Multiple Dose Effects
A10227	Acute Toxicity
A10228	Acute Toxicity, Mutagenicity
A10229	Acute Toxicity
A10230	Acute Toxicity
A10231	Acute Toxicity
A10232	Acute Toxicity
A10233	Non-toxicity
A10234	Acute Toxicity, Reproductive Effects
A10235	Acute Toxicity
A10236	Mutagenicity
A10237	Acute Toxicity
A10238	Acute Toxicity, Multiple Dose Effects
A10239	Acute Toxicity, Mutagenicity, Skin and Eye Irritation
A10240	Acute Toxicity
A10241	Acute Toxicity, Skin and Eye Irritation, Reproductive Effects, Multiple Dose
A10242	Mutagenicity
A10243	Acute Toxicity
A10244	Acute Toxicity
A10245	Acute Toxicity
A10246	Acute Toxicity
A10247	Mutagenicity
A10248	Acute Toxicity
A10249	Acute Toxicity
A10250	Acute Toxicity, Tumorigenicity
A10251	Acute Toxicity
A10252	Mutagenicity
A10253	Acute Toxicity, Reproductive Effects, Multiple Dose Effects
A10254	Mutagenicity
A10255	Acute Toxicity, Multiple Dose Effects
A10256	Mutagenicity
A10257	Mutagenicity
A10258	Mutagenicity
A10259	Acute Toxicity
A10260	Acute Toxicity, Multiple Dose Effects
A10261	Tumorigenicity
A10262	Acute Toxicity
A10263	Reproductive Effects
A10264	Mutagenicity
A10265	Non-toxicity
A10266	Acute Toxicity
A10267	Acute Toxicity, Skin and Eye Irritation
A10268	Acute Toxicity

A10269	Acute Toxicity
A10270	Mutagenicity
A10271	Acute Toxicity
A10272	Mutagenicity, Multiple Dose Effects
A10273	Acute Toxicity
A10274	Mutagenicity, Tumorigenicity
A10275	Acute Toxicity
A10276	Mutagenicity, Tumorigenicity
A10277	Acute Toxicity
A10278	Mutagenicity, Tumorigenicity
A10279	Acute Toxicity, Skin and Eye Irritation
A10280	Acute Toxicity
A10281	Acute Toxicity, Mutagenicity
A10282	Acute Toxicity
A10283	Acute Toxicity
A10284	Mutagenicity
A10285	Acute Toxicity
A10286	Acute Toxicity, Mutagenicity, Skin and Eye Irritation
A10287	Acute Toxicity
A10288	Non-toxicity
A10289	Mutagenicity
A10290	Mutagenicity
A10291	Acute Toxicity, Mutagenicity
A10292	Mutagenicity
A10293	Acute Toxicity
A10294	Mutagenicity
A10295	Acute Toxicity, Tumorigenicity
A10296	Acute Toxicity
A10297	Acute Toxicity, Skin and Eye Irritation
A10298	Acute Toxicity
A10299	Mutagenicity
A10300	Tumorigenicity
A10301	Acute Toxicity
A10302	Acute Toxicity
A10303	Mutagenicity
A10304	Acute Toxicity
A10305	Acute Toxicity, Multiple Dose Effects
A10306	Acute Toxicity
A10307	Acute Toxicity
A10308	Mutagenicity
A10309	Mutagenicity
A10310	Acute Toxicity
A10311	Acute Toxicity
A10312	Acute Toxicity
A10313	Skin and Eye Irritation
A10314	Mutagenicity

A10315	Acute Toxicity, Multiple Dose Effects
A10316	Acute Toxicity, Multiple Dose Effects
A10317	Acute Toxicity, Skin and Eye Irritation, Reproductive Effects, Multiple Dose
A10318	Acute Toxicity, Mutagenicity, Reproductive Effects, Multiple Dose Effects
A10319	Acute Toxicity, Mutagenicity
A10320	Acute Toxicity, Multiple Dose Effects
A10321	Acute Toxicity
A10322	Acute Toxicity
A10323	Mutagenicity
A10324	Acute Toxicity
A10325	Acute Toxicity
A10326	Acute Toxicity
A10327	Skin and Eye Irritation, Multiple Dose Effects
A10328	Acute Toxicity
A10329	Mutagenicity
A10330	Acute Toxicity
A10331	Acute Toxicity
A10332	Mutagenicity
A10333	Acute Toxicity
A10334	Acute Toxicity, Multiple Dose Effects
A10335	Acute Toxicity, Mutagenicity, Tumorigenicity
A10336	Multiple Dose Effects
A10337	Acute Toxicity, Skin and Eye Irritation, Multiple Dose Effects
A10338	Acute Toxicity
A10339	Multiple Dose Effects
A10340	Non-toxicity
A10341	Mutagenicity
A10342	Non-toxicity
A10343	Acute Toxicity, Skin and Eye Irritation
A10344	Acute Toxicity, Mutagenicity, Tumorigenicity
A10345	Acute Toxicity, Mutagenicity, Tumorigenicity, Reproductive Effects
A10346	Acute Toxicity, Skin and Eye Irritation
A10347	Acute Toxicity
A10348	Acute Toxicity, Tumorigenicity, Multiple Dose Effects
A10349	Tumorigenicity
A10350	Acute Toxicity, Mutagenicity, Tumorigenicity
A10351	Acute Toxicity, Mutagenicity, Tumorigenicity
A10352	Acute Toxicity
A10353	Mutagenicity, Tumorigenicity
A10354	Acute Toxicity, Mutagenicity, Tumorigenicity
A10355	Acute Toxicity
A10356	Mutagenicity
A10357	Acute Toxicity, Mutagenicity
A10358	Acute Toxicity, Mutagenicity, Tumorigenicity, Reproductive Effects
A10359	Multiple Dose Effects
A10360	Acute Toxicity

A10361	Acute Toxicity, Tumorigenicity
A10362	Acute Toxicity
A10363	Mutagenicity
A10364	Non-toxicity
A10365	Multiple Dose Effects
A10366	Acute Toxicity
A10367	Acute Toxicity
A10368	Acute Toxicity
A10369	Mutagenicity
A10370	Acute Toxicity, Mutagenicity, Skin and Eye Irritation, Multiple Dose Effects
A10371	Acute Toxicity
A10372	Acute Toxicity, Multiple Dose Effects
A10373	Acute Toxicity, Mutagenicity, Tumorigenicity, Skin and Eye Irritation, Multiple Dose Effects
A10374	Acute Toxicity
A10375	Acute Toxicity
A10376	Acute Toxicity
A10377	Acute Toxicity
A10378	Acute Toxicity
A10379	Acute Toxicity
A10380	Acute Toxicity
A10381	Acute Toxicity
A10382	Acute Toxicity, Mutagenicity
A10383	Mutagenicity
A10384	Non-toxicity
A10385	Acute Toxicity
A10386	Mutagenicity
A10387	Mutagenicity
A10388	Acute Toxicity
A10389	Acute Toxicity
A10390	Acute Toxicity
A10391	Multiple Dose Effects
A10392	Acute Toxicity, Multiple Dose Effects
A10393	Acute Toxicity
A10394	Acute Toxicity
A10395	Acute Toxicity
A10396	Acute Toxicity
A10397	Acute Toxicity
A10398	Mutagenicity
A10399	Acute Toxicity
A10400	Acute Toxicity
A10401	Mutagenicity, Tumorigenicity
A10402	Acute Toxicity
A10403	Acute Toxicity
A10404	Mutagenicity, Tumorigenicity
A10405	Mutagenicity

A10406	Acute Toxicity
A10407	Acute Toxicity
A10408	Acute Toxicity
A10409	Acute Toxicity, Reproductive Effects, Multiple Dose Effects
A10410	Skin and Eye Irritation
A10411	Acute Toxicity, Mutagenicity, Tumorigenicity
A10412	Acute Toxicity
A10413	Acute Toxicity, Reproductive Effects, Multiple Dose Effects
A10414	Acute Toxicity, Skin and Eye Irritation, Multiple Dose Effects
A10415	Acute Toxicity
A10416	Acute Toxicity, Skin and Eye Irritation, Multiple Dose Effects
A10417	Acute Toxicity
A10418	Acute Toxicity, Mutagenicity, Skin and Eye Irritation, Reproductive Effects, Multiple Dose Effects
A10419	Acute Toxicity, Multiple Dose Effects
A10420	Acute Toxicity, Reproductive Effects, Multiple Dose Effects
A10421	Acute Toxicity, Skin and Eye Irritation, Reproductive Effects, Multiple Dose
A10422	Acute Toxicity
A10423	Acute Toxicity
A10424	Acute Toxicity, Tumorigenicity, Reproductive Effects, Multiple Dose Effects
A10425	Acute Toxicity, Reproductive Effects, Multiple Dose Effects
A10426	Multiple Dose Effects
A10427	Acute Toxicity, Reproductive Effects, Multiple Dose Effects
A10428	Acute Toxicity
A10429	Acute Toxicity, Multiple Dose Effects
A10430	Acute Toxicity, Multiple Dose Effects
A10431	Acute Toxicity, Reproductive Effects, Multiple Dose Effects
A10432	Multiple Dose Effects
A10433	Mutagenicity
A10434	Acute Toxicity
A10435	Acute Toxicity
A10436	Reproductive Effects
A10437	Acute Toxicity, Multiple Dose Effects
A10438	Multiple Dose Effects
A10439	Acute Toxicity, Multiple Dose Effects
A10440	Acute Toxicity, Mutagenicity, Multiple Dose Effects
A10441	Acute Toxicity, Skin and Eye Irritation, Multiple Dose Effects
A10442	Acute Toxicity, Skin and Eye Irritation, Multiple Dose Effects
A10443	Acute Toxicity, Multiple Dose Effects
A10444	Multiple Dose Effects
A10445	Mutagenicity
A10446	Acute Toxicity, Reproductive Effects, Multiple Dose Effects
A10447	Multiple Dose Effects
A10448	Acute Toxicity
A10449	Acute Toxicity, Multiple Dose Effects
A10450	Mutagenicity, Tumorigenicity

A10451	Reproductive Effects, Multiple Dose Effects
A10452	Reproductive Effects, Multiple Dose Effects
A10453	Tumorigenicity, Multiple Dose Effects
A10454	Multiple Dose Effects
A10455	Acute Toxicity, Multiple Dose Effects
A10456	Skin and Eye Irritation, Multiple Dose Effects
A10457	Acute Toxicity, Tumorigenicity, Multiple Dose Effects
A10458	Acute Toxicity, Reproductive Effects, Multiple Dose Effects
A10459	Acute Toxicity
A10460	Mutagenicity
A10461	Mutagenicity
A10462	Acute Toxicity
A10463	Acute Toxicity
A10464	Acute Toxicity
A10465	Acute Toxicity
A10466	Acute Toxicity, Multiple Dose Effects
A10467	Acute Toxicity
A10468	Acute Toxicity
A10469	Mutagenicity
A10470	Mutagenicity
A10471	Acute Toxicity, Multiple Dose Effects
A10472	Mutagenicity
A10473	Acute Toxicity, Mutagenicity, Skin and Eye Irritation
A10474	Acute Toxicity
A10475	Acute Toxicity, Mutagenicity, Reproductive Effects, Multiple Dose Effects
A10476	Acute Toxicity
A10477	Acute Toxicity, Reproductive Effects
A10478	Acute Toxicity
A10479	Acute Toxicity
A10480	Acute Toxicity
A10481	Acute Toxicity
A10482	Mutagenicity
A10483	Acute Toxicity
A10484	Acute Toxicity
A10485	Acute Toxicity
A10486	Tumorigenicity
A10487	Acute Toxicity
A10488	Acute Toxicity
A10489	Skin and Eye Irritation
A10490	Acute Toxicity
A10491	Acute Toxicity, Skin and Eye Irritation
A10492	Acute Toxicity
A10493	Acute Toxicity
A10494	Acute Toxicity
A10495	Acute Toxicity
A10496	Acute Toxicity

A10497	Acute Toxicity
A10498	Acute Toxicity, Reproductive Effects
A10499	Non-toxicity
A10500	Mutagenicity
A10501	Acute Toxicity
A10502	Mutagenicity, Tumorigenicity, Reproductive Effects
A10503	Acute Toxicity
A10504	Acute Toxicity
A10505	Acute Toxicity
A10506	Acute Toxicity
A10507	Acute Toxicity
A10508	Multiple Dose Effects
A10509	Acute Toxicity
A10510	Non-toxicity
A10511	Mutagenicity, Tumorigenicity
A10512	Acute Toxicity
A10513	Acute Toxicity
A10514	Acute Toxicity, Mutagenicity, Reproductive Effects
A10515	Mutagenicity, Tumorigenicity
A10516	Acute Toxicity
A10517	Acute Toxicity
A10518	Acute Toxicity
A10519	Acute Toxicity
A10520	Acute Toxicity
A10521	Mutagenicity, Tumorigenicity
A10522	Acute Toxicity
A10523	Acute Toxicity
A10524	Acute Toxicity
A10525	Acute Toxicity, Mutagenicity, Multiple Dose Effects
A10526	Acute Toxicity
A10527	Acute Toxicity, Mutagenicity, Reproductive Effects, Multiple Dose Effects
A10528	Mutagenicity
A10529	Acute Toxicity, Multiple Dose Effects
A10530	Mutagenicity
A10531	Mutagenicity
A10532	Acute Toxicity, Mutagenicity, Tumorigenicity, Skin and Eye Irritation,
A10533	Acute Toxicity
A10534	Acute Toxicity
A10535	Acute Toxicity
A10536	Acute Toxicity
A10537	Acute Toxicity
A10538	Acute Toxicity, Reproductive Effects, Multiple Dose Effects
A10539	Acute Toxicity
A10540	Mutagenicity
A10541	Acute Toxicity
A10542	Acute Toxicity

A10543	Acute Toxicity
A10544	Non-toxicity
A10545	Acute Toxicity, Skin and Eye Irritation
A10546	Acute Toxicity, Multiple Dose Effects
A10547	Acute Toxicity, Mutagenicity, Tumorigenicity
A10548	Acute Toxicity
A10549	Acute Toxicity
A10550	Acute Toxicity
A10551	Acute Toxicity, Mutagenicity
A10552	Skin and Eye Irritation
A10553	Tumorigenicity
A10554	Acute Toxicity
A10555	Skin and Eye Irritation
A10556	Acute Toxicity, Skin and Eye Irritation, Multiple Dose Effects
A10557	Multiple Dose Effects
A10558	Acute Toxicity
A10559	Mutagenicity
A10560	Acute Toxicity
A10561	Acute Toxicity, Mutagenicity, Tumorigenicity
A10562	Acute Toxicity, Reproductive Effects
A10563	Acute Toxicity
A10564	Acute Toxicity
A10565	Acute Toxicity, Multiple Dose Effects
A10566	Acute Toxicity
A10567	Acute Toxicity
A10568	Acute Toxicity
A10569	Acute Toxicity
A10570	Acute Toxicity
A10571	Acute Toxicity
A10572	Acute Toxicity
A10573	Acute Toxicity, Skin and Eye Irritation
A10574	Acute Toxicity
A10575	Acute Toxicity, Mutagenicity
A10576	Acute Toxicity, Mutagenicity
A10577	Acute Toxicity, Mutagenicity
A10578	Reproductive Effects
A10579	Mutagenicity
A10580	Reproductive Effects, Multiple Dose Effects
A10581	Mutagenicity, Reproductive Effects
A10582	Mutagenicity
A10583	Acute Toxicity, Multiple Dose Effects
A10584	Reproductive Effects
A10585	Acute Toxicity
A10586	Acute Toxicity
A10587	Acute Toxicity
A10588	Acute Toxicity

A10589	Acute Toxicity, Mutagenicity, Multiple Dose Effects
A10590	Acute Toxicity, Tumorigenicity
A10591	Acute Toxicity, Multiple Dose Effects
A10592	Acute Toxicity, Multiple Dose Effects
A10593	Acute Toxicity
A10594	Acute Toxicity, Mutagenicity, Reproductive Effects, Multiple Dose Effects
A10595	Acute Toxicity
A10596	Mutagenicity, Tumorigenicity
A10597	Mutagenicity
A10598	Acute Toxicity
A10599	Acute Toxicity
A10600	Acute Toxicity, Mutagenicity, Tumorigenicity
A10601	Non-toxicity
A10602	Mutagenicity
A10603	Mutagenicity, Skin and Eye Irritation
A10604	Acute Toxicity
A10605	Acute Toxicity
A10606	Acute Toxicity, Mutagenicity, Multiple Dose Effects
A10607	Acute Toxicity, Reproductive Effects
A10608	Acute Toxicity
A10609	Mutagenicity
A10610	Mutagenicity
A10611	Mutagenicity, Tumorigenicity
A10612	Acute Toxicity, Mutagenicity, Skin and Eye Irritation, Reproductive Effects, Multiple Dose Effects
A10613	Mutagenicity, Skin and Eye Irritation
A10614	Acute Toxicity, Mutagenicity
A10615	Acute Toxicity
A10616	Acute Toxicity
A10617	Acute Toxicity, Skin and Eye Irritation
A10618	Acute Toxicity
A10619	Mutagenicity, Multiple Dose Effects
A10620	Mutagenicity
A10621	Acute Toxicity, Mutagenicity, Reproductive Effects, Multiple Dose Effects
A10622	Acute Toxicity, Multiple Dose Effects
A10623	Acute Toxicity, Mutagenicity, Reproductive Effects, Multiple Dose Effects
A10624	Acute Toxicity
A10625	Non-toxicity
A10626	Acute Toxicity, Mutagenicity, Reproductive Effects
A10627	Acute Toxicity, Mutagenicity
A10628	Mutagenicity, Tumorigenicity
A10629	Mutagenicity, Tumorigenicity
A10630	Acute Toxicity, Reproductive Effects
A10631	Acute Toxicity, Multiple Dose Effects
A10632	Acute Toxicity
A10633	Mutagenicity

A10634	Acute Toxicity, Mutagenicity
A10635	Skin and Eye Irritation
A10636	Mutagenicity, Tumorigenicity, Multiple Dose Effects
A10637	Acute Toxicity
A10638	Acute Toxicity, Reproductive Effects, Multiple Dose Effects
A10639	Mutagenicity, Tumorigenicity
A10640	Acute Toxicity, Skin and Eye Irritation
A10641	Mutagenicity, Tumorigenicity
A10642	Acute Toxicity, Mutagenicity, Reproductive Effects, Multiple Dose Effects
A10643	Tumorigenicity, Multiple Dose Effects
A10644	Skin and Eye Irritation
A10645	Acute Toxicity, Mutagenicity
A10646	Acute Toxicity
A10647	Acute Toxicity, Mutagenicity, Tumorigenicity
A10648	Acute Toxicity, Mutagenicity, Tumorigenicity
A10649	Acute Toxicity
A10650	Non-toxicity
A10651	Acute Toxicity, Mutagenicity, Reproductive Effects, Multiple Dose Effects
A10652	Acute Toxicity, Skin and Eye Irritation, Multiple Dose Effects
A10653	Mutagenicity, Tumorigenicity
A10654	Mutagenicity, Tumorigenicity
A10655	Reproductive Effects, Multiple Dose Effects
A10656	Reproductive Effects, Multiple Dose Effects
A10657	Acute Toxicity, Multiple Dose Effects
A10658	Acute Toxicity, Multiple Dose Effects
A10659	Acute Toxicity
A10660	Acute Toxicity
A10661	Mutagenicity, Tumorigenicity
A10662	Acute Toxicity, Mutagenicity, Reproductive Effects, Multiple Dose Effects
A10663	Acute Toxicity, Reproductive Effects
A10664	Acute Toxicity, Skin and Eye Irritation, Reproductive Effects, Multiple Dose
A10665	Mutagenicity
A10666	Acute Toxicity
A10667	Acute Toxicity, Mutagenicity, Tumorigenicity
A10668	Acute Toxicity
A10669	Acute Toxicity
A10670	Acute Toxicity, Reproductive Effects, Multiple Dose Effects
A10671	Skin and Eye Irritation
A10672	Acute Toxicity, Multiple Dose Effects
A10673	Acute Toxicity, Multiple Dose Effects
A10674	Acute Toxicity, Reproductive Effects, Multiple Dose Effects
A10675	Skin and Eye Irritation
A10676	Acute Toxicity, Multiple Dose Effects
A10677	Acute Toxicity, Reproductive Effects, Multiple Dose Effects
A10678	Acute Toxicity, Mutagenicity, Skin and Eye Irritation
A10679	Multiple Dose Effects

A10680	Acute Toxicity
A10681	Acute Toxicity, Mutagenicity, Skin and Eye Irritation, Multiple Dose Effects
A10682	Mutagenicity, Tumorigenicity
A10683	Acute Toxicity
A10684	Acute Toxicity, Reproductive Effects, Multiple Dose Effects
A10685	Acute Toxicity, Multiple Dose Effects
A10686	Acute Toxicity
A10687	Acute Toxicity
A10688	Acute Toxicity, Skin and Eye Irritation, Reproductive Effects, Multiple Dose
A10689	Acute Toxicity, Multiple Dose Effects
A10690	Acute Toxicity, Reproductive Effects, Multiple Dose Effects
A10691	Acute Toxicity, Reproductive Effects, Multiple Dose Effects
A10692	Acute Toxicity, Reproductive Effects, Multiple Dose Effects
A10693	Acute Toxicity
A10694	Acute Toxicity, Multiple Dose Effects
A10695	Acute Toxicity, Reproductive Effects
A10696	Acute Toxicity, Reproductive Effects, Multiple Dose Effects
A10697	Acute Toxicity, Multiple Dose Effects
A10698	Acute Toxicity
A10699	Acute Toxicity
A10700	Reproductive Effects, Multiple Dose Effects
A10701	Acute Toxicity
A10702	Acute Toxicity, Reproductive Effects, Multiple Dose Effects
A10703	Acute Toxicity, Reproductive Effects, Multiple Dose Effects
A10704	Acute Toxicity, Tumorigenicity, Reproductive Effects
A10705	Acute Toxicity, Multiple Dose Effects
A10706	Acute Toxicity, Skin and Eye Irritation, Multiple Dose Effects
A10707	Acute Toxicity, Multiple Dose Effects
A10708	Acute Toxicity
A10709	Acute Toxicity, Multiple Dose Effects
A10710	Acute Toxicity
A10711	Acute Toxicity
A10712	Acute Toxicity, Reproductive Effects, Multiple Dose Effects
A10713	Tumorigenicity
A10714	Acute Toxicity, Tumorigenicity, Reproductive Effects, Multiple Dose Effects
A10715	Acute Toxicity, Mutagenicity
A10716	Mutagenicity
A10717	Reproductive Effects
A10718	Acute Toxicity
A10719	Acute Toxicity
A10720	Acute Toxicity, Skin and Eye Irritation
A10721	Reproductive Effects, Multiple Dose Effects
A10722	Acute Toxicity, Multiple Dose Effects
A10723	Acute Toxicity
A10724	Mutagenicity
A10725	Acute Toxicity

A10726	Mutagenicity
A10727	Non-toxicity
A10728	Acute Toxicity
A10729	Mutagenicity
A10730	Acute Toxicity
A10731	Acute Toxicity
A10732	Mutagenicity
A10733	Non-toxicity
A10734	Mutagenicity
A10735	Non-toxicity
A10736	Mutagenicity
A10737	Acute Toxicity, Reproductive Effects
A10738	Acute Toxicity, Reproductive Effects
A10739	Reproductive Effects
A10740	Skin and Eye Irritation, Reproductive Effects, Multiple Dose Effects
A10741	Mutagenicity
A10742	Mutagenicity
A10743	Tumorigenicity, Multiple Dose Effects
A10744	Mutagenicity
A10745	Acute Toxicity
A10746	Acute Toxicity
A10747	Acute Toxicity
A10748	Acute Toxicity, Multiple Dose Effects
A10749	Acute Toxicity, Mutagenicity
A10750	Acute Toxicity, Mutagenicity, Multiple Dose Effects
A10751	Reproductive Effects
A10752	Mutagenicity
A10753	Acute Toxicity, Mutagenicity
A10754	Mutagenicity
A10755	Acute Toxicity
A10756	Acute Toxicity
A10757	Acute Toxicity
A10758	Reproductive Effects, Multiple Dose Effects
A10759	Acute Toxicity
A10760	Acute Toxicity
A10761	Mutagenicity
A10762	Mutagenicity
A10763	Acute Toxicity
A10764	Acute Toxicity
A10765	Tumorigenicity
A10766	Acute Toxicity
A10767	Acute Toxicity, Mutagenicity, Tumorigenicity
A10768	Acute Toxicity, Reproductive Effects, Multiple Dose Effects
A10769	Mutagenicity, Multiple Dose Effects
A10770	Mutagenicity
A10771	Mutagenicity, Tumorigenicity

A10772	Mutagenicity, Tumorigenicity
A10773	Mutagenicity
A10774	Mutagenicity, Tumorigenicity
A10775	Mutagenicity, Tumorigenicity
A10776	Acute Toxicity
A10777	Acute Toxicity
A10778	Acute Toxicity
A10779	Acute Toxicity, Multiple Dose Effects
A10780	Acute Toxicity, Multiple Dose Effects
A10781	Acute Toxicity, Reproductive Effects, Multiple Dose Effects
A10782	Multiple Dose Effects
A10783	Acute Toxicity, Reproductive Effects, Multiple Dose Effects
A10784	Acute Toxicity
A10785	Acute Toxicity
A10786	Mutagenicity
A10787	Mutagenicity, Tumorigenicity, Multiple Dose Effects
A10788	Acute Toxicity, Skin and Eye Irritation
A10789	Non-toxicity
A10790	Acute Toxicity
A10791	Acute Toxicity
A10792	Acute Toxicity
A10793	Acute Toxicity
A10794	Reproductive Effects
A10795	Acute Toxicity
A10796	Acute Toxicity
A10797	Acute Toxicity, Multiple Dose Effects
A10798	Acute Toxicity
A10799	Acute Toxicity
A10800	Acute Toxicity
A10801	Acute Toxicity
A10802	Acute Toxicity
A10803	Non-toxicity
A10804	Mutagenicity, Multiple Dose Effects
A10805	Acute Toxicity
A10806	Acute Toxicity, Reproductive Effects
A10807	Reproductive Effects
A10808	Mutagenicity
A10809	Acute Toxicity, Reproductive Effects
A10810	Non-toxicity
A10811	Acute Toxicity, Mutagenicity
A10812	Non-toxicity
A10813	Non-toxicity
A10814	Acute Toxicity
A10815	Acute Toxicity
A10816	Acute Toxicity
A10817	Acute Toxicity

A10818	Acute Toxicity
A10819	Reproductive Effects
A10820	Mutagenicity
A10821	Acute Toxicity
A10822	Acute Toxicity
A10823	Acute Toxicity, Skin and Eye Irritation, Multiple Dose Effects
A10824	Acute Toxicity
A10825	Mutagenicity
A10826	Skin and Eye Irritation, Multiple Dose Effects
A10827	Mutagenicity
A10828	Acute Toxicity, Mutagenicity
A10829	Acute Toxicity
A10830	Acute Toxicity, Multiple Dose Effects
A10831	Mutagenicity
A10832	Acute Toxicity, Mutagenicity, Skin and Eye Irritation
A10833	Acute Toxicity
A10834	Non-toxicity
A10835	Mutagenicity
A10836	Acute Toxicity, Reproductive Effects
A10837	Acute Toxicity
A10838	Acute Toxicity, Skin and Eye Irritation
A10839	Mutagenicity
A10840	Multiple Dose Effects
A10841	Mutagenicity
A10842	Acute Toxicity, Reproductive Effects, Multiple Dose Effects
A10843	Mutagenicity
A10844	Acute Toxicity, Mutagenicity, Tumorigenicity
A10845	Multiple Dose Effects
A10846	Acute Toxicity
A10847	Acute Toxicity
A10848	Acute Toxicity, Reproductive Effects, Multiple Dose Effects
A10849	Acute Toxicity
A10850	Acute Toxicity, Mutagenicity, Skin and Eye Irritation, Multiple Dose Effects
A10851	Acute Toxicity, Mutagenicity, Tumorigenicity
A10852	Mutagenicity, Tumorigenicity
A10853	Acute Toxicity, Reproductive Effects
A10854	Acute Toxicity, Mutagenicity
A10855	Acute Toxicity
A10856	Acute Toxicity, Mutagenicity, Tumorigenicity
A10857	Mutagenicity
A10858	Mutagenicity
A10859	Reproductive Effects
A10860	Acute Toxicity
A10861	Acute Toxicity, Mutagenicity, Tumorigenicity
A10862	Mutagenicity
A10863	Acute Toxicity

A10864	Tumorigenicity
A10865	Mutagenicity
A10866	Acute Toxicity, Reproductive Effects, Multiple Dose Effects
A10867	Acute Toxicity, Multiple Dose Effects
A10868	Acute Toxicity, Skin and Eye Irritation, Reproductive Effects
A10869	Acute Toxicity, Mutagenicity, Reproductive Effects, Multiple Dose Effects
A10870	Acute Toxicity, Mutagenicity
A10871	Acute Toxicity
A10872	Mutagenicity
A10873	Acute Toxicity, Skin and Eye Irritation, Reproductive Effects
A10874	Acute Toxicity, Mutagenicity, Skin and Eye Irritation, Reproductive Effects
A10875	Acute Toxicity, Reproductive Effects, Multiple Dose Effects
A10876	Acute Toxicity, Reproductive Effects, Multiple Dose Effects
A10877	Acute Toxicity, Skin and Eye Irritation
A10878	Acute Toxicity
A10879	Acute Toxicity
A10880	Multiple Dose Effects
A10881	Acute Toxicity
A10882	Multiple Dose Effects
A10883	Acute Toxicity
A10884	Acute Toxicity
A10885	Acute Toxicity, Multiple Dose Effects
A10886	Reproductive Effects, Multiple Dose Effects
A10887	Acute Toxicity, Mutagenicity
A10888	Multiple Dose Effects
A10889	Acute Toxicity
A10890	Acute Toxicity
A10891	Mutagenicity
A10892	Acute Toxicity
A10893	Acute Toxicity
A10894	Acute Toxicity
A10895	Acute Toxicity
A10896	Acute Toxicity
A10897	Acute Toxicity, Mutagenicity, Reproductive Effects
A10898	Acute Toxicity
A10899	Reproductive Effects
A10900	Mutagenicity
A10901	Acute Toxicity
A10902	Acute Toxicity
A10903	Acute Toxicity
A10904	Acute Toxicity, Skin and Eye Irritation, Multiple Dose Effects
A10905	Skin and Eye Irritation
A10906	Mutagenicity
A10907	Acute Toxicity
A10908	Acute Toxicity, Skin and Eye Irritation
A10909	Multiple Dose Effects

A10910	Acute Toxicity, Reproductive Effects, Multiple Dose Effects
A10911	Acute Toxicity, Multiple Dose Effects
A10912	Acute Toxicity
A10913	Acute Toxicity
A10914	Tumorigenicity
A10915	Acute Toxicity
A10916	Non-toxicity
A10917	Acute Toxicity
A10918	Acute Toxicity, Mutagenicity, Multiple Dose Effects
A10919	Acute Toxicity
A10920	Acute Toxicity
A10921	Acute Toxicity, Mutagenicity
A10922	Acute Toxicity, Multiple Dose Effects
A10923	Skin and Eye Irritation
A10924	Acute Toxicity
A10925	Acute Toxicity, Reproductive Effects, Multiple Dose Effects
A10926	Reproductive Effects, Multiple Dose Effects
A10927	Mutagenicity
A10928	Acute Toxicity
A10929	Mutagenicity
A10930	Skin and Eye Irritation
A10931	Mutagenicity
A10932	Mutagenicity
A10933	Acute Toxicity, Skin and Eye Irritation, Reproductive Effects, Multiple Dose
A10934	Acute Toxicity
A10935	Acute Toxicity
A10936	Acute Toxicity
A10937	Acute Toxicity
A10938	Acute Toxicity
A10939	Acute Toxicity
A10940	Acute Toxicity, Mutagenicity
A10941	Acute Toxicity
A10942	Mutagenicity
A10943	Mutagenicity
A10944	Acute Toxicity, Skin and Eye Irritation
A10945	Mutagenicity
A10946	Mutagenicity
A10947	Reproductive Effects, Multiple Dose Effects
A10948	Mutagenicity
A10949	Acute Toxicity
A10950	Acute Toxicity
A10951	Mutagenicity
A10952	Acute Toxicity
A10953	Acute Toxicity
A10954	Mutagenicity
A10955	Acute Toxicity

A10956	Acute Toxicity
A10957	Acute Toxicity
A10958	Acute Toxicity
A10959	Mutagenicity
A10960	Acute Toxicity
A10961	Non-toxicity
A10962	Mutagenicity
A10963	Acute Toxicity
A10964	Acute Toxicity
A10965	Acute Toxicity, Multiple Dose Effects
A10966	Acute Toxicity, Multiple Dose Effects
A10967	Acute Toxicity, Mutagenicity, Reproductive Effects, Multiple Dose Effects
A10968	Acute Toxicity
A10969	Acute Toxicity
A10970	Acute Toxicity
A10971	Acute Toxicity
A10972	Acute Toxicity
A10973	Acute Toxicity
A10974	Mutagenicity, Tumorigenicity
A10975	Acute Toxicity
A10976	Acute Toxicity
A10977	Acute Toxicity
A10978	Acute Toxicity
A10979	Acute Toxicity, Skin and Eye Irritation
A10980	Acute Toxicity
A10981	Acute Toxicity
A10982	Acute Toxicity
A10983	Acute Toxicity
A10984	Acute Toxicity
A10985	Acute Toxicity
A10986	Acute Toxicity
A10987	Acute Toxicity, Mutagenicity
A10988	Acute Toxicity, Mutagenicity, Tumorigenicity
A10989	Mutagenicity
A10990	Acute Toxicity
A10991	Mutagenicity
A10992	Acute Toxicity, Mutagenicity
A10993	Acute Toxicity, Mutagenicity
A10994	Acute Toxicity
A10995	Acute Toxicity
A10996	Tumorigenicity
A10997	Acute Toxicity
A10998	Mutagenicity
A10999	Acute Toxicity
A11000	Acute Toxicity, Multiple Dose Effects
A11001	Acute Toxicity

A11002	Acute Toxicity
A11003	Acute Toxicity
A11004	Acute Toxicity
A11005	Mutagenicity, Tumorigenicity
A11006	Acute Toxicity
A11007	Acute Toxicity
A11008	Mutagenicity
A11009	Acute Toxicity
A11010	Reproductive Effects
A11011	Mutagenicity
A11012	Mutagenicity, Tumorigenicity
A11013	Acute Toxicity
A11014	Mutagenicity
A11015	Reproductive Effects
A11016	Acute Toxicity
A11017	Acute Toxicity
A11018	Mutagenicity
A11019	Acute Toxicity
A11020	Mutagenicity, Tumorigenicity
A11021	Mutagenicity
A11022	Acute Toxicity, Mutagenicity, Reproductive Effects
A11023	Acute Toxicity, Mutagenicity
A11024	Acute Toxicity
A11025	Mutagenicity, Tumorigenicity
A11026	Mutagenicity
A11027	Mutagenicity, Multiple Dose Effects
A11028	Mutagenicity
A11029	Acute Toxicity
A11030	Reproductive Effects
A11031	Mutagenicity
A11032	Multiple Dose Effects
A11033	Acute Toxicity, Mutagenicity
A11034	Mutagenicity
A11035	Acute Toxicity
A11036	Mutagenicity, Tumorigenicity
A11037	Acute Toxicity
A11038	Acute Toxicity, Mutagenicity
A11039	Mutagenicity
A11040	Mutagenicity
A11041	Acute Toxicity
A11042	Acute Toxicity
A11043	Acute Toxicity
A11044	Mutagenicity
A11045	Acute Toxicity
A11046	Mutagenicity
A11047	Acute Toxicity

A11048	Mutagenicity
A11049	Mutagenicity, Tumorigenicity
A11050	Acute Toxicity
A11051	Acute Toxicity
A11052	Acute Toxicity
A11053	Mutagenicity
A11054	Mutagenicity
A11055	Acute Toxicity, Mutagenicity
A11056	Mutagenicity
A11057	Acute Toxicity
A11058	Mutagenicity
A11059	Mutagenicity
A11060	Acute Toxicity
A11061	Acute Toxicity
A11062	Acute Toxicity, Multiple Dose Effects
A11063	Skin and Eye Irritation
A11064	Non-toxicity
A11065	Acute Toxicity
A11066	Mutagenicity
A11067	Acute Toxicity
A11068	Mutagenicity
A11069	Mutagenicity
A11070	Mutagenicity
A11071	Acute Toxicity
A11072	Mutagenicity, Multiple Dose Effects
A11073	Mutagenicity
A11074	Acute Toxicity
A11075	Acute Toxicity
A11076	Reproductive Effects
A11077	Acute Toxicity
A11078	Acute Toxicity
A11079	Acute Toxicity
A11080	Acute Toxicity
A11081	Mutagenicity
A11082	Mutagenicity
A11083	Tumorigenicity
A11084	Acute Toxicity, Skin and Eye Irritation
A11085	Acute Toxicity
A11086	Acute Toxicity, Multiple Dose Effects
A11087	Acute Toxicity
A11088	Reproductive Effects
A11089	Tumorigenicity
A11090	Mutagenicity
A11091	Mutagenicity
A11092	Acute Toxicity, Mutagenicity
A11093	Acute Toxicity

A11094	Acute Toxicity
A11095	Acute Toxicity
A11096	Acute Toxicity
A11097	Acute Toxicity
A11098	Acute Toxicity
A11099	Acute Toxicity
A11100	Acute Toxicity
A11101	Acute Toxicity, Mutagenicity
A11102	Acute Toxicity
A11103	Mutagenicity, Tumorigenicity
A11104	Mutagenicity
A11105	Acute Toxicity
A11106	Acute Toxicity
A11107	Mutagenicity
A11108	Non-toxicity
A11109	Acute Toxicity
A11110	Acute Toxicity
A11111	Mutagenicity
A11112	Acute Toxicity
A11113	Acute Toxicity
A11114	Acute Toxicity
A11115	Acute Toxicity
A11116	Acute Toxicity
A11117	Acute Toxicity
A11118	Acute Toxicity, Multiple Dose Effects
A11119	Acute Toxicity
A11120	Acute Toxicity
A11121	Acute Toxicity, Mutagenicity
A11122	Acute Toxicity
A11123	Non-toxicity
A11124	Acute Toxicity
A11125	Acute Toxicity
A11126	Acute Toxicity
A11127	Mutagenicity
A11128	Mutagenicity
A11129	Non-toxicity
A11130	Reproductive Effects
A11131	Acute Toxicity, Mutagenicity
A11132	Acute Toxicity, Multiple Dose Effects
A11133	Tumorigenicity
A11134	Acute Toxicity
A11135	Mutagenicity
A11136	Tumorigenicity
A11137	Acute Toxicity
A11138	Mutagenicity
A11139	Acute Toxicity

A11140	Acute Toxicity, Mutagenicity
A11141	Acute Toxicity
A11142	Acute Toxicity
A11143	Mutagenicity
A11144	Acute Toxicity, Skin and Eye Irritation
A11145	Acute Toxicity
A11146	Acute Toxicity
A11147	Non-toxicity
A11148	Non-toxicity
A11149	Acute Toxicity, Mutagenicity
A11150	Acute Toxicity
A11151	Acute Toxicity
A11152	Acute Toxicity
A11153	Acute Toxicity, Reproductive Effects
A11154	Mutagenicity
A11155	Acute Toxicity
A11156	Multiple Dose Effects
A11157	Acute Toxicity
A11158	Acute Toxicity, Tumorigenicity, Multiple Dose Effects
A11159	Multiple Dose Effects
A11160	Acute Toxicity, Mutagenicity
A11161	Mutagenicity
A11162	Acute Toxicity
A11163	Acute Toxicity
A11164	Tumorigenicity
A11165	Acute Toxicity
A11166	Acute Toxicity, Mutagenicity
A11167	Acute Toxicity, Mutagenicity
A11168	Mutagenicity, Multiple Dose Effects
A11169	Acute Toxicity
A11170	Non-toxicity
A11171	Mutagenicity
A11172	Mutagenicity, Tumorigenicity
A11173	Mutagenicity
A11174	Acute Toxicity
A11175	Acute Toxicity
A11176	Acute Toxicity
A11177	Acute Toxicity, Mutagenicity
A11178	Mutagenicity
A11179	Mutagenicity
A11180	Acute Toxicity, Mutagenicity, Multiple Dose Effects
A11181	Mutagenicity
A11182	Reproductive Effects
A11183	Mutagenicity
A11184	Acute Toxicity
A11185	Acute Toxicity, Mutagenicity

A11186	Acute Toxicity
A11187	Mutagenicity
A11188	Acute Toxicity, Mutagenicity, Reproductive Effects
A11189	Acute Toxicity, Multiple Dose Effects
A11190	Acute Toxicity
A11191	Tumorigenicity, Multiple Dose Effects
A11192	Mutagenicity
A11193	Acute Toxicity, Reproductive Effects, Multiple Dose Effects
A11194	Mutagenicity
A11195	Reproductive Effects
A11196	Acute Toxicity, Mutagenicity
A11197	Acute Toxicity
A11198	Mutagenicity
A11199	Acute Toxicity
A11200	Acute Toxicity, Multiple Dose Effects
A11201	Mutagenicity
A11202	Multiple Dose Effects
A11203	Mutagenicity
A11204	Acute Toxicity
A11205	Acute Toxicity, Mutagenicity, Reproductive Effects, Multiple Dose Effects
A11206	Non-toxicity
A11207	Mutagenicity
A11208	Mutagenicity
A11209	Acute Toxicity
A11210	Acute Toxicity
A11211	Acute Toxicity, Mutagenicity
A11212	Skin and Eye Irritation
A11213	Acute Toxicity
A11214	Mutagenicity
A11215	Mutagenicity
A11216	Mutagenicity
A11217	Acute Toxicity
A11218	Acute Toxicity
A11219	Acute Toxicity
A11220	Acute Toxicity
A11221	Acute Toxicity
A11222	Mutagenicity
A11223	Mutagenicity
A11224	Acute Toxicity
A11225	Mutagenicity
A11226	Acute Toxicity
A11227	Reproductive Effects
A11228	Multiple Dose Effects
A11229	Multiple Dose Effects
A11230	Acute Toxicity
A11231	Acute Toxicity

A11232	Acute Toxicity
A11233	Acute Toxicity, Mutagenicity
A11234	Multiple Dose Effects
A11235	Acute Toxicity, Multiple Dose Effects
A11236	Mutagenicity, Multiple Dose Effects
A11237	Acute Toxicity, Reproductive Effects
A11238	Acute Toxicity, Skin and Eye Irritation
A11239	Acute Toxicity
A11240	Acute Toxicity
A11241	Tumorigenicity
A11242	Acute Toxicity
A11243	Skin and Eye Irritation
A11244	Acute Toxicity
A11245	Acute Toxicity
A11246	Acute Toxicity
A11247	Acute Toxicity, Reproductive Effects
A11248	Acute Toxicity
A11249	Acute Toxicity, Multiple Dose Effects
A11250	Non-toxicity
A11251	Reproductive Effects
A11252	Multiple Dose Effects
A11253	Acute Toxicity, Reproductive Effects
A11254	Acute Toxicity
A11255	Acute Toxicity
A11256	Acute Toxicity
A11257	Acute Toxicity
A11258	Tumorigenicity
A11259	Acute Toxicity
A11260	Acute Toxicity
A11261	Acute Toxicity
A11262	Acute Toxicity
A11263	Acute Toxicity
A11264	Acute Toxicity
A11265	Acute Toxicity, Multiple Dose Effects
A11266	Acute Toxicity, Reproductive Effects
A11267	Tumorigenicity
A11268	Acute Toxicity
A11269	Acute Toxicity
A11270	Acute Toxicity
A11271	Acute Toxicity
A11272	Acute Toxicity
A11273	Acute Toxicity
A11274	Mutagenicity
A11275	Acute Toxicity
A11276	Acute Toxicity, Mutagenicity, Skin and Eye Irritation
A11277	Acute Toxicity, Reproductive Effects, Multiple Dose Effects

A11278	Mutagenicity, Tumorigenicity
A11279	Acute Toxicity, Mutagenicity, Tumorigenicity, Reproductive Effects,
A11280	Mutagenicity, Tumorigenicity
A11281	Mutagenicity
A11282	Mutagenicity
A11283	Non-toxicity
A11284	Acute Toxicity, Mutagenicity, Reproductive Effects, Multiple Dose Effects
A11285	Acute Toxicity, Mutagenicity, Tumorigenicity, Multiple Dose Effects
A11286	Acute Toxicity
A11287	Acute Toxicity, Mutagenicity
A11288	Acute Toxicity, Multiple Dose Effects
A11289	Mutagenicity, Tumorigenicity
A11290	Acute Toxicity, Multiple Dose Effects
A11291	Acute Toxicity, Multiple Dose Effects
A11292	Acute Toxicity, Mutagenicity, Multiple Dose Effects
A11293	Acute Toxicity, Mutagenicity
A11294	Mutagenicity
A11295	Multiple Dose Effects
A11296	Acute Toxicity, Multiple Dose Effects
A11297	Acute Toxicity, Multiple Dose Effects
A11298	Mutagenicity
A11299	Mutagenicity, Tumorigenicity
A11300	Acute Toxicity, Mutagenicity
A11301	Acute Toxicity, Reproductive Effects, Multiple Dose Effects
A11302	Acute Toxicity
A11303	Acute Toxicity, Reproductive Effects
A11304	Acute Toxicity, Mutagenicity, Multiple Dose Effects
A11305	Mutagenicity, Tumorigenicity
A11306	Mutagenicity
A11307	Acute Toxicity
A11308	Acute Toxicity, Mutagenicity, Tumorigenicity
A11309	Acute Toxicity, Mutagenicity, Tumorigenicity, Reproductive Effects,
A11310	Mutagenicity, Tumorigenicity
A11311	Acute Toxicity, Mutagenicity, Reproductive Effects, Multiple Dose Effects
A11312	Acute Toxicity, Multiple Dose Effects
A11313	Mutagenicity, Tumorigenicity
A11314	Mutagenicity
A11315	Mutagenicity, Tumorigenicity
A11316	Acute Toxicity, Multiple Dose Effects
A11317	Mutagenicity, Tumorigenicity
A11318	Mutagenicity, Tumorigenicity
A11319	Mutagenicity
A11320	Mutagenicity, Tumorigenicity
A11321	Mutagenicity
A11322	Acute Toxicity, Reproductive Effects
A11323	Acute Toxicity

A11324	Acute Toxicity
A11325	Acute Toxicity, Multiple Dose Effects
A11326	Acute Toxicity, Multiple Dose Effects
A11327	Acute Toxicity, Mutagenicity
A11328	Mutagenicity
A11329	Tumorigenicity
A11330	Acute Toxicity, Mutagenicity, Tumorigenicity, Multiple Dose Effects
A11331	Acute Toxicity
A11332	Mutagenicity
A11333	Mutagenicity
A11334	Acute Toxicity
A11335	Mutagenicity
A11336	Mutagenicity, Tumorigenicity
A11337	Acute Toxicity, Multiple Dose Effects
A11338	Acute Toxicity
A11339	Mutagenicity, Tumorigenicity, Multiple Dose Effects
A11340	Mutagenicity
A11341	Mutagenicity
A11342	Mutagenicity
A11343	Tumorigenicity
A11344	Acute Toxicity
A11345	Acute Toxicity
A11346	Mutagenicity, Reproductive Effects
A11347	Acute Toxicity, Mutagenicity
A11348	Mutagenicity, Tumorigenicity
A11349	Acute Toxicity
A11350	Acute Toxicity
A11351	Acute Toxicity
A11352	Acute Toxicity
A11353	Tumorigenicity
A11354	Acute Toxicity, Skin and Eye Irritation
A11355	Acute Toxicity
A11356	Acute Toxicity
A11357	Acute Toxicity
A11358	Acute Toxicity
A11359	Acute Toxicity
A11360	Multiple Dose Effects
A11361	Acute Toxicity, Skin and Eye Irritation
A11362	Acute Toxicity
A11363	Non-toxicity
A11364	Acute Toxicity
A11365	Acute Toxicity
A11366	Mutagenicity
A11367	Acute Toxicity, Mutagenicity
A11368	Acute Toxicity
A11369	Acute Toxicity

A11370	Acute Toxicity, Multiple Dose Effects
A11371	Reproductive Effects, Multiple Dose Effects
A11372	Acute Toxicity
A11373	Non-toxicity
A11374	Acute Toxicity
A11375	Mutagenicity, Tumorigenicity
A11376	Acute Toxicity
A11377	Mutagenicity
A11378	Mutagenicity
A11379	Acute Toxicity, Mutagenicity
A11380	Acute Toxicity
A11381	Acute Toxicity, Reproductive Effects, Multiple Dose Effects
A11382	Mutagenicity, Tumorigenicity
A11383	Acute Toxicity, Tumorigenicity
A11384	Acute Toxicity, Multiple Dose Effects
A11385	Acute Toxicity, Mutagenicity
A11386	Acute Toxicity
A11387	Mutagenicity
A11388	Mutagenicity
A11389	Mutagenicity
A11390	Acute Toxicity
A11391	Mutagenicity, Tumorigenicity
A11392	Reproductive Effects
A11393	Acute Toxicity
A11394	Acute Toxicity, Mutagenicity, Multiple Dose Effects
A11395	Acute Toxicity, Reproductive Effects, Multiple Dose Effects
A11396	Mutagenicity, Tumorigenicity
A11397	Multiple Dose Effects
A11398	Mutagenicity
A11399	Multiple Dose Effects
A11400	Non-toxicity
A11401	Acute Toxicity
A11402	Mutagenicity
A11403	Acute Toxicity
A11404	Acute Toxicity
A11405	Mutagenicity
A11406	Skin and Eye Irritation
A11407	Acute Toxicity
A11408	Acute Toxicity, Mutagenicity, Tumorigenicity, Multiple Dose Effects
A11409	Acute Toxicity
A11410	Non-toxicity
A11411	Tumorigenicity
A11412	Mutagenicity
A11413	Mutagenicity, Tumorigenicity
A11414	Acute Toxicity
A11415	Acute Toxicity, Multiple Dose Effects

A11416	Acute Toxicity, Multiple Dose Effects
A11417	Mutagenicity, Tumorigenicity
A11418	Mutagenicity
A11419	Acute Toxicity
A11420	Acute Toxicity, Multiple Dose Effects
A11421	Acute Toxicity, Tumorigenicity, Multiple Dose Effects
A11422	Mutagenicity
A11423	Mutagenicity
A11424	Acute Toxicity, Mutagenicity
A11425	Acute Toxicity
A11426	Mutagenicity, Tumorigenicity
A11427	Acute Toxicity
A11428	Acute Toxicity, Mutagenicity
A11429	Acute Toxicity
A11430	Acute Toxicity, Reproductive Effects, Multiple Dose Effects
A11431	Acute Toxicity, Tumorigenicity
A11432	Acute Toxicity, Mutagenicity
A11433	Acute Toxicity
A11434	Acute Toxicity, Mutagenicity, Reproductive Effects
A11435	Mutagenicity
A11436	Acute Toxicity, Mutagenicity, Multiple Dose Effects
A11437	Mutagenicity, Tumorigenicity
A11438	Acute Toxicity
A11439	Acute Toxicity
A11440	Acute Toxicity
A11441	Mutagenicity, Tumorigenicity
A11442	Reproductive Effects
A11443	Multiple Dose Effects
A11444	Mutagenicity
A11445	Mutagenicity
A11446	Mutagenicity
A11447	Mutagenicity
A11448	Multiple Dose Effects
A11449	Mutagenicity
A11450	Mutagenicity, Tumorigenicity
A11451	Acute Toxicity, Reproductive Effects, Multiple Dose Effects
A11452	Acute Toxicity
A11453	Acute Toxicity, Reproductive Effects
A11454	Mutagenicity, Tumorigenicity, Multiple Dose Effects
A11455	Acute Toxicity, Mutagenicity
A11456	Reproductive Effects
A11457	Mutagenicity
A11458	Mutagenicity
A11459	Acute Toxicity
A11460	Acute Toxicity, Mutagenicity
A11461	Mutagenicity

A11462	Acute Toxicity
A11463	Mutagenicity
A11464	Mutagenicity, Tumorigenicity
A11465	Mutagenicity
A11466	Mutagenicity
A11467	Mutagenicity
A11468	Mutagenicity
A11469	Acute Toxicity, Multiple Dose Effects
A11470	Multiple Dose Effects
A11471	Acute Toxicity, Multiple Dose Effects
A11472	Non-toxicity
A11473	Acute Toxicity, Multiple Dose Effects
A11474	Acute Toxicity
A11475	Acute Toxicity, Mutagenicity, Multiple Dose Effects
A11476	Acute Toxicity, Multiple Dose Effects
A11477	Acute Toxicity, Mutagenicity, Multiple Dose Effects
A11478	Mutagenicity
A11479	Multiple Dose Effects
A11480	Acute Toxicity
A11481	Mutagenicity, Tumorigenicity
A11482	Acute Toxicity, Mutagenicity
A11483	Mutagenicity, Tumorigenicity
A11484	Mutagenicity
A11485	Acute Toxicity, Mutagenicity, Multiple Dose Effects
A11486	Non-toxicity
A11487	Acute Toxicity, Tumorigenicity, Reproductive Effects, Multiple Dose Effects
A11488	Skin and Eye Irritation, Multiple Dose Effects
A11489	Acute Toxicity
A11490	Mutagenicity
A11491	Mutagenicity
A11492	Non-toxicity
A11493	Acute Toxicity, Mutagenicity
A11494	Acute Toxicity
A11495	Acute Toxicity
A11496	Multiple Dose Effects
A11497	Acute Toxicity, Multiple Dose Effects
A11498	Mutagenicity
A11499	Acute Toxicity, Mutagenicity, Skin and Eye Irritation
A11500	Acute Toxicity
A11501	Non-toxicity
A11502	Acute Toxicity, Skin and Eye Irritation, Reproductive Effects, Multiple Dose
A11503	Acute Toxicity
A11504	Mutagenicity
A11505	Non-toxicity
A11506	Skin and Eye Irritation
A11507	Acute Toxicity

A11508	Non-toxicity
A11509	Acute Toxicity
A11510	Acute Toxicity
A11511	Acute Toxicity
A11512	Acute Toxicity
A11513	Acute Toxicity
A11514	Acute Toxicity
A11515	Acute Toxicity
A11516	Acute Toxicity
A11517	Acute Toxicity
A11518	Acute Toxicity
A11519	Acute Toxicity
A11520	Acute Toxicity
A11521	Acute Toxicity
A11522	Acute Toxicity
A11523	Acute Toxicity
A11524	Mutagenicity
A11525	Acute Toxicity, Mutagenicity, Multiple Dose Effects
A11526	Skin and Eye Irritation, Multiple Dose Effects
A11527	Mutagenicity, Tumorigenicity
A11528	Acute Toxicity
A11529	Acute Toxicity
A11530	Mutagenicity, Tumorigenicity
A11531	Mutagenicity
A11532	Tumorigenicity
A11533	Reproductive Effects
A11534	Mutagenicity
A11535	Non-toxicity
A11536	Mutagenicity
A11537	Acute Toxicity
A11538	Acute Toxicity
A11539	Multiple Dose Effects
A11540	Acute Toxicity
A11541	Acute Toxicity, Reproductive Effects
A11542	Multiple Dose Effects
A11543	Mutagenicity, Tumorigenicity
A11544	Acute Toxicity
A11545	Acute Toxicity
A11546	Mutagenicity
A11547	Acute Toxicity, Mutagenicity
A11548	Tumorigenicity
A11549	Acute Toxicity
A11550	Acute Toxicity
A11551	Acute Toxicity, Multiple Dose Effects
A11552	Acute Toxicity
A11553	Acute Toxicity

A11554	Acute Toxicity, Multiple Dose Effects
A11555	Acute Toxicity
A11556	Tumorigenicity
A11557	Mutagenicity
A11558	Multiple Dose Effects
A11559	Multiple Dose Effects
A11560	Non-toxicity
A11561	Mutagenicity
A11562	Mutagenicity
A11563	Mutagenicity
A11564	Acute Toxicity, Mutagenicity, Tumorigenicity
A11565	Acute Toxicity
A11566	Mutagenicity
A11567	Mutagenicity, Tumorigenicity
A11568	Mutagenicity
A11569	Mutagenicity
A11570	Acute Toxicity, Mutagenicity, Reproductive Effects, Multiple Dose Effects
A11571	Mutagenicity
A11572	Acute Toxicity
A11573	Tumorigenicity
A11574	Mutagenicity
A11575	Mutagenicity
A11576	Mutagenicity
A11577	Mutagenicity
A11578	Acute Toxicity
A11579	Mutagenicity
A11580	Acute Toxicity, Reproductive Effects, Multiple Dose Effects
A11581	Mutagenicity, Tumorigenicity
A11582	Mutagenicity
A11583	Skin and Eye Irritation
A11584	Non-toxicity
A11585	Mutagenicity
A11586	Mutagenicity
A11587	Mutagenicity, Tumorigenicity
A11588	Acute Toxicity, Multiple Dose Effects
A11589	Acute Toxicity, Skin and Eye Irritation
A11590	Acute Toxicity
A11591	Acute Toxicity, Multiple Dose Effects
A11592	Mutagenicity
A11593	Acute Toxicity
A11594	Mutagenicity, Tumorigenicity
A11595	Mutagenicity
A11596	Acute Toxicity, Multiple Dose Effects
A11597	Acute Toxicity
A11598	Acute Toxicity, Tumorigenicity, Multiple Dose Effects
A11599	Mutagenicity

A11600	Mutagenicity, Tumorigenicity
A11601	Non-toxicity
A11602	Mutagenicity
A11603	Mutagenicity
A11604	Non-toxicity
A11605	Tumorigenicity
A11606	Multiple Dose Effects
A11607	Acute Toxicity
A11608	Acute Toxicity
A11609	Mutagenicity
A11610	Mutagenicity, Tumorigenicity
A11611	Mutagenicity
A11612	Acute Toxicity
A11613	Acute Toxicity
A11614	Acute Toxicity, Multiple Dose Effects
A11615	Acute Toxicity
A11616	Acute Toxicity, Multiple Dose Effects
A11617	Acute Toxicity
A11618	Acute Toxicity
A11619	Acute Toxicity
A11620	Acute Toxicity
A11621	Acute Toxicity, Multiple Dose Effects
A11622	Acute Toxicity
A11623	Acute Toxicity
A11624	Acute Toxicity
A11625	Acute Toxicity
A11626	Acute Toxicity
A11627	Acute Toxicity
A11628	Acute Toxicity
A11629	Acute Toxicity
A11630	Acute Toxicity, Skin and Eye Irritation, Multiple Dose Effects
A11631	Acute Toxicity
A11632	Acute Toxicity
A11633	Acute Toxicity
A11634	Acute Toxicity
A11635	Acute Toxicity
A11636	Acute Toxicity
A11637	Acute Toxicity
A11638	Acute Toxicity
A11639	Acute Toxicity
A11640	Acute Toxicity
A11641	Acute Toxicity
A11642	Acute Toxicity
A11643	Acute Toxicity
A11644	Acute Toxicity
A11645	Acute Toxicity

A11646	Acute Toxicity
A11647	Acute Toxicity
A11648	Acute Toxicity, Multiple Dose Effects
A11649	Acute Toxicity, Multiple Dose Effects
A11650	Reproductive Effects
A11651	Multiple Dose Effects
A11652	Tumorigenicity
A11653	Tumorigenicity, Multiple Dose Effects
A11654	Mutagenicity, Tumorigenicity
A11655	Mutagenicity
A11656	Acute Toxicity, Mutagenicity, Multiple Dose Effects
A11657	Acute Toxicity, Mutagenicity
A11658	Acute Toxicity
A11659	Acute Toxicity
A11660	Reproductive Effects
A11661	Mutagenicity
A11662	Reproductive Effects
A11663	Mutagenicity, Multiple Dose Effects
A11664	Acute Toxicity
A11665	Acute Toxicity
A11666	Mutagenicity
A11667	Mutagenicity
A11668	Mutagenicity
A11669	Mutagenicity
A11670	Acute Toxicity
A11671	Acute Toxicity
A11672	Acute Toxicity, Tumorigenicity, Reproductive Effects, Multiple Dose Effects
A11673	Acute Toxicity
A11674	Mutagenicity, Tumorigenicity
A11675	Mutagenicity, Multiple Dose Effects
A11676	Mutagenicity
A11677	Acute Toxicity
A11678	Tumorigenicity, Skin and Eye Irritation
A11679	Reproductive Effects
A11680	Mutagenicity
A11681	Mutagenicity
A11682	Mutagenicity, Tumorigenicity
A11683	Acute Toxicity, Mutagenicity, Tumorigenicity, Skin and Eye Irritation,
A11684	Acute Toxicity, Multiple Dose Effects
A11685	Acute Toxicity
A11686	Mutagenicity
A11687	Mutagenicity, Tumorigenicity
A11688	Acute Toxicity
A11689	Acute Toxicity, Mutagenicity, Multiple Dose Effects
A11690	Acute Toxicity
A11691	Reproductive Effects

A11692	Acute Toxicity, Reproductive Effects, Multiple Dose Effects
A11693	Mutagenicity
A11694	Non-toxicity
A11695	Acute Toxicity
A11696	Mutagenicity
A11697	Acute Toxicity
A11698	Mutagenicity
A11699	Acute Toxicity
A11700	Mutagenicity
A11701	Mutagenicity
A11702	Acute Toxicity
A11703	Mutagenicity
A11704	Acute Toxicity
A11705	Acute Toxicity, Multiple Dose Effects
A11706	Acute Toxicity
A11707	Mutagenicity
A11708	Acute Toxicity, Reproductive Effects
A11709	Mutagenicity
A11710	Mutagenicity
A11711	Acute Toxicity, Skin and Eye Irritation
A11712	Mutagenicity
A11713	Mutagenicity, Reproductive Effects
A11714	Multiple Dose Effects
A11715	Mutagenicity
A11716	Acute Toxicity, Mutagenicity
A11717	Mutagenicity, Tumorigenicity
A11718	Acute Toxicity
A11719	Mutagenicity, Tumorigenicity
A11720	Mutagenicity
A11721	Mutagenicity, Tumorigenicity
A11722	Mutagenicity
A11723	Mutagenicity
A11724	Acute Toxicity
A11725	Acute Toxicity
A11726	Mutagenicity, Tumorigenicity
A11727	Acute Toxicity, Mutagenicity, Reproductive Effects, Multiple Dose Effects
A11728	Acute Toxicity, Multiple Dose Effects
A11729	Acute Toxicity, Reproductive Effects, Multiple Dose Effects
A11730	Tumorigenicity
A11731	Acute Toxicity
A11732	Mutagenicity
A11733	Acute Toxicity
A11734	Acute Toxicity, Multiple Dose Effects
A11735	Acute Toxicity, Multiple Dose Effects
A11736	Acute Toxicity
A11737	Acute Toxicity

A11738	Mutagenicity
A11739	Skin and Eye Irritation
A11740	Acute Toxicity
A11741	Acute Toxicity, Reproductive Effects, Multiple Dose Effects
A11742	Acute Toxicity, Reproductive Effects, Multiple Dose Effects
A11743	Acute Toxicity
A11744	Acute Toxicity
A11745	Acute Toxicity, Skin and Eye Irritation
A11746	Acute Toxicity
A11747	Acute Toxicity, Reproductive Effects
A11748	Acute Toxicity
A11749	Acute Toxicity
A11750	Acute Toxicity, Multiple Dose Effects
A11751	Acute Toxicity
A11752	Acute Toxicity
A11753	Acute Toxicity
A11754	Acute Toxicity
A11755	Acute Toxicity
A11756	Acute Toxicity
A11757	Acute Toxicity
A11758	Acute Toxicity
A11759	Acute Toxicity
A11760	Acute Toxicity
A11761	Acute Toxicity
A11762	Acute Toxicity
A11763	Acute Toxicity
A11764	Acute Toxicity
A11765	Acute Toxicity
A11766	Acute Toxicity
A11767	Acute Toxicity
A11768	Acute Toxicity
A11769	Acute Toxicity
A11770	Acute Toxicity
A11771	Acute Toxicity
A11772	Acute Toxicity
A11773	Acute Toxicity
A11774	Acute Toxicity
A11775	Acute Toxicity
A11776	Acute Toxicity
A11777	Acute Toxicity
A11778	Acute Toxicity
A11779	Acute Toxicity
A11780	Acute Toxicity
A11781	Acute Toxicity
A11782	Acute Toxicity
A11783	Acute Toxicity

A11784	Acute Toxicity
A11785	Acute Toxicity
A11786	Acute Toxicity
A11787	Acute Toxicity
A11788	Acute Toxicity
A11789	Acute Toxicity
A11790	Acute Toxicity
A11791	Acute Toxicity
A11792	Acute Toxicity
A11793	Acute Toxicity
A11794	Acute Toxicity
A11795	Acute Toxicity
A11796	Acute Toxicity
A11797	Acute Toxicity
A11798	Acute Toxicity, Multiple Dose Effects
A11799	Acute Toxicity
A11800	Acute Toxicity
A11801	Acute Toxicity
A11802	Acute Toxicity
A11803	Acute Toxicity
A11804	Acute Toxicity
A11805	Acute Toxicity
A11806	Acute Toxicity
A11807	Acute Toxicity
A11808	Acute Toxicity
A11809	Acute Toxicity, Reproductive Effects
A11810	Acute Toxicity
A11811	Acute Toxicity
A11812	Acute Toxicity
A11813	Acute Toxicity
A11814	Acute Toxicity
A11815	Acute Toxicity
A11816	Acute Toxicity
A11817	Acute Toxicity
A11818	Acute Toxicity
A11819	Acute Toxicity
A11820	Acute Toxicity
A11821	Acute Toxicity
A11822	Acute Toxicity
A11823	Acute Toxicity
A11824	Acute Toxicity
A11825	Skin and Eye Irritation
A11826	Non-toxicity
A11827	Acute Toxicity
A11828	Acute Toxicity
A11829	Multiple Dose Effects

A11830	Mutagenicity
A11831	Multiple Dose Effects
A11832	Acute Toxicity, Multiple Dose Effects
A11833	Acute Toxicity
A11834	Non-toxicity
A11835	Mutagenicity
A11836	Acute Toxicity
A11837	Acute Toxicity, Multiple Dose Effects
A11838	Acute Toxicity, Multiple Dose Effects
A11839	Non-toxicity
A11840	Mutagenicity
A11841	Acute Toxicity
A11842	Acute Toxicity
A11843	Tumorigenicity
A11844	Tumorigenicity
A11845	Acute Toxicity, Multiple Dose Effects
A11846	Acute Toxicity
A11847	Acute Toxicity, Multiple Dose Effects
A11848	Mutagenicity
A11849	Acute Toxicity
A11850	Non-toxicity
A11851	Acute Toxicity
A11852	Acute Toxicity
A11853	Non-toxicity
A11854	Acute Toxicity
A11855	Acute Toxicity, Multiple Dose Effects
A11856	Non-toxicity
A11857	Acute Toxicity
A11858	Acute Toxicity
A11859	Multiple Dose Effects
A11860	Acute Toxicity
A11861	Acute Toxicity
A11862	Multiple Dose Effects
A11863	Acute Toxicity
A11864	Acute Toxicity
A11865	Acute Toxicity
A11866	Acute Toxicity
A11867	Acute Toxicity
A11868	Tumorigenicity
A11869	Mutagenicity
A11870	Acute Toxicity
A11871	Acute Toxicity
A11872	Mutagenicity
A11873	Acute Toxicity, Multiple Dose Effects
A11874	Skin and Eye Irritation
A11875	Acute Toxicity

A11876	Acute Toxicity
A11877	Acute Toxicity
A11878	Acute Toxicity
A11879	Acute Toxicity
A11880	Acute Toxicity
A11881	Mutagenicity
A11882	Acute Toxicity
A11883	Acute Toxicity
A11884	Multiple Dose Effects
A11885	Acute Toxicity, Tumorigenicity, Skin and Eye Irritation, Multiple Dose
A11886	Mutagenicity
A11887	Mutagenicity
A11888	Tumorigenicity
A11889	Mutagenicity
A11890	Mutagenicity, Tumorigenicity
A11891	Mutagenicity
A11892	Mutagenicity, Reproductive Effects, Multiple Dose Effects
A11893	Mutagenicity
A11894	Multiple Dose Effects
A11895	Mutagenicity
A11896	Mutagenicity
A11897	Acute Toxicity, Mutagenicity, Tumorigenicity, Skin and Eye Irritation,
A11898	Mutagenicity
A11899	Mutagenicity
A11900	Acute Toxicity, Multiple Dose Effects
A11901	Mutagenicity
A11902	Mutagenicity
A11903	Acute Toxicity
A11904	Tumorigenicity
A11905	Acute Toxicity, Multiple Dose Effects
A11906	Non-toxicity
A11907	Acute Toxicity
A11908	Acute Toxicity
A11909	Acute Toxicity
A11910	Acute Toxicity
A11911	Acute Toxicity, Multiple Dose Effects
A11912	Mutagenicity
A11913	Acute Toxicity, Multiple Dose Effects
A11914	Acute Toxicity
A11915	Acute Toxicity, Mutagenicity
A11916	Acute Toxicity, Mutagenicity, Reproductive Effects, Multiple Dose Effects
A11917	Acute Toxicity, Tumorigenicity, Multiple Dose Effects
A11918	Acute Toxicity
A11919	Acute Toxicity, Multiple Dose Effects
A11920	Multiple Dose Effects
A11921	Non-toxicity

A11922	Acute Toxicity, Multiple Dose Effects
A11923	Acute Toxicity
A11924	Acute Toxicity
A11925	Mutagenicity
A11926	Acute Toxicity
A11927	Acute Toxicity
A11928	Acute Toxicity
A11929	Acute Toxicity
A11930	Acute Toxicity
A11931	Acute Toxicity, Mutagenicity, Tumorigenicity, Multiple Dose Effects
A11932	Non-toxicity
A11933	Acute Toxicity
A11934	Acute Toxicity
A11935	Acute Toxicity, Multiple Dose Effects
A11936	Non-toxicity
A11937	Acute Toxicity, Multiple Dose Effects
A11938	Acute Toxicity
A11939	Acute Toxicity
A11940	Acute Toxicity, Multiple Dose Effects
A11941	Non-toxicity
A11942	Multiple Dose Effects
A11943	Non-toxicity
A11944	Acute Toxicity
A11945	Acute Toxicity
A11946	Acute Toxicity, Mutagenicity
A11947	Tumorigenicity, Skin and Eye Irritation
A11948	Acute Toxicity, Reproductive Effects
A11949	Acute Toxicity
A11950	Acute Toxicity
A11951	Mutagenicity, Tumorigenicity, Multiple Dose Effects
A11952	Multiple Dose Effects
A11953	Multiple Dose Effects
A11954	Multiple Dose Effects
A11955	Acute Toxicity, Mutagenicity
A11956	Mutagenicity
A11957	Acute Toxicity
A11958	Acute Toxicity
A11959	Acute Toxicity
A11960	Mutagenicity, Multiple Dose Effects
A11961	Mutagenicity
A11962	Acute Toxicity
A11963	Mutagenicity
A11964	Acute Toxicity
A11965	Acute Toxicity
A11966	Acute Toxicity
A11967	Mutagenicity

A11968	Multiple Dose Effects
A11969	Acute Toxicity
A11970	Mutagenicity
A11971	Tumorigenicity
A11972	Mutagenicity, Tumorigenicity
A11973	Acute Toxicity
A11974	Acute Toxicity
A11975	Acute Toxicity
A11976	Mutagenicity, Tumorigenicity
A11977	Mutagenicity
A11978	Mutagenicity
A11979	Mutagenicity, Tumorigenicity
A11980	Acute Toxicity
A11981	Multiple Dose Effects
A11982	Tumorigenicity, Multiple Dose Effects
A11983	Acute Toxicity, Mutagenicity, Tumorigenicity, Skin and Eye Irritation,
A11984	Acute Toxicity
A11985	Mutagenicity, Tumorigenicity
A11986	Acute Toxicity, Mutagenicity
A11987	Mutagenicity
A11988	Mutagenicity
A11989	Mutagenicity
A11990	Mutagenicity
A11991	Acute Toxicity
A11992	Non-toxicity
A11993	Non-toxicity
A11994	Tumorigenicity, Reproductive Effects, Multiple Dose Effects
A11995	Acute Toxicity, Mutagenicity
A11996	Acute Toxicity, Mutagenicity
A11997	Mutagenicity
A11998	Mutagenicity
A11999	Mutagenicity
A12000	Acute Toxicity
A12001	Acute Toxicity
A12002	Mutagenicity, Tumorigenicity
A12003	Mutagenicity
A12004	Acute Toxicity
A12005	Acute Toxicity, Mutagenicity, Skin and Eye Irritation, Multiple Dose Effects
A12006	Mutagenicity
A12007	Acute Toxicity
A12008	Acute Toxicity
A12009	Acute Toxicity, Skin and Eye Irritation, Multiple Dose Effects
A12010	Multiple Dose Effects
A12011	Acute Toxicity
A12012	Acute Toxicity
A12013	Reproductive Effects

A12014	Acute Toxicity, Mutagenicity
A12015	Acute Toxicity
A12016	Mutagenicity
A12017	Acute Toxicity, Reproductive Effects, Multiple Dose Effects
A12018	Acute Toxicity
A12019	Mutagenicity
A12020	Acute Toxicity
A12021	Mutagenicity
A12022	Mutagenicity
A12023	Tumorigenicity
A12024	Mutagenicity
A12025	Mutagenicity
A12026	Acute Toxicity
A12027	Acute Toxicity
A12028	Acute Toxicity
A12029	Acute Toxicity
A12030	Acute Toxicity
A12031	Mutagenicity
A12032	Tumorigenicity
A12033	Acute Toxicity
A12034	Acute Toxicity
A12035	Mutagenicity
A12036	Mutagenicity
A12037	Multiple Dose Effects
A12038	Skin and Eye Irritation
A12039	Acute Toxicity
A12040	Mutagenicity
A12041	Acute Toxicity
A12042	Acute Toxicity
A12043	Acute Toxicity
A12044	Acute Toxicity
A12045	Mutagenicity, Tumorigenicity
A12046	Mutagenicity, Tumorigenicity
A12047	Acute Toxicity, Mutagenicity, Tumorigenicity, Skin and Eye Irritation,
A12048	Acute Toxicity
A12049	Acute Toxicity
A12050	Acute Toxicity
A12051	Mutagenicity
A12052	Acute Toxicity, Multiple Dose Effects
A12053	Acute Toxicity
A12054	Acute Toxicity
A12055	Mutagenicity
A12056	Acute Toxicity
A12057	Reproductive Effects
A12058	Acute Toxicity, Multiple Dose Effects
A12059	Acute Toxicity

A12060	Acute Toxicity
A12061	Reproductive Effects
A12062	Acute Toxicity, Mutagenicity
A12063	Acute Toxicity
A12064	Mutagenicity
A12065	Mutagenicity, Tumorigenicity
A12066	Mutagenicity, Tumorigenicity
A12067	Multiple Dose Effects
A12068	Multiple Dose Effects
A12069	Acute Toxicity
A12070	Acute Toxicity
A12071	Acute Toxicity
A12072	Mutagenicity, Tumorigenicity
A12073	Multiple Dose Effects
A12074	Acute Toxicity
A12075	Acute Toxicity
A12076	Acute Toxicity
A12077	Acute Toxicity
A12078	Mutagenicity
A12079	Multiple Dose Effects
A12080	Acute Toxicity
A12081	Acute Toxicity, Multiple Dose Effects
A12082	Acute Toxicity
A12083	Acute Toxicity
A12084	Acute Toxicity
A12085	Acute Toxicity, Mutagenicity
A12086	Acute Toxicity
A12087	Mutagenicity
A12088	Acute Toxicity
A12089	Acute Toxicity
A12090	Acute Toxicity
A12091	Acute Toxicity
A12092	Skin and Eye Irritation
A12093	Acute Toxicity
A12094	Acute Toxicity
A12095	Acute Toxicity
A12096	Multiple Dose Effects
A12097	Reproductive Effects
A12098	Mutagenicity
A12099	Acute Toxicity
A12100	Acute Toxicity
A12101	Acute Toxicity
A12102	Acute Toxicity
A12103	Acute Toxicity
A12104	Acute Toxicity
A12105	Acute Toxicity

A12106	Acute Toxicity
A12107	Acute Toxicity, Multiple Dose Effects
A12108	Mutagenicity
A12109	Acute Toxicity
A12110	Acute Toxicity
A12111	Mutagenicity
A12112	Mutagenicity
A12113	Acute Toxicity
A12114	Acute Toxicity
A12115	Acute Toxicity
A12116	Acute Toxicity
A12117	Mutagenicity, Tumorigenicity
A12118	Acute Toxicity
A12119	Mutagenicity, Tumorigenicity
A12120	Acute Toxicity, Mutagenicity
A12121	Mutagenicity, Reproductive Effects
A12122	Non-toxicity
A12123	Mutagenicity
A12124	Mutagenicity
A12125	Acute Toxicity
A12126	Acute Toxicity
A12127	Acute Toxicity, Reproductive Effects
A12128	Mutagenicity
A12129	Mutagenicity
A12130	Acute Toxicity
A12131	Mutagenicity, Tumorigenicity
A12132	Mutagenicity, Tumorigenicity
A12133	Mutagenicity
A12134	Mutagenicity
A12135	Mutagenicity
A12136	Acute Toxicity
A12137	Acute Toxicity
A12138	Acute Toxicity, Multiple Dose Effects
A12139	Acute Toxicity
A12140	Mutagenicity
A12141	Non-toxicity
A12142	Acute Toxicity
A12143	Acute Toxicity
A12144	Mutagenicity
A12145	Acute Toxicity
A12146	Acute Toxicity, Multiple Dose Effects
A12147	Mutagenicity
A12148	Acute Toxicity
A12149	Acute Toxicity
A12150	Mutagenicity
A12151	Acute Toxicity

A12152	Acute Toxicity, Multiple Dose Effects
A12153	Acute Toxicity
A12154	Mutagenicity, Tumorigenicity
A12155	Acute Toxicity
A12156	Mutagenicity
A12157	Acute Toxicity
A12158	Acute Toxicity, Multiple Dose Effects
A12159	Acute Toxicity
A12160	Acute Toxicity
A12161	Mutagenicity
A12162	Acute Toxicity
A12163	Multiple Dose Effects
A12164	Mutagenicity
A12165	Multiple Dose Effects
A12166	Mutagenicity
A12167	Acute Toxicity
A12168	Acute Toxicity
A12169	Mutagenicity, Tumorigenicity
A12170	Acute Toxicity
A12171	Mutagenicity
A12172	Acute Toxicity
A12173	Acute Toxicity, Multiple Dose Effects
A12174	Mutagenicity
A12175	Acute Toxicity
A12176	Mutagenicity
A12177	Acute Toxicity, Multiple Dose Effects
A12178	Acute Toxicity
A12179	Acute Toxicity
A12180	Multiple Dose Effects
A12181	Acute Toxicity
A12182	Mutagenicity
A12183	Acute Toxicity
A12184	Acute Toxicity
A12185	Mutagenicity
A12186	Acute Toxicity
A12187	Mutagenicity
A12188	Mutagenicity
A12189	Acute Toxicity
A12190	Mutagenicity
A12191	Acute Toxicity, Mutagenicity
A12192	Mutagenicity
A12193	Mutagenicity
A12194	Mutagenicity
A12195	Acute Toxicity
A12196	Acute Toxicity
A12197	Acute Toxicity

A12198	Acute Toxicity
A12199	Multiple Dose Effects
A12200	Multiple Dose Effects
A12201	Acute Toxicity
A12202	Mutagenicity
A12203	Mutagenicity
A12204	Acute Toxicity, Mutagenicity, Skin and Eye Irritation
A12205	Acute Toxicity, Mutagenicity
A12206	Reproductive Effects
A12207	Mutagenicity, Tumorigenicity
A12208	Acute Toxicity, Multiple Dose Effects
A12209	Acute Toxicity
A12210	Acute Toxicity
A12211	Multiple Dose Effects
A12212	Tumorigenicity
A12213	Acute Toxicity
A12214	Multiple Dose Effects
A12215	Acute Toxicity
A12216	Acute Toxicity
A12217	Acute Toxicity
A12218	Acute Toxicity
A12219	Reproductive Effects
A12220	Non-toxicity
A12221	Acute Toxicity, Multiple Dose Effects
A12222	Acute Toxicity
A12223	Acute Toxicity
A12224	Mutagenicity
A12225	Acute Toxicity
A12226	Acute Toxicity
A12227	Acute Toxicity, Multiple Dose Effects
A12228	Acute Toxicity
A12229	Acute Toxicity
A12230	Acute Toxicity
A12231	Acute Toxicity
A12232	Acute Toxicity
A12233	Acute Toxicity
A12234	Mutagenicity
A12235	Non-toxicity
A12236	Acute Toxicity
A12237	Acute Toxicity
A12238	Acute Toxicity
A12239	Acute Toxicity
A12240	Acute Toxicity
A12241	Acute Toxicity
A12242	Acute Toxicity
A12243	Acute Toxicity

A12244	Acute Toxicity
A12245	Acute Toxicity
A12246	Multiple Dose Effects
A12247	Non-toxicity
A12248	Acute Toxicity, Multiple Dose Effects
A12249	Acute Toxicity
A12250	Acute Toxicity
A12251	Acute Toxicity
A12252	Acute Toxicity
A12253	Acute Toxicity
A12254	Acute Toxicity, Multiple Dose Effects
A12255	Acute Toxicity, Multiple Dose Effects
A12256	Acute Toxicity
A12257	Acute Toxicity
A12258	Acute Toxicity
A12259	Acute Toxicity
A12260	Acute Toxicity
A12261	Acute Toxicity
A12262	Mutagenicity
A12263	Non-toxicity
A12264	Mutagenicity
A12265	Acute Toxicity, Mutagenicity
A12266	Acute Toxicity
A12267	Mutagenicity
A12268	Acute Toxicity
A12269	Acute Toxicity
A12270	Acute Toxicity
A12271	Acute Toxicity
A12272	Acute Toxicity
A12273	Mutagenicity
A12274	Acute Toxicity
A12275	Acute Toxicity
A12276	Acute Toxicity
A12277	Acute Toxicity
A12278	Acute Toxicity, Mutagenicity, Tumorigenicity
A12279	Acute Toxicity
A12280	Acute Toxicity
A12281	Acute Toxicity
A12282	Tumorigenicity, Multiple Dose Effects
A12283	Mutagenicity
A12284	Multiple Dose Effects
A12285	Acute Toxicity
A12286	Acute Toxicity
A12287	Acute Toxicity
A12288	Acute Toxicity
A12289	Reproductive Effects

A12290	Acute Toxicity
A12291	Acute Toxicity
A12292	Multiple Dose Effects
A12293	Acute Toxicity
A12294	Acute Toxicity
A12295	Reproductive Effects, Multiple Dose Effects
A12296	Acute Toxicity, Reproductive Effects
A12297	Reproductive Effects
A12298	Acute Toxicity, Mutagenicity
A12299	Reproductive Effects
A12300	Acute Toxicity
A12301	Reproductive Effects
A12302	Reproductive Effects
A12303	Reproductive Effects
A12304	Reproductive Effects, Multiple Dose Effects
A12305	Reproductive Effects
A12306	Reproductive Effects
A12307	Reproductive Effects
A12308	Reproductive Effects
A12309	Mutagenicity
A12310	Acute Toxicity
A12311	Acute Toxicity
A12312	Reproductive Effects
A12313	Acute Toxicity
A12314	Acute Toxicity
A12315	Acute Toxicity, Mutagenicity, Reproductive Effects, Multiple Dose Effects
A12316	Mutagenicity
A12317	Mutagenicity
A12318	Tumorigenicity
A12319	Acute Toxicity
A12320	Acute Toxicity, Mutagenicity, Multiple Dose Effects
A12321	Tumorigenicity
A12322	Reproductive Effects
A12323	Acute Toxicity
A12324	Acute Toxicity
A12325	Acute Toxicity
A12326	Mutagenicity
A12327	Acute Toxicity, Reproductive Effects
A12328	Acute Toxicity, Multiple Dose Effects
A12329	Acute Toxicity, Multiple Dose Effects
A12330	Acute Toxicity
A12331	Acute Toxicity
A12332	Mutagenicity
A12333	Mutagenicity, Tumorigenicity
A12334	Acute Toxicity
A12335	Tumorigenicity

A12336	Mutagenicity
A12337	Mutagenicity
A12338	Acute Toxicity, Mutagenicity, Tumorigenicity
A12339	Mutagenicity
A12340	Mutagenicity
A12341	Non-toxicity
A12342	Mutagenicity
A12343	Mutagenicity, Tumorigenicity
A12344	Mutagenicity
A12345	Mutagenicity
A12346	Mutagenicity
A12347	Skin and Eye Irritation
A12348	Mutagenicity
A12349	Acute Toxicity
A12350	Acute Toxicity
A12351	Mutagenicity
A12352	Mutagenicity
A12353	Non-toxicity
A12354	Acute Toxicity, Multiple Dose Effects
A12355	Mutagenicity
A12356	Mutagenicity
A12357	Skin and Eye Irritation
A12358	Acute Toxicity
A12359	Non-toxicity
A12360	Mutagenicity, Tumorigenicity
A12361	Acute Toxicity, Mutagenicity, Skin and Eye Irritation, Multiple Dose Effects
A12362	Acute Toxicity
A12363	Acute Toxicity, Mutagenicity, Multiple Dose Effects
A12364	Non-toxicity
A12365	Acute Toxicity, Mutagenicity, Multiple Dose Effects
A12366	Acute Toxicity
A12367	Acute Toxicity
A12368	Acute Toxicity
A12369	Acute Toxicity
A12370	Acute Toxicity
A12371	Acute Toxicity
A12372	Tumorigenicity
A12373	Acute Toxicity, Mutagenicity, Tumorigenicity, Multiple Dose Effects
A12374	Mutagenicity
A12375	Non-toxicity
A12376	Mutagenicity
A12377	Mutagenicity
A12378	Multiple Dose Effects
A12379	Mutagenicity
A12380	Mutagenicity
A12381	Acute Toxicity

A12382	Mutagenicity
A12383	Mutagenicity
A12384	Mutagenicity
A12385	Mutagenicity
A12386	Mutagenicity
A12387	Mutagenicity
A12388	Mutagenicity
A12389	Mutagenicity
A12390	Acute Toxicity, Multiple Dose Effects
A12391	Mutagenicity
A12392	Mutagenicity
A12393	Acute Toxicity
A12394	Mutagenicity
A12395	Acute Toxicity
A12396	Acute Toxicity
A12397	Acute Toxicity, Mutagenicity
A12398	Mutagenicity
A12399	Mutagenicity
A12400	Acute Toxicity, Mutagenicity
A12401	Mutagenicity
A12402	Mutagenicity
A12403	Acute Toxicity
A12404	Reproductive Effects
A12405	Mutagenicity
A12406	Mutagenicity
A12407	Acute Toxicity
A12408	Acute Toxicity
A12409	Acute Toxicity
A12410	Mutagenicity
A12411	Acute Toxicity, Mutagenicity
A12412	Acute Toxicity
A12413	Acute Toxicity, Mutagenicity
A12414	Mutagenicity
A12415	Mutagenicity
A12416	Acute Toxicity
A12417	Acute Toxicity
A12418	Acute Toxicity
A12419	Acute Toxicity, Mutagenicity, Multiple Dose Effects
A12420	Mutagenicity
A12421	Mutagenicity
A12422	Mutagenicity
A12423	Acute Toxicity, Mutagenicity, Tumorigenicity
A12424	Mutagenicity
A12425	Mutagenicity
A12426	Mutagenicity, Tumorigenicity
A12427	Acute Toxicity

A12428	Mutagenicity, Tumorigenicity
A12429	Mutagenicity
A12430	Tumorigenicity
A12431	Acute Toxicity, Mutagenicity
A12432	Mutagenicity
A12433	Acute Toxicity, Multiple Dose Effects
A12434	Mutagenicity
A12435	Mutagenicity
A12436	Mutagenicity
A12437	Mutagenicity
A12438	Mutagenicity
A12439	Mutagenicity, Tumorigenicity
A12440	Mutagenicity
A12441	Mutagenicity
A12442	Mutagenicity
A12443	Acute Toxicity
A12444	Acute Toxicity
A12445	Mutagenicity
A12446	Mutagenicity, Tumorigenicity
A12447	Acute Toxicity
A12448	Mutagenicity
A12449	Mutagenicity
A12450	Acute Toxicity
A12451	Skin and Eye Irritation
A12452	Acute Toxicity
A12453	Mutagenicity
A12454	Mutagenicity
A12455	Mutagenicity
A12456	Mutagenicity
A12457	Mutagenicity
A12458	Mutagenicity
A12459	Mutagenicity, Tumorigenicity
A12460	Tumorigenicity, Reproductive Effects
A12461	Mutagenicity
A12462	Mutagenicity
A12463	Mutagenicity
A12464	Mutagenicity
A12465	Mutagenicity
A12466	Mutagenicity
A12467	Tumorigenicity
A12468	Mutagenicity
A12469	Mutagenicity
A12470	Mutagenicity
A12471	Mutagenicity
A12472	Mutagenicity
A12473	Acute Toxicity

A12474	Mutagenicity
A12475	Acute Toxicity, Mutagenicity
A12476	Mutagenicity
A12477	Acute Toxicity
A12478	Mutagenicity
A12479	Mutagenicity, Tumorigenicity
A12480	Mutagenicity
A12481	Mutagenicity
A12482	Mutagenicity
A12483	Mutagenicity
A12484	Mutagenicity
A12485	Mutagenicity
A12486	Mutagenicity, Tumorigenicity
A12487	Acute Toxicity
A12488	Mutagenicity
A12489	Mutagenicity
A12490	Mutagenicity
A12491	Mutagenicity
A12492	Non-toxicity
A12493	Mutagenicity
A12494	Mutagenicity
A12495	Mutagenicity
A12496	Mutagenicity
A12497	Mutagenicity, Multiple Dose Effects
A12498	Mutagenicity
A12499	Multiple Dose Effects
A12500	Acute Toxicity
A12501	Multiple Dose Effects
A12502	Mutagenicity, Multiple Dose Effects
A12503	Acute Toxicity, Multiple Dose Effects
A12504	Mutagenicity
A12505	Mutagenicity
A12506	Acute Toxicity, Multiple Dose Effects
A12507	Multiple Dose Effects
A12508	Multiple Dose Effects
A12509	Non-toxicity
A12510	Mutagenicity, Tumorigenicity
A12511	Mutagenicity
A12512	Mutagenicity
A12513	Mutagenicity
A12514	Acute Toxicity, Mutagenicity
A12515	Acute Toxicity, Mutagenicity
A12516	Acute Toxicity, Mutagenicity
A12517	Mutagenicity
A12518	Mutagenicity
A12519	Mutagenicity

A12520	Mutagenicity
A12521	Mutagenicity
A12522	Mutagenicity
A12523	Mutagenicity
A12524	Mutagenicity
A12525	Mutagenicity
A12526	Mutagenicity
A12527	Mutagenicity
A12528	Mutagenicity
A12529	Mutagenicity
A12530	Mutagenicity
A12531	Mutagenicity
A12532	Mutagenicity
A12533	Mutagenicity
A12534	Mutagenicity
A12535	Mutagenicity
A12536	Mutagenicity
A12537	Acute Toxicity, Mutagenicity
A12538	Acute Toxicity, Mutagenicity
A12539	Mutagenicity
A12540	Mutagenicity
A12541	Mutagenicity, Tumorigenicity
A12542	Mutagenicity
A12543	Mutagenicity
A12544	Reproductive Effects
A12545	Reproductive Effects
A12546	Reproductive Effects
A12547	Reproductive Effects
A12548	Acute Toxicity, Mutagenicity, Reproductive Effects
A12549	Mutagenicity
A12550	Mutagenicity
A12551	Mutagenicity, Tumorigenicity
A12552	Mutagenicity
A12553	Mutagenicity
A12554	Mutagenicity
A12555	Mutagenicity
A12556	Acute Toxicity, Mutagenicity, Skin and Eye Irritation, Reproductive Effects, Multiple Dose Effects
A12557	Acute Toxicity
A12558	Mutagenicity
A12559	Mutagenicity
A12560	Mutagenicity, Tumorigenicity
A12561	Mutagenicity
A12562	Mutagenicity
A12563	Mutagenicity
A12564	Non-toxicity

A12565	Mutagenicity
A12566	Mutagenicity
A12567	Mutagenicity
A12568	Mutagenicity
A12569	Mutagenicity
A12570	Mutagenicity
A12571	Mutagenicity
A12572	Mutagenicity
A12573	Mutagenicity, Tumorigenicity
A12574	Mutagenicity
A12575	Mutagenicity
A12576	Mutagenicity
A12577	Tumorigenicity
A12578	Mutagenicity
A12579	Mutagenicity
A12580	Mutagenicity
A12581	Mutagenicity
A12582	Mutagenicity
A12583	Mutagenicity, Tumorigenicity
A12584	Acute Toxicity, Mutagenicity
A12585	Mutagenicity
A12586	Mutagenicity
A12587	Mutagenicity
A12588	Acute Toxicity
A12589	Reproductive Effects
A12590	Mutagenicity
A12591	Mutagenicity, Tumorigenicity
A12592	Mutagenicity
A12593	Mutagenicity
A12594	Mutagenicity
A12595	Mutagenicity
A12596	Mutagenicity
A12597	Mutagenicity
A12598	Mutagenicity
A12599	Tumorigenicity
A12600	Mutagenicity
A12601	Mutagenicity
A12602	Mutagenicity
A12603	Mutagenicity
A12604	Mutagenicity
A12605	Mutagenicity
A12606	Multiple Dose Effects
A12607	Mutagenicity
A12608	Mutagenicity
A12609	Mutagenicity
A12610	Mutagenicity

A12611	Mutagenicity
A12612	Mutagenicity
A12613	Mutagenicity
A12614	Mutagenicity
A12615	Mutagenicity
A12616	Mutagenicity
A12617	Mutagenicity
A12618	Mutagenicity
A12619	Mutagenicity
A12620	Mutagenicity
A12621	Mutagenicity
A12622	Mutagenicity, Tumorigenicity
A12623	Mutagenicity
A12624	Mutagenicity
A12625	Non-toxicity
A12626	Acute Toxicity
A12627	Mutagenicity
A12628	Mutagenicity, Tumorigenicity
A12629	Mutagenicity
A12630	Tumorigenicity
A12631	Mutagenicity
A12632	Mutagenicity
A12633	Mutagenicity
A12634	Tumorigenicity
A12635	Mutagenicity
A12636	Mutagenicity
A12637	Mutagenicity
A12638	Mutagenicity
A12639	Mutagenicity
A12640	Mutagenicity
A12641	Mutagenicity
A12642	Mutagenicity
A12643	Acute Toxicity
A12644	Tumorigenicity, Multiple Dose Effects
A12645	Reproductive Effects
A12646	Non-toxicity
A12647	Acute Toxicity
A12648	Tumorigenicity
A12649	Mutagenicity
A12650	Mutagenicity
A12651	Tumorigenicity
A12652	Non-toxicity
A12653	Acute Toxicity
A12654	Reproductive Effects
A12655	Multiple Dose Effects
A12656	Acute Toxicity

A12657	Mutagenicity
A12658	Acute Toxicity, Multiple Dose Effects
A12659	Non-toxicity
A12660	Multiple Dose Effects
A12661	Acute Toxicity, Multiple Dose Effects
A12662	Multiple Dose Effects
A12663	Acute Toxicity
A12664	Non-toxicity
A12665	Multiple Dose Effects
A12666	Non-toxicity
A12667	Non-toxicity
A12668	Non-toxicity
A12669	Mutagenicity
A12670	Mutagenicity
A12671	Mutagenicity
A12672	Acute Toxicity
A12673	Acute Toxicity
A12674	Mutagenicity
A12675	Tumorigenicity
A12676	Mutagenicity
A12677	Acute Toxicity
A12678	Acute Toxicity, Mutagenicity
A12679	Mutagenicity
A12680	Non-toxicity
A12681	Non-toxicity
A12682	Mutagenicity
A12683	Mutagenicity
A12684	Mutagenicity
A12685	Mutagenicity
A12686	Acute Toxicity
A12687	Acute Toxicity
A12688	Mutagenicity
A12689	Tumorigenicity
A12690	Mutagenicity
A12691	Acute Toxicity
A12692	Acute Toxicity
A12693	Acute Toxicity, Mutagenicity
A12694	Acute Toxicity
A12695	Mutagenicity, Tumorigenicity
A12696	Mutagenicity
A12697	Acute Toxicity
A12698	Mutagenicity
A12699	Mutagenicity
A12700	Mutagenicity
A12701	Mutagenicity
A12702	Mutagenicity

A12703	Non-toxicity
A12704	Acute Toxicity
A12705	Non-toxicity
A12706	Acute Toxicity
A12707	Mutagenicity
A12708	Mutagenicity
A12709	Mutagenicity
A12710	Acute Toxicity
A12711	Mutagenicity
A12712	Mutagenicity
A12713	Non-toxicity
A12714	Acute Toxicity
A12715	Non-toxicity
A12716	Mutagenicity, Tumorigenicity
A12717	Mutagenicity
A12718	Mutagenicity
A12719	Mutagenicity
A12720	Mutagenicity
A12721	Mutagenicity
A12722	Acute Toxicity, Multiple Dose Effects
A12723	Acute Toxicity, Multiple Dose Effects
A12724	Acute Toxicity, Reproductive Effects, Multiple Dose Effects
A12725	Non-toxicity
A12726	Acute Toxicity, Multiple Dose Effects
A12727	Multiple Dose Effects
A12728	Acute Toxicity, Reproductive Effects, Multiple Dose Effects
A12729	Acute Toxicity
A12730	Mutagenicity
A12731	Mutagenicity
A12732	Mutagenicity
A12733	Mutagenicity
A12734	Mutagenicity
A12735	Mutagenicity
A12736	Mutagenicity
A12737	Acute Toxicity
A12738	Multiple Dose Effects
A12739	Acute Toxicity, Mutagenicity
A12740	Mutagenicity
A12741	Mutagenicity
A12742	Mutagenicity
A12743	Mutagenicity
A12744	Mutagenicity
A12745	Mutagenicity
A12746	Mutagenicity
A12747	Mutagenicity
A12748	Mutagenicity, Tumorigenicity

A12749	Mutagenicity
A12750	Mutagenicity
A12751	Mutagenicity
A12752	Mutagenicity
A12753	Tumorigenicity
A12754	Tumorigenicity
A12755	Tumorigenicity
A12756	Mutagenicity
A12757	Acute Toxicity, Tumorigenicity
A12758	Mutagenicity
A12759	Mutagenicity
A12760	Mutagenicity
A12761	Mutagenicity
A12762	Mutagenicity
A12763	Mutagenicity
A12764	Mutagenicity
A12765	Mutagenicity
A12766	Mutagenicity
A12767	Mutagenicity
A12768	Tumorigenicity
A12769	Tumorigenicity
A12770	Mutagenicity, Multiple Dose Effects
A12771	Mutagenicity
A12772	Mutagenicity
A12773	Mutagenicity
A12774	Mutagenicity
A12775	Mutagenicity
A12776	Mutagenicity
A12777	Mutagenicity
A12778	Mutagenicity
A12779	Mutagenicity, Tumorigenicity
A12780	Mutagenicity
A12781	Tumorigenicity
A12782	Tumorigenicity
A12783	Mutagenicity
A12784	Mutagenicity
A12785	Mutagenicity
A12786	Tumorigenicity
A12787	Tumorigenicity
A12788	Mutagenicity
A12789	Mutagenicity
A12790	Mutagenicity
A12791	Acute Toxicity
A12792	Multiple Dose Effects
A12793	Acute Toxicity, Mutagenicity
A12794	Skin and Eye Irritation

A12795	Acute Toxicity
A12796	Mutagenicity
A12797	Mutagenicity
A12798	Mutagenicity
A12799	Mutagenicity
A12800	Mutagenicity
A12801	Mutagenicity
A12802	Acute Toxicity
A12803	Mutagenicity
A12804	Tumorigenicity
A12805	Mutagenicity
A12806	Mutagenicity
A12807	Mutagenicity
A12808	Mutagenicity
A12809	Tumorigenicity
A12810	Acute Toxicity
A12811	Mutagenicity, Tumorigenicity
A12812	Acute Toxicity
A12813	Acute Toxicity
A12814	Acute Toxicity
A12815	Acute Toxicity
A12816	Acute Toxicity
A12817	Acute Toxicity, Mutagenicity
A12818	Non-toxicity
A12819	Acute Toxicity
A12820	Tumorigenicity
A12821	Acute Toxicity, Reproductive Effects, Multiple Dose Effects
A12822	Acute Toxicity
A12823	Acute Toxicity
A12824	Acute Toxicity, Mutagenicity, Tumorigenicity, Skin and Eye Irritation,
A12825	Multiple Dose Effects
A12826	Acute Toxicity
A12827	Acute Toxicity
A12828	Acute Toxicity
A12829	Acute Toxicity
A12830	Acute Toxicity
A12831	Acute Toxicity
A12832	Acute Toxicity
A12833	Mutagenicity
A12834	Mutagenicity, Reproductive Effects
A12835	Acute Toxicity, Tumorigenicity, Reproductive Effects, Multiple Dose Effects
A12836	Mutagenicity
A12837	Acute Toxicity
A12838	Mutagenicity
A12839	Acute Toxicity
A12840	Skin and Eye Irritation

A12841	Acute Toxicity
A12842	Skin and Eye Irritation
A12843	Mutagenicity
A12844	Acute Toxicity
A12845	Mutagenicity
A12846	Reproductive Effects
A12847	Acute Toxicity
A12848	Acute Toxicity
A12849	Multiple Dose Effects
A12850	Acute Toxicity
A12851	Acute Toxicity
A12852	Acute Toxicity
A12853	Acute Toxicity, Multiple Dose Effects
A12854	Acute Toxicity, Multiple Dose Effects
A12855	Mutagenicity
A12856	Acute Toxicity
A12857	Tumorigenicity
A12858	Acute Toxicity, Multiple Dose Effects
A12859	Mutagenicity
A12860	Mutagenicity
A12861	Mutagenicity
A12862	Tumorigenicity
A12863	Acute Toxicity
A12864	Acute Toxicity
A12865	Acute Toxicity
A12866	Acute Toxicity
A12867	Acute Toxicity
A12868	Acute Toxicity, Skin and Eye Irritation
A12869	Reproductive Effects
A12870	Acute Toxicity
A12871	Acute Toxicity, Multiple Dose Effects
A12872	Acute Toxicity
A12873	Acute Toxicity, Multiple Dose Effects
A12874	Acute Toxicity, Mutagenicity
A12875	Multiple Dose Effects
A12876	Mutagenicity
A12877	Mutagenicity
A12878	Acute Toxicity
A12879	Mutagenicity
A12880	Acute Toxicity
A12881	Acute Toxicity
A12882	Acute Toxicity, Reproductive Effects
A12883	Reproductive Effects
A12884	Mutagenicity
A12885	Acute Toxicity, Multiple Dose Effects
A12886	Non-toxicity

A12887	Multiple Dose Effects
A12888	Mutagenicity
A12889	Reproductive Effects
A12890	Acute Toxicity
A12891	Acute Toxicity
A12892	Mutagenicity
A12893	Mutagenicity
A12894	Acute Toxicity
A12895	Acute Toxicity, Multiple Dose Effects
A12896	Mutagenicity
A12897	Mutagenicity
A12898	Mutagenicity
A12899	Acute Toxicity, Mutagenicity
A12900	Acute Toxicity, Mutagenicity
A12901	Mutagenicity
A12902	Acute Toxicity
A12903	Acute Toxicity
A12904	Acute Toxicity
A12905	Mutagenicity
A12906	Mutagenicity
A12907	Mutagenicity
A12908	Mutagenicity
A12909	Acute Toxicity
A12910	Acute Toxicity
A12911	Acute Toxicity
A12912	Acute Toxicity
A12913	Mutagenicity
A12914	Acute Toxicity
A12915	Acute Toxicity, Mutagenicity
A12916	Mutagenicity
A12917	Mutagenicity
A12918	Acute Toxicity
A12919	Acute Toxicity
A12920	Mutagenicity
A12921	Skin and Eye Irritation
A12922	Reproductive Effects
A12923	Acute Toxicity
A12924	Acute Toxicity, Mutagenicity
A12925	Acute Toxicity, Multiple Dose Effects
A12926	Reproductive Effects
A12927	Acute Toxicity
A12928	Mutagenicity
A12929	Acute Toxicity, Reproductive Effects
A12930	Acute Toxicity
A12931	Acute Toxicity, Reproductive Effects
A12932	Acute Toxicity

A12933	Skin and Eye Irritation
A12934	Mutagenicity
A12935	Acute Toxicity, Mutagenicity
A12936	Mutagenicity, Tumorigenicity
A12937	Acute Toxicity
A12938	Acute Toxicity
A12939	Acute Toxicity
A12940	Acute Toxicity, Multiple Dose Effects
A12941	Mutagenicity, Tumorigenicity
A12942	Acute Toxicity
A12943	Acute Toxicity
A12944	Mutagenicity, Reproductive Effects, Multiple Dose Effects
A12945	Acute Toxicity
A12946	Acute Toxicity
A12947	Reproductive Effects
A12948	Acute Toxicity, Mutagenicity
A12949	Acute Toxicity
A12950	Mutagenicity
A12951	Acute Toxicity
A12952	Acute Toxicity, Multiple Dose Effects
A12953	Acute Toxicity
A12954	Acute Toxicity
A12955	Mutagenicity
A12956	Mutagenicity
A12957	Reproductive Effects
A12958	Reproductive Effects
A12959	Mutagenicity
A12960	Mutagenicity
A12961	Multiple Dose Effects
A12962	Tumorigenicity
A12963	Mutagenicity
A12964	Tumorigenicity
A12965	Mutagenicity
A12966	Mutagenicity
A12967	Mutagenicity, Tumorigenicity
A12968	Acute Toxicity
A12969	Non-toxicity
A12970	Non-toxicity
A12971	Non-toxicity
A12972	Acute Toxicity
A12973	Non-toxicity
A12974	Acute Toxicity
A12975	Mutagenicity
A12976	Mutagenicity
A12977	Multiple Dose Effects
A12978	Acute Toxicity

A12979	Mutagenicity
A12980	Acute Toxicity
A12981	Mutagenicity
A12982	Mutagenicity
A12983	Mutagenicity
A12984	Mutagenicity
A12985	Acute Toxicity, Multiple Dose Effects
A12986	Acute Toxicity
A12987	Acute Toxicity
A12988	Acute Toxicity
A12989	Acute Toxicity
A12990	Acute Toxicity
A12991	Mutagenicity
A12992	Non-toxicity
A12993	Acute Toxicity, Mutagenicity
A12994	Acute Toxicity
A12995	Reproductive Effects
A12996	Reproductive Effects
A12997	Acute Toxicity
A12998	Acute Toxicity
A12999	Mutagenicity
A13000	Acute Toxicity
A13001	Acute Toxicity
A13002	Acute Toxicity
A13003	Acute Toxicity
A13004	Acute Toxicity
A13005	Skin and Eye Irritation
A13006	Acute Toxicity
A13007	Acute Toxicity
A13008	Acute Toxicity
A13009	Mutagenicity
A13010	Multiple Dose Effects
A13011	Acute Toxicity
A13012	Acute Toxicity
A13013	Acute Toxicity
A13014	Acute Toxicity
A13015	Acute Toxicity
A13016	Acute Toxicity
A13017	Acute Toxicity, Reproductive Effects
A13018	Mutagenicity
A13019	Acute Toxicity
A13020	Mutagenicity
A13021	Mutagenicity
A13022	Reproductive Effects
A13023	Acute Toxicity, Mutagenicity
A13024	Acute Toxicity

A13025	Acute Toxicity
A13026	Acute Toxicity, Skin and Eye Irritation, Multiple Dose Effects
A13027	Acute Toxicity
A13028	Acute Toxicity
A13029	Acute Toxicity
A13030	Acute Toxicity
A13031	Acute Toxicity
A13032	Acute Toxicity
A13033	Acute Toxicity, Multiple Dose Effects
A13034	Acute Toxicity, Skin and Eye Irritation
A13035	Tumorigenicity
A13036	Acute Toxicity
A13037	Acute Toxicity
A13038	Acute Toxicity
A13039	Acute Toxicity
A13040	Acute Toxicity
A13041	Reproductive Effects
A13042	Acute Toxicity
A13043	Acute Toxicity
A13044	Acute Toxicity
A13045	Acute Toxicity
A13046	Acute Toxicity
A13047	Acute Toxicity
A13048	Reproductive Effects
A13049	Acute Toxicity
A13050	Acute Toxicity
A13051	Mutagenicity
A13052	Acute Toxicity
A13053	Mutagenicity
A13054	Multiple Dose Effects
A13055	Mutagenicity
A13056	Multiple Dose Effects
A13057	Acute Toxicity
A13058	Acute Toxicity
A13059	Acute Toxicity
A13060	Acute Toxicity
A13061	Acute Toxicity
A13062	Acute Toxicity, Mutagenicity
A13063	Non-toxicity
A13064	Mutagenicity
A13065	Mutagenicity
A13066	Acute Toxicity
A13067	Acute Toxicity
A13068	Acute Toxicity
A13069	Acute Toxicity
A13070	Acute Toxicity

A13071	Acute Toxicity
A13072	Acute Toxicity
A13073	Acute Toxicity
A13074	Mutagenicity, Tumorigenicity
A13075	Acute Toxicity
A13076	Acute Toxicity
A13077	Acute Toxicity
A13078	Acute Toxicity
A13079	Acute Toxicity
A13080	Acute Toxicity
A13081	Acute Toxicity
A13082	Mutagenicity
A13083	Acute Toxicity
A13084	Acute Toxicity, Skin and Eye Irritation
A13085	Mutagenicity
A13086	Mutagenicity
A13087	Acute Toxicity
A13088	Acute Toxicity
A13089	Acute Toxicity
A13090	Acute Toxicity
A13091	Reproductive Effects, Multiple Dose Effects
A13092	Acute Toxicity, Multiple Dose Effects
A13093	Mutagenicity
A13094	Mutagenicity
A13095	Mutagenicity
A13096	Acute Toxicity
A13097	Acute Toxicity
A13098	Acute Toxicity
A13099	Acute Toxicity, Reproductive Effects, Multiple Dose Effects
A13100	Mutagenicity
A13101	Mutagenicity
A13102	Mutagenicity
A13103	Acute Toxicity
A13104	Acute Toxicity
A13105	Multiple Dose Effects
A13106	Mutagenicity
A13107	Acute Toxicity
A13108	Acute Toxicity
A13109	Acute Toxicity
A13110	Multiple Dose Effects
A13111	Reproductive Effects, Multiple Dose Effects
A13112	Mutagenicity
A13113	Mutagenicity
A13114	Mutagenicity
A13115	Acute Toxicity
A13116	Acute Toxicity

A13117	Reproductive Effects, Multiple Dose Effects
A13118	Tumorigenicity
A13119	Non-toxicity
A13120	Mutagenicity
A13121	Tumorigenicity
A13122	Mutagenicity
A13123	Mutagenicity
A13124	Multiple Dose Effects
A13125	Non-toxicity
A13126	Non-toxicity
A13127	Acute Toxicity
A13128	Acute Toxicity
A13129	Reproductive Effects
A13130	Mutagenicity
A13131	Acute Toxicity, Mutagenicity
A13132	Acute Toxicity
A13133	Mutagenicity
A13134	Acute Toxicity
A13135	Acute Toxicity
A13136	Acute Toxicity
A13137	Acute Toxicity, Multiple Dose Effects
A13138	Mutagenicity
A13139	Acute Toxicity
A13140	Acute Toxicity
A13141	Acute Toxicity
A13142	Acute Toxicity
A13143	Acute Toxicity
A13144	Acute Toxicity
A13145	Acute Toxicity
A13146	Non-toxicity
A13147	Acute Toxicity
A13148	Acute Toxicity, Multiple Dose Effects
A13149	Non-toxicity
A13150	Acute Toxicity
A13151	Acute Toxicity
A13152	Mutagenicity
A13153	Multiple Dose Effects
A13154	Acute Toxicity
A13155	Acute Toxicity
A13156	Acute Toxicity
A13157	Acute Toxicity
A13158	Acute Toxicity
A13159	Acute Toxicity
A13160	Acute Toxicity, Mutagenicity, Tumorigenicity, Skin and Eye Irritation,
A13161	Multiple Dose Effects
A13162	Acute Toxicity

A13163	Acute Toxicity
A13164	Reproductive Effects
A13165	Acute Toxicity
A13166	Acute Toxicity
A13167	Acute Toxicity
A13168	Acute Toxicity
A13169	Acute Toxicity
A13170	Multiple Dose Effects
A13171	Multiple Dose Effects
A13172	Acute Toxicity
A13173	Mutagenicity
A13174	Acute Toxicity
A13175	Acute Toxicity
A13176	Acute Toxicity
A13177	Acute Toxicity, Multiple Dose Effects
A13178	Mutagenicity
A13179	Skin and Eye Irritation
A13180	Mutagenicity, Skin and Eye Irritation, Multiple Dose Effects
A13181	Acute Toxicity
A13182	Mutagenicity
A13183	Acute Toxicity
A13184	Skin and Eye Irritation
A13185	Acute Toxicity
A13186	Acute Toxicity
A13187	Acute Toxicity
A13188	Acute Toxicity
A13189	Acute Toxicity
A13190	Acute Toxicity
A13191	Acute Toxicity
A13192	Acute Toxicity
A13193	Acute Toxicity
A13194	Acute Toxicity
A13195	Acute Toxicity
A13196	Acute Toxicity
A13197	Acute Toxicity
A13198	Acute Toxicity
A13199	Acute Toxicity
A13200	Acute Toxicity
A13201	Tumorigenicity
A13202	Acute Toxicity
A13203	Acute Toxicity
A13204	Acute Toxicity
A13205	Acute Toxicity
A13206	Mutagenicity
A13207	Acute Toxicity
A13208	Acute Toxicity, Multiple Dose Effects

A13209	Mutagenicity
A13210	Acute Toxicity
A13211	Non-toxicity
A13212	Acute Toxicity, Mutagenicity, Skin and Eye Irritation, Reproductive Effects, Multiple Dose Effects
A13213	Acute Toxicity, Multiple Dose Effects
A13214	Acute Toxicity
A13215	Acute Toxicity, Mutagenicity
A13216	Non-toxicity
A13217	Acute Toxicity
A13218	Acute Toxicity
A13219	Acute Toxicity
A13220	Acute Toxicity
A13221	Acute Toxicity
A13222	Reproductive Effects, Multiple Dose Effects
A13223	Mutagenicity
A13224	Skin and Eye Irritation
A13225	Acute Toxicity
A13226	Mutagenicity
A13227	Acute Toxicity
A13228	Acute Toxicity
A13229	Mutagenicity, Multiple Dose Effects
A13230	Acute Toxicity
A13231	Acute Toxicity, Multiple Dose Effects
A13232	Acute Toxicity
A13233	Acute Toxicity
A13234	Non-toxicity
A13235	Multiple Dose Effects
A13236	Acute Toxicity
A13237	Mutagenicity
A13238	Mutagenicity, Tumorigenicity
A13239	Acute Toxicity
A13240	Acute Toxicity
A13241	Non-toxicity
A13242	Mutagenicity
A13243	Acute Toxicity
A13244	Acute Toxicity
A13245	Acute Toxicity
A13246	Acute Toxicity
A13247	Acute Toxicity
A13248	Reproductive Effects
A13249	Acute Toxicity, Reproductive Effects
A13250	Mutagenicity, Skin and Eye Irritation, Reproductive Effects, Multiple Dose
A13251	Acute Toxicity
A13252	Multiple Dose Effects
A13253	Acute Toxicity

A13254	Acute Toxicity
A13255	Non-toxicity
A13256	Acute Toxicity
A13257	Acute Toxicity
A13258	Acute Toxicity
A13259	Multiple Dose Effects
A13260	Acute Toxicity
A13261	Acute Toxicity
A13262	Acute Toxicity
A13263	Skin and Eye Irritation
A13264	Acute Toxicity
A13265	Acute Toxicity
A13266	Acute Toxicity
A13267	Acute Toxicity
A13268	Skin and Eye Irritation
A13269	Multiple Dose Effects
A13270	Acute Toxicity
A13271	Acute Toxicity
A13272	Acute Toxicity
A13273	Acute Toxicity
A13274	Acute Toxicity
A13275	Acute Toxicity
A13276	Acute Toxicity
A13277	Acute Toxicity
A13278	Acute Toxicity
A13279	Acute Toxicity
A13280	Acute Toxicity
A13281	Acute Toxicity
A13282	Acute Toxicity, Reproductive Effects
A13283	Reproductive Effects
A13284	Reproductive Effects
A13285	Mutagenicity
A13286	Acute Toxicity
A13287	Acute Toxicity, Skin and Eye Irritation
A13288	Skin and Eye Irritation
A13289	Acute Toxicity
A13290	Acute Toxicity
A13291	Acute Toxicity
A13292	Acute Toxicity
A13293	Reproductive Effects
A13294	Acute Toxicity
A13295	Acute Toxicity, Multiple Dose Effects
A13296	Acute Toxicity
A13297	Mutagenicity
A13298	Acute Toxicity, Multiple Dose Effects
A13299	Acute Toxicity

A13300	Mutagenicity
A13301	Acute Toxicity
A13302	Acute Toxicity, Multiple Dose Effects
A13303	Acute Toxicity
A13304	Skin and Eye Irritation
A13305	Mutagenicity
A13306	Acute Toxicity
A13307	Acute Toxicity
A13308	Acute Toxicity
A13309	Reproductive Effects, Multiple Dose Effects
A13310	Acute Toxicity
A13311	Acute Toxicity, Multiple Dose Effects
A13312	Acute Toxicity
A13313	Acute Toxicity, Multiple Dose Effects
A13314	Acute Toxicity, Tumorigenicity, Reproductive Effects, Multiple Dose Effects
A13315	Acute Toxicity
A13316	Acute Toxicity
A13317	Multiple Dose Effects
A13318	Acute Toxicity
A13319	Acute Toxicity
A13320	Acute Toxicity
A13321	Multiple Dose Effects
A13322	Reproductive Effects
A13323	Multiple Dose Effects
A13324	Acute Toxicity
A13325	Acute Toxicity
A13326	Reproductive Effects, Multiple Dose Effects
A13327	Reproductive Effects
A13328	Acute Toxicity, Multiple Dose Effects
A13329	Reproductive Effects
A13330	Acute Toxicity, Multiple Dose Effects
A13331	Acute Toxicity
A13332	Acute Toxicity, Reproductive Effects, Multiple Dose Effects
A13333	Acute Toxicity
A13334	Mutagenicity
A13335	Acute Toxicity
A13336	Non-toxicity
A13337	Mutagenicity
A13338	Tumorigenicity, Multiple Dose Effects
A13339	Acute Toxicity
A13340	Acute Toxicity, Multiple Dose Effects
A13341	Tumorigenicity
A13342	Acute Toxicity
A13343	Acute Toxicity
A13344	Non-toxicity
A13345	Acute Toxicity

A13346	Acute Toxicity, Mutagenicity, Tumorigenicity, Skin and Eye Irritation,
A13347	Acute Toxicity, Tumorigenicity, Reproductive Effects, Multiple Dose Effects
A13348	Acute Toxicity
A13349	Mutagenicity
A13350	Non-toxicity
A13351	Mutagenicity, Tumorigenicity
A13352	Acute Toxicity
A13353	Acute Toxicity
A13354	Acute Toxicity
A13355	Multiple Dose Effects
A13356	Acute Toxicity
A13357	Acute Toxicity
A13358	Acute Toxicity
A13359	Non-toxicity
A13360	Acute Toxicity
A13361	Acute Toxicity, Mutagenicity, Multiple Dose Effects
A13362	Acute Toxicity, Reproductive Effects
A13363	Multiple Dose Effects
A13364	Multiple Dose Effects
A13365	Acute Toxicity
A13366	Skin and Eye Irritation
A13367	Multiple Dose Effects
A13368	Non-toxicity
A13369	Acute Toxicity, Mutagenicity, Tumorigenicity, Reproductive Effects,
A13370	Mutagenicity
A13371	Mutagenicity
A13372	Acute Toxicity
A13373	Skin and Eye Irritation
A13374	Acute Toxicity
A13375	Multiple Dose Effects
A13376	Acute Toxicity
A13377	Acute Toxicity
A13378	Mutagenicity
A13379	Acute Toxicity
A13380	Multiple Dose Effects
A13381	Acute Toxicity, Multiple Dose Effects
A13382	Mutagenicity
A13383	Acute Toxicity, Reproductive Effects
A13384	Acute Toxicity, Tumorigenicity, Multiple Dose Effects
A13385	Multiple Dose Effects
A13386	Acute Toxicity
A13387	Acute Toxicity, Mutagenicity, Tumorigenicity, Reproductive Effects,
A13388	Acute Toxicity, Multiple Dose Effects
A13389	Acute Toxicity
A13390	Non-toxicity
A13391	Non-toxicity

A13392	Skin and Eye Irritation
A13393	Non-toxicity
A13394	Acute Toxicity
A13395	Non-toxicity
A13396	Mutagenicity
A13397	Acute Toxicity, Reproductive Effects, Multiple Dose Effects
A13398	Acute Toxicity
A13399	Multiple Dose Effects
A13400	Acute Toxicity
A13401	Non-toxicity
A13402	Multiple Dose Effects
A13403	Acute Toxicity
A13404	Acute Toxicity
A13405	Non-toxicity
A13406	Multiple Dose Effects
A13407	Mutagenicity
A13408	Mutagenicity
A13409	Mutagenicity
A13410	Mutagenicity
A13411	Mutagenicity
A13412	Mutagenicity
A13413	Acute Toxicity
A13414	Acute Toxicity
A13415	Mutagenicity
A13416	Acute Toxicity
A13417	Acute Toxicity, Mutagenicity
A13418	Acute Toxicity
A13419	Mutagenicity
A13420	Reproductive Effects, Multiple Dose Effects
A13421	Mutagenicity
A13422	Mutagenicity
A13423	Mutagenicity
A13424	Acute Toxicity, Reproductive Effects
A13425	Acute Toxicity
A13426	Acute Toxicity
A13427	Acute Toxicity
A13428	Acute Toxicity, Multiple Dose Effects
A13429	Acute Toxicity, Multiple Dose Effects
A13430	Reproductive Effects
A13431	Acute Toxicity, Mutagenicity
A13432	Acute Toxicity
A13433	Acute Toxicity, Skin and Eye Irritation, Multiple Dose Effects
A13434	Reproductive Effects
A13435	Acute Toxicity
A13436	Acute Toxicity, Skin and Eye Irritation
A13437	Acute Toxicity

A13438	Acute Toxicity
A13439	Acute Toxicity
A13440	Acute Toxicity
A13441	Acute Toxicity
A13442	Acute Toxicity
A13443	Acute Toxicity
A13444	Acute Toxicity
A13445	Acute Toxicity
A13446	Acute Toxicity
A13447	Acute Toxicity
A13448	Acute Toxicity
A13449	Acute Toxicity
A13450	Acute Toxicity
A13451	Acute Toxicity
A13452	Acute Toxicity
A13453	Mutagenicity
A13454	Acute Toxicity
A13455	Acute Toxicity
A13456	Acute Toxicity, Multiple Dose Effects
A13457	Acute Toxicity
A13458	Acute Toxicity
A13459	Acute Toxicity
A13460	Acute Toxicity
A13461	Acute Toxicity
A13462	Acute Toxicity
A13463	Acute Toxicity
A13464	Acute Toxicity
A13465	Acute Toxicity
A13466	Acute Toxicity
A13467	Acute Toxicity
A13468	Reproductive Effects
A13469	Acute Toxicity
A13470	Acute Toxicity
A13471	Acute Toxicity
A13472	Acute Toxicity
A13473	Acute Toxicity
A13474	Acute Toxicity
A13475	Acute Toxicity
A13476	Acute Toxicity
A13477	Acute Toxicity
A13478	Acute Toxicity
A13479	Multiple Dose Effects
A13480	Acute Toxicity
A13481	Acute Toxicity
A13482	Multiple Dose Effects
A13483	Acute Toxicity

A13484	Acute Toxicity
A13485	Mutagenicity
A13486	Acute Toxicity
A13487	Acute Toxicity
A13488	Multiple Dose Effects
A13489	Acute Toxicity
A13490	Acute Toxicity, Reproductive Effects, Multiple Dose Effects
A13491	Acute Toxicity
A13492	Acute Toxicity
A13493	Acute Toxicity
A13494	Acute Toxicity
A13495	Acute Toxicity, Mutagenicity
A13496	Mutagenicity
A13497	Acute Toxicity
A13498	Acute Toxicity
A13499	Acute Toxicity
A13500	Acute Toxicity, Skin and Eye Irritation
A13501	Acute Toxicity
A13502	Acute Toxicity
A13503	Mutagenicity
A13504	Acute Toxicity
A13505	Acute Toxicity
A13506	Acute Toxicity
A13507	Acute Toxicity
A13508	Acute Toxicity
A13509	Acute Toxicity
A13510	Acute Toxicity
A13511	Acute Toxicity, Skin and Eye Irritation
A13512	Acute Toxicity
A13513	Tumorigenicity
A13514	Acute Toxicity
A13515	Acute Toxicity
A13516	Acute Toxicity
A13517	Acute Toxicity
A13518	Acute Toxicity
A13519	Acute Toxicity
A13520	Acute Toxicity
A13521	Acute Toxicity, Multiple Dose Effects
A13522	Acute Toxicity
A13523	Acute Toxicity, Multiple Dose Effects
A13524	Acute Toxicity
A13525	Acute Toxicity
A13526	Acute Toxicity
A13527	Acute Toxicity
A13528	Acute Toxicity, Multiple Dose Effects
A13529	Acute Toxicity, Multiple Dose Effects

A13530	Acute Toxicity
A13531	Acute Toxicity
A13532	Acute Toxicity
A13533	Acute Toxicity
A13534	Acute Toxicity
A13535	Acute Toxicity
A13536	Acute Toxicity
A13537	Acute Toxicity
A13538	Acute Toxicity, Multiple Dose Effects
A13539	Acute Toxicity
A13540	Acute Toxicity
A13541	Acute Toxicity
A13542	Acute Toxicity
A13543	Acute Toxicity
A13544	Acute Toxicity
A13545	Acute Toxicity
A13546	Acute Toxicity
A13547	Acute Toxicity
A13548	Acute Toxicity
A13549	Acute Toxicity
A13550	Acute Toxicity
A13551	Acute Toxicity
A13552	Acute Toxicity
A13553	Acute Toxicity, Multiple Dose Effects
A13554	Acute Toxicity
A13555	Acute Toxicity
A13556	Acute Toxicity
A13557	Acute Toxicity
A13558	Acute Toxicity
A13559	Acute Toxicity
A13560	Acute Toxicity
A13561	Acute Toxicity
A13562	Acute Toxicity
A13563	Acute Toxicity
A13564	Acute Toxicity
A13565	Acute Toxicity
A13566	Acute Toxicity
A13567	Acute Toxicity
A13568	Reproductive Effects
A13569	Acute Toxicity
A13570	Acute Toxicity
A13571	Acute Toxicity
A13572	Acute Toxicity
A13573	Acute Toxicity
A13574	Acute Toxicity
A13575	Acute Toxicity

A13576	Acute Toxicity
A13577	Acute Toxicity
A13578	Acute Toxicity
A13579	Acute Toxicity
A13580	Acute Toxicity
A13581	Acute Toxicity
A13582	Acute Toxicity
A13583	Acute Toxicity
A13584	Acute Toxicity
A13585	Acute Toxicity
A13586	Acute Toxicity
A13587	Acute Toxicity
A13588	Acute Toxicity
A13589	Acute Toxicity
A13590	Acute Toxicity
A13591	Acute Toxicity
A13592	Acute Toxicity
A13593	Acute Toxicity
A13594	Acute Toxicity
A13595	Acute Toxicity
A13596	Acute Toxicity
A13597	Acute Toxicity
A13598	Acute Toxicity
A13599	Acute Toxicity
A13600	Acute Toxicity
A13601	Acute Toxicity
A13602	Acute Toxicity
A13603	Acute Toxicity
A13604	Acute Toxicity
A13605	Acute Toxicity
A13606	Acute Toxicity
A13607	Acute Toxicity
A13608	Acute Toxicity
A13609	Acute Toxicity, Skin and Eye Irritation
A13610	Acute Toxicity
A13611	Acute Toxicity
A13612	Acute Toxicity
A13613	Acute Toxicity
A13614	Acute Toxicity, Multiple Dose Effects
A13615	Acute Toxicity
A13616	Acute Toxicity
A13617	Acute Toxicity, Multiple Dose Effects
A13618	Acute Toxicity
A13619	Acute Toxicity
A13620	Acute Toxicity
A13621	Acute Toxicity

A13622	Acute Toxicity
A13623	Acute Toxicity
A13624	Acute Toxicity, Multiple Dose Effects
A13625	Acute Toxicity
A13626	Acute Toxicity
A13627	Acute Toxicity
A13628	Acute Toxicity
A13629	Acute Toxicity
A13630	Acute Toxicity
A13631	Acute Toxicity
A13632	Acute Toxicity
A13633	Acute Toxicity
A13634	Acute Toxicity
A13635	Acute Toxicity
A13636	Acute Toxicity
A13637	Acute Toxicity
A13638	Acute Toxicity
A13639	Acute Toxicity
A13640	Acute Toxicity
A13641	Acute Toxicity
A13642	Acute Toxicity
A13643	Acute Toxicity
A13644	Acute Toxicity
A13645	Acute Toxicity
A13646	Acute Toxicity
A13647	Acute Toxicity, Multiple Dose Effects
A13648	Mutagenicity
A13649	Acute Toxicity
A13650	Acute Toxicity
A13651	Acute Toxicity
A13652	Acute Toxicity
A13653	Acute Toxicity
A13654	Acute Toxicity
A13655	Acute Toxicity
A13656	Acute Toxicity
A13657	Acute Toxicity
A13658	Acute Toxicity
A13659	Acute Toxicity
A13660	Acute Toxicity
A13661	Mutagenicity
A13662	Acute Toxicity
A13663	Acute Toxicity
A13664	Acute Toxicity
A13665	Acute Toxicity
A13666	Acute Toxicity
A13667	Acute Toxicity

A13668	Acute Toxicity
A13669	Acute Toxicity
A13670	Acute Toxicity
A13671	Acute Toxicity
A13672	Acute Toxicity
A13673	Acute Toxicity
A13674	Acute Toxicity
A13675	Acute Toxicity
A13676	Acute Toxicity
A13677	Acute Toxicity, Multiple Dose Effects
A13678	Acute Toxicity
A13679	Acute Toxicity
A13680	Acute Toxicity
A13681	Acute Toxicity
A13682	Acute Toxicity
A13683	Acute Toxicity, Mutagenicity
A13684	Acute Toxicity, Mutagenicity
A13685	Acute Toxicity
A13686	Acute Toxicity
A13687	Acute Toxicity
A13688	Acute Toxicity
A13689	Acute Toxicity
A13690	Acute Toxicity
A13691	Acute Toxicity
A13692	Acute Toxicity
A13693	Acute Toxicity
A13694	Acute Toxicity
A13695	Acute Toxicity
A13696	Acute Toxicity
A13697	Acute Toxicity
A13698	Acute Toxicity
A13699	Acute Toxicity
A13700	Acute Toxicity
A13701	Acute Toxicity
A13702	Acute Toxicity
A13703	Acute Toxicity
A13704	Mutagenicity
A13705	Acute Toxicity
A13706	Multiple Dose Effects
A13707	Acute Toxicity
A13708	Multiple Dose Effects
A13709	Acute Toxicity
A13710	Acute Toxicity
A13711	Acute Toxicity
A13712	Acute Toxicity
A13713	Acute Toxicity

A13714	Acute Toxicity
A13715	Acute Toxicity
A13716	Acute Toxicity
A13717	Acute Toxicity, Reproductive Effects, Multiple Dose Effects
A13718	Acute Toxicity
A13719	Acute Toxicity
A13720	Acute Toxicity
A13721	Acute Toxicity
A13722	Acute Toxicity
A13723	Acute Toxicity
A13724	Acute Toxicity
A13725	Tumorigenicity
A13726	Acute Toxicity, Multiple Dose Effects
A13727	Acute Toxicity
A13728	Acute Toxicity, Multiple Dose Effects
A13729	Reproductive Effects
A13730	Acute Toxicity
A13731	Acute Toxicity
A13732	Acute Toxicity
A13733	Acute Toxicity, Tumorigenicity
A13734	Reproductive Effects, Multiple Dose Effects
A13735	Acute Toxicity, Reproductive Effects, Multiple Dose Effects
A13736	Mutagenicity, Multiple Dose Effects
A13737	Reproductive Effects
A13738	Multiple Dose Effects
A13739	Acute Toxicity
A13740	Acute Toxicity
A13741	Acute Toxicity
A13742	Acute Toxicity
A13743	Acute Toxicity
A13744	Acute Toxicity
A13745	Mutagenicity
A13746	Acute Toxicity
A13747	Acute Toxicity
A13748	Acute Toxicity
A13749	Acute Toxicity
A13750	Acute Toxicity
A13751	Acute Toxicity, Multiple Dose Effects
A13752	Acute Toxicity
A13753	Acute Toxicity
A13754	Acute Toxicity
A13755	Acute Toxicity, Skin and Eye Irritation
A13756	Multiple Dose Effects
A13757	Acute Toxicity
A13758	Acute Toxicity
A13759	Acute Toxicity

A13760	Mutagenicity
A13761	Acute Toxicity
A13762	Acute Toxicity
A13763	Acute Toxicity
A13764	Acute Toxicity
A13765	Acute Toxicity
A13766	Mutagenicity
A13767	Acute Toxicity
A13768	Acute Toxicity
A13769	Acute Toxicity
A13770	Acute Toxicity
A13771	Acute Toxicity
A13772	Acute Toxicity
A13773	Acute Toxicity
A13774	Acute Toxicity
A13775	Acute Toxicity
A13776	Acute Toxicity
A13777	Acute Toxicity
A13778	Acute Toxicity, Mutagenicity
A13779	Acute Toxicity
A13780	Acute Toxicity, Multiple Dose Effects
A13781	Multiple Dose Effects
A13782	Acute Toxicity
A13783	Multiple Dose Effects
A13784	Reproductive Effects, Multiple Dose Effects
A13785	Multiple Dose Effects
A13786	Reproductive Effects, Multiple Dose Effects
A13787	Acute Toxicity
A13788	Multiple Dose Effects
A13789	Acute Toxicity
A13790	Mutagenicity
A13791	Acute Toxicity
A13792	Acute Toxicity
A13793	Acute Toxicity
A13794	Acute Toxicity
A13795	Acute Toxicity
A13796	Acute Toxicity
A13797	Acute Toxicity, Skin and Eye Irritation
A13798	Acute Toxicity
A13799	Acute Toxicity
A13800	Acute Toxicity
A13801	Acute Toxicity
A13802	Acute Toxicity
A13803	Acute Toxicity
A13804	Acute Toxicity
A13805	Acute Toxicity

A13806	Multiple Dose Effects
A13807	Acute Toxicity
A13808	Acute Toxicity
A13809	Acute Toxicity
A13810	Non-toxicity
A13811	Acute Toxicity, Tumorigenicity
A13812	Acute Toxicity
A13813	Acute Toxicity
A13814	Acute Toxicity, Mutagenicity, Reproductive Effects
A13815	Tumorigenicity
A13816	Acute Toxicity
A13817	Acute Toxicity, Skin and Eye Irritation
A13818	Skin and Eye Irritation
A13819	Acute Toxicity, Mutagenicity, Tumorigenicity, Reproductive Effects
A13820	Mutagenicity
A13821	Acute Toxicity, Skin and Eye Irritation
A13822	Acute Toxicity
A13823	Acute Toxicity
A13824	Acute Toxicity, Mutagenicity, Tumorigenicity, Multiple Dose Effects
A13825	Acute Toxicity
A13826	Acute Toxicity, Tumorigenicity, Reproductive Effects, Multiple Dose Effects
A13827	Acute Toxicity, Mutagenicity, Tumorigenicity
A13828	Acute Toxicity, Mutagenicity, Skin and Eye Irritation
A13829	Acute Toxicity, Mutagenicity, Tumorigenicity, Reproductive Effects,
A13830	Acute Toxicity, Mutagenicity, Tumorigenicity, Reproductive Effects,
A13831	Mutagenicity, Reproductive Effects, Multiple Dose Effects
A13832	Mutagenicity, Tumorigenicity, Multiple Dose Effects
A13833	Tumorigenicity
A13834	Acute Toxicity, Multiple Dose Effects
A13835	Skin and Eye Irritation, Reproductive Effects
A13836	Acute Toxicity
A13837	Acute Toxicity, Skin and Eye Irritation, Multiple Dose Effects
A13838	Acute Toxicity
A13839	Mutagenicity, Tumorigenicity
A13840	Acute Toxicity
A13841	Acute Toxicity
A13842	Acute Toxicity
A13843	Mutagenicity
A13844	Acute Toxicity, Reproductive Effects
A13845	Acute Toxicity, Mutagenicity, Tumorigenicity, Reproductive Effects,
A13846	Acute Toxicity, Reproductive Effects
A13847	Multiple Dose Effects
A13848	Mutagenicity
A13849	Mutagenicity
A13850	Multiple Dose Effects
A13851	Mutagenicity

A13852	Acute Toxicity, Mutagenicity
A13853	Acute Toxicity
A13854	Acute Toxicity
A13855	Acute Toxicity, Mutagenicity, Reproductive Effects, Multiple Dose Effects
A13856	Acute Toxicity
A13857	Multiple Dose Effects
A13858	Acute Toxicity, Skin and Eye Irritation, Reproductive Effects
A13859	Reproductive Effects
A13860	Acute Toxicity, Mutagenicity, Tumorigenicity, Multiple Dose Effects
A13861	Acute Toxicity
A13862	Acute Toxicity
A13863	Acute Toxicity
A13864	Acute Toxicity, Reproductive Effects
A13865	Acute Toxicity, Mutagenicity
A13866	Acute Toxicity, Mutagenicity, Reproductive Effects, Multiple Dose Effects
A13867	Reproductive Effects, Multiple Dose Effects
A13868	Acute Toxicity, Mutagenicity
A13869	Mutagenicity
A13870	Acute Toxicity
A13871	Acute Toxicity, Mutagenicity, Reproductive Effects, Multiple Dose Effects
A13872	Acute Toxicity, Mutagenicity, Skin and Eye Irritation
A13873	Reproductive Effects
A13874	Mutagenicity, Tumorigenicity, Reproductive Effects, Multiple Dose Effects
A13875	Acute Toxicity
A13876	Acute Toxicity, Skin and Eye Irritation
A13877	Acute Toxicity
A13878	Reproductive Effects
A13879	Acute Toxicity, Mutagenicity, Multiple Dose Effects
A13880	Multiple Dose Effects
A13881	Tumorigenicity, Reproductive Effects, Multiple Dose Effects
A13882	Tumorigenicity, Reproductive Effects
A13883	Acute Toxicity
A13884	Acute Toxicity, Tumorigenicity
A13885	Acute Toxicity, Mutagenicity, Tumorigenicity
A13886	Acute Toxicity, Mutagenicity, Multiple Dose Effects
A13887	Acute Toxicity, Reproductive Effects
A13888	Acute Toxicity
A13889	Acute Toxicity
A13890	Mutagenicity, Tumorigenicity
A13891	Reproductive Effects, Multiple Dose Effects
A13892	Acute Toxicity, Reproductive Effects, Multiple Dose Effects
A13893	Mutagenicity, Skin and Eye Irritation
A13894	Mutagenicity, Multiple Dose Effects
A13895	Mutagenicity, Multiple Dose Effects
A13896	Acute Toxicity
A13897	Acute Toxicity

A13898	Acute Toxicity
A13899	Acute Toxicity, Multiple Dose Effects
A13900	Acute Toxicity
A13901	Acute Toxicity
A13902	Mutagenicity
A13903	Acute Toxicity, Mutagenicity
A13904	Acute Toxicity
A13905	Acute Toxicity, Mutagenicity, Multiple Dose Effects
A13906	Acute Toxicity, Mutagenicity, Tumorigenicity, Multiple Dose Effects
A13907	Acute Toxicity, Skin and Eye Irritation
A13908	Acute Toxicity
A13909	Acute Toxicity
A13910	Acute Toxicity
A13911	Mutagenicity
A13912	Acute Toxicity, Multiple Dose Effects
A13913	Non-toxicity
A13914	Acute Toxicity, Mutagenicity, Skin and Eye Irritation
A13915	Acute Toxicity, Multiple Dose Effects
A13916	Acute Toxicity
A13917	Acute Toxicity, Mutagenicity, Multiple Dose Effects
A13918	Acute Toxicity
A13919	Acute Toxicity
A13920	Acute Toxicity
A13921	Skin and Eye Irritation
A13922	Mutagenicity
A13923	Acute Toxicity, Multiple Dose Effects
A13924	Mutagenicity
A13925	Acute Toxicity
A13926	Acute Toxicity, Mutagenicity, Multiple Dose Effects
A13927	Acute Toxicity
A13928	Acute Toxicity, Multiple Dose Effects
A13929	Acute Toxicity, Mutagenicity, Reproductive Effects, Multiple Dose Effects
A13930	Acute Toxicity
A13931	Mutagenicity
A13932	Acute Toxicity, Mutagenicity
A13933	Acute Toxicity, Mutagenicity
A13934	Acute Toxicity
A13935	Mutagenicity
A13936	Acute Toxicity, Mutagenicity
A13937	Multiple Dose Effects
A13938	Acute Toxicity, Mutagenicity
A13939	Acute Toxicity, Mutagenicity
A13940	Tumorigenicity
A13941	Mutagenicity
A13942	Acute Toxicity, Mutagenicity, Tumorigenicity
A13943	Acute Toxicity

A13944	Mutagenicity, Multiple Dose Effects
A13945	Acute Toxicity, Tumorigenicity
A13946	Acute Toxicity
A13947	Acute Toxicity
A13948	Reproductive Effects, Multiple Dose Effects
A13949	Acute Toxicity, Multiple Dose Effects
A13950	Acute Toxicity
A13951	Mutagenicity
A13952	Acute Toxicity
A13953	Mutagenicity
A13954	Acute Toxicity
A13955	Acute Toxicity
A13956	Acute Toxicity
A13957	Acute Toxicity
A13958	Non-toxicity
A13959	Mutagenicity, Multiple Dose Effects
A13960	Acute Toxicity
A13961	Acute Toxicity
A13962	Non-toxicity
A13963	Acute Toxicity, Multiple Dose Effects
A13964	Acute Toxicity
A13965	Multiple Dose Effects
A13966	Acute Toxicity
A13967	Mutagenicity, Multiple Dose Effects
A13968	Multiple Dose Effects
A13969	Multiple Dose Effects
A13970	Acute Toxicity, Skin and Eye Irritation
A13971	Acute Toxicity
A13972	Acute Toxicity, Mutagenicity, Tumorigenicity
A13973	Acute Toxicity, Skin and Eye Irritation
A13974	Acute Toxicity
A13975	Non-toxicity
A13976	Multiple Dose Effects
A13977	Acute Toxicity, Mutagenicity
A13978	Mutagenicity
A13979	Acute Toxicity
A13980	Non-toxicity
A13981	Acute Toxicity
A13982	Non-toxicity
A13983	Non-toxicity
A13984	Non-toxicity
A13985	Non-toxicity
A13986	Reproductive Effects
A13987	Mutagenicity, Reproductive Effects, Multiple Dose Effects
A13988	Skin and Eye Irritation, Multiple Dose Effects

A13989	Acute Toxicity, Mutagenicity, Skin and Eye Irritation, Reproductive Effects, Multiple Dose Effects
A13990	Non-toxicity
A13991	Non-toxicity
A13992	Acute Toxicity, Mutagenicity, Reproductive Effects, Multiple Dose Effects
A13993	Acute Toxicity, Reproductive Effects, Multiple Dose Effects
A13994	Non-toxicity
A13995	Acute Toxicity
A13996	Non-toxicity
A13997	Acute Toxicity, Mutagenicity
A13998	Non-toxicity
A13999	Non-toxicity
A14000	Acute Toxicity, Mutagenicity, Multiple Dose Effects
A14001	Non-toxicity
A14002	Acute Toxicity
A14003	Tumorigenicity
A14004	Non-toxicity
A14005	Mutagenicity
A14006	Acute Toxicity, Mutagenicity, Multiple Dose Effects
A14007	Acute Toxicity
A14008	Acute Toxicity
A14009	Acute Toxicity, Mutagenicity, Skin and Eye Irritation, Reproductive Effects
A14010	Acute Toxicity, Mutagenicity, Multiple Dose Effects
A14011	Multiple Dose Effects
A14012	Acute Toxicity, Skin and Eye Irritation
A14013	Non-toxicity
A14014	Acute Toxicity
A14015	Non-toxicity
A14016	Acute Toxicity, Reproductive Effects
A14017	Acute Toxicity
A14018	Acute Toxicity
A14019	Mutagenicity
A14020	Non-toxicity
A14021	Non-toxicity
A14022	Non-toxicity
A14023	Non-toxicity
A14024	Acute Toxicity, Multiple Dose Effects
A14025	Non-toxicity
A14026	Acute Toxicity, Mutagenicity
A14027	Non-toxicity
A14028	Non-toxicity
A14029	Mutagenicity
A14030	Non-toxicity
A14031	Non-toxicity
A14032	Non-toxicity
A14033	Non-toxicity

A14034	Non-toxicity
A14035	Non-toxicity
A14036	Mutagenicity
A14037	Mutagenicity
A14038	Non-toxicity
A14039	Acute Toxicity
A14040	Non-toxicity
A14041	Acute Toxicity
A14042	Non-toxicity
A14043	Non-toxicity
A14044	Non-toxicity
A14045	Non-toxicity
A14046	Non-toxicity
A14047	Non-toxicity
A14048	Mutagenicity, Reproductive Effects
A14049	Acute Toxicity
A14050	Non-toxicity
A14051	Mutagenicity
A14052	Mutagenicity
A14053	Non-toxicity
A14054	Mutagenicity
A14055	Acute Toxicity
A14056	Acute Toxicity, Mutagenicity, Multiple Dose Effects
A14057	Non-toxicity
A14058	Multiple Dose Effects
A14059	Non-toxicity
A14060	Non-toxicity
A14061	Non-toxicity
A14062	Non-toxicity
A14063	Non-toxicity
A14064	Non-toxicity
A14065	Non-toxicity
A14066	Non-toxicity
A14067	Non-toxicity
A14068	Non-toxicity
A14069	Non-toxicity
A14070	Acute Toxicity, Reproductive Effects
A14071	Acute Toxicity, Mutagenicity, Tumorigenicity, Skin and Eye Irritation,
A14072	Acute Toxicity, Mutagenicity, Reproductive Effects
A14073	Acute Toxicity, Mutagenicity, Reproductive Effects
A14074	Acute Toxicity, Mutagenicity, Skin and Eye Irritation, Multiple Dose Effects
A14075	Non-toxicity
A14076	Acute Toxicity
A14077	Acute Toxicity, Mutagenicity, Tumorigenicity, Skin and Eye Irritation,
A14078	Non-toxicity
A14079	Acute Toxicity, Skin and Eye Irritation, Multiple Dose Effects

A14080	Acute Toxicity, Tumorigenicity, Skin and Eye Irritation, Reproductive Effects, Multiple Dose Effects
A14081	Non-toxicity
A14082	Mutagenicity
A14083	Acute Toxicity
A14084	Mutagenicity
A14085	Acute Toxicity, Multiple Dose Effects
A14086	Acute Toxicity
A14087	Non-toxicity
A14088	Acute Toxicity, Skin and Eye Irritation, Reproductive Effects, Multiple Dose
A14089	Non-toxicity
A14090	Non-toxicity
A14091	Acute Toxicity, Multiple Dose Effects
A14092	Skin and Eye Irritation
A14093	Non-toxicity
A14094	Multiple Dose Effects
A14095	Acute Toxicity, Mutagenicity, Reproductive Effects, Multiple Dose Effects
A14096	Acute Toxicity, Multiple Dose Effects
A14097	Acute Toxicity, Mutagenicity, Multiple Dose Effects
A14098	Acute Toxicity
A14099	Acute Toxicity
A14100	Acute Toxicity
A14101	Acute Toxicity
A14102	Acute Toxicity
A14103	Acute Toxicity, Mutagenicity
A14104	Multiple Dose Effects
A14105	Acute Toxicity
A14106	Acute Toxicity
A14107	Acute Toxicity
A14108	Acute Toxicity
A14109	Acute Toxicity, Multiple Dose Effects
A14110	Acute Toxicity
A14111	Tumorigenicity, Skin and Eye Irritation, Multiple Dose Effects
A14112	Skin and Eye Irritation
A14113	Mutagenicity, Tumorigenicity, Skin and Eye Irritation, Reproductive Effects
A14114	Acute Toxicity
A14115	Acute Toxicity
A14116	Skin and Eye Irritation
A14117	Skin and Eye Irritation
A14118	Acute Toxicity, Multiple Dose Effects
A14119	Mutagenicity
A14120	Acute Toxicity, Mutagenicity
A14121	Acute Toxicity
A14122	Acute Toxicity, Multiple Dose Effects
A14123	Acute Toxicity
A14124	Acute Toxicity, Mutagenicity

A14125	Acute Toxicity, Mutagenicity, Multiple Dose Effects
A14126	Multiple Dose Effects
A14127	Mutagenicity, Tumorigenicity
A14128	Acute Toxicity, Mutagenicity, Tumorigenicity, Reproductive Effects,
A14129	Acute Toxicity, Tumorigenicity, Multiple Dose Effects
A14130	Acute Toxicity
A14131	Acute Toxicity, Reproductive Effects
A14132	Acute Toxicity, Mutagenicity
A14133	Multiple Dose Effects
A14134	Mutagenicity
A14135	Acute Toxicity, Mutagenicity, Tumorigenicity
A14136	Acute Toxicity
A14137	Acute Toxicity
A14138	Acute Toxicity, Reproductive Effects
A14139	Tumorigenicity
A14140	Acute Toxicity, Multiple Dose Effects
A14141	Acute Toxicity
A14142	Acute Toxicity
A14143	Acute Toxicity, Reproductive Effects, Multiple Dose Effects
A14144	Acute Toxicity, Mutagenicity
A14145	Multiple Dose Effects
A14146	Acute Toxicity
A14147	Acute Toxicity, Multiple Dose Effects
A14148	Acute Toxicity
A14149	Multiple Dose Effects
A14150	Acute Toxicity, Tumorigenicity, Reproductive Effects
A14151	Acute Toxicity, Mutagenicity
A14152	Acute Toxicity
A14153	Acute Toxicity, Reproductive Effects
A14154	Acute Toxicity, Skin and Eye Irritation, Reproductive Effects
A14155	Non-toxicity
A14156	Acute Toxicity, Mutagenicity
A14157	Non-toxicity
A14158	Skin and Eye Irritation
A14159	Acute Toxicity
A14160	Acute Toxicity, Mutagenicity
A14161	Acute Toxicity, Multiple Dose Effects
A14162	Mutagenicity
A14163	Acute Toxicity, Mutagenicity, Reproductive Effects, Multiple Dose Effects
A14164	Non-toxicity
A14165	Acute Toxicity, Reproductive Effects, Multiple Dose Effects
A14166	Acute Toxicity, Reproductive Effects, Multiple Dose Effects
A14167	Acute Toxicity, Multiple Dose Effects
A14168	Acute Toxicity, Mutagenicity, Multiple Dose Effects
A14169	Acute Toxicity
A14170	Acute Toxicity, Skin and Eye Irritation, Reproductive Effects

A14171	Acute Toxicity, Skin and Eye Irritation, Reproductive Effects, Multiple Dose
A14172	Non-toxicity
A14173	Acute Toxicity, Mutagenicity, Skin and Eye Irritation, Multiple Dose Effects
A14174	Acute Toxicity, Tumorigenicity, Reproductive Effects
A14175	Acute Toxicity, Reproductive Effects, Multiple Dose Effects
A14176	Acute Toxicity, Reproductive Effects, Multiple Dose Effects
A14177	Acute Toxicity, Skin and Eye Irritation, Multiple Dose Effects
A14178	Acute Toxicity, Reproductive Effects, Multiple Dose Effects
A14179	Acute Toxicity, Skin and Eye Irritation
A14180	Acute Toxicity, Skin and Eye Irritation
A14181	Non-toxicity
A14182	Multiple Dose Effects
A14183	Non-toxicity
A14184	Acute Toxicity, Reproductive Effects, Multiple Dose Effects
A14185	Non-toxicity
A14186	Acute Toxicity, Skin and Eye Irritation, Reproductive Effects, Multiple Dose
A14187	Acute Toxicity, Mutagenicity
A14188	Acute Toxicity, Mutagenicity, Skin and Eye Irritation, Multiple Dose Effects
A14189	Non-toxicity
A14190	Acute Toxicity, Mutagenicity, Tumorigenicity, Skin and Eye Irritation, Multiple Dose Effects
A14191	Tumorigenicity
A14192	Mutagenicity, Reproductive Effects, Multiple Dose Effects
A14193	Acute Toxicity, Mutagenicity, Tumorigenicity, Skin and Eye Irritation,
A14194	Non-toxicity
A14195	Mutagenicity, Tumorigenicity, Multiple Dose Effects
A14196	Acute Toxicity, Mutagenicity, Tumorigenicity, Skin and Eye Irritation,
A14197	Acute Toxicity, Multiple Dose Effects
A14198	Acute Toxicity, Mutagenicity, Tumorigenicity, Skin and Eye Irritation, Multiple Dose Effects
A14199	Acute Toxicity, Mutagenicity, Skin and Eye Irritation, Multiple Dose Effects
A14200	Acute Toxicity, Mutagenicity
A14201	Acute Toxicity
A14202	Acute Toxicity, Mutagenicity, Tumorigenicity, Reproductive Effects,
A14203	Multiple Dose Effects
A14204	Acute Toxicity, Mutagenicity, Tumorigenicity, Skin and Eye Irritation,
A14205	Multiple Dose Effects
A14206	Acute Toxicity
A14207	Tumorigenicity
A14208	Acute Toxicity, Mutagenicity, Tumorigenicity, Skin and Eye Irritation, Multiple Dose Effects
A14209	Acute Toxicity, Tumorigenicity, Reproductive Effects, Multiple Dose Effects
A14210	Acute Toxicity, Mutagenicity, Tumorigenicity, Skin and Eye Irritation,
A14211	Acute Toxicity, Mutagenicity, Tumorigenicity
A14212	Multiple Dose Effects
A14213	Multiple Dose Effects

A14214	Non-toxicity
A14215	Acute Toxicity
A14216	Acute Toxicity, Skin and Eye Irritation, Reproductive Effects, Multiple Dose
A14217	Non-toxicity
A14218	Acute Toxicity, Tumorigenicity, Multiple Dose Effects
A14219	Multiple Dose Effects
A14220	Acute Toxicity
A14221	Acute Toxicity, Mutagenicity, Tumorigenicity, Multiple Dose Effects
A14222	Acute Toxicity, Mutagenicity, Reproductive Effects, Multiple Dose Effects
A14223	Acute Toxicity, Skin and Eye Irritation
A14224	Acute Toxicity, Skin and Eye Irritation, Multiple Dose Effects
A14225	Non-toxicity
A14226	Mutagenicity
A14227	Acute Toxicity, Mutagenicity, Multiple Dose Effects
A14228	Acute Toxicity
A14229	Acute Toxicity, Mutagenicity, Tumorigenicity, Multiple Dose Effects
A14230	Non-toxicity
A14231	Non-toxicity
A14232	Acute Toxicity
A14233	Acute Toxicity
A14234	Acute Toxicity
A14235	Multiple Dose Effects
A14236	Acute Toxicity, Mutagenicity, Tumorigenicity, Skin and Eye Irritation, Multiple Dose Effects
A14237	Acute Toxicity, Multiple Dose Effects
A14238	Multiple Dose Effects
A14239	Acute Toxicity, Skin and Eye Irritation, Multiple Dose Effects
A14240	Acute Toxicity
A14241	Acute Toxicity
A14242	Non-toxicity
A14243	Acute Toxicity, Multiple Dose Effects
A14244	Multiple Dose Effects
A14245	Non-toxicity
A14246	Acute Toxicity, Mutagenicity, Reproductive Effects
A14247	Acute Toxicity, Mutagenicity, Tumorigenicity, Reproductive Effects,
A14248	Acute Toxicity, Tumorigenicity
A14249	Acute Toxicity, Multiple Dose Effects
A14250	Mutagenicity
A14251	Acute Toxicity
A14252	Non-toxicity
A14253	Acute Toxicity, Tumorigenicity, Multiple Dose Effects
A14254	Multiple Dose Effects
A14255	Acute Toxicity, Multiple Dose Effects
A14256	Acute Toxicity, Multiple Dose Effects
A14257	Multiple Dose Effects
A14258	Tumorigenicity

A14259	Non-toxicity
A14260	Multiple Dose Effects
A14261	Acute Toxicity, Multiple Dose Effects
A14262	Mutagenicity
A14263	Acute Toxicity, Mutagenicity, Tumorigenicity, Reproductive Effects,
A14264	Acute Toxicity, Reproductive Effects, Multiple Dose Effects
A14265	Mutagenicity, Tumorigenicity, Skin and Eye Irritation
A14266	Acute Toxicity, Mutagenicity, Reproductive Effects, Multiple Dose Effects
A14267	Acute Toxicity
A14268	Non-toxicity
A14269	Non-toxicity
A14270	Acute Toxicity, Multiple Dose Effects
A14271	Acute Toxicity, Mutagenicity, Multiple Dose Effects
A14272	Acute Toxicity, Mutagenicity, Skin and Eye Irritation, Reproductive Effects, Multiple Dose Effects
A14273	Skin and Eye Irritation, Reproductive Effects, Multiple Dose Effects
A14274	Acute Toxicity, Multiple Dose Effects
A14275	Non-toxicity
A14276	Non-toxicity
A14277	Acute Toxicity, Tumorigenicity
A14278	Acute Toxicity, Mutagenicity, Reproductive Effects, Multiple Dose Effects
A14279	Mutagenicity
A14280	Multiple Dose Effects
A14281	Acute Toxicity, Mutagenicity, Tumorigenicity, Skin and Eye Irritation,
A14282	Acute Toxicity
A14283	Acute Toxicity, Tumorigenicity
A14284	Acute Toxicity
A14285	Multiple Dose Effects
A14286	Reproductive Effects, Multiple Dose Effects
A14287	Acute Toxicity
A14288	Multiple Dose Effects
A14289	Acute Toxicity, Mutagenicity, Multiple Dose Effects
A14290	Acute Toxicity, Multiple Dose Effects
A14291	Mutagenicity
A14292	Non-toxicity
A14293	Acute Toxicity
A14294	Mutagenicity
A14295	Acute Toxicity, Mutagenicity
A14296	Multiple Dose Effects
A14297	Acute Toxicity
A14298	Multiple Dose Effects
A14299	Acute Toxicity, Mutagenicity, Tumorigenicity, Skin and Eye Irritation, Multiple Dose Effects
A14300	Mutagenicity, Reproductive Effects
A14301	Mutagenicity
A14302	Mutagenicity

A14303	Skin and Eye Irritation
A14304	Reproductive Effects, Multiple Dose Effects
A14305	Reproductive Effects
A14306	Mutagenicity
A14307	Mutagenicity
A14308	Mutagenicity
A14309	Mutagenicity
A14310	Acute Toxicity, Mutagenicity, Reproductive Effects
A14311	Acute Toxicity, Skin and Eye Irritation, Multiple Dose Effects
A14312	Acute Toxicity
A14313	Acute Toxicity, Mutagenicity, Multiple Dose Effects
A14314	Non-toxicity
A14315	Acute Toxicity, Mutagenicity
A14316	Acute Toxicity
A14317	Acute Toxicity
A14318	Acute Toxicity
A14319	Mutagenicity
A14320	Skin and Eye Irritation
A14321	Mutagenicity
A14322	Mutagenicity
A14323	Mutagenicity
A14324	Acute Toxicity, Multiple Dose Effects
A14325	Acute Toxicity
A14326	Acute Toxicity
A14327	Non-toxicity
A14328	Acute Toxicity
A14329	Mutagenicity
A14330	Acute Toxicity, Mutagenicity
A14331	Multiple Dose Effects
A14332	Mutagenicity, Reproductive Effects, Multiple Dose Effects
A14333	Acute Toxicity
A14334	Non-toxicity
A14335	Mutagenicity
A14336	Mutagenicity
A14337	Acute Toxicity, Mutagenicity, Multiple Dose Effects
A14338	Acute Toxicity, Mutagenicity, Tumorigenicity, Multiple Dose Effects
A14339	Mutagenicity
A14340	Acute Toxicity
A14341	Acute Toxicity
A14342	Mutagenicity
A14343	Acute Toxicity
A14344	Acute Toxicity, Mutagenicity, Tumorigenicity, Reproductive Effects,
A14345	Acute Toxicity
A14346	Acute Toxicity
A14347	Multiple Dose Effects
A14348	Acute Toxicity

A14349	Mutagenicity
A14350	Acute Toxicity
A14351	Acute Toxicity
A14352	Acute Toxicity, Reproductive Effects, Multiple Dose Effects
A14353	Tumorigenicity
A14354	Mutagenicity
A14355	Acute Toxicity
A14356	Acute Toxicity, Mutagenicity, Skin and Eye Irritation, Reproductive Effects, Multiple Dose Effects
A14357	Acute Toxicity, Mutagenicity
A14358	Acute Toxicity, Skin and Eye Irritation, Reproductive Effects, Multiple Dose
A14359	Acute Toxicity
A14360	Acute Toxicity
A14361	Acute Toxicity
A14362	Acute Toxicity, Mutagenicity, Tumorigenicity, Reproductive Effects,
A14363	Acute Toxicity
A14364	Acute Toxicity, Mutagenicity, Skin and Eye Irritation
A14365	Acute Toxicity
A14366	Acute Toxicity, Mutagenicity, Tumorigenicity, Skin and Eye Irritation,
A14367	Acute Toxicity
A14368	Acute Toxicity
A14369	Acute Toxicity, Tumorigenicity
A14370	Non-toxicity
A14371	Acute Toxicity, Skin and Eye Irritation, Reproductive Effects, Multiple Dose
A14372	Acute Toxicity, Mutagenicity, Tumorigenicity, Skin and Eye Irritation,
A14373	Acute Toxicity, Mutagenicity, Tumorigenicity, Multiple Dose Effects
A14374	Acute Toxicity, Mutagenicity, Tumorigenicity, Skin and Eye Irritation,
A14375	Acute Toxicity, Skin and Eye Irritation, Multiple Dose Effects
A14376	Acute Toxicity, Multiple Dose Effects
A14377	Acute Toxicity, Mutagenicity, Skin and Eye Irritation
A14378	Acute Toxicity, Mutagenicity, Tumorigenicity, Multiple Dose Effects
A14379	Non-toxicity
A14380	Acute Toxicity, Skin and Eye Irritation, Multiple Dose Effects
A14381	Acute Toxicity, Mutagenicity, Tumorigenicity, Skin and Eye Irritation, Multiple Dose Effects
A14382	Acute Toxicity, Mutagenicity
A14383	Acute Toxicity
A14384	Acute Toxicity, Mutagenicity, Multiple Dose Effects
A14385	Acute Toxicity
A14386	Acute Toxicity, Multiple Dose Effects
A14387	Acute Toxicity, Mutagenicity
A14388	Mutagenicity
A14389	Acute Toxicity, Mutagenicity
A14390	Acute Toxicity, Mutagenicity
A14391	Acute Toxicity, Mutagenicity, Tumorigenicity, Skin and Eye Irritation,
A14392	Acute Toxicity, Mutagenicity, Tumorigenicity, Reproductive Effects,

A14393	Mutagenicity
A14394	Acute Toxicity, Mutagenicity, Tumorigenicity, Reproductive Effects,
A14395	Acute Toxicity, Mutagenicity, Tumorigenicity, Skin and Eye Irritation,
A14396	Mutagenicity
A14397	Acute Toxicity, Tumorigenicity, Skin and Eye Irritation, Multiple Dose
A14398	Acute Toxicity, Skin and Eye Irritation, Reproductive Effects, Multiple Dose
A14399	Acute Toxicity, Mutagenicity, Reproductive Effects, Multiple Dose Effects
A14400	Acute Toxicity
A14401	Acute Toxicity, Mutagenicity
A14402	Acute Toxicity
A14403	Acute Toxicity, Skin and Eye Irritation
A14404	Acute Toxicity, Mutagenicity, Skin and Eye Irritation, Multiple Dose Effects
A14405	Acute Toxicity, Mutagenicity, Skin and Eye Irritation, Reproductive Effects, Multiple Dose Effects
A14406	Acute Toxicity, Tumorigenicity
A14407	Acute Toxicity, Multiple Dose Effects
A14408	Acute Toxicity, Mutagenicity, Tumorigenicity, Multiple Dose Effects
A14409	Acute Toxicity
A14410	Acute Toxicity, Skin and Eye Irritation
A14411	Acute Toxicity, Mutagenicity, Tumorigenicity
A14412	Mutagenicity, Multiple Dose Effects
A14413	Multiple Dose Effects
A14414	Acute Toxicity, Mutagenicity, Tumorigenicity
A14415	Acute Toxicity, Skin and Eye Irritation
A14416	Acute Toxicity, Skin and Eye Irritation
A14417	Skin and Eye Irritation
A14418	Acute Toxicity, Mutagenicity, Skin and Eye Irritation
A14419	Acute Toxicity, Skin and Eye Irritation
A14420	Non-toxicity
A14421	Mutagenicity
A14422	Mutagenicity, Skin and Eye Irritation
A14423	Acute Toxicity, Mutagenicity, Tumorigenicity, Reproductive Effects,
A14424	Acute Toxicity, Skin and Eye Irritation
A14425	Acute Toxicity
A14426	Acute Toxicity
A14427	Skin and Eye Irritation
A14428	Acute Toxicity
A14429	Acute Toxicity, Mutagenicity
A14430	Acute Toxicity, Mutagenicity, Tumorigenicity, Skin and Eye Irritation
A14431	Tumorigenicity, Skin and Eye Irritation
A14432	Acute Toxicity, Mutagenicity, Skin and Eye Irritation
A14433	Non-toxicity
A14434	Acute Toxicity, Multiple Dose Effects
A14435	Mutagenicity
A14436	Acute Toxicity
A14437	Acute Toxicity, Mutagenicity

A14438	Acute Toxicity
A14439	Acute Toxicity, Skin and Eye Irritation
A14440	Acute Toxicity, Mutagenicity
A14441	Acute Toxicity, Multiple Dose Effects
A14442	Acute Toxicity
A14443	Acute Toxicity
A14444	Acute Toxicity, Mutagenicity
A14445	Acute Toxicity, Mutagenicity, Reproductive Effects
A14446	Acute Toxicity, Multiple Dose Effects
A14447	Acute Toxicity, Multiple Dose Effects
A14448	Acute Toxicity, Mutagenicity, Reproductive Effects, Multiple Dose Effects
A14449	Acute Toxicity
A14450	Reproductive Effects
A14451	Acute Toxicity
A14452	Acute Toxicity, Mutagenicity, Tumorigenicity
A14453	Reproductive Effects
A14454	Acute Toxicity
A14455	Multiple Dose Effects
A14456	Non-toxicity
A14457	Acute Toxicity
A14458	Acute Toxicity
A14459	Acute Toxicity, Mutagenicity
A14460	Multiple Dose Effects
A14461	Acute Toxicity, Reproductive Effects, Multiple Dose Effects
A14462	Acute Toxicity
A14463	Acute Toxicity, Mutagenicity
A14464	Acute Toxicity, Mutagenicity, Tumorigenicity, Reproductive Effects,
A14465	Non-toxicity
A14466	Acute Toxicity
A14467	Acute Toxicity
A14468	Acute Toxicity
A14469	Skin and Eye Irritation, Reproductive Effects
A14470	Acute Toxicity, Reproductive Effects
A14471	Mutagenicity
A14472	Non-toxicity
A14473	Mutagenicity, Tumorigenicity, Reproductive Effects
A14474	Acute Toxicity, Mutagenicity, Multiple Dose Effects
A14475	Acute Toxicity, Mutagenicity, Tumorigenicity, Reproductive Effects,
A14476	Acute Toxicity
A14477	Acute Toxicity, Multiple Dose Effects
A14478	Non-toxicity
A14479	Acute Toxicity, Mutagenicity, Reproductive Effects, Multiple Dose Effects
A14480	Mutagenicity
A14481	Mutagenicity, Tumorigenicity, Reproductive Effects, Multiple Dose Effects
A14482	Acute Toxicity, Skin and Eye Irritation, Multiple Dose Effects
A14483	Acute Toxicity

A14484	Non-toxicity
A14485	Acute Toxicity, Mutagenicity, Tumorigenicity, Reproductive Effects,
A14486	Acute Toxicity
A14487	Acute Toxicity
A14488	Acute Toxicity
A14489	Acute Toxicity, Skin and Eye Irritation, Multiple Dose Effects
A14490	Acute Toxicity, Multiple Dose Effects
A14491	Acute Toxicity
A14492	Acute Toxicity
A14493	Mutagenicity
A14494	Non-toxicity
A14495	Acute Toxicity
A14496	Acute Toxicity, Mutagenicity, Tumorigenicity, Reproductive Effects
A14497	Acute Toxicity
A14498	Acute Toxicity, Mutagenicity, Tumorigenicity
A14499	Acute Toxicity
A14500	Acute Toxicity, Mutagenicity
A14501	Acute Toxicity
A14502	Acute Toxicity, Skin and Eye Irritation
A14503	Acute Toxicity
A14504	Reproductive Effects
A14505	Acute Toxicity, Multiple Dose Effects
A14506	Non-toxicity
A14507	Acute Toxicity
A14508	Acute Toxicity
A14509	Acute Toxicity, Mutagenicity
A14510	Acute Toxicity
A14511	Acute Toxicity, Mutagenicity, Tumorigenicity, Reproductive Effects,
A14512	Acute Toxicity
A14513	Acute Toxicity
A14514	Acute Toxicity
A14515	Acute Toxicity, Mutagenicity
A14516	Acute Toxicity
A14517	Mutagenicity, Tumorigenicity
A14518	Acute Toxicity
A14519	Acute Toxicity, Reproductive Effects, Multiple Dose Effects
A14520	Acute Toxicity
A14521	Acute Toxicity, Mutagenicity, Skin and Eye Irritation, Reproductive Effects
A14522	Acute Toxicity
A14523	Mutagenicity
A14524	Acute Toxicity, Mutagenicity, Skin and Eye Irritation, Reproductive Effects, Multiple Dose Effects
A14525	Acute Toxicity
A14526	Non-toxicity
A14527	Mutagenicity
A14528	Acute Toxicity, Mutagenicity

A14529	Mutagenicity
A14530	Acute Toxicity
A14531	Acute Toxicity, Mutagenicity
A14532	Multiple Dose Effects
A14533	Mutagenicity, Tumorigenicity
A14534	Mutagenicity
A14535	Acute Toxicity
A14536	Acute Toxicity
A14537	Acute Toxicity, Mutagenicity
A14538	Acute Toxicity
A14539	Acute Toxicity
A14540	Acute Toxicity, Mutagenicity, Tumorigenicity
A14541	Acute Toxicity
A14542	Acute Toxicity
A14543	Acute Toxicity
A14544	Acute Toxicity, Mutagenicity
A14545	Mutagenicity
A14546	Multiple Dose Effects
A14547	Acute Toxicity
A14548	Mutagenicity, Tumorigenicity
A14549	Acute Toxicity, Multiple Dose Effects
A14550	Acute Toxicity
A14551	Acute Toxicity
A14552	Acute Toxicity
A14553	Non-toxicity
A14554	Acute Toxicity, Mutagenicity, Skin and Eye Irritation, Reproductive Effects, Multiple Dose Effects
A14555	Acute Toxicity
A14556	Acute Toxicity
A14557	Acute Toxicity, Mutagenicity
A14558	Acute Toxicity
A14559	Acute Toxicity, Multiple Dose Effects
A14560	Acute Toxicity, Mutagenicity
A14561	Acute Toxicity
A14562	Tumorigenicity
A14563	Acute Toxicity
A14564	Acute Toxicity
A14565	Acute Toxicity
A14566	Acute Toxicity, Multiple Dose Effects
A14567	Acute Toxicity
A14568	Acute Toxicity, Skin and Eye Irritation, Reproductive Effects, Multiple Dose
A14569	Acute Toxicity, Multiple Dose Effects
A14570	Acute Toxicity, Mutagenicity, Tumorigenicity, Skin and Eye Irritation,
A14571	Acute Toxicity
A14572	Acute Toxicity
A14573	Acute Toxicity

A14574	Acute Toxicity
A14575	Acute Toxicity
A14576	Acute Toxicity
A14577	Non-toxicity
A14578	Acute Toxicity
A14579	Acute Toxicity, Mutagenicity, Skin and Eye Irritation
A14580	Acute Toxicity
A14581	Non-toxicity
A14582	Acute Toxicity, Multiple Dose Effects
A14583	Acute Toxicity
A14584	Acute Toxicity
A14585	Acute Toxicity, Mutagenicity, Tumorigenicity, Reproductive Effects,
A14586	Mutagenicity, Tumorigenicity
A14587	Acute Toxicity
A14588	Acute Toxicity
A14589	Acute Toxicity, Mutagenicity, Tumorigenicity, Reproductive Effects,
A14590	Mutagenicity
A14591	Acute Toxicity, Multiple Dose Effects
A14592	Acute Toxicity
A14593	Acute Toxicity
A14594	Acute Toxicity, Reproductive Effects, Multiple Dose Effects
A14595	Acute Toxicity
A14596	Acute Toxicity, Mutagenicity, Tumorigenicity, Skin and Eye Irritation, Multiple Dose Effects
A14597	Acute Toxicity, Mutagenicity
A14598	Acute Toxicity, Skin and Eye Irritation
A14599	Acute Toxicity, Mutagenicity, Tumorigenicity, Skin and Eye Irritation, Multiple Dose Effects
A14600	Acute Toxicity, Skin and Eye Irritation, Multiple Dose Effects
A14601	Acute Toxicity
A14602	Acute Toxicity
A14603	Acute Toxicity
A14604	Non-toxicity
A14605	Acute Toxicity, Mutagenicity
A14606	Acute Toxicity
A14607	Acute Toxicity
A14608	Acute Toxicity, Skin and Eye Irritation
A14609	Acute Toxicity
A14610	Acute Toxicity
A14611	Acute Toxicity, Tumorigenicity, Multiple Dose Effects
A14612	Tumorigenicity
A14613	Acute Toxicity, Skin and Eye Irritation
A14614	Acute Toxicity
A14615	Acute Toxicity, Skin and Eye Irritation, Multiple Dose Effects
A14616	Acute Toxicity, Mutagenicity, Skin and Eye Irritation
A14617	Acute Toxicity

A14618	Acute Toxicity
A14619	Acute Toxicity
A14620	Acute Toxicity
A14621	Acute Toxicity, Skin and Eye Irritation, Multiple Dose Effects
A14622	Non-toxicity
A14623	Acute Toxicity
A14624	Skin and Eye Irritation
A14625	Non-toxicity
A14626	Acute Toxicity, Reproductive Effects
A14627	Non-toxicity
A14628	Acute Toxicity, Mutagenicity, Multiple Dose Effects
A14629	Acute Toxicity
A14630	Acute Toxicity
A14631	Acute Toxicity
A14632	Acute Toxicity
A14633	Non-toxicity
A14634	Acute Toxicity, Skin and Eye Irritation, Reproductive Effects
A14635	Acute Toxicity
A14636	Acute Toxicity, Reproductive Effects, Multiple Dose Effects
A14637	Acute Toxicity, Mutagenicity, Tumorigenicity, Skin and Eye Irritation,
A14638	Acute Toxicity
A14639	Acute Toxicity, Reproductive Effects
A14640	Acute Toxicity, Mutagenicity
A14641	Acute Toxicity
A14642	Acute Toxicity, Mutagenicity, Tumorigenicity
A14643	Acute Toxicity, Mutagenicity, Tumorigenicity, Skin and Eye Irritation,
A14644	Acute Toxicity, Mutagenicity
A14645	Acute Toxicity
A14646	Acute Toxicity
A14647	Mutagenicity
A14648	Acute Toxicity, Mutagenicity, Tumorigenicity, Reproductive Effects,
A14649	Mutagenicity
A14650	Acute Toxicity, Mutagenicity, Skin and Eye Irritation
A14651	Mutagenicity
A14652	Acute Toxicity, Mutagenicity, Skin and Eye Irritation
A14653	Acute Toxicity, Reproductive Effects, Multiple Dose Effects
A14654	Acute Toxicity, Mutagenicity, Tumorigenicity
A14655	Acute Toxicity, Mutagenicity, Reproductive Effects, Multiple Dose Effects
A14656	Acute Toxicity, Mutagenicity, Tumorigenicity
A14657	Tumorigenicity
A14658	Acute Toxicity
A14659	Acute Toxicity, Multiple Dose Effects
A14660	Acute Toxicity
A14661	Acute Toxicity, Mutagenicity, Tumorigenicity, Reproductive Effects,
A14662	Acute Toxicity, Mutagenicity
A14663	Reproductive Effects

A14664	Reproductive Effects, Multiple Dose Effects
A14665	Acute Toxicity, Mutagenicity, Multiple Dose Effects
A14666	Acute Toxicity, Mutagenicity, Tumorigenicity, Skin and Eye Irritation, Multiple Dose Effects
A14667	Acute Toxicity
A14668	Mutagenicity
A14669	Acute Toxicity
A14670	Acute Toxicity
A14671	Acute Toxicity
A14672	Acute Toxicity, Multiple Dose Effects
A14673	Acute Toxicity
A14674	Acute Toxicity, Mutagenicity, Tumorigenicity, Reproductive Effects,
A14675	Mutagenicity
A14676	Mutagenicity
A14677	Mutagenicity
A14678	Mutagenicity
A14679	Acute Toxicity
A14680	Acute Toxicity
A14681	Acute Toxicity
A14682	Acute Toxicity
A14683	Acute Toxicity, Tumorigenicity
A14684	Acute Toxicity
A14685	Acute Toxicity, Mutagenicity, Tumorigenicity, Multiple Dose Effects
A14686	Non-toxicity
A14687	Acute Toxicity, Mutagenicity
A14688	Acute Toxicity
A14689	Acute Toxicity, Mutagenicity, Tumorigenicity, Multiple Dose Effects
A14690	Tumorigenicity
A14691	Acute Toxicity, Multiple Dose Effects
A14692	Mutagenicity
A14693	Acute Toxicity, Mutagenicity, Reproductive Effects, Multiple Dose Effects
A14694	Acute Toxicity, Mutagenicity, Reproductive Effects, Multiple Dose Effects
A14695	Mutagenicity
A14696	Acute Toxicity, Reproductive Effects, Multiple Dose Effects
A14697	Acute Toxicity
A14698	Acute Toxicity
A14699	Acute Toxicity, Mutagenicity, Reproductive Effects
A14700	Acute Toxicity
A14701	Acute Toxicity, Skin and Eye Irritation, Multiple Dose Effects
A14702	Acute Toxicity
A14703	Acute Toxicity
A14704	Acute Toxicity
A14705	Acute Toxicity
A14706	Multiple Dose Effects
A14707	Acute Toxicity, Skin and Eye Irritation, Reproductive Effects, Multiple Dose
A14708	Non-toxicity

A14709	Acute Toxicity, Mutagenicity, Reproductive Effects, Multiple Dose Effects
A14710	Mutagenicity, Reproductive Effects, Multiple Dose Effects
A14711	Acute Toxicity, Mutagenicity, Tumorigenicity, Multiple Dose Effects
A14712	Mutagenicity
A14713	Mutagenicity
A14714	Mutagenicity
A14715	Mutagenicity
A14716	Acute Toxicity
A14717	Multiple Dose Effects
A14718	Non-toxicity
A14719	Multiple Dose Effects
A14720	Acute Toxicity, Multiple Dose Effects
A14721	Multiple Dose Effects
A14722	Mutagenicity
A14723	Acute Toxicity
A14724	Mutagenicity
A14725	Acute Toxicity
A14726	Mutagenicity
A14727	Skin and Eye Irritation
A14728	Acute Toxicity
A14729	Acute Toxicity
A14730	Acute Toxicity
A14731	Acute Toxicity
A14732	Acute Toxicity
A14733	Mutagenicity
A14734	Tumorigenicity
A14735	Multiple Dose Effects
A14736	Tumorigenicity
A14737	Acute Toxicity
A14738	Acute Toxicity, Multiple Dose Effects
A14739	Mutagenicity
A14740	Mutagenicity
A14741	Acute Toxicity
A14742	Acute Toxicity
A14743	Acute Toxicity
A14744	Acute Toxicity, Multiple Dose Effects
A14745	Acute Toxicity
A14746	Mutagenicity, Tumorigenicity
A14747	Acute Toxicity
A14748	Acute Toxicity
A14749	Acute Toxicity
A14750	Acute Toxicity
A14751	Acute Toxicity
A14752	Acute Toxicity
A14753	Acute Toxicity
A14754	Acute Toxicity

A14755	Acute Toxicity
A14756	Acute Toxicity
A14757	Acute Toxicity
A14758	Acute Toxicity
A14759	Acute Toxicity
A14760	Acute Toxicity
A14761	Acute Toxicity
A14762	Acute Toxicity
A14763	Acute Toxicity
A14764	Acute Toxicity
A14765	Acute Toxicity
A14766	Acute Toxicity
A14767	Tumorigenicity
A14768	Acute Toxicity
A14769	Acute Toxicity
A14770	Acute Toxicity
A14771	Acute Toxicity
A14772	Acute Toxicity
A14773	Acute Toxicity
A14774	Acute Toxicity
A14775	Acute Toxicity
A14776	Acute Toxicity, Skin and Eye Irritation, Multiple Dose Effects
A14777	Acute Toxicity, Multiple Dose Effects
A14778	Non-toxicity
A14779	Acute Toxicity
A14780	Acute Toxicity
A14781	Acute Toxicity, Reproductive Effects, Multiple Dose Effects
A14782	Acute Toxicity, Reproductive Effects
A14783	Acute Toxicity, Mutagenicity, Reproductive Effects
A14784	Acute Toxicity
A14785	Acute Toxicity, Mutagenicity, Reproductive Effects
A14786	Mutagenicity
A14787	Acute Toxicity, Multiple Dose Effects
A14788	Mutagenicity, Tumorigenicity, Multiple Dose Effects
A14789	Mutagenicity
A14790	Acute Toxicity
A14791	Acute Toxicity
A14792	Reproductive Effects
A14793	Acute Toxicity, Mutagenicity, Tumorigenicity, Reproductive Effects
A14794	Acute Toxicity, Multiple Dose Effects
A14795	Acute Toxicity
A14796	Acute Toxicity
A14797	Acute Toxicity
A14798	Tumorigenicity
A14799	Acute Toxicity, Mutagenicity, Reproductive Effects, Multiple Dose Effects
A14800	Acute Toxicity, Reproductive Effects

A14801	Acute Toxicity
A14802	Reproductive Effects
A14803	Acute Toxicity, Mutagenicity, Reproductive Effects, Multiple Dose Effects
A14804	Acute Toxicity, Mutagenicity, Reproductive Effects, Multiple Dose Effects
A14805	Acute Toxicity
A14806	Skin and Eye Irritation
A14807	Acute Toxicity, Mutagenicity, Reproductive Effects
A14808	Acute Toxicity
A14809	Acute Toxicity
A14810	Mutagenicity
A14811	Acute Toxicity, Skin and Eye Irritation
A14812	Mutagenicity
A14813	Acute Toxicity, Reproductive Effects, Multiple Dose Effects
A14814	Acute Toxicity
A14815	Acute Toxicity
A14816	Acute Toxicity
A14817	Acute Toxicity
A14818	Acute Toxicity
A14819	Acute Toxicity, Reproductive Effects, Multiple Dose Effects
A14820	Acute Toxicity
A14821	Acute Toxicity
A14822	Acute Toxicity
A14823	Acute Toxicity
A14824	Mutagenicity
A14825	Acute Toxicity
A14826	Acute Toxicity
A14827	Acute Toxicity, Mutagenicity, Tumorigenicity, Reproductive Effects,
A14828	Acute Toxicity, Tumorigenicity, Multiple Dose Effects
A14829	Acute Toxicity, Skin and Eye Irritation, Multiple Dose Effects
A14830	Acute Toxicity
A14831	Acute Toxicity
A14832	Mutagenicity
A14833	Acute Toxicity
A14834	Acute Toxicity, Mutagenicity
A14835	Reproductive Effects
A14836	Acute Toxicity
A14837	Acute Toxicity, Multiple Dose Effects
A14838	Mutagenicity, Reproductive Effects, Multiple Dose Effects
A14839	Mutagenicity
A14840	Skin and Eye Irritation
A14841	Acute Toxicity
A14842	Acute Toxicity
A14843	Acute Toxicity, Multiple Dose Effects
A14844	Mutagenicity, Reproductive Effects, Multiple Dose Effects
A14845	Acute Toxicity
A14846	Acute Toxicity

A14847	Acute Toxicity, Mutagenicity
A14848	Acute Toxicity
A14849	Acute Toxicity
A14850	Acute Toxicity
A14851	Acute Toxicity
A14852	Acute Toxicity
A14853	Acute Toxicity
A14854	Mutagenicity
A14855	Mutagenicity
A14856	Acute Toxicity, Multiple Dose Effects
A14857	Acute Toxicity, Skin and Eye Irritation, Reproductive Effects, Multiple Dose
A14858	Acute Toxicity
A14859	Acute Toxicity
A14860	Acute Toxicity, Skin and Eye Irritation
A14861	Acute Toxicity, Reproductive Effects, Multiple Dose Effects
A14862	Acute Toxicity, Multiple Dose Effects
A14863	Acute Toxicity
A14864	Mutagenicity
A14865	Acute Toxicity
A14866	Acute Toxicity
A14867	Acute Toxicity, Multiple Dose Effects
A14868	Reproductive Effects
A14869	Acute Toxicity
A14870	Acute Toxicity
A14871	Acute Toxicity
A14872	Acute Toxicity
A14873	Mutagenicity
A14874	Acute Toxicity
A14875	Mutagenicity, Skin and Eye Irritation
A14876	Mutagenicity, Tumorigenicity
A14877	Acute Toxicity, Mutagenicity, Multiple Dose Effects
A14878	Acute Toxicity, Multiple Dose Effects
A14879	Skin and Eye Irritation
A14880	Skin and Eye Irritation
A14881	Acute Toxicity, Skin and Eye Irritation, Multiple Dose Effects
A14882	Acute Toxicity
A14883	Mutagenicity
A14884	Mutagenicity, Tumorigenicity
A14885	Mutagenicity
A14886	Mutagenicity
A14887	Mutagenicity
A14888	Acute Toxicity, Mutagenicity
A14889	Acute Toxicity
A14890	Acute Toxicity
A14891	Acute Toxicity
A14892	Mutagenicity

A14893	Tumorigenicity
A14894	Acute Toxicity
A14895	Acute Toxicity, Multiple Dose Effects
A14896	Acute Toxicity
A14897	Acute Toxicity
A14898	Acute Toxicity
A14899	Acute Toxicity
A14900	Acute Toxicity
A14901	Mutagenicity, Tumorigenicity
A14902	Acute Toxicity
A14903	Acute Toxicity, Multiple Dose Effects
A14904	Acute Toxicity
A14905	Acute Toxicity
A14906	Acute Toxicity
A14907	Acute Toxicity, Mutagenicity
A14908	Acute Toxicity
A14909	Acute Toxicity, Mutagenicity
A14910	Mutagenicity
A14911	Acute Toxicity
A14912	Acute Toxicity
A14913	Mutagenicity, Tumorigenicity
A14914	Reproductive Effects
A14915	Acute Toxicity, Reproductive Effects
A14916	Mutagenicity
A14917	Tumorigenicity
A14918	Mutagenicity
A14919	Acute Toxicity
A14920	Skin and Eye Irritation
A14921	Acute Toxicity
A14922	Acute Toxicity
A14923	Acute Toxicity, Reproductive Effects, Multiple Dose Effects
A14924	Mutagenicity
A14925	Acute Toxicity
A14926	Mutagenicity
A14927	Acute Toxicity
A14928	Acute Toxicity
A14929	Reproductive Effects, Multiple Dose Effects
A14930	Acute Toxicity
A14931	Acute Toxicity
A14932	Acute Toxicity
A14933	Reproductive Effects
A14934	Acute Toxicity
A14935	Acute Toxicity
A14936	Tumorigenicity
A14937	Multiple Dose Effects
A14938	Acute Toxicity

A14939	Acute Toxicity
A14940	Acute Toxicity
A14941	Acute Toxicity
A14942	Acute Toxicity
A14943	Acute Toxicity
A14944	Acute Toxicity
A14945	Acute Toxicity
A14946	Acute Toxicity
A14947	Acute Toxicity
A14948	Acute Toxicity
A14949	Acute Toxicity
A14950	Acute Toxicity
A14951	Acute Toxicity
A14952	Acute Toxicity, Tumorigenicity
A14953	Acute Toxicity
A14954	Acute Toxicity
A14955	Acute Toxicity
A14956	Acute Toxicity
A14957	Acute Toxicity
A14958	Acute Toxicity
A14959	Acute Toxicity
A14960	Acute Toxicity
A14961	Acute Toxicity
A14962	Acute Toxicity
A14963	Acute Toxicity
A14964	Acute Toxicity
A14965	Acute Toxicity
A14966	Acute Toxicity
A14967	Acute Toxicity
A14968	Acute Toxicity
A14969	Acute Toxicity
A14970	Acute Toxicity
A14971	Acute Toxicity
A14972	Acute Toxicity
A14973	Acute Toxicity
A14974	Acute Toxicity
A14975	Acute Toxicity
A14976	Acute Toxicity
A14977	Acute Toxicity
A14978	Acute Toxicity
A14979	Acute Toxicity
A14980	Acute Toxicity
A14981	Acute Toxicity
A14982	Acute Toxicity
A14983	Acute Toxicity
A14984	Acute Toxicity

A14985	Acute Toxicity, Mutagenicity
A14986	Acute Toxicity
A14987	Acute Toxicity
A14988	Acute Toxicity
A14989	Acute Toxicity
A14990	Acute Toxicity
A14991	Acute Toxicity
A14992	Acute Toxicity
A14993	Acute Toxicity
A14994	Mutagenicity
A14995	Non-toxicity
A14996	Acute Toxicity
A14997	Acute Toxicity
A14998	Acute Toxicity
A14999	Acute Toxicity
A15000	Acute Toxicity
A15001	Acute Toxicity
A15002	Acute Toxicity
A15003	Acute Toxicity
A15004	Acute Toxicity
A15005	Acute Toxicity
A15006	Acute Toxicity
A15007	Acute Toxicity
A15008	Acute Toxicity
A15009	Acute Toxicity
A15010	Acute Toxicity
A15011	Acute Toxicity
A15012	Acute Toxicity
A15013	Acute Toxicity
A15014	Acute Toxicity
A15015	Acute Toxicity
A15016	Acute Toxicity
A15017	Acute Toxicity
A15018	Mutagenicity, Tumorigenicity
A15019	Acute Toxicity
A15020	Acute Toxicity
A15021	Acute Toxicity
A15022	Acute Toxicity
A15023	Acute Toxicity, Multiple Dose Effects
A15024	Acute Toxicity
A15025	Acute Toxicity
A15026	Acute Toxicity
A15027	Acute Toxicity
A15028	Acute Toxicity
A15029	Acute Toxicity
A15030	Acute Toxicity

A15031	Acute Toxicity
A15032	Acute Toxicity
A15033	Acute Toxicity
A15034	Acute Toxicity
A15035	Acute Toxicity, Mutagenicity
A15036	Acute Toxicity
A15037	Acute Toxicity
A15038	Acute Toxicity
A15039	Acute Toxicity
A15040	Acute Toxicity
A15041	Acute Toxicity
A15042	Acute Toxicity
A15043	Acute Toxicity
A15044	Acute Toxicity
A15045	Acute Toxicity
A15046	Acute Toxicity
A15047	Acute Toxicity
A15048	Acute Toxicity
A15049	Acute Toxicity
A15050	Acute Toxicity
A15051	Acute Toxicity
A15052	Acute Toxicity
A15053	Acute Toxicity
A15054	Acute Toxicity
A15055	Acute Toxicity
A15056	Tumorigenicity
A15057	Acute Toxicity
A15058	Acute Toxicity
A15059	Acute Toxicity
A15060	Acute Toxicity
A15061	Reproductive Effects
A15062	Acute Toxicity
A15063	Acute Toxicity
A15064	Acute Toxicity
A15065	Acute Toxicity
A15066	Acute Toxicity
A15067	Acute Toxicity
A15068	Acute Toxicity
A15069	Acute Toxicity
A15070	Acute Toxicity
A15071	Acute Toxicity
A15072	Acute Toxicity
A15073	Acute Toxicity
A15074	Acute Toxicity
A15075	Acute Toxicity
A15076	Acute Toxicity

A15077	Acute Toxicity
A15078	Acute Toxicity
A15079	Acute Toxicity
A15080	Acute Toxicity
A15081	Acute Toxicity
A15082	Acute Toxicity
A15083	Acute Toxicity
A15084	Acute Toxicity
A15085	Acute Toxicity
A15086	Acute Toxicity
A15087	Acute Toxicity
A15088	Acute Toxicity
A15089	Acute Toxicity
A15090	Acute Toxicity
A15091	Acute Toxicity
A15092	Acute Toxicity
A15093	Acute Toxicity
A15094	Acute Toxicity
A15095	Acute Toxicity
A15096	Acute Toxicity
A15097	Acute Toxicity
A15098	Acute Toxicity
A15099	Acute Toxicity
A15100	Acute Toxicity
A15101	Acute Toxicity
A15102	Acute Toxicity
A15103	Acute Toxicity
A15104	Mutagenicity
A15105	Multiple Dose Effects
A15106	Acute Toxicity
A15107	Acute Toxicity
A15108	Mutagenicity
A15109	Acute Toxicity
A15110	Acute Toxicity
A15111	Acute Toxicity
A15112	Acute Toxicity
A15113	Acute Toxicity
A15114	Acute Toxicity
A15115	Acute Toxicity
A15116	Acute Toxicity
A15117	Acute Toxicity
A15118	Acute Toxicity
A15119	Acute Toxicity
A15120	Acute Toxicity
A15121	Acute Toxicity, Multiple Dose Effects
A15122	Acute Toxicity

A15123	Acute Toxicity
A15124	Mutagenicity
A15125	Acute Toxicity
A15126	Acute Toxicity
A15127	Acute Toxicity
A15128	Acute Toxicity
A15129	Acute Toxicity
A15130	Acute Toxicity
A15131	Acute Toxicity
A15132	Acute Toxicity
A15133	Acute Toxicity
A15134	Acute Toxicity
A15135	Acute Toxicity
A15136	Acute Toxicity
A15137	Acute Toxicity
A15138	Acute Toxicity
A15139	Acute Toxicity
A15140	Acute Toxicity
A15141	Acute Toxicity
A15142	Acute Toxicity
A15143	Acute Toxicity
A15144	Acute Toxicity
A15145	Acute Toxicity
A15146	Acute Toxicity
A15147	Acute Toxicity
A15148	Acute Toxicity
A15149	Acute Toxicity
A15150	Acute Toxicity
A15151	Acute Toxicity
A15152	Acute Toxicity
A15153	Acute Toxicity
A15154	Acute Toxicity
A15155	Acute Toxicity
A15156	Acute Toxicity
A15157	Acute Toxicity
A15158	Acute Toxicity
A15159	Acute Toxicity
A15160	Acute Toxicity
A15161	Acute Toxicity
A15162	Acute Toxicity
A15163	Acute Toxicity
A15164	Acute Toxicity
A15165	Acute Toxicity
A15166	Acute Toxicity
A15167	Acute Toxicity
A15168	Acute Toxicity

A15169	Acute Toxicity
A15170	Acute Toxicity
A15171	Acute Toxicity
A15172	Acute Toxicity
A15173	Acute Toxicity
A15174	Acute Toxicity
A15175	Multiple Dose Effects
A15176	Acute Toxicity
A15177	Acute Toxicity, Reproductive Effects, Multiple Dose Effects
A15178	Mutagenicity
A15179	Skin and Eye Irritation
A15180	Acute Toxicity
A15181	Mutagenicity
A15182	Non-toxicity
A15183	Acute Toxicity
A15184	Acute Toxicity
A15185	Acute Toxicity
A15186	Acute Toxicity
A15187	Multiple Dose Effects
A15188	Acute Toxicity, Multiple Dose Effects
A15189	Acute Toxicity, Multiple Dose Effects
A15190	Mutagenicity, Multiple Dose Effects
A15191	Acute Toxicity
A15192	Acute Toxicity, Skin and Eye Irritation
A15193	Acute Toxicity
A15194	Multiple Dose Effects
A15195	Acute Toxicity
A15196	Acute Toxicity
A15197	Acute Toxicity
A15198	Acute Toxicity
A15199	Acute Toxicity
A15200	Mutagenicity
A15201	Mutagenicity
A15202	Multiple Dose Effects
A15203	Acute Toxicity
A15204	Acute Toxicity
A15205	Acute Toxicity
A15206	Acute Toxicity
A15207	Acute Toxicity
A15208	Acute Toxicity
A15209	Acute Toxicity
A15210	Acute Toxicity
A15211	Acute Toxicity
A15212	Acute Toxicity, Multiple Dose Effects
A15213	Acute Toxicity
A15214	Acute Toxicity

A15215	Acute Toxicity
A15216	Acute Toxicity
A15217	Multiple Dose Effects
A15218	Acute Toxicity, Multiple Dose Effects
A15219	Acute Toxicity
A15220	Acute Toxicity
A15221	Acute Toxicity
A15222	Acute Toxicity, Reproductive Effects
A15223	Acute Toxicity, Multiple Dose Effects
A15224	Acute Toxicity
A15225	Acute Toxicity, Multiple Dose Effects
A15226	Acute Toxicity, Mutagenicity, Tumorigenicity, Skin and Eye Irritation
A15227	Acute Toxicity, Mutagenicity
A15228	Mutagenicity
A15229	Acute Toxicity
A15230	Non-toxicity
A15231	Acute Toxicity, Skin and Eye Irritation
A15232	Acute Toxicity, Mutagenicity, Tumorigenicity, Multiple Dose Effects
A15233	Acute Toxicity, Mutagenicity, Tumorigenicity, Skin and Eye Irritation, Multiple Dose Effects
A15234	Mutagenicity
A15235	Acute Toxicity
A15236	Acute Toxicity
A15237	Acute Toxicity, Tumorigenicity
A15238	Acute Toxicity, Mutagenicity, Tumorigenicity, Skin and Eye Irritation, Multiple Dose Effects
A15239	Acute Toxicity
A15240	Skin and Eye Irritation
A15241	Acute Toxicity, Mutagenicity, Skin and Eye Irritation
A15242	Skin and Eye Irritation
A15243	Acute Toxicity, Skin and Eye Irritation, Multiple Dose Effects
A15244	Acute Toxicity
A15245	Multiple Dose Effects
A15246	Acute Toxicity
A15247	Acute Toxicity
A15248	Mutagenicity
A15249	Mutagenicity
A15250	Acute Toxicity
A15251	Acute Toxicity
A15252	Multiple Dose Effects
A15253	Skin and Eye Irritation
A15254	Acute Toxicity
A15255	Acute Toxicity
A15256	Acute Toxicity
A15257	Acute Toxicity
A15258	Acute Toxicity, Mutagenicity, Tumorigenicity, Skin and Eye Irritation,

A15259	Acute Toxicity, Tumorigenicity
A15260	Mutagenicity
A15261	Acute Toxicity
A15262	Acute Toxicity
A15263	Mutagenicity
A15264	Acute Toxicity
A15265	Acute Toxicity
A15266	Acute Toxicity, Reproductive Effects, Multiple Dose Effects
A15267	Mutagenicity, Tumorigenicity
A15268	Acute Toxicity, Mutagenicity, Reproductive Effects, Multiple Dose Effects
A15269	Mutagenicity
A15270	Acute Toxicity, Multiple Dose Effects
A15271	Acute Toxicity, Mutagenicity
A15272	Acute Toxicity, Mutagenicity, Tumorigenicity, Multiple Dose Effects
A15273	Acute Toxicity, Mutagenicity
A15274	Acute Toxicity
A15275	Acute Toxicity, Multiple Dose Effects
A15276	Mutagenicity
A15277	Acute Toxicity, Mutagenicity, Tumorigenicity, Reproductive Effects
A15278	Multiple Dose Effects
A15279	Mutagenicity
A15280	Acute Toxicity
A15281	Acute Toxicity, Skin and Eye Irritation, Multiple Dose Effects
A15282	Acute Toxicity, Mutagenicity, Tumorigenicity, Skin and Eye Irritation, Multiple Dose Effects
A15283	Acute Toxicity, Mutagenicity, Skin and Eye Irritation
A15284	Multiple Dose Effects
A15285	Acute Toxicity, Skin and Eye Irritation
A15286	Acute Toxicity
A15287	Acute Toxicity
A15288	Acute Toxicity
A15289	Multiple Dose Effects
A15290	Acute Toxicity, Skin and Eye Irritation
A15291	Tumorigenicity
A15292	Acute Toxicity, Mutagenicity, Tumorigenicity, Reproductive Effects,
A15293	Non-toxicity
A15294	Non-toxicity
A15295	Acute Toxicity, Mutagenicity, Reproductive Effects
A15296	Non-toxicity
A15297	Acute Toxicity, Mutagenicity, Reproductive Effects, Multiple Dose Effects
A15298	Mutagenicity
A15299	Acute Toxicity
A15300	Non-toxicity
A15301	Mutagenicity, Tumorigenicity, Multiple Dose Effects
A15302	Reproductive Effects, Multiple Dose Effects
A15303	Non-toxicity

A15304	Mutagenicity
A15305	Mutagenicity
A15306	Acute Toxicity
A15307	Non-toxicity
A15308	Acute Toxicity, Tumorigenicity, Skin and Eye Irritation, Multiple Dose
A15309	Acute Toxicity, Multiple Dose Effects
A15310	Acute Toxicity, Mutagenicity
A15311	Acute Toxicity, Mutagenicity, Reproductive Effects
A15312	Acute Toxicity, Mutagenicity, Tumorigenicity, Multiple Dose Effects
A15313	Acute Toxicity, Mutagenicity, Tumorigenicity, Reproductive Effects,
A15314	Acute Toxicity, Mutagenicity, Tumorigenicity, Skin and Eye Irritation, Multiple Dose Effects
A15315	Acute Toxicity, Mutagenicity, Tumorigenicity, Reproductive Effects,
A15316	Acute Toxicity, Mutagenicity
A15317	Acute Toxicity, Mutagenicity
A15318	Acute Toxicity, Mutagenicity, Tumorigenicity
A15319	Acute Toxicity
A15320	Acute Toxicity, Multiple Dose Effects
A15321	Acute Toxicity, Reproductive Effects, Multiple Dose Effects
A15322	Acute Toxicity, Mutagenicity, Tumorigenicity, Skin and Eye Irritation, Multiple Dose Effects
A15323	Acute Toxicity, Multiple Dose Effects
A15324	Acute Toxicity, Mutagenicity
A15325	Non-toxicity
A15326	Reproductive Effects
A15327	Non-toxicity
A15328	Acute Toxicity, Mutagenicity, Tumorigenicity, Skin and Eye Irritation,
A15329	Acute Toxicity, Mutagenicity, Multiple Dose Effects
A15330	Acute Toxicity
A15331	Mutagenicity
A15332	Mutagenicity
A15333	Mutagenicity
A15334	Acute Toxicity
A15335	Acute Toxicity, Multiple Dose Effects
A15336	Mutagenicity
A15337	Mutagenicity
A15338	Acute Toxicity, Mutagenicity, Reproductive Effects, Multiple Dose Effects
A15339	Mutagenicity
A15340	Non-toxicity
A15341	Mutagenicity
A15342	Non-toxicity
A15343	Non-toxicity
A15344	Non-toxicity
A15345	Acute Toxicity, Tumorigenicity, Reproductive Effects, Multiple Dose Effects
A15346	Acute Toxicity, Tumorigenicity, Multiple Dose Effects
A15347	Acute Toxicity, Mutagenicity, Tumorigenicity, Reproductive Effects,

A15348	Acute Toxicity, Mutagenicity, Tumorigenicity, Multiple Dose Effects
A15349	Acute Toxicity, Mutagenicity, Tumorigenicity, Multiple Dose Effects
A15350	Non-toxicity
A15351	Non-toxicity
A15352	Acute Toxicity, Mutagenicity, Tumorigenicity, Multiple Dose Effects
A15353	Acute Toxicity, Mutagenicity
A15354	Acute Toxicity, Reproductive Effects
A15355	Acute Toxicity
A15356	Acute Toxicity
A15357	Multiple Dose Effects
A15358	Acute Toxicity, Mutagenicity, Reproductive Effects
A15359	Acute Toxicity
A15360	Mutagenicity, Tumorigenicity, Multiple Dose Effects
A15361	Mutagenicity, Tumorigenicity, Multiple Dose Effects
A15362	Reproductive Effects
A15363	Mutagenicity, Reproductive Effects, Multiple Dose Effects
A15364	Multiple Dose Effects
A15365	Acute Toxicity, Mutagenicity, Reproductive Effects
A15366	Acute Toxicity, Mutagenicity, Tumorigenicity, Reproductive Effects,
A15367	Acute Toxicity, Mutagenicity, Skin and Eye Irritation, Reproductive Effects, Multiple Dose Effects
A15368	Mutagenicity, Tumorigenicity
A15369	Acute Toxicity, Reproductive Effects, Multiple Dose Effects
A15370	Acute Toxicity, Tumorigenicity, Skin and Eye Irritation, Reproductive Effects, Multiple Dose Effects
A15371	Acute Toxicity, Multiple Dose Effects
A15372	Acute Toxicity, Mutagenicity, Reproductive Effects, Multiple Dose Effects
A15373	Acute Toxicity, Multiple Dose Effects
A15374	Acute Toxicity, Multiple Dose Effects
A15375	Acute Toxicity
A15376	Acute Toxicity, Mutagenicity
A15377	Acute Toxicity
A15378	Acute Toxicity, Reproductive Effects
A15379	Acute Toxicity, Reproductive Effects, Multiple Dose Effects
A15380	Acute Toxicity, Multiple Dose Effects
A15381	Acute Toxicity, Multiple Dose Effects
A15382	Non-toxicity
A15383	Non-toxicity
A15384	Acute Toxicity, Multiple Dose Effects
A15385	Multiple Dose Effects
A15386	Multiple Dose Effects
A15387	Multiple Dose Effects
A15388	Non-toxicity
A15389	Acute Toxicity
A15390	Mutagenicity
A15391	Skin and Eye Irritation

A15392	Skin and Eye Irritation
A15393	Acute Toxicity
A15394	Acute Toxicity, Multiple Dose Effects
A15395	Acute Toxicity, Mutagenicity, Skin and Eye Irritation, Multiple Dose Effects
A15396	Acute Toxicity, Multiple Dose Effects
A15397	Acute Toxicity, Tumorigenicity, Multiple Dose Effects
A15398	Acute Toxicity, Mutagenicity, Tumorigenicity
A15399	Tumorigenicity, Multiple Dose Effects
A15400	Multiple Dose Effects
A15401	Mutagenicity, Tumorigenicity
A15402	Multiple Dose Effects
A15403	Acute Toxicity, Multiple Dose Effects
A15404	Multiple Dose Effects
A15405	Acute Toxicity
A15406	Acute Toxicity
A15407	Tumorigenicity
A15408	Acute Toxicity, Multiple Dose Effects
A15409	Non-toxicity
A15410	Non-toxicity
A15411	Acute Toxicity, Multiple Dose Effects
A15412	Multiple Dose Effects
A15413	Acute Toxicity, Tumorigenicity, Multiple Dose Effects
A15414	Acute Toxicity
A15415	Acute Toxicity, Mutagenicity
A15416	Acute Toxicity, Mutagenicity, Tumorigenicity, Multiple Dose Effects
A15417	Mutagenicity
A15418	Acute Toxicity
A15419	Acute Toxicity, Mutagenicity, Tumorigenicity, Multiple Dose Effects
A15420	Acute Toxicity, Mutagenicity, Tumorigenicity, Skin and Eye Irritation
A15421	Tumorigenicity
A15422	Acute Toxicity, Mutagenicity, Tumorigenicity, Multiple Dose Effects
A15423	Acute Toxicity, Mutagenicity, Skin and Eye Irritation
A15424	Mutagenicity
A15425	Acute Toxicity, Mutagenicity, Skin and Eye Irritation
A15426	Non-toxicity
A15427	Skin and Eye Irritation
A15428	Acute Toxicity
A15429	Acute Toxicity
A15430	Acute Toxicity, Mutagenicity, Tumorigenicity, Skin and Eye Irritation,
A15431	Acute Toxicity, Mutagenicity, Multiple Dose Effects
A15432	Mutagenicity, Multiple Dose Effects
A15433	Acute Toxicity
A15434	Acute Toxicity, Mutagenicity, Tumorigenicity
A15435	Acute Toxicity, Mutagenicity, Multiple Dose Effects
A15436	Acute Toxicity, Mutagenicity
A15437	Acute Toxicity

A15438	Mutagenicity
A15439	Mutagenicity
A15440	Mutagenicity, Multiple Dose Effects
A15441	Mutagenicity
A15442	Mutagenicity
A15443	Mutagenicity
A15444	Mutagenicity
A15445	Acute Toxicity, Multiple Dose Effects
A15446	Mutagenicity
A15447	Acute Toxicity, Mutagenicity
A15448	Mutagenicity
A15449	Mutagenicity
A15450	Mutagenicity
A15451	Acute Toxicity, Mutagenicity
A15452	Mutagenicity, Tumorigenicity, Multiple Dose Effects
A15453	Mutagenicity
A15454	Acute Toxicity, Mutagenicity
A15455	Acute Toxicity
A15456	Mutagenicity
A15457	Mutagenicity, Tumorigenicity
A15458	Mutagenicity
A15459	Multiple Dose Effects
A15460	Mutagenicity
A15461	Acute Toxicity, Mutagenicity, Multiple Dose Effects
A15462	Acute Toxicity, Mutagenicity, Reproductive Effects, Multiple Dose Effects
A15463	Acute Toxicity, Mutagenicity, Tumorigenicity, Reproductive Effects,
A15464	Multiple Dose Effects
A15465	Acute Toxicity
A15466	Mutagenicity
A15467	Mutagenicity
A15468	Acute Toxicity, Mutagenicity, Multiple Dose Effects
A15469	Acute Toxicity, Mutagenicity, Multiple Dose Effects
A15470	Acute Toxicity, Mutagenicity, Tumorigenicity, Multiple Dose Effects
A15471	Acute Toxicity, Mutagenicity, Tumorigenicity, Reproductive Effects,
A15472	Acute Toxicity, Mutagenicity, Multiple Dose Effects
A15473	Acute Toxicity, Mutagenicity, Tumorigenicity
A15474	Acute Toxicity
A15475	Mutagenicity, Tumorigenicity
A15476	Acute Toxicity, Reproductive Effects, Multiple Dose Effects
A15477	Acute Toxicity
A15478	Acute Toxicity, Mutagenicity, Multiple Dose Effects
A15479	Multiple Dose Effects
A15480	Acute Toxicity, Multiple Dose Effects
A15481	Multiple Dose Effects
A15482	Acute Toxicity, Mutagenicity, Multiple Dose Effects
A15483	Acute Toxicity

A15484	Acute Toxicity, Mutagenicity, Reproductive Effects, Multiple Dose Effects
A15485	Acute Toxicity
A15486	Acute Toxicity, Mutagenicity
A15487	Acute Toxicity, Multiple Dose Effects
A15488	Acute Toxicity
A15489	Acute Toxicity, Mutagenicity
A15490	Acute Toxicity
A15491	Acute Toxicity
A15492	Acute Toxicity, Reproductive Effects, Multiple Dose Effects
A15493	Mutagenicity
A15494	Mutagenicity
A15495	Acute Toxicity, Mutagenicity, Tumorigenicity, Reproductive Effects,
A15496	Acute Toxicity, Multiple Dose Effects
A15497	Acute Toxicity, Reproductive Effects, Multiple Dose Effects
A15498	Multiple Dose Effects
A15499	Acute Toxicity
A15500	Acute Toxicity
A15501	Multiple Dose Effects
A15502	Acute Toxicity
A15503	Acute Toxicity
A15504	Acute Toxicity
A15505	Mutagenicity, Reproductive Effects
A15506	Acute Toxicity, Mutagenicity, Reproductive Effects, Multiple Dose Effects
A15507	Acute Toxicity, Reproductive Effects, Multiple Dose Effects
A15508	Multiple Dose Effects
A15509	Acute Toxicity
A15510	Acute Toxicity, Mutagenicity
A15511	Acute Toxicity, Tumorigenicity
A15512	Acute Toxicity
A15513	Acute Toxicity, Mutagenicity
A15514	Acute Toxicity
A15515	Mutagenicity, Multiple Dose Effects
A15516	Acute Toxicity
A15517	Acute Toxicity, Skin and Eye Irritation
A15518	Acute Toxicity, Skin and Eye Irritation
A15519	Acute Toxicity, Multiple Dose Effects
A15520	Acute Toxicity, Reproductive Effects, Multiple Dose Effects
A15521	Acute Toxicity, Reproductive Effects, Multiple Dose Effects
A15522	Acute Toxicity
A15523	Acute Toxicity
A15524	Acute Toxicity, Tumorigenicity, Skin and Eye Irritation, Reproductive Effects, Multiple Dose Effects
A15525	Acute Toxicity, Multiple Dose Effects
A15526	Acute Toxicity
A15527	Acute Toxicity
A15528	Non-toxicity

A15529	Acute Toxicity, Multiple Dose Effects
A15530	Acute Toxicity, Reproductive Effects, Multiple Dose Effects
A15531	Acute Toxicity, Skin and Eye Irritation
A15532	Acute Toxicity, Multiple Dose Effects
A15533	Acute Toxicity, Mutagenicity, Reproductive Effects, Multiple Dose Effects
A15534	Acute Toxicity, Multiple Dose Effects
A15535	Acute Toxicity, Multiple Dose Effects
A15536	Acute Toxicity
A15537	Acute Toxicity
A15538	Acute Toxicity, Reproductive Effects
A15539	Acute Toxicity, Mutagenicity, Skin and Eye Irritation, Reproductive Effects, Multiple Dose Effects
A15540	Acute Toxicity, Reproductive Effects, Multiple Dose Effects
A15541	Multiple Dose Effects
A15542	Mutagenicity, Reproductive Effects, Multiple Dose Effects
A15543	Acute Toxicity, Reproductive Effects, Multiple Dose Effects
A15544	Acute Toxicity
A15545	Acute Toxicity, Skin and Eye Irritation
A15546	Acute Toxicity, Reproductive Effects, Multiple Dose Effects
A15547	Acute Toxicity
A15548	Acute Toxicity
A15549	Acute Toxicity
A15550	Acute Toxicity
A15551	Acute Toxicity
A15552	Mutagenicity, Tumorigenicity, Reproductive Effects, Multiple Dose Effects
A15553	Acute Toxicity, Mutagenicity
A15554	Mutagenicity
A15555	Acute Toxicity, Reproductive Effects, Multiple Dose Effects
A15556	Acute Toxicity, Mutagenicity, Tumorigenicity, Reproductive Effects,
A15557	Acute Toxicity, Mutagenicity, Tumorigenicity, Reproductive Effects,
A15558	Acute Toxicity, Tumorigenicity, Skin and Eye Irritation, Reproductive Effects, Multiple Dose Effects
A15559	Acute Toxicity, Reproductive Effects, Multiple Dose Effects
A15560	Acute Toxicity, Reproductive Effects, Multiple Dose Effects
A15561	Acute Toxicity, Multiple Dose Effects
A15562	Acute Toxicity, Reproductive Effects, Multiple Dose Effects
A15563	Acute Toxicity, Reproductive Effects, Multiple Dose Effects
A15564	Acute Toxicity, Reproductive Effects, Multiple Dose Effects
A15565	Acute Toxicity, Reproductive Effects, Multiple Dose Effects
A15566	Mutagenicity
A15567	Acute Toxicity, Reproductive Effects, Multiple Dose Effects
A15568	Reproductive Effects
A15569	Skin and Eye Irritation
A15570	Acute Toxicity
A15571	Non-toxicity
A15572	Non-toxicity

A15573	Non-toxicity
A15574	Non-toxicity
A15575	Non-toxicity
A15576	Non-toxicity
A15577	Non-toxicity
A15578	Tumorigenicity
A15579	Multiple Dose Effects
A15580	Non-toxicity
A15581	Non-toxicity
A15582	Non-toxicity
A15583	Non-toxicity
A15584	Non-toxicity
A15585	Non-toxicity
A15586	Non-toxicity
A15587	Non-toxicity
A15588	Acute Toxicity
A15589	Acute Toxicity, Mutagenicity, Multiple Dose Effects
A15590	Acute Toxicity
A15591	Reproductive Effects
A15592	Mutagenicity, Reproductive Effects
A15593	Reproductive Effects
A15594	Acute Toxicity
A15595	Acute Toxicity, Reproductive Effects
A15596	Acute Toxicity
A15597	Acute Toxicity
A15598	Acute Toxicity, Mutagenicity, Multiple Dose Effects
A15599	Acute Toxicity, Mutagenicity
A15600	Mutagenicity
A15601	Acute Toxicity, Mutagenicity
A15602	Acute Toxicity, Skin and Eye Irritation
A15603	Mutagenicity
A15604	Acute Toxicity, Mutagenicity
A15605	Acute Toxicity, Mutagenicity, Skin and Eye Irritation
A15606	Acute Toxicity, Mutagenicity, Tumorigenicity
A15607	Acute Toxicity, Skin and Eye Irritation
A15608	Mutagenicity
A15609	Acute Toxicity, Skin and Eye Irritation
A15610	Acute Toxicity
A15611	Acute Toxicity, Multiple Dose Effects
A15612	Acute Toxicity
A15613	Skin and Eye Irritation
A15614	Non-toxicity
A15615	Non-toxicity
A15616	Non-toxicity
A15617	Multiple Dose Effects
A15618	Acute Toxicity, Mutagenicity, Skin and Eye Irritation, Multiple Dose Effects

A15619	Acute Toxicity, Tumorigenicity, Multiple Dose Effects
A15620	Mutagenicity
A15621	Mutagenicity
A15622	Mutagenicity, Tumorigenicity
A15623	Acute Toxicity, Mutagenicity, Tumorigenicity, Skin and Eye Irritation, Multiple Dose Effects
A15624	Acute Toxicity, Tumorigenicity, Skin and Eye Irritation, Reproductive Effects, Multiple Dose Effects
A15625	Acute Toxicity, Mutagenicity, Tumorigenicity, Reproductive Effects,
A15626	Acute Toxicity, Mutagenicity, Tumorigenicity
A15627	Mutagenicity, Tumorigenicity
A15628	Acute Toxicity, Mutagenicity, Tumorigenicity, Reproductive Effects,
A15629	Acute Toxicity, Tumorigenicity, Multiple Dose Effects
A15630	Tumorigenicity
A15631	Acute Toxicity, Reproductive Effects, Multiple Dose Effects
A15632	Tumorigenicity
A15633	Acute Toxicity, Mutagenicity, Tumorigenicity, Multiple Dose Effects
A15634	Acute Toxicity, Mutagenicity, Tumorigenicity, Multiple Dose Effects
A15635	Acute Toxicity, Mutagenicity
A15636	Acute Toxicity, Mutagenicity, Tumorigenicity, Skin and Eye Irritation,
A15637	Acute Toxicity, Mutagenicity, Tumorigenicity, Multiple Dose Effects
A15638	Mutagenicity, Tumorigenicity
A15639	Skin and Eye Irritation
A15640	Acute Toxicity
A15641	Mutagenicity
A15642	Acute Toxicity, Tumorigenicity, Skin and Eye Irritation
A15643	Acute Toxicity
A15644	Acute Toxicity, Tumorigenicity, Reproductive Effects, Multiple Dose Effects
A15645	Acute Toxicity, Reproductive Effects, Multiple Dose Effects
A15646	Skin and Eye Irritation
A15647	Reproductive Effects
A15648	Acute Toxicity, Reproductive Effects, Multiple Dose Effects
A15649	Acute Toxicity
A15650	Reproductive Effects
A15651	Acute Toxicity
A15652	Acute Toxicity, Skin and Eye Irritation, Multiple Dose Effects
A15653	Acute Toxicity, Reproductive Effects, Multiple Dose Effects
A15654	Acute Toxicity, Multiple Dose Effects
A15655	Acute Toxicity, Reproductive Effects
A15656	Skin and Eye Irritation, Reproductive Effects
A15657	Acute Toxicity, Reproductive Effects, Multiple Dose Effects
A15658	Acute Toxicity, Multiple Dose Effects
A15659	Acute Toxicity
A15660	Acute Toxicity, Mutagenicity, Reproductive Effects, Multiple Dose Effects
A15661	Acute Toxicity, Multiple Dose Effects
A15662	Acute Toxicity

A15663	Acute Toxicity, Skin and Eye Irritation, Multiple Dose Effects
A15664	Acute Toxicity, Skin and Eye Irritation, Reproductive Effects, Multiple Dose
A15665	Acute Toxicity, Mutagenicity, Tumorigenicity, Multiple Dose Effects
A15666	Acute Toxicity, Multiple Dose Effects
A15667	Acute Toxicity
A15668	Acute Toxicity
A15669	Mutagenicity, Reproductive Effects, Multiple Dose Effects
A15670	Mutagenicity
A15671	Mutagenicity
A15672	Mutagenicity
A15673	Acute Toxicity
A15674	Acute Toxicity
A15675	Acute Toxicity, Mutagenicity
A15676	Tumorigenicity, Reproductive Effects
A15677	Mutagenicity
A15678	Acute Toxicity
A15679	Tumorigenicity, Reproductive Effects, Multiple Dose Effects
A15680	Acute Toxicity, Mutagenicity, Tumorigenicity, Reproductive Effects,
A15681	Multiple Dose Effects
A15682	Acute Toxicity, Mutagenicity
A15683	Acute Toxicity, Mutagenicity, Skin and Eye Irritation, Reproductive Effects, Multiple Dose Effects
A15684	Acute Toxicity, Mutagenicity, Skin and Eye Irritation, Reproductive Effects, Multiple Dose Effects
A15685	Acute Toxicity, Mutagenicity
A15686	Non-toxicity
A15687	Non-toxicity
A15688	Reproductive Effects
A15689	Acute Toxicity
A15690	Tumorigenicity, Reproductive Effects, Multiple Dose Effects
A15691	Mutagenicity
A15692	Non-toxicity
A15693	Acute Toxicity
A15694	Acute Toxicity
A15695	Acute Toxicity
A15696	Acute Toxicity, Tumorigenicity
A15697	Acute Toxicity
A15698	Acute Toxicity
A15699	Acute Toxicity
A15700	Acute Toxicity
A15701	Acute Toxicity, Multiple Dose Effects
A15702	Reproductive Effects
A15703	Non-toxicity
A15704	Acute Toxicity, Mutagenicity
A15705	Acute Toxicity, Multiple Dose Effects
A15706	Acute Toxicity, Reproductive Effects, Multiple Dose Effects

A15707	Acute Toxicity
A15708	Acute Toxicity
A15709	Acute Toxicity, Multiple Dose Effects
A15710	Acute Toxicity
A15711	Acute Toxicity
A15712	Non-toxicity
A15713	Non-toxicity
A15714	Acute Toxicity, Mutagenicity, Skin and Eye Irritation, Multiple Dose Effects
A15715	Acute Toxicity, Multiple Dose Effects
A15716	Acute Toxicity, Reproductive Effects, Multiple Dose Effects
A15717	Acute Toxicity
A15718	Acute Toxicity, Skin and Eye Irritation
A15719	Skin and Eye Irritation, Reproductive Effects
A15720	Acute Toxicity, Multiple Dose Effects
A15721	Acute Toxicity
A15722	Acute Toxicity
A15723	Acute Toxicity
A15724	Acute Toxicity, Multiple Dose Effects
A15725	Acute Toxicity
A15726	Mutagenicity, Reproductive Effects, Multiple Dose Effects
A15727	Acute Toxicity
A15728	Acute Toxicity
A15729	Acute Toxicity, Reproductive Effects, Multiple Dose Effects
A15730	Acute Toxicity
A15731	Acute Toxicity
A15732	Acute Toxicity
A15733	Acute Toxicity, Reproductive Effects, Multiple Dose Effects
A15734	Acute Toxicity
A15735	Acute Toxicity, Skin and Eye Irritation, Multiple Dose Effects
A15736	Acute Toxicity, Reproductive Effects
A15737	Acute Toxicity
A15738	Acute Toxicity, Multiple Dose Effects
A15739	Multiple Dose Effects
A15740	Multiple Dose Effects
A15741	Acute Toxicity
A15742	Acute Toxicity
A15743	Acute Toxicity
A15744	Acute Toxicity
A15745	Multiple Dose Effects
A15746	Acute Toxicity, Mutagenicity
A15747	Acute Toxicity
A15748	Acute Toxicity
A15749	Acute Toxicity, Reproductive Effects
A15750	Acute Toxicity, Skin and Eye Irritation, Multiple Dose Effects
A15751	Acute Toxicity
A15752	Tumorigenicity

A15753	Acute Toxicity
A15754	Acute Toxicity
A15755	Acute Toxicity
A15756	Acute Toxicity, Multiple Dose Effects
A15757	Reproductive Effects
A15758	Acute Toxicity
A15759	Acute Toxicity, Multiple Dose Effects
A15760	Acute Toxicity
A15761	Acute Toxicity
A15762	Acute Toxicity, Multiple Dose Effects
A15763	Acute Toxicity
A15764	Acute Toxicity
A15765	Acute Toxicity, Reproductive Effects
A15766	Acute Toxicity
A15767	Acute Toxicity
A15768	Acute Toxicity, Skin and Eye Irritation, Multiple Dose Effects
A15769	Acute Toxicity
A15770	Mutagenicity, Reproductive Effects
A15771	Acute Toxicity, Multiple Dose Effects
A15772	Acute Toxicity
A15773	Acute Toxicity
A15774	Acute Toxicity
A15775	Acute Toxicity
A15776	Acute Toxicity
A15777	Acute Toxicity
A15778	Acute Toxicity, Multiple Dose Effects
A15779	Acute Toxicity, Reproductive Effects
A15780	Non-toxicity
A15781	Non-toxicity
A15782	Non-toxicity
A15783	Non-toxicity
A15784	Non-toxicity
A15785	Non-toxicity
A15786	Non-toxicity
A15787	Non-toxicity
A15788	Mutagenicity
A15789	Acute Toxicity
A15790	Mutagenicity
A15791	Acute Toxicity
A15792	Acute Toxicity
A15793	Acute Toxicity, Multiple Dose Effects
A15794	Acute Toxicity
A15795	Acute Toxicity, Mutagenicity, Skin and Eye Irritation, Reproductive Effects, Multiple Dose Effects
A15796	Reproductive Effects
A15797	Acute Toxicity

A15798	Acute Toxicity
A15799	Mutagenicity
A15800	Acute Toxicity
A15801	Acute Toxicity
A15802	Acute Toxicity
A15803	Skin and Eye Irritation
A15804	Multiple Dose Effects
A15805	Acute Toxicity, Multiple Dose Effects
A15806	Acute Toxicity, Skin and Eye Irritation
A15807	Multiple Dose Effects
A15808	Acute Toxicity
A15809	Acute Toxicity, Mutagenicity, Multiple Dose Effects
A15810	Acute Toxicity
A15811	Mutagenicity, Reproductive Effects
A15812	Acute Toxicity
A15813	Acute Toxicity
A15814	Multiple Dose Effects
A15815	Acute Toxicity
A15816	Multiple Dose Effects
A15817	Multiple Dose Effects
A15818	Acute Toxicity, Multiple Dose Effects
A15819	Acute Toxicity
A15820	Acute Toxicity, Multiple Dose Effects
A15821	Acute Toxicity, Skin and Eye Irritation
A15822	Acute Toxicity
A15823	Mutagenicity
A15824	Acute Toxicity, Multiple Dose Effects
A15825	Skin and Eye Irritation
A15826	Acute Toxicity, Mutagenicity, Multiple Dose Effects
A15827	Multiple Dose Effects
A15828	Acute Toxicity, Mutagenicity, Multiple Dose Effects
A15829	Acute Toxicity
A15830	Mutagenicity
A15831	Acute Toxicity, Multiple Dose Effects
A15832	Mutagenicity
A15833	Acute Toxicity
A15834	Skin and Eye Irritation
A15835	Acute Toxicity
A15836	Acute Toxicity
A15837	Acute Toxicity, Mutagenicity, Skin and Eye Irritation, Reproductive Effects, Multiple Dose Effects
A15838	Acute Toxicity, Mutagenicity, Tumorigenicity, Skin and Eye Irritation,
A15839	Acute Toxicity, Mutagenicity
A15840	Acute Toxicity, Mutagenicity, Tumorigenicity
A15841	Acute Toxicity
A15842	Acute Toxicity

A15843	Acute Toxicity, Skin and Eye Irritation
A15844	Mutagenicity
A15845	Acute Toxicity
A15846	Acute Toxicity, Skin and Eye Irritation
A15847	Multiple Dose Effects
A15848	Non-toxicity
A15849	Mutagenicity
A15850	Multiple Dose Effects
A15851	Acute Toxicity, Mutagenicity
A15852	Acute Toxicity
A15853	Acute Toxicity, Multiple Dose Effects
A15854	Acute Toxicity
A15855	Acute Toxicity, Multiple Dose Effects
A15856	Multiple Dose Effects
A15857	Multiple Dose Effects
A15858	Acute Toxicity, Mutagenicity, Multiple Dose Effects
A15859	Acute Toxicity
A15860	Mutagenicity
A15861	Multiple Dose Effects
A15862	Non-toxicity
A15863	Acute Toxicity
A15864	Acute Toxicity
A15865	Acute Toxicity
A15866	Acute Toxicity
A15867	Acute Toxicity
A15868	Non-toxicity
A15869	Non-toxicity
A15870	Acute Toxicity, Skin and Eye Irritation
A15871	Multiple Dose Effects
A15872	Acute Toxicity
A15873	Acute Toxicity
A15874	Acute Toxicity, Mutagenicity, Skin and Eye Irritation, Reproductive Effects, Multiple Dose Effects
A15875	Acute Toxicity, Mutagenicity
A15876	Non-toxicity
A15877	Skin and Eye Irritation
A15878	Acute Toxicity
A15879	Acute Toxicity
A15880	Acute Toxicity
A15881	Acute Toxicity
A15882	Acute Toxicity, Skin and Eye Irritation
A15883	Acute Toxicity, Mutagenicity
A15884	Acute Toxicity
A15885	Acute Toxicity, Mutagenicity, Skin and Eye Irritation, Multiple Dose Effects
A15886	Mutagenicity
A15887	Skin and Eye Irritation

A15888	Skin and Eye Irritation
A15889	Acute Toxicity
A15890	Multiple Dose Effects
A15891	Acute Toxicity
A15892	Acute Toxicity
A15893	Multiple Dose Effects
A15894	Mutagenicity
A15895	Skin and Eye Irritation
A15896	Mutagenicity, Skin and Eye Irritation, Multiple Dose Effects
A15897	Skin and Eye Irritation
A15898	Acute Toxicity
A15899	Multiple Dose Effects
A15900	Mutagenicity
A15901	Acute Toxicity
A15902	Acute Toxicity
A15903	Acute Toxicity
A15904	Acute Toxicity, Multiple Dose Effects
A15905	Acute Toxicity, Mutagenicity, Multiple Dose Effects
A15906	Acute Toxicity
A15907	Mutagenicity
A15908	Acute Toxicity
A15909	Mutagenicity, Tumorigenicity
A15910	Acute Toxicity
A15911	Acute Toxicity, Mutagenicity
A15912	Acute Toxicity, Reproductive Effects, Multiple Dose Effects
A15913	Mutagenicity
A15914	Acute Toxicity, Skin and Eye Irritation
A15915	Acute Toxicity
A15916	Acute Toxicity
A15917	Acute Toxicity
A15918	Acute Toxicity, Reproductive Effects, Multiple Dose Effects
A15919	Acute Toxicity
A15920	Acute Toxicity
A15921	Acute Toxicity
A15922	Acute Toxicity, Multiple Dose Effects
A15923	Acute Toxicity
A15924	Acute Toxicity
A15925	Acute Toxicity, Mutagenicity, Reproductive Effects
A15926	Acute Toxicity
A15927	Acute Toxicity
A15928	Acute Toxicity
A15929	Acute Toxicity, Multiple Dose Effects
A15930	Non-toxicity
A15931	Acute Toxicity, Mutagenicity, Tumorigenicity, Reproductive Effects,
A15932	Acute Toxicity, Reproductive Effects, Multiple Dose Effects
A15933	Acute Toxicity

A15934	Mutagenicity, Reproductive Effects, Multiple Dose Effects
A15935	Acute Toxicity
A15936	Acute Toxicity
A15937	Acute Toxicity
A15938	Non-toxicity
A15939	Skin and Eye Irritation
A15940	Acute Toxicity
A15941	Acute Toxicity, Multiple Dose Effects
A15942	Acute Toxicity, Reproductive Effects, Multiple Dose Effects
A15943	Acute Toxicity, Mutagenicity, Reproductive Effects, Multiple Dose Effects
A15944	Acute Toxicity, Reproductive Effects, Multiple Dose Effects
A15945	Acute Toxicity, Reproductive Effects, Multiple Dose Effects
A15946	Acute Toxicity, Skin and Eye Irritation, Multiple Dose Effects
A15947	Non-toxicity
A15948	Multiple Dose Effects
A15949	Multiple Dose Effects
A15950	Acute Toxicity
A15951	Non-toxicity
A15952	Acute Toxicity
A15953	Acute Toxicity, Multiple Dose Effects
A15954	Acute Toxicity, Skin and Eye Irritation, Multiple Dose Effects
A15955	Acute Toxicity, Skin and Eye Irritation
A15956	Acute Toxicity, Mutagenicity
A15957	Skin and Eye Irritation, Multiple Dose Effects
A15958	Acute Toxicity, Skin and Eye Irritation
A15959	Acute Toxicity
A15960	Skin and Eye Irritation
A15961	Acute Toxicity
A15962	Acute Toxicity
A15963	Mutagenicity
A15964	Acute Toxicity
A15965	Acute Toxicity, Skin and Eye Irritation
A15966	Acute Toxicity
A15967	Acute Toxicity
A15968	Acute Toxicity
A15969	Acute Toxicity
A15970	Acute Toxicity, Reproductive Effects, Multiple Dose Effects
A15971	Acute Toxicity, Mutagenicity
A15972	Acute Toxicity
A15973	Acute Toxicity
A15974	Acute Toxicity
A15975	Multiple Dose Effects
A15976	Mutagenicity, Tumorigenicity
A15977	Acute Toxicity
A15978	Acute Toxicity
A15979	Acute Toxicity

A15980	Acute Toxicity
A15981	Acute Toxicity, Mutagenicity
A15982	Acute Toxicity, Mutagenicity
A15983	Skin and Eye Irritation
A15984	Skin and Eye Irritation
A15985	Acute Toxicity
A15986	Reproductive Effects
A15987	Acute Toxicity
A15988	Acute Toxicity, Reproductive Effects
A15989	Acute Toxicity
A15990	Acute Toxicity, Multiple Dose Effects
A15991	Acute Toxicity
A15992	Acute Toxicity, Mutagenicity, Skin and Eye Irritation
A15993	Acute Toxicity
A15994	Acute Toxicity, Mutagenicity, Skin and Eye Irritation
A15995	Acute Toxicity, Skin and Eye Irritation
A15996	Acute Toxicity, Skin and Eye Irritation
A15997	Non-toxicity
A15998	Acute Toxicity
A15999	Skin and Eye Irritation, Multiple Dose Effects
A16000	Acute Toxicity
A16001	Acute Toxicity
A16002	Acute Toxicity, Mutagenicity, Reproductive Effects, Multiple Dose Effects
A16003	Mutagenicity
A16004	Multiple Dose Effects
A16005	Acute Toxicity, Mutagenicity
A16006	Acute Toxicity
A16007	Acute Toxicity, Skin and Eye Irritation
A16008	Acute Toxicity
A16009	Acute Toxicity, Skin and Eye Irritation
A16010	Skin and Eye Irritation
A16011	Acute Toxicity, Skin and Eye Irritation
A16012	Acute Toxicity
A16013	Non-toxicity
A16014	Acute Toxicity, Skin and Eye Irritation
A16015	Acute Toxicity
A16016	Acute Toxicity
A16017	Multiple Dose Effects
A16018	Acute Toxicity, Mutagenicity, Tumorigenicity, Multiple Dose Effects
A16019	Acute Toxicity
A16020	Acute Toxicity
A16021	Acute Toxicity, Mutagenicity, Skin and Eye Irritation, Multiple Dose Effects
A16022	Acute Toxicity
A16023	Acute Toxicity
A16024	Acute Toxicity, Multiple Dose Effects
A16025	Non-toxicity

A16026	Acute Toxicity, Mutagenicity, Reproductive Effects, Multiple Dose Effects
A16027	Acute Toxicity, Mutagenicity, Reproductive Effects, Multiple Dose Effects
A16028	Acute Toxicity, Mutagenicity, Multiple Dose Effects
A16029	Acute Toxicity
A16030	Tumorigenicity
A16031	Acute Toxicity, Skin and Eye Irritation
A16032	Acute Toxicity
A16033	Acute Toxicity, Multiple Dose Effects
A16034	Acute Toxicity, Mutagenicity, Reproductive Effects
A16035	Acute Toxicity
A16036	Acute Toxicity, Mutagenicity, Skin and Eye Irritation
A16037	Acute Toxicity
A16038	Mutagenicity, Skin and Eye Irritation
A16039	Acute Toxicity
A16040	Acute Toxicity
A16041	Multiple Dose Effects
A16042	Mutagenicity, Tumorigenicity
A16043	Mutagenicity
A16044	Acute Toxicity
A16045	Acute Toxicity
A16046	Mutagenicity
A16047	Acute Toxicity, Mutagenicity
A16048	Acute Toxicity, Mutagenicity, Multiple Dose Effects
A16049	Mutagenicity
A16050	Mutagenicity
A16051	Acute Toxicity
A16052	Mutagenicity
A16053	Acute Toxicity
A16054	Acute Toxicity
A16055	Acute Toxicity
A16056	Reproductive Effects, Multiple Dose Effects
A16057	Skin and Eye Irritation
A16058	Mutagenicity
A16059	Acute Toxicity
A16060	Acute Toxicity
A16061	Reproductive Effects
A16062	Acute Toxicity, Mutagenicity
A16063	Acute Toxicity, Multiple Dose Effects
A16064	Multiple Dose Effects
A16065	Acute Toxicity
A16066	Skin and Eye Irritation
A16067	Acute Toxicity, Skin and Eye Irritation, Reproductive Effects, Multiple Dose
A16068	Acute Toxicity, Mutagenicity, Tumorigenicity, Skin and Eye Irritation,
A16069	Acute Toxicity, Mutagenicity
A16070	Acute Toxicity, Mutagenicity, Reproductive Effects
A16071	Skin and Eye Irritation

A16072	Acute Toxicity
A16073	Acute Toxicity, Mutagenicity, Reproductive Effects, Multiple Dose Effects
A16074	Acute Toxicity, Mutagenicity, Tumorigenicity
A16075	Acute Toxicity
A16076	Acute Toxicity, Multiple Dose Effects
A16077	Mutagenicity, Multiple Dose Effects
A16078	Non-toxicity
A16079	Mutagenicity
A16080	Non-toxicity
A16081	Acute Toxicity
A16082	Non-toxicity
A16083	Non-toxicity
A16084	Non-toxicity
A16085	Non-toxicity
A16086	Acute Toxicity
A16087	Acute Toxicity, Mutagenicity, Tumorigenicity, Skin and Eye Irritation,
A16088	Acute Toxicity, Mutagenicity, Skin and Eye Irritation, Reproductive Effects, Multiple Dose Effects
A16089	Non-toxicity
A16090	Acute Toxicity
A16091	Acute Toxicity
A16092	Acute Toxicity, Multiple Dose Effects
A16093	Acute Toxicity
A16094	Acute Toxicity
A16095	Acute Toxicity
A16096	Acute Toxicity
A16097	Acute Toxicity
A16098	Acute Toxicity, Skin and Eye Irritation
A16099	Acute Toxicity, Mutagenicity, Skin and Eye Irritation, Multiple Dose Effects
A16100	Acute Toxicity, Multiple Dose Effects
A16101	Mutagenicity
A16102	Acute Toxicity
A16103	Acute Toxicity
A16104	Acute Toxicity
A16105	Acute Toxicity
A16106	Acute Toxicity
A16107	Acute Toxicity
A16108	Acute Toxicity
A16109	Multiple Dose Effects
A16110	Acute Toxicity
A16111	Acute Toxicity, Skin and Eye Irritation
A16112	Acute Toxicity
A16113	Multiple Dose Effects
A16114	Acute Toxicity, Multiple Dose Effects
A16115	Multiple Dose Effects
A16116	Acute Toxicity, Mutagenicity

A16117	Tumorigenicity, Multiple Dose Effects
A16118	Acute Toxicity
A16119	Mutagenicity
A16120	Acute Toxicity
A16121	Acute Toxicity, Mutagenicity, Tumorigenicity, Multiple Dose Effects
A16122	Reproductive Effects
A16123	Acute Toxicity, Tumorigenicity
A16124	Non-toxicity
A16125	Acute Toxicity
A16126	Acute Toxicity
A16127	Acute Toxicity, Skin and Eye Irritation, Reproductive Effects, Multiple Dose
A16128	Acute Toxicity, Skin and Eye Irritation, Multiple Dose Effects
A16129	Multiple Dose Effects
A16130	Multiple Dose Effects
A16131	Acute Toxicity
A16132	Acute Toxicity, Mutagenicity, Multiple Dose Effects
A16133	Acute Toxicity
A16134	Acute Toxicity, Mutagenicity, Skin and Eye Irritation, Multiple Dose Effects
A16135	Tumorigenicity
A16136	Acute Toxicity, Multiple Dose Effects
A16137	Acute Toxicity
A16138	Mutagenicity
A16139	Acute Toxicity
A16140	Mutagenicity
A16141	Tumorigenicity
A16142	Mutagenicity
A16143	Acute Toxicity, Mutagenicity
A16144	Acute Toxicity, Mutagenicity, Reproductive Effects, Multiple Dose Effects
A16145	Acute Toxicity
A16146	Acute Toxicity
A16147	Mutagenicity
A16148	Mutagenicity
A16149	Acute Toxicity
A16150	Mutagenicity
A16151	Acute Toxicity
A16152	Acute Toxicity, Mutagenicity
A16153	Acute Toxicity
A16154	Mutagenicity
A16155	Mutagenicity
A16156	Acute Toxicity
A16157	Acute Toxicity, Multiple Dose Effects
A16158	Mutagenicity
A16159	Acute Toxicity
A16160	Mutagenicity
A16161	Acute Toxicity, Multiple Dose Effects
A16162	Acute Toxicity

A16163	Acute Toxicity
A16164	Acute Toxicity
A16165	Mutagenicity
A16166	Mutagenicity
A16167	Acute Toxicity, Mutagenicity
A16168	Mutagenicity
A16169	Mutagenicity
A16170	Mutagenicity
A16171	Mutagenicity
A16172	Mutagenicity
A16173	Mutagenicity
A16174	Mutagenicity
A16175	Mutagenicity
A16176	Mutagenicity
A16177	Non-toxicity
A16178	Mutagenicity
A16179	Mutagenicity
A16180	Mutagenicity
A16181	Mutagenicity
A16182	Mutagenicity
A16183	Mutagenicity
A16184	Mutagenicity
A16185	Mutagenicity
A16186	Mutagenicity
A16187	Acute Toxicity
A16188	Acute Toxicity
A16189	Tumorigenicity
A16190	Mutagenicity
A16191	Acute Toxicity
A16192	Acute Toxicity
A16193	Acute Toxicity
A16194	Mutagenicity
A16195	Mutagenicity
A16196	Acute Toxicity
A16197	Acute Toxicity
A16198	Acute Toxicity
A16199	Acute Toxicity
A16200	Acute Toxicity
A16201	Acute Toxicity
A16202	Acute Toxicity
A16203	Acute Toxicity
A16204	Acute Toxicity
A16205	Acute Toxicity
A16206	Acute Toxicity
A16207	Acute Toxicity
A16208	Acute Toxicity

A16209	Acute Toxicity
A16210	Acute Toxicity
A16211	Acute Toxicity
A16212	Acute Toxicity, Multiple Dose Effects
A16213	Acute Toxicity
A16214	Mutagenicity
A16215	Acute Toxicity
A16216	Acute Toxicity, Tumorigenicity, Skin and Eye Irritation, Multiple Dose
A16217	Acute Toxicity
A16218	Acute Toxicity
A16219	Acute Toxicity
A16220	Mutagenicity
A16221	Acute Toxicity
A16222	Acute Toxicity
A16223	Acute Toxicity
A16224	Acute Toxicity, Multiple Dose Effects
A16225	Acute Toxicity, Mutagenicity, Skin and Eye Irritation, Multiple Dose Effects
A16226	Acute Toxicity, Skin and Eye Irritation, Reproductive Effects, Multiple Dose
A16227	Tumorigenicity
A16228	Acute Toxicity, Reproductive Effects, Multiple Dose Effects
A16229	Acute Toxicity
A16230	Acute Toxicity
A16231	Acute Toxicity, Skin and Eye Irritation, Multiple Dose Effects
A16232	Acute Toxicity
A16233	Acute Toxicity
A16234	Acute Toxicity, Multiple Dose Effects
A16235	Acute Toxicity
A16236	Acute Toxicity
A16237	Acute Toxicity
A16238	Acute Toxicity
A16239	Acute Toxicity
A16240	Acute Toxicity
A16241	Acute Toxicity
A16242	Acute Toxicity, Skin and Eye Irritation
A16243	Acute Toxicity
A16244	Acute Toxicity
A16245	Acute Toxicity
A16246	Acute Toxicity
A16247	Acute Toxicity
A16248	Mutagenicity
A16249	Acute Toxicity
A16250	Acute Toxicity
A16251	Acute Toxicity
A16252	Acute Toxicity
A16253	Acute Toxicity
A16254	Acute Toxicity

A16255	Acute Toxicity
A16256	Acute Toxicity
A16257	Acute Toxicity
A16258	Reproductive Effects
A16259	Acute Toxicity
A16260	Acute Toxicity
A16261	Acute Toxicity
A16262	Acute Toxicity, Mutagenicity, Tumorigenicity, Reproductive Effects,
A16263	Acute Toxicity, Tumorigenicity, Multiple Dose Effects
A16264	Mutagenicity
A16265	Acute Toxicity
A16266	Acute Toxicity
A16267	Mutagenicity
A16268	Mutagenicity
A16269	Acute Toxicity, Multiple Dose Effects
A16270	Mutagenicity
A16271	Mutagenicity
A16272	Acute Toxicity
A16273	Acute Toxicity
A16274	Acute Toxicity, Mutagenicity, Tumorigenicity, Reproductive Effects
A16275	Acute Toxicity
A16276	Acute Toxicity
A16277	Acute Toxicity, Multiple Dose Effects
A16278	Acute Toxicity
A16279	Acute Toxicity, Mutagenicity
A16280	Acute Toxicity
A16281	Acute Toxicity, Mutagenicity, Tumorigenicity
A16282	Acute Toxicity, Skin and Eye Irritation
A16283	Acute Toxicity, Mutagenicity, Multiple Dose Effects
A16284	Acute Toxicity, Mutagenicity, Multiple Dose Effects
A16285	Acute Toxicity
A16286	Acute Toxicity, Tumorigenicity, Reproductive Effects, Multiple Dose Effects
A16287	Acute Toxicity
A16288	Reproductive Effects, Multiple Dose Effects
A16289	Non-toxicity
A16290	Acute Toxicity, Mutagenicity
A16291	Acute Toxicity
A16292	Mutagenicity, Tumorigenicity, Reproductive Effects, Multiple Dose Effects
A16293	Acute Toxicity
A16294	Acute Toxicity, Reproductive Effects, Multiple Dose Effects
A16295	Acute Toxicity, Reproductive Effects
A16296	Non-toxicity
A16297	Acute Toxicity, Multiple Dose Effects
A16298	Acute Toxicity
A16299	Acute Toxicity, Mutagenicity, Skin and Eye Irritation, Reproductive Effects, Multiple Dose Effects

A16300	Multiple Dose Effects
A16301	Acute Toxicity
A16302	Acute Toxicity
A16303	Multiple Dose Effects
A16304	Acute Toxicity, Multiple Dose Effects
A16305	Acute Toxicity
A16306	Acute Toxicity
A16307	Acute Toxicity, Multiple Dose Effects
A16308	Mutagenicity
A16309	Acute Toxicity
A16310	Multiple Dose Effects
A16311	Acute Toxicity
A16312	Acute Toxicity
A16313	Non-toxicity
A16314	Multiple Dose Effects
A16315	Non-toxicity
A16316	Acute Toxicity
A16317	Multiple Dose Effects
A16318	Acute Toxicity
A16319	Acute Toxicity
A16320	Acute Toxicity
A16321	Acute Toxicity, Skin and Eye Irritation
A16322	Acute Toxicity, Reproductive Effects, Multiple Dose Effects
A16323	Acute Toxicity
A16324	Tumorigenicity
A16325	Acute Toxicity, Multiple Dose Effects
A16326	Acute Toxicity
A16327	Acute Toxicity
A16328	Acute Toxicity, Mutagenicity, Multiple Dose Effects
A16329	Acute Toxicity
A16330	Acute Toxicity
A16331	Acute Toxicity
A16332	Acute Toxicity, Mutagenicity, Skin and Eye Irritation, Reproductive Effects, Multiple Dose Effects
A16333	Acute Toxicity, Mutagenicity, Multiple Dose Effects
A16334	Acute Toxicity
A16335	Acute Toxicity, Reproductive Effects, Multiple Dose Effects
A16336	Reproductive Effects
A16337	Mutagenicity
A16338	Mutagenicity
A16339	Mutagenicity
A16340	Mutagenicity
A16341	Mutagenicity
A16342	Skin and Eye Irritation
A16343	Acute Toxicity
A16344	Acute Toxicity

A16345	Acute Toxicity, Multiple Dose Effects
A16346	Acute Toxicity
A16347	Acute Toxicity
A16348	Acute Toxicity
A16349	Acute Toxicity
A16350	Mutagenicity
A16351	Acute Toxicity
A16352	Mutagenicity
A16353	Acute Toxicity, Mutagenicity, Skin and Eye Irritation
A16354	Acute Toxicity
A16355	Mutagenicity
A16356	Acute Toxicity, Mutagenicity
A16357	Acute Toxicity
A16358	Acute Toxicity, Reproductive Effects, Multiple Dose Effects
A16359	Acute Toxicity
A16360	Acute Toxicity, Mutagenicity, Skin and Eye Irritation, Reproductive Effects, Multiple Dose Effects
A16361	Acute Toxicity
A16362	Acute Toxicity
A16363	Acute Toxicity
A16364	Acute Toxicity, Reproductive Effects, Multiple Dose Effects
A16365	Mutagenicity
A16366	Acute Toxicity
A16367	Mutagenicity, Tumorigenicity, Reproductive Effects
A16368	Acute Toxicity, Mutagenicity, Multiple Dose Effects
A16369	Acute Toxicity
A16370	Reproductive Effects
A16371	Acute Toxicity
A16372	Acute Toxicity
A16373	Reproductive Effects
A16374	Acute Toxicity
A16375	Mutagenicity, Reproductive Effects
A16376	Acute Toxicity
A16377	Acute Toxicity
A16378	Non-toxicity
A16379	Acute Toxicity
A16380	Reproductive Effects
A16381	Tumorigenicity, Reproductive Effects
A16382	Acute Toxicity, Mutagenicity
A16383	Reproductive Effects
A16384	Acute Toxicity
A16385	Acute Toxicity
A16386	Acute Toxicity
A16387	Acute Toxicity
A16388	Acute Toxicity
A16389	Acute Toxicity

A16390	Reproductive Effects
A16391	Acute Toxicity
A16392	Acute Toxicity
A16393	Non-toxicity
A16394	Acute Toxicity, Mutagenicity, Reproductive Effects, Multiple Dose Effects
A16395	Acute Toxicity
A16396	Acute Toxicity, Mutagenicity
A16397	Reproductive Effects
A16398	Skin and Eye Irritation
A16399	Acute Toxicity
A16400	Acute Toxicity, Mutagenicity, Multiple Dose Effects
A16401	Acute Toxicity
A16402	Acute Toxicity, Mutagenicity
A16403	Acute Toxicity, Reproductive Effects
A16404	Acute Toxicity
A16405	Non-toxicity
A16406	Acute Toxicity
A16407	Acute Toxicity
A16408	Acute Toxicity, Mutagenicity, Reproductive Effects, Multiple Dose Effects
A16409	Acute Toxicity
A16410	Multiple Dose Effects
A16411	Multiple Dose Effects
A16412	Acute Toxicity
A16413	Acute Toxicity
A16414	Reproductive Effects
A16415	Acute Toxicity
A16416	Mutagenicity, Tumorigenicity
A16417	Acute Toxicity, Multiple Dose Effects
A16418	Acute Toxicity, Skin and Eye Irritation
A16419	Acute Toxicity
A16420	Mutagenicity
A16421	Skin and Eye Irritation
A16422	Acute Toxicity, Multiple Dose Effects
A16423	Mutagenicity
A16424	Non-toxicity
A16425	Skin and Eye Irritation
A16426	Acute Toxicity
A16427	Tumorigenicity
A16428	Acute Toxicity
A16429	Reproductive Effects
A16430	Acute Toxicity, Reproductive Effects
A16431	Acute Toxicity, Multiple Dose Effects
A16432	Mutagenicity, Tumorigenicity, Reproductive Effects, Multiple Dose Effects
A16433	Multiple Dose Effects
A16434	Reproductive Effects
A16435	Acute Toxicity

A16436	Mutagenicity
A16437	Acute Toxicity
A16438	Mutagenicity
A16439	Mutagenicity
A16440	Non-toxicity
A16441	Acute Toxicity
A16442	Acute Toxicity
A16443	Acute Toxicity
A16444	Acute Toxicity
A16445	Mutagenicity, Tumorigenicity
A16446	Acute Toxicity
A16447	Tumorigenicity, Reproductive Effects, Multiple Dose Effects
A16448	Acute Toxicity
A16449	Reproductive Effects, Multiple Dose Effects
A16450	Acute Toxicity, Mutagenicity, Multiple Dose Effects
A16451	Reproductive Effects
A16452	Acute Toxicity, Mutagenicity, Reproductive Effects, Multiple Dose Effects
A16453	Acute Toxicity
A16454	Acute Toxicity
A16455	Reproductive Effects
A16456	Reproductive Effects
A16457	Acute Toxicity
A16458	Acute Toxicity, Multiple Dose Effects
A16459	Multiple Dose Effects
A16460	Acute Toxicity
A16461	Acute Toxicity
A16462	Acute Toxicity, Multiple Dose Effects
A16463	Acute Toxicity
A16464	Acute Toxicity
A16465	Acute Toxicity
A16466	Multiple Dose Effects
A16467	Acute Toxicity, Multiple Dose Effects
A16468	Acute Toxicity
A16469	Acute Toxicity
A16470	Mutagenicity
A16471	Reproductive Effects
A16472	Acute Toxicity, Multiple Dose Effects
A16473	Multiple Dose Effects
A16474	Acute Toxicity
A16475	Acute Toxicity
A16476	Acute Toxicity
A16477	Acute Toxicity, Multiple Dose Effects
A16478	Acute Toxicity, Mutagenicity, Reproductive Effects, Multiple Dose Effects
A16479	Acute Toxicity
A16480	Tumorigenicity
A16481	Reproductive Effects

A16482	Acute Toxicity
A16483	Acute Toxicity
A16484	Acute Toxicity
A16485	Acute Toxicity
A16486	Tumorigenicity, Reproductive Effects
A16487	Acute Toxicity
A16488	Acute Toxicity
A16489	Mutagenicity
A16490	Acute Toxicity
A16491	Acute Toxicity
A16492	Acute Toxicity
A16493	Tumorigenicity
A16494	Mutagenicity
A16495	Tumorigenicity
A16496	Acute Toxicity
A16497	Multiple Dose Effects
A16498	Acute Toxicity
A16499	Acute Toxicity
A16500	Acute Toxicity, Multiple Dose Effects
A16501	Acute Toxicity
A16502	Tumorigenicity
A16503	Mutagenicity
A16504	Multiple Dose Effects
A16505	Reproductive Effects
A16506	Reproductive Effects
A16507	Multiple Dose Effects
A16508	Acute Toxicity
A16509	Multiple Dose Effects
A16510	Acute Toxicity
A16511	Mutagenicity, Tumorigenicity
A16512	Mutagenicity
A16513	Acute Toxicity, Mutagenicity
A16514	Mutagenicity
A16515	Mutagenicity
A16516	Mutagenicity
A16517	Mutagenicity
A16518	Mutagenicity
A16519	Mutagenicity
A16520	Tumorigenicity
A16521	Tumorigenicity
A16522	Mutagenicity
A16523	Mutagenicity
A16524	Mutagenicity
A16525	Mutagenicity
A16526	Multiple Dose Effects
A16527	Acute Toxicity, Mutagenicity

A16528	Acute Toxicity, Multiple Dose Effects
A16529	Acute Toxicity, Mutagenicity, Reproductive Effects, Multiple Dose Effects
A16530	Reproductive Effects
A16531	Acute Toxicity
A16532	Multiple Dose Effects
A16533	Acute Toxicity, Multiple Dose Effects
A16534	Tumorigenicity
A16535	Mutagenicity
A16536	Acute Toxicity
A16537	Acute Toxicity
A16538	Acute Toxicity
A16539	Acute Toxicity
A16540	Acute Toxicity
A16541	Acute Toxicity
A16542	Acute Toxicity
A16543	Mutagenicity
A16544	Acute Toxicity
A16545	Skin and Eye Irritation
A16546	Multiple Dose Effects
A16547	Non-toxicity
A16548	Acute Toxicity
A16549	Acute Toxicity
A16550	Acute Toxicity
A16551	Acute Toxicity
A16552	Mutagenicity, Multiple Dose Effects
A16553	Multiple Dose Effects
A16554	Non-toxicity
A16555	Multiple Dose Effects
A16556	Acute Toxicity
A16557	Non-toxicity
A16558	Multiple Dose Effects
A16559	Multiple Dose Effects
A16560	Acute Toxicity, Reproductive Effects
A16561	Mutagenicity
A16562	Mutagenicity, Tumorigenicity
A16563	Reproductive Effects
A16564	Non-toxicity
A16565	Acute Toxicity, Multiple Dose Effects
A16566	Multiple Dose Effects
A16567	Acute Toxicity, Multiple Dose Effects
A16568	Acute Toxicity
A16569	Acute Toxicity
A16570	Acute Toxicity
A16571	Acute Toxicity
A16572	Acute Toxicity
A16573	Acute Toxicity

A16574	Acute Toxicity
A16575	Acute Toxicity
A16576	Acute Toxicity
A16577	Acute Toxicity
A16578	Acute Toxicity
A16579	Multiple Dose Effects
A16580	Acute Toxicity
A16581	Acute Toxicity
A16582	Multiple Dose Effects
A16583	Acute Toxicity, Multiple Dose Effects
A16584	Tumorigenicity
A16585	Acute Toxicity
A16586	Acute Toxicity
A16587	Acute Toxicity
A16588	Acute Toxicity
A16589	Acute Toxicity, Multiple Dose Effects
A16590	Acute Toxicity
A16591	Acute Toxicity
A16592	Non-toxicity
A16593	Non-toxicity
A16594	Non-toxicity
A16595	Mutagenicity
A16596	Acute Toxicity, Multiple Dose Effects
A16597	Reproductive Effects, Multiple Dose Effects
A16598	Acute Toxicity
A16599	Acute Toxicity
A16600	Acute Toxicity
A16601	Acute Toxicity
A16602	Mutagenicity
A16603	Acute Toxicity, Multiple Dose Effects
A16604	Acute Toxicity
A16605	Mutagenicity
A16606	Acute Toxicity, Multiple Dose Effects
A16607	Tumorigenicity, Multiple Dose Effects
A16608	Acute Toxicity, Multiple Dose Effects
A16609	Tumorigenicity
A16610	Acute Toxicity
A16611	Skin and Eye Irritation, Multiple Dose Effects
A16612	Acute Toxicity, Reproductive Effects, Multiple Dose Effects
A16613	Acute Toxicity, Skin and Eye Irritation
A16614	Tumorigenicity
A16615	Acute Toxicity
A16616	Mutagenicity
A16617	Reproductive Effects, Multiple Dose Effects
A16618	Acute Toxicity
A16619	Acute Toxicity

A16620	Acute Toxicity
A16621	Non-toxicity
A16622	Acute Toxicity
A16623	Multiple Dose Effects
A16624	Acute Toxicity
A16625	Non-toxicity
A16626	Acute Toxicity, Mutagenicity, Tumorigenicity, Reproductive Effects,
A16627	Acute Toxicity, Multiple Dose Effects
A16628	Acute Toxicity
A16629	Acute Toxicity
A16630	Acute Toxicity
A16631	Acute Toxicity, Multiple Dose Effects
A16632	Acute Toxicity
A16633	Acute Toxicity, Multiple Dose Effects
A16634	Skin and Eye Irritation
A16635	Acute Toxicity, Mutagenicity, Tumorigenicity, Reproductive Effects,
A16636	Acute Toxicity, Mutagenicity, Tumorigenicity, Skin and Eye Irritation,
A16637	Acute Toxicity
A16638	Acute Toxicity
A16639	Non-toxicity
A16640	Non-toxicity
A16641	Non-toxicity
A16642	Acute Toxicity, Mutagenicity
A16643	Acute Toxicity, Mutagenicity
A16644	Acute Toxicity, Mutagenicity
A16645	Acute Toxicity
A16646	Acute Toxicity, Reproductive Effects, Multiple Dose Effects
A16647	Acute Toxicity
A16648	Acute Toxicity
A16649	Acute Toxicity, Mutagenicity, Reproductive Effects, Multiple Dose Effects
A16650	Acute Toxicity, Multiple Dose Effects
A16651	Acute Toxicity, Reproductive Effects
A16652	Reproductive Effects
A16653	Acute Toxicity
A16654	Acute Toxicity
A16655	Acute Toxicity, Reproductive Effects, Multiple Dose Effects
A16656	Reproductive Effects
A16657	Acute Toxicity, Mutagenicity
A16658	Acute Toxicity
A16659	Acute Toxicity
A16660	Acute Toxicity, Multiple Dose Effects
A16661	Acute Toxicity
A16662	Acute Toxicity
A16663	Non-toxicity
A16664	Acute Toxicity, Multiple Dose Effects
A16665	Acute Toxicity

A16666	Acute Toxicity
A16667	Acute Toxicity, Mutagenicity
A16668	Acute Toxicity, Multiple Dose Effects
A16669	Acute Toxicity, Tumorigenicity, Multiple Dose Effects
A16670	Mutagenicity
A16671	Acute Toxicity
A16672	Mutagenicity
A16673	Acute Toxicity
A16674	Acute Toxicity
A16675	Acute Toxicity, Multiple Dose Effects
A16676	Multiple Dose Effects
A16677	Acute Toxicity, Multiple Dose Effects
A16678	Multiple Dose Effects
A16679	Acute Toxicity
A16680	Acute Toxicity
A16681	Multiple Dose Effects
A16682	Acute Toxicity
A16683	Acute Toxicity
A16684	Acute Toxicity, Multiple Dose Effects
A16685	Acute Toxicity
A16686	Acute Toxicity
A16687	Acute Toxicity
A16688	Acute Toxicity
A16689	Multiple Dose Effects
A16690	Non-toxicity
A16691	Multiple Dose Effects
A16692	Non-toxicity
A16693	Acute Toxicity, Multiple Dose Effects
A16694	Multiple Dose Effects
A16695	Multiple Dose Effects
A16696	Acute Toxicity, Multiple Dose Effects
A16697	Acute Toxicity
A16698	Acute Toxicity
A16699	Acute Toxicity
A16700	Acute Toxicity
A16701	Non-toxicity
A16702	Acute Toxicity, Multiple Dose Effects
A16703	Non-toxicity
A16704	Acute Toxicity
A16705	Acute Toxicity, Reproductive Effects, Multiple Dose Effects
A16706	Acute Toxicity
A16707	Multiple Dose Effects
A16708	Multiple Dose Effects
A16709	Acute Toxicity, Multiple Dose Effects
A16710	Multiple Dose Effects
A16711	Acute Toxicity, Multiple Dose Effects

A16712	Multiple Dose Effects
A16713	Multiple Dose Effects
A16714	Multiple Dose Effects
A16715	Multiple Dose Effects
A16716	Mutagenicity
A16717	Acute Toxicity
A16718	Mutagenicity
A16719	Mutagenicity
A16720	Acute Toxicity
A16721	Mutagenicity
A16722	Mutagenicity
A16723	Acute Toxicity, Mutagenicity, Tumorigenicity
A16724	Acute Toxicity
A16725	Acute Toxicity
A16726	Acute Toxicity, Reproductive Effects
A16727	Acute Toxicity
A16728	Acute Toxicity
A16729	Acute Toxicity
A16730	Acute Toxicity, Tumorigenicity
A16731	Acute Toxicity, Mutagenicity
A16732	Acute Toxicity, Mutagenicity
A16733	Acute Toxicity
A16734	Acute Toxicity
A16735	Acute Toxicity
A16736	Acute Toxicity, Mutagenicity, Tumorigenicity
A16737	Acute Toxicity
A16738	Acute Toxicity, Mutagenicity, Multiple Dose Effects
A16739	Acute Toxicity, Multiple Dose Effects
A16740	Mutagenicity
A16741	Acute Toxicity, Mutagenicity, Tumorigenicity, Reproductive Effects,
A16742	Acute Toxicity, Reproductive Effects
A16743	Acute Toxicity, Mutagenicity, Skin and Eye Irritation, Multiple Dose Effects
A16744	Acute Toxicity, Multiple Dose Effects
A16745	Acute Toxicity, Mutagenicity, Tumorigenicity
A16746	Acute Toxicity
A16747	Acute Toxicity
A16748	Acute Toxicity
A16749	Acute Toxicity
A16750	Acute Toxicity
A16751	Skin and Eye Irritation
A16752	Acute Toxicity, Tumorigenicity
A16753	Acute Toxicity
A16754	Non-toxicity
A16755	Acute Toxicity
A16756	Acute Toxicity, Multiple Dose Effects
A16757	Acute Toxicity

A16758	Reproductive Effects
A16759	Acute Toxicity
A16760	Acute Toxicity
A16761	Mutagenicity
A16762	Multiple Dose Effects
A16763	Acute Toxicity, Mutagenicity, Multiple Dose Effects
A16764	Acute Toxicity, Multiple Dose Effects
A16765	Acute Toxicity
A16766	Acute Toxicity, Reproductive Effects, Multiple Dose Effects
A16767	Acute Toxicity, Multiple Dose Effects
A16768	Acute Toxicity
A16769	Acute Toxicity, Mutagenicity, Reproductive Effects
A16770	Acute Toxicity
A16771	Mutagenicity
A16772	Acute Toxicity
A16773	Acute Toxicity
A16774	Acute Toxicity, Multiple Dose Effects
A16775	Tumorigenicity
A16776	Mutagenicity, Multiple Dose Effects
A16777	Skin and Eye Irritation
A16778	Acute Toxicity
A16779	Mutagenicity
A16780	Acute Toxicity
A16781	Acute Toxicity
A16782	Acute Toxicity
A16783	Acute Toxicity, Multiple Dose Effects
A16784	Acute Toxicity
A16785	Acute Toxicity
A16786	Acute Toxicity
A16787	Acute Toxicity
A16788	Multiple Dose Effects
A16789	Acute Toxicity
A16790	Acute Toxicity
A16791	Acute Toxicity
A16792	Acute Toxicity, Tumorigenicity, Multiple Dose Effects
A16793	Acute Toxicity, Multiple Dose Effects
A16794	Acute Toxicity
A16795	Acute Toxicity
A16796	Acute Toxicity
A16797	Multiple Dose Effects
A16798	Acute Toxicity
A16799	Multiple Dose Effects
A16800	Multiple Dose Effects
A16801	Acute Toxicity, Multiple Dose Effects
A16802	Multiple Dose Effects
A16803	Acute Toxicity

A16804	Multiple Dose Effects
A16805	Acute Toxicity, Multiple Dose Effects
A16806	Multiple Dose Effects
A16807	Acute Toxicity, Mutagenicity
A16808	Acute Toxicity, Multiple Dose Effects
A16809	Acute Toxicity
A16810	Multiple Dose Effects
A16811	Acute Toxicity
A16812	Non-toxicity
A16813	Acute Toxicity
A16814	Acute Toxicity
A16815	Multiple Dose Effects
A16816	Acute Toxicity
A16817	Multiple Dose Effects
A16818	Acute Toxicity
A16819	Acute Toxicity
A16820	Acute Toxicity, Multiple Dose Effects
A16821	Reproductive Effects
A16822	Acute Toxicity, Reproductive Effects, Multiple Dose Effects
A16823	Reproductive Effects
A16824	Acute Toxicity, Multiple Dose Effects
A16825	Multiple Dose Effects
A16826	Acute Toxicity
A16827	Acute Toxicity, Multiple Dose Effects
A16828	Acute Toxicity, Multiple Dose Effects
A16829	Multiple Dose Effects
A16830	Acute Toxicity, Multiple Dose Effects
A16831	Acute Toxicity, Multiple Dose Effects
A16832	Multiple Dose Effects
A16833	Acute Toxicity
A16834	Acute Toxicity
A16835	Non-toxicity
A16836	Reproductive Effects, Multiple Dose Effects
A16837	Acute Toxicity
A16838	Acute Toxicity, Multiple Dose Effects
A16839	Multiple Dose Effects
A16840	Acute Toxicity, Multiple Dose Effects
A16841	Acute Toxicity, Multiple Dose Effects
A16842	Acute Toxicity
A16843	Skin and Eye Irritation
A16844	Acute Toxicity, Multiple Dose Effects
A16845	Acute Toxicity
A16846	Multiple Dose Effects
A16847	Acute Toxicity
A16848	Multiple Dose Effects
A16849	Acute Toxicity, Multiple Dose Effects

A16850	Non-toxicity
A16851	Acute Toxicity
A16852	Multiple Dose Effects
A16853	Multiple Dose Effects
A16854	Acute Toxicity, Multiple Dose Effects
A16855	Acute Toxicity
A16856	Acute Toxicity
A16857	Non-toxicity
A16858	Acute Toxicity
A16859	Acute Toxicity, Multiple Dose Effects
A16860	Acute Toxicity, Multiple Dose Effects
A16861	Acute Toxicity
A16862	Acute Toxicity, Multiple Dose Effects
A16863	Non-toxicity
A16864	Multiple Dose Effects
A16865	Acute Toxicity
A16866	Non-toxicity
A16867	Multiple Dose Effects
A16868	Acute Toxicity, Multiple Dose Effects
A16869	Acute Toxicity, Multiple Dose Effects
A16870	Multiple Dose Effects
A16871	Acute Toxicity, Multiple Dose Effects
A16872	Multiple Dose Effects
A16873	Acute Toxicity
A16874	Acute Toxicity, Multiple Dose Effects
A16875	Multiple Dose Effects
A16876	Acute Toxicity, Multiple Dose Effects
A16877	Acute Toxicity
A16878	Multiple Dose Effects
A16879	Acute Toxicity, Multiple Dose Effects
A16880	Multiple Dose Effects
A16881	Multiple Dose Effects
A16882	Multiple Dose Effects
A16883	Multiple Dose Effects
A16884	Multiple Dose Effects
A16885	Acute Toxicity, Multiple Dose Effects
A16886	Acute Toxicity, Multiple Dose Effects
A16887	Acute Toxicity
A16888	Acute Toxicity
A16889	Acute Toxicity
A16890	Non-toxicity
A16891	Non-toxicity
A16892	Multiple Dose Effects
A16893	Acute Toxicity
A16894	Multiple Dose Effects
A16895	Acute Toxicity

A16896	Acute Toxicity, Multiple Dose Effects
A16897	Acute Toxicity
A16898	Multiple Dose Effects
A16899	Acute Toxicity, Multiple Dose Effects
A16900	Acute Toxicity
A16901	Multiple Dose Effects
A16902	Acute Toxicity
A16903	Acute Toxicity
A16904	Tumorigenicity, Multiple Dose Effects
A16905	Multiple Dose Effects
A16906	Multiple Dose Effects
A16907	Multiple Dose Effects
A16908	Acute Toxicity
A16909	Acute Toxicity
A16910	Non-toxicity
A16911	Acute Toxicity
A16912	Acute Toxicity
A16913	Acute Toxicity, Multiple Dose Effects
A16914	Acute Toxicity
A16915	Acute Toxicity, Multiple Dose Effects
A16916	Non-toxicity
A16917	Acute Toxicity, Multiple Dose Effects
A16918	Multiple Dose Effects
A16919	Skin and Eye Irritation
A16920	Acute Toxicity
A16921	Mutagenicity, Skin and Eye Irritation
A16922	Acute Toxicity
A16923	Acute Toxicity
A16924	Multiple Dose Effects
A16925	Acute Toxicity, Multiple Dose Effects
A16926	Acute Toxicity
A16927	Reproductive Effects
A16928	Multiple Dose Effects
A16929	Multiple Dose Effects
A16930	Acute Toxicity
A16931	Acute Toxicity
A16932	Acute Toxicity
A16933	Acute Toxicity
A16934	Acute Toxicity, Multiple Dose Effects
A16935	Skin and Eye Irritation
A16936	Acute Toxicity, Multiple Dose Effects
A16937	Acute Toxicity
A16938	Acute Toxicity, Mutagenicity, Multiple Dose Effects
A16939	Acute Toxicity
A16940	Reproductive Effects
A16941	Acute Toxicity

A16942	Acute Toxicity
A16943	Non-toxicity
A16944	Multiple Dose Effects
A16945	Acute Toxicity
A16946	Skin and Eye Irritation
A16947	Acute Toxicity
A16948	Acute Toxicity
A16949	Acute Toxicity
A16950	Acute Toxicity
A16951	Mutagenicity, Tumorigenicity, Multiple Dose Effects
A16952	Acute Toxicity
A16953	Acute Toxicity
A16954	Multiple Dose Effects
A16955	Multiple Dose Effects
A16956	Multiple Dose Effects
A16957	Multiple Dose Effects
A16958	Acute Toxicity
A16959	Acute Toxicity, Tumorigenicity, Reproductive Effects
A16960	Acute Toxicity
A16961	Acute Toxicity
A16962	Acute Toxicity
A16963	Acute Toxicity, Mutagenicity
A16964	Acute Toxicity
A16965	Acute Toxicity
A16966	Acute Toxicity
A16967	Acute Toxicity
A16968	Acute Toxicity, Reproductive Effects, Multiple Dose Effects
A16969	Multiple Dose Effects
A16970	Acute Toxicity
A16971	Multiple Dose Effects
A16972	Acute Toxicity, Multiple Dose Effects
A16973	Acute Toxicity, Multiple Dose Effects
A16974	Multiple Dose Effects
A16975	Acute Toxicity
A16976	Acute Toxicity
A16977	Reproductive Effects
A16978	Acute Toxicity
A16979	Acute Toxicity, Multiple Dose Effects
A16980	Acute Toxicity
A16981	Mutagenicity
A16982	Reproductive Effects
A16983	Tumorigenicity
A16984	Acute Toxicity, Reproductive Effects, Multiple Dose Effects
A16985	Mutagenicity
A16986	Acute Toxicity
A16987	Acute Toxicity, Mutagenicity, Multiple Dose Effects

A16988	Mutagenicity
A16989	Non-toxicity
A16990	Non-toxicity
A16991	Non-toxicity
A16992	Acute Toxicity, Multiple Dose Effects
A16993	Non-toxicity
A16994	Reproductive Effects
A16995	Non-toxicity
A16996	Multiple Dose Effects
A16997	Acute Toxicity
A16998	Acute Toxicity
A16999	Acute Toxicity, Reproductive Effects, Multiple Dose Effects
A17000	Acute Toxicity
A17001	Mutagenicity
A17002	Acute Toxicity
A17003	Acute Toxicity, Reproductive Effects, Multiple Dose Effects
A17004	Acute Toxicity, Mutagenicity
A17005	Reproductive Effects
A17006	Reproductive Effects, Multiple Dose Effects
A17007	Multiple Dose Effects
A17008	Multiple Dose Effects
A17009	Acute Toxicity, Mutagenicity, Tumorigenicity, Reproductive Effects
A17010	Acute Toxicity
A17011	Multiple Dose Effects
A17012	Acute Toxicity, Multiple Dose Effects
A17013	Multiple Dose Effects
A17014	Multiple Dose Effects
A17015	Non-toxicity
A17016	Acute Toxicity
A17017	Acute Toxicity
A17018	Multiple Dose Effects
A17019	Acute Toxicity
A17020	Acute Toxicity, Mutagenicity
A17021	Acute Toxicity, Multiple Dose Effects
A17022	Acute Toxicity
A17023	Acute Toxicity
A17024	Acute Toxicity
A17025	Multiple Dose Effects
A17026	Acute Toxicity
A17027	Acute Toxicity
A17028	Acute Toxicity
A17029	Acute Toxicity, Multiple Dose Effects
A17030	Acute Toxicity, Multiple Dose Effects
A17031	Acute Toxicity
A17032	Non-toxicity
A17033	Multiple Dose Effects

A17034	Multiple Dose Effects
A17035	Acute Toxicity, Multiple Dose Effects
A17036	Acute Toxicity, Reproductive Effects, Multiple Dose Effects
A17037	Acute Toxicity, Reproductive Effects
A17038	Multiple Dose Effects
A17039	Multiple Dose Effects
A17040	Acute Toxicity, Reproductive Effects, Multiple Dose Effects
A17041	Acute Toxicity, Reproductive Effects, Multiple Dose Effects
A17042	Acute Toxicity
A17043	Reproductive Effects, Multiple Dose Effects
A17044	Acute Toxicity
A17045	Multiple Dose Effects
A17046	Multiple Dose Effects
A17047	Multiple Dose Effects
A17048	Acute Toxicity, Multiple Dose Effects
A17049	Acute Toxicity
A17050	Acute Toxicity
A17051	Acute Toxicity, Reproductive Effects, Multiple Dose Effects
A17052	Tumorigenicity
A17053	Tumorigenicity, Multiple Dose Effects
A17054	Acute Toxicity
A17055	Acute Toxicity, Reproductive Effects, Multiple Dose Effects
A17056	Acute Toxicity
A17057	Multiple Dose Effects
A17058	Multiple Dose Effects
A17059	Non-toxicity
A17060	Non-toxicity
A17061	Mutagenicity, Tumorigenicity, Reproductive Effects
A17062	Reproductive Effects
A17063	Acute Toxicity, Mutagenicity
A17064	Acute Toxicity
A17065	Multiple Dose Effects
A17066	Acute Toxicity
A17067	Acute Toxicity
A17068	Acute Toxicity
A17069	Acute Toxicity
A17070	Acute Toxicity
A17071	Acute Toxicity, Multiple Dose Effects
A17072	Acute Toxicity
A17073	Acute Toxicity
A17074	Mutagenicity
A17075	Acute Toxicity
A17076	Acute Toxicity
A17077	Acute Toxicity
A17078	Acute Toxicity, Mutagenicity, Tumorigenicity, Reproductive Effects,
A17079	Acute Toxicity, Reproductive Effects, Multiple Dose Effects

A17080	Mutagenicity, Tumorigenicity
A17081	Acute Toxicity
A17082	Acute Toxicity
A17083	Mutagenicity, Tumorigenicity
A17084	Acute Toxicity
A17085	Acute Toxicity, Mutagenicity, Tumorigenicity
A17086	Acute Toxicity, Mutagenicity
A17087	Acute Toxicity
A17088	Acute Toxicity
A17089	Acute Toxicity, Reproductive Effects, Multiple Dose Effects
A17090	Mutagenicity
A17091	Mutagenicity, Reproductive Effects
A17092	Acute Toxicity, Reproductive Effects, Multiple Dose Effects
A17093	Non-toxicity
A17094	Non-toxicity
A17095	Acute Toxicity
A17096	Mutagenicity
A17097	Multiple Dose Effects
A17098	Acute Toxicity
A17099	Acute Toxicity, Mutagenicity
A17100	Acute Toxicity, Mutagenicity
A17101	Acute Toxicity, Mutagenicity
A17102	Non-toxicity
A17103	Acute Toxicity
A17104	Acute Toxicity
A17105	Acute Toxicity
A17106	Acute Toxicity
A17107	Multiple Dose Effects
A17108	Acute Toxicity
A17109	Non-toxicity
A17110	Acute Toxicity
A17111	Acute Toxicity, Multiple Dose Effects
A17112	Acute Toxicity, Multiple Dose Effects
A17113	Tumorigenicity, Multiple Dose Effects
A17114	Acute Toxicity
A17115	Acute Toxicity, Reproductive Effects
A17116	Multiple Dose Effects
A17117	Acute Toxicity
A17118	Multiple Dose Effects
A17119	Acute Toxicity
A17120	Multiple Dose Effects
A17121	Acute Toxicity, Reproductive Effects, Multiple Dose Effects
A17122	Acute Toxicity, Tumorigenicity, Reproductive Effects, Multiple Dose Effects
A17123	Acute Toxicity, Mutagenicity, Reproductive Effects, Multiple Dose Effects
A17124	Acute Toxicity, Mutagenicity, Tumorigenicity, Skin and Eye Irritation,
A17125	Acute Toxicity, Tumorigenicity, Multiple Dose Effects

A17126	Acute Toxicity, Reproductive Effects
A17127	Acute Toxicity
A17128	Acute Toxicity, Multiple Dose Effects
A17129	Reproductive Effects
A17130	Reproductive Effects, Multiple Dose Effects
A17131	Acute Toxicity
A17132	Acute Toxicity
A17133	Acute Toxicity
A17134	Acute Toxicity
A17135	Acute Toxicity
A17136	Acute Toxicity
A17137	Acute Toxicity
A17138	Acute Toxicity
A17139	Acute Toxicity
A17140	Acute Toxicity
A17141	Acute Toxicity
A17142	Acute Toxicity
A17143	Acute Toxicity, Multiple Dose Effects
A17144	Acute Toxicity
A17145	Acute Toxicity
A17146	Acute Toxicity
A17147	Skin and Eye Irritation
A17148	Acute Toxicity
A17149	Acute Toxicity
A17150	Acute Toxicity
A17151	Mutagenicity
A17152	Reproductive Effects
A17153	Acute Toxicity
A17154	Acute Toxicity
A17155	Mutagenicity, Tumorigenicity
A17156	Acute Toxicity
A17157	Mutagenicity
A17158	Acute Toxicity, Multiple Dose Effects
A17159	Acute Toxicity
A17160	Non-toxicity
A17161	Acute Toxicity, Multiple Dose Effects
A17162	Non-toxicity
A17163	Acute Toxicity
A17164	Non-toxicity
A17165	Non-toxicity
A17166	Skin and Eye Irritation
A17167	Skin and Eye Irritation
A17168	Acute Toxicity, Mutagenicity
A17169	Reproductive Effects
A17170	Acute Toxicity
A17171	Acute Toxicity, Multiple Dose Effects

A17172	Acute Toxicity
A17173	Acute Toxicity, Multiple Dose Effects
A17174	Mutagenicity
A17175	Acute Toxicity, Mutagenicity
A17176	Acute Toxicity
A17177	Acute Toxicity
A17178	Acute Toxicity
A17179	Acute Toxicity
A17180	Acute Toxicity
A17181	Acute Toxicity
A17182	Multiple Dose Effects
A17183	Mutagenicity
A17184	Multiple Dose Effects
A17185	Acute Toxicity
A17186	Acute Toxicity
A17187	Non-toxicity
A17188	Non-toxicity
A17189	Non-toxicity
A17190	Multiple Dose Effects
A17191	Acute Toxicity
A17192	Acute Toxicity
A17193	Acute Toxicity
A17194	Acute Toxicity
A17195	Non-toxicity
A17196	Tumorigenicity
A17197	Acute Toxicity
A17198	Mutagenicity, Tumorigenicity
A17199	Acute Toxicity
A17200	Acute Toxicity
A17201	Acute Toxicity
A17202	Acute Toxicity
A17203	Acute Toxicity
A17204	Acute Toxicity
A17205	Acute Toxicity
A17206	Acute Toxicity
A17207	Acute Toxicity
A17208	Acute Toxicity
A17209	Mutagenicity
A17210	Multiple Dose Effects
A17211	Acute Toxicity, Multiple Dose Effects
A17212	Non-toxicity
A17213	Acute Toxicity
A17214	Non-toxicity
A17215	Non-toxicity
A17216	Non-toxicity
A17217	Non-toxicity

A17218	Multiple Dose Effects
A17219	Acute Toxicity
A17220	Multiple Dose Effects
A17221	Acute Toxicity
A17222	Non-toxicity
A17223	Acute Toxicity, Mutagenicity
A17224	Non-toxicity
A17225	Acute Toxicity
A17226	Acute Toxicity
A17227	Acute Toxicity
A17228	Acute Toxicity
A17229	Acute Toxicity
A17230	Acute Toxicity
A17231	Acute Toxicity
A17232	Non-toxicity
A17233	Non-toxicity
