# Supplementary material for: Predicting Chemical Toxicity Effects Based on Chemical-Chemical Interactions
Source: PLoS One. 2013 Feb 15;8(2):e56517. doi: 10.1371/journal.pone.0056517 (PMC3574107; doi:10.1371/journal.pone.0056517)
Supplement: Table S3 — List of SAs (Benigni) and examples matching SAs in our dataset. (PDF) [file pone.0056517.s003.pdf]

Table S3: SAs (Benigni) and examples matching SAs in our dataset

SAs	Examples
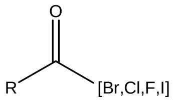<br>acyl halides	dimethylcarbamyl chloride (CID000006598)<br>benzoyl chloride (CID000007412)
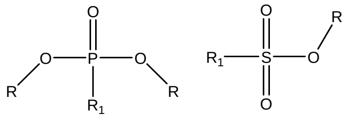<br>alkyl (C<5) or benzyl ester of sulphonic or phosphonic acid	trichlorfon (CID000005853)<br>ethyl p-toluenesulfonate (CID000006638)
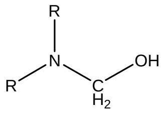<br>*N*-methylol derivatives	*N*-methylolacrylamide (CID000013543)
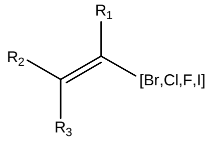<br>monohaloalkene	vinyl fluoride (CID000006339)<br>1-chloro-2-methylpropene (CID000010555)
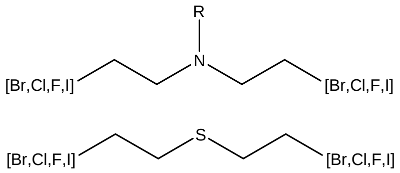<br>S or N mustard	2,2',2''-trichlorotriethylamine (CID000005561)<br>sulfur mustard (CID000010461)
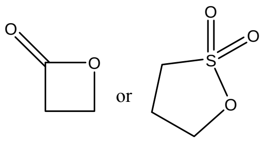<br>propiolactones or propiosultones	propiolactone (CID000002365)<br>1,3-propane sultone (CID000014264)
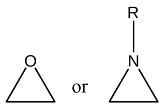<br>epoxides and aziridines	3,4-epoxy-1-butene (CID000013586)<br>aziridine (CID000009033)
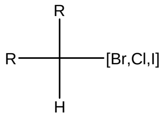<br>aliphatic halogens	2-bromobutane (CID000006554)<br>2-iodobutane (CID000010559)
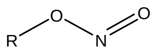<br>alkyl nitrite	isobutyl nitrite (CID000010958)

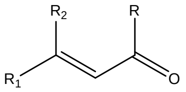 α,β unsaturated carbonyls	acrylamide (CID000006579)<br>2-butenal (CID000447466)
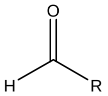 simple aldehyde	acetaldehyde (CID000000177)<br>bromoacetaldehyde (CID000105131)
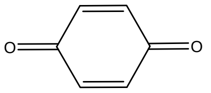 quinones	phenylbenzoquinone (CID000009688)<br>chloranil (CID000008371)
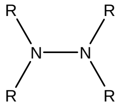 hydrazine	1,1-dibutylhydrazine (CID000023902)<br>hydrazine (CID000009321)
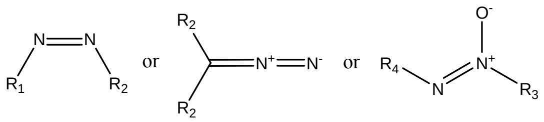 aliphatic azo and azoxy	diazomethane (CID000009550)<br>methylazoxymethanol acetate (CID005964719)
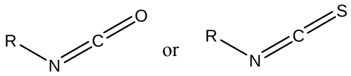 isocyanate and isothiocyanate groups	toluene 2,4-diisocyanate (CID000011443)<br>benzyl isothiocyanate (CID000002346)
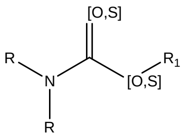 alkyl carbamate and thiocarbamate	ethyl butylcarbamate (CID000011577)<br>pebulate (CID000014215)
polycyclic aromatic hydrocarbons	dibenzo(a,i)pyrene (CID000009106)<br>dibenzo(a,h)pyrene (CID000009108)
heterocyclic polycyclic aromatic hydrocarbons	benzo(f)quinoline (CID000006796)<br>benzo(b)naphtho(2,1-d)thiophene (CID000009198)
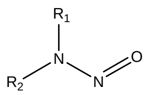 alkyl and aryl *N*-nitroso groups	2-methylnitrosopiperidine (CID000023677)<br>*N*-nitroso-*N*-butyl-*N*-propylamine (CID000032965)
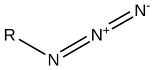 triazene groups	zidovudine (CID000035370)

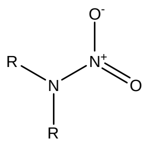<br>aliphatic *N*-nitro group	dimethylnitramine (CID000020120)<br>*N*-nitrodiethylamine (CID000023505)
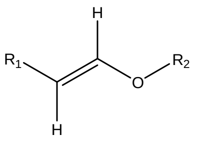<br>α,β unsaturated aliphatic alkoxy group	sterigmatocystin (CID005284457)<br>aflatoxin G1 (CID000014421)
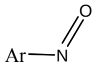<br>aromatic nitroso group	2-nitrosofluorene (CID000017271)<br>3,2'-dimethyl-4-nitrosobiphenyl (CID000051168)
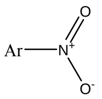<br>nitro-aromatic	4,4'-dinitrobiphenyl (CID000015216)<br>4-nitrophenyl (CID000119211)
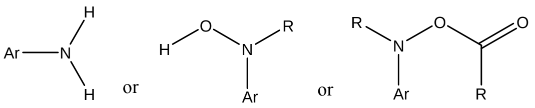<br>primary aromatic amine, hydroxyl amine and its derived esters<br>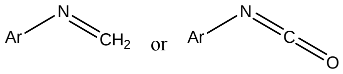<br>or amine generating group	4-aminophenyl (CID0000181201)<br>*N*-hydroxy-4-aminobiphenyl (CID000081261)<br>*N*-acetoxy-4-acetylaminobiphenyl (CID000091584)<br>toluene 2,4-diisocyanate (CID000011443)<br>DADI (CID000007069)
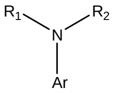<br>aromatic mono- and dialkylamine	*N,N,N',N'*-tetramethyl-4,4'-methylenedianiline (CID000007567)<br>4-dimethylaminostilbene (CID000640024)
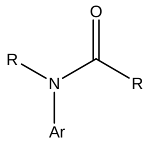<br>aromatic *N*-alkyl amine	*N,N'*-diacetylbenzidine (CID000011942)<br>4-acetylaminobiphenyl (CID000019998)
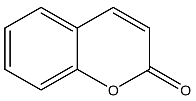<br>coumarins and Furocoumarins	6-methylcoumarin (CID000007092)<br>coumarin (CID000000323)
