# Supplementary material for: Predicting Chemical Toxicity Effects Based on Chemical-Chemical Interactions
Source: PLoS One. 2013 Feb 15;8(2):e56517. doi: 10.1371/journal.pone.0056517 (PMC3574107; doi:10.1371/journal.pone.0056517)
Supplement: Table S2 — List of 323,432 interaction units used to predict compound toxicity in this study. (PDF) [file pone.0056517.s002.pdf]

Table S2: 323,432 interaction units used to predict compound toxicity, each unit contains two compounds and one confidence score.

Compound #1	Compound #2	Confidence score	Compound #1	Compound #2	Confidence score
A00001	A00009	374	A00001	A00010	374
A00001	A00026	561	A00001	A00041	489
A00001	A00042	490	A00001	A00046	263
A00001	A00047	558	A00001	A00058	465
A00001	A00064	154	A00001	A00073	631
A00001	A00074	489	A00001	A00090	618
A00001	A00138	491	A00001	A00162	202
A00001	A00183	559	A00001	A00185	221
A00001	A00198	291	A00001	A00207	383
A00001	A00210	291	A00001	A00214	486
A00001	A00234	240	A00001	A00239	291
A00001	A00240	291	A00001	A00241	291
A00001	A00243	291	A00001	A00254	283
A00001	A00261	291	A00001	A00316	152
A00001	A00371	334	A00001	A00382	63
A00001	A00433	334	A00001	A00600	185
A00001	A00641	550	A00001	A00696	366
A00001	A00706	334	A00001	A00973	334
A00001	A01113	334	A00001	A01192	334
A00001	A01474	260	A00001	A01560	185
A00001	A01596	291	A00001	A01648	334
A00001	A01792	219	A00001	A01914	484
A00001	A01920	291	A00001	A02342	188
A00001	A03446	63	A00001	A03560	334
A00001	A03784	180	A00001	A04012	945
A00001	A04567	165	A00001	A04731	174
A00001	A05029	334	A00001	A05633	488
A00001	A06197	229	A00001	A06200	195
A00001	A07027	177	A00001	A07167	271
A00001	A07311	391	A00001	A07378	334
A00001	A08091	235	A00001	A08118	291
A00001	A09591	334	A00001	A11129	226
A00001	A11929	235	A00001	A12054	358
A00001	A12988	488	A00001	A13198	477
A00001	A13199	374	A00001	A13250	291
A00001	A13511	229	A00001	A13743	465
A00001	A14178	334	A00001	A14198	193
A00001	A14299	291	A00001	A14704	291
A00001	A15347	187	A00001	A15636	241
A00001	A16594	373	A00001	A17088	334
A00002	A00035	160	A00002	A00042	915

A00002	A00052	904	A00002	A00067	899
A00002	A00123	371	A00002	A00140	350
A00002	A00148	353	A00002	A00151	941
A00002	A00152	63	A00002	A00159	63
A00002	A00189	281	A00002	A00222	193
A00002	A00224	902	A00002	A00226	375
A00002	A00381	187	A00002	A00759	208
A00002	A00760	235	A00002	A01189	279
A00002	A01527	904	A00002	A01558	899
A00002	A01588	899	A00002	A01597	191
A00002	A01709	477	A00002	A01844	63
A00002	A02400	346	A00002	A02415	226
A00002	A02487	230	A00002	A02564	146
A00002	A02641	291	A00002	A02737	162
A00002	A02836	160	A00002	A02881	414
A00002	A02904	316	A00002	A02908	162
A00002	A03010	224	A00002	A03338	457
A00002	A03654	230	A00002	A03809	168
A00002	A03965	229	A00002	A04853	265
A00002	A05065	232	A00002	A06149	274
A00002	A06398	408	A00002	A06601	215
A00002	A07187	252	A00002	A08768	401
A00002	A10899	249	A00002	A15230	236
A00003	A00045	483	A00003	A00167	491
A00003	A00210	311	A00003	A00212	490
A00003	A00316	458	A00003	A00338	481
A00003	A00395	375	A00003	A00449	385
A00003	A00471	170	A00003	A00472	480
A00003	A00473	409	A00003	A00477	390
A00003	A00485	435	A00003	A00546	229
A00003	A00562	445	A00003	A00576	179
A00003	A00625	447	A00003	A00733	491
A00003	A00752	480	A00003	A00887	193
A00003	A00981	463	A00003	A01004	232
A00003	A01009	447	A00003	A01018	424
A00003	A01052	488	A00003	A01161	490
A00003	A01212	241	A00003	A01256	163
A00003	A01332	249	A00003	A01363	229
A00003	A01367	229	A00003	A01478	260
A00003	A01557	241	A00003	A01578	213
A00003	A01582	217	A00003	A01602	899
A00003	A01656	259	A00003	A01688	213
A00003	A01694	491	A00003	A01725	601
A00003	A01728	614	A00003	A01743	606
A00003	A01749	162	A00003	A01758	260
A00003	A01790	484	A00003	A01931	491

A00003	A01936	202	A00003	A01972	63
A00003	A02028	480	A00003	A02075	343
A00003	A02096	152	A00003	A02104	152
A00003	A02109	366	A00003	A02116	486
A00003	A02117	165	A00003	A02120	457
A00003	A02148	484	A00003	A02194	238
A00003	A02222	461	A00003	A02332	160
A00003	A02333	490	A00003	A02434	428
A00003	A02436	441	A00003	A02443	491
A00003	A02450	491	A00003	A02469	343
A00003	A02476	491	A00003	A02477	434
A00003	A02492	307	A00003	A02535	401
A00003	A02540	291	A00003	A02673	485
A00003	A02691	400	A00003	A02698	258
A00003	A02727	218	A00003	A02753	243
A00003	A02785	185	A00003	A02885	201
A00003	A02921	174	A00003	A02931	218
A00003	A03028	404	A00003	A03043	188
A00003	A03056	123	A00003	A03104	430
A00003	A03107	216	A00003	A03186	330
A00003	A03202	193	A00003	A03223	484
A00003	A03246	462	A00003	A03417	219
A00003	A03434	232	A00003	A03451	481
A00003	A03709	288	A00003	A03811	238
A00003	A03819	409	A00003	A03926	427
A00003	A03928	238	A00003	A03945	180
A00003	A03968	308	A00003	A04019	259
A00003	A04062	388	A00003	A04075	491
A00003	A04210	363	A00003	A04212	485
A00003	A04220	313	A00003	A04228	427
A00003	A04389	486	A00003	A04442	246
A00003	A04449	483	A00003	A04460	156
A00003	A04520	151	A00003	A04736	174
A00003	A04867	358	A00003	A04879	470
A00003	A05096	152	A00003	A05128	467
A00003	A05155	470	A00003	A05293	291
A00003	A05297	414	A00003	A05355	156
A00003	A05381	223	A00003	A05439	191
A00003	A05838	481	A00003	A05929	291
A00003	A06277	488	A00003	A06281	353
A00003	A06298	491	A00003	A06405	283
A00003	A06660	485	A00003	A06718	154
A00003	A06729	353	A00003	A06731	252
A00003	A06819	475	A00003	A06855	462
A00003	A06953	200	A00003	A07209	187
A00003	A07286	223	A00003	A07391	152

A00003	A07442	435	A00003	A07605	480
A00003	A07613	302	A00003	A08097	350
A00003	A08163	288	A00003	A08690	480
A00003	A08725	238	A00003	A08758	163
A00003	A08857	488	A00003	A08867	485
A00003	A08878	480	A00003	A08951	185
A00003	A09216	238	A00003	A09364	180
A00003	A09452	446	A00003	A09457	238
A00003	A09878	195	A00003	A10004	291
A00003	A10126	398	A00003	A10180	212
A00003	A10407	291	A00003	A10414	187
A00003	A10696	152	A00003	A10752	296
A00003	A10768	193	A00003	A10882	394
A00003	A11068	945	A00003	A11101	441
A00003	A11282	199	A00003	A11300	160
A00003	A11313	196	A00003	A11317	400
A00003	A11454	176	A00003	A11602	187
A00003	A11727	188	A00003	A11900	152
A00003	A11983	945	A00003	A12010	156
A00003	A12033	201	A00003	A12273	213
A00003	A12402	291	A00003	A12464	223
A00003	A13110	223	A00003	A13154	212
A00003	A13212	457	A00003	A13338	154
A00003	A13361	409	A00003	A13369	475
A00003	A14055	152	A00003	A14246	208
A00003	A14281	177	A00003	A14366	434
A00003	A14373	270	A00003	A14377	491
A00003	A14406	249	A00003	A14554	483
A00003	A14570	185	A00003	A14603	180
A00003	A14621	491	A00003	A14634	160
A00003	A14828	441	A00003	A15226	163
A00003	A15241	171	A00003	A15270	152
A00003	A15272	160	A00003	A15282	488
A00003	A15295	416	A00003	A15324	450
A00003	A15395	183	A00003	A15396	340
A00003	A15422	319	A00003	A15495	288
A00003	A15498	251	A00003	A15586	377
A00003	A15605	388	A00003	A15606	486
A00003	A15608	165	A00003	A15875	254
A00003	A15883	259	A00003	A15917	172
A00003	A15956	291	A00003	A16004	156
A00003	A16036	223	A00003	A16477	160
A00003	A16533	168	A00004	A00007	945
A00004	A00008	945	A00004	A00041	337
A00004	A00045	473	A00004	A00052	396
A00004	A00068	899	A00004	A00071	483

A00004	A00142	277	A00004	A00152	491
A00004	A00171	152	A00004	A00190	477
A00004	A00204	404	A00004	A00227	218
A00004	A00264	489	A00004	A00309	317
A00004	A00351	485	A00004	A00408	247
A00004	A00449	257	A00004	A00657	472
A00004	A00659	254	A00004	A00662	400
A00004	A00663	205	A00004	A00669	407
A00004	A00688	252	A00004	A00694	196
A00004	A00736	361	A00004	A00747	452
A00004	A00794	401	A00004	A00820	490
A00004	A00914	226	A00004	A00997	159
A00004	A01005	406	A00004	A01138	218
A00004	A01161	224	A00004	A01400	429
A00004	A01404	388	A00004	A01428	246
A00004	A01460	272	A00004	A01461	475
A00004	A01515	452	A00004	A01551	491
A00004	A01573	488	A00004	A01575	204
A00004	A01668	491	A00004	A01670	486
A00004	A01675	221	A00004	A01676	184
A00004	A01705	491	A00004	A01725	310
A00004	A01726	485	A00004	A01728	333
A00004	A01729	236	A00004	A01730	296
A00004	A01731	364	A00004	A01732	344
A00004	A01736	488	A00004	A01737	491
A00004	A01741	486	A00004	A01743	491
A00004	A01746	396	A00004	A01751	286
A00004	A01753	351	A00004	A01754	213
A00004	A01757	489	A00004	A01758	491
A00004	A01762	124	A00004	A01767	229
A00004	A01769	330	A00004	A01772	180
A00004	A01776	172	A00004	A01787	201
A00004	A01788	110	A00004	A01794	447
A00004	A01802	65	A00004	A01850	182
A00004	A01872	373	A00004	A01874	249
A00004	A01883	490	A00004	A01893	491
A00004	A01894	491	A00004	A01895	266
A00004	A01896	91	A00004	A01899	151
A00004	A01909	491	A00004	A01926	491
A00004	A02006	165	A00004	A02075	402
A00004	A02117	440	A00004	A02167	252
A00004	A02300	462	A00004	A02307	154
A00004	A02315	277	A00004	A02333	170
A00004	A02335	467	A00004	A02338	446
A00004	A02402	277	A00004	A02419	172
A00004	A02435	191	A00004	A02436	187

A00004	A02490	232	A00004	A02491	406
A00004	A02613	249	A00004	A02668	260
A00004	A02687	356	A00004	A02691	491
A00004	A02700	486	A00004	A02701	351
A00004	A02705	223	A00004	A02706	380
A00004	A02707	107	A00004	A02752	368
A00004	A02771	205	A00004	A02785	480
A00004	A02814	412	A00004	A02817	168
A00004	A02836	202	A00004	A02837	423
A00004	A02859	486	A00004	A02874	405
A00004	A02893	205	A00004	A02895	157
A00004	A02906	269	A00004	A02988	162
A00004	A03034	279	A00004	A03225	318
A00004	A03238	457	A00004	A03259	297
A00004	A03268	475	A00004	A03284	185
A00004	A03520	160	A00004	A03585	274
A00004	A03593	249	A00004	A03594	378
A00004	A03611	491	A00004	A03624	249
A00004	A03653	154	A00004	A03866	168
A00004	A03870	205	A00004	A03872	351
A00004	A03890	405	A00004	A03983	191
A00004	A03990	443	A00004	A04005	434
A00004	A04045	235	A00004	A04062	338
A00004	A04118	270	A00004	A04135	263
A00004	A04209	291	A00004	A04279	345
A00004	A04285	344	A00004	A04286	299
A00004	A04304	290	A00004	A04314	319
A00004	A04397	213	A00004	A04443	266
A00004	A04449	167	A00004	A04510	241
A00004	A04530	329	A00004	A04591	270
A00004	A04620	463	A00004	A04729	319
A00004	A04763	358	A00004	A04797	270
A00004	A04822	343	A00004	A04953	270
A00004	A04973	205	A00004	A05057	213
A00004	A05122	279	A00004	A05309	236
A00004	A05376	352	A00004	A05415	491
A00004	A05459	176	A00004	A05665	238
A00004	A05666	154	A00004	A05709	260
A00004	A05746	249	A00004	A05767	216
A00004	A05858	249	A00004	A05901	198
A00004	A05911	154	A00004	A06021	293
A00004	A06554	196	A00004	A06579	232
A00004	A06607	462	A00004	A06635	455
A00004	A06659	299	A00004	A06678	205
A00004	A06852	213	A00004	A06868	183
A00004	A06897	319	A00004	A06949	294

A00004	A07165	473	A00004	A07167	160
A00004	A07171	235	A00004	A07177	251
A00004	A07178	210	A00004	A07200	491
A00004	A07350	270	A00004	A07486	271
A00004	A07552	180	A00004	A07663	270
A00004	A07736	484	A00004	A07848	210
A00004	A07943	301	A00004	A08070	185
A00004	A08484	165	A00004	A08626	163
A00004	A08724	279	A00004	A08989	259
A00004	A09145	216	A00004	A09147	180
A00004	A09162	285	A00004	A09209	160
A00004	A09404	185	A00004	A09406	213
A00004	A09492	270	A00004	A09699	180
A00004	A09790	247	A00004	A09882	274
A00004	A09929	191	A00004	A09955	172
A00004	A09989	160	A00004	A10018	368
A00004	A10247	491	A00004	A10382	210
A00004	A10424	160	A00004	A10495	301
A00004	A10575	201	A00004	A10743	210
A00004	A10950	301	A00004	A11279	185
A00004	A11316	235	A00004	A11348	210
A00004	A11476	243	A00004	A12335	249
A00004	A13348	391	A00004	A13548	270
A00004	A13897	249	A00004	A14120	399
A00004	A14247	467	A00004	A14389	310
A00004	A14405	491	A00004	A14440	484
A00004	A14790	249	A00004	A15282	193
A00004	A15368	310	A00004	A15564	388
A00004	A15623	301	A00004	A15717	414
A00004	A15747	246	A00004	A15838	168
A00004	A16877	212	A00005	A00012	294
A00005	A00013	356	A00005	A00085	333
A00005	A00106	423	A00005	A00155	488
A00005	A00196	468	A00005	A00204	328
A00005	A00212	297	A00005	A00216	101
A00005	A00218	451	A00005	A00219	348
A00005	A00264	460	A00005	A00301	154
A00005	A00661	187	A00005	A00663	152
A00005	A00914	151	A00005	A01137	213
A00005	A01138	491	A00005	A01400	154
A00005	A01590	201	A00005	A01668	208
A00005	A01669	299	A00005	A01670	173
A00005	A01726	372	A00005	A01894	218
A00005	A01982	277	A00005	A02071	491
A00005	A02074	173	A00005	A02075	452
A00005	A02077	358	A00005	A02083	340

A00005	A02129	324	A00005	A02133	384
A00005	A02187	256	A00005	A02215	375
A00005	A02298	236	A00005	A02300	489
A00005	A02306	213	A00005	A02314	330
A00005	A02317	374	A00005	A02329	490
A00005	A02330	413	A00005	A02443	151
A00005	A02545	228	A00005	A02644	210
A00005	A02665	384	A00005	A02669	382
A00005	A02671	63	A00005	A02754	170
A00005	A02774	484	A00005	A02785	491
A00005	A02788	213	A00005	A02859	268
A00005	A02874	213	A00005	A02918	152
A00005	A02929	191	A00005	A02947	267
A00005	A02951	230	A00005	A03050	490
A00005	A03099	213	A00005	A03213	221
A00005	A03275	170	A00005	A03346	165
A00005	A03427	221	A00005	A03486	219
A00005	A03545	232	A00005	A03744	163
A00005	A04023	484	A00005	A04051	276
A00005	A04162	299	A00005	A04256	196
A00005	A04267	163	A00005	A04369	490
A00005	A04371	412	A00005	A04436	455
A00005	A04555	400	A00005	A04608	281
A00005	A04612	243	A00005	A04614	210
A00005	A04633	481	A00005	A04635	477
A00005	A04865	229	A00005	A04974	409
A00005	A04975	466	A00005	A05070	223
A00005	A05217	446	A00005	A05390	226
A00005	A05494	183	A00005	A05505	366
A00005	A05659	195	A00005	A05868	240
A00005	A05992	360	A00005	A06607	408
A00005	A06747	193	A00005	A06762	224
A00005	A06874	398	A00005	A07158	240
A00005	A07200	337	A00005	A07217	281
A00005	A07297	470	A00005	A07407	228
A00005	A07442	217	A00005	A07491	193
A00005	A07515	406	A00005	A07541	319
A00005	A07542	199	A00005	A07543	205
A00005	A07561	174	A00005	A07630	288
A00005	A07634	165	A00005	A09020	245
A00005	A14423	358	A00005	A14430	299
A00005	A17078	290	A00006	A00015	481
A00006	A00018	283	A00006	A00021	312
A00006	A00034	389	A00006	A00053	475
A00006	A00067	899	A00006	A00069	945
A00006	A00074	411	A00006	A00081	491

A00006	A00089	210	A00006	A00127	424
A00006	A00140	899	A00006	A00150	486
A00006	A00159	899	A00006	A00167	462
A00006	A00178	170	A00006	A00209	899
A00006	A00210	149	A00006	A00219	379
A00006	A00230	900	A00006	A00246	899
A00006	A00294	363	A00006	A00309	405
A00006	A00315	455	A00006	A00328	247
A00006	A00335	163	A00006	A00370	174
A00006	A00400	341	A00006	A00446	307
A00006	A00642	159	A00006	A00643	490
A00006	A00747	191	A00006	A00806	223
A00006	A00815	491	A00006	A00967	154
A00006	A00989	63	A00006	A01052	899
A00006	A01112	347	A00006	A01257	279
A00006	A01277	273	A00006	A01425	347
A00006	A01527	899	A00006	A01550	943
A00006	A01554	936	A00006	A01558	899
A00006	A01601	230	A00006	A01615	989
A00006	A01640	187	A00006	A01709	472
A00006	A01722	104	A00006	A01740	157
A00006	A02213	218	A00006	A02223	154
A00006	A02397	168	A00006	A02424	115
A00006	A02425	371	A00006	A02429	308
A00006	A02465	121	A00006	A02860	157
A00006	A03053	193	A00006	A03054	283
A00006	A03110	154	A00006	A03188	196
A00006	A03209	193	A00006	A03244	207
A00006	A03445	235	A00006	A03458	407
A00006	A03750	434	A00006	A03818	226
A00006	A03963	232	A00006	A03981	328
A00006	A04033	196	A00006	A04218	219
A00006	A04348	269	A00006	A04351	202
A00006	A04380	378	A00006	A04471	238
A00006	A05158	296	A00006	A05165	374
A00006	A05229	285	A00006	A05377	177
A00006	A05949	174	A00006	A06814	229
A00006	A07143	263	A00006	A07311	281
A00006	A07568	318	A00006	A07726	488
A00006	A08092	308	A00006	A08583	213
A00006	A09078	63	A00006	A09104	213
A00006	A09644	182	A00006	A09983	245
A00006	A10645	174	A00006	A10683	179
A00006	A10711	256	A00006	A10766	236
A00006	A11125	899	A00006	A11392	165
A00006	A11444	480	A00006	A11604	386

A00006	A12227	294	A00006	A12299	291
A00006	A13340	311	A00006	A13443	319
A00006	A14212	357	A00006	A14410	159
A00006	A14693	484	A00006	A15430	489
A00006	A15669	480	A00006	A16436	481
A00007	A00008	945	A00007	A00042	475
A00007	A00071	945	A00007	A00123	361
A00007	A00135	489	A00007	A00153	191
A00007	A00160	425	A00007	A00161	926
A00007	A00263	151	A00007	A00269	154
A00007	A00337	434	A00007	A00416	302
A00007	A00485	196	A00007	A00561	443
A00007	A00625	474	A00007	A00669	366
A00007	A00799	174	A00007	A00877	491
A00007	A01125	267	A00007	A01161	221
A00007	A01527	899	A00007	A01650	319
A00007	A01705	91	A00007	A01736	425
A00007	A01737	491	A00007	A01757	196
A00007	A01758	193	A00007	A01759	279
A00007	A01787	85	A00007	A01796	129
A00007	A01839	241	A00007	A01890	329
A00007	A01895	366	A00007	A01899	154
A00007	A01909	263	A00007	A01914	380
A00007	A02382	152	A00007	A02501	157
A00007	A02687	196	A00007	A02691	412
A00007	A02698	490	A00007	A02700	366
A00007	A02701	168	A00007	A02711	147
A00007	A02714	168	A00007	A02873	246
A00007	A02877	152	A00007	A02893	302
A00007	A02906	456	A00007	A02926	296
A00007	A02986	226	A00007	A03518	296
A00007	A03575	254	A00007	A03585	247
A00007	A03654	152	A00007	A03866	270
A00007	A03986	291	A00007	A04004	204
A00007	A04035	205	A00007	A04118	347
A00007	A04256	163	A00007	A04279	162
A00007	A04287	369	A00007	A04303	347
A00007	A04304	379	A00007	A04449	230
A00007	A04623	180	A00007	A04805	414
A00007	A04953	347	A00007	A05008	286
A00007	A05015	279	A00007	A05094	170
A00007	A05305	202	A00007	A06277	236
A00007	A06379	329	A00007	A06603	491
A00007	A06607	318	A00007	A06868	312
A00007	A06926	247	A00007	A07167	467
A00007	A07182	302	A00007	A07465	347

A00007	A08503	338	A00007	A08571	470
A00007	A08615	314	A00007	A08717	274
A00007	A08934	329	A00007	A09554	369
A00007	A09728	341	A00007	A10602	263
A00007	A11023	241	A00007	A11150	424
A00007	A11180	473	A00007	A11258	157
A00007	A11281	234	A00007	A11348	462
A00007	A11450	390	A00007	A11478	467
A00007	A11547	480	A00007	A11665	457
A00007	A11915	226	A00007	A12335	423
A00007	A12586	369	A00007	A13927	468
A00007	A13978	281	A00007	A14236	398
A00007	A14405	360	A00007	A15035	252
A00007	A15368	278	A00007	A15383	213
A00007	A15603	196	A00007	A15605	163
A00007	A15623	369	A00008	A00042	462
A00008	A00071	945	A00008	A00152	343
A00008	A00153	293	A00008	A00161	334
A00008	A00190	334	A00008	A00233	208
A00008	A00546	321	A00008	A00799	191
A00008	A00887	200	A00008	A00914	121
A00008	A00951	190	A00008	A01044	306
A00008	A01364	188	A00008	A01393	160
A00008	A01450	369	A00008	A01527	899
A00008	A01565	177	A00008	A01629	154
A00008	A01676	174	A00008	A01705	63
A00008	A01726	469	A00008	A01730	152
A00008	A01737	481	A00008	A01749	91
A00008	A01755	152	A00008	A01757	210
A00008	A01768	227	A00008	A01769	200
A00008	A01775	226	A00008	A01782	154
A00008	A01789	454	A00008	A01794	213
A00008	A01796	80	A00008	A01861	256
A00008	A01867	201	A00008	A01873	324
A00008	A01883	439	A00008	A01893	245
A00008	A01895	374	A00008	A01899	299
A00008	A01902	157	A00008	A01914	157
A00008	A02109	205	A00008	A02154	232
A00008	A02301	183	A00008	A02319	324
A00008	A02336	247	A00008	A02341	324
A00008	A02342	232	A00008	A02392	301
A00008	A02417	281	A00008	A02469	185
A00008	A02488	160	A00008	A02541	229
A00008	A02546	285	A00008	A02565	152
A00008	A02574	162	A00008	A02629	208
A00008	A02686	201	A00008	A02687	367

A00008	A02691	483	A00008	A02692	185
A00008	A02700	374	A00008	A02701	180
A00008	A02702	165	A00008	A02707	221
A00008	A02710	188	A00008	A02714	182
A00008	A02758	210	A00008	A02785	270
A00008	A02786	160	A00008	A02811	213
A00008	A02814	281	A00008	A02820	258
A00008	A02873	258	A00008	A02893	311
A00008	A02906	432	A00008	A02908	386
A00008	A02943	296	A00008	A02988	337
A00008	A03007	279	A00008	A03086	234
A00008	A03120	311	A00008	A03224	316
A00008	A03264	236	A00008	A03488	258
A00008	A03537	301	A00008	A03583	278
A00008	A03585	479	A00008	A03653	168
A00008	A03847	333	A00008	A03870	311
A00008	A03984	353	A00008	A04004	490
A00008	A04005	288	A00008	A04031	265
A00008	A04118	356	A00008	A04123	311
A00008	A04125	267	A00008	A04287	374
A00008	A04304	489	A00008	A04314	216
A00008	A04372	306	A00008	A04443	254
A00008	A04531	386	A00008	A04541	288
A00008	A04560	278	A00008	A04574	329
A00008	A04620	263	A00008	A04728	167
A00008	A04805	229	A00008	A04870	216
A00008	A04905	363	A00008	A04939	232
A00008	A04953	356	A00008	A05036	296
A00008	A05310	167	A00008	A05438	246
A00008	A05550	182	A00008	A06211	273
A00008	A06379	163	A00008	A06558	333
A00008	A06603	443	A00008	A06708	193
A00008	A06765	267	A00008	A06778	196
A00008	A06877	245	A00008	A07167	469
A00008	A07199	377	A00008	A07200	224
A00008	A07943	374	A00008	A08615	324
A00008	A10332	226	A00008	A11166	407
A00008	A11167	278	A00008	A11285	305
A00008	A11292	262	A00008	A11348	412
A00008	A11725	235	A00008	A12024	324
A00008	A13906	263	A00008	A14405	371
A00008	A14685	311	A00008	A15282	156
A00008	A15470	160	A00008	A16152	187
A00008	A17155	247	A00009	A00010	945
A00009	A00011	490	A00009	A00014	439
A00009	A00041	299	A00009	A00044	170

A00009	A00046	193	A00009	A00052	901
A00009	A00067	899	A00009	A00074	311
A00009	A00090	422	A00009	A00093	910
A00009	A00116	385	A00009	A00130	258
A00009	A00136	113	A00009	A00138	377
A00009	A00145	74	A00009	A00153	899
A00009	A00159	899	A00009	A00160	104
A00009	A00161	87	A00009	A00162	249
A00009	A00175	283	A00009	A00183	252
A00009	A00185	121	A00009	A00205	254
A00009	A00207	409	A00009	A00211	899
A00009	A00214	63	A00009	A00224	216
A00009	A00234	386	A00009	A00240	343
A00009	A00242	904	A00009	A00246	939
A00009	A00254	462	A00009	A00261	193
A00009	A00273	179	A00009	A00601	357
A00009	A01052	899	A00009	A01524	912
A00009	A01527	943	A00009	A01553	901
A00009	A01605	899	A00009	A01628	945
A00009	A01640	899	A00009	A01680	899
A00009	A01688	899	A00009	A01692	177
A00009	A01708	945	A00009	A01719	943
A00009	A01879	918	A00009	A01880	933
A00009	A01908	432	A00009	A01920	944
A00009	A02304	187	A00009	A02337	213
A00009	A02803	157	A00009	A03019	132
A00009	A03204	193	A00009	A03271	143
A00009	A03817	919	A00009	A03854	899
A00009	A03965	154	A00009	A04018	246
A00009	A04091	160	A00009	A04138	916
A00009	A06289	170	A00009	A07157	476
A00009	A07311	926	A00009	A07760	177
A00009	A07803	188	A00009	A08417	159
A00009	A09218	213	A00009	A09220	121
A00009	A09401	143	A00009	A09643	937
A00009	A09660	163	A00009	A09937	490
A00009	A10580	918	A00009	A10639	293
A00009	A11124	401	A00009	A11369	246
A00009	A12009	210	A00009	A12886	486
A00009	A12932	165	A00009	A13198	386
A00009	A14008	143	A00009	A14033	318
A00009	A14034	389	A00009	A14043	899
A00009	A14187	899	A00009	A14208	219
A00009	A14426	204	A00009	A14433	899
A00009	A14443	899	A00009	A15773	195
A00009	A15841	303	A00009	A16084	930

A00010	A00011	945	A00010	A00014	945
A00010	A00015	419	A00010	A00019	903
A00010	A00021	63	A00010	A00022	936
A00010	A00025	942	A00010	A00029	945
A00010	A00031	198	A00010	A00035	407
A00010	A00036	933	A00010	A00041	945
A00010	A00042	931	A00010	A00046	906
A00010	A00048	944	A00010	A00050	324
A00010	A00052	967	A00010	A00058	305
A00010	A00064	385	A00010	A00066	924
A00010	A00067	953	A00010	A00074	975
A00010	A00081	167	A00010	A00082	174
A00010	A00090	488	A00010	A00091	402
A00010	A00093	913	A00010	A00098	443
A00010	A00099	899	A00010	A00100	900
A00010	A00113	945	A00010	A00114	198
A00010	A00115	899	A00010	A00116	404
A00010	A00118	204	A00010	A00120	162
A00010	A00122	416	A00010	A00124	146
A00010	A00130	899	A00010	A00132	477
A00010	A00133	899	A00010	A00136	188
A00010	A00137	235	A00010	A00138	543
A00010	A00139	430	A00010	A00140	899
A00010	A00141	262	A00010	A00145	417
A00010	A00146	369	A00010	A00147	269
A00010	A00152	420	A00010	A00153	900
A00010	A00156	960	A00010	A00157	901
A00010	A00158	358	A00010	A00159	949
A00010	A00161	947	A00010	A00162	952
A00010	A00163	951	A00010	A00166	937
A00010	A00167	928	A00010	A00169	993
A00010	A00171	255	A00010	A00172	899
A00010	A00174	919	A00010	A00175	944
A00010	A00182	941	A00010	A00183	551
A00010	A00184	899	A00010	A00185	945
A00010	A00186	901	A00010	A00189	176
A00010	A00190	899	A00010	A00200	899
A00010	A00205	945	A00010	A00207	956
A00010	A00208	518	A00010	A00209	994
A00010	A00211	943	A00010	A00214	322
A00010	A00220	899	A00010	A00221	319
A00010	A00224	949	A00010	A00230	928
A00010	A00233	899	A00010	A00234	488
A00010	A00236	912	A00010	A00239	543
A00010	A00240	994	A00010	A00241	477
A00010	A00242	994	A00010	A00243	159

A00010	A00246	945	A00010	A00252	899
A00010	A00254	960	A00010	A00255	957
A00010	A00257	994	A00010	A00261	571
A00010	A00263	585	A00010	A00270	355
A00010	A00271	899	A00010	A00276	185
A00010	A00312	940	A00010	A00316	477
A00010	A00324	993	A00010	A00388	899
A00010	A00418	942	A00010	A00669	939
A00010	A00696	426	A00010	A00808	942
A00010	A00811	191	A00010	A00932	919
A00010	A00933	899	A00010	A00981	417
A00010	A01052	935	A00010	A01176	899
A00010	A01212	562	A00010	A01286	63
A00010	A01289	899	A00010	A01307	63
A00010	A01310	65	A00010	A01357	151
A00010	A01435	218	A00010	A01512	912
A00010	A01524	997	A00010	A01527	994
A00010	A01536	900	A00010	A01540	162
A00010	A01553	949	A00010	A01554	938
A00010	A01558	994	A00010	A01560	962
A00010	A01561	949	A00010	A01562	945
A00010	A01572	466	A00010	A01588	917
A00010	A01592	910	A00010	A01601	899
A00010	A01602	900	A00010	A01605	945
A00010	A01612	899	A00010	A01615	917
A00010	A01628	949	A00010	A01636	907
A00010	A01637	899	A00010	A01638	899
A00010	A01639	914	A00010	A01640	944
A00010	A01651	899	A00010	A01665	899
A00010	A01680	899	A00010	A01688	908
A00010	A01692	945	A00010	A01697	367
A00010	A01698	63	A00010	A01702	914
A00010	A01708	943	A00010	A01709	908
A00010	A01718	939	A00010	A01719	941
A00010	A01730	353	A00010	A01742	899
A00010	A01844	344	A00010	A01880	899
A00010	A01914	464	A00010	A01995	491
A00010	A02088	223	A00010	A02159	176
A00010	A02254	385	A00010	A02284	901
A00010	A02337	899	A00010	A02411	247
A00010	A02419	63	A00010	A02482	249
A00010	A02502	925	A00010	A02642	899
A00010	A02678	419	A00010	A02699	899
A00010	A02702	187	A00010	A02711	182
A00010	A02723	905	A00010	A02803	308
A00010	A03019	899	A00010	A03087	270

A00010	A03099	899	A00010	A03195	63
A00010	A03225	165	A00010	A03271	110
A00010	A03292	182	A00010	A03436	267
A00010	A03445	899	A00010	A03451	205
A00010	A03536	182	A00010	A03591	942
A00010	A03651	199	A00010	A03748	454
A00010	A03784	462	A00010	A03809	950
A00010	A03854	913	A00010	A03910	151
A00010	A04012	943	A00010	A04248	899
A00010	A04314	165	A00010	A04422	917
A00010	A04451	157	A00010	A04484	899
A00010	A04622	255	A00010	A04686	899
A00010	A04699	899	A00010	A05025	238
A00010	A05065	191	A00010	A05082	918
A00010	A05090	899	A00010	A05208	902
A00010	A05216	899	A00010	A05326	899
A00010	A05363	162	A00010	A05453	945
A00010	A05633	489	A00010	A05867	213
A00010	A06077	154	A00010	A06197	228
A00010	A06289	139	A00010	A06397	154
A00010	A06409	198	A00010	A06427	168
A00010	A06588	191	A00010	A06608	899
A00010	A06610	475	A00010	A06762	899
A00010	A06893	899	A00010	A07118	160
A00010	A07165	899	A00010	A07275	243
A00010	A07311	978	A00010	A07422	899
A00010	A07460	899	A00010	A07565	176
A00010	A08280	407	A00010	A08608	418
A00010	A08701	79	A00010	A08902	291
A00010	A08941	301	A00010	A09149	177
A00010	A09181	922	A00010	A09201	899
A00010	A09218	435	A00010	A09220	915
A00010	A09319	899	A00010	A09349	160
A00010	A09383	259	A00010	A09401	91
A00010	A09506	202	A00010	A09511	208
A00010	A09542	899	A00010	A09556	152
A00010	A09568	937	A00010	A09660	945
A00010	A09803	305	A00010	A09804	294
A00010	A09937	452	A00010	A10174	899
A00010	A10175	302	A00010	A10205	486
A00010	A10233	899	A00010	A10365	925
A00010	A10580	899	A00010	A10735	215
A00010	A10756	338	A00010	A10801	933
A00010	A10813	900	A00010	A11089	213
A00010	A11124	460	A00010	A11125	899
A00010	A11198	235	A00010	A11369	468

A00010	A11707	326	A00010	A11708	176
A00010	A11983	938	A00010	A12383	946
A00010	A12409	151	A00010	A12705	899
A00010	A12886	419	A00010	A12932	945
A00010	A12969	945	A00010	A12971	900
A00010	A12990	353	A00010	A12992	76
A00010	A13003	899	A00010	A13181	157
A00010	A13362	353	A00010	A13390	899
A00010	A13391	941	A00010	A13393	899
A00010	A13401	899	A00010	A13511	466
A00010	A13605	900	A00010	A13924	230
A00010	A13958	899	A00010	A13975	215
A00010	A13980	221	A00010	A13981	899
A00010	A13984	942	A00010	A13985	910
A00010	A13987	992	A00010	A13991	899
A00010	A13999	899	A00010	A14004	945
A00010	A14008	90	A00010	A14010	992
A00010	A14027	176	A00010	A14031	921
A00010	A14032	969	A00010	A14033	185
A00010	A14035	911	A00010	A14038	925
A00010	A14042	899	A00010	A14043	899
A00010	A14045	916	A00010	A14061	899
A00010	A14065	899	A00010	A14066	899
A00010	A14069	899	A00010	A14103	198
A00010	A14128	174	A00010	A14187	943
A00010	A14188	944	A00010	A14198	953
A00010	A14208	338	A00010	A14242	899
A00010	A14400	383	A00010	A14402	266
A00010	A14426	162	A00010	A14433	945
A00010	A14443	992	A00010	A14444	907
A00010	A14452	135	A00010	A14472	899
A00010	A14827	152	A00010	A15292	899
A00010	A15312	899	A00010	A15325	414
A00010	A15726	63	A00010	A15786	274
A00010	A16084	901	A00010	A16085	899
A00010	A17188	899	A00011	A00014	456
A00011	A00021	104	A00011	A00022	281
A00011	A00025	232	A00011	A00029	171
A00011	A00041	942	A00011	A00042	449
A00011	A00046	274	A00011	A00052	945
A00011	A00067	906	A00011	A00073	263
A00011	A00074	900	A00011	A00094	928
A00011	A00116	485	A00011	A00124	945
A00011	A00133	899	A00011	A00138	469
A00011	A00140	899	A00011	A00141	207
A00011	A00145	65	A00011	A00156	71

A00011	A00160	199	A00011	A00161	135
A00011	A00162	945	A00011	A00165	940
A00011	A00175	265	A00011	A00184	899
A00011	A00185	99	A00011	A00188	944
A00011	A00205	490	A00011	A00207	945
A00011	A00208	118	A00011	A00209	899
A00011	A00211	228	A00011	A00214	63
A00011	A00224	929	A00011	A00230	901
A00011	A00233	323	A00011	A00234	945
A00011	A00239	633	A00011	A00240	958
A00011	A00241	318	A00011	A00242	378
A00011	A00246	944	A00011	A00248	232
A00011	A00254	940	A00011	A00261	281
A00011	A01512	908	A00011	A01524	903
A00011	A01527	938	A00011	A01553	477
A00011	A01554	944	A00011	A01558	911
A00011	A01560	461	A00011	A01561	63
A00011	A01562	908	A00011	A01588	899
A00011	A01596	207	A00011	A01615	899
A00011	A01628	935	A00011	A01638	899
A00011	A01639	944	A00011	A01651	899
A00011	A01708	943	A00011	A01709	966
A00011	A01719	944	A00011	A01726	446
A00011	A01748	383	A00011	A01793	95
A00011	A01883	162	A00011	A01908	65
A00011	A01920	324	A00011	A02171	217
A00011	A02178	154	A00011	A02304	165
A00011	A02326	210	A00011	A02734	185
A00011	A03019	63	A00011	A03204	177
A00011	A03263	124	A00011	A03465	71
A00011	A03598	252	A00011	A03656	252
A00011	A03685	339	A00011	A03780	210
A00011	A04138	205	A00011	A04280	247
A00011	A04314	191	A00011	A04320	327
A00011	A04699	941	A00011	A05150	188
A00011	A05453	296	A00011	A05944	249
A00011	A06055	154	A00011	A06289	301
A00011	A07212	154	A00011	A07311	942
A00011	A08114	266	A00011	A08278	480
A00011	A08279	252	A00011	A08571	196
A00011	A08608	135	A00011	A09162	154
A00011	A09181	461	A00011	A09470	156
A00011	A09542	63	A00011	A09577	917
A00011	A09804	899	A00011	A09937	491
A00011	A10294	174	A00011	A10560	899
A00011	A10623	905	A00011	A10791	201

A00011	A10801	168	A00011	A10982	407
A00011	A11124	174	A00011	A11125	930
A00011	A11369	441	A00011	A11943	899
A00011	A12664	899	A00011	A12886	447
A00011	A12980	232	A00011	A13440	340
A00011	A13510	187	A00011	A13917	190
A00011	A13946	193	A00011	A13977	252
A00011	A13980	93	A00011	A14022	899
A00011	A14027	297	A00011	A14043	899
A00011	A14188	165	A00011	A14242	899
A00011	A14364	255	A00011	A14402	188
A00011	A14426	439	A00011	A14438	937
A00011	A16084	200	A00011	A16994	156
A00012	A00013	458	A00012	A00021	274
A00012	A00072	942	A00012	A00155	469
A00012	A00162	899	A00012	A00168	407
A00012	A00209	899	A00012	A00310	926
A00012	A00567	399	A00012	A01138	373
A00012	A01527	899	A00012	A01590	223
A00012	A02083	252	A00012	A02306	452
A00012	A02330	942	A00012	A02877	154
A00012	A04441	469	A00012	A08878	899
A00012	A09072	308	A00012	A11983	899
A00012	A14443	915	A00012	A14444	218
A00013	A00021	207	A00013	A00034	249
A00013	A00080	201	A00013	A00085	307
A00013	A00087	172	A00013	A00142	172
A00013	A00155	267	A00013	A00196	160
A00013	A00216	484	A00013	A00219	477
A00013	A00264	283	A00013	A00312	385
A00013	A00498	217	A00013	A01117	474
A00013	A01137	427	A00013	A01138	490
A00013	A01230	254	A00013	A01404	441
A00013	A01462	343	A00013	A01590	479
A00013	A01626	159	A00013	A01679	288
A00013	A01722	216	A00013	A01867	391
A00013	A01884	334	A00013	A01926	443
A00013	A02074	481	A00013	A02083	486
A00013	A02148	279	A00013	A02156	469
A00013	A02183	157	A00013	A02213	319
A00013	A02268	400	A00013	A02275	241
A00013	A02305	430	A00013	A02306	483
A00013	A02309	286	A00013	A02317	468
A00013	A02330	488	A00013	A02454	321
A00013	A02524	254	A00013	A02751	288
A00013	A02759	434	A00013	A02785	274

A00013	A03091	257	A00013	A03099	488
A00013	A03186	374	A00013	A03238	343
A00013	A03468	297	A00013	A03484	271
A00013	A04010	296	A00013	A04162	468
A00013	A04163	270	A00013	A04258	463
A00013	A04850	240	A00013	A04981	454
A00013	A05277	417	A00013	A05370	277
A00013	A05533	249	A00013	A05610	360
A00013	A06101	296	A00013	A06134	288
A00013	A06263	438	A00013	A06473	249
A00013	A06607	306	A00013	A06769	341
A00013	A07037	327	A00013	A07156	395
A00013	A07757	277	A00013	A08746	252
A00013	A11646	274	A00013	A14071	316
A00013	A14198	196	A00013	A14423	465
A00013	A14525	202	A00013	A15498	389
A00013	A16219	279	A00014	A00018	173
A00014	A00019	391	A00014	A00021	195
A00014	A00048	697	A00014	A00052	899
A00014	A00067	899	A00014	A00074	492
A00014	A00082	228	A00014	A00091	698
A00014	A00113	984	A00014	A00116	246
A00014	A00156	961	A00014	A00162	291
A00014	A00180	172	A00014	A00182	899
A00014	A00185	152	A00014	A00205	207
A00014	A00207	669	A00014	A00214	63
A00014	A00224	899	A00014	A00228	296
A00014	A00240	505	A00014	A00247	212
A00014	A00254	416	A00014	A00255	202
A00014	A00451	183	A00014	A00932	213
A00014	A01310	193	A00014	A01485	183
A00014	A01524	929	A00014	A01527	905
A00014	A01558	899	A00014	A01560	151
A00014	A01561	73	A00014	A01562	457
A00014	A01588	899	A00014	A01718	154
A00014	A02239	247	A00014	A02254	230
A00014	A02304	154	A00014	A02586	177
A00014	A03087	232	A00014	A03784	281
A00014	A03830	213	A00014	A04012	187
A00014	A04323	269	A00014	A05165	255
A00014	A06660	170	A00014	A07311	909
A00014	A09660	243	A00014	A09906	357
A00014	A09937	441	A00014	A10342	346
A00014	A10801	263	A00014	A10813	272
A00014	A11708	362	A00014	A12886	302
A00014	A12932	243	A00014	A12969	379

A00014	A12971	899	A00014	A12990	152
A00014	A13362	335	A00014	A13364	268
A00014	A14004	430	A00014	A14198	180
A00014	A14433	224	A00014	A14443	899
A00014	A15325	446	A00014	A16084	288
A00015	A00018	126	A00015	A00020	369
A00015	A00021	491	A00015	A00023	899
A00015	A00026	335	A00015	A00029	899
A00015	A00030	452	A00015	A00031	945
A00015	A00032	475	A00015	A00034	945
A00015	A00041	420	A00015	A00045	246
A00015	A00052	162	A00015	A00053	443
A00015	A00056	317	A00015	A00057	177
A00015	A00063	167	A00015	A00067	899
A00015	A00069	491	A00015	A00074	215
A00015	A00076	380	A00015	A00079	299
A00015	A00081	490	A00015	A00083	462
A00015	A00085	286	A00015	A00087	396
A00015	A00089	491	A00015	A00106	941
A00015	A00111	488	A00015	A00112	235
A00015	A00125	296	A00015	A00127	486
A00015	A00131	198	A00015	A00150	252
A00015	A00153	899	A00015	A00159	899
A00015	A00166	480	A00015	A00167	941
A00015	A00168	475	A00015	A00177	379
A00015	A00185	408	A00015	A00190	489
A00015	A00196	400	A00015	A00203	63
A00015	A00207	436	A00015	A00209	938
A00015	A00210	224	A00015	A00218	480
A00015	A00219	491	A00015	A00221	480
A00015	A00227	945	A00015	A00231	899
A00015	A00245	456	A00015	A00254	483
A00015	A00274	945	A00015	A00292	432
A00015	A00315	483	A00015	A00324	899
A00015	A00340	258	A00015	A00349	346
A00015	A00440	162	A00015	A00616	488
A00015	A00752	324	A00015	A00815	491
A00015	A00969	232	A00015	A01257	461
A00015	A01463	378	A00015	A01476	313
A00015	A01485	380	A00015	A01493	408
A00015	A01524	931	A00015	A01527	904
A00015	A01557	177	A00015	A01590	162
A00015	A01601	232	A00015	A01605	358
A00015	A01626	176	A00015	A01629	458
A00015	A01640	235	A00015	A01722	480
A00015	A01741	260	A00015	A01790	193

A00015	A01844	899	A00015	A01925	288
A00015	A01929	185	A00015	A02048	424
A00015	A02126	230	A00015	A02174	213
A00015	A02176	162	A00015	A02213	177
A00015	A02215	301	A00015	A02236	489
A00015	A02251	196	A00015	A02282	193
A00015	A02324	235	A00015	A02406	230
A00015	A02423	110	A00015	A02424	84
A00015	A02425	945	A00015	A02429	358
A00015	A02431	461	A00015	A02433	205
A00015	A02454	129	A00015	A02464	390
A00015	A02465	296	A00015	A02468	462
A00015	A02472	234	A00015	A02481	944
A00015	A02508	188	A00015	A02624	144
A00015	A02671	491	A00015	A02759	228
A00015	A02772	174	A00015	A03071	424
A00015	A03079	205	A00015	A03080	191
A00015	A03081	238	A00015	A03085	276
A00015	A03109	149	A00015	A03110	945
A00015	A03115	249	A00015	A03126	399
A00015	A03127	317	A00015	A03156	221
A00015	A03183	308	A00015	A03196	241
A00015	A03209	933	A00015	A03219	945
A00015	A03259	483	A00015	A03314	173
A00015	A03458	294	A00015	A03633	319
A00015	A03639	488	A00015	A03644	174
A00015	A03744	286	A00015	A03782	162
A00015	A03799	446	A00015	A03800	337
A00015	A03893	226	A00015	A03910	168
A00015	A03942	491	A00015	A03961	488
A00015	A03975	180	A00015	A04169	321
A00015	A04180	185	A00015	A04202	301
A00015	A04216	314	A00015	A04218	179
A00015	A04256	224	A00015	A04380	450
A00015	A04398	283	A00015	A04422	340
A00015	A04454	205	A00015	A04471	199
A00015	A04488	226	A00015	A04490	455
A00015	A04492	151	A00015	A04647	308
A00015	A04676	490	A00015	A04696	185
A00015	A05091	306	A00015	A05118	265
A00015	A05182	177	A00015	A05229	249
A00015	A05246	230	A00015	A05248	276
A00015	A05288	317	A00015	A05359	180
A00015	A05491	230	A00015	A05517	159
A00015	A05533	447	A00015	A05706	173
A00015	A05784	333	A00015	A05965	276

A00015	A06086	223	A00015	A06118	321
A00015	A06344	173	A00015	A06997	232
A00015	A07141	213	A00015	A07209	236
A00015	A07311	899	A00015	A07422	932
A00015	A07726	311	A00015	A07803	470
A00015	A08031	333	A00015	A08714	334
A00015	A08834	388	A00015	A08840	366
A00015	A08859	243	A00015	A09036	188
A00015	A09062	483	A00015	A09085	191
A00015	A09127	218	A00015	A09208	361
A00015	A09217	389	A00015	A09222	177
A00015	A09239	485	A00015	A09374	180
A00015	A09390	301	A00015	A09480	199
A00015	A09732	489	A00015	A09733	217
A00015	A09744	288	A00015	A09984	265
A00015	A10040	201	A00015	A10090	249
A00015	A10164	308	A00015	A10208	477
A00015	A10336	440	A00015	A10580	923
A00015	A10716	333	A00015	A11190	326
A00015	A11412	369	A00015	A11415	226
A00015	A11537	230	A00015	A11558	401
A00015	A12393	401	A00015	A13084	156
A00015	A13124	422	A00015	A13225	899
A00015	A13336	226	A00015	A13363	202
A00015	A13435	241	A00015	A13546	288
A00015	A13826	385	A00015	A13849	257
A00015	A13860	263	A00015	A13899	235
A00015	A13944	420	A00015	A13987	401
A00015	A14010	390	A00015	A14190	490
A00015	A14202	367	A00015	A14210	491
A00015	A14369	485	A00015	A14370	465
A00015	A14371	491	A00015	A14372	165
A00015	A14379	452	A00015	A14408	281
A00015	A14443	900	A00015	A14444	899
A00015	A14511	491	A00015	A14536	337
A00015	A14539	353	A00015	A14577	201
A00015	A14604	433	A00015	A14667	305
A00015	A14722	276	A00015	A14930	165
A00015	A15230	388	A00015	A15292	491
A00015	A15312	318	A00015	A15313	469
A00015	A15317	441	A00015	A15318	409
A00015	A15333	227	A00015	A15348	406
A00015	A15349	488	A00015	A15352	454
A00015	A15381	324	A00015	A15396	334
A00015	A15404	435	A00015	A15419	323
A00015	A15431	162	A00015	A15435	254

A00015	A15441	213	A00015	A15444	400
A00015	A15445	274	A00015	A15447	414
A00015	A15452	273	A00015	A15480	436
A00015	A15481	279	A00015	A15485	157
A00015	A15495	486	A00015	A15515	184
A00015	A15792	171	A00015	A15800	241
A00015	A15804	329	A00015	A15813	154
A00015	A15830	160	A00015	A15913	170
A00015	A16107	246	A00015	A16177	188
A00015	A16300	350	A00015	A16493	371
A00016	A00029	307	A00016	A00036	201
A00016	A00048	173	A00016	A00052	899
A00016	A00059	477	A00016	A00100	910
A00016	A00119	930	A00016	A00123	167
A00016	A00167	936	A00016	A00180	184
A00016	A00195	942	A00016	A00209	899
A00016	A00228	176	A00016	A00230	899
A00016	A00236	469	A00016	A00252	945
A00016	A00253	945	A00016	A00255	345
A00016	A00323	411	A00016	A00329	322
A00016	A00454	450	A00016	A00762	241
A00016	A00910	343	A00016	A01042	352
A00016	A01083	180	A00016	A01300	305
A00016	A01378	170	A00016	A01527	899
A00016	A01558	899	A00016	A01615	909
A00016	A01920	928	A00016	A02698	419
A00016	A02703	172	A00016	A03740	418
A00016	A03820	157	A00016	A04914	268
A00016	A08054	232	A00016	A09818	379
A00016	A09888	345	A00016	A09964	357
A00016	A11921	273	A00016	A11932	254
A00016	A14013	327	A00016	A14319	273
A00016	A15275	314	A00016	A15699	319
A00016	A16080	438	A00016	A16764	468
A00017	A00052	899	A00017	A00163	424
A00017	A00198	905	A00017	A02495	335
A00017	A02507	480	A00017	A02638	277
A00017	A02784	254	A00017	A02790	435
A00017	A02819	347	A00017	A02861	318
A00017	A03602	221	A00017	A03609	207
A00017	A04512	384	A00017	A04516	329
A00017	A04529	322	A00017	A04560	347
A00017	A04810	391	A00017	A05120	328
A00017	A05122	226	A00017	A09073	270
A00017	A14378	157	A00017	A14543	299
A00017	A14674	291	A00017	A15838	350

A00017	A15840	260	A00018	A00019	945
A00018	A00037	205	A00018	A00053	975
A00018	A00067	899	A00018	A00069	427
A00018	A00095	344	A00018	A00127	409
A00018	A00149	334	A00018	A00156	262
A00018	A00159	899	A00018	A00166	369
A00018	A00167	391	A00018	A00175	478
A00018	A00198	241	A00018	A00209	919
A00018	A00210	88	A00018	A00228	473
A00018	A00237	156	A00018	A00240	399
A00018	A00242	204	A00018	A00243	536
A00018	A00244	293	A00018	A00247	967
A00018	A00400	334	A00018	A00900	165
A00018	A00932	491	A00018	A00967	334
A00018	A01151	334	A00018	A01485	469
A00018	A01524	903	A00018	A01527	378
A00018	A01553	922	A00018	A01629	259
A00018	A01718	491	A00018	A01844	899
A00018	A01926	334	A00018	A02035	185
A00018	A02174	184	A00018	A02239	286
A00018	A02324	281	A00018	A02397	176
A00018	A02425	488	A00018	A02481	334
A00018	A02624	193	A00018	A02673	221
A00018	A03315	205	A00018	A03571	277
A00018	A03873	334	A00018	A04074	940
A00018	A04202	185	A00018	A04347	972
A00018	A04415	205	A00018	A04449	334
A00018	A04665	221	A00018	A04780	238
A00018	A06212	187	A00018	A07107	168
A00018	A07803	162	A00018	A08850	334
A00018	A09247	188	A00018	A09906	445
A00018	A10040	249	A00018	A10210	281
A00018	A11559	334	A00018	A12373	334
A00018	A12465	340	A00018	A12990	701
A00018	A14210	334	A00018	A14371	334
A00018	A14636	334	A00018	A14722	314
A00018	A16025	286	A00018	A16916	259
A00019	A00037	454	A00019	A00053	945
A00019	A00113	199	A00019	A00116	156
A00019	A00156	267	A00019	A00162	227
A00019	A00167	401	A00019	A00175	363
A00019	A00191	270	A00019	A00192	281
A00019	A00198	374	A00019	A00209	901
A00019	A00228	286	A00019	A00240	640
A00019	A00242	208	A00019	A00243	676
A00019	A00244	297	A00019	A00247	491

A00019	A00275	374	A00019	A00356	462
A00019	A00643	207	A00019	A00932	747
A00019	A01291	458	A00019	A01485	462
A00019	A01524	899	A00019	A01553	900
A00019	A01561	328	A00019	A01623	260
A00019	A01718	712	A00019	A01974	337
A00019	A02151	208	A00019	A02239	157
A00019	A02407	174	A00019	A02425	210
A00019	A03571	149	A00019	A04202	294
A00019	A04347	446	A00019	A04761	246
A00019	A06219	301	A00019	A06466	263
A00019	A06950	162	A00019	A07311	911
A00019	A07803	168	A00019	A09906	401
A00019	A10210	283	A00019	A11279	258
A00019	A12618	208	A00019	A12752	419
A00019	A12897	170	A00019	A12990	491
A00019	A14061	899	A00019	A15498	216
A00019	A15551	260	A00019	A16025	288
A00020	A00026	484	A00020	A00032	466
A00020	A00034	358	A00020	A00085	184
A00020	A00089	223	A00020	A00112	375
A00020	A00122	159	A00020	A00127	401
A00020	A00209	899	A00020	A00221	273
A00020	A00353	182	A00020	A00616	281
A00020	A01527	899	A00020	A01688	899
A00020	A01880	254	A00020	A02049	157
A00020	A02152	243	A00020	A02236	291
A00020	A02425	409	A00020	A02675	184
A00020	A02785	152	A00020	A03014	247
A00020	A03110	435	A00020	A03470	310
A00020	A03639	229	A00020	A03800	474
A00020	A04490	489	A00020	A04914	277
A00020	A04929	267	A00020	A08408	249
A00020	A09218	305	A00020	A09238	218
A00020	A09550	348	A00020	A10164	324
A00020	A11412	196	A00020	A11415	269
A00020	A13511	358	A00020	A13824	187
A00020	A13944	406	A00020	A13987	154
A00020	A14210	438	A00020	A14259	440
A00020	A14370	489	A00020	A14371	446
A00020	A14379	357	A00020	A14430	156
A00020	A14511	467	A00020	A15292	152
A00020	A15312	254	A00020	A15313	270
A00020	A15335	274	A00020	A15452	176
A00020	A15457	334	A00020	A15459	291
A00020	A15480	196	A00020	A15498	265

A00021	A00030	177	A00021	A00034	491
A00021	A00038	218	A00021	A00041	232
A00021	A00048	445	A00021	A00053	469
A00021	A00056	440	A00021	A00057	307
A00021	A00067	899	A00021	A00069	491
A00021	A00081	475	A00021	A00083	167
A00021	A00097	238	A00021	A00106	477
A00021	A00131	201	A00021	A00166	323
A00021	A00168	481	A00021	A00174	195
A00021	A00196	273	A00021	A00207	63
A00021	A00209	899	A00021	A00212	470
A00021	A00219	481	A00021	A00221	329
A00021	A00227	165	A00021	A00254	945
A00021	A00264	180	A00021	A00274	240
A00021	A00292	452	A00021	A00309	157
A00021	A00312	344	A00021	A00315	329
A00021	A00616	475	A00021	A00815	477
A00021	A01125	350	A00021	A01257	419
A00021	A01527	899	A00021	A01590	274
A00021	A01603	213	A00021	A01629	283
A00021	A01722	409	A00021	A01973	314
A00021	A02109	201	A00021	A02126	400
A00021	A02176	232	A00021	A02215	208
A00021	A02423	435	A00021	A02425	466
A00021	A02444	257	A00021	A02464	171
A00021	A02465	449	A00021	A02468	301
A00021	A02644	187	A00021	A02668	294
A00021	A02671	490	A00021	A02756	212
A00021	A02759	176	A00021	A02760	218
A00021	A02785	263	A00021	A02984	235
A00021	A03047	269	A00021	A03053	221
A00021	A03110	481	A00021	A03114	269
A00021	A03115	340	A00021	A03141	162
A00021	A03224	179	A00021	A03402	226
A00021	A03498	418	A00021	A03584	162
A00021	A03633	255	A00021	A03639	443
A00021	A03784	210	A00021	A03961	945
A00021	A04023	199	A00021	A04216	249
A00021	A04256	173	A00021	A04398	446
A00021	A04647	245	A00021	A05048	329
A00021	A05184	219	A00021	A05288	263
A00021	A05494	160	A00021	A05533	480
A00021	A06762	202	A00021	A07367	170
A00021	A07509	238	A00021	A09062	274
A00021	A09217	329	A00021	A09660	170
A00021	A09812	269	A00021	A10079	432

A00021	A10208	353	A00021	A10841	353
A00021	A12932	171	A00021	A14198	165
A00021	A14210	299	A00021	A14369	195
A00021	A15230	468	A00021	A15325	232
A00021	A15913	262	A00021	A16636	185
A00022	A00093	215	A00022	A00094	710
A00022	A00098	163	A00022	A00116	224
A00022	A00133	899	A00022	A00160	157
A00022	A00161	152	A00022	A00163	968
A00022	A00188	224	A00022	A00205	184
A00022	A00207	338	A00022	A00246	899
A00022	A00257	238	A00022	A00262	420
A00022	A00324	378	A00022	A00336	249
A00022	A00477	185	A00022	A01507	208
A00022	A01512	935	A00022	A01560	188
A00022	A01606	157	A00022	A01628	162
A00022	A01698	307	A00022	A01776	322
A00022	A01850	255	A00022	A02250	230
A00022	A02774	245	A00022	A04023	249
A00022	A04288	249	A00022	A04449	223
A00022	A05556	160	A00022	A05601	291
A00022	A05854	204	A00022	A06579	273
A00022	A07311	899	A00022	A07561	204
A00022	A08093	229	A00022	A08692	252
A00022	A09162	440	A00022	A10623	187
A00022	A10808	406	A00022	A10982	405
A00022	A11313	193	A00022	A13977	198
A00022	A13980	159	A00022	A14925	185
A00022	A16438	290	A00022	A16835	486
A00023	A00024	943	A00023	A00031	457
A00023	A00045	152	A00023	A00056	414
A00023	A00057	163	A00023	A00061	251
A00023	A00085	937	A00023	A00109	270
A00023	A00123	216	A00023	A00125	281
A00023	A00141	177	A00023	A00145	199
A00023	A00160	241	A00023	A00209	899
A00023	A00246	171	A00023	A00264	274
A00023	A00274	391	A00023	A00440	200
A00023	A00815	914	A00023	A00870	195
A00023	A01092	299	A00023	A01142	346
A00023	A01524	902	A00023	A01527	899
A00023	A01654	340	A00023	A01880	288
A00023	A01889	274	A00023	A02141	353
A00023	A02143	419	A00023	A02179	213
A00023	A02288	366	A00023	A02313	302
A00023	A02425	932	A00023	A02447	357

A00023	A02495	240	A00023	A02711	191
A00023	A02837	266	A00023	A02917	288
A00023	A03081	408	A00023	A03305	319
A00023	A03651	198	A00023	A03661	210
A00023	A03744	274	A00023	A04492	334
A00023	A04759	316	A00023	A06623	246
A00023	A07173	263	A00023	A08572	375
A00023	A08911	307	A00023	A09570	477
A00023	A13370	441	A00023	A14236	255
A00023	A14393	162	A00023	A15952	412
A00024	A00030	417	A00024	A00034	199
A00024	A00057	191	A00024	A00069	247
A00024	A00081	88	A00024	A00085	942
A00024	A00209	899	A00024	A00616	213
A00024	A00815	221	A00024	A01142	477
A00024	A01277	319	A00024	A01524	899
A00024	A01527	904	A00024	A01790	165
A00024	A02313	416	A00024	A02425	934
A00024	A02772	363	A00024	A02839	274
A00024	A02864	246	A00024	A02897	348
A00024	A04147	417	A00024	A06858	337
A00024	A09570	443	A00024	A12111	269
A00024	A13370	480	A00024	A14077	174
A00024	A14604	286	A00024	A15795	302
A00025	A00140	899	A00025	A00157	899
A00025	A00205	380	A00025	A00207	262
A00025	A00221	177	A00025	A00224	909
A00025	A00239	358	A00025	A00240	213
A00025	A00243	329	A00025	A00533	193
A00025	A01461	177	A00025	A01527	932
A00025	A01554	963	A00025	A01574	201
A00025	A05216	899	A00025	A07311	916
A00025	A07706	249	A00025	A09319	967
A00025	A09804	297	A00025	A13368	369
A00025	A13980	241	A00025	A13991	335
A00026	A00031	246	A00026	A00032	483
A00026	A00034	480	A00026	A00041	353
A00026	A00048	201	A00026	A00052	179
A00026	A00053	154	A00026	A00057	219
A00026	A00069	317	A00026	A00079	152
A00026	A00081	425	A00026	A00087	302
A00026	A00090	414	A00026	A00091	173
A00026	A00112	398	A00026	A00116	173
A00026	A00127	188	A00026	A00138	208
A00026	A00166	389	A00026	A00173	283
A00026	A00177	63	A00026	A00209	899

A00026	A00211	255	A00026	A00217	91
A00026	A00219	451	A00026	A00221	491
A00026	A00223	400	A00026	A00230	899
A00026	A00234	450	A00026	A00240	899
A00026	A00254	305	A00026	A00341	285
A00026	A00401	257	A00026	A00446	185
A00026	A00616	427	A00026	A00641	561
A00026	A00651	63	A00026	A00696	428
A00026	A00752	226	A00026	A00766	232
A00026	A00791	305	A00026	A00815	165
A00026	A00868	221	A00026	A00884	369
A00026	A00939	157	A00026	A00975	163
A00026	A01014	235	A00026	A01128	132
A00026	A01339	347	A00026	A01527	989
A00026	A01558	899	A00026	A01604	355
A00026	A01605	119	A00026	A01615	899
A00026	A01640	474	A00026	A01908	173
A00026	A02044	182	A00026	A02054	188
A00026	A02120	246	A00026	A02236	332
A00026	A02268	223	A00026	A02292	160
A00026	A02415	157	A00026	A02419	312
A00026	A02423	176	A00026	A02425	232
A00026	A02510	278	A00026	A02544	162
A00026	A02579	154	A00026	A02582	374
A00026	A02790	168	A00026	A03110	441
A00026	A03126	217	A00026	A03183	159
A00026	A03260	213	A00026	A03470	224
A00026	A03630	288	A00026	A03788	382
A00026	A03800	469	A00026	A03944	246
A00026	A04176	234	A00026	A04223	259
A00026	A04310	285	A00026	A04422	185
A00026	A04490	457	A00026	A04682	201
A00026	A04706	293	A00026	A04731	204
A00026	A04827	297	A00026	A05091	353
A00026	A05129	340	A00026	A05283	293
A00026	A05337	335	A00026	A05477	274
A00026	A05506	254	A00026	A05606	462
A00026	A05679	243	A00026	A05741	254
A00026	A06997	179	A00026	A07138	238
A00026	A07141	316	A00026	A07143	162
A00026	A07457	165	A00026	A08580	340
A00026	A08714	180	A00026	A08949	168
A00026	A08990	263	A00026	A09201	174
A00026	A09206	226	A00026	A09644	428
A00026	A10581	213	A00026	A10599	173
A00026	A10744	384	A00026	A11307	184

A00026	A11381	398	A00026	A11986	328
A00026	A12009	216	A00026	A12419	216
A00026	A12824	219	A00026	A13484	162
A00026	A13944	217	A00026	A13987	263
A00026	A14190	491	A00026	A14198	249
A00026	A14210	475	A00026	A14364	279
A00026	A14369	193	A00026	A14370	449
A00026	A14371	445	A00026	A14374	200
A00026	A14379	413	A00026	A14418	281
A00026	A14511	355	A00026	A14532	243
A00026	A14553	353	A00026	A15292	238
A00026	A15347	238	A00026	A15432	221
A00026	A15440	204	A00026	A15459	201
A00026	A15607	238	A00026	A15608	185
A00026	A15639	151	A00026	A15780	274
A00026	A15862	329	A00026	A15904	302
A00026	A16001	373	A00026	A16540	226
A00026	A16650	279	A00026	A16880	322
A00026	A16989	373	A00026	A17105	288
A00027	A00028	899	A00027	A00104	380
A00027	A00228	232	A00027	A00247	162
A00027	A00485	167	A00027	A01410	283
A00027	A02499	395	A00027	A02749	190
A00027	A09215	424	A00027	A10532	226
A00027	A10907	407	A00027	A13879	288
A00027	A14132	428	A00027	A15696	341
A00027	A16124	899	A00028	A16124	899
A00029	A00035	622	A00029	A00036	491
A00029	A00037	273	A00029	A00041	903
A00029	A00047	949	A00029	A00048	899
A00029	A00052	933	A00029	A00058	945
A00029	A00064	65	A00029	A00065	154
A00029	A00067	899	A00029	A00073	648
A00029	A00074	135	A00029	A00089	899
A00029	A00091	899	A00029	A00093	286
A00029	A00113	445	A00029	A00114	490
A00029	A00120	941	A00029	A00122	63
A00029	A00124	63	A00029	A00127	490
A00029	A00128	291	A00029	A00130	483
A00029	A00132	489	A00029	A00136	63
A00029	A00137	290	A00029	A00138	95
A00029	A00146	157	A00029	A00150	949
A00029	A00152	888	A00029	A00155	480
A00029	A00156	899	A00029	A00157	907
A00029	A00159	899	A00029	A00167	903
A00029	A00169	899	A00029	A00180	388

A00029	A00185	918	A00029	A00191	924
A00029	A00207	324	A00029	A00208	899
A00029	A00209	899	A00029	A00227	899
A00029	A00234	512	A00029	A00236	958
A00029	A00239	506	A00029	A00240	954
A00029	A00242	427	A00029	A00243	319
A00029	A00244	286	A00029	A00246	899
A00029	A00247	463	A00029	A00250	425
A00029	A00252	469	A00029	A00253	452
A00029	A00254	976	A00029	A00255	955
A00029	A00257	566	A00029	A00263	167
A00029	A00271	899	A00029	A00280	199
A00029	A00284	255	A00029	A00285	477
A00029	A00288	466	A00029	A00298	466
A00029	A00304	213	A00029	A00323	491
A00029	A00329	491	A00029	A00334	356
A00029	A00336	455	A00029	A00340	274
A00029	A00343	489	A00029	A00345	167
A00029	A00348	584	A00029	A00352	526
A00029	A00356	490	A00029	A00405	490
A00029	A00409	297	A00029	A00419	489
A00029	A00433	343	A00029	A00439	285
A00029	A00448	204	A00029	A00451	899
A00029	A00454	613	A00029	A00457	249
A00029	A00460	395	A00029	A00517	333
A00029	A00521	319	A00029	A00537	530
A00029	A00543	383	A00029	A00553	159
A00029	A00561	235	A00029	A00604	542
A00029	A00605	534	A00029	A00621	63
A00029	A00626	390	A00029	A00648	434
A00029	A00651	435	A00029	A00653	788
A00029	A00697	567	A00029	A00698	383
A00029	A00700	348	A00029	A00724	168
A00029	A00741	687	A00029	A00747	165
A00029	A00758	481	A00029	A00759	427
A00029	A00761	394	A00029	A00773	486
A00029	A00783	491	A00029	A00785	491
A00029	A00786	163	A00029	A00787	261
A00029	A00790	491	A00029	A00805	425
A00029	A00808	945	A00029	A00809	491
A00029	A00811	490	A00029	A00833	432
A00029	A00839	491	A00029	A00841	486
A00029	A00843	207	A00029	A00848	486
A00029	A00849	249	A00029	A00870	449
A00029	A00876	193	A00029	A00878	174
A00029	A00909	484	A00029	A00910	491

A00029	A00911	481	A00029	A00912	472
A00029	A00922	585	A00029	A00923	366
A00029	A00932	491	A00029	A00935	485
A00029	A00953	480	A00029	A00970	425
A00029	A00971	499	A00029	A01027	491
A00029	A01038	170	A00029	A01042	491
A00029	A01052	905	A00029	A01054	490
A00029	A01055	95	A00029	A01083	491
A00029	A01090	260	A00029	A01098	481
A00029	A01112	490	A00029	A01115	473
A00029	A01127	422	A00029	A01148	110
A00029	A01150	491	A00029	A01161	546
A00029	A01178	490	A00029	A01189	154
A00029	A01192	575	A00029	A01194	416
A00029	A01206	328	A00029	A01216	385
A00029	A01232	584	A00029	A01249	168
A00029	A01256	355	A00029	A01261	296
A00029	A01265	491	A00029	A01287	252
A00029	A01289	450	A00029	A01291	949
A00029	A01300	560	A00029	A01306	430
A00029	A01335	484	A00029	A01354	417
A00029	A01390	63	A00029	A01399	475
A00029	A01413	283	A00029	A01431	491
A00029	A01434	204	A00029	A01435	664
A00029	A01443	411	A00029	A01446	491
A00029	A01447	358	A00029	A01448	469
A00029	A01470	495	A00029	A01492	903
A00029	A01522	229	A00029	A01524	916
A00029	A01527	899	A00029	A01539	473
A00029	A01540	491	A00029	A01553	965
A00029	A01554	302	A00029	A01558	303
A00029	A01560	529	A00029	A01561	416
A00029	A01562	385	A00029	A01565	199
A00029	A01569	480	A00029	A01574	477
A00029	A01577	399	A00029	A01579	488
A00029	A01588	899	A00029	A01628	922
A00029	A01631	475	A00029	A01638	899
A00029	A01640	899	A00029	A01646	352
A00029	A01647	176	A00029	A01649	401
A00029	A01688	899	A00029	A01692	491
A00029	A01697	63	A00029	A01698	899
A00029	A01707	491	A00029	A01708	292
A00029	A01709	294	A00029	A01719	294
A00029	A01730	499	A00029	A01776	174
A00029	A01787	285	A00029	A01822	516
A00029	A01837	319	A00029	A01850	945

A00029	A01860	176	A00029	A02084	93
A00029	A02350	484	A00029	A02424	171
A00029	A02429	899	A00029	A02433	900
A00029	A02481	899	A00029	A02502	899
A00029	A02607	406	A00029	A02698	199
A00029	A02703	283	A00029	A02715	441
A00029	A02791	401	A00029	A02803	63
A00029	A02805	899	A00029	A02864	427
A00029	A02872	205	A00029	A02877	899
A00029	A03082	899	A00029	A03126	899
A00029	A03269	198	A00029	A03271	212
A00029	A03289	352	A00029	A03293	451
A00029	A03294	290	A00029	A03298	424
A00029	A03422	259	A00029	A03446	428
A00029	A03451	334	A00029	A03516	469
A00029	A03557	335	A00029	A03567	462
A00029	A03591	461	A00029	A03596	63
A00029	A03665	259	A00029	A03740	491
A00029	A03752	491	A00029	A03776	369
A00029	A03785	263	A00029	A03797	899
A00029	A03809	960	A00029	A03830	899
A00029	A03959	461	A00029	A03981	288
A00029	A04028	490	A00029	A04280	263
A00029	A04342	266	A00029	A04446	477
A00029	A04484	899	A00029	A04644	205
A00029	A04682	63	A00029	A04686	414
A00029	A04699	63	A00029	A04957	112
A00029	A05086	485	A00029	A05453	329
A00029	A05501	288	A00029	A05577	199
A00029	A05867	932	A00029	A05910	322
A00029	A05938	205	A00029	A05939	258
A00029	A06037	461	A00029	A06158	63
A00029	A06256	110	A00029	A06289	121
A00029	A06466	491	A00029	A06554	204
A00029	A06599	176	A00029	A06703	82
A00029	A06748	167	A00029	A06965	205
A00029	A07208	612	A00029	A07247	472
A00029	A07311	998	A00029	A07394	306
A00029	A07451	306	A00029	A07527	210
A00029	A07726	160	A00029	A07798	491
A00029	A08053	473	A00029	A08054	491
A00029	A08061	285	A00029	A08096	167
A00029	A08131	193	A00029	A08137	344
A00029	A08315	276	A00029	A08359	382
A00029	A08519	229	A00029	A08571	945
A00029	A08815	408	A00029	A08826	167

A00029	A08835	611	A00029	A08850	199
A00029	A08860	490	A00029	A08943	159
A00029	A08987	468	A00029	A09127	207
A00029	A09253	348	A00029	A09263	74
A00029	A09336	174	A00029	A09476	491
A00029	A09502	486	A00029	A09505	205
A00029	A09543	110	A00029	A09577	433
A00029	A09581	85	A00029	A09591	340
A00029	A09592	224	A00029	A09792	306
A00029	A09804	307	A00029	A09873	357
A00029	A09896	205	A00029	A09902	343
A00029	A09933	63	A00029	A10014	63
A00029	A10175	204	A00029	A10233	941
A00029	A10328	196	A00029	A10365	491
A00029	A10532	634	A00029	A10623	152
A00029	A10728	488	A00029	A10735	63
A00029	A10797	491	A00029	A10798	491
A00029	A10799	328	A00029	A10902	141
A00029	A10913	68	A00029	A10971	167
A00029	A11124	71	A00029	A11125	63
A00029	A11244	486	A00029	A11286	252
A00029	A11296	491	A00029	A11297	369
A00029	A11302	491	A00029	A11322	491
A00029	A11337	490	A00029	A11338	486
A00029	A11356	205	A00029	A11372	491
A00029	A11374	490	A00029	A11419	486
A00029	A11427	490	A00029	A11677	475
A00029	A11707	63	A00029	A11724	489
A00029	A11731	187	A00029	A11821	205
A00029	A11833	254	A00029	A11974	353
A00029	A11975	218	A00029	A12167	232
A00029	A12221	165	A00029	A12328	491
A00029	A12383	455	A00029	A12791	205
A00029	A12988	299	A00029	A12990	212
A00029	A13023	199	A00029	A13149	447
A00029	A13255	291	A00029	A13282	205
A00029	A13320	159	A00029	A13335	272
A00029	A13340	491	A00029	A13521	445
A00029	A13830	165	A00029	A13903	174
A00029	A13970	899	A00029	A13983	127
A00029	A13984	219	A00029	A13989	950
A00029	A14006	468	A00029	A14008	118
A00029	A14013	138	A00029	A14031	205
A00029	A14073	491	A00029	A14138	347
A00029	A14148	899	A00029	A14150	202
A00029	A14188	906	A00029	A14198	909

A00029	A14216	471	A00029	A14246	899
A00029	A14275	79	A00029	A14366	199
A00029	A14596	535	A00029	A14642	439
A00029	A14738	151	A00029	A14845	489
A00029	A15119	205	A00029	A15244	489
A00029	A15275	462	A00029	A15297	899
A00029	A15324	899	A00029	A15325	899
A00029	A15353	899	A00029	A15359	899
A00029	A15490	167	A00029	A15546	180
A00029	A15547	900	A00029	A15614	899
A00029	A15653	445	A00029	A15660	490
A00029	A15666	472	A00029	A15684	475
A00029	A15694	361	A00029	A15695	301
A00029	A15699	481	A00029	A15726	139
A00029	A15769	307	A00029	A15938	440
A00029	A16060	486	A00029	A16157	490
A00029	A16289	167	A00029	A16371	486
A00029	A16430	174	A00029	A16449	404
A00029	A16540	269	A00029	A16587	168
A00029	A16633	418	A00029	A16640	200
A00029	A16830	152	A00029	A16993	167
A00029	A17128	375	A00029	A17140	240
A00029	A17188	907	A00030	A00031	491
A00030	A00032	177	A00030	A00034	491
A00030	A00056	269	A00030	A00057	460
A00030	A00067	899	A00030	A00069	200
A00030	A00080	305	A00030	A00081	71
A00030	A00085	274	A00030	A00089	77
A00030	A00166	291	A00030	A00190	380
A00030	A00211	185	A00030	A00219	456
A00030	A00230	899	A00030	A00246	899
A00030	A00274	489	A00030	A00324	179
A00030	A00616	945	A00030	A00701	256
A00030	A00736	179	A00030	A01137	258
A00030	A01162	151	A00030	A01192	321
A00030	A01276	168	A00030	A01277	229
A00030	A01605	914	A00030	A01639	899
A00030	A01944	326	A00030	A01982	180
A00030	A02186	229	A00030	A02229	260
A00030	A02265	183	A00030	A02454	159
A00030	A02464	229	A00030	A02476	263
A00030	A02667	196	A00030	A02772	401
A00030	A02791	213	A00030	A02868	265
A00030	A02897	272	A00030	A02983	340
A00030	A03093	263	A00030	A03110	445
A00030	A03219	408	A00030	A03224	235

A00030	A03259	184	A00030	A03314	193
A00030	A03373	205	A00030	A03435	156
A00030	A03436	246	A00030	A03639	323
A00030	A03799	307	A00030	A03800	240
A00030	A03949	249	A00030	A04076	208
A00030	A04487	374	A00030	A04504	343
A00030	A04813	279	A00030	A05118	358
A00030	A05533	221	A00030	A06209	398
A00030	A06769	182	A00030	A06798	449
A00030	A07143	221	A00030	A07415	363
A00030	A07422	899	A00030	A07834	180
A00030	A08911	241	A00030	A10018	201
A00030	A10336	162	A00030	A10469	347
A00030	A10998	358	A00030	A11578	490
A00030	A12521	398	A00030	A13348	272
A00030	A13826	185	A00030	A13978	899
A00030	A14072	162	A00030	A14210	299
A00030	A14371	452	A00030	A14554	196
A00030	A15795	207	A00030	A16224	202
A00030	A16877	263	A00030	A17060	343
A00031	A00032	438	A00031	A00034	945
A00031	A00038	218	A00031	A00041	423
A00031	A00042	162	A00031	A00045	213
A00031	A00052	183	A00031	A00056	491
A00031	A00057	436	A00031	A00067	899
A00031	A00069	443	A00031	A00076	285
A00031	A00080	339	A00031	A00081	102
A00031	A00085	95	A00031	A00089	63
A00031	A00095	191	A00031	A00111	328
A00031	A00125	423	A00031	A00150	899
A00031	A00152	216	A00031	A00162	176
A00031	A00166	324	A00031	A00168	269
A00031	A00189	174	A00031	A00190	457
A00031	A00209	903	A00031	A00211	458
A00031	A00212	260	A00031	A00219	486
A00031	A00221	330	A00031	A00224	902
A00031	A00230	899	A00031	A00246	899
A00031	A00255	353	A00031	A00264	926
A00031	A00274	491	A00031	A00440	435
A00031	A00616	945	A00031	A00736	345
A00031	A01125	196	A00031	A01163	247
A00031	A01257	241	A00031	A01278	96
A00031	A01289	168	A00031	A01425	291
A00031	A01524	907	A00031	A01527	908
A00031	A01605	406	A00031	A01640	246
A00031	A01741	193	A00031	A01767	221

A00031	A01926	256	A00031	A02052	270
A00031	A02088	436	A00031	A02143	483
A00031	A02174	213	A00031	A02217	314
A00031	A02282	262	A00031	A02331	156
A00031	A02382	451	A00031	A02423	177
A00031	A02425	113	A00031	A02429	205
A00031	A02454	324	A00031	A02461	256
A00031	A02464	484	A00031	A02468	168
A00031	A02472	63	A00031	A02481	68
A00031	A02494	152	A00031	A02582	156
A00031	A02600	330	A00031	A02615	229
A00031	A02616	361	A00031	A02624	216
A00031	A02711	263	A00031	A02732	184
A00031	A02768	188	A00031	A02772	246
A00031	A02983	167	A00031	A03055	260
A00031	A03081	185	A00031	A03093	411
A00031	A03109	417	A00031	A03110	491
A00031	A03183	491	A00031	A03185	389
A00031	A03219	489	A00031	A03259	353
A00031	A03305	332	A00031	A03314	265
A00031	A03458	232	A00031	A03639	262
A00031	A03753	394	A00031	A03800	184
A00031	A03942	490	A00031	A03955	213
A00031	A03964	240	A00031	A04007	205
A00031	A04076	152	A00031	A04078	193
A00031	A04257	436	A00031	A04422	319
A00031	A04473	428	A00031	A04478	221
A00031	A04487	406	A00031	A04490	184
A00031	A04492	221	A00031	A04643	323
A00031	A04759	196	A00031	A05366	330
A00031	A05533	163	A00031	A05706	414
A00031	A06118	258	A00031	A06798	256
A00031	A07143	163	A00031	A07145	419
A00031	A07753	234	A00031	A09070	340
A00031	A09092	246	A00031	A09570	270
A00031	A10336	229	A00031	A11007	308
A00031	A11544	193	A00031	A11578	473
A00031	A11750	413	A00031	A11757	323
A00031	A11986	196	A00031	A12419	218
A00031	A13768	243	A00031	A13826	460
A00031	A13860	412	A00031	A13987	162
A00031	A14004	182	A00031	A14038	235
A00031	A14126	252	A00031	A14190	458
A00031	A14210	490	A00031	A14366	288
A00031	A14371	491	A00031	A14378	165
A00031	A14379	263	A00031	A14427	340

A00031	A14444	901	A00031	A14490	241
A00031	A14511	401	A00031	A14604	260
A00031	A14605	200	A00031	A15285	252
A00031	A15318	428	A00031	A15346	286
A00031	A15411	213	A00031	A15486	221
A00031	A15487	319	A00031	A15518	260
A00031	A15792	241	A00031	A15840	165
A00031	A15916	235	A00031	A16021	340
A00031	A16114	218	A00031	A16224	369
A00031	A17060	303	A00032	A00034	491
A00032	A00041	279	A00032	A00048	419
A00032	A00053	255	A00032	A00067	899
A00032	A00069	427	A00032	A00081	285
A00032	A00083	167	A00032	A00085	377
A00032	A00087	200	A00032	A00089	180
A00032	A00091	173	A00032	A00112	266
A00032	A00127	945	A00032	A00150	379
A00032	A00166	486	A00032	A00167	489
A00032	A00168	191	A00032	A00200	200
A00032	A00209	909	A00032	A00211	969
A00032	A00212	260	A00032	A00219	477
A00032	A00221	491	A00032	A00223	480
A00032	A00254	416	A00032	A00255	469
A00032	A00270	306	A00032	A00277	174
A00032	A00292	272	A00032	A00315	329
A00032	A00324	899	A00032	A00333	188
A00032	A00335	307	A00032	A00356	358
A00032	A00400	208	A00032	A00568	162
A00032	A00616	945	A00032	A00815	165
A00032	A00868	63	A00032	A00870	165
A00032	A01056	160	A00032	A01098	324
A00032	A01140	207	A00032	A01299	260
A00032	A01425	490	A00032	A01477	308
A00032	A01524	196	A00032	A01527	899
A00032	A01605	469	A00032	A01629	152
A00032	A01640	372	A00032	A02111	270
A00032	A02243	262	A00032	A02265	252
A00032	A02275	154	A00032	A02305	165
A00032	A02306	288	A00032	A02356	235
A00032	A02397	198	A00032	A02411	428
A00032	A02425	454	A00032	A02464	899
A00032	A02467	174	A00032	A02597	286
A00032	A02678	193	A00032	A02791	156
A00032	A03029	219	A00032	A03083	160
A00032	A03091	172	A00032	A03110	458
A00032	A03470	451	A00032	A03584	294

A00032	A03633	255	A00032	A03799	483
A00032	A03800	477	A00032	A03873	162
A00032	A03942	351	A00032	A04038	196
A00032	A04230	391	A00032	A04323	198
A00032	A04422	477	A00032	A04490	491
A00032	A04562	195	A00032	A04600	179
A00032	A04813	226	A00032	A04843	416
A00032	A04914	318	A00032	A05118	322
A00032	A05143	249	A00032	A05183	281
A00032	A05288	416	A00032	A05337	205
A00032	A05373	322	A00032	A05407	388
A00032	A05459	286	A00032	A05869	243
A00032	A06588	378	A00032	A06814	196
A00032	A07024	259	A00032	A07143	162
A00032	A07159	213	A00032	A07581	152
A00032	A08052	293	A00032	A08714	313
A00032	A08724	180	A00032	A08869	341
A00032	A09077	288	A00032	A09247	208
A00032	A09829	210	A00032	A10336	394
A00032	A11646	440	A00032	A12009	217
A00032	A13944	347	A00032	A14038	477
A00032	A14190	328	A00032	A14194	195
A00032	A14198	165	A00032	A14210	213
A00032	A14370	221	A00032	A14371	423
A00032	A14433	151	A00032	A14443	992
A00032	A14444	899	A00032	A14511	400
A00032	A15230	168	A00032	A15292	165
A00032	A15310	160	A00032	A15440	205
A00032	A15459	201	A00032	A15485	228
A00033	A00158	200	A00033	A00189	154
A00033	A00209	899	A00033	A00417	195
A00033	A00799	229	A00033	A01524	903
A00033	A01575	213	A00033	A01576	452
A00033	A02698	154	A00033	A03293	922
A00033	A08137	347	A00033	A08300	296
A00033	A11543	291	A00033	A15606	468
A00034	A00038	263	A00034	A00041	480
A00034	A00048	177	A00034	A00052	204
A00034	A00053	481	A00034	A00056	461
A00034	A00057	384	A00034	A00063	337
A00034	A00064	221	A00034	A00067	899
A00034	A00069	491	A00034	A00076	452
A00034	A00080	436	A00034	A00081	491
A00034	A00083	302	A00034	A00085	479
A00034	A00087	439	A00034	A00089	491
A00034	A00091	310	A00034	A00095	319

A00034	A00106	445	A00034	A00111	366
A00034	A00112	344	A00034	A00127	364
A00034	A00166	429	A00034	A00167	484
A00034	A00168	488	A00034	A00190	944
A00034	A00196	246	A00034	A00204	360
A00034	A00209	943	A00034	A00210	490
A00034	A00211	443	A00034	A00212	156
A00034	A00219	945	A00034	A00221	488
A00034	A00224	910	A00034	A00227	389
A00034	A00230	914	A00034	A00234	307
A00034	A00245	413	A00034	A00246	899
A00034	A00254	483	A00034	A00264	473
A00034	A00274	491	A00034	A00292	475
A00034	A00315	490	A00034	A00324	899
A00034	A00349	202	A00034	A00354	252
A00034	A00420	405	A00034	A00440	221
A00034	A00484	217	A00034	A00509	380
A00034	A00616	945	A00034	A00669	236
A00034	A00736	363	A00034	A00815	491
A00034	A00861	162	A00034	A00989	63
A00034	A01009	63	A00034	A01070	278
A00034	A01124	63	A00034	A01125	436
A00034	A01137	366	A00034	A01163	429
A00034	A01235	173	A00034	A01257	463
A00034	A01277	307	A00034	A01278	180
A00034	A01404	249	A00034	A01475	71
A00034	A01476	299	A00034	A01477	235
A00034	A01485	301	A00034	A01493	324
A00034	A01524	922	A00034	A01527	899
A00034	A01558	899	A00034	A01599	205
A00034	A01602	899	A00034	A01605	443
A00034	A01615	899	A00034	A01629	407
A00034	A01640	314	A00034	A01701	185
A00034	A01722	945	A00034	A01741	465
A00034	A01797	294	A00034	A01856	202
A00034	A01923	152	A00034	A01924	165
A00034	A01925	278	A00034	A01926	458
A00034	A01929	306	A00034	A01985	127
A00034	A02104	195	A00034	A02125	278
A00034	A02126	347	A00034	A02143	102
A00034	A02156	183	A00034	A02183	234
A00034	A02236	435	A00034	A02251	179
A00034	A02260	266	A00034	A02262	160
A00034	A02268	491	A00034	A02272	486
A00034	A02275	491	A00034	A02278	467
A00034	A02282	313	A00034	A02305	195

A00034	A02306	154	A00034	A02419	424
A00034	A02423	383	A00034	A02424	74
A00034	A02425	491	A00034	A02429	457
A00034	A02431	185	A00034	A02451	235
A00034	A02454	491	A00034	A02464	489
A00034	A02465	452	A00034	A02468	199
A00034	A02472	473	A00034	A02481	430
A00034	A02490	238	A00034	A02508	174
A00034	A02524	200	A00034	A02624	132
A00034	A02644	107	A00034	A02668	160
A00034	A02669	294	A00034	A02671	484
A00034	A02756	276	A00034	A02772	162
A00034	A02782	207	A00034	A02783	188
A00034	A02787	191	A00034	A02859	216
A00034	A02875	265	A00034	A03014	210
A00034	A03053	362	A00034	A03054	216
A00034	A03055	177	A00034	A03081	104
A00034	A03091	491	A00034	A03099	238
A00034	A03109	266	A00034	A03110	945
A00034	A03126	283	A00034	A03141	281
A00034	A03154	170	A00034	A03165	157
A00034	A03173	278	A00034	A03183	479
A00034	A03209	408	A00034	A03219	472
A00034	A03259	440	A00034	A03314	393
A00034	A03458	420	A00034	A03470	271
A00034	A03633	389	A00034	A03634	243
A00034	A03639	424	A00034	A03750	163
A00034	A03753	213	A00034	A03799	465
A00034	A03800	450	A00034	A03809	313
A00034	A03845	154	A00034	A03942	491
A00034	A03961	469	A00034	A04087	160
A00034	A04111	223	A00034	A04155	223
A00034	A04156	263	A00034	A04160	235
A00034	A04169	294	A00034	A04180	172
A00034	A04196	207	A00034	A04216	301
A00034	A04218	468	A00034	A04297	217
A00034	A04380	409	A00034	A04398	396
A00034	A04407	188	A00034	A04422	390
A00034	A04423	157	A00034	A04454	191
A00034	A04468	202	A00034	A04471	438
A00034	A04472	418	A00034	A04473	162
A00034	A04484	212	A00034	A04487	340
A00034	A04490	427	A00034	A04504	229
A00034	A04647	380	A00034	A04676	424
A00034	A04679	182	A00034	A04751	944
A00034	A04813	446	A00034	A05112	254

A00034	A05140	182	A00034	A05288	299
A00034	A05311	202	A00034	A05407	195
A00034	A05494	188	A00034	A05506	172
A00034	A05533	296	A00034	A05617	258
A00034	A05706	256	A00034	A05784	324
A00034	A05965	263	A00034	A06118	307
A00034	A06159	465	A00034	A06167	485
A00034	A06312	182	A00034	A06578	165
A00034	A06610	160	A00034	A07140	168
A00034	A07145	165	A00034	A07422	160
A00034	A07658	195	A00034	A07803	438
A00034	A08031	324	A00034	A08600	278
A00034	A08714	385	A00034	A08840	193
A00034	A09022	238	A00034	A09036	174
A00034	A09062	328	A00034	A09202	87
A00034	A09217	263	A00034	A09375	195
A00034	A09480	185	A00034	A09535	195
A00034	A09559	223	A00034	A09732	425
A00034	A09733	193	A00034	A10018	165
A00034	A10039	170	A00034	A10208	385
A00034	A10408	263	A00034	A10638	177
A00034	A10716	324	A00034	A10744	188
A00034	A11007	235	A00034	A11039	162
A00034	A11123	182	A00034	A11415	208
A00034	A11713	199	A00034	A11885	269
A00034	A12393	344	A00034	A12518	243
A00034	A13178	296	A00034	A13218	202
A00034	A13826	439	A00034	A13849	243
A00034	A13948	306	A00034	A13981	899
A00034	A13987	205	A00034	A14007	195
A00034	A14072	361	A00034	A14126	170
A00034	A14190	490	A00034	A14198	419
A00034	A14210	491	A00034	A14364	353
A00034	A14369	481	A00034	A14370	470
A00034	A14371	491	A00034	A14379	484
A00034	A14382	263	A00034	A14427	278
A00034	A14430	488	A00034	A14438	157
A00034	A14443	905	A00034	A14444	899
A00034	A14511	491	A00034	A14523	162
A00034	A14536	324	A00034	A14604	249
A00034	A14667	183	A00034	A15230	200
A00034	A15292	481	A00034	A15294	419
A00034	A15312	221	A00034	A15313	401
A00034	A15318	352	A00034	A15320	438
A00034	A15327	240	A00034	A15334	154
A00034	A15346	363	A00034	A15349	249

A00034	A15352	430	A00034	A15495	457
A00034	A15498	218	A00034	A15506	480
A00034	A15515	165	A00034	A15645	235
A00034	A15792	425	A00034	A15909	285
A00034	A16192	199	A00034	A16224	271
A00034	A17060	917	A00035	A00036	143
A00035	A00052	905	A00035	A00067	899
A00035	A00068	194	A00035	A00074	281
A00035	A00075	490	A00035	A00112	238
A00035	A00114	132	A00035	A00137	63
A00035	A00149	205	A00035	A00157	899
A00035	A00166	288	A00035	A00170	968
A00035	A00191	481	A00035	A00206	204
A00035	A00211	219	A00035	A00221	259
A00035	A00229	994	A00035	A00236	263
A00035	A00239	434	A00035	A00240	977
A00035	A00242	469	A00035	A00243	173
A00035	A00254	707	A00035	A00255	163
A00035	A00272	758	A00035	A00275	235
A00035	A00294	904	A00035	A00309	205
A00035	A00338	276	A00035	A00391	241
A00035	A00483	241	A00035	A00548	241
A00035	A00565	157	A00035	A00571	340
A00035	A00621	63	A00035	A00643	558
A00035	A00653	373	A00035	A00688	241
A00035	A00808	945	A00035	A00811	310
A00035	A00868	176	A00035	A00875	302
A00035	A00929	204	A00035	A00987	219
A00035	A01004	241	A00035	A01083	63
A00035	A01086	313	A00035	A01161	537
A00035	A01178	252	A00035	A01305	363
A00035	A01310	741	A00035	A01409	302
A00035	A01417	241	A00035	A01452	219
A00035	A01507	266	A00035	A01553	457
A00035	A01558	899	A00035	A01560	63
A00035	A01562	63	A00035	A01582	337
A00035	A01588	899	A00035	A01602	467
A00035	A01631	63	A00035	A01660	266
A00035	A01661	241	A00035	A01719	63
A00035	A01724	440	A00035	A01763	252
A00035	A02077	188	A00035	A02289	218
A00035	A02411	191	A00035	A02416	241
A00035	A02419	66	A00035	A02473	422
A00035	A02526	252	A00035	A02634	185
A00035	A02755	173	A00035	A02836	758
A00035	A03050	576	A00035	A03157	486

A00035	A03233	327	A00035	A03263	279
A00035	A03304	159	A00035	A03435	219
A00035	A03440	491	A00035	A03465	380
A00035	A03591	154	A00035	A04033	425
A00035	A04160	187	A00035	A04436	187
A00035	A04449	243	A00035	A04630	468
A00035	A05854	291	A00035	A06039	199
A00035	A07158	252	A00035	A07311	773
A00035	A07366	274	A00035	A07442	213
A00035	A07471	399	A00035	A07475	366
A00035	A07511	232	A00035	A07515	157
A00035	A08141	362	A00035	A08377	154
A00035	A08463	389	A00035	A08550	174
A00035	A08621	235	A00035	A08785	168
A00035	A08861	241	A00035	A09156	152
A00035	A09933	63	A00035	A10253	402
A00035	A10365	63	A00035	A10848	217
A00035	A11211	167	A00035	A11346	199
A00035	A11414	195	A00035	A11433	277
A00035	A11496	160	A00035	A11614	424
A00035	A11707	491	A00035	A11735	296
A00035	A11834	449	A00035	A11914	219
A00035	A11943	424	A00035	A11964	157
A00035	A11983	377	A00035	A12115	252
A00035	A12254	235	A00035	A12858	296
A00035	A12897	488	A00035	A13111	367
A00035	A13323	152	A00035	A13553	217
A00035	A13809	213	A00035	A13983	219
A00035	A14013	126	A00035	A14062	761
A00035	A14150	176	A00035	A14191	271
A00035	A14265	486	A00035	A14289	260
A00035	A14299	241	A00035	A14540	180
A00035	A14635	454	A00035	A14653	483
A00035	A14739	187	A00035	A15294	204
A00035	A15295	335	A00035	A15296	449
A00035	A15376	319	A00035	A15519	160
A00035	A15541	167	A00035	A15613	238
A00035	A15626	199	A00035	A15660	457
A00035	A15711	168	A00035	A16157	183
A00035	A16343	294	A00035	A16565	185
A00035	A16587	199	A00035	A16788	232
A00035	A16815	157	A00035	A16908	187
A00036	A00041	925	A00036	A00048	90
A00036	A00052	905	A00036	A00064	212
A00036	A00065	934	A00036	A00067	899
A00036	A00082	281	A00036	A00091	168

A00036	A00098	899	A00036	A00113	319
A00036	A00114	296	A00036	A00120	445
A00036	A00130	479	A00036	A00136	115
A00036	A00139	905	A00036	A00156	944
A00036	A00167	899	A00036	A00180	383
A00036	A00183	899	A00036	A00184	899
A00036	A00209	899	A00036	A00234	177
A00036	A00236	173	A00036	A00257	467
A00036	A00276	414	A00036	A00323	456
A00036	A00329	489	A00036	A00336	326
A00036	A00419	475	A00036	A00454	491
A00036	A00470	262	A00036	A00653	288
A00036	A00697	112	A00036	A00747	241
A00036	A00783	404	A00036	A00811	160
A00036	A00848	316	A00036	A00910	480
A00036	A00912	351	A00036	A01042	491
A00036	A01083	420	A00036	A01150	251
A00036	A01390	373	A00036	A01435	63
A00036	A01470	187	A00036	A01524	899
A00036	A01527	899	A00036	A01540	258
A00036	A01553	232	A00036	A01560	63
A00036	A01561	63	A00036	A01562	945
A00036	A01568	477	A00036	A01692	470
A00036	A01709	74	A00036	A01719	82
A00036	A01850	362	A00036	A02803	260
A00036	A02805	247	A00036	A03271	65
A00036	A03591	307	A00036	A03740	491
A00036	A03798	151	A00036	A03965	157
A00036	A04061	202	A00036	A04280	170
A00036	A04446	195	A00036	A04699	168
A00036	A04706	377	A00036	A04957	452
A00036	A05462	210	A00036	A05673	249
A00036	A05867	337	A00036	A06289	63
A00036	A07054	170	A00036	A07220	188
A00036	A07311	903	A00036	A07709	490
A00036	A08053	419	A00036	A08054	477
A00036	A08525	201	A00036	A09441	265
A00036	A09476	165	A00036	A09502	229
A00036	A09660	165	A00036	A10233	210
A00036	A10365	82	A00036	A10645	200
A00036	A10813	196	A00036	A11302	268
A00036	A12383	906	A00036	A12969	184
A00036	A12971	196	A00036	A12988	165
A00036	A13255	285	A00036	A13340	167
A00036	A13401	899	A00036	A13604	294
A00036	A13999	899	A00036	A14031	932

A00036	A14033	927	A00036	A14035	941
A00036	A14073	391	A00036	A14642	208
A00036	A15660	218	A00036	A15699	366
A00036	A15938	285	A00036	A16218	252
A00037	A00038	208	A00037	A00041	566
A00037	A00047	219	A00037	A00067	899
A00037	A00069	160	A00037	A00105	514
A00037	A00127	491	A00037	A00150	697
A00037	A00173	491	A00037	A00174	177
A00037	A00175	372	A00037	A00177	631
A00037	A00191	491	A00037	A00192	945
A00037	A00205	184	A00037	A00209	914
A00037	A00223	430	A00037	A00240	389
A00037	A00243	246	A00037	A00258	972
A00037	A00268	457	A00037	A00280	491
A00037	A00283	578	A00037	A00299	488
A00037	A00325	473	A00037	A00334	491
A00037	A00335	490	A00037	A00355	350
A00037	A00356	790	A00037	A00357	491
A00037	A00358	491	A00037	A00360	257
A00037	A00361	355	A00037	A00362	294
A00037	A00390	469	A00037	A00403	274
A00037	A00427	383	A00037	A00454	334
A00037	A00467	491	A00037	A00489	172
A00037	A00521	489	A00037	A00571	502
A00037	A00590	663	A00037	A00603	491
A00037	A00604	604	A00037	A00605	491
A00037	A00629	739	A00037	A00648	491
A00037	A00651	488	A00037	A00653	581
A00037	A00697	324	A00037	A00700	491
A00037	A00701	491	A00037	A00705	337
A00037	A00713	170	A00037	A00763	491
A00037	A00783	240	A00037	A00787	665
A00037	A00806	180	A00037	A00839	491
A00037	A00870	461	A00037	A00878	491
A00037	A00900	167	A00037	A00902	489
A00037	A00906	283	A00037	A00923	491
A00037	A00965	637	A00037	A00966	182
A00037	A00972	286	A00037	A00983	151
A00037	A00990	351	A00037	A01000	490
A00037	A01012	198	A00037	A01017	160
A00037	A01025	664	A00037	A01042	419
A00037	A01047	488	A00037	A01056	488
A00037	A01067	383	A00037	A01072	488
A00037	A01085	241	A00037	A01090	481
A00037	A01098	547	A00037	A01101	483

A00037	A01112	489	A00037	A01115	475
A00037	A01127	491	A00037	A01140	491
A00037	A01150	466	A00037	A01166	484
A00037	A01184	445	A00037	A01186	238
A00037	A01189	260	A00037	A01192	412
A00037	A01206	252	A00037	A01212	488
A00037	A01216	207	A00037	A01234	445
A00037	A01238	160	A00037	A01240	63
A00037	A01249	665	A00037	A01291	988
A00037	A01292	288	A00037	A01294	212
A00037	A01306	578	A00037	A01335	514
A00037	A01360	165	A00037	A01392	605
A00037	A01425	560	A00037	A01431	357
A00037	A01433	383	A00037	A01436	450
A00037	A01437	485	A00037	A01457	485
A00037	A01458	441	A00037	A01464	483
A00037	A01468	491	A00037	A01470	462
A00037	A01478	160	A00037	A01492	469
A00037	A01496	475	A00037	A01540	451
A00037	A01569	466	A00037	A01579	491
A00037	A01586	491	A00037	A01601	749
A00037	A01605	369	A00037	A01617	394
A00037	A01618	63	A00037	A01649	486
A00037	A01718	945	A00037	A02350	398
A00037	A02597	165	A00037	A02601	290
A00037	A03048	85	A00037	A03069	479
A00037	A03171	63	A00037	A03304	290
A00037	A03331	168	A00037	A03332	489
A00037	A03437	441	A00037	A03461	180
A00037	A03531	462	A00037	A03567	582
A00037	A03570	435	A00037	A03705	451
A00037	A03740	422	A00037	A03754	395
A00037	A03885	165	A00037	A03913	221
A00037	A03939	452	A00037	A03981	441
A00037	A04099	457	A00037	A04754	384
A00037	A04761	485	A00037	A04827	180
A00037	A05044	212	A00037	A05121	165
A00037	A05411	204	A00037	A05443	714
A00037	A05501	475	A00037	A05996	452
A00037	A06279	486	A00037	A06351	414
A00037	A06428	452	A00037	A06814	200
A00037	A06861	565	A00037	A06965	491
A00037	A06973	346	A00037	A07243	154
A00037	A07323	290	A00037	A07398	682
A00037	A07449	317	A00037	A07451	491
A00037	A07968	212	A00037	A08011	654

A00037	A08055	395	A00037	A08357	424
A00037	A08359	332	A00037	A08398	414
A00037	A08452	479	A00037	A08547	260
A00037	A08717	160	A00037	A08773	199
A00037	A08972	228	A00037	A09282	297
A00037	A09322	246	A00037	A09332	457
A00037	A09581	317	A00037	A09722	224
A00037	A09852	243	A00037	A10234	224
A00037	A10599	389	A00037	A10616	283
A00037	A10949	419	A00037	A11190	249
A00037	A11390	382	A00037	A11400	283
A00037	A11438	468	A00037	A11480	191
A00037	A11704	302	A00037	A12079	193
A00037	A12433	154	A00037	A12643	302
A00037	A12651	269	A00037	A12653	443
A00037	A12925	174	A00037	A12990	395
A00037	A13249	440	A00037	A13549	212
A00037	A13989	548	A00037	A13992	491
A00037	A14029	321	A00037	A14172	411
A00037	A14482	191	A00037	A14505	358
A00037	A15221	157	A00037	A15660	490
A00037	A15684	490	A00037	A15894	491
A00037	A15988	490	A00037	A16118	490
A00037	A16159	334	A00037	A16418	279
A00037	A16631	254	A00037	A17095	187
A00038	A00039	928	A00038	A00150	340
A00038	A00209	246	A00038	A00258	374
A00038	A00292	159	A00038	A00306	196
A00038	A00324	243	A00038	A00642	180
A00038	A00643	157	A00038	A00736	257
A00038	A01002	899	A00038	A01605	486
A00038	A01640	380	A00038	A01697	184
A00038	A01718	290	A00038	A02984	208
A00038	A03281	393	A00038	A03512	199
A00038	A03600	246	A00038	A03929	299
A00038	A06240	152	A00038	A10460	299
A00038	A14025	241	A00038	A15649	273
A00038	A16420	348	A00038	A16766	162
A00039	A00210	374	A00039	A00230	313
A00039	A00347	173	A00039	A01002	899
A00039	A01296	291	A00039	A01315	201
A00039	A01325	379	A00039	A01596	217
A00039	A01605	160	A00039	A01697	224
A00039	A02797	291	A00039	A02908	230
A00039	A05986	165	A00039	A09524	377
A00039	A09785	341	A00039	A10580	458

A00039	A15649	316	A00040	A00041	469
A00040	A00042	205	A00040	A00043	232
A00040	A00045	475	A00040	A00052	312
A00040	A00055	195	A00040	A00057	165
A00040	A00061	208	A00040	A00062	441
A00040	A00063	404	A00040	A00064	156
A00040	A00067	199	A00040	A00068	199
A00040	A00073	113	A00040	A00074	488
A00040	A00081	414	A00040	A00123	340
A00040	A00127	486	A00040	A00141	243
A00040	A00149	366	A00040	A00152	491
A00040	A00156	277	A00040	A00157	63
A00040	A00159	491	A00040	A00160	907
A00040	A00161	951	A00040	A00162	63
A00040	A00180	160	A00040	A00184	334
A00040	A00190	486	A00040	A00204	291
A00040	A00208	343	A00040	A00213	341
A00040	A00214	351	A00040	A00219	313
A00040	A00224	527	A00040	A00225	245
A00040	A00232	491	A00040	A00233	468
A00040	A00234	193	A00040	A00254	491
A00040	A00310	296	A00040	A00324	210
A00040	A00335	360	A00040	A00356	491
A00040	A00440	241	A00040	A00450	185
A00040	A00495	489	A00040	A00556	463
A00040	A00557	260	A00040	A00691	260
A00040	A00736	491	A00040	A00747	462
A00040	A00799	384	A00040	A00812	152
A00040	A00820	483	A00040	A00878	195
A00040	A00914	489	A00040	A00926	167
A00040	A00993	480	A00040	A01046	151
A00040	A01075	185	A00040	A01082	167
A00040	A01164	355	A00040	A01209	205
A00040	A01213	236	A00040	A01239	185
A00040	A01286	257	A00040	A01307	480
A00040	A01396	399	A00040	A01452	194
A00040	A01468	258	A00040	A01473	480
A00040	A01527	899	A00040	A01557	236
A00040	A01558	199	A00040	A01559	236
A00040	A01572	491	A00040	A01592	158
A00040	A01668	474	A00040	A01676	269
A00040	A01697	179	A00040	A01718	177
A00040	A01726	491	A00040	A01741	352
A00040	A01743	462	A00040	A01749	63
A00040	A01769	452	A00040	A01775	241
A00040	A01789	63	A00040	A01796	218

A00040	A01799	63	A00040	A01844	488
A00040	A01846	350	A00040	A01847	483
A00040	A01887	63	A00040	A01888	240
A00040	A01900	491	A00040	A01941	485
A00040	A01959	240	A00040	A02011	374
A00040	A02086	491	A00040	A02114	177
A00040	A02226	382	A00040	A02250	314
A00040	A02331	490	A00040	A02345	152
A00040	A02377	489	A00040	A02380	235
A00040	A02481	480	A00040	A02491	451
A00040	A02513	347	A00040	A02521	468
A00040	A02535	401	A00040	A02564	294
A00040	A02637	213	A00040	A02639	383
A00040	A02650	202	A00040	A02651	162
A00040	A02689	210	A00040	A02690	180
A00040	A02697	162	A00040	A02704	406
A00040	A02710	63	A00040	A02717	489
A00040	A02731	343	A00040	A02735	430
A00040	A02739	441	A00040	A02745	63
A00040	A02753	418	A00040	A02754	174
A00040	A02770	324	A00040	A02777	486
A00040	A02807	401	A00040	A02808	157
A00040	A02825	63	A00040	A02828	491
A00040	A02837	288	A00040	A02847	317
A00040	A02856	424	A00040	A02864	479
A00040	A02869	441	A00040	A02872	489
A00040	A02877	296	A00040	A02882	486
A00040	A02883	157	A00040	A02888	477
A00040	A02896	237	A00040	A02897	322
A00040	A02908	491	A00040	A02918	491
A00040	A02919	417	A00040	A02925	481
A00040	A02929	447	A00040	A02941	228
A00040	A02942	475	A00040	A02947	213
A00040	A02948	329	A00040	A02961	269
A00040	A02968	486	A00040	A02975	196
A00040	A02976	307	A00040	A02982	480
A00040	A02983	477	A00040	A03001	95
A00040	A03019	491	A00040	A03223	472
A00040	A03259	486	A00040	A03287	326
A00040	A03321	491	A00040	A03335	358
A00040	A03611	475	A00040	A03742	458
A00040	A03803	489	A00040	A03821	480
A00040	A03837	218	A00040	A03965	358
A00040	A04020	490	A00040	A04115	260
A00040	A04117	486	A00040	A04139	177
A00040	A04161	489	A00040	A04207	424

A00040	A04362	173	A00040	A04446	228
A00040	A04540	480	A00040	A04553	477
A00040	A04568	477	A00040	A04600	246
A00040	A04622	419	A00040	A04809	204
A00040	A04867	475	A00040	A04902	491
A00040	A04908	238	A00040	A04938	204
A00040	A05008	177	A00040	A05015	242
A00040	A05114	291	A00040	A05280	165
A00040	A05410	167	A00040	A05428	461
A00040	A05443	324	A00040	A05456	202
A00040	A05568	477	A00040	A05570	213
A00040	A05584	193	A00040	A05591	418
A00040	A05760	479	A00040	A05767	213
A00040	A05817	199	A00040	A05832	207
A00040	A05853	490	A00040	A05876	419
A00040	A05878	245	A00040	A05879	401
A00040	A05958	238	A00040	A06004	172
A00040	A06094	255	A00040	A06131	297
A00040	A06190	401	A00040	A06339	260
A00040	A06414	191	A00040	A06416	205
A00040	A06438	202	A00040	A06591	339
A00040	A06610	491	A00040	A06631	263
A00040	A06642	475	A00040	A06644	461
A00040	A06971	224	A00040	A07135	360
A00040	A07140	234	A00040	A07157	63
A00040	A07164	167	A00040	A07165	484
A00040	A07186	132	A00040	A07201	430
A00040	A07218	379	A00040	A07242	486
A00040	A07311	199	A00040	A07483	475
A00040	A07486	241	A00040	A07488	202
A00040	A07570	240	A00040	A07593	491
A00040	A07900	484	A00040	A07961	414
A00040	A08004	202	A00040	A08133	199
A00040	A08193	199	A00040	A08402	157
A00040	A08714	251	A00040	A08732	486
A00040	A08735	202	A00040	A08917	296
A00040	A09242	384	A00040	A09488	165
A00040	A09535	162	A00040	A09559	199
A00040	A09766	441	A00040	A09988	489
A00040	A09999	360	A00040	A10159	195
A00040	A10247	480	A00040	A10431	184
A00040	A10601	218	A00040	A10626	235
A00040	A10791	172	A00040	A10798	236
A00040	A10823	102	A00040	A11248	202
A00040	A11369	447	A00040	A11546	311
A00040	A11604	199	A00040	A11639	165

A00040	A11750	154	A00040	A11964	177
A00040	A12111	404	A00040	A12365	274
A00040	A12544	202	A00040	A12546	202
A00040	A12547	202	A00040	A12717	165
A00040	A12902	240	A00040	A13202	202
A00040	A13212	423	A00040	A13615	202
A00040	A13734	152	A00040	A13824	183
A00040	A13829	311	A00040	A13869	352
A00040	A13983	185	A00040	A13989	427
A00040	A14197	469	A00040	A14279	160
A00040	A14346	165	A00040	A14431	276
A00040	A14540	152	A00040	A14570	335
A00040	A14575	340	A00040	A14652	235
A00040	A14886	402	A00040	A15296	358
A00040	A15356	490	A00040	A15532	171
A00040	A15613	385	A00040	A15962	180
A00040	A16012	202	A00040	A16453	205
A00041	A00042	985	A00041	A00043	491
A00041	A00044	491	A00041	A00045	491
A00041	A00046	945	A00041	A00047	899
A00041	A00048	466	A00041	A00050	395
A00041	A00052	945	A00041	A00053	334
A00041	A00055	190	A00041	A00056	480
A00041	A00057	353	A00041	A00058	439
A00041	A00061	414	A00041	A00062	491
A00041	A00063	491	A00041	A00064	971
A00041	A00066	899	A00041	A00067	951
A00041	A00068	152	A00041	A00069	441
A00041	A00071	486	A00041	A00073	945
A00041	A00074	945	A00041	A00075	463
A00041	A00079	185	A00041	A00081	479
A00041	A00083	899	A00041	A00085	361
A00041	A00087	458	A00041	A00089	435
A00041	A00090	491	A00041	A00091	461
A00041	A00094	945	A00041	A00099	899
A00041	A00106	217	A00041	A00109	334
A00041	A00112	217	A00041	A00118	285
A00041	A00122	491	A00041	A00123	362
A00041	A00126	899	A00041	A00127	491
A00041	A00132	480	A00041	A00133	899
A00041	A00135	452	A00041	A00136	266
A00041	A00138	626	A00041	A00140	899
A00041	A00141	945	A00041	A00145	405
A00041	A00146	205	A00041	A00147	475
A00041	A00152	977	A00041	A00153	488
A00041	A00154	283	A00041	A00156	486

A00041	A00157	899	A00041	A00158	390
A00041	A00159	578	A00041	A00160	391
A00041	A00161	490	A00041	A00162	961
A00041	A00163	945	A00041	A00165	899
A00041	A00166	238	A00041	A00167	954
A00041	A00168	468	A00041	A00169	473
A00041	A00171	472	A00041	A00173	945
A00041	A00183	262	A00041	A00184	900
A00041	A00185	945	A00041	A00186	147
A00041	A00188	936	A00041	A00189	82
A00041	A00190	945	A00041	A00191	899
A00041	A00192	417	A00041	A00193	196
A00041	A00196	490	A00041	A00204	405
A00041	A00205	63	A00041	A00207	960
A00041	A00208	491	A00041	A00209	909
A00041	A00211	190	A00041	A00212	401
A00041	A00214	950	A00041	A00215	290
A00041	A00216	491	A00041	A00219	952
A00041	A00220	455	A00041	A00221	582
A00041	A00224	958	A00041	A00225	274
A00041	A00227	391	A00041	A00228	312
A00041	A00230	942	A00041	A00231	450
A00041	A00232	481	A00041	A00233	491
A00041	A00234	953	A00041	A00236	899
A00041	A00238	205	A00041	A00240	188
A00041	A00246	945	A00041	A00248	485
A00041	A00252	899	A00041	A00254	945
A00041	A00255	901	A00041	A00257	456
A00041	A00262	357	A00041	A00264	491
A00041	A00265	491	A00041	A00272	457
A00041	A00274	485	A00041	A00292	425
A00041	A00294	422	A00041	A00305	454
A00041	A00309	424	A00041	A00310	218
A00041	A00312	469	A00041	A00316	479
A00041	A00324	944	A00041	A00333	285
A00041	A00356	491	A00041	A00358	899
A00041	A00370	422	A00041	A00375	430
A00041	A00377	170	A00041	A00381	347
A00041	A00436	154	A00041	A00457	488
A00041	A00517	159	A00041	A00525	327
A00041	A00552	446	A00041	A00556	475
A00041	A00557	152	A00041	A00558	481
A00041	A00574	352	A00041	A00601	229
A00041	A00616	475	A00041	A00641	484
A00041	A00643	152	A00041	A00650	319
A00041	A00654	254	A00041	A00660	157

A00041	A00671	162	A00041	A00696	217
A00041	A00719	157	A00041	A00722	380
A00041	A00728	187	A00041	A00736	491
A00041	A00747	491	A00041	A00752	414
A00041	A00761	314	A00041	A00791	208
A00041	A00799	238	A00041	A00815	191
A00041	A00820	489	A00041	A00826	416
A00041	A00832	461	A00041	A00866	402
A00041	A00868	428	A00041	A00883	490
A00041	A00887	368	A00041	A00914	491
A00041	A00926	393	A00041	A00930	455
A00041	A00939	953	A00041	A00961	441
A00041	A00981	319	A00041	A00985	213
A00041	A00993	378	A00041	A01009	260
A00041	A01022	200	A00041	A01052	914
A00041	A01070	903	A00041	A01102	223
A00041	A01125	457	A00041	A01127	439
A00041	A01137	224	A00041	A01140	468
A00041	A01142	307	A00041	A01144	265
A00041	A01169	235	A00041	A01172	223
A00041	A01178	324	A00041	A01212	411
A00041	A01257	438	A00041	A01275	154
A00041	A01277	243	A00041	A01291	949
A00041	A01307	491	A00041	A01317	162
A00041	A01318	332	A00041	A01320	285
A00041	A01324	291	A00041	A01339	477
A00041	A01396	486	A00041	A01409	254
A00041	A01422	344	A00041	A01423	252
A00041	A01438	460	A00041	A01450	276
A00041	A01452	572	A00041	A01468	195
A00041	A01473	486	A00041	A01492	899
A00041	A01499	468	A00041	A01501	278
A00041	A01509	172	A00041	A01512	922
A00041	A01515	166	A00041	A01524	974
A00041	A01527	974	A00041	A01548	163
A00041	A01553	954	A00041	A01554	933
A00041	A01555	213	A00041	A01558	919
A00041	A01560	959	A00041	A01561	899
A00041	A01562	929	A00041	A01572	491
A00041	A01573	467	A00041	A01576	620
A00041	A01588	899	A00041	A01593	899
A00041	A01602	945	A00041	A01604	475
A00041	A01606	424	A00041	A01615	899
A00041	A01626	467	A00041	A01628	185
A00041	A01638	899	A00041	A01639	920
A00041	A01640	382	A00041	A01651	899

A00041	A01653	302	A00041	A01654	463
A00041	A01668	491	A00041	A01675	63
A00041	A01676	488	A00041	A01677	486
A00041	A01678	259	A00041	A01683	199
A00041	A01691	195	A00041	A01692	934
A00041	A01702	436	A00041	A01704	278
A00041	A01706	581	A00041	A01708	443
A00041	A01709	440	A00041	A01710	263
A00041	A01719	283	A00041	A01726	489
A00041	A01727	430	A00041	A01729	432
A00041	A01730	489	A00041	A01731	290
A00041	A01733	677	A00041	A01737	455
A00041	A01740	162	A00041	A01741	491
A00041	A01742	167	A00041	A01743	491
A00041	A01749	296	A00041	A01754	279
A00041	A01759	457	A00041	A01767	236
A00041	A01770	216	A00041	A01775	413
A00041	A01781	443	A00041	A01792	452
A00041	A01796	490	A00041	A01797	491
A00041	A01798	488	A00041	A01813	357
A00041	A01830	198	A00041	A01844	491
A00041	A01847	238	A00041	A01850	168
A00041	A01870	481	A00041	A01876	373
A00041	A01879	473	A00041	A01880	98
A00041	A01887	399	A00041	A01888	455
A00041	A01890	200	A00041	A01894	470
A00041	A01897	260	A00041	A01900	484
A00041	A01902	477	A00041	A01903	486
A00041	A01905	252	A00041	A01908	491
A00041	A01914	428	A00041	A01920	271
A00041	A01926	238	A00041	A01941	260
A00041	A01978	385	A00041	A01983	360
A00041	A02008	165	A00041	A02010	371
A00041	A02011	407	A00041	A02049	154
A00041	A02054	450	A00041	A02056	260
A00041	A02076	318	A00041	A02083	215
A00041	A02088	272	A00041	A02117	443
A00041	A02133	156	A00041	A02138	187
A00041	A02143	201	A00041	A02148	234
A00041	A02158	328	A00041	A02180	205
A00041	A02183	491	A00041	A02208	350
A00041	A02217	165	A00041	A02224	159
A00041	A02226	441	A00041	A02228	185
A00041	A02254	347	A00041	A02268	252
A00041	A02278	352	A00041	A02303	200
A00041	A02304	308	A00041	A02306	462

A00041	A02337	159	A00041	A02345	63
A00041	A02353	352	A00041	A02361	215
A00041	A02367	156	A00041	A02369	269
A00041	A02370	212	A00041	A02377	347
A00041	A02382	491	A00041	A02411	174
A00041	A02416	319	A00041	A02425	457
A00041	A02436	274	A00041	A02454	288
A00041	A02481	433	A00041	A02488	201
A00041	A02491	312	A00041	A02502	230
A00041	A02553	177	A00041	A02564	413
A00041	A02578	439	A00041	A02630	440
A00041	A02632	219	A00041	A02635	473
A00041	A02636	279	A00041	A02637	443
A00041	A02638	324	A00041	A02639	475
A00041	A02654	267	A00041	A02659	407
A00041	A02672	251	A00041	A02695	347
A00041	A02698	195	A00041	A02699	247
A00041	A02705	162	A00041	A02706	491
A00041	A02709	388	A00041	A02711	162
A00041	A02714	138	A00041	A02715	490
A00041	A02718	213	A00041	A02731	229
A00041	A02732	63	A00041	A02735	283
A00041	A02739	491	A00041	A02740	243
A00041	A02745	288	A00041	A02746	63
A00041	A02747	177	A00041	A02749	491
A00041	A02753	193	A00041	A02759	193
A00041	A02768	480	A00041	A02770	319
A00041	A02787	218	A00041	A02788	224
A00041	A02801	156	A00041	A02803	945
A00041	A02808	446	A00041	A02816	335
A00041	A02828	360	A00041	A02830	454
A00041	A02834	156	A00041	A02836	469
A00041	A02837	489	A00041	A02842	159
A00041	A02843	172	A00041	A02854	213
A00041	A02856	172	A00041	A02859	582
A00041	A02864	486	A00041	A02872	236
A00041	A02874	469	A00041	A02877	414
A00041	A02887	395	A00041	A02896	504
A00041	A02902	491	A00041	A02908	165
A00041	A02918	314	A00041	A02927	323
A00041	A02933	281	A00041	A02936	263
A00041	A02944	212	A00041	A02945	340
A00041	A02970	455	A00041	A02982	355
A00041	A02983	440	A00041	A02991	190
A00041	A03006	319	A00041	A03010	195
A00041	A03019	422	A00041	A03034	262

A00041	A03050	200	A00041	A03076	177
A00041	A03084	185	A00041	A03095	235
A00041	A03099	473	A00041	A03110	486
A00041	A03112	491	A00041	A03121	361
A00041	A03134	270	A00041	A03157	234
A00041	A03171	472	A00041	A03174	177
A00041	A03183	408	A00041	A03186	188
A00041	A03204	256	A00041	A03209	319
A00041	A03213	347	A00041	A03219	357
A00041	A03223	402	A00041	A03224	456
A00041	A03225	337	A00041	A03243	252
A00041	A03259	491	A00041	A03263	355
A00041	A03270	162	A00041	A03271	960
A00041	A03275	484	A00041	A03280	566
A00041	A03327	457	A00041	A03332	474
A00041	A03451	477	A00041	A03452	472
A00041	A03477	386	A00041	A03520	375
A00041	A03523	162	A00041	A03570	489
A00041	A03587	63	A00041	A03591	932
A00041	A03596	270	A00041	A03598	63
A00041	A03609	274	A00041	A03610	235
A00041	A03611	465	A00041	A03618	486
A00041	A03626	204	A00041	A03639	899
A00041	A03655	156	A00041	A03657	217
A00041	A03661	389	A00041	A03722	314
A00041	A03725	480	A00041	A03726	899
A00041	A03732	154	A00041	A03739	380
A00041	A03799	344	A00041	A03803	200
A00041	A03809	944	A00041	A03815	393
A00041	A03817	491	A00041	A03818	324
A00041	A03821	490	A00041	A03843	422
A00041	A03846	408	A00041	A03851	180
A00041	A03887	185	A00041	A03889	406
A00041	A03894	260	A00041	A03904	202
A00041	A03910	177	A00041	A03922	243
A00041	A03942	451	A00041	A03961	294
A00041	A03965	154	A00041	A03990	470
A00041	A04010	348	A00041	A04015	224
A00041	A04038	305	A00041	A04040	198
A00041	A04050	587	A00041	A04051	409
A00041	A04062	227	A00041	A04078	168
A00041	A04117	945	A00041	A04121	165
A00041	A04130	210	A00041	A04138	251
A00041	A04160	154	A00041	A04161	468
A00041	A04238	195	A00041	A04248	398
A00041	A04264	400	A00041	A04287	156

A00041	A04292	432	A00041	A04293	154
A00041	A04300	156	A00041	A04342	232
A00041	A04426	156	A00041	A04442	241
A00041	A04476	156	A00041	A04536	469
A00041	A04540	409	A00041	A04568	216
A00041	A04587	177	A00041	A04589	306
A00041	A04611	385	A00041	A04613	177
A00041	A04622	491	A00041	A04699	899
A00041	A04730	177	A00041	A04761	195
A00041	A04814	165	A00041	A04879	483
A00041	A04880	428	A00041	A04902	251
A00041	A04969	217	A00041	A05020	352
A00041	A05024	261	A00041	A05037	912
A00041	A05044	160	A00041	A05054	165
A00041	A05082	903	A00041	A05107	163
A00041	A05122	258	A00041	A05174	263
A00041	A05200	247	A00041	A05202	247
A00041	A05216	491	A00041	A05221	486
A00041	A05280	243	A00041	A05283	224
A00041	A05288	491	A00041	A05309	440
A00041	A05336	156	A00041	A05421	156
A00041	A05443	662	A00041	A05446	157
A00041	A05490	299	A00041	A05656	156
A00041	A05750	215	A00041	A05795	324
A00041	A05816	188	A00041	A05818	156
A00041	A05854	899	A00041	A05867	916
A00041	A05944	353	A00041	A05985	229
A00041	A06001	195	A00041	A06037	246
A00041	A06039	199	A00041	A06054	267
A00041	A06055	252	A00041	A06190	227
A00041	A06197	154	A00041	A06251	154
A00041	A06289	199	A00041	A06294	156
A00041	A06298	452	A00041	A06359	352
A00041	A06410	177	A00041	A06415	446
A00041	A06416	63	A00041	A06428	366
A00041	A06449	195	A00041	A06465	215
A00041	A06491	345	A00041	A06521	154
A00041	A06581	491	A00041	A06588	152
A00041	A06595	219	A00041	A06597	491
A00041	A06607	396	A00041	A06608	486
A00041	A06610	488	A00041	A06615	213
A00041	A06623	484	A00041	A06648	281
A00041	A06733	157	A00041	A06769	364
A00041	A06849	195	A00041	A06854	154
A00041	A06872	156	A00041	A06949	296
A00041	A07070	156	A00041	A07118	243

A00041	A07127	156	A00041	A07135	945
A00041	A07145	348	A00041	A07148	274
A00041	A07151	296	A00041	A07152	240
A00041	A07157	486	A00041	A07158	455
A00041	A07161	428	A00041	A07163	156
A00041	A07164	575	A00041	A07165	488
A00041	A07166	483	A00041	A07173	445
A00041	A07180	226	A00041	A07200	468
A00041	A07201	406	A00041	A07209	335
A00041	A07240	156	A00041	A07311	954
A00041	A07339	483	A00041	A07388	156
A00041	A07422	899	A00041	A07461	307
A00041	A07574	154	A00041	A07622	195
A00041	A07643	352	A00041	A07771	279
A00041	A07900	167	A00041	A07935	160
A00041	A07942	340	A00041	A07999	234
A00041	A08338	156	A00041	A08413	156
A00041	A08418	286	A00041	A08563	167
A00041	A08597	156	A00041	A08608	345
A00041	A08611	156	A00041	A08616	156
A00041	A08692	126	A00041	A08697	156
A00041	A08707	477	A00041	A08821	314
A00041	A08836	204	A00041	A08837	346
A00041	A08860	549	A00041	A08880	171
A00041	A08896	312	A00041	A08902	899
A00041	A08972	260	A00041	A09003	247
A00041	A09018	256	A00041	A09050	297
A00041	A09124	215	A00041	A09126	173
A00041	A09137	156	A00041	A09218	230
A00041	A09220	472	A00041	A09401	63
A00041	A09405	156	A00041	A09406	165
A00041	A09509	232	A00041	A09556	314
A00041	A09568	899	A00041	A09804	302
A00041	A09823	156	A00041	A09833	430
A00041	A09841	156	A00041	A09842	485
A00041	A10006	198	A00041	A10159	899
A00041	A10174	441	A00041	A10336	455
A00041	A10368	156	A00041	A10392	899
A00041	A10464	156	A00041	A10519	154
A00041	A10530	195	A00041	A10537	156
A00041	A10556	353	A00041	A10580	915
A00041	A10596	159	A00041	A10611	302
A00041	A10623	899	A00041	A10625	288
A00041	A10735	475	A00041	A10739	208
A00041	A10740	288	A00041	A10801	918
A00041	A10813	899	A00041	A10854	352

A00041	A10870	177	A00041	A11079	199
A00041	A11133	243	A00041	A11369	963
A00041	A11405	491	A00041	A11431	185
A00041	A11474	174	A00041	A11487	170
A00041	A11526	172	A00041	A11604	899
A00041	A11614	290	A00041	A11646	167
A00041	A11715	268	A00041	A11789	156
A00041	A11801	156	A00041	A11921	899
A00041	A11943	411	A00041	A11978	156
A00041	A11983	899	A00041	A12009	154
A00041	A12101	379	A00041	A12255	256
A00041	A12329	223	A00041	A12383	433
A00041	A12408	199	A00041	A12453	156
A00041	A12680	156	A00041	A12732	156
A00041	A12824	416	A00041	A13039	156
A00041	A13040	156	A00041	A13058	154
A00041	A13364	180	A00041	A13373	236
A00041	A13390	899	A00041	A13391	899
A00041	A13393	899	A00041	A13401	899
A00041	A13511	480	A00041	A13531	191
A00041	A13593	252	A00041	A13734	190
A00041	A13826	402	A00041	A13827	183
A00041	A13828	156	A00041	A13840	156
A00041	A13860	489	A00041	A13873	486
A00041	A13899	347	A00041	A13975	307
A00041	A13980	943	A00041	A13981	899
A00041	A13982	934	A00041	A13983	572
A00041	A13984	899	A00041	A13986	449
A00041	A13989	899	A00041	A13997	257
A00041	A14007	229	A00041	A14027	899
A00041	A14031	909	A00041	A14035	899
A00041	A14131	195	A00041	A14140	156
A00041	A14143	232	A00041	A14146	283
A00041	A14187	414	A00041	A14188	486
A00041	A14190	475	A00041	A14191	433
A00041	A14198	491	A00041	A14199	279
A00041	A14201	196	A00041	A14208	955
A00041	A14210	480	A00041	A14236	234
A00041	A14364	491	A00041	A14366	414
A00041	A14371	439	A00041	A14374	379
A00041	A14395	484	A00041	A14399	306
A00041	A14402	923	A00041	A14426	408
A00041	A14430	647	A00041	A14433	180
A00041	A14440	479	A00041	A14452	480
A00041	A14472	899	A00041	A14511	408
A00041	A14513	156	A00041	A14564	156

A00041	A14596	483	A00041	A14599	441
A00041	A14642	386	A00041	A14643	466
A00041	A14647	373	A00041	A14662	899
A00041	A14741	329	A00041	A14767	156
A00041	A14899	154	A00041	A14930	899
A00041	A14943	463	A00041	A14964	361
A00041	A14992	156	A00041	A15075	156
A00041	A15076	156	A00041	A15150	156
A00041	A15232	156	A00041	A15291	230
A00041	A15293	899	A00041	A15294	899
A00041	A15297	899	A00041	A15302	152
A00041	A15308	384	A00041	A15314	396
A00041	A15323	922	A00041	A15324	899
A00041	A15325	899	A00041	A15328	179
A00041	A15331	174	A00041	A15346	451
A00041	A15349	435	A00041	A15353	899
A00041	A15359	899	A00041	A15369	199
A00041	A15394	473	A00041	A15395	537
A00041	A15403	154	A00041	A15420	177
A00041	A15425	424	A00041	A15487	221
A00041	A15498	422	A00041	A15510	385
A00041	A15521	154	A00041	A15529	252
A00041	A15576	154	A00041	A15597	299
A00041	A15624	205	A00041	A15638	286
A00041	A15647	460	A00041	A15660	489
A00041	A15726	491	A00041	A15801	182
A00041	A15806	288	A00041	A15896	247
A00041	A15935	480	A00041	A15963	156
A00041	A15964	330	A00041	A15966	319
A00041	A15967	156	A00041	A15974	419
A00041	A15979	412	A00041	A15991	156
A00041	A16010	156	A00041	A16074	291
A00041	A16216	334	A00041	A16224	162
A00041	A16228	296	A00041	A16230	230
A00041	A16269	400	A00041	A16274	490
A00041	A16300	386	A00041	A16310	195
A00041	A16562	156	A00041	A16623	159
A00041	A16636	484	A00041	A16845	156
A00041	A16901	475	A00041	A17052	156
A00041	A17063	406	A00041	A17160	899
A00041	A17198	399	A00042	A00043	63
A00042	A00044	945	A00042	A00045	491
A00042	A00046	943	A00042	A00052	994
A00042	A00056	945	A00042	A00057	262
A00042	A00058	899	A00042	A00059	937
A00042	A00060	340	A00042	A00061	491

A00042	A00062	485	A00042	A00063	490
A00042	A00064	302	A00042	A00065	899
A00042	A00067	972	A00042	A00068	951
A00042	A00069	371	A00042	A00070	899
A00042	A00073	899	A00042	A00074	942
A00042	A00082	917	A00042	A00086	353
A00042	A00094	921	A00042	A00098	216
A00042	A00109	489	A00042	A00111	154
A00042	A00122	270	A00042	A00123	962
A00042	A00124	899	A00042	A00125	296
A00042	A00138	491	A00042	A00141	491
A00042	A00145	454	A00042	A00147	160
A00042	A00149	899	A00042	A00150	201
A00042	A00151	992	A00042	A00152	999
A00042	A00153	491	A00042	A00157	899
A00042	A00158	486	A00042	A00159	958
A00042	A00160	490	A00042	A00161	63
A00042	A00162	944	A00042	A00163	944
A00042	A00167	945	A00042	A00169	271
A00042	A00171	899	A00042	A00174	462
A00042	A00185	903	A00042	A00186	443
A00042	A00189	604	A00042	A00190	945
A00042	A00193	165	A00042	A00200	915
A00042	A00201	899	A00042	A00204	911
A00042	A00205	262	A00042	A00207	586
A00042	A00208	63	A00042	A00209	993
A00042	A00214	899	A00042	A00219	171
A00042	A00220	457	A00042	A00224	910
A00042	A00226	154	A00042	A00230	928
A00042	A00231	207	A00042	A00232	195
A00042	A00233	491	A00042	A00238	929
A00042	A00239	443	A00042	A00240	481
A00042	A00246	994	A00042	A00248	424
A00042	A00250	899	A00042	A00254	942
A00042	A00255	456	A00042	A00256	425
A00042	A00261	721	A00042	A00264	945
A00042	A00270	899	A00042	A00274	302
A00042	A00275	899	A00042	A00324	905
A00042	A00381	899	A00042	A00400	191
A00042	A00561	944	A00042	A00572	485
A00042	A00576	154	A00042	A00643	335
A00042	A00694	747	A00042	A00736	405
A00042	A00799	945	A00042	A00887	152
A00042	A00914	945	A00042	A00963	205
A00042	A00981	162	A00042	A00985	255
A00042	A01088	217	A00042	A01138	240

A00042	A01140	460	A00042	A01161	462
A00042	A01198	899	A00042	A01211	196
A00042	A01289	480	A00042	A01473	369
A00042	A01489	899	A00042	A01507	938
A00042	A01512	899	A00042	A01522	899
A00042	A01524	997	A00042	A01527	997
A00042	A01551	414	A00042	A01553	918
A00042	A01554	916	A00042	A01558	989
A00042	A01559	263	A00042	A01560	904
A00042	A01588	989	A00042	A01604	434
A00042	A01606	199	A00042	A01615	990
A00042	A01631	906	A00042	A01632	390
A00042	A01634	899	A00042	A01640	899
A00042	A01647	199	A00042	A01651	899
A00042	A01654	491	A00042	A01668	227
A00042	A01683	899	A00042	A01692	180
A00042	A01702	899	A00042	A01704	483
A00042	A01708	899	A00042	A01709	944
A00042	A01718	903	A00042	A01726	486
A00042	A01727	929	A00042	A01729	225
A00042	A01740	210	A00042	A01741	943
A00042	A01743	339	A00042	A01746	208
A00042	A01747	180	A00042	A01759	149
A00042	A01765	899	A00042	A01775	944
A00042	A01787	279	A00042	A01789	156
A00042	A01790	358	A00042	A01797	911
A00042	A01802	154	A00042	A01844	909
A00042	A01870	368	A00042	A01873	168
A00042	A01876	491	A00042	A01878	167
A00042	A01879	490	A00042	A01880	490
A00042	A01881	449	A00042	A01887	475
A00042	A01888	491	A00042	A01889	385
A00042	A01895	90	A00042	A01902	445
A00042	A01905	904	A00042	A01908	162
A00042	A01914	228	A00042	A01931	227
A00042	A02056	452	A00042	A02176	273
A00042	A02179	456	A00042	A02278	382
A00042	A02298	454	A00042	A02319	168
A00042	A02337	252	A00042	A02340	329
A00042	A02372	221	A00042	A02382	679
A00042	A02384	216	A00042	A02416	291
A00042	A02447	379	A00042	A02481	409
A00042	A02490	481	A00042	A02491	452
A00042	A02594	180	A00042	A02608	196
A00042	A02615	430	A00042	A02619	899
A00042	A02631	247	A00042	A02637	475

A00042	A02639	486	A00042	A02696	245
A00042	A02697	481	A00042	A02698	491
A00042	A02709	288	A00042	A02711	491
A00042	A02714	274	A00042	A02730	440
A00042	A02732	348	A00042	A02739	489
A00042	A02741	63	A00042	A02749	428
A00042	A02769	190	A00042	A02787	243
A00042	A02788	405	A00042	A02812	469
A00042	A02832	319	A00042	A02837	152
A00042	A02859	899	A00042	A02863	945
A00042	A02870	260	A00042	A02873	201
A00042	A02896	432	A00042	A02908	238
A00042	A02917	486	A00042	A02942	899
A00042	A02945	477	A00042	A02962	246
A00042	A02964	218	A00042	A02982	345
A00042	A02983	312	A00042	A02985	123
A00042	A02988	277	A00042	A03010	306
A00042	A03033	213	A00042	A03051	260
A00042	A03095	346	A00042	A03096	218
A00042	A03183	154	A00042	A03188	213
A00042	A03204	472	A00042	A03211	452
A00042	A03259	491	A00042	A03275	252
A00042	A03293	899	A00042	A03304	429
A00042	A03331	899	A00042	A03332	455
A00042	A03420	899	A00042	A03436	274
A00042	A03451	475	A00042	A03585	205
A00042	A03610	462	A00042	A03611	491
A00042	A03632	198	A00042	A03650	168
A00042	A03661	251	A00042	A03783	196
A00042	A03790	374	A00042	A03809	159
A00042	A03822	440	A00042	A03934	266
A00042	A03966	235	A00042	A04004	328
A00042	A04010	255	A00042	A04016	159
A00042	A04033	491	A00042	A04041	196
A00042	A04117	481	A00042	A04118	218
A00042	A04138	163	A00042	A04206	196
A00042	A04248	489	A00042	A04280	399
A00042	A04284	180	A00042	A04287	254
A00042	A04302	212	A00042	A04315	196
A00042	A04320	446	A00042	A04324	180
A00042	A04398	337	A00042	A04419	180
A00042	A04478	154	A00042	A04484	252
A00042	A04529	361	A00042	A04535	205
A00042	A04536	235	A00042	A04548	288
A00042	A04569	168	A00042	A04585	254
A00042	A04652	281	A00042	A04699	903

A00042	A04717	168	A00042	A04718	420
A00042	A04754	247	A00042	A04791	219
A00042	A04800	218	A00042	A04877	252
A00042	A04878	434	A00042	A04879	472
A00042	A04909	165	A00042	A04953	218
A00042	A05103	247	A00042	A05174	171
A00042	A05219	899	A00042	A05272	254
A00042	A05309	416	A00042	A05315	899
A00042	A05317	168	A00042	A05463	152
A00042	A05490	899	A00042	A05943	180
A00042	A05944	439	A00042	A06054	278
A00042	A06142	218	A00042	A06156	180
A00042	A06175	180	A00042	A06212	235
A00042	A06289	899	A00042	A06324	308
A00042	A06414	171	A00042	A06415	355
A00042	A06422	229	A00042	A06448	168
A00042	A06506	168	A00042	A06601	899
A00042	A06608	902	A00042	A06610	243
A00042	A06618	218	A00042	A06635	232
A00042	A06650	483	A00042	A06686	218
A00042	A07006	159	A00042	A07061	899
A00042	A07091	899	A00042	A07110	196
A00042	A07135	441	A00042	A07145	281
A00042	A07152	378	A00042	A07154	152
A00042	A07157	757	A00042	A07158	346
A00042	A07160	473	A00042	A07161	470
A00042	A07173	480	A00042	A07200	303
A00042	A07269	347	A00042	A07310	196
A00042	A07339	932	A00042	A07422	939
A00042	A07565	159	A00042	A07622	313
A00042	A07668	218	A00042	A07747	196
A00042	A07902	291	A00042	A08030	420
A00042	A08117	413	A00042	A08216	302
A00042	A08289	328	A00042	A08360	179
A00042	A08378	168	A00042	A08445	188
A00042	A08588	430	A00042	A08607	218
A00042	A08615	168	A00042	A08675	254
A00042	A08697	254	A00042	A08730	168
A00042	A08835	361	A00042	A08869	899
A00042	A08880	168	A00042	A08941	281
A00042	A09014	180	A00042	A09112	281
A00042	A09263	419	A00042	A09319	899
A00042	A09405	254	A00042	A09438	152
A00042	A09499	159	A00042	A09523	218
A00042	A09558	899	A00042	A09568	229
A00042	A09575	302	A00042	A09577	899

A00042	A09644	899	A00042	A09732	306
A00042	A09804	899	A00042	A09884	143
A00042	A09898	196	A00042	A09959	180
A00042	A10049	208	A00042	A10174	899
A00042	A10233	246	A00042	A10307	159
A00042	A10365	899	A00042	A10459	177
A00042	A10496	254	A00042	A10580	899
A00042	A10584	899	A00042	A10615	899
A00042	A10633	491	A00042	A10754	899
A00042	A10791	484	A00042	A10808	899
A00042	A10811	899	A00042	A10844	268
A00042	A10899	193	A00042	A11090	260
A00042	A11124	899	A00042	A11125	942
A00042	A11306	260	A00042	A11348	235
A00042	A11400	339	A00042	A11411	185
A00042	A11413	236	A00042	A11431	353
A00042	A11474	260	A00042	A11543	285
A00042	A11983	923	A00042	A12296	196
A00042	A12383	327	A00042	A12393	303
A00042	A12897	357	A00042	A12913	196
A00042	A12927	223	A00042	A13002	232
A00042	A13182	168	A00042	A13464	254
A00042	A13828	254	A00042	A13860	301
A00042	A13946	330	A00042	A13976	168
A00042	A13978	154	A00042	A13983	229
A00042	A13984	463	A00042	A14002	899
A00042	A14008	899	A00042	A14094	899
A00042	A14191	185	A00042	A14193	245
A00042	A14204	518	A00042	A14208	899
A00042	A14216	193	A00042	A14220	899
A00042	A14232	899	A00042	A14236	491
A00042	A14273	378	A00042	A14364	901
A00042	A14366	297	A00042	A14373	216
A00042	A14391	428	A00042	A14392	212
A00042	A14393	938	A00042	A14401	414
A00042	A14426	382	A00042	A14432	299
A00042	A14442	238	A00042	A14444	989
A00042	A14498	187	A00042	A14527	899
A00042	A14635	361	A00042	A14643	302
A00042	A14724	254	A00042	A14784	254
A00042	A15029	159	A00042	A15136	180
A00042	A15241	491	A00042	A15290	196
A00042	A15323	504	A00042	A15394	230
A00042	A15395	457	A00042	A15416	440
A00042	A15601	256	A00042	A15603	232
A00042	A15605	335	A00042	A15606	486

A00042	A15607	177	A00042	A15902	205
A00042	A15910	168	A00042	A15981	353
A00042	A15994	218	A00042	A16044	180
A00042	A16397	152	A00042	A16636	485
A00042	A16791	899	A00042	A17050	218
A00043	A00045	389	A00043	A00050	152
A00043	A00052	490	A00043	A00057	162
A00043	A00058	63	A00043	A00064	190
A00043	A00074	262	A00043	A00149	435
A00043	A00154	491	A00043	A00157	74
A00043	A00159	945	A00043	A00161	63
A00043	A00163	452	A00043	A00169	396
A00043	A00171	430	A00043	A00173	425
A00043	A00175	193	A00043	A00190	486
A00043	A00193	457	A00043	A00208	63
A00043	A00214	899	A00043	A00219	210
A00043	A00232	490	A00043	A00233	208
A00043	A00234	241	A00043	A00271	262
A00043	A00298	165	A00043	A00305	205
A00043	A00322	163	A00043	A00377	191
A00043	A00379	276	A00043	A00381	457
A00043	A00384	162	A00043	A00395	467
A00043	A00433	260	A00043	A00449	302
A00043	A00469	485	A00043	A00556	377
A00043	A00597	424	A00043	A00616	208
A00043	A00747	221	A00043	A00800	235
A00043	A00887	473	A00043	A00914	196
A00043	A01018	488	A00043	A01021	199
A00043	A01038	238	A00043	A01051	274
A00043	A01113	416	A00043	A01115	288
A00043	A01156	234	A00043	A01173	479
A00043	A01192	490	A00043	A01222	154
A00043	A01225	311	A00043	A01276	414
A00043	A01289	357	A00043	A01483	345
A00043	A01527	899	A00043	A01553	63
A00043	A01557	302	A00043	A01561	899
A00043	A01572	255	A00043	A01576	899
A00043	A01608	208	A00043	A01676	489
A00043	A01690	474	A00043	A01730	430
A00043	A01740	180	A00043	A01741	486
A00043	A01755	82	A00043	A01768	368
A00043	A01878	285	A00043	A01890	255
A00043	A01897	491	A00043	A01898	76
A00043	A01899	303	A00043	A01900	259
A00043	A01901	491	A00043	A01902	291
A00043	A01913	281	A00043	A01983	200

A00043	A02026	307	A00043	A02027	183
A00043	A02052	193	A00043	A02184	229
A00043	A02213	168	A00043	A02214	271
A00043	A02303	333	A00043	A02344	283
A00043	A02484	154	A00043	A02495	367
A00043	A02592	414	A00043	A02598	407
A00043	A02618	154	A00043	A02629	255
A00043	A02642	172	A00043	A02658	235
A00043	A02703	63	A00043	A02705	454
A00043	A02706	333	A00043	A02709	157
A00043	A02733	346	A00043	A02743	246
A00043	A02796	168	A00043	A02816	302
A00043	A02817	218	A00043	A02827	218
A00043	A02861	378	A00043	A02862	396
A00043	A02864	899	A00043	A02869	272
A00043	A02874	636	A00043	A02877	224
A00043	A02884	191	A00043	A02901	177
A00043	A02968	899	A00043	A03144	160
A00043	A03222	218	A00043	A03259	257
A00043	A03321	63	A00043	A03392	241
A00043	A03412	160	A00043	A03421	215
A00043	A03511	205	A00043	A03585	180
A00043	A03646	258	A00043	A03795	475
A00043	A03883	328	A00043	A03922	434
A00043	A03928	283	A00043	A04017	223
A00043	A04046	213	A00043	A04121	249
A00043	A04179	191	A00043	A04228	322
A00043	A04305	486	A00043	A04307	180
A00043	A04316	405	A00043	A04382	258
A00043	A04383	180	A00043	A04423	163
A00043	A04446	486	A00043	A04476	328
A00043	A04502	393	A00043	A04542	241
A00043	A04576	360	A00043	A04579	159
A00043	A04595	177	A00043	A04618	167
A00043	A04622	288	A00043	A04627	266
A00043	A04661	467	A00043	A04706	301
A00043	A04739	286	A00043	A04743	205
A00043	A04816	258	A00043	A04838	177
A00043	A05045	218	A00043	A05071	258
A00043	A05163	241	A00043	A05388	347
A00043	A05545	193	A00043	A05670	313
A00043	A05677	213	A00043	A06109	360
A00043	A06158	283	A00043	A06439	340
A00043	A06649	176	A00043	A06663	208
A00043	A06701	254	A00043	A06821	416
A00043	A06846	477	A00043	A07014	162

A00043	A07049	328	A00043	A07143	375
A00043	A07153	488	A00043	A07163	446
A00043	A07165	152	A00043	A07201	489
A00043	A07232	445	A00043	A07239	313
A00043	A07281	356	A00043	A07335	301
A00043	A07739	235	A00043	A08064	191
A00043	A08261	265	A00043	A08390	452
A00043	A08999	283	A00043	A09104	165
A00043	A09263	384	A00043	A09323	162
A00043	A09438	141	A00043	A09456	328
A00043	A09471	193	A00043	A09591	490
A00043	A09784	165	A00043	A09911	177
A00043	A09917	229	A00043	A09942	328
A00043	A09999	165	A00043	A10186	269
A00043	A10316	168	A00043	A10458	198
A00043	A10696	201	A00043	A11021	356
A00043	A11064	193	A00043	A11113	411
A00043	A11135	241	A00043	A11206	174
A00043	A11211	223	A00043	A11344	256
A00043	A11548	201	A00043	A11759	204
A00043	A12060	283	A00043	A12080	223
A00043	A12329	479	A00043	A12710	269
A00043	A13044	235	A00043	A13232	269
A00043	A13592	193	A00043	A13623	269
A00043	A13738	333	A00043	A13986	360
A00043	A13997	200	A00043	A14014	188
A00043	A14094	425	A00043	A14151	152
A00043	A14273	188	A00043	A14443	899
A00043	A14513	328	A00043	A14643	491
A00043	A14648	489	A00043	A14686	198
A00043	A15519	398	A00043	A15629	160
A00043	A15653	485	A00043	A15660	278
A00043	A15684	219	A00043	A15970	154
A00043	A16163	283	A00043	A16258	198
A00043	A16325	218	A00043	A16676	316
A00044	A00045	461	A00044	A00046	313
A00044	A00056	419	A00044	A00061	276
A00044	A00062	491	A00044	A00063	423
A00044	A00064	414	A00044	A00067	944
A00044	A00074	491	A00044	A00079	221
A00044	A00086	185	A00044	A00109	305
A00044	A00111	157	A00044	A00122	63
A00044	A00138	491	A00044	A00141	945
A00044	A00145	424	A00044	A00152	945
A00044	A00156	63	A00044	A00158	246
A00044	A00159	488	A00044	A00160	260

A00044	A00161	63	A00044	A00162	63
A00044	A00173	483	A00044	A00183	301
A00044	A00185	171	A00044	A00188	173
A00044	A00189	450	A00044	A00190	236
A00044	A00205	195	A00044	A00207	296
A00044	A00220	193	A00044	A00224	162
A00044	A00232	276	A00044	A00233	330
A00044	A00234	363	A00044	A00246	943
A00044	A00248	269	A00044	A00254	463
A00044	A00261	405	A00044	A00391	201
A00044	A00616	173	A00044	A00914	210
A00044	A00981	260	A00044	A01452	191
A00044	A01473	430	A00044	A01524	924
A00044	A01527	943	A00044	A01553	82
A00044	A01554	63	A00044	A01560	63
A00044	A01572	405	A00044	A01604	425
A00044	A01654	450	A00044	A01704	236
A00044	A01709	193	A00044	A01870	396
A00044	A01876	160	A00044	A01879	473
A00044	A01880	229	A00044	A01887	454
A00044	A01888	485	A00044	A01908	63
A00044	A02141	201	A00044	A02337	167
A00044	A02340	243	A00044	A02382	441
A00044	A02383	165	A00044	A02416	202
A00044	A02438	165	A00044	A02578	191
A00044	A02582	224	A00044	A02639	249
A00044	A02658	259	A00044	A02659	290
A00044	A02711	263	A00044	A02713	182
A00044	A02730	246	A00044	A02731	185
A00044	A02769	328	A00044	A02795	176
A00044	A02816	157	A00044	A02851	191
A00044	A02864	491	A00044	A02884	218
A00044	A02945	378	A00044	A02962	163
A00044	A03010	486	A00044	A03204	449
A00044	A03259	463	A00044	A03271	63
A00044	A03316	218	A00044	A03470	173
A00044	A03610	363	A00044	A03817	297
A00044	A03821	163	A00044	A04138	176
A00044	A04207	218	A00044	A04244	312
A00044	A04248	401	A00044	A04263	218
A00044	A04280	199	A00044	A04320	488
A00044	A04321	281	A00044	A04374	303
A00044	A04473	193	A00044	A04478	386
A00044	A04518	210	A00044	A04536	324
A00044	A04682	151	A00044	A04736	247
A00044	A04865	377	A00044	A04866	235

A00044	A05127	383	A00044	A05741	205
A00044	A05816	227	A00044	A05931	281
A00044	A05944	210	A00044	A05985	407
A00044	A06095	207	A00044	A06107	243
A00044	A06415	246	A00044	A06422	285
A00044	A06465	303	A00044	A06780	235
A00044	A06818	191	A00044	A07031	379
A00044	A07138	188	A00044	A07148	438
A00044	A07152	165	A00044	A07157	489
A00044	A07158	63	A00044	A07173	191
A00044	A07421	418	A00044	A08360	457
A00044	A09123	171	A00044	A09220	184
A00044	A10083	361	A00044	A10650	291
A00044	A10744	221	A00044	A10791	484
A00044	A10827	319	A00044	A11064	221
A00044	A11123	215	A00044	A11369	296
A00044	A11543	210	A00044	A12886	187
A00044	A13181	208	A00044	A13860	296
A00044	A13986	456	A00044	A14007	458
A00044	A14198	447	A00044	A14299	379
A00044	A14432	200	A00044	A14452	195
A00044	A14981	345	A00044	A15394	327
A00044	A15395	195	A00044	A15420	440
A00044	A15603	286	A00044	A15605	346
A00044	A15608	358	A00044	A15640	281
A00044	A15726	441	A00044	A15981	182
A00044	A16636	171	A00045	A00052	489
A00045	A00056	490	A00045	A00057	412
A00045	A00061	491	A00045	A00062	411
A00045	A00063	491	A00045	A00064	490
A00045	A00067	945	A00045	A00068	243
A00045	A00074	369	A00045	A00084	177
A00045	A00086	446	A00045	A00087	358
A00045	A00089	480	A00045	A00092	452
A00045	A00094	285	A00045	A00109	313
A00045	A00111	424	A00045	A00123	491
A00045	A00125	480	A00045	A00138	182
A00045	A00141	481	A00045	A00145	335
A00045	A00147	430	A00045	A00149	484
A00045	A00151	168	A00045	A00152	491
A00045	A00153	687	A00045	A00154	435
A00045	A00155	419	A00045	A00159	490
A00045	A00160	229	A00045	A00162	63
A00045	A00163	944	A00045	A00167	486
A00045	A00169	173	A00045	A00171	385
A00045	A00188	297	A00045	A00189	943

A00045	A00190	491	A00045	A00196	467
A00045	A00204	343	A00045	A00209	925
A00045	A00212	491	A00045	A00214	443
A00045	A00215	435	A00045	A00216	246
A00045	A00218	321	A00045	A00219	491
A00045	A00224	945	A00045	A00230	899
A00045	A00232	490	A00045	A00233	491
A00045	A00234	385	A00045	A00238	254
A00045	A00246	184	A00045	A00248	465
A00045	A00254	291	A00045	A00264	491
A00045	A00274	457	A00045	A00292	290
A00045	A00294	418	A00045	A00305	208
A00045	A00309	452	A00045	A00322	184
A00045	A00324	486	A00045	A00338	486
A00045	A00376	232	A00045	A00395	213
A00045	A00437	432	A00045	A00449	187
A00045	A00470	407	A00045	A00472	490
A00045	A00485	479	A00045	A00541	396
A00045	A00556	294	A00045	A00557	249
A00045	A00574	475	A00045	A00576	474
A00045	A00577	234	A00045	A00600	162
A00045	A00614	302	A00045	A00616	438
A00045	A00628	434	A00045	A00641	218
A00045	A00654	409	A00045	A00659	241
A00045	A00663	307	A00045	A00681	430
A00045	A00688	480	A00045	A00695	235
A00045	A00736	491	A00045	A00738	254
A00045	A00770	301	A00045	A00773	353
A00045	A00799	469	A00045	A00820	491
A00045	A00826	188	A00045	A00914	945
A00045	A00939	405	A00045	A00962	429
A00045	A00995	243	A00045	A01022	236
A00045	A01161	490	A00045	A01172	466
A00045	A01174	199	A00045	A01178	157
A00045	A01230	183	A00045	A01256	480
A00045	A01257	296	A00045	A01307	398
A00045	A01317	334	A00045	A01318	411
A00045	A01320	377	A00045	A01324	260
A00045	A01328	360	A00045	A01396	358
A00045	A01432	332	A00045	A01481	156
A00045	A01485	240	A00045	A01524	929
A00045	A01527	374	A00045	A01551	491
A00045	A01556	408	A00045	A01557	318
A00045	A01558	899	A00045	A01567	260
A00045	A01572	316	A00045	A01578	491
A00045	A01582	373	A00045	A01590	204

A00045	A01602	208	A00045	A01604	259
A00045	A01615	899	A00045	A01632	489
A00045	A01635	402	A00045	A01654	486
A00045	A01661	899	A00045	A01668	491
A00045	A01676	491	A00045	A01694	341
A00045	A01704	455	A00045	A01705	474
A00045	A01713	188	A00045	A01726	279
A00045	A01728	160	A00045	A01729	462
A00045	A01730	246	A00045	A01733	491
A00045	A01741	491	A00045	A01743	491
A00045	A01746	486	A00045	A01748	357
A00045	A01752	157	A00045	A01754	63
A00045	A01755	283	A00045	A01757	171
A00045	A01758	236	A00045	A01759	351
A00045	A01764	286	A00045	A01766	165
A00045	A01767	467	A00045	A01769	484
A00045	A01774	63	A00045	A01775	491
A00045	A01784	152	A00045	A01785	393
A00045	A01786	935	A00045	A01789	190
A00045	A01791	457	A00045	A01792	65
A00045	A01796	486	A00045	A01797	469
A00045	A01798	485	A00045	A01844	473
A00045	A01872	165	A00045	A01875	461
A00045	A01876	491	A00045	A01879	480
A00045	A01880	491	A00045	A01881	425
A00045	A01883	290	A00045	A01887	486
A00045	A01888	747	A00045	A01889	400
A00045	A01890	255	A00045	A01894	414
A00045	A01896	156	A00045	A01900	388
A00045	A01902	491	A00045	A01905	224
A00045	A01908	341	A00045	A01909	269
A00045	A01923	177	A00045	A01926	395
A00045	A01931	323	A00045	A01941	475
A00045	A01961	171	A00045	A01972	290
A00045	A02011	215	A00045	A02049	188
A00045	A02071	191	A00045	A02086	204
A00045	A02098	157	A00045	A02117	380
A00045	A02122	322	A00045	A02156	241
A00045	A02199	230	A00045	A02251	458
A00045	A02252	183	A00045	A02278	490
A00045	A02298	435	A00045	A02300	247
A00045	A02316	185	A00045	A02331	154
A00045	A02333	384	A00045	A02337	105
A00045	A02340	490	A00045	A02347	235
A00045	A02351	236	A00045	A02372	243
A00045	A02380	260	A00045	A02382	452

A00045	A02384	165	A00045	A02415	299
A00045	A02416	490	A00045	A02466	245
A00045	A02489	162	A00045	A02490	489
A00045	A02491	491	A00045	A02495	168
A00045	A02513	218	A00045	A02564	341
A00045	A02571	293	A00045	A02582	294
A00045	A02586	165	A00045	A02591	157
A00045	A02605	165	A00045	A02615	293
A00045	A02620	173	A00045	A02639	451
A00045	A02668	454	A00045	A02685	171
A00045	A02686	313	A00045	A02687	172
A00045	A02691	386	A00045	A02695	477
A00045	A02696	223	A00045	A02697	443
A00045	A02698	490	A00045	A02705	405
A00045	A02706	227	A00045	A02709	352
A00045	A02711	266	A00045	A02729	419
A00045	A02730	491	A00045	A02732	210
A00045	A02735	218	A00045	A02738	378
A00045	A02741	490	A00045	A02745	382
A00045	A02746	310	A00045	A02749	480
A00045	A02753	213	A00045	A02754	407
A00045	A02756	405	A00045	A02770	263
A00045	A02780	235	A00045	A02783	241
A00045	A02785	481	A00045	A02787	425
A00045	A02788	490	A00045	A02808	182
A00045	A02812	491	A00045	A02828	440
A00045	A02830	271	A00045	A02832	172
A00045	A02834	190	A00045	A02836	294
A00045	A02837	491	A00045	A02840	337
A00045	A02841	483	A00045	A02854	477
A00045	A02856	168	A00045	A02859	491
A00045	A02862	218	A00045	A02863	484
A00045	A02869	363	A00045	A02874	491
A00045	A02875	364	A00045	A02877	151
A00045	A02883	193	A00045	A02886	357
A00045	A02896	399	A00045	A02908	404
A00045	A02917	303	A00045	A02918	238
A00045	A02924	294	A00045	A02929	322
A00045	A02953	363	A00045	A02969	152
A00045	A02982	462	A00045	A02983	357
A00045	A02984	408	A00045	A02985	91
A00045	A02986	218	A00045	A02992	160
A00045	A03008	180	A00045	A03010	945
A00045	A03019	193	A00045	A03034	188
A00045	A03050	163	A00045	A03056	318
A00045	A03080	460	A00045	A03095	372

A00045	A03100	484	A00045	A03107	168
A00045	A03112	490	A00045	A03126	486
A00045	A03131	340	A00045	A03144	241
A00045	A03167	449	A00045	A03168	337
A00045	A03184	232	A00045	A03204	245
A00045	A03211	485	A00045	A03223	462
A00045	A03243	193	A00045	A03259	491
A00045	A03260	339	A00045	A03275	489
A00045	A03293	168	A00045	A03304	441
A00045	A03356	251	A00045	A03436	152
A00045	A03451	373	A00045	A03477	283
A00045	A03520	443	A00045	A03534	274
A00045	A03539	157	A00045	A03583	182
A00045	A03590	187	A00045	A03602	218
A00045	A03609	404	A00045	A03610	401
A00045	A03611	316	A00045	A03620	143
A00045	A03661	191	A00045	A03739	445
A00045	A03803	307	A00045	A03807	374
A00045	A03821	379	A00045	A03851	255
A00045	A03923	185	A00045	A03934	218
A00045	A03964	227	A00045	A03984	159
A00045	A03991	228	A00045	A04010	481
A00045	A04033	443	A00045	A04041	157
A00045	A04080	207	A00045	A04091	369
A00045	A04117	488	A00045	A04138	481
A00045	A04144	212	A00045	A04169	252
A00045	A04206	329	A00045	A04211	182
A00045	A04238	187	A00045	A04248	402
A00045	A04263	489	A00045	A04274	212
A00045	A04275	412	A00045	A04276	357
A00045	A04280	434	A00045	A04288	243
A00045	A04302	345	A00045	A04303	180
A00045	A04309	256	A00045	A04320	274
A00045	A04334	232	A00045	A04337	396
A00045	A04342	439	A00045	A04361	265
A00045	A04380	199	A00045	A04398	439
A00045	A04410	353	A00045	A04484	205
A00045	A04520	182	A00045	A04535	154
A00045	A04536	463	A00045	A04559	218
A00045	A04567	405	A00045	A04589	165
A00045	A04603	154	A00045	A04622	489
A00045	A04685	152	A00045	A04714	260
A00045	A04715	210	A00045	A04734	401
A00045	A04783	157	A00045	A04798	218
A00045	A04800	272	A00045	A04805	204
A00045	A04806	274	A00045	A04839	152

A00045	A04851	182	A00045	A04855	259
A00045	A04865	330	A00045	A04878	302
A00045	A04891	313	A00045	A04902	486
A00045	A04908	419	A00045	A04927	167
A00045	A04939	171	A00045	A04949	296
A00045	A05007	296	A00045	A05032	172
A00045	A05094	205	A00045	A05098	223
A00045	A05102	272	A00045	A05106	281
A00045	A05155	364	A00045	A05214	180
A00045	A05254	218	A00045	A05279	157
A00045	A05297	475	A00045	A05305	180
A00045	A05309	491	A00045	A05356	404
A00045	A05406	180	A00045	A05416	362
A00045	A05444	218	A00045	A05451	187
A00045	A05462	185	A00045	A05472	163
A00045	A05473	288	A00045	A05515	218
A00045	A05516	157	A00045	A05551	157
A00045	A05582	274	A00045	A05601	177
A00045	A05603	441	A00045	A05643	286
A00045	A05659	329	A00045	A05679	204
A00045	A05742	428	A00045	A05744	180
A00045	A05759	255	A00045	A05838	434
A00045	A05891	360	A00045	A05929	218
A00045	A05933	218	A00045	A05958	281
A00045	A05969	157	A00045	A05985	488
A00045	A06065	157	A00045	A06067	177
A00045	A06072	218	A00045	A06088	168
A00045	A06339	286	A00045	A06346	185
A00045	A06358	291	A00045	A06377	157
A00045	A06402	162	A00045	A06415	313
A00045	A06422	413	A00045	A06445	218
A00045	A06525	180	A00045	A06581	335
A00045	A06592	269	A00045	A06597	165
A00045	A06605	157	A00045	A06607	218
A00045	A06610	491	A00045	A06615	372
A00045	A06623	249	A00045	A06626	212
A00045	A06650	439	A00045	A06686	156
A00045	A06716	218	A00045	A06731	218
A00045	A06834	198	A00045	A06835	180
A00045	A06866	180	A00045	A06931	157
A00045	A06949	305	A00045	A06984	218
A00045	A07031	899	A00045	A07061	198
A00045	A07109	319	A00045	A07122	393
A00045	A07127	277	A00045	A07135	443
A00045	A07146	408	A00045	A07153	416
A00045	A07154	477	A00045	A07157	454

A00045	A07158	477	A00045	A07160	208
A00045	A07163	205	A00045	A07164	435
A00045	A07165	491	A00045	A07173	383
A00045	A07191	358	A00045	A07193	180
A00045	A07197	382	A00045	A07198	424
A00045	A07200	486	A00045	A07201	413
A00045	A07209	306	A00045	A07211	218
A00045	A07218	306	A00045	A07222	187
A00045	A07413	321	A00045	A07440	252
A00045	A07442	368	A00045	A07477	345
A00045	A07494	180	A00045	A07501	457
A00045	A07590	277	A00045	A07605	160
A00045	A07736	391	A00045	A07790	218
A00045	A07800	432	A00045	A07803	215
A00045	A07810	180	A00045	A07931	347
A00045	A07973	235	A00045	A08099	279
A00045	A08238	232	A00045	A08296	263
A00045	A08595	180	A00045	A08623	180
A00045	A08649	180	A00045	A08670	180
A00045	A08751	177	A00045	A08764	180
A00045	A08857	323	A00045	A08867	460
A00045	A09018	256	A00045	A09046	229
A00045	A09070	157	A00045	A09073	196
A00045	A09123	458	A00045	A09124	202
A00045	A09170	180	A00045	A09199	324
A00045	A09261	218	A00045	A09263	63
A00045	A09357	191	A00045	A09403	157
A00045	A09420	157	A00045	A09732	480
A00045	A09733	204	A00045	A09826	216
A00045	A09833	272	A00045	A09882	154
A00045	A09951	180	A00045	A10027	218
A00045	A10033	157	A00045	A10060	157
A00045	A10066	291	A00045	A10096	157
A00045	A10189	180	A00045	A10215	193
A00045	A10219	313	A00045	A10230	218
A00045	A10326	218	A00045	A10335	180
A00045	A10337	152	A00045	A10364	314
A00045	A10397	157	A00045	A10435	218
A00045	A10456	221	A00045	A10611	396
A00045	A10637	180	A00045	A10656	218
A00045	A10658	241	A00045	A10672	154
A00045	A10677	246	A00045	A10702	350
A00045	A10712	157	A00045	A10735	455
A00045	A10986	165	A00045	A11005	180
A00045	A11051	218	A00045	A11136	218
A00045	A11309	366	A00045	A11331	182

A00045	A11412	461	A00045	A11591	216
A00045	A11683	263	A00045	A12038	218
A00045	A12144	182	A00045	A12324	157
A00045	A12383	228	A00045	A12418	218
A00045	A12428	218	A00045	A12586	218
A00045	A12648	157	A00045	A12656	157
A00045	A12698	180	A00045	A12832	218
A00045	A12844	218	A00045	A12897	382
A00045	A12898	157	A00045	A12913	157
A00045	A12927	465	A00045	A12967	218
A00045	A13084	204	A00045	A13181	201
A00045	A13262	218	A00045	A13355	218
A00045	A13369	452	A00045	A13435	228
A00045	A13464	218	A00045	A13471	218
A00045	A13593	276	A00045	A13627	180
A00045	A13667	477	A00045	A13735	283
A00045	A13824	238	A00045	A13826	475
A00045	A13829	435	A00045	A13871	324
A00045	A13935	900	A00045	A13996	274
A00045	A14202	210	A00045	A14210	332
A00045	A14229	243	A00045	A14236	483
A00045	A14245	314	A00045	A14303	213
A00045	A14313	236	A00045	A14361	218
A00045	A14371	312	A00045	A14392	299
A00045	A14394	268	A00045	A14405	205
A00045	A14541	180	A00045	A14632	157
A00045	A14643	369	A00045	A14664	180
A00045	A14741	417	A00045	A14784	218
A00045	A14857	277	A00045	A14930	294
A00045	A14934	180	A00045	A14986	160
A00045	A15241	491	A00045	A15277	179
A00045	A15292	380	A00045	A15314	414
A00045	A15322	484	A00045	A15346	229
A00045	A15349	276	A00045	A15352	263
A00045	A15357	396	A00045	A15398	303
A00045	A15402	447	A00045	A15480	213
A00045	A15497	212	A00045	A15804	165
A00045	A15881	157	A00045	A15917	271
A00045	A15952	180	A00045	A16002	210
A00045	A16034	267	A00045	A16052	157
A00045	A16113	180	A00045	A16121	255
A00045	A16216	396	A00045	A16286	256
A00045	A16300	339	A00045	A16346	157
A00045	A16432	184	A00045	A16529	322
A00045	A16636	491	A00045	A16965	314
A00045	A17078	335	A00045	A17198	452

A00046	A00052	915	A00046	A00064	915
A00046	A00066	491	A00046	A00067	933
A00046	A00074	930	A00046	A00126	332
A00046	A00135	190	A00046	A00138	460
A00046	A00141	340	A00046	A00157	899
A00046	A00162	899	A00046	A00167	919
A00046	A00169	332	A00046	A00207	913
A00046	A00208	215	A00046	A00209	899
A00046	A00210	221	A00046	A00224	945
A00046	A00230	943	A00046	A00234	917
A00046	A00246	899	A00046	A00254	358
A00046	A00257	912	A00046	A00341	255
A00046	A00381	198	A00046	A00669	173
A00046	A01052	902	A00046	A01452	224
A00046	A01524	924	A00046	A01527	907
A00046	A01548	63	A00046	A01553	899
A00046	A01558	944	A00046	A01560	474
A00046	A01562	899	A00046	A01588	935
A00046	A01602	899	A00046	A01615	899
A00046	A01638	899	A00046	A01651	899
A00046	A01655	154	A00046	A01844	899
A00046	A02747	236	A00046	A03790	168
A00046	A04195	274	A00046	A04282	168
A00046	A04807	227	A00046	A04809	160
A00046	A05150	174	A00046	A05621	286
A00046	A05822	243	A00046	A05958	363
A00046	A06148	221	A00046	A06610	274
A00046	A07155	356	A00046	A07311	899
A00046	A08571	316	A00046	A09121	347
A00046	A09937	177	A00046	A10542	274
A00046	A10580	899	A00046	A10615	357
A00046	A10801	899	A00046	A13984	384
A00046	A14006	198	A00046	A14198	257
A00046	A14443	347	A00046	A14548	190
A00046	A15120	355	A00046	A15325	196
A00046	A15422	256	A00046	A15726	162
A00046	A16560	226	A00047	A00055	490
A00047	A00058	520	A00047	A00064	927
A00047	A00073	981	A00047	A00127	483
A00047	A00146	63	A00047	A00150	967
A00047	A00169	170	A00047	A00191	899
A00047	A00199	372	A00047	A00203	452
A00047	A00214	899	A00047	A00215	475
A00047	A00225	527	A00047	A00234	904
A00047	A00245	435	A00047	A00258	485
A00047	A00261	201	A00047	A00265	457

A00047	A00280	452	A00047	A00285	480
A00047	A00286	490	A00047	A00288	486
A00047	A00289	188	A00047	A00296	488
A00047	A00303	364	A00047	A00308	491
A00047	A00316	238	A00047	A00323	271
A00047	A00325	472	A00047	A00329	452
A00047	A00330	167	A00047	A00339	152
A00047	A00348	491	A00047	A00355	371
A00047	A00356	488	A00047	A00358	458
A00047	A00366	463	A00047	A00367	260
A00047	A00368	334	A00047	A00369	486
A00047	A00371	491	A00047	A00372	467
A00047	A00382	659	A00047	A00389	229
A00047	A00397	552	A00047	A00403	378
A00047	A00412	468	A00047	A00421	491
A00047	A00430	274	A00047	A00433	356
A00047	A00440	604	A00047	A00444	659
A00047	A00448	163	A00047	A00452	223
A00047	A00453	411	A00047	A00454	486
A00047	A00457	195	A00047	A00458	491
A00047	A00469	265	A00047	A00489	659
A00047	A00491	180	A00047	A00494	165
A00047	A00496	477	A00047	A00500	489
A00047	A00501	466	A00047	A00508	168
A00047	A00517	455	A00047	A00520	199
A00047	A00527	439	A00047	A00531	538
A00047	A00532	532	A00047	A00535	201
A00047	A00553	486	A00047	A00560	472
A00047	A00570	477	A00047	A00571	434
A00047	A00574	469	A00047	A00589	338
A00047	A00605	491	A00047	A00609	455
A00047	A00629	452	A00047	A00640	491
A00047	A00648	438	A00047	A00651	520
A00047	A00652	251	A00047	A00655	463
A00047	A00658	402	A00047	A00666	457
A00047	A00667	488	A00047	A00670	224
A00047	A00687	484	A00047	A00691	402
A00047	A00693	299	A00047	A00700	486
A00047	A00703	447	A00047	A00705	462
A00047	A00706	491	A00047	A00740	154
A00047	A00746	196	A00047	A00764	199
A00047	A00767	312	A00047	A00810	475
A00047	A00822	491	A00047	A00826	399
A00047	A00831	491	A00047	A00835	486
A00047	A00837	184	A00047	A00839	530
A00047	A00841	486	A00047	A00843	609

A00047	A00848	491	A00047	A00849	374
A00047	A00853	177	A00047	A00856	341
A00047	A00871	217	A00047	A00875	157
A00047	A00883	491	A00047	A00905	491
A00047	A00909	361	A00047	A00916	540
A00047	A00918	479	A00047	A00922	283
A00047	A00923	491	A00047	A00928	451
A00047	A00932	469	A00047	A00939	914
A00047	A00954	472	A00047	A00958	477
A00047	A00962	205	A00047	A00970	491
A00047	A00975	188	A00047	A00980	475
A00047	A00987	404	A00047	A01000	479
A00047	A01017	480	A00047	A01025	351
A00047	A01031	188	A00047	A01038	205
A00047	A01040	310	A00047	A01042	491
A00047	A01052	238	A00047	A01069	558
A00047	A01075	491	A00047	A01077	263
A00047	A01082	246	A00047	A01087	443
A00047	A01088	491	A00047	A01090	484
A00047	A01091	490	A00047	A01097	440
A00047	A01098	580	A00047	A01101	396
A00047	A01107	180	A00047	A01112	491
A00047	A01113	420	A00047	A01115	659
A00047	A01119	440	A00047	A01120	401
A00047	A01127	299	A00047	A01136	491
A00047	A01140	252	A00047	A01150	491
A00047	A01157	251	A00047	A01166	491
A00047	A01181	177	A00047	A01189	274
A00047	A01192	425	A00047	A01194	491
A00047	A01196	273	A00047	A01201	165
A00047	A01206	491	A00047	A01211	484
A00047	A01215	491	A00047	A01219	307
A00047	A01221	193	A00047	A01224	174
A00047	A01231	171	A00047	A01234	530
A00047	A01242	732	A00047	A01243	491
A00047	A01249	172	A00047	A01259	283
A00047	A01261	207	A00047	A01266	202
A00047	A01267	251	A00047	A01268	235
A00047	A01291	958	A00047	A01299	491
A00047	A01305	162	A00047	A01306	483
A00047	A01311	491	A00047	A01326	323
A00047	A01335	490	A00047	A01339	177
A00047	A01347	154	A00047	A01351	340
A00047	A01353	477	A00047	A01354	491
A00047	A01384	445	A00047	A01393	291
A00047	A01406	485	A00047	A01411	404

A00047	A01418	400	A00047	A01420	481
A00047	A01425	581	A00047	A01431	491
A00047	A01439	159	A00047	A01441	165
A00047	A01468	491	A00047	A01470	491
A00047	A01474	224	A00047	A01492	955
A00047	A01495	465	A00047	A01496	224
A00047	A01506	480	A00047	A01508	467
A00047	A01539	659	A00047	A01540	361
A00047	A01541	608	A00047	A01548	491
A00047	A01555	477	A00047	A01558	945
A00047	A01560	489	A00047	A01562	899
A00047	A01569	491	A00047	A01577	582
A00047	A01579	491	A00047	A01586	154
A00047	A01588	949	A00047	A01593	899
A00047	A01597	345	A00047	A01598	530
A00047	A01612	611	A00047	A01614	491
A00047	A01615	174	A00047	A01638	937
A00047	A01649	491	A00047	A01684	480
A00047	A01692	899	A00047	A01707	491
A00047	A01747	462	A00047	A01749	199
A00047	A01770	491	A00047	A01822	528
A00047	A01841	391	A00047	A01917	479
A00047	A02003	168	A00047	A02064	463
A00047	A02212	258	A00047	A02353	491
A00047	A02452	429	A00047	A02512	281
A00047	A02715	416	A00047	A02718	484
A00047	A02719	252	A00047	A02737	606
A00047	A02787	279	A00047	A02803	899
A00047	A02825	259	A00047	A02830	347
A00047	A02877	401	A00047	A02916	322
A00047	A02980	251	A00047	A03271	899
A00047	A03298	488	A00047	A03304	358
A00047	A03438	230	A00047	A03446	659
A00047	A03448	208	A00047	A03453	393
A00047	A03476	480	A00047	A03477	491
A00047	A03488	294	A00047	A03494	473
A00047	A03557	159	A00047	A03560	491
A00047	A03567	491	A00047	A03569	491
A00047	A03570	308	A00047	A03572	288
A00047	A03586	443	A00047	A03591	184
A00047	A03665	326	A00047	A03669	288
A00047	A03705	360	A00047	A03738	491
A00047	A03740	491	A00047	A03752	491
A00047	A03783	215	A00047	A03785	463
A00047	A03817	429	A00047	A03859	435
A00047	A03860	589	A00047	A03863	490

A00047	A04021	363	A00047	A04124	385
A00047	A04563	218	A00047	A04754	201
A00047	A04949	251	A00047	A05025	404
A00047	A05029	572	A00047	A05037	899
A00047	A05288	579	A00047	A05331	199
A00047	A05417	372	A00047	A05449	157
A00047	A05501	402	A00047	A05557	213
A00047	A05712	491	A00047	A05760	899
A00047	A05995	465	A00047	A06039	199
A00047	A06051	281	A00047	A06109	236
A00047	A06182	272	A00047	A06198	151
A00047	A06275	267	A00047	A06287	302
A00047	A06342	151	A00047	A06359	174
A00047	A06466	491	A00047	A06489	396
A00047	A06531	223	A00047	A06543	475
A00047	A06554	393	A00047	A06599	344
A00047	A06819	409	A00047	A06880	151
A00047	A06892	260	A00047	A06907	510
A00047	A06965	369	A00047	A07004	535
A00047	A07225	151	A00047	A07247	491
A00047	A07311	613	A00047	A07359	151
A00047	A07451	227	A00047	A07487	238
A00047	A07583	293	A00047	A07594	98
A00047	A07604	490	A00047	A07706	559
A00047	A07744	455	A00047	A07746	472
A00047	A07798	488	A00047	A07851	419
A00047	A08022	432	A00047	A08061	491
A00047	A08098	241	A00047	A08118	446
A00047	A08131	173	A00047	A08137	528
A00047	A08178	159	A00047	A08267	188
A00047	A08281	438	A00047	A08357	489
A00047	A08359	491	A00047	A08382	285
A00047	A08390	408	A00047	A08439	185
A00047	A08476	374	A00047	A08546	641
A00047	A08567	196	A00047	A08571	958
A00047	A08679	330	A00047	A08896	445
A00047	A08978	480	A00047	A08987	230
A00047	A08991	340	A00047	A09024	486
A00047	A09032	151	A00047	A09252	208
A00047	A09269	217	A00047	A09327	163
A00047	A09587	329	A00047	A09591	480
A00047	A09619	307	A00047	A09641	367
A00047	A09708	243	A00047	A09857	458
A00047	A09902	314	A00047	A10006	490
A00047	A10159	907	A00047	A10208	199
A00047	A10238	484	A00047	A10266	291

A00047	A10321	371	A00047	A10331	151
A00047	A10399	491	A00047	A10456	462
A00047	A10490	419	A00047	A10532	866
A00047	A10574	338	A00047	A10586	208
A00047	A10615	390	A00047	A10624	491
A00047	A10728	441	A00047	A10847	423
A00047	A10853	224	A00047	A11240	372
A00047	A11277	457	A00047	A11291	377
A00047	A11297	486	A00047	A11301	405
A00047	A11302	348	A00047	A11312	456
A00047	A11316	489	A00047	A11337	236
A00047	A11344	198	A00047	A11368	380
A00047	A11387	427	A00047	A11409	491
A00047	A11420	377	A00047	A11476	446
A00047	A11535	162	A00047	A11621	446
A00047	A11670	401	A00047	A11704	441
A00047	A11705	405	A00047	A11811	201
A00047	A11822	172	A00047	A11933	277
A00047	A11945	221	A00047	A11965	154
A00047	A12096	151	A00047	A12111	460
A00047	A12242	329	A00047	A12325	458
A00047	A12329	286	A00047	A12365	489
A00047	A12390	174	A00047	A12647	172
A00047	A12672	353	A00047	A12722	170
A00047	A12813	247	A00047	A12839	467
A00047	A13135	348	A00047	A13210	151
A00047	A13310	330	A00047	A13311	328
A00047	A13313	154	A00047	A13321	439
A00047	A13340	485	A00047	A13353	151
A00047	A13376	238	A00047	A13388	427
A00047	A13478	151	A00047	A13498	188
A00047	A13552	188	A00047	A13584	151
A00047	A13608	151	A00047	A13609	491
A00047	A13616	151	A00047	A13654	151
A00047	A13661	107	A00047	A13662	151
A00047	A13723	151	A00047	A13870	447
A00047	A13875	187	A00047	A13928	245
A00047	A13989	950	A00047	A14006	491
A00047	A14073	491	A00047	A14074	262
A00047	A14147	208	A00047	A14148	899
A00047	A14150	299	A00047	A14151	246
A00047	A14169	491	A00047	A14182	251
A00047	A14192	204	A00047	A14196	527
A00047	A14200	486	A00047	A14208	899
A00047	A14216	448	A00047	A14220	491
A00047	A14221	150	A00047	A14246	938

A00047	A14257	462	A00047	A14351	457
A00047	A14395	154	A00047	A14476	339
A00047	A14481	199	A00047	A14508	281
A00047	A14525	243	A00047	A14551	423
A00047	A14559	273	A00047	A14594	223
A00047	A14596	491	A00047	A14597	479
A00047	A14636	273	A00047	A14659	337
A00047	A14662	899	A00047	A14672	363
A00047	A14701	324	A00047	A14819	276
A00047	A14836	171	A00047	A14939	151
A00047	A15141	168	A00047	A15142	151
A00047	A15156	167	A00047	A15209	151
A00047	A15244	477	A00047	A15257	265
A00047	A15297	952	A00047	A15309	152
A00047	A15323	899	A00047	A15324	945
A00047	A15338	490	A00047	A15353	945
A00047	A15359	899	A00047	A15366	209
A00047	A15500	229	A00047	A15507	379
A00047	A15547	899	A00047	A15559	637
A00047	A15564	486	A00047	A15614	903
A00047	A15659	308	A00047	A15660	491
A00047	A15684	491	A00047	A15696	353
A00047	A15698	628	A00047	A15717	285
A00047	A15720	168	A00047	A15725	229
A00047	A15729	473	A00047	A15744	151
A00047	A15747	484	A00047	A15752	221
A00047	A15763	489	A00047	A15769	433
A00047	A15773	241	A00047	A15867	190
A00047	A15918	363	A00047	A15933	327
A00047	A15935	294	A00047	A15946	226
A00047	A15947	276	A00047	A16060	458
A00047	A16096	477	A00047	A16118	249
A00047	A16144	226	A00047	A16320	389
A00047	A16394	179	A00047	A16400	163
A00047	A16490	217	A00047	A16756	154
A00047	A16798	352	A00047	A16824	343
A00047	A16834	441	A00047	A17063	491
A00047	A17127	347	A00047	A17131	302
A00047	A17135	173	A00047	A17140	246
A00047	A17142	363	A00048	A00052	174
A00048	A00064	363	A00048	A00067	915
A00048	A00074	588	A00048	A00085	157
A00048	A00090	470	A00048	A00091	770
A00048	A00113	708	A00048	A00118	221
A00048	A00122	219	A00048	A00130	906
A00048	A00138	152	A00048	A00156	682

A00048	A00161	190	A00048	A00162	195
A00048	A00180	172	A00048	A00184	130
A00048	A00185	491	A00048	A00207	662
A00048	A00208	470	A00048	A00214	63
A00048	A00221	424	A00048	A00224	260
A00048	A00227	462	A00048	A00234	285
A00048	A00254	491	A00048	A00292	218
A00048	A00333	374	A00048	A00451	490
A00048	A00478	450	A00048	A00641	414
A00048	A00659	480	A00048	A00697	63
A00048	A00747	183	A00048	A00911	374
A00048	A00939	176	A00048	A01212	180
A00048	A01286	770	A00048	A01307	345
A00048	A01310	93	A00048	A01357	167
A00048	A01390	63	A00048	A01473	63
A00048	A01524	901	A00048	A01527	899
A00048	A01560	430	A00048	A01562	274
A00048	A01692	63	A00048	A01737	396
A00048	A01752	208	A00048	A01754	176
A00048	A01769	251	A00048	A01832	338
A00048	A01846	335	A00048	A01849	193
A00048	A01908	224	A00048	A02011	446
A00048	A02012	299	A00048	A02014	210
A00048	A02015	256	A00048	A02027	227
A00048	A02419	277	A00048	A02425	188
A00048	A02481	480	A00048	A02574	491
A00048	A02598	245	A00048	A02611	483
A00048	A02649	446	A00048	A02661	219
A00048	A02678	160	A00048	A02689	193
A00048	A02696	312	A00048	A02787	477
A00048	A02788	477	A00048	A02803	215
A00048	A02811	176	A00048	A02849	355
A00048	A02864	481	A00048	A02874	450
A00048	A02875	340	A00048	A02887	63
A00048	A02908	348	A00048	A02915	414
A00048	A02931	84	A00048	A02944	196
A00048	A03019	63	A00048	A03034	490
A00048	A03036	382	A00048	A03071	190
A00048	A03092	401	A00048	A03126	160
A00048	A03144	321	A00048	A03195	479
A00048	A03247	198	A00048	A03251	355
A00048	A03254	396	A00048	A03271	474
A00048	A03282	162	A00048	A03480	265
A00048	A03611	371	A00048	A03683	363
A00048	A03684	324	A00048	A03752	371
A00048	A03784	391	A00048	A03803	443

A00048	A03809	177	A00048	A03830	491
A00048	A03831	281	A00048	A03940	281
A00048	A03973	296	A00048	A03990	151
A00048	A04012	224	A00048	A04037	260
A00048	A04080	247	A00048	A04111	270
A00048	A04242	270	A00048	A04323	454
A00048	A04422	152	A00048	A04578	456
A00048	A04579	202	A00048	A04600	368
A00048	A04615	195	A00048	A04697	200
A00048	A04731	432	A00048	A04807	329
A00048	A04830	188	A00048	A04958	288
A00048	A04968	256	A00048	A04972	213
A00048	A05111	265	A00048	A05218	185
A00048	A05339	382	A00048	A05617	425
A00048	A06053	270	A00048	A06138	340
A00048	A06187	333	A00048	A06289	63
A00048	A07056	219	A00048	A07141	168
A00048	A07142	457	A00048	A07155	229
A00048	A07311	940	A00048	A07342	168
A00048	A07373	198	A00048	A07697	485
A00048	A07798	408	A00048	A07860	306
A00048	A08731	422	A00048	A09115	355
A00048	A09229	191	A00048	A09334	260
A00048	A09401	240	A00048	A09890	232
A00048	A09897	333	A00048	A09901	157
A00048	A09983	241	A00048	A09997	176
A00048	A10214	165	A00048	A10365	384
A00048	A10510	252	A00048	A10937	306
A00048	A11129	221	A00048	A11307	151
A00048	A11900	245	A00048	A12969	154
A00048	A12971	167	A00048	A13216	208
A00048	A13605	172	A00048	A13829	63
A00048	A13983	63	A00048	A14071	170
A00048	A14188	358	A00048	A14198	446
A00048	A14344	401	A00048	A14402	176
A00048	A14433	241	A00048	A14438	208
A00048	A14452	63	A00048	A15325	330
A00048	A15382	162	A00048	A15726	63
A00048	A15862	306	A00048	A15913	218
A00048	A16300	229	A00048	A16547	339
A00048	A16594	247	A00048	A16989	355
A00049	A00050	945	A00049	A00052	944
A00049	A00067	918	A00049	A00162	945
A00049	A00167	899	A00049	A00205	177
A00049	A00209	902	A00049	A00224	899
A00049	A00235	327	A00049	A00270	945

A00049	A00271	899	A00049	A00381	382
A00049	A00400	447	A00049	A00531	406
A00049	A01139	294	A00049	A01359	435
A00049	A01558	899	A00049	A01560	249
A00049	A01588	899	A00049	A02502	443
A00049	A03651	228	A00049	A03939	235
A00049	A06610	207	A00049	A09258	252
A00049	A09377	317	A00049	A09451	210
A00049	A09515	208	A00049	A09538	402
A00049	A10340	319	A00049	A10833	470
A00049	A11492	357	A00049	A13250	173
A00049	A13981	899	A00049	A13990	945
A00049	A14278	162	A00050	A00052	755
A00050	A00058	172	A00050	A00066	257
A00050	A00067	953	A00050	A00112	384
A00050	A00132	400	A00050	A00138	191
A00050	A00146	165	A00050	A00147	274
A00050	A00162	945	A00050	A00167	927
A00050	A00206	341	A00050	A00209	899
A00050	A00235	455	A00050	A00254	258
A00050	A00257	291	A00050	A00263	174
A00050	A00269	582	A00050	A00270	968
A00050	A00271	899	A00050	A00275	899
A00050	A00381	274	A00050	A00400	491
A00050	A00437	260	A00050	A00471	408
A00050	A00531	277	A00050	A00874	174
A00050	A01044	256	A00050	A01139	273
A00050	A01212	374	A00050	A01359	400
A00050	A01410	207	A00050	A01524	174
A00050	A01527	899	A00050	A01560	208
A00050	A01591	333	A00050	A01703	212
A00050	A02195	224	A00050	A02473	202
A00050	A02502	302	A00050	A03101	159
A00050	A03307	270	A00050	A03492	391
A00050	A03651	456	A00050	A03768	235
A00050	A04025	185	A00050	A04491	160
A00050	A05315	205	A00050	A05467	174
A00050	A05734	193	A00050	A06118	204
A00050	A07311	269	A00050	A07655	168
A00050	A08148	224	A00050	A08571	461
A00050	A08688	302	A00050	A09218	296
A00050	A09229	170	A00050	A09258	286
A00050	A09377	232	A00050	A09411	221
A00050	A09538	316	A00050	A10340	235
A00050	A10384	343	A00050	A10833	286
A00050	A11492	288	A00050	A11621	185

A00050	A11696	291	A00050	A12204	380
A00050	A12284	238	A00050	A12532	301
A00050	A12534	270	A00050	A13511	195
A00050	A13826	183	A00050	A13929	281
A00050	A13980	394	A00050	A13990	945
A00050	A14290	196	A00050	A14496	499
A00050	A14584	245	A00050	A15916	180
A00050	A16665	212	A00051	A00094	899
A00051	A00095	918	A00051	A00142	919
A00051	A00143	924	A00051	A00168	899
A00051	A00200	899	A00051	A00212	467
A00051	A00215	945	A00051	A00457	205
A00051	A00666	205	A00051	A00962	154
A00051	A01008	141	A00051	A01211	218
A00051	A01482	390	A00051	A01508	210
A00051	A03477	461	A00051	A04202	256
A00051	A05310	313	A00051	A05703	408
A00051	A06608	899	A00051	A06979	344
A00051	A07189	395	A00051	A10420	241
A00051	A13474	404	A00051	A14122	252
A00051	A14511	180	A00052	A00053	944
A00052	A00056	416	A00052	A00058	483
A00052	A00064	491	A00052	A00065	944
A00052	A00066	969	A00052	A00067	980
A00052	A00068	489	A00052	A00069	928
A00052	A00071	326	A00052	A00073	491
A00052	A00074	945	A00052	A00081	435
A00052	A00085	296	A00052	A00086	185
A00052	A00090	944	A00052	A00093	899
A00052	A00094	968	A00052	A00099	899
A00052	A00100	936	A00052	A00111	335
A00052	A00113	154	A00052	A00117	899
A00052	A00120	899	A00052	A00121	899
A00052	A00122	380	A00052	A00123	904
A00052	A00124	938	A00052	A00127	899
A00052	A00128	926	A00052	A00132	475
A00052	A00133	899	A00052	A00135	899
A00052	A00136	371	A00052	A00137	200
A00052	A00138	796	A00052	A00140	899
A00052	A00141	902	A00052	A00145	901
A00052	A00146	201	A00052	A00147	491
A00052	A00149	899	A00052	A00150	903
A00052	A00151	944	A00052	A00152	575
A00052	A00153	945	A00052	A00154	945
A00052	A00156	281	A00052	A00157	913
A00052	A00158	196	A00052	A00159	995

A00052	A00162	945	A00052	A00163	945
A00052	A00165	899	A00052	A00167	949
A00052	A00169	945	A00052	A00170	899
A00052	A00171	945	A00052	A00173	945
A00052	A00174	899	A00052	A00175	899
A00052	A00184	900	A00052	A00185	484
A00052	A00186	902	A00052	A00188	945
A00052	A00189	899	A00052	A00190	491
A00052	A00191	904	A00052	A00196	321
A00052	A00198	899	A00052	A00200	945
A00052	A00201	491	A00052	A00203	899
A00052	A00204	306	A00052	A00205	917
A00052	A00206	822	A00052	A00207	968
A00052	A00208	994	A00052	A00209	994
A00052	A00210	907	A00052	A00211	899
A00052	A00219	945	A00052	A00220	899
A00052	A00221	972	A00052	A00223	945
A00052	A00224	958	A00052	A00225	185
A00052	A00229	930	A00052	A00230	904
A00052	A00231	271	A00052	A00233	940
A00052	A00234	949	A00052	A00235	323
A00052	A00236	951	A00052	A00237	899
A00052	A00239	479	A00052	A00240	945
A00052	A00241	899	A00052	A00242	899
A00052	A00246	938	A00052	A00248	944
A00052	A00250	925	A00052	A00252	944
A00052	A00253	945	A00052	A00254	945
A00052	A00255	910	A00052	A00256	491
A00052	A00257	945	A00052	A00261	911
A00052	A00263	164	A00052	A00264	490
A00052	A00265	491	A00052	A00266	903
A00052	A00268	899	A00052	A00269	926
A00052	A00270	949	A00052	A00271	943
A00052	A00272	933	A00052	A00275	899
A00052	A00276	901	A00052	A00294	909
A00052	A00316	477	A00052	A00324	992
A00052	A00337	230	A00052	A00340	304
A00052	A00348	168	A00052	A00356	899
A00052	A00358	899	A00052	A00378	942
A00052	A00381	485	A00052	A00469	199
A00052	A00509	247	A00052	A00517	449
A00052	A00552	312	A00052	A00556	491
A00052	A00558	488	A00052	A00616	485
A00052	A00624	227	A00052	A00630	228
A00052	A00640	484	A00052	A00651	899
A00052	A00655	218	A00052	A00660	154

A00052	A00669	899	A00052	A00696	208
A00052	A00697	712	A00052	A00724	290
A00052	A00736	491	A00052	A00747	945
A00052	A00786	899	A00052	A00820	335
A00052	A00825	310	A00052	A00826	230
A00052	A00832	490	A00052	A00839	185
A00052	A00851	899	A00052	A00914	486
A00052	A00930	157	A00052	A00933	899
A00052	A00939	899	A00052	A00980	200
A00052	A00999	423	A00052	A01002	899
A00052	A01052	910	A00052	A01056	899
A00052	A01070	899	A00052	A01077	177
A00052	A01098	165	A00052	A01102	481
A00052	A01125	165	A00052	A01131	277
A00052	A01168	899	A00052	A01176	929
A00052	A01178	491	A00052	A01189	435
A00052	A01244	899	A00052	A01289	960
A00052	A01291	903	A00052	A01309	230
A00052	A01310	180	A00052	A01354	491
A00052	A01400	316	A00052	A01404	272
A00052	A01405	249	A00052	A01415	899
A00052	A01425	905	A00052	A01452	439
A00052	A01485	229	A00052	A01496	899
A00052	A01507	899	A00052	A01512	909
A00052	A01524	945	A00052	A01527	994
A00052	A01553	949	A00052	A01554	949
A00052	A01558	901	A00052	A01560	976
A00052	A01561	949	A00052	A01562	944
A00052	A01563	160	A00052	A01571	228
A00052	A01572	680	A00052	A01588	989
A00052	A01591	989	A00052	A01592	938
A00052	A01593	899	A00052	A01594	230
A00052	A01600	401	A00052	A01602	908
A00052	A01604	899	A00052	A01605	945
A00052	A01607	204	A00052	A01615	994
A00052	A01626	377	A00052	A01628	945
A00052	A01629	235	A00052	A01632	469
A00052	A01633	171	A00052	A01636	899
A00052	A01638	899	A00052	A01639	945
A00052	A01640	945	A00052	A01650	991
A00052	A01651	899	A00052	A01661	391
A00052	A01665	899	A00052	A01668	489
A00052	A01675	905	A00052	A01676	467
A00052	A01680	899	A00052	A01686	899
A00052	A01688	899	A00052	A01692	982
A00052	A01697	974	A00052	A01702	951

A00052	A01708	941	A00052	A01709	929
A00052	A01717	899	A00052	A01718	921
A00052	A01719	932	A00052	A01726	490
A00052	A01727	467	A00052	A01729	489
A00052	A01730	951	A00052	A01731	377
A00052	A01733	241	A00052	A01739	218
A00052	A01740	491	A00052	A01741	491
A00052	A01742	163	A00052	A01743	489
A00052	A01746	435	A00052	A01747	283
A00052	A01748	286	A00052	A01752	486
A00052	A01755	229	A00052	A01760	272
A00052	A01763	260	A00052	A01770	491
A00052	A01774	380	A00052	A01775	363
A00052	A01781	266	A00052	A01796	165
A00052	A01797	475	A00052	A01798	259
A00052	A01812	474	A00052	A01844	942
A00052	A01876	369	A00052	A01877	407
A00052	A01878	180	A00052	A01894	191
A00052	A01897	345	A00052	A01898	899
A00052	A01900	938	A00052	A01903	446
A00052	A01905	919	A00052	A01907	160
A00052	A01908	489	A00052	A01930	171
A00052	A02020	394	A00052	A02045	154
A00052	A02054	162	A00052	A02094	902
A00052	A02110	200	A00052	A02117	343
A00052	A02143	317	A00052	A02180	407
A00052	A02185	188	A00052	A02196	154
A00052	A02218	154	A00052	A02278	414
A00052	A02388	152	A00052	A02390	224
A00052	A02407	168	A00052	A02411	922
A00052	A02463	162	A00052	A02469	207
A00052	A02470	236	A00052	A02491	152
A00052	A02493	316	A00052	A02494	490
A00052	A02495	445	A00052	A02501	279
A00052	A02502	906	A00052	A02512	443
A00052	A02544	273	A00052	A02562	318
A00052	A02564	480	A00052	A02568	435
A00052	A02575	201	A00052	A02586	160
A00052	A02593	440	A00052	A02614	152
A00052	A02621	229	A00052	A02632	294
A00052	A02636	162	A00052	A02672	154
A00052	A02687	160	A00052	A02695	445
A00052	A02698	489	A00052	A02702	151
A00052	A02703	490	A00052	A02704	454
A00052	A02705	383	A00052	A02706	921
A00052	A02711	229	A00052	A02715	267

A00052	A02722	255	A00052	A02737	251
A00052	A02741	353	A00052	A02749	475
A00052	A02752	210	A00052	A02754	221
A00052	A02758	201	A00052	A02766	193
A00052	A02779	944	A00052	A02788	180
A00052	A02792	400	A00052	A02795	249
A00052	A02803	489	A00052	A02815	152
A00052	A02816	490	A00052	A02819	180
A00052	A02827	364	A00052	A02830	490
A00052	A02836	469	A00052	A02837	330
A00052	A02842	156	A00052	A02859	411
A00052	A02860	467	A00052	A02861	190
A00052	A02874	249	A00052	A02877	491
A00052	A02878	491	A00052	A02895	457
A00052	A02902	229	A00052	A02904	389
A00052	A02908	899	A00052	A02914	360
A00052	A02924	465	A00052	A02927	435
A00052	A02930	229	A00052	A02968	899
A00052	A02982	396	A00052	A02995	235
A00052	A03012	452	A00052	A03028	172
A00052	A03056	443	A00052	A03057	447
A00052	A03112	491	A00052	A03115	167
A00052	A03121	472	A00052	A03157	328
A00052	A03211	481	A00052	A03212	378
A00052	A03213	156	A00052	A03259	245
A00052	A03271	923	A00052	A03276	319
A00052	A03288	283	A00052	A03321	490
A00052	A03332	899	A00052	A03338	215
A00052	A03415	472	A00052	A03423	226
A00052	A03431	457	A00052	A03436	452
A00052	A03451	491	A00052	A03523	391
A00052	A03568	279	A00052	A03575	899
A00052	A03591	987	A00052	A03596	406
A00052	A03609	180	A00052	A03611	473
A00052	A03622	154	A00052	A03649	385
A00052	A03661	419	A00052	A03662	928
A00052	A03663	157	A00052	A03725	168
A00052	A03726	899	A00052	A03739	489
A00052	A03741	899	A00052	A03752	899
A00052	A03796	213	A00052	A03817	480
A00052	A03843	491	A00052	A03913	899
A00052	A03936	157	A00052	A03990	291
A00052	A04006	293	A00052	A04012	553
A00052	A04046	207	A00052	A04117	260
A00052	A04161	582	A00052	A04209	245
A00052	A04245	163	A00052	A04280	489

A00052	A04308	297	A00052	A04342	374
A00052	A04367	163	A00052	A04536	439
A00052	A04571	215	A00052	A04600	899
A00052	A04622	491	A00052	A04699	901
A00052	A04722	165	A00052	A04761	899
A00052	A04861	369	A00052	A05008	899
A00052	A05009	899	A00052	A05015	899
A00052	A05029	899	A00052	A05032	430
A00052	A05034	412	A00052	A05045	899
A00052	A05058	202	A00052	A05062	899
A00052	A05070	313	A00052	A05105	154
A00052	A05112	424	A00052	A05122	205
A00052	A05216	935	A00052	A05288	734
A00052	A05336	154	A00052	A05418	443
A00052	A05432	490	A00052	A05453	945
A00052	A05508	202	A00052	A05573	290
A00052	A05574	163	A00052	A05656	154
A00052	A05719	171	A00052	A05795	157
A00052	A05832	323	A00052	A05866	172
A00052	A05867	899	A00052	A05871	249
A00052	A05913	154	A00052	A05933	154
A00052	A05960	267	A00052	A05961	394
A00052	A05996	899	A00052	A06039	199
A00052	A06132	152	A00052	A06216	162
A00052	A06241	215	A00052	A06258	232
A00052	A06286	402	A00052	A06313	152
A00052	A06333	165	A00052	A06442	154
A00052	A06449	296	A00052	A06511	154
A00052	A06581	488	A00052	A06588	491
A00052	A06597	491	A00052	A06600	212
A00052	A06606	165	A00052	A06607	491
A00052	A06608	945	A00052	A06609	901
A00052	A06610	582	A00052	A06615	63
A00052	A06623	452	A00052	A06646	154
A00052	A06648	438	A00052	A06650	488
A00052	A06677	176	A00052	A06688	491
A00052	A06714	154	A00052	A06748	243
A00052	A06795	313	A00052	A06797	466
A00052	A06818	207	A00052	A06949	491
A00052	A07059	157	A00052	A07096	425
A00052	A07123	350	A00052	A07135	553
A00052	A07141	383	A00052	A07158	439
A00052	A07163	490	A00052	A07165	940
A00052	A07177	156	A00052	A07311	980
A00052	A07320	213	A00052	A07339	489
A00052	A07381	247	A00052	A07393	154

A00052	A07422	945	A00052	A07476	154
A00052	A07486	385	A00052	A07546	193
A00052	A07628	154	A00052	A07695	199
A00052	A07723	462	A00052	A07964	154
A00052	A07971	371	A00052	A08065	154
A00052	A08076	243	A00052	A08221	346
A00052	A08349	154	A00052	A08438	290
A00052	A08571	903	A00052	A08572	436
A00052	A08608	402	A00052	A08643	268
A00052	A08701	232	A00052	A08738	258
A00052	A08782	154	A00052	A08839	899
A00052	A08858	899	A00052	A08862	386
A00052	A08865	899	A00052	A08904	199
A00052	A08943	180	A00052	A09016	154
A00052	A09020	358	A00052	A09033	416
A00052	A09095	213	A00052	A09220	904
A00052	A09260	154	A00052	A09261	154
A00052	A09319	899	A00052	A09383	930
A00052	A09428	200	A00052	A09542	899
A00052	A09568	966	A00052	A09577	927
A00052	A09644	283	A00052	A09804	346
A00052	A09931	154	A00052	A09937	217
A00052	A10014	200	A00052	A10018	207
A00052	A10103	469	A00052	A10148	434
A00052	A10174	899	A00052	A10182	243
A00052	A10185	899	A00052	A10233	899
A00052	A10238	246	A00052	A10247	466
A00052	A10313	294	A00052	A10365	932
A00052	A10392	899	A00052	A10430	290
A00052	A10463	154	A00052	A10510	200
A00052	A10512	154	A00052	A10560	899
A00052	A10580	899	A00052	A10623	899
A00052	A10625	455	A00052	A10744	176
A00052	A10756	422	A00052	A10801	899
A00052	A10808	917	A00052	A10811	899
A00052	A10833	928	A00052	A10857	154
A00052	A10888	213	A00052	A11078	213
A00052	A11124	414	A00052	A11125	899
A00052	A11174	243	A00052	A11335	308
A00052	A11369	475	A00052	A11392	174
A00052	A11485	196	A00052	A11487	167
A00052	A11499	154	A00052	A11820	704
A00052	A11896	243	A00052	A11943	945
A00052	A11981	160	A00052	A12124	374
A00052	A12373	301	A00052	A12383	942
A00052	A12393	334	A00052	A12510	154

A00052	A12654	160	A00052	A12664	899
A00052	A12705	899	A00052	A12835	480
A00052	A12890	243	A00052	A12915	243
A00052	A12916	213	A00052	A12971	899
A00052	A12988	234	A00052	A12993	154
A00052	A13207	188	A00052	A13240	212
A00052	A13250	899	A00052	A13391	916
A00052	A13393	899	A00052	A13511	236
A00052	A13531	420	A00052	A13584	154
A00052	A13615	154	A00052	A13712	154
A00052	A13734	413	A00052	A13917	475
A00052	A13945	899	A00052	A13977	899
A00052	A13978	930	A00052	A13981	989
A00052	A13985	899	A00052	A13989	899
A00052	A13990	916	A00052	A13999	899
A00052	A14000	899	A00052	A14005	205
A00052	A14015	899	A00052	A14019	899
A00052	A14024	899	A00052	A14027	899
A00052	A14033	912	A00052	A14035	899
A00052	A14038	899	A00052	A14062	240
A00052	A14065	899	A00052	A14188	899
A00052	A14190	945	A00052	A14198	952
A00052	A14208	899	A00052	A14366	182
A00052	A14371	486	A00052	A14399	345
A00052	A14402	943	A00052	A14443	989
A00052	A14444	899	A00052	A14496	229
A00052	A14506	899	A00052	A14511	174
A00052	A14605	490	A00052	A14638	174
A00052	A14643	486	A00052	A14648	485
A00052	A14784	154	A00052	A14808	243
A00052	A14938	154	A00052	A14975	154
A00052	A15233	314	A00052	A15242	154
A00052	A15293	899	A00052	A15294	907
A00052	A15295	544	A00052	A15498	330
A00052	A15611	908	A00052	A15665	245
A00052	A15726	944	A00052	A15869	152
A00052	A15910	177	A00052	A15932	200
A00052	A15935	207	A00052	A16003	154
A00052	A16029	154	A00052	A16100	215
A00052	A16281	235	A00052	A16399	193
A00052	A16636	190	A00052	A16741	449
A00052	A16847	290	A00052	A16909	899
A00052	A17160	899	A00052	A17188	899
A00052	A17203	163	A00052	A17204	154
A00053	A00056	74	A00053	A00057	63
A00053	A00067	899	A00053	A00069	945

A00053	A00081	490	A00053	A00095	456
A00053	A00131	199	A00053	A00157	899
A00053	A00159	899	A00053	A00173	945
A00053	A00175	204	A00053	A00196	372
A00053	A00209	899	A00053	A00210	491
A00053	A00212	256	A00053	A00219	288
A00053	A00221	286	A00053	A00224	904
A00053	A00227	154	A00053	A00228	461
A00053	A00230	902	A00053	A00239	252
A00053	A00240	551	A00053	A00243	412
A00053	A00246	905	A00053	A00247	491
A00053	A00254	154	A00053	A00267	324
A00053	A00309	165	A00053	A00358	238
A00053	A00385	279	A00053	A00413	213
A00053	A00446	228	A00053	A00469	191
A00053	A00470	156	A00053	A00473	171
A00053	A00776	268	A00053	A00781	475
A00053	A00805	195	A00053	A00816	446
A00053	A00932	491	A00053	A00975	458
A00053	A00989	63	A00053	A01009	63
A00053	A01014	299	A00053	A01057	294
A00053	A01077	162	A00053	A01124	107
A00053	A01157	191	A00053	A01163	177
A00053	A01169	219	A00053	A01289	156
A00053	A01325	395	A00053	A01363	98
A00053	A01462	213	A00053	A01485	483
A00053	A01524	902	A00053	A01527	929
A00053	A01553	917	A00053	A01558	899
A00053	A01561	927	A00053	A01593	157
A00053	A01605	241	A00053	A01615	899
A00053	A01624	235	A00053	A01629	90
A00053	A01640	905	A00053	A01718	945
A00053	A01722	165	A00053	A01974	167
A00053	A01985	141	A00053	A02017	170
A00053	A02035	267	A00053	A02049	468
A00053	A02072	240	A00053	A02091	266
A00053	A02106	489	A00053	A02124	419
A00053	A02126	230	A00053	A02148	241
A00053	A02185	469	A00053	A02189	165
A00053	A02191	455	A00053	A02233	288
A00053	A02239	227	A00053	A02251	91
A00053	A02260	149	A00053	A02278	363
A00053	A02291	152	A00053	A02303	396
A00053	A02304	347	A00053	A02322	199
A00053	A02360	174	A00053	A02392	235
A00053	A02423	110	A00053	A02424	288

A00053	A02425	238	A00053	A02463	151
A00053	A02465	191	A00053	A02467	942
A00053	A02481	63	A00053	A02501	215
A00053	A02583	180	A00053	A02666	160
A00053	A02671	193	A00053	A02760	279
A00053	A03041	380	A00053	A03045	180
A00053	A03053	152	A00053	A03054	398
A00053	A03055	191	A00053	A03096	307
A00053	A03097	167	A00053	A03110	346
A00053	A03115	333	A00053	A03126	63
A00053	A03176	307	A00053	A03177	472
A00053	A03209	467	A00053	A03331	429
A00053	A03531	434	A00053	A03633	319
A00053	A03642	276	A00053	A03753	230
A00053	A03820	382	A00053	A03942	345
A00053	A04074	479	A00053	A04098	333
A00053	A04171	213	A00053	A04180	185
A00053	A04181	213	A00053	A04218	180
A00053	A04280	168	A00053	A04347	759
A00053	A04351	162	A00053	A04381	276
A00053	A04388	333	A00053	A04394	230
A00053	A04417	230	A00053	A04423	172
A00053	A04465	241	A00053	A04471	199
A00053	A04751	173	A00053	A04759	252
A00053	A04808	230	A00053	A04813	156
A00053	A04911	299	A00053	A05005	159
A00053	A05052	204	A00053	A05276	165
A00053	A05826	235	A00053	A05899	257
A00053	A05965	276	A00053	A06234	241
A00053	A06243	257	A00053	A06409	276
A00053	A06489	319	A00053	A06517	266
A00053	A06538	333	A00053	A06623	327
A00053	A06648	172	A00053	A07063	213
A00053	A07091	899	A00053	A07311	899
A00053	A07485	333	A00053	A07614	288
A00053	A07615	333	A00053	A08070	340
A00053	A08194	307	A00053	A08810	274
A00053	A08869	360	A00053	A09076	196
A00053	A09111	350	A00053	A09196	266
A00053	A09200	184	A00053	A09230	235
A00053	A09360	307	A00053	A09784	174
A00053	A09814	199	A00053	A09906	249
A00053	A09943	196	A00053	A09970	288
A00053	A10210	235	A00053	A10221	230
A00053	A10665	205	A00053	A10949	272
A00053	A11014	168	A00053	A11352	276

A00053	A11584	202	A00053	A12651	480
A00053	A12990	730	A00053	A13443	288
A00053	A13537	266	A00053	A13925	333
A00053	A14096	218	A00053	A14190	283
A00053	A14267	308	A00053	A14352	184
A00053	A14371	390	A00053	A14443	900
A00053	A14642	215	A00053	A14722	276
A00053	A15277	216	A00053	A15493	235
A00053	A15498	385	A00053	A15543	491
A00053	A15551	208	A00053	A15704	213
A00053	A15795	193	A00053	A15809	358
A00053	A16042	165	A00053	A16911	210
A00054	A01307	201	A00054	A03569	183
A00054	A14196	118	A00054	A14208	168
A00054	A15314	174	A00054	A15330	310
A00054	A15360	290	A00054	A15361	257
A00055	A00073	165	A00055	A00153	208
A00055	A00163	279	A00055	A00166	352
A00055	A00167	438	A00055	A00168	359
A00055	A00171	302	A00055	A00211	290
A00055	A00219	247	A00055	A00253	366
A00055	A00286	243	A00055	A00294	256
A00055	A00316	454	A00055	A00372	488
A00055	A00386	281	A00055	A00396	744
A00055	A00400	201	A00055	A00413	378
A00055	A00421	202	A00055	A00426	490
A00055	A00429	486	A00055	A00445	489
A00055	A00446	483	A00055	A00496	154
A00055	A00498	417	A00055	A00532	491
A00055	A00541	223	A00055	A00552	441
A00055	A00560	232	A00055	A00565	658
A00055	A00568	317	A00055	A00571	491
A00055	A00609	227	A00055	A00614	223
A00055	A00645	190	A00055	A00658	490
A00055	A00664	544	A00055	A00670	262
A00055	A00684	313	A00055	A00718	165
A00055	A00719	536	A00055	A00805	408
A00055	A00826	163	A00055	A00831	165
A00055	A00835	486	A00055	A00853	488
A00055	A00855	649	A00055	A00862	490
A00055	A00868	353	A00055	A00878	241
A00055	A00883	491	A00055	A00888	396
A00055	A00916	307	A00055	A00939	179
A00055	A00957	257	A00055	A00968	553
A00055	A00971	232	A00055	A00981	291
A00055	A01021	491	A00055	A01022	313

A00055	A01036	207	A00055	A01052	217
A00055	A01074	709	A00055	A01075	429
A00055	A01088	301	A00055	A01091	491
A00055	A01097	165	A00055	A01105	317
A00055	A01125	322	A00055	A01132	224
A00055	A01143	162	A00055	A01172	157
A00055	A01189	223	A00055	A01209	642
A00055	A01211	480	A00055	A01215	447
A00055	A01234	458	A00055	A01243	441
A00055	A01244	463	A00055	A01259	490
A00055	A01352	224	A00055	A01353	488
A00055	A01354	256	A00055	A01373	441
A00055	A01375	491	A00055	A01386	241
A00055	A01406	491	A00055	A01451	486
A00055	A01558	417	A00055	A01588	268
A00055	A01598	204	A00055	A01612	443
A00055	A01622	327	A00055	A01660	409
A00055	A01679	317	A00055	A01705	168
A00055	A01730	327	A00055	A01995	413
A00055	A02180	461	A00055	A02206	177
A00055	A02212	456	A00055	A02219	481
A00055	A02335	223	A00055	A02452	491
A00055	A02500	177	A00055	A02789	223
A00055	A03057	486	A00055	A03297	217
A00055	A03390	236	A00055	A03401	491
A00055	A03436	346	A00055	A03569	252
A00055	A03710	367	A00055	A03719	223
A00055	A03728	486	A00055	A03752	223
A00055	A03764	481	A00055	A03817	301
A00055	A03990	454	A00055	A04171	249
A00055	A04178	218	A00055	A04189	263
A00055	A04190	190	A00055	A04403	205
A00055	A04640	312	A00055	A04773	235
A00055	A04844	254	A00055	A05016	232
A00055	A05058	196	A00055	A05327	258
A00055	A05332	317	A00055	A05609	317
A00055	A05690	163	A00055	A05967	276
A00055	A06010	173	A00055	A06085	328
A00055	A06162	491	A00055	A06472	428
A00055	A06502	312	A00055	A06660	201
A00055	A06689	223	A00055	A06819	223
A00055	A06907	428	A00055	A06924	486
A00055	A06990	165	A00055	A07054	317
A00055	A07222	317	A00055	A07333	317
A00055	A07339	361	A00055	A07571	317
A00055	A07598	174	A00055	A07604	423

A00055	A07706	180	A00055	A07880	481
A00055	A07927	263	A00055	A07942	157
A00055	A07953	263	A00055	A07956	274
A00055	A07974	321	A00055	A08097	317
A00055	A08137	451	A00055	A08281	367
A00055	A08376	168	A00055	A08571	424
A00055	A08982	419	A00055	A09067	285
A00055	A09340	317	A00055	A09346	202
A00055	A09781	263	A00055	A10090	160
A00055	A11121	355	A00055	A11277	224
A00055	A11291	361	A00055	A11385	380
A00055	A11394	245	A00055	A11398	201
A00055	A11574	411	A00055	A11614	414
A00055	A11767	191	A00055	A12020	240
A00055	A12363	245	A00055	A12415	263
A00055	A12598	190	A00055	A12966	312
A00055	A13081	168	A00055	A13822	223
A00055	A13876	224	A00055	A14006	267
A00055	A14196	548	A00055	A14221	271
A00055	A14266	260	A00055	A14272	188
A00055	A14394	274	A00055	A14452	443
A00055	A14511	396	A00055	A14525	223
A00055	A14690	290	A00055	A14804	223
A00055	A15232	263	A00055	A15237	374
A00055	A15273	235	A00055	A15297	491
A00055	A15309	488	A00055	A15323	537
A00055	A15324	324	A00055	A15338	291
A00055	A15355	208	A00055	A15366	473
A00055	A15372	310	A00055	A15419	451
A00055	A15431	408	A00055	A15510	180
A00055	A15559	486	A00055	A15564	310
A00055	A15747	380	A00055	A15788	512
A00055	A15837	491	A00055	A15867	223
A00055	A16219	223	A00055	A16410	249
A00055	A16531	223	A00055	A16861	190
A00055	A17063	475	A00056	A00057	945
A00056	A00058	899	A00056	A00059	900
A00056	A00061	491	A00056	A00063	463
A00056	A00067	909	A00056	A00069	443
A00056	A00073	899	A00056	A00076	200
A00056	A00078	124	A00056	A00080	375
A00056	A00081	63	A00056	A00085	198
A00056	A00086	213	A00056	A00109	491
A00056	A00111	173	A00056	A00112	407
A00056	A00119	204	A00056	A00123	945
A00056	A00125	490	A00056	A00152	952

A00056	A00153	490	A00056	A00157	915
A00056	A00158	447	A00056	A00163	488
A00056	A00167	937	A00056	A00173	256
A00056	A00174	293	A00056	A00189	396
A00056	A00190	484	A00056	A00195	283
A00056	A00196	419	A00056	A00198	398
A00056	A00204	929	A00056	A00209	942
A00056	A00210	115	A00056	A00219	484
A00056	A00220	491	A00056	A00221	180
A00056	A00233	239	A00056	A00234	251
A00056	A00238	899	A00056	A00239	362
A00056	A00240	154	A00056	A00246	899
A00056	A00248	332	A00056	A00255	346
A00056	A00261	372	A00056	A00264	945
A00056	A00274	491	A00056	A00292	486
A00056	A00309	260	A00056	A00331	207
A00056	A00338	339	A00056	A00472	308
A00056	A00476	460	A00056	A00561	899
A00056	A00616	574	A00056	A00625	195
A00056	A00630	356	A00056	A00688	465
A00056	A00694	451	A00056	A00736	347
A00056	A00752	475	A00056	A00786	202
A00056	A00799	899	A00056	A00804	160
A00056	A00875	356	A00056	A00911	208
A00056	A00914	369	A00056	A00981	541
A00056	A01009	479	A00056	A01049	385
A00056	A01125	163	A00056	A01138	213
A00056	A01161	276	A00056	A01169	537
A00056	A01178	65	A00056	A01277	260
A00056	A01289	457	A00056	A01461	190
A00056	A01514	188	A00056	A01515	216
A00056	A01524	945	A00056	A01527	945
A00056	A01574	224	A00056	A01575	168
A00056	A01585	185	A00056	A01604	490
A00056	A01640	160	A00056	A01650	152
A00056	A01654	491	A00056	A01658	469
A00056	A01663	383	A00056	A01668	277
A00056	A01683	256	A00056	A01691	399
A00056	A01704	416	A00056	A01709	899
A00056	A01729	229	A00056	A01741	479
A00056	A01743	373	A00056	A01747	470
A00056	A01767	297	A00056	A01799	213
A00056	A01867	297	A00056	A01870	488
A00056	A01876	179	A00056	A01879	383
A00056	A01880	485	A00056	A01881	409
A00056	A01887	172	A00056	A01888	488

A00056	A01889	297	A00056	A01890	307
A00056	A01936	215	A00056	A01984	177
A00056	A02000	191	A00056	A02006	245
A00056	A02015	173	A00056	A02044	77
A00056	A02052	374	A00056	A02111	296
A00056	A02138	174	A00056	A02141	135
A00056	A02143	491	A00056	A02164	229
A00056	A02170	369	A00056	A02176	238
A00056	A02186	157	A00056	A02217	210
A00056	A02247	322	A00056	A02248	474
A00056	A02249	190	A00056	A02251	473
A00056	A02263	177	A00056	A02278	401
A00056	A02281	267	A00056	A02288	157
A00056	A02298	205	A00056	A02299	173
A00056	A02300	162	A00056	A02307	249
A00056	A02333	461	A00056	A02337	221
A00056	A02382	490	A00056	A02384	279
A00056	A02416	491	A00056	A02417	297
A00056	A02418	138	A00056	A02423	347
A00056	A02424	63	A00056	A02439	380
A00056	A02464	71	A00056	A02465	129
A00056	A02471	154	A00056	A02473	363
A00056	A02481	63	A00056	A02488	322
A00056	A02490	486	A00056	A02491	490
A00056	A02493	396	A00056	A02494	490
A00056	A02495	234	A00056	A02497	322
A00056	A02515	201	A00056	A02575	341
A00056	A02579	234	A00056	A02582	378
A00056	A02585	168	A00056	A02590	301
A00056	A02593	177	A00056	A02606	399
A00056	A02609	188	A00056	A02615	484
A00056	A02616	468	A00056	A02624	386
A00056	A02629	202	A00056	A02632	260
A00056	A02638	435	A00056	A02639	458
A00056	A02659	208	A00056	A02668	177
A00056	A02674	177	A00056	A02693	268
A00056	A02698	488	A00056	A02711	454
A00056	A02730	260	A00056	A02733	196
A00056	A02745	228	A00056	A02749	184
A00056	A02756	288	A00056	A02769	353
A00056	A02771	112	A00056	A02783	327
A00056	A02787	391	A00056	A02788	486
A00056	A02812	205	A00056	A02819	463
A00056	A02836	452	A00056	A02837	291
A00056	A02854	245	A00056	A02859	363
A00056	A02863	477	A00056	A02874	188

A00056	A02875	199	A00056	A02884	151
A00056	A02896	456	A00056	A02917	484
A00056	A02931	366	A00056	A02944	252
A00056	A02945	486	A00056	A02955	174
A00056	A03014	308	A00056	A03051	283
A00056	A03053	107	A00056	A03055	366
A00056	A03063	201	A00056	A03068	944
A00056	A03081	480	A00056	A03093	208
A00056	A03095	205	A00056	A03110	446
A00056	A03128	219	A00056	A03129	173
A00056	A03183	486	A00056	A03185	380
A00056	A03186	416	A00056	A03218	216
A00056	A03219	435	A00056	A03224	480
A00056	A03228	400	A00056	A03243	208
A00056	A03259	507	A00056	A03263	408
A00056	A03282	191	A00056	A03305	297
A00056	A03315	232	A00056	A03319	157
A00056	A03386	297	A00056	A03390	223
A00056	A03399	180	A00056	A03436	151
A00056	A03438	477	A00056	A03451	463
A00056	A03488	452	A00056	A03537	188
A00056	A03540	168	A00056	A03610	412
A00056	A03630	435	A00056	A03636	238
A00056	A03639	196	A00056	A03695	201
A00056	A03748	243	A00056	A03753	270
A00056	A03790	375	A00056	A03797	173
A00056	A03844	165	A00056	A03848	157
A00056	A03898	267	A00056	A03934	322
A00056	A03942	343	A00056	A03949	490
A00056	A03976	251	A00056	A03989	151
A00056	A04030	210	A00056	A04040	246
A00056	A04078	185	A00056	A04138	232
A00056	A04159	219	A00056	A04201	271
A00056	A04226	246	A00056	A04237	195
A00056	A04238	297	A00056	A04244	335
A00056	A04248	475	A00056	A04257	451
A00056	A04263	313	A00056	A04267	210
A00056	A04276	230	A00056	A04280	451
A00056	A04289	219	A00056	A04334	167
A00056	A04370	177	A00056	A04372	195
A00056	A04398	945	A00056	A04402	195
A00056	A04417	167	A00056	A04439	201
A00056	A04449	212	A00056	A04462	210
A00056	A04468	168	A00056	A04473	352
A00056	A04484	435	A00056	A04488	177
A00056	A04492	224	A00056	A04517	335

A00056	A04536	345	A00056	A04539	188
A00056	A04541	173	A00056	A04567	191
A00056	A04589	393	A00056	A04609	168
A00056	A04614	283	A00056	A04622	432
A00056	A04643	348	A00056	A04690	246
A00056	A04750	201	A00056	A04759	188
A00056	A04762	267	A00056	A04794	246
A00056	A04795	154	A00056	A04842	162
A00056	A04855	219	A00056	A04865	380
A00056	A04878	401	A00056	A04909	335
A00056	A04961	265	A00056	A04985	481
A00056	A04989	183	A00056	A04992	201
A00056	A04996	210	A00056	A05066	177
A00056	A05090	183	A00056	A05155	682
A00056	A05236	246	A00056	A05250	307
A00056	A05283	183	A00056	A05309	229
A00056	A05350	201	A00056	A05431	452
A00056	A05545	154	A00056	A05558	201
A00056	A05607	483	A00056	A05613	173
A00056	A05679	414	A00056	A05709	257
A00056	A05901	195	A00056	A05959	173
A00056	A05960	210	A00056	A05985	383
A00056	A05989	219	A00056	A06095	271
A00056	A06211	286	A00056	A06313	363
A00056	A06414	401	A00056	A06415	484
A00056	A06623	461	A00056	A06650	229
A00056	A06672	297	A00056	A06728	210
A00056	A06807	177	A00056	A06825	265
A00056	A06901	246	A00056	A06955	157
A00056	A07116	168	A00056	A07131	383
A00056	A07134	246	A00056	A07142	168
A00056	A07145	490	A00056	A07152	318
A00056	A07157	435	A00056	A07158	345
A00056	A07161	262	A00056	A07164	383
A00056	A07165	351	A00056	A07166	355
A00056	A07173	491	A00056	A07192	232
A00056	A07205	162	A00056	A07227	384
A00056	A07242	167	A00056	A07331	195
A00056	A07372	165	A00056	A07421	483
A00056	A07495	163	A00056	A07557	177
A00056	A07587	173	A00056	A07665	229
A00056	A08254	267	A00056	A08612	334
A00056	A08704	201	A00056	A08828	188
A00056	A08930	160	A00056	A09011	307
A00056	A09017	183	A00056	A09070	246
A00056	A09112	201	A00056	A09120	195

A00056	A09208	188	A00056	A09263	176
A00056	A09390	246	A00056	A09433	383
A00056	A09449	199	A00056	A09464	204
A00056	A09513	210	A00056	A09523	267
A00056	A09548	389	A00056	A09555	246
A00056	A09570	424	A00056	A09871	908
A00056	A09959	232	A00056	A09967	246
A00056	A09970	246	A00056	A10055	173
A00056	A10074	267	A00056	A10084	210
A00056	A10166	297	A00056	A10273	232
A00056	A10455	173	A00056	A10655	356
A00056	A10731	246	A00056	A10791	491
A00056	A10940	195	A00056	A10977	246
A00056	A10985	232	A00056	A11004	297
A00056	A11023	341	A00056	A11086	154
A00056	A11090	183	A00056	A11125	906
A00056	A11150	255	A00056	A11281	417
A00056	A11400	210	A00056	A11487	199
A00056	A11779	456	A00056	A11937	165
A00056	A12365	356	A00056	A12529	232
A00056	A12913	246	A00056	A12971	196
A00056	A13369	587	A00056	A13617	180
A00056	A13860	311	A00056	A13872	177
A00056	A13902	356	A00056	A14071	422
A00056	A14146	212	A00056	A14190	469
A00056	A14194	294	A00056	A14202	308
A00056	A14236	488	A00056	A14366	491
A00056	A14372	191	A00056	A14374	467
A00056	A14378	408	A00056	A14391	483
A00056	A14399	236	A00056	A14439	160
A00056	A14443	899	A00056	A14511	174
A00056	A14535	177	A00056	A14544	232
A00056	A14554	208	A00056	A14606	232
A00056	A14613	177	A00056	A14642	215
A00056	A14643	226	A00056	A14680	297
A00056	A14784	297	A00056	A14924	297
A00056	A15241	479	A00056	A15278	210
A00056	A15336	293	A00056	A15394	485
A00056	A15395	486	A00056	A15425	423
A00056	A15427	363	A00056	A15506	173
A00056	A15518	366	A00056	A15601	326
A00056	A15603	446	A00056	A15605	396
A00056	A15608	279	A00056	A15630	159
A00056	A15641	368	A00056	A15795	438
A00056	A15806	408	A00056	A15964	446
A00056	A15973	210	A00056	A16047	246

A00056	A16112	383	A00056	A16222	358
A00056	A16300	238	A00056	A16450	224
A00056	A16636	458	A00056	A16738	240
A00057	A00063	488	A00057	A00069	469
A00057	A00077	167	A00057	A00079	168
A00057	A00080	486	A00057	A00081	221
A00057	A00083	291	A00057	A00085	477
A00057	A00106	63	A00057	A00111	235
A00057	A00112	458	A00057	A00125	267
A00057	A00152	491	A00057	A00153	373
A00057	A00167	943	A00057	A00168	157
A00057	A00190	491	A00057	A00196	388
A00057	A00204	488	A00057	A00209	935
A00057	A00210	63	A00057	A00212	200
A00057	A00219	491	A00057	A00220	477
A00057	A00227	195	A00057	A00232	486
A00057	A00233	465	A00057	A00264	945
A00057	A00274	417	A00057	A00292	477
A00057	A00352	329	A00057	A00438	151
A00057	A00473	412	A00057	A00476	491
A00057	A00478	322	A00057	A00509	374
A00057	A00519	223	A00057	A00524	223
A00057	A00526	223	A00057	A00616	491
A00057	A00658	223	A00057	A00659	301
A00057	A00687	231	A00057	A00715	223
A00057	A00736	477	A00057	A00752	474
A00057	A00797	163	A00057	A00799	226
A00057	A00871	530	A00057	A00914	480
A00057	A00986	223	A00057	A01006	154
A00057	A01009	479	A00057	A01111	223
A00057	A01118	223	A00057	A01161	313
A00057	A01202	223	A00057	A01208	223
A00057	A01215	230	A00057	A01228	223
A00057	A01235	160	A00057	A01257	159
A00057	A01277	443	A00057	A01307	281
A00057	A01353	323	A00057	A01524	152
A00057	A01527	944	A00057	A01567	424
A00057	A01604	491	A00057	A01628	213
A00057	A01629	167	A00057	A01654	396
A00057	A01658	281	A00057	A01663	191
A00057	A01668	404	A00057	A01676	352
A00057	A01704	462	A00057	A01736	223
A00057	A01741	435	A00057	A01743	450
A00057	A01775	270	A00057	A01791	157
A00057	A01809	401	A00057	A01867	329
A00057	A01870	491	A00057	A01879	218

A00057	A01899	182	A00057	A01931	63
A00057	A01944	195	A00057	A02030	299
A00057	A02041	223	A00057	A02051	223
A00057	A02074	165	A00057	A02089	200
A00057	A02111	151	A00057	A02143	481
A00057	A02170	228	A00057	A02174	177
A00057	A02186	193	A00057	A02242	174
A00057	A02248	107	A00057	A02250	95
A00057	A02251	477	A00057	A02255	318
A00057	A02260	256	A00057	A02262	152
A00057	A02268	467	A00057	A02275	171
A00057	A02278	204	A00057	A02281	396
A00057	A02298	436	A00057	A02299	200
A00057	A02307	288	A00057	A02309	238
A00057	A02319	246	A00057	A02331	469
A00057	A02333	441	A00057	A02350	156
A00057	A02367	318	A00057	A02380	168
A00057	A02381	159	A00057	A02382	269
A00057	A02415	405	A00057	A02416	463
A00057	A02417	324	A00057	A02418	173
A00057	A02423	201	A00057	A02424	70
A00057	A02427	218	A00057	A02454	457
A00057	A02460	345	A00057	A02465	162
A00057	A02468	293	A00057	A02471	180
A00057	A02488	356	A00057	A02490	485
A00057	A02491	457	A00057	A02493	443
A00057	A02494	462	A00057	A02498	337
A00057	A02505	171	A00057	A02546	195
A00057	A02549	177	A00057	A02575	314
A00057	A02578	152	A00057	A02579	404
A00057	A02582	353	A00057	A02590	333
A00057	A02607	63	A00057	A02620	180
A00057	A02624	488	A00057	A02654	469
A00057	A02667	151	A00057	A02668	457
A00057	A02669	154	A00057	A02709	215
A00057	A02727	263	A00057	A02745	165
A00057	A02754	263	A00057	A02756	384
A00057	A02771	243	A00057	A02772	154
A00057	A02787	485	A00057	A02788	296
A00057	A02820	297	A00057	A02839	213
A00057	A02847	238	A00057	A02851	152
A00057	A02859	455	A00057	A02864	246
A00057	A02865	174	A00057	A02874	204
A00057	A02875	165	A00057	A02885	236
A00057	A02896	486	A00057	A02901	163
A00057	A02916	180	A00057	A02920	291

A00057	A02931	198	A00057	A02944	363
A00057	A02945	389	A00057	A02947	156
A00057	A02953	221	A00057	A02961	297
A00057	A02971	317	A00057	A02972	323
A00057	A02975	174	A00057	A03045	425
A00057	A03049	160	A00057	A03053	132
A00057	A03063	229	A00057	A03081	210
A00057	A03110	367	A00057	A03124	290
A00057	A03127	162	A00057	A03128	246
A00057	A03156	193	A00057	A03157	198
A00057	A03169	223	A00057	A03173	271
A00057	A03185	188	A00057	A03186	345
A00057	A03206	356	A00057	A03224	945
A00057	A03259	208	A00057	A03271	313
A00057	A03275	157	A00057	A03312	317
A00057	A03316	311	A00057	A03540	195
A00057	A03610	221	A00057	A03630	335
A00057	A03639	419	A00057	A03749	154
A00057	A03788	382	A00057	A03803	306
A00057	A03821	202	A00057	A03904	238
A00057	A03913	285	A00057	A03935	204
A00057	A03949	324	A00057	A03977	154
A00057	A03989	177	A00057	A04040	278
A00057	A04051	234	A00057	A04092	205
A00057	A04110	322	A00057	A04159	246
A00057	A04206	160	A00057	A04218	157
A00057	A04221	210	A00057	A04225	215
A00057	A04226	271	A00057	A04237	221
A00057	A04244	269	A00057	A04247	215
A00057	A04257	180	A00057	A04304	180
A00057	A04372	221	A00057	A04398	424
A00057	A04402	221	A00057	A04447	223
A00057	A04450	195	A00057	A04462	236
A00057	A04467	271	A00057	A04468	195
A00057	A04481	204	A00057	A04508	290
A00057	A04566	257	A00057	A04613	180
A00057	A04620	168	A00057	A04741	221
A00057	A04748	215	A00057	A04750	229
A00057	A04756	243	A00057	A04854	257
A00057	A04912	236	A00057	A04916	257
A00057	A04985	308	A00057	A04989	210
A00057	A05079	200	A00057	A05124	185
A00057	A05353	157	A00057	A05463	353
A00057	A05477	188	A00057	A05641	168
A00057	A05709	156	A00057	A05952	246
A00057	A05959	200	A00057	A05965	257

A00057	A05968	200	A00057	A05985	409
A00057	A06095	167	A00057	A06149	341
A00057	A06211	180	A00057	A06370	191
A00057	A06414	446	A00057	A06415	422
A00057	A06558	257	A00057	A06807	204
A00057	A06901	271	A00057	A06911	236
A00057	A06927	318	A00057	A07006	236
A00057	A07087	215	A00057	A07131	396
A00057	A07135	323	A00057	A07138	481
A00057	A07140	394	A00057	A07141	177
A00057	A07145	393	A00057	A07152	204
A00057	A07157	413	A00057	A07164	218
A00057	A07173	457	A00057	A07209	489
A00057	A07265	223	A00057	A07421	243
A00057	A07588	223	A00057	A07655	260
A00057	A07748	191	A00057	A08163	191
A00057	A08612	356	A00057	A08667	271
A00057	A08688	443	A00057	A08929	241
A00057	A08955	223	A00057	A08971	223
A00057	A09013	318	A00057	A09017	210
A00057	A09148	246	A00057	A09163	318
A00057	A09219	172	A00057	A09263	219
A00057	A09388	251	A00057	A09390	447
A00057	A09433	191	A00057	A09890	174
A00057	A09979	271	A00057	A09985	180
A00057	A10018	154	A00057	A10190	271
A00057	A10254	271	A00057	A10470	345
A00057	A10640	267	A00057	A10755	223
A00057	A10791	441	A00057	A10905	160
A00057	A11020	246	A00057	A11070	215
A00057	A11140	299	A00057	A11195	215
A00057	A11202	223	A00057	A11206	160
A00057	A11262	167	A00057	A11318	366
A00057	A11556	163	A00057	A11566	257
A00057	A11667	375	A00057	A12581	271
A00057	A12719	229	A00057	A13298	223
A00057	A13419	257	A00057	A13447	204
A00057	A13726	252	A00057	A13872	213
A00057	A13906	168	A00057	A14190	163
A00057	A14199	201	A00057	A14216	223
A00057	A14366	424	A00057	A14374	489
A00057	A14387	383	A00057	A14391	328
A00057	A14416	167	A00057	A14430	333
A00057	A14439	469	A00057	A14554	333
A00057	A14599	385	A00057	A14603	215
A00057	A14604	322	A00057	A14643	188

A00057	A14650	229	A00057	A14685	229
A00057	A15285	428	A00057	A15290	160
A00057	A15394	480	A00057	A15395	383
A00057	A15425	182	A00057	A15516	294
A00057	A15517	236	A00057	A15518	168
A00057	A15603	339	A00057	A15640	246
A00057	A15642	180	A00057	A15789	210
A00057	A15795	485	A00057	A15800	385
A00057	A15806	154	A00057	A15826	191
A00057	A15832	236	A00057	A15962	165
A00057	A16007	160	A00057	A16008	172
A00057	A16010	318	A00057	A16014	257
A00057	A16037	257	A00057	A16112	191
A00057	A16232	221	A00057	A16323	152
A00057	A16636	460	A00057	A16887	229
A00057	A17155	154	A00058	A00059	945
A00058	A00067	899	A00058	A00073	958
A00058	A00074	455	A00058	A00094	571
A00058	A00118	711	A00058	A00129	341
A00058	A00132	707	A00058	A00138	477
A00058	A00140	65	A00058	A00146	971
A00058	A00147	441	A00058	A00150	199
A00058	A00151	88	A00058	A00152	484
A00058	A00153	899	A00058	A00157	294
A00058	A00159	576	A00058	A00161	63
A00058	A00162	63	A00058	A00163	213
A00058	A00165	870	A00058	A00167	907
A00058	A00171	899	A00058	A00180	404
A00058	A00187	438	A00058	A00190	343
A00058	A00201	297	A00058	A00208	63
A00058	A00209	899	A00058	A00210	193
A00058	A00220	899	A00058	A00224	899
A00058	A00225	576	A00058	A00226	348
A00058	A00230	899	A00058	A00232	179
A00058	A00239	225	A00058	A00240	197
A00058	A00243	560	A00058	A00248	228
A00058	A00250	945	A00058	A00254	485
A00058	A00257	724	A00058	A00260	288
A00058	A00265	491	A00058	A00420	324
A00058	A00691	314	A00058	A00736	205
A00058	A00843	457	A00058	A00883	163
A00058	A01083	472	A00058	A01088	241
A00058	A01173	263	A00058	A01213	290
A00058	A01410	489	A00058	A01473	582
A00058	A01527	899	A00058	A01553	288
A00058	A01554	85	A00058	A01558	899

A00058	A01560	229	A00058	A01572	477
A00058	A01588	899	A00058	A01596	821
A00058	A01615	327	A00058	A01631	138
A00058	A01639	945	A00058	A01709	63
A00058	A01730	477	A00058	A01770	900
A00058	A02183	183	A00058	A02236	208
A00058	A02487	71	A00058	A02564	121
A00058	A02724	324	A00058	A02736	173
A00058	A02737	966	A00058	A02805	462
A00058	A02806	63	A00058	A02825	964
A00058	A02860	190	A00058	A02904	180
A00058	A03213	63	A00058	A03223	469
A00058	A03575	193	A00058	A03802	154
A00058	A04012	532	A00058	A04205	174
A00058	A04248	899	A00058	A04346	190
A00058	A04740	154	A00058	A05029	655
A00058	A05104	367	A00058	A05579	204
A00058	A05620	311	A00058	A05645	241
A00058	A05712	911	A00058	A06004	236
A00058	A06601	232	A00058	A06642	190
A00058	A06914	314	A00058	A06936	255
A00058	A07201	63	A00058	A07207	307
A00058	A07311	488	A00058	A07324	176
A00058	A07422	961	A00058	A07748	168
A00058	A08074	185	A00058	A08571	899
A00058	A08742	408	A00058	A08858	230
A00058	A09121	144	A00058	A09162	374
A00058	A09218	396	A00058	A09383	173
A00058	A09577	63	A00058	A09934	255
A00058	A10014	79	A00058	A10580	941
A00058	A10623	944	A00058	A10801	899
A00058	A11369	63	A00058	A11522	384
A00058	A11601	281	A00058	A11872	173
A00058	A11879	255	A00058	A11894	240
A00058	A12383	469	A00058	A12550	240
A00058	A12680	925	A00058	A12720	255
A00058	A12794	305	A00058	A12882	152
A00058	A12988	685	A00058	A13465	229
A00058	A13511	490	A00058	A13521	196
A00058	A13551	255	A00058	A13986	235
A00058	A14364	236	A00058	A14385	196
A00058	A14443	930	A00058	A14925	213
A00058	A16513	162	A00059	A00061	160
A00059	A00073	945	A00059	A00109	180
A00059	A00123	407	A00059	A00146	452
A00059	A00151	279	A00059	A00158	205

A00059	A00160	281	A00059	A00162	146
A00059	A00167	916	A00059	A00180	272
A00059	A00209	936	A00059	A00220	899
A00059	A00250	345	A00059	A00255	173
A00059	A00265	190	A00059	A00324	240
A00059	A00694	409	A00059	A00696	179
A00059	A00843	319	A00059	A01410	322
A00059	A01475	180	A00059	A01524	455
A00059	A01527	993	A00059	A01615	183
A00059	A01688	200	A00059	A01770	899
A00059	A02737	937	A00059	A02825	929
A00059	A02863	198	A00059	A02883	184
A00059	A02904	159	A00059	A03436	395
A00059	A04248	899	A00059	A04537	307
A00059	A05712	899	A00059	A05854	196
A00059	A06610	343	A00059	A07173	174
A00059	A10072	249	A00059	A10623	179
A00059	A12971	243	A00059	A14211	216
A00059	A14443	899	A00059	A14665	188
A00060	A00095	386	A00060	A00193	235
A00060	A00212	360	A00060	A00215	65
A00060	A00224	490	A00060	A00294	224
A00060	A00301	152	A00060	A00740	187
A00060	A00911	274	A00060	A01156	152
A00060	A01161	386	A00060	A01172	479
A00060	A01396	157	A00060	A01481	152
A00060	A01529	328	A00060	A01530	246
A00060	A01544	296	A00060	A01548	475
A00060	A01569	449	A00060	A01832	329
A00060	A01855	263	A00060	A02075	157
A00060	A02231	245	A00060	A02325	188
A00060	A02353	383	A00060	A02678	414
A00060	A02739	423	A00060	A02803	193
A00060	A03216	329	A00060	A03451	152
A00060	A03477	479	A00060	A03575	218
A00060	A04140	201	A00060	A04182	229
A00060	A04362	249	A00060	A04364	352
A00060	A04435	196	A00060	A04486	310
A00060	A04492	241	A00060	A04519	327
A00060	A04556	259	A00060	A04578	445
A00060	A04850	174	A00060	A04879	454
A00060	A04880	475	A00060	A04888	369
A00060	A05065	318	A00060	A05111	450
A00060	A05240	196	A00060	A05284	195
A00060	A05701	160	A00060	A06086	193
A00060	A06516	335	A00060	A06610	159

A00060	A06634	290	A00060	A06773	266
A00060	A07091	202	A00060	A07660	281
A00060	A07737	252	A00060	A07746	208
A00060	A07834	367	A00060	A07935	318
A00060	A08240	383	A00060	A08693	196
A00060	A09236	259	A00060	A09647	405
A00060	A09738	273	A00060	A09897	364
A00060	A09911	273	A00060	A10540	313
A00060	A10547	176	A00060	A10894	383
A00060	A11335	245	A00060	A11553	273
A00060	A11590	281	A00060	A11683	341
A00060	A11750	232	A00060	A11889	383
A00060	A12520	383	A00060	A13228	449
A00060	A13330	228	A00060	A13331	335
A00060	A13373	324	A00060	A13403	353
A00060	A14369	215	A00060	A15277	187
A00060	A16109	160	A00060	A16216	154
A00061	A00063	945	A00061	A00064	481
A00061	A00067	218	A00061	A00068	319
A00061	A00109	491	A00061	A00110	165
A00061	A00123	491	A00061	A00141	521
A00061	A00152	489	A00061	A00153	451
A00061	A00158	324	A00061	A00159	263
A00061	A00160	324	A00061	A00163	199
A00061	A00167	160	A00061	A00189	249
A00061	A00190	419	A00061	A00220	458
A00061	A00233	467	A00061	A00248	157
A00061	A00255	252	A00061	A00264	384
A00061	A00317	351	A00061	A00407	217
A00061	A00478	255	A00061	A00799	180
A00061	A00820	466	A00061	A00914	456
A00061	A01211	259	A00061	A01524	939
A00061	A01527	942	A00061	A01654	491
A00061	A01704	201	A00061	A01741	405
A00061	A01747	179	A00061	A01879	339
A00061	A01880	489	A00061	A01881	319
A00061	A01887	338	A00061	A01888	580
A00061	A01909	270	A00061	A01936	210
A00061	A02065	170	A00061	A02105	215
A00061	A02191	177	A00061	A02220	305
A00061	A02307	160	A00061	A02337	345
A00061	A02382	462	A00061	A02416	174
A00061	A02497	294	A00061	A02498	332
A00061	A02568	177	A00061	A02569	351
A00061	A02582	151	A00061	A02606	226
A00061	A02615	224	A00061	A02616	230

A00061	A02637	296	A00061	A02639	385
A00061	A02658	299	A00061	A02659	205
A00061	A02661	249	A00061	A02695	452
A00061	A02696	345	A00061	A02698	490
A00061	A02709	173	A00061	A02711	425
A00061	A02730	385	A00061	A02732	177
A00061	A02739	176	A00061	A02741	96
A00061	A02780	241	A00061	A02785	156
A00061	A02788	281	A00061	A02803	71
A00061	A02808	208	A00061	A02812	258
A00061	A02816	323	A00061	A02817	173
A00061	A02826	152	A00061	A02830	173
A00061	A02834	213	A00061	A02854	290
A00061	A02863	491	A00061	A02896	246
A00061	A02917	491	A00061	A02927	311
A00061	A02945	480	A00061	A02962	210
A00061	A03010	152	A00061	A03081	180
A00061	A03083	156	A00061	A03100	273
A00061	A03259	361	A00061	A03275	232
A00061	A03451	185	A00061	A03531	177
A00061	A03612	180	A00061	A03790	406
A00061	A03817	167	A00061	A03878	281
A00061	A03934	294	A00061	A03966	152
A00061	A04011	235	A00061	A04033	449
A00061	A04110	187	A00061	A04238	388
A00061	A04248	486	A00061	A04249	266
A00061	A04263	278	A00061	A04280	171
A00061	A04320	343	A00061	A04364	199
A00061	A04493	170	A00061	A04536	205
A00061	A04567	191	A00061	A04598	272
A00061	A04865	204	A00061	A04878	356
A00061	A04909	224	A00061	A04944	334
A00061	A05113	157	A00061	A05349	182
A00061	A05487	154	A00061	A05860	319
A00061	A05944	363	A00061	A06054	177
A00061	A06088	263	A00061	A06211	266
A00061	A06415	241	A00061	A06422	200
A00061	A06530	276	A00061	A06650	241
A00061	A06654	201	A00061	A06686	193
A00061	A06780	278	A00061	A07146	356
A00061	A07161	168	A00061	A07164	240
A00061	A07165	205	A00061	A07173	490
A00061	A07192	149	A00061	A07310	338
A00061	A07311	199	A00061	A07390	226
A00061	A07685	338	A00061	A08133	199
A00061	A08193	199	A00061	A09570	266

A00061	A09959	327	A00061	A10344	351
A00061	A10626	255	A00061	A10813	198
A00061	A11604	199	A00061	A14236	491
A00061	A14366	281	A00061	A14369	191
A00061	A14387	251	A00061	A14635	249
A00061	A15241	486	A00061	A15370	163
A00061	A15395	458	A00061	A15599	190
A00061	A15601	347	A00061	A15602	266
A00061	A15603	201	A00061	A15604	380
A00061	A15606	441	A00061	A15806	241
A00061	A15839	228	A00061	A15885	338
A00061	A15981	475	A00061	A16527	286
A00061	A16636	177	A00062	A00063	428
A00062	A00064	330	A00062	A00067	380
A00062	A00074	458	A00062	A00086	156
A00062	A00108	138	A00062	A00138	490
A00062	A00141	491	A00062	A00145	162
A00062	A00152	491	A00062	A00159	491
A00062	A00190	299	A00062	A00220	251
A00062	A00232	491	A00062	A00233	424
A00062	A00234	173	A00062	A00246	306
A00062	A00254	485	A00062	A00914	463
A00062	A01473	162	A00062	A01553	63
A00062	A01572	294	A00062	A01604	267
A00062	A01654	279	A00062	A01704	198
A00062	A01775	63	A00062	A01790	63
A00062	A01844	63	A00062	A01849	218
A00062	A01879	335	A00062	A01887	486
A00062	A01888	486	A00062	A01908	267
A00062	A02340	162	A00062	A02372	191
A00062	A02382	154	A00062	A02464	163
A00062	A02630	196	A00062	A02639	238
A00062	A02659	334	A00062	A02717	119
A00062	A02730	165	A00062	A02731	486
A00062	A02787	95	A00062	A02800	235
A00062	A02808	205	A00062	A02828	63
A00062	A02837	63	A00062	A02843	260
A00062	A02853	310	A00062	A02864	491
A00062	A02884	260	A00062	A02896	180
A00062	A02908	306	A00062	A02932	310
A00062	A02977	179	A00062	A02986	228
A00062	A03010	420	A00062	A03019	63
A00062	A03195	174	A00062	A03204	326
A00062	A03259	216	A00062	A03470	218
A00062	A03610	185	A00062	A03816	369
A00062	A03817	162	A00062	A03821	491

A00062	A04018	246	A00062	A04040	230
A00062	A04110	184	A00062	A04138	221
A00062	A04207	458	A00062	A04248	291
A00062	A04320	210	A00062	A04518	252
A00062	A04519	167	A00062	A04968	279
A00062	A05888	266	A00062	A06149	201
A00062	A06415	321	A00062	A06422	329
A00062	A07138	232	A00062	A07148	230
A00062	A07157	458	A00062	A07161	165
A00062	A07166	256	A00062	A07186	247
A00062	A10605	228	A00062	A10639	291
A00062	A11369	245	A00062	A13872	180
A00062	A14452	279	A00062	A15394	279
A00062	A15726	154	A00062	A15806	238
A00063	A00064	491	A00063	A00073	390
A00063	A00074	152	A00063	A00081	193
A00063	A00086	491	A00063	A00089	383
A00063	A00094	357	A00063	A00123	434
A00063	A00138	462	A00063	A00141	310
A00063	A00145	63	A00063	A00152	582
A00063	A00154	204	A00063	A00159	489
A00063	A00167	451	A00063	A00176	490
A00063	A00182	168	A00063	A00190	491
A00063	A00204	491	A00063	A00212	265
A00063	A00218	399	A00063	A00219	491
A00063	A00224	489	A00063	A00227	191
A00063	A00231	417	A00063	A00232	486
A00063	A00233	582	A00063	A00234	488
A00063	A00238	180	A00063	A00248	302
A00063	A00254	454	A00063	A00264	491
A00063	A00274	424	A00063	A00322	378
A00063	A00324	281	A00063	A00338	433
A00063	A00438	188	A00063	A00457	207
A00063	A00472	435	A00063	A00478	216
A00063	A00496	407	A00063	A00556	473
A00063	A00557	63	A00063	A00558	224
A00063	A00560	202	A00063	A00658	187
A00063	A00724	151	A00063	A00736	490
A00063	A00799	230	A00063	A00914	491
A00063	A01052	384	A00063	A01150	224
A00063	A01161	176	A00063	A01178	195
A00063	A01234	408	A00063	A01287	271
A00063	A01307	357	A00063	A01353	188
A00063	A01396	228	A00063	A01452	357
A00063	A01473	68	A00063	A01474	329
A00063	A01524	904	A00063	A01527	936

A00063	A01551	347	A00063	A01553	899
A00063	A01572	223	A00063	A01604	483
A00063	A01640	400	A00063	A01654	340
A00063	A01668	491	A00063	A01676	364
A00063	A01704	491	A00063	A01705	243
A00063	A01733	230	A00063	A01741	491
A00063	A01743	491	A00063	A01749	63
A00063	A01759	177	A00063	A01767	152
A00063	A01775	491	A00063	A01785	311
A00063	A01789	405	A00063	A01790	63
A00063	A01796	438	A00063	A01797	285
A00063	A01798	200	A00063	A01836	174
A00063	A01844	188	A00063	A01849	84
A00063	A01879	491	A00063	A01880	180
A00063	A01882	167	A00063	A01887	491
A00063	A01888	486	A00063	A01900	160
A00063	A01902	172	A00063	A01908	395
A00063	A01945	63	A00063	A02183	283
A00063	A02221	363	A00063	A02298	202
A00063	A02300	177	A00063	A02340	200
A00063	A02349	71	A00063	A02372	330
A00063	A02382	249	A00063	A02416	333
A00063	A02490	477	A00063	A02491	402
A00063	A02569	234	A00063	A02582	454
A00063	A02586	160	A00063	A02605	252
A00063	A02611	322	A00063	A02637	469
A00063	A02649	212	A00063	A02659	263
A00063	A02686	65	A00063	A02695	460
A00063	A02699	243	A00063	A02709	217
A00063	A02714	215	A00063	A02717	63
A00063	A02726	269	A00063	A02730	357
A00063	A02731	63	A00063	A02735	63
A00063	A02741	407	A00063	A02742	193
A00063	A02745	490	A00063	A02746	375
A00063	A02756	378	A00063	A02780	210
A00063	A02785	299	A00063	A02787	480
A00063	A02788	479	A00063	A02800	380
A00063	A02803	443	A00063	A02808	451
A00063	A02811	162	A00063	A02812	407
A00063	A02814	408	A00063	A02816	302
A00063	A02826	177	A00063	A02837	479
A00063	A02847	314	A00063	A02851	207
A00063	A02854	343	A00063	A02859	491
A00063	A02864	439	A00063	A02874	486
A00063	A02883	151	A00063	A02886	411
A00063	A02888	246	A00063	A02896	491

A00063	A02897	77	A00063	A02916	491
A00063	A02917	269	A00063	A02922	374
A00063	A02935	160	A00063	A02944	491
A00063	A02951	439	A00063	A02953	238
A00063	A02961	488	A00063	A02971	249
A00063	A02972	441	A00063	A02976	91
A00063	A02988	246	A00063	A03001	63
A00063	A03010	96	A00063	A03110	246
A00063	A03112	367	A00063	A03139	165
A00063	A03157	353	A00063	A03183	182
A00063	A03204	488	A00063	A03206	167
A00063	A03223	446	A00063	A03256	373
A00063	A03259	582	A00063	A03271	475
A00063	A03274	167	A00063	A03275	491
A00063	A03279	372	A00063	A03280	393
A00063	A03282	443	A00063	A03739	408
A00063	A03817	218	A00063	A03821	363
A00063	A03851	447	A00063	A03910	302
A00063	A03942	283	A00063	A03996	269
A00063	A03999	234	A00063	A04010	456
A00063	A04027	152	A00063	A04030	174
A00063	A04047	297	A00063	A04088	296
A00063	A04111	152	A00063	A04169	389
A00063	A04206	427	A00063	A04247	152
A00063	A04248	195	A00063	A04265	196
A00063	A04273	167	A00063	A04298	196
A00063	A04304	245	A00063	A04528	213
A00063	A04536	276	A00063	A04538	308
A00063	A04542	167	A00063	A04566	196
A00063	A04567	385	A00063	A04568	165
A00063	A04589	489	A00063	A04613	245
A00063	A04622	380	A00063	A04657	196
A00063	A04734	241	A00063	A04741	160
A00063	A04798	152	A00063	A04826	196
A00063	A04865	343	A00063	A04866	262
A00063	A04879	199	A00063	A04951	213
A00063	A04955	185	A00063	A05150	162
A00063	A05163	167	A00063	A05284	207
A00063	A05309	247	A00063	A05349	430
A00063	A05860	185	A00063	A05929	269
A00063	A06001	229	A00063	A06127	213
A00063	A06149	263	A00063	A06286	176
A00063	A06295	356	A00063	A06422	481
A00063	A06486	152	A00063	A06610	486
A00063	A06642	406	A00063	A06643	185
A00063	A06770	167	A00063	A06854	167

A00063	A06907	219	A00063	A07045	213
A00063	A07135	379	A00063	A07146	193
A00063	A07154	174	A00063	A07157	491
A00063	A07161	358	A00063	A07164	491
A00063	A07165	445	A00063	A07166	424
A00063	A07200	160	A00063	A07209	432
A00063	A07218	177	A00063	A07236	201
A00063	A07651	234	A00063	A08301	391
A00063	A08368	196	A00063	A08458	160
A00063	A08594	63	A00063	A08657	234
A00063	A08786	185	A00063	A08805	152
A00063	A08828	152	A00063	A08829	234
A00063	A08834	456	A00063	A09038	174
A00063	A09380	205	A00063	A09732	201
A00063	A09799	185	A00063	A09835	213
A00063	A09847	215	A00063	A09848	441
A00063	A09863	152	A00063	A10009	107
A00063	A10069	174	A00063	A10078	291
A00063	A10101	234	A00063	A10278	157
A00063	A10401	291	A00063	A10823	167
A00063	A11191	271	A00063	A11291	447
A00063	A11412	152	A00063	A11461	152
A00063	A11466	174	A00063	A11487	208
A00063	A11537	301	A00063	A11567	252
A00063	A11658	221	A00063	A11848	154
A00063	A12003	196	A00063	A12330	174
A00063	A12342	196	A00063	A12560	234
A00063	A13269	269	A00063	A13816	63
A00063	A13824	170	A00063	A13826	462
A00063	A13827	124	A00063	A13829	352
A00063	A13851	152	A00063	A13860	162
A00063	A13909	234	A00063	A13975	519
A00063	A13987	184	A00063	A14072	165
A00063	A14198	469	A00063	A14208	474
A00063	A14210	343	A00063	A14220	171
A00063	A14236	271	A00063	A14290	218
A00063	A14364	322	A00063	A14371	154
A00063	A14387	224	A00063	A14394	152
A00063	A14511	324	A00063	A14515	234
A00063	A14632	213	A00063	A14635	413
A00063	A14643	200	A00063	A14667	418
A00063	A14689	461	A00063	A14852	269
A00063	A15089	234	A00063	A15292	483
A00063	A15310	269	A00063	A15312	317
A00063	A15313	408	A00063	A15317	424
A00063	A15318	345	A00063	A15331	411

A00063	A15333	210	A00063	A15336	172
A00063	A15348	480	A00063	A15349	443
A00063	A15352	486	A00063	A15366	401
A00063	A15394	176	A00063	A15413	188
A00063	A15447	353	A00063	A15451	193
A00063	A15454	360	A00063	A15460	180
A00063	A15484	255	A00063	A15488	281
A00063	A15489	152	A00063	A15515	430
A00063	A15523	196	A00063	A15614	234
A00063	A15639	379	A00063	A15640	185
A00063	A15795	165	A00063	A15799	268
A00063	A15800	374	A00063	A15806	210
A00063	A15815	152	A00063	A15817	167
A00063	A15887	299	A00063	A15916	299
A00063	A16000	269	A00063	A16224	371
A00063	A16300	389	A00063	A16549	185
A00063	A16636	488	A00063	A16687	285
A00063	A16717	234	A00063	A17086	167
A00063	A17175	234	A00063	A17230	152
A00064	A00065	165	A00064	A00067	491
A00064	A00071	457	A00064	A00074	479
A00064	A00081	352	A00064	A00085	269
A00064	A00090	491	A00064	A00091	170
A00064	A00094	486	A00064	A00114	63
A00064	A00118	144	A00064	A00122	93
A00064	A00123	63	A00064	A00124	63
A00064	A00126	385	A00064	A00136	215
A00064	A00138	613	A00064	A00141	291
A00064	A00145	63	A00064	A00152	542
A00064	A00153	286	A00064	A00156	526
A00064	A00158	63	A00064	A00159	951
A00064	A00161	63	A00064	A00162	262
A00064	A00173	489	A00064	A00185	419
A00064	A00186	63	A00064	A00187	432
A00064	A00188	441	A00064	A00189	63
A00064	A00190	463	A00064	A00204	180
A00064	A00205	63	A00064	A00207	360
A00064	A00208	63	A00064	A00214	952
A00064	A00215	358	A00064	A00219	199
A00064	A00221	618	A00064	A00224	899
A00064	A00225	195	A00064	A00233	490
A00064	A00234	965	A00064	A00236	205
A00064	A00246	902	A00064	A00248	398
A00064	A00252	168	A00064	A00253	224
A00064	A00254	491	A00064	A00265	469
A00064	A00270	224	A00064	A00295	245

A00064	A00314	416	A00064	A00419	260
A00064	A00421	396	A00064	A00553	296
A00064	A00562	207	A00064	A00574	420
A00064	A00616	469	A00064	A00641	491
A00064	A00652	426	A00064	A00654	322
A00064	A00660	196	A00064	A00697	469
A00064	A00752	157	A00064	A00757	185
A00064	A00852	491	A00064	A00864	553
A00064	A00868	190	A00064	A00905	252
A00064	A00914	419	A00064	A00939	945
A00064	A00989	297	A00064	A00993	63
A00064	A01041	414	A00064	A01097	427
A00064	A01267	426	A00064	A01286	63
A00064	A01291	899	A00064	A01307	472
A00064	A01309	344	A00064	A01310	63
A00064	A01332	201	A00064	A01423	208
A00064	A01451	362	A00064	A01452	489
A00064	A01492	899	A00064	A01527	899
A00064	A01537	388	A00064	A01542	168
A00064	A01553	355	A00064	A01558	899
A00064	A01560	419	A00064	A01562	917
A00064	A01566	246	A00064	A01572	477
A00064	A01588	899	A00064	A01593	899
A00064	A01603	491	A00064	A01612	477
A00064	A01628	283	A00064	A01631	63
A00064	A01639	344	A00064	A01640	363
A00064	A01654	400	A00064	A01660	543
A00064	A01676	399	A00064	A01692	936
A00064	A01697	63	A00064	A01704	63
A00064	A01709	899	A00064	A01719	238
A00064	A01785	104	A00064	A01792	396
A00064	A01793	63	A00064	A01797	63
A00064	A01850	63	A00064	A01870	152
A00064	A01879	219	A00064	A01880	63
A00064	A01887	87	A00064	A01888	99
A00064	A01900	441	A00064	A01905	353
A00064	A01908	491	A00064	A01909	198
A00064	A01943	154	A00064	A01983	484
A00064	A02183	435	A00064	A02228	469
A00064	A02254	297	A00064	A02305	156
A00064	A02340	63	A00064	A02350	90
A00064	A02353	420	A00064	A02369	221
A00064	A02370	285	A00064	A02419	63
A00064	A02425	441	A00064	A02608	188
A00064	A02610	191	A00064	A02630	218
A00064	A02634	63	A00064	A02635	63

A00064	A02637	486	A00064	A02645	174
A00064	A02656	144	A00064	A02659	229
A00064	A02662	191	A00064	A02681	198
A00064	A02695	190	A00064	A02718	276
A00064	A02730	252	A00064	A02731	63
A00064	A02734	130	A00064	A02739	329
A00064	A02740	343	A00064	A02741	63
A00064	A02751	63	A00064	A02769	286
A00064	A02787	63	A00064	A02788	71
A00064	A02800	436	A00064	A02803	994
A00064	A02808	321	A00064	A02812	180
A00064	A02837	63	A00064	A02860	279
A00064	A02863	139	A00064	A02864	204
A00064	A02887	481	A00064	A02896	63
A00064	A02914	152	A00064	A02917	229
A00064	A02931	317	A00064	A02940	191
A00064	A02945	188	A00064	A02950	341
A00064	A02975	63	A00064	A03010	76
A00064	A03019	466	A00064	A03110	351
A00064	A03116	213	A00064	A03117	174
A00064	A03171	443	A00064	A03174	217
A00064	A03195	63	A00064	A03224	265
A00064	A03263	63	A00064	A03270	205
A00064	A03271	945	A00064	A03327	372
A00064	A03335	459	A00064	A03477	467
A00064	A03496	680	A00064	A03569	596
A00064	A03596	66	A00064	A03625	82
A00064	A03784	232	A00064	A03809	486
A00064	A03817	491	A00064	A03821	468
A00064	A03942	236	A00064	A03962	274
A00064	A04042	162	A00064	A04049	198
A00064	A04050	485	A00064	A04105	227
A00064	A04138	157	A00064	A04161	452
A00064	A04205	329	A00064	A04216	188
A00064	A04245	154	A00064	A04248	159
A00064	A04249	217	A00064	A04323	405
A00064	A04519	328	A00064	A04536	404
A00064	A04540	278	A00064	A04551	154
A00064	A04587	296	A00064	A04589	306
A00064	A04634	191	A00064	A04673	191
A00064	A04682	408	A00064	A04699	899
A00064	A04730	296	A00064	A04755	379
A00064	A04880	479	A00064	A05008	234
A00064	A05020	420	A00064	A05037	899
A00064	A05224	246	A00064	A05270	191
A00064	A05288	226	A00064	A05470	191

A00064	A05490	174	A00064	A05607	322
A00064	A05617	414	A00064	A05712	174
A00064	A05760	899	A00064	A05773	432
A00064	A05944	199	A00064	A05985	413
A00064	A06054	269	A00064	A06055	416
A00064	A06095	198	A00064	A06108	249
A00064	A06224	362	A00064	A06289	77
A00064	A06359	420	A00064	A06449	308
A00064	A06676	154	A00064	A06701	226
A00064	A06737	213	A00064	A06758	346
A00064	A06854	276	A00064	A06899	188
A00064	A06900	249	A00064	A06907	539
A00064	A06955	167	A00064	A07131	213
A00064	A07135	445	A00064	A07143	152
A00064	A07146	74	A00064	A07147	405
A00064	A07157	274	A00064	A07158	157
A00064	A07161	469	A00064	A07164	384
A00064	A07166	319	A00064	A07200	386
A00064	A07201	173	A00064	A07218	455
A00064	A07311	483	A00064	A07390	160
A00064	A07618	899	A00064	A07798	491
A00064	A07885	257	A00064	A08137	401
A00064	A08206	154	A00064	A08280	279
A00064	A08535	491	A00064	A08608	221
A00064	A08616	249	A00064	A09220	96
A00064	A09263	344	A00064	A09401	63
A00064	A09625	395	A00064	A09842	247
A00064	A09937	138	A00064	A10014	63
A00064	A10159	899	A00064	A10174	899
A00064	A10208	491	A00064	A10365	63
A00064	A10510	235	A00064	A10528	262
A00064	A10735	361	A00064	A10744	319
A00064	A10801	110	A00064	A10854	420
A00064	A11089	191	A00064	A11125	899
A00064	A11369	967	A00064	A11591	165
A00064	A12009	65	A00064	A12287	174
A00064	A12383	254	A00064	A12664	899
A00064	A12835	277	A00064	A13146	899
A00064	A13561	174	A00064	A13587	167
A00064	A13592	217	A00064	A13743	176
A00064	A13744	249	A00064	A13824	229
A00064	A13849	154	A00064	A13980	63
A00064	A13983	244	A00064	A13985	899
A00064	A13986	340	A00064	A13989	899
A00064	A14164	213	A00064	A14190	335
A00064	A14192	557	A00064	A14193	544

A00064	A14196	196	A00064	A14198	491
A00064	A14208	945	A00064	A14210	274
A00064	A14263	572	A00064	A14299	185
A00064	A14303	430	A00064	A14364	945
A00064	A14366	419	A00064	A14395	303
A00064	A14440	301	A00064	A14452	63
A00064	A14516	283	A00064	A14647	226
A00064	A14662	899	A00064	A15101	191
A00064	A15297	918	A00064	A15314	406
A00064	A15323	899	A00064	A15324	899
A00064	A15353	899	A00064	A15359	899
A00064	A15388	193	A00064	A15506	152
A00064	A15559	195	A00064	A15578	332
A00064	A15620	183	A00064	A15712	224
A00064	A15726	418	A00064	A15839	162
A00064	A15865	368	A00064	A15904	268
A00064	A15935	472	A00064	A15968	162
A00064	A15999	174	A00064	A16009	249
A00064	A16083	191	A00064	A16188	174
A00064	A16216	291	A00064	A16308	374
A00064	A16310	174	A00064	A16716	191
A00064	A16853	154	A00065	A00067	899
A00065	A00100	491	A00065	A00113	313
A00065	A00119	485	A00065	A00130	922
A00065	A00138	452	A00065	A00147	323
A00065	A00156	185	A00065	A00157	899
A00065	A00167	355	A00065	A00169	305
A00065	A00180	219	A00065	A00184	903
A00065	A00190	351	A00065	A00195	404
A00065	A00223	491	A00065	A00224	914
A00065	A00236	968	A00065	A00239	369
A00065	A00240	939	A00065	A00252	945
A00065	A00253	491	A00065	A00265	447
A00065	A00268	279	A00065	A00293	390
A00065	A00324	901	A00065	A00331	210
A00065	A00335	204	A00065	A00358	157
A00065	A00430	299	A00065	A00630	364
A00065	A00652	491	A00065	A00747	481
A00065	A00848	63	A00065	A00887	286
A00065	A00902	163	A00065	A01098	340
A00065	A01140	302	A00065	A01178	488
A00065	A01289	407	A00065	A01291	433
A00065	A01425	491	A00065	A01524	899
A00065	A01527	899	A00065	A01550	489
A00065	A01554	899	A00065	A01558	899
A00065	A01562	945	A00065	A01588	899

A00065	A01606	193	A00065	A01692	964
A00065	A01730	451	A00065	A01740	477
A00065	A01741	268	A00065	A01755	90
A00065	A01796	436	A00065	A01877	446
A00065	A01886	134	A00065	A02325	491
A00065	A02418	446	A00065	A02421	258
A00065	A02434	291	A00065	A02494	485
A00065	A02502	906	A00065	A02647	243
A00065	A02703	399	A00065	A02786	226
A00065	A02816	456	A00065	A02827	227
A00065	A02860	456	A00065	A02877	308
A00065	A02895	389	A00065	A02912	162
A00065	A02965	195	A00065	A03057	374
A00065	A03112	303	A00065	A03286	277
A00065	A03451	371	A00065	A03472	196
A00065	A03575	899	A00065	A03591	170
A00065	A03739	343	A00065	A04020	236
A00065	A04312	307	A00065	A04342	491
A00065	A04964	243	A00065	A05077	334
A00065	A05418	218	A00065	A05621	451
A00065	A05811	297	A00065	A05958	232
A00065	A06138	174	A00065	A06324	190
A00065	A07163	240	A00065	A07311	899
A00065	A07399	190	A00065	A07422	914
A00065	A07791	227	A00065	A08858	899
A00065	A08899	945	A00065	A09092	177
A00065	A09121	185	A00065	A09123	154
A00065	A09423	407	A00065	A09562	162
A00065	A09923	307	A00065	A10233	278
A00065	A10247	238	A00065	A10311	267
A00065	A10346	227	A00065	A10580	899
A00065	A10588	173	A00065	A11921	469
A00065	A12996	290	A00065	A13255	451
A00065	A13795	249	A00065	A13977	899
A00065	A14035	249	A00065	A14164	408
A00065	A15483	258	A00065	A16718	171
A00066	A00067	914	A00066	A00074	188
A00066	A00099	899	A00066	A00126	398
A00066	A00159	926	A00066	A00163	165
A00066	A00200	899	A00066	A00206	677
A00066	A00207	399	A00066	A00221	443
A00066	A00224	945	A00066	A00230	944
A00066	A00236	932	A00066	A00240	326
A00066	A00252	311	A00066	A00254	945
A00066	A00269	486	A00066	A00271	899
A00066	A00341	224	A00066	A00400	436

A00066	A00697	265	A00066	A01524	899
A00066	A01533	335	A00066	A01558	934
A00066	A01560	945	A00066	A01561	943
A00066	A01568	414	A00066	A01588	941
A00066	A01591	386	A00066	A01592	945
A00066	A01602	899	A00066	A01615	899
A00066	A01638	455	A00066	A01639	899
A00066	A01651	993	A00066	A01692	960
A00066	A01697	207	A00066	A02564	191
A00066	A03591	961	A00066	A04195	246
A00066	A04305	218	A00066	A04699	899
A00066	A05082	190	A00066	A05453	374
A00066	A06148	190	A00066	A06608	899
A00066	A07311	945	A00066	A07762	491
A00066	A07876	224	A00066	A08608	422
A00066	A08862	283	A00066	A09568	971
A00066	A10365	899	A00066	A10833	971
A00066	A11478	224	A00066	A11820	302
A00066	A11983	899	A00066	A12383	899
A00066	A12987	291	A00066	A13390	899
A00066	A13391	912	A00066	A13511	283
A00066	A13917	477	A00066	A13984	944
A00066	A13990	899	A00066	A14039	213
A00066	A14188	162	A00066	A14472	906
A00066	A14548	159	A00066	A14577	470
A00066	A16741	488	A00067	A00068	955
A00067	A00069	899	A00067	A00074	973
A00067	A00075	899	A00067	A00079	899
A00067	A00081	899	A00067	A00085	165
A00067	A00087	899	A00067	A00089	899
A00067	A00091	921	A00067	A00094	945
A00067	A00099	899	A00067	A00100	899
A00067	A00113	899	A00067	A00116	899
A00067	A00117	899	A00067	A00118	150
A00067	A00120	899	A00067	A00121	899
A00067	A00122	490	A00067	A00124	939
A00067	A00128	899	A00067	A00130	899
A00067	A00133	899	A00067	A00134	899
A00067	A00135	931	A00067	A00138	528
A00067	A00140	899	A00067	A00141	923
A00067	A00149	899	A00067	A00150	903
A00067	A00151	942	A00067	A00152	959
A00067	A00153	942	A00067	A00156	918
A00067	A00157	899	A00067	A00158	308
A00067	A00159	944	A00067	A00160	196
A00067	A00161	955	A00067	A00162	954

A00067	A00163	945	A00067	A00166	899
A00067	A00167	903	A00067	A00169	899
A00067	A00170	899	A00067	A00172	899
A00067	A00173	899	A00067	A00174	899
A00067	A00175	904	A00067	A00180	159
A00067	A00182	899	A00067	A00184	899
A00067	A00185	945	A00067	A00186	925
A00067	A00189	899	A00067	A00190	945
A00067	A00192	899	A00067	A00196	404
A00067	A00200	931	A00067	A00201	491
A00067	A00203	899	A00067	A00205	908
A00067	A00206	899	A00067	A00207	997
A00067	A00208	951	A00067	A00209	945
A00067	A00210	899	A00067	A00211	899
A00067	A00214	929	A00067	A00218	389
A00067	A00219	942	A00067	A00220	899
A00067	A00221	899	A00067	A00223	899
A00067	A00224	932	A00067	A00226	899
A00067	A00227	899	A00067	A00229	899
A00067	A00230	899	A00067	A00233	266
A00067	A00234	950	A00067	A00236	899
A00067	A00240	906	A00067	A00244	899
A00067	A00245	899	A00067	A00246	940
A00067	A00247	899	A00067	A00248	942
A00067	A00250	899	A00067	A00252	899
A00067	A00253	899	A00067	A00254	956
A00067	A00255	923	A00067	A00256	491
A00067	A00257	899	A00067	A00258	899
A00067	A00261	588	A00067	A00264	490
A00067	A00265	934	A00067	A00266	899
A00067	A00268	899	A00067	A00270	899
A00067	A00271	899	A00067	A00272	899
A00067	A00275	899	A00067	A00312	899
A00067	A00324	899	A00067	A00335	899
A00067	A00378	952	A00067	A00449	191
A00067	A00451	899	A00067	A00616	924
A00067	A00665	318	A00067	A00679	899
A00067	A00696	490	A00067	A00700	899
A00067	A00704	232	A00067	A00707	438
A00067	A00724	489	A00067	A00743	322
A00067	A00744	491	A00067	A00747	899
A00067	A00769	598	A00067	A00841	578
A00067	A00849	199	A00067	A00871	168
A00067	A00887	899	A00067	A00909	631
A00067	A00914	934	A00067	A00922	224
A00067	A00983	358	A00067	A01002	899

A00067	A01009	480	A00067	A01022	202
A00067	A01024	216	A00067	A01027	156
A00067	A01052	899	A00067	A01070	899
A00067	A01148	899	A00067	A01150	470
A00067	A01173	899	A00067	A01232	629
A00067	A01244	899	A00067	A01291	903
A00067	A01293	491	A00067	A01311	514
A00067	A01325	234	A00067	A01404	899
A00067	A01415	899	A00067	A01425	899
A00067	A01439	435	A00067	A01452	447
A00067	A01477	899	A00067	A01512	899
A00067	A01524	950	A00067	A01527	904
A00067	A01542	212	A00067	A01550	899
A00067	A01553	906	A00067	A01554	902
A00067	A01558	992	A00067	A01560	945
A00067	A01561	899	A00067	A01562	899
A00067	A01571	467	A00067	A01572	578
A00067	A01588	899	A00067	A01592	899
A00067	A01593	899	A00067	A01601	899
A00067	A01602	899	A00067	A01604	899
A00067	A01605	899	A00067	A01612	904
A00067	A01613	288	A00067	A01615	899
A00067	A01617	899	A00067	A01628	899
A00067	A01636	899	A00067	A01637	899
A00067	A01638	899	A00067	A01639	899
A00067	A01640	899	A00067	A01651	899
A00067	A01665	899	A00067	A01680	899
A00067	A01688	899	A00067	A01692	899
A00067	A01698	899	A00067	A01702	899
A00067	A01708	899	A00067	A01709	899
A00067	A01718	899	A00067	A01719	899
A00067	A01725	168	A00067	A01726	945
A00067	A01729	167	A00067	A01730	462
A00067	A01733	490	A00067	A01737	414
A00067	A01743	202	A00067	A01744	356
A00067	A01746	419	A00067	A01755	914
A00067	A01765	329	A00067	A01769	490
A00067	A01778	195	A00067	A01779	302
A00067	A01812	215	A00067	A01844	899
A00067	A01850	899	A00067	A01870	230
A00067	A01875	358	A00067	A01876	484
A00067	A01879	904	A00067	A01880	899
A00067	A01888	357	A00067	A01894	337
A00067	A01908	260	A00067	A01920	899
A00067	A01944	212	A00067	A02104	899
A00067	A02105	899	A00067	A02126	899

A00067	A02183	173	A00067	A02284	899
A00067	A02337	899	A00067	A02382	372
A00067	A02401	162	A00067	A02416	899
A00067	A02429	899	A00067	A02433	899
A00067	A02464	899	A00067	A02481	909
A00067	A02490	363	A00067	A02502	899
A00067	A02508	163	A00067	A02544	899
A00067	A02637	199	A00067	A02641	191
A00067	A02695	288	A00067	A02711	63
A00067	A02714	400	A00067	A02723	899
A00067	A02748	187	A00067	A02754	468
A00067	A02756	196	A00067	A02779	899
A00067	A02783	168	A00067	A02803	187
A00067	A02825	899	A00067	A02834	168
A00067	A02849	162	A00067	A02859	485
A00067	A02875	167	A00067	A02896	199
A00067	A02982	484	A00067	A03010	899
A00067	A03014	472	A00067	A03019	899
A00067	A03050	575	A00067	A03054	899
A00067	A03056	179	A00067	A03099	899
A00067	A03110	904	A00067	A03121	400
A00067	A03126	899	A00067	A03204	899
A00067	A03259	199	A00067	A03275	380
A00067	A03417	486	A00067	A03451	899
A00067	A03478	374	A00067	A03498	899
A00067	A03544	212	A00067	A03575	899
A00067	A03591	900	A00067	A03633	899
A00067	A03639	899	A00067	A03662	945
A00067	A03799	899	A00067	A03830	899
A00067	A03843	358	A00067	A03854	899
A00067	A03861	899	A00067	A03913	899
A00067	A03942	912	A00067	A04012	899
A00067	A04051	191	A00067	A04062	899
A00067	A04130	230	A00067	A04147	899
A00067	A04248	899	A00067	A04286	195
A00067	A04287	162	A00067	A04316	202
A00067	A04320	899	A00067	A04346	446
A00067	A04398	927	A00067	A04422	899
A00067	A04444	396	A00067	A04536	277
A00067	A04751	899	A00067	A04783	157
A00067	A04807	899	A00067	A04879	173
A00067	A05082	899	A00067	A05141	216
A00067	A05208	899	A00067	A05216	899
A00067	A05288	477	A00067	A05326	899
A00067	A05418	229	A00067	A05533	899
A00067	A05742	441	A00067	A05854	899

A00067	A05944	899	A00067	A05981	477
A00067	A05985	491	A00067	A06039	199
A00067	A06415	179	A00067	A06591	899
A00067	A06597	451	A00067	A06608	899
A00067	A06609	899	A00067	A06650	491
A00067	A06762	899	A00067	A06795	168
A00067	A06926	163	A00067	A07091	899
A00067	A07157	899	A00067	A07165	899
A00067	A07166	209	A00067	A07311	914
A00067	A07370	418	A00067	A07422	899
A00067	A07460	899	A00067	A07744	469
A00067	A07911	489	A00067	A08133	199
A00067	A08193	207	A00067	A08228	452
A00067	A08278	899	A00067	A08548	368
A00067	A08571	899	A00067	A08589	162
A00067	A08714	899	A00067	A08858	899
A00067	A08869	302	A00067	A08899	899
A00067	A08904	199	A00067	A09181	899
A00067	A09201	899	A00067	A09220	945
A00067	A09319	899	A00067	A09363	899
A00067	A09383	899	A00067	A09542	899
A00067	A09577	899	A00067	A09660	899
A00067	A09842	277	A00067	A09919	162
A00067	A10028	162	A00067	A10185	899
A00067	A10392	899	A00067	A10512	162
A00067	A10542	899	A00067	A10560	899
A00067	A10580	943	A00067	A10601	899
A00067	A10623	899	A00067	A10801	912
A00067	A10919	162	A00067	A10941	243
A00067	A11125	899	A00067	A11326	488
A00067	A11369	933	A00067	A11522	899
A00067	A11604	914	A00067	A11834	899
A00067	A11931	333	A00067	A11983	899
A00067	A12383	899	A00067	A12680	899
A00067	A12932	899	A00067	A12988	899
A00067	A13003	899	A00067	A13250	899
A00067	A13307	416	A00067	A13391	899
A00067	A13401	899	A00067	A13605	899
A00067	A13667	185	A00067	A13734	223
A00067	A13788	185	A00067	A13958	899
A00067	A13977	899	A00067	A13978	899
A00067	A13980	955	A00067	A13981	899
A00067	A13983	899	A00067	A13985	899
A00067	A13987	899	A00067	A13989	899
A00067	A13990	925	A00067	A13991	899
A00067	A13992	899	A00067	A13995	899

A00067	A13999	899	A00067	A14001	899
A00067	A14010	899	A00067	A14027	901
A00067	A14030	899	A00067	A14031	899
A00067	A14032	899	A00067	A14033	899
A00067	A14038	899	A00067	A14043	899
A00067	A14044	899	A00067	A14046	899
A00067	A14055	899	A00067	A14059	899
A00067	A14062	899	A00067	A14063	989
A00067	A14065	899	A00067	A14111	201
A00067	A14187	899	A00067	A14188	899
A00067	A14194	259	A00067	A14198	945
A00067	A14210	899	A00067	A14242	899
A00067	A14245	168	A00067	A14330	207
A00067	A14371	899	A00067	A14408	899
A00067	A14433	899	A00067	A14438	899
A00067	A14443	989	A00067	A14444	899
A00067	A14654	899	A00067	A14699	660
A00067	A14778	258	A00067	A14930	899
A00067	A14943	377	A00067	A14975	162
A00067	A14992	162	A00067	A15242	162
A00067	A15292	899	A00067	A15293	899
A00067	A15294	899	A00067	A15304	899
A00067	A15308	325	A00067	A15312	899
A00067	A15321	899	A00067	A15325	899
A00067	A15340	899	A00067	A15350	899
A00067	A15356	485	A00067	A15357	157
A00067	A15634	899	A00067	A15746	201
A00067	A15841	899	A00067	A15974	199
A00067	A16030	899	A00067	A16048	401
A00067	A16081	329	A00067	A16084	899
A00067	A16636	201	A00067	A16912	159
A00067	A16988	899	A00067	A17195	899
A00067	A17196	152	A00067	A17215	899
A00067	A17216	899	A00068	A00076	260
A00068	A00086	198	A00068	A00094	491
A00068	A00105	202	A00068	A00129	899
A00068	A00131	469	A00068	A00141	199
A00068	A00147	218	A00068	A00151	256
A00068	A00153	914	A00068	A00159	899
A00068	A00163	945	A00068	A00167	481
A00068	A00169	335	A00068	A00171	485
A00068	A00184	458	A00068	A00190	486
A00068	A00199	217	A00068	A00200	491
A00068	A00201	491	A00068	A00209	945
A00068	A00212	428	A00068	A00224	899
A00068	A00246	899	A00068	A00248	188

A00068	A00254	329	A00068	A00256	491
A00068	A00264	945	A00068	A00294	899
A00068	A00301	328	A00068	A00308	491
A00068	A00338	490	A00068	A00377	168
A00068	A00472	485	A00068	A00485	480
A00068	A00535	322	A00068	A00600	170
A00068	A00643	405	A00068	A00799	396
A00068	A00805	154	A00068	A00909	296
A00068	A00931	899	A00068	A00939	347
A00068	A00958	234	A00068	A00981	208
A00068	A00985	283	A00068	A00986	322
A00068	A01022	491	A00068	A01161	557
A00068	A01211	491	A00068	A01299	174
A00068	A01441	291	A00068	A01451	208
A00068	A01481	480	A00068	A01500	195
A00068	A01512	899	A00068	A01524	933
A00068	A01551	313	A00068	A01558	899
A00068	A01582	432	A00068	A01588	899
A00068	A01590	167	A00068	A01639	899
A00068	A01668	899	A00068	A01726	452
A00068	A01728	183	A00068	A01729	85
A00068	A01733	488	A00068	A01737	480
A00068	A01743	490	A00068	A01746	472
A00068	A01754	160	A00068	A01763	446
A00068	A01775	270	A00068	A01844	899
A00068	A01876	227	A00068	A01888	249
A00068	A02124	191	A00068	A02306	251
A00068	A02411	899	A00068	A02477	473
A00068	A02482	273	A00068	A02485	380
A00068	A02490	368	A00068	A02491	288
A00068	A02493	899	A00068	A02548	268
A00068	A02637	199	A00068	A02695	312
A00068	A02697	429	A00068	A02698	480
A00068	A02714	258	A00068	A02785	940
A00068	A02822	899	A00068	A02896	199
A00068	A02908	899	A00068	A02924	489
A00068	A02982	388	A00068	A02983	151
A00068	A03259	199	A00068	A03268	899
A00068	A03292	226	A00068	A03304	329
A00068	A03402	380	A00068	A03417	374
A00068	A03459	303	A00068	A03661	341
A00068	A03662	452	A00068	A03676	251
A00068	A03686	227	A00068	A04096	899
A00068	A04337	490	A00068	A04449	188
A00068	A04532	402	A00068	A04614	906
A00068	A04652	432	A00068	A04659	906

A00068	A04737	199	A00068	A04962	234
A00068	A05115	208	A00068	A05117	156
A00068	A05245	317	A00068	A05288	480
A00068	A05295	213	A00068	A05309	485
A00068	A05331	324	A00068	A05356	573
A00068	A05418	436	A00068	A05577	288
A00068	A05891	229	A00068	A06544	327
A00068	A06607	486	A00068	A06615	899
A00068	A06628	385	A00068	A06650	491
A00068	A06954	215	A00068	A06999	170
A00068	A07011	358	A00068	A07311	257
A00068	A07339	488	A00068	A07422	899
A00068	A07442	481	A00068	A07697	899
A00068	A07911	465	A00068	A07997	200
A00068	A08093	449	A00068	A08133	199
A00068	A08158	170	A00068	A08193	199
A00068	A08255	296	A00068	A08404	486
A00068	A08627	343	A00068	A09170	170
A00068	A09592	420	A00068	A09978	268
A00068	A10532	553	A00068	A10580	899
A00068	A10667	425	A00068	A10778	375
A00068	A11299	455	A00068	A11308	339
A00068	A11313	241	A00068	A11324	371
A00068	A11326	363	A00068	A11403	215
A00068	A11411	435	A00068	A11513	467
A00068	A11604	199	A00068	A11982	180
A00068	A12373	486	A00068	A12883	246
A00068	A12897	151	A00068	A13824	899
A00068	A13873	157	A00068	A13978	899
A00068	A14211	899	A00068	A14236	246
A00068	A14272	369	A00068	A14398	170
A00068	A14444	152	A00068	A14449	284
A00068	A14759	174	A00068	A15282	241
A00068	A15292	899	A00068	A15295	958
A00068	A15296	973	A00068	A15322	200
A00068	A15422	444	A00068	A15596	269
A00068	A15766	170	A00068	A15917	391
A00068	A16016	208	A00068	A16083	899
A00068	A16120	156	A00068	A16636	432
A00069	A00072	294	A00069	A00074	402
A00069	A00076	341	A00069	A00079	157
A00069	A00080	491	A00069	A00081	945
A00069	A00083	491	A00069	A00085	491
A00069	A00087	480	A00069	A00088	229
A00069	A00089	491	A00069	A00095	481
A00069	A00097	457	A00069	A00106	489

A00069	A00111	945	A00069	A00127	491
A00069	A00131	456	A00069	A00150	578
A00069	A00159	899	A00069	A00166	406
A00069	A00167	547	A00069	A00168	491
A00069	A00173	481	A00069	A00177	642
A00069	A00178	235	A00069	A00190	479
A00069	A00191	195	A00069	A00193	252
A00069	A00196	491	A00069	A00203	193
A00069	A00208	252	A00069	A00209	945
A00069	A00212	480	A00069	A00216	246
A00069	A00218	483	A00069	A00219	945
A00069	A00221	456	A00069	A00222	374
A00069	A00223	457	A00069	A00227	369
A00069	A00245	491	A00069	A00254	477
A00069	A00264	491	A00069	A00274	483
A00069	A00292	438	A00069	A00294	171
A00069	A00309	455	A00069	A00315	230
A00069	A00324	947	A00069	A00327	283
A00069	A00328	462	A00069	A00335	414
A00069	A00338	363	A00069	A00350	177
A00069	A00352	243	A00069	A00356	162
A00069	A00363	389	A00069	A00370	297
A00069	A00377	252	A00069	A00405	314
A00069	A00406	183	A00069	A00440	299
A00069	A00472	385	A00069	A00485	316
A00069	A00500	360	A00069	A00532	221
A00069	A00616	491	A00069	A00643	477
A00069	A00651	330	A00069	A00668	171
A00069	A00700	241	A00069	A00736	486
A00069	A00747	294	A00069	A00752	201
A00069	A00760	472	A00069	A00815	491
A00069	A00820	172	A00069	A00831	291
A00069	A00882	294	A00069	A00916	483
A00069	A00931	350	A00069	A00981	270
A00069	A00997	204	A00069	A01005	279
A00069	A01022	344	A00069	A01075	290
A00069	A01091	435	A00069	A01112	431
A00069	A01125	642	A00069	A01137	484
A00069	A01140	477	A00069	A01157	228
A00069	A01161	547	A00069	A01166	399
A00069	A01196	281	A00069	A01211	221
A00069	A01225	185	A00069	A01235	462
A00069	A01248	193	A00069	A01257	491
A00069	A01277	346	A00069	A01291	288
A00069	A01419	290	A00069	A01425	484
A00069	A01460	480	A00069	A01461	589

A00069	A01468	474	A00069	A01470	256
A00069	A01476	204	A00069	A01485	490
A00069	A01487	435	A00069	A01488	229
A00069	A01492	553	A00069	A01515	491
A00069	A01524	920	A00069	A01525	159
A00069	A01527	914	A00069	A01531	451
A00069	A01553	899	A00069	A01558	899
A00069	A01573	571	A00069	A01579	470
A00069	A01588	899	A00069	A01590	379
A00069	A01597	321	A00069	A01598	162
A00069	A01601	623	A00069	A01605	484
A00069	A01612	152	A00069	A01640	154
A00069	A01722	449	A00069	A01740	344
A00069	A01741	424	A00069	A01743	162
A00069	A01775	899	A00069	A01790	241
A00069	A01925	204	A00069	A01926	446
A00069	A01931	439	A00069	A01995	262
A00069	A02044	207	A00069	A02048	205
A00069	A02049	286	A00069	A02074	238
A00069	A02083	193	A00069	A02104	906
A00069	A02105	901	A00069	A02111	475
A00069	A02126	270	A00069	A02133	260
A00069	A02143	419	A00069	A02148	465
A00069	A02156	251	A00069	A02161	159
A00069	A02174	483	A00069	A02176	361
A00069	A02179	424	A00069	A02180	202
A00069	A02186	277	A00069	A02213	445
A00069	A02215	490	A00069	A02228	446
A00069	A02229	474	A00069	A02236	235
A00069	A02245	255	A00069	A02251	243
A00069	A02265	263	A00069	A02278	283
A00069	A02298	251	A00069	A02303	187
A00069	A02304	468	A00069	A02305	96
A00069	A02306	480	A00069	A02309	245
A00069	A02313	485	A00069	A02318	196
A00069	A02326	82	A00069	A02364	414
A00069	A02377	259	A00069	A02383	160
A00069	A02390	906	A00069	A02393	204
A00069	A02397	490	A00069	A02401	159
A00069	A02405	414	A00069	A02419	201
A00069	A02423	474	A00069	A02425	486
A00069	A02431	375	A00069	A02449	177
A00069	A02465	489	A00069	A02468	183
A00069	A02482	157	A00069	A02490	449
A00069	A02491	216	A00069	A02504	193
A00069	A02545	168	A00069	A02572	160

A00069	A02588	167	A00069	A02644	63
A00069	A02667	217	A00069	A02668	423
A00069	A02671	404	A00069	A02672	356
A00069	A02673	490	A00069	A02756	439
A00069	A02758	329	A00069	A02759	196
A00069	A02772	91	A00069	A02785	433
A00069	A02836	360	A00069	A02864	238
A00069	A02877	301	A00069	A02959	344
A00069	A02983	371	A00069	A03053	168
A00069	A03054	945	A00069	A03070	438
A00069	A03076	400	A00069	A03077	152
A00069	A03080	283	A00069	A03083	170
A00069	A03099	395	A00069	A03104	483
A00069	A03110	491	A00069	A03112	213
A00069	A03115	245	A00069	A03141	219
A00069	A03160	168	A00069	A03183	254
A00069	A03186	383	A00069	A03191	226
A00069	A03201	215	A00069	A03203	223
A00069	A03219	472	A00069	A03259	316
A00069	A03300	413	A00069	A03304	213
A00069	A03310	99	A00069	A03314	484
A00069	A03315	79	A00069	A03379	322
A00069	A03402	465	A00069	A03413	215
A00069	A03438	191	A00069	A03451	160
A00069	A03458	283	A00069	A03498	168
A00069	A03573	243	A00069	A03578	143
A00069	A03584	174	A00069	A03588	167
A00069	A03633	927	A00069	A03639	491
A00069	A03726	165	A00069	A03744	221
A00069	A03748	205	A00069	A03749	223
A00069	A03752	179	A00069	A03786	188
A00069	A03787	159	A00069	A03799	454
A00069	A03818	341	A00069	A03880	424
A00069	A03893	341	A00069	A03910	708
A00069	A03942	466	A00069	A03960	229
A00069	A03961	491	A00069	A03963	477
A00069	A04082	168	A00069	A04092	204
A00069	A04163	301	A00069	A04166	291
A00069	A04169	433	A00069	A04180	213
A00069	A04202	945	A00069	A04225	276
A00069	A04230	350	A00069	A04256	347
A00069	A04326	152	A00069	A04337	420
A00069	A04366	168	A00069	A04380	353
A00069	A04423	297	A00069	A04430	276
A00069	A04449	489	A00069	A04464	188
A00069	A04490	208	A00069	A04504	283

A00069	A04605	152	A00069	A04626	204
A00069	A04633	173	A00069	A04643	177
A00069	A04668	314	A00069	A04676	291
A00069	A04697	362	A00069	A04751	343
A00069	A04816	177	A00069	A04843	177
A00069	A04976	480	A00069	A05011	204
A00069	A05015	263	A00069	A05048	188
A00069	A05091	226	A00069	A05118	310
A00069	A05131	159	A00069	A05155	332
A00069	A05182	305	A00069	A05184	486
A00069	A05208	183	A00069	A05216	899
A00069	A05234	421	A00069	A05288	272
A00069	A05337	385	A00069	A05356	583
A00069	A05366	188	A00069	A05377	468
A00069	A05418	278	A00069	A05439	451
A00069	A05443	299	A00069	A05457	167
A00069	A05494	380	A00069	A05533	491
A00069	A05534	167	A00069	A05535	291
A00069	A05588	188	A00069	A05653	435
A00069	A05654	401	A00069	A05812	411
A00069	A05906	260	A00069	A05944	226
A00069	A05959	258	A00069	A05992	447
A00069	A06008	204	A00069	A06033	204
A00069	A06100	290	A00069	A06249	204
A00069	A06286	414	A00069	A06340	427
A00069	A06343	177	A00069	A06371	384
A00069	A06379	157	A00069	A06423	258
A00069	A06452	226	A00069	A06534	294
A00069	A06606	180	A00069	A06607	235
A00069	A06610	391	A00069	A06719	297
A00069	A06762	467	A00069	A06922	177
A00069	A07047	162	A00069	A07115	270
A00069	A07143	174	A00069	A07163	165
A00069	A07189	152	A00069	A07209	450
A00069	A07296	283	A00069	A07297	159
A00069	A07398	346	A00069	A07422	945
A00069	A07460	322	A00069	A07495	262
A00069	A07581	156	A00069	A07623	188
A00069	A07726	230	A00069	A07803	389
A00069	A07815	238	A00069	A07968	177
A00069	A08284	243	A00069	A08288	188
A00069	A08408	318	A00069	A08424	152
A00069	A08484	159	A00069	A08630	229
A00069	A08667	180	A00069	A08714	115
A00069	A08859	479	A00069	A09028	491
A00069	A09054	692	A00069	A09070	204

A00069	A09075	335	A00069	A09198	188
A00069	A09213	389	A00069	A09235	152
A00069	A09238	255	A00069	A09245	455
A00069	A09254	314	A00069	A09319	899
A00069	A09429	93	A00069	A09480	330
A00069	A09550	475	A00069	A09552	299
A00069	A09732	490	A00069	A09733	462
A00069	A09743	352	A00069	A09868	159
A00069	A09942	260	A00069	A09958	260
A00069	A10040	234	A00069	A10090	159
A00069	A10113	177	A00069	A10145	322
A00069	A10306	226	A00069	A10336	490
A00069	A10482	226	A00069	A10525	472
A00069	A10633	456	A00069	A10645	457
A00069	A10779	226	A00069	A10841	226
A00069	A10928	204	A00069	A11170	167
A00069	A11190	480	A00069	A11279	154
A00069	A11313	456	A00069	A11327	294
A00069	A11387	291	A00069	A11415	281
A00069	A11522	353	A00069	A11527	319
A00069	A11548	718	A00069	A11846	384
A00069	A11857	180	A00069	A11893	167
A00069	A11913	152	A00069	A11983	512
A00069	A12082	187	A00069	A12108	167
A00069	A12246	247	A00069	A12265	177
A00069	A12273	491	A00069	A12393	334
A00069	A12459	167	A00069	A12560	899
A00069	A12682	335	A00069	A12897	337
A00069	A13063	657	A00069	A13108	310
A00069	A13344	204	A00069	A13667	224
A00069	A13832	480	A00069	A13849	167
A00069	A13874	491	A00069	A13875	167
A00069	A13888	226	A00069	A13944	462
A00069	A13987	221	A00069	A13989	719
A00069	A13994	462	A00069	A14007	201
A00069	A14010	462	A00069	A14051	226
A00069	A14052	486	A00069	A14053	260
A00069	A14190	462	A00069	A14198	165
A00069	A14202	351	A00069	A14210	490
A00069	A14247	617	A00069	A14371	477
A00069	A14378	228	A00069	A14399	430
A00069	A14443	246	A00069	A14490	243
A00069	A14496	366	A00069	A14511	491
A00069	A14579	156	A00069	A14604	435
A00069	A14693	157	A00069	A14722	188
A00069	A14901	177	A00069	A15229	429

A00069	A15230	480	A00069	A15268	466
A00069	A15292	635	A00069	A15312	414
A00069	A15313	489	A00069	A15317	156
A00069	A15321	230	A00069	A15322	255
A00069	A15345	262	A00069	A15349	469
A00069	A15352	385	A00069	A15381	461
A00069	A15384	414	A00069	A15396	205
A00069	A15419	460	A00069	A15431	184
A00069	A15432	277	A00069	A15435	468
A00069	A15436	440	A00069	A15437	207
A00069	A15447	216	A00069	A15452	475
A00069	A15456	238	A00069	A15457	216
A00069	A15463	210	A00069	A15478	433
A00069	A15480	205	A00069	A15495	341
A00069	A15498	489	A00069	A15552	484
A00069	A15598	467	A00069	A15671	213
A00069	A15904	152	A00069	A15909	412
A00069	A15913	491	A00069	A16001	260
A00069	A16107	369	A00069	A16254	260
A00069	A16257	351	A00069	A16636	470
A00069	A16648	177	A00069	A16778	252
A00069	A17061	450	A00069	A17201	226
A00070	A00157	899	A00070	A00190	899
A00070	A00209	899	A00070	A01640	899
A00070	A09644	899	A00071	A00074	168
A00071	A00127	466	A00071	A00160	73
A00071	A00161	944	A00071	A00162	99
A00071	A00190	398	A00071	A00208	63
A00071	A00262	367	A00071	A00561	274
A00071	A00747	293	A00071	A00911	196
A00071	A01191	154	A00071	A01400	374
A00071	A01527	899	A00071	A01551	174
A00071	A01668	294	A00071	A01675	491
A00071	A01691	441	A00071	A01722	443
A00071	A01729	156	A00071	A01737	372
A00071	A01741	340	A00071	A01746	218
A00071	A01751	302	A00071	A01757	157
A00071	A01758	474	A00071	A01768	174
A00071	A01787	137	A00071	A01793	271
A00071	A01796	491	A00071	A01797	443
A00071	A01873	285	A00071	A01890	425
A00071	A01894	363	A00071	A01895	443
A00071	A01896	473	A00071	A01899	467
A00071	A01908	112	A00071	A01909	213
A00071	A01914	491	A00071	A01951	276
A00071	A02109	152	A00071	A02183	296

A00071	A02208	486	A00071	A02228	229
A00071	A02327	195	A00071	A02338	154
A00071	A02341	417	A00071	A02342	162
A00071	A02392	257	A00071	A02417	234
A00071	A02425	168	A00071	A02435	277
A00071	A02498	156	A00071	A02586	396
A00071	A02616	319	A00071	A02629	290
A00071	A02632	201	A00071	A02638	263
A00071	A02687	419	A00071	A02700	335
A00071	A02702	236	A00071	A02707	168
A00071	A02734	452	A00071	A02749	379
A00071	A02758	157	A00071	A02766	188
A00071	A02811	162	A00071	A02819	183
A00071	A02905	174	A00071	A02906	205
A00071	A02943	251	A00071	A02988	401
A00071	A03024	230	A00071	A03041	182
A00071	A03120	269	A00071	A03186	447
A00071	A03225	475	A00071	A03242	160
A00071	A03263	319	A00071	A03434	450
A00071	A03537	257	A00071	A03583	230
A00071	A03585	210	A00071	A03593	307
A00071	A03594	224	A00071	A03598	355
A00071	A03611	154	A00071	A03638	202
A00071	A03847	294	A00071	A03870	269
A00071	A03964	401	A00071	A04004	297
A00071	A04031	215	A00071	A04053	154
A00071	A04123	269	A00071	A04228	258
A00071	A04304	418	A00071	A04314	480
A00071	A04315	202	A00071	A04432	294
A00071	A04443	335	A00071	A04560	230
A00071	A04594	307	A00071	A04623	489
A00071	A04880	451	A00071	A04904	246
A00071	A04905	319	A00071	A05036	251
A00071	A05216	187	A00071	A05247	162
A00071	A05253	241	A00071	A05390	154
A00071	A05440	416	A00071	A05463	172
A00071	A05493	218	A00071	A05494	235
A00071	A05533	238	A00071	A05575	238
A00071	A05683	389	A00071	A05899	276
A00071	A05940	195	A00071	A05984	294
A00071	A06157	323	A00071	A06579	177
A00071	A06603	422	A00071	A06607	472
A00071	A06623	434	A00071	A06703	241
A00071	A07167	439	A00071	A07177	339
A00071	A07199	208	A00071	A07200	423
A00071	A07335	196	A00071	A08397	294

A00071	A08911	135	A00071	A09084	424
A00071	A09438	170	A00071	A10239	294
A00071	A10332	174	A00071	A10645	156
A00071	A11408	168	A00071	A11460	218
A00071	A11539	458	A00071	A11606	469
A00071	A11646	366	A00071	A11900	230
A00071	A13917	154	A00071	A14036	154
A00071	A14332	409	A00071	A14405	449
A00071	A14426	294	A00071	A14440	190
A00071	A14533	307	A00071	A14642	389
A00071	A14643	348	A00071	A14647	159
A00071	A14942	152	A00071	A16281	491
A00071	A16438	196	A00071	A16739	157
A00071	A16744	165	A00071	A16792	174
A00072	A00085	207	A00072	A00155	423
A00072	A00168	475	A00072	A00209	899
A00072	A00216	452	A00072	A00219	361
A00072	A00312	257	A00072	A00567	479
A00072	A01137	324	A00072	A01257	160
A00072	A01404	160	A00072	A01527	899
A00072	A01590	396	A00072	A02083	213
A00072	A02143	210	A00072	A02306	461
A00072	A02313	443	A00072	A02330	229
A00072	A02416	190	A00072	A02667	258
A00072	A02749	165	A00072	A02759	288
A00072	A02785	308	A00072	A03051	251
A00072	A03087	274	A00072	A03099	441
A00072	A03183	274	A00072	A03402	224
A00072	A03408	180	A00072	A03584	278
A00072	A03639	251	A00072	A03961	374
A00072	A04162	260	A00072	A04428	362
A00072	A04463	344	A00072	A04746	324
A00072	A05533	278	A00072	A06607	168
A00072	A08878	899	A00072	A09072	474
A00072	A09442	462	A00072	A09480	353
A00072	A10018	260	A00072	A11485	210
A00072	A11983	899	A00072	A15913	251
A00073	A00074	480	A00073	A00094	156
A00073	A00110	899	A00073	A00127	374
A00073	A00132	664	A00073	A00138	550
A00073	A00140	901	A00073	A00146	678
A00073	A00147	475	A00073	A00150	949
A00073	A00151	190	A00073	A00152	485
A00073	A00156	164	A00073	A00157	63
A00073	A00159	965	A00073	A00163	234
A00073	A00165	702	A00073	A00167	944

A00073	A00180	168	A00073	A00187	480
A00073	A00198	243	A00073	A00206	701
A00073	A00209	899	A00073	A00210	151
A00073	A00213	486	A00073	A00214	631
A00073	A00220	899	A00073	A00224	950
A00073	A00225	976	A00073	A00226	491
A00073	A00230	899	A00073	A00233	159
A00073	A00234	434	A00073	A00239	184
A00073	A00243	196	A00073	A00246	899
A00073	A00248	324	A00073	A00250	552
A00073	A00252	901	A00073	A00253	941
A00073	A00254	486	A00073	A00257	491
A00073	A00261	488	A00073	A00265	597
A00073	A00285	462	A00073	A00286	417
A00073	A00306	470	A00073	A00316	485
A00073	A00348	483	A00073	A00356	353
A00073	A00371	628	A00073	A00378	424
A00073	A00382	469	A00073	A00395	191
A00073	A00420	457	A00073	A00433	334
A00073	A00436	234	A00073	A00444	481
A00073	A00489	470	A00073	A00500	296
A00073	A00517	486	A00073	A00531	180
A00073	A00532	434	A00073	A00552	305
A00073	A00557	269	A00073	A00587	215
A00073	A00640	481	A00073	A00658	473
A00073	A00706	530	A00073	A00736	491
A00073	A00740	346	A00073	A00757	346
A00073	A00764	195	A00073	A00805	473
A00073	A00831	275	A00073	A00832	902
A00073	A00835	353	A00073	A00843	647
A00073	A00847	469	A00073	A00848	490
A00073	A00878	205	A00073	A00905	249
A00073	A00932	372	A00073	A00962	261
A00073	A00970	491	A00073	A00971	709
A00073	A00973	390	A00073	A01021	207
A00073	A01022	180	A00073	A01042	307
A00073	A01052	483	A00073	A01069	491
A00073	A01077	408	A00073	A01083	422
A00073	A01088	170	A00073	A01090	361
A00073	A01112	285	A00073	A01113	577
A00073	A01115	491	A00073	A01141	210
A00073	A01150	232	A00073	A01161	241
A00073	A01173	355	A00073	A01192	627
A00073	A01194	488	A00073	A01212	401
A00073	A01214	185	A00073	A01215	435
A00073	A01242	207	A00073	A01291	943

A00073	A01307	481	A00073	A01311	486
A00073	A01353	251	A00073	A01354	491
A00073	A01361	384	A00073	A01410	301
A00073	A01419	327	A00073	A01425	185
A00073	A01438	313	A00073	A01457	156
A00073	A01473	465	A00073	A01492	903
A00073	A01506	270	A00073	A01508	229
A00073	A01527	899	A00073	A01539	473
A00073	A01541	234	A00073	A01543	344
A00073	A01548	483	A00073	A01551	407
A00073	A01553	327	A00073	A01554	950
A00073	A01558	900	A00073	A01560	565
A00073	A01562	346	A00073	A01569	491
A00073	A01572	491	A00073	A01574	180
A00073	A01576	560	A00073	A01577	475
A00073	A01579	172	A00073	A01580	327
A00073	A01588	899	A00073	A01591	443
A00073	A01592	270	A00073	A01596	687
A00073	A01610	469	A00073	A01628	305
A00073	A01636	906	A00073	A01637	490
A00073	A01639	490	A00073	A01640	221
A00073	A01648	334	A00073	A01650	490
A00073	A01651	940	A00073	A01655	440
A00073	A01664	491	A00073	A01668	449
A00073	A01707	490	A00073	A01709	294
A00073	A01730	720	A00073	A01770	945
A00073	A01781	193	A00073	A01844	942
A00073	A01920	486	A00073	A01995	156
A00073	A02030	329	A00073	A02164	162
A00073	A02353	461	A00073	A02452	407
A00073	A02470	240	A00073	A02487	409
A00073	A02512	160	A00073	A02564	159
A00073	A02715	491	A00073	A02736	274
A00073	A02737	962	A00073	A02825	962
A00073	A02826	79	A00073	A02833	469
A00073	A02870	90	A00073	A02881	66
A00073	A02904	472	A00073	A03112	63
A00073	A03214	63	A00073	A03321	358
A00073	A03335	324	A00073	A03338	432
A00073	A03436	156	A00073	A03446	486
A00073	A03453	152	A00073	A03476	267
A00073	A03477	481	A00073	A03560	614
A00073	A03568	208	A00073	A03569	407
A00073	A03575	152	A00073	A03586	367
A00073	A03652	196	A00073	A03665	195
A00073	A03738	463	A00073	A03740	347

A00073	A03752	465	A00073	A03837	200
A00073	A03863	224	A00073	A03886	434
A00073	A03893	291	A00073	A03990	254
A00073	A04012	949	A00073	A04050	358
A00073	A04115	252	A00073	A04161	229
A00073	A04248	899	A00073	A04346	270
A00073	A04537	462	A00073	A04697	446
A00073	A05029	975	A00073	A05232	168
A00073	A05313	396	A00073	A05633	322
A00073	A05645	477	A00073	A05683	230
A00073	A05712	945	A00073	A05760	899
A00073	A05857	205	A00073	A05995	252
A00073	A06025	301	A00073	A06051	218
A00073	A06115	168	A00073	A06198	205
A00073	A06235	154	A00073	A06398	452
A00073	A06466	252	A00073	A06489	486
A00073	A06581	252	A00073	A06597	488
A00073	A06701	491	A00073	A06892	332
A00073	A06907	457	A00073	A07247	490
A00073	A07309	398	A00073	A07311	491
A00073	A07378	334	A00073	A07422	932
A00073	A07589	130	A00073	A07594	368
A00073	A07604	319	A00073	A07726	939
A00073	A07798	227	A00073	A07803	351
A00073	A07814	201	A00073	A07985	399
A00073	A08061	491	A00073	A08261	408
A00073	A08293	162	A00073	A08541	258
A00073	A08546	195	A00073	A08860	692
A00073	A08896	380	A00073	A08978	204
A00073	A09162	360	A00073	A09218	414
A00073	A09323	430	A00073	A09591	334
A00073	A09641	259	A00073	A09837	170
A00073	A09873	252	A00073	A09977	218
A00073	A10006	486	A00073	A10014	63
A00073	A10082	99	A00073	A10159	343
A00073	A10174	899	A00073	A10183	205
A00073	A10238	486	A00073	A10244	168
A00073	A10266	130	A00073	A10365	202
A00073	A10459	202	A00073	A10480	205
A00073	A10490	235	A00073	A10532	567
A00073	A10575	899	A00073	A10580	259
A00073	A10601	183	A00073	A10623	278
A00073	A10847	274	A00073	A11028	168
A00073	A11409	344	A00073	A11474	447
A00073	A11487	334	A00073	A11622	168
A00073	A11623	168	A00073	A11639	168

A00073	A12383	903	A00073	A12988	481
A00073	A13210	205	A00073	A13231	263
A00073	A13395	205	A00073	A13465	436
A00073	A13511	610	A00073	A13552	355
A00073	A13609	490	A00073	A13694	188
A00073	A13824	398	A00073	A13829	173
A00073	A13975	373	A00073	A13989	945
A00073	A14006	491	A00073	A14073	263
A00073	A14074	324	A00073	A14178	334
A00073	A14196	491	A00073	A14207	199
A00073	A14208	609	A00073	A14218	291
A00073	A14246	162	A00073	A14299	540
A00073	A14351	267	A00073	A14379	367
A00073	A14399	202	A00073	A14443	949
A00073	A14559	154	A00073	A15244	180
A00073	A15291	486	A00073	A15314	656
A00073	A15336	180	A00073	A15361	246
A00073	A15386	333	A00073	A15612	188
A00073	A15696	180	A00073	A15698	322
A00073	A16216	496	A00073	A16217	311
A00073	A16705	210	A00073	A16784	205
A00073	A17063	311	A00073	A17088	334
A00074	A00076	184	A00074	A00081	441
A00074	A00089	232	A00074	A00090	489
A00074	A00091	438	A00074	A00094	450
A00074	A00099	899	A00074	A00108	63
A00074	A00113	301	A00074	A00114	63
A00074	A00122	491	A00074	A00132	491
A00074	A00137	63	A00074	A00138	614
A00074	A00140	63	A00074	A00141	491
A00074	A00145	374	A00074	A00146	351
A00074	A00147	430	A00074	A00149	899
A00074	A00152	945	A00074	A00153	165
A00074	A00156	563	A00074	A00157	905
A00074	A00158	188	A00074	A00159	950
A00074	A00161	490	A00074	A00162	945
A00074	A00163	994	A00074	A00164	187
A00074	A00167	554	A00074	A00171	899
A00074	A00173	486	A00074	A00183	362
A00074	A00185	969	A00074	A00189	213
A00074	A00190	945	A00074	A00205	907
A00074	A00206	296	A00074	A00207	977
A00074	A00208	491	A00074	A00211	899
A00074	A00214	950	A00074	A00216	184
A00074	A00221	369	A00074	A00224	953
A00074	A00227	163	A00074	A00228	475

A00074	A00230	945	A00074	A00232	232
A00074	A00234	945	A00074	A00240	936
A00074	A00246	944	A00074	A00250	899
A00074	A00253	419	A00074	A00254	985
A00074	A00255	382	A00074	A00257	462
A00074	A00261	249	A00074	A00265	355
A00074	A00270	532	A00074	A00274	212
A00074	A00294	374	A00074	A00316	462
A00074	A00324	912	A00074	A00370	465
A00074	A00378	293	A00074	A00400	478
A00074	A00470	899	A00074	A00552	201
A00074	A00553	899	A00074	A00556	307
A00074	A00566	427	A00074	A00641	907
A00074	A00643	556	A00074	A00670	439
A00074	A00671	486	A00074	A00687	486
A00074	A00697	109	A00074	A00747	486
A00074	A00814	160	A00074	A00820	262
A00074	A00875	254	A00074	A00914	913
A00074	A00981	160	A00074	A01052	901
A00074	A01158	184	A00074	A01164	200
A00074	A01209	200	A00074	A01275	489
A00074	A01286	188	A00074	A01357	63
A00074	A01410	228	A00074	A01452	474
A00074	A01473	490	A00074	A01474	419
A00074	A01489	899	A00074	A01524	971
A00074	A01527	995	A00074	A01553	263
A00074	A01554	76	A00074	A01558	994
A00074	A01560	945	A00074	A01561	905
A00074	A01562	932	A00074	A01572	950
A00074	A01576	201	A00074	A01587	899
A00074	A01588	994	A00074	A01592	899
A00074	A01602	945	A00074	A01605	899
A00074	A01615	992	A00074	A01628	899
A00074	A01636	899	A00074	A01637	899
A00074	A01638	899	A00074	A01639	899
A00074	A01640	989	A00074	A01650	899
A00074	A01688	899	A00074	A01692	486
A00074	A01697	171	A00074	A01702	212
A00074	A01708	899	A00074	A01709	899
A00074	A01737	187	A00074	A01741	463
A00074	A01775	899	A00074	A01789	899
A00074	A01796	198	A00074	A01797	899
A00074	A01844	945	A00074	A01845	324
A00074	A01846	347	A00074	A01847	434
A00074	A01848	321	A00074	A01900	341
A00074	A01914	475	A00074	A01920	899

A00074	A01995	544	A00074	A02086	475
A00074	A02121	196	A00074	A02162	272
A00074	A02163	324	A00074	A02183	212
A00074	A02268	224	A00074	A02304	201
A00074	A02411	914	A00074	A02424	899
A00074	A02564	326	A00074	A02678	655
A00074	A02749	199	A00074	A02802	294
A00074	A02803	210	A00074	A02872	899
A00074	A02887	63	A00074	A02902	191
A00074	A02908	899	A00074	A02942	916
A00074	A02968	899	A00074	A03014	298
A00074	A03019	251	A00074	A03049	165
A00074	A03050	571	A00074	A03110	200
A00074	A03112	326	A00074	A03213	491
A00074	A03219	184	A00074	A03271	379
A00074	A03273	319	A00074	A03275	210
A00074	A03292	359	A00074	A03304	435
A00074	A03321	184	A00074	A03435	180
A00074	A03465	420	A00074	A03575	899
A00074	A03591	911	A00074	A03592	162
A00074	A03719	184	A00074	A03784	363
A00074	A03809	210	A00074	A03854	899
A00074	A04012	386	A00074	A04051	297
A00074	A04269	162	A00074	A04622	456
A00074	A04696	372	A00074	A04796	200
A00074	A04797	162	A00074	A05111	188
A00074	A05158	413	A00074	A05216	945
A00074	A05339	188	A00074	A05428	899
A00074	A05453	314	A00074	A05454	177
A00074	A05560	899	A00074	A05633	382
A00074	A05760	908	A00074	A05762	200
A00074	A06067	184	A00074	A06082	360
A00074	A06159	180	A00074	A06167	154
A00074	A06408	380	A00074	A06496	285
A00074	A06578	184	A00074	A06581	301
A00074	A06597	535	A00074	A06610	488
A00074	A06783	358	A00074	A07004	491
A00074	A07011	899	A00074	A07017	163
A00074	A07154	63	A00074	A07165	989
A00074	A07183	420	A00074	A07190	436
A00074	A07311	994	A00074	A07339	929
A00074	A07350	162	A00074	A07399	232
A00074	A07421	262	A00074	A07568	238
A00074	A07598	184	A00074	A07833	238
A00074	A08207	363	A00074	A08345	226
A00074	A08360	246	A00074	A08521	252

A00074	A08571	899	A00074	A08648	200
A00074	A08732	899	A00074	A08905	229
A00074	A08993	232	A00074	A09078	246
A00074	A09119	200	A00074	A09176	141
A00074	A09181	899	A00074	A09188	271
A00074	A09218	461	A00074	A09220	338
A00074	A09251	413	A00074	A09319	899
A00074	A09351	184	A00074	A09392	385
A00074	A09401	63	A00074	A09542	899
A00074	A09577	899	A00074	A09580	174
A00074	A09598	151	A00074	A09643	899
A00074	A09937	82	A00074	A10115	162
A00074	A10155	296	A00074	A10365	899
A00074	A10576	63	A00074	A10615	899
A00074	A10623	899	A00074	A10666	180
A00074	A10735	383	A00074	A10756	291
A00074	A10801	992	A00074	A10899	63
A00074	A11042	162	A00074	A11316	429
A00074	A11369	486	A00074	A11476	260
A00074	A11487	177	A00074	A11558	184
A00074	A11591	273	A00074	A11604	899
A00074	A11729	432	A00074	A11851	184
A00074	A11983	909	A00074	A12246	266
A00074	A12383	928	A00074	A12680	200
A00074	A12927	899	A00074	A13225	899
A00074	A13356	184	A00074	A13364	488
A00074	A13433	271	A00074	A13511	490
A00074	A13605	141	A00074	A13841	296
A00074	A13882	352	A00074	A13902	254
A00074	A13975	480	A00074	A13980	462
A00074	A13981	989	A00074	A13983	63
A00074	A13986	488	A00074	A13998	899
A00074	A14004	469	A00074	A14007	472
A00074	A14019	115	A00074	A14047	350
A00074	A14187	945	A00074	A14188	945
A00074	A14198	955	A00074	A14202	899
A00074	A14208	475	A00074	A14210	184
A00074	A14299	171	A00074	A14400	207
A00074	A14405	228	A00074	A14430	443
A00074	A14433	945	A00074	A14440	236
A00074	A14443	899	A00074	A14444	899
A00074	A14452	452	A00074	A14596	556
A00074	A14635	274	A00074	A14643	302
A00074	A14930	913	A00074	A15182	924
A00074	A15294	167	A00074	A15420	446
A00074	A15624	391	A00074	A15675	899

A00074	A15726	577	A00074	A16068	899
A00074	A16073	929	A00074	A16152	184
A00074	A16298	243	A00074	A16304	270
A00074	A16421	200	A00074	A16770	200
A00074	A16791	899	A00074	A17042	180
A00075	A00167	899	A00075	A00170	918
A00075	A00209	929	A00075	A00216	188
A00075	A00229	484	A00075	A00234	481
A00075	A00272	945	A00075	A00298	321
A00075	A00343	236	A00075	A00731	188
A00075	A01639	909	A00075	A01798	152
A00075	A02329	215	A00075	A02545	396
A00075	A02836	467	A00075	A03050	472
A00075	A03070	184	A00075	A03172	271
A00075	A03322	224	A00075	A03854	232
A00075	A04074	170	A00075	A05081	385
A00075	A05701	165	A00075	A06701	170
A00075	A07422	916	A00075	A09072	187
A00075	A09220	281	A00075	A10392	899
A00075	A11834	973	A00075	A13978	899
A00075	A14062	472	A00075	A14063	327
A00075	A14964	188	A00076	A00089	458
A00076	A00144	488	A00076	A00159	899
A00076	A00164	548	A00076	A00167	899
A00076	A00168	185	A00076	A00190	484
A00076	A00196	396	A00076	A00202	477
A00076	A00209	899	A00076	A00212	470
A00076	A00216	184	A00076	A00218	247
A00076	A00219	184	A00076	A00238	411
A00076	A00274	490	A00076	A00338	374
A00076	A00377	236	A00076	A00385	313
A00076	A00434	357	A00076	A00446	486
A00076	A00472	374	A00076	A00476	210
A00076	A00477	446	A00076	A00483	489
A00076	A00485	469	A00076	A00576	491
A00076	A00577	251	A00076	A00591	475
A00076	A00661	288	A00076	A00697	180
A00076	A00736	399	A00076	A00752	457
A00076	A00799	307	A00076	A00802	486
A00076	A00849	179	A00076	A00863	201
A00076	A00882	425	A00076	A00981	177
A00076	A00985	477	A00076	A00986	391
A00076	A01004	491	A00076	A01022	423
A00076	A01027	299	A00076	A01043	238
A00076	A01102	285	A00076	A01156	486
A00076	A01158	637	A00076	A01161	578

A00076	A01169	319	A00076	A01183	270
A00076	A01252	899	A00076	A01276	466
A00076	A01288	265	A00076	A01326	488
A00076	A01329	389	A00076	A01343	302
A00076	A01385	486	A00076	A01465	218
A00076	A01524	899	A00076	A01543	281
A00076	A01578	251	A00076	A01585	474
A00076	A01593	899	A00076	A01602	184
A00076	A01632	434	A00076	A01661	489
A00076	A01727	235	A00076	A01741	943
A00076	A01743	391	A00076	A01870	288
A00076	A01970	184	A00076	A01971	366
A00076	A01997	402	A00076	A02046	157
A00076	A02056	485	A00076	A02062	191
A00076	A02099	241	A00076	A02143	384
A00076	A02158	258	A00076	A02180	443
A00076	A02182	480	A00076	A02214	474
A00076	A02240	371	A00076	A02249	152
A00076	A02292	260	A00076	A02418	276
A00076	A02511	168	A00076	A02604	154
A00076	A02609	380	A00076	A02624	358
A00076	A02654	435	A00076	A02655	154
A00076	A02727	441	A00076	A02859	232
A00076	A02885	182	A00076	A03045	326
A00076	A03079	488	A00076	A03080	335
A00076	A03081	319	A00076	A03093	273
A00076	A03109	456	A00076	A03110	479
A00076	A03170	165	A00076	A03211	215
A00076	A03219	184	A00076	A03224	226
A00076	A03239	375	A00076	A03259	265
A00076	A03304	402	A00076	A03314	152
A00076	A03399	288	A00076	A03408	458
A00076	A03412	151	A00076	A03501	347
A00076	A03719	244	A00076	A03721	167
A00076	A03724	490	A00076	A03731	344
A00076	A03732	491	A00076	A03746	193
A00076	A03763	489	A00076	A03831	173
A00076	A03879	398	A00076	A03899	432
A00076	A03910	480	A00076	A03942	423
A00076	A03945	490	A00076	A03946	440
A00076	A04020	480	A00076	A04061	485
A00076	A04169	340	A00076	A04192	173
A00076	A04252	480	A00076	A04337	486
A00076	A04351	201	A00076	A04362	454
A00076	A04418	154	A00076	A04422	488
A00076	A04448	204	A00076	A04492	185

A00076	A04652	232	A00076	A04668	345
A00076	A04671	260	A00076	A04985	286
A00076	A04987	486	A00076	A05047	274
A00076	A05155	467	A00076	A05289	152
A00076	A05309	154	A00076	A05356	254
A00076	A05397	218	A00076	A05439	152
A00076	A05492	274	A00076	A05601	429
A00076	A05621	458	A00076	A05688	218
A00076	A05689	324	A00076	A05777	159
A00076	A05786	154	A00076	A06067	184
A00076	A06126	241	A00076	A06177	445
A00076	A06253	265	A00076	A06578	184
A00076	A06623	329	A00076	A06719	213
A00076	A06750	157	A00076	A06828	390
A00076	A06956	340	A00076	A07145	251
A00076	A07180	314	A00076	A07209	263
A00076	A07273	154	A00076	A07296	165
A00076	A07339	361	A00076	A07379	191
A00076	A07442	491	A00076	A07483	174
A00076	A07598	184	A00076	A07703	241
A00076	A08093	712	A00076	A08097	401
A00076	A08168	364	A00076	A08630	152
A00076	A08817	180	A00076	A08836	294
A00076	A08866	451	A00076	A09205	489
A00076	A09206	193	A00076	A09217	204
A00076	A09233	469	A00076	A09254	152
A00076	A09334	486	A00076	A09351	285
A00076	A09734	205	A00076	A09777	274
A00076	A09863	160	A00076	A10083	180
A00076	A10532	951	A00076	A10589	204
A00076	A10640	417	A00076	A10754	474
A00076	A10927	364	A00076	A11044	266
A00076	A11173	299	A00076	A11299	160
A00076	A11422	385	A00076	A11495	274
A00076	A11558	184	A00076	A11738	430
A00076	A11835	408	A00076	A11851	184
A00076	A12121	182	A00076	A12458	182
A00076	A12861	160	A00076	A12975	160
A00076	A13007	218	A00076	A13214	274
A00076	A13220	374	A00076	A13338	226
A00076	A13344	899	A00076	A13356	200
A00076	A13369	484	A00076	A13371	182
A00076	A13372	204	A00076	A13644	218
A00076	A13826	418	A00076	A13899	183
A00076	A13935	338	A00076	A13987	463
A00076	A14060	180	A00076	A14115	467

A00076	A14122	341	A00076	A14125	335
A00076	A14190	491	A00076	A14199	217
A00076	A14202	394	A00076	A14210	556
A00076	A14272	358	A00076	A14350	440
A00076	A14366	481	A00076	A14368	180
A00076	A14369	489	A00076	A14370	290
A00076	A14371	486	A00076	A14372	358
A00076	A14374	330	A00076	A14378	269
A00076	A14379	307	A00076	A14391	340
A00076	A14399	306	A00076	A14418	270
A00076	A14427	218	A00076	A14444	899
A00076	A14449	409	A00076	A14511	490
A00076	A14544	204	A00076	A14550	241
A00076	A14554	408	A00076	A14557	899
A00076	A14598	199	A00076	A14611	486
A00076	A14642	157	A00076	A14648	223
A00076	A14667	188	A00076	A14838	196
A00076	A15285	330	A00076	A15292	485
A00076	A15300	221	A00076	A15312	462
A00076	A15313	367	A00076	A15315	174
A00076	A15318	491	A00076	A15321	719
A00076	A15322	269	A00076	A15329	481
A00076	A15346	463	A00076	A15348	154
A00076	A15349	488	A00076	A15352	429
A00076	A15365	232	A00076	A15391	255
A00076	A15411	174	A00076	A15414	360
A00076	A15415	174	A00076	A15417	326
A00076	A15419	491	A00076	A15420	486
A00076	A15421	451	A00076	A15422	971
A00076	A15425	160	A00076	A15436	241
A00076	A15442	165	A00076	A15452	318
A00076	A15459	160	A00076	A15475	347
A00076	A15480	165	A00076	A15495	338
A00076	A15498	391	A00076	A15513	185
A00076	A15515	252	A00076	A15516	296
A00076	A15795	470	A00076	A15808	307
A00076	A15813	468	A00076	A15820	414
A00076	A15886	380	A00076	A15897	241
A00076	A15902	454	A00076	A15904	165
A00076	A15932	489	A00076	A16001	274
A00076	A16014	204	A00076	A16031	182
A00076	A16117	420	A00076	A16139	204
A00076	A16152	552	A00076	A16224	170
A00076	A16300	455	A00076	A16383	274
A00076	A16414	252	A00076	A16662	191
A00076	A16983	160	A00076	A17196	165

A00077	A00078	942	A00077	A00167	234
A00077	A00209	914	A00077	A00274	393
A00077	A00752	303	A00077	A01046	172
A00077	A01527	899	A00077	A01733	171
A00077	A01752	260	A00077	A01790	363
A00077	A01870	335	A00077	A01931	485
A00077	A01983	232	A00077	A02136	345
A00077	A02138	394	A00077	A02247	382
A00077	A02333	339	A00077	A02412	434
A00077	A02413	308	A00077	A02416	463
A00077	A02458	270	A00077	A02460	944
A00077	A02468	297	A00077	A02654	278
A00077	A02655	385	A00077	A02669	351
A00077	A02697	159	A00077	A02812	195
A00077	A02826	288	A00077	A02983	196
A00077	A02984	249	A00077	A03239	277
A00077	A03540	373	A00077	A03788	357
A00077	A03882	259	A00077	A03900	343
A00077	A03975	931	A00077	A04040	347
A00077	A04260	263	A00077	A04337	184
A00077	A04454	367	A00077	A04510	343
A00077	A04737	312	A00077	A05079	374
A00077	A05577	187	A00077	A06414	344
A00077	A06415	317	A00077	A06604	366
A00077	A07442	213	A00077	A10731	294
A00077	A13419	400	A00077	A14199	200
A00077	A14372	265	A00077	A14374	223
A00077	A14391	228	A00077	A14439	302
A00077	A14599	313	A00077	A14930	265
A00078	A00079	269	A00078	A00190	227
A00078	A00209	899	A00078	A00274	380
A00078	A00666	162	A00078	A00752	450
A00078	A01046	154	A00078	A01211	176
A00078	A01527	899	A00078	A01870	470
A00078	A02136	333	A00078	A02138	428
A00078	A02236	307	A00078	A02240	257
A00078	A02412	243	A00078	A02439	236
A00078	A02456	319	A00078	A02458	479
A00078	A02460	943	A00078	A02606	329
A00078	A02615	328	A00078	A02654	379
A00078	A02655	374	A00078	A03081	294
A00078	A03104	168	A00078	A03239	260
A00078	A03788	412	A00078	A03934	374
A00078	A03975	929	A00078	A04040	335
A00078	A04130	260	A00078	A04484	368
A00078	A05037	252	A00078	A05124	279

A00078	A05517	456	A00078	A05577	385
A00078	A05679	362	A00078	A06414	332
A00078	A06415	430	A00078	A06604	356
A00078	A07126	409	A00078	A07152	420
A00078	A07885	165	A00078	A08596	313
A00078	A09421	341	A00078	A10585	319
A00078	A10586	297	A00078	A11740	418
A00078	A12927	185	A00078	A13903	398
A00078	A13988	412	A00078	A14012	422
A00078	A14125	268	A00078	A14146	413
A00078	A14199	182	A00078	A14366	191
A00078	A14371	165	A00078	A14372	247
A00078	A14374	445	A00078	A14391	411
A00078	A14394	347	A00078	A14439	286
A00078	A14599	299	A00078	A15317	229
A00078	A15336	196	A00078	A16738	294
A00078	A17213	317	A00079	A00089	319
A00079	A00109	168	A00079	A00111	486
A00079	A00140	185	A00079	A00219	323
A00079	A00220	162	A00079	A00245	302
A00079	A00274	467	A00079	A00752	491
A00079	A00848	163	A00079	A01604	386
A00079	A01654	227	A00079	A01870	218
A00079	A01880	191	A00079	A01889	174
A00079	A02074	183	A00079	A02138	290
A00079	A02142	265	A00079	A02154	259
A00079	A02174	254	A00079	A02240	475
A00079	A02242	399	A00079	A02245	455
A00079	A02278	229	A00079	A02337	249
A00079	A02356	266	A00079	A02370	274
A00079	A02383	371	A00079	A02431	323
A00079	A02439	372	A00079	A02491	345
A00079	A02608	279	A00079	A02659	364
A00079	A02769	276	A00079	A02795	258
A00079	A02851	272	A00079	A03010	188
A00079	A03049	179	A00079	A03093	241
A00079	A03110	434	A00079	A03183	191
A00079	A03239	469	A00079	A03259	224
A00079	A03310	283	A00079	A03757	296
A00079	A03788	251	A00079	A03817	228
A00079	A03873	195	A00079	A03942	159
A00079	A03961	170	A00079	A04103	358
A00079	A04130	174	A00079	A04248	405
A00079	A04415	258	A00079	A04603	228
A00079	A04748	322	A00079	A04870	243
A00079	A05390	423	A00079	A05517	259

A00079	A05653	299	A00079	A05679	372
A00079	A05944	310	A00079	A05985	283
A00079	A06054	413	A00079	A06055	409
A00079	A06780	307	A00079	A07031	173
A00079	A07143	485	A00079	A07148	268
A00079	A07161	205	A00079	A07166	167
A00079	A07173	162	A00079	A08465	313
A00079	A08677	262	A00079	A08714	491
A00079	A09238	154	A00079	A09422	317
A00079	A09480	294	A00079	A09855	216
A00079	A12393	319	A00079	A13119	324
A00079	A13151	246	A00079	A14190	291
A00079	A14210	945	A00079	A14366	393
A00079	A14370	252	A00079	A14371	457
A00079	A14372	294	A00079	A14376	271
A00079	A14379	165	A00079	A14381	163
A00079	A14391	347	A00079	A14442	241
A00079	A14511	337	A00079	A14554	480
A00079	A14570	190	A00079	A14599	219
A00079	A14603	322	A00079	A14604	162
A00079	A14667	188	A00079	A15292	229
A00079	A15394	188	A00079	A15395	165
A00079	A15425	258	A00079	A15605	205
A00079	A15608	347	A00079	A15642	193
A00079	A15795	182	A00079	A16040	350
A00080	A00081	144	A00080	A00083	945
A00080	A00085	491	A00080	A00091	167
A00080	A00095	455	A00080	A00111	489
A00080	A00112	491	A00080	A00123	185
A00080	A00167	290	A00080	A00168	475
A00080	A00190	256	A00080	A00196	479
A00080	A00209	899	A00080	A00212	474
A00080	A00216	215	A00080	A00219	491
A00080	A00245	247	A00080	A00264	945
A00080	A00292	262	A00080	A00350	455
A00080	A00420	219	A00080	A00455	193
A00080	A00506	157	A00080	A00568	154
A00080	A00616	491	A00080	A00799	407
A00080	A01137	490	A00080	A01138	204
A00080	A01232	63	A00080	A01257	489
A00080	A01277	480	A00080	A01278	91
A00080	A01485	391	A00080	A01527	899
A00080	A01590	346	A00080	A01741	234
A00080	A01855	452	A00080	A01856	297
A00080	A01888	163	A00080	A01894	379
A00080	A01926	241	A00080	A01982	241

A00080	A01983	204	A00080	A01985	204
A00080	A02083	420	A00080	A02092	246
A00080	A02102	335	A00080	A02107	379
A00080	A02111	486	A00080	A02122	196
A00080	A02132	310	A00080	A02134	310
A00080	A02138	232	A00080	A02142	488
A00080	A02143	419	A00080	A02146	401
A00080	A02147	256	A00080	A02148	428
A00080	A02156	91	A00080	A02180	170
A00080	A02245	337	A00080	A02250	174
A00080	A02251	278	A00080	A02278	165
A00080	A02291	235	A00080	A02298	485
A00080	A02300	227	A00080	A02305	291
A00080	A02306	490	A00080	A02309	473
A00080	A02313	475	A00080	A02326	419
A00080	A02352	290	A00080	A02396	369
A00080	A02405	152	A00080	A02423	432
A00080	A02465	273	A00080	A02468	294
A00080	A02490	484	A00080	A02491	451
A00080	A02554	293	A00080	A02582	279
A00080	A02587	380	A00080	A02600	326
A00080	A02644	488	A00080	A02667	262
A00080	A02668	466	A00080	A02751	201
A00080	A02756	420	A00080	A02759	386
A00080	A02772	454	A00080	A02788	174
A00080	A02841	185	A00080	A02961	230
A00080	A03053	414	A00080	A03054	174
A00080	A03063	406	A00080	A03099	485
A00080	A03107	218	A00080	A03186	347
A00080	A03310	429	A00080	A03314	353
A00080	A03408	160	A00080	A03540	276
A00080	A03578	138	A00080	A03584	156
A00080	A03639	480	A00080	A03914	165
A00080	A03915	445	A00080	A03919	451
A00080	A03934	293	A00080	A03968	452
A00080	A04086	329	A00080	A04092	433
A00080	A04125	258	A00080	A04163	489
A00080	A04256	299	A00080	A04258	196
A00080	A04392	335	A00080	A04422	179
A00080	A04423	235	A00080	A04437	218
A00080	A04482	279	A00080	A04510	224
A00080	A04562	188	A00080	A04600	306
A00080	A04652	176	A00080	A04673	335
A00080	A04976	385	A00080	A05117	260
A00080	A05208	353	A00080	A05459	279
A00080	A05533	157	A00080	A05751	193

A00080	A05934	303	A00080	A06055	168
A00080	A06762	196	A00080	A06831	279
A00080	A06922	317	A00080	A07143	377
A00080	A08655	229	A00080	A08905	204
A00080	A09217	326	A00080	A09236	232
A00080	A09429	363	A00080	A09480	434
A00080	A09559	293	A00080	A10044	317
A00080	A10045	317	A00080	A10046	418
A00080	A10258	293	A00080	A10306	350
A00080	A10650	326	A00080	A11064	380
A00080	A11123	258	A00080	A13909	350
A00080	A14405	268	A00080	A14430	257
A00080	A14438	235	A00080	A15230	162
A00080	A15233	221	A00080	A15809	198
A00080	A15930	191	A00080	A16257	168
A00080	A16636	463	A00080	A16802	168
A00081	A00083	63	A00081	A00087	184
A00081	A00089	435	A00081	A00090	407
A00081	A00095	316	A00081	A00106	63
A00081	A00111	470	A00081	A00112	391
A00081	A00122	216	A00081	A00124	489
A00081	A00131	477	A00081	A00133	899
A00081	A00152	440	A00081	A00159	906
A00081	A00161	662	A00081	A00166	232
A00081	A00167	491	A00081	A00168	246
A00081	A00173	228	A00081	A00184	180
A00081	A00185	432	A00081	A00190	943
A00081	A00193	201	A00081	A00196	491
A00081	A00204	369	A00081	A00208	406
A00081	A00209	899	A00081	A00210	489
A00081	A00212	201	A00081	A00218	488
A00081	A00219	491	A00081	A00221	407
A00081	A00227	485	A00081	A00230	899
A00081	A00232	418	A00081	A00234	247
A00081	A00246	989	A00081	A00254	452
A00081	A00264	363	A00081	A00270	480
A00081	A00292	307	A00081	A00294	213
A00081	A00304	251	A00081	A00312	286
A00081	A00315	486	A00081	A00316	489
A00081	A00338	296	A00081	A00352	490
A00081	A00370	345	A00081	A00377	491
A00081	A00378	299	A00081	A00400	332
A00081	A00418	432	A00081	A00421	488
A00081	A00437	490	A00081	A00438	488
A00081	A00446	788	A00081	A00451	256
A00081	A00457	383	A00081	A00473	241

A00081	A00506	183	A00081	A00531	490
A00081	A00537	279	A00081	A00548	316
A00081	A00553	302	A00081	A00556	483
A00081	A00558	430	A00081	A00568	180
A00081	A00572	326	A00081	A00600	267
A00081	A00601	454	A00081	A00616	228
A00081	A00641	322	A00081	A00643	486
A00081	A00653	408	A00081	A00656	238
A00081	A00671	167	A00081	A00674	545
A00081	A00680	243	A00081	A00681	238
A00081	A00691	314	A00081	A00697	485
A00081	A00714	160	A00081	A00735	335
A00081	A00736	269	A00081	A00751	254
A00081	A00761	212	A00081	A00766	519
A00081	A00781	474	A00081	A00791	542
A00081	A00801	425	A00081	A00805	486
A00081	A00813	329	A00081	A00814	212
A00081	A00815	945	A00081	A00821	218
A00081	A00868	621	A00081	A00871	176
A00081	A00883	545	A00081	A00884	185
A00081	A00914	240	A00081	A00926	544
A00081	A00927	160	A00081	A00930	445
A00081	A00975	544	A00081	A00989	491
A00081	A01009	945	A00081	A01039	160
A00081	A01058	184	A00081	A01077	483
A00081	A01080	434	A00081	A01088	247
A00081	A01091	430	A00081	A01097	408
A00081	A01117	218	A00081	A01122	484
A00081	A01124	477	A00081	A01137	251
A00081	A01156	491	A00081	A01161	524
A00081	A01164	411	A00081	A01169	491
A00081	A01212	491	A00081	A01235	291
A00081	A01257	462	A00081	A01267	169
A00081	A01277	489	A00081	A01278	697
A00081	A01281	491	A00081	A01317	208
A00081	A01322	172	A00081	A01327	484
A00081	A01329	486	A00081	A01331	470
A00081	A01339	160	A00081	A01342	544
A00081	A01359	419	A00081	A01363	368
A00081	A01370	452	A00081	A01385	435
A00081	A01404	276	A00081	A01417	232
A00081	A01476	68	A00081	A01477	436
A00081	A01485	193	A00081	A01507	252
A00081	A01512	899	A00081	A01524	899
A00081	A01527	899	A00081	A01536	221
A00081	A01552	470	A00081	A01558	899

A00081	A01582	486	A00081	A01588	193
A00081	A01593	205	A00081	A01598	324
A00081	A01602	310	A00081	A01605	196
A00081	A01615	899	A00081	A01640	159
A00081	A01661	486	A00081	A01722	63
A00081	A01726	491	A00081	A01741	454
A00081	A01796	201	A00081	A01861	156
A00081	A01941	157	A00081	A01985	185
A00081	A02044	353	A00081	A02048	174
A00081	A02054	354	A00081	A02062	137
A00081	A02104	87	A00081	A02112	63
A00081	A02125	163	A00081	A02126	472
A00081	A02143	945	A00081	A02148	343
A00081	A02156	201	A00081	A02212	160
A00081	A02215	374	A00081	A02223	443
A00081	A02238	259	A00081	A02251	480
A00081	A02260	63	A00081	A02268	375
A00081	A02272	63	A00081	A02278	465
A00081	A02282	168	A00081	A02313	63
A00081	A02361	420	A00081	A02410	74
A00081	A02423	346	A00081	A02425	490
A00081	A02429	177	A00081	A02431	63
A00081	A02454	234	A00081	A02464	63
A00081	A02465	226	A00081	A02472	63
A00081	A02481	205	A00081	A02847	324
A00081	A03043	330	A00081	A03044	241
A00081	A03046	452	A00081	A03047	466
A00081	A03053	358	A00081	A03054	352
A00081	A03061	351	A00081	A03082	283
A00081	A03091	124	A00081	A03110	481
A00081	A03115	277	A00081	A03126	63
A00081	A03152	159	A00081	A03163	174
A00081	A03175	457	A00081	A03177	63
A00081	A03185	87	A00081	A03186	177
A00081	A03188	405	A00081	A03209	258
A00081	A03213	235	A00081	A03219	63
A00081	A03223	255	A00081	A03271	393
A00081	A03305	63	A00081	A03340	163
A00081	A03379	204	A00081	A03402	180
A00081	A03458	463	A00081	A03462	223
A00081	A03497	328	A00081	A03498	481
A00081	A03503	485	A00081	A03633	71
A00081	A03639	187	A00081	A03676	441
A00081	A03749	491	A00081	A03750	491
A00081	A03880	462	A00081	A03902	296
A00081	A03942	413	A00081	A03960	176

A00081	A04033	424	A00081	A04050	899
A00081	A04147	288	A00081	A04180	462
A00081	A04216	152	A00081	A04379	380
A00081	A04449	234	A00081	A04562	160
A00081	A04666	283	A00081	A04853	165
A00081	A04962	378	A00081	A05158	243
A00081	A05249	449	A00081	A05377	266
A00081	A05418	489	A00081	A05457	254
A00081	A05465	185	A00081	A05497	369
A00081	A05546	307	A00081	A05577	369
A00081	A05675	185	A00081	A05784	223
A00081	A06028	223	A00081	A06032	174
A00081	A06214	223	A00081	A06261	163
A00081	A06272	488	A00081	A06286	241
A00081	A06513	223	A00081	A06581	210
A00081	A06588	184	A00081	A06642	347
A00081	A06650	389	A00081	A06750	369
A00081	A06822	163	A00081	A06907	247
A00081	A06914	491	A00081	A07034	185
A00081	A07062	252	A00081	A07096	160
A00081	A07226	160	A00081	A07311	180
A00081	A07315	311	A00081	A07422	904
A00081	A07433	899	A00081	A07575	427
A00081	A07621	193	A00081	A07726	473
A00081	A07756	255	A00081	A07803	316
A00081	A07813	218	A00081	A07902	190
A00081	A07920	159	A00081	A07961	185
A00081	A08032	188	A00081	A08123	185
A00081	A08182	357	A00081	A08608	167
A00081	A08612	119	A00081	A08701	356
A00081	A08834	160	A00081	A08875	226
A00081	A08882	303	A00081	A09062	188
A00081	A09078	412	A00081	A09203	163
A00081	A09387	272	A00081	A09627	160
A00081	A09644	232	A00081	A09801	87
A00081	A09807	185	A00081	A09811	185
A00081	A09999	65	A00081	A10021	406
A00081	A10037	185	A00081	A10161	163
A00081	A10372	160	A00081	A10453	491
A00081	A10491	163	A00081	A10539	163
A00081	A10632	469	A00081	A11060	185
A00081	A11270	223	A00081	A11365	160
A00081	A11598	236	A00081	A11649	460
A00081	A11735	160	A00081	A11737	160
A00081	A11747	266	A00081	A11914	160
A00081	A12319	243	A00081	A12325	168

A00081	A12395	223	A00081	A12658	160
A00081	A12824	545	A00081	A12900	163
A00081	A12994	223	A00081	A13084	213
A00081	A13248	163	A00081	A13268	223
A00081	A13290	314	A00081	A13447	314
A00081	A13492	223	A00081	A13511	358
A00081	A13613	223	A00081	A13829	174
A00081	A13831	362	A00081	A14007	541
A00081	A14020	185	A00081	A14080	303
A00081	A14154	234	A00081	A14180	391
A00081	A14190	490	A00081	A14193	462
A00081	A14196	536	A00081	A14210	480
A00081	A14345	319	A00081	A14366	199
A00081	A14369	318	A00081	A14371	473
A00081	A14379	190	A00081	A14410	433
A00081	A14430	450	A00081	A14443	899
A00081	A14511	470	A00081	A14570	205
A00081	A14636	427	A00081	A15136	279
A00081	A15257	307	A00081	A15292	358
A00081	A15295	501	A00081	A15297	602
A00081	A15318	332	A00081	A15324	220
A00081	A15338	246	A00081	A15340	899
A00081	A15349	265	A00081	A15350	899
A00081	A15356	491	A00081	A15379	217
A00081	A15427	154	A00081	A15498	596
A00081	A15556	462	A00081	A15559	393
A00081	A15624	395	A00081	A15644	529
A00081	A15683	416	A00081	A15788	174
A00081	A15795	154	A00081	A15809	256
A00081	A15858	172	A00081	A15896	305
A00081	A16636	340	A00081	A16782	279
A00081	A17215	899	A00081	A17216	899
A00082	A00126	467	A00082	A00151	268
A00082	A00214	435	A00082	A00224	941
A00082	A00226	488	A00082	A00246	912
A00082	A00257	451	A00082	A00439	152
A00082	A00825	162	A00082	A00933	483
A00082	A01307	406	A00082	A01553	916
A00082	A01558	899	A00082	A01588	899
A00082	A01762	269	A00082	A01951	338
A00082	A02018	229	A00082	A02094	290
A00082	A03739	291	A00082	A04115	163
A00082	A04783	190	A00082	A05064	406
A00082	A05284	207	A00082	A06597	485
A00082	A06608	177	A00082	A06735	240
A00082	A07155	290	A00082	A09417	294

A00082	A10506	470	A00082	A11850	271
A00082	A12886	269	A00082	A14996	180
A00082	A15291	345	A00082	A16331	267
A00083	A00085	488	A00083	A00087	132
A00083	A00106	176	A00083	A00111	379
A00083	A00123	254	A00083	A00159	899
A00083	A00168	63	A00083	A00196	428
A00083	A00204	363	A00083	A00209	919
A00083	A00218	151	A00083	A00219	475
A00083	A00245	480	A00083	A00264	945
A00083	A00294	185	A00083	A00616	486
A00083	A00815	110	A00083	A01257	307
A00083	A01527	899	A00083	A01734	185
A00083	A01775	902	A00083	A01985	255
A00083	A02028	179	A00083	A02070	367
A00083	A02108	452	A00083	A02176	274
A00083	A02215	481	A00083	A02251	200
A00083	A02298	361	A00083	A02303	305
A00083	A02309	251	A00083	A02310	251
A00083	A02311	238	A00083	A02313	450
A00083	A02383	262	A00083	A02397	367
A00083	A02423	224	A00083	A02425	156
A00083	A02465	402	A00083	A02490	443
A00083	A02582	201	A00083	A02615	271
A00083	A02668	407	A00083	A02672	293
A00083	A02756	446	A00083	A02785	326
A00083	A02944	162	A00083	A02983	228
A00083	A03107	152	A00083	A03219	193
A00083	A03282	241	A00083	A03402	151
A00083	A03639	491	A00083	A03818	180
A00083	A03915	332	A00083	A03961	470
A00083	A04147	224	A00083	A04237	335
A00083	A04365	335	A00083	A04914	230
A00083	A04976	411	A00083	A05288	173
A00083	A05494	207	A00083	A05533	490
A00083	A05534	346	A00083	A05535	425
A00083	A05923	272	A00083	A05992	375
A00083	A06033	367	A00083	A06762	457
A00083	A07141	272	A00083	A09213	299
A00083	A09480	305	A00083	A09550	477
A00083	A13879	199	A00083	A14511	173
A00083	A15230	216	A00083	A16636	340
A00084	A00089	943	A00084	A00274	196
A00084	A02431	302	A00084	A03110	235
A00084	A07803	171	A00084	A08859	151
A00084	A09732	329	A00084	A09733	251

A00084	A11412	374	A00084	A14369	329
A00084	A15292	229	A00084	A15348	247
A00084	A15349	167	A00084	A15452	234
A00084	A15515	291	A00084	A15876	369
A00085	A00087	154	A00085	A00106	215
A00085	A00109	224	A00085	A00111	490
A00085	A00112	406	A00085	A00153	213
A00085	A00154	899	A00085	A00167	171
A00085	A00168	472	A00085	A00173	391
A00085	A00196	479	A00085	A00209	899
A00085	A00212	481	A00085	A00219	491
A00085	A00221	191	A00085	A00245	401
A00085	A00254	217	A00085	A00264	491
A00085	A00350	486	A00085	A00455	157
A00085	A00476	157	A00085	A00616	491
A00085	A00730	159	A00085	A00752	452
A00085	A00815	462	A00085	A00820	302
A00085	A00914	188	A00085	A01124	247
A00085	A01137	489	A00085	A01138	168
A00085	A01142	333	A00085	A01163	413
A00085	A01257	490	A00085	A01277	139
A00085	A01425	63	A00085	A01485	366
A00085	A01527	899	A00085	A01567	228
A00085	A01590	223	A00085	A01605	156
A00085	A01766	213	A00085	A01797	385
A00085	A01903	230	A00085	A01946	199
A00085	A01983	472	A00085	A02083	350
A00085	A02107	235	A00085	A02111	465
A00085	A02132	288	A00085	A02134	288
A00085	A02138	443	A00085	A02142	443
A00085	A02143	74	A00085	A02146	297
A00085	A02148	452	A00085	A02245	312
A00085	A02246	306	A00085	A02268	179
A00085	A02278	198	A00085	A02293	205
A00085	A02298	462	A00085	A02299	256
A00085	A02300	191	A00085	A02306	486
A00085	A02309	484	A00085	A02313	481
A00085	A02314	288	A00085	A02317	330
A00085	A02324	270	A00085	A02326	483
A00085	A02405	351	A00085	A02423	366
A00085	A02425	479	A00085	A02454	407
A00085	A02461	223	A00085	A02465	240
A00085	A02470	201	A00085	A02490	455
A00085	A02491	383	A00085	A02505	246
A00085	A02604	265	A00085	A02620	363
A00085	A02644	474	A00085	A02668	468

A00085	A02691	154	A00085	A02756	480
A00085	A02757	356	A00085	A02759	418
A00085	A02760	184	A00085	A02772	446
A00085	A02785	405	A00085	A02787	168
A00085	A02788	235	A00085	A02789	180
A00085	A02803	302	A00085	A02811	177
A00085	A02918	217	A00085	A02945	329
A00085	A02952	207	A00085	A03053	322
A00085	A03099	441	A00085	A03129	256
A00085	A03184	210	A00085	A03186	413
A00085	A03212	157	A00085	A03310	418
A00085	A03314	451	A00085	A03484	154
A00085	A03550	205	A00085	A03633	223
A00085	A03639	486	A00085	A03788	165
A00085	A03799	198	A00085	A03817	195
A00085	A03818	428	A00085	A03887	160
A00085	A03915	385	A00085	A03919	332
A00085	A04007	170	A00085	A04086	388
A00085	A04092	463	A00085	A04157	218
A00085	A04163	285	A00085	A04256	477
A00085	A04326	276	A00085	A04391	235
A00085	A04422	152	A00085	A04482	472
A00085	A04484	260	A00085	A04487	299
A00085	A04679	232	A00085	A04746	188
A00085	A04914	151	A00085	A04975	245
A00085	A04976	368	A00085	A04981	218
A00085	A05117	235	A00085	A05138	185
A00085	A05208	406	A00085	A05709	213
A00085	A05768	182	A00085	A05815	260
A00085	A05825	256	A00085	A05826	270
A00085	A05934	281	A00085	A05985	419
A00085	A06033	318	A00085	A06091	160
A00085	A06588	188	A00085	A06818	210
A00085	A06944	167	A00085	A07037	224
A00085	A07143	441	A00085	A07154	185
A00085	A07460	306	A00085	A07757	160
A00085	A08749	356	A00085	A08905	310
A00085	A09026	219	A00085	A09480	423
A00085	A09570	363	A00085	A10040	238
A00085	A10103	190	A00085	A10609	348
A00085	A10650	306	A00085	A10801	157
A00085	A11064	238	A00085	A11123	232
A00085	A11485	163	A00085	A12667	171
A00085	A13222	165	A00085	A13370	432
A00085	A13872	152	A00085	A13909	333
A00085	A14027	288	A00085	A14194	294

A00085	A14198	324	A00085	A14220	162
A00085	A14392	188	A00085	A14402	177
A00085	A14432	218	A00085	A14438	208
A00085	A15230	265	A00085	A15426	318
A00085	A15607	205	A00085	A15637	154
A00085	A15966	270	A00085	A16636	474
A00086	A00090	931	A00086	A00109	913
A00086	A00152	435	A00086	A00159	899
A00086	A00190	479	A00086	A00196	413
A00086	A00204	945	A00086	A00209	899
A00086	A00218	212	A00086	A00219	285
A00086	A00233	399	A00086	A00245	157
A00086	A00264	491	A00086	A00556	468
A00086	A00627	226	A00086	A00736	238
A00086	A00820	160	A00086	A00914	467
A00086	A00939	899	A00086	A01527	899
A00086	A01551	196	A00086	A01588	899
A00086	A01604	180	A00086	A01615	899
A00086	A01626	191	A00086	A01654	424
A00086	A01668	391	A00086	A01704	411
A00086	A01726	160	A00086	A01733	416
A00086	A01741	475	A00086	A01743	404
A00086	A01744	154	A00086	A01745	247
A00086	A01752	353	A00086	A01775	484
A00086	A01784	195	A00086	A01785	347
A00086	A01873	274	A00086	A01875	296
A00086	A01879	163	A00086	A01902	210
A00086	A01907	281	A00086	A01931	152
A00086	A01936	152	A00086	A02080	162
A00086	A02145	168	A00086	A02177	79
A00086	A02298	484	A00086	A02300	255
A00086	A02307	226	A00086	A02337	157
A00086	A02347	396	A00086	A02385	183
A00086	A02411	899	A00086	A02412	171
A00086	A02439	162	A00086	A02490	480
A00086	A02491	424	A00086	A02582	424
A00086	A02605	272	A00086	A02615	167
A00086	A02646	188	A00086	A02668	477
A00086	A02674	235	A00086	A02695	486
A00086	A02696	404	A00086	A02716	257
A00086	A02729	374	A00086	A02745	207
A00086	A02756	439	A00086	A02764	205
A00086	A02783	389	A00086	A02785	165
A00086	A02787	229	A00086	A02812	945
A00086	A02813	334	A00086	A02830	272
A00086	A02837	407	A00086	A02843	208

A00086	A02859	945	A00086	A02863	313
A00086	A02866	238	A00086	A02874	491
A00086	A02883	193	A00086	A02884	339
A00086	A02896	486	A00086	A02898	141
A00086	A02905	162	A00086	A02921	238
A00086	A02922	491	A00086	A02923	382
A00086	A02932	265	A00086	A02936	172
A00086	A02944	449	A00086	A02951	945
A00086	A02953	260	A00086	A02961	340
A00086	A02963	362	A00086	A02966	152
A00086	A02974	350	A00086	A02977	252
A00086	A02982	299	A00086	A02984	154
A00086	A03112	180	A00086	A03129	229
A00086	A03204	269	A00086	A03275	945
A00086	A03281	257	A00086	A03282	358
A00086	A03415	488	A00086	A03419	195
A00086	A03426	221	A00086	A03475	396
A00086	A03664	366	A00086	A03746	165
A00086	A03799	160	A00086	A03865	174
A00086	A03914	396	A00086	A03927	176
A00086	A04010	490	A00086	A04011	179
A00086	A04040	177	A00086	A04051	945
A00086	A04052	195	A00086	A04088	171
A00086	A04138	299	A00086	A04142	235
A00086	A04143	368	A00086	A04240	283
A00086	A04244	386	A00086	A04262	257
A00086	A04267	193	A00086	A04278	430
A00086	A04288	416	A00086	A04332	238
A00086	A04391	409	A00086	A04449	899
A00086	A04494	200	A00086	A04520	279
A00086	A04526	257	A00086	A04548	252
A00086	A04567	263	A00086	A04589	176
A00086	A04598	218	A00086	A04607	933
A00086	A04608	945	A00086	A04609	919
A00086	A04614	408	A00086	A04622	154
A00086	A04652	483	A00086	A04727	200
A00086	A04735	251	A00086	A04745	257
A00086	A04851	218	A00086	A04906	174
A00086	A04941	157	A00086	A04961	193
A00086	A05071	274	A00086	A05113	945
A00086	A05202	202	A00086	A05309	491
A00086	A05314	283	A00086	A05315	899
A00086	A05349	945	A00086	A05506	195
A00086	A05861	347	A00086	A05878	154
A00086	A05879	369	A00086	A05920	257
A00086	A05931	274	A00086	A05985	165

A00086	A06055	238	A00086	A06073	329
A00086	A06149	279	A00086	A06286	213
A00086	A06465	296	A00086	A06642	219
A00086	A06682	296	A00086	A06793	198
A00086	A06980	296	A00086	A07025	283
A00086	A07141	374	A00086	A07147	179
A00086	A07148	177	A00086	A07157	218
A00086	A07164	268	A00086	A07165	324
A00086	A07173	281	A00086	A07191	160
A00086	A07200	193	A00086	A08420	283
A00086	A08603	183	A00086	A08627	474
A00086	A08657	313	A00086	A08677	171
A00086	A08755	274	A00086	A08827	165
A00086	A08829	313	A00086	A09319	899
A00086	A09381	167	A00086	A10173	193
A00086	A10791	160	A00086	A11476	216
A00086	A12897	447	A00086	A14208	899
A00086	A14236	165	A00086	A14393	899
A00086	A14440	173	A00086	A14658	265
A00086	A14735	185	A00086	A15314	900
A00086	A15607	177	A00086	A15981	172
A00086	A16636	484	A00086	A16965	238
A00087	A00089	943	A00087	A00095	384
A00087	A00111	389	A00087	A00127	310
A00087	A00168	475	A00087	A00190	393
A00087	A00219	486	A00087	A00221	188
A00087	A00245	945	A00087	A00274	441
A00087	A00315	380	A00087	A00616	286
A00087	A00931	185	A00087	A01137	63
A00087	A01157	247	A00087	A01173	443
A00087	A01235	252	A00087	A01257	491
A00087	A01485	185	A00087	A01524	899
A00087	A01547	170	A00087	A01610	371
A00087	A01616	190	A00087	A01668	416
A00087	A01817	221	A00087	A02174	74
A00087	A02213	212	A00087	A02236	163
A00087	A02306	63	A00087	A02425	290
A00087	A02429	386	A00087	A02440	330
A00087	A02498	385	A00087	A02544	352
A00087	A02550	249	A00087	A02639	191
A00087	A02673	198	A00087	A02859	356
A00087	A02959	151	A00087	A03056	210
A00087	A03110	246	A00087	A03115	195
A00087	A03259	466	A00087	A03314	322
A00087	A03466	279	A00087	A03639	449
A00087	A03748	234	A00087	A03800	463

A00087	A03818	450	A00087	A03910	236
A00087	A03942	380	A00087	A03961	490
A00087	A04169	323	A00087	A04256	267
A00087	A04482	254	A00087	A04616	200
A00087	A05052	247	A00087	A05184	184
A00087	A05270	316	A00087	A05404	353
A00087	A05546	480	A00087	A05736	216
A00087	A05881	213	A00087	A05992	176
A00087	A06762	167	A00087	A07019	291
A00087	A07021	303	A00087	A07051	317
A00087	A07296	274	A00087	A07297	279
A00087	A07315	154	A00087	A07559	272
A00087	A08730	446	A00087	A08859	180
A00087	A09062	455	A00087	A09074	254
A00087	A09211	313	A00087	A09394	198
A00087	A09729	176	A00087	A09732	480
A00087	A09733	472	A00087	A09734	198
A00087	A09955	372	A00087	A10665	240
A00087	A11014	407	A00087	A11412	402
A00087	A11537	274	A00087	A11868	449
A00087	A11875	154	A00087	A12108	286
A00087	A12478	294	A00087	A13861	303
A00087	A13942	366	A00087	A13944	182
A00087	A13987	294	A00087	A14072	283
A00087	A14139	240	A00087	A14190	185
A00087	A14210	367	A00087	A14364	399
A00087	A14371	345	A00087	A14378	405
A00087	A14716	486	A00087	A15292	484
A00087	A15310	251	A00087	A15313	185
A00087	A15317	363	A00087	A15327	163
A00087	A15348	462	A00087	A15349	469
A00087	A15352	367	A00087	A15376	486
A00087	A15432	278	A00087	A15440	168
A00087	A15456	165	A00087	A15459	165
A00087	A15515	229	A00087	A15641	160
A00087	A15671	221	A00087	A15799	176
A00087	A16300	399	A00087	A16323	208
A00087	A17117	279	A00088	A00196	489
A00088	A00472	193	A00088	A00725	205
A00088	A01556	200	A00088	A02000	274
A00088	A02148	463	A00088	A03141	408
A00088	A03186	414	A00088	A03379	223
A00088	A03651	210	A00088	A03906	475
A00088	A05891	281	A00088	A13816	262
A00088	A14838	229	A00089	A00095	184
A00089	A00122	291	A00089	A00127	221

A00089	A00150	210	A00089	A00152	489
A00089	A00167	491	A00089	A00168	483
A00089	A00179	286	A00089	A00190	491
A00089	A00209	899	A00089	A00210	63
A00089	A00219	491	A00089	A00221	252
A00089	A00233	180	A00089	A00245	945
A00089	A00274	489	A00089	A00315	195
A00089	A00382	402	A00089	A00531	489
A00089	A00722	162	A00089	A00752	333
A00089	A00815	488	A00089	A00916	332
A00089	A00930	497	A00089	A00989	63
A00089	A01009	63	A00089	A01091	229
A00089	A01124	63	A00089	A01125	286
A00089	A01235	491	A00089	A01257	486
A00089	A01354	486	A00089	A01359	400
A00089	A01539	472	A00089	A01593	899
A00089	A01610	363	A00089	A01654	249
A00089	A01659	247	A00089	A01668	422
A00089	A01676	183	A00089	A01741	297
A00089	A01763	173	A00089	A01790	424
A00089	A01822	226	A00089	A01936	218
A00089	A02006	165	A00089	A02052	157
A00089	A02138	246	A00089	A02139	123
A00089	A02143	63	A00089	A02144	177
A00089	A02236	374	A00089	A02251	235
A00089	A02253	205	A00089	A02260	226
A00089	A02288	160	A00089	A02425	246
A00089	A02429	310	A00089	A02431	491
A00089	A02433	160	A00089	A02454	63
A00089	A02464	73	A00089	A02467	347
A00089	A02471	157	A00089	A02472	63
A00089	A02839	259	A00089	A02859	465
A00089	A02959	491	A00089	A03104	480
A00089	A03110	491	A00089	A03129	308
A00089	A03136	288	A00089	A03157	221
A00089	A03183	422	A00089	A03209	321
A00089	A03219	223	A00089	A03259	491
A00089	A03275	174	A00089	A03305	91
A00089	A03580	185	A00089	A03639	446
A00089	A03748	249	A00089	A03788	213
A00089	A03799	485	A00089	A03818	202
A00089	A03851	417	A00089	A03887	281
A00089	A03910	446	A00089	A03925	351
A00089	A03942	945	A00089	A03961	404
A00089	A03963	160	A00089	A04033	288
A00089	A04076	440	A00089	A04166	205

A00089	A04169	491	A00089	A04253	185
A00089	A04305	152	A00089	A04361	216
A00089	A04380	434	A00089	A04449	452
A00089	A04472	191	A00089	A04490	176
A00089	A04881	490	A00089	A04976	162
A00089	A05093	462	A00089	A05157	491
A00089	A05184	226	A00089	A05221	291
A00089	A05229	205	A00089	A05270	249
A00089	A05339	185	A00089	A05439	420
A00089	A05786	185	A00089	A05944	307
A00089	A06207	213	A00089	A06494	223
A00089	A06569	179	A00089	A06588	290
A00089	A06762	202	A00089	A06997	274
A00089	A07021	234	A00089	A07209	491
A00089	A07229	177	A00089	A07296	198
A00089	A07297	205	A00089	A07311	899
A00089	A07803	491	A00089	A07912	185
A00089	A08465	313	A00089	A08561	191
A00089	A08600	249	A00089	A08630	491
A00089	A08641	218	A00089	A08707	605
A00089	A08714	174	A00089	A08814	157
A00089	A08834	445	A00089	A08840	296
A00089	A08859	791	A00089	A08875	182
A00089	A09022	469	A00089	A09062	296
A00089	A09208	324	A00089	A09238	481
A00089	A09239	490	A00089	A09245	425
A00089	A09387	147	A00089	A09422	477
A00089	A09550	167	A00089	A09559	191
A00089	A09570	157	A00089	A09609	160
A00089	A09732	491	A00089	A09733	491
A00089	A09734	385	A00089	A09742	208
A00089	A09828	173	A00089	A09843	408
A00089	A09855	177	A00089	A09875	180
A00089	A09970	249	A00089	A09984	223
A00089	A10164	489	A00089	A10167	180
A00089	A10202	384	A00089	A10336	240
A00089	A10579	260	A00089	A10580	906
A00089	A10619	177	A00089	A10723	217
A00089	A10858	234	A00089	A10985	234
A00089	A11144	154	A00089	A11412	491
A00089	A11415	491	A00089	A11471	193
A00089	A11537	352	A00089	A11542	165
A00089	A11616	168	A00089	A11735	257
A00089	A11737	174	A00089	A11875	467
A00089	A11914	168	A00089	A11946	223
A00089	A11983	374	A00089	A12254	182

A00089	A12393	491	A00089	A12826	269
A00089	A12850	162	A00089	A12854	332
A00089	A12858	329	A00089	A13003	321
A00089	A13119	474	A00089	A13151	219
A00089	A13169	183	A00089	A13546	491
A00089	A13587	351	A00089	A13826	469
A00089	A13860	316	A00089	A13899	408
A00089	A13944	223	A00089	A13973	205
A00089	A13987	488	A00089	A13989	283
A00089	A14000	457	A00089	A14010	263
A00089	A14072	447	A00089	A14111	265
A00089	A14122	474	A00089	A14129	473
A00089	A14133	263	A00089	A14190	490
A00089	A14202	491	A00089	A14210	491
A00089	A14221	201	A00089	A14229	286
A00089	A14251	171	A00089	A14270	198
A00089	A14364	313	A00089	A14369	475
A00089	A14370	473	A00089	A14371	491
A00089	A14379	460	A00089	A14399	345
A00089	A14490	382	A00089	A14511	491
A00089	A14523	258	A00089	A14536	160
A00089	A14539	290	A00089	A14569	152
A00089	A14570	486	A00089	A14579	232
A00089	A14635	477	A00089	A14667	486
A00089	A14733	238	A00089	A14739	205
A00089	A14930	491	A00089	A15184	249
A00089	A15230	259	A00089	A15233	335
A00089	A15260	234	A00089	A15277	427
A00089	A15292	491	A00089	A15310	385
A00089	A15312	489	A00089	A15313	489
A00089	A15314	489	A00089	A15317	491
A00089	A15318	425	A00089	A15322	486
A00089	A15331	228	A00089	A15335	173
A00089	A15346	171	A00089	A15348	489
A00089	A15349	491	A00089	A15352	491
A00089	A15366	456	A00089	A15396	355
A00089	A15400	196	A00089	A15402	368
A00089	A15404	360	A00089	A15413	176
A00089	A15419	247	A00089	A15431	297
A00089	A15435	473	A00089	A15436	208
A00089	A15444	436	A00089	A15445	461
A00089	A15447	458	A00089	A15452	491
A00089	A15454	347	A00089	A15456	303
A00089	A15478	154	A00089	A15480	488
A00089	A15495	491	A00089	A15498	207
A00089	A15515	463	A00089	A15630	409

A00089	A15638	165	A00089	A15684	322
A00089	A15711	193	A00089	A15799	385
A00089	A15804	151	A00089	A15819	168
A00089	A15828	226	A00089	A15850	198
A00089	A15886	191	A00089	A15916	245
A00089	A16157	226	A00089	A16177	418
A00089	A16187	174	A00089	A16192	167
A00089	A16260	205	A00089	A16565	215
A00089	A16585	157	A00089	A16587	324
A00089	A16691	167	A00089	A16695	185
A00089	A16711	303	A00089	A16788	249
A00089	A16815	174	A00089	A16904	567
A00089	A17081	191	A00089	A17120	269
A00089	A17124	249	A00090	A00091	79
A00090	A00098	101	A00090	A00109	917
A00090	A00113	63	A00090	A00138	491
A00090	A00145	463	A00090	A00152	451
A00090	A00156	63	A00090	A00159	945
A00090	A00171	899	A00090	A00182	463
A00090	A00183	488	A00090	A00185	481
A00090	A00204	943	A00090	A00207	432
A00090	A00213	63	A00090	A00214	491
A00090	A00219	151	A00090	A00220	215
A00090	A00230	899	A00090	A00232	190
A00090	A00234	491	A00090	A00254	490
A00090	A00267	174	A00090	A00274	198
A00090	A00316	418	A00090	A00418	369
A00090	A00433	230	A00090	A00600	196
A00090	A00641	662	A00090	A00696	200
A00090	A00697	488	A00090	A00939	491
A00090	A01164	420	A00090	A01203	208
A00090	A01212	469	A00090	A01286	299
A00090	A01307	491	A00090	A01419	160
A00090	A01423	160	A00090	A01428	429
A00090	A01520	63	A00090	A01521	63
A00090	A01527	993	A00090	A01553	297
A00090	A01558	899	A00090	A01560	456
A00090	A01562	168	A00090	A01603	366
A00090	A01604	456	A00090	A01605	189
A00090	A01612	178	A00090	A01615	899
A00090	A01628	174	A00090	A01639	899
A00090	A01654	65	A00090	A01692	305
A00090	A01718	255	A00090	A01719	66
A00090	A01850	200	A00090	A01908	318
A00090	A01914	470	A00090	A02043	221
A00090	A02147	157	A00090	A02251	249

A00090	A02419	184	A00090	A02425	265
A00090	A02604	195	A00090	A02610	258
A00090	A02611	241	A00090	A02637	241
A00090	A02651	112	A00090	A02659	477
A00090	A02740	277	A00090	A02798	170
A00090	A02801	307	A00090	A02803	491
A00090	A02816	168	A00090	A02847	80
A00090	A02851	361	A00090	A02853	221
A00090	A02854	344	A00090	A02859	226
A00090	A02887	490	A00090	A02896	340
A00090	A02916	63	A00090	A02917	259
A00090	A02920	144	A00090	A02924	362
A00090	A02931	490	A00090	A02944	391
A00090	A02945	132	A00090	A02950	396
A00090	A02961	187	A00090	A02972	93
A00090	A02975	256	A00090	A03014	285
A00090	A03019	216	A00090	A03028	243
A00090	A03109	238	A00090	A03183	157
A00090	A03195	413	A00090	A03271	491
A00090	A03275	263	A00090	A03282	96
A00090	A03428	205	A00090	A03523	391
A00090	A03568	265	A00090	A03569	355
A00090	A03625	63	A00090	A03666	190
A00090	A03686	63	A00090	A03817	277
A00090	A03830	373	A00090	A03834	254
A00090	A03835	208	A00090	A03984	489
A00090	A03985	218	A00090	A04010	201
A00090	A04012	530	A00090	A04031	157
A00090	A04042	232	A00090	A04050	491
A00090	A04115	213	A00090	A04244	251
A00090	A04251	258	A00090	A04300	307
A00090	A04450	180	A00090	A04494	154
A00090	A04536	363	A00090	A04587	358
A00090	A04657	257	A00090	A04682	216
A00090	A04730	232	A00090	A04731	218
A00090	A04755	157	A00090	A04854	243
A00090	A04860	221	A00090	A04864	333
A00090	A05211	333	A00090	A05212	371
A00090	A05331	165	A00090	A05333	207
A00090	A05349	899	A00090	A05475	277
A00090	A05606	302	A00090	A05633	484
A00090	A05902	165	A00090	A05904	258
A00090	A05946	258	A00090	A06028	307
A00090	A06224	409	A00090	A06237	232
A00090	A06289	63	A00090	A06567	221
A00090	A06610	484	A00090	A07135	163

A00090	A07148	260	A00090	A07157	358
A00090	A07161	466	A00090	A07166	221
A00090	A07167	172	A00090	A07171	243
A00090	A07173	406	A00090	A07207	63
A00090	A07311	483	A00090	A07422	899
A00090	A07807	207	A00090	A08345	391
A00090	A08608	486	A00090	A09060	259
A00090	A09145	329	A00090	A09263	414
A00090	A09279	99	A00090	A09392	162
A00090	A09395	241	A00090	A09441	165
A00090	A09488	277	A00090	A09549	358
A00090	A09581	343	A00090	A09754	188
A00090	A09937	110	A00090	A10214	98
A00090	A10288	232	A00090	A10562	277
A00090	A10735	212	A00090	A11129	622
A00090	A11307	441	A00090	A11308	162
A00090	A11369	274	A00090	A11929	317
A00090	A13198	310	A00090	A13437	258
A00090	A13774	277	A00090	A13824	152
A00090	A13872	195	A00090	A13978	900
A00090	A13983	63	A00090	A13994	160
A00090	A14196	252	A00090	A14208	491
A00090	A14364	384	A00090	A14367	118
A00090	A14369	307	A00090	A14400	269
A00090	A14426	224	A00090	A14431	224
A00090	A14439	177	A00090	A14540	207
A00090	A14830	484	A00090	A15314	491
A00090	A15337	258	A00090	A15361	310
A00090	A15386	85	A00090	A15510	223
A00090	A15603	219	A00090	A15609	213
A00090	A15630	265	A00090	A15780	469
A00090	A15781	277	A00090	A15782	243
A00090	A15832	221	A00090	A15834	265
A00090	A16216	384	A00090	A16430	240
A00090	A16461	213	A00090	A16594	391
A00091	A00113	578	A00091	A00156	712
A00091	A00184	216	A00091	A00185	480
A00091	A00204	63	A00091	A00207	565
A00091	A00208	177	A00091	A00221	217
A00091	A00230	899	A00091	A00234	228
A00091	A00254	470	A00091	A00291	269
A00091	A00333	240	A00091	A00451	488
A00091	A00616	226	A00091	A01286	316
A00091	A01307	63	A00091	A01553	908
A00091	A01558	899	A00091	A01615	899
A00091	A01645	201	A00091	A01908	243

A00091	A02419	452	A00091	A02425	162
A00091	A02511	196	A00091	A02636	218
A00091	A02783	213	A00091	A02787	147
A00091	A02788	252	A00091	A02803	115
A00091	A02874	429	A00091	A02875	446
A00091	A02887	107	A00091	A02961	167
A00091	A03054	232	A00091	A03271	104
A00091	A03282	247	A00091	A03683	411
A00091	A03684	388	A00091	A03830	477
A00091	A03974	199	A00091	A03975	296
A00091	A04012	273	A00091	A04037	329
A00091	A04422	238	A00091	A04451	196
A00091	A04578	317	A00091	A04600	232
A00091	A04639	249	A00091	A04864	335
A00091	A04965	174	A00091	A04972	293
A00091	A05111	332	A00091	A05679	171
A00091	A07056	297	A00091	A07148	395
A00091	A07167	223	A00091	A07311	899
A00091	A07860	362	A00091	A08070	176
A00091	A09902	218	A00091	A10898	408
A00091	A13605	257	A00091	A14004	232
A00091	A14163	174	A00091	A14190	215
A00091	A14198	252	A00091	A14210	190
A00091	A14364	443	A00091	A14404	274
A00091	A15834	216	A00092	A00095	322
A00092	A00163	265	A00092	A00200	408
A00092	A00212	475	A00092	A00216	269
A00092	A00219	347	A00092	A00301	154
A00092	A00316	462	A00092	A00348	318
A00092	A00351	491	A00092	A00370	157
A00092	A00474	201	A00092	A00482	195
A00092	A00509	196	A00092	A00568	184
A00092	A01161	305	A00092	A01249	205
A00092	A01475	291	A00092	A01485	411
A00092	A01551	274	A00092	A01582	213
A00092	A01629	173	A00092	A01656	364
A00092	A01733	160	A00092	A01755	305
A00092	A01767	485	A00092	A01772	207
A00092	A01857	251	A00092	A01876	176
A00092	A01880	180	A00092	A01905	491
A00092	A02074	173	A00092	A02075	157
A00092	A02111	490	A00092	A02181	276
A00092	A02216	168	A00092	A02306	213
A00092	A02317	252	A00092	A02320	346
A00092	A02330	262	A00092	A02338	224
A00092	A02427	213	A00092	A02436	176

A00092	A02468	190	A00092	A02691	329
A00092	A02701	199	A00092	A02738	491
A00092	A02759	198	A00092	A02830	208
A00092	A02924	151	A00092	A03099	316
A00092	A03213	306	A00092	A03314	157
A00092	A03511	296	A00092	A03904	228
A00092	A03919	254	A00092	A04077	337
A00092	A04078	213	A00092	A04092	407
A00092	A04258	352	A00092	A04289	473
A00092	A04321	473	A00092	A04349	172
A00092	A04374	432	A00092	A04460	296
A00092	A04510	252	A00092	A04569	424
A00092	A04580	462	A00092	A04683	319
A00092	A04840	445	A00092	A04870	360
A00092	A04965	274	A00092	A05106	171
A00092	A05123	455	A00092	A05172	432
A00092	A05288	483	A00092	A05351	276
A00092	A05381	344	A00092	A05865	243
A00092	A05944	165	A00092	A06293	260
A00092	A06346	335	A00092	A06608	344
A00092	A06711	268	A00092	A06891	260
A00092	A07200	152	A00092	A07384	232
A00092	A07705	323	A00092	A08671	462
A00092	A08746	188	A00092	A08961	272
A00092	A09123	488	A00092	A09283	314
A00092	A09364	314	A00092	A09440	364
A00092	A09927	383	A00092	A10013	462
A00092	A10049	440	A00092	A10140	311
A00092	A10203	364	A00092	A10417	183
A00092	A10619	205	A00092	A10744	396
A00092	A10825	460	A00092	A10827	438
A00092	A11318	255	A00092	A11388	438
A00092	A11709	353	A00092	A12387	383
A00092	A12898	353	A00092	A12963	383
A00092	A13173	474	A00092	A13820	432
A00092	A14255	168	A00092	A14377	278
A00092	A14430	193	A00092	A14443	177
A00092	A14444	902	A00092	A14886	232
A00092	A14918	224	A00092	A15258	252
A00092	A15277	188	A00092	A15398	241
A00092	A15989	344	A00092	A16764	167
A00093	A00116	217	A00093	A00133	899
A00093	A00240	419	A00093	A00242	152
A00093	A00246	899	A00093	A00336	241
A00093	A00399	254	A00093	A00446	228
A00093	A00487	249	A00093	A00562	224

A00093	A00648	177	A00093	A00709	232
A00093	A00725	221	A00093	A00808	401
A00093	A00897	208	A00093	A01064	283
A00093	A01070	899	A00093	A01133	171
A00093	A01369	227	A00093	A01512	909
A00093	A01553	937	A00093	A01554	433
A00093	A01560	424	A00093	A01680	491
A00093	A01708	899	A00093	A01914	210
A00093	A02667	183	A00093	A02791	199
A00093	A03244	162	A00093	A04650	357
A00093	A06564	243	A00093	A07311	933
A00093	A08255	177	A00093	A08418	179
A00093	A09220	168	A00093	A10601	479
A00093	A10808	193	A00093	A10982	323
A00093	A11559	218	A00093	A11658	227
A00093	A11850	151	A00093	A11929	185
A00093	A14030	283	A00093	A14073	213
A00093	A14256	63	A00093	A14490	159
A00093	A14546	238	A00093	A15188	167
A00093	A15291	490	A00093	A15293	899
A00093	A15294	899	A00093	A15501	262
A00093	A15612	171	A00093	A15614	334
A00093	A15649	205	A00093	A15680	338
A00093	A15722	245	A00093	A16293	190
A00093	A16467	461	A00094	A00095	899
A00094	A00098	413	A00094	A00116	167
A00094	A00129	340	A00094	A00133	944
A00094	A00135	361	A00094	A00136	168
A00094	A00138	486	A00094	A00140	899
A00094	A00142	899	A00094	A00143	899
A00094	A00146	316	A00094	A00149	462
A00094	A00152	356	A00094	A00157	899
A00094	A00159	906	A00094	A00163	958
A00094	A00165	981	A00094	A00167	490
A00094	A00168	154	A00094	A00169	944
A00094	A00171	490	A00094	A00173	491
A00094	A00179	256	A00094	A00183	456
A00094	A00185	346	A00094	A00187	481
A00094	A00188	491	A00094	A00190	490
A00094	A00200	474	A00094	A00201	396
A00094	A00209	945	A00094	A00212	899
A00094	A00215	899	A00094	A00219	455
A00094	A00224	484	A00094	A00230	205
A00094	A00233	306	A00094	A00234	489
A00094	A00239	458	A00094	A00240	679
A00094	A00246	899	A00094	A00248	491

A00094	A00254	945	A00094	A00256	491
A00094	A00257	583	A00094	A00259	449
A00094	A00261	429	A00094	A00262	486
A00094	A00264	490	A00094	A00265	480
A00094	A00266	263	A00094	A00272	529
A00094	A00274	193	A00094	A00309	450
A00094	A00324	491	A00094	A00327	400
A00094	A00328	238	A00094	A00400	234
A00094	A00476	187	A00094	A00548	389
A00094	A00616	441	A00094	A00630	306
A00094	A00656	490	A00094	A00679	401
A00094	A00693	221	A00094	A00696	465
A00094	A00736	388	A00094	A00738	152
A00094	A00743	252	A00094	A00773	488
A00094	A00791	462	A00094	A00822	481
A00094	A00868	461	A00094	A00887	474
A00094	A00922	491	A00094	A00926	466
A00094	A00981	418	A00094	A01021	329
A00094	A01052	466	A00094	A01064	191
A00094	A01070	160	A00094	A01102	425
A00094	A01125	174	A00094	A01178	232
A00094	A01225	230	A00094	A01329	446
A00094	A01364	457	A00094	A01378	384
A00094	A01452	462	A00094	A01475	221
A00094	A01507	944	A00094	A01512	945
A00094	A01524	903	A00094	A01553	930
A00094	A01554	943	A00094	A01560	368
A00094	A01571	396	A00094	A01589	393
A00094	A01604	391	A00094	A01606	485
A00094	A01615	899	A00094	A01626	236
A00094	A01639	994	A00094	A01653	240
A00094	A01668	201	A00094	A01669	348
A00094	A01688	475	A00094	A01692	235
A00094	A01698	460	A00094	A01729	469
A00094	A01741	477	A00094	A01742	490
A00094	A01746	491	A00094	A01756	432
A00094	A01763	232	A00094	A01775	174
A00094	A01776	357	A00094	A01797	165
A00094	A01850	429	A00094	A01894	193
A00094	A01908	391	A00094	A01910	195
A00094	A01932	168	A00094	A01983	352
A00094	A01995	185	A00094	A02180	160
A00094	A02183	481	A00094	A02235	190
A00094	A02298	340	A00094	A02343	185
A00094	A02383	413	A00094	A02420	210
A00094	A02467	358	A00094	A02490	429

A00094	A02498	229	A00094	A02502	899
A00094	A02505	475	A00094	A02581	182
A00094	A02639	224	A00094	A02668	156
A00094	A02670	361	A00094	A02695	324
A00094	A02699	486	A00094	A02715	224
A00094	A02724	195	A00094	A02756	351
A00094	A02789	477	A00094	A02821	434
A00094	A02834	254	A00094	A02859	265
A00094	A02866	195	A00094	A02871	205
A00094	A02873	416	A00094	A02909	449
A00094	A02963	449	A00094	A03004	176
A00094	A03007	240	A00094	A03014	417
A00094	A03095	491	A00094	A03172	307
A00094	A03181	187	A00094	A03218	477
A00094	A03244	449	A00094	A03268	160
A00094	A03304	314	A00094	A03312	344
A00094	A03420	229	A00094	A03422	170
A00094	A03451	288	A00094	A03500	195
A00094	A03520	210	A00094	A03541	488
A00094	A03598	160	A00094	A03612	475
A00094	A03632	224	A00094	A03653	221
A00094	A03662	945	A00094	A03726	922
A00094	A03741	971	A00094	A03778	483
A00094	A03822	400	A00094	A03826	385
A00094	A03832	491	A00094	A03846	361
A00094	A03861	257	A00094	A03869	243
A00094	A03871	259	A00094	A03917	296
A00094	A03921	180	A00094	A03922	234
A00094	A03930	168	A00094	A03970	168
A00094	A03989	460	A00094	A04020	213
A00094	A04049	441	A00094	A04071	180
A00094	A04089	156	A00094	A04248	152
A00094	A04249	156	A00094	A04275	462
A00094	A04310	182	A00094	A04334	422
A00094	A04391	152	A00094	A04445	279
A00094	A04449	583	A00094	A04481	188
A00094	A04513	168	A00094	A04536	491
A00094	A04577	168	A00094	A04603	440
A00094	A04654	205	A00094	A04699	911
A00094	A04728	305	A00094	A04732	195
A00094	A04858	185	A00094	A04865	152
A00094	A04911	200	A00094	A04922	434
A00094	A04996	447	A00094	A05012	205
A00094	A05073	221	A00094	A05216	177
A00094	A05288	758	A00094	A05312	168
A00094	A05398	235	A00094	A05407	165

A00094	A05418	480	A00094	A05489	358
A00094	A05556	485	A00094	A05755	205
A00094	A05771	210	A00094	A05805	168
A00094	A05963	205	A00094	A06055	489
A00094	A06254	386	A00094	A06295	170
A00094	A06362	205	A00094	A06394	205
A00094	A06550	207	A00094	A06581	393
A00094	A06583	205	A00094	A06588	424
A00094	A06597	491	A00094	A06601	388
A00094	A06606	227	A00094	A06607	491
A00094	A06628	379	A00094	A06650	274
A00094	A06703	182	A00094	A06760	230
A00094	A06801	168	A00094	A06877	450
A00094	A06986	205	A00094	A07047	326
A00094	A07166	216	A00094	A07201	208
A00094	A07339	488	A00094	A07390	424
A00094	A07422	974	A00094	A07695	180
A00094	A07834	380	A00094	A07955	168
A00094	A08274	152	A00094	A08527	224
A00094	A08658	182	A00094	A08692	299
A00094	A08850	433	A00094	A08878	941
A00094	A08902	171	A00094	A09016	205
A00094	A09018	276	A00094	A09046	361
A00094	A09162	486	A00094	A09492	168
A00094	A09509	171	A00094	A09573	339
A00094	A09607	491	A00094	A09804	480
A00094	A09814	152	A00094	A09976	201
A00094	A09986	205	A00094	A10001	191
A00094	A10166	205	A00094	A10173	229
A00094	A10174	899	A00094	A10207	205
A00094	A10263	205	A00094	A10368	205
A00094	A10417	212	A00094	A10424	160
A00094	A10501	188	A00094	A10506	180
A00094	A10573	168	A00094	A10580	899
A00094	A10623	945	A00094	A10728	486
A00094	A10745	168	A00094	A10808	945
A00094	A10811	899	A00094	A10914	205
A00094	A10982	491	A00094	A11184	199
A00094	A11335	180	A00094	A11513	249
A00094	A11559	474	A00094	A11673	160
A00094	A11805	205	A00094	A11943	486
A00094	A11983	954	A00094	A12357	185
A00094	A12373	463	A00094	A12514	168
A00094	A12515	205	A00094	A12516	205
A00094	A12517	205	A00094	A12662	235
A00094	A12664	279	A00094	A12687	202

A00094	A12897	440	A00094	A13111	156
A00094	A13146	926	A00094	A13397	380
A00094	A13652	205	A00094	A13734	307
A00094	A13864	156	A00094	A13946	165
A00094	A13978	993	A00094	A13986	422
A00094	A13997	351	A00094	A14005	480
A00094	A14007	411	A00094	A14164	168
A00094	A14198	491	A00094	A14364	183
A00094	A14396	277	A00094	A14440	190
A00094	A14443	943	A00094	A14444	378
A00094	A14452	341	A00094	A14489	198
A00094	A14498	317	A00094	A14502	338
A00094	A14524	277	A00094	A14539	490
A00094	A14585	218	A00094	A14589	205
A00094	A14620	168	A00094	A14623	156
A00094	A14643	481	A00094	A14648	446
A00094	A14653	152	A00094	A14659	236
A00094	A14669	168	A00094	A14674	430
A00094	A14784	205	A00094	A14797	168
A00094	A14916	205	A00094	A14924	205
A00094	A14925	399	A00094	A15237	362
A00094	A15293	385	A00094	A15294	246
A00094	A15324	234	A00094	A15353	268
A00094	A15420	429	A00094	A15465	210
A00094	A15506	411	A00094	A15726	477
A00094	A15821	205	A00094	A15954	474
A00094	A16427	205	A00094	A16512	168
A00094	A16636	425	A00094	A16738	202
A00094	A16874	185	A00094	A17050	168
A00094	A17160	899	A00094	A17174	205
A00095	A00142	900	A00095	A00143	904
A00095	A00166	163	A00095	A00167	390
A00095	A00168	945	A00095	A00169	188
A00095	A00193	486	A00095	A00200	899
A00095	A00202	73	A00095	A00209	249
A00095	A00210	489	A00095	A00212	491
A00095	A00215	899	A00095	A00219	486
A00095	A00224	488	A00095	A00245	188
A00095	A00274	168	A00095	A00316	193
A00095	A00322	308	A00095	A00347	160
A00095	A00348	484	A00095	A00349	243
A00095	A00350	188	A00095	A00351	452
A00095	A00377	491	A00095	A00426	443
A00095	A00472	258	A00095	A00570	230
A00095	A00616	391	A00095	A00630	195
A00095	A00712	213	A00095	A00863	188

A00095	A00989	378	A00095	A01124	229
A00095	A01125	488	A00095	A01137	221
A00095	A01156	490	A00095	A01161	271
A00095	A01163	205	A00095	A01211	283
A00095	A01257	475	A00095	A01481	306
A00095	A01485	491	A00095	A01502	157
A00095	A01527	286	A00095	A01547	452
A00095	A01582	179	A00095	A01632	241
A00095	A01700	485	A00095	A01790	255
A00095	A01889	218	A00095	A01910	375
A00095	A02082	208	A00095	A02091	288
A00095	A02111	425	A00095	A02148	400
A00095	A02154	185	A00095	A02188	346
A00095	A02192	173	A00095	A02221	443
A00095	A02301	360	A00095	A02303	196
A00095	A02304	235	A00095	A02310	162
A00095	A02312	484	A00095	A02392	262
A00095	A02467	259	A00095	A02470	443
A00095	A02477	154	A00095	A02501	249
A00095	A02504	490	A00095	A02531	314
A00095	A02541	183	A00095	A02583	207
A00095	A02584	218	A00095	A02598	235
A00095	A02618	485	A00095	A02621	407
A00095	A02671	369	A00095	A02672	408
A00095	A02673	491	A00095	A02699	154
A00095	A02703	384	A00095	A02760	174
A00095	A02761	246	A00095	A02822	195
A00095	A03068	198	A00095	A03115	185
A00095	A03178	96	A00095	A03186	118
A00095	A03209	176	A00095	A03211	204
A00095	A03264	191	A00095	A03314	208
A00095	A03334	488	A00095	A03477	307
A00095	A03535	383	A00095	A03573	235
A00095	A03579	311	A00095	A03583	361
A00095	A03589	256	A00095	A03609	174
A00095	A03642	299	A00095	A03713	191
A00095	A03723	154	A00095	A03795	195
A00095	A03961	424	A00095	A03992	407
A00095	A04081	391	A00095	A04092	414
A00095	A04183	232	A00095	A04222	480
A00095	A04256	490	A00095	A04418	286
A00095	A04423	405	A00095	A04424	160
A00095	A04445	188	A00095	A04491	390
A00095	A04556	196	A00095	A04723	201
A00095	A04751	208	A00095	A04813	183
A00095	A04821	246	A00095	A04990	246

A00095	A05075	281	A00095	A05249	213
A00095	A05252	288	A00095	A05306	350
A00095	A05442	311	A00095	A05462	172
A00095	A05825	456	A00095	A06277	308
A00095	A06400	446	A00095	A06606	163
A00095	A06608	899	A00095	A06671	205
A00095	A06739	353	A00095	A06922	288
A00095	A07014	196	A00095	A08064	353
A00095	A08136	246	A00095	A09118	326
A00095	A09240	246	A00095	A09269	382
A00095	A09366	350	A00095	A09471	229
A00095	A09812	229	A00095	A10072	162
A00095	A10141	288	A00095	A10237	262
A00095	A10476	409	A00095	A10541	350
A00095	A10622	262	A00095	A10844	185
A00095	A10887	262	A00095	A11353	243
A00095	A11378	252	A00095	A11467	267
A00095	A12453	350	A00095	A12654	484
A00095	A13025	350	A00095	A13346	491
A00095	A13917	344	A00095	A13965	420
A00095	A14002	326	A00095	A15230	252
A00095	A15233	185	A00095	A15498	486
A00095	A16075	283	A00095	A16087	238
A00095	A16374	188	A00095	A16433	216
A00095	A16627	335	A00096	A00103	338
A00096	A00104	307	A00096	A00199	413
A00096	A00236	229	A00096	A00430	491
A00096	A00675	176	A00096	A00863	254
A00096	A00970	377	A00096	A01380	140
A00096	A03584	185	A00096	A03738	343
A00096	A03747	449	A00096	A03790	227
A00096	A04191	491	A00096	A04192	455
A00096	A04280	208	A00096	A04573	301
A00096	A04614	212	A00096	A04964	265
A00096	A05288	195	A00096	A05830	312
A00096	A05995	240	A00096	A06359	160
A00096	A06473	185	A00096	A07163	484
A00096	A07245	301	A00096	A07247	340
A00096	A07634	167	A00096	A08093	481
A00096	A08679	283	A00096	A08978	215
A00096	A09210	483	A00096	A09271	338
A00096	A10490	193	A00096	A10519	326
A00096	A10532	491	A00096	A10589	460
A00096	A10830	366	A00096	A10907	433
A00096	A11240	347	A00096	A11299	296
A00096	A11705	235	A00096	A12818	353

A00096	A12839	475	A00096	A13084	249
A00096	A13344	353	A00096	A13875	182
A00096	A13944	241	A00096	A14132	491
A00096	A15696	369	A00096	A17085	480
A00097	A00125	266	A00097	A00141	293
A00097	A00158	162	A00097	A00168	374
A00097	A00219	205	A00097	A01520	171
A00097	A01524	461	A00097	A01527	268
A00097	A02000	246	A00097	A02668	195
A00097	A02785	307	A00097	A06463	329
A00097	A07011	204	A00097	A09749	390
A00097	A11313	458	A00097	A12531	417
A00097	A12560	406	A00097	A14471	174
A00097	A15317	353	A00097	A15505	258
A00097	A15598	307	A00097	A15913	249
A00097	A16559	398	A00098	A00165	167
A00098	A00167	485	A00098	A00182	490
A00098	A00183	945	A00098	A00214	63
A00098	A00219	324	A00098	A00235	899
A00098	A00262	369	A00098	A00324	374
A00098	A00679	351	A00098	A01088	152
A00098	A01213	226	A00098	A01378	187
A00098	A01507	372	A00098	A01524	388
A00098	A01527	942	A00098	A01606	246
A00098	A01688	227	A00098	A01698	240
A00098	A01850	177	A00098	A01995	257
A00098	A02210	308	A00098	A02427	183
A00098	A02698	157	A00098	A02822	452
A00098	A03451	177	A00098	A03778	491
A00098	A03893	297	A00098	A03895	271
A00098	A04007	196	A00098	A04202	202
A00098	A04449	490	A00098	A05181	173
A00098	A05221	389	A00098	A05489	272
A00098	A06254	396	A00098	A06317	236
A00098	A06588	460	A00098	A07422	899
A00098	A07803	340	A00098	A08022	174
A00098	A08878	408	A00098	A09072	154
A00098	A09235	272	A00098	A09256	900
A00098	A09363	165	A00098	A10014	168
A00098	A11983	443	A00098	A12897	446
A00098	A13946	207	A00098	A13994	285
A00098	A13997	445	A00098	A14055	472
A00098	A14211	296	A00098	A14316	245
A00098	A14636	180	A00098	A14925	254
A00098	A15293	379	A00098	A15726	899
A00098	A16904	190	A00099	A00122	118

A00099	A00134	899	A00099	A00136	433
A00099	A00137	328	A00099	A00138	63
A00099	A00139	170	A00099	A00140	409
A00099	A00141	899	A00099	A00157	905
A00099	A00167	899	A00099	A00169	899
A00099	A00172	899	A00099	A00182	899
A00099	A00184	916	A00099	A00187	138
A00099	A00207	899	A00099	A00208	899
A00099	A00209	899	A00099	A00224	899
A00099	A00230	899	A00099	A00233	899
A00099	A00239	899	A00099	A00240	899
A00099	A00241	899	A00099	A00242	899
A00099	A00246	899	A00099	A00247	899
A00099	A00254	899	A00099	A00458	360
A00099	A00669	899	A00099	A00871	179
A00099	A00933	899	A00099	A01052	899
A00099	A01075	176	A00099	A01176	899
A00099	A01214	163	A00099	A01524	899
A00099	A01527	899	A00099	A01553	928
A00099	A01554	912	A00099	A01558	899
A00099	A01560	943	A00099	A01561	899
A00099	A01562	921	A00099	A01588	899
A00099	A01592	899	A00099	A01602	899
A00099	A01605	191	A00099	A01615	899
A00099	A01628	901	A00099	A01629	207
A00099	A01651	899	A00099	A01680	899
A00099	A01688	899	A00099	A01692	924
A00099	A01697	908	A00099	A01702	899
A00099	A01709	899	A00099	A01718	899
A00099	A01844	899	A00099	A01920	899
A00099	A02717	180	A00099	A03331	179
A00099	A03338	238	A00099	A03435	152
A00099	A03451	899	A00099	A03525	165
A00099	A03565	278	A00099	A03591	900
A00099	A04447	190	A00099	A04699	899
A00099	A04853	252	A00099	A05086	212
A00099	A05216	899	A00099	A05453	899
A00099	A07311	917	A00099	A09577	905
A00099	A10365	899	A00099	A10801	899
A00099	A10833	899	A00099	A11125	899
A00099	A11983	899	A00099	A12383	207
A00099	A12473	308	A00099	A12705	899
A00099	A13605	899	A00099	A13985	899
A00099	A13991	899	A00099	A14024	899
A00099	A14188	899	A00099	A14198	899
A00099	A14452	85	A00099	A14472	899

A00099	A15195	363	A00099	A17168	443
A00100	A00114	249	A00100	A00119	477
A00100	A00163	430	A00100	A00167	909
A00100	A00195	366	A00100	A00209	899
A00100	A00224	899	A00100	A00236	945
A00100	A00240	401	A00100	A00252	945
A00100	A00253	994	A00100	A00264	396
A00100	A00294	218	A00100	A00309	479
A00100	A00652	488	A00100	A00736	299
A00100	A00747	491	A00100	A01104	341
A00100	A01189	167	A00100	A01405	163
A00100	A01498	286	A00100	A01524	899
A00100	A01527	905	A00100	A01550	945
A00100	A01660	380	A00100	A01692	480
A00100	A01730	207	A00100	A01740	63
A00100	A01741	379	A00100	A01763	162
A00100	A01775	358	A00100	A01886	451
A00100	A01897	102	A00100	A01904	165
A00100	A02304	232	A00100	A02325	486
A00100	A02336	200	A00100	A02339	308
A00100	A02349	162	A00100	A02359	296
A00100	A02410	216	A00100	A02418	235
A00100	A02421	279	A00100	A02494	177
A00100	A02507	334	A00100	A02525	363
A00100	A02647	265	A00100	A02676	221
A00100	A02695	177	A00100	A02703	441
A00100	A02744	84	A00100	A02766	191
A00100	A02775	308	A00100	A02786	183
A00100	A02789	267	A00100	A02816	165
A00100	A02827	249	A00100	A02830	212
A00100	A02836	213	A00100	A02837	480
A00100	A02842	491	A00100	A02859	420
A00100	A02868	297	A00100	A02869	316
A00100	A02880	279	A00100	A02902	344
A00100	A02903	229	A00100	A02912	187
A00100	A02921	254	A00100	A02958	213
A00100	A03112	347	A00100	A03186	207
A00100	A03229	236	A00100	A03286	296
A00100	A03287	174	A00100	A03305	285
A00100	A03321	63	A00100	A03338	270
A00100	A03415	165	A00100	A03427	424
A00100	A03450	241	A00100	A03451	443
A00100	A03657	249	A00100	A03725	273
A00100	A03919	188	A00100	A03922	329
A00100	A03976	179	A00100	A04059	201
A00100	A04085	234	A00100	A04180	210

A00100	A04216	205	A00100	A04277	348
A00100	A04440	182	A00100	A04710	317
A00100	A04720	308	A00100	A04729	168
A00100	A04818	156	A00100	A04847	396
A00100	A05036	254	A00100	A05039	279
A00100	A05077	348	A00100	A05122	299
A00100	A05141	413	A00100	A05185	173
A00100	A05302	345	A00100	A05578	236
A00100	A05868	486	A00100	A05902	353
A00100	A05967	180	A00100	A06200	180
A00100	A06581	330	A00100	A06595	201
A00100	A06683	279	A00100	A06949	152
A00100	A07167	252	A00100	A07311	899
A00100	A08576	205	A00100	A08655	191
A00100	A08837	199	A00100	A08899	446
A00100	A09125	308	A00100	A09127	257
A00100	A09221	154	A00100	A09476	483
A00100	A09504	177	A00100	A09668	218
A00100	A10081	245	A00100	A10148	213
A00100	A10233	386	A00100	A10311	286
A00100	A10337	230	A00100	A10346	249
A00100	A11921	462	A00100	A12246	236
A00100	A12705	899	A00100	A13161	348
A00100	A13255	457	A00100	A13985	899
A00100	A14013	213	A00100	A14449	308
A00100	A14648	324	A00100	A14726	348
A00100	A15282	177	A00100	A15675	254
A00100	A16300	283	A00100	A16624	234
A00100	A16973	229	A00100	A17088	151
A00101	A00104	944	A00101	A00209	899
A00101	A01524	899	A00101	A06608	251
A00101	A07163	363	A00101	A08093	385
A00101	A08679	328	A00101	A10532	939
A00102	A00104	899	A00102	A00209	899
A00102	A01161	208	A00102	A01524	899
A00102	A10532	193	A00102	A10589	899
A00102	A10830	899	A00102	A17214	899
A00103	A00104	899	A00103	A00173	160
A00103	A00209	899	A00103	A00430	341
A00103	A01125	232	A00103	A01524	899
A00103	A02006	314	A00103	A02836	204
A00103	A03817	218	A00103	A10532	329
A00103	A14557	899	A00104	A00430	313
A00104	A02499	311	A00104	A02749	221
A00104	A04191	357	A00104	A05601	391
A00104	A07163	329	A00104	A08093	475

A00104	A09215	432	A00104	A10532	472
A00104	A11299	406	A00104	A14132	436
A00104	A16124	899	A00105	A00106	899
A00105	A00107	926	A00105	A00150	587
A00105	A00157	899	A00105	A00199	182
A00105	A00209	899	A00105	A00288	424
A00105	A00405	249	A00105	A00414	475
A00105	A00426	390	A00105	A00427	657
A00105	A00521	480	A00105	A00537	461
A00105	A00571	179	A00105	A00576	424
A00105	A00590	664	A00105	A00603	445
A00105	A00604	176	A00105	A00605	490
A00105	A00648	738	A00105	A00651	816
A00105	A00653	418	A00105	A00697	450
A00105	A00700	417	A00105	A00706	179
A00105	A00763	330	A00105	A00787	665
A00105	A00809	445	A00105	A00839	486
A00105	A00870	396	A00105	A00878	491
A00105	A00923	212	A00105	A00935	469
A00105	A00953	176	A00105	A00965	662
A00105	A00966	687	A00105	A00970	484
A00105	A00985	224	A00105	A00986	534
A00105	A00994	411	A00105	A01012	835
A00105	A01025	664	A00105	A01027	455
A00105	A01034	491	A00105	A01037	488
A00105	A01038	475	A00105	A01067	665
A00105	A01072	199	A00105	A01085	486
A00105	A01090	688	A00105	A01095	346
A00105	A01101	162	A00105	A01108	212
A00105	A01112	312	A00105	A01119	457
A00105	A01140	460	A00105	A01144	716
A00105	A01156	446	A00105	A01168	462
A00105	A01206	373	A00105	A01238	196
A00105	A01247	329	A00105	A01249	383
A00105	A01263	337	A00105	A01291	597
A00105	A01294	486	A00105	A01295	333
A00105	A01335	600	A00105	A01366	224
A00105	A01372	374	A00105	A01385	355
A00105	A01392	665	A00105	A01416	436
A00105	A01433	665	A00105	A01436	660
A00105	A01447	468	A00105	A01468	380
A00105	A01496	688	A00105	A01524	899
A00105	A01579	368	A00105	A01586	207
A00105	A01601	346	A00105	A01649	208
A00105	A01718	383	A00105	A02164	63
A00105	A02165	462	A00105	A03438	113

A00105	A03738	157	A00105	A03981	682
A00105	A05501	218	A00105	A05577	221
A00105	A06084	247	A00105	A06351	479
A00105	A06356	303	A00105	A06814	358
A00105	A06825	229	A00105	A06965	157
A00105	A07237	227	A00105	A08011	665
A00105	A08055	187	A00105	A08150	196
A00105	A08301	165	A00105	A08359	441
A00105	A08377	491	A00105	A08543	319
A00105	A08550	684	A00105	A08556	168
A00105	A08653	127	A00105	A08773	491
A00105	A08812	456	A00105	A08973	489
A00105	A09332	182	A00105	A09592	162
A00105	A10316	375	A00105	A10532	491
A00105	A11240	450	A00105	A11254	307
A00105	A11297	481	A00105	A11334	457
A00105	A11550	190	A00105	A11728	208
A00105	A11992	389	A00105	A12433	489
A00105	A13375	267	A00105	A13720	920
A00105	A13963	382	A00105	A13989	491
A00105	A14172	200	A00105	A14216	773
A00105	A14447	368	A00105	A14482	477
A00105	A14569	165	A00105	A14843	423
A00105	A14937	324	A00105	A15666	488
A00105	A15931	489	A00105	A15932	483
A00105	A15988	475	A00105	A16157	174
A00106	A00107	899	A00106	A00157	899
A00106	A00209	904	A00106	A00210	121
A00106	A00216	246	A00106	A00219	457
A00106	A00264	327	A00106	A00310	218
A00106	A00312	491	A00106	A00616	350
A00106	A00661	307	A00106	A00815	912
A00106	A01137	372	A00106	A01138	327
A00106	A01462	286	A00106	A01463	190
A00106	A01466	314	A00106	A01475	447
A00106	A01476	432	A00106	A01485	243
A00106	A01490	483	A00106	A01493	462
A00106	A01527	899	A00106	A01590	411
A00106	A01594	188	A00106	A01629	160
A00106	A01722	491	A00106	A01731	199
A00106	A01883	193	A00106	A02071	182
A00106	A02083	224	A00106	A02104	283
A00106	A02215	489	A00106	A02236	210
A00106	A02251	162	A00106	A02284	419
A00106	A02299	293	A00106	A02301	400
A00106	A02306	480	A00106	A02329	199

A00106	A02413	324	A00106	A02419	188
A00106	A02423	458	A00106	A02424	152
A00106	A02425	939	A00106	A02465	488
A00106	A02472	127	A00106	A02481	73
A00106	A02621	177	A00106	A02666	238
A00106	A02669	252	A00106	A02671	193
A00106	A02758	216	A00106	A02759	438
A00106	A02785	435	A00106	A03053	452
A00106	A03054	491	A00106	A03099	373
A00106	A03110	310	A00106	A03498	358
A00106	A03583	283	A00106	A03633	435
A00106	A03639	272	A00106	A03776	190
A00106	A03873	172	A00106	A04001	279
A00106	A04218	375	A00106	A04226	899
A00106	A04271	152	A00106	A04369	157
A00106	A04471	274	A00106	A04612	230
A00106	A04614	198	A00106	A04633	235
A00106	A04842	184	A00106	A04910	346
A00106	A05217	286	A00106	A05277	260
A00106	A05487	301	A00106	A05493	484
A00106	A05494	386	A00106	A05533	491
A00106	A05573	165	A00106	A05992	221
A00106	A06037	224	A00106	A06607	438
A00106	A06734	263	A00106	A06762	412
A00106	A06912	232	A00106	A07061	151
A00106	A07200	379	A00106	A07471	322
A00106	A07475	168	A00106	A07538	286
A00106	A07908	358	A00106	A08210	265
A00106	A08385	210	A00106	A08444	277
A00106	A09031	321	A00106	A09509	110
A00106	A10131	224	A00106	A10306	358
A00106	A10841	358	A00106	A13162	159
A00106	A13734	286	A00106	A13879	428
A00106	A14187	205	A00106	A14402	218
A00106	A14735	255	A00106	A15230	177
A00106	A16636	286	A00107	A00157	899
A00107	A02695	299	A00107	A15498	262
A00108	A00141	176	A00108	A00159	243
A00108	A00224	177	A00108	A01473	491
A00108	A01527	921	A00108	A02076	938
A00108	A02120	269	A00108	A02864	112
A00108	A02897	241	A00108	A03722	168
A00108	A03894	183	A00108	A04687	259
A00108	A07135	154	A00108	A10587	327
A00108	A10721	305	A00108	A10899	207
A00108	A11415	199	A00108	A12843	384

A00108	A12848	433	A00108	A14011	195
A00108	A14300	308	A00108	A14452	328
A00108	A15626	484	A00108	A15726	74
A00108	A16994	218	A00109	A00111	462
A00109	A00123	490	A00109	A00125	167
A00109	A00141	347	A00109	A00153	317
A00109	A00188	283	A00109	A00204	942
A00109	A00209	899	A00109	A00213	74
A00109	A00214	99	A00109	A00220	449
A00109	A00230	899	A00109	A00233	229
A00109	A00248	364	A00109	A00274	346
A00109	A00616	179	A00109	A00752	252
A00109	A01009	344	A00109	A01043	204
A00109	A01524	162	A00109	A01527	927
A00109	A01558	899	A00109	A01604	443
A00109	A01615	899	A00109	A01654	491
A00109	A01702	162	A00109	A01704	330
A00109	A01743	263	A00109	A01802	176
A00109	A01870	488	A00109	A01880	391
A00109	A01888	458	A00109	A01902	170
A00109	A02033	311	A00109	A02152	162
A00109	A02220	321	A00109	A02251	170
A00109	A02337	423	A00109	A02382	440
A00109	A02416	378	A00109	A02439	443
A00109	A02458	373	A00109	A02459	200
A00109	A02460	243	A00109	A02479	208
A00109	A02481	171	A00109	A02490	157
A00109	A02578	259	A00109	A02582	172
A00109	A02604	307	A00109	A02606	369
A00109	A02608	382	A00109	A02611	297
A00109	A02615	243	A00109	A02637	483
A00109	A02639	172	A00109	A02654	294
A00109	A02655	465	A00109	A02659	226
A00109	A02661	268	A00109	A02698	286
A00109	A02711	362	A00109	A02730	481
A00109	A02769	380	A00109	A02788	205
A00109	A02803	188	A00109	A02808	357
A00109	A02851	259	A00109	A02854	477
A00109	A02863	491	A00109	A02876	165
A00109	A02886	427	A00109	A02896	477
A00109	A02898	219	A00109	A02917	491
A00109	A02927	199	A00109	A02931	249
A00109	A02932	327	A00109	A02935	353
A00109	A02944	443	A00109	A02945	491
A00109	A02955	445	A00109	A02962	480
A00109	A03049	162	A00109	A03113	240

A00109	A03177	183	A00109	A03185	290
A00109	A03195	199	A00109	A03239	160
A00109	A03259	433	A00109	A03271	435
A00109	A03276	246	A00109	A03280	221
A00109	A03305	216	A00109	A03610	446
A00109	A03783	266	A00109	A03900	247
A00109	A04010	212	A00109	A04033	193
A00109	A04130	160	A00109	A04138	243
A00109	A04238	293	A00109	A04248	458
A00109	A04257	394	A00109	A04263	385
A00109	A04267	291	A00109	A04272	257
A00109	A04320	358	A00109	A04478	363
A00109	A04519	191	A00109	A04598	290
A00109	A04600	196	A00109	A04609	196
A00109	A04659	260	A00109	A04734	441
A00109	A04865	224	A00109	A04878	249
A00109	A04962	218	A00109	A05113	179
A00109	A05177	316	A00109	A05305	420
A00109	A05487	176	A00109	A05577	174
A00109	A05679	263	A00109	A05860	333
A00109	A05944	475	A00109	A05985	486
A00109	A06054	405	A00109	A06055	324
A00109	A06314	274	A00109	A06344	259
A00109	A06414	249	A00109	A06415	489
A00109	A06422	350	A00109	A06650	152
A00109	A06747	188	A00109	A06899	382
A00109	A07031	290	A00109	A07142	296
A00109	A07146	249	A00109	A07148	254
A00109	A07152	484	A00109	A07157	430
A00109	A07161	400	A00109	A07164	266
A00109	A07166	152	A00109	A07173	491
A00109	A07372	293	A00109	A07390	246
A00109	A09570	283	A00109	A09981	380
A00109	A10214	216	A00109	A13821	281
A00109	A13872	204	A00109	A14071	399
A00109	A14146	356	A00109	A14231	176
A00109	A14366	308	A00109	A14373	279
A00109	A14374	391	A00109	A14376	257
A00109	A14391	466	A00109	A14392	352
A00109	A14393	246	A00109	A14432	382
A00109	A14439	324	A00109	A14442	227
A00109	A14554	156	A00109	A14598	246
A00109	A14599	408	A00109	A15241	322
A00109	A15394	389	A00109	A15395	479
A00109	A15403	272	A00109	A15426	430
A00109	A15475	221	A00109	A15601	454

A00109	A15602	283	A00109	A15603	484
A00109	A15605	472	A00109	A15606	368
A00109	A15607	483	A00109	A15608	462
A00109	A15795	299	A00109	A15819	193
A00109	A15865	321	A00109	A15959	363
A00109	A15964	353	A00109	A15981	371
A00110	A00150	899	A00110	A00198	901
A00110	A00252	899	A00110	A00253	899
A00110	A00261	364	A00110	A00267	221
A00110	A00380	196	A00110	A00469	303
A00110	A00832	899	A00110	A00981	227
A00110	A01267	190	A00110	A01291	899
A00110	A01354	265	A00110	A01380	165
A00110	A01410	401	A00110	A01492	899
A00110	A01527	430	A00110	A01664	180
A00110	A01770	906	A00110	A01974	469
A00110	A02231	271	A00110	A02428	491
A00110	A03512	372	A00110	A03536	162
A00110	A03886	260	A00110	A04361	191
A00110	A04928	247	A00110	A05240	224
A00110	A06088	307	A00110	A06120	329
A00110	A06346	269	A00110	A06403	338
A00110	A07004	391	A00110	A07422	899
A00110	A07726	932	A00110	A09778	290
A00110	A10525	329	A00110	A10575	899
A00110	A10604	219	A00110	A11129	296
A00110	A13147	168	A00110	A13989	899
A00110	A14299	418	A00110	A14334	394
A00110	A14526	368	A00110	A15257	170
A00110	A15559	255	A00111	A00152	224
A00111	A00159	317	A00111	A00163	483
A00111	A00167	613	A00111	A00168	481
A00111	A00171	257	A00111	A00176	625
A00111	A00177	177	A00111	A00181	338
A00111	A00190	441	A00111	A00196	265
A00111	A00200	152	A00111	A00212	168
A00111	A00216	191	A00111	A00219	491
A00111	A00220	395	A00111	A00245	491
A00111	A00264	443	A00111	A00274	491
A00111	A00324	899	A00111	A00420	409
A00111	A00440	307	A00111	A00503	232
A00111	A00556	286	A00111	A00616	490
A00111	A00736	481	A00111	A00752	491
A00111	A00815	297	A00111	A00829	201
A00111	A00850	367	A00111	A01009	257
A00111	A01091	200	A00111	A01137	477

A00111	A01163	386	A00111	A01164	256
A00111	A01235	183	A00111	A01257	472
A00111	A01277	316	A00111	A01524	201
A00111	A01530	208	A00111	A01534	205
A00111	A01542	359	A00111	A01590	401
A00111	A01604	402	A00111	A01605	151
A00111	A01654	322	A00111	A01741	227
A00111	A01766	154	A00111	A01870	310
A00111	A01903	241	A00111	A02010	212
A00111	A02092	176	A00111	A02099	185
A00111	A02111	351	A00111	A02134	249
A00111	A02138	472	A00111	A02142	157
A00111	A02143	281	A00111	A02144	109
A00111	A02148	224	A00111	A02174	491
A00111	A02176	479	A00111	A02213	168
A00111	A02228	481	A00111	A02236	439
A00111	A02245	491	A00111	A02283	198
A00111	A02298	347	A00111	A02303	333
A00111	A02304	481	A00111	A02306	435
A00111	A02309	352	A00111	A02313	319
A00111	A02326	154	A00111	A02381	185
A00111	A02382	475	A00111	A02383	270
A00111	A02416	159	A00111	A02425	224
A00111	A02482	373	A00111	A02490	177
A00111	A02491	374	A00111	A02502	322
A00111	A02505	474	A00111	A02545	63
A00111	A02578	299	A00111	A02587	327
A00111	A02604	223	A00111	A02608	390
A00111	A02618	154	A00111	A02621	207
A00111	A02624	138	A00111	A02637	283
A00111	A02644	232	A00111	A02655	163
A00111	A02668	171	A00111	A02673	484
A00111	A02759	319	A00111	A02764	188
A00111	A02769	302	A00111	A02851	165
A00111	A02854	199	A00111	A02917	195
A00111	A02945	271	A00111	A03049	407
A00111	A03076	491	A00111	A03077	180
A00111	A03081	228	A00111	A03099	435
A00111	A03104	436	A00111	A03109	364
A00111	A03110	491	A00111	A03183	469
A00111	A03219	177	A00111	A03271	276
A00111	A03284	223	A00111	A03292	232
A00111	A03305	121	A00111	A03310	484
A00111	A03314	480	A00111	A03315	422
A00111	A03319	198	A00111	A03402	63
A00111	A03413	112	A00111	A03458	154

A00111	A03578	174	A00111	A03584	199
A00111	A03639	483	A00111	A03771	358
A00111	A03799	236	A00111	A03818	263
A00111	A03919	154	A00111	A03942	460
A00111	A03961	269	A00111	A04033	491
A00111	A04092	223	A00111	A04130	173
A00111	A04163	396	A00111	A04180	177
A00111	A04216	173	A00111	A04248	308
A00111	A04256	213	A00111	A04257	193
A00111	A04423	163	A00111	A04478	366
A00111	A04504	235	A00111	A04637	316
A00111	A04676	425	A00111	A04748	229
A00111	A04755	185	A00111	A04756	260
A00111	A04976	407	A00111	A05149	188
A00111	A05249	177	A00111	A05408	299
A00111	A05418	190	A00111	A05538	249
A00111	A05545	193	A00111	A05588	269
A00111	A05679	382	A00111	A05696	433
A00111	A05706	345	A00111	A05736	390
A00111	A05737	269	A00111	A05786	418
A00111	A05815	466	A00111	A05944	406
A00111	A05985	443	A00111	A06054	451
A00111	A06095	313	A00111	A06415	195
A00111	A06581	191	A00111	A06588	339
A00111	A06610	445	A00111	A06727	154
A00111	A06847	159	A00111	A06922	258
A00111	A07031	402	A00111	A07126	440
A00111	A07143	469	A00111	A07145	355
A00111	A07148	160	A00111	A07152	227
A00111	A07157	191	A00111	A07161	213
A00111	A07166	162	A00111	A07173	447
A00111	A07209	278	A00111	A07339	449
A00111	A07372	335	A00111	A07390	374
A00111	A07422	900	A00111	A07833	182
A00111	A07912	351	A00111	A08078	162
A00111	A08264	269	A00111	A08714	326
A00111	A08749	328	A00111	A08878	384
A00111	A09078	274	A00111	A09125	283
A00111	A09480	440	A00111	A09509	293
A00111	A09870	208	A00111	A10021	191
A00111	A10168	249	A00111	A10287	299
A00111	A10336	305	A00111	A11007	241
A00111	A11014	160	A00111	A11983	422
A00111	A12682	283	A00111	A14025	606
A00111	A14055	374	A00111	A14190	377
A00111	A14210	491	A00111	A14371	379

A00111	A14376	163	A00111	A14410	268
A00111	A14423	260	A00111	A14432	173
A00111	A14475	449	A00111	A14511	456
A00111	A14536	198	A00111	A14554	486
A00111	A14589	391	A00111	A14604	489
A00111	A14635	435	A00111	A15230	208
A00111	A15264	351	A00111	A15296	198
A00111	A15312	235	A00111	A15327	246
A00111	A15330	170	A00111	A15395	160
A00111	A15426	283	A00111	A15605	213
A00111	A15607	293	A00111	A15916	157
A00111	A15969	241	A00111	A16043	183
A00111	A16055	167	A00111	A16125	208
A00111	A16138	201	A00111	A16194	249
A00111	A16636	185	A00112	A00127	179
A00112	A00128	177	A00112	A00157	899
A00112	A00190	443	A00112	A00219	479
A00112	A00221	457	A00112	A00234	193
A00112	A00264	491	A00112	A00270	473
A00112	A00292	491	A00112	A00352	193
A00112	A00418	455	A00112	A00420	243
A00112	A00478	177	A00112	A00577	260
A00112	A00616	489	A00112	A00815	167
A00112	A00926	152	A00112	A00932	251
A00112	A00999	229	A00112	A01164	212
A00112	A01212	316	A00112	A01888	439
A00112	A01936	152	A00112	A02088	246
A00112	A02105	156	A00112	A02250	341
A00112	A02260	157	A00112	A02278	429
A00112	A02388	328	A00112	A02416	185
A00112	A02419	340	A00112	A02490	474
A00112	A02491	488	A00112	A02496	294
A00112	A02581	228	A00112	A02668	458
A00112	A02749	302	A00112	A02756	480
A00112	A02841	430	A00112	A02859	368
A00112	A03053	160	A00112	A03156	232
A00112	A03224	456	A00112	A03259	481
A00112	A03458	168	A00112	A03539	294
A00112	A03750	468	A00112	A03780	168
A00112	A03798	210	A00112	A03834	212
A00112	A04038	357	A00112	A04169	196
A00112	A04194	168	A00112	A04200	272
A00112	A04201	196	A00112	A04242	243
A00112	A04254	283	A00112	A04255	283
A00112	A04288	266	A00112	A04398	490
A00112	A04416	355	A00112	A04419	283

A00112	A04471	207	A00112	A04490	438
A00112	A04888	163	A00112	A05084	249
A00112	A05144	296	A00112	A05780	249
A00112	A06008	294	A00112	A06251	256
A00112	A06306	193	A00112	A06622	327
A00112	A06742	272	A00112	A06825	240
A00112	A06955	232	A00112	A07138	177
A00112	A07164	162	A00112	A07589	283
A00112	A08022	398	A00112	A08194	424
A00112	A08221	183	A00112	A08541	210
A00112	A08738	157	A00112	A08817	480
A00112	A08880	227	A00112	A09062	217
A00112	A09085	198	A00112	A09218	377
A00112	A09390	176	A00112	A09493	263
A00112	A09654	107	A00112	A09778	185
A00112	A10182	312	A00112	A10650	283
A00112	A10756	446	A00112	A10791	293
A00112	A11064	488	A00112	A11123	488
A00112	A11129	193	A00112	A11725	168
A00112	A13348	154	A00112	A13396	272
A00112	A13743	313	A00112	A13986	200
A00112	A14007	230	A00112	A14190	456
A00112	A14366	391	A00112	A14368	154
A00112	A14370	291	A00112	A14418	224
A00112	A14511	170	A00112	A14526	446
A00112	A15420	433	A00112	A15429	179
A00112	A15498	480	A00112	A15913	438
A00112	A16636	491	A00112	A16664	188
A00112	A16674	302	A00112	A17171	338
A00113	A00130	82	A00113	A00133	899
A00113	A00156	938	A00113	A00165	63
A00113	A00167	899	A00113	A00180	491
A00113	A00185	172	A00113	A00207	554
A00113	A00209	899	A00113	A00230	899
A00113	A00231	586	A00113	A00240	360
A00113	A00250	162	A00113	A00257	405
A00113	A00284	160	A00113	A00285	258
A00113	A00300	223	A00113	A00388	273
A00113	A00439	427	A00113	A00698	433
A00113	A00809	160	A00113	A00833	160
A00113	A00876	160	A00113	A00922	160
A00113	A00932	402	A00113	A00933	365
A00113	A00980	160	A00113	A01161	160
A00113	A01261	160	A00113	A01307	63
A00113	A01357	182	A00113	A01410	351
A00113	A01423	246	A00113	A01435	104

A00113	A01479	167	A00113	A01507	63
A00113	A01512	899	A00113	A01527	899
A00113	A01536	915	A00113	A01558	899
A00113	A01560	486	A00113	A01561	113
A00113	A01562	842	A00113	A01588	899
A00113	A01615	899	A00113	A01674	486
A00113	A01692	447	A00113	A01708	934
A00113	A01709	66	A00113	A01719	74
A00113	A01822	160	A00113	A02058	291
A00113	A02411	235	A00113	A02473	249
A00113	A02732	163	A00113	A02790	151
A00113	A02887	63	A00113	A03451	157
A00113	A03591	386	A00113	A03677	160
A00113	A03752	457	A00113	A04012	168
A00113	A04073	374	A00113	A04553	277
A00113	A06289	149	A00113	A06466	408
A00113	A06950	160	A00113	A07311	945
A00113	A07798	463	A00113	A08259	171
A00113	A08533	160	A00113	A08902	484
A00113	A09018	213	A00113	A09218	200
A00113	A09263	221	A00113	A09297	160
A00113	A09424	317	A00113	A09568	219
A00113	A09643	258	A00113	A09647	159
A00113	A09660	488	A00113	A09803	219
A00113	A09804	286	A00113	A09902	532
A00113	A10047	232	A00113	A10332	204
A00113	A10365	266	A00113	A10728	467
A00113	A10801	172	A00113	A10813	183
A00113	A10982	255	A00113	A11604	263
A00113	A12043	160	A00113	A12227	160
A00113	A12383	112	A00113	A12932	423
A00113	A12969	491	A00113	A12971	945
A00113	A12990	270	A00113	A13149	419
A00113	A13340	160	A00113	A13397	168
A00113	A13511	243	A00113	A14004	489
A00113	A14013	238	A00113	A14738	160
A00113	A15004	266	A00113	A15106	283
A00113	A15244	160	A00113	A15677	204
A00113	A15932	160	A00113	A15933	160
A00113	A16084	202	A00113	A16272	160
A00113	A16318	160	A00113	A16437	160
A00113	A16446	160	A00113	A16585	160
A00113	A16678	262	A00114	A00120	256
A00114	A00122	112	A00114	A00130	71
A00114	A00165	63	A00114	A00257	229
A00114	A00419	223	A00114	A00454	288

A00114	A00851	179	A00114	A00910	173
A00114	A00912	271	A00114	A00981	483
A00114	A01042	433	A00114	A01083	489
A00114	A01357	246	A00114	A01628	71
A00114	A01631	385	A00114	A01692	467
A00114	A01709	113	A00114	A01719	124
A00114	A01797	157	A00114	A01889	188
A00114	A03649	170	A00114	A03740	438
A00114	A03785	230	A00114	A05037	182
A00114	A06289	257	A00114	A06748	443
A00114	A06950	419	A00114	A07311	205
A00114	A09476	488	A00114	A10047	291
A00114	A11124	367	A00114	A12383	196
A00114	A12988	217	A00114	A13240	210
A00114	A13429	234	A00114	A14006	163
A00114	A15573	324	A00114	A16060	265
A00115	A00932	926	A00115	A01983	360
A00115	A04415	414	A00115	A07311	899
A00115	A14188	899	A00116	A00124	402
A00116	A00129	474	A00116	A00152	224
A00116	A00162	260	A00116	A00165	301
A00116	A00167	441	A00116	A00179	144
A00116	A00187	266	A00116	A00188	465
A00116	A00205	185	A00116	A00207	340
A00116	A00209	899	A00116	A00211	395
A00116	A00224	899	A00116	A00239	301
A00116	A00240	938	A00116	A00242	296
A00116	A00248	396	A00116	A00643	188
A00116	A01527	902	A00116	A01639	944
A00116	A01640	919	A00116	A01726	489
A00116	A01790	344	A00116	A01889	195
A00116	A01931	324	A00116	A02075	322
A00116	A02343	202	A00116	A02412	177
A00116	A03436	241	A00116	A03528	224
A00116	A03719	176	A00116	A04205	228
A00116	A05944	945	A00116	A07311	165
A00116	A07422	160	A00116	A09937	380
A00116	A10392	936	A00116	A10623	188
A00116	A10677	217	A00116	A11335	388
A00116	A12886	395	A00116	A13181	196
A00116	A13917	152	A00116	A13977	479
A00116	A14426	379	A00116	A15241	414
A00116	A15813	274	A00116	A16083	940
A00117	A00121	899	A00117	A00152	195
A00117	A00171	364	A00117	A00261	369
A00117	A00422	235	A00117	A01600	184

A00117	A01615	339	A00117	A01691	263
A00117	A02836	291	A00117	A03419	163
A00117	A03566	172	A00117	A04279	260
A00117	A04639	281	A00117	A08289	257
A00117	A08571	224	A00117	A08681	278
A00117	A08753	266	A00117	A10525	236
A00117	A11332	255	A00117	A13672	288
A00117	A15416	193	A00117	A15726	245
A00118	A00122	99	A00118	A00132	165
A00118	A00138	432	A00118	A00146	263
A00118	A00156	401	A00118	A00158	63
A00118	A00161	63	A00118	A00162	899
A00118	A00165	848	A00118	A00185	619
A00118	A00186	156	A00118	A00198	362
A00118	A00201	271	A00118	A00208	104
A00118	A00234	857	A00118	A00243	475
A00118	A00254	959	A00118	A00261	157
A00118	A00340	193	A00118	A01553	63
A00118	A01554	63	A00118	A01580	241
A00118	A01596	763	A00118	A01709	63
A00118	A01792	82	A00118	A01888	335
A00118	A01908	63	A00118	A02988	156
A00118	A03019	63	A00118	A03470	385
A00118	A03784	286	A00118	A04012	165
A00118	A04088	174	A00118	A04293	201
A00118	A04323	477	A00118	A05009	469
A00118	A06077	247	A00118	A06914	297
A00118	A07422	165	A00118	A08494	204
A00118	A08552	257	A00118	A09220	623
A00118	A09401	313	A00118	A09577	63
A00118	A10285	340	A00118	A10510	229
A00118	A10580	152	A00118	A10735	183
A00118	A10913	212	A00118	A11129	330
A00118	A12009	159	A00118	A12499	159
A00118	A13216	318	A00118	A13340	240
A00118	A13980	256	A00118	A14032	338
A00118	A14157	316	A00118	A14299	167
A00118	A14433	903	A00118	A14438	185
A00118	A15512	154	A00118	A16547	588
A00118	A16560	204	A00119	A00167	912
A00119	A00195	945	A00119	A00209	899
A00119	A00236	968	A00119	A00252	969
A00119	A00253	970	A00119	A00293	377
A00119	A00295	432	A00119	A00652	490
A00119	A00852	213	A00119	A01045	469
A00119	A01550	457	A00119	A01551	408

A00119	A01566	213	A00119	A01660	269
A00119	A01692	479	A00119	A01772	201
A00119	A02323	360	A00119	A02325	438
A00119	A02647	314	A00119	A02854	177
A00119	A03819	171	A00119	A04015	182
A00119	A04209	232	A00119	A04342	441
A00119	A08881	152	A00119	A10311	457
A00119	A11921	491	A00119	A13255	447
A00119	A14443	899	A00119	A14648	165
A00119	A16080	941	A00119	A16764	160
A00120	A00128	945	A00120	A00132	485
A00120	A00209	899	A00120	A00236	338
A00120	A00271	899	A00120	A00419	380
A00120	A01527	899	A00120	A01562	424
A00120	A01692	341	A00120	A03436	152
A00120	A03643	329	A00120	A03740	241
A00120	A04957	313	A00120	A07311	63
A00120	A08845	180	A00120	A09124	152
A00120	A09253	432	A00120	A10813	286
A00120	A11125	899	A00120	A13984	283
A00121	A00157	899	A00121	A00160	267
A00121	A00171	196	A00121	A00189	491
A00121	A00261	201	A00121	A00309	210
A00121	A00879	213	A00121	A01029	288
A00121	A02249	227	A00121	A02293	268
A00121	A02711	490	A00121	A03181	182
A00121	A03276	260	A00121	A03399	156
A00121	A03651	212	A00121	A04737	218
A00121	A06731	156	A00121	A07004	263
A00121	A07524	162	A00121	A08327	407
A00121	A08416	486	A00121	A09038	187
A00121	A09431	305	A00121	A10059	350
A00121	A10580	899	A00121	A10599	205
A00121	A10813	234	A00121	A11543	491
A00121	A12897	267	A00121	A12971	232
A00121	A14236	152	A00121	A14621	424
A00121	A14925	165	A00121	A15606	243
A00121	A16476	302	A00122	A00138	491
A00122	A00145	63	A00122	A00152	454
A00122	A00156	356	A00122	A00159	341
A00122	A00161	469	A00122	A00185	490
A00122	A00207	489	A00122	A00208	491
A00122	A00224	450	A00122	A00234	308
A00122	A00246	489	A00122	A00254	491
A00122	A01275	265	A00122	A01473	402
A00122	A01524	450	A00122	A01527	241

A00122	A01553	126	A00122	A01554	63
A00122	A01560	104	A00122	A01561	63
A00122	A01572	490	A00122	A01628	63
A00122	A01692	63	A00122	A01697	219
A00122	A01698	77	A00122	A01719	63
A00122	A01887	165	A00122	A01908	63
A00122	A02326	174	A00122	A02350	63
A00122	A02411	170	A00122	A02578	185
A00122	A02656	260	A00122	A02659	151
A00122	A02676	208	A00122	A02678	135
A00122	A02800	185	A00122	A02958	200
A00122	A03209	427	A00122	A03465	113
A00122	A03685	171	A00122	A03809	196
A00122	A04107	208	A00122	A04111	246
A00122	A04556	182	A00122	A05072	260
A00122	A05247	152	A00122	A05339	241
A00122	A05888	217	A00122	A06138	185
A00122	A06289	63	A00122	A07148	313
A00122	A07311	63	A00122	A07370	417
A00122	A09220	79	A00122	A09540	208
A00122	A09863	246	A00122	A09913	213
A00122	A10437	224	A00122	A10801	357
A00122	A11369	337	A00122	A13151	157
A00122	A13291	412	A00122	A13312	191
A00122	A13605	369	A00122	A13827	217
A00122	A13860	198	A00122	A13980	391
A00122	A13983	63	A00122	A14004	347
A00122	A14019	260	A00122	A14027	267
A00122	A14187	361	A00122	A14188	475
A00122	A14198	491	A00122	A14371	402
A00122	A14402	152	A00122	A14430	452
A00122	A14433	313	A00122	A14438	183
A00122	A14452	63	A00122	A14511	179
A00122	A14543	152	A00122	A14686	217
A00122	A14930	82	A00122	A15290	193
A00122	A15352	269	A00122	A15387	196
A00122	A15515	200	A00122	A15726	110
A00122	A15916	177	A00123	A00125	466
A00123	A00141	479	A00123	A00152	486
A00123	A00153	486	A00123	A00158	475
A00123	A00159	179	A00123	A00160	472
A00123	A00167	258	A00123	A00174	154
A00123	A00189	688	A00123	A00190	475
A00123	A00212	256	A00123	A00220	480
A00123	A00224	899	A00123	A00226	247
A00123	A00232	486	A00123	A00233	491

A00123	A00234	943	A00123	A00248	353
A00123	A00255	446	A00123	A00264	424
A00123	A00338	274	A00123	A00407	180
A00123	A00561	463	A00123	A00600	165
A00123	A00694	490	A00123	A00739	249
A00123	A00820	281	A00123	A00887	205
A00123	A00914	469	A00123	A01138	159
A00123	A01140	219	A00123	A01161	457
A00123	A01524	491	A00123	A01527	945
A00123	A01559	467	A00123	A01615	918
A00123	A01654	491	A00123	A01704	152
A00123	A01726	191	A00123	A01733	408
A00123	A01734	319	A00123	A01741	457
A00123	A01747	196	A00123	A01767	165
A00123	A01769	419	A00123	A01776	246
A00123	A01792	96	A00123	A01876	266
A00123	A01879	191	A00123	A01880	491
A00123	A01881	449	A00123	A01882	276
A00123	A01884	229	A00123	A01887	294
A00123	A01888	490	A00123	A01889	428
A00123	A01890	165	A00123	A01894	188
A00123	A01895	210	A00123	A01908	63
A00123	A01936	307	A00123	A02143	256
A00123	A02220	276	A00123	A02247	263
A00123	A02293	198	A00123	A02337	177
A00123	A02338	162	A00123	A02340	353
A00123	A02382	766	A00123	A02383	177
A00123	A02384	353	A00123	A02416	221
A00123	A02460	168	A00123	A02464	259
A00123	A02497	263	A00123	A02515	276
A00123	A02542	193	A00123	A02606	323
A00123	A02615	439	A00123	A02616	195
A00123	A02639	456	A00123	A02657	249
A00123	A02661	213	A00123	A02694	159
A00123	A02695	188	A00123	A02696	200
A00123	A02698	491	A00123	A02709	369
A00123	A02711	474	A00123	A02730	412
A00123	A02732	143	A00123	A02741	71
A00123	A02766	196	A00123	A02788	311
A00123	A02798	172	A00123	A02808	173
A00123	A02812	399	A00123	A02816	172
A00123	A02828	326	A00123	A02851	202
A00123	A02854	346	A00123	A02863	491
A00123	A02917	490	A00123	A02945	450
A00123	A02955	193	A00123	A02982	430
A00123	A02984	182	A00123	A02986	195

A00123	A02988	281	A00123	A03010	240
A00123	A03019	63	A00123	A03035	196
A00123	A03100	110	A00123	A03259	358
A00123	A03275	172	A00123	A03451	179
A00123	A03537	263	A00123	A03610	283
A00123	A03611	361	A00123	A03639	208
A00123	A03780	191	A00123	A03790	382
A00123	A03807	249	A00123	A03934	380
A00123	A03965	246	A00123	A04016	283
A00123	A04023	159	A00123	A04033	486
A00123	A04041	313	A00123	A04088	191
A00123	A04105	196	A00123	A04138	187
A00123	A04206	210	A00123	A04207	229
A00123	A04238	241	A00123	A04248	486
A00123	A04263	334	A00123	A04267	221
A00123	A04280	367	A00123	A04364	162
A00123	A04473	205	A00123	A04474	254
A00123	A04484	374	A00123	A04493	262
A00123	A04510	191	A00123	A04517	322
A00123	A04518	221	A00123	A04529	200
A00123	A04536	156	A00123	A04545	270
A00123	A04567	156	A00123	A04659	205
A00123	A04723	202	A00123	A04794	313
A00123	A04798	263	A00123	A04865	168
A00123	A04878	326	A00123	A04879	424
A00123	A04888	185	A00123	A04944	185
A00123	A05278	338	A00123	A05305	388
A00123	A05309	379	A00123	A05741	216
A00123	A05860	291	A00123	A05944	327
A00123	A06055	263	A00123	A06211	232
A00123	A06422	163	A00123	A06610	258
A00123	A06635	193	A00123	A06650	458
A00123	A06654	165	A00123	A06728	283
A00123	A07145	223	A00123	A07146	326
A00123	A07173	490	A00123	A07182	276
A00123	A07199	345	A00123	A07200	152
A00123	A07227	172	A00123	A07295	276
A00123	A07310	313	A00123	A07311	899
A00123	A07626	185	A00123	A07985	190
A00123	A08623	328	A00123	A09112	276
A00123	A09220	91	A00123	A09559	263
A00123	A09570	232	A00123	A09959	301
A00123	A09981	352	A00123	A10598	157
A00123	A10626	221	A00123	A10901	259
A00123	A11023	201	A00123	A11348	168
A00123	A11369	63	A00123	A11411	335

A00123	A11707	154	A00123	A11881	285
A00123	A12913	313	A00123	A12971	293
A00123	A13050	208	A00123	A13391	207
A00123	A13555	208	A00123	A13667	285
A00123	A13978	245	A00123	A14029	902
A00123	A14236	491	A00123	A14391	152
A00123	A14393	235	A00123	A14426	302
A00123	A14635	343	A00123	A15241	490
A00123	A15395	480	A00123	A15486	180
A00123	A15598	380	A00123	A15599	286
A00123	A15601	394	A00123	A15602	358
A00123	A15603	299	A00123	A15604	356
A00123	A15605	262	A00123	A15606	461
A00123	A15630	283	A00123	A15641	154
A00123	A15981	472	A00123	A16527	254
A00123	A16741	402	A00124	A00136	77
A00124	A00163	929	A00124	A00167	288
A00124	A00185	378	A00124	A00209	899
A00124	A00224	911	A00124	A00230	899
A00124	A00236	385	A00124	A00239	345
A00124	A00240	945	A00124	A00252	439
A00124	A00253	400	A00124	A00284	425
A00124	A00300	398	A00124	A00312	396
A00124	A00316	165	A00124	A00552	302
A00124	A00601	202	A00124	A00621	401
A00124	A00624	296	A00124	A00660	205
A00124	A00698	323	A00124	A00930	486
A00124	A01235	436	A00124	A01553	268
A00124	A01554	185	A00124	A01558	899
A00124	A01560	63	A00124	A01631	942
A00124	A01639	937	A00124	A01660	380
A00124	A01680	424	A00124	A01717	291
A00124	A01726	945	A00124	A01748	473
A00124	A01897	93	A00124	A02054	360
A00124	A02323	163	A00124	A02696	408
A00124	A02982	357	A00124	A03164	372
A00124	A03331	283	A00124	A05363	288
A00124	A05424	160	A00124	A06000	491
A00124	A06466	486	A00124	A06650	265
A00124	A06914	490	A00124	A07257	157
A00124	A07311	385	A00124	A07366	204
A00124	A07422	945	A00124	A07771	383
A00124	A08707	470	A00124	A09464	151
A00124	A09577	379	A00124	A10392	91
A00124	A10534	475	A00124	A10632	171
A00124	A10698	288	A00124	A10720	252

A00124	A10808	99	A00124	A13340	185
A00124	A13684	279	A00124	A13946	160
A00124	A13977	942	A00124	A14055	899
A00124	A14073	488	A00124	A14252	466
A00124	A14961	318	A00124	A15356	491
A00124	A15726	63	A00124	A15964	152
A00124	A16272	382	A00124	A16331	182
A00124	A16453	272	A00125	A00153	386
A00125	A00158	484	A00125	A00160	358
A00125	A00174	224	A00125	A00189	411
A00125	A00210	66	A00125	A00212	63
A00125	A00220	160	A00125	A00255	475
A00125	A00407	249	A00125	A00694	283
A00125	A00697	177	A00125	A00733	258
A00125	A00911	154	A00125	A00937	172
A00125	A00981	458	A00125	A01022	219
A00125	A01125	245	A00125	A01520	374
A00125	A01524	958	A00125	A01527	384
A00125	A01629	481	A00125	A01658	269
A00125	A01699	223	A00125	A01767	257
A00125	A01792	159	A00125	A01799	173
A00125	A01880	322	A00125	A01889	171
A00125	A01905	375	A00125	A02000	789
A00125	A02046	229	A00125	A02141	279
A00125	A02143	405	A00125	A02171	266
A00125	A02217	335	A00125	A02247	321
A00125	A02249	353	A00125	A02337	247
A00125	A02382	307	A00125	A02416	322
A00125	A02447	441	A00125	A02468	473
A00125	A02473	388	A00125	A02479	484
A00125	A02494	191	A00125	A02502	179
A00125	A02615	375	A00125	A02616	380
A00125	A02620	294	A00125	A02624	243
A00125	A02632	274	A00125	A02639	188
A00125	A02669	272	A00125	A02732	213
A00125	A02788	429	A00125	A02819	151
A00125	A02837	223	A00125	A03041	258
A00125	A03071	430	A00125	A03081	344
A00125	A03093	238	A00125	A03100	310
A00125	A03195	212	A00125	A03219	176
A00125	A03451	252	A00125	A03522	411
A00125	A03790	468	A00125	A03906	318
A00125	A03926	457	A00125	A04007	232
A00125	A04078	466	A00125	A04201	283
A00125	A04280	462	A00125	A04473	385
A00125	A04517	257	A00125	A04681	151

A00125	A04759	224	A00125	A04764	358
A00125	A04960	160	A00125	A05030	291
A00125	A05065	193	A00125	A05082	452
A00125	A05155	418	A00125	A05741	281
A00125	A05868	246	A00125	A06107	313
A00125	A06578	173	A00125	A06813	474
A00125	A07054	154	A00125	A07127	246
A00125	A07145	475	A00125	A07163	154
A00125	A07570	286	A00125	A08926	224
A00125	A09240	308	A00125	A09390	945
A00125	A09419	251	A00125	A09428	267
A00125	A10148	170	A00125	A10156	191
A00125	A10369	159	A00125	A11038	404
A00125	A11576	160	A00125	A12383	277
A00125	A13110	348	A00125	A13181	307
A00125	A13369	642	A00125	A13387	174
A00125	A13555	274	A00125	A14366	223
A00125	A14452	390	A00125	A15641	224
A00125	A16003	386	A00125	A16039	386
A00126	A00186	174	A00126	A00206	152
A00126	A00224	944	A00126	A00230	490
A00126	A00341	457	A00126	A00388	491
A00126	A00748	173	A00126	A00812	457
A00126	A00933	416	A00126	A00937	343
A00126	A01052	467	A00126	A01071	343
A00126	A01478	452	A00126	A01537	489
A00126	A01558	899	A00126	A01588	899
A00126	A01595	352	A00126	A01660	490
A00126	A01687	486	A00126	A01689	173
A00126	A02030	316	A00126	A02381	490
A00126	A02676	227	A00126	A03225	172
A00126	A03575	271	A00126	A03927	198
A00126	A04080	240	A00126	A05009	245
A00126	A05064	254	A00126	A05185	313
A00126	A05278	258	A00126	A05284	461
A00126	A05454	425	A00126	A05880	343
A00126	A05895	419	A00126	A05987	238
A00126	A06348	491	A00126	A06572	343
A00126	A06597	362	A00126	A07425	432
A00126	A07433	352	A00126	A07593	276
A00126	A08097	343	A00126	A08184	299
A00126	A08196	343	A00126	A08503	343
A00126	A08549	343	A00126	A08560	343
A00126	A08812	343	A00126	A08839	174
A00126	A08844	343	A00126	A08853	393
A00126	A08854	311	A00126	A08855	316

A00126	A11369	899	A00126	A11656	352
A00126	A11766	302	A00126	A11884	191
A00126	A14048	302	A00126	A14084	343
A00126	A14133	340	A00126	A14227	474
A00126	A14279	491	A00126	A14285	343
A00126	A14309	355	A00126	A15533	258
A00126	A16391	208	A00126	A16602	313
A00127	A00150	950	A00127	A00152	538
A00127	A00166	475	A00127	A00167	899
A00127	A00177	491	A00127	A00191	427
A00127	A00192	466	A00127	A00203	137
A00127	A00209	899	A00127	A00211	126
A00127	A00221	489	A00127	A00223	490
A00127	A00245	163	A00127	A00255	205
A00127	A00257	447	A00127	A00270	477
A00127	A00279	326	A00127	A00280	467
A00127	A00284	247	A00127	A00285	455
A00127	A00288	488	A00127	A00289	152
A00127	A00304	491	A00127	A00318	480
A00127	A00324	899	A00127	A00327	228
A00127	A00328	480	A00127	A00334	491
A00127	A00335	491	A00127	A00340	212
A00127	A00356	491	A00127	A00366	481
A00127	A00411	326	A00127	A00414	491
A00127	A00454	424	A00127	A00467	488
A00127	A00469	174	A00127	A00521	417
A00127	A00561	481	A00127	A00571	457
A00127	A00605	480	A00127	A00648	489
A00127	A00651	945	A00127	A00653	417
A00127	A00700	491	A00127	A00701	458
A00127	A00705	396	A00127	A00713	446
A00127	A00763	461	A00127	A00782	456
A00127	A00783	391	A00127	A00787	438
A00127	A00812	491	A00127	A00815	184
A00127	A00839	491	A00127	A00841	486
A00127	A00851	286	A00127	A00870	380
A00127	A00878	327	A00127	A00923	414
A00127	A00966	450	A00127	A00970	479
A00127	A00972	447	A00127	A01012	577
A00127	A01025	177	A00127	A01037	420
A00127	A01042	475	A00127	A01054	427
A00127	A01056	945	A00127	A01072	157
A00127	A01090	491	A00127	A01098	374
A00127	A01112	491	A00127	A01115	457
A00127	A01121	290	A00127	A01127	443
A00127	A01140	491	A00127	A01168	899

A00127	A01196	435	A00127	A01206	313
A00127	A01212	490	A00127	A01216	479
A00127	A01243	249	A00127	A01249	319
A00127	A01261	279	A00127	A01263	262
A00127	A01291	491	A00127	A01306	704
A00127	A01335	491	A00127	A01365	462
A00127	A01400	286	A00127	A01411	168
A00127	A01425	485	A00127	A01431	456
A00127	A01447	281	A00127	A01457	491
A00127	A01461	180	A00127	A01468	491
A00127	A01478	468	A00127	A01492	491
A00127	A01496	945	A00127	A01527	899
A00127	A01560	435	A00127	A01569	162
A00127	A01579	673	A00127	A01586	380
A00127	A01595	199	A00127	A01597	409
A00127	A01601	491	A00127	A01604	63
A00127	A01605	481	A00127	A01606	160
A00127	A01649	446	A00127	A01675	310
A00127	A01718	278	A00127	A01777	157
A00127	A01787	418	A00127	A01796	420
A00127	A01822	472	A00127	A01914	477
A00127	A01995	156	A00127	A02179	409
A00127	A02243	229	A00127	A02265	198
A00127	A02350	491	A00127	A02397	241
A00127	A02405	63	A00127	A02597	135
A00127	A03069	156	A00127	A03110	299
A00127	A03219	154	A00127	A03314	217
A00127	A03332	450	A00127	A03445	491
A00127	A03461	380	A00127	A03639	475
A00127	A03645	157	A00127	A03740	395
A00127	A03752	475	A00127	A03799	369
A00127	A03800	303	A00127	A03809	218
A00127	A03942	277	A00127	A03961	223
A00127	A03963	434	A00127	A03981	609
A00127	A04202	406	A00127	A04230	430
A00127	A04422	411	A00127	A04446	185
A00127	A04490	491	A00127	A04623	374
A00127	A04761	224	A00127	A04843	182
A00127	A05076	340	A00127	A05091	230
A00127	A05373	182	A00127	A05407	347
A00127	A05443	491	A00127	A05449	340
A00127	A05450	238	A00127	A05501	360
A00127	A05863	210	A00127	A05866	180
A00127	A05996	227	A00127	A06037	427
A00127	A06351	435	A00127	A06466	481
A00127	A06534	302	A00127	A06564	407

A00127	A06607	477	A00127	A06814	491
A00127	A06897	394	A00127	A06965	167
A00127	A06973	330	A00127	A07028	210
A00127	A07075	182	A00127	A07306	210
A00127	A07307	174	A00127	A07311	490
A00127	A07323	467	A00127	A07398	491
A00127	A07422	899	A00127	A07451	468
A00127	A07706	199	A00127	A07726	491
A00127	A08052	265	A00127	A08131	475
A00127	A08301	420	A00127	A08309	328
A00127	A08315	313	A00127	A08357	461
A00127	A08358	443	A00127	A08359	491
A00127	A08452	254	A00127	A08457	424
A00127	A08476	232	A00127	A08487	210
A00127	A08685	386	A00127	A08686	490
A00127	A08753	433	A00127	A08761	152
A00127	A08835	467	A00127	A08869	229
A00127	A09062	249	A00127	A09263	188
A00127	A09297	154	A00127	A09308	327
A00127	A09332	241	A00127	A09591	271
A00127	A09620	477	A00127	A09674	198
A00127	A09704	182	A00127	A09741	188
A00127	A09829	428	A00127	A09893	182
A00127	A09960	193	A00127	A10336	489
A00127	A10518	182	A00127	A10532	486
A00127	A10633	375	A00127	A10645	424
A00127	A10748	271	A00127	A11096	217
A00127	A11190	460	A00127	A11263	337
A00127	A11278	458	A00127	A11298	322
A00127	A11333	404	A00127	A11392	236
A00127	A11463	172	A00127	A11846	428
A00127	A11974	193	A00127	A12305	230
A00127	A12310	230	A00127	A12329	302
A00127	A12404	157	A00127	A12653	226
A00127	A12813	163	A00127	A12970	383
A00127	A13107	322	A00127	A13126	152
A00127	A13336	270	A00127	A13340	489
A00127	A13830	278	A00127	A13944	472
A00127	A13989	491	A00127	A13992	441
A00127	A14010	259	A00127	A14038	191
A00127	A14055	463	A00127	A14172	188
A00127	A14210	294	A00127	A14216	545
A00127	A14247	270	A00127	A14443	899
A00127	A14508	326	A00127	A14511	446
A00127	A14566	177	A00127	A15229	330
A00127	A15236	182	A00127	A15268	485

A00127	A15278	172	A00127	A15292	469
A00127	A15348	272	A00127	A15349	413
A00127	A15381	485	A00127	A15416	388
A00127	A15498	191	A00127	A15500	446
A00127	A15660	485	A00127	A15684	491
A00127	A15988	177	A00127	A16107	414
A00127	A16272	241	A00127	A16281	491
A00127	A16437	301	A00127	A16575	274
A00127	A16686	188	A00127	A16778	469
A00128	A00132	474	A00128	A00138	171
A00128	A00167	899	A00128	A00209	899
A00128	A00246	899	A00128	A00697	252
A00128	A01527	899	A00128	A01553	918
A00128	A01692	521	A00128	A03436	183
A00128	A03591	373	A00128	A03643	347
A00128	A03739	162	A00128	A09253	283
A00128	A11322	246	A00129	A00135	152
A00129	A00146	350	A00129	A00153	899
A00129	A00165	319	A00129	A00187	323
A00129	A00188	944	A00129	A00209	900
A00129	A00248	480	A00129	A00621	223
A00129	A01604	172	A00129	A01639	484
A00129	A01664	238	A00129	A01717	180
A00129	A01850	899	A00129	A01900	915
A00129	A01902	259	A00129	A02059	323
A00129	A02578	329	A00129	A02608	335
A00129	A02637	254	A00129	A02889	341
A00129	A03049	252	A00129	A03076	202
A00129	A03224	281	A00129	A05634	337
A00129	A05944	249	A00129	A05953	348
A00129	A06055	278	A00129	A06601	252
A00129	A09256	162	A00129	A09937	308
A00129	A10392	469	A00129	A10580	899
A00129	A13181	249	A00129	A13977	252
A00129	A14376	329	A00129	A14444	167
A00129	A15605	276	A00129	A16083	899
A00130	A00159	899	A00130	A00164	217
A00130	A00184	899	A00130	A00216	217
A00130	A00240	167	A00130	A00347	291
A00130	A00384	440	A00130	A00419	168
A00130	A00446	368	A00130	A00470	488
A00130	A00583	217	A00130	A00852	326
A00130	A00981	502	A00130	A01172	460
A00130	A01372	217	A00130	A01390	898
A00130	A01524	899	A00130	A01548	358
A00130	A01561	63	A00130	A01562	945

A00130	A01628	63	A00130	A01640	899
A00130	A01692	228	A00130	A01705	546
A00130	A01719	63	A00130	A01844	210
A00130	A01907	196	A00130	A02208	269
A00130	A02278	199	A00130	A02366	213
A00130	A02419	77	A00130	A02437	269
A00130	A02598	163	A00130	A02642	941
A00130	A02687	414	A00130	A02803	88
A00130	A02860	183	A00130	A02895	159
A00130	A02942	173	A00130	A03019	63
A00130	A03271	491	A00130	A03416	163
A00130	A03611	234	A00130	A03625	63
A00130	A03873	217	A00130	A03892	141
A00130	A04015	154	A00130	A04104	174
A00130	A04957	486	A00130	A04972	262
A00130	A04986	270	A00130	A05765	410
A00130	A06135	177	A00130	A06159	180
A00130	A06167	160	A00130	A06236	270
A00130	A06289	205	A00130	A06675	492
A00130	A06941	638	A00130	A07311	899
A00130	A07866	301	A00130	A07956	217
A00130	A08096	270	A00130	A08248	217
A00130	A08484	165	A00130	A08627	322
A00130	A08899	899	A00130	A08982	217
A00130	A09084	204	A00130	A09441	157
A00130	A09826	460	A00130	A10174	900
A00130	A10247	383	A00130	A10286	269
A00130	A10766	152	A00130	A10813	307
A00130	A11544	217	A00130	A11649	217
A00130	A12383	229	A00130	A12764	224
A00130	A13250	899	A00130	A13282	301
A00130	A13892	217	A00130	A13977	899
A00130	A14016	338	A00130	A14033	224
A00130	A14362	217	A00130	A15321	502
A00130	A15338	217	A00130	A15495	259
A00130	A15544	157	A00130	A15555	269
A00130	A15564	217	A00130	A15966	191
A00130	A15979	185	A00130	A16255	269
A00130	A16614	217	A00131	A00155	270
A00131	A00196	491	A00131	A00204	215
A00131	A00209	903	A00131	A00216	486
A00131	A00218	491	A00131	A00219	488
A00131	A00264	319	A00131	A00301	216
A00131	A00311	254	A00131	A00338	288
A00131	A00385	223	A00131	A00472	368
A00131	A00656	491	A00131	A00662	408

A00131	A00736	452	A00131	A00815	301
A00131	A01373	196	A00131	A01432	474
A00131	A01527	899	A00131	A01829	188
A00131	A01934	152	A00131	A01939	240
A00131	A01982	475	A00131	A02002	151
A00131	A02010	228	A00131	A02037	240
A00131	A02048	491	A00131	A02049	491
A00131	A02074	241	A00131	A02083	269
A00131	A02145	441	A00131	A02146	467
A00131	A02158	422	A00131	A02186	445
A00131	A02190	255	A00131	A02215	491
A00131	A02226	485	A00131	A02291	374
A00131	A02305	441	A00131	A02306	485
A00131	A02317	196	A00131	A02328	193
A00131	A02385	208	A00131	A02495	283
A00131	A02545	486	A00131	A02759	272
A00131	A02776	173	A00131	A02785	462
A00131	A03050	414	A00131	A03080	489
A00131	A03099	235	A00131	A03319	240
A00131	A03369	213	A00131	A03370	481
A00131	A03371	212	A00131	A03373	400
A00131	A03379	418	A00131	A03390	238
A00131	A03401	457	A00131	A03402	491
A00131	A03403	450	A00131	A03408	435
A00131	A03498	400	A00131	A03725	394
A00131	A03744	452	A00131	A03976	193
A00131	A03989	234	A00131	A04437	400
A00131	A04633	191	A00131	A04672	317
A00131	A04775	255	A00131	A04843	296
A00131	A05087	196	A00131	A05184	185
A00131	A05190	332	A00131	A05337	170
A00131	A05356	491	A00131	A05377	171
A00131	A05457	288	A00131	A05494	408
A00131	A05581	255	A00131	A05731	213
A00131	A05945	490	A00131	A06033	317
A00131	A06331	355	A00131	A06607	491
A00131	A06696	314	A00131	A06797	151
A00131	A07218	473	A00131	A07292	483
A00131	A07376	210	A00131	A07438	411
A00131	A07468	200	A00131	A07471	429
A00131	A07475	407	A00131	A07496	263
A00131	A07509	408	A00131	A07510	432
A00131	A07567	269	A00131	A07634	333
A00131	A07635	167	A00131	A07652	407
A00131	A07653	463	A00131	A07656	447
A00131	A07660	232	A00131	A07673	472

A00131	A07740	446	A00131	A07741	281
A00131	A07754	491	A00131	A07827	332
A00131	A07913	491	A00131	A07914	483
A00131	A07916	414	A00131	A07928	205
A00131	A07950	305	A00131	A07958	260
A00131	A08165	355	A00131	A08173	407
A00131	A08210	224	A00131	A08217	355
A00131	A08218	441	A00131	A08246	443
A00131	A08288	468	A00131	A08347	355
A00131	A08395	332	A00131	A08404	491
A00131	A08785	428	A00131	A09131	255
A00131	A09199	302	A00131	A09394	199
A00131	A09807	332	A00131	A09831	269
A00131	A09928	205	A00131	A10021	234
A00131	A10436	355	A00131	A10563	327
A00131	A10944	281	A00131	A10951	355
A00131	A11428	251	A00131	A11442	420
A00131	A11552	200	A00131	A11738	170
A00131	A12347	281	A00131	A12641	420
A00131	A12758	355	A00131	A13872	152
A00131	A15803	281	A00132	A00138	529
A00132	A00141	313	A00132	A00146	346
A00132	A00147	435	A00132	A00150	199
A00132	A00153	899	A00132	A00157	899
A00132	A00159	199	A00132	A00165	424
A00132	A00167	904	A00132	A00169	271
A00132	A00183	424	A00132	A00209	908
A00132	A00224	624	A00132	A00225	490
A00132	A00226	375	A00132	A00234	174
A00132	A00239	162	A00132	A00250	938
A00132	A00254	490	A00132	A00257	698
A00132	A00265	457	A00132	A00270	527
A00132	A00271	899	A00132	A00318	417
A00132	A00322	307	A00132	A00340	466
A00132	A00440	369	A00132	A00531	549
A00132	A00769	246	A00132	A00841	402
A00132	A00871	179	A00132	A00887	474
A00132	A00909	307	A00132	A00962	271
A00132	A01141	245	A00132	A01157	369
A00132	A01232	307	A00132	A01311	202
A00132	A01358	168	A00132	A01473	207
A00132	A01522	286	A00132	A01558	945
A00132	A01560	491	A00132	A01562	218
A00132	A01588	994	A00132	A01615	485
A00132	A01692	951	A00132	A01694	245
A00132	A01914	333	A00132	A02117	235

A00132	A02164	193	A00132	A02434	167
A00132	A02702	162	A00132	A03575	154
A00132	A03591	462	A00132	A04012	390
A00132	A04072	180	A00132	A04077	171
A00132	A04337	172	A00132	A04449	155
A00132	A04540	218	A00132	A04610	199
A00132	A04949	369	A00132	A05164	263
A00132	A05418	179	A00132	A05603	185
A00132	A06554	307	A00132	A06597	581
A00132	A06599	271	A00132	A06721	208
A00132	A07309	404	A00132	A07311	527
A00132	A07422	899	A00132	A07552	369
A00132	A08171	475	A00132	A08172	486
A00132	A08299	256	A00132	A08309	401
A00132	A08453	316	A00132	A08459	219
A00132	A08571	455	A00132	A08635	278
A00132	A08685	372	A00132	A08686	483
A00132	A08753	347	A00132	A08860	729
A00132	A09218	447	A00132	A09253	486
A00132	A09401	484	A00132	A09585	160
A00132	A10735	324	A00132	A11207	152
A00132	A11278	440	A00132	A11298	205
A00132	A11321	358	A00132	A11333	332
A00132	A11487	296	A00132	A12156	256
A00132	A12478	388	A00132	A12619	219
A00132	A12620	256	A00132	A12824	236
A00132	A13511	488	A00132	A13551	199
A00132	A13928	152	A00132	A14331	199
A00132	A14334	256	A00132	A14699	369
A00132	A15295	260	A00132	A15345	173
A00132	A15347	215	A00132	A15506	483
A00132	A15638	491	A00132	A16281	167
A00132	A16682	177	A00132	A16766	170
A00132	A16908	152	A00132	A17209	219
A00133	A00134	899	A00133	A00136	208
A00133	A00140	930	A00133	A00141	899
A00133	A00150	899	A00133	A00157	899
A00133	A00158	63	A00133	A00163	920
A00133	A00165	491	A00133	A00167	902
A00133	A00171	391	A00133	A00178	899
A00133	A00179	174	A00133	A00184	899
A00133	A00187	462	A00133	A00190	899
A00133	A00203	899	A00133	A00209	904
A00133	A00224	899	A00133	A00230	899
A00133	A00246	899	A00133	A00254	899
A00133	A00305	183	A00133	A00324	904

A00133	A00378	475	A00133	A00887	899
A00133	A00997	185	A00133	A01002	899
A00133	A01148	377	A00133	A01178	283
A00133	A01291	899	A00133	A01492	899
A00133	A01512	930	A00133	A01524	899
A00133	A01527	899	A00133	A01553	899
A00133	A01554	910	A00133	A01558	899
A00133	A01562	899	A00133	A01588	899
A00133	A01591	899	A00133	A01596	356
A00133	A01601	899	A00133	A01602	899
A00133	A01615	899	A00133	A01628	899
A00133	A01636	899	A00133	A01639	902
A00133	A01651	899	A00133	A01680	899
A00133	A01688	899	A00133	A01698	102
A00133	A01708	899	A00133	A01709	899
A00133	A01920	899	A00133	A02343	251
A00133	A02411	899	A00133	A03699	174
A00133	A04320	899	A00133	A04449	127
A00133	A04699	899	A00133	A04744	277
A00133	A04807	899	A00133	A05082	899
A00133	A05169	256	A00133	A05216	899
A00133	A05312	338	A00133	A06216	196
A00133	A06277	899	A00133	A06305	899
A00133	A06601	246	A00133	A07311	899
A00133	A07422	899	A00133	A08878	899
A00133	A09162	251	A00133	A09319	899
A00133	A10580	899	A00133	A10623	905
A00133	A10743	182	A00133	A10808	936
A00133	A11125	899	A00133	A11379	165
A00133	A11440	228	A00133	A11604	899
A00133	A11983	899	A00133	A12373	199
A00133	A12664	899	A00133	A13971	277
A00133	A13985	899	A00133	A13989	899
A00133	A14005	929	A00133	A14044	899
A00133	A14055	899	A00133	A14059	899
A00133	A16786	184	A00133	A17160	899
A00134	A00184	899	A00134	A00209	213
A00134	A00224	899	A00134	A00246	899
A00134	A01512	914	A00134	A01524	899
A00134	A01527	899	A00134	A01558	899
A00134	A01560	899	A00134	A01562	899
A00134	A01574	174	A00134	A01588	899
A00134	A01628	914	A00134	A01639	484
A00134	A01709	908	A00134	A04699	899
A00134	A06550	235	A00134	A09162	422
A00134	A09542	899	A00134	A11125	899

A00134	A12705	899	A00134	A13985	899
A00135	A00147	430	A00135	A00159	899
A00135	A00165	390	A00135	A00190	944
A00135	A00209	899	A00135	A00224	165
A00135	A00246	899	A00135	A00262	260
A00135	A00265	469	A00135	A00324	302
A00135	A00416	488	A00135	A00450	458
A00135	A00546	217	A00135	A00625	491
A00135	A00679	163	A00135	A00786	321
A00135	A00877	491	A00135	A00971	213
A00135	A01017	163	A00135	A01036	488
A00135	A01217	165	A00135	A01378	472
A00135	A01451	174	A00135	A01452	281
A00135	A01454	333	A00135	A01507	395
A00135	A01524	989	A00135	A01527	899
A00135	A01568	490	A00135	A01580	326
A00135	A01606	480	A00135	A01687	286
A00135	A01698	251	A00135	A01724	135
A00135	A01730	433	A00135	A01732	160
A00135	A01736	310	A00135	A01742	401
A00135	A01749	177	A00135	A01769	465
A00135	A01850	260	A00135	A02400	230
A00135	A02698	491	A00135	A02715	118
A00135	A02724	353	A00135	A02822	162
A00135	A02908	899	A00135	A02982	940
A00135	A03304	398	A00135	A03632	171
A00135	A03983	230	A00135	A04249	329
A00135	A04449	491	A00135	A04905	160
A00135	A06277	235	A00135	A06601	902
A00135	A07227	361	A00135	A07242	491
A00135	A07483	475	A00135	A07495	491
A00135	A07665	470	A00135	A07788	226
A00135	A07816	413	A00135	A07893	486
A00135	A07944	216	A00135	A08022	302
A00135	A08056	338	A00135	A08536	162
A00135	A08934	467	A00135	A08962	252
A00135	A09162	255	A00135	A09624	447
A00135	A09804	916	A00135	A09842	408
A00135	A11023	461	A00135	A11149	414
A00135	A11150	455	A00135	A11180	413
A00135	A11281	458	A00135	A11293	480
A00135	A11346	152	A00135	A11389	201
A00135	A11547	452	A00135	A11665	470
A00135	A11940	165	A00135	A11983	291
A00135	A12047	473	A00135	A12048	272
A00135	A12051	260	A00135	A12091	272

A00135	A12102	272	A00135	A12215	185
A00135	A12420	899	A00135	A12421	916
A00135	A13213	208	A00135	A13511	271
A00135	A13667	485	A00135	A13927	452
A00135	A14002	160	A00135	A14059	256
A00135	A14260	162	A00135	A14389	216
A00135	A14443	900	A00135	A14539	399
A00135	A14925	400	A00135	A15035	399
A00135	A16343	221	A00135	A16647	226
A00135	A16806	226	A00135	A17019	230
A00135	A17030	168	A00136	A00137	414
A00136	A00138	286	A00136	A00139	235
A00136	A00140	481	A00136	A00157	486
A00136	A00159	110	A00136	A00161	165
A00136	A00162	614	A00136	A00165	63
A00136	A00175	154	A00136	A00184	159
A00136	A00185	63	A00136	A00187	457
A00136	A00207	63	A00136	A00208	308
A00136	A00217	469	A00136	A00240	217
A00136	A00267	302	A00136	A00273	491
A00136	A00275	168	A00136	A01010	193
A00136	A01512	259	A00136	A01553	572
A00136	A01554	468	A00136	A01560	263
A00136	A01561	293	A00136	A01562	259
A00136	A01605	402	A00136	A01628	454
A00136	A01639	204	A00136	A01640	238
A00136	A01667	176	A00136	A01680	420
A00136	A01692	258	A00136	A01697	374
A00136	A01702	319	A00136	A01708	450
A00136	A01709	452	A00136	A01718	344
A00136	A01719	409	A00136	A01907	252
A00136	A02413	179	A00136	A02616	232
A00136	A02669	241	A00136	A02803	73
A00136	A03213	63	A00136	A03273	63
A00136	A03417	152	A00136	A04015	162
A00136	A04439	306	A00136	A04452	328
A00136	A04968	283	A00136	A05878	212
A00136	A07051	216	A00136	A07174	232
A00136	A07311	278	A00136	A08077	180
A00136	A08194	154	A00136	A08238	240
A00136	A09162	408	A00136	A09504	219
A00136	A09577	68	A00136	A09643	276
A00136	A10808	146	A00136	A11278	200
A00136	A11369	63	A00136	A11767	235
A00136	A12383	438	A00136	A13729	229
A00136	A13872	317	A00136	A14008	146

A00136	A14038	232	A00136	A14303	171
A00136	A17014	383	A00137	A00140	307
A00137	A00157	190	A00137	A00187	224
A00137	A00217	226	A00137	A00267	193
A00137	A00273	205	A00137	A00284	171
A00137	A01358	154	A00137	A01554	63
A00137	A01560	364	A00137	A01561	463
A00137	A01562	119	A00137	A01570	205
A00137	A01605	245	A00137	A01628	216
A00137	A01692	63	A00137	A01700	157
A00137	A01702	168	A00137	A02088	202
A00137	A02411	87	A00137	A02862	207
A00137	A06727	252	A00137	A07311	436
A00137	A08171	210	A00137	A08172	162
A00137	A09568	139	A00137	A09894	323
A00137	A11820	254	A00137	A12383	263
A00137	A14452	63	A00137	A14925	154
A00137	A15726	63	A00138	A00140	63
A00138	A00141	491	A00138	A00145	491
A00138	A00146	274	A00138	A00147	391
A00138	A00152	761	A00138	A00153	340
A00138	A00156	352	A00138	A00157	99
A00138	A00158	483	A00138	A00159	785
A00138	A00160	63	A00138	A00161	491
A00138	A00162	446	A00138	A00163	527
A00138	A00167	179	A00138	A00183	658
A00138	A00185	490	A00138	A00186	63
A00138	A00189	765	A00138	A00201	323
A00138	A00205	208	A00138	A00207	751
A00138	A00208	490	A00138	A00214	554
A00138	A00224	792	A00138	A00225	488
A00138	A00232	484	A00138	A00233	477
A00138	A00234	562	A00138	A00235	199
A00138	A00236	224	A00138	A00246	956
A00138	A00247	319	A00138	A00254	785
A00138	A00256	369	A00138	A00257	491
A00138	A00261	831	A00138	A00265	468
A00138	A00270	706	A00138	A00292	449
A00138	A00316	491	A00138	A00340	432
A00138	A00351	218	A00138	A00378	410
A00138	A00418	273	A00138	A00448	241
A00138	A00464	201	A00138	A00471	183
A00138	A00552	332	A00138	A00561	218
A00138	A00641	296	A00138	A00654	199
A00138	A00662	152	A00138	A00691	191
A00138	A00696	157	A00138	A00697	193

A00138	A00724	462	A00138	A00744	184
A00138	A00757	318	A00138	A00769	401
A00138	A00799	405	A00138	A00841	491
A00138	A00875	321	A00138	A00879	384
A00138	A00909	484	A00138	A00916	520
A00138	A00935	171	A00138	A00981	538
A00138	A01017	218	A00138	A01020	343
A00138	A01061	274	A00138	A01150	589
A00138	A01173	475	A00138	A01212	424
A00138	A01234	489	A00138	A01329	233
A00138	A01396	405	A00138	A01419	308
A00138	A01425	446	A00138	A01452	490
A00138	A01458	491	A00138	A01473	491
A00138	A01474	480	A00138	A01492	536
A00138	A01524	631	A00138	A01527	949
A00138	A01553	485	A00138	A01558	572
A00138	A01560	558	A00138	A01572	526
A00138	A01588	489	A00138	A01610	452
A00138	A01615	490	A00138	A01640	530
A00138	A01692	524	A00138	A01697	63
A00138	A01843	63	A00138	A01844	63
A00138	A01879	63	A00138	A01880	63
A00138	A01887	110	A00138	A01888	63
A00138	A01894	402	A00138	A01908	486
A00138	A01914	687	A00138	A01995	562
A00138	A02076	205	A00138	A02163	578
A00138	A02183	490	A00138	A02223	171
A00138	A02331	491	A00138	A02342	414
A00138	A02372	432	A00138	A02382	392
A00138	A02411	246	A00138	A02425	165
A00138	A02630	117	A00138	A02732	63
A00138	A02734	259	A00138	A02803	490
A00138	A02864	454	A00138	A03010	63
A00138	A03110	241	A00138	A03225	326
A00138	A03271	479	A00138	A03292	231
A00138	A03327	491	A00138	A03436	477
A00138	A03470	160	A00138	A03478	433
A00138	A03512	363	A00138	A03569	393
A00138	A03591	525	A00138	A03598	93
A00138	A03784	369	A00138	A03803	474
A00138	A03809	596	A00138	A03817	469
A00138	A03821	489	A00138	A03962	196
A00138	A04012	531	A00138	A04033	371
A00138	A04060	352	A00138	A04066	171
A00138	A04161	288	A00138	A04288	238
A00138	A04314	469	A00138	A04323	296

A00138	A04536	179	A00138	A04914	314
A00138	A05212	267	A00138	A05288	466
A00138	A05453	257	A00138	A05638	384
A00138	A05880	481	A00138	A05985	447
A00138	A06197	399	A00138	A06258	165
A00138	A06273	339	A00138	A06400	265
A00138	A06543	322	A00138	A06572	286
A00138	A06597	425	A00138	A07004	501
A00138	A07167	162	A00138	A07200	333
A00138	A07311	580	A00138	A07409	491
A00138	A07524	473	A00138	A07726	441
A00138	A07732	474	A00138	A07803	218
A00138	A07924	286	A00138	A08118	303
A00138	A08185	204	A00138	A08196	224
A00138	A08280	172	A00138	A08345	422
A00138	A08450	243	A00138	A08571	536
A00138	A08608	234	A00138	A08701	447
A00138	A08860	734	A00138	A08878	293
A00138	A09176	208	A00138	A09181	273
A00138	A09220	452	A00138	A09341	340
A00138	A09401	382	A00138	A09542	289
A00138	A09764	218	A00138	A09888	191
A00138	A09937	137	A00138	A10014	63
A00138	A10365	63	A00138	A10510	347
A00138	A10557	202	A00138	A10666	191
A00138	A10711	191	A00138	A10721	257
A00138	A10735	491	A00138	A10744	168
A00138	A10766	157	A00138	A10801	330
A00138	A10854	218	A00138	A10899	63
A00138	A11124	250	A00138	A11369	592
A00138	A11487	543	A00138	A11591	316
A00138	A11983	678	A00138	A12009	63
A00138	A12548	294	A00138	A12660	176
A00138	A12835	200	A00138	A13216	193
A00138	A13391	63	A00138	A13511	488
A00138	A13605	63	A00138	A13743	193
A00138	A13910	208	A00138	A13975	491
A00138	A13980	479	A00138	A13986	475
A00138	A13989	527	A00138	A14006	467
A00138	A14007	321	A00138	A14019	63
A00138	A14034	63	A00138	A14187	291
A00138	A14188	378	A00138	A14190	313
A00138	A14198	491	A00138	A14208	602
A00138	A14215	63	A00138	A14299	377
A00138	A14364	436	A00138	A14426	441
A00138	A14430	481	A00138	A14433	317

A00138	A14440	380	A00138	A14444	906
A00138	A14452	286	A00138	A14635	368
A00138	A14986	177	A00138	A15294	747
A00138	A15386	218	A00138	A15506	819
A00138	A15692	115	A00138	A15726	491
A00138	A15854	218	A00138	A15935	254
A00138	A16048	361	A00138	A16084	63
A00138	A16529	182	A00138	A16994	521
A00138	A17102	252	A00139	A00157	257
A00139	A00180	313	A00139	A00187	294
A00139	A00912	221	A00139	A01527	899
A00139	A01562	63	A00139	A01692	63
A00139	A01789	361	A00139	A05128	361
A00139	A06615	198	A00139	A07163	200
A00139	A07311	63	A00139	A08321	154
A00139	A09127	366	A00139	A10247	157
A00139	A12383	603	A00139	A14268	215
A00140	A00151	899	A00140	A00153	899
A00140	A00157	933	A00140	A00161	899
A00140	A00162	899	A00140	A00165	63
A00140	A00167	899	A00140	A00173	900
A00140	A00184	928	A00140	A00187	340
A00140	A00205	910	A00140	A00208	77
A00140	A00209	899	A00140	A00217	401
A00140	A00224	899	A00140	A00226	906
A00140	A00230	899	A00140	A00244	215
A00140	A00246	901	A00140	A00248	899
A00140	A00250	899	A00140	A00266	899
A00140	A00267	205	A00140	A00273	475
A00140	A01512	927	A00140	A01524	899
A00140	A01527	899	A00140	A01553	899
A00140	A01554	943	A00140	A01558	899
A00140	A01560	63	A00140	A01588	899
A00140	A01605	263	A00140	A01615	899
A00140	A01623	183	A00140	A01628	236
A00140	A01636	899	A00140	A01639	900
A00140	A01651	899	A00140	A01697	121
A00140	A01702	185	A00140	A01709	484
A00140	A01718	913	A00140	A01844	63
A00140	A02825	914	A00140	A03019	63
A00140	A03331	157	A00140	A03575	899
A00140	A06601	179	A00140	A06648	165
A00140	A06708	351	A00140	A07311	899
A00140	A07422	899	A00140	A08571	899
A00140	A08858	899	A00140	A09162	394
A00140	A09319	906	A00140	A09577	914

A00140	A09643	180	A00140	A10392	911
A00140	A10580	902	A00140	A10601	899
A00140	A10623	899	A00140	A10808	182
A00140	A10934	221	A00140	A11125	899
A00140	A11767	268	A00140	A12383	177
A00140	A13729	380	A00140	A13980	908
A00140	A13985	899	A00140	A14030	899
A00140	A14157	899	A00140	A14539	299
A00140	A16095	154	A00141	A00145	229
A00141	A00152	479	A00141	A00157	899
A00141	A00158	269	A00141	A00159	389
A00141	A00160	435	A00141	A00169	480
A00141	A00183	173	A00141	A00185	165
A00141	A00188	283	A00141	A00189	491
A00141	A00220	449	A00141	A00224	462
A00141	A00230	274	A00141	A00239	162
A00141	A00240	468	A00141	A00246	926
A00141	A00248	470	A00141	A00254	223
A00141	A00274	324	A00141	A00669	491
A00141	A00754	299	A00141	A00887	400
A00141	A00914	302	A00141	A00997	170
A00141	A01052	360	A00141	A01070	899
A00141	A01146	386	A00141	A01512	899
A00141	A01524	944	A00141	A01527	945
A00141	A01548	183	A00141	A01557	160
A00141	A01560	899	A00141	A01588	297
A00141	A01615	188	A00141	A01654	486
A00141	A01688	260	A00141	A01697	899
A00141	A01709	901	A00141	A01828	263
A00141	A01835	205	A00141	A01846	157
A00141	A01870	404	A00141	A01879	383
A00141	A01880	475	A00141	A01887	323
A00141	A01888	579	A00141	A01889	171
A00141	A01891	249	A00141	A01902	291
A00141	A01907	70	A00141	A01946	154
A00141	A02141	174	A00141	A02148	224
A00141	A02178	162	A00141	A02304	263
A00141	A02337	462	A00141	A02340	98
A00141	A02370	385	A00141	A02382	455
A00141	A02383	269	A00141	A02439	238
A00141	A02510	205	A00141	A02578	297
A00141	A02592	188	A00141	A02604	223
A00141	A02617	328	A00141	A02630	251
A00141	A02637	551	A00141	A02654	174
A00141	A02657	413	A00141	A02659	259
A00141	A02668	171	A00141	A02681	313

A00141	A02695	177	A00141	A02708	223
A00141	A02711	491	A00141	A02730	212
A00141	A02749	413	A00141	A02788	88
A00141	A02795	281	A00141	A02798	216
A00141	A02808	263	A00141	A02841	263
A00141	A02851	297	A00141	A02854	460
A00141	A02863	440	A00141	A02864	389
A00141	A02874	172	A00141	A02886	369
A00141	A02896	448	A00141	A02917	443
A00141	A02935	355	A00141	A02945	433
A00141	A03010	457	A00141	A03019	63
A00141	A03049	358	A00141	A03163	195
A00141	A03181	409	A00141	A03204	346
A00141	A03259	551	A00141	A03552	171
A00141	A03611	221	A00141	A03744	171
A00141	A03790	252	A00141	A03807	213
A00141	A03817	296	A00141	A03845	160
A00141	A03854	475	A00141	A03966	193
A00141	A04062	246	A00141	A04176	466
A00141	A04248	489	A00141	A04263	439
A00141	A04265	157	A00141	A04320	491
A00141	A04426	328	A00141	A04432	269
A00141	A04450	208	A00141	A04473	167
A00141	A04518	316	A00141	A04536	396
A00141	A04734	401	A00141	A04736	223
A00141	A04748	228	A00141	A04755	185
A00141	A04807	491	A00141	A04865	469
A00141	A04866	208	A00141	A04968	213
A00141	A05064	899	A00141	A05082	491
A00141	A05278	396	A00141	A05329	196
A00141	A05558	241	A00141	A05621	160
A00141	A05701	433	A00141	A05741	177
A00141	A05815	218	A00141	A05940	165
A00141	A05944	488	A00141	A05985	241
A00141	A06054	223	A00141	A06055	385
A00141	A06107	414	A00141	A06142	299
A00141	A06344	165	A00141	A06404	316
A00141	A06422	408	A00141	A06451	218
A00141	A06779	201	A00141	A06780	208
A00141	A06818	384	A00141	A07135	229
A00141	A07148	160	A00141	A07157	480
A00141	A07158	485	A00141	A07161	462
A00141	A07166	265	A00141	A07173	257
A00141	A07311	199	A00141	A07338	269
A00141	A07390	283	A00141	A07683	258
A00141	A08133	199	A00141	A08193	199

A00141	A08360	391	A00141	A08497	258
A00141	A08623	299	A00141	A08644	269
A00141	A08658	213	A00141	A08711	228
A00141	A08729	446	A00141	A09124	457
A00141	A09803	293	A00141	A10504	185
A00141	A10721	177	A00141	A10852	299
A00141	A10894	405	A00141	A10901	223
A00141	A11125	899	A00141	A11206	899
A00141	A11369	380	A00141	A11543	278
A00141	A11604	199	A00141	A11771	180
A00141	A11854	177	A00141	A11901	299
A00141	A11983	899	A00141	A12560	299
A00141	A12720	281	A00141	A12900	281
A00141	A13181	480	A00141	A13344	281
A00141	A13860	322	A00141	A13981	899
A00141	A14029	326	A00141	A14132	151
A00141	A14236	243	A00141	A14299	475
A00141	A14373	188	A00141	A14432	173
A00141	A15293	899	A00141	A15294	899
A00141	A15394	190	A00141	A15403	180
A00141	A15605	316	A00141	A15608	229
A00141	A15952	299	A00141	A15981	154
A00141	A16328	465	A00141	A16839	446
A00142	A00143	945	A00142	A00152	900
A00142	A00212	939	A00142	A00215	944
A00142	A00224	473	A00142	A00351	377
A00142	A00574	474	A00142	A00654	418
A00142	A00786	249	A00142	A01461	318
A00142	A01573	418	A00142	A01741	343
A00142	A01859	273	A00142	A01866	207
A00142	A01926	262	A00142	A02046	165
A00142	A02353	306	A00142	A02695	490
A00142	A02718	317	A00142	A02723	353
A00142	A03004	188	A00142	A03059	229
A00142	A03238	208	A00142	A03431	479
A00142	A03477	945	A00142	A03500	263
A00142	A03861	340	A00142	A03890	201
A00142	A04193	193	A00142	A04784	380
A00142	A04834	321	A00142	A04849	323
A00142	A04850	491	A00142	A04888	440
A00142	A05277	218	A00142	A05356	213
A00142	A05515	200	A00142	A05937	226
A00142	A06170	317	A00142	A06359	473
A00142	A06473	475	A00142	A06577	332
A00142	A06597	191	A00142	A06634	240
A00142	A06775	201	A00142	A07128	172

A00142	A07256	240	A00142	A08785	259
A00142	A09236	205	A00142	A09913	931
A00142	A10229	355	A00142	A10952	355
A00142	A11683	352	A00142	A13234	383
A00142	A13330	172	A00142	A14247	193
A00142	A16048	162	A00142	A16799	229
A00143	A00168	899	A00143	A00200	899
A00143	A00212	924	A00143	A00215	943
A00143	A00224	162	A00143	A00461	246
A00143	A00574	481	A00143	A00614	483
A00143	A00654	470	A00143	A00739	318
A00143	A01667	312	A00143	A01859	199
A00143	A01866	260	A00143	A02109	223
A00143	A02488	179	A00143	A02695	484
A00143	A02719	201	A00143	A02723	390
A00143	A03053	243	A00143	A03431	477
A00143	A03477	255	A00143	A04784	451
A00143	A04834	366	A00143	A04850	491
A00143	A04995	215	A00143	A06086	193
A00143	A06359	291	A00143	A06473	477
A00143	A06608	899	A00143	A06756	195
A00143	A07109	380	A00143	A07542	198
A00143	A07543	204	A00143	A07710	221
A00143	A09878	229	A00143	A10229	383
A00143	A10701	180	A00143	A16134	252
A00144	A00166	307	A00144	A00167	271
A00144	A00221	661	A00144	A00232	173
A00144	A00385	210	A00144	A00483	173
A00144	A00561	210	A00144	A00719	165
A00144	A00752	329	A00144	A01277	246
A00144	A01500	414	A00144	A01759	230
A00144	A01841	184	A00144	A01856	245
A00144	A01970	288	A00144	A02048	350
A00144	A02092	313	A00144	A02099	321
A00144	A02116	156	A00144	A02143	165
A00144	A02157	185	A00144	A02158	152
A00144	A02214	286	A00144	A02330	185
A00144	A02336	307	A00144	A02338	273
A00144	A02353	213	A00144	A02355	307
A00144	A02492	187	A00144	A02608	313
A00144	A02727	400	A00144	A02738	201
A00144	A03037	404	A00144	A03183	232
A00144	A03314	208	A00144	A03399	198
A00144	A03477	391	A00144	A03719	196
A00144	A03744	414	A00144	A04202	170
A00144	A04361	216	A00144	A04422	258

A00144	A04428	335	A00144	A04879	296
A00144	A04928	271	A00144	A04965	196
A00144	A05020	260	A00144	A05027	601
A00144	A05439	167	A00144	A05457	362
A00144	A05473	374	A00144	A05688	380
A00144	A05689	367	A00144	A06067	240
A00144	A06346	163	A00144	A06609	246
A00144	A06956	321	A00144	A07409	213
A00144	A09028	272	A00144	A09205	274
A00144	A09211	283	A00144	A09559	348
A00144	A14007	172	A00144	A14196	263
A00144	A14350	367	A00144	A14366	369
A00144	A14368	285	A00144	A14369	382
A00144	A14374	159	A00144	A14391	163
A00144	A14554	343	A00144	A15329	288
A00144	A15419	184	A00144	A15422	380
A00144	A15795	352	A00144	A15820	293
A00145	A00152	665	A00145	A00153	910
A00145	A00158	758	A00145	A00159	996
A00145	A00160	491	A00145	A00161	63
A00145	A00162	286	A00145	A00163	249
A00145	A00189	490	A00145	A00190	488
A00145	A00205	85	A00145	A00207	480
A00145	A00208	63	A00145	A00224	960
A00145	A00232	263	A00145	A00234	159
A00145	A00246	907	A00145	A00294	899
A00145	A00316	160	A00145	A00370	899
A00145	A00533	176	A00145	A00930	193
A00145	A01052	899	A00145	A01473	491
A00145	A01474	190	A00145	A01524	441
A00145	A01527	941	A00145	A01553	63
A00145	A01554	255	A00145	A01558	899
A00145	A01560	440	A00145	A01572	213
A00145	A01588	899	A00145	A01914	243
A00145	A02076	467	A00145	A02639	156
A00145	A02711	480	A00145	A02731	63
A00145	A02732	127	A00145	A02865	208
A00145	A03010	477	A00145	A03019	63
A00145	A03141	438	A00145	A03178	188
A00145	A03569	367	A00145	A03687	255
A00145	A03780	174	A00145	A03821	461
A00145	A04011	183	A00145	A04138	170
A00145	A04221	243	A00145	A04320	159
A00145	A05704	229	A00145	A06414	176
A00145	A06610	151	A00145	A07062	273
A00145	A07165	900	A00145	A09888	356

A00145	A10062	234	A00145	A10514	217
A00145	A10544	168	A00145	A10721	469
A00145	A10735	84	A00145	A10899	440
A00145	A11369	457	A00145	A11487	246
A00145	A11591	310	A00145	A11885	465
A00145	A12383	291	A00145	A13491	260
A00145	A13581	176	A00145	A13975	726
A00145	A13980	479	A00145	A13986	290
A00145	A14208	481	A00145	A14250	412
A00145	A14452	184	A00145	A14825	215
A00145	A15263	316	A00145	A15726	469
A00145	A15896	217	A00145	A15952	316
A00145	A16994	353	A00145	A17212	254
A00146	A00147	399	A00146	A00153	899
A00146	A00157	899	A00146	A00165	770
A00146	A00167	899	A00146	A00209	899
A00146	A00224	903	A00146	A00225	441
A00146	A00226	296	A00146	A00230	899
A00146	A00234	168	A00146	A00243	217
A00146	A00250	976	A00146	A00254	454
A00146	A00257	460	A00146	A00265	408
A00146	A00736	162	A00146	A01410	362
A00146	A01527	899	A00146	A01553	63
A00146	A01558	899	A00146	A01596	712
A00146	A01639	905	A00146	A01730	215
A00146	A01768	217	A00146	A01898	156
A00146	A02678	319	A00146	A02702	154
A00146	A02737	461	A00146	A02825	296
A00146	A02993	263	A00146	A03213	93
A00146	A04012	202	A00146	A04288	193
A00146	A04537	266	A00146	A04578	385
A00146	A04639	191	A00146	A05064	159
A00146	A05111	396	A00146	A06654	200
A00146	A07422	899	A00146	A08571	899
A00146	A09218	212	A00146	A09383	273
A00146	A09502	227	A00146	A09543	310
A00146	A09577	66	A00146	A10580	942
A00146	A10596	200	A00146	A10623	920
A00146	A10735	249	A00146	A10756	196
A00146	A11601	251	A00146	A11639	348
A00146	A11731	221	A00146	A12341	899
A00146	A12383	409	A00146	A13244	317
A00146	A13511	441	A00146	A14187	188
A00146	A14443	936	A00146	A14444	899
A00146	A14666	243	A00146	A15064	174
A00146	A15321	167	A00146	A15555	232

A00147	A00152	188	A00147	A00154	168
A00147	A00163	339	A00147	A00169	223
A00147	A00171	230	A00147	A00190	486
A00147	A00200	420	A00147	A00202	327
A00147	A00219	213	A00147	A00223	184
A00147	A00236	251	A00147	A00248	332
A00147	A00250	173	A00147	A00254	419
A00147	A00257	396	A00147	A00264	257
A00147	A00265	945	A00147	A00266	219
A00147	A00312	491	A00147	A00314	154
A00147	A00574	160	A00147	A00660	202
A00147	A00680	286	A00147	A00688	152
A00147	A00747	208	A00147	A00912	172
A00147	A01157	215	A00147	A01211	174
A00147	A01276	160	A00147	A01354	173
A00147	A01543	338	A00147	A01566	469
A00147	A01582	466	A00147	A01600	473
A00147	A01607	249	A00147	A01632	491
A00147	A01676	945	A00147	A01685	174
A00147	A01729	299	A00147	A01730	945
A00147	A01740	491	A00147	A01741	351
A00147	A01755	483	A00147	A01760	182
A00147	A01768	168	A00147	A01770	462
A00147	A01775	205	A00147	A01776	230
A00147	A01841	159	A00147	A01877	465
A00147	A01896	272	A00147	A01915	165
A00147	A01916	374	A00147	A02068	263
A00147	A02082	196	A00147	A02085	288
A00147	A02310	371	A00147	A02344	173
A00147	A02407	266	A00147	A02434	172
A00147	A02494	461	A00147	A02501	454
A00147	A02511	307	A00147	A02564	210
A00147	A02583	193	A00147	A02618	176
A00147	A02664	240	A00147	A02687	434
A00147	A02695	296	A00147	A02702	227
A00147	A02703	484	A00147	A02736	419
A00147	A02737	418	A00147	A02744	168
A00147	A02786	165	A00147	A02805	198
A00147	A02816	486	A00147	A02825	238
A00147	A02827	363	A00147	A02830	491
A00147	A02836	188	A00147	A02867	288
A00147	A02877	491	A00147	A02878	491
A00147	A02904	268	A00147	A02921	243
A00147	A02927	458	A00147	A02939	413
A00147	A02943	243	A00147	A02962	291
A00147	A03001	193	A00147	A03082	160

A00147	A03112	447	A00147	A03126	278
A00147	A03211	491	A00147	A03223	212
A00147	A03276	456	A00147	A03321	63
A00147	A03415	152	A00147	A03447	165
A00147	A03451	447	A00147	A03591	945
A00147	A03611	82	A00147	A03613	227
A00147	A03839	179	A00147	A04229	207
A00147	A04235	288	A00147	A04282	279
A00147	A04305	433	A00147	A04308	441
A00147	A04310	235	A00147	A04342	195
A00147	A04493	243	A00147	A04533	279
A00147	A04543	163	A00147	A04576	256
A00147	A04675	262	A00147	A04737	355
A00147	A04815	217	A00147	A04944	400
A00147	A04964	344	A00147	A04965	356
A00147	A04972	191	A00147	A05106	210
A00147	A05247	154	A00147	A05250	360
A00147	A05288	491	A00147	A05296	235
A00147	A05315	204	A00147	A05462	483
A00147	A05490	288	A00147	A05645	170
A00147	A05703	288	A00147	A05866	170
A00147	A06010	168	A00147	A06078	179
A00147	A06151	389	A00147	A06152	262
A00147	A06400	258	A00147	A06442	341
A00147	A06535	360	A00147	A06607	436
A00147	A06608	394	A00147	A06626	243
A00147	A06648	191	A00147	A06650	308
A00147	A06666	371	A00147	A06688	481
A00147	A06871	301	A00147	A07016	205
A00147	A07066	256	A00147	A07163	489
A00147	A07179	270	A00147	A07217	208
A00147	A07399	311	A00147	A07684	157
A00147	A07856	240	A00147	A08135	305
A00147	A08417	333	A00147	A08655	180
A00147	A09058	323	A00147	A09218	296
A00147	A09247	152	A00147	A09248	160
A00147	A09402	288	A00147	A09409	288
A00147	A09441	217	A00147	A09457	301
A00147	A09575	278	A00147	A09770	404
A00147	A09976	371	A00147	A10082	256
A00147	A10103	430	A00147	A10284	353
A00147	A11104	165	A00147	A11404	190
A00147	A11411	188	A00147	A11468	288
A00147	A11513	210	A00147	A11621	490
A00147	A12049	217	A00147	A12246	227
A00147	A13157	288	A00147	A13362	353

A00147	A13511	491	A00147	A13886	185
A00147	A14302	341	A00147	A14444	179
A00147	A14800	404	A00147	A15018	227
A00147	A15910	278	A00147	A15929	317
A00147	A16877	152	A00147	A16969	341
A00148	A00163	297	A00148	A00165	193
A00148	A00171	469	A00148	A00209	901
A00148	A00224	899	A00148	A00232	218
A00148	A00305	263	A00148	A00476	347
A00148	A01483	480	A00148	A01527	899
A00148	A01733	179	A00148	A01748	167
A00148	A02308	176	A00148	A02488	193
A00148	A03338	221	A00148	A03861	188
A00148	A04429	288	A00148	A04495	196
A00148	A04769	299	A00148	A04977	217
A00148	A06203	340	A00148	A06379	311
A00148	A07277	361	A00148	A09431	307
A00148	A10580	196	A00148	A10899	218
A00148	A13978	329	A00148	A14299	483
A00148	A14444	927	A00148	A15726	314
A00149	A00151	238	A00149	A00152	489
A00149	A00153	899	A00149	A00154	916
A00149	A00159	945	A00149	A00163	989
A00149	A00167	424	A00149	A00171	195
A00149	A00175	334	A00149	A00188	204
A00149	A00190	490	A00149	A00209	899
A00149	A00219	899	A00149	A00228	210
A00149	A00232	491	A00149	A00233	243
A00149	A00248	945	A00149	A00260	423
A00149	A00309	440	A00149	A00324	899
A00149	A00336	198	A00149	A00400	619
A00149	A00410	165	A00149	A00472	258
A00149	A00496	199	A00149	A00628	199
A00149	A00643	419	A00149	A00704	171
A00149	A00781	899	A00149	A00820	196
A00149	A00852	446	A00149	A00883	899
A00149	A00887	899	A00149	A00914	937
A00149	A00967	334	A00149	A00968	218
A00149	A01052	207	A00149	A01151	334
A00149	A01361	174	A00149	A01524	989
A00149	A01527	989	A00149	A01537	404
A00149	A01558	199	A00149	A01562	899
A00149	A01572	486	A00149	A01588	899
A00149	A01628	899	A00149	A01638	899
A00149	A01668	411	A00149	A01669	945
A00149	A01676	491	A00149	A01693	313

A00149	A01708	899	A00149	A01729	297
A00149	A01741	940	A00149	A01743	157
A00149	A01775	210	A00149	A01781	899
A00149	A01797	152	A00149	A01844	899
A00149	A01882	205	A00149	A01908	899
A00149	A01926	334	A00149	A01992	160
A00149	A02086	356	A00149	A02159	213
A00149	A02411	899	A00149	A02481	334
A00149	A02491	384	A00149	A02606	899
A00149	A02625	301	A00149	A02717	345
A00149	A02718	199	A00149	A02753	467
A00149	A02797	899	A00149	A02837	193
A00149	A02903	160	A00149	A02908	901
A00149	A02968	899	A00149	A03095	489
A00149	A03097	899	A00149	A03302	191
A00149	A03401	207	A00149	A03406	183
A00149	A03434	356	A00149	A03539	249
A00149	A03873	334	A00149	A03893	313
A00149	A04187	899	A00149	A04395	176
A00149	A04410	171	A00149	A04449	380
A00149	A04477	899	A00149	A04488	180
A00149	A04512	235	A00149	A04622	274
A00149	A04627	207	A00149	A04743	168
A00149	A04847	223	A00149	A04908	313
A00149	A04996	317	A00149	A05307	198
A00149	A05367	305	A00149	A05583	191
A00149	A06039	199	A00149	A06289	899
A00149	A06476	235	A00149	A06581	196
A00149	A06609	899	A00149	A07091	899
A00149	A07096	288	A00149	A07153	344
A00149	A07165	260	A00149	A07185	176
A00149	A07187	179	A00149	A07201	462
A00149	A07209	279	A00149	A07222	160
A00149	A07307	899	A00149	A07311	899
A00149	A07339	903	A00149	A07495	199
A00149	A07799	154	A00149	A07961	286
A00149	A07974	213	A00149	A08133	199
A00149	A08567	899	A00149	A08571	899
A00149	A08572	185	A00149	A08751	160
A00149	A08850	334	A00149	A08878	899
A00149	A09490	213	A00149	A09577	899
A00149	A09603	198	A00149	A09654	899
A00149	A10174	899	A00149	A10234	235
A00149	A10240	198	A00149	A10272	301
A00149	A10291	383	A00149	A10508	154
A00149	A10773	152	A00149	A11242	223

A00149	A11339	198	A00149	A11343	185
A00149	A11559	334	A00149	A11687	154
A00149	A11749	270	A00149	A11905	191
A00149	A11907	301	A00149	A11964	176
A00149	A11983	937	A00149	A12373	334
A00149	A12390	160	A00149	A12662	367
A00149	A13225	899	A00149	A13737	899
A00149	A13750	899	A00149	A13963	899
A00149	A13975	899	A00149	A14019	899
A00149	A14023	899	A00149	A14098	191
A00149	A14148	174	A00149	A14193	469
A00149	A14194	899	A00149	A14210	334
A00149	A14247	899	A00149	A14371	334
A00149	A14444	989	A00149	A14636	555
A00149	A14643	180	A00149	A15856	899
A00149	A15949	899	A00149	A16068	899
A00149	A16297	899	A00149	A16644	154
A00149	A17047	899	A00150	A00152	633
A00150	A00159	199	A00150	A00162	466
A00150	A00167	903	A00150	A00177	741
A00150	A00178	906	A00150	A00191	950
A00150	A00192	486	A00150	A00203	945
A00150	A00204	199	A00150	A00209	914
A00150	A00212	720	A00150	A00219	375
A00150	A00221	238	A00150	A00222	479
A00150	A00223	967	A00150	A00231	899
A00150	A00240	395	A00150	A00243	200
A00150	A00247	536	A00150	A00252	899
A00150	A00253	899	A00150	A00257	610
A00150	A00258	986	A00150	A00268	396
A00150	A00270	154	A00150	A00279	491
A00150	A00280	491	A00150	A00281	491
A00150	A00283	648	A00150	A00284	488
A00150	A00285	491	A00150	A00286	193
A00150	A00288	641	A00150	A00289	491
A00150	A00292	188	A00150	A00296	452
A00150	A00299	489	A00150	A00304	491
A00150	A00323	467	A00150	A00325	429
A00150	A00327	443	A00150	A00328	491
A00150	A00329	404	A00150	A00334	491
A00150	A00335	956	A00150	A00337	163
A00150	A00340	586	A00150	A00348	446
A00150	A00355	180	A00150	A00356	865
A00150	A00357	490	A00150	A00358	488
A00150	A00366	490	A00150	A00368	401
A00150	A00371	340	A00150	A00374	170

A00150	A00404	481	A00150	A00406	302
A00150	A00409	384	A00150	A00411	491
A00150	A00414	491	A00150	A00423	491
A00150	A00427	420	A00150	A00433	306
A00150	A00439	152	A00150	A00444	351
A00150	A00454	491	A00150	A00458	368
A00150	A00467	491	A00150	A00469	491
A00150	A00489	347	A00150	A00494	465
A00150	A00500	417	A00150	A00501	193
A00150	A00505	329	A00150	A00507	418
A00150	A00520	207	A00150	A00521	491
A00150	A00531	620	A00150	A00554	170
A00150	A00560	404	A00150	A00561	491
A00150	A00571	582	A00150	A00589	157
A00150	A00590	651	A00150	A00593	490
A00150	A00595	170	A00150	A00603	491
A00150	A00605	491	A00150	A00629	581
A00150	A00643	167	A00150	A00648	595
A00150	A00651	984	A00150	A00653	528
A00150	A00694	548	A00150	A00698	427
A00150	A00700	945	A00150	A00701	491
A00150	A00705	491	A00150	A00711	486
A00150	A00713	550	A00150	A00727	177
A00150	A00749	170	A00150	A00763	491
A00150	A00767	170	A00150	A00782	491
A00150	A00783	460	A00150	A00787	553
A00150	A00788	313	A00150	A00805	391
A00150	A00806	489	A00150	A00809	236
A00150	A00811	414	A00150	A00812	341
A00150	A00826	163	A00150	A00831	461
A00150	A00832	899	A00150	A00833	428
A00150	A00839	802	A00150	A00841	489
A00150	A00843	414	A00150	A00848	485
A00150	A00861	499	A00150	A00870	479
A00150	A00872	170	A00150	A00876	380
A00150	A00878	491	A00150	A00879	490
A00150	A00902	454	A00150	A00905	490
A00150	A00910	230	A00150	A00916	600
A00150	A00918	170	A00150	A00919	423
A00150	A00922	491	A00150	A00923	491
A00150	A00932	490	A00150	A00934	170
A00150	A00945	480	A00150	A00955	585
A00150	A00965	481	A00150	A00966	491
A00150	A00970	491	A00150	A00972	489
A00150	A00973	187	A00150	A00980	546
A00150	A00983	210	A00150	A00986	281

A00150	A00990	383	A00150	A00991	279
A00150	A00992	389	A00150	A01000	490
A00150	A01012	586	A00150	A01017	491
A00150	A01025	491	A00150	A01028	210
A00150	A01029	486	A00150	A01032	236
A00150	A01034	384	A00150	A01037	526
A00150	A01038	578	A00150	A01042	491
A00150	A01047	424	A00150	A01054	469
A00150	A01056	956	A00150	A01069	307
A00150	A01072	491	A00150	A01082	377
A00150	A01083	433	A00150	A01085	491
A00150	A01090	491	A00150	A01091	899
A00150	A01092	408	A00150	A01098	627
A00150	A01101	489	A00150	A01112	695
A00150	A01115	491	A00150	A01120	484
A00150	A01121	475	A00150	A01127	587
A00150	A01129	170	A00150	A01130	208
A00150	A01139	218	A00150	A01140	491
A00150	A01144	184	A00150	A01150	491
A00150	A01154	486	A00150	A01160	176
A00150	A01166	491	A00150	A01168	943
A00150	A01184	480	A00150	A01186	430
A00150	A01189	490	A00150	A01192	400
A00150	A01193	154	A00150	A01194	438
A00150	A01196	491	A00150	A01201	363
A00150	A01206	491	A00150	A01212	491
A00150	A01216	563	A00150	A01217	414
A00150	A01219	480	A00150	A01221	246
A00150	A01234	491	A00150	A01238	240
A00150	A01243	470	A00150	A01247	491
A00150	A01249	491	A00150	A01261	390
A00150	A01263	625	A00150	A01270	491
A00150	A01275	435	A00150	A01282	390
A00150	A01288	472	A00150	A01291	991
A00150	A01294	294	A00150	A01306	661
A00150	A01309	163	A00150	A01335	491
A00150	A01337	485	A00150	A01351	170
A00150	A01356	170	A00150	A01362	306
A00150	A01365	491	A00150	A01369	490
A00150	A01384	488	A00150	A01392	318
A00150	A01393	215	A00150	A01406	475
A00150	A01407	358	A00150	A01411	549
A00150	A01425	962	A00150	A01431	491
A00150	A01433	485	A00150	A01435	443
A00150	A01436	296	A00150	A01437	491
A00150	A01447	457	A00150	A01449	458

A00150	A01457	543	A00150	A01458	534
A00150	A01461	624	A00150	A01464	481
A00150	A01468	563	A00150	A01470	491
A00150	A01473	199	A00150	A01487	350
A00150	A01488	485	A00150	A01492	975
A00150	A01495	369	A00150	A01496	945
A00150	A01503	527	A00150	A01512	899
A00150	A01522	307	A00150	A01527	903
A00150	A01530	330	A00150	A01531	413
A00150	A01538	535	A00150	A01539	535
A00150	A01540	401	A00150	A01558	899
A00150	A01560	491	A00150	A01561	621
A00150	A01569	491	A00150	A01572	333
A00150	A01579	651	A00150	A01586	563
A00150	A01588	899	A00150	A01597	467
A00150	A01598	581	A00150	A01601	991
A00150	A01605	949	A00150	A01606	455
A00150	A01612	537	A00150	A01614	540
A00150	A01618	277	A00150	A01638	899
A00150	A01649	587	A00150	A01652	212
A00150	A01684	475	A00150	A01707	491
A00150	A01718	489	A00150	A01740	396
A00150	A01764	210	A00150	A01770	899
A00150	A01787	491	A00150	A01822	534
A00150	A01917	378	A00150	A01995	615
A00150	A02148	260	A00150	A02179	491
A00150	A02216	452	A00150	A02227	363
A00150	A02265	529	A00150	A02270	401
A00150	A02350	491	A00150	A02397	293
A00150	A02437	63	A00150	A02494	579
A00150	A02592	77	A00150	A02675	152
A00150	A02877	486	A00150	A02928	281
A00150	A02950	398	A00150	A02978	404
A00150	A03048	185	A00150	A03049	210
A00150	A03069	491	A00150	A03070	483
A00150	A03083	470	A00150	A03164	486
A00150	A03188	260	A00150	A03298	470
A00150	A03304	490	A00150	A03332	324
A00150	A03438	540	A00150	A03445	491
A00150	A03446	488	A00150	A03461	486
A00150	A03493	404	A00150	A03567	582
A00150	A03569	486	A00150	A03571	461
A00150	A03625	899	A00150	A03639	259
A00150	A03645	199	A00150	A03652	170
A00150	A03686	899	A00150	A03726	935
A00150	A03738	457	A00150	A03740	491

A00150	A03752	533	A00150	A03754	308
A00150	A03809	491	A00150	A03858	183
A00150	A03859	480	A00150	A03860	183
A00150	A03939	218	A00150	A03963	940
A00150	A03981	621	A00150	A04099	454
A00150	A04202	467	A00150	A04230	491
A00150	A04846	455	A00150	A04856	231
A00150	A04858	255	A00150	A04988	455
A00150	A05029	283	A00150	A05076	286
A00150	A05086	213	A00150	A05090	179
A00150	A05149	382	A00150	A05230	180
A00150	A05298	259	A00150	A05411	218
A00150	A05418	407	A00150	A05443	491
A00150	A05449	491	A00150	A05450	373
A00150	A05499	391	A00150	A05501	491
A00150	A05557	489	A00150	A05654	486
A00150	A06037	480	A00150	A06121	382
A00150	A06275	193	A00150	A06277	327
A00150	A06279	490	A00150	A06286	330
A00150	A06351	489	A00150	A06356	170
A00150	A06466	527	A00150	A06534	490
A00150	A06564	490	A00150	A06696	160
A00150	A06779	210	A00150	A06785	215
A00150	A06812	199	A00150	A06814	608
A00150	A06861	536	A00150	A06897	491
A00150	A06965	491	A00150	A06973	491
A00150	A07047	491	A00150	A07201	569
A00150	A07243	393	A00150	A07244	170
A00150	A07247	490	A00150	A07307	413
A00150	A07311	848	A00150	A07323	491
A00150	A07349	475	A00150	A07398	563
A00150	A07422	899	A00150	A07451	491
A00150	A07506	436	A00150	A07524	714
A00150	A07533	160	A00150	A07536	481
A00150	A07581	491	A00150	A07726	958
A00150	A07798	373	A00150	A07803	195
A00150	A07924	468	A00150	A07962	207
A00150	A07965	176	A00150	A08011	587
A00150	A08052	274	A00150	A08061	463
A00150	A08131	491	A00150	A08137	530
A00150	A08154	480	A00150	A08211	170
A00150	A08273	212	A00150	A08277	452
A00150	A08301	490	A00150	A08315	272
A00150	A08321	382	A00150	A08327	477
A00150	A08357	491	A00150	A08358	491
A00150	A08359	491	A00150	A08369	563

A00150	A08377	486	A00150	A08398	454
A00150	A08408	491	A00150	A08419	457
A00150	A08452	484	A00150	A08457	491
A00150	A08462	452	A00150	A08466	452
A00150	A08476	491	A00150	A08489	395
A00150	A08501	490	A00150	A08521	302
A00150	A08533	441	A00150	A08543	491
A00150	A08546	385	A00150	A08550	314
A00150	A08556	491	A00150	A08559	491
A00150	A08567	163	A00150	A08571	955
A00150	A08682	475	A00150	A08707	210
A00150	A08748	241	A00150	A08760	247
A00150	A08761	258	A00150	A08773	581
A00150	A08835	479	A00150	A08859	210
A00150	A08869	418	A00150	A08872	401
A00150	A08929	303	A00150	A08973	583
A00150	A08983	372	A00150	A08991	229
A00150	A09044	286	A00150	A09054	163
A00150	A09263	247	A00150	A09277	154
A00150	A09285	335	A00150	A09297	468
A00150	A09308	488	A00150	A09322	581
A00150	A09332	241	A00150	A09337	279
A00150	A09352	63	A00150	A09539	165
A00150	A09550	79	A00150	A09577	353
A00150	A09590	177	A00150	A09592	290
A00150	A09607	210	A00150	A09610	297
A00150	A09613	157	A00150	A09620	467
A00150	A09622	475	A00150	A09655	268
A00150	A09657	170	A00150	A09674	283
A00150	A09697	170	A00150	A09722	259
A00150	A09726	449	A00150	A09741	193
A00150	A09808	177	A00150	A09852	490
A00150	A09862	452	A00150	A09865	243
A00150	A09893	63	A00150	A09902	291
A00150	A09960	157	A00150	A09999	291
A00150	A10001	581	A00150	A10034	160
A00150	A10336	452	A00150	A10362	450
A00150	A10367	306	A00150	A10417	63
A00150	A10425	247	A00150	A10430	210
A00150	A10490	210	A00150	A10518	388
A00150	A10532	826	A00150	A10564	63
A00150	A10575	899	A00150	A10616	328
A00150	A10633	491	A00150	A10645	490
A00150	A10711	406	A00150	A10728	185
A00150	A10748	483	A00150	A10797	217
A00150	A10798	180	A00150	A11096	424

A00150	A11139	347	A00150	A11190	491
A00150	A11254	224	A00150	A11263	340
A00150	A11286	310	A00150	A11295	201
A00150	A11297	490	A00150	A11302	204
A00150	A11331	302	A00150	A11334	491
A00150	A11344	488	A00150	A11346	254
A00150	A11372	414	A00150	A11390	195
A00150	A11392	447	A00150	A11400	361
A00150	A11420	491	A00150	A11438	466
A00150	A11440	587	A00150	A11462	313
A00150	A11480	451	A00150	A11487	189
A00150	A11522	374	A00150	A11535	480
A00150	A11544	446	A00150	A11550	491
A00150	A11562	486	A00150	A11572	440
A00150	A11593	193	A00150	A11597	378
A00150	A11616	488	A00150	A11688	462
A00150	A11705	151	A00150	A11728	491
A00150	A11808	369	A00150	A11846	486
A00150	A11853	944	A00150	A11857	490
A00150	A11861	485	A00150	A11922	224
A00150	A11924	428	A00150	A11936	468
A00150	A11937	445	A00150	A11945	462
A00150	A11981	389	A00150	A11983	903
A00150	A12011	335	A00150	A12042	160
A00150	A12068	423	A00150	A12079	297
A00150	A12160	224	A00150	A12168	362
A00150	A12193	281	A00150	A12259	405
A00150	A12269	486	A00150	A12270	491
A00150	A12305	207	A00150	A12328	483
A00150	A12329	490	A00150	A12404	286
A00150	A12618	180	A00150	A12813	210
A00150	A12821	491	A00150	A12970	362
A00150	A13002	299	A00150	A13012	210
A00150	A13052	170	A00150	A13063	962
A00150	A13107	252	A00150	A13126	480
A00150	A13207	174	A00150	A13311	486
A00150	A13335	388	A00150	A13340	491
A00150	A13357	396	A00150	A13375	221
A00150	A13403	177	A00150	A13425	424
A00150	A13525	168	A00150	A13527	177
A00150	A13581	269	A00150	A13609	208
A00150	A13626	152	A00150	A13702	207
A00150	A13708	163	A00150	A13710	491
A00150	A13752	351	A00150	A13824	899
A00150	A13829	899	A00150	A13830	491
A00150	A13874	358	A00150	A13875	435

A00150	A13889	189	A00150	A13944	163
A00150	A13989	989	A00150	A13992	587
A00150	A13994	358	A00150	A14025	340
A00150	A14038	940	A00150	A14055	925
A00150	A14073	488	A00150	A14090	468
A00150	A14148	899	A00150	A14150	406
A00150	A14152	461	A00150	A14169	488
A00150	A14172	558	A00150	A14176	491
A00150	A14188	489	A00150	A14192	151
A00150	A14206	401	A00150	A14216	915
A00150	A14246	899	A00150	A14249	185
A00150	A14257	389	A00150	A14386	224
A00150	A14410	379	A00150	A14443	989
A00150	A14447	157	A00150	A14476	154
A00150	A14496	216	A00150	A14505	283
A00150	A14508	491	A00150	A14530	457
A00150	A14566	477	A00150	A14568	291
A00150	A14591	267	A00150	A14738	480
A00150	A14758	199	A00150	A14908	286
A00150	A14923	412	A00150	A14937	586
A00150	A14948	199	A00150	A15034	277
A00150	A15183	162	A00150	A15202	491
A00150	A15225	491	A00150	A15229	378
A00150	A15235	481	A00150	A15236	383
A00150	A15244	581	A00150	A15268	945
A00150	A15297	905	A00150	A15324	899
A00150	A15353	899	A00150	A15359	899
A00150	A15376	291	A00150	A15381	491
A00150	A15499	368	A00150	A15500	582
A00150	A15507	513	A00150	A15547	924
A00150	A15559	899	A00150	A15614	903
A00150	A15658	174	A00150	A15660	539
A00150	A15684	738	A00150	A15708	319
A00150	A15711	483	A00150	A15732	491
A00150	A15743	379	A00150	A15751	207
A00150	A15769	409	A00150	A15771	286
A00150	A15778	419	A00150	A15931	491
A00150	A15932	229	A00150	A15933	235
A00150	A15938	256	A00150	A15971	297
A00150	A15988	488	A00150	A16053	177
A00150	A16060	465	A00150	A16088	489
A00150	A16092	173	A00150	A16107	943
A00150	A16118	489	A00150	A16218	462
A00150	A16266	232	A00150	A16272	477
A00150	A16318	180	A00150	A16410	199
A00150	A16418	332	A00150	A16437	435

A00150	A16462	249	A00150	A16575	228
A00150	A16627	339	A00150	A16658	484
A00150	A16682	474	A00150	A16686	491
A00150	A16724	246	A00150	A16768	393
A00150	A16778	313	A00150	A16822	285
A00150	A16837	246	A00150	A16839	468
A00150	A16847	210	A00150	A16904	163
A00150	A16937	179	A00150	A17134	446
A00150	A17140	477	A00150	A17171	199
A00150	A17189	249	A00151	A00152	920
A00151	A00157	324	A00151	A00159	911
A00151	A00160	205	A00151	A00190	921
A00151	A00208	63	A00151	A00224	923
A00151	A00226	902	A00151	A00230	899
A00151	A00233	912	A00151	A00236	151
A00151	A00246	899	A00151	A00315	180
A00151	A00324	188	A00151	A00336	316
A00151	A00581	490	A00151	A00609	185
A00151	A00747	395	A00151	A00914	157
A00151	A01092	291	A00151	A01189	226
A00151	A01553	71	A00151	A01554	937
A00151	A01558	899	A00151	A01588	899
A00151	A01636	899	A00151	A01651	899
A00151	A01661	204	A00151	A01709	252
A00151	A01730	196	A00151	A01779	185
A00151	A01856	154	A00151	A02116	174
A00151	A02170	182	A00151	A02325	152
A00151	A02353	180	A00151	A02487	146
A00151	A02564	491	A00151	A02641	446
A00151	A02695	236	A00151	A02703	80
A00151	A02737	942	A00151	A02775	329
A00151	A02795	212	A00151	A02816	420
A00151	A02825	942	A00151	A02826	141
A00151	A02847	219	A00151	A02860	171
A00151	A02866	279	A00151	A02878	245
A00151	A02881	249	A00151	A02904	491
A00151	A02908	190	A00151	A02918	240
A00151	A03123	285	A00151	A03185	260
A00151	A03305	183	A00151	A03321	121
A00151	A03338	488	A00151	A03436	151
A00151	A04226	329	A00151	A04288	184
A00151	A04346	456	A00151	A04517	213
A00151	A04541	385	A00151	A04714	191
A00151	A04956	439	A00151	A05222	279
A00151	A05769	364	A00151	A06404	243
A00151	A07146	218	A00151	A07422	899

A00151	A07426	291	A00151	A07693	490
A00151	A07900	174	A00151	A08182	252
A00151	A08403	221	A00151	A08572	395
A00151	A08660	171	A00151	A08667	216
A00151	A08911	168	A00151	A09009	182
A00151	A09643	899	A00151	A09937	198
A00151	A10034	176	A00151	A10363	183
A00151	A10717	344	A00151	A10744	255
A00151	A10811	163	A00151	A10825	319
A00151	A11452	177	A00151	A11564	296
A00151	A11639	344	A00151	A11808	241
A00151	A13191	899	A00151	A13978	274
A00151	A14008	899	A00151	A14013	241
A00151	A14092	180	A00151	A14196	899
A00151	A15230	152	A00151	A15463	179
A00151	A15507	899	A00151	A15877	172
A00151	A16145	302	A00152	A00153	490
A00152	A00154	485	A00152	A00158	206
A00152	A00159	974	A00152	A00160	107
A00152	A00161	299	A00152	A00162	731
A00152	A00163	454	A00152	A00165	185
A00152	A00167	958	A00152	A00174	263
A00152	A00182	234	A00152	A00190	997
A00152	A00204	666	A00152	A00207	725
A00152	A00209	922	A00152	A00212	491
A00152	A00214	480	A00152	A00219	491
A00152	A00220	232	A00152	A00224	970
A00152	A00231	477	A00152	A00232	491
A00152	A00233	582	A00152	A00234	528
A00152	A00238	945	A00152	A00245	252
A00152	A00246	919	A00152	A00248	229
A00152	A00254	491	A00152	A00256	272
A00152	A00257	472	A00152	A00261	698
A00152	A00264	491	A00152	A00274	457
A00152	A00294	363	A00152	A00309	271
A00152	A00316	423	A00152	A00324	474
A00152	A00328	259	A00152	A00338	439
A00152	A00352	382	A00152	A00356	528
A00152	A00377	534	A00152	A00421	198
A00152	A00438	412	A00152	A00446	949
A00152	A00454	393	A00152	A00457	489
A00152	A00458	177	A00152	A00485	486
A00152	A00489	187	A00152	A00496	361
A00152	A00521	462	A00152	A00531	603
A00152	A00556	489	A00152	A00558	473
A00152	A00560	157	A00152	A00561	625

A00152	A00565	258	A00152	A00572	529
A00152	A00576	490	A00152	A00600	180
A00152	A00604	150	A00152	A00643	460
A00152	A00648	165	A00152	A00651	489
A00152	A00653	753	A00152	A00671	259
A00152	A00688	182	A00152	A00694	581
A00152	A00700	327	A00152	A00704	485
A00152	A00722	236	A00152	A00724	369
A00152	A00728	428	A00152	A00736	491
A00152	A00741	228	A00152	A00783	491
A00152	A00785	491	A00152	A00790	432
A00152	A00799	945	A00152	A00820	491
A00152	A00849	475	A00152	A00876	480
A00152	A00883	491	A00152	A00914	945
A00152	A00919	208	A00152	A00926	228
A00152	A00953	430	A00152	A00963	217
A00152	A00970	297	A00152	A00973	152
A00152	A00980	215	A00152	A00981	401
A00152	A00985	173	A00152	A00987	230
A00152	A01009	221	A00152	A01011	228
A00152	A01012	154	A00152	A01022	396
A00152	A01024	165	A00152	A01042	490
A00152	A01046	477	A00152	A01052	319
A00152	A01054	483	A00152	A01071	198
A00152	A01082	432	A00152	A01087	449
A00152	A01091	213	A00152	A01098	177
A00152	A01100	357	A00152	A01108	252
A00152	A01127	377	A00152	A01140	373
A00152	A01150	627	A00152	A01161	532
A00152	A01176	228	A00152	A01192	210
A00152	A01198	899	A00152	A01211	162
A00152	A01235	286	A00152	A01252	199
A00152	A01265	491	A00152	A01275	491
A00152	A01287	288	A00152	A01289	406
A00152	A01291	521	A00152	A01303	317
A00152	A01306	293	A00152	A01307	463
A00152	A01399	477	A00152	A01431	489
A00152	A01443	193	A00152	A01446	486
A00152	A01448	446	A00152	A01452	593
A00152	A01457	544	A00152	A01461	199
A00152	A01473	651	A00152	A01489	899
A00152	A01492	168	A00152	A01496	467
A00152	A01524	997	A00152	A01527	995
A00152	A01540	580	A00152	A01543	406
A00152	A01551	491	A00152	A01553	224
A00152	A01560	404	A00152	A01561	363

A00152	A01572	619	A00152	A01579	242
A00152	A01582	483	A00152	A01588	899
A00152	A01599	163	A00152	A01604	486
A00152	A01606	486	A00152	A01612	253
A00152	A01615	989	A00152	A01627	174
A00152	A01631	899	A00152	A01632	491
A00152	A01645	480	A00152	A01654	423
A00152	A01668	491	A00152	A01676	491
A00152	A01704	491	A00152	A01705	301
A00152	A01711	363	A00152	A01712	162
A00152	A01727	903	A00152	A01733	406
A00152	A01741	491	A00152	A01743	491
A00152	A01746	443	A00152	A01767	151
A00152	A01769	458	A00152	A01775	491
A00152	A01778	172	A00152	A01782	198
A00152	A01785	427	A00152	A01789	491
A00152	A01790	379	A00152	A01796	491
A00152	A01797	469	A00152	A01798	433
A00152	A01802	277	A00152	A01822	366
A00152	A01844	944	A00152	A01859	180
A00152	A01860	198	A00152	A01863	260
A00152	A01870	314	A00152	A01875	252
A00152	A01876	409	A00152	A01879	491
A00152	A01880	274	A00152	A01887	491
A00152	A01888	491	A00152	A01894	364
A00152	A01902	430	A00152	A01903	472
A00152	A01905	157	A00152	A01908	399
A00152	A01931	249	A00152	A01941	263
A00152	A02031	241	A00152	A02086	475
A00152	A02117	217	A00152	A02138	227
A00152	A02163	261	A00152	A02167	218
A00152	A02179	364	A00152	A02183	475
A00152	A02252	260	A00152	A02260	172
A00152	A02268	168	A00152	A02298	390
A00152	A02370	232	A00152	A02371	185
A00152	A02372	460	A00152	A02380	432
A00152	A02382	698	A00152	A02411	899
A00152	A02416	291	A00152	A02427	899
A00152	A02436	491	A00152	A02447	372
A00152	A02454	241	A00152	A02462	172
A00152	A02477	401	A00152	A02490	474
A00152	A02491	481	A00152	A02564	281
A00152	A02567	176	A00152	A02578	154
A00152	A02582	160	A00152	A02611	385
A00152	A02633	312	A00152	A02637	466
A00152	A02659	419	A00152	A02695	419

A00152	A02698	302	A00152	A02705	63
A00152	A02709	483	A00152	A02710	63
A00152	A02714	240	A00152	A02730	440
A00152	A02731	281	A00152	A02735	266
A00152	A02739	405	A00152	A02742	135
A00152	A02745	412	A00152	A02746	380
A00152	A02749	235	A00152	A02751	210
A00152	A02756	384	A00152	A02769	302
A00152	A02787	481	A00152	A02788	438
A00152	A02798	457	A00152	A02803	389
A00152	A02812	485	A00152	A02819	360
A00152	A02820	63	A00152	A02828	416
A00152	A02830	411	A00152	A02834	167
A00152	A02835	418	A00152	A02837	491
A00152	A02847	480	A00152	A02851	458
A00152	A02859	491	A00152	A02863	210
A00152	A02864	488	A00152	A02869	63
A00152	A02872	481	A00152	A02874	490
A00152	A02896	491	A00152	A02898	308
A00152	A02904	184	A00152	A02908	942
A00152	A02912	479	A00152	A02916	475
A00152	A02918	452	A00152	A02925	457
A00152	A02929	260	A00152	A02944	489
A00152	A02945	154	A00152	A02951	420
A00152	A02953	283	A00152	A02961	466
A00152	A02968	899	A00152	A02972	366
A00152	A02983	180	A00152	A02988	489
A00152	A03010	308	A00152	A03018	413
A00152	A03044	154	A00152	A03046	246
A00152	A03051	174	A00152	A03070	152
A00152	A03104	435	A00152	A03110	326
A00152	A03112	457	A00152	A03131	190
A00152	A03157	379	A00152	A03162	180
A00152	A03183	384	A00152	A03188	449
A00152	A03204	481	A00152	A03211	491
A00152	A03223	473	A00152	A03224	183
A00152	A03259	582	A00152	A03271	475
A00152	A03275	491	A00152	A03280	313
A00152	A03282	338	A00152	A03293	307
A00152	A03304	489	A00152	A03332	489
A00152	A03336	174	A00152	A03431	326
A00152	A03436	279	A00152	A03451	276
A00152	A03557	247	A00152	A03585	324
A00152	A03611	491	A00152	A03657	329
A00152	A03676	379	A00152	A03739	221
A00152	A03740	489	A00152	A03752	199

A00152	A03776	899	A00152	A03788	263
A00152	A03809	490	A00152	A03821	490
A00152	A03843	172	A00152	A03851	470
A00152	A03858	211	A00152	A03887	202
A00152	A03910	334	A00152	A03940	213
A00152	A03942	195	A00152	A03959	486
A00152	A03981	276	A00152	A04010	480
A00152	A04015	196	A00152	A04033	491
A00152	A04051	462	A00152	A04062	491
A00152	A04110	162	A00152	A04117	441
A00152	A04164	363	A00152	A04169	350
A00152	A04206	311	A00152	A04248	419
A00152	A04275	456	A00152	A04304	288
A00152	A04418	294	A00152	A04449	366
A00152	A04504	201	A00152	A04536	191
A00152	A04548	356	A00152	A04589	380
A00152	A04622	491	A00152	A04650	159
A00152	A04652	379	A00152	A04668	154
A00152	A04718	406	A00152	A04798	190
A00152	A04848	451	A00152	A04879	236
A00152	A04880	224	A00152	A04902	447
A00152	A04908	477	A00152	A04931	343
A00152	A04939	330	A00152	A04949	542
A00152	A04964	414	A00152	A04985	467
A00152	A05044	899	A00152	A05220	173
A00152	A05221	268	A00152	A05234	219
A00152	A05278	477	A00152	A05288	281
A00152	A05309	486	A00152	A05349	446
A00152	A05398	208	A00152	A05418	284
A00152	A05449	486	A00152	A05577	557
A00152	A05679	157	A00152	A05724	400
A00152	A05982	207	A00152	A06039	199
A00152	A06082	374	A00152	A06286	236
A00152	A06324	260	A00152	A06339	180
A00152	A06415	463	A00152	A06422	414
A00152	A06466	491	A00152	A06569	162
A00152	A06610	491	A00152	A06615	266
A00152	A06623	477	A00152	A06631	151
A00152	A06642	472	A00152	A06686	240
A00152	A06758	193	A00152	A06997	243
A00152	A07061	195	A00152	A07062	346
A00152	A07126	306	A00152	A07140	174
A00152	A07149	174	A00152	A07153	167
A00152	A07157	752	A00152	A07160	466
A00152	A07161	481	A00152	A07164	417
A00152	A07165	491	A00152	A07166	375

A00152	A07200	223	A00152	A07201	430
A00152	A07208	605	A00152	A07209	484
A00152	A07229	368	A00152	A07258	353
A00152	A07311	482	A00152	A07314	433
A00152	A07339	571	A00152	A07422	468
A00152	A07442	417	A00152	A07451	329
A00152	A07747	283	A00152	A07803	452
A00152	A07961	488	A00152	A08133	195
A00152	A08137	199	A00152	A08280	549
A00152	A08438	281	A00152	A08445	159
A00152	A08452	375	A00152	A08461	260
A00152	A08533	172	A00152	A08591	201
A00152	A08718	157	A00152	A08732	218
A00152	A08834	430	A00152	A08835	422
A00152	A08850	243	A00152	A08851	458
A00152	A08878	314	A00152	A08885	228
A00152	A08957	162	A00152	A08987	165
A00152	A09120	288	A00152	A09180	254
A00152	A09201	364	A00152	A09263	254
A00152	A09422	201	A00152	A09556	899
A00152	A09592	491	A00152	A09643	899
A00152	A09670	213	A00152	A09732	481
A00152	A09852	310	A00152	A09860	332
A00152	A09913	907	A00152	A10156	185
A00152	A10214	441	A00152	A10279	180
A00152	A10314	160	A00152	A10532	491
A00152	A10556	395	A00152	A10624	899
A00152	A10633	491	A00152	A10797	480
A00152	A10798	491	A00152	A10905	171
A00152	A10935	180	A00152	A11244	335
A00152	A11296	339	A00152	A11297	456
A00152	A11300	385	A00152	A11322	196
A00152	A11337	396	A00152	A11346	195
A00152	A11369	457	A00152	A11400	205
A00152	A11419	384	A00152	A11431	469
A00152	A11487	207	A00152	A11673	218
A00152	A11684	422	A00152	A11714	419
A00152	A11983	634	A00152	A12047	899
A00152	A12216	180	A00152	A12897	488
A00152	A13002	425	A00152	A13328	182
A00152	A13340	486	A00152	A13617	199
A00152	A13620	198	A00152	A13826	451
A00152	A13829	299	A00152	A13860	576
A00152	A13975	422	A00152	A13986	678
A00152	A13989	170	A00152	A14008	899
A00152	A14071	468	A00152	A14073	352

A00152	A14122	371	A00152	A14191	472
A00152	A14196	414	A00152	A14198	490
A00152	A14202	480	A00152	A14204	486
A00152	A14208	577	A00152	A14210	475
A00152	A14216	752	A00152	A14231	749
A00152	A14232	899	A00152	A14236	296
A00152	A14364	358	A00152	A14366	420
A00152	A14371	413	A00152	A14374	184
A00152	A14381	454	A00152	A14387	180
A00152	A14391	252	A00152	A14394	545
A00152	A14395	363	A00152	A14410	400
A00152	A14430	519	A00152	A14439	168
A00152	A14443	899	A00152	A14449	165
A00152	A14452	491	A00152	A14475	325
A00152	A14481	199	A00152	A14483	435
A00152	A14496	355	A00152	A14511	486
A00152	A14570	357	A00152	A14596	542
A00152	A14624	159	A00152	A14635	491
A00152	A14643	585	A00152	A14648	252
A00152	A14659	518	A00152	A14667	310
A00152	A14701	486	A00152	A14716	195
A00152	A14738	226	A00152	A14930	358
A00152	A15029	226	A00152	A15220	180
A00152	A15230	208	A00152	A15282	451
A00152	A15292	553	A00152	A15293	242
A00152	A15297	491	A00152	A15302	215
A00152	A15310	321	A00152	A15312	313
A00152	A15313	355	A00152	A15314	489
A00152	A15317	241	A00152	A15322	486
A00152	A15323	583	A00152	A15324	455
A00152	A15328	441	A00152	A15331	358
A00152	A15346	200	A00152	A15348	306
A00152	A15349	479	A00152	A15352	447
A00152	A15376	299	A00152	A15416	435
A00152	A15432	311	A00152	A15436	323
A00152	A15452	286	A00152	A15454	226
A00152	A15480	334	A00152	A15484	177
A00152	A15487	249	A00152	A15495	358
A00152	A15507	428	A00152	A15535	474
A00152	A15537	163	A00152	A15540	457
A00152	A15601	174	A00152	A15606	489
A00152	A15613	152	A00152	A15660	556
A00152	A15684	524	A00152	A15726	491
A00152	A15795	372	A00152	A15806	159
A00152	A15896	210	A00152	A15930	188
A00152	A15931	491	A00152	A15962	491

A00152	A15988	480	A00152	A16006	401
A00152	A16114	167	A00152	A16218	318
A00152	A16224	377	A00152	A16272	232
A00152	A16300	343	A00152	A16491	213
A00152	A16520	171	A00152	A16636	491
A00152	A16791	899	A00152	A16910	215
A00152	A16916	152	A00152	A16960	157
A00152	A17085	269	A00152	A17134	224
A00152	A17136	223	A00153	A00154	88
A00153	A00157	899	A00153	A00158	438
A00153	A00159	329	A00153	A00160	456
A00153	A00162	151	A00153	A00163	483
A00153	A00167	938	A00153	A00169	469
A00153	A00171	229	A00153	A00173	271
A00153	A00174	152	A00153	A00180	252
A00153	A00184	899	A00153	A00189	409
A00153	A00190	945	A00153	A00191	899
A00153	A00192	899	A00153	A00200	485
A00153	A00201	382	A00153	A00207	245
A00153	A00209	921	A00153	A00214	375
A00153	A00219	475	A00153	A00220	306
A00153	A00224	915	A00153	A00226	899
A00153	A00234	238	A00153	A00240	326
A00153	A00246	899	A00153	A00250	899
A00153	A00254	899	A00153	A00256	425
A00153	A00264	398	A00153	A00268	156
A00153	A00270	899	A00153	A00271	899
A00153	A00274	221	A00153	A00275	899
A00153	A00531	899	A00153	A00557	462
A00153	A00597	201	A00153	A00694	457
A00153	A00736	491	A00153	A00799	449
A00153	A00820	488	A00153	A00911	216
A00153	A00914	340	A00153	A01003	360
A00153	A01139	899	A00153	A01161	213
A00153	A01257	434	A00153	A01289	944
A00153	A01297	199	A00153	A01359	899
A00153	A01472	899	A00153	A01524	943
A00153	A01527	949	A00153	A01554	925
A00153	A01558	899	A00153	A01588	899
A00153	A01593	899	A00153	A01632	235
A00153	A01639	899	A00153	A01654	356
A00153	A01669	899	A00153	A01718	899
A00153	A01730	935	A00153	A01732	899
A00153	A01743	294	A00153	A01747	355
A00153	A01781	899	A00153	A01790	402
A00153	A01791	327	A00153	A01858	167

A00153	A01880	446	A00153	A01881	246
A00153	A01888	648	A00153	A01890	167
A00153	A02006	267	A00153	A02124	899
A00153	A02143	224	A00153	A02180	163
A00153	A02208	323	A00153	A02217	183
A00153	A02247	160	A00153	A02382	208
A00153	A02416	348	A00153	A02425	263
A00153	A02497	160	A00153	A02501	899
A00153	A02609	160	A00153	A02639	475
A00153	A02698	481	A00153	A02709	411
A00153	A02711	433	A00153	A02714	228
A00153	A02723	899	A00153	A02730	303
A00153	A02732	319	A00153	A02741	228
A00153	A02788	305	A00153	A02826	191
A00153	A02836	322	A00153	A02863	274
A00153	A02886	195	A00153	A02917	196
A00153	A02945	319	A00153	A02983	213
A00153	A03010	190	A00153	A03025	151
A00153	A03051	151	A00153	A03095	173
A00153	A03101	177	A00153	A03110	907
A00153	A03121	488	A00153	A03211	279
A00153	A03255	288	A00153	A03259	363
A00153	A03451	454	A00153	A03457	200
A00153	A03576	151	A00153	A03611	486
A00153	A03711	205	A00153	A03726	899
A00153	A03753	313	A00153	A03766	270
A00153	A03790	267	A00153	A03809	305
A00153	A03821	188	A00153	A03822	443
A00153	A03846	380	A00153	A03858	199
A00153	A03994	160	A00153	A04117	491
A00153	A04138	196	A00153	A04144	156
A00153	A04192	174	A00153	A04201	236
A00153	A04250	174	A00153	A04263	268
A00153	A04280	382	A00153	A04294	183
A00153	A04303	241	A00153	A04351	201
A00153	A04367	183	A00153	A04489	241
A00153	A04530	167	A00153	A04551	183
A00153	A04584	156	A00153	A04614	900
A00153	A04659	960	A00153	A04759	152
A00153	A04796	276	A00153	A04797	241
A00153	A04806	205	A00153	A04855	193
A00153	A04859	241	A00153	A04872	241
A00153	A04996	173	A00153	A05373	193
A00153	A05795	483	A00153	A05869	319
A00153	A06142	241	A00153	A06226	174
A00153	A06607	328	A00153	A06650	417

A00153	A06658	276	A00153	A06686	156
A00153	A06783	157	A00153	A06795	483
A00153	A07004	207	A00153	A07145	168
A00153	A07154	185	A00153	A07158	416
A00153	A07173	361	A00153	A07189	167
A00153	A07192	472	A00153	A07270	221
A00153	A07422	899	A00153	A07426	167
A00153	A07960	221	A00153	A08275	276
A00153	A08403	208	A00153	A08589	276
A00153	A08609	151	A00153	A08730	193
A00153	A09047	221	A00153	A09112	174
A00153	A09258	899	A00153	A09365	205
A00153	A09377	899	A00153	A09419	188
A00153	A09526	475	A00153	A09781	276
A00153	A09884	301	A00153	A09900	385
A00153	A09941	167	A00153	A09957	276
A00153	A09964	294	A00153	A10005	319
A00153	A10160	151	A00153	A10340	899
A00153	A10489	193	A00153	A10532	899
A00153	A10580	944	A00153	A10589	899
A00153	A10601	899	A00153	A10623	899
A00153	A10735	484	A00153	A10830	899
A00153	A10907	221	A00153	A11104	291
A00153	A11582	335	A00153	A11850	899
A00153	A11894	205	A00153	A11983	899
A00153	A12086	272	A00153	A12275	156
A00153	A12341	205	A00153	A12415	276
A00153	A12983	160	A00153	A13390	183
A00153	A13795	174	A00153	A13924	241
A00153	A13972	160	A00153	A14016	208
A00153	A14038	205	A00153	A14127	454
A00153	A14158	221	A00153	A14208	252
A00153	A14231	899	A00153	A14236	210
A00153	A14330	477	A00153	A14366	246
A00153	A14393	335	A00153	A14444	899
A00153	A14484	899	A00153	A14644	463
A00153	A14893	201	A00153	A15241	469
A00153	A15292	899	A00153	A15295	899
A00153	A15296	899	A00153	A15549	174
A00153	A15605	218	A00153	A15606	468
A00153	A15609	174	A00153	A15795	179
A00153	A15933	899	A00153	A15951	899
A00153	A16081	436	A00153	A16083	899
A00153	A16112	267	A00153	A16442	221
A00153	A16636	316	A00153	A16671	156
A00153	A16887	174	A00153	A16910	210

A00153	A17136	311	A00154	A00159	489
A00154	A00163	945	A00154	A00171	457
A00154	A00173	246	A00154	A00185	899
A00154	A00190	491	A00154	A00196	159
A00154	A00209	899	A00154	A00216	899
A00154	A00219	274	A00154	A00232	470
A00154	A00233	475	A00154	A00254	899
A00154	A00260	156	A00154	A00263	461
A00154	A00266	165	A00154	A00294	899
A00154	A00298	407	A00154	A00343	391
A00154	A00385	408	A00154	A00469	446
A00154	A00736	272	A00154	A00804	168
A00154	A00832	226	A00154	A00914	329
A00154	A01070	899	A00154	A01157	278
A00154	A01189	258	A00154	A01257	899
A00154	A01325	190	A00154	A01527	899
A00154	A01572	491	A00154	A01595	302
A00154	A01668	420	A00154	A01676	491
A00154	A01730	210	A00154	A01741	481
A00154	A01745	486	A00154	A01746	221
A00154	A01796	377	A00154	A01835	172
A00154	A01844	224	A00154	A01897	472
A00154	A01901	491	A00154	A01906	262
A00154	A02086	243	A00154	A02145	243
A00154	A02169	247	A00154	A02180	277
A00154	A02219	400	A00154	A02377	254
A00154	A02384	156	A00154	A02549	159
A00154	A02586	160	A00154	A02633	347
A00154	A02676	156	A00154	A02689	179
A00154	A02703	210	A00154	A02711	457
A00154	A02714	281	A00154	A02733	310
A00154	A02743	204	A00154	A02749	371
A00154	A02754	234	A00154	A02819	190
A00154	A02836	385	A00154	A02844	196
A00154	A02869	221	A00154	A02877	345
A00154	A03070	443	A00154	A03275	252
A00154	A03309	190	A00154	A03401	414
A00154	A03611	246	A00154	A03614	176
A00154	A03744	224	A00154	A03814	162
A00154	A03821	329	A00154	A03883	303
A00154	A03947	172	A00154	A04017	323
A00154	A04045	238	A00154	A04260	202
A00154	A04305	343	A00154	A04307	234
A00154	A04316	310	A00154	A04351	346
A00154	A04355	157	A00154	A04375	195
A00154	A04417	177	A00154	A04476	303

A00154	A04573	176	A00154	A04576	462
A00154	A04622	288	A00154	A04627	213
A00154	A04952	238	A00154	A04977	283
A00154	A04990	180	A00154	A05112	212
A00154	A05163	210	A00154	A05219	165
A00154	A05282	218	A00154	A05283	190
A00154	A05415	160	A00154	A05473	184
A00154	A05681	185	A00154	A05798	165
A00154	A05967	238	A00154	A06199	254
A00154	A06231	414	A00154	A06295	899
A00154	A06330	238	A00154	A06606	184
A00154	A06608	299	A00154	A06623	262
A00154	A06736	363	A00154	A07127	330
A00154	A07153	491	A00154	A07165	469
A00154	A07201	477	A00154	A07217	152
A00154	A07243	174	A00154	A07282	491
A00154	A07311	899	A00154	A07339	288
A00154	A07641	234	A00154	A08567	899
A00154	A08851	406	A00154	A09120	334
A00154	A09370	152	A00154	A09402	238
A00154	A09492	274	A00154	A09900	191
A00154	A09989	165	A00154	A10005	184
A00154	A10279	254	A00154	A10284	172
A00154	A10525	235	A00154	A10529	185
A00154	A10564	180	A00154	A10625	385
A00154	A11394	302	A00154	A11404	470
A00154	A11852	382	A00154	A12079	210
A00154	A12192	254	A00154	A14014	156
A00154	A14035	210	A00154	A14198	899
A00154	A14596	899	A00154	A14643	457
A00154	A15272	176	A00154	A15293	899
A00154	A15294	899	A00154	A15596	224
A00154	A15716	185	A00154	A16110	159
A00154	A16275	274	A00155	A00196	190
A00155	A00204	475	A00155	A00215	491
A00155	A00216	491	A00155	A00218	446
A00155	A00310	196	A00155	A00312	378
A00155	A00338	386	A00155	A00343	274
A00155	A00351	167	A00155	A00395	489
A00155	A00449	491	A00155	A00472	480
A00155	A00476	481	A00155	A00541	489
A00155	A00567	241	A00155	A00574	491
A00155	A00600	157	A00155	A00614	445
A00155	A00628	475	A00155	A00633	455
A00155	A00645	333	A00155	A00654	491
A00155	A00660	162	A00155	A00662	491

A00155	A00663	491	A00155	A00666	486
A00155	A00681	490	A00155	A00695	391
A00155	A00696	356	A00155	A00738	429
A00155	A00770	457	A00155	A00771	414
A00155	A00773	486	A00155	A00785	383
A00155	A00807	188	A00155	A00962	491
A00155	A00995	283	A00155	A01005	491
A00155	A01008	491	A00155	A01018	483
A00155	A01035	491	A00155	A01138	413
A00155	A01161	491	A00155	A01174	409
A00155	A01175	414	A00155	A01207	307
A00155	A01223	335	A00155	A01227	165
A00155	A01233	441	A00155	A01256	491
A00155	A01297	479	A00155	A01347	257
A00155	A01367	353	A00155	A01397	294
A00155	A01409	490	A00155	A01481	430
A00155	A01508	474	A00155	A01520	187
A00155	A01540	466	A00155	A01551	243
A00155	A01556	296	A00155	A01590	477
A00155	A01600	291	A00155	A01635	490
A00155	A01670	174	A00155	A01705	465
A00155	A01713	491	A00155	A01714	238
A00155	A01738	238	A00155	A01743	307
A00155	A01857	196	A00155	A01866	210
A00155	A01882	171	A00155	A01926	452
A00155	A01947	232	A00155	A01972	484
A00155	A02012	154	A00155	A02066	273
A00155	A02071	484	A00155	A02075	363
A00155	A02083	484	A00155	A02168	335
A00155	A02215	491	A00155	A02254	340
A00155	A02257	179	A00155	A02306	299
A00155	A02316	480	A00155	A02317	196
A00155	A02329	240	A00155	A02330	324
A00155	A02338	71	A00155	A02402	252
A00155	A02443	394	A00155	A02521	352
A00155	A02538	180	A00155	A02600	201
A00155	A02601	435	A00155	A02774	247
A00155	A02785	489	A00155	A02814	91
A00155	A02859	489	A00155	A02874	286
A00155	A02992	491	A00155	A03016	462
A00155	A03027	276	A00155	A03050	491
A00155	A03080	241	A00155	A03099	347
A00155	A03167	477	A00155	A03303	217
A00155	A03347	243	A00155	A03353	405
A00155	A03369	223	A00155	A03370	386
A00155	A03379	301	A00155	A03402	310

A00155	A03429	180	A00155	A03441	256
A00155	A03513	369	A00155	A03545	455
A00155	A03563	296	A00155	A03618	428
A00155	A03740	414	A00155	A03917	383
A00155	A04010	180	A00155	A04023	154
A00155	A04027	290	A00155	A04053	260
A00155	A04211	446	A00155	A04212	180
A00155	A04258	157	A00155	A04369	491
A00155	A04371	157	A00155	A04556	208
A00155	A04633	457	A00155	A04645	188
A00155	A04660	188	A00155	A04744	157
A00155	A04791	335	A00155	A04834	185
A00155	A04839	491	A00155	A04883	274
A00155	A04927	445	A00155	A04995	246
A00155	A05007	389	A00155	A05036	152
A00155	A05045	219	A00155	A05102	217
A00155	A05153	361	A00155	A05207	217
A00155	A05356	490	A00155	A05390	449
A00155	A05393	378	A00155	A05416	390
A00155	A05418	283	A00155	A05487	423
A00155	A05532	188	A00155	A05573	390
A00155	A05586	188	A00155	A05659	212
A00155	A05770	263	A00155	A05774	235
A00155	A05858	217	A00155	A05926	424
A00155	A05957	163	A00155	A06092	462
A00155	A06237	188	A00155	A06252	213
A00155	A06263	382	A00155	A06295	260
A00155	A06316	217	A00155	A06358	419
A00155	A06405	159	A00155	A06439	424
A00155	A06527	489	A00155	A06592	213
A00155	A06607	481	A00155	A06630	180
A00155	A06633	174	A00155	A06641	274
A00155	A06689	366	A00155	A06696	177
A00155	A06709	201	A00155	A06756	451
A00155	A06874	351	A00155	A06908	402
A00155	A06970	218	A00155	A06979	401
A00155	A07059	322	A00155	A07122	480
A00155	A07209	159	A00155	A07218	241
A00155	A07238	283	A00155	A07297	171
A00155	A07300	383	A00155	A07407	486
A00155	A07413	371	A00155	A07452	472
A00155	A07471	430	A00155	A07475	190
A00155	A07477	385	A00155	A07520	396
A00155	A07527	163	A00155	A07541	491
A00155	A07542	489	A00155	A07543	490
A00155	A07544	163	A00155	A07553	205

A00155	A07561	486	A00155	A07610	480
A00155	A07629	213	A00155	A07630	249
A00155	A07631	322	A00155	A07632	238
A00155	A07633	157	A00155	A07634	485
A00155	A07635	157	A00155	A07662	188
A00155	A07674	398	A00155	A07736	434
A00155	A07800	480	A00155	A07823	171
A00155	A07958	406	A00155	A07973	274
A00155	A08404	401	A00155	A08656	180
A00155	A08693	366	A00155	A08785	208
A00155	A08858	366	A00155	A09711	217
A00155	A09908	273	A00155	A10056	480
A00155	A10429	188	A00155	A10456	317
A00155	A10536	273	A00155	A10686	221
A00155	A10967	477	A00155	A11309	279
A00155	A11414	180	A00155	A11983	205
A00155	A12793	340	A00155	A13033	217
A00155	A13369	364	A00155	A13530	238
A00155	A13871	491	A00155	A13879	335
A00155	A14121	201	A00155	A14247	228
A00155	A14502	488	A00155	A14635	279
A00155	A14827	160	A00155	A15675	152
A00155	A15699	218	A00155	A15992	249
A00155	A16027	208	A00155	A16028	193
A00155	A16371	234	A00155	A16649	238
A00155	A16741	323	A00155	A17078	491
A00156	A00161	168	A00156	A00180	550
A00156	A00183	292	A00156	A00184	119
A00156	A00185	491	A00156	A00207	960
A00156	A00208	944	A00156	A00214	194
A00156	A00224	906	A00156	A00227	352
A00156	A00228	246	A00156	A00234	469
A00156	A00239	445	A00156	A00240	945
A00156	A00241	396	A00156	A00242	922
A00156	A00246	932	A00156	A00247	419
A00156	A00254	491	A00156	A00261	194
A00156	A00276	288	A00156	A00379	391
A00156	A00418	479	A00156	A00451	472
A00156	A00460	285	A00156	A00600	157
A00156	A00696	234	A00156	A00805	192
A00156	A00820	162	A00156	A00821	257
A00156	A00883	191	A00156	A00978	152
A00156	A01212	630	A00156	A01286	460
A00156	A01524	938	A00156	A01527	942
A00156	A01553	224	A00156	A01558	899
A00156	A01560	475	A00156	A01562	718

A00156	A01588	899	A00156	A01615	899
A00156	A01628	148	A00156	A01636	216
A00156	A01692	63	A00156	A01718	597
A00156	A01813	252	A00156	A01822	340
A00156	A01908	360	A00156	A01914	308
A00156	A02027	217	A00156	A02056	188
A00156	A02344	185	A00156	A02481	263
A00156	A02511	219	A00156	A02702	241
A00156	A02751	163	A00156	A02766	401
A00156	A02788	124	A00156	A02803	110
A00156	A02805	210	A00156	A02841	63
A00156	A02874	176	A00156	A02875	385
A00156	A02881	221	A00156	A02887	63
A00156	A02897	389	A00156	A02931	77
A00156	A03001	210	A00156	A03019	135
A00156	A03100	223	A00156	A03271	335
A00156	A03282	152	A00156	A03335	509
A00156	A03683	355	A00156	A03684	394
A00156	A03784	484	A00156	A03817	247
A00156	A03830	480	A00156	A03831	272
A00156	A03892	63	A00156	A04012	433
A00156	A04037	249	A00156	A04111	260
A00156	A04123	272	A00156	A04242	260
A00156	A04314	168	A00156	A04323	465
A00156	A04517	185	A00156	A04578	235
A00156	A04600	263	A00156	A04686	208
A00156	A04697	188	A00156	A04731	160
A00156	A04807	215	A00156	A04972	202
A00156	A05111	255	A00156	A05208	188
A00156	A05216	899	A00156	A05337	160
A00156	A05339	255	A00156	A05364	310
A00156	A05621	255	A00156	A06256	279
A00156	A06269	235	A00156	A06289	63
A00156	A06610	297	A00156	A06691	324
A00156	A06707	162	A00156	A07056	210
A00156	A07311	949	A00156	A07860	297
A00156	A08621	201	A00156	A09076	221
A00156	A09220	297	A00156	A09401	328
A00156	A09568	185	A00156	A09897	324
A00156	A10294	151	A00156	A10510	366
A00156	A10630	230	A00156	A10898	221
A00156	A11108	324	A00156	A11124	452
A00156	A11129	446	A00156	A11369	63
A00156	A12047	278	A00156	A12383	85
A00156	A12969	276	A00156	A12971	288
A00156	A13216	329	A00156	A13335	156

A00156	A13340	213	A00156	A13511	208
A00156	A13555	205	A00156	A13824	63
A00156	A13980	172	A00156	A13983	63
A00156	A14032	466	A00156	A14035	934
A00156	A14071	156	A00156	A14128	436
A00156	A14164	324	A00156	A14188	480
A00156	A14198	490	A00156	A14292	179
A00156	A14299	552	A00156	A14364	393
A00156	A14402	165	A00156	A14433	329
A00156	A14438	198	A00156	A14443	903
A00156	A14930	93	A00156	A14944	249
A00156	A15325	490	A00156	A15506	173
A00156	A15726	63	A00156	A16547	198
A00156	A16939	434	A00157	A00159	899
A00157	A00161	914	A00157	A00162	922
A00157	A00163	899	A00157	A00167	899
A00157	A00190	899	A00157	A00198	899
A00157	A00205	899	A00157	A00206	188
A00157	A00208	441	A00157	A00209	899
A00157	A00217	207	A00157	A00221	899
A00157	A00224	903	A00157	A00229	899
A00157	A00230	899	A00157	A00236	901
A00157	A00240	990	A00157	A00246	899
A00157	A00248	899	A00157	A00250	930
A00157	A00252	899	A00157	A00253	899
A00157	A00254	902	A00157	A00257	930
A00157	A00266	899	A00157	A00271	899
A00157	A00273	346	A00157	A00324	899
A00157	A00334	228	A00157	A00408	199
A00157	A00587	229	A00157	A00630	297
A00157	A00651	187	A00157	A00668	160
A00157	A00747	899	A00157	A00875	297
A00157	A00922	207	A00157	A00981	193
A00157	A01002	899	A00157	A01244	899
A00157	A01283	198	A00157	A01345	199
A00157	A01415	899	A00157	A01478	199
A00157	A01512	915	A00157	A01524	899
A00157	A01527	989	A00157	A01553	995
A00157	A01554	994	A00157	A01558	989
A00157	A01560	993	A00157	A01561	960
A00157	A01562	994	A00157	A01588	899
A00157	A01602	182	A00157	A01605	993
A00157	A01615	989	A00157	A01628	994
A00157	A01639	994	A00157	A01640	994
A00157	A01683	199	A00157	A01688	899
A00157	A01692	918	A00157	A01697	993

A00157	A01702	941	A00157	A01708	942
A00157	A01709	993	A00157	A01718	993
A00157	A01719	938	A00157	A01844	899
A00157	A02344	165	A00157	A02390	899
A00157	A02517	899	A00157	A02877	899
A00157	A03082	899	A00157	A03213	916
A00157	A03247	168	A00157	A03273	899
A00157	A03331	168	A00157	A03575	899
A00157	A03750	899	A00157	A04104	340
A00157	A04320	899	A00157	A04449	899
A00157	A04699	899	A00157	A04807	899
A00157	A05064	899	A00157	A05082	899
A00157	A05216	899	A00157	A06277	899
A00157	A06466	175	A00157	A06559	255
A00157	A06851	457	A00157	A07311	994
A00157	A07339	899	A00157	A07398	201
A00157	A07422	899	A00157	A07797	899
A00157	A08571	199	A00157	A08732	899
A00157	A08858	899	A00157	A08878	899
A00157	A08969	249	A00157	A09162	345
A00157	A09218	899	A00157	A09297	299
A00157	A09319	899	A00157	A09577	902
A00157	A09804	899	A00157	A09871	899
A00157	A10047	188	A00157	A10185	899
A00157	A10365	913	A00157	A10392	902
A00157	A10580	914	A00157	A10615	899
A00157	A10623	902	A00157	A10655	297
A00157	A11125	899	A00157	A11206	899
A00157	A11376	481	A00157	A11549	272
A00157	A11937	226	A00157	A11983	899
A00157	A12000	226	A00157	A12341	899
A00157	A12383	994	A00157	A12408	190
A00157	A12990	286	A00157	A13340	162
A00157	A13729	302	A00157	A13824	989
A00157	A13902	297	A00157	A13980	899
A00157	A13981	989	A00157	A14005	899
A00157	A14073	820	A00157	A14268	156
A00157	A14404	160	A00157	A14443	899
A00157	A14444	899	A00157	A14843	335
A00157	A15324	899	A00157	A15336	238
A00157	A15353	899	A00157	A15932	297
A00157	A15944	899	A00157	A16093	899
A00157	A16585	559	A00158	A00159	955
A00158	A00160	491	A00158	A00161	296
A00158	A00162	414	A00158	A00167	288
A00158	A00185	63	A00158	A00189	491

A00158	A00205	162	A00158	A00207	173
A00158	A00208	63	A00158	A00220	263
A00158	A00224	687	A00158	A00230	229
A00158	A00231	475	A00158	A00232	152
A00158	A00240	343	A00158	A00246	912
A00158	A00254	184	A00158	A00255	428
A00158	A00407	488	A00158	A00421	401
A00158	A00457	272	A00158	A00532	152
A00158	A00655	467	A00158	A00694	260
A00158	A00696	299	A00158	A00697	191
A00158	A00905	432	A00158	A00981	356
A00158	A01289	228	A00158	A01385	477
A00158	A01406	259	A00158	A01445	156
A00158	A01473	491	A00158	A01524	491
A00158	A01527	944	A00158	A01553	152
A00158	A01554	85	A00158	A01558	899
A00158	A01560	63	A00158	A01572	168
A00158	A01588	899	A00158	A01628	73
A00158	A01688	191	A00158	A01719	63
A00158	A01880	198	A00158	A01975	162
A00158	A02000	340	A00158	A02076	263
A00158	A02141	177	A00158	A02382	299
A00158	A02698	296	A00158	A02709	160
A00158	A02711	491	A00158	A02732	110
A00158	A02795	152	A00158	A02803	63
A00158	A03001	76	A00158	A03010	441
A00158	A03436	310	A00158	A03685	152
A00158	A03790	484	A00158	A03822	305
A00158	A04033	281	A00158	A04078	241
A00158	A04105	249	A00158	A04280	243
A00158	A04320	271	A00158	A04805	281
A00158	A05285	200	A00158	A05986	162
A00158	A06147	243	A00158	A06404	185
A00158	A07004	335	A00158	A07173	159
A00158	A08137	185	A00158	A08300	481
A00158	A08926	457	A00158	A09562	156
A00158	A10265	219	A00158	A10544	151
A00158	A10570	329	A00158	A10721	466
A00158	A10735	71	A00158	A10813	350
A00158	A10899	486	A00158	A11121	216
A00158	A11262	183	A00158	A11369	173
A00158	A11487	313	A00158	A11543	485
A00158	A12548	348	A00158	A12560	302
A00158	A12927	156	A00158	A12992	944
A00158	A13074	152	A00158	A13110	271
A00158	A13555	469	A00158	A13860	378

A00158	A13975	371	A00158	A13980	489
A00158	A13983	405	A00158	A13986	341
A00158	A14236	246	A00158	A14250	193
A00158	A14290	176	A00158	A14452	491
A00158	A15263	302	A00158	A15292	357
A00158	A15599	243	A00158	A15726	491
A00158	A16192	207	A00158	A16476	204
A00158	A16994	414	A00158	A16996	353
A00159	A00161	913	A00159	A00162	600
A00159	A00163	905	A00159	A00167	913
A00159	A00171	906	A00159	A00173	165
A00159	A00182	327	A00159	A00185	932
A00159	A00189	717	A00159	A00190	945
A00159	A00200	899	A00159	A00205	903
A00159	A00206	922	A00159	A00207	974
A00159	A00208	900	A00159	A00209	899
A00159	A00212	905	A00159	A00213	481
A00159	A00214	950	A00159	A00219	945
A00159	A00220	899	A00159	A00221	908
A00159	A00223	899	A00159	A00224	980
A00159	A00225	491	A00159	A00226	363
A00159	A00230	424	A00159	A00231	900
A00159	A00232	636	A00159	A00233	945
A00159	A00234	994	A00159	A00239	899
A00159	A00240	899	A00159	A00241	899
A00159	A00242	899	A00159	A00246	993
A00159	A00252	899	A00159	A00253	899
A00159	A00254	491	A00159	A00256	899
A00159	A00257	914	A00159	A00263	903
A00159	A00264	899	A00159	A00265	358
A00159	A00269	914	A00159	A00270	906
A00159	A00294	989	A00159	A00306	302
A00159	A00316	356	A00159	A00324	945
A00159	A00330	899	A00159	A00340	899
A00159	A00370	899	A00159	A00378	899
A00159	A00395	176	A00159	A00408	173
A00159	A00420	229	A00159	A00421	458
A00159	A00470	899	A00159	A00482	899
A00159	A00495	235	A00159	A00496	899
A00159	A00531	913	A00159	A00533	941
A00159	A00552	263	A00159	A00557	443
A00159	A00587	191	A00159	A00597	899
A00159	A00640	899	A00159	A00641	942
A00159	A00652	302	A00159	A00736	491
A00159	A00744	899	A00159	A00786	185
A00159	A00820	488	A00159	A00832	910

A00159	A00853	899	A00159	A00874	404
A00159	A00883	899	A00159	A00914	945
A00159	A00916	685	A00159	A00939	932
A00159	A00981	176	A00159	A01022	165
A00159	A01052	945	A00159	A01070	913
A00159	A01077	899	A00159	A01088	552
A00159	A01137	347	A00159	A01149	899
A00159	A01172	475	A00159	A01173	465
A00159	A01198	899	A00159	A01234	469
A00159	A01235	243	A00159	A01257	899
A00159	A01259	435	A00159	A01275	899
A00159	A01281	484	A00159	A01286	347
A00159	A01295	899	A00159	A01307	491
A00159	A01308	899	A00159	A01361	176
A00159	A01396	480	A00159	A01415	899
A00159	A01419	306	A00159	A01438	243
A00159	A01450	251	A00159	A01451	905
A00159	A01452	576	A00159	A01458	472
A00159	A01470	375	A00159	A01473	582
A00159	A01486	899	A00159	A01489	899
A00159	A01491	899	A00159	A01492	542
A00159	A01494	899	A00159	A01503	905
A00159	A01512	899	A00159	A01521	899
A00159	A01524	945	A00159	A01527	994
A00159	A01528	899	A00159	A01539	157
A00159	A01550	899	A00159	A01553	943
A00159	A01554	930	A00159	A01557	276
A00159	A01558	995	A00159	A01559	470
A00159	A01560	957	A00159	A01561	927
A00159	A01562	899	A00159	A01570	320
A00159	A01572	966	A00159	A01575	235
A00159	A01576	502	A00159	A01585	904
A00159	A01588	989	A00159	A01591	899
A00159	A01592	899	A00159	A01593	899
A00159	A01601	899	A00159	A01602	934
A00159	A01603	475	A00159	A01605	899
A00159	A01612	955	A00159	A01615	908
A00159	A01626	918	A00159	A01628	901
A00159	A01634	899	A00159	A01636	900
A00159	A01637	910	A00159	A01638	899
A00159	A01639	899	A00159	A01640	899
A00159	A01646	899	A00159	A01650	297
A00159	A01651	905	A00159	A01668	416
A00159	A01676	945	A00159	A01686	899
A00159	A01688	906	A00159	A01692	931
A00159	A01702	899	A00159	A01708	899

A00159	A01709	909	A00159	A01718	899
A00159	A01719	899	A00159	A01730	418
A00159	A01733	375	A00159	A01741	899
A00159	A01742	900	A00159	A01775	940
A00159	A01785	283	A00159	A01796	249
A00159	A01797	899	A00159	A01804	152
A00159	A01844	945	A00159	A01849	360
A00159	A01850	899	A00159	A01877	899
A00159	A01879	427	A00159	A01900	941
A00159	A01905	899	A00159	A01908	204
A00159	A01914	283	A00159	A01920	899
A00159	A01926	899	A00159	A02002	285
A00159	A02054	212	A00159	A02058	899
A00159	A02076	427	A00159	A02086	484
A00159	A02088	899	A00159	A02124	899
A00159	A02148	899	A00159	A02163	921
A00159	A02174	899	A00159	A02183	454
A00159	A02215	899	A00159	A02226	278
A00159	A02268	218	A00159	A02305	899
A00159	A02341	291	A00159	A02342	481
A00159	A02343	485	A00159	A02350	899
A00159	A02362	247	A00159	A02382	353
A00159	A02389	899	A00159	A02411	899
A00159	A02429	899	A00159	A02432	491
A00159	A02465	899	A00159	A02481	154
A00159	A02494	899	A00159	A02513	318
A00159	A02517	899	A00159	A02564	302
A00159	A02578	174	A00159	A02635	199
A00159	A02639	470	A00159	A02680	361
A00159	A02682	183	A00159	A02686	63
A00159	A02698	283	A00159	A02709	302
A00159	A02717	398	A00159	A02731	466
A00159	A02749	156	A00159	A02760	210
A00159	A02778	899	A00159	A02789	899
A00159	A02803	901	A00159	A02828	899
A00159	A02833	429	A00159	A02864	943
A00159	A02869	900	A00159	A02872	899
A00159	A02897	132	A00159	A02904	152
A00159	A02908	943	A00159	A02919	899
A00159	A02942	901	A00159	A02949	899
A00159	A02951	154	A00159	A02958	899
A00159	A02968	899	A00159	A02975	369
A00159	A02976	196	A00159	A03001	472
A00159	A03010	457	A00159	A03014	176
A00159	A03098	199	A00159	A03171	899
A00159	A03204	105	A00159	A03214	899

A00159	A03222	340	A00159	A03223	449
A00159	A03259	899	A00159	A03271	241
A00159	A03292	63	A00159	A03331	899
A00159	A03338	63	A00159	A03406	899
A00159	A03419	356	A00159	A03569	419
A00159	A03575	929	A00159	A03591	899
A00159	A03606	184	A00159	A03639	899
A00159	A03685	899	A00159	A03686	899
A00159	A03726	899	A00159	A03741	899
A00159	A03752	899	A00159	A03771	249
A00159	A03776	899	A00159	A03817	899
A00159	A03821	945	A00159	A03834	212
A00159	A03835	246	A00159	A03837	363
A00159	A03873	290	A00159	A03892	899
A00159	A03984	446	A00159	A03985	372
A00159	A03990	460	A00159	A04010	386
A00159	A04012	177	A00159	A04050	944
A00159	A04051	468	A00159	A04073	899
A00159	A04096	404	A00159	A04097	429
A00159	A04105	477	A00159	A04115	353
A00159	A04117	463	A00159	A04207	367
A00159	A04318	899	A00159	A04368	899
A00159	A04527	378	A00159	A04600	899
A00159	A04608	188	A00159	A04622	484
A00159	A04652	377	A00159	A04668	899
A00159	A04683	899	A00159	A04687	408
A00159	A04914	486	A00159	A04949	545
A00159	A05009	899	A00159	A05029	940
A00159	A05035	899	A00159	A05044	899
A00159	A05062	899	A00159	A05212	446
A00159	A05309	151	A00159	A05315	899
A00159	A05349	936	A00159	A05370	899
A00159	A05414	910	A00159	A05428	899
A00159	A05709	899	A00159	A05760	900
A00159	A05762	183	A00159	A05888	439
A00159	A05938	899	A00159	A05993	899
A00159	A06046	899	A00159	A06118	899
A00159	A06161	208	A00159	A06289	899
A00159	A06295	899	A00159	A06314	899
A00159	A06438	183	A00159	A06458	183
A00159	A06597	442	A00159	A06601	899
A00159	A06610	491	A00159	A06615	474
A00159	A06631	288	A00159	A06642	452
A00159	A06770	190	A00159	A07004	201
A00159	A07034	899	A00159	A07051	213
A00159	A07061	899	A00159	A07091	899

A00159	A07135	491	A00159	A07155	899
A00159	A07157	303	A00159	A07165	942
A00159	A07176	258	A00159	A07187	338
A00159	A07190	395	A00159	A07201	485
A00159	A07303	183	A00159	A07307	899
A00159	A07311	945	A00159	A07314	390
A00159	A07335	470	A00159	A07339	928
A00159	A07590	188	A00159	A07798	899
A00159	A07803	302	A00159	A08133	914
A00159	A08259	899	A00159	A08280	537
A00159	A08400	910	A00159	A08557	899
A00159	A08566	899	A00159	A08567	899
A00159	A08571	957	A00159	A08598	259
A00159	A08732	939	A00159	A08745	165
A00159	A08755	317	A00159	A08818	157
A00159	A08839	899	A00159	A08858	899
A00159	A08859	899	A00159	A08863	899
A00159	A09078	474	A00159	A09127	899
A00159	A09195	216	A00159	A09220	369
A00159	A09298	899	A00159	A09319	899
A00159	A09335	190	A00159	A09388	417
A00159	A09391	305	A00159	A09395	294
A00159	A09524	157	A00159	A09542	899
A00159	A09545	899	A00159	A09556	914
A00159	A09558	899	A00159	A09568	899
A00159	A09577	899	A00159	A09627	152
A00159	A09644	899	A00159	A09654	899
A00159	A09727	899	A00159	A09754	899
A00159	A09785	467	A00159	A09804	899
A00159	A09913	899	A00159	A10159	165
A00159	A10174	923	A00159	A10175	899
A00159	A10218	168	A00159	A10355	165
A00159	A10365	899	A00159	A10392	899
A00159	A10452	371	A00159	A10466	317
A00159	A10501	71	A00159	A10615	899
A00159	A10624	899	A00159	A10721	347
A00159	A10732	71	A00159	A10735	450
A00159	A10757	252	A00159	A10789	899
A00159	A10800	276	A00159	A10801	910
A00159	A10808	899	A00159	A10828	183
A00159	A10869	899	A00159	A10893	183
A00159	A10899	82	A00159	A10904	101
A00159	A10913	63	A00159	A11206	899
A00159	A11262	899	A00159	A11283	899
A00159	A11369	475	A00159	A11487	475
A00159	A11507	183	A00159	A11513	411

A00159	A11612	899	A00159	A11735	899
A00159	A11866	899	A00159	A11931	367
A00159	A11964	152	A00159	A11968	183
A00159	A11983	915	A00159	A12047	899
A00159	A12341	899	A00159	A12383	926
A00159	A12507	899	A00159	A12548	340
A00159	A12652	899	A00159	A12705	899
A00159	A12990	899	A00159	A13099	213
A00159	A13146	899	A00159	A13191	899
A00159	A13225	899	A00159	A13231	243
A00159	A13234	203	A00159	A13250	899
A00159	A13397	899	A00159	A13508	183
A00159	A13511	594	A00159	A13555	184
A00159	A13737	899	A00159	A13824	992
A00159	A13827	63	A00159	A13829	914
A00159	A13849	899	A00159	A13963	899
A00159	A13975	997	A00159	A13978	899
A00159	A13980	490	A00159	A13981	899
A00159	A13982	347	A00159	A13984	899
A00159	A13986	729	A00159	A13989	528
A00159	A13991	899	A00159	A13997	913
A00159	A13998	899	A00159	A14002	899
A00159	A14007	462	A00159	A14008	899
A00159	A14019	917	A00159	A14022	899
A00159	A14023	899	A00159	A14025	899
A00159	A14039	899	A00159	A14086	899
A00159	A14088	899	A00159	A14096	899
A00159	A14159	899	A00159	A14191	909
A00159	A14195	899	A00159	A14196	993
A00159	A14197	905	A00159	A14198	945
A00159	A14200	899	A00159	A14202	899
A00159	A14206	899	A00159	A14207	899
A00159	A14208	951	A00159	A14210	902
A00159	A14218	266	A00159	A14226	899
A00159	A14247	903	A00159	A14252	899
A00159	A14265	185	A00159	A14300	199
A00159	A14303	210	A00159	A14364	191
A00159	A14367	930	A00159	A14379	899
A00159	A14393	899	A00159	A14395	191
A00159	A14430	256	A00159	A14431	363
A00159	A14443	992	A00159	A14444	899
A00159	A14452	944	A00159	A14471	283
A00159	A14490	899	A00159	A14496	190
A00159	A14527	899	A00159	A14643	260
A00159	A14741	217	A00159	A14771	276
A00159	A14856	899	A00159	A15092	183

A00159	A15105	899	A00159	A15291	455
A00159	A15292	899	A00159	A15293	932
A00159	A15294	990	A00159	A15314	641
A00159	A15323	185	A00159	A15336	165
A00159	A15356	899	A00159	A15386	320
A00159	A15420	408	A00159	A15506	921
A00159	A15507	318	A00159	A15510	425
A00159	A15511	263	A00159	A15543	899
A00159	A15547	973	A00159	A15563	899
A00159	A15575	162	A00159	A15578	208
A00159	A15612	193	A00159	A15613	486
A00159	A15617	905	A00159	A15665	899
A00159	A15670	899	A00159	A15681	899
A00159	A15701	474	A00159	A15726	491
A00159	A15834	473	A00159	A15944	899
A00159	A15948	899	A00159	A15949	899
A00159	A16073	899	A00159	A16084	915
A00159	A16211	899	A00159	A16216	577
A00159	A16297	899	A00159	A16363	307
A00159	A16450	212	A00159	A16791	899
A00159	A16809	199	A00159	A16909	899
A00159	A16994	241	A00159	A17011	899
A00159	A17013	899	A00159	A17017	899
A00159	A17034	899	A00159	A17113	267
A00159	A17198	152	A00159	A17210	899
A00160	A00161	945	A00160	A00162	945
A00160	A00167	255	A00160	A00189	491
A00160	A00190	271	A00160	A00200	168
A00160	A00205	489	A00160	A00208	485
A00160	A00224	925	A00160	A00239	350
A00160	A00241	384	A00160	A00243	477
A00160	A00244	299	A00160	A00407	205
A00160	A00799	165	A00160	A01524	286
A00160	A01527	919	A00160	A01553	132
A00160	A01554	384	A00160	A01558	899
A00160	A01588	899	A00160	A01600	193
A00160	A01602	899	A00160	A01615	899
A00160	A01623	263	A00160	A01709	152
A00160	A01876	369	A00160	A01880	274
A00160	A01881	174	A00160	A01889	254
A00160	A02295	199	A00160	A02304	174
A00160	A02382	468	A00160	A02383	202
A00160	A02697	183	A00160	A02698	466
A00160	A02706	263	A00160	A02709	260
A00160	A02711	491	A00160	A02795	213
A00160	A02820	241	A00160	A02825	154

A00160	A02906	238	A00160	A02986	348
A00160	A03010	490	A00160	A03081	168
A00160	A03218	200	A00160	A03536	218
A00160	A03587	196	A00160	A03650	311
A00160	A03685	344	A00160	A04033	294
A00160	A04279	157	A00160	A04320	333
A00160	A04419	319	A00160	A04449	152
A00160	A04478	205	A00160	A04535	243
A00160	A04567	180	A00160	A04699	159
A00160	A04922	198	A00160	A05070	185
A00160	A05094	165	A00160	A05106	358
A00160	A05272	366	A00160	A05335	156
A00160	A05587	256	A00160	A05847	216
A00160	A06225	411	A00160	A06404	245
A00160	A06603	317	A00160	A06708	174
A00160	A06868	306	A00160	A07293	344
A00160	A07390	216	A00160	A07891	262
A00160	A08171	174	A00160	A08926	180
A00160	A09487	202	A00160	A09884	291
A00160	A09937	198	A00160	A10358	173
A00160	A10626	245	A00160	A10813	187
A00160	A10899	488	A00160	A11109	277
A00160	A11348	193	A00160	A11369	79
A00160	A11413	435	A00160	A11450	386
A00160	A11543	409	A00160	A11654	311
A00160	A12086	269	A00160	A12371	219
A00160	A12586	366	A00160	A12668	925
A00160	A12992	198	A00160	A13860	347
A00160	A13978	274	A00160	A13980	313
A00160	A14250	373	A00160	A15241	279
A00160	A15263	344	A00160	A15726	474
A00160	A15952	344	A00160	A15981	218
A00160	A16844	201	A00161	A00162	973
A00161	A00167	955	A00161	A00168	424
A00161	A00180	268	A00161	A00185	936
A00161	A00190	297	A00161	A00205	971
A00161	A00207	480	A00161	A00208	983
A00161	A00209	947	A00161	A00214	193
A00161	A00224	949	A00161	A00230	186
A00161	A00232	195	A00161	A00234	328
A00161	A00240	190	A00161	A00243	357
A00161	A00246	909	A00161	A00254	943
A00161	A00255	184	A00161	A00262	217
A00161	A00292	375	A00161	A00451	429
A00161	A00566	249	A00161	A00696	301
A00161	A00911	151	A00161	A00931	446

A00161	A01131	257	A00161	A01155	366
A00161	A01286	187	A00161	A01307	333
A00161	A01342	462	A00161	A01493	154
A00161	A01524	993	A00161	A01527	899
A00161	A01553	903	A00161	A01554	900
A00161	A01560	234	A00161	A01572	343
A00161	A01726	394	A00161	A01796	466
A00161	A01824	173	A00161	A01914	434
A00161	A01941	234	A00161	A02013	263
A00161	A02086	489	A00161	A02331	491
A00161	A02420	191	A00161	A02502	283
A00161	A02658	324	A00161	A02708	313
A00161	A02711	440	A00161	A02746	118
A00161	A02877	435	A00161	A02878	205
A00161	A02882	121	A00161	A02908	340
A00161	A02982	491	A00161	A03019	63
A00161	A03047	151	A00161	A03225	446
A00161	A03234	167	A00161	A03259	63
A00161	A03681	247	A00161	A03685	229
A00161	A03742	234	A00161	A03800	168
A00161	A03803	473	A00161	A03809	454
A00161	A03821	152	A00161	A03837	346
A00161	A03880	462	A00161	A04006	412
A00161	A04115	391	A00161	A04117	260
A00161	A04288	294	A00161	A04521	243
A00161	A04956	174	A00161	A05339	179
A00161	A05621	191	A00161	A05816	157
A00161	A05876	328	A00161	A07311	930
A00161	A08324	243	A00161	A09220	335
A00161	A09363	899	A00161	A09577	63
A00161	A09719	154	A00161	A09913	151
A00161	A10379	294	A00161	A10724	157
A00161	A10801	899	A00161	A10899	167
A00161	A10913	147	A00161	A10941	458
A00161	A11120	191	A00161	A11369	491
A00161	A11413	160	A00161	A13504	217
A00161	A13980	796	A00161	A13990	252
A00161	A14033	217	A00161	A14062	152
A00161	A14127	324	A00161	A14193	644
A00161	A14198	266	A00161	A14209	191
A00161	A14393	191	A00161	A14433	906
A00161	A14443	899	A00161	A14444	933
A00161	A14644	440	A00161	A14686	154
A00161	A15263	263	A00161	A15319	174
A00161	A15323	243	A00161	A15556	462
A00161	A15644	462	A00161	A16986	372

A00162	A00163	283	A00162	A00166	340
A00162	A00167	941	A00162	A00169	245
A00162	A00180	291	A00162	A00184	899
A00162	A00185	945	A00162	A00186	99
A00162	A00189	394	A00162	A00190	266
A00162	A00205	970	A00162	A00207	969
A00162	A00208	977	A00162	A00209	899
A00162	A00211	185	A00162	A00214	238
A00162	A00224	965	A00162	A00230	911
A00162	A00234	615	A00162	A00239	201
A00162	A00240	639	A00162	A00241	205
A00162	A00242	156	A00162	A00243	363
A00162	A00246	942	A00162	A00250	922
A00162	A00254	945	A00162	A00255	457
A00162	A00261	407	A00162	A00270	334
A00162	A00271	899	A00162	A00292	281
A00162	A00310	899	A00162	A00312	903
A00162	A00324	266	A00162	A00340	918
A00162	A00381	256	A00162	A00566	224
A00162	A00820	249	A00162	A00825	185
A00162	A01052	899	A00162	A01070	899
A00162	A01112	445	A00162	A01291	312
A00162	A01310	354	A00162	A01452	742
A00162	A01473	899	A00162	A01498	218
A00162	A01524	945	A00162	A01527	944
A00162	A01553	976	A00162	A01554	944
A00162	A01558	899	A00162	A01560	481
A00162	A01562	899	A00162	A01572	491
A00162	A01588	899	A00162	A01606	238
A00162	A01615	899	A00162	A01624	187
A00162	A01628	899	A00162	A01688	899
A00162	A01708	899	A00162	A01709	929
A00162	A01730	918	A00162	A01740	899
A00162	A01747	201	A00162	A01759	234
A00162	A01787	378	A00162	A01794	205
A00162	A01800	208	A00162	A01894	247
A00162	A01910	180	A00162	A01914	644
A00162	A02284	899	A00162	A02304	323
A00162	A02330	899	A00162	A02502	454
A00162	A02711	489	A00162	A02747	152
A00162	A02749	63	A00162	A02803	208
A00162	A02985	172	A00162	A03010	232
A00162	A03099	926	A00162	A03123	187
A00162	A03213	930	A00162	A03225	423
A00162	A03263	216	A00162	A03273	927
A00162	A03280	200	A00162	A03598	288

A00162	A03646	218	A00162	A03685	454
A00162	A03804	162	A00162	A03822	228
A00162	A03878	172	A00162	A03971	152
A00162	A04006	229	A00162	A04051	296
A00162	A04280	228	A00162	A04301	326
A00162	A04451	213	A00162	A04623	180
A00162	A04857	296	A00162	A05064	332
A00162	A05208	899	A00162	A05621	172
A00162	A05780	193	A00162	A06039	199
A00162	A06166	193	A00162	A06202	167
A00162	A06610	458	A00162	A06616	172
A00162	A06655	172	A00162	A06762	899
A00162	A06926	363	A00162	A07311	945
A00162	A07460	899	A00162	A09181	212
A00162	A09214	218	A00162	A09220	942
A00162	A09363	899	A00162	A09377	167
A00162	A09418	201	A00162	A09542	925
A00162	A09577	63	A00162	A09694	193
A00162	A09801	160	A00162	A09937	462
A00162	A10249	208	A00162	A10340	172
A00162	A10580	912	A00162	A10801	989
A00162	A10899	170	A00162	A10941	460
A00162	A11125	899	A00162	A11369	394
A00162	A11392	188	A00162	A11512	296
A00162	A11535	223	A00162	A11707	187
A00162	A11758	230	A00162	A12040	246
A00162	A12494	266	A00162	A12765	246
A00162	A13131	152	A00162	A13975	208
A00162	A13980	977	A00162	A13989	486
A00162	A13990	945	A00162	A14027	208
A00162	A14033	218	A00162	A14043	899
A00162	A14198	945	A00162	A14208	426
A00162	A14209	172	A00162	A14389	172
A00162	A14402	422	A00162	A14433	917
A00162	A14442	208	A00162	A14443	899
A00162	A14444	991	A00162	A14503	218
A00162	A14776	266	A00162	A14943	232
A00162	A15241	185	A00162	A15263	266
A00162	A15293	899	A00162	A15294	899
A00162	A15315	157	A00162	A15394	440
A00162	A15395	350	A00162	A15952	266
A00162	A15964	420	A00162	A16231	347
A00163	A00165	152	A00163	A00167	491
A00163	A00168	486	A00163	A00169	945
A00163	A00171	491	A00163	A00173	452
A00163	A00176	198	A00163	A00185	490

A00163	A00189	299	A00163	A00190	491
A00163	A00200	994	A00163	A00207	276
A00163	A00208	343	A00163	A00209	955
A00163	A00216	395	A00163	A00219	486
A00163	A00224	944	A00163	A00230	899
A00163	A00231	157	A00163	A00239	322
A00163	A00240	202	A00163	A00243	195
A00163	A00245	425	A00163	A00246	899
A00163	A00248	900	A00163	A00254	565
A00163	A00256	351	A00163	A00257	200
A00163	A00264	475	A00163	A00265	916
A00163	A00270	899	A00163	A00271	899
A00163	A00294	490	A00163	A00301	173
A00163	A00305	411	A00163	A00309	491
A00163	A00316	491	A00163	A00324	944
A00163	A00331	339	A00163	A00340	274
A00163	A00370	447	A00163	A00378	291
A00163	A00400	324	A00163	A00552	491
A00163	A00597	422	A00163	A00600	469
A00163	A00601	183	A00163	A00628	260
A00163	A00643	395	A00163	A00649	283
A00163	A00661	247	A00163	A00662	218
A00163	A00691	346	A00163	A00696	402
A00163	A00733	168	A00163	A00736	475
A00163	A00747	451	A00163	A00757	491
A00163	A00822	418	A00163	A00883	185
A00163	A00887	944	A00163	A00914	257
A00163	A00939	404	A00163	A00981	489
A00163	A00997	433	A00163	A01022	358
A00163	A01052	490	A00163	A01088	270
A00163	A01125	408	A00163	A01157	468
A00163	A01178	156	A00163	A01211	458
A00163	A01213	319	A00163	A01235	480
A00163	A01256	475	A00163	A01257	252
A00163	A01289	283	A00163	A01512	991
A00163	A01524	944	A00163	A01527	945
A00163	A01531	247	A00163	A01558	485
A00163	A01559	218	A00163	A01560	209
A00163	A01571	461	A00163	A01602	430
A00163	A01626	180	A00163	A01655	277
A00163	A01665	899	A00163	A01667	383
A00163	A01668	465	A00163	A01676	432
A00163	A01688	433	A00163	A01700	435
A00163	A01718	899	A00163	A01726	945
A00163	A01727	446	A00163	A01729	440
A00163	A01730	477	A00163	A01733	172

A00163	A01737	243	A00163	A01740	218
A00163	A01741	491	A00163	A01743	489
A00163	A01747	491	A00163	A01748	465
A00163	A01754	177	A00163	A01763	467
A00163	A01767	412	A00163	A01784	151
A00163	A01786	945	A00163	A01797	162
A00163	A01841	441	A00163	A01844	904
A00163	A01876	307	A00163	A01878	485
A00163	A01886	162	A00163	A01888	905
A00163	A01891	345	A00163	A01897	322
A00163	A01900	488	A00163	A01903	170
A00163	A01931	283	A00163	A01990	187
A00163	A01995	599	A00163	A02006	246
A00163	A02117	353	A00163	A02180	472
A00163	A02199	371	A00163	A02228	260
A00163	A02369	177	A00163	A02411	899
A00163	A02477	196	A00163	A02482	491
A00163	A02491	324	A00163	A02494	283
A00163	A02495	491	A00163	A02502	380
A00163	A02507	457	A00163	A02548	196
A00163	A02559	172	A00163	A02574	162
A00163	A02611	335	A00163	A02614	235
A00163	A02628	259	A00163	A02639	317
A00163	A02673	291	A00163	A02694	159
A00163	A02695	267	A00163	A02696	157
A00163	A02697	477	A00163	A02698	468
A00163	A02705	491	A00163	A02706	491
A00163	A02707	443	A00163	A02708	210
A00163	A02731	455	A00163	A02733	160
A00163	A02812	224	A00163	A02817	491
A00163	A02818	488	A00163	A02819	481
A00163	A02820	390	A00163	A02822	899
A00163	A02830	402	A00163	A02836	488
A00163	A02837	457	A00163	A02855	483
A00163	A02859	468	A00163	A02861	481
A00163	A02862	475	A00163	A02874	347
A00163	A02915	443	A00163	A02921	210
A00163	A02924	293	A00163	A02928	469
A00163	A02968	899	A00163	A03014	218
A00163	A03027	268	A00163	A03034	227
A00163	A03051	174	A00163	A03068	286
A00163	A03070	235	A00163	A03076	458
A00163	A03095	212	A00163	A03112	434
A00163	A03141	312	A00163	A03167	170
A00163	A03228	486	A00163	A03244	207
A00163	A03275	290	A00163	A03292	491

A00163	A03304	485	A00163	A03434	455
A00163	A03435	208	A00163	A03436	489
A00163	A03451	473	A00163	A03459	313
A00163	A03520	372	A00163	A03587	418
A00163	A03611	461	A00163	A03649	491
A00163	A03653	174	A00163	A03662	412
A00163	A03719	215	A00163	A03723	156
A00163	A03725	316	A00163	A03726	899
A00163	A03739	471	A00163	A03749	483
A00163	A03762	154	A00163	A03771	491
A00163	A03775	279	A00163	A03811	157
A00163	A03841	489	A00163	A03926	291
A00163	A03933	321	A00163	A03949	490
A00163	A03976	308	A00163	A03990	674
A00163	A04006	414	A00163	A04020	240
A00163	A04046	418	A00163	A04080	346
A00163	A04091	491	A00163	A04096	899
A00163	A04134	307	A00163	A04142	204
A00163	A04174	152	A00163	A04270	273
A00163	A04280	445	A00163	A04355	441
A00163	A04417	201	A00163	A04449	221
A00163	A04456	180	A00163	A04483	235
A00163	A04500	180	A00163	A04516	245
A00163	A04532	475	A00163	A04533	423
A00163	A04539	321	A00163	A04561	227
A00163	A04590	180	A00163	A04606	180
A00163	A04607	899	A00163	A04622	469
A00163	A04683	227	A00163	A04684	180
A00163	A04698	462	A00163	A04731	167
A00163	A04740	263	A00163	A04747	154
A00163	A04758	218	A00163	A04763	326
A00163	A04804	218	A00163	A04810	157
A00163	A04813	291	A00163	A05018	157
A00163	A05034	443	A00163	A05052	468
A00163	A05070	485	A00163	A05093	235
A00163	A05216	491	A00163	A05219	324
A00163	A05240	272	A00163	A05250	198
A00163	A05287	218	A00163	A05288	491
A00163	A05331	232	A00163	A05351	256
A00163	A05353	241	A00163	A05392	157
A00163	A05418	489	A00163	A05470	157
A00163	A05473	286	A00163	A05537	218
A00163	A05546	488	A00163	A05574	246
A00163	A05592	313	A00163	A05671	313
A00163	A05866	291	A00163	A05877	218
A00163	A05940	317	A00163	A05979	196

A00163	A06092	329	A00163	A06195	299
A00163	A06214	218	A00163	A06225	477
A00163	A06258	232	A00163	A06261	157
A00163	A06332	218	A00163	A06379	371
A00163	A06392	218	A00163	A06400	491
A00163	A06520	218	A00163	A06543	263
A00163	A06544	422	A00163	A06581	472
A00163	A06588	454	A00163	A06592	330
A00163	A06597	361	A00163	A06606	480
A00163	A06607	472	A00163	A06608	900
A00163	A06609	913	A00163	A06610	438
A00163	A06615	388	A00163	A06623	348
A00163	A06683	246	A00163	A06688	335
A00163	A06798	469	A00163	A06949	196
A00163	A07006	246	A00163	A07163	162
A00163	A07165	400	A00163	A07197	486
A00163	A07198	486	A00163	A07311	940
A00163	A07315	472	A00163	A07339	709
A00163	A07350	180	A00163	A07381	301
A00163	A07400	218	A00163	A07421	491
A00163	A07422	899	A00163	A07442	291
A00163	A07564	157	A00163	A07576	218
A00163	A07659	329	A00163	A08070	475
A00163	A08389	198	A00163	A08630	152
A00163	A08727	204	A00163	A08837	469
A00163	A08861	201	A00163	A08897	218
A00163	A09014	366	A00163	A09020	199
A00163	A09078	491	A00163	A09170	313
A00163	A09235	337	A00163	A09319	899
A00163	A09363	267	A00163	A09367	157
A00163	A09371	157	A00163	A09408	180
A00163	A09457	157	A00163	A09462	180
A00163	A09561	404	A00163	A09832	157
A00163	A09882	371	A00163	A09944	218
A00163	A09951	180	A00163	A10049	157
A00163	A10072	379	A00163	A10102	218
A00163	A10114	226	A00163	A10223	157
A00163	A10233	185	A00163	A10324	313
A00163	A10360	218	A00163	A10532	446
A00163	A10631	484	A00163	A10936	218
A00163	A10960	185	A00163	A10975	218
A00163	A10986	281	A00163	A11169	154
A00163	A11193	405	A00163	A11215	180
A00163	A11275	177	A00163	A11487	491
A00163	A11663	198	A00163	A11779	489
A00163	A11932	182	A00163	A11978	218

A00163	A11983	472	A00163	A12147	157
A00163	A12373	944	A00163	A12523	291
A00163	A12592	291	A00163	A12752	157
A00163	A12796	180	A00163	A12897	420
A00163	A12898	291	A00163	A13079	180
A00163	A13080	180	A00163	A13146	899
A00163	A13147	396	A00163	A13168	180
A00163	A13229	321	A00163	A13250	900
A00163	A13373	185	A00163	A13437	157
A00163	A13617	299	A00163	A13648	180
A00163	A13824	899	A00163	A13865	180
A00163	A13945	430	A00163	A13977	899
A00163	A13980	899	A00163	A14006	491
A00163	A14024	291	A00163	A14055	899
A00163	A14198	614	A00163	A14210	899
A00163	A14308	180	A00163	A14332	151
A00163	A14386	483	A00163	A14390	438
A00163	A14399	188	A00163	A14434	455
A00163	A14436	210	A00163	A14443	313
A00163	A14577	296	A00163	A14589	204
A00163	A14643	466	A00163	A14649	462
A00163	A14674	357	A00163	A14712	345
A00163	A14715	180	A00163	A14729	259
A00163	A14742	218	A00163	A14765	218
A00163	A14811	378	A00163	A14824	180
A00163	A14857	188	A00163	A14930	899
A00163	A15041	218	A00163	A15046	218
A00163	A15293	585	A00163	A15294	177
A00163	A15318	152	A00163	A15504	256
A00163	A15506	533	A00163	A15675	210
A00163	A15840	486	A00163	A15841	235
A00163	A15927	157	A00163	A15971	235
A00163	A16300	469	A00163	A16317	157
A00163	A16347	180	A00163	A16348	218
A00163	A16363	360	A00163	A16500	218
A00163	A16677	157	A00163	A16679	177
A00163	A16718	226	A00163	A16741	241
A00163	A16795	187	A00163	A16887	235
A00164	A00216	262	A00164	A00446	599
A00164	A00583	262	A00164	A00691	193
A00164	A00981	217	A00164	A01158	233
A00164	A01358	241	A00164	A01372	597
A00164	A01390	217	A00164	A01602	184
A00164	A02565	408	A00164	A02658	157
A00164	A02942	422	A00164	A03219	184
A00164	A03484	224	A00164	A03719	184

A00164	A03859	486	A00164	A03873	262
A00164	A06067	184	A00164	A06578	184
A00164	A06608	441	A00164	A07346	486
A00164	A07598	546	A00164	A07956	262
A00164	A08248	262	A00164	A08524	491
A00164	A08982	262	A00164	A09351	364
A00164	A11544	262	A00164	A11558	184
A00164	A11649	262	A00164	A11675	151
A00164	A11851	532	A00164	A12052	182
A00164	A12723	236	A00164	A13356	556
A00164	A13781	271	A00164	A13784	375
A00164	A13855	340	A00164	A13892	262
A00164	A14167	208	A00164	A14210	184
A00164	A14214	221	A00164	A14362	262
A00164	A15321	575	A00164	A15338	262
A00164	A15559	268	A00164	A15564	262
A00164	A16152	184	A00164	A16215	419
A00164	A16588	163	A00164	A16611	191
A00164	A16614	262	A00164	A16690	450
A00164	A16866	171	A00164	A17133	173
A00165	A00167	213	A00165	A00179	177
A00165	A00183	273	A00165	A00187	319
A00165	A00188	422	A00165	A00198	491
A00165	A00201	560	A00165	A00224	919
A00165	A00230	913	A00165	A00239	593
A00165	A00240	704	A00165	A00243	601
A00165	A00246	899	A00165	A00248	335
A00165	A00257	535	A00165	A00262	262
A00165	A00269	183	A00165	A00270	488
A00165	A00324	483	A00165	A00440	350
A00165	A00590	265	A00165	A00621	301
A00165	A00691	232	A00165	A00765	485
A00165	A00813	172	A00165	A00822	163
A00165	A01088	172	A00165	A01148	157
A00165	A01178	458	A00165	A01213	208
A00165	A01252	443	A00165	A01263	232
A00165	A01312	356	A00165	A01378	413
A00165	A01410	243	A00165	A01507	480
A00165	A01512	944	A00165	A01524	932
A00165	A01527	899	A00165	A01554	936
A00165	A01558	899	A00165	A01560	224
A00165	A01576	642	A00165	A01580	517
A00165	A01588	899	A00165	A01596	949
A00165	A01601	488	A00165	A01606	489
A00165	A01628	63	A00165	A01639	945
A00165	A01688	317	A00165	A01697	63

A00165	A01809	327	A00165	A01850	402
A00165	A01974	375	A00165	A02361	386
A00165	A02382	417	A00165	A02411	159
A00165	A02703	265	A00165	A03329	252
A00165	A03445	162	A00165	A03575	677
A00165	A03741	497	A00165	A03822	271
A00165	A04033	489	A00165	A04449	543
A00165	A04686	156	A00165	A04699	917
A00165	A05009	463	A00165	A05015	218
A00165	A05029	152	A00165	A05032	485
A00165	A05553	208	A00165	A06277	168
A00165	A06348	229	A00165	A06466	230
A00165	A06601	469	A00165	A06701	480
A00165	A06779	327	A00165	A06914	232
A00165	A07311	138	A00165	A07422	951
A00165	A08308	160	A00165	A08356	165
A00165	A08552	236	A00165	A08571	950
A00165	A08692	394	A00165	A08858	445
A00165	A08869	177	A00165	A08878	229
A00165	A09162	446	A00165	A09767	435
A00165	A09804	301	A00165	A09976	241
A00165	A10365	63	A00165	A10392	154
A00165	A10580	952	A00165	A10623	491
A00165	A10728	491	A00165	A10743	200
A00165	A10808	480	A00165	A10982	469
A00165	A11300	251	A00165	A11379	489
A00165	A11621	491	A00165	A11983	703
A00165	A12036	234	A00165	A12047	509
A00165	A12664	324	A00165	A12824	386
A00165	A12920	317	A00165	A12971	104
A00165	A13274	160	A00165	A13977	363
A00165	A13997	185	A00165	A14005	598
A00165	A14055	356	A00165	A14182	188
A00165	A14208	235	A00165	A14229	246
A00165	A14299	488	A00165	A14443	237
A00165	A14571	193	A00165	A14635	436
A00165	A14653	63	A00165	A15322	463
A00165	A15357	333	A00165	A15381	468
A00165	A15741	305	A00165	A15945	265
A00165	A16400	208	A00165	A17160	899
A00166	A00168	598	A00166	A00209	926
A00166	A00211	982	A00166	A00217	353
A00166	A00219	296	A00166	A00221	491
A00166	A00229	329	A00166	A00230	899
A00166	A00815	467	A00166	A01125	510
A00166	A01258	286	A00166	A01310	794

A00166	A01605	945	A00166	A01640	452
A00166	A01914	403	A00166	A02088	162
A00166	A02313	288	A00166	A02323	199
A00166	A02507	228	A00166	A02673	224
A00166	A03228	157	A00166	A03690	176
A00166	A03799	217	A00166	A03800	296
A00166	A03942	229	A00166	A04012	168
A00166	A04367	299	A00166	A04422	468
A00166	A04490	428	A00166	A04749	249
A00166	A04756	323	A00166	A04790	406
A00166	A05090	277	A00166	A05091	341
A00166	A05221	490	A00166	A05337	185
A00166	A05407	258	A00166	A06032	184
A00166	A06392	363	A00166	A06578	347
A00166	A07141	187	A00166	A07311	899
A00166	A07367	281	A00166	A07422	899
A00166	A09077	273	A00166	A10040	251
A00166	A11313	247	A00166	A11392	176
A00166	A11614	818	A00166	A11646	171
A00166	A12897	238	A00166	A13370	269
A00166	A14038	215	A00166	A14198	288
A00166	A14371	216	A00166	A15304	899
A00166	A15305	927	A00166	A15342	218
A00166	A15455	235	A00166	A16904	435
A00166	A17102	185	A00167	A00168	491
A00167	A00169	663	A00167	A00171	491
A00167	A00172	899	A00167	A00173	352
A00167	A00174	899	A00167	A00175	899
A00167	A00176	961	A00167	A00177	266
A00167	A00178	899	A00167	A00179	491
A00167	A00180	176	A00167	A00181	171
A00167	A00182	369	A00167	A00183	486
A00167	A00184	899	A00167	A00185	943
A00167	A00189	944	A00167	A00190	945
A00167	A00191	944	A00167	A00195	936
A00167	A00199	899	A00167	A00200	491
A00167	A00202	488	A00167	A00203	899
A00167	A00205	899	A00167	A00206	899
A00167	A00207	899	A00167	A00208	945
A00167	A00209	998	A00167	A00212	276
A00167	A00216	278	A00167	A00219	491
A00167	A00220	990	A00167	A00221	910
A00167	A00223	993	A00167	A00224	962
A00167	A00228	363	A00167	A00230	899
A00167	A00231	931	A00167	A00233	281
A00167	A00236	944	A00167	A00239	899

A00167	A00240	899	A00167	A00241	901
A00167	A00242	899	A00167	A00243	899
A00167	A00244	899	A00167	A00245	491
A00167	A00246	899	A00167	A00247	899
A00167	A00250	899	A00167	A00252	945
A00167	A00253	945	A00167	A00254	491
A00167	A00256	383	A00167	A00257	474
A00167	A00258	436	A00167	A00262	424
A00167	A00263	486	A00167	A00268	899
A00167	A00270	949	A00167	A00271	920
A00167	A00272	899	A00167	A00274	914
A00167	A00275	899	A00167	A00276	899
A00167	A00294	491	A00167	A00304	239
A00167	A00308	327	A00167	A00309	490
A00167	A00322	294	A00167	A00324	491
A00167	A00330	560	A00167	A00337	257
A00167	A00351	266	A00167	A00356	905
A00167	A00358	899	A00167	A00370	404
A00167	A00377	418	A00167	A00400	491
A00167	A00411	204	A00167	A00420	491
A00167	A00440	612	A00167	A00449	404
A00167	A00472	477	A00167	A00482	412
A00167	A00485	154	A00167	A00496	274
A00167	A00531	899	A00167	A00546	489
A00167	A00556	472	A00167	A00560	286
A00167	A00587	422	A00167	A00600	432
A00167	A00616	474	A00167	A00630	162
A00167	A00643	537	A00167	A00651	905
A00167	A00656	245	A00167	A00669	335
A00167	A00671	491	A00167	A00696	471
A00167	A00719	191	A00167	A00743	363
A00167	A00747	385	A00167	A00752	342
A00167	A00757	362	A00167	A00781	352
A00167	A00799	399	A00167	A00815	477
A00167	A00820	491	A00167	A00835	157
A00167	A00851	899	A00167	A00853	367
A00167	A00875	370	A00167	A00883	490
A00167	A00887	556	A00167	A00900	277
A00167	A00914	486	A00167	A00921	398
A00167	A00937	454	A00167	A00957	480
A00167	A00967	180	A00167	A00981	539
A00167	A00997	202	A00167	A01009	259
A00167	A01011	609	A00167	A01022	229
A00167	A01052	491	A00167	A01056	937
A00167	A01070	937	A00167	A01091	491
A00167	A01112	549	A00167	A01125	491

A00167	A01137	491	A00167	A01139	899
A00167	A01140	591	A00167	A01143	491
A00167	A01157	411	A00167	A01163	213
A00167	A01164	490	A00167	A01168	899
A00167	A01209	530	A00167	A01211	200
A00167	A01213	477	A00167	A01235	490
A00167	A01252	909	A00167	A01257	462
A00167	A01289	958	A00167	A01291	903
A00167	A01295	176	A00167	A01299	491
A00167	A01305	472	A00167	A01332	316
A00167	A01359	899	A00167	A01378	461
A00167	A01405	200	A00167	A01406	251
A00167	A01425	942	A00167	A01454	506
A00167	A01474	465	A00167	A01475	238
A00167	A01478	364	A00167	A01485	310
A00167	A01492	899	A00167	A01494	905
A00167	A01496	899	A00167	A01500	185
A00167	A01507	484	A00167	A01512	934
A00167	A01524	958	A00167	A01527	957
A00167	A01531	472	A00167	A01542	903
A00167	A01543	396	A00167	A01554	899
A00167	A01557	541	A00167	A01558	904
A00167	A01560	899	A00167	A01562	935
A00167	A01565	259	A00167	A01576	899
A00167	A01582	483	A00167	A01588	899
A00167	A01590	475	A00167	A01593	899
A00167	A01602	488	A00167	A01604	899
A00167	A01605	945	A00167	A01606	480
A00167	A01607	154	A00167	A01615	899
A00167	A01630	288	A00167	A01632	152
A00167	A01639	380	A00167	A01640	902
A00167	A01654	485	A00167	A01658	899
A00167	A01660	681	A00167	A01661	223
A00167	A01668	254	A00167	A01670	174
A00167	A01676	373	A00167	A01688	481
A00167	A01698	489	A00167	A01702	480
A00167	A01718	899	A00167	A01722	303
A00167	A01723	468	A00167	A01726	490
A00167	A01729	255	A00167	A01730	940
A00167	A01733	448	A00167	A01740	899
A00167	A01741	462	A00167	A01747	490
A00167	A01763	395	A00167	A01775	491
A00167	A01781	159	A00167	A01790	491
A00167	A01796	358	A00167	A01844	942
A00167	A01850	276	A00167	A01892	154
A00167	A01902	334	A00167	A01903	491

A00167	A01905	330	A00167	A01926	160
A00167	A01931	491	A00167	A01936	340
A00167	A01959	480	A00167	A01974	899
A00167	A01994	461	A00167	A01995	539
A00167	A02000	157	A00167	A02004	296
A00167	A02010	472	A00167	A02020	472
A00167	A02044	899	A00167	A02074	230
A00167	A02083	475	A00167	A02148	348
A00167	A02149	173	A00167	A02156	307
A00167	A02163	925	A00167	A02174	379
A00167	A02192	151	A00167	A02198	179
A00167	A02199	491	A00167	A02212	347
A00167	A02226	322	A00167	A02228	491
A00167	A02229	367	A00167	A02235	176
A00167	A02278	469	A00167	A02304	491
A00167	A02306	483	A00167	A02313	383
A00167	A02333	340	A00167	A02382	473
A00167	A02397	305	A00167	A02411	905
A00167	A02412	491	A00167	A02431	252
A00167	A02443	255	A00167	A02452	293
A00167	A02467	441	A00167	A02477	162
A00167	A02481	167	A00167	A02482	375
A00167	A02490	293	A00167	A02491	457
A00167	A02494	610	A00167	A02501	160
A00167	A02502	489	A00167	A02505	488
A00167	A02531	167	A00167	A02564	168
A00167	A02621	165	A00167	A02624	433
A00167	A02673	486	A00167	A02687	184
A00167	A02698	612	A00167	A02704	303
A00167	A02711	484	A00167	A02759	305
A00167	A02761	475	A00167	A02787	441
A00167	A02788	422	A00167	A02812	420
A00167	A02833	391	A00167	A02836	474
A00167	A02839	491	A00167	A02874	435
A00167	A02875	480	A00167	A02877	462
A00167	A02887	899	A00167	A02902	321
A00167	A02917	899	A00167	A02924	157
A00167	A02945	191	A00167	A02959	235
A00167	A02981	427	A00167	A03007	154
A00167	A03018	281	A00167	A03034	297
A00167	A03051	270	A00167	A03056	398
A00167	A03076	945	A00167	A03077	441
A00167	A03080	241	A00167	A03081	945
A00167	A03095	491	A00167	A03099	490
A00167	A03104	491	A00167	A03136	469
A00167	A03141	483	A00167	A03186	217

A00167	A03188	491	A00167	A03211	468
A00167	A03213	481	A00167	A03219	899
A00167	A03292	198	A00167	A03304	491
A00167	A03312	180	A00167	A03314	279
A00167	A03315	363	A00167	A03332	899
A00167	A03334	357	A00167	A03415	463
A00167	A03434	603	A00167	A03435	240
A00167	A03436	489	A00167	A03444	899
A00167	A03451	684	A00167	A03584	152
A00167	A03591	441	A00167	A03611	385
A00167	A03639	269	A00167	A03697	205
A00167	A03719	485	A00167	A03726	544
A00167	A03739	204	A00167	A03771	440
A00167	A03778	791	A00167	A03799	439
A00167	A03800	435	A00167	A03819	339
A00167	A03854	469	A00167	A03878	205
A00167	A03882	184	A00167	A03893	613
A00167	A03906	477	A00167	A03909	234
A00167	A03913	899	A00167	A03946	438
A00167	A03961	469	A00167	A04002	488
A00167	A04020	160	A00167	A04033	491
A00167	A04050	428	A00167	A04071	277
A00167	A04079	357	A00167	A04082	235
A00167	A04152	157	A00167	A04162	477
A00167	A04163	329	A00167	A04169	333
A00167	A04202	483	A00167	A04222	424
A00167	A04250	152	A00167	A04259	176
A00167	A04275	200	A00167	A04282	262
A00167	A04316	184	A00167	A04334	422
A00167	A04361	200	A00167	A04441	199
A00167	A04449	625	A00167	A04454	266
A00167	A04548	460	A00167	A04569	176
A00167	A04622	424	A00167	A04737	216
A00167	A04746	235	A00167	A04761	899
A00167	A04770	174	A00167	A04773	486
A00167	A04818	312	A00167	A04830	162
A00167	A04873	407	A00167	A04949	386
A00167	A05027	259	A00167	A05029	899
A00167	A05087	318	A00167	A05221	489
A00167	A05264	277	A00167	A05288	491
A00167	A05309	428	A00167	A05315	899
A00167	A05377	324	A00167	A05418	864
A00167	A05439	472	A00167	A05449	159
A00167	A05450	474	A00167	A05533	212
A00167	A05556	344	A00167	A05607	176
A00167	A05666	436	A00167	A05701	196

A00167	A05721	402	A00167	A05809	900
A00167	A05810	447	A00167	A05853	486
A00167	A05854	416	A00167	A05867	899
A00167	A05869	480	A00167	A05886	160
A00167	A05887	173	A00167	A05891	473
A00167	A05944	486	A00167	A05979	235
A00167	A05996	899	A00167	A06038	283
A00167	A06057	162	A00167	A06092	406
A00167	A06190	323	A00167	A06277	491
A00167	A06286	217	A00167	A06298	402
A00167	A06305	899	A00167	A06317	469
A00167	A06415	160	A00167	A06554	218
A00167	A06578	258	A00167	A06581	512
A00167	A06588	645	A00167	A06591	495
A00167	A06599	186	A00167	A06607	491
A00167	A06608	425	A00167	A06609	899
A00167	A06610	455	A00167	A06615	489
A00167	A06628	232	A00167	A06650	491
A00167	A06688	491	A00167	A06731	324
A00167	A06769	484	A00167	A06814	428
A00167	A06907	491	A00167	A06914	364
A00167	A06999	241	A00167	A07054	274
A00167	A07132	260	A00167	A07149	302
A00167	A07165	413	A00167	A07173	210
A00167	A07176	491	A00167	A07192	399
A00167	A07206	157	A00167	A07209	491
A00167	A07242	644	A00167	A07311	952
A00167	A07314	404	A00167	A07339	491
A00167	A07387	154	A00167	A07422	899
A00167	A07726	546	A00167	A07803	491
A00167	A07833	490	A00167	A07934	283
A00167	A07967	199	A00167	A08280	457
A00167	A08315	160	A00167	A08325	241
A00167	A08402	294	A00167	A08643	204
A00167	A08659	154	A00167	A08718	491
A00167	A08746	163	A00167	A08837	208
A00167	A08850	274	A00167	A08859	684
A00167	A08869	488	A00167	A08875	490
A00167	A08878	966	A00167	A08929	353
A00167	A08943	899	A00167	A09018	407
A00167	A09078	474	A00167	A09124	308
A00167	A09149	187	A00167	A09162	424
A00167	A09199	193	A00167	A09215	154
A00167	A09235	224	A00167	A09236	335
A00167	A09238	180	A00167	A09258	899
A00167	A09377	899	A00167	A09380	277

A00167	A09382	265	A00167	A09416	477
A00167	A09479	252	A00167	A09493	162
A00167	A09509	491	A00167	A09513	398
A00167	A09732	484	A00167	A09733	443
A00167	A09734	267	A00167	A09742	171
A00167	A09791	229	A00167	A09825	249
A00167	A10018	151	A00167	A10156	151
A00167	A10174	899	A00167	A10175	427
A00167	A10228	900	A00167	A10233	899
A00167	A10336	423	A00167	A10340	899
A00167	A10365	899	A00167	A10453	216
A00167	A10532	899	A00167	A10580	899
A00167	A10596	313	A00167	A10597	914
A00167	A10599	297	A00167	A10619	469
A00167	A10645	193	A00167	A10731	467
A00167	A10743	899	A00167	A10833	899
A00167	A11007	152	A00167	A11104	160
A00167	A11287	157	A00167	A11291	177
A00167	A11293	335	A00167	A11300	485
A00167	A11369	294	A00167	A11387	368
A00167	A11405	324	A00167	A11415	346
A00167	A11487	385	A00167	A11513	276
A00167	A11522	432	A00167	A11523	465
A00167	A11531	296	A00167	A11548	185
A00167	A11560	294	A00167	A11684	217
A00167	A11727	190	A00167	A11834	899
A00167	A11846	303	A00167	A11875	224
A00167	A11897	277	A00167	A11899	191
A00167	A11932	435	A00167	A11983	982
A00167	A12111	490	A00167	A12356	154
A00167	A12365	569	A00167	A12371	263
A00167	A12373	490	A00167	A12385	154
A00167	A12393	363	A00167	A12410	154
A00167	A12466	154	A00167	A12474	279
A00167	A12488	154	A00167	A12490	154
A00167	A12507	187	A00167	A12513	316
A00167	A12666	899	A00167	A12699	154
A00167	A12818	899	A00167	A12854	151
A00167	A12897	613	A00167	A12971	899
A00167	A13063	370	A00167	A13241	944
A00167	A13287	152	A00167	A13344	899
A00167	A13587	490	A00167	A13605	899
A00167	A13874	217	A00167	A13889	184
A00167	A13928	477	A00167	A13944	152
A00167	A13975	905	A00167	A13987	196
A00167	A13989	904	A00167	A13994	313

A00167	A13997	247	A00167	A14010	168
A00167	A14017	899	A00167	A14025	511
A00167	A14032	899	A00167	A14033	912
A00167	A14038	913	A00167	A14055	945
A00167	A14120	266	A00167	A14148	490
A00167	A14188	899	A00167	A14190	905
A00167	A14196	531	A00167	A14202	553
A00167	A14208	581	A00167	A14210	490
A00167	A14211	238	A00167	A14220	465
A00167	A14221	473	A00167	A14229	343
A00167	A14236	338	A00167	A14247	277
A00167	A14258	159	A00167	A14299	489
A00167	A14366	916	A00167	A14371	475
A00167	A14378	281	A00167	A14382	191
A00167	A14391	899	A00167	A14399	173
A00167	A14410	612	A00167	A14430	312
A00167	A14442	406	A00167	A14443	994
A00167	A14444	993	A00167	A14457	263
A00167	A14472	380	A00167	A14474	204
A00167	A14498	195	A00167	A14506	899
A00167	A14511	490	A00167	A14557	899
A00167	A14570	484	A00167	A14589	473
A00167	A14604	485	A00167	A14635	491
A00167	A14636	491	A00167	A14643	491
A00167	A14650	408	A00167	A14659	204
A00167	A14660	457	A00167	A14667	473
A00167	A14886	274	A00167	A14925	456
A00167	A14930	157	A00167	A14949	154
A00167	A15002	154	A00167	A15056	154
A00167	A15233	246	A00167	A15237	227
A00167	A15238	310	A00167	A15241	230
A00167	A15277	366	A00167	A15282	491
A00167	A15290	899	A00167	A15292	491
A00167	A15293	949	A00167	A15294	925
A00167	A15296	557	A00167	A15309	163
A00167	A15312	430	A00167	A15313	445
A00167	A15314	491	A00167	A15318	491
A00167	A15322	490	A00167	A15323	430
A00167	A15337	491	A00167	A15339	380
A00167	A15349	479	A00167	A15352	435
A00167	A15355	435	A00167	A15356	945
A00167	A15360	162	A00167	A15361	180
A00167	A15366	490	A00167	A15403	308
A00167	A15431	386	A00167	A15447	291
A00167	A15452	481	A00167	A15478	255
A00167	A15480	229	A00167	A15481	154

A00167	A15484	151	A00167	A15492	167
A00167	A15495	418	A00167	A15506	528
A00167	A15559	358	A00167	A15599	345
A00167	A15605	180	A00167	A15606	584
A00167	A15608	368	A00167	A15630	488
A00167	A15834	269	A00167	A15852	238
A00167	A15868	229	A00167	A15909	408
A00167	A15911	269	A00167	A15917	406
A00167	A16080	935	A00167	A16087	302
A00167	A16257	452	A00167	A16379	326
A00167	A16453	272	A00167	A16511	435
A00167	A16625	154	A00167	A16626	463
A00167	A16635	312	A00167	A16792	165
A00167	A16812	899	A00167	A16814	160
A00167	A16911	356	A00167	A16916	432
A00167	A17063	436	A00167	A17188	899
A00167	A17190	176	A00167	A17198	435
A00167	A17214	899	A00168	A00169	388
A00168	A00176	219	A00168	A00177	434
A00168	A00185	232	A00168	A00190	447
A00168	A00200	899	A00168	A00208	348
A00168	A00209	489	A00168	A00211	350
A00168	A00212	491	A00168	A00216	438
A00168	A00219	632	A00168	A00231	447
A00168	A00245	491	A00168	A00254	486
A00168	A00264	395	A00168	A00294	165
A00168	A00309	303	A00168	A00315	384
A00168	A00316	152	A00168	A00349	260
A00168	A00370	385	A00168	A00451	488
A00168	A00485	369	A00168	A00491	484
A00168	A00561	484	A00168	A00567	401
A00168	A00612	195	A00168	A00616	489
A00168	A00664	163	A00168	A00718	486
A00168	A00736	356	A00168	A00752	168
A00168	A00757	299	A00168	A00815	443
A00168	A00875	235	A00168	A00883	185
A00168	A00921	170	A00168	A00931	491
A00168	A00937	307	A00168	A00940	207
A00168	A00958	258	A00168	A00981	639
A00168	A01070	474	A00168	A01125	785
A00168	A01137	491	A00168	A01161	353
A00168	A01209	305	A00168	A01235	477
A00168	A01244	457	A00168	A01257	491
A00168	A01360	473	A00168	A01454	337
A00168	A01475	488	A00168	A01485	422
A00168	A01512	174	A00168	A01524	417

A00168	A01531	174	A00168	A01546	252
A00168	A01547	475	A00168	A01590	433
A00168	A01600	520	A00168	A01601	319
A00168	A01640	180	A00168	A01647	445
A00168	A01671	470	A00168	A01741	318
A00168	A01775	348	A00168	A01925	210
A00168	A01926	465	A00168	A01929	221
A00168	A01941	380	A00168	A01995	409
A00168	A02000	345	A00168	A02006	172
A00168	A02010	438	A00168	A02063	168
A00168	A02074	323	A00168	A02083	208
A00168	A02111	401	A00168	A02143	201
A00168	A02156	199	A00168	A02161	165
A00168	A02212	458	A00168	A02215	227
A00168	A02228	226	A00168	A02229	461
A00168	A02278	457	A00168	A02304	177
A00168	A02306	461	A00168	A02313	486
A00168	A02317	188	A00168	A02397	420
A00168	A02425	360	A00168	A02452	157
A00168	A02464	428	A00168	A02466	468
A00168	A02482	398	A00168	A02502	374
A00168	A02559	162	A00168	A02572	452
A00168	A02588	173	A00168	A02660	463
A00168	A02673	490	A00168	A02716	165
A00168	A02731	472	A00168	A02759	360
A00168	A02785	179	A00168	A02836	154
A00168	A03051	350	A00168	A03076	212
A00168	A03099	374	A00168	A03104	489
A00168	A03110	176	A00168	A03141	485
A00168	A03165	93	A00168	A03181	163
A00168	A03183	335	A00168	A03186	90
A00168	A03219	157	A00168	A03259	179
A00168	A03292	177	A00168	A03304	446
A00168	A03310	104	A00168	A03314	491
A00168	A03315	456	A00168	A03402	230
A00168	A03436	260	A00168	A03451	193
A00168	A03457	412	A00168	A03459	232
A00168	A03578	63	A00168	A03584	271
A00168	A03639	475	A00168	A03688	308
A00168	A03694	330	A00168	A03723	468
A00168	A03771	243	A00168	A03818	352
A00168	A03862	303	A00168	A03906	329
A00168	A03961	491	A00168	A04012	340
A00168	A04086	252	A00168	A04092	364
A00168	A04101	205	A00168	A04133	185
A00168	A04152	405	A00168	A04162	236

A00168	A04202	477	A00168	A04282	252
A00168	A04365	157	A00168	A04449	475
A00168	A04491	168	A00168	A04492	174
A00168	A04504	157	A00168	A04626	210
A00168	A04749	400	A00168	A04770	240
A00168	A04790	160	A00168	A04813	182
A00168	A04976	347	A00168	A05048	195
A00168	A05087	187	A00168	A05118	183
A00168	A05221	184	A00168	A05228	173
A00168	A05288	445	A00168	A05297	154
A00168	A05356	290	A00168	A05366	195
A00168	A05377	469	A00168	A05439	491
A00168	A05494	268	A00168	A05533	472
A00168	A05534	390	A00168	A05571	232
A00168	A05629	218	A00168	A05653	345
A00168	A05737	195	A00168	A05886	230
A00168	A05887	456	A00168	A05965	195
A00168	A06008	210	A00168	A06038	422
A00168	A06156	195	A00168	A06245	152
A00168	A06253	151	A00168	A06277	328
A00168	A06578	446	A00168	A06607	307
A00168	A06608	899	A00168	A06609	201
A00168	A06610	428	A00168	A06769	468
A00168	A06777	195	A00168	A06815	165
A00168	A07054	310	A00168	A07143	273
A00168	A07209	486	A00168	A07211	267
A00168	A07339	446	A00168	A07454	210
A00168	A07803	268	A00168	A07893	515
A00168	A07934	435	A00168	A08039	183
A00168	A08244	173	A00168	A08358	180
A00168	A08630	240	A00168	A09036	326
A00168	A09072	420	A00168	A09078	215
A00168	A09235	486	A00168	A09236	303
A00168	A09241	455	A00168	A09246	355
A00168	A09272	477	A00168	A09363	290
A00168	A09429	382	A00168	A09480	338
A00168	A09493	173	A00168	A09729	488
A00168	A09812	243	A00168	A09837	256
A00168	A09956	232	A00168	A09970	210
A00168	A09971	183	A00168	A10079	358
A00168	A10470	152	A00168	A10645	252
A00168	A11132	310	A00168	A11237	267
A00168	A11313	457	A00168	A11327	200
A00168	A11614	462	A00168	A11788	210
A00168	A11819	232	A00168	A11893	307
A00168	A11983	486	A00168	A12265	317

A00168	A12298	307	A00168	A12366	232
A00168	A12650	277	A00168	A12654	396
A00168	A12897	466	A00168	A13299	396
A00168	A13344	210	A00168	A13736	196
A00168	A13782	195	A00168	A13811	163
A00168	A13849	173	A00168	A13853	243
A00168	A13994	183	A00168	A14060	433
A00168	A14072	491	A00168	A14128	218
A00168	A14188	382	A00168	A14193	486
A00168	A14196	401	A00168	A14198	486
A00168	A14299	279	A00168	A14399	177
A00168	A14423	160	A00168	A14443	477
A00168	A14444	488	A00168	A14511	447
A00168	A14589	185	A00168	A14596	302
A00168	A14604	212	A00168	A14635	208
A00168	A14667	174	A00168	A14933	267
A00168	A15230	486	A00168	A15282	157
A00168	A15293	491	A00168	A15294	319
A00168	A15321	622	A00168	A15337	263
A00168	A15496	307	A00168	A15498	488
A00168	A15506	490	A00168	A15555	322
A00168	A15598	489	A00168	A15600	183
A00168	A15682	182	A00168	A15913	491
A00168	A15971	165	A00168	A16260	165
A00168	A16261	401	A00168	A16519	210
A00168	A16608	396	A00168	A16916	469
A00169	A00171	484	A00169	A00185	993
A00169	A00190	452	A00169	A00200	994
A00169	A00201	484	A00169	A00207	408
A00169	A00208	301	A00169	A00209	936
A00169	A00213	279	A00169	A00214	491
A00169	A00216	900	A00169	A00219	303
A00169	A00224	490	A00169	A00229	180
A00169	A00230	931	A00169	A00234	211
A00169	A00239	467	A00169	A00240	491
A00169	A00245	252	A00169	A00246	930
A00169	A00254	935	A00169	A00255	899
A00169	A00263	395	A00169	A00294	919
A00169	A00308	406	A00169	A00309	159
A00169	A00315	154	A00169	A00316	313
A00169	A00324	491	A00169	A00366	307
A00169	A00381	474	A00169	A00411	485
A00169	A00440	539	A00169	A00469	204
A00169	A00486	196	A00169	A00638	486
A00169	A00643	512	A00169	A00669	757
A00169	A00696	489	A00169	A00832	420

A00169	A00864	480	A00169	A00875	452
A00169	A00887	469	A00169	A00905	321
A00169	A00911	405	A00169	A00958	383
A00169	A00981	177	A00169	A00997	491
A00169	A01003	491	A00169	A01011	162
A00169	A01052	480	A00169	A01070	899
A00169	A01078	324	A00169	A01157	534
A00169	A01161	235	A00169	A01172	257
A00169	A01214	395	A00169	A01289	469
A00169	A01307	399	A00169	A01322	490
A00169	A01325	454	A00169	A01450	171
A00169	A01512	301	A00169	A01524	938
A00169	A01527	945	A00169	A01529	368
A00169	A01548	217	A00169	A01553	363
A00169	A01554	937	A00169	A01558	899
A00169	A01560	942	A00169	A01561	389
A00169	A01571	339	A00169	A01588	899
A00169	A01602	230	A00169	A01615	903
A00169	A01629	419	A00169	A01630	433
A00169	A01636	899	A00169	A01639	218
A00169	A01650	378	A00169	A01668	322
A00169	A01688	445	A00169	A01692	223
A00169	A01697	335	A00169	A01709	402
A00169	A01730	488	A00169	A01747	491
A00169	A01796	218	A00169	A01822	249
A00169	A01839	347	A00169	A01842	154
A00169	A01897	249	A00169	A01920	899
A00169	A02211	165	A00169	A02217	182
A00169	A02221	330	A00169	A02493	306
A00169	A02494	378	A00169	A02502	488
A00169	A02504	475	A00169	A02598	260
A00169	A02618	303	A00169	A02639	236
A00169	A02640	488	A00169	A02673	247
A00169	A02687	165	A00169	A02711	198
A00169	A02738	296	A00169	A02748	191
A00169	A02749	469	A00169	A02752	176
A00169	A02788	174	A00169	A02826	188
A00169	A02840	174	A00169	A02877	729
A00169	A02895	210	A00169	A03056	435
A00169	A03188	419	A00169	A03203	241
A00169	A03216	182	A00169	A03226	483
A00169	A03244	205	A00169	A03292	270
A00169	A03304	380	A00169	A03321	224
A00169	A03401	152	A00169	A03435	366
A00169	A03436	460	A00169	A03451	491
A00169	A03477	184	A00169	A03591	366

A00169	A03611	486	A00169	A03758	321
A00169	A03771	173	A00169	A03854	488
A00169	A03904	165	A00169	A04006	184
A00169	A04038	254	A00169	A04050	491
A00169	A04062	256	A00169	A04133	196
A00169	A04140	414	A00169	A04222	217
A00169	A04239	327	A00169	A04266	174
A00169	A04280	165	A00169	A04282	491
A00169	A04349	177	A00169	A04358	160
A00169	A04359	191	A00169	A04360	281
A00169	A04413	174	A00169	A04414	274
A00169	A04449	340	A00169	A04484	899
A00169	A04486	154	A00169	A04513	241
A00169	A04534	165	A00169	A04569	191
A00169	A04680	174	A00169	A04699	305
A00169	A04704	245	A00169	A04719	191
A00169	A04807	477	A00169	A04841	218
A00169	A04853	156	A00169	A04949	534
A00169	A04973	174	A00169	A05082	450
A00169	A05247	171	A00169	A05288	491
A00169	A05415	443	A00169	A05418	633
A00169	A05432	180	A00169	A05453	167
A00169	A05519	218	A00169	A05680	191
A00169	A05701	491	A00169	A05721	351
A00169	A05722	324	A00169	A05763	218
A00169	A05990	401	A00169	A06147	174
A00169	A06161	467	A00169	A06212	168
A00169	A06268	343	A00169	A06295	899
A00169	A06400	322	A00169	A06554	485
A00169	A06597	224	A00169	A06599	452
A00169	A06608	945	A00169	A06610	363
A00169	A06623	329	A00169	A06648	151
A00169	A06660	160	A00169	A06701	208
A00169	A06728	182	A00169	A06752	398
A00169	A06767	204	A00169	A07019	251
A00169	A07158	346	A00169	A07163	407
A00169	A07165	388	A00169	A07206	327
A00169	A07260	274	A00169	A07311	949
A00169	A07339	491	A00169	A07668	241
A00169	A07706	216	A00169	A07782	218
A00169	A07908	241	A00169	A08047	274
A00169	A08175	174	A00169	A08236	160
A00169	A08310	174	A00169	A08342	218
A00169	A08720	191	A00169	A08747	191
A00169	A08946	154	A00169	A09018	272
A00169	A09078	238	A00169	A09123	468

A00169	A09124	467	A00169	A09149	477
A00169	A09319	277	A00169	A09361	409
A00169	A09380	213	A00169	A09464	165
A00169	A09479	174	A00169	A09500	165
A00169	A09577	193	A00169	A09746	160
A00169	A09964	160	A00169	A09982	241
A00169	A10072	165	A00169	A10111	165
A00169	A10203	241	A00169	A10247	395
A00169	A10252	364	A00169	A10278	401
A00169	A10364	154	A00169	A10525	454
A00169	A10743	167	A00169	A10808	903
A00169	A10941	160	A00169	A10982	380
A00169	A10986	174	A00169	A11014	208
A00169	A11125	310	A00169	A11252	204
A00169	A11262	335	A00169	A11264	249
A00169	A11300	360	A00169	A11342	165
A00169	A11483	160	A00169	A11513	176
A00169	A11586	314	A00169	A11619	274
A00169	A11788	218	A00169	A11977	204
A00169	A12010	477	A00169	A12108	182
A00169	A12365	488	A00169	A12373	446
A00169	A12432	218	A00169	A12434	241
A00169	A12435	218	A00169	A12518	182
A00169	A12535	241	A00169	A12638	174
A00169	A12656	218	A00169	A12657	154
A00169	A12743	274	A00169	A12778	274
A00169	A12830	274	A00169	A12860	274
A00169	A12965	218	A00169	A13138	274
A00169	A13234	191	A00169	A13795	174
A00169	A13862	174	A00169	A13997	294
A00169	A14006	323	A00169	A14045	899
A00169	A14198	930	A00169	A14319	443
A00169	A14321	314	A00169	A14393	470
A00169	A14410	425	A00169	A14596	899
A00169	A14605	340	A00169	A14642	417
A00169	A14650	462	A00169	A14660	278
A00169	A14689	165	A00169	A14732	204
A00169	A14834	274	A00169	A15293	905
A00169	A15294	899	A00169	A15325	201
A00169	A15606	279	A00169	A15651	267
A00169	A15838	266	A00169	A15840	429
A00169	A15952	241	A00169	A16043	435
A00169	A16138	260	A00169	A16150	241
A00169	A16171	218	A00169	A16174	204
A00169	A16195	204	A00169	A16339	241
A00169	A16397	279	A00169	A16442	218

A00169	A16489	204	A00169	A16718	383
A00169	A16721	395	A00169	A16732	266
A00169	A16775	241	A00169	A16887	307
A00169	A17096	274	A00169	A17188	899
A00170	A00229	984	A00170	A00240	953
A00170	A00272	991	A00170	A00653	372
A00170	A02473	362	A00170	A02836	739
A00170	A07153	184	A00170	A07311	547
A00170	A07422	899	A00170	A14062	974
A00170	A14191	201	A00171	A00173	491
A00171	A00185	393	A00171	A00190	480
A00171	A00196	188	A00171	A00200	238
A00171	A00208	932	A00171	A00209	952
A00171	A00212	293	A00171	A00214	899
A00171	A00219	491	A00171	A00224	491
A00171	A00230	296	A00171	A00234	941
A00171	A00235	463	A00171	A00238	475
A00171	A00239	157	A00171	A00240	462
A00171	A00242	911	A00171	A00246	899
A00171	A00258	252	A00171	A00262	899
A00171	A00294	483	A00171	A00301	154
A00171	A00305	491	A00171	A00309	484
A00171	A00316	899	A00171	A00324	944
A00171	A00338	399	A00171	A00366	443
A00171	A00417	439	A00171	A00421	490
A00171	A00422	183	A00171	A00431	152
A00171	A00446	156	A00171	A00447	286
A00171	A00457	162	A00171	A00470	162
A00171	A00485	470	A00171	A00493	491
A00171	A00531	445	A00171	A00562	290
A00171	A00581	262	A00171	A00589	241
A00171	A00604	452	A00171	A00605	486
A00171	A00609	491	A00171	A00616	188
A00171	A00617	475	A00171	A00669	491
A00171	A00680	218	A00171	A00697	313
A00171	A00718	491	A00171	A00736	422
A00171	A00747	430	A00171	A00778	480
A00171	A00779	485	A00171	A00797	474
A00171	A00832	485	A00171	A00851	154
A00171	A00853	188	A00171	A00868	254
A00171	A00883	491	A00171	A00887	369
A00171	A00905	419	A00171	A00907	488
A00171	A00914	907	A00171	A00937	174
A00171	A00942	286	A00171	A00954	480
A00171	A00997	491	A00171	A01021	490
A00171	A01022	445	A00171	A01026	491

A00171	A01065	450	A00171	A01091	438
A00171	A01100	393	A00171	A01120	307
A00171	A01136	436	A00171	A01161	467
A00171	A01189	467	A00171	A01211	346
A00171	A01243	288	A00171	A01269	229
A00171	A01313	486	A00171	A01325	235
A00171	A01356	163	A00171	A01359	199
A00171	A01375	466	A00171	A01377	491
A00171	A01384	322	A00171	A01386	375
A00171	A01472	193	A00171	A01483	491
A00171	A01527	937	A00171	A01529	154
A00171	A01554	899	A00171	A01560	472
A00171	A01582	362	A00171	A01591	172
A00171	A01592	176	A00171	A01593	899
A00171	A01601	276	A00171	A01612	486
A00171	A01615	937	A00171	A01626	899
A00171	A01632	389	A00171	A01634	254
A00171	A01649	224	A00171	A01666	393
A00171	A01675	319	A00171	A01676	391
A00171	A01688	335	A00171	A01694	277
A00171	A01702	945	A00171	A01718	901
A00171	A01730	430	A00171	A01741	488
A00171	A01747	416	A00171	A01748	274
A00171	A01767	151	A00171	A01789	899
A00171	A01797	317	A00171	A01822	346
A00171	A01839	286	A00171	A01844	945
A00171	A01900	258	A00171	A02049	160
A00171	A02180	468	A00171	A02212	199
A00171	A02215	257	A00171	A02278	361
A00171	A02292	200	A00171	A02293	157
A00171	A02333	299	A00171	A02352	490
A00171	A02354	180	A00171	A02361	257
A00171	A02411	915	A00171	A02477	263
A00171	A02494	385	A00171	A02563	286
A00171	A02640	312	A00171	A02675	241
A00171	A02704	353	A00171	A02711	299
A00171	A02749	273	A00171	A02763	185
A00171	A02784	436	A00171	A02789	224
A00171	A02797	210	A00171	A02816	339
A00171	A02836	491	A00171	A02837	213
A00171	A02864	899	A00171	A02872	899
A00171	A02877	466	A00171	A02878	457
A00171	A02908	899	A00171	A02942	899
A00171	A02968	899	A00171	A03014	462
A00171	A03095	324	A00171	A03101	221
A00171	A03102	245	A00171	A03112	391

A00171	A03181	191	A00171	A03211	358
A00171	A03213	490	A00171	A03214	913
A00171	A03232	152	A00171	A03258	154
A00171	A03273	481	A00171	A03321	313
A00171	A03406	488	A00171	A03412	350
A00171	A03415	443	A00171	A03420	491
A00171	A03421	335	A00171	A03422	479
A00171	A03423	63	A00171	A03446	303
A00171	A03451	283	A00171	A03494	291
A00171	A03515	188	A00171	A03566	285
A00171	A03569	414	A00171	A03591	439
A00171	A03611	480	A00171	A03725	485
A00171	A03736	218	A00171	A03771	260
A00171	A03778	152	A00171	A03854	373
A00171	A03860	318	A00171	A03922	454
A00171	A03977	168	A00171	A04006	196
A00171	A04050	899	A00171	A04080	162
A00171	A04145	461	A00171	A04160	414
A00171	A04175	484	A00171	A04249	307
A00171	A04288	396	A00171	A04310	228
A00171	A04337	402	A00171	A04429	288
A00171	A04507	202	A00171	A04622	451
A00171	A04629	439	A00171	A04645	154
A00171	A04681	469	A00171	A04737	491
A00171	A04743	386	A00171	A04879	428
A00171	A04977	375	A00171	A05015	303
A00171	A05052	386	A00171	A05122	339
A00171	A05141	162	A00171	A05155	343
A00171	A05182	170	A00171	A05216	324
A00171	A05226	180	A00171	A05240	241
A00171	A05307	256	A00171	A05315	899
A00171	A05353	477	A00171	A05443	899
A00171	A05450	278	A00171	A05474	374
A00171	A05504	274	A00171	A05525	235
A00171	A05568	247	A00171	A05621	199
A00171	A05701	398	A00171	A05767	162
A00171	A05873	180	A00171	A05891	420
A00171	A05939	165	A00171	A06045	240
A00171	A06189	274	A00171	A06268	384
A00171	A06379	486	A00171	A06458	240
A00171	A06472	157	A00171	A06478	180
A00171	A06581	440	A00171	A06590	202
A00171	A06606	418	A00171	A06609	235
A00171	A06615	479	A00171	A06752	452
A00171	A06814	515	A00171	A06819	201
A00171	A06924	411	A00171	A06949	434

A00171	A07091	899	A00171	A07111	221
A00171	A07153	185	A00171	A07163	307
A00171	A07165	314	A00171	A07209	177
A00171	A07232	429	A00171	A07311	176
A00171	A07339	491	A00171	A07558	474
A00171	A07575	454	A00171	A07583	249
A00171	A07621	218	A00171	A07693	267
A00171	A07848	274	A00171	A07973	208
A00171	A08051	180	A00171	A08101	240
A00171	A08113	473	A00171	A08171	388
A00171	A08172	483	A00171	A08222	468
A00171	A08263	474	A00171	A08281	483
A00171	A08299	240	A00171	A08319	408
A00171	A08327	356	A00171	A08346	285
A00171	A08357	472	A00171	A08402	483
A00171	A08416	313	A00171	A08453	299
A00171	A08460	240	A00171	A08484	446
A00171	A08506	160	A00171	A08571	452
A00171	A08615	154	A00171	A08694	154
A00171	A08732	899	A00171	A08960	475
A00171	A08975	188	A00171	A09019	202
A00171	A09028	286	A00171	A09118	202
A00171	A09124	172	A00171	A09142	240
A00171	A09170	202	A00171	A09242	380
A00171	A09265	441	A00171	A09271	154
A00171	A09292	228	A00171	A09298	296
A00171	A09327	188	A00171	A09343	456
A00171	A09387	191	A00171	A09423	343
A00171	A09431	427	A00171	A09448	174
A00171	A09449	547	A00171	A09490	274
A00171	A09525	202	A00171	A09537	274
A00171	A09556	458	A00171	A09644	899
A00171	A09707	247	A00171	A09727	899
A00171	A09816	265	A00171	A09876	481
A00171	A09934	314	A00171	A09995	179
A00171	A10015	443	A00171	A10078	291
A00171	A10123	202	A00171	A10148	424
A00171	A10156	486	A00171	A10160	335
A00171	A10174	907	A00171	A10408	165
A00171	A10496	899	A00171	A10731	218
A00171	A10801	899	A00171	A10894	177
A00171	A10955	240	A00171	A10996	202
A00171	A11082	240	A00171	A11284	470
A00171	A11382	256	A00171	A11465	180
A00171	A11712	180	A00171	A11942	257
A00171	A12020	423	A00171	A12045	154

A00171	A12046	154	A00171	A12070	154
A00171	A12089	240	A00171	A12178	240
A00171	A12179	367	A00171	A12371	154
A00171	A12373	483	A00171	A12413	202
A00171	A12632	240	A00171	A12634	154
A00171	A12685	240	A00171	A12702	240
A00171	A12759	202	A00171	A12762	335
A00171	A12807	202	A00171	A12858	254
A00171	A12879	240	A00171	A12897	477
A00171	A12955	202	A00171	A13120	202
A00171	A13172	165	A00171	A13369	281
A00171	A13468	180	A00171	A13587	574
A00171	A13623	165	A00171	A13725	202
A00171	A13737	899	A00171	A13824	899
A00171	A13873	457	A00171	A13968	307
A00171	A13981	899	A00171	A13991	899
A00171	A14004	165	A00171	A14039	903
A00171	A14077	423	A00171	A14092	424
A00171	A14191	489	A00171	A14196	491
A00171	A14202	899	A00171	A14220	187
A00171	A14272	491	A00171	A14381	227
A00171	A14443	899	A00171	A14444	899
A00171	A14459	240	A00171	A14484	260
A00171	A14589	374	A00171	A14597	446
A00171	A14636	362	A00171	A14643	419
A00171	A14649	251	A00171	A14664	174
A00171	A14701	488	A00171	A14707	477
A00171	A14840	329	A00171	A14925	436
A00171	A14949	240	A00171	A15256	307
A00171	A15269	154	A00171	A15292	899
A00171	A15296	224	A00171	A15297	432
A00171	A15305	165	A00171	A15338	232
A00171	A15367	489	A00171	A15452	899
A00171	A15490	202	A00171	A15559	485
A00171	A15564	472	A00171	A15638	245
A00171	A15678	165	A00171	A15726	480
A00171	A15877	481	A00171	A16300	422
A00171	A16311	374	A00171	A16325	235
A00171	A16418	480	A00171	A16423	274
A00171	A16816	335	A00171	A16819	899
A00171	A16915	456	A00172	A00207	924
A00172	A00209	899	A00172	A00224	899
A00172	A00227	899	A00172	A00230	899
A00172	A00247	944	A00172	A00254	915
A00172	A01524	916	A00172	A01527	935
A00172	A01560	943	A00172	A07311	899

A00172	A10365	363	A00172	A13605	929
A00172	A14188	899	A00172	A14198	924
A00173	A00174	468	A00173	A00175	486
A00173	A00185	477	A00173	A00190	489
A00173	A00191	491	A00173	A00192	491
A00173	A00196	491	A00173	A00209	989
A00173	A00219	491	A00173	A00221	190
A00173	A00223	462	A00173	A00234	479
A00173	A00239	234	A00173	A00240	491
A00173	A00246	932	A00173	A00248	328
A00173	A00254	329	A00173	A00262	215
A00173	A00264	460	A00173	A00267	247
A00173	A00268	477	A00173	A00280	469
A00173	A00325	374	A00173	A00334	160
A00173	A00355	481	A00173	A00356	491
A00173	A00357	477	A00173	A00358	491
A00173	A00360	479	A00173	A00361	488
A00173	A00375	329	A00173	A00403	313
A00173	A00469	398	A00173	A00484	265
A00173	A00499	151	A00173	A00616	490
A00173	A00632	382	A00173	A00656	302
A00173	A00675	489	A00173	A00719	367
A00173	A00736	408	A00173	A00751	293
A00173	A00814	490	A00173	A00832	439
A00173	A00882	491	A00173	A00900	491
A00173	A00923	450	A00173	A00930	310
A00173	A00938	221	A00173	A00990	332
A00173	A01000	456	A00173	A01009	313
A00173	A01047	399	A00173	A01064	226
A00173	A01101	321	A00173	A01127	185
A00173	A01140	385	A00173	A01184	396
A00173	A01189	477	A00173	A01240	458
A00173	A01249	312	A00173	A01291	491
A00173	A01325	340	A00173	A01338	461
A00173	A01425	204	A00173	A01442	443
A00173	A01464	368	A00173	A01467	188
A00173	A01468	424	A00173	A01469	460
A00173	A01473	488	A00173	A01483	491
A00173	A01524	899	A00173	A01527	899
A00173	A01531	265	A00173	A01536	204
A00173	A01553	313	A00173	A01554	945
A00173	A01568	477	A00173	A01572	467
A00173	A01589	491	A00173	A01594	337
A00173	A01604	588	A00173	A01605	301
A00173	A01617	260	A00173	A01618	483
A00173	A01624	353	A00173	A01649	245

A00173	A01659	455	A00173	A01661	468
A00173	A01669	193	A00173	A01686	489
A00173	A01692	460	A00173	A01709	195
A00173	A01718	960	A00173	A01729	167
A00173	A01740	454	A00173	A01741	179
A00173	A01747	377	A00173	A01797	272
A00173	A01870	469	A00173	A01897	408
A00173	A01908	424	A00173	A01983	491
A00173	A01986	160	A00173	A02006	366
A00173	A02049	491	A00173	A02054	171
A00173	A02068	457	A00173	A02128	246
A00173	A02178	393	A00173	A02180	491
A00173	A02183	273	A00173	A02184	441
A00173	A02185	491	A00173	A02213	180
A00173	A02278	294	A00173	A02291	469
A00173	A02292	276	A00173	A02293	489
A00173	A02352	472	A00173	A02393	188
A00173	A02473	446	A00173	A02501	152
A00173	A02505	290	A00173	A02542	215
A00173	A02668	174	A00173	A02675	469
A00173	A02788	218	A00173	A02803	333
A00173	A02836	491	A00173	A02858	201
A00173	A02874	255	A00173	A02877	486
A00173	A02878	182	A00173	A02935	224
A00173	A02978	210	A00173	A02983	180
A00173	A02988	191	A00173	A03048	210
A00173	A03069	328	A00173	A03070	488
A00173	A03095	193	A00173	A03102	154
A00173	A03126	377	A00173	A03171	484
A00173	A03177	236	A00173	A03190	188
A00173	A03209	396	A00173	A03228	380
A00173	A03269	162	A00173	A03271	252
A00173	A03331	489	A00173	A03332	484
A00173	A03356	63	A00173	A03390	68
A00173	A03391	63	A00173	A03395	160
A00173	A03401	63	A00173	A03406	483
A00173	A03407	466	A00173	A03408	491
A00173	A03411	380	A00173	A03412	311
A00173	A03413	274	A00173	A03434	302
A00173	A03437	216	A00173	A03451	372
A00173	A03531	486	A00173	A03567	473
A00173	A03651	296	A00173	A03661	228
A00173	A03705	270	A00173	A03753	491
A00173	A03754	457	A00173	A03773	176
A00173	A03775	491	A00173	A03813	335
A00173	A03817	440	A00173	A03888	428

A00173	A03913	489	A00173	A03922	191
A00173	A04007	216	A00173	A04020	446
A00173	A04034	366	A00173	A04117	328
A00173	A04158	151	A00173	A04175	196
A00173	A04234	151	A00173	A04276	151
A00173	A04280	353	A00173	A04363	160
A00173	A04367	151	A00173	A04415	152
A00173	A04536	369	A00173	A04706	425
A00173	A04754	399	A00173	A04761	491
A00173	A04792	151	A00173	A04795	157
A00173	A04886	160	A00173	A04977	177
A00173	A04993	273	A00173	A05004	484
A00173	A05018	188	A00173	A05044	480
A00173	A05052	353	A00173	A05174	160
A00173	A05195	340	A00173	A05252	160
A00173	A05288	469	A00173	A05301	469
A00173	A05411	411	A00173	A05414	899
A00173	A05427	366	A00173	A05443	491
A00173	A05723	160	A00173	A05996	483
A00173	A06055	254	A00173	A06113	188
A00173	A06242	491	A00173	A06371	191
A00173	A06409	173	A00173	A06591	372
A00173	A06601	273	A00173	A06606	408
A00173	A06615	299	A00173	A06750	321
A00173	A06793	193	A00173	A06864	455
A00173	A06870	246	A00173	A06943	372
A00173	A06965	439	A00173	A07025	173
A00173	A07068	210	A00173	A07079	259
A00173	A07098	246	A00173	A07143	457
A00173	A07152	260	A00173	A07163	424
A00173	A07165	260	A00173	A07173	163
A00173	A07201	218	A00173	A07238	288
A00173	A07305	173	A00173	A07319	490
A00173	A07421	247	A00173	A07466	461
A00173	A07480	475	A00173	A07534	469
A00173	A07582	160	A00173	A07603	481
A00173	A07647	241	A00173	A07902	477
A00173	A07989	188	A00173	A07996	486
A00173	A08000	247	A00173	A08024	307
A00173	A08030	486	A00173	A08117	486
A00173	A08172	188	A00173	A08194	491
A00173	A08208	307	A00173	A08402	346
A00173	A08452	260	A00173	A08458	414
A00173	A08572	151	A00173	A08580	188
A00173	A08624	188	A00173	A08669	424
A00173	A08753	174	A00173	A08810	232

A00173	A08835	490	A00173	A08849	188
A00173	A08921	249	A00173	A08929	398
A00173	A08949	240	A00173	A09032	246
A00173	A09095	188	A00173	A09124	463
A00173	A09322	274	A00173	A09409	173
A00173	A09469	477	A00173	A09499	151
A00173	A09601	151	A00173	A09661	188
A00173	A09704	160	A00173	A09758	396
A00173	A09790	165	A00173	A09802	246
A00173	A09808	188	A00173	A09809	486
A00173	A09840	332	A00173	A09865	210
A00173	A09895	246	A00173	A09968	188
A00173	A10080	173	A00173	A10088	210
A00173	A10297	246	A00173	A10314	193
A00173	A10333	246	A00173	A10529	243
A00173	A10616	168	A00173	A10743	369
A00173	A10887	130	A00173	A10949	414
A00173	A11087	246	A00173	A11195	353
A00173	A11323	479	A00173	A11324	477
A00173	A11400	470	A00173	A11480	318
A00173	A11522	479	A00173	A11612	447
A00173	A11699	201	A00173	A11704	344
A00173	A11784	170	A00173	A11791	347
A00173	A11943	488	A00173	A12197	173
A00173	A12233	159	A00173	A12241	152
A00173	A12493	188	A00173	A12540	173
A00173	A12592	321	A00173	A12651	422
A00173	A12653	443	A00173	A12729	188
A00173	A12738	266	A00173	A12822	173
A00173	A12831	172	A00173	A12897	256
A00173	A12990	699	A00173	A13166	188
A00173	A13207	491	A00173	A13241	434
A00173	A13460	491	A00173	A13561	173
A00173	A13663	173	A00173	A13716	177
A00173	A13930	929	A00173	A13945	490
A00173	A13975	899	A00173	A13986	481
A00173	A13992	229	A00173	A13998	246
A00173	A14007	490	A00173	A14060	321
A00173	A14079	491	A00173	A14149	347
A00173	A14151	259	A00173	A14273	490
A00173	A14366	219	A00173	A14372	162
A00173	A14374	479	A00173	A14392	193
A00173	A14417	273	A00173	A14452	458
A00173	A14460	467	A00173	A14598	259
A00173	A14642	157	A00173	A14643	268
A00173	A14721	221	A00173	A14737	210

A00173	A14755	210	A00173	A14827	218
A00173	A14893	469	A00173	A14927	446
A00173	A14930	162	A00173	A14932	472
A00173	A14941	347	A00173	A15237	558
A00173	A15245	210	A00173	A15251	188
A00173	A15274	167	A00173	A15369	296
A00173	A15394	393	A00173	A15395	361
A00173	A15416	465	A00173	A15418	389
A00173	A15420	491	A00173	A15427	196
A00173	A15496	151	A00173	A15516	241
A00173	A15607	167	A00173	A15638	452
A00173	A15641	429	A00173	A15726	469
A00173	A15809	731	A00173	A15894	302
A00173	A15922	488	A00173	A15964	454
A00173	A16030	488	A00173	A16040	173
A00173	A16042	398	A00173	A16073	414
A00173	A16127	469	A00173	A16128	435
A00173	A16129	440	A00173	A16159	151
A00173	A16218	317	A00173	A16225	174
A00173	A16261	251	A00173	A16290	210
A00173	A16300	283	A00173	A16317	188
A00173	A16331	165	A00173	A16598	467
A00173	A16606	371	A00173	A16613	446
A00173	A16679	217	A00173	A16754	417
A00173	A16878	188	A00173	A16928	210
A00173	A16930	151	A00173	A16960	396
A00173	A16997	210	A00173	A17059	297
A00173	A17107	162	A00173	A17136	489
A00173	A17145	306	A00173	A17195	213
A00173	A17198	195	A00174	A00175	945
A00174	A00190	520	A00174	A00204	532
A00174	A00207	185	A00174	A00209	899
A00174	A00220	681	A00174	A00246	396
A00174	A00255	202	A00174	A00268	944
A00174	A00312	198	A00174	A00356	218
A00174	A00694	347	A00174	A00802	217
A00174	A00882	290	A00174	A01011	470
A00174	A01140	424	A00174	A01291	163
A00174	A01524	899	A00174	A01527	905
A00174	A01605	217	A00174	A01640	901
A00174	A01718	944	A00174	A01983	168
A00174	A02185	163	A00174	A02247	348
A00174	A02826	230	A00174	A03331	330
A00174	A03332	224	A00174	A03753	470
A00174	A03775	198	A00174	A03822	177
A00174	A03913	945	A00174	A04398	213

A00174	A05424	185	A00174	A05438	307
A00174	A06324	318	A00174	A07157	485
A00174	A07311	899	A00174	A08280	477
A00174	A09469	394	A00174	A10214	532
A00174	A10233	283	A00174	A11149	228
A00174	A11300	532	A00174	A11983	428
A00174	A13860	523	A00174	A13945	422
A00174	A14231	515	A00174	A15323	401
A00174	A16030	310	A00175	A00192	249
A00175	A00205	303	A00175	A00207	319
A00175	A00209	899	A00175	A00211	454
A00175	A00239	174	A00175	A00240	379
A00175	A00241	316	A00175	A00246	932
A00175	A00261	212	A00175	A00268	306
A00175	A00283	168	A00175	A00356	369
A00175	A00400	334	A00175	A00900	224
A00175	A00930	303	A00175	A00932	430
A00175	A00967	334	A00175	A01151	334
A00175	A01527	899	A00175	A01553	899
A00175	A01605	270	A00175	A01640	906
A00175	A01718	945	A00175	A01926	334
A00175	A01983	356	A00175	A02110	183
A00175	A02481	334	A00175	A03126	489
A00175	A03331	428	A00175	A03470	260
A00175	A03753	345	A00175	A03873	334
A00175	A03913	944	A00175	A04347	265
A00175	A04398	435	A00175	A04415	263
A00175	A04449	334	A00175	A04878	268
A00175	A05996	226	A00175	A07311	899
A00175	A08850	334	A00175	A09469	251
A00175	A11559	334	A00175	A12373	334
A00175	A12500	224	A00175	A13716	390
A00175	A13945	335	A00175	A13992	235
A00175	A14210	334	A00175	A14371	334
A00175	A14636	334	A00175	A16030	279
A00176	A00181	768	A00176	A00250	328
A00176	A00371	278	A00176	A00415	491
A00176	A00418	278	A00176	A00471	250
A00176	A00534	486	A00176	A00597	491
A00176	A00730	391	A00176	A00885	294
A00176	A00886	491	A00176	A00889	491
A00176	A00890	485	A00176	A00891	488
A00176	A00892	491	A00176	A00893	485
A00176	A00894	488	A00176	A00895	473
A00176	A00896	299	A00176	A00897	475
A00176	A00898	491	A00176	A00899	182

A00176	A00914	223	A00176	A00999	347
A00176	A01169	204	A00176	A01212	191
A00176	A01236	229	A00176	A01329	340
A00176	A01494	864	A00176	A01524	899
A00176	A01537	430	A00176	A01542	732
A00176	A01596	393	A00176	A01605	903
A00176	A01606	191	A00176	A01668	457
A00176	A01729	475	A00176	A01745	232
A00176	A01766	468	A00176	A01797	318
A00176	A01830	339	A00176	A01888	278
A00176	A01903	305	A00176	A02006	484
A00176	A02086	491	A00176	A02105	232
A00176	A02175	417	A00176	A02358	434
A00176	A02627	193	A00176	A03051	438
A00176	A03151	479	A00176	A03205	180
A00176	A03300	179	A00176	A03339	215
A00176	A03408	460	A00176	A03551	193
A00176	A03569	333	A00176	A03809	204
A00176	A03834	172	A00176	A03854	383
A00176	A04117	291	A00176	A04260	480
A00176	A04334	185	A00176	A04575	154
A00176	A04698	435	A00176	A05216	172
A00176	A05386	191	A00176	A05449	219
A00176	A05526	193	A00176	A05623	243
A00176	A05859	481	A00176	A06368	443
A00176	A06492	173	A00176	A06581	825
A00176	A06588	340	A00176	A06599	479
A00176	A06606	491	A00176	A06607	491
A00176	A06621	490	A00176	A06673	441
A00176	A06688	353	A00176	A06737	154
A00176	A06992	154	A00176	A07111	491
A00176	A07314	202	A00176	A07339	188
A00176	A07446	257	A00176	A07768	488
A00176	A07791	171	A00176	A07815	396
A00176	A07844	204	A00176	A07874	154
A00176	A08137	157	A00176	A08435	406
A00176	A08572	355	A00176	A08891	491
A00176	A08903	326	A00176	A08907	154
A00176	A09008	424	A00176	A09073	217
A00176	A09124	449	A00176	A09149	168
A00176	A09209	243	A00176	A09882	411
A00176	A09923	154	A00176	A09930	154
A00176	A09986	193	A00176	A10087	486
A00176	A10466	491	A00176	A10731	314
A00176	A10810	443	A00176	A10964	154
A00176	A11288	457	A00176	A11440	288

A00176	A11475	208	A00176	A11692	347
A00176	A11735	183	A00176	A11759	440
A00176	A11931	243	A00176	A12891	218
A00176	A13017	324	A00176	A13365	286
A00176	A13671	193	A00176	A13706	489
A00176	A14025	978	A00176	A14127	204
A00176	A14148	243	A00176	A14174	491
A00176	A14197	286	A00176	A14204	491
A00176	A14286	154	A00176	A14299	288
A00176	A14449	204	A00176	A14475	745
A00176	A14485	486	A00176	A14496	278
A00176	A14569	191	A00176	A14585	490
A00176	A14589	491	A00176	A14638	322
A00176	A14643	620	A00176	A14674	179
A00176	A14792	294	A00176	A15189	167
A00176	A15578	193	A00176	A15626	199
A00176	A15726	302	A00176	A16400	219
A00176	A16547	278	A00176	A16664	191
A00176	A16977	154	A00176	A17191	193
A00177	A00178	401	A00177	A00203	105
A00177	A00211	146	A00177	A00217	63
A00177	A00219	483	A00177	A00223	196
A00177	A00240	479	A00177	A00243	241
A00177	A00245	435	A00177	A00258	543
A00177	A00270	168	A00177	A00279	378
A00177	A00288	374	A00177	A00304	475
A00177	A00334	491	A00177	A00335	489
A00177	A00356	610	A00177	A00404	152
A00177	A00414	291	A00177	A00467	468
A00177	A00531	180	A00177	A00550	232
A00177	A00605	454	A00177	A00634	224
A00177	A00648	357	A00177	A00651	414
A00177	A00700	491	A00177	A00742	279
A00177	A00812	488	A00177	A00839	490
A00177	A00878	157	A00177	A00930	224
A00177	A00931	408	A00177	A00970	185
A00177	A00972	201	A00177	A01056	340
A00177	A01072	314	A00177	A01090	168
A00177	A01098	249	A00177	A01112	660
A00177	A01120	154	A00177	A01127	409
A00177	A01128	152	A00177	A01140	489
A00177	A01196	205	A00177	A01257	176
A00177	A01291	599	A00177	A01306	232
A00177	A01320	260	A00177	A01415	366
A00177	A01425	272	A00177	A01457	195
A00177	A01468	490	A00177	A01478	185

A00177	A01492	636	A00177	A01496	378
A00177	A01531	219	A00177	A01579	491
A00177	A01580	302	A00177	A01586	163
A00177	A01601	861	A00177	A01605	486
A00177	A01640	416	A00177	A01649	363
A00177	A01684	180	A00177	A01718	362
A00177	A02179	322	A00177	A02213	163
A00177	A02318	160	A00177	A02350	489
A00177	A02397	435	A00177	A02405	188
A00177	A03083	188	A00177	A03219	352
A00177	A03304	348	A00177	A03314	257
A00177	A03332	174	A00177	A03445	347
A00177	A03639	424	A00177	A03787	236
A00177	A03961	260	A00177	A04099	457
A00177	A04202	395	A00177	A04321	254
A00177	A04580	278	A00177	A04689	236
A00177	A04856	485	A00177	A05356	254
A00177	A05359	302	A00177	A05443	355
A00177	A05906	324	A00177	A06371	456
A00177	A06814	255	A00177	A07398	473
A00177	A07696	344	A00177	A07726	409
A00177	A08357	286	A00177	A08359	469
A00177	A08869	384	A00177	A09249	229
A00177	A09644	63	A00177	A10580	268
A00177	A10599	422	A00177	A10660	278
A00177	A10725	127	A00177	A11190	380
A00177	A11392	481	A00177	A11400	162
A00177	A11522	470	A00177	A11846	306
A00177	A11932	419	A00177	A12047	212
A00177	A12137	236	A00177	A12329	254
A00177	A13336	213	A00177	A13549	374
A00177	A13944	358	A00177	A13989	730
A00177	A13994	490	A00177	A14060	262
A00177	A14072	296	A00177	A14172	379
A00177	A14216	200	A00177	A14294	213
A00177	A14371	184	A00177	A14511	461
A00177	A14633	254	A00177	A15229	180
A00177	A15256	188	A00177	A15671	173
A00177	A15684	189	A00177	A15884	160
A00177	A16682	152	A00177	A16686	196
A00178	A00202	177	A00178	A00203	941
A00178	A00209	899	A00178	A00441	329
A00178	A00605	168	A00178	A00690	455
A00178	A00916	445	A00178	A01120	283
A00178	A01166	171	A00178	A01206	425
A00178	A01234	168	A00178	A01235	162

A00178	A01384	272	A00178	A01425	160
A00178	A01470	425	A00178	A01488	294
A00178	A01492	945	A00178	A01512	899
A00178	A01527	899	A00178	A01601	152
A00178	A01605	223	A00178	A01649	463
A00178	A01730	899	A00178	A01734	217
A00178	A02265	335	A00178	A02501	243
A00178	A02564	157	A00178	A03480	168
A00178	A03617	226	A00178	A03863	195
A00178	A05563	185	A00178	A07347	185
A00178	A07422	899	A00178	A10616	177
A00178	A11209	306	A00178	A11983	899
A00178	A13875	236	A00178	A13989	933
A00178	A14055	899	A00178	A14151	299
A00178	A14410	162	A00178	A15660	291
A00179	A00187	395	A00179	A00248	369
A00179	A00260	236	A00179	A01248	213
A00179	A01639	486	A00179	A01653	367
A00179	A01790	208	A00179	A03071	195
A00179	A03095	491	A00179	A03612	154
A00179	A04109	260	A00179	A04446	162
A00179	A04449	177	A00179	A04969	263
A00179	A05367	256	A00179	A05418	578
A00179	A05453	285	A00179	A05944	339
A00179	A06289	440	A00179	A06588	742
A00179	A06650	174	A00179	A06688	314
A00179	A07206	340	A00179	A09018	201
A00179	A09778	218	A00179	A10392	452
A00179	A10599	276	A00179	A11304	215
A00179	A11379	162	A00179	A12667	174
A00179	A12897	285	A00179	A12971	172
A00179	A14201	174	A00179	A14511	236
A00179	A16792	195	A00180	A00184	140
A00180	A00185	163	A00180	A00208	191
A00180	A00234	844	A00180	A00243	183
A00180	A00250	484	A00180	A00276	936
A00180	A00900	162	A00180	A00912	335
A00180	A01083	466	A00180	A01128	232
A00180	A01410	247	A00180	A01489	385
A00180	A01524	899	A00180	A01562	610
A00180	A01628	77	A00180	A01631	215
A00180	A01692	379	A00180	A01718	168
A00180	A01719	71	A00180	A01790	376
A00180	A01822	161	A00180	A01904	321
A00180	A02088	160	A00180	A02179	160
A00180	A02411	439	A00180	A02470	218

A00180	A02473	245	A00180	A02675	172
A00180	A02877	666	A00180	A03632	401
A00180	A03739	401	A00180	A03812	347
A00180	A03837	662	A00180	A04115	649
A00180	A04342	173	A00180	A05530	446
A00180	A06289	141	A00180	A06748	339
A00180	A07061	245	A00180	A07163	469
A00180	A07311	232	A00180	A07399	347
A00180	A08259	400	A00180	A09218	196
A00180	A09504	201	A00180	A10332	199
A00180	A10506	191	A00180	A10822	290
A00180	A10844	205	A00180	A10949	188
A00180	A11124	174	A00180	A11359	339
A00180	A11608	230	A00180	A11694	249
A00180	A12383	491	A00180	A12969	489
A00180	A12971	469	A00180	A13824	183
A00180	A14004	157	A00180	A14034	262
A00180	A14035	290	A00180	A14205	279
A00180	A14209	441	A00180	A14495	290
A00180	A15308	366	A00180	A15321	191
A00180	A15323	191	A00180	A15512	400
A00180	A15665	470	A00180	A15677	406
A00180	A16840	281	A00181	A00191	533
A00181	A00212	470	A00181	A00216	303
A00181	A00270	195	A00181	A00312	219
A00181	A00316	270	A00181	A00352	473
A00181	A00374	313	A00181	A00457	322
A00181	A00497	235	A00181	A00521	228
A00181	A00537	446	A00181	A00600	307
A00181	A00662	316	A00181	A00674	185
A00181	A00891	491	A00181	A00916	480
A00181	A01022	210	A00181	A01161	466
A00181	A01216	259	A00181	A01234	490
A00181	A01244	229	A00181	A01311	162
A00181	A01361	183	A00181	A01451	486
A00181	A01453	408	A00181	A01457	531
A00181	A01458	615	A00181	A01461	532
A00181	A01492	526	A00181	A01494	835
A00181	A01503	528	A00181	A01515	418
A00181	A01520	247	A00181	A01522	322
A00181	A01542	893	A00181	A01573	157
A00181	A01574	486	A00181	A01576	546
A00181	A01585	548	A00181	A01593	450
A00181	A01601	124	A00181	A01607	313
A00181	A01612	382	A00181	A01798	462
A00181	A01923	208	A00181	A01927	196

A00181	A02148	236	A00181	A02188	313
A00181	A02215	486	A00181	A02545	405
A00181	A02607	366	A00181	A03050	430
A00181	A03186	320	A00181	A03205	490
A00181	A03444	263	A00181	A03524	259
A00181	A03569	425	A00181	A03597	256
A00181	A03962	171	A00181	A04368	154
A00181	A04686	491	A00181	A05153	160
A00181	A05234	240	A00181	A05356	490
A00181	A05807	204	A00181	A05914	219
A00181	A06581	567	A00181	A06735	313
A00181	A07020	167	A00181	A07111	491
A00181	A07452	400	A00181	A07468	469
A00181	A07471	416	A00181	A07541	439
A00181	A07610	193	A00181	A07633	201
A00181	A07815	432	A00181	A08193	172
A00181	A08404	201	A00181	A08785	473
A00181	A08817	185	A00181	A08882	458
A00181	A08903	491	A00181	A09583	218
A00181	A09932	334	A00181	A10451	204
A00181	A10458	159	A00181	A10616	227
A00181	A10643	299	A00181	A10810	640
A00181	A10886	204	A00181	A10967	414
A00181	A11311	159	A00181	A11363	399
A00181	A11508	276	A00181	A11621	183
A00181	A12107	467	A00181	A13327	201
A00181	A13329	241	A00181	A13428	167
A00181	A13830	461	A00181	A13831	481
A00181	A13989	490	A00181	A14025	590
A00181	A14127	183	A00181	A14204	528
A00181	A14247	347	A00181	A14475	633
A00181	A14485	486	A00181	A14562	252
A00181	A14585	491	A00181	A14589	573
A00181	A14637	353	A00181	A14643	490
A00181	A14655	178	A00181	A14907	167
A00181	A14986	286	A00181	A15295	294
A00181	A15498	154	A00181	A15617	228
A00181	A16082	430	A00181	A16876	208
A00182	A00183	491	A00182	A00187	180
A00182	A00190	234	A00182	A00204	188
A00182	A00207	902	A00182	A00214	430
A00182	A00224	899	A00182	A00246	899
A00182	A00630	329	A00182	A00641	207
A00182	A00671	196	A00182	A00887	172
A00182	A00921	217	A00182	A00939	218
A00182	A01052	187	A00182	A01103	205

A00182	A01125	260	A00182	A01148	168
A00182	A01307	340	A00182	A01507	176
A00182	A01524	294	A00182	A01527	945
A00182	A01558	899	A00182	A01560	899
A00182	A01588	899	A00182	A01603	196
A00182	A01615	899	A00182	A01639	188
A00182	A01724	217	A00182	A01730	436
A00182	A01775	199	A00182	A01995	246
A00182	A02340	157	A00182	A02621	154
A00182	A02640	160	A00182	A02739	165
A00182	A02769	235	A00182	A02790	157
A00182	A02822	226	A00182	A02859	490
A00182	A02889	247	A00182	A02896	174
A00182	A02912	218	A00182	A02954	279
A00182	A02961	223	A00182	A03018	391
A00182	A03224	165	A00182	A03275	305
A00182	A03279	223	A00182	A03280	191
A00182	A03316	255	A00182	A03778	458
A00182	A03834	274	A00182	A03984	337
A00182	A03985	440	A00182	A04096	460
A00182	A04097	469	A00182	A04375	174
A00182	A04449	335	A00182	A04494	247
A00182	A04557	213	A00182	A04678	263
A00182	A04682	190	A00182	A04692	188
A00182	A04731	191	A00182	A04954	271
A00182	A05065	152	A00182	A05168	217
A00182	A05169	267	A00182	A05212	307
A00182	A05583	288	A00182	A06254	313
A00182	A06317	232	A00182	A06434	217
A00182	A07106	249	A00182	A07149	162
A00182	A07157	188	A00182	A07167	157
A00182	A07311	899	A00182	A07314	443
A00182	A07560	395	A00182	A08759	321
A00182	A08878	238	A00182	A09241	176
A00182	A09395	340	A00182	A09542	899
A00182	A09647	165	A00182	A10109	260
A00182	A10580	615	A00182	A10811	168
A00182	A11983	221	A00182	A12997	313
A00182	A13667	313	A00182	A13860	168
A00182	A14208	463	A00182	A14211	380
A00182	A14218	380	A00182	A14443	926
A00182	A14646	183	A00182	A15103	208
A00182	A15314	218	A00182	A15361	173
A00182	A15578	201	A00182	A15639	445
A00182	A15834	151	A00182	A15976	271
A00182	A16068	180	A00182	A16216	429

A00182	A16635	204	A00183	A00185	205
A00183	A00191	451	A00183	A00198	558
A00183	A00207	391	A00183	A00210	460
A00183	A00230	899	A00183	A00235	899
A00183	A00239	313	A00183	A00240	492
A00183	A00241	568	A00183	A00243	316
A00183	A00246	374	A00183	A00254	427
A00183	A00257	423	A00183	A00261	869
A00183	A00324	272	A00183	A00416	157
A00183	A00445	198	A00183	A00537	173
A00183	A00630	330	A00183	A00641	216
A00183	A00643	313	A00183	A00679	439
A00183	A00696	243	A00183	A00809	432
A00183	A00931	159	A00183	A00981	328
A00183	A01052	450	A00183	A01151	195
A00183	A01213	234	A00183	A01507	358
A00183	A01524	439	A00183	A01527	944
A00183	A01539	353	A00183	A01558	924
A00183	A01596	579	A00183	A01606	399
A00183	A01615	899	A00183	A01636	224
A00183	A01676	174	A00183	A01688	235
A00183	A01730	329	A00183	A01790	246
A00183	A01850	366	A00183	A01914	433
A00183	A01920	706	A00183	A01995	319
A00183	A02210	210	A00183	A02698	235
A00183	A02803	213	A00183	A02968	246
A00183	A03188	245	A00183	A03591	205
A00183	A03752	170	A00183	A03778	491
A00183	A03893	204	A00183	A04012	506
A00183	A04033	491	A00183	A04449	491
A00183	A05221	468	A00183	A05288	201
A00183	A05583	439	A00183	A05944	216
A00183	A06190	360	A00183	A06197	271
A00183	A06254	486	A00183	A06277	466
A00183	A06373	391	A00183	A06588	460
A00183	A06814	224	A00183	A07004	490
A00183	A07149	162	A00183	A07229	400
A00183	A07422	913	A00183	A07495	170
A00183	A07803	484	A00183	A07966	297
A00183	A08079	151	A00183	A08118	291
A00183	A08420	232	A00183	A08550	407
A00183	A08571	225	A00183	A08859	360
A00183	A08869	269	A00183	A08878	480
A00183	A09256	899	A00183	A09460	219
A00183	A09750	321	A00183	A09870	168
A00183	A10165	157	A00183	A10580	435

A00183	A10666	278	A00183	A10913	151
A00183	A11124	496	A00183	A11487	236
A00183	A11727	303	A00183	A11983	491
A00183	A12125	188	A00183	A12365	366
A00183	A12534	210	A00183	A12897	291
A00183	A13250	899	A00183	A13809	238
A00183	A14055	413	A00183	A14202	450
A00183	A14220	224	A00183	A14299	614
A00183	A14410	377	A00183	A14570	167
A00183	A14635	475	A00183	A14653	373
A00183	A14704	291	A00183	A14925	353
A00183	A15322	393	A00183	A15357	160
A00183	A15360	472	A00183	A15402	218
A00183	A15506	759	A00183	A15510	199
A00183	A15606	430	A00183	A15726	899
A00183	A16036	232	A00183	A16157	446
A00184	A00187	252	A00184	A00207	899
A00184	A00209	899	A00184	A00224	899
A00184	A00236	903	A00184	A00246	899
A00184	A00252	903	A00184	A00253	165
A00184	A00267	215	A00184	A00271	899
A00184	A00273	355	A00184	A00276	930
A00184	A00308	367	A00184	A00548	154
A00184	A00679	899	A00184	A00868	235
A00184	A00926	154	A00184	A01052	899
A00184	A01148	899	A00184	A01254	177
A00184	A01512	899	A00184	A01524	899
A00184	A01527	899	A00184	A01553	899
A00184	A01554	165	A00184	A01558	899
A00184	A01560	899	A00184	A01562	944
A00184	A01588	899	A00184	A01602	899
A00184	A01615	899	A00184	A01628	899
A00184	A01639	173	A00184	A01665	899
A00184	A01688	899	A00184	A01692	904
A00184	A01697	130	A00184	A01708	904
A00184	A01709	899	A00184	A01719	906
A00184	A01844	899	A00184	A02492	154
A00184	A02717	167	A00184	A03126	899
A00184	A03517	338	A00184	A03703	420
A00184	A04699	899	A00184	A07101	216
A00184	A07166	177	A00184	A07311	899
A00184	A08899	899	A00184	A09577	899
A00184	A09766	201	A00184	A10560	899
A00184	A10813	899	A00184	A11125	899
A00184	A11983	899	A00184	A12664	899
A00184	A12705	899	A00184	A12969	899

A00184	A12971	899	A00184	A13401	899
A00184	A13977	899	A00184	A13978	201
A00184	A13985	899	A00184	A13999	899
A00184	A14033	899	A00184	A14043	899
A00184	A14188	899	A00184	A14242	899
A00184	A15296	461	A00184	A15726	63
A00184	A16993	899	A00185	A00186	454
A00185	A00190	435	A00185	A00200	899
A00185	A00205	82	A00185	A00207	945
A00185	A00208	945	A00185	A00209	899
A00185	A00213	447	A00185	A00214	481
A00185	A00224	945	A00185	A00227	481
A00185	A00234	491	A00185	A00240	931
A00185	A00246	909	A00185	A00247	486
A00185	A00254	969	A00185	A00271	899
A00185	A00285	156	A00185	A00294	446
A00185	A00309	475	A00185	A00316	491
A00185	A00320	227	A00185	A00324	899
A00185	A00333	241	A00185	A00340	491
A00185	A00385	241	A00185	A00418	369
A00185	A00439	452	A00185	A00451	491
A00185	A00552	215	A00185	A00557	193
A00185	A00624	409	A00185	A00688	232
A00185	A00736	221	A00185	A00747	316
A00185	A00887	413	A00185	A00932	469
A00185	A00933	373	A00185	A00939	302
A00185	A00963	443	A00185	A00997	307
A00185	A01009	157	A00185	A01036	180
A00185	A01052	486	A00185	A01070	899
A00185	A01104	201	A00185	A01212	463
A00185	A01286	485	A00185	A01307	185
A00185	A01473	369	A00185	A01524	994
A00185	A01527	935	A00185	A01553	63
A00185	A01554	63	A00185	A01558	989
A00185	A01560	491	A00185	A01562	899
A00185	A01572	368	A00185	A01588	989
A00185	A01605	899	A00185	A01615	899
A00185	A01640	899	A00185	A01651	899
A00185	A01667	899	A00185	A01675	171
A00185	A01676	908	A00185	A01702	899
A00185	A01709	63	A00185	A01718	899
A00185	A01741	312	A00185	A01748	278
A00185	A01832	428	A00185	A01844	899
A00185	A01890	269	A00185	A01907	302
A00185	A01946	201	A00185	A01995	491
A00185	A02027	240	A00185	A02115	241

A00185	A02215	272	A00185	A02278	154
A00185	A02401	307	A00185	A02414	226
A00185	A02481	486	A00185	A02482	408
A00185	A02586	176	A00185	A02629	165
A00185	A02634	352	A00185	A02636	480
A00185	A02638	417	A00185	A02639	180
A00185	A02678	696	A00185	A02695	483
A00185	A02743	340	A00185	A02749	440
A00185	A02752	252	A00185	A02753	424
A00185	A02768	485	A00185	A02819	347
A00185	A02836	238	A00185	A02842	273
A00185	A02877	477	A00185	A02882	174
A00185	A02908	899	A00185	A02981	198
A00185	A03080	154	A00185	A03099	180
A00185	A03126	433	A00185	A03254	183
A00185	A03271	406	A00185	A03292	272
A00185	A03309	154	A00185	A03331	218
A00185	A03413	241	A00185	A03415	367
A00185	A03419	252	A00185	A03436	404
A00185	A03451	486	A00185	A03459	241
A00185	A03634	162	A00185	A03685	302
A00185	A03752	490	A00185	A03784	485
A00185	A03809	167	A00185	A03830	450
A00185	A03864	288	A00185	A03910	172
A00185	A03976	198	A00185	A03990	316
A00185	A04020	216	A00185	A04080	367
A00185	A04117	388	A00185	A04229	257
A00185	A04293	399	A00185	A04297	154
A00185	A04314	174	A00185	A04323	339
A00185	A04442	216	A00185	A04616	205
A00185	A04651	219	A00185	A04696	234
A00185	A04706	198	A00185	A04731	480
A00185	A04795	212	A00185	A04801	219
A00185	A04804	276	A00185	A04864	294
A00185	A04965	391	A00185	A04991	344
A00185	A05072	174	A00185	A05082	316
A00185	A05084	301	A00185	A05165	417
A00185	A05216	902	A00185	A05339	154
A00185	A05373	193	A00185	A05400	241
A00185	A05440	241	A00185	A05458	228
A00185	A05473	319	A00185	A05570	260
A00185	A05633	196	A00185	A05701	163
A00185	A06077	160	A00185	A06256	183
A00185	A06466	491	A00185	A06586	157
A00185	A06609	899	A00185	A06610	257
A00185	A06665	278	A00185	A06879	276

A00185	A07033	241	A00185	A07091	899
A00185	A07118	167	A00185	A07127	286
A00185	A07135	367	A00185	A07163	318
A00185	A07201	899	A00185	A07279	167
A00185	A07311	945	A00185	A07339	483
A00185	A07421	154	A00185	A07555	241
A00185	A07726	329	A00185	A07762	255
A00185	A07798	490	A00185	A08070	350
A00185	A08183	435	A00185	A08541	254
A00185	A08608	190	A00185	A08640	276
A00185	A08732	899	A00185	A08869	313
A00185	A08878	904	A00185	A08911	235
A00185	A08998	263	A00185	A09018	198
A00185	A09078	180	A00185	A09176	219
A00185	A09220	694	A00185	A09239	460
A00185	A09249	167	A00185	A09251	154
A00185	A09399	174	A00185	A09418	174
A00185	A09486	241	A00185	A09488	241
A00185	A09536	335	A00185	A09577	85
A00185	A09742	490	A00185	A09791	238
A00185	A09804	219	A00185	A09902	291
A00185	A09937	177	A00185	A10125	219
A00185	A10126	167	A00185	A10133	193
A00185	A10174	899	A00185	A10220	276
A00185	A10337	185	A00185	A10365	361
A00185	A10468	276	A00185	A10471	326
A00185	A10538	193	A00185	A10728	294
A00185	A10735	146	A00185	A10801	423
A00185	A10833	899	A00185	A10913	124
A00185	A11206	329	A00185	A11286	486
A00185	A11369	246	A00185	A11431	477
A00185	A11504	219	A00185	A11604	945
A00185	A11672	154	A00185	A11769	183
A00185	A11891	219	A00185	A11911	286
A00185	A11983	372	A00185	A12328	273
A00185	A12343	307	A00185	A12393	469
A00185	A12409	160	A00185	A13145	174
A00185	A13149	488	A00185	A13172	335
A00185	A13335	162	A00185	A13340	485
A00185	A13364	154	A00185	A13394	205
A00185	A13413	167	A00185	A13445	276
A00185	A13605	432	A00185	A13625	276
A00185	A13718	276	A00185	A13974	205
A00185	A13975	234	A00185	A13980	260
A00185	A13983	91	A00185	A13986	405
A00185	A13997	229	A00185	A14007	419

A00185	A14019	928	A00185	A14157	241
A00185	A14187	447	A00185	A14188	491
A00185	A14198	945	A00185	A14210	173
A00185	A14364	177	A00185	A14371	374
A00185	A14378	396	A00185	A14399	436
A00185	A14401	224	A00185	A14402	276
A00185	A14410	273	A00185	A14426	173
A00185	A14433	943	A00185	A14438	425
A00185	A14443	899	A00185	A14452	157
A00185	A14511	378	A00185	A14635	174
A00185	A14643	353	A00185	A14738	152
A00185	A14827	170	A00185	A15230	212
A00185	A15293	911	A00185	A15294	916
A00185	A15301	422	A00185	A15302	467
A00185	A15325	377	A00185	A15335	229
A00185	A15366	433	A00185	A15413	485
A00185	A15420	281	A00185	A15428	167
A00185	A15463	462	A00185	A15487	160
A00185	A15506	362	A00185	A15510	488
A00185	A15512	174	A00185	A15674	196
A00185	A15734	259	A00185	A15832	183
A00185	A15862	335	A00185	A15913	152
A00185	A15927	219	A00185	A15977	326
A00185	A16300	447	A00185	A16450	385
A00185	A16985	183	A00186	A00224	899
A00186	A00230	899	A00186	A00234	213
A00186	A00246	899	A00186	A00255	221
A00186	A00340	382	A00186	A01128	224
A00186	A01475	229	A00186	A01524	906
A00186	A01527	917	A00186	A01553	902
A00186	A01558	899	A00186	A01588	899
A00186	A01615	899	A00186	A01727	162
A00186	A01749	171	A00186	A01787	177
A00186	A01895	326	A00186	A01900	296
A00186	A01920	905	A00186	A02335	404
A00186	A03661	168	A00186	A03821	202
A00186	A04002	378	A00186	A04315	480
A00186	A04323	285	A00186	A04490	272
A00186	A04879	180	A00186	A04933	344
A00186	A05866	307	A00186	A05944	236
A00186	A06635	486	A00186	A07311	899
A00186	A09220	944	A00186	A13593	177
A00186	A14401	490	A00187	A00217	388
A00187	A00243	154	A00187	A00248	291
A00187	A00257	450	A00187	A00267	443
A00187	A00273	488	A00187	A01512	472

A00187	A01554	294	A00187	A01560	63
A00187	A01562	491	A00187	A01596	319
A00187	A01628	413	A00187	A01639	491
A00187	A01640	171	A00187	A01653	265
A00187	A01664	477	A00187	A01692	63
A00187	A01697	152	A00187	A01702	385
A00187	A01708	363	A00187	A01709	480
A00187	A01718	481	A00187	A01719	441
A00187	A01730	152	A00187	A01740	330
A00187	A01908	218	A00187	A02341	151
A00187	A03095	414	A00187	A03656	238
A00187	A03960	232	A00187	A04205	491
A00187	A04452	302	A00187	A04536	160
A00187	A04858	424	A00187	A06289	65
A00187	A06601	332	A00187	A06610	152
A00187	A06701	438	A00187	A07654	188
A00187	A08218	193	A00187	A08902	433
A00187	A09162	491	A00187	A10392	490
A00187	A10595	159	A00187	A10808	113
A00187	A11252	302	A00187	A11379	154
A00187	A11696	177	A00187	A11767	201
A00187	A11830	218	A00187	A11852	165
A00187	A12383	452	A00187	A13539	323
A00188	A00190	486	A00188	A00240	321
A00188	A00248	491	A00188	A00256	449
A00188	A00265	475	A00188	A00266	208
A00188	A00324	170	A00188	A00336	259
A00188	A01560	165	A00188	A01639	945
A00188	A01654	338	A00188	A01669	468
A00188	A01729	276	A00188	A01730	279
A00188	A01742	491	A00188	A01746	483
A00188	A01756	412	A00188	A01776	218
A00188	A01781	340	A00188	A01850	390
A00188	A01880	208	A00188	A01888	252
A00188	A01900	899	A00188	A01902	200
A00188	A01983	408	A00188	A02021	154
A00188	A02337	281	A00188	A02383	463
A00188	A02498	266	A00188	A02630	263
A00188	A02645	278	A00188	A02662	291
A00188	A02699	486	A00188	A02821	277
A00188	A02843	201	A00188	A02854	314
A00188	A02866	232	A00188	A02873	321
A00188	A02886	291	A00188	A02909	388
A00188	A02945	180	A00188	A03541	477
A00188	A03662	485	A00188	A03846	445
A00188	A03869	383	A00188	A03871	390

A00188	A03942	451	A00188	A04049	190
A00188	A04088	163	A00188	A04248	463
A00188	A04320	369	A00188	A04528	291
A00188	A04536	491	A00188	A04584	232
A00188	A04734	201	A00188	A04865	270
A00188	A04976	210	A00188	A05038	218
A00188	A05270	291	A00188	A05389	193
A00188	A05556	241	A00188	A05558	251
A00188	A05583	238	A00188	A05634	185
A00188	A05854	302	A00188	A05944	479
A00188	A06054	399	A00188	A06055	491
A00188	A06601	468	A00188	A06780	218
A00188	A07157	358	A00188	A07173	272
A00188	A07372	215	A00188	A07390	452
A00188	A07422	265	A00188	A08615	267
A00188	A08633	185	A00188	A08711	363
A00188	A08804	185	A00188	A09046	456
A00188	A09062	210	A00188	A09162	267
A00188	A09842	229	A00188	A10097	334
A00188	A10392	923	A00188	A10623	279
A00188	A10969	258	A00188	A10982	369
A00188	A11300	274	A00188	A14376	173
A00188	A14432	183	A00188	A15605	227
A00188	A15608	243	A00189	A00208	899
A00189	A00209	919	A00189	A00220	277
A00189	A00224	943	A00189	A00230	899
A00189	A00232	373	A00189	A00236	488
A00189	A00241	462	A00189	A00246	928
A00189	A00252	491	A00189	A00253	489
A00189	A00254	213	A00189	A00261	335
A00189	A00324	899	A00189	A00531	193
A00189	A00588	183	A00189	A00604	308
A00189	A00643	283	A00189	A00652	490
A00189	A00653	276	A00189	A00814	310
A00189	A00820	205	A00189	A01052	899
A00189	A01289	489	A00189	A01524	937
A00189	A01527	913	A00189	A01559	383
A00189	A01614	265	A00189	A01616	491
A00189	A01659	432	A00189	A01688	899
A00189	A01692	388	A00189	A01709	340
A00189	A01730	468	A00189	A01876	240
A00189	A01881	360	A00189	A01889	208
A00189	A02178	419	A00189	A02304	475
A00189	A02382	731	A00189	A02416	159
A00189	A02698	489	A00189	A02704	168
A00189	A02711	491	A00189	A02863	229

A00189	A03010	945	A00189	A03255	180
A00189	A03683	152	A00189	A03790	301
A00189	A03817	480	A00189	A03821	154
A00189	A03914	154	A00189	A03972	218
A00189	A04033	486	A00189	A04117	162
A00189	A04176	339	A00189	A04250	200
A00189	A04320	462	A00189	A04449	486
A00189	A04451	210	A00189	A04473	252
A00189	A04668	329	A00189	A04736	180
A00189	A04807	293	A00189	A05082	491
A00189	A05417	210	A00189	A05679	168
A00189	A06107	174	A00189	A06270	265
A00189	A06277	221	A00189	A06552	356
A00189	A06650	293	A00189	A06982	486
A00189	A07004	358	A00189	A07151	154
A00189	A07158	436	A00189	A07375	247
A00189	A08416	489	A00189	A08679	185
A00189	A08729	171	A00189	A08812	259
A00189	A09470	162	A00189	A09524	174
A00189	A09544	332	A00189	A09732	232
A00189	A09764	441	A00189	A10055	171
A00189	A10224	208	A00189	A10813	299
A00189	A10899	433	A00189	A11369	943
A00189	A11543	491	A00189	A11707	467
A00189	A11875	179	A00189	A11901	382
A00189	A11983	980	A00189	A12971	371
A00189	A13066	218	A00189	A13110	229
A00189	A13181	297	A00189	A13247	174
A00189	A13860	469	A00189	A13949	165
A00189	A13975	154	A00189	A13981	899
A00189	A13985	899	A00189	A13996	229
A00189	A14029	903	A00189	A14094	455
A00189	A14198	354	A00189	A14236	462
A00189	A14426	205	A00189	A14472	183
A00189	A14517	286	A00189	A14570	303
A00189	A14645	265	A00189	A14694	479
A00189	A15241	429	A00189	A15599	184
A00189	A15605	157	A00189	A15606	486
A00189	A15665	168	A00189	A15726	422
A00189	A16354	229	A00189	A16476	480
A00189	A16513	152	A00190	A00196	489
A00190	A00197	157	A00190	A00200	232
A00190	A00204	747	A00190	A00207	899
A00190	A00208	451	A00190	A00209	944
A00190	A00210	249	A00190	A00212	374
A00190	A00214	430	A00190	A00216	416

A00190	A00218	483	A00190	A00219	491
A00190	A00220	527	A00190	A00224	945
A00190	A00227	413	A00190	A00230	899
A00190	A00231	491	A00190	A00232	491
A00190	A00233	945	A00190	A00234	489
A00190	A00238	205	A00190	A00245	296
A00190	A00248	491	A00190	A00250	899
A00190	A00254	488	A00190	A00256	245
A00190	A00262	901	A00190	A00264	945
A00190	A00265	491	A00190	A00274	488
A00190	A00292	364	A00190	A00294	944
A00190	A00305	463	A00190	A00309	490
A00190	A00324	943	A00190	A00327	457
A00190	A00328	255	A00190	A00351	445
A00190	A00365	249	A00190	A00370	324
A00190	A00377	457	A00190	A00395	226
A00190	A00420	179	A00190	A00449	488
A00190	A00457	246	A00190	A00472	398
A00190	A00478	160	A00190	A00482	427
A00190	A00483	199	A00190	A00531	368
A00190	A00537	165	A00190	A00538	379
A00190	A00541	296	A00190	A00556	488
A00190	A00558	283	A00190	A00570	430
A00190	A00574	355	A00190	A00576	191
A00190	A00591	286	A00190	A00595	445
A00190	A00601	257	A00190	A00616	223
A00190	A00649	360	A00190	A00659	266
A00190	A00674	196	A00190	A00688	481
A00190	A00709	174	A00190	A00722	468
A00190	A00730	241	A00190	A00731	311
A00190	A00736	491	A00190	A00747	374
A00190	A00765	404	A00190	A00784	440
A00190	A00791	332	A00190	A00799	491
A00190	A00815	332	A00190	A00820	486
A00190	A00858	254	A00190	A00863	210
A00190	A00868	461	A00190	A00869	165
A00190	A00914	491	A00190	A00926	423
A00190	A00931	361	A00190	A00950	374
A00190	A00993	470	A00190	A01003	154
A00190	A01009	934	A00190	A01011	540
A00190	A01018	174	A00190	A01020	180
A00190	A01080	899	A00190	A01086	339
A00190	A01093	352	A00190	A01108	469
A00190	A01114	412	A00190	A01116	156
A00190	A01117	316	A00190	A01125	394
A00190	A01137	252	A00190	A01156	377

A00190	A01157	432	A00190	A01178	491
A00190	A01204	162	A00190	A01210	286
A00190	A01223	396	A00190	A01230	435
A00190	A01240	204	A00190	A01253	154
A00190	A01256	202	A00190	A01257	265
A00190	A01281	340	A00190	A01296	311
A00190	A01297	363	A00190	A01307	480
A00190	A01317	406	A00190	A01318	441
A00190	A01320	486	A00190	A01323	213
A00190	A01324	210	A00190	A01325	235
A00190	A01328	454	A00190	A01346	219
A00190	A01354	371	A00190	A01359	327
A00190	A01360	294	A00190	A01416	168
A00190	A01427	168	A00190	A01452	479
A00190	A01460	249	A00190	A01463	405
A00190	A01468	491	A00190	A01473	344
A00190	A01483	466	A00190	A01489	899
A00190	A01504	378	A00190	A01512	899
A00190	A01524	899	A00190	A01527	943
A00190	A01551	491	A00190	A01553	899
A00190	A01554	455	A00190	A01558	899
A00190	A01567	185	A00190	A01572	199
A00190	A01583	369	A00190	A01590	409
A00190	A01591	899	A00190	A01593	899
A00190	A01602	942	A00190	A01604	427
A00190	A01606	899	A00190	A01626	454
A00190	A01629	221	A00190	A01631	926
A00190	A01635	446	A00190	A01643	430
A00190	A01647	199	A00190	A01666	208
A00190	A01668	491	A00190	A01676	491
A00190	A01677	347	A00190	A01688	899
A00190	A01704	491	A00190	A01725	160
A00190	A01726	473	A00190	A01727	277
A00190	A01728	420	A00190	A01729	483
A00190	A01730	582	A00190	A01733	486
A00190	A01737	262	A00190	A01739	252
A00190	A01740	338	A00190	A01741	945
A00190	A01742	177	A00190	A01743	491
A00190	A01746	390	A00190	A01747	366
A00190	A01749	157	A00190	A01752	395
A00190	A01754	307	A00190	A01755	414
A00190	A01759	480	A00190	A01767	475
A00190	A01769	404	A00190	A01775	491
A00190	A01781	429	A00190	A01785	357
A00190	A01789	491	A00190	A01796	491
A00190	A01797	491	A00190	A01798	374

A00190	A01802	182	A00190	A01815	196
A00190	A01844	484	A00190	A01863	314
A00190	A01870	183	A00190	A01875	339
A00190	A01876	480	A00190	A01879	491
A00190	A01880	180	A00190	A01887	491
A00190	A01888	491	A00190	A01889	191
A00190	A01894	473	A00190	A01900	394
A00190	A01902	489	A00190	A01908	259
A00190	A01923	168	A00190	A01926	479
A00190	A01971	218	A00190	A01980	157
A00190	A01981	322	A00190	A01982	364
A00190	A02006	449	A00190	A02010	433
A00190	A02011	363	A00190	A02020	480
A00190	A02034	168	A00190	A02063	319
A00190	A02065	334	A00190	A02074	160
A00190	A02080	195	A00190	A02083	180
A00190	A02086	439	A00190	A02117	238
A00190	A02119	199	A00190	A02138	202
A00190	A02143	469	A00190	A02148	429
A00190	A02156	208	A00190	A02158	374
A00190	A02161	353	A00190	A02168	180
A00190	A02174	374	A00190	A02179	157
A00190	A02180	489	A00190	A02183	374
A00190	A02215	452	A00190	A02229	157
A00190	A02235	160	A00190	A02236	340
A00190	A02248	205	A00190	A02251	447
A00190	A02254	235	A00190	A02268	480
A00190	A02275	334	A00190	A02278	468
A00190	A02292	207	A00190	A02298	385
A00190	A02300	247	A00190	A02304	402
A00190	A02340	273	A00190	A02345	340
A00190	A02361	308	A00190	A02372	283
A00190	A02377	485	A00190	A02380	180
A00190	A02382	466	A00190	A02390	318
A00190	A02407	268	A00190	A02412	414
A00190	A02414	165	A00190	A02416	443
A00190	A02425	191	A00190	A02431	466
A00190	A02435	427	A00190	A02454	485
A00190	A02460	256	A00190	A02464	215
A00190	A02473	377	A00190	A02481	296
A00190	A02488	177	A00190	A02490	481
A00190	A02491	489	A00190	A02492	414
A00190	A02494	335	A00190	A02495	269
A00190	A02499	317	A00190	A02505	420
A00190	A02506	210	A00190	A02508	333
A00190	A02512	396	A00190	A02521	485

A00190	A02524	208	A00190	A02564	481
A00190	A02582	170	A00190	A02585	238
A00190	A02586	168	A00190	A02594	212
A00190	A02605	152	A00190	A02624	252
A00190	A02630	152	A00190	A02639	484
A00190	A02668	424	A00190	A02672	255
A00190	A02675	193	A00190	A02695	480
A00190	A02696	337	A00190	A02697	419
A00190	A02698	187	A00190	A02703	436
A00190	A02709	481	A00190	A02711	127
A00190	A02714	491	A00190	A02730	439
A00190	A02741	307	A00190	A02745	477
A00190	A02746	224	A00190	A02749	489
A00190	A02754	429	A00190	A02756	395
A00190	A02772	238	A00190	A02777	278
A00190	A02784	176	A00190	A02785	447
A00190	A02787	481	A00190	A02788	454
A00190	A02789	216	A00190	A02812	490
A00190	A02814	452	A00190	A02816	451
A00190	A02828	935	A00190	A02829	358
A00190	A02830	491	A00190	A02836	469
A00190	A02837	491	A00190	A02842	302
A00190	A02859	491	A00190	A02864	395
A00190	A02869	338	A00190	A02874	491
A00190	A02875	321	A00190	A02877	457
A00190	A02878	380	A00190	A02895	208
A00190	A02896	491	A00190	A02897	182
A00190	A02904	159	A00190	A02908	461
A00190	A02913	183	A00190	A02916	468
A00190	A02918	213	A00190	A02920	245
A00190	A02922	154	A00190	A02923	183
A00190	A02927	176	A00190	A02942	899
A00190	A02944	486	A00190	A02951	190
A00190	A02961	458	A00190	A02972	267
A00190	A02982	469	A00190	A02983	491
A00190	A02984	366	A00190	A02988	240
A00190	A03001	176	A00190	A03003	283
A00190	A03028	154	A00190	A03034	341
A00190	A03046	218	A00190	A03051	271
A00190	A03056	256	A00190	A03068	196
A00190	A03070	199	A00190	A03074	162
A00190	A03080	468	A00190	A03087	344
A00190	A03091	466	A00190	A03094	210
A00190	A03095	479	A00190	A03099	329
A00190	A03100	205	A00190	A03104	469
A00190	A03110	489	A00190	A03112	491

A00190	A03115	218	A00190	A03121	251
A00190	A03126	297	A00190	A03131	266
A00190	A03132	168	A00190	A03141	247
A00190	A03144	427	A00190	A03181	217
A00190	A03183	182	A00190	A03186	260
A00190	A03188	177	A00190	A03196	162
A00190	A03204	218	A00190	A03211	366
A00190	A03219	408	A00190	A03223	477
A00190	A03238	251	A00190	A03259	945
A00190	A03271	200	A00190	A03275	491
A00190	A03282	375	A00190	A03304	210
A00190	A03305	215	A00190	A03315	414
A00190	A03332	273	A00190	A03347	246
A00190	A03361	193	A00190	A03370	388
A00190	A03406	180	A00190	A03412	221
A00190	A03415	346	A00190	A03419	182
A00190	A03423	185	A00190	A03436	473
A00190	A03438	302	A00190	A03451	481
A00190	A03513	358	A00190	A03520	491
A00190	A03523	366	A00190	A03537	154
A00190	A03544	63	A00190	A03596	458
A00190	A03604	263	A00190	A03609	481
A00190	A03611	374	A00190	A03626	388
A00190	A03644	238	A00190	A03661	306
A00190	A03690	407	A00190	A03703	379
A00190	A03721	424	A00190	A03722	465
A00190	A03724	395	A00190	A03725	491
A00190	A03726	899	A00190	A03732	190
A00190	A03739	473	A00190	A03748	416
A00190	A03750	247	A00190	A03763	191
A00190	A03795	224	A00190	A03797	899
A00190	A03799	167	A00190	A03813	263
A00190	A03821	386	A00190	A03834	337
A00190	A03843	486	A00190	A03846	369
A00190	A03851	489	A00190	A03861	417
A00190	A03887	450	A00190	A03894	460
A00190	A03902	335	A00190	A03910	485
A00190	A03922	185	A00190	A03942	945
A00190	A03965	249	A00190	A03981	455
A00190	A03990	485	A00190	A03992	154
A00190	A04004	173	A00190	A04009	168
A00190	A04010	490	A00190	A04048	196
A00190	A04049	176	A00190	A04050	269
A00190	A04051	454	A00190	A04060	378
A00190	A04080	475	A00190	A04082	268
A00190	A04085	296	A00190	A04088	390

A00190	A04113	168	A00190	A04117	477
A00190	A04169	491	A00190	A04202	276
A00190	A04206	322	A00190	A04216	163
A00190	A04248	202	A00190	A04304	286
A00190	A04334	463	A00190	A04342	483
A00190	A04352	373	A00190	A04398	241
A00190	A04410	340	A00190	A04417	234
A00190	A04444	485	A00190	A04453	157
A00190	A04536	425	A00190	A04548	395
A00190	A04567	277	A00190	A04614	317
A00190	A04616	455	A00190	A04622	491
A00190	A04627	173	A00190	A04637	180
A00190	A04656	260	A00190	A04714	162
A00190	A04750	172	A00190	A04751	170
A00190	A04771	276	A00190	A04776	204
A00190	A04783	402	A00190	A04873	199
A00190	A04879	580	A00190	A04880	515
A00190	A04883	177	A00190	A04891	260
A00190	A04893	268	A00190	A04902	173
A00190	A04908	399	A00190	A04911	221
A00190	A04927	247	A00190	A04939	489
A00190	A05016	296	A00190	A05034	277
A00190	A05050	199	A00190	A05112	168
A00190	A05113	251	A00190	A05122	374
A00190	A05155	489	A00190	A05194	212
A00190	A05216	899	A00190	A05220	424
A00190	A05221	485	A00190	A05261	183
A00190	A05268	168	A00190	A05278	168
A00190	A05288	446	A00190	A05309	491
A00190	A05336	168	A00190	A05349	308
A00190	A05362	168	A00190	A05418	185
A00190	A05421	168	A00190	A05439	218
A00190	A05444	168	A00190	A05472	243
A00190	A05506	172	A00190	A05568	223
A00190	A05577	245	A00190	A05603	401
A00190	A05735	398	A00190	A05824	168
A00190	A05869	151	A00190	A05933	168
A00190	A05961	245	A00190	A05999	172
A00190	A06001	446	A00190	A06053	154
A00190	A06056	183	A00190	A06067	263
A00190	A06072	168	A00190	A06118	176
A00190	A06149	238	A00190	A06289	899
A00190	A06292	168	A00190	A06346	208
A00190	A06405	246	A00190	A06422	467
A00190	A06439	213	A00190	A06569	424
A00190	A06581	491	A00190	A06595	213

A00190	A06597	388	A00190	A06606	302
A00190	A06607	435	A00190	A06608	219
A00190	A06610	484	A00190	A06615	477
A00190	A06623	251	A00190	A06642	263
A00190	A06648	168	A00190	A06660	400
A00190	A06689	152	A00190	A06693	369
A00190	A06792	168	A00190	A06797	252
A00190	A06825	299	A00190	A06949	486
A00190	A06997	296	A00190	A07062	217
A00190	A07135	485	A00190	A07138	358
A00190	A07141	396	A00190	A07145	449
A00190	A07153	483	A00190	A07157	685
A00190	A07158	454	A00190	A07159	176
A00190	A07160	174	A00190	A07163	262
A00190	A07165	945	A00190	A07191	348
A00190	A07200	475	A00190	A07201	480
A00190	A07209	491	A00190	A07218	449
A00190	A07249	286	A00190	A07314	340
A00190	A07339	480	A00190	A07363	168
A00190	A07399	452	A00190	A07400	168
A00190	A07421	323	A00190	A07422	899
A00190	A07486	228	A00190	A07488	168
A00190	A07503	188	A00190	A07520	215
A00190	A07525	232	A00190	A07540	299
A00190	A07553	180	A00190	A07566	260
A00190	A07572	151	A00190	A07723	363
A00190	A07766	484	A00190	A07794	307
A00190	A07803	489	A00190	A07927	168
A00190	A07973	180	A00190	A07993	268
A00190	A08280	644	A00190	A08366	232
A00190	A08411	168	A00190	A08559	168
A00190	A08572	484	A00190	A08732	899
A00190	A08782	168	A00190	A08791	168
A00190	A08834	489	A00190	A08837	207
A00190	A08840	432	A00190	A08841	288
A00190	A08842	462	A00190	A08863	199
A00190	A08887	177	A00190	A09001	241
A00190	A09018	340	A00190	A09022	408
A00190	A09028	447	A00190	A09058	238
A00190	A09073	337	A00190	A09137	168
A00190	A09145	246	A00190	A09196	196
A00190	A09205	293	A00190	A09206	200
A00190	A09211	252	A00190	A09215	168
A00190	A09222	157	A00190	A09238	182
A00190	A09239	345	A00190	A09243	352
A00190	A09275	212	A00190	A09379	168

A00190	A09381	278	A00190	A09394	290
A00190	A09422	327	A00190	A09429	157
A00190	A09509	405	A00190	A09573	337
A00190	A09598	469	A00190	A09612	246
A00190	A09643	899	A00190	A09644	899
A00190	A09647	165	A00190	A09672	162
A00190	A09732	488	A00190	A09740	252
A00190	A09742	474	A00190	A09744	347
A00190	A09753	160	A00190	A09804	899
A00190	A09817	314	A00190	A09833	405
A00190	A09842	165	A00190	A09847	160
A00190	A09848	379	A00190	A09855	246
A00190	A09873	391	A00190	A09875	185
A00190	A09879	180	A00190	A09882	188
A00190	A09896	168	A00190	A09907	168
A00190	A09932	311	A00190	A10044	299
A00190	A10045	299	A00190	A10046	314
A00190	A10068	168	A00190	A10087	152
A00190	A10174	299	A00190	A10214	609
A00190	A10271	168	A00190	A10332	191
A00190	A10353	172	A00190	A10382	162
A00190	A10421	152	A00190	A10435	168
A00190	A10504	162	A00190	A10506	391
A00190	A10544	200	A00190	A10579	157
A00190	A10580	944	A00190	A10587	326
A00190	A10591	160	A00190	A10592	263
A00190	A10612	449	A00190	A10625	481
A00190	A10638	266	A00190	A10658	245
A00190	A10702	163	A00190	A10789	355
A00190	A10835	168	A00190	A10914	168
A00190	A10984	168	A00190	A11069	168
A00190	A11073	162	A00190	A11110	168
A00190	A11113	223	A00190	A11122	246
A00190	A11190	219	A00190	A11191	364
A00190	A11250	229	A00190	A11288	172
A00190	A11294	180	A00190	A11300	734
A00190	A11326	223	A00190	A11404	154
A00190	A11412	422	A00190	A11431	483
A00190	A11435	469	A00190	A11444	210
A00190	A11469	278	A00190	A11526	327
A00190	A11537	443	A00190	A11545	283
A00190	A11556	172	A00190	A11558	424
A00190	A11578	338	A00190	A11607	205
A00190	A11683	191	A00190	A11768	168
A00190	A11789	168	A00190	A11835	255
A00190	A11848	228	A00190	A11850	419

A00190	A11885	162	A00190	A11926	326
A00190	A11943	899	A00190	A11978	168
A00190	A11983	534	A00190	A12041	163
A00190	A12187	168	A00190	A12195	205
A00190	A12271	168	A00190	A12361	200
A00190	A12373	374	A00190	A12393	486
A00190	A12635	168	A00190	A12719	172
A00190	A12792	163	A00190	A12824	439
A00190	A12854	385	A00190	A12897	489
A00190	A12927	475	A00190	A13003	401
A00190	A13123	212	A00190	A13124	416
A00190	A13161	168	A00190	A13259	168
A00190	A13334	168	A00190	A13338	294
A00190	A13369	488	A00190	A13371	183
A00190	A13386	168	A00190	A13460	224
A00190	A13542	168	A00190	A13595	168
A00190	A13610	168	A00190	A13658	168
A00190	A13749	168	A00190	A13816	212
A00190	A13824	157	A00190	A13826	491
A00190	A13860	678	A00190	A13899	486
A00190	A13951	302	A00190	A13975	899
A00190	A13987	463	A00190	A13991	899
A00190	A14008	899	A00190	A14010	316
A00190	A14071	235	A00190	A14072	348
A00190	A14111	154	A00190	A14122	422
A00190	A14123	313	A00190	A14126	326
A00190	A14128	491	A00190	A14129	467
A00190	A14190	468	A00190	A14191	438
A00190	A14198	379	A00190	A14202	467
A00190	A14208	632	A00190	A14210	490
A00190	A14215	263	A00190	A14229	462
A00190	A14231	836	A00190	A14232	899
A00190	A14290	249	A00190	A14320	232
A00190	A14330	221	A00190	A14343	172
A00190	A14368	180	A00190	A14371	486
A00190	A14374	187	A00190	A14378	466
A00190	A14392	193	A00190	A14394	152
A00190	A14395	469	A00190	A14399	413
A00190	A14406	160	A00190	A14408	182
A00190	A14413	163	A00190	A14416	176
A00190	A14430	377	A00190	A14440	338
A00190	A14443	224	A00190	A14470	170
A00190	A14490	162	A00190	A14511	490
A00190	A14512	168	A00190	A14520	207
A00190	A14534	168	A00190	A14564	168
A00190	A14570	455	A00190	A14604	267

A00190	A14605	339	A00190	A14635	467
A00190	A14643	457	A00190	A14654	901
A00190	A14664	446	A00190	A14667	465
A00190	A14671	260	A00190	A14693	241
A00190	A14726	168	A00190	A14741	260
A00190	A14799	439	A00190	A14928	386
A00190	A14930	477	A00190	A15130	193
A00190	A15241	475	A00190	A15277	263
A00190	A15287	352	A00190	A15289	168
A00190	A15291	446	A00190	A15292	662
A00190	A15294	899	A00190	A15301	435
A00190	A15302	475	A00190	A15310	460
A00190	A15312	490	A00190	A15313	490
A00190	A15314	490	A00190	A15317	486
A00190	A15318	329	A00190	A15322	490
A00190	A15323	281	A00190	A15328	190
A00190	A15331	470	A00190	A15333	406
A00190	A15335	385	A00190	A15336	152
A00190	A15345	327	A00190	A15346	484
A00190	A15348	489	A00190	A15349	491
A00190	A15352	491	A00190	A15357	420
A00190	A15365	246	A00190	A15366	173
A00190	A15376	470	A00190	A15396	173
A00190	A15398	428	A00190	A15399	176
A00190	A15400	423	A00190	A15402	352
A00190	A15404	374	A00190	A15413	479
A00190	A15416	344	A00190	A15417	160
A00190	A15422	235	A00190	A15428	162
A00190	A15429	230	A00190	A15430	489
A00190	A15431	174	A00190	A15432	254
A00190	A15433	191	A00190	A15440	156
A00190	A15445	278	A00190	A15447	475
A00190	A15452	446	A00190	A15454	424
A00190	A15460	195	A00190	A15461	385
A00190	A15463	479	A00190	A15464	385
A00190	A15480	475	A00190	A15484	469
A00190	A15487	470	A00190	A15488	188
A00190	A15489	152	A00190	A15495	486
A00190	A15498	489	A00190	A15515	481
A00190	A15546	369	A00190	A15624	490
A00190	A15630	470	A00190	A15634	899
A00190	A15638	196	A00190	A15641	208
A00190	A15649	255	A00190	A15669	373
A00190	A15673	313	A00190	A15726	352
A00190	A15784	168	A00190	A15795	554
A00190	A15800	188	A00190	A15804	345

A00190	A15813	196	A00190	A15814	176
A00190	A15819	340	A00190	A15824	151
A00190	A15825	183	A00190	A15828	326
A00190	A15843	430	A00190	A15858	290
A00190	A15874	303	A00190	A15884	151
A00190	A15896	334	A00190	A15913	273
A00190	A15932	338	A00190	A15935	345
A00190	A15977	196	A00190	A15996	154
A00190	A16054	245	A00190	A16055	154
A00190	A16081	340	A00190	A16095	484
A00190	A16120	160	A00190	A16121	229
A00190	A16136	249	A00190	A16165	232
A00190	A16199	168	A00190	A16224	383
A00190	A16270	168	A00190	A16271	232
A00190	A16300	489	A00190	A16418	256
A00190	A16419	234	A00190	A16636	489
A00190	A16657	199	A00190	A16739	299
A00190	A16744	314	A00190	A16904	345
A00190	A16916	223	A00190	A16948	232
A00190	A16988	899	A00190	A16989	168
A00190	A17022	168	A00190	A17066	286
A00190	A17136	302	A00190	A17146	240
A00190	A17154	168	A00191	A00192	964
A00191	A00209	899	A00191	A00229	177
A00191	A00247	357	A00191	A00257	224
A00191	A00258	192	A00191	A00268	241
A00191	A00283	162	A00191	A00288	191
A00191	A00298	276	A00191	A00304	201
A00191	A00337	296	A00191	A00340	395
A00191	A00356	557	A00191	A00357	212
A00191	A00358	945	A00191	A00360	491
A00191	A00361	945	A00191	A00362	490
A00191	A00366	245	A00191	A00374	358
A00191	A00433	648	A00191	A00458	157
A00191	A00483	408	A00191	A00531	341
A00191	A00604	430	A00191	A00629	283
A00191	A00630	260	A00191	A00643	207
A00191	A00648	401	A00191	A00653	419
A00191	A00700	274	A00191	A00709	229
A00191	A00783	480	A00191	A00787	473
A00191	A00826	296	A00191	A00839	172
A00191	A00878	180	A00191	A00900	477
A00191	A00905	412	A00191	A00916	491
A00191	A00923	312	A00191	A00980	260
A00191	A00981	165	A00191	A00982	230
A00191	A01012	205	A00191	A01034	257

A00191	A01038	302	A00191	A01056	469
A00191	A01112	402	A00191	A01125	407
A00191	A01127	474	A00191	A01140	401
A00191	A01206	480	A00191	A01216	483
A00191	A01234	512	A00191	A01249	205
A00191	A01252	218	A00191	A01276	294
A00191	A01291	960	A00191	A01299	154
A00191	A01306	407	A00191	A01309	296
A00191	A01322	358	A00191	A01335	443
A00191	A01346	198	A00191	A01399	391
A00191	A01415	236	A00191	A01443	401
A00191	A01446	469	A00191	A01448	372
A00191	A01457	575	A00191	A01458	595
A00191	A01461	528	A00191	A01492	905
A00191	A01494	303	A00191	A01522	485
A00191	A01524	899	A00191	A01542	457
A00191	A01574	490	A00191	A01575	373
A00191	A01578	481	A00191	A01579	204
A00191	A01585	491	A00191	A01588	899
A00191	A01593	899	A00191	A01598	423
A00191	A01601	280	A00191	A01612	491
A00191	A01638	899	A00191	A01718	491
A00191	A01905	203	A00191	A01995	176
A00191	A02110	260	A00191	A02971	174
A00191	A03126	174	A00191	A03332	491
A00191	A03437	63	A00191	A03567	232
A00191	A03569	475	A00191	A03571	249
A00191	A03726	899	A00191	A03752	463
A00191	A03754	183	A00191	A03910	358
A00191	A03913	396	A00191	A03955	484
A00191	A04033	491	A00191	A04449	532
A00191	A04686	152	A00191	A04715	291
A00191	A04754	458	A00191	A04761	491
A00191	A05044	484	A00191	A05288	366
A00191	A05356	152	A00191	A05427	236
A00191	A05443	490	A00191	A05996	491
A00191	A06564	224	A00191	A06588	312
A00191	A06689	196	A00191	A06814	559
A00191	A06965	154	A00191	A07004	183
A00191	A07111	235	A00191	A07149	190
A00191	A07311	338	A00191	A07422	903
A00191	A07603	362	A00191	A07726	353
A00191	A07803	491	A00191	A07959	236
A00191	A08018	417	A00191	A08137	536
A00191	A08359	462	A00191	A08550	344
A00191	A08560	165	A00191	A08571	906

A00191	A08814	165	A00191	A08869	204
A00191	A08878	456	A00191	A08929	296
A00191	A09078	343	A00191	A09609	277
A00191	A10154	460	A00191	A10316	491
A00191	A10430	358	A00191	A10525	366
A00191	A10616	406	A00191	A11190	324
A00191	A11400	419	A00191	A11472	184
A00191	A11487	218	A00191	A11496	157
A00191	A11548	358	A00191	A11612	491
A00191	A11735	428	A00191	A11914	299
A00191	A11983	491	A00191	A12073	486
A00191	A12215	154	A00191	A12284	365
A00191	A12506	266	A00191	A12508	154
A00191	A12606	263	A00191	A12653	481
A00191	A12658	212	A00191	A12826	177
A00191	A12858	347	A00191	A12897	440
A00191	A13241	945	A00191	A13680	215
A00191	A13707	491	A00191	A13787	215
A00191	A13804	154	A00191	A13806	490
A00191	A13811	177	A00191	A13949	296
A00191	A13989	958	A00191	A13992	283
A00191	A14148	899	A00191	A14202	413
A00191	A14204	260	A00191	A14246	899
A00191	A14247	477	A00191	A14270	154
A00191	A14279	160	A00191	A14393	176
A00191	A14410	347	A00191	A14443	908
A00191	A14569	422	A00191	A14635	490
A00191	A14917	281	A00191	A15247	486
A00191	A15255	490	A00191	A15292	241
A00191	A15297	899	A00191	A15324	899
A00191	A15353	899	A00191	A15359	899
A00191	A15541	163	A00191	A15547	899
A00191	A15614	899	A00191	A15660	358
A00191	A15682	160	A00191	A15709	266
A00191	A15731	311	A00191	A15931	313
A00191	A16157	357	A00191	A16565	188
A00191	A16567	157	A00191	A16587	271
A00191	A16611	276	A00191	A16664	249
A00191	A16693	201	A00191	A16695	208
A00191	A16774	272	A00191	A16788	154
A00191	A16815	193	A00191	A16829	360
A00191	A16847	358	A00192	A00207	899
A00192	A00209	899	A00192	A00258	486
A00192	A00268	324	A00192	A00303	152
A00192	A00335	157	A00192	A00356	491
A00192	A00357	435	A00192	A00358	491

A00192	A00360	491	A00192	A00361	491
A00192	A00362	468	A00192	A00634	488
A00192	A00900	374	A00192	A00916	306
A00192	A00923	341	A00192	A01056	469
A00192	A01098	456	A00192	A01127	340
A00192	A01206	438	A00192	A01234	240
A00192	A01249	228	A00192	A01291	945
A00192	A01320	177	A00192	A01425	380
A00192	A01457	313	A00192	A01470	177
A00192	A01492	422	A00192	A01524	989
A00192	A01593	899	A00192	A01612	374
A00192	A01718	475	A00192	A02068	156
A00192	A02185	160	A00192	A02265	312
A00192	A02293	204	A00192	A03126	160
A00192	A03332	491	A00192	A03334	299
A00192	A03437	168	A00192	A03571	262
A00192	A03580	205	A00192	A03726	899
A00192	A03754	393	A00192	A03913	183
A00192	A03919	199	A00192	A04033	334
A00192	A04099	168	A00192	A04754	483
A00192	A04761	491	A00192	A05044	396
A00192	A05443	486	A00192	A05996	491
A00192	A06279	294	A00192	A06814	218
A00192	A07422	922	A00192	A08137	398
A00192	A09494	269	A00192	A10234	305
A00192	A11190	490	A00192	A11400	228
A00192	A11612	491	A00192	A11808	352
A00192	A12653	451	A00192	A12667	170
A00192	A13241	430	A00192	A13424	174
A00192	A13707	257	A00192	A13989	490
A00192	A13992	240	A00192	A15247	470
A00192	A15255	452	A00192	A15432	176
A00192	A15498	243	A00193	A00202	80
A00193	A00210	363	A00193	A00212	384
A00193	A00216	168	A00193	A00219	208
A00193	A00312	218	A00193	A00322	328
A00193	A00338	490	A00193	A00377	674
A00193	A00379	480	A00193	A00387	230
A00193	A00395	490	A00193	A00420	293
A00193	A00438	462	A00193	A00446	307
A00193	A00449	418	A00193	A00469	183
A00193	A00472	484	A00193	A00473	238
A00193	A00485	461	A00193	A00531	425
A00193	A00576	263	A00193	A00600	185
A00193	A00638	457	A00193	A00802	337
A00193	A00849	255	A00193	A00899	208

A00193	A00906	434	A00193	A00962	258
A00193	A01018	490	A00193	A01022	274
A00193	A01139	307	A00193	A01156	730
A00193	A01161	489	A00193	A01169	249
A00193	A01215	267	A00193	A01222	204
A00193	A01225	488	A00193	A01227	409
A00193	A01258	279	A00193	A01278	165
A00193	A01297	235	A00193	A01320	185
A00193	A01344	171	A00193	A01485	296
A00193	A01530	180	A00193	A01556	208
A00193	A01582	486	A00193	A01585	286
A00193	A01627	224	A00193	A01629	247
A00193	A01713	273	A00193	A01901	213
A00193	A02061	151	A00193	A02077	180
A00193	A02091	299	A00193	A02188	180
A00193	A02191	152	A00193	A02192	319
A00193	A02215	412	A00193	A02221	263
A00193	A02261	191	A00193	A02283	245
A00193	A02301	372	A00193	A02303	213
A00193	A02392	273	A00193	A02435	163
A00193	A02477	465	A00193	A02484	201
A00193	A02501	443	A00193	A02504	462
A00193	A02521	443	A00193	A02548	193
A00193	A02583	219	A00193	A02584	468
A00193	A02592	252	A00193	A02594	408
A00193	A02595	319	A00193	A02598	429
A00193	A02605	173	A00193	A02656	285
A00193	A02666	201	A00193	A02672	374
A00193	A02747	241	A00193	A02758	391
A00193	A02760	188	A00193	A02774	165
A00193	A02785	217	A00193	A02874	205
A00193	A03050	213	A00193	A03094	201
A00193	A03115	307	A00193	A03211	226
A00193	A03313	191	A00193	A03334	170
A00193	A03351	177	A00193	A03413	157
A00193	A03642	408	A00193	A03795	463
A00193	A03860	173	A00193	A03992	414
A00193	A04172	291	A00193	A04177	308
A00193	A04179	363	A00193	A04222	317
A00193	A04228	481	A00193	A04231	299
A00193	A04234	291	A00193	A04256	274
A00193	A04282	174	A00193	A04337	399
A00193	A04373	256	A00193	A04416	260
A00193	A04423	446	A00193	A04429	229
A00193	A04436	285	A00193	A04485	419
A00193	A04491	398	A00193	A04519	367

A00193	A04532	174	A00193	A04555	249
A00193	A04647	216	A00193	A04791	168
A00193	A04816	299	A00193	A04879	432
A00193	A05139	319	A00193	A05227	251
A00193	A05356	475	A00193	A05538	291
A00193	A05549	319	A00193	A05721	177
A00193	A05763	319	A00193	A05901	279
A00193	A06038	216	A00193	A06057	291
A00193	A06141	207	A00193	A06284	285
A00193	A06299	195	A00193	A06379	213
A00193	A06439	390	A00193	A06546	170
A00193	A06655	260	A00193	A06696	187
A00193	A07088	254	A00193	A07136	260
A00193	A07165	162	A00193	A07239	488
A00193	A07281	386	A00193	A07376	216
A00193	A07407	308	A00193	A07438	213
A00193	A07442	489	A00193	A07645	319
A00193	A07674	290	A00193	A07737	204
A00193	A07739	279	A00193	A08172	236
A00193	A09008	184	A00193	A09088	358
A00193	A09494	273	A00193	A10228	210
A00193	A10458	368	A00193	A10646	335
A00193	A10651	159	A00193	A10652	332
A00193	A10687	249	A00193	A10696	369
A00193	A10781	216	A00193	A11021	273
A00193	A11606	263	A00193	A11722	319
A00193	A12371	205	A00193	A12453	358
A00193	A13065	335	A00193	A13965	391
A00193	A14381	429	A00193	A14413	249
A00193	A14449	247	A00193	A15195	308
A00193	A15498	419	A00193	A15698	152
A00193	A15917	428	A00193	A16112	252
A00193	A17006	213	A00193	A17078	210
A00194	A00220	317	A00194	A00221	243
A00194	A00222	943	A00194	A00223	347
A00194	A00763	185	A00194	A01056	254
A00194	A01492	157	A00194	A01730	184
A00194	A07422	899	A00194	A08012	404
A00194	A13989	63	A00195	A00209	899
A00195	A00236	491	A00195	A00252	945
A00195	A00253	945	A00195	A00293	302
A00195	A00420	174	A00195	A00652	468
A00195	A01045	419	A00195	A01405	171
A00195	A01550	420	A00195	A01551	199
A00195	A01664	200	A00195	A01692	226
A00195	A02325	235	A00195	A02647	348

A00195	A03472	218	A00195	A03596	213
A00195	A04209	160	A00195	A04342	208
A00195	A05811	294	A00195	A08815	191
A00195	A08881	204	A00195	A10233	279
A00195	A10311	363	A00195	A11412	167
A00195	A11921	491	A00195	A13255	339
A00195	A14443	989	A00195	A16080	944
A00195	A16764	213	A00196	A00202	347
A00196	A00204	486	A00196	A00209	913
A00196	A00212	432	A00196	A00216	441
A00196	A00218	491	A00196	A00219	491
A00196	A00227	430	A00196	A00264	491
A00196	A00294	483	A00196	A00312	441
A00196	A00338	412	A00196	A00449	321
A00196	A00472	491	A00196	A00485	401
A00196	A00556	367	A00196	A00574	246
A00196	A00616	486	A00196	A00656	491
A00196	A00662	217	A00196	A00663	159
A00196	A00688	454	A00196	A00815	484
A00196	A00921	252	A00196	A00930	414
A00196	A01048	235	A00196	A01058	180
A00196	A01125	317	A00196	A01138	491
A00196	A01161	159	A00196	A01211	188
A00196	A01325	484	A00196	A01406	441
A00196	A01477	168	A00196	A01481	321
A00196	A01483	174	A00196	A01526	491
A00196	A01527	902	A00196	A01556	491
A00196	A01581	154	A00196	A01635	462
A00196	A01640	241	A00196	A01658	422
A00196	A01663	355	A00196	A01668	468
A00196	A01676	486	A00196	A01726	472
A00196	A01727	221	A00196	A01741	480
A00196	A01743	485	A00196	A01748	152
A00196	A01758	328	A00196	A01891	177
A00196	A01894	424	A00196	A01946	193
A00196	A01982	491	A00196	A02000	215
A00196	A02006	318	A00196	A02010	465
A00196	A02019	297	A00196	A02028	221
A00196	A02044	223	A00196	A02046	486
A00196	A02048	491	A00196	A02049	486
A00196	A02054	299	A00196	A02065	208
A00196	A02074	171	A00196	A02075	228
A00196	A02108	430	A00196	A02111	152
A00196	A02126	281	A00196	A02143	407
A00196	A02145	441	A00196	A02146	491
A00196	A02147	434	A00196	A02148	491

A00196	A02149	188	A00196	A02176	193
A00196	A02177	212	A00196	A02180	491
A00196	A02183	338	A00196	A02186	491
A00196	A02187	418	A00196	A02188	389
A00196	A02205	489	A00196	A02213	213
A00196	A02215	491	A00196	A02222	249
A00196	A02223	477	A00196	A02224	176
A00196	A02233	213	A00196	A02234	435
A00196	A02237	404	A00196	A02238	490
A00196	A02245	167	A00196	A02278	229
A00196	A02291	491	A00196	A02292	184
A00196	A02295	402	A00196	A02298	491
A00196	A02300	477	A00196	A02305	281
A00196	A02306	293	A00196	A02307	396
A00196	A02309	157	A00196	A02328	291
A00196	A02338	256	A00196	A02352	347
A00196	A02385	313	A00196	A02390	296
A00196	A02393	213	A00196	A02400	286
A00196	A02401	168	A00196	A02409	154
A00196	A02412	416	A00196	A02416	452
A00196	A02423	213	A00196	A02450	286
A00196	A02465	378	A00196	A02468	286
A00196	A02478	199	A00196	A02490	491
A00196	A02491	491	A00196	A02536	177
A00196	A02545	317	A00196	A02559	360
A00196	A02582	385	A00196	A02587	345
A00196	A02605	391	A00196	A02639	162
A00196	A02643	213	A00196	A02668	491
A00196	A02669	212	A00196	A02691	363
A00196	A02697	452	A00196	A02698	217
A00196	A02704	171	A00196	A02756	488
A00196	A02771	443	A00196	A02774	420
A00196	A02785	486	A00196	A02832	263
A00196	A02836	299	A00196	A02859	472
A00196	A02869	152	A00196	A02874	480
A00196	A02877	411	A00196	A02922	475
A00196	A02938	160	A00196	A02945	152
A00196	A02946	270	A00196	A02948	177
A00196	A02951	488	A00196	A02961	185
A00196	A02977	229	A00196	A02982	152
A00196	A03021	160	A00196	A03027	401
A00196	A03053	285	A00196	A03067	330
A00196	A03069	473	A00196	A03070	486
A00196	A03073	263	A00196	A03077	168
A00196	A03080	491	A00196	A03082	404
A00196	A03084	297	A00196	A03099	341

A00196	A03107	358	A00196	A03141	491
A00196	A03145	154	A00196	A03171	422
A00196	A03172	217	A00196	A03178	456
A00196	A03184	208	A00196	A03186	491
A00196	A03275	408	A00196	A03310	226
A00196	A03314	226	A00196	A03346	490
A00196	A03347	491	A00196	A03348	235
A00196	A03351	480	A00196	A03353	489
A00196	A03358	394	A00196	A03361	490
A00196	A03369	491	A00196	A03370	491
A00196	A03371	491	A00196	A03373	491
A00196	A03374	251	A00196	A03378	483
A00196	A03379	491	A00196	A03390	167
A00196	A03391	157	A00196	A03392	302
A00196	A03394	302	A00196	A03396	168
A00196	A03397	433	A00196	A03402	488
A00196	A03403	229	A00196	A03408	483
A00196	A03414	490	A00196	A03479	271
A00196	A03532	187	A00196	A03634	479
A00196	A03639	424	A00196	A03661	171
A00196	A03719	201	A00196	A03722	245
A00196	A03733	457	A00196	A03744	313
A00196	A03755	274	A00196	A03773	360
A00196	A03824	232	A00196	A03906	491
A00196	A03914	469	A00196	A03915	375
A00196	A03936	357	A00196	A03944	213
A00196	A03976	430	A00196	A04048	193
A00196	A04051	491	A00196	A04070	319
A00196	A04080	307	A00196	A04086	157
A00196	A04092	306	A00196	A04152	407
A00196	A04159	438	A00196	A04160	168
A00196	A04193	468	A00196	A04260	213
A00196	A04271	159	A00196	A04278	396
A00196	A04327	340	A00196	A04333	176
A00196	A04338	235	A00196	A04342	435
A00196	A04344	286	A00196	A04351	193
A00196	A04353	235	A00196	A04366	183
A00196	A04391	243	A00196	A04409	333
A00196	A04437	288	A00196	A04449	188
A00196	A04456	235	A00196	A04457	199
A00196	A04492	179	A00196	A04607	337
A00196	A04608	452	A00196	A04609	409
A00196	A04614	318	A00196	A04618	314
A00196	A04672	213	A00196	A04742	177
A00196	A04763	386	A00196	A04830	408
A00196	A04875	263	A00196	A04882	235

A00196	A04923	213	A00196	A04989	479
A00196	A04992	168	A00196	A05113	457
A00196	A05117	341	A00196	A05131	302
A00196	A05136	430	A00196	A05264	165
A00196	A05309	303	A00196	A05349	463
A00196	A05356	462	A00196	A05373	187
A00196	A05427	404	A00196	A05469	457
A00196	A05494	343	A00196	A05521	270
A00196	A05533	347	A00196	A05543	213
A00196	A05578	355	A00196	A05612	154
A00196	A05731	317	A00196	A05858	213
A00196	A05901	160	A00196	A05920	168
A00196	A05999	302	A00196	A06033	213
A00196	A06346	207	A00196	A06358	176
A00196	A06415	274	A00196	A06463	191
A00196	A06473	191	A00196	A06607	447
A00196	A06642	451	A00196	A06681	235
A00196	A06732	235	A00196	A06782	177
A00196	A06847	200	A00196	A06935	213
A00196	A07049	270	A00196	A07141	380
A00196	A07163	445	A00196	A07165	205
A00196	A07200	272	A00196	A07217	398
A00196	A07218	491	A00196	A07263	270
A00196	A07273	281	A00196	A07420	199
A00196	A07440	226	A00196	A07620	310
A00196	A07754	218	A00196	A07765	213
A00196	A07771	167	A00196	A08044	199
A00196	A08192	202	A00196	A08200	330
A00196	A08222	235	A00196	A08288	199
A00196	A08300	168	A00196	A08404	271
A00196	A08632	154	A00196	A08707	449
A00196	A08755	187	A00196	A08861	243
A00196	A08926	385	A00196	A09028	152
A00196	A09084	154	A00196	A09090	168
A00196	A09145	167	A00196	A09200	324
A00196	A09266	344	A00196	A09480	243
A00196	A09939	238	A00196	A09983	251
A00196	A10020	213	A00196	A10074	235
A00196	A10080	199	A00196	A10148	154
A00196	A10486	235	A00196	A10625	232
A00196	A10639	154	A00196	A10698	199
A00196	A10943	154	A00196	A11101	159
A00196	A11231	270	A00196	A11313	368
A00196	A11334	157	A00196	A11370	235
A00196	A11371	235	A00196	A11748	154
A00196	A11897	314	A00196	A12179	310

A00196	A12183	270	A00196	A12459	177
A00196	A12605	177	A00196	A12674	168
A00196	A12824	310	A00196	A12897	483
A00196	A12927	274	A00196	A12949	177
A00196	A13063	177	A00196	A13734	456
A00196	A13879	330	A00196	A13886	207
A00196	A13926	193	A00196	A14047	199
A00196	A14194	328	A00196	A14198	296
A00196	A14399	288	A00196	A14423	163
A00196	A14430	386	A00196	A14443	276
A00196	A14470	177	A00196	A15137	235
A00196	A15282	368	A00196	A15356	180
A00196	A15649	361	A00196	A15809	462
A00196	A15914	270	A00196	A16051	260
A00196	A16636	491	A00196	A16862	177
A00197	A00245	179	A00197	A00705	243
A00197	A01060	324	A00197	A01173	303
A00197	A01252	324	A00197	A01352	172
A00197	A01610	154	A00197	A01673	324
A00197	A02859	180	A00197	A02983	185
A00197	A03748	462	A00197	A03851	191
A00197	A03910	151	A00197	A06941	324
A00197	A08484	324	A00197	A08785	324
A00197	A09734	368	A00197	A11279	384
A00197	A11287	324	A00197	A11542	445
A00197	A12289	401	A00197	A13222	324
A00197	A13987	486	A00197	A14010	368
A00197	A14122	218	A00197	A14182	307
A00197	A14258	324	A00197	A14378	319
A00197	A14392	236	A00197	A14408	380
A00197	A14511	276	A00197	A14661	325
A00197	A14711	307	A00197	A14844	324
A00197	A14877	324	A00197	A15292	369
A00197	A15301	353	A00197	A15312	377
A00197	A15313	432	A00197	A15352	450
A00197	A15366	411	A00197	A15396	299
A00197	A15404	347	A00197	A15413	243
A00197	A15419	236	A00197	A15432	355
A00197	A15435	328	A00197	A15447	230
A00197	A15451	337	A00197	A15452	205
A00197	A15462	241	A00197	A15463	369
A00197	A15495	173	A00197	A15513	329
A00197	A15669	278	A00197	A15804	357
A00197	A15833	372	A00197	A17061	324
A00198	A00208	196	A00198	A00210	581
A00198	A00224	907	A00198	A00228	224

A00198	A00230	899	A00198	A00239	616
A00198	A00240	616	A00198	A00241	616
A00198	A00242	489	A00198	A00243	615
A00198	A00244	467	A00198	A00261	616
A00198	A00263	249	A00198	A00330	363
A00198	A00451	260	A00198	A00472	443
A00198	A00625	319	A00198	A00865	152
A00198	A00931	417	A00198	A01169	443
A00198	A01198	899	A00198	A01512	899
A00198	A01524	407	A00198	A01527	995
A00198	A01558	899	A00198	A01561	899
A00198	A01574	201	A00198	A01588	899
A00198	A01596	616	A00198	A01615	989
A00198	A01623	443	A00198	A01718	296
A00198	A01844	899	A00198	A01920	614
A00198	A01974	491	A00198	A02674	188
A00198	A02942	899	A00198	A02968	899
A00198	A03314	218	A00198	A03512	246
A00198	A03536	481	A00198	A03654	159
A00198	A03762	151	A00198	A03832	460
A00198	A04155	199	A00198	A04226	256
A00198	A04449	335	A00198	A05026	168
A00198	A05155	443	A00198	A05431	561
A00198	A06191	396	A00198	A06379	152
A00198	A07004	243	A00198	A07242	281
A00198	A07311	899	A00198	A07422	899
A00198	A08118	598	A00198	A09596	205
A00198	A10580	204	A00198	A12204	232
A00198	A12365	485	A00198	A13250	291
A00198	A13369	462	A00198	A14072	436
A00198	A14153	321	A00198	A14202	451
A00198	A14204	491	A00198	A14299	616
A00198	A14444	903	A00198	A14704	394
A00198	A14705	157	A00198	A14777	243
A00198	A15347	348	A00198	A15665	232
A00198	A15712	152	A00198	A15726	899
A00198	A16764	456	A00199	A00209	899
A00199	A00371	179	A00199	A00382	372
A00199	A00430	363	A00199	A00444	372
A00199	A00489	372	A00199	A00970	454
A00199	A01115	402	A00199	A01119	319
A00199	A01150	176	A00199	A01539	372
A00199	A01684	191	A00199	A02192	207
A00199	A02352	154	A00199	A03446	372
A00199	A03747	277	A00199	A04191	340
A00199	A04192	301	A00199	A05168	218

A00199	A05995	207	A00199	A06084	217
A00199	A07247	198	A00199	A08102	174
A00199	A08524	408	A00199	A08546	372
A00199	A08570	234	A00199	A09210	218
A00199	A09423	183	A00199	A10519	404
A00199	A10532	730	A00199	A10589	417
A00199	A11240	583	A00199	A12818	334
A00199	A12839	314	A00199	A14132	218
A00199	A14482	152	A00199	A14709	179
A00199	A15698	502	A00199	A16690	213
A00199	A17085	177	A00200	A00201	945
A00200	A00209	941	A00200	A00212	274
A00200	A00218	183	A00200	A00219	408
A00200	A00224	932	A00200	A00246	899
A00200	A00256	491	A00200	A00258	408
A00200	A00264	944	A00200	A00382	305
A00200	A00455	473	A00200	A00472	273
A00200	A00634	318	A00200	A00643	162
A00200	A00763	452	A00200	A00822	462
A00200	A00887	899	A00200	A01088	942
A00200	A01274	160	A00200	A01299	427
A00200	A01385	443	A00200	A01419	462
A00200	A01524	899	A00200	A01527	899
A00200	A01558	899	A00200	A01561	899
A00200	A01571	374	A00200	A01576	160
A00200	A01588	899	A00200	A01600	470
A00200	A01632	428	A00200	A01733	238
A00200	A01741	177	A00200	A01743	355
A00200	A01747	156	A00200	A01767	389
A00200	A01844	899	A00200	A01905	937
A00200	A01976	318	A00200	A02111	405
A00200	A02143	212	A00200	A02220	168
A00200	A02298	208	A00200	A02411	899
A00200	A02427	238	A00200	A02470	291
A00200	A02483	288	A00200	A02490	301
A00200	A02501	198	A00200	A02564	465
A00200	A02697	200	A00200	A02698	398
A00200	A02711	246	A00200	A02738	213
A00200	A02835	316	A00200	A02878	462
A00200	A02968	899	A00200	A03080	185
A00200	A03105	236	A00200	A03107	899
A00200	A03121	446	A00200	A03211	435
A00200	A03221	188	A00200	A03304	154
A00200	A03451	215	A00200	A03591	579
A00200	A03661	240	A00200	A03928	215
A00200	A03961	151	A00200	A03968	326

A00200	A04044	271	A00200	A04047	301
A00200	A04077	188	A00200	A04159	188
A00200	A04193	191	A00200	A04250	302
A00200	A04289	321	A00200	A04309	229
A00200	A04329	205	A00200	A04382	188
A00200	A04507	236	A00200	A04525	901
A00200	A04527	899	A00200	A04535	229
A00200	A04543	179	A00200	A04545	162
A00200	A04928	335	A00200	A05247	400
A00200	A05288	705	A00200	A05346	177
A00200	A05399	200	A00200	A05432	278
A00200	A05521	271	A00200	A05621	199
A00200	A05660	162	A00200	A05701	154
A00200	A05795	425	A00200	A05869	210
A00200	A06151	191	A00200	A06153	345
A00200	A06154	215	A00200	A06181	188
A00200	A06346	463	A00200	A06581	404
A00200	A06588	462	A00200	A06597	414
A00200	A06607	157	A00200	A06608	945
A00200	A06609	899	A00200	A06628	436
A00200	A06650	491	A00200	A06711	218
A00200	A06712	200	A00200	A06841	168
A00200	A06885	271	A00200	A06903	195
A00200	A06998	200	A00200	A07218	341
A00200	A07311	899	A00200	A07339	200
A00200	A07676	271	A00200	A07704	307
A00200	A07705	355	A00200	A07706	308
A00200	A07828	215	A00200	A08128	271
A00200	A08203	247	A00200	A08224	168
A00200	A08292	281	A00200	A08611	271
A00200	A08629	215	A00200	A08679	216
A00200	A08738	427	A00200	A08869	486
A00200	A09391	899	A00200	A09409	200
A00200	A09456	271	A00200	A09470	408
A00200	A09518	188	A00200	A09705	340
A00200	A10174	899	A00200	A10218	899
A00200	A10290	271	A00200	A10449	162
A00200	A10450	399	A00200	A10900	236
A00200	A11056	236	A00200	A11300	446
A00200	A11310	208	A00200	A11320	227
A00200	A11510	271	A00200	A11621	207
A00200	A11970	271	A00200	A11983	294
A00200	A12134	271	A00200	A12135	271
A00200	A12298	177	A00200	A12373	945
A00200	A12388	271	A00200	A12552	321
A00200	A12797	271	A00200	A12803	271

A00200	A12878	188	A00200	A12897	199
A00200	A12898	215	A00200	A12984	236
A00200	A13173	200	A00200	A14198	208
A00200	A14330	201	A00200	A14444	899
A00200	A14605	151	A00200	A14660	466
A00200	A14883	399	A00200	A15297	319
A00200	A15570	188	A00200	A15847	177
A00200	A15981	196	A00200	A16140	271
A00200	A16168	271	A00200	A16169	271
A00200	A16172	271	A00200	A16175	271
A00200	A16178	271	A00200	A16179	271
A00200	A16184	271	A00200	A16185	271
A00200	A16186	271	A00200	A16636	441
A00200	A16648	188	A00201	A00209	486
A00201	A00256	489	A00201	A00263	380
A00201	A00519	491	A00201	A00561	486
A00201	A00605	457	A00201	A00621	274
A00201	A00653	616	A00201	A00658	151
A00201	A00711	491	A00201	A00724	708
A00201	A00736	703	A00201	A00769	616
A00201	A00783	488	A00201	A00785	491
A00201	A00810	414	A00201	A00824	491
A00201	A00841	708	A00201	A00865	469
A00201	A00871	491	A00201	A00909	708
A00201	A00922	616	A00201	A00927	400
A00201	A00934	398	A00201	A00953	228
A00201	A00983	491	A00201	A00986	488
A00201	A01006	708	A00201	A01017	341
A00201	A01027	616	A00201	A01069	491
A00201	A01088	470	A00201	A01101	469
A00201	A01150	491	A00201	A01208	393
A00201	A01221	438	A00201	A01232	616
A00201	A01242	335	A00201	A01293	708
A00201	A01305	157	A00201	A01311	491
A00201	A01346	229	A00201	A01431	291
A00201	A01439	353	A00201	A01458	228
A00201	A01492	491	A00201	A01512	899
A00201	A01538	213	A00201	A01572	171
A00201	A01577	491	A00201	A01596	544
A00201	A01598	414	A00201	A01613	435
A00201	A01727	430	A00201	A01748	490
A00201	A01764	232	A00201	A01765	176
A00201	A01775	434	A00201	A01778	187
A00201	A01779	348	A00201	A01780	296
A00201	A01844	899	A00201	A01894	644
A00201	A02235	299	A00201	A02321	218

A00201	A02479	156	A00201	A03121	489
A00201	A03169	210	A00201	A03291	258
A00201	A03438	477	A00201	A03454	490
A00201	A03557	208	A00201	A03608	477
A00201	A03677	323	A00201	A03809	291
A00201	A03901	210	A00201	A04885	303
A00201	A04895	283	A00201	A05009	473
A00201	A05227	604	A00201	A05288	491
A00201	A05318	299	A00201	A05432	446
A00201	A05702	249	A00201	A05742	486
A00201	A05795	475	A00201	A05853	160
A00201	A06287	484	A00201	A06543	488
A00201	A06608	937	A00201	A06609	201
A00201	A06637	491	A00201	A06650	491
A00201	A06783	156	A00201	A06795	475
A00201	A07339	368	A00201	A07360	380
A00201	A07744	491	A00201	A07881	616
A00201	A07911	708	A00201	A08098	491
A00201	A08178	486	A00201	A08228	616
A00201	A08491	480	A00201	A08548	615
A00201	A08763	480	A00201	A09210	216
A00201	A10580	454	A00201	A10851	163
A00201	A11296	191	A00201	A11300	353
A00201	A11326	180	A00201	A11381	317
A00201	A11387	272	A00201	A11555	246
A00201	A11560	288	A00201	A11907	223
A00201	A12373	945	A00201	A13773	185
A00201	A13989	279	A00201	A14083	491
A00201	A14330	466	A00201	A14443	263
A00201	A14605	254	A00201	A14633	290
A00201	A14699	616	A00201	A14778	616
A00201	A15375	463	A00201	A15660	490
A00201	A15684	491	A00201	A15746	480
A00201	A15937	457	A00201	A16048	458
A00201	A16081	433	A00201	A16104	185
A00201	A16912	213	A00202	A00209	900
A00202	A00219	177	A00202	A00259	259
A00202	A00264	351	A00202	A00265	483
A00202	A00338	439	A00202	A00420	303
A00202	A00485	174	A00202	A00524	188
A00202	A00571	177	A00202	A00849	269
A00202	A00878	406	A00202	A01022	420
A00202	A01026	152	A00202	A01157	165
A00202	A01161	483	A00202	A01481	185
A00202	A01524	927	A00202	A01547	317
A00202	A01566	265	A00202	A01582	486

A00202	A01629	258	A00202	A01632	419
A00202	A01730	185	A00202	A01741	340
A00202	A01743	243	A00202	A01755	408
A00202	A01774	302	A00202	A01877	399
A00202	A01896	176	A00202	A01916	188
A00202	A01931	470	A00202	A01936	193
A00202	A01941	329	A00202	A01957	413
A00202	A02033	279	A00202	A02044	160
A00202	A02045	362	A00202	A02048	307
A00202	A02049	319	A00202	A02068	168
A00202	A02072	429	A00202	A02091	306
A00202	A02110	251	A00202	A02144	160
A00202	A02170	176	A00202	A02174	184
A00202	A02179	265	A00202	A02192	479
A00202	A02221	171	A00202	A02250	159
A00202	A02278	296	A00202	A02301	157
A00202	A02303	391	A00202	A02310	436
A00202	A02318	417	A00202	A02376	228
A00202	A02392	279	A00202	A02429	184
A00202	A02459	160	A00202	A02466	457
A00202	A02467	374	A00202	A02471	249
A00202	A02477	489	A00202	A02495	302
A00202	A02501	491	A00202	A02505	262
A00202	A02525	163	A00202	A02531	202
A00202	A02532	422	A00202	A02568	294
A00202	A02583	355	A00202	A02621	369
A00202	A02666	208	A00202	A02672	423
A00202	A02758	185	A00202	A02760	404
A00202	A02773	279	A00202	A02787	185
A00202	A02814	251	A00202	A02816	301
A00202	A02817	154	A00202	A02830	333
A00202	A02837	218	A00202	A02874	296
A00202	A02924	466	A00202	A02927	160
A00202	A03076	185	A00202	A03080	424
A00202	A03094	340	A00202	A03112	414
A00202	A03211	323	A00202	A03276	340
A00202	A03377	340	A00202	A03576	385
A00202	A03642	314	A00202	A03798	263
A00202	A03857	326	A00202	A03904	185
A00202	A04087	221	A00202	A04172	297
A00202	A04229	343	A00202	A04256	152
A00202	A04333	356	A00202	A04418	391
A00202	A04423	219	A00202	A04477	430
A00202	A04479	306	A00202	A04491	195
A00202	A04493	152	A00202	A04548	165
A00202	A04710	245	A00202	A04763	228

A00202	A04816	306	A00202	A04942	350
A00202	A05184	198	A00202	A05259	221
A00202	A05260	416	A00202	A05467	208
A00202	A05472	267	A00202	A05736	229
A00202	A06126	340	A00202	A06400	429
A00202	A06620	171	A00202	A06648	335
A00202	A06654	185	A00202	A06722	418
A00202	A07218	479	A00202	A07442	395
A00202	A08694	407	A00202	A08718	472
A00202	A09149	262	A00202	A09243	251
A00202	A09340	163	A00202	A09394	212
A00202	A09464	185	A00202	A09507	344
A00202	A09573	205	A00202	A09777	267
A00202	A09978	201	A00202	A10033	326
A00202	A10071	314	A00202	A10156	363
A00202	A10731	418	A00202	A11211	274
A00202	A11911	358	A00202	A12654	241
A00202	A13397	165	A00202	A14060	195
A00202	A14381	234	A00202	A14410	168
A00202	A14498	486	A00202	A14521	151
A00202	A14589	481	A00202	A14643	439
A00202	A14648	207	A00202	A14886	188
A00202	A15422	173	A00202	A15911	160
A00202	A15917	433	A00202	A16257	374
A00202	A16739	185	A00202	A16744	193
A00203	A00209	899	A00203	A00223	316
A00203	A00302	259	A00203	A00501	360
A00203	A00518	151	A00203	A00559	152
A00203	A00605	483	A00203	A00626	183
A00203	A00648	491	A00203	A00713	160
A00203	A00835	218	A00203	A00839	188
A00203	A00916	412	A00203	A00932	247
A00203	A00970	429	A00203	A01085	470
A00203	A01112	486	A00203	A01113	260
A00203	A01140	469	A00203	A01166	252
A00203	A01206	445	A00203	A01224	185
A00203	A01234	400	A00203	A01291	480
A00203	A01425	468	A00203	A01433	162
A00203	A01468	465	A00203	A01470	473
A00203	A01487	260	A00203	A01488	396
A00203	A01491	63	A00203	A01492	944
A00203	A01505	159	A00203	A01512	899
A00203	A01527	899	A00203	A01541	467
A00203	A01601	899	A00203	A01614	401
A00203	A01649	490	A00203	A01684	170
A00203	A01707	477	A00203	A01730	899

A00203	A01740	162	A00203	A02265	218
A00203	A02638	157	A00203	A03298	208
A00203	A03446	201	A00203	A03461	170
A00203	A03738	283	A00203	A06340	372
A00203	A06466	483	A00203	A06897	196
A00203	A07247	176	A00203	A07311	260
A00203	A07422	899	A00203	A07451	195
A00203	A07677	210	A00203	A08137	170
A00203	A08357	332	A00203	A08571	318
A00203	A09577	218	A00203	A09655	162
A00203	A10365	154	A00203	A10532	456
A00203	A11379	165	A00203	A11745	205
A00203	A11942	210	A00203	A11983	899
A00203	A12020	249	A00203	A12167	180
A00203	A12328	193	A00203	A12329	201
A00203	A13340	363	A00203	A13944	191
A00203	A13989	945	A00203	A14006	413
A00203	A14055	899	A00203	A14151	205
A00203	A14216	485	A00203	A14467	157
A00203	A14506	899	A00203	A15146	323
A00203	A15391	171	A00203	A15660	154
A00203	A15777	263	A00203	A16060	170
A00203	A16092	245	A00203	A16463	154
A00204	A00209	899	A00204	A00210	346
A00204	A00212	299	A00204	A00214	63
A00204	A00215	224	A00204	A00216	477
A00204	A00218	489	A00204	A00219	490
A00204	A00220	592	A00204	A00224	945
A00204	A00232	481	A00204	A00233	491
A00204	A00264	491	A00204	A00311	201
A00204	A00312	235	A00204	A00316	401
A00204	A00351	246	A00204	A00374	375
A00204	A00406	407	A00204	A00438	317
A00204	A00448	458	A00204	A00449	452
A00204	A00459	235	A00204	A00472	249
A00204	A00473	302	A00204	A00531	172
A00204	A00556	386	A00204	A00557	380
A00204	A00571	345	A00204	A00574	224
A00204	A00641	262	A00204	A00642	210
A00204	A00658	274	A00204	A00659	188
A00204	A00660	171	A00204	A00662	470
A00204	A00663	369	A00204	A00674	218
A00204	A00696	180	A00204	A00732	318
A00204	A00736	467	A00204	A00781	391
A00204	A00841	469	A00204	A00864	199
A00204	A00868	461	A00204	A00871	339

A00204	A00904	174	A00204	A00909	355
A00204	A00914	475	A00204	A00926	361
A00204	A00939	218	A00204	A01009	162
A00204	A01011	470	A00204	A01020	420
A00204	A01077	229	A00204	A01087	185
A00204	A01116	317	A00204	A01137	249
A00204	A01184	358	A00204	A01201	367
A00204	A01203	157	A00204	A01215	373
A00204	A01234	423	A00204	A01291	199
A00204	A01307	311	A00204	A01353	451
A00204	A01400	310	A00204	A01473	208
A00204	A01485	190	A00204	A01527	899
A00204	A01551	384	A00204	A01554	481
A00204	A01572	199	A00204	A01573	188
A00204	A01590	484	A00204	A01603	160
A00204	A01604	455	A00204	A01612	199
A00204	A01615	899	A00204	A01626	183
A00204	A01667	177	A00204	A01668	491
A00204	A01676	290	A00204	A01700	474
A00204	A01704	491	A00204	A01713	416
A00204	A01741	489	A00204	A01743	358
A00204	A01747	179	A00204	A01775	420
A00204	A01781	281	A00204	A01792	230
A00204	A01822	154	A00204	A01861	232
A00204	A01870	566	A00204	A01879	390
A00204	A01887	418	A00204	A01888	380
A00204	A01923	156	A00204	A01980	224
A00204	A01982	218	A00204	A02011	217
A00204	A02048	445	A00204	A02049	154
A00204	A02083	419	A00204	A02111	375
A00204	A02174	353	A00204	A02180	221
A00204	A02215	490	A00204	A02220	176
A00204	A02275	346	A00204	A02279	241
A00204	A02298	414	A00204	A02300	272
A00204	A02306	358	A00204	A02314	185
A00204	A02324	162	A00204	A02329	247
A00204	A02364	174	A00204	A02382	213
A00204	A02402	266	A00204	A02412	221
A00204	A02447	400	A00204	A02454	364
A00204	A02457	160	A00204	A02465	180
A00204	A02472	409	A00204	A02490	488
A00204	A02521	324	A00204	A02545	333
A00204	A02578	219	A00204	A02582	477
A00204	A02601	173	A00204	A02605	451
A00204	A02611	337	A00204	A02637	180
A00204	A02644	243	A00204	A02653	157

A00204	A02654	329	A00204	A02655	243
A00204	A02659	355	A00204	A02668	423
A00204	A02681	238	A00204	A02699	157
A00204	A02714	316	A00204	A02730	306
A00204	A02739	307	A00204	A02751	241
A00204	A02756	353	A00204	A02785	477
A00204	A02803	216	A00204	A02812	271
A00204	A02837	369	A00204	A02847	480
A00204	A02851	219	A00204	A02859	491
A00204	A02874	490	A00204	A02876	182
A00204	A02877	240	A00204	A02878	154
A00204	A02883	252	A00204	A02886	301
A00204	A02896	491	A00204	A02897	185
A00204	A02905	193	A00204	A02916	491
A00204	A02922	337	A00204	A02923	185
A00204	A02924	230	A00204	A02932	185
A00204	A02936	461	A00204	A02938	302
A00204	A02944	491	A00204	A02946	277
A00204	A02951	481	A00204	A02953	484
A00204	A02961	491	A00204	A02966	360
A00204	A02971	443	A00204	A02972	479
A00204	A02976	183	A00204	A02982	477
A00204	A03019	269	A00204	A03034	188
A00204	A03050	474	A00204	A03080	417
A00204	A03087	198	A00204	A03099	435
A00204	A03112	274	A00204	A03144	405
A00204	A03157	373	A00204	A03189	185
A00204	A03224	229	A00204	A03253	277
A00204	A03259	432	A00204	A03275	490
A00204	A03279	312	A00204	A03280	270
A00204	A03281	424	A00204	A03282	491
A00204	A03346	170	A00204	A03347	168
A00204	A03370	435	A00204	A03379	313
A00204	A03401	160	A00204	A03408	157
A00204	A03410	219	A00204	A03432	243
A00204	A03520	167	A00204	A03522	283
A00204	A03523	208	A00204	A03534	207
A00204	A03545	491	A00204	A03557	193
A00204	A03568	217	A00204	A03635	221
A00204	A03682	468	A00204	A03831	176
A00204	A03922	424	A00204	A03923	182
A00204	A03991	168	A00204	A04010	475
A00204	A04023	160	A00204	A04045	338
A00204	A04051	486	A00204	A04079	204
A00204	A04095	238	A00204	A04110	249
A00204	A04144	157	A00204	A04155	162

A00204	A04157	229	A00204	A04159	195
A00204	A04168	185	A00204	A04200	195
A00204	A04206	330	A00204	A04240	207
A00204	A04244	460	A00204	A04247	162
A00204	A04254	207	A00204	A04298	207
A00204	A04326	302	A00204	A04369	385
A00204	A04392	221	A00204	A04436	176
A00204	A04481	152	A00204	A04526	176
A00204	A04554	243	A00204	A04566	207
A00204	A04567	156	A00204	A04584	157
A00204	A04589	298	A00204	A04605	168
A00204	A04612	191	A00204	A04613	353
A00204	A04645	195	A00204	A04734	350
A00204	A04735	168	A00204	A04813	196
A00204	A04839	245	A00204	A04854	207
A00204	A04855	195	A00204	A04860	185
A00204	A04862	195	A00204	A04865	353
A00204	A04877	152	A00204	A04923	221
A00204	A04955	195	A00204	A04981	229
A00204	A05051	199	A00204	A05107	279
A00204	A05110	277	A00204	A05112	156
A00204	A05113	202	A00204	A05136	176
A00204	A05153	462	A00204	A05211	162
A00204	A05307	168	A00204	A05349	475
A00204	A05356	277	A00204	A05455	162
A00204	A05467	201	A00204	A05469	185
A00204	A05485	157	A00204	A05532	195
A00204	A05577	385	A00204	A05592	243
A00204	A05608	185	A00204	A05668	221
A00204	A05826	162	A00204	A05945	156
A00204	A05957	168	A00204	A05985	419
A00204	A05999	176	A00204	A06149	405
A00204	A06199	351	A00204	A06224	168
A00204	A06284	176	A00204	A06286	424
A00204	A06312	347	A00204	A06395	277
A00204	A06415	378	A00204	A06422	350
A00204	A06567	318	A00204	A06607	414
A00204	A06642	332	A00204	A06709	207
A00204	A06752	402	A00204	A06778	152
A00204	A06800	221	A00204	A06980	221
A00204	A07130	165	A00204	A07135	398
A00204	A07138	215	A00204	A07141	162
A00204	A07147	215	A00204	A07157	653
A00204	A07161	369	A00204	A07165	474
A00204	A07166	369	A00204	A07171	207
A00204	A07173	401	A00204	A07200	245

A00204	A07218	477	A00204	A07308	221
A00204	A07347	321	A00204	A07349	174
A00204	A07468	419	A00204	A07484	207
A00204	A07520	252	A00204	A07541	258
A00204	A07555	243	A00204	A07563	195
A00204	A07568	283	A00204	A07700	151
A00204	A07750	277	A00204	A07807	168
A00204	A07919	157	A00204	A07961	364
A00204	A08063	207	A00204	A08137	199
A00204	A08199	210	A00204	A08280	508
A00204	A08403	210	A00204	A08404	229
A00204	A08647	215	A00204	A09062	265
A00204	A09153	185	A00204	A09435	294
A00204	A09540	249	A00204	A09556	423
A00204	A09644	174	A00204	A09857	457
A00204	A10011	277	A00204	A10163	195
A00204	A10214	683	A00204	A10413	277
A00204	A10521	168	A00204	A10527	269
A00204	A10541	277	A00204	A10553	221
A00204	A10567	243	A00204	A10635	193
A00204	A10673	172	A00204	A10675	162
A00204	A10682	185	A00204	A10692	168
A00204	A10700	157	A00204	A10791	296
A00204	A10823	176	A00204	A10963	243
A00204	A10967	413	A00204	A10973	243
A00204	A11009	207	A00204	A11062	162
A00204	A11098	243	A00204	A11300	542
A00204	A11432	176	A00204	A11442	243
A00204	A11452	252	A00204	A11567	265
A00204	A11770	277	A00204	A11896	243
A00204	A11983	428	A00204	A12136	152
A00204	A12307	243	A00204	A12392	199
A00204	A12419	184	A00204	A12439	243
A00204	A12793	279	A00204	A12809	195
A00204	A12824	469	A00204	A12970	176
A00204	A13191	152	A00204	A13383	157
A00204	A13782	207	A00204	A13824	63
A00204	A13860	527	A00204	A13872	385
A00204	A13909	243	A00204	A13944	185
A00204	A13954	340	A00204	A14231	677
A00204	A14374	463	A00204	A14430	151
A00204	A14440	224	A00204	A14566	272
A00204	A14599	246	A00204	A14603	162
A00204	A14720	176	A00204	A14722	207
A00204	A15111	185	A00204	A15323	401
A00204	A15394	279	A00204	A15395	154

A00204	A15611	165	A00204	A15639	422
A00204	A15786	152	A00204	A15795	182
A00204	A15806	324	A00204	A15832	185
A00204	A15956	277	A00204	A15992	259
A00204	A15993	168	A00204	A16104	243
A00204	A16216	301	A00204	A16343	152
A00204	A16423	185	A00204	A16636	357
A00204	A16693	152	A00204	A16695	157
A00204	A16959	210	A00204	A17019	162
A00204	A17078	313	A00204	A17108	347
A00204	A17171	199	A00205	A00207	945
A00205	A00208	685	A00205	A00209	899
A00205	A00211	271	A00205	A00224	296
A00205	A00230	387	A00205	A00240	344
A00205	A00246	900	A00205	A00254	277
A00205	A00261	235	A00205	A00262	152
A00205	A00314	224	A00205	A01512	63
A00205	A01524	930	A00205	A01527	940
A00205	A01553	942	A00205	A01554	945
A00205	A01558	899	A00205	A01560	113
A00205	A01572	229	A00205	A01588	899
A00205	A01615	900	A00205	A01697	218
A00205	A01730	167	A00205	A01914	168
A00205	A02711	177	A00205	A02790	182
A00205	A02791	168	A00205	A03654	174
A00205	A03784	224	A00205	A04117	160
A00205	A04162	156	A00205	A04222	267
A00205	A04490	195	A00205	A06610	408
A00205	A07311	937	A00205	A09181	353
A00205	A09542	344	A00205	A09577	356
A00205	A09888	291	A00205	A09937	419
A00205	A10580	899	A00205	A10791	235
A00205	A10941	307	A00205	A11369	457
A00205	A12086	291	A00205	A13605	218
A00205	A13980	974	A00205	A13985	219
A00205	A13991	989	A00205	A14033	329
A00205	A14198	187	A00205	A15726	63
A00205	A16257	188	A00206	A00209	899
A00206	A00221	669	A00206	A00229	249
A00206	A00230	940	A00206	A00235	269
A00206	A00254	296	A00206	A00263	477
A00206	A00269	655	A00206	A00270	473
A00206	A00295	436	A00206	A00400	594
A00206	A00600	174	A00206	A00697	465
A00206	A00786	458	A00206	A00941	452
A00206	A00973	396	A00206	A01070	903

A00206	A01161	342	A00206	A01192	435
A00206	A01310	319	A00206	A01478	488
A00206	A01479	170	A00206	A01527	899
A00206	A01533	490	A00206	A01551	427
A00206	A01558	899	A00206	A01560	480
A00206	A01568	270	A00206	A01572	430
A00206	A01576	186	A00206	A01588	899
A00206	A01591	680	A00206	A01592	975
A00206	A01596	257	A00206	A01615	900
A00206	A01636	516	A00206	A01638	479
A00206	A01650	491	A00206	A01660	413
A00206	A01667	391	A00206	A01669	156
A00206	A01692	655	A00206	A01703	173
A00206	A02411	188	A00206	A02429	235
A00206	A02507	154	A00206	A03422	470
A00206	A03471	441	A00206	A03492	202
A00206	A03496	221	A00206	A03566	467
A00206	A03575	450	A00206	A03591	820
A00206	A03656	183	A00206	A04175	243
A00206	A04740	171	A00206	A05008	396
A00206	A05009	428	A00206	A05015	247
A00206	A05029	307	A00206	A05101	229
A00206	A05202	171	A00206	A05315	899
A00206	A06701	413	A00206	A07339	899
A00206	A07762	425	A00206	A07997	271
A00206	A08518	156	A00206	A08571	425
A00206	A08862	440	A00206	A09195	474
A00206	A09370	171	A00206	A09449	450
A00206	A09551	235	A00206	A09568	302
A00206	A09820	361	A00206	A10183	317
A00206	A10510	193	A00206	A10833	994
A00206	A11027	160	A00206	A11736	288
A00206	A11820	383	A00206	A12047	176
A00206	A12902	899	A00206	A13391	154
A00206	A13488	202	A00206	A13530	288
A00206	A13917	465	A00206	A13997	899
A00206	A14198	407	A00206	A14352	160
A00206	A14443	899	A00206	A14444	991
A00206	A14577	709	A00206	A15293	899
A00206	A15294	918	A00206	A15295	196
A00206	A15604	176	A00206	A15649	490
A00206	A15726	926	A00207	A00208	945
A00207	A00209	899	A00207	A00214	171
A00207	A00221	385	A00207	A00224	976
A00207	A00230	906	A00207	A00233	899
A00207	A00234	945	A00207	A00239	942

A00207	A00240	945	A00207	A00241	932
A00207	A00242	993	A00207	A00246	994
A00207	A00247	422	A00207	A00254	945
A00207	A00261	631	A00207	A00316	440
A00207	A00324	936	A00207	A00332	243
A00207	A00340	232	A00207	A00341	170
A00207	A00669	932	A00207	A00696	249
A00207	A00887	154	A00207	A00933	899
A00207	A00997	208	A00207	A01027	187
A00207	A01052	438	A00207	A01452	557
A00207	A01474	196	A00207	A01524	994
A00207	A01527	994	A00207	A01551	224
A00207	A01553	470	A00207	A01554	329
A00207	A01558	993	A00207	A01560	970
A00207	A01561	916	A00207	A01562	939
A00207	A01572	456	A00207	A01588	936
A00207	A01602	902	A00207	A01612	899
A00207	A01615	919	A00207	A01628	907
A00207	A01636	899	A00207	A01637	899
A00207	A01638	899	A00207	A01640	152
A00207	A01688	924	A00207	A01692	388
A00207	A01697	939	A00207	A01709	923
A00207	A01718	252	A00207	A01719	63
A00207	A01790	183	A00207	A01844	931
A00207	A01914	249	A00207	A01995	488
A00207	A02369	154	A00207	A02482	238
A00207	A02678	288	A00207	A03110	255
A00207	A03292	160	A00207	A03436	340
A00207	A03591	921	A00207	A03778	201
A00207	A03809	204	A00207	A03942	339
A00207	A04012	505	A00207	A04117	473
A00207	A04530	191	A00207	A04699	899
A00207	A04796	296	A00207	A04797	265
A00207	A05124	329	A00207	A05216	899
A00207	A05633	318	A00207	A06295	899
A00207	A06582	208	A00207	A06610	469
A00207	A07118	191	A00207	A07135	190
A00207	A07165	899	A00207	A07311	955
A00207	A07339	909	A00207	A08280	296
A00207	A08732	899	A00207	A09181	926
A00207	A09188	243	A00207	A09241	281
A00207	A09434	416	A00207	A09542	900
A00207	A09577	63	A00207	A09804	157
A00207	A09937	210	A00207	A10365	899
A00207	A10735	63	A00207	A10756	443
A00207	A10801	994	A00207	A11125	899

A00207	A11369	945	A00207	A11652	174
A00207	A11934	154	A00207	A12705	899
A00207	A12924	157	A00207	A13250	899
A00207	A13364	441	A00207	A13433	243
A00207	A13605	937	A00207	A13841	265
A00207	A13975	386	A00207	A13980	429
A00207	A13981	899	A00207	A13983	63
A00207	A13991	899	A00207	A14004	270
A00207	A14013	138	A00207	A14019	358
A00207	A14027	899	A00207	A14032	274
A00207	A14033	218	A00207	A14047	229
A00207	A14059	167	A00207	A14187	440
A00207	A14188	943	A00207	A14198	945
A00207	A14207	899	A00207	A14208	510
A00207	A14426	310	A00207	A14433	945
A00207	A14443	899	A00207	A14452	63
A00207	A14496	452	A00207	A15182	912
A00207	A15294	899	A00207	A15325	235
A00207	A15361	899	A00207	A15726	163
A00207	A15848	243	A00207	A15893	457
A00207	A16084	104	A00208	A00209	899
A00208	A00211	899	A00208	A00216	201
A00208	A00224	949	A00208	A00227	383
A00208	A00230	541	A00208	A00233	168
A00208	A00234	436	A00208	A00240	295
A00208	A00243	491	A00208	A00246	942
A00208	A00254	945	A00208	A00261	212
A00208	A00270	538	A00208	A00292	208
A00208	A00294	395	A00208	A00309	477
A00208	A00400	477	A00208	A00418	377
A00208	A00463	230	A00208	A00491	168
A00208	A00511	195	A00208	A00556	219
A00208	A00566	566	A00208	A00643	236
A00208	A00747	450	A00208	A00799	246
A00208	A00805	272	A00208	A00858	503
A00208	A00922	212	A00208	A00931	208
A00208	A01006	430	A00208	A01052	899
A00208	A01061	452	A00208	A01104	243
A00208	A01114	276	A00208	A01125	361
A00208	A01137	294	A00208	A01155	278
A00208	A01212	440	A00208	A01406	159
A00208	A01473	283	A00208	A01498	168
A00208	A01524	989	A00208	A01527	992
A00208	A01553	63	A00208	A01554	85
A00208	A01558	989	A00208	A01560	899
A00208	A01572	466	A00208	A01588	899

A00208	A01602	486	A00208	A01612	899
A00208	A01615	989	A00208	A01639	899
A00208	A01688	899	A00208	A01741	452
A00208	A01796	234	A00208	A01797	170
A00208	A01800	157	A00208	A01914	490
A00208	A01949	252	A00208	A01970	152
A00208	A01974	180	A00208	A02072	198
A00208	A02111	199	A00208	A02304	204
A00208	A02327	184	A00208	A02342	265
A00208	A02361	290	A00208	A02481	463
A00208	A02562	408	A00208	A02564	899
A00208	A02671	202	A00208	A02695	162
A00208	A02711	401	A00208	A02715	179
A00208	A02877	346	A00208	A02882	288
A00208	A02902	188	A00208	A02908	296
A00208	A02988	274	A00208	A03213	484
A00208	A03225	419	A00208	A03259	262
A00208	A03273	247	A00208	A03514	301
A00208	A03637	346	A00208	A03681	377
A00208	A03685	451	A00208	A03725	469
A00208	A03784	238	A00208	A03837	268
A00208	A04085	396	A00208	A04092	188
A00208	A04115	302	A00208	A04163	199
A00208	A04209	344	A00208	A04238	235
A00208	A04314	383	A00208	A04622	283
A00208	A04685	201	A00208	A04770	215
A00208	A04814	157	A00208	A04943	157
A00208	A05107	221	A00208	A05112	430
A00208	A05184	152	A00208	A05216	945
A00208	A05280	217	A00208	A05288	213
A00208	A05533	160	A00208	A05618	217
A00208	A05760	899	A00208	A05891	433
A00208	A06513	252	A00208	A06579	159
A00208	A06581	371	A00208	A06597	491
A00208	A06649	198	A00208	A06650	439
A00208	A06769	241	A00208	A06911	157
A00208	A06926	204	A00208	A06949	385
A00208	A07033	217	A00208	A07191	224
A00208	A07311	901	A00208	A07339	489
A00208	A07422	899	A00208	A07460	180
A00208	A07900	207	A00208	A08732	899
A00208	A08911	467	A00208	A09072	157
A00208	A09123	156	A00208	A09236	179
A00208	A09241	201	A00208	A10161	195
A00208	A10174	152	A00208	A10203	217
A00208	A11369	479	A00208	A11460	213

A00208	A11512	252	A00208	A11764	252
A00208	A11832	324	A00208	A11983	901
A00208	A12108	157	A00208	A12965	195
A00208	A13386	252	A00208	A13605	907
A00208	A13860	243	A00208	A13899	272
A00208	A13978	899	A00208	A13980	753
A00208	A13981	899	A00208	A13991	899
A00208	A14055	420	A00208	A14073	230
A00208	A14188	491	A00208	A14198	945
A00208	A14220	481	A00208	A14399	267
A00208	A14433	924	A00208	A14444	899
A00208	A14683	177	A00208	A15315	193
A00208	A15323	172	A00208	A15347	177
A00208	A15621	168	A00208	A15764	489
A00208	A15862	180	A00208	A15913	185
A00208	A15932	302	A00208	A15935	335
A00208	A16257	268	A00208	A16438	185
A00208	A16734	157	A00209	A00211	911
A00209	A00216	899	A00209	A00218	384
A00209	A00219	945	A00209	A00220	912
A00209	A00221	900	A00209	A00223	899
A00209	A00224	924	A00209	A00225	899
A00209	A00227	899	A00209	A00230	915
A00209	A00231	921	A00209	A00236	899
A00209	A00239	899	A00209	A00240	899
A00209	A00241	899	A00209	A00242	899
A00209	A00243	899	A00209	A00244	899
A00209	A00246	899	A00209	A00247	899
A00209	A00248	899	A00209	A00250	899
A00209	A00252	899	A00209	A00253	899
A00209	A00254	949	A00209	A00255	899
A00209	A00256	369	A00209	A00257	899
A00209	A00258	934	A00209	A00264	994
A00209	A00265	899	A00209	A00268	899
A00209	A00269	899	A00209	A00270	909
A00209	A00271	899	A00209	A00272	899
A00209	A00274	899	A00209	A00275	899
A00209	A00276	899	A00209	A00283	199
A00209	A00294	943	A00209	A00301	318
A00209	A00309	405	A00209	A00310	899
A00209	A00312	899	A00209	A00324	343
A00209	A00356	899	A00209	A00358	899
A00209	A00361	899	A00209	A00377	899
A00209	A00396	899	A00209	A00400	215
A00209	A00440	398	A00209	A00472	176
A00209	A00531	899	A00209	A00571	199

A00209	A00616	932	A00209	A00625	899
A00209	A00629	199	A00209	A00635	162
A00209	A00643	557	A00209	A00651	899
A00209	A00656	913	A00209	A00671	191
A00209	A00688	401	A00209	A00696	188
A00209	A00700	899	A00209	A00815	899
A00209	A00841	918	A00209	A00851	899
A00209	A00914	922	A00209	A00931	899
A00209	A00939	912	A00209	A00981	944
A00209	A01022	483	A00209	A01026	899
A00209	A01056	899	A00209	A01070	930
A00209	A01098	207	A00209	A01125	489
A00209	A01138	899	A00209	A01139	899
A00209	A01157	461	A00209	A01161	414
A00209	A01168	899	A00209	A01211	483
A00209	A01252	899	A00209	A01257	908
A00209	A01289	899	A00209	A01291	914
A00209	A01305	154	A00209	A01306	199
A00209	A01359	899	A00209	A01425	914
A00209	A01457	899	A00209	A01458	899
A00209	A01461	899	A00209	A01472	899
A00209	A01475	466	A00209	A01477	899
A00209	A01485	899	A00209	A01492	899
A00209	A01493	899	A00209	A01496	899
A00209	A01503	899	A00209	A01512	899
A00209	A01515	899	A00209	A01522	899
A00209	A01524	995	A00209	A01527	944
A00209	A01531	208	A00209	A01553	899
A00209	A01554	899	A00209	A01558	899
A00209	A01560	899	A00209	A01562	899
A00209	A01568	221	A00209	A01572	899
A00209	A01574	899	A00209	A01576	899
A00209	A01585	899	A00209	A01588	899
A00209	A01593	899	A00209	A01601	914
A00209	A01602	899	A00209	A01604	899
A00209	A01605	899	A00209	A01606	922
A00209	A01615	899	A00209	A01626	899
A00209	A01634	899	A00209	A01636	899
A00209	A01639	899	A00209	A01640	916
A00209	A01646	899	A00209	A01658	899
A00209	A01661	900	A00209	A01662	215
A00209	A01665	899	A00209	A01669	899
A00209	A01676	937	A00209	A01683	899
A00209	A01688	899	A00209	A01692	899
A00209	A01708	899	A00209	A01718	914
A00209	A01719	899	A00209	A01722	173

A00209	A01726	899	A00209	A01730	899
A00209	A01737	899	A00209	A01740	899
A00209	A01741	475	A00209	A01743	160
A00209	A01745	188	A00209	A01749	899
A00209	A01755	899	A00209	A01775	249
A00209	A01781	899	A00209	A01786	899
A00209	A01790	473	A00209	A01844	907
A00209	A01860	899	A00209	A01870	899
A00209	A01878	899	A00209	A01880	428
A00209	A01905	903	A00209	A01931	473
A00209	A01974	899	A00209	A01983	899
A00209	A01995	263	A00209	A02010	439
A00209	A02043	156	A00209	A02044	899
A00209	A02083	900	A00209	A02094	903
A00209	A02104	905	A00209	A02105	899
A00209	A02124	899	A00209	A02126	899
A00209	A02142	170	A00209	A02148	256
A00209	A02156	899	A00209	A02163	903
A00209	A02172	177	A00209	A02174	899
A00209	A02215	912	A00209	A02221	899
A00209	A02226	418	A00209	A02278	277
A00209	A02284	899	A00209	A02326	165
A00209	A02330	899	A00209	A02333	168
A00209	A02356	172	A00209	A02387	157
A00209	A02390	899	A00209	A02393	193
A00209	A02412	235	A00209	A02416	330
A00209	A02425	901	A00209	A02460	899
A00209	A02464	899	A00209	A02465	899
A00209	A02481	899	A00209	A02482	204
A00209	A02490	229	A00209	A02491	455
A00209	A02502	187	A00209	A02504	174
A00209	A02505	424	A00209	A02590	916
A00209	A02615	900	A00209	A02668	901
A00209	A02723	899	A00209	A02754	285
A00209	A02756	918	A00209	A02785	187
A00209	A02787	900	A00209	A02788	918
A00209	A02836	393	A00209	A02874	190
A00209	A02887	899	A00209	A02917	899
A00209	A02982	910	A00209	A03010	899
A00209	A03054	913	A00209	A03076	899
A00209	A03095	465	A00209	A03099	899
A00209	A03104	319	A00209	A03107	901
A00209	A03110	899	A00209	A03141	419
A00209	A03186	360	A00209	A03188	257
A00209	A03209	899	A00209	A03213	899
A00209	A03219	899	A00209	A03224	899

A00209	A03273	899	A00209	A03292	335
A00209	A03293	899	A00209	A03304	259
A00209	A03315	343	A00209	A03331	899
A00209	A03332	899	A00209	A03415	438
A00209	A03434	386	A00209	A03436	466
A00209	A03498	899	A00209	A03509	899
A00209	A03567	199	A00209	A03591	953
A00209	A03625	899	A00209	A03633	899
A00209	A03639	920	A00209	A03723	899
A00209	A03726	899	A00209	A03778	296
A00209	A03794	185	A00209	A03803	899
A00209	A03893	217	A00209	A03904	374
A00209	A03913	899	A00209	A03942	899
A00209	A03946	152	A00209	A03961	936
A00209	A04012	899	A00209	A04033	196
A00209	A04063	424	A00209	A04202	939
A00209	A04225	899	A00209	A04226	899
A00209	A04237	899	A00209	A04294	156
A00209	A04337	208	A00209	A04347	899
A00209	A04403	899	A00209	A04422	899
A00209	A04449	195	A00209	A04473	899
A00209	A04484	906	A00209	A04548	196
A00209	A04569	165	A00209	A04571	249
A00209	A04761	899	A00209	A04818	218
A00209	A04832	469	A00209	A04873	357
A00209	A04949	638	A00209	A04965	159
A00209	A04981	902	A00209	A05029	899
A00209	A05040	251	A00209	A05208	901
A00209	A05216	899	A00209	A05221	401
A00209	A05264	380	A00209	A05288	486
A00209	A05372	156	A00209	A05418	799
A00209	A05474	165	A00209	A05533	902
A00209	A05597	899	A00209	A05621	199
A00209	A05723	165	A00209	A05809	908
A00209	A05867	899	A00209	A05886	311
A00209	A05887	262	A00209	A05965	177
A00209	A05979	152	A00209	A05988	899
A00209	A05992	899	A00209	A05996	899
A00209	A06038	184	A00209	A06219	156
A00209	A06295	899	A00209	A06305	899
A00209	A06554	329	A00209	A06578	201
A00209	A06581	913	A00209	A06588	450
A00209	A06599	293	A00209	A06603	899
A00209	A06608	899	A00209	A06609	899
A00209	A06615	491	A00209	A06628	352
A00209	A06650	481	A00209	A06731	235

A00209	A06762	906	A00209	A06818	180
A00209	A06861	199	A00209	A06986	251
A00209	A06999	215	A00209	A07006	899
A00209	A07165	899	A00209	A07201	899
A00209	A07209	152	A00209	A07283	899
A00209	A07297	899	A00209	A07311	903
A00209	A07339	944	A00209	A07370	168
A00209	A07422	899	A00209	A07460	899
A00209	A07483	899	A00209	A07803	360
A00209	A07999	263	A00209	A08575	251
A00209	A08602	899	A00209	A08718	419
A00209	A08859	199	A00209	A08875	350
A00209	A08878	908	A00209	A08943	899
A00209	A09028	452	A00209	A09036	201
A00209	A09072	902	A00209	A09173	193
A00209	A09184	193	A00209	A09201	899
A00209	A09210	899	A00209	A09235	347
A00209	A09241	899	A00209	A09258	899
A00209	A09322	199	A00209	A09377	899
A00209	A09453	165	A00209	A09467	177
A00209	A09509	176	A00209	A09513	156
A00209	A09543	899	A00209	A09660	902
A00209	A10005	494	A00209	A10064	165
A00209	A10113	165	A00209	A10148	196
A00209	A10214	899	A00209	A10228	899
A00209	A10233	899	A00209	A10278	899
A00209	A10340	899	A00209	A10365	899
A00209	A10392	899	A00209	A10532	899
A00209	A10542	908	A00209	A10580	899
A00209	A10584	899	A00209	A10597	899
A00209	A10601	899	A00209	A10666	212
A00209	A10731	314	A00209	A10743	899
A00209	A10801	899	A00209	A10825	177
A00209	A10833	899	A00209	A10875	156
A00209	A11125	899	A00209	A11369	904
A00209	A11392	210	A00209	A11492	177
A00209	A11522	899	A00209	A11834	931
A00209	A11839	899	A00209	A11850	899
A00209	A11893	156	A00209	A11897	195
A00209	A11911	219	A00209	A11932	469
A00209	A11951	179	A00209	A11983	943
A00209	A12265	165	A00209	A12273	254
A00209	A12365	326	A00209	A12373	932
A00209	A12383	899	A00209	A12420	899
A00209	A12421	899	A00209	A12459	156
A00209	A12474	165	A00209	A12605	156

A00209	A12666	899	A00209	A12693	193
A00209	A12818	899	A00209	A12883	165
A00209	A12897	491	A00209	A12932	903
A00209	A12959	156	A00209	A12971	899
A00209	A12990	899	A00209	A13003	899
A00209	A13063	899	A00209	A13180	165
A00209	A13241	224	A00209	A13344	899
A00209	A13369	899	A00209	A13580	899
A00209	A13605	899	A00209	A13720	899
A00209	A13824	899	A00209	A13860	899
A00209	A13873	914	A00209	A13886	179
A00209	A13924	196	A00209	A13930	899
A00209	A13935	903	A00209	A13958	899
A00209	A13975	899	A00209	A13977	899
A00209	A13978	899	A00209	A13981	899
A00209	A13987	899	A00209	A13988	899
A00209	A13989	899	A00209	A13992	899
A00209	A13994	899	A00209	A13999	899
A00209	A14003	899	A00209	A14010	899
A00209	A14017	899	A00209	A14030	899
A00209	A14032	899	A00209	A14033	899
A00209	A14038	901	A00209	A14046	899
A00209	A14055	914	A00209	A14056	899
A00209	A14060	908	A00209	A14061	899
A00209	A14071	900	A00209	A14128	268
A00209	A14133	899	A00209	A14139	899
A00209	A14188	899	A00209	A14190	899
A00209	A14191	917	A00209	A14193	899
A00209	A14196	913	A00209	A14198	952
A00209	A14208	899	A00209	A14229	927
A00209	A14245	899	A00209	A14246	899
A00209	A14299	477	A00209	A14319	288
A00209	A14366	899	A00209	A14369	899
A00209	A14371	899	A00209	A14374	899
A00209	A14391	899	A00209	A14392	901
A00209	A14393	928	A00209	A14397	899
A00209	A14410	265	A00209	A14443	995
A00209	A14444	994	A00209	A14456	899
A00209	A14484	899	A00209	A14506	899
A00209	A14511	899	A00209	A14557	899
A00209	A14561	177	A00209	A14577	358
A00209	A14596	899	A00209	A14604	899
A00209	A14608	177	A00209	A14635	221
A00209	A14650	313	A00209	A14654	899
A00209	A14661	899	A00209	A14925	183
A00209	A15290	899	A00209	A15291	899

A00209	A15292	903	A00209	A15293	943
A00209	A15294	899	A00209	A15295	899
A00209	A15296	938	A00209	A15297	899
A00209	A15300	899	A00209	A15301	899
A00209	A15302	899	A00209	A15304	899
A00209	A15305	899	A00209	A15307	899
A00209	A15312	899	A00209	A15314	942
A00209	A15315	899	A00209	A15318	245
A00209	A15322	943	A00209	A15323	899
A00209	A15337	899	A00209	A15340	899
A00209	A15341	911	A00209	A15342	902
A00209	A15343	904	A00209	A15344	899
A00209	A15347	899	A00209	A15350	899
A00209	A15355	899	A00209	A15356	899
A00209	A15357	912	A00209	A15361	899
A00209	A15366	899	A00209	A15398	900
A00209	A15400	899	A00209	A15402	899
A00209	A15422	899	A00209	A15480	899
A00209	A15496	156	A00209	A15506	414
A00209	A15617	899	A00209	A15620	899
A00209	A15634	899	A00209	A15726	199
A00209	A15821	251	A00209	A15854	391
A00209	A15933	899	A00209	A15951	899
A00209	A15997	899	A00209	A16030	899
A00209	A16080	899	A00209	A16083	899
A00209	A16253	899	A00209	A16323	180
A00209	A16357	251	A00209	A16424	899
A00209	A16581	899	A00209	A16734	156
A00209	A16812	899	A00209	A16916	899
A00209	A16988	899	A00209	A17060	899
A00209	A17188	899	A00209	A17214	899
A00209	A17215	899	A00209	A17216	899
A00209	A17217	899	A00210	A00212	367
A00210	A00219	236	A00210	A00224	910
A00210	A00227	168	A00210	A00230	936
A00210	A00239	536	A00210	A00240	470
A00210	A00241	542	A00210	A00243	291
A00210	A00246	899	A00210	A00261	586
A00210	A00273	193	A00210	A00304	515
A00210	A00315	424	A00210	A00316	204
A00210	A00322	747	A00210	A00347	755
A00210	A00418	477	A00210	A00473	491
A00210	A00565	208	A00210	A00593	172
A00210	A00638	467	A00210	A00688	278
A00210	A00815	310	A00210	A00856	490
A00210	A00911	731	A00210	A00989	305

A00210	A01050	353	A00210	A01117	458
A00210	A01124	975	A00210	A01214	486
A00210	A01215	491	A00210	A01244	488
A00210	A01278	229	A00210	A01296	746
A00210	A01312	177	A00210	A01317	278
A00210	A01318	274	A00210	A01320	827
A00210	A01322	468	A00210	A01325	945
A00210	A01327	413	A00210	A01328	480
A00210	A01331	198	A00210	A01415	449
A00210	A01477	216	A00210	A01485	441
A00210	A01529	283	A00210	A01530	411
A00210	A01558	899	A00210	A01559	168
A00210	A01561	899	A00210	A01588	899
A00210	A01593	899	A00210	A01596	840
A00210	A01615	899	A00210	A01629	490
A00210	A01722	364	A00210	A01741	257
A00210	A01920	611	A00210	A01929	152
A00210	A01930	165	A00210	A01993	296
A00210	A02051	162	A00210	A02072	299
A00210	A02091	234	A00210	A02104	174
A00210	A02110	324	A00210	A02143	151
A00210	A02144	191	A00210	A02147	159
A00210	A02148	312	A00210	A02188	483
A00210	A02221	490	A00210	A02228	184
A00210	A02231	246	A00210	A02267	308
A00210	A02278	170	A00210	A02303	207
A00210	A02392	201	A00210	A02424	467
A00210	A02425	475	A00210	A02435	205
A00210	A02454	168	A00210	A02463	340
A00210	A02465	329	A00210	A02470	152
A00210	A02477	156	A00210	A02487	430
A00210	A02501	168	A00210	A02531	246
A00210	A02589	168	A00210	A02595	260
A00210	A02666	252	A00210	A02672	343
A00210	A02673	449	A00210	A02760	236
A00210	A02830	265	A00210	A03017	208
A00210	A03043	218	A00210	A03044	416
A00210	A03046	215	A00210	A03055	157
A00210	A03090	168	A00210	A03114	168
A00210	A03115	473	A00210	A03152	152
A00210	A03155	208	A00210	A03186	371
A00210	A03193	456	A00210	A03209	441
A00210	A03328	450	A00210	A03334	385
A00210	A03434	210	A00210	A03451	456
A00210	A03642	245	A00210	A03820	268
A00210	A04074	238	A00210	A04184	152

A00210	A04218	276	A00210	A04305	168
A00210	A04380	187	A00210	A04423	267
A00210	A04429	154	A00210	A04468	182
A00210	A04471	165	A00210	A04715	190
A00210	A04911	260	A00210	A04948	260
A00210	A05183	182	A00210	A05294	179
A00210	A05297	324	A00210	A05357	201
A00210	A05685	288	A00210	A05690	488
A00210	A05825	187	A00210	A06028	308
A00210	A06137	393	A00210	A06212	391
A00210	A06270	279	A00210	A06455	234
A00210	A06616	187	A00210	A06739	279
A00210	A06822	260	A00210	A07062	462
A00210	A07118	208	A00210	A07311	901
A00210	A07339	308	A00210	A07540	234
A00210	A07605	157	A00210	A07646	308
A00210	A08118	291	A00210	A08126	224
A00210	A08209	367	A00210	A08317	216
A00210	A08884	291	A00210	A08951	208
A00210	A09375	307	A00210	A09387	159
A00210	A09441	168	A00210	A09652	182
A00210	A09668	159	A00210	A09854	177
A00210	A09917	201	A00210	A10039	281
A00210	A10082	208	A00210	A10141	234
A00210	A10183	308	A00210	A10278	935
A00210	A10580	474	A00210	A10671	182
A00210	A10881	245	A00210	A10884	216
A00210	A11107	260	A00210	A11166	208
A00210	A11167	174	A00210	A11315	188
A00210	A11467	208	A00210	A11483	443
A00210	A11486	212	A00210	A11744	378
A00210	A11840	377	A00210	A12047	293
A00210	A12720	260	A00210	A12971	385
A00210	A13250	291	A00210	A13382	899
A00210	A13925	308	A00210	A14190	374
A00210	A14210	163	A00210	A14299	560
A00210	A14366	196	A00210	A14443	899
A00210	A14452	215	A00210	A14697	177
A00210	A14704	291	A00210	A14888	157
A00210	A15195	245	A00210	A15498	249
A00210	A15641	312	A00210	A15896	446
A00210	A15989	245	A00211	A00219	259
A00211	A00221	394	A00211	A00229	316
A00211	A00230	899	A00211	A00240	308
A00211	A00246	899	A00211	A00400	221
A00211	A00867	183	A00211	A01125	433

A00211	A01310	599	A00211	A01524	923
A00211	A01527	939	A00211	A01553	899
A00211	A01560	229	A00211	A01601	63
A00211	A01605	945	A00211	A01628	899
A00211	A01639	899	A00211	A01640	945
A00211	A01718	400	A00211	A01902	293
A00211	A02313	183	A00211	A02323	899
A00211	A02411	283	A00211	A02636	173
A00211	A03219	152	A00211	A03470	969
A00211	A03799	899	A00211	A04062	899
A00211	A04117	235	A00211	A04196	291
A00211	A04271	151	A00211	A04490	323
A00211	A04790	261	A00211	A05090	299
A00211	A05221	480	A00211	A06578	372
A00211	A06601	152	A00211	A06914	589
A00211	A06989	345	A00211	A07081	229
A00211	A07149	183	A00211	A07311	903
A00211	A07367	177	A00211	A07422	899
A00211	A07718	152	A00211	A07935	303
A00211	A09644	112	A00211	A11544	200
A00211	A11614	787	A00211	A12886	427
A00211	A13181	151	A00211	A13716	252
A00211	A13978	899	A00211	A14032	899
A00211	A14038	932	A00211	A14190	256
A00211	A14371	920	A00211	A14426	347
A00211	A15304	899	A00211	A15343	188
A00211	A15480	229	A00211	A16904	383
A00212	A00214	296	A00212	A00215	945
A00212	A00216	477	A00212	A00218	182
A00212	A00219	848	A00212	A00221	351
A00212	A00224	944	A00212	A00225	185
A00212	A00238	373	A00212	A00264	411
A00212	A00274	165	A00212	A00312	408
A00212	A00313	200	A00212	A00316	491
A00212	A00318	174	A00212	A00338	491
A00212	A00349	167	A00212	A00350	329
A00212	A00351	491	A00212	A00377	491
A00212	A00449	185	A00212	A00472	483
A00212	A00485	491	A00212	A00556	388
A00212	A00558	187	A00212	A00561	229
A00212	A00574	463	A00212	A00576	491
A00212	A00577	290	A00212	A00600	404
A00212	A00601	462	A00212	A00609	184
A00212	A00616	490	A00212	A00661	329
A00212	A00694	232	A00212	A00736	213
A00212	A00752	323	A00212	A00770	343

A00212	A00799	490	A00212	A00802	296
A00212	A00849	479	A00212	A00911	446
A00212	A00939	486	A00212	A00985	333
A00212	A01008	945	A00212	A01022	391
A00212	A01032	699	A00212	A01052	205
A00212	A01102	288	A00212	A01125	483
A00212	A01137	491	A00212	A01156	469
A00212	A01161	491	A00212	A01162	491
A00212	A01230	335	A00212	A01257	455
A00212	A01278	274	A00212	A01320	285
A00212	A01326	235	A00212	A01385	461
A00212	A01404	293	A00212	A01425	383
A00212	A01451	302	A00212	A01460	369
A00212	A01461	601	A00212	A01485	491
A00212	A01515	798	A00212	A01520	661
A00212	A01522	779	A00212	A01527	279
A00212	A01529	260	A00212	A01530	793
A00212	A01542	562	A00212	A01543	385
A00212	A01551	480	A00212	A01559	477
A00212	A01582	491	A00212	A01585	539
A00212	A01591	472	A00212	A01593	945
A00212	A01603	389	A00212	A01627	281
A00212	A01629	413	A00212	A01632	491
A00212	A01656	265	A00212	A01676	278
A00212	A01686	472	A00212	A01729	160
A00212	A01743	224	A00212	A01758	413
A00212	A01844	191	A00212	A01894	297
A00212	A01895	356	A00212	A01926	278
A00212	A02046	193	A00212	A02083	452
A00212	A02084	187	A00212	A02104	159
A00212	A02111	491	A00212	A02117	435
A00212	A02148	840	A00212	A02156	201
A00212	A02188	577	A00212	A02215	243
A00212	A02221	447	A00212	A02222	491
A00212	A02257	228	A00212	A02299	171
A00212	A02306	484	A00212	A02313	160
A00212	A02314	208	A00212	A02330	432
A00212	A02333	234	A00212	A02377	160
A00212	A02427	182	A00212	A02428	273
A00212	A02442	449	A00212	A02443	480
A00212	A02464	154	A00212	A02468	334
A00212	A02469	273	A00212	A02470	480
A00212	A02477	491	A00212	A02485	266
A00212	A02491	208	A00212	A02492	160
A00212	A02511	213	A00212	A02533	208
A00212	A02543	200	A00212	A02598	159

A00212	A02618	398	A00212	A02644	454
A00212	A02667	358	A00212	A02695	340
A00212	A02759	450	A00212	A02785	201
A00212	A02792	200	A00212	A02935	308
A00212	A02942	899	A00212	A02949	180
A00212	A02953	184	A00212	A03047	152
A00212	A03053	223	A00212	A03063	332
A00212	A03099	486	A00212	A03107	361
A00212	A03174	152	A00212	A03178	281
A00212	A03184	251	A00212	A03186	724
A00212	A03211	486	A00212	A03304	488
A00212	A03314	477	A00212	A03391	201
A00212	A03473	343	A00212	A03477	945
A00212	A03511	382	A00212	A03537	187
A00212	A03584	243	A00212	A03598	156
A00212	A03726	994	A00212	A03910	347
A00212	A03935	176	A00212	A03945	190
A00212	A03959	184	A00212	A03961	488
A00212	A03968	469	A00212	A03990	238
A00212	A03998	234	A00212	A04074	301
A00212	A04092	491	A00212	A04157	470
A00212	A04162	212	A00212	A04168	340
A00212	A04183	156	A00212	A04185	193
A00212	A04215	229	A00212	A04222	268
A00212	A04256	428	A00212	A04258	301
A00212	A04275	474	A00212	A04326	193
A00212	A04337	469	A00212	A04378	159
A00212	A04379	247	A00212	A04383	362
A00212	A04427	296	A00212	A04444	160
A00212	A04463	412	A00212	A04468	167
A00212	A04486	180	A00212	A04487	193
A00212	A04493	260	A00212	A04520	165
A00212	A04551	208	A00212	A04622	156
A00212	A04668	475	A00212	A04671	297
A00212	A04717	218	A00212	A04750	332
A00212	A04813	229	A00212	A04850	414
A00212	A04879	490	A00212	A04880	152
A00212	A04944	374	A00212	A04964	371
A00212	A04969	176	A00212	A04975	159
A00212	A04976	159	A00212	A04985	262
A00212	A05006	480	A00212	A05094	165
A00212	A05126	193	A00212	A05284	159
A00212	A05288	480	A00212	A05317	347
A00212	A05333	193	A00212	A05356	276
A00212	A05374	245	A00212	A05398	205
A00212	A05504	208	A00212	A05525	176

A00212	A05556	313	A00212	A05577	185
A00212	A05601	235	A00212	A05606	156
A00212	A05703	229	A00212	A05751	490
A00212	A05829	208	A00212	A05919	193
A00212	A05986	168	A00212	A06277	224
A00212	A06283	245	A00212	A06359	288
A00212	A06473	385	A00212	A06474	176
A00212	A06530	221	A00212	A06535	388
A00212	A06610	219	A00212	A06650	180
A00212	A06729	484	A00212	A06769	267
A00212	A06868	177	A00212	A06925	208
A00212	A06953	313	A00212	A06956	274
A00212	A07122	411	A00212	A07189	193
A00212	A07310	245	A00212	A07442	486
A00212	A07471	276	A00212	A07613	176
A00212	A07707	265	A00212	A07834	265
A00212	A08093	267	A00212	A08163	162
A00212	A08581	193	A00212	A08602	162
A00212	A08913	152	A00212	A09204	324
A00212	A09277	187	A00212	A09416	257
A00212	A09539	411	A00212	A09637	377
A00212	A09939	278	A00212	A09989	156
A00212	A10079	382	A00212	A10090	332
A00212	A10371	274	A00212	A10882	319
A00212	A11311	156	A00212	A11318	240
A00212	A11321	314	A00212	A11424	171
A00212	A11432	200	A00212	A11485	279
A00212	A11654	218	A00212	A11655	162
A00212	A11683	490	A00212	A11765	252
A00212	A11913	307	A00212	A12449	229
A00212	A12581	245	A00212	A12590	296
A00212	A12715	218	A00212	A13045	200
A00212	A13154	218	A00212	A13228	176
A00212	A13485	218	A00212	A13824	160
A00212	A13987	270	A00212	A14048	205
A00212	A14197	168	A00212	A14272	156
A00212	A14381	395	A00212	A14410	187
A00212	A14418	167	A00212	A14443	177
A00212	A14444	899	A00212	A14936	183
A00212	A15226	305	A00212	A15230	491
A00212	A15233	229	A00212	A15271	162
A00212	A15272	167	A00212	A15292	443
A00212	A15295	491	A00212	A15381	159
A00212	A15422	481	A00212	A15498	491
A00212	A15624	260	A00212	A15684	489
A00212	A15917	480	A00212	A15932	429

A00212	A16112	162	A00212	A16751	176
A00212	A16757	265	A00212	A17078	191
A00213	A00214	820	A00213	A00224	922
A00213	A00225	388	A00213	A00226	913
A00213	A00234	327	A00213	A00405	199
A00213	A00557	480	A00213	A00601	322
A00213	A00736	583	A00213	A00939	273
A00213	A01052	232	A00213	A01307	488
A00213	A01524	901	A00213	A01527	902
A00213	A01558	899	A00213	A01576	615
A00213	A01588	902	A00213	A02833	208
A00213	A02962	361	A00213	A02974	205
A00213	A02977	230	A00213	A03457	152
A00213	A03625	63	A00213	A03699	199
A00213	A03817	170	A00213	A03892	63
A00213	A03965	302	A00213	A03998	283
A00213	A04012	423	A00213	A04050	364
A00213	A04115	157	A00213	A04218	171
A00213	A04471	191	A00213	A04611	360
A00213	A04659	489	A00213	A05082	426
A00213	A05467	152	A00213	A05633	489
A00213	A06161	459	A00213	A06695	258
A00213	A06891	162	A00213	A06903	154
A00213	A07174	466	A00213	A07210	252
A00213	A07276	177	A00213	A08133	199
A00213	A09642	283	A00213	A09743	168
A00213	A09754	456	A00213	A10217	299
A00213	A10247	355	A00213	A10460	260
A00213	A10601	936	A00213	A10871	205
A00213	A11676	165	A00213	A11850	221
A00213	A11906	188	A00213	A12054	328
A00213	A12175	393	A00213	A12727	185
A00213	A13199	208	A00213	A13824	63
A00213	A13826	205	A00213	A13975	430
A00213	A14030	301	A00213	A14196	508
A00213	A14204	191	A00213	A14208	484
A00213	A14262	223	A00213	A14276	198
A00213	A14395	517	A00213	A14456	928
A00213	A14882	157	A00213	A15241	180
A00213	A15291	420	A00213	A15303	270
A00213	A15314	574	A00213	A15507	457
A00213	A15510	423	A00213	A15547	396
A00213	A15575	241	A00213	A15597	269
A00213	A15607	160	A00213	A15612	252
A00213	A15614	905	A00213	A15972	283
A00213	A15973	249	A00213	A15974	177

A00213	A15978	235	A00213	A16216	162
A00213	A16316	228	A00213	A16397	352
A00213	A16496	241	A00213	A16832	201
A00214	A00224	394	A00214	A00225	353
A00214	A00226	343	A00214	A00230	899
A00214	A00234	996	A00214	A00246	899
A00214	A00254	490	A00214	A00258	899
A00214	A00310	270	A00214	A00316	435
A00214	A00324	932	A00214	A00395	196
A00214	A00451	174	A00214	A00557	962
A00214	A00587	398	A00214	A00600	489
A00214	A00601	441	A00214	A00641	491
A00214	A00655	401	A00214	A00736	445
A00214	A00757	477	A00214	A00765	450
A00214	A00818	414	A00214	A00868	229
A00214	A00901	199	A00214	A00916	240
A00214	A00939	945	A00214	A00987	167
A00214	A01052	241	A00214	A01070	899
A00214	A01088	174	A00214	A01132	193
A00214	A01133	457	A00214	A01173	263
A00214	A01286	224	A00214	A01291	899
A00214	A01307	954	A00214	A01385	263
A00214	A01419	463	A00214	A01438	449
A00214	A01474	226	A00214	A01492	899
A00214	A01520	63	A00214	A01524	902
A00214	A01527	920	A00214	A01558	899
A00214	A01560	917	A00214	A01562	899
A00214	A01572	471	A00214	A01576	958
A00214	A01588	899	A00214	A01593	989
A00214	A01603	318	A00214	A01615	903
A00214	A01628	85	A00214	A01660	260
A00214	A01668	469	A00214	A01692	904
A00214	A01719	65	A00214	A01748	479
A00214	A01785	174	A00214	A01792	151
A00214	A01870	238	A00214	A01914	486
A00214	A01920	252	A00214	A01995	407
A00214	A02460	157	A00214	A02604	241
A00214	A02651	227	A00214	A02803	934
A00214	A02850	241	A00214	A02859	441
A00214	A02864	899	A00214	A02877	270
A00214	A02887	187	A00214	A02912	151
A00214	A02920	174	A00214	A02931	345
A00214	A02950	401	A00214	A02956	299
A00214	A02962	311	A00214	A02968	899
A00214	A02972	272	A00214	A02975	490
A00214	A02976	193	A00214	A02977	333

A00214	A03034	324	A00214	A03128	154
A00214	A03271	945	A00214	A03275	368
A00214	A03279	154	A00214	A03335	476
A00214	A03569	339	A00214	A03625	63
A00214	A03699	399	A00214	A03834	490
A00214	A03835	491	A00214	A03836	182
A00214	A03837	678	A00214	A03838	290
A00214	A03851	232	A00214	A03892	104
A00214	A03900	151	A00214	A03984	213
A00214	A04012	529	A00214	A04050	996
A00214	A04051	489	A00214	A04096	469
A00214	A04097	491	A00214	A04115	714
A00214	A04278	299	A00214	A04362	157
A00214	A04608	216	A00214	A04609	375
A00214	A04614	441	A00214	A04618	168
A00214	A04657	427	A00214	A04659	366
A00214	A04685	409	A00214	A04864	249
A00214	A05029	436	A00214	A05037	945
A00214	A05213	154	A00214	A05315	899
A00214	A05401	196	A00214	A05446	479
A00214	A05633	491	A00214	A05674	335
A00214	A05679	196	A00214	A05747	241
A00214	A05776	379	A00214	A05936	182
A00214	A05994	241	A00214	A06092	276
A00214	A06161	436	A00214	A06163	182
A00214	A06273	224	A00214	A06333	196
A00214	A06415	352	A00214	A06492	205
A00214	A06597	157	A00214	A06608	229
A00214	A06615	154	A00214	A06631	177
A00214	A06737	204	A00214	A06903	151
A00214	A07000	154	A00214	A07004	481
A00214	A07069	180	A00214	A07135	358
A00214	A07167	429	A00214	A07173	152
A00214	A07175	157	A00214	A07311	431
A00214	A07346	332	A00214	A07697	473
A00214	A07803	338	A00214	A08133	195
A00214	A08280	279	A00214	A08293	187
A00214	A08327	199	A00214	A08345	462
A00214	A08608	461	A00214	A08780	188
A00214	A08834	319	A00214	A08840	208
A00214	A08863	484	A00214	A08877	177
A00214	A09176	314	A00214	A09279	428
A00214	A09400	241	A00214	A09405	241
A00214	A09411	208	A00214	A09769	204
A00214	A09785	332	A00214	A09804	899
A00214	A09818	165	A00214	A09833	369

A00214	A09993	208	A00214	A10159	941
A00214	A10174	899	A00214	A10178	335
A00214	A10247	406	A00214	A10459	260
A00214	A10571	154	A00214	A10601	484
A00214	A10735	223	A00214	A10871	218
A00214	A11042	204	A00214	A11487	389
A00214	A11559	483	A00214	A11591	254
A00214	A11604	313	A00214	A11850	445
A00214	A11906	373	A00214	A11929	491
A00214	A11983	899	A00214	A12523	182
A00214	A12652	317	A00214	A12833	154
A00214	A12988	232	A00214	A13018	457
A00214	A13491	266	A00214	A13824	934
A00214	A13826	491	A00214	A13829	157
A00214	A13899	463	A00214	A13975	568
A00214	A13983	227	A00214	A13989	899
A00214	A14030	168	A00214	A14110	165
A00214	A14153	210	A00214	A14191	452
A00214	A14193	906	A00214	A14196	955
A00214	A14199	324	A00214	A14204	528
A00214	A14207	929	A00214	A14208	997
A00214	A14218	945	A00214	A14238	245
A00214	A14276	391	A00214	A14339	241
A00214	A14364	265	A00214	A14367	945
A00214	A14392	245	A00214	A14393	899
A00214	A14395	477	A00214	A14400	168
A00214	A14424	165	A00214	A14431	165
A00214	A14443	899	A00214	A14444	899
A00214	A14662	899	A00214	A14691	899
A00214	A14843	425	A00214	A15224	256
A00214	A15291	491	A00214	A15293	899
A00214	A15294	899	A00214	A15297	899
A00214	A15303	235	A00214	A15308	479
A00214	A15314	954	A00214	A15323	907
A00214	A15324	899	A00214	A15328	491
A00214	A15330	944	A00214	A15346	486
A00214	A15353	899	A00214	A15359	899
A00214	A15360	945	A00214	A15361	945
A00214	A15374	899	A00214	A15386	649
A00214	A15387	180	A00214	A15388	488
A00214	A15400	235	A00214	A15402	311
A00214	A15410	285	A00214	A15411	368
A00214	A15425	419	A00214	A15505	316
A00214	A15506	201	A00214	A15510	491
A00214	A15566	205	A00214	A15575	490
A00214	A15576	412	A00214	A15578	484

A00214	A15597	165	A00214	A15612	285
A00214	A15613	405	A00214	A15630	433
A00214	A15639	195	A00214	A15642	216
A00214	A15676	360	A00214	A15726	425
A00214	A15834	394	A00214	A15914	241
A00214	A15974	228	A00214	A15976	435
A00214	A16216	589	A00214	A16217	942
A00214	A16224	465	A00214	A16467	433
A00214	A16802	241	A00214	A17056	204
A00215	A00216	436	A00215	A00221	515
A00215	A00224	423	A00215	A00312	202
A00215	A00338	199	A00215	A00376	463
A00215	A00395	467	A00215	A00449	490
A00215	A00453	491	A00215	A00485	301
A00215	A00541	489	A00215	A00573	322
A00215	A00574	771	A00215	A00614	488
A00215	A00654	731	A00215	A00658	561
A00215	A00661	174	A00215	A00662	402
A00215	A00663	475	A00215	A00666	491
A00215	A00681	491	A00215	A00695	480
A00215	A00696	245	A00215	A00738	491
A00215	A00739	435	A00215	A00770	491
A00215	A00911	270	A00215	A00962	491
A00215	A00995	477	A00215	A01005	422
A00215	A01008	491	A00215	A01046	199
A00215	A01069	216	A00215	A01109	474
A00215	A01156	185	A00215	A01161	484
A00215	A01174	488	A00215	A01175	485
A00215	A01211	176	A00215	A01223	260
A00215	A01227	263	A00215	A01230	473
A00215	A01233	485	A00215	A01242	156
A00215	A01272	205	A00215	A01277	185
A00215	A01297	427	A00215	A01311	421
A00215	A01360	171	A00215	A01409	313
A00215	A01481	458	A00215	A01482	407
A00215	A01508	486	A00215	A01548	462
A00215	A01569	446	A00215	A01582	346
A00215	A01635	491	A00215	A01642	173
A00215	A01714	297	A00215	A01735	210
A00215	A01743	269	A00215	A01746	251
A00215	A01747	462	A00215	A01841	445
A00215	A01855	488	A00215	A01933	302
A00215	A01986	238	A00215	A02053	151
A00215	A02168	162	A00215	A02252	360
A00215	A02285	167	A00215	A02316	235
A00215	A02353	767	A00215	A02363	467

A00215	A02477	165	A00215	A02520	306
A00215	A02695	762	A00215	A02718	694
A00215	A02719	372	A00215	A02761	174
A00215	A02859	411	A00215	A02991	188
A00215	A02992	408	A00215	A03002	151
A00215	A03004	439	A00215	A03008	283
A00215	A03016	391	A00215	A03050	256
A00215	A03167	420	A00215	A03168	191
A00215	A03223	170	A00215	A03311	317
A00215	A03322	219	A00215	A03429	356
A00215	A03430	229	A00215	A03441	335
A00215	A03477	771	A00215	A03487	272
A00215	A03513	190	A00215	A03586	429
A00215	A03783	285	A00215	A03861	157
A00215	A03863	201	A00215	A03968	198
A00215	A04079	154	A00215	A04229	393
A00215	A04299	238	A00215	A04307	152
A00215	A04674	229	A00215	A04779	466
A00215	A04782	204	A00215	A04784	201
A00215	A04791	219	A00215	A04834	480
A00215	A04850	396	A00215	A04883	268
A00215	A04937	285	A00215	A04995	477
A00215	A05006	486	A00215	A05007	491
A00215	A05013	221	A00215	A05020	767
A00215	A05023	195	A00215	A05088	191
A00215	A05098	207	A00215	A05102	488
A00215	A05216	402	A00215	A05277	154
A00215	A05310	488	A00215	A05416	446
A00215	A05418	358	A00215	A05472	270
A00215	A05515	360	A00215	A05516	489
A00215	A05573	347	A00215	A05600	276
A00215	A05615	162	A00215	A05699	201
A00215	A05703	249	A00215	A05751	391
A00215	A05759	452	A00215	A05772	173
A00215	A05774	483	A00215	A05937	294
A00215	A06063	313	A00215	A06088	303
A00215	A06089	356	A00215	A06091	384
A00215	A06098	221	A00215	A06252	462
A00215	A06358	488	A00215	A06359	770
A00215	A06405	241	A00215	A06428	208
A00215	A06439	226	A00215	A06473	477
A00215	A06474	196	A00215	A06597	413
A00215	A06632	201	A00215	A06633	489
A00215	A06634	310	A00215	A06641	236
A00215	A06668	188	A00215	A06689	157
A00215	A06692	180	A00215	A06699	303

A00215	A06700	422	A00215	A06756	474
A00215	A06860	170	A00215	A06934	157
A00215	A06970	378	A00215	A07080	477
A00215	A07109	384	A00215	A07122	491
A00215	A07135	180	A00215	A07189	344
A00215	A07238	406	A00215	A07256	469
A00215	A07267	475	A00215	A07288	399
A00215	A07304	391	A00215	A07322	278
A00215	A07413	384	A00215	A07477	449
A00215	A07530	268	A00215	A07590	334
A00215	A07688	439	A00215	A07721	238
A00215	A07736	447	A00215	A07746	484
A00215	A07809	229	A00215	A07973	432
A00215	A08751	310	A00215	A08817	339
A00215	A09043	177	A00215	A09089	313
A00215	A09425	154	A00215	A09507	266
A00215	A09603	213	A00215	A09639	313
A00215	A09640	238	A00215	A09774	173
A00215	A09819	313	A00215	A10020	265
A00215	A10027	313	A00215	A10147	313
A00215	A10355	201	A00215	A10550	383
A00215	A10694	151	A00215	A11304	408
A00215	A11414	229	A00215	A11432	221
A00215	A11433	180	A00215	A11632	313
A00215	A11683	256	A00215	A12055	409
A00215	A13154	238	A00215	A13474	238
A00215	A13609	352	A00215	A14196	207
A00215	A14827	413	A00215	A15230	360
A00215	A15874	353	A00215	A15905	484
A00215	A16026	486	A00215	A16027	462
A00215	A16325	196	A00215	A16346	265
A00215	A16741	446	A00215	A17078	488
A00215	A17194	265	A00216	A00218	440
A00216	A00219	491	A00216	A00224	899
A00216	A00254	939	A00216	A00264	434
A00216	A00272	274	A00216	A00310	485
A00216	A00311	484	A00216	A00312	488
A00216	A00313	184	A00216	A00314	290
A00216	A00316	664	A00216	A00318	279
A00216	A00322	270	A00216	A00350	399
A00216	A00351	486	A00216	A00395	436
A00216	A00446	262	A00216	A00449	490
A00216	A00472	473	A00216	A00478	321
A00216	A00485	347	A00216	A00552	608
A00216	A00574	484	A00216	A00583	262
A00216	A00616	329	A00216	A00647	491

A00216	A00654	152	A00216	A00659	330
A00216	A00660	340	A00216	A00662	486
A00216	A00663	488	A00216	A00681	286
A00216	A00696	408	A00216	A00719	383
A00216	A00736	384	A00216	A00747	213
A00216	A00770	313	A00216	A00825	434
A00216	A00846	345	A00216	A00962	391
A00216	A00981	217	A00216	A01005	408
A00216	A01008	198	A00216	A01043	154
A00216	A01070	904	A00216	A01137	491
A00216	A01138	316	A00216	A01158	184
A00216	A01230	374	A00216	A01276	346
A00216	A01297	396	A00216	A01372	262
A00216	A01390	217	A00216	A01404	409
A00216	A01409	270	A00216	A01426	383
A00216	A01490	329	A00216	A01524	899
A00216	A01527	899	A00216	A01529	380
A00216	A01530	486	A00216	A01550	367
A00216	A01551	232	A00216	A01558	899
A00216	A01588	899	A00216	A01590	491
A00216	A01602	184	A00216	A01635	465
A00216	A01676	899	A00216	A01713	361
A00216	A01743	385	A00216	A01793	205
A00216	A01884	262	A00216	A01923	435
A00216	A01924	399	A00216	A01926	488
A00216	A01972	474	A00216	A01982	151
A00216	A01995	576	A00216	A02008	305
A00216	A02011	232	A00216	A02014	229
A00216	A02028	165	A00216	A02071	416
A00216	A02074	480	A00216	A02083	491
A00216	A02111	470	A00216	A02114	306
A00216	A02117	436	A00216	A02148	173
A00216	A02156	168	A00216	A02186	230
A00216	A02215	490	A00216	A02240	177
A00216	A02252	312	A00216	A02254	205
A00216	A02257	286	A00216	A02284	259
A00216	A02299	154	A00216	A02306	491
A00216	A02314	191	A00216	A02316	416
A00216	A02317	484	A00216	A02329	462
A00216	A02330	491	A00216	A02359	213
A00216	A02405	406	A00216	A02410	407
A00216	A02449	201	A00216	A02468	224
A00216	A02545	490	A00216	A02644	425
A00216	A02671	240	A00216	A02698	484
A00216	A02749	457	A00216	A02759	489
A00216	A02774	399	A00216	A02784	294

A00216	A02785	428	A00216	A02859	443
A00216	A02902	491	A00216	A02969	268
A00216	A02992	460	A00216	A03016	174
A00216	A03034	266	A00216	A03050	491
A00216	A03051	491	A00216	A03066	165
A00216	A03080	286	A00216	A03087	362
A00216	A03099	491	A00216	A03123	170
A00216	A03144	165	A00216	A03146	235
A00216	A03148	297	A00216	A03172	489
A00216	A03174	266	A00216	A03175	290
A00216	A03216	191	A00216	A03219	184
A00216	A03223	217	A00216	A03238	430
A00216	A03239	420	A00216	A03370	227
A00216	A03402	488	A00216	A03427	168
A00216	A03465	278	A00216	A03513	205
A00216	A03545	419	A00216	A03719	184
A00216	A03725	491	A00216	A03726	480
A00216	A03873	262	A00216	A03917	329
A00216	A03940	184	A00216	A03961	332
A00216	A03968	640	A00216	A04070	407
A00216	A04079	281	A00216	A04087	330
A00216	A04162	488	A00216	A04163	254
A00216	A04258	486	A00216	A04326	177
A00216	A04337	316	A00216	A04349	195
A00216	A04365	177	A00216	A04366	202
A00216	A04368	414	A00216	A04369	491
A00216	A04371	468	A00216	A04373	151
A00216	A04377	383	A00216	A04437	205
A00216	A04486	165	A00216	A04597	159
A00216	A04633	422	A00216	A04635	372
A00216	A04637	252	A00216	A04646	229
A00216	A04822	386	A00216	A04883	208
A00216	A04920	249	A00216	A04974	454
A00216	A04975	446	A00216	A04981	490
A00216	A05123	184	A00216	A05153	491
A00216	A05184	490	A00216	A05222	475
A00216	A05224	159	A00216	A05237	447
A00216	A05277	243	A00216	A05330	440
A00216	A05356	491	A00216	A05365	243
A00216	A05376	419	A00216	A05389	252
A00216	A05390	491	A00216	A05393	218
A00216	A05394	474	A00216	A05416	384
A00216	A05418	434	A00216	A05424	388
A00216	A05459	286	A00216	A05696	201
A00216	A05736	479	A00216	A05737	213
A00216	A05777	173	A00216	A05786	165

A00216	A05820	213	A00216	A05878	191
A00216	A05926	461	A00216	A05945	491
A00216	A05954	283	A00216	A05992	291
A00216	A06063	252	A00216	A06067	184
A00216	A06196	201	A00216	A06245	170
A00216	A06263	491	A00216	A06293	327
A00216	A06410	201	A00216	A06473	435
A00216	A06571	249	A00216	A06578	184
A00216	A06607	491	A00216	A06609	899
A00216	A06641	366	A00216	A06663	151
A00216	A06769	467	A00216	A06831	154
A00216	A06874	481	A00216	A06970	251
A00216	A06979	185	A00216	A07010	334
A00216	A07059	185	A00216	A07117	159
A00216	A07135	263	A00216	A07201	899
A00216	A07217	255	A00216	A07218	386
A00216	A07239	313	A00216	A07296	177
A00216	A07316	283	A00216	A07339	182
A00216	A07407	436	A00216	A07452	291
A00216	A07455	213	A00216	A07468	491
A00216	A07469	380	A00216	A07471	396
A00216	A07520	269	A00216	A07529	269
A00216	A07541	274	A00216	A07548	356
A00216	A07564	229	A00216	A07567	490
A00216	A07598	184	A00216	A07617	246
A00216	A07638	283	A00216	A07652	323
A00216	A07653	329	A00216	A07654	422
A00216	A07673	457	A00216	A07754	435
A00216	A07764	344	A00216	A07808	249
A00216	A07912	429	A00216	A07913	462
A00216	A07928	327	A00216	A07956	262
A00216	A07958	162	A00216	A08002	317
A00216	A08248	262	A00216	A08310	396
A00216	A08404	491	A00216	A08746	412
A00216	A08785	439	A00216	A08878	899
A00216	A08982	262	A00216	A09072	945
A00216	A09080	201	A00216	A09119	283
A00216	A09173	229	A00216	A09351	184
A00216	A09561	154	A00216	A09791	383
A00216	A10013	229	A00216	A10105	184
A00216	A10252	249	A00216	A10346	291
A00216	A10700	165	A00216	A10757	154
A00216	A10967	491	A00216	A11315	329
A00216	A11424	375	A00216	A11466	191
A00216	A11544	262	A00216	A11558	184
A00216	A11646	260	A00216	A11649	262

A00216	A11851	184	A00216	A11911	358
A00216	A11983	899	A00216	A12115	249
A00216	A12622	283	A00216	A12793	480
A00216	A12909	283	A00216	A13140	191
A00216	A13258	249	A00216	A13280	213
A00216	A13327	303	A00216	A13329	201
A00216	A13356	184	A00216	A13734	299
A00216	A13892	262	A00216	A14071	180
A00216	A14198	899	A00216	A14210	184
A00216	A14362	268	A00216	A14380	165
A00216	A14381	323	A00216	A14436	165
A00216	A14443	899	A00216	A14600	184
A00216	A14625	151	A00216	A14652	333
A00216	A14767	283	A00216	A15060	201
A00216	A15293	908	A00216	A15294	899
A00216	A15321	217	A00216	A15338	262
A00216	A15422	160	A00216	A15475	179
A00216	A15498	291	A00216	A15532	346
A00216	A15564	262	A00216	A16152	184
A00216	A16171	229	A00216	A16174	213
A00216	A16195	213	A00216	A16562	283
A00216	A16614	262	A00216	A16741	174
A00216	A17052	283	A00216	A17078	152
A00216	A17108	435	A00217	A00221	187
A00217	A00252	441	A00217	A00267	265
A00217	A00273	481	A00217	A00407	185
A00217	A01281	418	A00217	A01512	195
A00217	A01550	254	A00217	A01553	168
A00217	A01559	414	A00217	A01572	436
A00217	A01592	491	A00217	A01594	285
A00217	A01604	307	A00217	A01605	151
A00217	A01628	337	A00217	A01640	424
A00217	A01660	345	A00217	A01702	180
A00217	A03057	367	A00217	A04452	303
A00217	A04668	215	A00217	A04809	475
A00217	A04957	200	A00217	A06253	167
A00217	A06919	154	A00217	A08827	190
A00217	A08899	221	A00217	A08926	159
A00217	A09111	252	A00217	A09124	218
A00217	A09162	234	A00217	A09413	196
A00217	A09644	276	A00217	A09752	165
A00217	A09866	252	A00217	A11549	400
A00217	A11574	252	A00217	A11767	201
A00217	A12014	294	A00217	A13741	460
A00217	A14009	490	A00217	A14594	249
A00217	A14694	389	A00217	A15030	208

A00217	A16051	246	A00217	A17075	221
A00218	A00219	452	A00218	A00227	486
A00218	A00258	562	A00218	A00264	489
A00218	A00294	491	A00218	A00301	348
A00218	A00311	243	A00218	A00338	488
A00218	A00359	451	A00218	A00385	290
A00218	A00449	456	A00218	A00472	491
A00218	A00485	348	A00218	A00556	226
A00218	A00656	491	A00218	A00659	312
A00218	A00662	329	A00218	A00663	196
A00218	A00688	177	A00218	A01138	347
A00218	A01157	185	A00218	A01209	185
A00218	A01478	270	A00218	A01526	491
A00218	A01556	491	A00218	A01578	454
A00218	A01640	491	A00218	A01663	163
A00218	A01726	373	A00218	A01741	443
A00218	A01743	483	A00218	A01758	201
A00218	A01900	191	A00218	A01982	491
A00218	A02000	490	A00218	A02010	291
A00218	A02021	481	A00218	A02046	299
A00218	A02048	491	A00218	A02049	488
A00218	A02071	260	A00218	A02126	182
A00218	A02146	486	A00218	A02148	481
A00218	A02157	159	A00218	A02180	473
A00218	A02183	481	A00218	A02186	490
A00218	A02205	486	A00218	A02215	491
A00218	A02223	435	A00218	A02238	302
A00218	A02291	382	A00218	A02295	213
A00218	A02300	461	A00218	A02328	232
A00218	A02329	455	A00218	A02450	348
A00218	A02478	232	A00218	A02490	223
A00218	A02542	174	A00218	A02575	390
A00218	A02587	154	A00218	A02620	154
A00218	A02668	252	A00218	A02756	390
A00218	A02774	468	A00218	A02785	272
A00218	A02859	480	A00218	A02874	243
A00218	A02876	310	A00218	A02951	379
A00218	A02977	358	A00218	A02982	208
A00218	A03028	223	A00218	A03034	389
A00218	A03067	439	A00218	A03070	252
A00218	A03071	452	A00218	A03080	491
A00218	A03107	232	A00218	A03186	435
A00218	A03259	196	A00218	A03346	490
A00218	A03347	491	A00218	A03348	447
A00218	A03349	307	A00218	A03351	491
A00218	A03353	491	A00218	A03357	160

A00218	A03358	468	A00218	A03360	210
A00218	A03361	491	A00218	A03362	188
A00218	A03363	368	A00218	A03365	383
A00218	A03369	491	A00218	A03370	491
A00218	A03371	491	A00218	A03373	491
A00218	A03374	378	A00218	A03375	348
A00218	A03376	477	A00218	A03378	490
A00218	A03379	491	A00218	A03388	236
A00218	A03390	484	A00218	A03391	485
A00218	A03397	364	A00218	A03401	479
A00218	A03402	230	A00218	A03414	274
A00218	A03499	201	A00218	A03504	193
A00218	A03532	443	A00218	A03697	274
A00218	A03729	486	A00218	A03733	491
A00218	A03744	484	A00218	A03755	177
A00218	A03895	163	A00218	A04051	491
A00218	A04070	348	A00218	A04154	348
A00218	A04159	219	A00218	A04193	234
A00218	A04278	475	A00218	A04329	247
A00218	A04337	353	A00218	A04338	266
A00218	A04344	188	A00218	A04385	316
A00218	A04407	154	A00218	A04409	418
A00218	A04456	266	A00218	A04614	179
A00218	A04617	160	A00218	A04618	254
A00218	A04672	246	A00218	A04708	219
A00218	A04790	406	A00218	A04830	327
A00218	A04875	168	A00218	A04882	383
A00218	A04923	369	A00218	A04928	195
A00218	A04989	182	A00218	A05000	266
A00218	A05084	193	A00218	A05117	151
A00218	A05136	334	A00218	A05156	172
A00218	A05189	266	A00218	A05286	266
A00218	A05356	429	A00218	A05382	232
A00218	A05390	200	A00218	A05427	461
A00218	A05457	340	A00218	A05469	210
A00218	A05543	246	A00218	A05578	163
A00218	A05612	188	A00218	A05716	160
A00218	A05723	219	A00218	A05731	489
A00218	A05968	172	A00218	A05999	201
A00218	A06069	177	A00218	A06192	297
A00218	A06322	266	A00218	A06346	416
A00218	A06401	177	A00218	A06483	297
A00218	A06527	154	A00218	A06574	279
A00218	A06597	259	A00218	A06606	191
A00218	A06642	486	A00218	A06712	232
A00218	A06729	407	A00218	A06782	210

A00218	A06838	172	A00218	A06935	246
A00218	A07066	193	A00218	A07218	491
A00218	A07249	446	A00218	A07442	247
A00218	A07561	228	A00218	A07572	418
A00218	A07601	296	A00218	A07605	238
A00218	A07620	210	A00218	A08033	297
A00218	A08034	201	A00218	A08295	232
A00218	A09067	193	A00218	A09141	400
A00218	A09951	266	A00218	A09983	290
A00218	A10000	369	A00218	A10666	402
A00218	A10937	232	A00218	A10948	297
A00218	A11031	297	A00218	A11154	383
A00218	A11313	210	A00218	A11317	480
A00218	A11394	160	A00218	A11446	290
A00218	A11558	193	A00218	A11573	151
A00218	A11599	266	A00218	A11713	163
A00218	A11872	163	A00218	A11879	246
A00218	A12019	369	A00218	A12351	266
A00218	A12430	297	A00218	A12459	463
A00218	A12496	297	A00218	A12594	297
A00218	A12917	210	A00218	A12982	302
A00218	A12991	266	A00218	A13124	323
A00218	A13597	296	A00218	A13734	465
A00218	A13851	188	A00218	A14128	655
A00218	A14190	569	A00218	A14399	291
A00218	A14430	474	A00218	A14661	179
A00218	A14820	193	A00218	A15649	177
A00218	A15880	160	A00218	A15911	276
A00218	A16040	232	A00218	A16636	259
A00218	A16811	201	A00218	A16967	219
A00219	A00221	423	A00219	A00223	168
A00219	A00224	945	A00219	A00227	380
A00219	A00233	281	A00219	A00234	451
A00219	A00240	469	A00219	A00245	491
A00219	A00246	901	A00219	A00256	326
A00219	A00264	491	A00219	A00274	489
A00219	A00292	249	A00219	A00305	176
A00219	A00310	347	A00219	A00311	475
A00219	A00312	484	A00219	A00314	160
A00219	A00315	441	A00219	A00316	485
A00219	A00324	943	A00219	A00338	454
A00219	A00349	177	A00219	A00350	486
A00219	A00351	481	A00219	A00370	279
A00219	A00377	943	A00219	A00420	491
A00219	A00449	232	A00219	A00454	156
A00219	A00455	193	A00219	A00470	899

A00219	A00472	491	A00219	A00556	490
A00219	A00558	243	A00219	A00568	465
A00219	A00597	171	A00219	A00602	168
A00219	A00616	491	A00219	A00659	337
A00219	A00669	262	A00219	A00688	434
A00219	A00736	491	A00219	A00747	180
A00219	A00752	490	A00219	A00786	177
A00219	A00815	356	A00219	A00820	480
A00219	A00832	462	A00219	A00914	491
A00219	A01032	485	A00219	A01091	184
A00219	A01125	614	A00219	A01137	491
A00219	A01138	385	A00219	A01161	198
A00219	A01162	216	A00219	A01163	307
A00219	A01164	488	A00219	A01213	319
A00219	A01230	162	A00219	A01232	190
A00219	A01235	363	A00219	A01257	491
A00219	A01277	396	A00219	A01278	400
A00219	A01379	168	A00219	A01404	484
A00219	A01425	577	A00219	A01461	303
A00219	A01483	215	A00219	A01485	474
A00219	A01515	657	A00219	A01518	426
A00219	A01520	465	A00219	A01522	839
A00219	A01524	899	A00219	A01530	561
A00219	A01551	196	A00219	A01553	183
A00219	A01556	358	A00219	A01567	232
A00219	A01571	446	A00219	A01572	433
A00219	A01590	491	A00219	A01593	934
A00219	A01601	229	A00219	A01602	379
A00219	A01604	372	A00219	A01605	945
A00219	A01626	193	A00219	A01640	460
A00219	A01668	491	A00219	A01676	474
A00219	A01740	182	A00219	A01741	945
A00219	A01743	488	A00219	A01747	227
A00219	A01775	481	A00219	A01790	230
A00219	A01796	491	A00219	A01797	416
A00219	A01817	235	A00219	A01844	323
A00219	A01894	490	A00219	A01903	445
A00219	A01907	319	A00219	A01923	217
A00219	A01924	224	A00219	A01926	491
A00219	A01931	238	A00219	A01937	238
A00219	A01939	162	A00219	A01944	177
A00219	A01983	477	A00219	A02048	205
A00219	A02074	490	A00219	A02083	491
A00219	A02086	187	A00219	A02088	188
A00219	A02092	440	A00219	A02107	256
A00219	A02111	491	A00219	A02117	483

A00219	A02138	485	A00219	A02142	480
A00219	A02143	489	A00219	A02145	278
A00219	A02148	859	A00219	A02156	484
A00219	A02161	223	A00219	A02174	486
A00219	A02176	196	A00219	A02180	399
A00219	A02196	207	A00219	A02215	477
A00219	A02222	480	A00219	A02228	353
A00219	A02229	485	A00219	A02245	434
A00219	A02268	401	A00219	A02271	207
A00219	A02278	294	A00219	A02284	224
A00219	A02298	483	A00219	A02300	216
A00219	A02304	227	A00219	A02305	484
A00219	A02306	491	A00219	A02309	481
A00219	A02313	314	A00219	A02316	159
A00219	A02317	468	A00219	A02326	446
A00219	A02329	202	A00219	A02330	491
A00219	A02331	204	A00219	A02356	401
A00219	A02377	185	A00219	A02381	288
A00219	A02382	480	A00219	A02383	171
A00219	A02390	276	A00219	A02393	279
A00219	A02397	353	A00219	A02405	488
A00219	A02411	899	A00219	A02412	462
A00219	A02416	461	A00219	A02423	367
A00219	A02425	461	A00219	A02429	151
A00219	A02451	223	A00219	A02454	446
A00219	A02460	243	A00219	A02461	466
A00219	A02464	452	A00219	A02465	314
A00219	A02467	246	A00219	A02468	232
A00219	A02481	185	A00219	A02490	490
A00219	A02491	490	A00219	A02493	213
A00219	A02494	398	A00219	A02501	154
A00219	A02505	425	A00219	A02544	223
A00219	A02545	440	A00219	A02552	440
A00219	A02558	274	A00219	A02572	170
A00219	A02582	402	A00219	A02587	395
A00219	A02618	468	A00219	A02624	252
A00219	A02644	491	A00219	A02660	171
A00219	A02667	485	A00219	A02668	481
A00219	A02672	364	A00219	A02673	205
A00219	A02698	168	A00219	A02711	174
A00219	A02749	394	A00219	A02756	408
A00219	A02759	491	A00219	A02771	156
A00219	A02772	435	A00219	A02785	491
A00219	A02787	488	A00219	A02788	484
A00219	A02789	490	A00219	A02791	205
A00219	A02803	191	A00219	A02836	379

A00219	A02837	314	A00219	A02859	465
A00219	A02874	485	A00219	A02875	196
A00219	A02877	156	A00219	A02878	193
A00219	A02888	221	A00219	A02896	308
A00219	A02908	899	A00219	A02935	241
A00219	A02945	232	A00219	A02959	411
A00219	A02982	274	A00219	A02983	240
A00219	A03003	312	A00219	A03034	384
A00219	A03063	223	A00219	A03080	348
A00219	A03082	170	A00219	A03086	279
A00219	A03087	212	A00219	A03095	188
A00219	A03099	491	A00219	A03103	168
A00219	A03104	467	A00219	A03110	489
A00219	A03112	457	A00219	A03144	213
A00219	A03174	159	A00219	A03183	408
A00219	A03186	491	A00219	A03219	247
A00219	A03223	443	A00219	A03238	432
A00219	A03259	472	A00219	A03304	152
A00219	A03305	353	A00219	A03310	418
A00219	A03314	491	A00219	A03319	162
A00219	A03321	196	A00219	A03402	486
A00219	A03408	362	A00219	A03451	384
A00219	A03458	283	A00219	A03459	168
A00219	A03511	173	A00219	A03520	168
A00219	A03578	243	A00219	A03584	477
A00219	A03586	326	A00219	A03633	380
A00219	A03639	489	A00219	A03653	435
A00219	A03725	461	A00219	A03726	945
A00219	A03739	291	A00219	A03757	341
A00219	A03760	168	A00219	A03766	481
A00219	A03771	184	A00219	A03788	480
A00219	A03799	486	A00219	A03800	375
A00219	A03818	475	A00219	A03854	185
A00219	A03893	418	A00219	A03910	316
A00219	A03914	167	A00219	A03919	446
A00219	A03942	486	A00219	A03961	491
A00219	A03968	456	A00219	A03976	396
A00219	A04002	451	A00219	A04037	190
A00219	A04050	899	A00219	A04051	327
A00219	A04079	195	A00219	A04092	485
A00219	A04101	215	A00219	A04117	440
A00219	A04157	414	A00219	A04162	488
A00219	A04163	488	A00219	A04169	391
A00219	A04180	319	A00219	A04196	184
A00219	A04202	491	A00219	A04230	249
A00219	A04256	454	A00219	A04258	472

A00219	A04295	207	A00219	A04318	168
A00219	A04326	213	A00219	A04343	177
A00219	A04348	196	A00219	A04366	182
A00219	A04368	159	A00219	A04371	205
A00219	A04373	281	A00219	A04378	271
A00219	A04379	363	A00219	A04387	207
A00219	A04437	466	A00219	A04454	162
A00219	A04482	288	A00219	A04487	221
A00219	A04490	188	A00219	A04504	213
A00219	A04562	215	A00219	A04622	470
A00219	A04679	446	A00219	A04746	266
A00219	A04749	154	A00219	A04751	945
A00219	A04770	393	A00219	A04780	420
A00219	A04813	457	A00219	A04822	398
A00219	A04888	273	A00219	A04965	347
A00219	A04974	301	A00219	A04975	401
A00219	A04976	433	A00219	A04981	480
A00219	A05049	207	A00219	A05117	256
A00219	A05151	207	A00219	A05157	168
A00219	A05158	286	A00219	A05208	458
A00219	A05221	341	A00219	A05224	190
A00219	A05277	232	A00219	A05288	480
A00219	A05311	358	A00219	A05315	177
A00219	A05330	318	A00219	A05355	173
A00219	A05356	154	A00219	A05376	402
A00219	A05377	485	A00219	A05408	168
A00219	A05409	260	A00219	A05439	382
A00219	A05446	383	A00219	A05459	363
A00219	A05533	475	A00219	A05577	353
A00219	A05587	159	A00219	A05653	162
A00219	A05658	207	A00219	A05706	221
A00219	A05736	228	A00219	A05744	168
A00219	A05815	190	A00219	A05891	162
A00219	A05923	191	A00219	A05985	296
A00219	A06033	369	A00219	A06037	188
A00219	A06086	168	A00219	A06160	168
A00219	A06248	168	A00219	A06263	485
A00219	A06581	234	A00219	A06588	490
A00219	A06591	195	A00219	A06601	182
A00219	A06607	491	A00219	A06608	294
A00219	A06610	457	A00219	A06615	307
A00219	A06642	160	A00219	A06650	480
A00219	A06688	412	A00219	A06729	446
A00219	A06731	468	A00219	A06769	490
A00219	A06798	235	A00219	A06831	184
A00219	A06874	462	A00219	A06953	374

A00219	A07109	383	A00219	A07135	943
A00219	A07138	404	A00219	A07140	389
A00219	A07141	165	A00219	A07143	491
A00219	A07157	467	A00219	A07158	313
A00219	A07164	296	A00219	A07165	441
A00219	A07200	416	A00219	A07209	491
A00219	A07210	362	A00219	A07339	466
A00219	A07343	207	A00219	A07353	290
A00219	A07407	151	A00219	A07577	168
A00219	A07601	173	A00219	A07605	277
A00219	A07641	177	A00219	A07650	281
A00219	A07653	212	A00219	A07745	207
A00219	A07803	489	A00219	A07833	314
A00219	A07834	305	A00219	A07912	301
A00219	A08280	260	A00219	A08289	162
A00219	A08311	260	A00219	A08404	302
A00219	A08455	168	A00219	A08551	276
A00219	A08714	407	A00219	A08738	262
A00219	A08746	436	A00219	A08749	207
A00219	A08826	302	A00219	A08851	491
A00219	A08859	491	A00219	A08864	401
A00219	A08949	160	A00219	A09013	207
A00219	A09028	457	A00219	A09072	286
A00219	A09125	279	A00219	A09212	246
A00219	A09217	260	A00219	A09260	207
A00219	A09442	159	A00219	A09467	260
A00219	A09480	256	A00219	A09509	232
A00219	A09539	212	A00219	A09540	252
A00219	A09550	173	A00219	A09559	307
A00219	A09732	417	A00219	A09733	489
A00219	A09797	278	A00219	A09886	168
A00219	A09890	151	A00219	A09942	207
A00219	A10002	260	A00219	A10004	207
A00219	A10010	207	A00219	A10018	241
A00219	A10044	404	A00219	A10045	404
A00219	A10046	412	A00219	A10074	168
A00219	A10145	260	A00219	A10237	205
A00219	A10278	902	A00219	A10336	491
A00219	A10508	154	A00219	A10540	205
A00219	A10594	152	A00219	A10609	317
A00219	A10611	177	A00219	A10625	201
A00219	A10725	243	A00219	A10743	312
A00219	A10963	168	A00219	A11123	151
A00219	A11124	899	A00219	A11132	385
A00219	A11313	485	A00219	A11415	313
A00219	A11424	184	A00219	A11446	162

A00219	A11485	380	A00219	A11523	162
A00219	A11548	168	A00219	A11602	302
A00219	A11614	358	A00219	A11683	229
A00219	A11787	168	A00219	A12261	168
A00219	A12317	207	A00219	A12361	177
A00219	A12393	385	A00219	A12412	168
A00219	A12578	207	A00219	A12622	207
A00219	A12897	458	A00219	A13140	234
A00219	A13176	207	A00219	A13224	168
A00219	A13225	899	A00219	A13252	207
A00219	A13281	207	A00219	A13310	165
A00219	A13734	216	A00219	A13812	152
A00219	A13829	330	A00219	A13849	234
A00219	A13860	294	A00219	A13904	168
A00219	A13944	171	A00219	A13987	182
A00219	A13994	446	A00219	A14072	380
A00219	A14111	168	A00219	A14135	162
A00219	A14188	366	A00219	A14190	485
A00219	A14198	219	A00219	A14202	265
A00219	A14210	491	A00219	A14258	446
A00219	A14371	491	A00219	A14379	371
A00219	A14399	174	A00219	A14405	306
A00219	A14430	190	A00219	A14440	388
A00219	A14443	912	A00219	A14483	212
A00219	A14511	491	A00219	A14554	408
A00219	A14570	191	A00219	A14667	205
A00219	A14733	193	A00219	A14892	207
A00219	A14930	477	A00219	A15024	207
A00219	A15089	168	A00219	A15230	353
A00219	A15233	188	A00219	A15268	420
A00219	A15292	486	A00219	A15295	660
A00219	A15312	404	A00219	A15313	462
A00219	A15318	426	A00219	A15348	162
A00219	A15349	419	A00219	A15352	318
A00219	A15381	420	A00219	A15422	245
A00219	A15463	180	A00219	A15484	221
A00219	A15495	490	A00219	A15498	491
A00219	A15506	160	A00219	A15598	457
A00219	A15630	210	A00219	A15726	439
A00219	A15913	491	A00219	A16001	207
A00219	A16114	171	A00219	A16257	401
A00219	A16300	252	A00219	A16636	491
A00219	A17076	163	A00219	A17106	207
A00219	A17155	218	A00219	A17183	207
A00220	A00221	945	A00220	A00222	360
A00220	A00223	994	A00220	A00230	899

A00220	A00231	210	A00220	A00246	899
A00220	A00274	427	A00220	A00318	205
A00220	A00476	319	A00220	A00616	274
A00220	A00694	323	A00220	A00752	443
A00220	A00870	408	A00220	A00914	210
A00220	A01009	446	A00220	A01011	470
A00220	A01198	369	A00220	A01425	308
A00220	A01524	447	A00220	A01527	918
A00220	A01553	899	A00220	A01558	899
A00220	A01604	945	A00220	A01615	899
A00220	A01640	942	A00220	A01654	490
A00220	A01669	154	A00220	A01755	160
A00220	A01867	210	A00220	A01870	486
A00220	A01877	221	A00220	A01880	468
A00220	A02012	210	A00220	A02065	185
A00220	A02074	380	A00220	A02105	229
A00220	A02111	372	A00220	A02247	307
A00220	A02251	383	A00220	A02333	490
A00220	A02337	475	A00220	A02370	374
A00220	A02381	258	A00220	A02382	484
A00220	A02384	386	A00220	A02416	475
A00220	A02457	210	A00220	A02458	277
A00220	A02459	193	A00220	A02460	337
A00220	A02490	460	A00220	A02491	490
A00220	A02494	165	A00220	A02498	215
A00220	A02502	154	A00220	A02505	165
A00220	A02590	212	A00220	A02592	165
A00220	A02606	425	A00220	A02608	260
A00220	A02617	378	A00220	A02630	213
A00220	A02637	427	A00220	A02639	163
A00220	A02654	154	A00220	A02657	294
A00220	A02659	416	A00220	A02661	262
A00220	A02711	174	A00220	A02731	180
A00220	A02732	193	A00220	A02751	218
A00220	A02761	174	A00220	A02764	274
A00220	A02812	177	A00220	A02851	252
A00220	A02854	389	A00220	A02863	384
A00220	A02896	205	A00220	A02917	455
A00220	A02945	463	A00220	A02977	196
A00220	A03006	324	A00220	A03035	246
A00220	A03049	290	A00220	A03100	157
A00220	A03109	230	A00220	A03177	177
A00220	A03224	400	A00220	A03228	322
A00220	A03316	276	A00220	A03451	216
A00220	A03610	202	A00220	A03753	323
A00220	A03771	157	A00220	A03817	424

A00220	A03898	358	A00220	A03919	243
A00220	A03937	202	A00220	A03976	348
A00220	A04082	232	A00220	A04130	154
A00220	A04217	180	A00220	A04238	288
A00220	A04244	425	A00220	A04248	490
A00220	A04263	294	A00220	A04320	227
A00220	A04398	281	A00220	A04415	363
A00220	A04478	422	A00220	A04490	193
A00220	A04492	358	A00220	A04854	337
A00220	A04909	238	A00220	A05078	285
A00220	A05124	176	A00220	A05305	351
A00220	A05679	353	A00220	A05815	297
A00220	A05944	491	A00220	A05959	294
A00220	A05985	477	A00220	A06054	485
A00220	A06055	319	A00220	A06095	267
A00220	A06404	384	A00220	A06414	427
A00220	A06415	263	A00220	A06780	291
A00220	A06818	374	A00220	A06968	160
A00220	A07143	174	A00220	A07145	286
A00220	A07151	182	A00220	A07157	641
A00220	A07161	396	A00220	A07166	277
A00220	A07173	481	A00220	A07311	899
A00220	A07372	396	A00220	A07390	241
A00220	A08012	302	A00220	A08280	477
A00220	A08518	154	A00220	A08580	347
A00220	A08585	347	A00220	A08612	413
A00220	A08714	191	A00220	A08908	229
A00220	A09259	257	A00220	A09419	422
A00220	A09939	274	A00220	A10214	532
A00220	A10249	323	A00220	A10319	152
A00220	A10469	317	A00220	A10760	170
A00220	A11096	262	A00220	A11300	532
A00220	A11309	260	A00220	A11544	202
A00220	A11561	276	A00220	A11881	335
A00220	A11911	221	A00220	A11932	313
A00220	A11983	428	A00220	A12913	347
A00220	A13860	523	A00220	A13872	405
A00220	A13886	251	A00220	A14038	428
A00220	A14231	515	A00220	A14236	260
A00220	A14266	167	A00220	A14366	443
A00220	A14373	274	A00220	A14376	251
A00220	A14392	247	A00220	A14439	185
A00220	A14442	221	A00220	A14443	899
A00220	A14554	281	A00220	A14598	241
A00220	A14599	199	A00220	A15323	401
A00220	A15394	165	A00220	A15395	419

A00220	A15425	474	A00220	A15427	202
A00220	A15518	269	A00220	A15601	422
A00220	A15603	475	A00220	A15605	477
A00220	A15607	247	A00220	A15608	441
A00220	A15642	306	A00220	A15795	160
A00220	A15832	323	A00220	A15958	358
A00220	A15964	218	A00220	A15981	243
A00220	A16010	378	A00220	A16024	323
A00221	A00222	425	A00221	A00223	945
A00221	A00229	308	A00221	A00230	899
A00221	A00231	183	A00221	A00234	472
A00221	A00254	435	A00221	A00255	157
A00221	A00264	357	A00221	A00292	485
A00221	A00335	279	A00221	A00356	277
A00221	A00400	299	A00221	A00574	646
A00221	A00601	168	A00221	A00616	462
A00221	A00654	420	A00221	A00688	198
A00221	A00697	375	A00221	A00752	174
A00221	A00868	299	A00221	A00870	399
A00221	A00926	195	A00221	A01056	219
A00221	A01098	190	A00221	A01127	256
A00221	A01140	221	A00221	A01161	210
A00221	A01172	184	A00221	A01310	408
A00221	A01339	152	A00221	A01425	466
A00221	A01500	270	A00221	A01524	989
A00221	A01527	903	A00221	A01536	491
A00221	A01558	899	A00221	A01561	899
A00221	A01568	332	A00221	A01604	486
A00221	A01605	388	A00221	A01615	899
A00221	A01640	490	A00221	A01652	162
A00221	A01692	358	A00221	A01908	473
A00221	A01939	200	A00221	A01978	165
A00221	A02054	330	A00221	A02058	446
A00221	A02088	229	A00221	A02111	173
A00221	A02151	422	A00221	A02170	249
A00221	A02243	188	A00221	A02251	369
A00221	A02278	246	A00221	A02333	469
A00221	A02353	633	A00221	A02369	200
A00221	A02419	327	A00221	A02425	314
A00221	A02465	185	A00221	A02490	445
A00221	A02491	489	A00221	A02494	207
A00221	A02507	172	A00221	A02547	362
A00221	A02570	252	A00221	A02592	475
A00221	A02597	216	A00221	A02608	176
A00221	A02718	500	A00221	A02730	216
A00221	A02769	172	A00221	A02803	445

A00221	A02812	223	A00221	A03029	276
A00221	A03071	279	A00221	A03110	268
A00221	A03165	165	A00221	A03171	157
A00221	A03228	391	A00221	A03271	474
A00221	A03470	281	A00221	A03477	737
A00221	A03591	623	A00221	A03639	162
A00221	A03731	191	A00221	A03744	174
A00221	A03798	443	A00221	A03800	330
A00221	A03817	485	A00221	A03826	321
A00221	A03937	241	A00221	A03942	154
A00221	A04016	252	A00221	A04069	236
A00221	A04073	491	A00221	A04161	234
A00221	A04169	172	A00221	A04218	174
A00221	A04230	219	A00221	A04249	196
A00221	A04398	942	A00221	A04422	489
A00221	A04450	212	A00221	A04471	193
A00221	A04490	490	A00221	A04492	277
A00221	A04755	188	A00221	A04819	230
A00221	A04827	230	A00221	A04879	350
A00221	A04914	232	A00221	A04917	252
A00221	A05020	735	A00221	A05027	497
A00221	A05082	174	A00221	A05091	302
A00221	A05129	285	A00221	A05183	212
A00221	A05276	160	A00221	A05337	353
A00221	A05373	260	A00221	A05407	408
A00221	A05422	236	A00221	A05606	406
A00221	A05670	396	A00221	A05815	221
A00221	A05944	281	A00221	A06037	483
A00221	A06054	430	A00221	A06286	193
A00221	A06306	313	A00221	A06359	723
A00221	A06392	329	A00221	A06769	152
A00221	A06814	330	A00221	A06852	252
A00221	A06899	176	A00221	A06902	329
A00221	A07135	302	A00221	A07138	165
A00221	A07143	204	A00221	A07164	246
A00221	A07174	160	A00221	A07390	154
A00221	A07751	243	A00221	A08052	374
A00221	A08199	380	A00221	A08293	380
A00221	A08570	168	A00221	A08574	226
A00221	A08882	335	A00221	A08932	185
A00221	A09077	221	A00221	A09218	943
A00221	A09295	236	A00221	A09305	221
A00221	A09644	366	A00221	A09672	468
A00221	A09983	200	A00221	A10231	443
A00221	A10599	218	A00221	A10668	216
A00221	A10679	162	A00221	A10756	945

A00221	A10923	216	A00221	A11086	196
A00221	A11422	165	A00221	A11561	193
A00221	A11628	160	A00221	A11771	183
A00221	A11792	177	A00221	A11940	165
A00221	A12155	285	A00221	A12215	185
A00221	A12658	367	A00221	A13162	364
A00221	A13225	899	A00221	A13546	151
A00221	A13904	302	A00221	A13983	338
A00221	A14190	488	A00221	A14194	344
A00221	A14196	299	A00221	A14198	340
A00221	A14210	390	A00221	A14260	162
A00221	A14371	276	A00221	A14438	165
A00221	A14511	283	A00221	A15292	167
A00221	A15294	299	A00221	A15498	246
A00221	A15607	162	A00221	A16024	252
A00221	A16343	221	A00221	A17019	230
A00222	A00223	491	A00222	A00292	174
A00222	A00595	246	A00222	A00651	283
A00222	A00690	165	A00222	A00700	205
A00222	A00870	77	A00222	A00916	344
A00222	A00992	79	A00222	A01053	174
A00222	A01065	246	A00222	A01092	486
A00222	A01098	491	A00222	A01120	293
A00222	A01127	190	A00222	A01140	301
A00222	A01206	205	A00222	A01234	230
A00222	A01291	363	A00222	A01351	96
A00222	A01425	486	A00222	A01457	174
A00222	A01468	224	A00222	A01470	208
A00222	A01492	491	A00222	A01496	110
A00222	A01598	207	A00222	A01599	177
A00222	A01649	241	A00222	A01877	215
A00222	A02164	63	A00222	A02179	374
A00222	A02250	185	A00222	A02265	258
A00222	A02355	249	A00222	A02414	436
A00222	A02415	294	A00222	A02494	259
A00222	A02584	263	A00222	A02895	173
A00222	A03338	188	A00222	A03438	101
A00222	A03571	172	A00222	A04230	313
A00222	A05370	330	A00222	A06821	221
A00222	A06825	265	A00222	A07139	324
A00222	A07157	210	A00222	A07422	912
A00222	A08012	296	A00222	A11853	268
A00222	A13126	149	A00222	A13989	490
A00222	A14428	63	A00222	A15764	180
A00223	A00236	491	A00223	A00252	491
A00223	A00253	491	A00223	A00265	188

A00223	A00268	486	A00223	A00277	285
A00223	A00283	469	A00223	A00292	252
A00223	A00304	401	A00223	A00327	470
A00223	A00328	490	A00223	A00334	162
A00223	A00335	741	A00223	A00356	746
A00223	A00357	171	A00223	A00358	441
A00223	A00362	324	A00223	A00414	263
A00223	A00501	286	A00223	A00504	157
A00223	A00531	358	A00223	A00571	229
A00223	A00589	389	A00223	A00603	162
A00223	A00605	477	A00223	A00648	477
A00223	A00651	491	A00223	A00653	216
A00223	A00700	486	A00223	A00701	271
A00223	A00713	216	A00223	A00759	899
A00223	A00763	477	A00223	A00782	245
A00223	A00787	434	A00223	A00839	477
A00223	A00861	473	A00223	A00870	489
A00223	A00872	364	A00223	A00878	470
A00223	A00902	369	A00223	A00906	319
A00223	A00916	401	A00223	A00923	355
A00223	A00966	457	A00223	A00972	469
A00223	A00990	491	A00223	A01012	452
A00223	A01037	468	A00223	A01053	279
A00223	A01056	747	A00223	A01065	281
A00223	A01072	436	A00223	A01088	352
A00223	A01090	473	A00223	A01092	361
A00223	A01098	688	A00223	A01112	469
A00223	A01120	461	A00223	A01127	714
A00223	A01129	401	A00223	A01130	477
A00223	A01140	491	A00223	A01160	313
A00223	A01166	470	A00223	A01168	486
A00223	A01178	371	A00223	A01198	263
A00223	A01206	456	A00223	A01234	452
A00223	A01238	199	A00223	A01240	452
A00223	A01249	265	A00223	A01288	463
A00223	A01289	491	A00223	A01291	969
A00223	A01294	188	A00223	A01306	379
A00223	A01423	296	A00223	A01425	773
A00223	A01433	373	A00223	A01464	486
A00223	A01468	490	A00223	A01470	462
A00223	A01492	953	A00223	A01496	490
A00223	A01527	899	A00223	A01538	316
A00223	A01540	321	A00223	A01546	316
A00223	A01558	899	A00223	A01579	484
A00223	A01586	419	A00223	A01598	364
A00223	A01601	486	A00223	A01604	944

A00223	A01605	408	A00223	A01614	313
A00223	A01618	406	A00223	A01620	310
A00223	A01640	944	A00223	A01649	470
A00223	A01718	159	A00223	A01730	490
A00223	A01740	486	A00223	A01741	330
A00223	A01755	249	A00223	A01822	196
A00223	A01877	466	A00223	A01888	184
A00223	A02089	188	A00223	A02164	394
A00223	A02165	63	A00223	A02179	475
A00223	A02216	245	A00223	A02243	160
A00223	A02265	481	A00223	A02325	405
A00223	A02413	88	A00223	A02414	290
A00223	A02416	63	A00223	A02418	157
A00223	A02434	252	A00223	A02437	157
A00223	A02490	63	A00223	A02491	160
A00223	A02494	747	A00223	A02550	184
A00223	A02582	156	A00223	A02592	71
A00223	A02597	188	A00223	A02703	424
A00223	A02815	217	A00223	A02816	470
A00223	A02860	460	A00223	A02877	159
A00223	A02895	465	A00223	A02914	249
A00223	A03069	384	A00223	A03083	430
A00223	A03112	157	A00223	A03228	187
A00223	A03308	310	A00223	A03331	208
A00223	A03334	386	A00223	A03437	323
A00223	A03438	490	A00223	A03451	230
A00223	A03461	210	A00223	A03544	184
A00223	A03567	347	A00223	A03571	483
A00223	A03582	362	A00223	A03604	210
A00223	A03625	899	A00223	A03645	210
A00223	A03671	308	A00223	A03686	899
A00223	A03705	196	A00223	A03753	188
A00223	A03773	313	A00223	A03774	204
A00223	A03796	260	A00223	A03888	350
A00223	A03977	273	A00223	A03981	491
A00223	A04116	198	A00223	A04230	491
A00223	A04234	226	A00223	A04312	279
A00223	A04342	475	A00223	A04415	126
A00223	A04418	212	A00223	A04422	374
A00223	A04490	441	A00223	A04492	243
A00223	A04507	279	A00223	A04622	159
A00223	A05085	246	A00223	A05086	428
A00223	A05090	198	A00223	A05360	335
A00223	A05370	313	A00223	A05385	157
A00223	A05411	326	A00223	A05477	434
A00223	A05501	335	A00223	A05601	99

A00223	A05616	310	A00223	A05654	87
A00223	A06279	187	A00223	A06324	375
A00223	A06351	361	A00223	A06486	204
A00223	A06812	210	A00223	A06814	491
A00223	A06825	489	A00223	A06965	160
A00223	A06987	168	A00223	A07001	179
A00223	A07138	361	A00223	A07143	167
A00223	A07163	160	A00223	A07201	491
A00223	A07243	467	A00223	A07251	180
A00223	A07307	404	A00223	A07398	188
A00223	A07412	329	A00223	A07506	210
A00223	A07536	151	A00223	A07546	246
A00223	A07726	346	A00223	A07935	168
A00223	A08052	480	A00223	A08070	207
A00223	A08273	414	A00223	A08315	307
A00223	A08357	385	A00223	A08559	438
A00223	A08571	899	A00223	A08653	130
A00223	A08662	279	A00223	A08682	146
A00223	A08835	385	A00223	A09044	163
A00223	A09248	338	A00223	A09292	262
A00223	A09494	335	A00223	A09550	180
A00223	A09620	419	A00223	A09644	327
A00223	A09722	246	A00223	A09960	246
A00223	A10034	308	A00223	A10080	246
A00223	A10092	310	A00223	A10247	171
A00223	A10249	226	A00223	A10417	486
A00223	A10569	235	A00223	A10633	202
A00223	A10748	238	A00223	A10814	157
A00223	A10839	374	A00223	A11096	283
A00223	A11331	355	A00223	A11392	383
A00223	A11400	441	A00223	A11932	490
A00223	A11981	377	A00223	A11992	152
A00223	A12661	259	A00223	A12687	265
A00223	A12970	217	A00223	A13052	184
A00223	A13107	152	A00223	A13126	419
A00223	A13581	259	A00223	A13824	899
A00223	A13829	899	A00223	A13862	217
A00223	A13875	262	A00223	A13989	974
A00223	A13992	205	A00223	A14038	260
A00223	A14169	215	A00223	A14216	486
A00223	A14246	899	A00223	A14443	899
A00223	A14561	246	A00223	A14843	174
A00223	A15063	279	A00223	A15174	260
A00223	A15235	351	A00223	A15297	899
A00223	A15559	899	A00223	A15743	339
A00223	A16024	226	A00223	A16686	170

A00223	A17193	475	A00224	A00225	945
A00224	A00226	945	A00224	A00227	899
A00224	A00228	899	A00224	A00230	965
A00224	A00231	226	A00224	A00232	475
A00224	A00233	483	A00224	A00234	926
A00224	A00235	955	A00224	A00236	909
A00224	A00239	972	A00224	A00240	959
A00224	A00241	955	A00224	A00242	485
A00224	A00243	493	A00224	A00244	951
A00224	A00245	235	A00224	A00246	932
A00224	A00247	899	A00224	A00248	899
A00224	A00250	899	A00224	A00252	943
A00224	A00253	945	A00224	A00254	949
A00224	A00257	904	A00224	A00261	959
A00224	A00262	899	A00224	A00263	950
A00224	A00264	899	A00224	A00265	458
A00224	A00266	899	A00224	A00269	955
A00224	A00270	950	A00224	A00271	903
A00224	A00275	899	A00224	A00283	199
A00224	A00285	228	A00224	A00294	943
A00224	A00309	235	A00224	A00316	491
A00224	A00324	945	A00224	A00341	227
A00224	A00378	452	A00224	A00381	302
A00224	A00418	425	A00224	A00431	173
A00224	A00440	283	A00224	A00457	474
A00224	A00482	404	A00224	A00523	899
A00224	A00526	899	A00224	A00531	245
A00224	A00533	945	A00224	A00552	463
A00224	A00556	490	A00224	A00557	154
A00224	A00561	899	A00224	A00566	160
A00224	A00638	199	A00224	A00643	512
A00224	A00658	283	A00224	A00666	481
A00224	A00669	944	A00224	A00671	276
A00224	A00696	174	A00224	A00736	579
A00224	A00747	942	A00224	A00748	489
A00224	A00757	480	A00224	A00786	899
A00224	A00801	340	A00224	A00815	180
A00224	A00820	491	A00224	A00825	942
A00224	A00829	209	A00224	A00832	945
A00224	A00835	430	A00224	A00858	174
A00224	A00887	943	A00224	A00914	945
A00224	A00926	461	A00224	A00930	162
A00224	A00933	417	A00224	A00939	165
A00224	A00993	413	A00224	A00997	443
A00224	A01002	899	A00224	A01016	899
A00224	A01017	173	A00224	A01052	949

A00224	A01053	172	A00224	A01070	899
A00224	A01109	187	A00224	A01124	273
A00224	A01125	256	A00224	A01162	199
A00224	A01172	408	A00224	A01173	333
A00224	A01176	899	A00224	A01178	472
A00224	A01194	378	A00224	A01210	156
A00224	A01237	199	A00224	A01244	899
A00224	A01259	165	A00224	A01277	480
A00224	A01291	920	A00224	A01297	199
A00224	A01302	193	A00224	A01307	419
A00224	A01396	188	A00224	A01406	435
A00224	A01415	899	A00224	A01425	899
A00224	A01430	393	A00224	A01445	173
A00224	A01450	180	A00224	A01452	490
A00224	A01461	254	A00224	A01473	954
A00224	A01478	908	A00224	A01501	154
A00224	A01512	926	A00224	A01524	966
A00224	A01527	970	A00224	A01540	199
A00224	A01548	398	A00224	A01553	940
A00224	A01554	940	A00224	A01558	971
A00224	A01560	955	A00224	A01561	903
A00224	A01562	918	A00224	A01570	173
A00224	A01572	949	A00224	A01576	905
A00224	A01588	945	A00224	A01591	950
A00224	A01592	955	A00224	A01593	936
A00224	A01600	171	A00224	A01602	945
A00224	A01605	905	A00224	A01610	269
A00224	A01612	967	A00224	A01615	949
A00224	A01628	899	A00224	A01636	952
A00224	A01637	922	A00224	A01638	943
A00224	A01639	899	A00224	A01640	948
A00224	A01645	193	A00224	A01647	199
A00224	A01650	949	A00224	A01651	944
A00224	A01660	199	A00224	A01665	899
A00224	A01668	491	A00224	A01675	899
A00224	A01676	451	A00224	A01680	899
A00224	A01688	942	A00224	A01692	947
A00224	A01694	254	A00224	A01697	263
A00224	A01700	273	A00224	A01702	943
A00224	A01705	198	A00224	A01708	899
A00224	A01709	914	A00224	A01712	442
A00224	A01718	899	A00224	A01719	899
A00224	A01741	491	A00224	A01743	491
A00224	A01789	329	A00224	A01790	480
A00224	A01796	491	A00224	A01797	488
A00224	A01798	369	A00224	A01812	168

A00224	A01819	213	A00224	A01839	279
A00224	A01844	955	A00224	A01850	152
A00224	A01855	491	A00224	A01856	462
A00224	A01859	489	A00224	A01861	152
A00224	A01864	481	A00224	A01876	380
A00224	A01900	489	A00224	A01941	449
A00224	A01959	338	A00224	A01974	160
A00224	A01995	467	A00224	A01996	899
A00224	A02030	491	A00224	A02050	160
A00224	A02086	299	A00224	A02148	281
A00224	A02163	954	A00224	A02211	899
A00224	A02226	202	A00224	A02268	323
A00224	A02304	433	A00224	A02308	199
A00224	A02311	340	A00224	A02331	481
A00224	A02361	216	A00224	A02362	467
A00224	A02377	432	A00224	A02381	472
A00224	A02382	946	A00224	A02393	163
A00224	A02411	899	A00224	A02416	899
A00224	A02419	899	A00224	A02454	446
A00224	A02464	899	A00224	A02520	358
A00224	A02562	246	A00224	A02564	486
A00224	A02639	157	A00224	A02671	477
A00224	A02695	447	A00224	A02703	313
A00224	A02711	200	A00224	A02723	358
A00224	A02741	235	A00224	A02776	291
A00224	A02829	171	A00224	A02830	361
A00224	A02836	184	A00224	A02837	486
A00224	A02859	418	A00224	A02895	270
A00224	A02981	190	A00224	A03003	475
A00224	A03007	438	A00224	A03019	441
A00224	A03057	373	A00224	A03091	281
A00224	A03110	899	A00224	A03112	491
A00224	A03113	217	A00224	A03186	460
A00224	A03209	902	A00224	A03213	443
A00224	A03214	340	A00224	A03223	489
A00224	A03259	489	A00224	A03267	223
A00224	A03275	446	A00224	A03292	162
A00224	A03321	490	A00224	A03422	943
A00224	A03432	430	A00224	A03434	330
A00224	A03436	488	A00224	A03451	425
A00224	A03477	489	A00224	A03480	251
A00224	A03500	160	A00224	A03512	490
A00224	A03536	230	A00224	A03569	472
A00224	A03575	949	A00224	A03586	413
A00224	A03591	919	A00224	A03611	488
A00224	A03641	223	A00224	A03686	899

A00224	A03726	901	A00224	A03741	904
A00224	A03752	199	A00224	A03803	470
A00224	A03817	899	A00224	A03818	174
A00224	A03843	402	A00224	A03858	199
A00224	A03861	491	A00224	A03946	276
A00224	A03990	945	A00224	A04010	224
A00224	A04012	906	A00224	A04050	329
A00224	A04053	219	A00224	A04062	899
A00224	A04110	293	A00224	A04117	422
A00224	A04175	218	A00224	A04398	899
A00224	A04622	483	A00224	A04699	932
A00224	A04751	945	A00224	A04790	386
A00224	A04807	357	A00224	A04849	472
A00224	A04850	490	A00224	A04861	173
A00224	A04888	452	A00224	A04902	480
A00224	A04938	196	A00224	A04949	196
A00224	A05006	435	A00224	A05008	944
A00224	A05009	940	A00224	A05015	945
A00224	A05029	906	A00224	A05062	954
A00224	A05064	258	A00224	A05070	157
A00224	A05082	490	A00224	A05112	406
A00224	A05127	235	A00224	A05216	945
A00224	A05278	404	A00224	A05284	480
A00224	A05288	491	A00224	A05415	263
A00224	A05418	446	A00224	A05428	936
A00224	A05446	475	A00224	A05453	899
A00224	A05454	303	A00224	A05482	899
A00224	A05506	157	A00224	A05767	446
A00224	A05880	423	A00224	A06063	213
A00224	A06258	157	A00224	A06268	490
A00224	A06277	899	A00224	A06289	899
A00224	A06295	899	A00224	A06379	283
A00224	A06504	473	A00224	A06581	490
A00224	A06597	881	A00224	A06608	935
A00224	A06610	945	A00224	A06615	490
A00224	A06648	462	A00224	A06660	920
A00224	A06687	401	A00224	A06735	173
A00224	A06764	246	A00224	A06765	425
A00224	A06819	195	A00224	A06825	151
A00224	A06882	899	A00224	A06907	578
A00224	A07004	335	A00224	A07091	449
A00224	A07157	464	A00224	A07165	921
A00224	A07190	486	A00224	A07191	486
A00224	A07311	950	A00224	A07339	486
A00224	A07422	899	A00224	A07570	172
A00224	A07604	413	A00224	A07762	272

A00224	A07797	899	A00224	A07798	475
A00224	A07834	489	A00224	A08013	306
A00224	A08137	529	A00224	A08205	199
A00224	A08207	191	A00224	A08280	531
A00224	A08571	951	A00224	A08700	176
A00224	A08836	385	A00224	A08839	929
A00224	A08858	951	A00224	A08860	154
A00224	A08878	935	A00224	A08944	279
A00224	A09059	392	A00224	A09084	277
A00224	A09110	271	A00224	A09256	944
A00224	A09263	196	A00224	A09319	945
A00224	A09383	899	A00224	A09416	151
A00224	A09542	899	A00224	A09556	491
A00224	A09568	905	A00224	A09752	199
A00224	A09758	199	A00224	A09842	229
A00224	A09913	939	A00224	A09977	469
A00224	A10065	266	A00224	A10174	466
A00224	A10185	899	A00224	A10247	263
A00224	A10330	193	A00224	A10365	899
A00224	A10392	899	A00224	A10506	452
A00224	A10544	425	A00224	A10547	549
A00224	A10580	906	A00224	A10581	899
A00224	A10601	916	A00224	A10615	945
A00224	A10623	899	A00224	A10624	341
A00224	A10625	467	A00224	A10673	450
A00224	A10743	319	A00224	A10801	899
A00224	A10813	899	A00224	A10833	901
A00224	A10875	199	A00224	A10894	490
A00224	A10899	212	A00224	A11019	899
A00224	A11023	266	A00224	A11027	224
A00224	A11125	899	A00224	A11149	259
A00224	A11180	179	A00224	A11184	279
A00224	A11286	429	A00224	A11291	469
A00224	A11304	475	A00224	A11346	275
A00224	A11369	944	A00224	A11430	489
A00224	A11478	423	A00224	A11487	945
A00224	A11493	329	A00224	A11543	306
A00224	A11567	199	A00224	A11601	899
A00224	A11683	491	A00224	A11834	899
A00224	A11850	903	A00224	A11858	238
A00224	A11902	326	A00224	A11923	657
A00224	A11943	450	A00224	A11983	964
A00224	A11993	160	A00224	A12132	223
A00224	A12195	252	A00224	A12383	935
A00224	A12664	925	A00224	A12705	931
A00224	A12730	373	A00224	A12897	286

A00224	A13147	457	A00224	A13228	235
A00224	A13250	899	A00224	A13346	490
A00224	A13368	900	A00224	A13373	172
A00224	A13390	915	A00224	A13391	899
A00224	A13393	899	A00224	A13516	311
A00224	A13517	201	A00224	A13605	899
A00224	A13607	193	A00224	A13617	255
A00224	A13667	191	A00224	A13759	353
A00224	A13824	903	A00224	A13829	472
A00224	A13928	444	A00224	A13975	945
A00224	A13977	899	A00224	A13978	899
A00224	A13980	581	A00224	A13981	899
A00224	A13982	484	A00224	A13983	485
A00224	A13984	899	A00224	A13985	905
A00224	A13986	326	A00224	A13990	899
A00224	A13991	940	A00224	A13997	276
A00224	A14001	899	A00224	A14005	899
A00224	A14006	491	A00224	A14030	899
A00224	A14032	899	A00224	A14035	899
A00224	A14042	899	A00224	A14045	901
A00224	A14055	434	A00224	A14059	899
A00224	A14128	362	A00224	A14148	238
A00224	A14154	174	A00224	A14188	491
A00224	A14190	173	A00224	A14193	199
A00224	A14198	943	A00224	A14199	458
A00224	A14202	911	A00224	A14208	185
A00224	A14209	257	A00224	A14220	508
A00224	A14221	521	A00224	A14269	243
A00224	A14279	199	A00224	A14299	945
A00224	A14303	211	A00224	A14393	899
A00224	A14430	234	A00224	A14433	905
A00224	A14443	948	A00224	A14444	945
A00224	A14456	911	A00224	A14472	899
A00224	A14496	191	A00224	A14625	165
A00224	A14643	269	A00224	A14694	899
A00224	A14699	173	A00224	A14758	199
A00224	A14930	899	A00224	A14986	213
A00224	A15099	404	A00224	A15182	899
A00224	A15241	296	A00224	A15291	949
A00224	A15292	909	A00224	A15293	899
A00224	A15294	899	A00224	A15315	884
A00224	A15323	243	A00224	A15345	703
A00224	A15347	890	A00224	A15366	266
A00224	A15384	518	A00224	A15416	168
A00224	A15528	484	A00224	A15613	226
A00224	A15614	950	A00224	A15617	651

A00224	A15624	270	A00224	A15665	336
A00224	A15673	436	A00224	A15726	955
A00224	A15752	217	A00224	A15854	173
A00224	A15920	899	A00224	A15935	234
A00224	A16083	899	A00224	A16135	477
A00224	A16284	246	A00224	A16304	486
A00224	A16322	469	A00224	A16328	332
A00224	A16364	548	A00224	A16467	311
A00224	A16532	160	A00224	A16581	159
A00224	A16741	173	A00224	A16938	899
A00224	A17160	899	A00225	A00226	491
A00225	A00230	899	A00225	A00246	899
A00225	A00257	489	A00225	A00265	491
A00225	A00306	489	A00225	A00533	413
A00225	A00557	215	A00225	A00565	163
A00225	A00736	490	A00225	A00805	151
A00225	A00843	491	A00225	A00847	488
A00225	A00856	314	A00225	A01088	388
A00225	A01214	230	A00225	A01307	373
A00225	A01514	160	A00225	A01558	910
A00225	A01560	257	A00225	A01576	441
A00225	A01588	901	A00225	A01591	215
A00225	A01636	929	A00225	A01637	475
A00225	A01650	463	A00225	A01651	944
A00225	A01668	176	A00225	A01859	65
A00225	A01988	241	A00225	A02376	486
A00225	A02452	205	A00225	A02737	681
A00225	A02825	625	A00225	A02904	294
A00225	A03244	303	A00225	A03278	301
A00225	A03457	215	A00225	A03524	291
A00225	A03569	230	A00225	A04378	274
A00225	A04574	163	A00225	A04697	218
A00225	A04902	188	A00225	A05029	963
A00225	A05416	185	A00225	A05645	208
A00225	A05832	258	A00225	A06581	272
A00225	A06597	491	A00225	A06648	171
A00225	A06907	266	A00225	A06971	245
A00225	A06980	230	A00225	A07311	427
A00225	A07422	903	A00225	A07760	469
A00225	A07814	172	A00225	A08391	176
A00225	A08583	232	A00225	A08860	486
A00225	A09218	199	A00225	A09319	477
A00225	A09977	353	A00225	A10244	252
A00225	A10459	224	A00225	A10460	291
A00225	A10576	271	A00225	A10601	402
A00225	A11474	378	A00225	A11475	188

A00225	A12522	185	A00225	A12664	217
A00225	A12719	185	A00225	A13228	160
A00225	A13346	378	A00225	A13511	260
A00225	A13617	154	A00225	A13975	193
A00225	A14196	457	A00225	A14208	469
A00225	A14827	173	A00225	A15105	314
A00225	A15291	491	A00225	A15614	466
A00225	A16467	199	A00225	A16497	236
A00225	A16766	360	A00225	A16969	285
A00226	A00230	899	A00226	A00246	899
A00226	A00257	467	A00226	A00263	308
A00226	A00265	171	A00226	A00533	121
A00226	A00736	191	A00226	A00801	217
A00226	A01178	180	A00226	A01307	232
A00226	A01524	899	A00226	A01548	63
A00226	A01554	935	A00226	A01558	903
A00226	A01560	324	A00226	A01588	991
A00226	A01591	285	A00226	A01636	899
A00226	A01651	899	A00226	A01668	183
A00226	A01709	369	A00226	A02145	327
A00226	A02689	193	A00226	A02737	165
A00226	A02825	360	A00226	A02826	249
A00226	A02975	249	A00226	A03432	152
A00226	A04182	174	A00226	A04657	201
A00226	A04905	201	A00226	A05029	473
A00226	A05203	255	A00226	A06597	466
A00226	A06608	380	A00226	A06735	306
A00226	A07311	257	A00226	A07422	900
A00226	A08860	380	A00226	A09204	255
A00226	A09303	182	A00226	A09319	486
A00226	A09502	199	A00226	A09745	235
A00226	A10336	182	A00226	A10601	937
A00226	A11180	174	A00226	A11971	355
A00226	A11972	332	A00226	A12131	355
A00226	A12343	281	A00226	A12360	355
A00226	A12522	281	A00226	A13975	228
A00226	A13982	436	A00226	A14269	305
A00226	A14456	909	A00226	A14944	260
A00226	A15291	939	A00226	A15614	929
A00226	A16916	243	A00227	A00230	899
A00227	A00247	942	A00227	A00254	457
A00227	A00315	135	A00227	A00351	188
A00227	A00472	299	A00227	A00478	491
A00227	A00624	156	A00227	A00656	241
A00227	A00659	491	A00227	A00663	265
A00227	A00815	168	A00227	A01178	74

A00227	A01257	219	A00227	A01527	899
A00227	A01544	324	A00227	A01556	154
A00227	A01558	899	A00227	A01575	291
A00227	A01585	363	A00227	A01722	79
A00227	A01737	491	A00227	A01741	436
A00227	A01797	221	A00227	A01846	174
A00227	A01861	191	A00227	A01864	152
A00227	A01926	454	A00227	A01929	195
A00227	A01959	224	A00227	A01985	149
A00227	A02008	483	A00227	A02009	430
A00227	A02010	364	A00227	A02011	491
A00227	A02012	481	A00227	A02014	469
A00227	A02015	356	A00227	A02026	423
A00227	A02028	486	A00227	A02044	121
A00227	A02048	430	A00227	A02114	439
A00227	A02119	104	A00227	A02121	460
A00227	A02126	238	A00227	A02148	171
A00227	A02213	185	A00227	A02238	272
A00227	A02361	160	A00227	A02423	118
A00227	A02425	390	A00227	A02429	274
A00227	A02454	141	A00227	A02465	378
A00227	A02472	63	A00227	A02481	488
A00227	A02574	319	A00227	A02611	469
A00227	A02668	286	A00227	A02695	483
A00227	A02806	491	A00227	A02859	434
A00227	A02881	205	A00227	A02951	213
A00227	A03034	491	A00227	A03035	174
A00227	A03036	491	A00227	A03039	245
A00227	A03053	160	A00227	A03071	467
A00227	A03080	463	A00227	A03094	388
A00227	A03126	472	A00227	A03127	191
A00227	A03144	491	A00227	A03146	486
A00227	A03147	279	A00227	A03148	485
A00227	A03156	491	A00227	A03157	329
A00227	A03165	180	A00227	A03186	180
A00227	A03195	345	A00227	A03196	249
A00227	A03247	170	A00227	A03259	382
A00227	A03370	409	A00227	A03379	279
A00227	A03513	251	A00227	A03683	210
A00227	A03684	296	A00227	A03685	168
A00227	A03744	441	A00227	A03831	257
A00227	A03927	434	A00227	A03934	245
A00227	A03936	157	A00227	A04075	273
A00227	A04080	290	A00227	A04133	168
A00227	A04147	118	A00227	A04152	157
A00227	A04358	245	A00227	A04392	296

A00227	A04473	317	A00227	A04487	268
A00227	A04518	200	A00227	A04520	191
A00227	A04534	372	A00227	A04614	254
A00227	A04616	174	A00227	A05088	229
A00227	A05093	257	A00227	A05112	183
A00227	A05185	157	A00227	A05203	229
A00227	A05246	363	A00227	A05256	160
A00227	A05457	265	A00227	A05491	238
A00227	A05570	477	A00227	A05716	218
A00227	A05779	184	A00227	A05956	319
A00227	A05988	213	A00227	A06052	296
A00227	A06053	368	A00227	A06195	491
A00227	A06483	339	A00227	A06653	152
A00227	A06813	184	A00227	A06916	196
A00227	A07034	313	A00227	A07167	232
A00227	A07195	265	A00227	A07218	456
A00227	A07311	899	A00227	A07342	481
A00227	A07373	170	A00227	A07477	208
A00227	A07736	343	A00227	A09123	160
A00227	A09211	154	A00227	A09323	179
A00227	A09983	213	A00227	A09991	488
A00227	A10091	188	A00227	A10121	249
A00227	A10226	451	A00227	A10758	296
A00227	A11064	210	A00227	A11076	339
A00227	A11485	223	A00227	A12654	201
A00227	A12697	283	A00227	A12948	234
A00227	A12965	296	A00227	A13827	213
A00227	A14188	441	A00227	A14344	452
A00227	A15286	180	A00227	A15498	372
A00227	A15913	182	A00227	A15914	339
A00227	A16087	215	A00227	A16623	157
A00227	A16636	152	A00227	A16732	221
A00227	A16951	210	A00228	A00230	899
A00228	A00244	151	A00228	A00247	491
A00228	A00294	466	A00228	A00309	475
A00228	A00314	180	A00228	A00370	438
A00228	A00642	340	A00228	A00643	433
A00228	A00747	265	A00228	A00806	480
A00228	A00932	473	A00228	A01083	273
A00228	A01485	294	A00228	A01490	210
A00228	A01540	200	A00228	A01544	486
A00228	A01558	899	A00228	A01562	341
A00228	A01588	899	A00228	A01615	899
A00228	A01718	472	A00228	A01886	171
A00228	A01916	179	A00228	A01927	157
A00228	A02239	486	A00228	A02460	180

A00228	A02499	340	A00228	A02636	263
A00228	A02839	201	A00228	A03049	456
A00228	A03174	241	A00228	A03201	156
A00228	A03512	283	A00228	A03629	238
A00228	A03654	260	A00228	A03790	306
A00228	A03798	348	A00228	A04280	232
A00228	A04775	259	A00228	A04914	465
A00228	A04924	278	A00228	A05041	272
A00228	A05086	252	A00228	A05145	255
A00228	A05185	188	A00228	A05224	263
A00228	A05240	462	A00228	A05338	70
A00228	A05424	371	A00228	A05709	217
A00228	A07107	159	A00228	A07148	208
A00228	A07158	168	A00228	A08197	205
A00228	A08911	152	A00228	A09071	334
A00228	A09110	243	A00228	A09388	185
A00228	A09417	238	A00228	A09447	229
A00228	A09620	157	A00228	A09809	183
A00228	A09888	374	A00228	A09906	283
A00228	A10034	160	A00228	A10619	152
A00228	A10679	208	A00228	A10911	345
A00228	A11002	334	A00228	A11100	408
A00228	A11860	208	A00228	A12463	334
A00228	A12819	357	A00228	A12990	460
A00228	A13276	334	A00228	A13373	363
A00228	A13494	299	A00228	A13521	386
A00228	A13788	196	A00228	A14355	291
A00228	A14404	193	A00228	A14443	899
A00228	A14543	180	A00228	A14583	205
A00228	A14647	176	A00228	A15551	246
A00228	A15911	152	A00228	A15940	193
A00228	A16714	357	A00228	A16824	251
A00228	A16944	221	A00229	A00240	953
A00229	A00272	984	A00229	A00294	899
A00229	A00324	899	A00229	A00571	400
A00229	A00653	448	A00229	A00804	232
A00229	A00868	216	A00229	A00929	249
A00229	A00987	268	A00229	A01086	356
A00229	A01161	487	A00229	A01234	193
A00229	A01310	766	A00229	A01844	246
A00229	A02077	296	A00229	A02473	385
A00229	A02502	160	A00229	A02836	851
A00229	A03050	465	A00229	A03440	429
A00229	A03591	198	A00229	A03650	286
A00229	A03931	172	A00229	A04436	271
A00229	A04630	441	A00229	A05854	394

A00229	A06689	156	A00229	A07027	294
A00229	A07311	537	A00229	A07511	198
A00229	A10253	452	A00229	A10386	226
A00229	A11614	391	A00229	A11707	371
A00229	A11834	452	A00229	A14062	762
A00229	A14191	201	A00229	A14672	184
A00229	A15294	249	A00229	A15295	185
A00230	A00234	899	A00230	A00235	433
A00230	A00239	316	A00230	A00240	475
A00230	A00246	899	A00230	A00247	900
A00230	A00254	937	A00230	A00255	913
A00230	A00258	899	A00230	A00261	945
A00230	A00269	943	A00230	A00270	501
A00230	A00275	899	A00230	A00294	937
A00230	A00316	899	A00230	A00324	922
A00230	A00388	332	A00230	A00398	486
A00230	A00408	362	A00230	A00523	899
A00230	A00533	489	A00230	A00552	154
A00230	A00669	925	A00230	A00671	168
A00230	A00748	491	A00230	A00786	899
A00230	A00801	201	A00230	A00812	243
A00230	A00832	899	A00230	A00886	256
A00230	A00887	940	A00230	A00916	352
A00230	A00923	322	A00230	A00943	196
A00230	A00981	160	A00230	A00997	394
A00230	A01052	945	A00230	A01070	899
A00230	A01212	177	A00230	A01291	903
A00230	A01302	481	A00230	A01404	463
A00230	A01478	165	A00230	A01479	322
A00230	A01512	899	A00230	A01524	958
A00230	A01527	949	A00230	A01553	899
A00230	A01554	900	A00230	A01558	945
A00230	A01560	927	A00230	A01561	899
A00230	A01562	899	A00230	A01572	944
A00230	A01588	950	A00230	A01591	949
A00230	A01592	950	A00230	A01593	935
A00230	A01602	920	A00230	A01605	899
A00230	A01612	940	A00230	A01615	945
A00230	A01628	899	A00230	A01636	915
A00230	A01637	922	A00230	A01638	950
A00230	A01639	920	A00230	A01640	899
A00230	A01650	438	A00230	A01651	904
A00230	A01665	899	A00230	A01680	899
A00230	A01688	910	A00230	A01692	899
A00230	A01697	899	A00230	A01702	899
A00230	A01708	899	A00230	A01709	899

A00230	A01712	235	A00230	A01717	902
A00230	A01718	899	A00230	A01719	899
A00230	A01722	899	A00230	A01844	935
A00230	A01876	480	A00230	A01920	899
A00230	A01996	899	A00230	A02381	477
A00230	A02416	899	A00230	A02464	899
A00230	A02600	185	A00230	A02712	355
A00230	A02719	196	A00230	A03002	199
A00230	A03110	899	A00230	A03213	419
A00230	A03214	217	A00230	A03422	385
A00230	A03435	188	A00230	A03451	899
A00230	A03470	899	A00230	A03575	950
A00230	A03591	899	A00230	A03611	414
A00230	A03686	899	A00230	A03726	936
A00230	A03741	962	A00230	A03809	899
A00230	A03990	481	A00230	A04012	899
A00230	A04110	302	A00230	A04362	201
A00230	A04398	899	A00230	A04751	899
A00230	A04767	154	A00230	A04825	185
A00230	A04849	165	A00230	A04973	154
A00230	A05015	935	A00230	A05029	918
A00230	A05062	361	A00230	A05082	447
A00230	A05216	972	A00230	A05226	332
A00230	A05428	936	A00230	A05454	491
A00230	A05488	221	A00230	A05684	162
A00230	A05880	235	A00230	A05885	185
A00230	A05987	168	A00230	A06268	240
A00230	A06289	899	A00230	A06348	270
A00230	A06390	258	A00230	A06415	232
A00230	A06581	901	A00230	A06597	490
A00230	A06610	443	A00230	A06642	408
A00230	A06648	335	A00230	A06687	490
A00230	A06819	419	A00230	A07091	288
A00230	A07135	899	A00230	A07311	903
A00230	A07339	340	A00230	A07422	899
A00230	A07433	467	A00230	A07565	429
A00230	A07797	899	A00230	A07834	364
A00230	A08555	454	A00230	A08571	950
A00230	A08836	321	A00230	A08839	922
A00230	A08858	952	A00230	A08859	199
A00230	A08865	899	A00230	A08878	899
A00230	A09218	899	A00230	A09220	899
A00230	A09242	324	A00230	A09256	202
A00230	A09298	483	A00230	A09457	200
A00230	A09542	899	A00230	A09577	899
A00230	A09644	899	A00230	A09913	329

A00230	A10319	286	A00230	A10365	899
A00230	A10392	899	A00230	A10506	268
A00230	A10547	240	A00230	A10580	902
A00230	A10615	932	A00230	A10623	899
A00230	A10750	424	A00230	A10756	899
A00230	A10801	899	A00230	A10894	351
A00230	A11019	173	A00230	A11118	200
A00230	A11369	899	A00230	A11604	914
A00230	A11902	322	A00230	A11923	816
A00230	A11983	899	A00230	A12373	899
A00230	A12383	899	A00230	A12604	185
A00230	A13147	941	A00230	A13250	899
A00230	A13824	900	A00230	A13944	899
A00230	A13975	906	A00230	A13977	899
A00230	A13978	899	A00230	A13980	939
A00230	A13981	899	A00230	A13983	491
A00230	A14032	899	A00230	A14044	899
A00230	A14053	258	A00230	A14139	899
A00230	A14146	899	A00230	A14188	927
A00230	A14196	899	A00230	A14198	899
A00230	A14199	486	A00230	A14208	899
A00230	A14210	899	A00230	A14221	163
A00230	A14299	908	A00230	A14371	899
A00230	A14374	933	A00230	A14379	899
A00230	A14397	899	A00230	A14408	899
A00230	A14439	185	A00230	A14443	927
A00230	A14444	899	A00230	A14472	899
A00230	A14511	899	A00230	A14930	899
A00230	A15120	258	A00230	A15293	899
A00230	A15294	899	A00230	A15314	899
A00230	A15321	899	A00230	A15342	935
A00230	A15366	899	A00230	A15422	899
A00230	A15642	252	A00230	A15726	955
A00230	A15920	899	A00230	A15995	258
A00230	A16253	899	A00230	A16304	645
A00230	A16317	200	A00230	A16424	899
A00230	A16581	955	A00230	A16741	328
A00230	A17060	899	A00230	A17160	899
A00231	A00239	313	A00231	A00240	382
A00231	A00251	899	A00231	A00630	288
A00231	A00875	177	A00231	A00914	469
A00231	A01125	419	A00231	A01157	384
A00231	A01289	208	A00231	A01473	288
A00231	A01527	180	A00231	A01562	479
A00231	A01605	229	A00231	A01688	176
A00231	A01730	489	A00231	A01887	191

A00231	A02001	215	A00231	A02074	235
A00231	A02076	462	A00231	A02163	425
A00231	A02380	221	A00231	A02482	409
A00231	A02502	470	A00231	A02513	196
A00231	A02836	467	A00231	A02896	236
A00231	A03141	201	A00231	A03209	902
A00231	A03434	374	A00231	A03436	475
A00231	A03451	391	A00231	A04101	205
A00231	A04202	480	A00231	A04546	243
A00231	A04639	291	A00231	A04773	232
A00231	A05417	249	A00231	A05436	265
A00231	A05450	157	A00231	A05563	177
A00231	A05989	291	A00231	A06717	277
A00231	A07315	152	A00231	A10009	347
A00231	A10332	436	A00231	A10595	425
A00231	A10870	157	A00231	A10905	213
A00231	A11932	486	A00231	A12111	283
A00231	A12971	162	A00231	A13989	899
A00231	A14416	219	A00231	A14443	390
A00231	A15241	385	A00231	A15293	388
A00231	A15506	160	A00231	A16537	202
A00232	A00233	491	A00232	A00234	488
A00232	A00246	63	A00232	A00254	270
A00232	A00350	406	A00232	A00352	360
A00232	A00377	286	A00232	A00388	338
A00232	A00437	305	A00232	A00537	156
A00232	A00557	357	A00232	A00604	384
A00232	A00653	486	A00232	A00659	288
A00232	A00736	430	A00232	A00752	182
A00232	A00799	343	A00232	A00868	324
A00232	A00914	491	A00232	A00926	391
A00232	A00927	207	A00232	A00939	481
A00232	A00953	488	A00232	A00987	268
A00232	A01032	466	A00232	A01046	347
A00232	A01088	402	A00232	A01117	236
A00232	A01119	424	A00232	A01150	419
A00232	A01161	416	A00232	A01230	236
A00232	A01307	176	A00232	A01396	472
A00232	A01404	321	A00232	A01452	196
A00232	A01458	154	A00232	A01461	329
A00232	A01473	488	A00232	A01557	232
A00232	A01572	491	A00232	A01573	294
A00232	A01574	301	A00232	A01644	352
A00232	A01671	274	A00232	A01676	488
A00232	A01704	110	A00232	A01726	407
A00232	A01733	299	A00232	A01741	172

A00232	A01749	63	A00232	A01769	393
A00232	A01775	286	A00232	A01789	63
A00232	A01799	107	A00232	A01834	165
A00232	A01837	63	A00232	A01844	63
A00232	A01847	457	A00232	A01849	102
A00232	A01879	63	A00232	A01887	404
A00232	A01888	424	A00232	A01900	490
A00232	A01908	63	A00232	A02011	243
A00232	A02086	485	A00232	A02252	236
A00232	A02268	205	A00232	A02278	207
A00232	A02338	184	A00232	A02342	210
A00232	A02343	63	A00232	A02362	360
A00232	A02372	360	A00232	A02380	441
A00232	A02454	279	A00232	A02513	328
A00232	A02564	467	A00232	A02610	235
A00232	A02649	243	A00232	A02653	171
A00232	A02657	160	A00232	A02680	183
A00232	A02699	176	A00232	A02717	411
A00232	A02731	491	A00232	A02735	480
A00232	A02739	379	A00232	A02745	63
A00232	A02770	413	A00232	A02802	290
A00232	A02808	198	A00232	A02828	389
A00232	A02830	208	A00232	A02837	63
A00232	A02847	491	A00232	A02851	238
A00232	A02856	270	A00232	A02864	485
A00232	A02869	63	A00232	A02888	177
A00232	A02896	63	A00232	A02897	480
A00232	A02908	491	A00232	A02920	246
A00232	A02925	491	A00232	A02929	236
A00232	A02931	288	A00232	A02942	379
A00232	A02960	208	A00232	A02961	475
A00232	A02968	312	A00232	A02972	168
A00232	A02976	358	A00232	A02982	486
A00232	A03001	101	A00232	A03010	489
A00232	A03011	157	A00232	A03019	460
A00232	A03046	283	A00232	A03095	170
A00232	A03206	190	A00232	A03212	167
A00232	A03223	486	A00232	A03279	328
A00232	A03281	432	A00232	A03340	235
A00232	A03556	432	A00232	A03589	171
A00232	A03687	260	A00232	A03742	210
A00232	A03780	105	A00232	A03821	491
A00232	A03864	171	A00232	A04005	160
A00232	A04030	199	A00232	A04050	180
A00232	A04105	63	A00232	A04117	180
A00232	A04207	484	A00232	A04288	82

A00232	A04446	469	A00232	A04468	157
A00232	A04504	183	A00232	A04622	168
A00232	A04717	208	A00232	A04813	215
A00232	A04908	490	A00232	A05157	201
A00232	A05211	457	A00232	A05213	208
A00232	A05279	235	A00232	A05280	256
A00232	A05297	293	A00232	A05309	63
A00232	A05402	162	A00232	A05539	196
A00232	A05577	486	A00232	A05591	180
A00232	A05679	235	A00232	A05760	418
A00232	A05794	221	A00232	A05847	104
A00232	A05888	277	A00232	A05893	288
A00232	A05936	235	A00232	A06015	152
A00232	A06107	165	A00232	A06156	221
A00232	A06244	190	A00232	A06271	177
A00232	A06285	235	A00232	A06339	485
A00232	A06370	152	A00232	A06415	439
A00232	A06421	183	A00232	A06422	185
A00232	A06470	257	A00232	A06471	299
A00232	A06510	177	A00232	A06631	265
A00232	A06643	340	A00232	A06644	170
A00232	A06758	205	A00232	A07140	196
A00232	A07157	107	A00232	A07165	393
A00232	A07168	183	A00232	A07186	127
A00232	A07199	254	A00232	A07201	486
A00232	A07259	208	A00232	A07324	332
A00232	A07335	340	A00232	A07510	160
A00232	A07655	384	A00232	A07748	324
A00232	A07795	208	A00232	A07799	416
A00232	A07900	168	A00232	A07955	256
A00232	A07961	491	A00232	A08199	229
A00232	A08566	195	A00232	A08660	162
A00232	A08667	223	A00232	A08688	314
A00232	A08715	299	A00232	A08732	468
A00232	A08759	221	A00232	A09454	152
A00232	A09628	340	A00232	A09652	157
A00232	A10050	199	A00232	A10061	171
A00232	A10081	160	A00232	A10146	165
A00232	A10195	221	A00232	A10317	327
A00232	A10322	235	A00232	A10573	256
A00232	A10622	177	A00232	A10823	400
A00232	A10904	208	A00232	A10930	221
A00232	A11212	256	A00232	A11253	157
A00232	A11369	340	A00232	A11436	213
A00232	A11583	238	A00232	A11964	160
A00232	A12585	288	A00232	A12656	235

A00232	A13739	199	A00232	A13774	256
A00232	A13830	393	A00232	A13844	165
A00232	A13978	369	A00232	A13986	243
A00232	A13996	221	A00232	A14029	85
A00232	A14071	411	A00232	A14173	191
A00232	A14198	329	A00232	A14208	491
A00232	A14374	171	A00232	A14404	213
A00232	A14431	185	A00232	A14554	188
A00232	A14727	232	A00232	A14785	157
A00232	A15271	152	A00232	A15487	268
A00232	A15613	462	A00232	A15639	404
A00232	A15642	226	A00232	A15822	199
A00232	A15846	299	A00232	A15960	165
A00232	A15962	402	A00232	A16428	160
A00232	A16723	243	A00232	A16752	288
A00232	A16758	157	A00232	A16996	171
A00232	A17126	302	A00232	A17212	317
A00233	A00234	491	A00233	A00248	168
A00233	A00254	473	A00233	A00264	481
A00233	A00265	256	A00233	A00274	273
A00233	A00305	249	A00233	A00374	180
A00233	A00406	183	A00233	A00457	212
A00233	A00463	236	A00233	A00523	324
A00233	A00554	167	A00233	A00556	468
A00233	A00561	152	A00233	A00679	63
A00233	A00736	313	A00233	A00799	416
A00233	A00868	151	A00233	A00914	491
A00233	A00934	167	A00233	A01116	162
A00233	A01150	170	A00233	A01189	165
A00233	A01235	223	A00233	A01287	364
A00233	A01404	330	A00233	A01452	327
A00233	A01473	249	A00233	A01524	322
A00233	A01527	922	A00233	A01560	899
A00233	A01572	152	A00233	A01599	179
A00233	A01604	457	A00233	A01654	384
A00233	A01668	481	A00233	A01676	480
A00233	A01704	491	A00233	A01733	477
A00233	A01741	491	A00233	A01743	446
A00233	A01759	260	A00233	A01769	293
A00233	A01775	491	A00233	A01776	184
A00233	A01785	443	A00233	A01789	486
A00233	A01797	475	A00233	A01836	226
A00233	A01844	146	A00233	A01876	380
A00233	A01879	491	A00233	A01880	263
A00233	A01882	217	A00233	A01887	491
A00233	A01888	489	A00233	A01900	187

A00233	A01901	210	A00233	A01902	255
A00233	A01905	210	A00233	A01908	185
A00233	A02051	162	A00233	A02162	184
A00233	A02183	272	A00233	A02340	279
A00233	A02341	201	A00233	A02342	63
A00233	A02343	68	A00233	A02372	385
A00233	A02376	481	A00233	A02377	490
A00233	A02382	171	A00233	A02412	208
A00233	A02415	159	A00233	A02490	263
A00233	A02508	156	A00233	A02582	156
A00233	A02611	246	A00233	A02612	235
A00233	A02637	330	A00233	A02653	198
A00233	A02661	151	A00233	A02689	347
A00233	A02695	294	A00233	A02698	165
A00233	A02699	212	A00233	A02705	144
A00233	A02709	330	A00233	A02712	193
A00233	A02730	408	A00233	A02731	63
A00233	A02735	91	A00233	A02742	132
A00233	A02745	390	A00233	A02754	329
A00233	A02784	159	A00233	A02785	167
A00233	A02787	372	A00233	A02788	198
A00233	A02800	271	A00233	A02803	477
A00233	A02808	479	A00233	A02809	173
A00233	A02812	452	A00233	A02828	424
A00233	A02837	490	A00233	A02854	268
A00233	A02859	491	A00233	A02864	491
A00233	A02865	162	A00233	A02872	184
A00233	A02874	486	A00233	A02886	249
A00233	A02896	491	A00233	A02897	238
A00233	A02900	310	A00233	A02908	157
A00233	A02916	486	A00233	A02917	263
A00233	A02918	368	A00233	A02922	330
A00233	A02931	385	A00233	A02932	226
A00233	A02944	467	A00233	A02953	207
A00233	A02961	394	A00233	A02982	218
A00233	A02984	188	A00233	A02988	270
A00233	A03001	63	A00233	A03010	340
A00233	A03016	321	A00233	A03019	63
A00233	A03087	162	A00233	A03110	351
A00233	A03112	441	A00233	A03183	162
A00233	A03204	490	A00233	A03223	393
A00233	A03259	581	A00233	A03265	180
A00233	A03271	413	A00233	A03275	474
A00233	A03278	238	A00233	A03280	221
A00233	A03282	313	A00233	A03427	213
A00233	A03534	246	A00233	A03585	288

A00233	A03684	249	A00233	A03817	73
A00233	A03821	480	A00233	A03865	260
A00233	A03942	299	A00233	A03994	204
A00233	A04004	234	A00233	A04008	63
A00233	A04010	438	A00233	A04027	204
A00233	A04051	208	A00233	A04088	477
A00233	A04105	63	A00233	A04134	326
A00233	A04142	193	A00233	A04164	414
A00233	A04206	371	A00233	A04207	165
A00233	A04247	204	A00233	A04248	404
A00233	A04304	386	A00233	A04443	149
A00233	A04493	285	A00233	A04538	226
A00233	A04542	217	A00233	A04567	210
A00233	A04573	184	A00233	A04574	205
A00233	A04576	210	A00233	A04589	215
A00233	A04622	406	A00233	A04734	299
A00233	A04798	335	A00233	A04939	263
A00233	A04952	246	A00233	A04990	188
A00233	A05032	167	A00233	A05122	299
A00233	A05163	217	A00233	A05309	79
A00233	A05349	207	A00233	A05507	193
A00233	A05543	379	A00233	A05630	168
A00233	A05911	165	A00233	A06135	188
A00233	A06149	329	A00233	A06241	193
A00233	A06244	217	A00233	A06422	477
A00233	A06610	174	A00233	A06642	274
A00233	A06736	369	A00233	A06770	217
A00233	A06803	167	A00233	A07045	260
A00233	A07135	171	A00233	A07148	265
A00233	A07157	491	A00233	A07164	288
A00233	A07165	491	A00233	A07173	218
A00233	A07187	372	A00233	A07199	285
A00233	A07238	205	A00233	A07311	899
A00233	A07339	899	A00233	A07477	255
A00233	A07570	160	A00233	A07700	171
A00233	A07805	310	A00233	A07973	263
A00233	A07985	198	A00233	A08070	207
A00233	A08238	417	A00233	A08368	246
A00233	A08687	162	A00233	A08732	899
A00233	A08879	391	A00233	A09018	191
A00233	A09038	154	A00233	A09081	217
A00233	A09396	278	A00233	A09540	162
A00233	A09643	900	A00233	A10009	152
A00233	A10070	198	A00233	A10234	246
A00233	A10477	224	A00233	A10501	193
A00233	A10550	160	A00233	A10625	458

A00233	A10656	302	A00233	A10667	210
A00233	A10672	375	A00233	A10677	271
A00233	A10712	259	A00233	A10823	217
A00233	A11061	180	A00233	A11086	168
A00233	A11433	176	A00233	A11487	199
A00233	A11832	232	A00233	A12927	185
A00233	A13302	180	A00233	A13386	310
A00233	A13667	311	A00233	A13713	310
A00233	A13812	224	A00233	A13829	241
A00233	A14008	899	A00233	A14119	156
A00233	A14188	903	A00233	A14198	945
A00233	A14364	247	A00233	A14387	152
A00233	A14643	157	A00233	A14664	270
A00233	A14856	207	A00233	A15442	210
A00233	A15443	210	A00233	A15764	184
A00233	A15804	162	A00233	A15918	154
A00233	A16418	168	A00233	A16636	187
A00233	A17158	260	A00234	A00240	905
A00234	A00246	937	A00234	A00247	352
A00234	A00248	294	A00234	A00254	945
A00234	A00262	63	A00234	A00265	466
A00234	A00272	172	A00234	A00273	63
A00234	A00285	414	A00234	A00302	217
A00234	A00395	168	A00234	A00418	191
A00234	A00433	157	A00234	A00439	311
A00234	A00440	291	A00234	A00548	235
A00234	A00553	182	A00234	A00587	198
A00234	A00600	401	A00234	A00616	384
A00234	A00626	598	A00234	A00641	488
A00234	A00660	241	A00234	A00680	207
A00234	A00697	63	A00234	A00736	433
A00234	A00752	249	A00234	A00761	409
A00234	A00765	282	A00234	A00766	486
A00234	A00791	489	A00234	A00833	490
A00234	A00868	491	A00234	A00876	191
A00234	A00883	491	A00234	A00884	177
A00234	A00914	477	A00234	A00926	491
A00234	A00932	286	A00234	A00939	973
A00234	A00956	187	A00234	A00975	195
A00234	A00981	291	A00234	A01021	182
A00234	A01106	469	A00234	A01157	298
A00234	A01173	195	A00234	A01198	899
A00234	A01203	272	A00234	A01212	238
A00234	A01254	385	A00234	A01261	244
A00234	A01279	165	A00234	A01291	899
A00234	A01307	491	A00234	A01310	188

A00234	A01339	462	A00234	A01370	483
A00234	A01423	190	A00234	A01438	330
A00234	A01444	430	A00234	A01452	491
A00234	A01492	903	A00234	A01527	989
A00234	A01553	293	A00234	A01558	899
A00234	A01560	526	A00234	A01562	906
A00234	A01572	530	A00234	A01575	324
A00234	A01576	947	A00234	A01584	435
A00234	A01585	332	A00234	A01588	899
A00234	A01593	899	A00234	A01603	305
A00234	A01604	165	A00234	A01615	989
A00234	A01631	63	A00234	A01638	899
A00234	A01640	152	A00234	A01651	899
A00234	A01675	102	A00234	A01692	933
A00234	A01708	305	A00234	A01709	463
A00234	A01719	405	A00234	A01733	204
A00234	A01769	63	A00234	A01781	404
A00234	A01792	334	A00234	A01793	63
A00234	A01797	135	A00234	A01799	63
A00234	A01843	76	A00234	A01850	63
A00234	A01879	121	A00234	A01881	63
A00234	A01887	119	A00234	A01888	300
A00234	A01897	357	A00234	A01900	490
A00234	A01902	115	A00234	A01905	371
A00234	A01908	491	A00234	A01983	190
A00234	A02183	445	A00234	A02254	460
A00234	A02284	154	A00234	A02305	212
A00234	A02316	154	A00234	A02364	241
A00234	A02369	419	A00234	A02372	63
A00234	A02419	240	A00234	A02425	443
A00234	A02454	208	A00234	A02578	159
A00234	A02610	177	A00234	A02630	425
A00234	A02631	223	A00234	A02634	63
A00234	A02635	63	A00234	A02645	160
A00234	A02662	177	A00234	A02703	165
A00234	A02715	473	A00234	A02728	235
A00234	A02730	63	A00234	A02731	68
A00234	A02734	356	A00234	A02740	330
A00234	A02746	68	A00234	A02752	152
A00234	A02768	168	A00234	A02803	945
A00234	A02808	406	A00234	A02816	152
A00234	A02828	347	A00234	A02836	488
A00234	A02859	238	A00234	A02860	168
A00234	A02864	110	A00234	A02874	279
A00234	A02877	239	A00234	A02887	489
A00234	A02908	899	A00234	A02931	283

A00234	A02940	177	A00234	A02950	395
A00234	A02975	380	A00234	A03001	154
A00234	A03010	118	A00234	A03019	462
A00234	A03087	450	A00234	A03116	199
A00234	A03117	160	A00234	A03174	198
A00234	A03195	245	A00234	A03258	149
A00234	A03259	102	A00234	A03270	185
A00234	A03271	945	A00234	A03327	247
A00234	A03591	185	A00234	A03596	172
A00234	A03611	281	A00234	A03626	391
A00234	A03661	193	A00234	A03665	291
A00234	A03686	899	A00234	A03740	180
A00234	A03752	555	A00234	A03784	63
A00234	A03809	266	A00234	A03817	491
A00234	A03821	165	A00234	A03830	157
A00234	A03834	433	A00234	A03835	374
A00234	A03836	177	A00234	A03837	811
A00234	A03892	470	A00234	A03962	245
A00234	A04011	70	A00234	A04012	490
A00234	A04018	77	A00234	A04050	558
A00234	A04059	74	A00234	A04088	385
A00234	A04115	895	A00234	A04144	115
A00234	A04161	469	A00234	A04245	269
A00234	A04314	210	A00234	A04323	102
A00234	A04364	195	A00234	A04519	146
A00234	A04540	249	A00234	A04541	223
A00234	A04587	281	A00234	A04613	198
A00234	A04662	191	A00234	A04682	350
A00234	A04685	176	A00234	A04713	190
A00234	A04730	281	A00234	A04879	174
A00234	A04880	405	A00234	A04897	489
A00234	A04949	291	A00234	A05037	929
A00234	A05061	223	A00234	A05081	235
A00234	A05224	229	A00234	A05288	227
A00234	A05363	63	A00234	A05409	160
A00234	A05490	160	A00234	A05506	279
A00234	A05633	173	A00234	A05710	341
A00234	A06037	157	A00234	A06063	218
A00234	A06113	177	A00234	A06204	156
A00234	A06224	407	A00234	A06347	193
A00234	A06416	63	A00234	A06422	63
A00234	A06449	294	A00234	A06466	530
A00234	A06554	446	A00234	A06580	160
A00234	A06597	204	A00234	A06599	407
A00234	A06757	235	A00234	A06900	235
A00234	A06914	201	A00234	A07135	371

A00234	A07157	174	A00234	A07159	213
A00234	A07186	79	A00234	A07294	213
A00234	A07311	537	A00234	A07339	267
A00234	A07346	291	A00234	A07457	440
A00234	A07592	199	A00234	A07756	378
A00234	A07763	196	A00234	A07798	629
A00234	A07803	291	A00234	A07876	223
A00234	A08293	208	A00234	A08345	291
A00234	A08608	458	A00234	A08616	235
A00234	A08697	235	A00234	A08732	899
A00234	A08770	306	A00234	A08781	199
A00234	A09176	177	A00234	A09220	580
A00234	A09263	63	A00234	A09300	173
A00234	A09302	160	A00234	A09319	899
A00234	A09401	291	A00234	A09449	208
A00234	A09542	63	A00234	A09643	63
A00234	A09695	235	A00234	A09804	901
A00234	A09816	260	A00234	A09937	121
A00234	A10006	335	A00234	A10018	241
A00234	A10159	899	A00234	A10197	85
A00234	A10235	177	A00234	A10247	196
A00234	A10355	235	A00234	A10601	180
A00234	A10643	199	A00234	A10735	377
A00234	A10744	374	A00234	A10756	196
A00234	A10854	291	A00234	A11108	199
A00234	A11286	281	A00234	A11369	960
A00234	A11834	447	A00234	A11929	165
A00234	A11931	457	A00234	A12009	539
A00234	A12042	267	A00234	A12222	199
A00234	A12304	185	A00234	A12328	465
A00234	A12503	198	A00234	A12792	170
A00234	A12824	489	A00234	A12835	173
A00234	A12863	270	A00234	A12886	68
A00234	A13105	177	A00234	A13147	190
A00234	A13216	182	A00234	A13335	257
A00234	A13340	489	A00234	A13498	160
A00234	A13553	160	A00234	A13620	201
A00234	A13744	235	A00234	A13824	942
A00234	A13828	235	A00234	A13830	358
A00234	A13975	199	A00234	A13983	153
A00234	A13986	249	A00234	A13989	899
A00234	A14019	899	A00234	A14186	462
A00234	A14187	184	A00234	A14188	446
A00234	A14190	246	A00234	A14196	952
A00234	A14198	945	A00234	A14208	970
A00234	A14218	315	A00234	A14364	483

A00234	A14366	399	A00234	A14430	489
A00234	A14433	323	A00234	A14443	899
A00234	A14564	235	A00234	A14662	899
A00234	A14679	330	A00234	A14738	345
A00234	A15182	434	A00234	A15217	246
A00234	A15244	628	A00234	A15291	241
A00234	A15296	416	A00234	A15297	899
A00234	A15314	554	A00234	A15323	915
A00234	A15324	899	A00234	A15330	198
A00234	A15353	899	A00234	A15359	899
A00234	A15361	210	A00234	A15386	623
A00234	A15388	303	A00234	A15430	177
A00234	A15502	485	A00234	A15510	613
A00234	A15546	279	A00234	A15578	236
A00234	A15620	163	A00234	A15639	184
A00234	A15712	271	A00234	A15724	249
A00234	A15726	457	A00234	A15861	235
A00234	A15862	160	A00234	A15903	235
A00234	A15935	490	A00234	A15976	188
A00234	A15994	199	A00234	A16216	532
A00234	A16217	390	A00234	A16437	160
A00234	A16547	625	A00234	A16989	235
A00235	A00263	475	A00235	A00269	462
A00235	A00270	486	A00235	A00275	899
A00235	A00388	367	A00235	A00400	324
A00235	A00531	208	A00235	A00654	199
A00235	A00930	311	A00235	A01139	162
A00235	A01359	347	A00235	A01472	138
A00235	A01483	210	A00235	A01527	285
A00235	A01558	466	A00235	A01560	283
A00235	A01588	343	A00235	A01591	446
A00235	A01592	160	A00235	A01612	193
A00235	A01615	460	A00235	A01636	162
A00235	A01637	187	A00235	A01650	357
A00235	A01651	165	A00235	A01685	401
A00235	A01717	281	A00235	A02189	174
A00235	A02636	226	A00235	A03298	151
A00235	A03299	165	A00235	A03406	167
A00235	A03422	491	A00235	A03528	454
A00235	A03566	432	A00235	A03637	480
A00235	A03701	185	A00235	A05008	263
A00235	A05015	477	A00235	A05219	333
A00235	A05344	205	A00235	A06176	213
A00235	A06348	296	A00235	A06379	162
A00235	A06495	154	A00235	A07422	215
A00235	A07834	273	A00235	A08163	221

A00235	A08554	157	A00235	A08571	491
A00235	A08707	419	A00235	A08885	193
A00235	A09100	177	A00235	A09146	205
A00235	A09182	180	A00235	A09256	945
A00235	A09258	371	A00235	A09317	226
A00235	A09448	252	A00235	A09485	173
A00235	A09511	183	A00235	A09523	313
A00235	A09945	339	A00235	A09996	224
A00235	A10250	399	A00235	A10293	265
A00235	A10354	205	A00235	A10580	229
A00235	A10695	157	A00235	A10824	195
A00235	A11258	328	A00235	A11478	232
A00235	A12553	283	A00235	A12952	185
A00235	A13990	319	A00235	A14222	528
A00235	A14484	477	A00235	A14633	457
A00235	A15726	945	A00235	A15926	390
A00235	A16102	283	A00235	A16503	229
A00236	A00239	418	A00236	A00240	945
A00236	A00242	160	A00236	A00246	989
A00236	A00252	993	A00236	A00253	993
A00236	A00254	551	A00236	A00255	382
A00236	A00265	475	A00236	A00268	213
A00236	A00271	899	A00236	A00293	419
A00236	A00295	373	A00236	A00324	902
A00236	A00331	126	A00236	A00546	417
A00236	A00552	174	A00236	A00589	235
A00236	A00621	313	A00236	A00624	163
A00236	A00630	483	A00236	A00652	491
A00236	A00747	490	A00236	A00852	425
A00236	A00864	174	A00236	A00876	484
A00236	A00887	328	A00236	A01045	491
A00236	A01052	903	A00236	A01098	235
A00236	A01104	63	A00236	A01140	380
A00236	A01149	458	A00236	A01178	489
A00236	A01209	199	A00236	A01289	450
A00236	A01405	218	A00236	A01425	491
A00236	A01478	454	A00236	A01524	899
A00236	A01527	899	A00236	A01550	491
A00236	A01551	182	A00236	A01553	899
A00236	A01554	899	A00236	A01558	899
A00236	A01560	314	A00236	A01561	352
A00236	A01562	958	A00236	A01566	228
A00236	A01572	265	A00236	A01578	185
A00236	A01588	899	A00236	A01602	899
A00236	A01606	151	A00236	A01631	352
A00236	A01639	277	A00236	A01660	491

A00236	A01665	899	A00236	A01692	969
A00236	A01726	441	A00236	A01730	485
A00236	A01740	480	A00236	A01741	257
A00236	A01755	63	A00236	A01774	63
A00236	A01796	455	A00236	A01844	899
A00236	A01877	427	A00236	A01886	144
A00236	A01897	77	A00236	A02325	491
A00236	A02418	435	A00236	A02421	168
A00236	A02434	177	A00236	A02494	479
A00236	A02502	928	A00236	A02544	944
A00236	A02647	154	A00236	A02698	378
A00236	A02703	485	A00236	A02786	483
A00236	A02816	485	A00236	A02826	168
A00236	A02853	168	A00236	A02860	480
A00236	A02877	63	A00236	A02895	344
A00236	A02902	434	A00236	A02912	288
A00236	A02924	66	A00236	A02965	241
A00236	A03057	323	A00236	A03171	195
A00236	A03237	375	A00236	A03286	191
A00236	A03338	216	A00236	A03451	452
A00236	A03472	371	A00236	A03482	179
A00236	A03488	260	A00236	A03517	452
A00236	A03575	899	A00236	A03591	642
A00236	A03604	154	A00236	A03752	252
A00236	A03811	207	A00236	A03819	165
A00236	A03886	160	A00236	A03979	279
A00236	A04191	400	A00236	A04312	228
A00236	A04342	491	A00236	A04446	179
A00236	A04565	207	A00236	A04622	404
A00236	A04715	279	A00236	A04847	179
A00236	A05077	263	A00236	A05418	490
A00236	A05811	440	A00236	A05867	207
A00236	A05902	115	A00236	A06324	218
A00236	A06403	154	A00236	A06466	479
A00236	A06654	68	A00236	A07091	899
A00236	A07163	423	A00236	A07221	216
A00236	A07296	154	A00236	A07311	953
A00236	A07399	308	A00236	A07422	945
A00236	A08280	163	A00236	A08489	152
A00236	A08532	461	A00236	A08533	199
A00236	A08799	179	A00236	A08815	408
A00236	A08858	899	A00236	A08899	945
A00236	A09319	899	A00236	A09423	374
A00236	A09434	252	A00236	A09995	229
A00236	A10081	260	A00236	A10233	945
A00236	A10311	179	A00236	A10346	445

A00236	A10532	378	A00236	A10580	899
A00236	A10611	168	A00236	A10623	213
A00236	A11187	179	A00236	A11293	412
A00236	A11455	160	A00236	A11921	491
A00236	A13255	470	A00236	A13340	245
A00236	A13549	179	A00236	A13795	160
A00236	A13977	993	A00236	A13984	324
A00236	A14198	899	A00236	A14333	236
A00236	A14402	154	A00236	A14433	159
A00236	A14443	899	A00236	A14648	435
A00236	A15320	171	A00236	A15356	435
A00236	A15483	302	A00236	A15506	235
A00236	A16080	933	A00236	A16500	263
A00236	A17188	324	A00237	A00270	709
A00237	A00400	450	A00237	A00469	246
A00237	A00471	730	A00237	A00588	491
A00237	A00911	643	A00237	A01099	491
A00237	A01124	643	A00237	A01212	809
A00237	A01329	374	A00237	A01341	385
A00237	A01459	414	A00237	A01594	489
A00237	A01680	643	A00237	A02424	375
A00237	A05065	262	A00237	A05959	182
A00237	A06037	436	A00237	A07433	151
A00237	A07603	490	A00237	A07670	360
A00237	A08516	631	A00237	A08812	229
A00237	A09000	343	A00237	A09076	157
A00237	A09517	484	A00237	A09647	455
A00237	A09687	191	A00237	A09769	388
A00237	A10271	306	A00237	A10373	488
A00237	A11236	489	A00237	A11302	170
A00237	A12556	490	A00237	A12681	490
A00237	A14094	385	A00237	A14282	196
A00237	A14477	641	A00237	A14498	343
A00237	A14674	643	A00237	A14688	388
A00237	A14702	421	A00237	A15054	204
A00237	A16299	445	A00237	A16469	260
A00237	A16470	212	A00237	A16738	643
A00237	A17068	198	A00238	A00338	435
A00238	A00485	288	A00238	A00657	170
A00238	A00694	441	A00238	A00799	490
A00238	A00914	251	A00238	A01022	243
A00238	A01121	168	A00238	A01161	483
A00238	A01343	165	A00238	A01397	162
A00238	A01483	246	A00238	A01527	940
A00238	A01551	213	A00238	A01615	899
A00238	A01632	477	A00238	A01666	481

A00238	A01691	170	A00238	A01732	228
A00238	A01743	279	A00238	A01744	208
A00238	A01887	235	A00238	A02183	172
A00238	A02415	151	A00238	A02618	224
A00238	A02629	330	A00238	A02701	170
A00238	A02790	162	A00238	A02918	251
A00238	A03014	201	A00238	A03211	343
A00238	A03406	435	A00238	A03419	177
A00238	A03420	188	A00238	A03451	256
A00238	A03611	152	A00238	A03977	238
A00238	A04140	173	A00238	A04334	277
A00238	A04424	198	A00238	A04988	198
A00238	A05076	263	A00238	A05503	317
A00238	A05504	310	A00238	A05516	191
A00238	A05548	240	A00238	A05810	191
A00238	A05855	317	A00238	A06235	160
A00238	A07442	177	A00238	A07723	236
A00238	A07757	187	A00238	A07900	318
A00238	A07935	474	A00238	A08635	227
A00238	A09388	208	A00238	A09541	297
A00238	A11546	297	A00238	A11684	469
A00238	A14206	227	A00238	A14387	247
A00238	A15349	182	A00238	A15596	201
A00238	A15641	188	A00238	A16074	162
A00238	A16732	273	A00238	A17085	182
A00239	A00240	996	A00239	A00241	974
A00239	A00242	945	A00239	A00243	986
A00239	A00244	967	A00239	A00246	899
A00239	A00247	63	A00239	A00252	279
A00239	A00253	273	A00239	A00254	217
A00239	A00255	235	A00239	A00261	614
A00239	A00296	157	A00239	A00331	329
A00239	A00643	450	A00239	A00652	229
A00239	A00691	322	A00239	A00808	430
A00239	A00883	168	A00239	A00887	165
A00239	A00911	485	A00239	A00932	185
A00239	A00981	154	A00239	A01088	255
A00239	A01148	380	A00239	A01161	151
A00239	A01213	296	A00239	A01289	434
A00239	A01310	219	A00239	A01507	228
A00239	A01512	419	A00239	A01524	914
A00239	A01527	917	A00239	A01546	468
A00239	A01553	994	A00239	A01554	585
A00239	A01558	989	A00239	A01560	945
A00239	A01576	154	A00239	A01588	899
A00239	A01596	675	A00239	A01623	945

A00239	A01638	219	A00239	A01639	395
A00239	A01680	286	A00239	A01692	527
A00239	A01697	165	A00239	A01702	159
A00239	A01709	452	A00239	A01718	377
A00239	A01719	229	A00239	A01730	213
A00239	A01747	333	A00239	A01910	210
A00239	A01920	608	A00239	A01974	967
A00239	A02052	180	A00239	A02143	469
A00239	A02488	174	A00239	A02494	249
A00239	A02502	450	A00239	A02724	210
A00239	A02791	404	A00239	A03185	188
A00239	A03451	408	A00239	A03598	184
A00239	A03822	193	A00239	A04196	200
A00239	A04380	200	A00239	A04759	326
A00239	A04807	152	A00239	A04909	361
A00239	A05118	246	A00239	A06211	180
A00239	A06601	160	A00239	A06610	372
A00239	A06987	180	A00239	A07227	246
A00239	A07311	949	A00239	A07422	267
A00239	A07568	249	A00239	A07891	188
A00239	A08118	291	A00239	A08427	180
A00239	A08571	415	A00239	A09026	162
A00239	A09162	224	A00239	A09319	535
A00239	A09374	160	A00239	A09577	602
A00239	A09804	373	A00239	A09871	172
A00239	A10076	196	A00239	A10179	257
A00239	A10201	177	A00239	A10365	427
A00239	A10560	177	A00239	A10580	358
A00239	A10623	245	A00239	A10624	488
A00239	A10743	241	A00239	A10808	346
A00239	A10811	308	A00239	A10902	168
A00239	A10913	177	A00239	A11125	473
A00239	A11206	160	A00239	A11262	167
A00239	A11335	443	A00239	A11590	174
A00239	A11707	224	A00239	A11943	484
A00239	A12666	944	A00239	A13250	291
A00239	A13391	156	A00239	A13705	319
A00239	A13977	160	A00239	A13984	332
A00239	A14045	271	A00239	A14068	417
A00239	A14188	351	A00239	A14299	614
A00239	A14642	179	A00239	A14704	291
A00239	A15196	184	A00239	A15699	156
A00239	A16222	160	A00239	A17197	293
A00240	A00241	956	A00240	A00242	994
A00240	A00243	991	A00240	A00244	945
A00240	A00246	916	A00240	A00252	461

A00240	A00253	405	A00240	A00254	477
A00240	A00255	958	A00240	A00257	333
A00240	A00261	602	A00240	A00270	899
A00240	A00272	569	A00240	A00324	944
A00240	A00338	372	A00240	A00470	899
A00240	A00531	899	A00240	A00533	235
A00240	A00652	455	A00240	A00669	490
A00240	A00691	227	A00240	A00763	154
A00240	A00808	486	A00240	A00809	899
A00240	A00887	360	A00240	A00900	906
A00240	A00911	469	A00240	A00914	899
A00240	A00922	154	A00240	A00932	677
A00240	A00987	160	A00240	A00997	263
A00240	A01003	200	A00240	A01052	475
A00240	A01088	168	A00240	A01178	285
A00240	A01198	899	A00240	A01212	167
A00240	A01213	214	A00240	A01289	458
A00240	A01291	452	A00240	A01425	440
A00240	A01435	417	A00240	A01450	235
A00240	A01512	950	A00240	A01524	477
A00240	A01527	944	A00240	A01550	912
A00240	A01553	945	A00240	A01554	945
A00240	A01558	990	A00240	A01560	994
A00240	A01561	910	A00240	A01562	945
A00240	A01572	206	A00240	A01588	989
A00240	A01596	713	A00240	A01601	522
A00240	A01605	491	A00240	A01606	326
A00240	A01615	925	A00240	A01623	491
A00240	A01628	916	A00240	A01639	945
A00240	A01640	472	A00240	A01680	936
A00240	A01688	369	A00240	A01692	536
A00240	A01697	180	A00240	A01702	368
A00240	A01708	378	A00240	A01709	490
A00240	A01718	952	A00240	A01719	219
A00240	A01730	384	A00240	A01748	179
A00240	A01755	156	A00240	A01798	232
A00240	A01844	927	A00240	A01850	402
A00240	A01920	403	A00240	A01974	491
A00240	A02143	363	A00240	A02254	168
A00240	A02424	899	A00240	A02502	486
A00240	A02642	899	A00240	A02791	324
A00240	A02836	545	A00240	A02908	899
A00240	A02942	899	A00240	A02968	899
A00240	A03087	258	A00240	A03095	223
A00240	A03171	899	A00240	A03209	899
A00240	A03451	374	A00240	A03575	217

A00240	A03775	905	A00240	A03817	899
A00240	A03822	412	A00240	A03830	218
A00240	A03854	446	A00240	A03971	236
A00240	A03981	160	A00240	A04062	473
A00240	A04074	230	A00240	A04266	160
A00240	A04280	414	A00240	A04347	573
A00240	A04359	179	A00240	A04413	160
A00240	A04414	263	A00240	A04534	154
A00240	A04682	900	A00240	A04699	477
A00240	A04909	283	A00240	A05062	157
A00240	A05065	267	A00240	A05082	490
A00240	A05118	179	A00240	A05464	191
A00240	A05553	259	A00240	A05606	899
A00240	A05701	355	A00240	A05952	179
A00240	A05969	207	A00240	A05970	179
A00240	A06256	168	A00240	A06289	162
A00240	A06398	207	A00240	A06601	429
A00240	A06610	489	A00240	A06615	425
A00240	A06821	265	A00240	A07032	263
A00240	A07311	997	A00240	A07398	899
A00240	A07422	486	A00240	A07834	188
A00240	A08118	291	A00240	A08282	263
A00240	A08607	228	A00240	A08732	899
A00240	A09158	154	A00240	A09162	420
A00240	A09319	951	A00240	A09476	393
A00240	A09544	160	A00240	A09577	945
A00240	A09660	191	A00240	A09698	171
A00240	A09804	923	A00240	A09871	391
A00240	A09937	157	A00240	A10076	408
A00240	A10096	207	A00240	A10167	899
A00240	A10174	899	A00240	A10365	440
A00240	A10386	236	A00240	A10499	355
A00240	A10560	396	A00240	A10580	784
A00240	A10615	899	A00240	A10623	483
A00240	A10624	374	A00240	A10743	469
A00240	A10801	899	A00240	A10808	468
A00240	A10811	207	A00240	A10822	160
A00240	A10913	396	A00240	A10981	191
A00240	A10982	477	A00240	A11125	944
A00240	A11252	191	A00240	A11262	899
A00240	A11335	278	A00240	A11460	229
A00240	A11620	228	A00240	A11707	473
A00240	A11943	945	A00240	A12018	168
A00240	A12103	168	A00240	A12341	899
A00240	A12664	474	A00240	A12666	272
A00240	A12810	286	A00240	A12990	539

A00240	A12997	179	A00240	A13146	913
A00240	A13250	291	A00240	A13292	899
A00240	A13390	168	A00240	A13391	420
A00240	A13549	179	A00240	A13672	330
A00240	A13705	263	A00240	A13804	221
A00240	A13975	899	A00240	A13977	364
A00240	A13978	933	A00240	A13980	236
A00240	A13981	899	A00240	A13992	330
A00240	A14019	899	A00240	A14030	445
A00240	A14045	338	A00240	A14046	228
A00240	A14062	582	A00240	A14068	286
A00240	A14188	935	A00240	A14191	191
A00240	A14215	899	A00240	A14259	899
A00240	A14299	958	A00240	A14399	263
A00240	A14443	439	A00240	A14444	942
A00240	A14642	402	A00240	A14704	291
A00240	A14917	172	A00240	A14961	228
A00240	A14964	335	A00240	A15096	456
A00240	A15196	241	A00240	A16050	160
A00240	A16400	174	A00240	A16418	168
A00240	A16438	201	A00240	A16788	207
A00240	A16792	276	A00240	A16905	202
A00240	A16944	210	A00240	A17213	899
A00241	A00242	945	A00241	A00243	811
A00241	A00244	945	A00241	A00246	923
A00241	A00257	316	A00241	A00261	615
A00241	A00320	240	A00241	A00428	343
A00241	A00660	193	A00241	A00671	187
A00241	A00911	247	A00241	A01363	450
A00241	A01527	899	A00241	A01553	935
A00241	A01558	899	A00241	A01560	940
A00241	A01588	899	A00241	A01596	613
A00241	A01623	483	A00241	A01700	185
A00241	A01920	517	A00241	A01974	756
A00241	A01977	174	A00241	A02711	371
A00241	A02791	313	A00241	A03321	232
A00241	A04033	241	A00241	A04909	165
A00241	A05118	272	A00241	A05181	436
A00241	A07311	930	A00241	A07803	165
A00241	A07891	217	A00241	A08118	291
A00241	A08377	201	A00241	A08416	235
A00241	A08550	165	A00241	A09060	174
A00241	A09121	191	A00241	A09529	156
A00241	A09609	213	A00241	A10076	224
A00241	A10615	899	A00241	A10902	198
A00241	A11125	174	A00241	A11472	154

A00241	A11543	299	A00241	A11707	260
A00241	A11914	154	A00241	A12204	196
A00241	A12658	205	A00241	A12666	932
A00241	A13169	165	A00241	A13250	291
A00241	A13527	160	A00241	A13787	243
A00241	A13788	162	A00241	A13804	198
A00241	A13809	213	A00241	A14068	249
A00241	A14188	168	A00241	A14204	367
A00241	A14299	614	A00241	A14569	221
A00241	A14704	291	A00241	A14739	256
A00241	A14917	160	A00241	A15606	228
A00241	A15726	358	A00241	A16157	172
A00241	A16243	168	A00241	A16565	191
A00241	A16566	176	A00241	A16567	201
A00241	A16587	286	A00241	A16680	172
A00241	A16691	219	A00241	A16701	193
A00241	A16788	294	A00241	A16815	228
A00241	A16874	165	A00242	A00243	491
A00242	A00244	945	A00242	A00246	899
A00242	A00254	154	A00242	A00255	456
A00242	A00324	899	A00242	A00439	229
A00242	A00669	213	A00242	A00808	414
A00242	A00811	152	A00242	A00932	188
A00242	A00933	299	A00242	A01157	180
A00242	A01289	252	A00242	A01435	288
A00242	A01553	488	A00242	A01554	339
A00242	A01558	899	A00242	A01560	945
A00242	A01561	899	A00242	A01588	899
A00242	A01605	193	A00242	A01623	462
A00242	A01639	394	A00242	A01640	931
A00242	A01680	899	A00242	A01692	452
A00242	A01709	259	A00242	A01974	491
A00242	A02424	899	A00242	A02498	193
A00242	A03143	200	A00242	A03209	218
A00242	A03594	259	A00242	A03822	210
A00242	A04280	162	A00242	A04347	219
A00242	A04909	218	A00242	A05118	313
A00242	A06256	307	A00242	A07311	486
A00242	A08607	347	A00242	A09577	243
A00242	A10365	174	A00242	A10960	159
A00242	A11124	185	A00242	A11335	213
A00242	A11707	470	A00242	A12990	923
A00242	A13549	313	A00242	A14188	903
A00242	A14444	335	A00243	A00244	966
A00243	A00247	297	A00243	A00257	443
A00243	A00261	616	A00243	A00356	189

A00243	A00421	249	A00243	A00651	183
A00243	A00667	335	A00243	A00691	293
A00243	A00697	286	A00243	A00709	481
A00243	A00771	329	A00243	A00883	151
A00243	A00911	491	A00243	A00919	391
A00243	A00932	585	A00243	A00972	329
A00243	A00983	391	A00243	A01088	221
A00243	A01139	391	A00243	A01148	423
A00243	A01213	268	A00243	A01289	301
A00243	A01405	391	A00243	A01507	575
A00243	A01524	899	A00243	A01527	899
A00243	A01558	899	A00243	A01560	413
A00243	A01588	899	A00243	A01596	712
A00243	A01601	379	A00243	A01623	491
A00243	A01639	198	A00243	A01692	373
A00243	A01718	577	A00243	A01920	615
A00243	A01974	682	A00243	A02791	402
A00243	A03451	380	A00243	A03858	329
A00243	A03877	238	A00243	A03971	414
A00243	A04347	380	A00243	A04449	167
A00243	A05054	193	A00243	A05065	221
A00243	A05101	157	A00243	A06869	283
A00243	A06941	188	A00243	A06985	251
A00243	A07557	160	A00243	A08118	291
A00243	A08282	283	A00243	A09319	271
A00243	A10076	152	A00243	A10179	414
A00243	A10580	393	A00243	A10811	246
A00243	A10864	283	A00243	A11109	160
A00243	A11521	157	A00243	A11736	251
A00243	A12666	934	A00243	A12824	249
A00243	A12837	171	A00243	A12989	251
A00243	A12990	329	A00243	A13063	329
A00243	A13091	156	A00243	A13250	310
A00243	A13391	235	A00243	A13571	372
A00243	A14005	310	A00243	A14046	372
A00243	A14068	469	A00243	A14204	491
A00243	A14229	171	A00243	A14299	616
A00243	A14444	301	A00243	A14642	428
A00243	A14704	299	A00243	A15096	180
A00243	A15321	296	A00243	A15322	488
A00243	A15347	527	A00244	A00246	899
A00244	A01554	296	A00244	A01558	899
A00244	A01588	899	A00244	A01623	412
A00244	A01974	472	A00244	A05082	268
A00244	A06610	156	A00244	A10808	210
A00244	A12666	338	A00244	A14299	199

A00244	A14444	346	A00245	A00270	177
A00245	A00274	205	A00245	A00324	170
A00245	A00363	193	A00245	A00370	185
A00245	A00440	165	A00245	A00616	475
A00245	A00643	257	A00245	A00752	328
A00245	A00826	196	A00245	A00831	177
A00245	A00883	184	A00245	A00902	374
A00245	A00914	165	A00245	A00958	334
A00245	A01072	388	A00245	A01088	477
A00245	A01091	151	A00245	A01112	235
A00245	A01137	265	A00245	A01140	394
A00245	A01157	350	A00245	A01166	154
A00245	A01235	446	A00245	A01257	491
A00245	A01299	446	A00245	A01305	154
A00245	A01425	259	A00245	A01468	302
A00245	A01470	187	A00245	A01485	63
A00245	A01492	467	A00245	A01524	899
A00245	A01598	479	A00245	A01601	402
A00245	A01605	171	A00245	A01614	217
A00245	A01668	173	A00245	A01903	174
A00245	A01931	221	A00245	A02148	85
A00245	A02174	435	A00245	A02212	156
A00245	A02228	432	A00245	A02260	152
A00245	A02265	265	A00245	A02304	458
A00245	A02306	180	A00245	A02313	395
A00245	A02379	334	A00245	A02383	281
A00245	A02431	475	A00245	A02482	326
A00245	A02554	238	A00245	A02667	82
A00245	A02673	472	A00245	A02759	110
A00245	A02959	468	A00245	A03056	238
A00245	A03076	491	A00245	A03104	223
A00245	A03110	279	A00245	A03183	208
A00245	A03186	63	A00245	A03219	190
A00245	A03264	165	A00245	A03292	247
A00245	A03304	489	A00245	A03310	188
A00245	A03314	435	A00245	A03436	165
A00245	A03451	232	A00245	A03584	99
A00245	A03639	491	A00245	A03818	278
A00245	A03893	429	A00245	A03910	363
A00245	A03925	267	A00245	A03942	451
A00245	A03961	491	A00245	A03963	452
A00245	A04002	104	A00245	A04033	374
A00245	A04169	407	A00245	A04202	435
A00245	A04256	227	A00245	A04282	266
A00245	A04331	228	A00245	A04380	223
A00245	A04449	273	A00245	A04525	276

A00245	A04527	412	A00245	A04597	228
A00245	A04608	163	A00245	A04976	341
A00245	A05184	152	A00245	A05221	216
A00245	A05270	291	A00245	A05439	213
A00245	A05533	213	A00245	A05653	208
A00245	A05736	183	A00245	A05786	232
A00245	A05992	274	A00245	A06273	191
A00245	A06340	332	A00245	A06371	188
A00245	A06601	191	A00245	A06610	379
A00245	A06727	299	A00245	A06762	358
A00245	A07019	254	A00245	A07021	278
A00245	A07209	414	A00245	A07296	243
A00245	A07297	251	A00245	A07339	328
A00245	A07559	232	A00245	A07793	198
A00245	A07912	232	A00245	A08714	236
A00245	A08859	489	A00245	A09062	210
A00245	A09074	223	A00245	A09200	185
A00245	A09235	299	A00245	A09238	260
A00245	A09391	377	A00245	A09480	333
A00245	A09550	447	A00245	A09732	489
A00245	A09733	488	A00245	A09955	347
A00245	A10164	435	A00245	A10218	305
A00245	A10665	208	A00245	A11014	386
A00245	A11300	343	A00245	A11387	285
A00245	A11415	452	A00245	A11464	193
A00245	A11653	170	A00245	A11983	171
A00245	A12108	259	A00245	A12152	188
A00245	A12365	399	A00245	A12390	446
A00245	A12393	443	A00245	A12854	212
A00245	A12897	302	A00245	A13546	425
A00245	A13558	167	A00245	A13989	463
A00245	A14210	462	A00245	A14364	297
A00245	A14370	152	A00245	A14371	215
A00245	A14511	489	A00245	A14643	182
A00245	A14667	420	A00245	A15226	223
A00245	A15258	163	A00245	A15268	160
A00245	A15292	486	A00245	A15312	322
A00245	A15313	210	A00245	A15349	383
A00245	A15431	252	A00245	A15433	246
A00245	A15435	329	A00245	A15437	183
A00245	A15452	483	A00245	A15457	323
A00245	A15495	480	A00245	A15671	188
A00245	A15820	157	A00245	A16778	218
A00245	A17177	334	A00246	A00250	63
A00246	A00254	945	A00246	A00255	899
A00246	A00257	899	A00246	A00261	669

A00246	A00270	277	A00246	A00294	899
A00246	A00324	989	A00246	A00378	396
A00246	A00669	899	A00246	A00856	458
A00246	A00864	899	A00246	A00911	291
A00246	A00914	899	A00246	A01052	899
A00246	A01070	899	A00246	A01473	899
A00246	A01474	224	A00246	A01507	899
A00246	A01512	899	A00246	A01524	899
A00246	A01527	992	A00246	A01553	992
A00246	A01554	906	A00246	A01558	989
A00246	A01559	899	A00246	A01560	899
A00246	A01561	899	A00246	A01562	899
A00246	A01572	899	A00246	A01588	989
A00246	A01593	899	A00246	A01602	899
A00246	A01605	902	A00246	A01612	899
A00246	A01615	899	A00246	A01628	899
A00246	A01638	899	A00246	A01639	899
A00246	A01640	899	A00246	A01651	899
A00246	A01680	899	A00246	A01688	899
A00246	A01692	899	A00246	A01708	899
A00246	A01709	899	A00246	A01718	904
A00246	A01719	899	A00246	A01747	170
A00246	A01797	63	A00246	A01830	152
A00246	A01844	989	A00246	A01880	185
A00246	A01900	121	A00246	A01914	517
A00246	A01920	921	A00246	A02143	899
A00246	A02327	157	A00246	A02372	117
A00246	A02502	899	A00246	A02639	485
A00246	A02711	338	A00246	A02908	899
A00246	A02942	899	A00246	A02968	899
A00246	A03010	82	A00246	A03110	899
A00246	A03126	902	A00246	A03335	899
A00246	A03685	274	A00246	A03784	252
A00246	A04323	115	A00246	A04398	260
A00246	A04451	489	A00246	A04622	63
A00246	A04651	160	A00246	A04682	119
A00246	A04795	199	A00246	A05065	191
A00246	A05082	899	A00246	A05164	216
A00246	A05216	899	A00246	A05854	899
A00246	A06404	176	A00246	A06608	899
A00246	A06906	263	A00246	A07091	899
A00246	A07260	323	A00246	A07311	899
A00246	A07422	899	A00246	A07884	263
A00246	A08278	899	A00246	A08732	899
A00246	A08860	402	A00246	A08941	360
A00246	A08944	243	A00246	A09124	899

A00246	A09181	899	A00246	A09220	899
A00246	A09225	263	A00246	A09238	232
A00246	A09319	899	A00246	A09422	465
A00246	A09506	154	A00246	A09542	899
A00246	A09568	899	A00246	A09577	899
A00246	A09803	152	A00246	A09855	321
A00246	A09870	201	A00246	A09937	441
A00246	A09982	294	A00246	A10392	900
A00246	A10560	899	A00246	A10580	899
A00246	A10623	899	A00246	A10633	318
A00246	A10801	899	A00246	A10808	901
A00246	A10811	899	A00246	A11125	899
A00246	A11369	916	A00246	A11512	323
A00246	A11707	112	A00246	A11943	899
A00246	A11983	899	A00246	A12353	187
A00246	A12383	989	A00246	A12393	473
A00246	A12664	899	A00246	A12666	899
A00246	A12681	193	A00246	A12705	899
A00246	A12886	152	A00246	A12971	899
A00246	A12992	899	A00246	A13250	899
A00246	A13390	899	A00246	A13391	899
A00246	A13605	899	A00246	A13923	294
A00246	A13975	899	A00246	A13980	899
A00246	A13981	899	A00246	A13982	899
A00246	A13984	899	A00246	A13985	899
A00246	A13991	899	A00246	A14007	899
A00246	A14027	899	A00246	A14038	899
A00246	A14042	899	A00246	A14043	899
A00246	A14072	899	A00246	A14198	993
A00246	A14242	899	A00246	A14402	899
A00246	A14438	899	A00246	A14443	989
A00246	A14444	989	A00246	A14930	899
A00246	A15182	899	A00246	A15241	168
A00246	A15290	165	A00246	A15293	899
A00246	A15294	899	A00246	A15783	210
A00246	A15952	294	A00246	A16085	899
A00246	A16772	461	A00246	A17060	899
A00246	A17160	899	A00247	A00254	221
A00247	A00257	412	A00247	A00263	160
A00247	A00285	491	A00247	A00297	185
A00247	A00298	343	A00247	A00337	423
A00247	A00340	724	A00247	A00356	474
A00247	A00358	177	A00247	A00366	165
A00247	A00630	385	A00247	A00643	255
A00247	A00651	457	A00247	A00698	486
A00247	A00826	414	A00247	A00839	435

A00247	A00876	208	A00247	A00922	281
A00247	A00932	491	A00247	A00980	531
A00247	A01055	405	A00247	A01112	491
A00247	A01125	314	A00247	A01212	362
A00247	A01268	191	A00247	A01291	538
A00247	A01309	414	A00247	A01410	146
A00247	A01496	247	A00247	A01524	939
A00247	A01527	945	A00247	A01540	483
A00247	A01541	173	A00247	A01544	176
A00247	A01553	226	A00247	A01558	899
A00247	A01560	945	A00247	A01561	294
A00247	A01579	490	A00247	A01687	290
A00247	A01707	151	A00247	A01718	491
A00247	A01782	154	A00247	A01850	286
A00247	A01995	262	A00247	A02110	378
A00247	A02239	440	A00247	A03571	176
A00247	A03591	207	A00247	A03740	235
A00247	A03752	491	A00247	A04347	353
A00247	A04449	221	A00247	A05086	396
A00247	A05120	281	A00247	A05240	187
A00247	A05416	226	A00247	A06197	291
A00247	A06466	655	A00247	A07004	165
A00247	A07311	558	A00247	A07433	184
A00247	A07726	350	A00247	A07798	491
A00247	A08214	296	A00247	A09076	384
A00247	A09176	263	A00247	A09220	363
A00247	A09263	293	A00247	A09297	246
A00247	A09577	395	A00247	A09804	411
A00247	A09888	185	A00247	A09902	404
A00247	A09906	348	A00247	A10165	378
A00247	A10365	390	A00247	A10430	472
A00247	A10532	428	A00247	A10534	168
A00247	A10728	480	A00247	A10801	311
A00247	A11286	165	A00247	A11440	224
A00247	A11487	332	A00247	A11697	157
A00247	A11937	363	A00247	A12084	219
A00247	A12225	348	A00247	A12328	462
A00247	A12990	491	A00247	A13149	435
A00247	A13310	180	A00247	A13311	457
A00247	A13340	491	A00247	A13605	899
A00247	A14073	486	A00247	A14188	333
A00247	A14635	404	A00247	A14642	322
A00247	A14655	440	A00247	A15551	469
A00247	A15682	254	A00247	A15684	182
A00247	A15760	162	A00247	A15932	299
A00247	A16272	388	A00247	A16446	174

A00247	A16542	205	A00247	A16847	472
A00248	A00256	461	A00248	A00259	347
A00248	A00260	335	A00248	A00264	358
A00248	A00265	490	A00248	A00266	916
A00248	A00616	367	A00248	A00676	177
A00248	A00887	899	A00248	A00914	294
A00248	A01524	899	A00248	A01527	899
A00248	A01554	899	A00248	A01558	899
A00248	A01588	899	A00248	A01596	252
A00248	A01604	176	A00248	A01626	188
A00248	A01639	402	A00248	A01654	486
A00248	A01669	945	A00248	A01725	389
A00248	A01729	390	A00248	A01730	291
A00248	A01741	199	A00248	A01742	490
A00248	A01743	296	A00248	A01746	491
A00248	A01756	414	A00248	A01776	224
A00248	A01781	463	A00248	A01850	348
A00248	A01870	177	A00248	A01876	479
A00248	A01879	396	A00248	A01880	385
A00248	A01888	475	A00248	A01900	945
A00248	A01902	207	A00248	A01981	232
A00248	A01983	412	A00248	A02183	185
A00248	A02292	159	A00248	A02337	424
A00248	A02370	182	A00248	A02383	154
A00248	A02490	283	A00248	A02505	312
A00248	A02587	208	A00248	A02611	303
A00248	A02637	445	A00248	A02670	312
A00248	A02699	443	A00248	A02746	174
A00248	A02769	184	A00248	A02800	180
A00248	A02808	281	A00248	A02812	434
A00248	A02821	283	A00248	A02854	427
A00248	A02863	208	A00248	A02866	362
A00248	A02871	338	A00248	A02873	472
A00248	A02886	382	A00248	A02896	283
A00248	A02906	191	A00248	A02909	469
A00248	A02917	425	A00248	A02945	367
A00248	A02983	375	A00248	A03095	475
A00248	A03218	450	A00248	A03259	302
A00248	A03269	174	A00248	A03287	157
A00248	A03319	213	A00248	A03541	489
A00248	A03612	121	A00248	A03662	488
A00248	A03817	205	A00248	A03826	201
A00248	A03846	255	A00248	A03848	213
A00248	A03869	385	A00248	A03871	393
A00248	A03942	434	A00248	A04049	402
A00248	A04063	288	A00248	A04088	168

A00248	A04089	208	A00248	A04109	228
A00248	A04134	232	A00248	A04248	486
A00248	A04272	179	A00248	A04320	374
A00248	A04415	163	A00248	A04536	491
A00248	A04577	312	A00248	A04584	236
A00248	A04603	357	A00248	A04627	183
A00248	A04635	224	A00248	A04734	338
A00248	A04866	224	A00248	A04941	154
A00248	A04961	191	A00248	A04996	278
A00248	A05041	243	A00248	A05288	262
A00248	A05305	281	A00248	A05436	243
A00248	A05551	294	A00248	A05556	424
A00248	A05558	432	A00248	A05583	243
A00248	A05636	338	A00248	A05810	265
A00248	A05944	481	A00248	A05985	265
A00248	A06055	491	A00248	A06415	224
A00248	A06422	272	A00248	A06609	899
A00248	A06650	328	A00248	A07031	190
A00248	A07142	224	A00248	A07157	216
A00248	A07158	221	A00248	A07161	232
A00248	A07164	162	A00248	A07173	454
A00248	A07205	235	A00248	A07372	219
A00248	A07390	167	A00248	A08615	385
A00248	A08658	228	A00248	A08711	366
A00248	A09046	312	A00248	A09220	168
A00248	A09374	188	A00248	A09394	170
A00248	A09673	466	A00248	A10018	193
A00248	A10332	291	A00248	A10392	185
A00248	A10412	338	A00248	A10580	899
A00248	A10969	263	A00248	A11473	383
A00248	A11894	281	A00248	A12662	195
A00248	A14416	195	A00248	A14432	439
A00248	A14969	390	A00248	A15601	157
A00248	A15603	366	A00248	A15605	334
A00248	A15981	170	A00248	A16870	312
A00249	A14204	63	A00249	A15303	63
A00249	A16891	63	A00249	A16910	63
A00250	A00257	469	A00250	A00265	351
A00250	A00271	899	A00250	A00427	260
A00250	A00630	157	A00250	A00825	477
A00250	A01475	221	A00250	A01553	446
A00250	A01554	908	A00250	A01558	899
A00250	A01562	279	A00250	A01572	163
A00250	A01588	899	A00250	A01596	581
A00250	A01631	408	A00250	A01639	935
A00250	A01692	152	A00250	A01709	899

A00250	A01730	943	A00250	A01850	196
A00250	A01901	63	A00250	A01975	259
A00250	A02088	185	A00250	A02185	299
A00250	A02724	193	A00250	A02737	149
A00250	A02825	77	A00250	A02828	185
A00250	A02830	182	A00250	A03213	232
A00250	A03259	899	A00250	A03266	247
A00250	A03273	191	A00250	A03656	168
A00250	A03892	172	A00250	A04010	413
A00250	A04104	63	A00250	A04342	152
A00250	A04537	168	A00250	A04540	321
A00250	A04639	380	A00250	A05064	480
A00250	A05530	347	A00250	A06166	205
A00250	A06416	71	A00250	A06466	440
A00250	A06721	195	A00250	A06748	276
A00250	A06936	256	A00250	A07163	212
A00250	A07422	900	A00250	A07933	441
A00250	A08211	172	A00250	A09383	934
A00250	A09409	241	A00250	A09543	221
A00250	A09577	486	A00250	A09643	927
A00250	A10014	177	A00250	A10510	179
A00250	A10580	943	A00250	A10623	906
A00250	A10811	899	A00250	A10902	285
A00250	A11124	205	A00250	A11601	151
A00250	A11911	223	A00250	A11943	184
A00250	A12141	306	A00250	A12263	276
A00250	A12359	306	A00250	A12383	945
A00250	A12431	256	A00250	A12726	151
A00250	A12850	171	A00250	A13511	452
A00250	A13562	229	A00250	A13589	288
A00250	A13711	306	A00250	A13809	152
A00250	A14008	240	A00250	A14022	899
A00250	A14073	468	A00250	A14443	504
A00250	A14455	151	A00250	A14921	199
A00250	A15034	224	A00250	A15692	899
A00250	A15745	366	A00250	A16145	243
A00250	A16703	198	A00250	A16799	156
A00250	A16806	193	A00250	A16824	174
A00251	A01524	899	A00251	A01558	899
A00251	A01588	899	A00251	A03209	899
A00252	A00253	995	A00252	A00271	899
A00252	A00284	232	A00252	A00293	486
A00252	A00295	441	A00252	A00324	911
A00252	A00543	279	A00252	A00546	260
A00252	A00589	221	A00252	A00621	301
A00252	A00624	157	A00252	A00630	412

A00252	A00652	491	A00252	A00747	480
A00252	A00832	899	A00252	A00852	396
A00252	A00876	491	A00252	A00887	899
A00252	A00981	185	A00252	A00999	369
A00252	A01045	491	A00252	A01052	927
A00252	A01149	477	A00252	A01178	489
A00252	A01289	363	A00252	A01405	455
A00252	A01406	339	A00252	A01425	491
A00252	A01478	334	A00252	A01492	899
A00252	A01524	989	A00252	A01527	900
A00252	A01550	491	A00252	A01551	182
A00252	A01554	899	A00252	A01558	989
A00252	A01560	199	A00252	A01562	958
A00252	A01566	274	A00252	A01572	386
A00252	A01588	989	A00252	A01592	394
A00252	A01595	215	A00252	A01602	899
A00252	A01606	174	A00252	A01630	199
A00252	A01631	338	A00252	A01639	257
A00252	A01660	490	A00252	A01665	899
A00252	A01692	955	A00252	A01726	374
A00252	A01730	407	A00252	A01740	335
A00252	A01770	899	A00252	A01796	384
A00252	A01844	911	A00252	A02325	491
A00252	A02418	432	A00252	A02421	162
A00252	A02434	170	A00252	A02494	446
A00252	A02502	910	A00252	A02544	945
A00252	A02564	899	A00252	A02568	63
A00252	A02647	147	A00252	A02698	485
A00252	A02703	483	A00252	A02786	485
A00252	A02816	710	A00252	A02860	208
A00252	A02877	201	A00252	A02890	185
A00252	A02902	486	A00252	A02912	357
A00252	A02927	63	A00252	A02965	305
A00252	A03057	462	A00252	A03112	151
A00252	A03237	263	A00252	A03286	319
A00252	A03321	187	A00252	A03338	205
A00252	A03472	428	A00252	A03482	173
A00252	A03488	373	A00252	A03517	375
A00252	A03575	909	A00252	A03591	617
A00252	A03596	219	A00252	A03752	302
A00252	A03886	152	A00252	A04342	491
A00252	A04715	293	A00252	A04818	63
A00252	A05073	200	A00252	A05208	177
A00252	A05418	480	A00252	A05558	288
A00252	A05811	455	A00252	A05902	229
A00252	A06138	191	A00252	A06466	491

A00252	A06610	357	A00252	A06677	229
A00252	A07091	899	A00252	A07311	954
A00252	A07399	297	A00252	A07422	945
A00252	A07726	899	A00252	A07829	258
A00252	A08280	379	A00252	A08532	483
A00252	A08533	477	A00252	A08815	470
A00252	A08858	899	A00252	A08899	945
A00252	A09297	221	A00252	A09319	899
A00252	A09354	223	A00252	A09434	157
A00252	A09577	308	A00252	A09685	201
A00252	A09818	185	A00252	A09842	204
A00252	A09995	218	A00252	A10057	212
A00252	A10212	258	A00252	A10233	479
A00252	A10311	389	A00252	A10346	469
A00252	A10575	899	A00252	A10580	899
A00252	A10611	162	A00252	A11277	159
A00252	A11293	446	A00252	A11455	154
A00252	A11921	945	A00252	A11932	177
A00252	A11983	989	A00252	A12328	263
A00252	A12598	185	A00252	A13062	258
A00252	A13255	486	A00252	A13340	486
A00252	A13590	202	A00252	A13795	154
A00252	A13812	241	A00252	A13824	159
A00252	A13975	899	A00252	A13977	996
A00252	A13989	899	A00252	A14009	364
A00252	A14073	296	A00252	A14198	899
A00252	A14443	989	A00252	A14648	411
A00252	A15108	201	A00252	A15356	285
A00252	A15506	483	A00252	A16080	941
A00252	A16411	154	A00253	A00284	302
A00253	A00293	488	A00253	A00295	317
A00253	A00308	288	A00253	A00324	914
A00253	A00330	208	A00253	A00533	154
A00253	A00621	157	A00253	A00624	154
A00253	A00626	190	A00253	A00630	312
A00253	A00652	491	A00253	A00747	486
A00253	A00757	152	A00253	A00814	229
A00253	A00820	279	A00253	A00832	899
A00253	A00852	362	A00253	A00853	199
A00253	A00876	491	A00253	A00999	394
A00253	A01045	491	A00253	A01052	438
A00253	A01098	156	A00253	A01149	347
A00253	A01178	480	A00253	A01299	252
A00253	A01305	491	A00253	A01354	385
A00253	A01405	481	A00253	A01406	330
A00253	A01425	491	A00253	A01478	312

A00253	A01492	899	A00253	A01524	899
A00253	A01527	915	A00253	A01550	491
A00253	A01554	899	A00253	A01558	899
A00253	A01560	222	A00253	A01562	613
A00253	A01572	377	A00253	A01588	899
A00253	A01595	247	A00253	A01606	256
A00253	A01615	223	A00253	A01630	208
A00253	A01631	235	A00253	A01639	249
A00253	A01660	488	A00253	A01692	491
A00253	A01726	374	A00253	A01730	418
A00253	A01740	332	A00253	A01770	902
A00253	A01796	324	A00253	A01844	281
A00253	A01994	159	A00253	A02323	257
A00253	A02325	491	A00253	A02418	406
A00253	A02421	160	A00253	A02434	167
A00253	A02494	288	A00253	A02502	152
A00253	A02544	475	A00253	A02568	63
A00253	A02647	146	A00253	A02698	489
A00253	A02703	441	A00253	A02786	475
A00253	A02816	425	A00253	A02830	477
A00253	A02860	205	A00253	A02877	396
A00253	A02895	176	A00253	A02902	489
A00253	A02912	355	A00253	A02927	110
A00253	A02931	277	A00253	A02965	425
A00253	A03057	490	A00253	A03286	396
A00253	A03321	430	A00253	A03338	202
A00253	A03401	422	A00253	A03451	210
A00253	A03472	472	A00253	A03482	171
A00253	A03488	288	A00253	A03517	374
A00253	A03575	908	A00253	A03591	383
A00253	A03611	185	A00253	A03752	477
A00253	A04342	491	A00253	A04565	199
A00253	A04914	201	A00253	A05073	196
A00253	A05418	434	A00253	A05558	285
A00253	A05811	477	A00253	A05902	329
A00253	A06138	293	A00253	A06466	491
A00253	A06610	322	A00253	A06677	227
A00253	A06981	184	A00253	A07311	945
A00253	A07399	294	A00253	A07422	944
A00253	A07726	899	A00253	A07798	156
A00253	A08133	199	A00253	A08280	374
A00253	A08532	489	A00253	A08533	443
A00253	A08571	899	A00253	A08815	484
A00253	A08858	899	A00253	A08899	477
A00253	A09354	221	A00253	A09577	379
A00253	A09818	183	A00253	A09842	200

A00253	A09995	215	A00253	A10051	355
A00253	A10174	899	A00253	A10212	256
A00253	A10233	424	A00253	A10293	160
A00253	A10311	171	A00253	A10334	221
A00253	A10346	458	A00253	A10575	899
A00253	A10580	899	A00253	A10611	159
A00253	A10904	171	A00253	A11293	428
A00253	A11300	317	A00253	A11346	199
A00253	A11921	491	A00253	A11937	199
A00253	A11983	225	A00253	A12246	241
A00253	A12328	420	A00253	A12598	183
A00253	A13062	256	A00253	A13255	477
A00253	A13340	489	A00253	A13590	212
A00253	A13689	256	A00253	A13743	185
A00253	A13772	199	A00253	A13795	474
A00253	A13812	322	A00253	A13824	230
A00253	A13977	996	A00253	A13989	899
A00253	A14009	416	A00253	A14073	286
A00253	A14188	180	A00253	A14443	899
A00253	A14583	180	A00253	A14648	353
A00253	A15356	235	A00253	A16080	941
A00253	A16207	199	A00253	A16411	152
A00253	A17146	446	A00254	A00255	945
A00254	A00257	951	A00254	A00261	217
A00254	A00264	910	A00254	A00265	432
A00254	A00269	329	A00254	A00271	899
A00254	A00273	157	A00254	A00280	221
A00254	A00292	335	A00254	A00304	296
A00254	A00312	916	A00254	A00316	491
A00254	A00333	319	A00254	A00340	945
A00254	A00418	365	A00254	A00451	371
A00254	A00471	254	A00254	A00552	490
A00254	A00607	245	A00254	A00616	260
A00254	A00641	188	A00254	A00643	284
A00254	A00655	412	A00254	A00658	179
A00254	A00669	452	A00254	A00679	319
A00254	A00697	109	A00254	A00709	466
A00254	A00739	201	A00254	A00757	491
A00254	A00808	899	A00254	A00811	63
A00254	A00875	449	A00254	A00887	908
A00254	A00921	219	A00254	A00939	160
A00254	A00981	561	A00254	A00993	63
A00254	A00997	378	A00254	A01047	224
A00254	A01052	945	A00254	A01061	202
A00254	A01070	945	A00254	A01125	463
A00254	A01157	425	A00254	A01169	213

A00254	A01212	560	A00254	A01286	327
A00254	A01307	299	A00254	A01310	68
A00254	A01329	358	A00254	A01410	162
A00254	A01435	254	A00254	A01452	490
A00254	A01469	157	A00254	A01473	446
A00254	A01512	899	A00254	A01524	950
A00254	A01527	945	A00254	A01540	152
A00254	A01553	944	A00254	A01554	441
A00254	A01558	945	A00254	A01559	486
A00254	A01560	945	A00254	A01561	939
A00254	A01562	301	A00254	A01572	491
A00254	A01588	945	A00254	A01592	901
A00254	A01615	927	A00254	A01628	255
A00254	A01629	176	A00254	A01631	71
A00254	A01639	899	A00254	A01640	208
A00254	A01676	899	A00254	A01688	916
A00254	A01692	941	A00254	A01697	445
A00254	A01698	185	A00254	A01708	95
A00254	A01709	899	A00254	A01723	257
A00254	A01730	486	A00254	A01733	187
A00254	A01790	480	A00254	A01792	63
A00254	A01812	358	A00254	A01832	210
A00254	A01844	429	A00254	A01849	63
A00254	A01908	477	A00254	A01988	258
A00254	A01995	562	A00254	A02074	455
A00254	A02076	63	A00254	A02088	312
A00254	A02183	451	A00254	A02215	353
A00254	A02284	899	A00254	A02305	202
A00254	A02331	301	A00254	A02350	63
A00254	A02369	196	A00254	A02411	228
A00254	A02419	79	A00254	A02425	332
A00254	A02481	191	A00254	A02482	491
A00254	A02502	899	A00254	A02634	63
A00254	A02671	263	A00254	A02673	396
A00254	A02678	661	A00254	A02696	258
A00254	A02715	185	A00254	A02723	901
A00254	A02731	63	A00254	A02741	63
A00254	A02752	485	A00254	A02756	238
A00254	A02791	324	A00254	A02803	488
A00254	A02812	319	A00254	A02828	159
A00254	A02841	63	A00254	A02864	475
A00254	A02887	63	A00254	A03099	917
A00254	A03104	152	A00254	A03110	380
A00254	A03127	167	A00254	A03141	174
A00254	A03195	63	A00254	A03213	241
A00254	A03271	480	A00254	A03292	662

A00254	A03434	296	A00254	A03435	324
A00254	A03436	491	A00254	A03451	224
A00254	A03465	130	A00254	A03581	260
A00254	A03591	467	A00254	A03611	221
A00254	A03694	390	A00254	A03749	232
A00254	A03771	440	A00254	A03784	486
A00254	A03809	695	A00254	A03817	457
A00254	A03821	467	A00254	A03830	457
A00254	A03961	219	A00254	A03990	488
A00254	A04012	910	A00254	A04020	224
A00254	A04033	401	A00254	A04037	223
A00254	A04051	312	A00254	A04162	232
A00254	A04174	321	A00254	A04288	99
A00254	A04293	79	A00254	A04297	229
A00254	A04314	63	A00254	A04323	266
A00254	A04335	193	A00254	A04357	172
A00254	A04398	291	A00254	A04481	223
A00254	A04484	899	A00254	A04490	312
A00254	A04661	171	A00254	A04663	180
A00254	A04699	899	A00254	A04731	346
A00254	A04914	157	A00254	A05025	171
A00254	A05208	900	A00254	A05216	180
A00254	A05221	477	A00254	A05288	473
A00254	A05289	334	A00254	A05339	110
A00254	A05363	112	A00254	A05418	364
A00254	A05453	308	A00254	A05530	63
A00254	A05560	167	A00254	A05633	491
A00254	A05638	358	A00254	A05847	63
A00254	A05848	254	A00254	A06029	395
A00254	A06045	230	A00254	A06077	236
A00254	A06138	154	A00254	A06190	491
A00254	A06256	135	A00254	A06295	943
A00254	A06397	236	A00254	A06398	160
A00254	A06409	157	A00254	A06581	156
A00254	A06609	899	A00254	A06610	429
A00254	A06703	102	A00254	A06762	899
A00254	A06783	352	A00254	A06915	163
A00254	A07004	188	A00254	A07148	329
A00254	A07152	157	A00254	A07200	212
A00254	A07201	899	A00254	A07311	976
A00254	A07339	490	A00254	A07422	899
A00254	A07460	899	A00254	A07483	168
A00254	A07528	306	A00254	A07619	245
A00254	A07726	486	A00254	A07762	486
A00254	A07803	243	A00254	A07835	353
A00254	A08280	419	A00254	A08571	486

A00254	A08608	430	A00254	A08860	451
A00254	A08935	157	A00254	A09007	207
A00254	A09078	490	A00254	A09201	899
A00254	A09218	407	A00254	A09220	479
A00254	A09235	489	A00254	A09356	196
A00254	A09363	294	A00254	A09398	193
A00254	A09401	63	A00254	A09542	907
A00254	A09543	135	A00254	A09568	937
A00254	A09643	63	A00254	A09660	901
A00254	A09897	193	A00254	A09937	358
A00254	A09993	196	A00254	A10072	348
A00254	A10623	899	A00254	A10634	205
A00254	A10666	180	A00254	A10735	938
A00254	A10801	944	A00254	A10833	905
A00254	A10872	230	A00254	A10986	265
A00254	A11259	172	A00254	A11369	491
A00254	A11411	154	A00254	A11521	484
A00254	A11983	404	A00254	A11986	176
A00254	A12288	193	A00254	A12383	939
A00254	A12871	260	A00254	A12886	146
A00254	A12932	916	A00254	A13003	899
A00254	A13168	193	A00254	A13207	176
A00254	A13362	205	A00254	A13390	899
A00254	A13510	185	A00254	A13511	486
A00254	A13605	281	A00254	A13663	157
A00254	A13824	157	A00254	A13903	472
A00254	A13958	899	A00254	A13975	483
A00254	A13980	369	A00254	A13984	899
A00254	A13986	276	A00254	A13987	899
A00254	A13997	294	A00254	A14006	306
A00254	A14010	905	A00254	A14019	324
A00254	A14027	263	A00254	A14034	99
A00254	A14035	127	A00254	A14042	899
A00254	A14045	899	A00254	A14047	157
A00254	A14065	899	A00254	A14103	157
A00254	A14187	481	A00254	A14188	491
A00254	A14194	903	A00254	A14198	976
A00254	A14208	249	A00254	A14299	357
A00254	A14364	441	A00254	A14400	393
A00254	A14402	446	A00254	A14426	353
A00254	A14433	945	A00254	A14438	424
A00254	A14443	943	A00254	A14444	238
A00254	A14553	193	A00254	A14577	485
A00254	A14635	408	A00254	A14644	446
A00254	A14653	63	A00254	A15182	905
A00254	A15186	193	A00254	A15195	157

A00254	A15292	899	A00254	A15293	958
A00254	A15294	963	A00254	A15312	899
A00254	A15320	469	A00254	A15325	235
A00254	A15503	232	A00254	A15506	541
A00254	A15726	489	A00254	A15802	156
A00254	A16224	302	A00254	A16901	348
A00254	A16994	63	A00254	A17188	288
A00255	A00261	227	A00255	A00694	160
A00255	A00697	427	A00255	A00808	910
A00255	A00811	306	A00255	A00981	243
A00255	A01052	899	A00255	A01178	151
A00255	A01407	168	A00255	A01435	319
A00255	A01507	63	A00255	A01524	945
A00255	A01527	945	A00255	A01553	899
A00255	A01558	899	A00255	A01560	165
A00255	A01615	900	A00255	A01688	900
A00255	A01785	193	A00255	A01908	84
A00255	A01975	246	A00255	A02000	414
A00255	A02350	334	A00255	A02447	263
A00255	A02479	200	A00255	A02502	899
A00255	A02797	168	A00255	A02803	79
A00255	A02863	162	A00255	A02864	314
A00255	A02877	180	A00255	A02878	177
A00255	A02917	167	A00255	A03054	322
A00255	A03071	485	A00255	A03271	73
A00255	A03753	187	A00255	A03790	341
A00255	A03809	968	A00255	A03906	162
A00255	A03971	277	A00255	A04078	200
A00255	A04473	373	A00255	A04484	924
A00255	A04490	324	A00255	A04622	63
A00255	A05082	375	A00255	A05155	465
A00255	A06107	296	A00255	A06795	346
A00255	A07163	308	A00255	A07311	944
A00255	A09220	262	A00255	A09401	157
A00255	A09543	321	A00255	A09959	335
A00255	A10005	191	A00255	A11578	207
A00255	A13181	152	A00255	A13342	207
A00255	A13369	160	A00255	A14035	314
A00255	A14198	899	A00255	A15317	243
A00255	A15606	204	A00255	A17188	929
A00256	A00264	462	A00256	A00382	472
A00256	A00396	224	A00256	A00472	455
A00256	A00503	257	A00256	A00682	291
A00256	A00687	406	A00256	A01142	180
A00256	A01558	899	A00256	A01571	63
A00256	A01669	241	A00256	A01728	184

A00256	A01746	462	A00256	A01763	193
A00256	A01840	273	A00256	A02564	385
A00256	A02698	257	A00256	A02873	223
A00256	A02968	899	A00256	A03095	363
A00256	A03266	157	A00256	A03662	462
A00256	A03766	443	A00256	A03841	154
A00256	A03846	254	A00256	A04287	269
A00256	A04536	428	A00256	A05016	247
A00256	A05216	480	A00256	A05399	329
A00256	A05513	165	A00256	A05742	388
A00256	A05760	899	A00256	A05797	74
A00256	A05989	185	A00256	A06595	213
A00256	A06607	489	A00256	A06608	467
A00256	A06610	63	A00256	A06622	281
A00256	A06628	491	A00256	A06650	491
A00256	A06654	157	A00256	A06712	196
A00256	A06731	276	A00256	A06760	307
A00256	A07178	157	A00256	A07189	159
A00256	A08010	152	A00256	A08128	269
A00256	A08183	468	A00256	A08615	185
A00256	A08736	247	A00256	A08738	339
A00256	A08941	299	A00256	A08944	152
A00256	A09243	159	A00256	A09385	165
A00256	A11973	174	A00256	A12373	380
A00256	A13565	185	A00256	A14430	348
A00256	A14443	899	A00256	A15624	399
A00256	A15803	165	A00256	A15889	267
A00256	A16426	151	A00256	A16636	241
A00257	A00261	154	A00257	A00265	420
A00257	A00316	246	A00257	A00329	227
A00257	A00348	316	A00257	A00352	151
A00257	A00356	559	A00257	A00419	408
A00257	A00454	475	A00257	A00517	429
A00257	A00531	327	A00257	A00532	162
A00257	A00560	458	A00257	A00630	462
A00257	A00785	180	A00257	A00805	441
A00257	A00808	191	A00257	A00910	283
A00257	A00932	373	A00257	A01042	491
A00257	A01048	205	A00257	A01057	196
A00257	A01077	490	A00257	A01083	489
A00257	A01098	257	A00257	A01150	168
A00257	A01161	159	A00257	A01178	491
A00257	A01254	238	A00257	A01291	496
A00257	A01406	255	A00257	A01431	278
A00257	A01435	595	A00257	A01473	579
A00257	A01512	518	A00257	A01527	899

A00257	A01540	451	A00257	A01551	340
A00257	A01553	945	A00257	A01554	480
A00257	A01558	899	A00257	A01560	491
A00257	A01561	513	A00257	A01562	277
A00257	A01576	491	A00257	A01588	899
A00257	A01606	593	A00257	A01615	297
A00257	A01628	353	A00257	A01631	450
A00257	A01639	481	A00257	A01692	491
A00257	A01697	246	A00257	A01707	485
A00257	A01719	321	A00257	A01730	351
A00257	A01781	63	A00257	A01920	258
A00257	A01950	436	A00257	A02094	416
A00257	A02361	319	A00257	A02586	291
A00257	A02715	491	A00257	A02724	341
A00257	A02813	152	A00257	A03014	195
A00257	A03031	205	A00257	A03236	241
A00257	A03451	294	A00257	A03536	538
A00257	A03591	491	A00257	A03611	154
A00257	A03625	491	A00257	A03686	586
A00257	A03739	483	A00257	A03740	491
A00257	A03752	491	A00257	A03809	268
A00257	A03893	443	A00257	A04012	477
A00257	A04033	489	A00257	A04072	457
A00257	A04153	183	A00257	A04194	252
A00257	A04342	353	A00257	A04449	486
A00257	A04622	215	A00257	A05303	205
A00257	A05979	238	A00257	A06317	170
A00257	A06466	490	A00257	A06588	491
A00257	A06688	491	A00257	A06907	299
A00257	A07004	477	A00257	A07017	446
A00257	A07151	236	A00257	A07155	469
A00257	A07207	491	A00257	A07311	952
A00257	A07339	171	A00257	A07398	335
A00257	A07798	468	A00257	A07920	346
A00257	A08054	188	A00257	A08130	183
A00257	A08315	185	A00257	A08409	469
A00257	A08423	183	A00257	A08571	224
A00257	A08635	255	A00257	A08860	491
A00257	A08878	433	A00257	A09009	283
A00257	A09068	301	A00257	A09078	182
A00257	A09162	409	A00257	A09218	440
A00257	A09253	491	A00257	A09294	288
A00257	A09303	439	A00257	A09417	306
A00257	A09476	235	A00257	A09502	154
A00257	A09577	445	A00257	A09592	460
A00257	A09750	283	A00257	A09804	490

A00257	A09842	63	A00257	A09874	485
A00257	A09952	63	A00257	A10014	160
A00257	A10365	486	A00257	A10379	241
A00257	A10623	357	A00257	A10728	486
A00257	A10735	226	A00257	A10760	391
A00257	A10808	330	A00257	A10982	430
A00257	A11302	286	A00257	A11372	255
A00257	A11470	218	A00257	A11500	205
A00257	A11532	205	A00257	A11602	461
A00257	A11724	247	A00257	A11937	239
A00257	A11983	486	A00257	A12050	241
A00257	A12383	423	A00257	A12990	165
A00257	A13219	258	A00257	A13340	407
A00257	A13403	396	A00257	A13511	485
A00257	A13551	183	A00257	A13729	475
A00257	A13743	165	A00257	A13824	562
A00257	A13829	899	A00257	A13989	449
A00257	A14006	488	A00257	A14026	486
A00257	A14073	491	A00257	A14298	167
A00257	A14334	241	A00257	A14635	347
A00257	A14648	452	A00257	A14801	168
A00257	A15295	323	A00257	A15366	213
A00257	A15498	480	A00257	A15506	314
A00257	A15620	173	A00257	A15699	173
A00257	A15921	241	A00257	A16060	307
A00257	A16835	243	A00257	A16847	491
A00257	A17188	167	A00258	A00283	199
A00258	A00334	372	A00258	A00356	748
A00258	A00366	364	A00258	A00440	328
A00258	A00571	199	A00258	A00629	199
A00258	A00634	491	A00258	A00700	930
A00258	A01098	199	A00258	A01112	334
A00258	A01291	986	A00258	A01299	152
A00258	A01306	199	A00258	A01405	235
A00258	A01415	281	A00258	A01425	199
A00258	A01524	908	A00258	A01527	899
A00258	A01558	899	A00258	A01579	160
A00258	A01580	484	A00258	A01588	899
A00258	A01593	899	A00258	A01596	497
A00258	A01601	984	A00258	A01605	932
A00258	A01640	942	A00258	A01718	915
A00258	A02179	195	A00258	A02213	182
A00258	A03567	199	A00258	A03575	173
A00258	A03591	945	A00258	A03939	538
A00258	A04790	170	A00258	A05288	574
A00258	A06814	281	A00258	A06861	199

A00258	A07398	335	A00258	A07869	154
A00258	A08360	396	A00258	A08839	899
A00258	A09322	199	A00258	A09619	218
A00258	A10580	447	A00258	A10666	216
A00258	A12047	577	A00258	A12365	232
A00258	A12373	900	A00258	A13063	154
A00258	A13397	247	A00258	A13405	279
A00258	A13831	152	A00258	A13989	592
A00258	A13992	899	A00258	A13994	213
A00258	A14128	497	A00258	A14182	396
A00258	A14190	396	A00258	A14375	191
A00258	A14443	899	A00258	A14444	899
A00258	A16418	603	A00258	A17013	296
A00259	A00260	941	A00259	A00357	162
A00259	A00522	288	A00259	A00639	232
A00259	A01098	247	A00259	A01425	419
A00259	A01756	232	A00259	A01875	213
A00259	A02292	423	A00259	A02599	215
A00259	A02837	360	A00259	A02871	347
A00259	A02909	193	A00259	A03101	168
A00259	A03102	183	A00259	A03541	384
A00259	A03590	362	A00259	A03612	268
A00259	A03825	188	A00259	A03826	218
A00259	A03912	226	A00259	A03922	172
A00259	A04089	226	A00259	A04583	395
A00259	A05038	240	A00259	A05073	335
A00259	A05078	232	A00259	A05417	152
A00259	A06404	215	A00259	A06597	337
A00259	A06884	159	A00259	A07147	193
A00259	A07187	268	A00259	A09261	347
A00259	A09780	208	A00259	A10337	330
A00259	A10364	254	A00259	A10792	278
A00259	A11116	395	A00259	A12373	249
A00259	A13250	196	A00259	A14261	307
A00259	A14371	172	A00259	A15220	307
A00259	A16786	163	A00260	A00522	361
A00260	A01452	162	A00260	A01669	285
A00260	A02080	343	A00260	A02460	246
A00260	A03095	465	A00260	A03157	207
A00260	A03419	348	A00260	A04017	383
A00260	A04739	288	A00260	A04996	334
A00260	A07187	452	A00260	A09263	263
A00260	A11116	454	A00260	A15220	408
A00260	A16786	334	A00261	A00316	406
A00261	A00696	168	A00261	A00922	185
A00261	A00981	328	A00261	A01052	899

A00261	A01558	908	A00261	A01573	193
A00261	A01588	899	A00261	A01596	616
A00261	A01602	899	A00261	A01615	899
A00261	A01623	272	A00261	A01636	154
A00261	A01697	188	A00261	A01914	762
A00261	A01920	616	A00261	A01974	488
A00261	A02711	262	A00261	A02797	441
A00261	A03068	412	A00261	A03279	157
A00261	A03422	489	A00261	A04012	267
A00261	A04137	184	A00261	A04280	463
A00261	A04398	475	A00261	A04495	218
A00261	A04857	243	A00261	A05221	461
A00261	A05831	243	A00261	A06597	488
A00261	A07311	347	A00261	A07390	390
A00261	A07565	278	A00261	A07683	157
A00261	A08118	291	A00261	A08124	184
A00261	A08725	184	A00261	A08860	215
A00261	A09059	491	A00261	A09937	224
A00261	A10183	243	A00261	A10666	388
A00261	A11019	424	A00261	A11118	184
A00261	A11124	369	A00261	A12886	162
A00261	A13032	205	A00261	A13250	360
A00261	A14055	168	A00261	A14073	202
A00261	A14204	491	A00261	A14299	665
A00261	A14443	180	A00261	A14444	226
A00261	A14704	615	A00261	A14705	460
A00261	A14777	488	A00261	A14848	278
A00261	A14913	288	A00261	A15321	366
A00261	A15322	490	A00261	A15347	486
A00261	A15506	390	A00261	A15647	369
A00261	A15726	285	A00261	A15932	271
A00262	A00294	226	A00262	A00324	491
A00262	A00336	240	A00262	A00679	149
A00262	A01052	163	A00262	A01178	474
A00262	A01363	191	A00262	A01364	323
A00262	A01378	414	A00262	A01507	450
A00262	A01512	473	A00262	A01602	185
A00262	A01606	486	A00262	A01691	212
A00262	A01698	480	A00262	A01745	218
A00262	A01756	196	A00262	A01759	185
A00262	A01796	173	A00262	A01850	486
A00262	A01896	455	A00262	A01908	63
A00262	A02318	160	A00262	A02343	477
A00262	A02388	174	A00262	A02586	358
A00262	A02629	347	A00262	A02636	196
A00262	A02667	910	A00262	A02670	159

A00262	A02699	238	A00262	A02749	188
A00262	A02789	293	A00262	A02819	319
A00262	A02822	288	A00262	A03073	204
A00262	A03263	168	A00262	A03309	218
A00262	A03336	313	A00262	A03451	188
A00262	A03465	87	A00262	A03611	363
A00262	A03842	229	A00262	A03845	154
A00262	A03871	265	A00262	A03893	307
A00262	A04015	196	A00262	A04380	208
A00262	A04449	470	A00262	A04522	352
A00262	A04622	63	A00262	A05288	303
A00262	A05295	159	A00262	A05633	223
A00262	A05634	432	A00262	A05779	162
A00262	A05790	165	A00262	A06056	245
A00262	A06333	396	A00262	A06426	254
A00262	A06596	154	A00262	A06623	463
A00262	A06648	154	A00262	A06703	339
A00262	A06717	236	A00262	A06859	193
A00262	A07167	202	A00262	A07393	324
A00262	A07466	234	A00262	A08030	205
A00262	A08274	185	A00262	A08590	236
A00262	A08692	491	A00262	A09085	177
A00262	A09162	608	A00262	A09279	475
A00262	A09644	899	A00262	A09753	249
A00262	A09791	156	A00262	A10221	218
A00262	A10514	183	A00262	A10669	185
A00262	A10808	356	A00262	A11487	177
A00262	A11973	245	A00262	A11983	168
A00262	A13502	245	A00262	A13673	185
A00262	A13997	278	A00262	A14513	324
A00262	A14604	151	A00262	A14643	419
A00262	A14860	385	A00262	A14925	154
A00262	A16590	213	A00262	A16835	347
A00263	A00269	992	A00263	A00294	183
A00263	A00337	571	A00263	A00340	308
A00263	A00347	208	A00263	A00388	193
A00263	A00397	180	A00263	A00472	201
A00263	A00482	385	A00263	A00483	422
A00263	A00519	249	A00263	A00625	205
A00263	A00630	224	A00263	A00637	213
A00263	A00652	308	A00263	A00688	226
A00263	A00719	176	A00263	A00779	232
A00263	A00786	849	A00263	A00813	228
A00263	A00814	269	A00263	A00826	249
A00263	A00829	238	A00263	A00853	343
A00263	A00864	461	A00263	A00874	172

A00263	A00929	156	A00263	A00941	523
A00263	A00980	216	A00263	A01004	483
A00263	A01011	304	A00263	A01064	467
A00263	A01125	199	A00263	A01169	180
A00263	A01268	195	A00263	A01309	469
A00263	A01348	345	A00263	A01386	241
A00263	A01415	475	A00263	A01417	375
A00263	A01454	374	A00263	A01478	959
A00263	A01479	489	A00263	A01486	491
A00263	A01524	899	A00263	A01532	236
A00263	A01537	389	A00263	A01558	899
A00263	A01559	463	A00263	A01588	899
A00263	A01591	975	A00263	A01592	469
A00263	A01595	491	A00263	A01596	486
A00263	A01615	266	A00263	A01630	274
A00263	A01636	260	A00263	A01650	945
A00263	A01661	491	A00263	A01667	347
A00263	A01668	249	A00263	A01705	561
A00263	A01708	188	A00263	A01775	172
A00263	A01839	473	A00263	A01970	157
A00263	A02000	185	A00263	A02110	216
A00263	A02278	263	A00263	A02314	162
A00263	A02416	470	A00263	A02479	215
A00263	A02836	235	A00263	A02877	490
A00263	A03098	249	A00263	A03213	207
A00263	A03401	285	A00263	A03415	207
A00263	A03422	639	A00263	A03484	63
A00263	A03492	344	A00263	A03575	951
A00263	A03637	490	A00263	A03641	172
A00263	A03813	475	A00263	A03817	160
A00263	A03820	302	A00263	A03899	279
A00263	A04086	249	A00263	A04117	283
A00263	A04175	468	A00263	A04440	472
A00263	A04449	215	A00263	A04557	74
A00263	A04668	168	A00263	A04715	286
A00263	A04790	441	A00263	A04863	162
A00263	A04873	215	A00263	A04971	352
A00263	A05008	491	A00263	A05009	961
A00263	A05015	491	A00263	A05087	172
A00263	A05141	193	A00263	A05155	190
A00263	A05383	486	A00263	A05428	927
A00263	A05431	308	A00263	A05443	308
A00263	A05621	170	A00263	A05625	185
A00263	A05836	257	A00263	A05853	608
A00263	A05880	468	A00263	A05894	296
A00263	A05944	469	A00263	A06010	165

A00263	A06039	199	A00263	A06182	398
A00263	A06379	486	A00263	A06428	330
A00263	A06609	441	A00263	A06615	452
A00263	A06651	217	A00263	A06783	357
A00263	A07218	273	A00263	A07283	195
A00263	A07433	524	A00263	A07480	249
A00263	A07524	180	A00263	A07603	308
A00263	A07706	259	A00263	A07848	331
A00263	A08022	216	A00263	A08133	199
A00263	A08184	236	A00263	A08185	255
A00263	A08289	177	A00263	A08571	949
A00263	A08732	899	A00263	A08746	382
A00263	A08836	475	A00263	A08839	975
A00263	A08853	422	A00263	A08858	555
A00263	A08885	256	A00263	A09182	488
A00263	A09187	200	A00263	A09195	494
A00263	A09256	201	A00263	A09353	162
A00263	A09370	425	A00263	A09380	188
A00263	A09443	491	A00263	A09449	63
A00263	A09491	433	A00263	A09506	474
A00263	A09511	190	A00263	A09546	226
A00263	A09747	252	A00263	A10083	160
A00263	A10175	608	A00263	A10203	221
A00263	A10380	216	A00263	A10395	687
A00263	A10430	308	A00263	A10580	452
A00263	A10619	485	A00263	A10657	308
A00263	A11006	200	A00263	A11104	483
A00263	A11107	200	A00263	A11383	183
A00263	A11481	200	A00263	A11487	180
A00263	A11548	236	A00263	A11560	394
A00263	A11661	200	A00263	A11835	154
A00263	A11897	293	A00263	A12123	221
A00263	A12137	154	A00263	A12365	216
A00263	A12474	433	A00263	A12513	200
A00263	A12638	154	A00263	A12671	257
A00263	A12722	424	A00263	A12897	441
A00263	A13369	203	A00263	A13378	257
A00263	A13924	491	A00263	A13978	246
A00263	A14192	172	A00263	A14295	193
A00263	A14395	195	A00263	A14443	990
A00263	A14444	899	A00263	A14485	147
A00263	A14509	241	A00263	A14531	425
A00263	A14583	180	A00263	A14585	63
A00263	A14633	529	A00263	A14886	485
A00263	A15726	488	A00263	A15889	247
A00263	A15896	236	A00263	A15926	281

A00263	A16077	306	A00263	A16135	219
A00263	A16418	160	A00263	A16802	216
A00263	A16847	308	A00263	A17148	252
A00263	A17200	257	A00264	A00292	473
A00264	A00294	378	A00264	A00305	305
A00264	A00309	452	A00264	A00370	227
A00264	A00449	265	A00264	A00472	386
A00264	A00556	383	A00264	A00576	216
A00264	A00577	260	A00264	A00616	945
A00264	A00656	469	A00264	A00657	227
A00264	A00659	288	A00264	A00688	420
A00264	A00736	491	A00264	A00747	260
A00264	A00914	491	A00264	A00993	488
A00264	A01125	346	A00264	A01137	228
A00264	A01138	491	A00264	A01161	182
A00264	A01172	269	A00264	A01223	180
A00264	A01230	347	A00264	A01325	232
A00264	A01400	474	A00264	A01527	899
A00264	A01551	491	A00264	A01558	899
A00264	A01588	899	A00264	A01590	374
A00264	A01602	252	A00264	A01604	401
A00264	A01626	460	A00264	A01629	252
A00264	A01654	232	A00264	A01666	176
A00264	A01668	491	A00264	A01676	491
A00264	A01704	228	A00264	A01705	491
A00264	A01726	491	A00264	A01727	408
A00264	A01728	452	A00264	A01729	257
A00264	A01733	490	A00264	A01737	466
A00264	A01741	491	A00264	A01743	491
A00264	A01746	491	A00264	A01751	455
A00264	A01752	438	A00264	A01754	201
A00264	A01757	348	A00264	A01758	479
A00264	A01763	165	A00264	A01767	472
A00264	A01774	379	A00264	A01775	491
A00264	A01778	208	A00264	A01785	371
A00264	A01794	286	A00264	A01795	165
A00264	A01797	391	A00264	A01802	268
A00264	A01867	386	A00264	A01875	480
A00264	A01876	488	A00264	A01879	457
A00264	A01883	316	A00264	A01888	491
A00264	A01889	296	A00264	A01893	462
A00264	A01894	491	A00264	A01900	210
A00264	A01909	451	A00264	A01941	429
A00264	A01944	212	A00264	A01982	477
A00264	A02006	223	A00264	A02026	151
A00264	A02028	208	A00264	A02048	490

A00264	A02049	441	A00264	A02072	87
A00264	A02080	436	A00264	A02082	74
A00264	A02108	293	A00264	A02143	335
A00264	A02146	475	A00264	A02148	414
A00264	A02173	413	A00264	A02177	424
A00264	A02180	406	A00264	A02186	486
A00264	A02202	228	A00264	A02215	491
A00264	A02224	215	A00264	A02238	457
A00264	A02260	303	A00264	A02291	488
A00264	A02298	491	A00264	A02299	427
A00264	A02300	491	A00264	A02302	265
A00264	A02303	63	A00264	A02306	443
A00264	A02307	491	A00264	A02309	457
A00264	A02312	63	A00264	A02313	301
A00264	A02314	358	A00264	A02320	484
A00264	A02326	139	A00264	A02328	314
A00264	A02329	324	A00264	A02331	458
A00264	A02333	451	A00264	A02338	188
A00264	A02340	391	A00264	A02347	394
A00264	A02349	398	A00264	A02371	310
A00264	A02376	198	A00264	A02377	489
A00264	A02385	461	A00264	A02386	367
A00264	A02387	439	A00264	A02388	277
A00264	A02393	173	A00264	A02402	121
A00264	A02412	491	A00264	A02413	447
A00264	A02415	408	A00264	A02416	486
A00264	A02417	422	A00264	A02423	490
A00264	A02427	254	A00264	A02428	162
A00264	A02450	241	A00264	A02460	456
A00264	A02465	490	A00264	A02468	469
A00264	A02473	157	A00264	A02477	179
A00264	A02488	265	A00264	A02490	491
A00264	A02491	491	A00264	A02492	468
A00264	A02493	484	A00264	A02494	105
A00264	A02495	491	A00264	A02498	63
A00264	A02505	371	A00264	A02506	360
A00264	A02514	173	A00264	A02535	452
A00264	A02539	154	A00264	A02542	484
A00264	A02545	263	A00264	A02559	419
A00264	A02568	238	A00264	A02575	319
A00264	A02579	185	A00264	A02582	491
A00264	A02585	402	A00264	A02587	296
A00264	A02605	489	A00264	A02611	310
A00264	A02612	276	A00264	A02613	173
A00264	A02614	127	A00264	A02615	229
A00264	A02620	414	A00264	A02621	210

A00264	A02622	157	A00264	A02638	154
A00264	A02639	329	A00264	A02644	461
A00264	A02657	218	A00264	A02664	107
A00264	A02668	491	A00264	A02669	469
A00264	A02670	152	A00264	A02672	229
A00264	A02687	193	A00264	A02691	208
A00264	A02693	229	A00264	A02695	489
A00264	A02696	472	A00264	A02697	491
A00264	A02698	420	A00264	A02706	457
A00264	A02729	469	A00264	A02730	423
A00264	A02735	246	A00264	A02739	324
A00264	A02741	490	A00264	A02745	480
A00264	A02746	386	A00264	A02749	243
A00264	A02753	258	A00264	A02754	243
A00264	A02756	491	A00264	A02760	63
A00264	A02763	167	A00264	A02771	491
A00264	A02775	173	A00264	A02780	157
A00264	A02783	490	A00264	A02784	327
A00264	A02785	491	A00264	A02787	460
A00264	A02788	484	A00264	A02789	434
A00264	A02801	232	A00264	A02808	358
A00264	A02812	491	A00264	A02813	291
A00264	A02814	165	A00264	A02831	361
A00264	A02836	457	A00264	A02837	491
A00264	A02841	477	A00264	A02842	340
A00264	A02843	291	A00264	A02854	198
A00264	A02859	491	A00264	A02869	347
A00264	A02872	156	A00264	A02874	491
A00264	A02875	433	A00264	A02888	268
A00264	A02890	157	A00264	A02895	212
A00264	A02896	468	A00264	A02901	171
A00264	A02917	191	A00264	A02918	273
A00264	A02922	485	A00264	A02927	473
A00264	A02944	409	A00264	A02951	490
A00264	A02953	172	A00264	A02961	489
A00264	A02963	429	A00264	A02969	176
A00264	A02982	491	A00264	A02983	286
A00264	A02984	484	A00264	A03007	162
A00264	A03034	263	A00264	A03050	157
A00264	A03053	452	A00264	A03056	468
A00264	A03069	394	A00264	A03080	484
A00264	A03107	480	A00264	A03112	445
A00264	A03126	252	A00264	A03144	208
A00264	A03157	291	A00264	A03176	195
A00264	A03184	154	A00264	A03186	378
A00264	A03188	355	A00264	A03189	358

A00264	A03195	154	A00264	A03210	157
A00264	A03223	191	A00264	A03224	428
A00264	A03228	229	A00264	A03243	154
A00264	A03252	157	A00264	A03259	491
A00264	A03268	196	A00264	A03269	267
A00264	A03271	293	A00264	A03275	491
A00264	A03281	435	A00264	A03282	177
A00264	A03288	174	A00264	A03304	396
A00264	A03353	218	A00264	A03361	469
A00264	A03370	436	A00264	A03373	423
A00264	A03378	246	A00264	A03379	329
A00264	A03403	174	A00264	A03414	199
A00264	A03417	260	A00264	A03419	235
A00264	A03432	162	A00264	A03516	213
A00264	A03520	402	A00264	A03522	224
A00264	A03534	157	A00264	A03540	462
A00264	A03634	344	A00264	A03639	945
A00264	A03653	480	A00264	A03661	369
A00264	A03744	350	A00264	A03761	277
A00264	A03773	409	A00264	A03803	473
A00264	A03834	185	A00264	A03904	348
A00264	A03914	491	A00264	A03915	238
A00264	A03923	210	A00264	A03970	195
A00264	A03975	347	A00264	A03976	486
A00264	A03988	475	A00264	A03991	404
A00264	A04010	491	A00264	A04015	205
A00264	A04023	481	A00264	A04031	180
A00264	A04041	173	A00264	A04045	157
A00264	A04048	468	A00264	A04051	491
A00264	A04087	154	A00264	A04092	402
A00264	A04117	208	A00264	A04201	277
A00264	A04212	491	A00264	A04214	157
A00264	A04218	162	A00264	A04222	259
A00264	A04225	238	A00264	A04237	121
A00264	A04258	188	A00264	A04260	369
A00264	A04262	255	A00264	A04263	455
A00264	A04267	480	A00264	A04268	173
A00264	A04271	160	A00264	A04274	330
A00264	A04278	205	A00264	A04286	265
A00264	A04298	157	A00264	A04300	232
A00264	A04327	330	A00264	A04330	285
A00264	A04333	428	A00264	A04334	452
A00264	A04335	195	A00264	A04349	424
A00264	A04391	474	A00264	A04398	466
A00264	A04407	191	A00264	A04428	380
A00264	A04462	358	A00264	A04481	481

A00264	A04484	224	A00264	A04509	472
A00264	A04510	236	A00264	A04517	135
A00264	A04533	254	A00264	A04536	174
A00264	A04548	373	A00264	A04585	232
A00264	A04589	266	A00264	A04593	195
A00264	A04594	173	A00264	A04604	173
A00264	A04607	235	A00264	A04608	440
A00264	A04609	234	A00264	A04618	157
A00264	A04652	473	A00264	A04698	163
A00264	A04710	228	A00264	A04783	407
A00264	A04794	173	A00264	A04801	173
A00264	A04830	221	A00264	A04832	63
A00264	A04842	424	A00264	A04866	212
A00264	A04867	480	A00264	A04870	115
A00264	A04875	212	A00264	A04908	322
A00264	A04916	157	A00264	A04939	208
A00264	A04949	221	A00264	A04951	173
A00264	A04965	185	A00264	A04975	201
A00264	A04976	305	A00264	A04981	165
A00264	A04989	110	A00264	A05079	319
A00264	A05113	473	A00264	A05114	271
A00264	A05117	367	A00264	A05122	322
A00264	A05153	346	A00264	A05176	279
A00264	A05215	157	A00264	A05279	173
A00264	A05303	195	A00264	A05309	491
A00264	A05349	491	A00264	A05353	162
A00264	A05497	157	A00264	A05505	195
A00264	A05521	232	A00264	A05535	255
A00264	A05571	195	A00264	A05686	232
A00264	A05797	408	A00264	A05828	171
A00264	A05868	434	A00264	A05934	255
A00264	A05949	190	A00264	A06146	157
A00264	A06174	195	A00264	A06286	260
A00264	A06314	408	A00264	A06415	480
A00264	A06439	205	A00264	A06558	157
A00264	A06597	463	A00264	A06606	454
A00264	A06607	449	A00264	A06650	488
A00264	A06834	221	A00264	A06949	451
A00264	A07025	157	A00264	A07135	329
A00264	A07141	490	A00264	A07145	299
A00264	A07153	162	A00264	A07154	215
A00264	A07157	481	A00264	A07164	491
A00264	A07165	490	A00264	A07166	215
A00264	A07177	414	A00264	A07183	188
A00264	A07187	157	A00264	A07191	347
A00264	A07198	404	A00264	A07200	491

A00264	A07201	218	A00264	A07209	490
A00264	A07218	480	A00264	A07339	238
A00264	A07522	157	A00264	A07525	173
A00264	A07639	195	A00264	A07935	201
A00264	A08572	358	A00264	A08591	344
A00264	A08596	193	A00264	A08627	446
A00264	A08631	195	A00264	A08738	480
A00264	A08875	249	A00264	A08910	232
A00264	A09095	306	A00264	A09266	173
A00264	A09390	246	A00264	A09397	288
A00264	A09399	127	A00264	A09467	157
A00264	A09468	173	A00264	A09480	291
A00264	A09481	195	A00264	A09642	173
A00264	A09789	246	A00264	A10085	307
A00264	A10216	212	A00264	A10244	195
A00264	A10337	428	A00264	A10357	180
A00264	A10388	232	A00264	A10625	400
A00264	A10650	157	A00264	A10835	232
A00264	A11050	232	A00264	A11064	479
A00264	A11123	456	A00264	A11313	229
A00264	A11897	160	A00264	A11911	489
A00264	A12373	221	A00264	A12553	157
A00264	A12714	195	A00264	A12897	480
A00264	A12927	486	A00264	A13212	274
A00264	A13225	899	A00264	A13419	157
A00264	A13579	173	A00264	A13667	329
A00264	A13734	462	A00264	A13886	252
A00264	A13909	195	A00264	A14071	380
A00264	A14194	945	A00264	A14198	945
A00264	A14236	355	A00264	A14405	484
A00264	A14410	207	A00264	A14430	457
A00264	A14440	486	A00264	A14443	899
A00264	A14534	232	A00264	A14606	157
A00264	A14613	224	A00264	A14643	301
A00264	A14664	218	A00264	A15089	195
A00264	A15241	204	A00264	A15639	176
A00264	A15809	446	A00264	A15906	232
A00264	A15911	156	A00264	A15912	249
A00264	A15913	443	A00264	A15952	195
A00264	A15976	180	A00264	A16040	157
A00264	A16102	157	A00264	A16300	420
A00264	A16636	491	A00264	A17155	340
A00265	A00266	188	A00265	A00271	899
A00265	A00294	899	A00265	A00348	296
A00265	A00352	461	A00265	A00386	408
A00265	A00473	299	A00265	A00531	239

A00265	A00556	263	A00265	A00616	329
A00265	A00640	249	A00265	A00651	329
A00265	A00736	290	A00265	A00831	485
A00265	A00832	229	A00265	A00887	899
A00265	A00914	389	A00265	A01021	435
A00265	A01022	391	A00265	A01115	326
A00265	A01215	226	A00265	A01354	65
A00265	A01359	534	A00265	A01425	709
A00265	A01475	469	A00265	A01492	388
A00265	A01498	249	A00265	A01524	899
A00265	A01527	899	A00265	A01566	85
A00265	A01588	899	A00265	A01607	63
A00265	A01626	401	A00265	A01632	484
A00265	A01665	899	A00265	A01676	267
A00265	A01728	165	A00265	A01729	302
A00265	A01730	491	A00265	A01731	224
A00265	A01733	435	A00265	A01740	477
A00265	A01741	407	A00265	A01743	267
A00265	A01760	152	A00265	A01770	474
A00265	A01781	358	A00265	A01859	208
A00265	A01877	347	A00265	A01887	224
A00265	A01917	198	A00265	A01983	272
A00265	A02068	224	A00265	A02099	177
A00265	A02183	229	A00265	A02337	260
A00265	A02410	174	A00265	A02487	188
A00265	A02502	305	A00265	A02512	339
A00265	A02542	227	A00265	A02562	188
A00265	A02564	316	A00265	A02568	317
A00265	A02584	174	A00265	A02629	243
A00265	A02669	159	A00265	A02695	157
A00265	A02703	452	A00265	A02736	404
A00265	A02737	420	A00265	A02805	167
A00265	A02816	447	A00265	A02825	198
A00265	A02830	479	A00265	A02867	262
A00265	A02877	360	A00265	A02921	213
A00265	A02924	241	A00265	A02939	183
A00265	A02943	213	A00265	A03057	283
A00265	A03112	489	A00265	A03211	439
A00265	A03321	112	A00265	A03436	213
A00265	A03596	163	A00265	A03612	216
A00265	A03613	196	A00265	A03796	388
A00265	A03981	366	A00265	A04012	485
A00265	A04122	152	A00265	A04161	464
A00265	A04248	371	A00265	A04306	241
A00265	A04308	429	A00265	A04475	219
A00265	A04533	318	A00265	A04536	480

A00265	A04537	322	A00265	A04622	207
A00265	A04666	656	A00265	A04721	208
A00265	A04744	219	A00265	A04821	205
A00265	A04952	262	A00265	A05085	262
A00265	A05112	333	A00265	A05288	429
A00265	A05645	270	A00265	A05944	266
A00265	A06055	205	A00265	A06409	262
A00265	A06482	322	A00265	A06489	168
A00265	A06576	294	A00265	A06597	232
A00265	A06609	899	A00265	A06626	213
A00265	A06628	317	A00265	A06780	200
A00265	A06814	428	A00265	A06871	274
A00265	A06880	322	A00265	A06916	172
A00265	A07147	152	A00265	A07157	176
A00265	A07163	324	A00265	A07220	329
A00265	A07311	156	A00265	A07399	348
A00265	A07461	438	A00265	A07495	241
A00265	A08390	152	A00265	A08572	135
A00265	A09033	154	A00265	A09121	167
A00265	A09218	356	A00265	A09383	196
A00265	A09789	152	A00265	A09917	219
A00265	A09976	348	A00265	A10038	899
A00265	A10047	165	A00265	A10082	226
A00265	A10532	486	A00265	A10801	899
A00265	A11411	157	A00265	A11441	226
A00265	A12680	322	A00265	A12988	302
A00265	A13511	730	A00265	A13654	322
A00265	A13743	154	A00265	A13886	154
A00265	A14198	414	A00265	A14272	485
A00265	A14333	319	A00265	A14432	297
A00265	A14443	912	A00265	A14444	469
A00265	A14498	470	A00265	A14539	333
A00265	A14589	479	A00265	A14635	384
A00265	A14637	188	A00265	A14643	358
A00265	A14648	311	A00265	A14661	227
A00265	A14662	177	A00265	A14674	534
A00265	A15165	249	A00265	A15875	167
A00265	A16738	534	A00266	A00825	312
A00266	A01527	899	A00266	A01554	899
A00266	A01558	899	A00266	A01588	899
A00266	A01729	326	A00266	A01755	296
A00266	A01770	358	A00266	A02488	302
A00266	A02749	201	A00266	A02805	364
A00266	A02874	157	A00266	A03520	221
A00266	A03541	340	A00266	A04275	267
A00266	A04577	417	A00266	A07422	157

A00266	A07643	223	A00266	A09385	395
A00266	A10580	928	A00266	A13609	204
A00267	A00273	454	A00267	A00400	407
A00267	A01108	217	A00267	A01156	193
A00267	A01275	479	A00267	A01291	63
A00267	A01554	283	A00267	A01562	343
A00267	A01605	412	A00267	A01628	346
A00267	A01639	457	A00267	A01664	184
A00267	A01702	210	A00267	A01718	488
A00267	A02076	317	A00267	A02188	152
A00267	A02228	200	A00267	A02518	414
A00267	A03104	390	A00267	A03331	457
A00267	A04944	165	A00267	A05289	246
A00267	A06100	480	A00267	A07166	187
A00267	A07282	401	A00267	A07836	317
A00267	A08954	319	A00267	A10609	296
A00267	A10844	277	A00267	A11831	281
A00267	A12383	202	A00267	A13199	322
A00267	A14008	201	A00267	A14197	254
A00267	A15212	285	A00267	A15704	217
A00268	A00325	234	A00268	A00355	310
A00268	A00356	335	A00268	A00357	341
A00268	A00358	486	A00268	A00361	157
A00268	A00403	281	A00268	A00629	373
A00268	A00701	201	A00268	A00802	157
A00268	A00900	201	A00268	A00902	191
A00268	A00923	474	A00268	A00990	207
A00268	A01000	293	A00268	A01056	361
A00268	A01127	461	A00268	A01140	483
A00268	A01240	316	A00268	A01249	196
A00268	A01291	945	A00268	A01338	63
A00268	A01425	486	A00268	A01524	899
A00268	A01527	899	A00268	A01614	217
A00268	A01617	938	A00268	A01618	472
A00268	A01652	252	A00268	A01718	944
A00268	A01781	263	A00268	A01983	232
A00268	A02585	196	A00268	A02826	172
A00268	A03331	140	A00268	A03332	297
A00268	A03437	416	A00268	A03567	488
A00268	A03571	183	A00268	A03705	200
A00268	A03754	451	A00268	A03913	936
A00268	A04099	218	A00268	A04230	191
A00268	A04682	218	A00268	A04760	319
A00268	A04761	154	A00268	A04867	215
A00268	A05385	438	A00268	A05712	191
A00268	A06812	314	A00268	A06965	455

A00268	A07123	210	A00268	A07398	152
A00268	A07422	899	A00268	A07451	71
A00268	A08172	157	A00268	A08835	171
A00268	A09124	191	A00268	A09802	380
A00268	A10006	252	A00268	A10233	229
A00268	A11392	210	A00268	A11400	424
A00268	A13126	159	A00268	A13207	308
A00268	A13989	183	A00268	A13992	380
A00268	A15416	245	A00268	A15932	238
A00268	A16118	443	A00269	A00270	492
A00269	A00294	899	A00269	A00337	462
A00269	A00388	339	A00269	A00400	183
A00269	A00482	551	A00269	A00545	451
A00269	A00597	252	A00269	A00779	757
A00269	A00786	990	A00269	A00874	279
A00269	A00914	899	A00269	A00929	152
A00269	A00941	457	A00269	A01003	377
A00269	A01011	316	A00269	A01139	240
A00269	A01173	268	A00269	A01268	212
A00269	A01345	491	A00269	A01359	198
A00269	A01429	343	A00269	A01454	472
A00269	A01460	152	A00269	A01478	954
A00269	A01479	491	A00269	A01486	962
A00269	A01524	899	A00269	A01527	904
A00269	A01532	339	A00269	A01533	481
A00269	A01537	439	A00269	A01558	899
A00269	A01559	302	A00269	A01560	385
A00269	A01561	159	A00269	A01572	193
A00269	A01580	491	A00269	A01588	899
A00269	A01591	981	A00269	A01592	997
A00269	A01593	939	A00269	A01595	419
A00269	A01596	528	A00269	A01615	452
A00269	A01636	466	A00269	A01638	486
A00269	A01650	973	A00269	A01651	424
A00269	A01657	245	A00269	A01667	943
A00269	A01687	313	A00269	A01689	210
A00269	A01692	449	A00269	A01703	429
A00269	A01705	361	A00269	A01717	317
A00269	A01741	180	A00269	A01839	254
A00269	A02216	226	A00269	A02278	229
A00269	A02345	232	A00269	A02508	213
A00269	A02690	274	A00269	A02779	176
A00269	A03422	650	A00269	A03484	451
A00269	A03485	174	A00269	A03492	755
A00269	A03575	701	A00269	A03591	458
A00269	A03637	467	A00269	A03651	170

A00269	A03656	235	A00269	A04117	416
A00269	A04175	343	A00269	A04345	163
A00269	A04440	477	A00269	A04622	241
A00269	A04664	113	A00269	A04668	193
A00269	A04681	174	A00269	A04715	215
A00269	A04971	473	A00269	A05008	945
A00269	A05009	972	A00269	A05015	475
A00269	A05070	319	A00269	A05072	156
A00269	A05383	491	A00269	A05625	512
A00269	A05836	259	A00269	A05853	615
A00269	A05892	473	A00269	A05894	491
A00269	A05925	174	A00269	A06100	486
A00269	A06379	488	A00269	A06402	219
A00269	A06572	234	A00269	A06606	491
A00269	A06608	374	A00269	A06609	273
A00269	A06621	177	A00269	A06919	303
A00269	A07242	347	A00269	A07282	346
A00269	A07313	188	A00269	A07339	172
A00269	A07354	281	A00269	A07422	264
A00269	A07433	197	A00269	A07762	446
A00269	A07834	179	A00269	A07836	240
A00269	A08185	382	A00269	A08400	183
A00269	A08536	311	A00269	A08551	489
A00269	A08552	156	A00269	A08569	491
A00269	A08571	949	A00269	A08836	260
A00269	A08839	764	A00269	A08844	418
A00269	A08853	296	A00269	A08858	745
A00269	A08885	639	A00269	A08986	468
A00269	A09052	273	A00269	A09182	467
A00269	A09195	491	A00269	A09220	182
A00269	A09256	208	A00269	A09370	472
A00269	A09443	945	A00269	A09449	712
A00269	A09451	260	A00269	A09491	483
A00269	A09506	413	A00269	A09511	193
A00269	A09515	172	A00269	A09525	224
A00269	A09844	255	A00269	A10139	185
A00269	A10174	899	A00269	A10175	734
A00269	A10228	367	A00269	A10250	400
A00269	A10388	259	A00269	A10395	326
A00269	A10580	396	A00269	A10604	340
A00269	A10833	636	A00269	A11006	407
A00269	A11104	162	A00269	A11156	188
A00269	A11283	469	A00269	A11486	229
A00269	A11551	252	A00269	A11676	193
A00269	A11835	258	A00269	A11940	191
A00269	A11995	281	A00269	A12047	348

A00269	A12533	393	A00269	A12553	185
A00269	A12671	259	A00269	A13277	174
A00269	A13309	177	A00269	A13315	258
A00269	A13328	329	A00269	A13685	334
A00269	A13908	319	A00269	A13917	324
A00269	A13924	486	A00269	A13978	249
A00269	A13986	193	A00269	A14055	234
A00269	A14202	195	A00269	A14227	207
A00269	A14294	260	A00269	A14299	353
A00269	A14443	990	A00269	A14444	989
A00269	A14485	470	A00269	A14531	104
A00269	A14577	667	A00269	A14585	491
A00269	A14623	474	A00269	A14633	159
A00269	A14643	157	A00269	A14792	96
A00269	A15187	229	A00269	A15533	472
A00269	A15649	179	A00269	A15726	955
A00269	A15926	362	A00269	A16224	223
A00269	A16418	173	A00269	A16503	127
A00269	A16602	201	A00269	A16714	259
A00269	A16799	219	A00269	A16824	245
A00269	A16858	163	A00269	A16905	195
A00270	A00271	183	A00270	A00275	942
A00270	A00294	899	A00270	A00335	154
A00270	A00356	355	A00270	A00377	533
A00270	A00400	876	A00270	A00418	595
A00270	A00420	443	A00270	A00421	319
A00270	A00425	263	A00270	A00448	433
A00270	A00463	486	A00270	A00471	684
A00270	A00517	396	A00270	A00520	164
A00270	A00531	274	A00270	A00566	491
A00270	A00575	488	A00270	A00600	443
A00270	A00622	353	A00270	A00643	460
A00270	A00674	207	A00270	A00733	215
A00270	A00765	489	A00270	A00805	491
A00270	A00858	491	A00270	A00881	488
A00270	A00954	491	A00270	A00978	224
A00270	A01007	188	A00270	A01112	180
A00270	A01139	379	A00270	A01164	208
A00270	A01169	530	A00270	A01195	467
A00270	A01212	734	A00270	A01299	475
A00270	A01305	188	A00270	A01329	627
A00270	A01339	200	A00270	A01359	469
A00270	A01398	488	A00270	A01401	461
A00270	A01474	208	A00270	A01500	443
A00270	A01503	484	A00270	A01524	899
A00270	A01527	939	A00270	A01546	440

A00270	A01558	294	A00270	A01570	288
A00270	A01576	527	A00270	A01591	485
A00270	A01595	199	A00270	A01615	468
A00270	A01647	491	A00270	A01682	481
A00270	A01790	215	A00270	A01844	204
A00270	A02138	183	A00270	A02304	187
A00270	A02836	210	A00270	A03014	165
A00270	A03076	235	A00270	A03422	288
A00270	A03809	165	A00270	A03893	489
A00270	A03939	215	A00270	A04033	491
A00270	A04345	115	A00270	A04445	201
A00270	A04971	63	A00270	A05221	461
A00270	A05288	477	A00270	A05567	210
A00270	A06039	199	A00270	A06588	450
A00270	A06597	548	A00270	A06650	416
A00270	A06750	297	A00270	A06774	151
A00270	A06803	294	A00270	A07252	173
A00270	A07409	281	A00270	A07556	151
A00270	A07670	491	A00270	A07803	491
A00270	A07821	173	A00270	A07826	221
A00270	A07850	363	A00270	A07887	353
A00270	A07920	489	A00270	A08022	245
A00270	A08376	238	A00270	A08495	409
A00270	A08571	491	A00270	A08659	210
A00270	A08869	232	A00270	A08878	337
A00270	A09162	210	A00270	A09256	400
A00270	A09258	939	A00270	A09377	363
A00270	A09401	156	A00270	A09451	419
A00270	A09515	389	A00270	A09538	468
A00270	A09712	212	A00270	A09992	195
A00270	A10175	229	A00270	A10228	301
A00270	A10250	336	A00270	A10340	293
A00270	A10342	445	A00270	A10354	154
A00270	A10415	151	A00270	A10432	151
A00270	A10447	210	A00270	A10531	249
A00270	A10599	177	A00270	A11387	174
A00270	A11485	165	A00270	A11492	414
A00270	A11983	491	A00270	A12095	151
A00270	A12284	491	A00270	A12365	267
A00270	A12681	404	A00270	A12812	249
A00270	A12897	408	A00270	A13039	210
A00270	A13040	210	A00270	A13216	180
A00270	A13248	151	A00270	A13850	210
A00270	A13892	274	A00270	A14055	484
A00270	A14128	465	A00270	A14204	466
A00270	A14229	446	A00270	A14278	183

A00270	A14331	151	A00270	A14359	168
A00270	A14443	989	A00270	A14633	217
A00270	A14635	490	A00270	A14636	408
A00270	A14643	243	A00270	A14824	173
A00270	A14925	162	A00270	A14950	210
A00270	A15292	329	A00270	A15293	238
A00270	A15295	491	A00270	A15322	489
A00270	A15349	305	A00270	A15726	899
A00270	A16294	443	A00270	A16491	266
A00270	A16773	173	A00270	A16916	485
A00271	A00324	191	A00271	A00381	191
A00271	A00493	195	A00271	A00506	249
A00271	A00832	63	A00271	A00887	900
A00271	A01052	899	A00271	A01450	303
A00271	A01524	899	A00271	A01527	899
A00271	A01558	899	A00271	A01562	899
A00271	A01572	229	A00271	A01588	899
A00271	A01602	899	A00271	A01665	905
A00271	A01667	899	A00271	A01676	63
A00271	A01692	899	A00271	A01699	152
A00271	A02535	307	A00271	A02539	219
A00271	A02544	899	A00271	A02713	193
A00271	A02779	196	A00271	A02805	213
A00271	A03525	180	A00271	A03591	899
A00271	A03611	938	A00271	A03664	162
A00271	A03777	218	A00271	A03923	174
A00271	A04209	156	A00271	A04212	369
A00271	A04699	899	A00271	A04843	291
A00271	A04870	172	A00271	A05162	276
A00271	A05188	352	A00271	A05992	172
A00271	A06416	246	A00271	A06429	185
A00271	A06609	899	A00271	A07176	488
A00271	A07311	899	A00271	A07318	352
A00271	A08581	436	A00271	A09631	283
A00271	A10523	276	A00271	A10813	160
A00271	A11745	191	A00271	A12383	899
A00271	A13391	899	A00271	A13977	899
A00271	A13990	899	A00271	A14188	290
A00271	A14198	899	A00271	A14556	276
A00271	A14643	246	A00271	A14707	185
A00272	A00653	358	A00272	A00808	173
A00272	A01272	213	A00272	A01527	905
A00272	A01639	900	A00272	A02235	176
A00272	A02329	213	A00272	A02821	238
A00272	A02836	694	A00272	A03050	472
A00272	A03172	271	A00272	A03322	418

A00272	A05288	614	A00272	A07311	540
A00272	A07422	973	A00272	A07471	400
A00272	A07475	184	A00272	A07635	226
A00272	A09072	185	A00272	A10386	290
A00272	A10392	899	A00272	A11834	943
A00272	A13978	899	A00272	A14062	762
A00272	A14063	455	A00272	A14191	191
A00273	A00350	184	A00273	A01286	195
A00273	A01512	358	A00273	A01553	396
A00273	A01554	373	A00273	A01561	249
A00273	A01562	177	A00273	A01605	204
A00273	A01628	473	A00273	A01697	373
A00273	A01702	328	A00273	A01708	252
A00273	A01709	409	A00273	A01718	383
A00273	A01719	446	A00273	A01907	193
A00273	A01908	130	A00273	A03822	168
A00273	A04452	368	A00273	A06289	80
A00273	A06466	63	A00273	A07165	205
A00273	A09162	445	A00273	A09526	152
A00273	A09643	343	A00273	A09644	165
A00273	A11369	173	A00273	A11767	294
A00273	A12383	463	A00273	A13729	291
A00273	A13980	173	A00273	A14364	213
A00273	A14402	271	A00273	A14438	297
A00273	A16531	200	A00274	A00290	154
A00274	A00292	490	A00274	A00440	490
A00274	A00616	416	A00274	A00736	414
A00274	A00752	491	A00274	A00815	139
A00274	A01009	179	A00274	A01030	240
A00274	A01046	360	A00274	A01235	267
A00274	A01257	323	A00274	A01275	366
A00274	A01276	224	A00274	A01354	162
A00274	A01493	196	A00274	A01527	899
A00274	A01557	159	A00274	A01572	463
A00274	A01599	210	A00274	A01604	488
A00274	A01630	157	A00274	A01654	319
A00274	A01699	238	A00274	A01704	393
A00274	A01741	262	A00274	A01743	243
A00274	A01870	467	A00274	A01878	151
A00274	A01879	238	A00274	A01880	296
A00274	A01929	176	A00274	A01983	196
A00274	A02052	326	A00274	A02062	257
A00274	A02088	391	A00274	A02136	154
A00274	A02138	466	A00274	A02143	457
A00274	A02174	446	A00274	A02176	152
A00274	A02236	404	A00274	A02245	480

A00274	A02282	184	A00274	A02337	269
A00274	A02356	157	A00274	A02370	299
A00274	A02381	183	A00274	A02382	491
A00274	A02400	227	A00274	A02416	240
A00274	A02425	127	A00274	A02431	191
A00274	A02439	321	A00274	A02464	382
A00274	A02472	63	A00274	A02547	232
A00274	A02578	383	A00274	A02606	151
A00274	A02608	388	A00274	A02609	227
A00274	A02616	288	A00274	A02624	265
A00274	A02637	281	A00274	A02654	353
A00274	A02655	475	A00274	A02769	430
A00274	A02812	213	A00274	A02896	168
A00274	A02945	165	A00274	A03049	465
A00274	A03055	180	A00274	A03063	240
A00274	A03081	477	A00274	A03093	441
A00274	A03104	276	A00274	A03109	491
A00274	A03110	945	A00274	A03131	180
A00274	A03183	491	A00274	A03185	200
A00274	A03219	490	A00274	A03224	218
A00274	A03239	171	A00274	A03259	474
A00274	A03305	480	A00274	A03310	310
A00274	A03313	272	A00274	A03314	262
A00274	A03390	179	A00274	A03398	199
A00274	A03466	240	A00274	A03639	258
A00274	A03713	154	A00274	A03724	183
A00274	A03788	467	A00274	A03799	473
A00274	A03817	380	A00274	A03900	152
A00274	A03919	154	A00274	A03942	491
A00274	A03961	267	A00274	A04040	159
A00274	A04169	302	A00274	A04248	475
A00274	A04257	401	A00274	A04365	232
A00274	A04368	193	A00274	A04450	338
A00274	A04473	165	A00274	A04478	462
A00274	A04482	212	A00274	A04492	486
A00274	A04589	252	A00274	A04676	290
A00274	A04696	176	A00274	A04748	227
A00274	A04755	317	A00274	A04756	441
A00274	A04780	180	A00274	A04790	174
A00274	A04822	154	A00274	A04976	332
A00274	A05118	257	A00274	A05133	268
A00274	A05145	207	A00274	A05180	327
A00274	A05408	402	A00274	A05577	450
A00274	A05679	321	A00274	A05706	483
A00274	A05815	414	A00274	A05944	477
A00274	A06015	202	A00274	A06054	488

A00274	A06118	435	A00274	A06269	200
A00274	A06414	286	A00274	A06415	458
A00274	A06604	193	A00274	A06899	172
A00274	A07006	247	A00274	A07126	407
A00274	A07133	268	A00274	A07140	173
A00274	A07143	418	A00274	A07145	489
A00274	A07148	159	A00274	A07152	224
A00274	A07157	423	A00274	A07161	383
A00274	A07166	343	A00274	A07173	154
A00274	A07184	154	A00274	A07209	367
A00274	A07372	335	A00274	A07390	283
A00274	A07803	481	A00274	A08641	263
A00274	A08642	327	A00274	A08644	268
A00274	A08714	221	A00274	A08859	154
A00274	A08881	160	A00274	A09092	165
A00274	A09125	281	A00274	A09219	184
A00274	A09241	432	A00274	A09243	174
A00274	A09366	327	A00274	A09433	202
A00274	A09480	400	A00274	A09552	163
A00274	A09572	943	A00274	A09732	485
A00274	A09744	172	A00274	A09929	227
A00274	A10018	353	A00274	A10090	363
A00274	A10336	408	A00274	A10403	281
A00274	A10744	193	A00274	A10796	180
A00274	A10821	151	A00274	A11090	221
A00274	A11412	291	A00274	A11495	327
A00274	A11875	232	A00274	A12393	352
A00274	A13826	425	A00274	A13860	480
A00274	A13950	179	A00274	A14004	221
A00274	A14007	201	A00274	A14103	268
A00274	A14122	278	A00274	A14190	485
A00274	A14202	185	A00274	A14210	491
A00274	A14366	489	A00274	A14371	491
A00274	A14372	425	A00274	A14374	419
A00274	A14376	162	A00274	A14379	428
A00274	A14391	480	A00274	A14392	372
A00274	A14409	327	A00274	A14430	422
A00274	A14432	172	A00274	A14511	489
A00274	A14536	196	A00274	A14554	491
A00274	A14570	422	A00274	A14596	385
A00274	A14601	327	A00274	A14604	488
A00274	A14616	257	A00274	A14667	188
A00274	A14930	152	A00274	A15285	174
A00274	A15292	480	A00274	A15312	299
A00274	A15313	326	A00274	A15314	224
A00274	A15317	221	A00274	A15318	449

A00274	A15322	330	A00274	A15327	344
A00274	A15346	369	A00274	A15349	319
A00274	A15352	413	A00274	A15404	172
A00274	A15422	340	A00274	A15452	257
A00274	A15495	458	A00274	A15516	205
A00274	A15605	383	A00274	A15607	159
A00274	A15608	394	A00274	A15641	240
A00274	A15726	268	A00274	A15819	319
A00274	A15884	163	A00274	A15916	288
A00274	A15935	156	A00274	A16008	184
A00274	A16037	268	A00274	A16224	174
A00274	A16323	383	A00274	A17081	227
A00275	A00294	899	A00275	A00339	180
A00275	A00369	124	A00275	A00385	188
A00275	A00400	213	A00275	A00421	107
A00275	A00471	176	A00275	A00531	151
A00275	A00905	115	A00275	A01004	430
A00275	A01139	899	A00275	A01151	63
A00275	A01359	899	A00275	A01437	238
A00275	A01524	901	A00275	A01527	910
A00275	A01591	319	A00275	A01660	152
A00275	A02158	409	A00275	A02226	394
A00275	A02877	251	A00275	A03494	63
A00275	A04608	177	A00275	A05853	172
A00275	A06063	184	A00275	A06608	200
A00275	A07145	193	A00275	A08571	256
A00275	A08866	291	A00275	A09258	899
A00275	A09558	191	A00275	A12990	353
A00275	A13527	271	A00275	A14443	989
A00275	A14584	330	A00275	A15438	335
A00275	A15448	361	A00275	A15449	330
A00275	A15726	899	A00275	A16453	238
A00275	A16473	319	A00276	A01524	899
A00276	A01527	910	A00276	A01562	941
A00276	A01692	182	A00276	A02411	271
A00276	A04117	198	A00276	A04280	281
A00276	A04706	347	A00276	A05462	377
A00276	A14033	899	A00276	A15512	375
A00276	A15665	154	A00277	A00327	425
A00277	A00328	467	A00277	A00651	489
A00277	A00763	139	A00277	A00861	401
A00277	A00870	115	A00277	A00902	241
A00277	A01129	347	A00277	A01251	276
A00277	A01288	255	A00277	A01464	165
A00277	A01496	457	A00277	A01598	63
A00277	A01767	152	A00277	A02164	70

A00277	A02494	221	A00277	A03243	193
A00277	A03432	240	A00277	A03438	429
A00277	A03609	162	A00277	A03981	491
A00277	A04990	326	A00277	A05562	293
A00277	A06814	406	A00277	A06825	228
A00277	A07506	469	A00277	A08052	213
A00277	A08070	176	A00277	A08682	407
A00277	A08748	272	A00277	A08768	283
A00277	A10791	272	A00277	A11331	350
A00277	A11981	364	A00277	A12554	319
A00277	A13989	165	A00277	A14216	401
A00277	A15673	159	A00278	A00839	201
A00278	A00895	279	A00278	A01166	160
A00278	A01206	232	A00278	A01335	160
A00278	A01781	216	A00278	A03619	374
A00278	A07822	307	A00278	A08357	202
A00278	A11487	159	A00278	A15660	288
A00278	A15918	382	A00278	A15920	398
A00278	A15932	200	A00278	A15933	257
A00278	A16096	294	A00279	A00280	409
A00279	A00281	461	A00279	A00288	491
A00279	A00423	422	A00279	A00571	165
A00279	A00590	171	A00279	A00593	167
A00279	A00651	474	A00279	A00700	196
A00279	A00705	481	A00279	A00782	219
A00279	A00839	491	A00279	A00878	377
A00279	A00966	201	A00279	A01112	191
A00279	A01196	236	A00279	A01270	420
A00279	A01275	159	A00279	A01291	152
A00279	A01306	489	A00279	A01335	488
A00279	A01337	171	A00279	A01369	327
A00279	A01468	324	A00279	A01496	489
A00279	A01579	565	A00279	A03069	409
A00279	A03296	208	A00279	A03859	200
A00279	A04858	200	A00279	A05501	205
A00279	A06897	395	A00279	A06965	200
A00279	A06973	455	A00279	A07047	392
A00279	A07533	154	A00279	A07581	372
A00279	A08131	265	A00279	A08137	345
A00279	A08358	339	A00279	A08359	566
A00279	A08369	299	A00279	A08408	276
A00279	A08457	565	A00279	A08466	200
A00279	A08476	200	A00279	A08983	313
A00279	A09322	200	A00279	A11420	379
A00279	A11550	395	A00279	A11562	200
A00279	A11572	399	A00279	A11616	176

A00279	A11728	561	A00279	A11861	200
A00279	A11924	447	A00279	A12011	236
A00279	A12270	474	A00279	A12821	162
A00279	A13333	200	A00279	A14216	490
A00279	A14508	450	A00279	A14923	313
A00279	A14937	474	A00279	A15202	200
A00279	A15225	491	A00279	A15500	172
A00279	A15684	293	A00279	A15732	395
A00279	A15746	188	A00279	A15937	73
A00280	A00283	566	A00280	A00288	368
A00280	A00299	566	A00280	A00325	491
A00280	A00328	470	A00280	A00334	486
A00280	A00355	566	A00280	A00356	491
A00280	A00357	491	A00280	A00358	491
A00280	A00403	491	A00280	A00405	326
A00280	A00406	406	A00280	A00448	232
A00280	A00454	411	A00280	A00467	417
A00280	A00469	266	A00280	A00488	483
A00280	A00501	232	A00280	A00521	566
A00280	A00561	443	A00280	A00589	303
A00280	A00590	491	A00280	A00603	484
A00280	A00605	491	A00280	A00629	490
A00280	A00648	491	A00280	A00651	485
A00280	A00653	491	A00280	A00701	489
A00280	A00713	424	A00280	A00763	491
A00280	A00783	465	A00280	A00785	217
A00280	A00787	491	A00280	A00827	151
A00280	A00839	491	A00280	A00848	270
A00280	A00878	491	A00280	A00923	491
A00280	A00965	396	A00280	A00970	255
A00280	A00990	200	A00280	A01000	491
A00280	A01017	412	A00280	A01020	296
A00280	A01025	491	A00280	A01027	293
A00280	A01031	260	A00280	A01042	475
A00280	A01047	566	A00280	A01052	221
A00280	A01054	477	A00280	A01056	475
A00280	A01066	320	A00280	A01101	491
A00280	A01112	412	A00280	A01115	463
A00280	A01120	294	A00280	A01127	491
A00280	A01140	481	A00280	A01150	408
A00280	A01166	162	A00280	A01184	491
A00280	A01189	491	A00280	A01206	491
A00280	A01211	191	A00280	A01234	490
A00280	A01249	566	A00280	A01263	430
A00280	A01264	200	A00280	A01282	216
A00280	A01291	643	A00280	A01306	491

A00280	A01335	475	A00280	A01338	566
A00280	A01392	219	A00280	A01399	210
A00280	A01429	562	A00280	A01430	477
A00280	A01431	454	A00280	A01433	416
A00280	A01437	491	A00280	A01449	208
A00280	A01457	491	A00280	A01464	562
A00280	A01468	485	A00280	A01470	416
A00280	A01496	477	A00280	A01569	257
A00280	A01579	491	A00280	A01598	251
A00280	A01612	391	A00280	A01617	229
A00280	A01618	369	A00280	A01619	157
A00280	A01620	286	A00280	A01649	491
A00280	A01700	278	A00280	A01718	281
A00280	A01787	475	A00280	A01822	294
A00280	A01897	353	A00280	A02178	226
A00280	A02216	481	A00280	A02738	176
A00280	A02874	428	A00280	A02978	503
A00280	A03048	188	A00280	A03069	491
A00280	A03149	257	A00280	A03272	193
A00280	A03567	491	A00280	A03740	484
A00280	A03752	433	A00280	A03754	480
A00280	A03859	385	A00280	A04430	180
A00280	A04760	195	A00280	A05121	491
A00280	A05385	265	A00280	A05411	205
A00280	A05443	374	A00280	A05501	328
A00280	A06279	232	A00280	A06466	473
A00280	A06861	480	A00280	A06897	481
A00280	A06965	491	A00280	A06973	562
A00280	A07201	183	A00280	A07311	473
A00280	A07398	313	A00280	A07451	491
A00280	A07505	207	A00280	A07533	490
A00280	A07584	443	A00280	A08011	491
A00280	A08055	429	A00280	A08137	491
A00280	A08301	418	A00280	A08357	491
A00280	A08358	240	A00280	A08359	477
A00280	A08377	171	A00280	A08398	566
A00280	A08408	221	A00280	A08452	566
A00280	A08501	249	A00280	A08521	565
A00280	A08547	470	A00280	A08558	348
A00280	A08559	480	A00280	A08664	481
A00280	A08773	480	A00280	A08972	479
A00280	A08973	190	A00280	A09044	321
A00280	A09297	207	A00280	A09322	491
A00280	A09332	362	A00280	A09615	165
A00280	A09674	435	A00280	A09702	564
A00280	A09852	329	A00280	A10417	535

A00280	A10439	388	A00280	A10518	490
A00280	A10529	171	A00280	A10532	307
A00280	A10616	480	A00280	A11303	152
A00280	A11338	187	A00280	A11390	491
A00280	A11419	228	A00280	A11420	316
A00280	A11438	565	A00280	A11462	277
A00280	A11480	490	A00280	A11588	488
A00280	A11616	258	A00280	A11704	470
A00280	A11729	200	A00280	A11731	224
A00280	A11945	491	A00280	A11974	224
A00280	A12080	176	A00280	A12126	435
A00280	A12221	154	A00280	A12233	489
A00280	A12237	399	A00280	A12259	195
A00280	A12329	223	A00280	A12813	195
A00280	A13055	180	A00280	A13333	555
A00280	A13340	468	A00280	A13375	241
A00280	A13417	151	A00280	A13525	480
A00280	A13527	450	A00280	A13709	213
A00280	A13830	486	A00280	A13989	491
A00280	A13992	489	A00280	A14152	281
A00280	A14188	490	A00280	A14216	480
A00280	A14505	490	A00280	A15215	266
A00280	A15218	345	A00280	A15219	348
A00280	A15221	483	A00280	A15235	467
A00280	A15660	413	A00280	A15684	335
A00280	A15727	165	A00280	A15743	477
A00280	A15776	337	A00280	A15777	430
A00280	A15778	223	A00280	A15932	74
A00280	A15988	488	A00280	A16118	489
A00280	A16159	335	A00280	A16523	180
A00280	A16622	63	A00280	A16631	491
A00280	A16679	422	A00280	A16768	160
A00280	A16845	293	A00280	A17142	219
A00280	A17193	363	A00281	A00288	491
A00281	A00289	351	A00281	A00304	488
A00281	A00360	195	A00281	A00651	472
A00281	A00675	170	A00281	A00808	165
A00281	A00839	180	A00281	A01085	263
A00281	A01090	341	A00281	A01112	483
A00281	A01291	184	A00281	A01306	296
A00281	A01335	439	A00281	A01468	190
A00281	A01496	474	A00281	A01579	488
A00281	A01601	469	A00281	A01946	247
A00281	A03981	258	A00281	A06466	174
A00281	A06534	330	A00281	A07311	217
A00281	A07581	168	A00281	A08137	293

A00281	A08358	262	A00281	A08359	491
A00281	A08369	446	A00281	A08457	485
A00281	A08476	446	A00281	A08559	151
A00281	A08838	159	A00281	A09297	162
A00281	A11334	160	A00281	A11420	486
A00281	A11550	199	A00281	A11612	185
A00281	A11728	215	A00281	A11861	263
A00281	A11924	413	A00281	A12105	177
A00281	A12269	484	A00281	A12821	394
A00281	A13308	247	A00281	A13311	157
A00281	A13340	440	A00281	A14176	310
A00281	A14216	491	A00281	A14257	154
A00281	A15500	196	A00281	A16624	290
A00281	A16627	172	A00281	A16724	246
A00281	A16839	296	A00282	A00651	212
A00282	A00698	272	A00282	A00741	235
A00282	A00819	254	A00282	A00835	185
A00282	A00876	252	A00282	A01082	168
A00282	A01385	160	A00282	A01787	263
A00282	A01822	339	A00282	A06466	204
A00282	A07208	283	A00282	A07798	179
A00282	A09297	313	A00282	A10728	270
A00282	A11337	279	A00282	A12170	368
A00282	A12328	218	A00282	A15261	296
A00282	A15932	205	A00282	A16272	313
A00282	A16318	348	A00282	A16437	338
A00282	A16620	63	A00283	A00288	477
A00283	A00299	566	A00283	A00325	491
A00283	A00327	472	A00283	A00328	665
A00283	A00355	565	A00283	A00356	395
A00283	A00357	491	A00283	A00358	488
A00283	A00403	491	A00283	A00521	565
A00283	A00571	212	A00283	A00590	480
A00283	A00603	296	A00283	A00605	447
A00283	A00629	569	A00283	A00648	322
A00283	A00651	481	A00283	A00653	290
A00283	A00701	483	A00283	A00763	488
A00283	A00787	491	A00283	A00839	490
A00283	A00878	462	A00283	A00923	491
A00283	A00970	154	A00283	A00990	638
A00283	A01000	491	A00283	A01025	491
A00283	A01047	566	A00283	A01066	204
A00283	A01098	199	A00283	A01101	485
A00283	A01112	439	A00283	A01127	488
A00283	A01140	173	A00283	A01184	488
A00283	A01186	179	A00283	A01189	299

A00283	A01206	456	A00283	A01234	193
A00283	A01240	405	A00283	A01249	565
A00283	A01263	458	A00283	A01264	200
A00283	A01282	486	A00283	A01291	643
A00283	A01306	582	A00283	A01338	560
A00283	A01392	223	A00283	A01425	211
A00283	A01429	200	A00283	A01437	491
A00283	A01457	485	A00283	A01464	668
A00283	A01496	469	A00283	A01522	207
A00283	A01579	481	A00283	A01585	196
A00283	A01601	199	A00283	A01618	635
A00283	A01634	207	A00283	A01647	230
A00283	A01649	455	A00283	A01684	317
A00283	A01755	157	A00283	A01822	575
A00283	A02216	154	A00283	A02738	438
A00283	A02978	200	A00283	A03069	491
A00283	A03567	581	A00283	A03705	205
A00283	A03740	368	A00283	A03754	509
A00283	A03761	234	A00283	A03888	485
A00283	A03981	172	A00283	A05121	310
A00283	A05411	252	A00283	A05449	393
A00283	A05708	241	A00283	A06015	187
A00283	A06466	151	A00283	A06495	353
A00283	A06812	217	A00283	A06843	183
A00283	A06848	195	A00283	A06861	221
A00283	A06897	347	A00283	A06909	224
A00283	A06965	491	A00283	A06973	551
A00283	A07012	314	A00283	A07104	205
A00283	A07201	491	A00283	A07243	393
A00283	A07311	483	A00283	A07451	475
A00283	A08011	433	A00283	A08086	151
A00283	A08133	454	A00283	A08321	171
A00283	A08357	467	A00283	A08358	291
A00283	A08359	457	A00283	A08398	552
A00283	A08452	565	A00283	A08501	299
A00283	A08521	287	A00283	A08558	200
A00283	A08559	491	A00283	A08664	639
A00283	A08752	402	A00283	A08972	414
A00283	A09044	200	A00283	A09322	581
A00283	A09332	196	A00283	A09622	226
A00283	A09674	477	A00283	A09702	200
A00283	A09822	252	A00283	A09852	474
A00283	A10002	252	A00283	A10133	241
A00283	A10417	530	A00283	A10518	278
A00283	A10569	241	A00283	A10584	366
A00283	A11390	180	A00283	A11420	274

A00283	A11438	566	A00283	A11480	443
A00283	A11704	343	A00283	A11729	200
A00283	A11945	467	A00283	A11981	210
A00283	A12011	273	A00283	A12233	414
A00283	A12237	217	A00283	A12500	210
A00283	A12813	224	A00283	A13333	200
A00283	A13340	474	A00283	A13525	154
A00283	A13527	352	A00283	A13830	205
A00283	A13989	344	A00283	A13992	353
A00283	A14188	380	A00283	A14216	500
A00283	A14505	450	A00283	A14508	162
A00283	A14923	232	A00283	A15218	463
A00283	A15219	152	A00283	A15221	374
A00283	A15235	490	A00283	A15727	195
A00283	A15743	485	A00283	A16118	469
A00283	A16627	163	A00283	A16631	296
A00283	A16633	162	A00283	A16822	312
A00283	A16823	200	A00284	A00285	438
A00284	A00288	340	A00284	A00439	568
A00284	A00454	330	A00284	A00698	498
A00284	A00809	160	A00284	A00833	222
A00284	A00876	520	A00284	A00922	336
A00284	A00932	517	A00284	A00933	290
A00284	A00980	423	A00284	A01055	249
A00284	A01090	177	A00284	A01161	160
A00284	A01218	190	A00284	A01261	160
A00284	A01393	187	A00284	A01554	424
A00284	A01560	382	A00284	A01579	278
A00284	A01627	162	A00284	A01680	489
A00284	A01822	440	A00284	A02350	283
A00284	A03490	185	A00284	A03677	160
A00284	A03691	274	A00284	A03752	658
A00284	A03809	362	A00284	A04116	317
A00284	A06000	279	A00284	A06351	154
A00284	A06466	695	A00284	A06950	438
A00284	A07311	491	A00284	A07323	174
A00284	A07798	671	A00284	A08054	177
A00284	A08301	193	A00284	A08519	199
A00284	A08533	503	A00284	A09297	524
A00284	A09308	229	A00284	A09577	491
A00284	A09873	180	A00284	A09902	465
A00284	A10534	481	A00284	A11302	212
A00284	A11550	413	A00284	A12042	274
A00284	A12043	160	A00284	A12227	160
A00284	A12328	360	A00284	A12990	232
A00284	A13340	542	A00284	A14073	491

A00284	A14188	251	A00284	A14738	160
A00284	A15244	487	A00284	A15932	460
A00284	A15933	461	A00284	A16272	531
A00284	A16318	160	A00284	A16437	160
A00284	A16446	160	A00284	A16585	463
A00284	A16618	335	A00285	A00288	163
A00285	A00356	185	A00285	A00371	270
A00285	A00439	691	A00285	A00444	310
A00285	A00626	252	A00285	A00651	490
A00285	A00698	373	A00285	A00701	274
A00285	A00713	353	A00285	A00773	249
A00285	A00839	414	A00285	A00910	213
A00285	A00923	167	A00285	A00932	485
A00285	A00933	468	A00285	A01042	491
A00285	A01055	457	A00285	A01083	157
A00285	A01112	491	A00285	A01115	232
A00285	A01150	378	A00285	A01275	470
A00285	A01291	454	A00285	A01306	184
A00285	A01431	434	A00285	A01457	458
A00285	A01496	483	A00285	A01560	491
A00285	A01569	474	A00285	A01579	490
A00285	A01647	458	A00285	A01712	188
A00285	A01782	152	A00285	A01822	326
A00285	A02857	246	A00285	A03188	477
A00285	A03545	230	A00285	A03740	452
A00285	A03752	695	A00285	A05066	193
A00285	A06037	228	A00285	A06466	695
A00285	A06645	180	A00285	A06950	515
A00285	A07247	157	A00285	A07311	491
A00285	A07451	474	A00285	A07604	296
A00285	A07798	695	A00285	A08359	252
A00285	A08835	293	A00285	A09078	224
A00285	A09263	491	A00285	A09319	466
A00285	A09581	165	A00285	A09804	456
A00285	A09902	480	A00285	A10365	474
A00285	A10532	311	A00285	A10728	489
A00285	A11286	491	A00285	A12073	177
A00285	A12328	462	A00285	A13146	297
A00285	A13149	404	A00285	A13320	473
A00285	A13335	488	A00285	A13340	489
A00285	A13529	262	A00285	A13552	247
A00285	A13989	475	A00285	A14150	156
A00285	A14188	152	A00285	A14571	329
A00285	A14596	429	A00285	A15244	460
A00285	A15546	443	A00285	A16805	212
A00286	A00348	488	A00286	A00374	232

A00286	A00382	168	A00286	A00393	180
A00286	A00421	416	A00286	A00425	356
A00286	A00448	450	A00286	A00453	479
A00286	A00458	491	A00286	A00481	488
A00286	A00496	375	A00286	A00531	490
A00286	A00532	491	A00286	A00571	162
A00286	A00605	245	A00286	A00655	472
A00286	A00691	260	A00286	A00760	485
A00286	A00762	479	A00286	A00793	380
A00286	A00822	218	A00286	A00831	221
A00286	A00848	251	A00286	A00856	357
A00286	A00871	270	A00286	A00876	63
A00286	A00883	317	A00286	A00905	413
A00286	A00916	489	A00286	A00919	474
A00286	A00947	460	A00286	A01020	272
A00286	A01075	491	A00286	A01082	489
A00286	A01084	489	A00286	A01087	491
A00286	A01088	413	A00286	A01096	182
A00286	A01136	489	A00286	A01166	312
A00286	A01184	361	A00286	A01206	395
A00286	A01215	458	A00286	A01234	484
A00286	A01243	170	A00286	A01259	190
A00286	A01353	382	A00286	A01354	490
A00286	A01385	380	A00286	A01406	488
A00286	A01423	190	A00286	A01468	269
A00286	A01470	430	A00286	A01492	457
A00286	A01495	374	A00286	A01572	414
A00286	A01598	466	A00286	A01612	470
A00286	A01614	367	A00286	A01649	379
A00286	A01707	489	A00286	A01763	185
A00286	A02243	187	A00286	A02274	260
A00286	A02671	245	A00286	A03057	404
A00286	A03446	330	A00286	A03453	314
A00286	A03569	402	A00286	A03601	490
A00286	A03728	68	A00286	A05328	301
A00286	A05501	219	A00286	A05709	171
A00286	A07349	177	A00286	A07524	63
A00286	A07706	491	A00286	A08137	458
A00286	A08304	283	A00286	A08337	260
A00286	A08357	160	A00286	A08491	165
A00286	A08528	252	A00286	A08571	195
A00286	A09608	283	A00286	A09825	188
A00286	A10532	274	A00286	A10538	420
A00286	A11291	405	A00286	A11575	318
A00286	A11577	174	A00286	A11930	399
A00286	A13603	329	A00286	A13870	317

A00286	A13956	162	A00286	A13989	491
A00286	A14006	484	A00286	A14220	486
A00286	A14566	491	A00286	A14594	486
A00286	A15038	329	A00286	A15041	329
A00286	A15046	329	A00286	A15091	329
A00286	A15128	329	A00286	A15215	329
A00286	A15218	168	A00286	A15935	408
A00286	A16320	352	A00286	A17063	380
A00287	A00571	328	A00287	A00955	180
A00287	A01000	375	A00287	A01064	168
A00287	A01093	196	A00287	A01121	279
A00287	A01154	360	A00287	A01473	472
A00287	A01594	249	A00287	A01686	288
A00287	A02108	204	A00287	A03461	170
A00287	A04949	285	A00287	A05446	266
A00287	A08000	160	A00287	A08154	174
A00287	A08419	344	A00287	A09610	396
A00287	A09852	274	A00287	A11364	433
A00287	A12477	477	A00287	A13554	358
A00287	A15941	454	A00288	A00289	491
A00288	A00296	281	A00288	A00299	469
A00288	A00304	470	A00288	A00323	213
A00288	A00334	255	A00288	A00357	179
A00288	A00414	270	A00288	A00423	243
A00288	A00454	205	A00288	A00467	462
A00288	A00521	218	A00288	A00560	414
A00288	A00561	452	A00288	A00571	709
A00288	A00590	180	A00288	A00593	455
A00288	A00605	470	A00288	A00648	485
A00288	A00651	491	A00288	A00700	491
A00288	A00705	491	A00288	A00782	477
A00288	A00787	165	A00288	A00808	154
A00288	A00839	645	A00288	A00878	290
A00288	A00905	461	A00288	A00916	443
A00288	A00923	466	A00288	A00955	168
A00288	A00966	311	A00288	A00970	212
A00288	A01017	488	A00288	A01025	408
A00288	A01042	441	A00288	A01083	162
A00288	A01085	357	A00288	A01090	489
A00288	A01098	248	A00288	A01112	491
A00288	A01115	432	A00288	A01150	307
A00288	A01196	465	A00288	A01206	486
A00288	A01234	432	A00288	A01249	198
A00288	A01270	330	A00288	A01291	580
A00288	A01294	196	A00288	A01306	491
A00288	A01335	491	A00288	A01369	184

A00288	A01425	275	A00288	A01431	306
A00288	A01457	193	A00288	A01468	491
A00288	A01470	263	A00288	A01496	491
A00288	A01539	474	A00288	A01569	258
A00288	A01572	199	A00288	A01579	491
A00288	A01601	491	A00288	A01612	491
A00288	A01649	473	A00288	A01787	446
A00288	A02350	408	A00288	A03298	159
A00288	A03390	391	A00288	A03567	317
A00288	A03569	416	A00288	A03740	481
A00288	A03752	457	A00288	A03981	475
A00288	A04116	334	A00288	A05501	480
A00288	A06466	490	A00288	A06564	390
A00288	A06897	488	A00288	A06965	396
A00288	A06973	489	A00288	A07047	483
A00288	A07311	629	A00288	A07398	368
A00288	A07451	279	A00288	A07524	63
A00288	A07581	449	A00288	A08131	491
A00288	A08137	491	A00288	A08154	390
A00288	A08301	276	A00288	A08357	435
A00288	A08358	477	A00288	A08359	491
A00288	A08377	224	A00288	A08408	462
A00288	A08452	152	A00288	A08457	485
A00288	A08476	491	A00288	A08571	363
A00288	A08983	229	A00288	A09322	288
A00288	A09613	251	A00288	A09726	276
A00288	A10362	278	A00288	A10490	286
A00288	A10498	180	A00288	A10532	280
A00288	A10797	414	A00288	A11096	154
A00288	A11254	180	A00288	A11334	385
A00288	A11420	486	A00288	A11550	491
A00288	A11593	165	A00288	A11857	488
A00288	A11861	483	A00288	A12011	270
A00288	A12269	452	A00288	A12270	230
A00288	A12328	278	A00288	A12329	383
A00288	A12365	306	A00288	A12821	452
A00288	A13167	229	A00288	A13262	313
A00288	A13311	324	A00288	A13340	491
A00288	A13357	180	A00288	A13527	252
A00288	A13989	488	A00288	A14152	172
A00288	A14188	405	A00288	A14206	263
A00288	A14216	491	A00288	A14257	468
A00288	A14447	251	A00288	A14476	177
A00288	A14508	489	A00288	A14923	229
A00288	A15034	199	A00288	A15225	477
A00288	A15499	262	A00288	A15500	581

A00288	A15660	420	A00288	A15684	477
A00288	A15732	302	A00288	A15751	283
A00288	A15931	163	A00288	A16272	390
A00288	A16637	63	A00288	A16658	455
A00288	A16686	279	A00288	A16724	268
A00288	A16839	188	A00288	A17140	260
A00289	A00335	306	A00289	A00355	159
A00289	A00357	187	A00289	A00403	219
A00289	A00409	174	A00289	A00560	396
A00289	A00605	179	A00289	A00629	221
A00289	A00651	170	A00289	A00700	219
A00289	A00705	396	A00289	A00839	462
A00289	A00923	171	A00289	A00958	201
A00289	A00991	232	A00289	A01017	160
A00289	A01025	328	A00289	A01196	188
A00289	A01206	204	A00289	A01291	193
A00289	A01306	422	A00289	A01335	484
A00289	A01468	171	A00289	A01470	205
A00289	A01579	407	A00289	A01612	174
A00289	A02830	154	A00289	A03569	210
A00289	A03740	234	A00289	A03754	224
A00289	A04988	283	A00289	A05450	277
A00289	A05501	329	A00289	A06466	160
A00289	A06814	218	A00289	A06965	268
A00289	A06973	157	A00289	A07581	235
A00289	A07604	307	A00289	A08137	154
A00289	A08154	279	A00289	A08301	246
A00289	A08359	488	A00289	A09322	201
A00289	A10490	254	A00289	A11291	249
A00289	A11550	263	A00289	A11861	319
A00289	A12821	369	A00289	A14006	154
A00289	A14257	343	A00289	A15244	286
A00289	A15367	183	A00289	A16118	202
A00290	A00481	193	A00290	A00570	425
A00290	A00629	425	A00290	A00781	187
A00290	A00865	425	A00290	A00928	425
A00290	A00952	425	A00290	A01101	425
A00290	A01159	425	A00290	A01221	425
A00290	A01412	425	A00290	A01774	296
A00290	A02121	299	A00290	A02439	232
A00290	A02456	317	A00290	A02654	257
A00290	A02655	429	A00290	A02803	174
A00290	A02983	174	A00290	A03922	160
A00290	A04446	201	A00290	A05577	491
A00290	A05679	232	A00290	A06047	63
A00290	A06369	425	A00290	A06832	425

A00290	A07159	452	A00290	A08057	484
A00290	A13821	475	A00290	A14071	210
A00290	A14391	205	A00290	A14596	478
A00290	A14659	187	A00290	A14741	217
A00291	A00799	173	A00291	A00940	227
A00291	A01143	152	A00291	A01576	165
A00291	A01717	172	A00291	A01876	274
A00291	A02438	406	A00291	A02439	414
A00291	A02460	200	A00291	A03171	165
A00291	A03974	491	A00291	A03975	329
A00291	A05517	313	A00291	A05679	346
A00291	A06177	168	A00291	A06415	490
A00291	A06598	256	A00291	A08293	470
A00291	A08356	187	A00291	A08362	224
A00291	A08821	322	A00291	A09829	446
A00291	A11291	266	A00291	A12858	213
A00291	A13928	173	A00291	A13983	483
A00291	A14071	489	A00291	A14199	160
A00291	A14220	154	A00291	A15314	63
A00292	A00356	274	A00292	A00619	483
A00292	A00868	63	A00292	A00914	351
A00292	A00960	152	A00292	A00999	311
A00292	A01492	467	A00292	A01524	216
A00292	A01527	238	A00292	A01604	183
A00292	A01640	400	A00292	A01818	127
A00292	A02151	299	A00292	A02170	139
A00292	A02250	490	A00292	A02278	285
A00292	A02333	489	A00292	A02361	265
A00292	A02414	243	A00292	A02415	319
A00292	A02416	191	A00292	A02425	338
A00292	A02490	489	A00292	A02491	491
A00292	A02581	232	A00292	A02592	101
A00292	A02624	156	A00292	A02729	154
A00292	A02785	168	A00292	A02841	361
A00292	A02901	196	A00292	A03009	456
A00292	A03029	160	A00292	A03053	162
A00292	A03061	267	A00292	A03110	208
A00292	A03185	219	A00292	A03438	218
A00292	A03506	195	A00292	A03771	307
A00292	A03937	262	A00292	A03949	484
A00292	A04327	323	A00292	A04398	491
A00292	A04422	441	A00292	A04490	249
A00292	A04638	274	A00292	A04826	286
A00292	A04990	232	A00292	A05082	202
A00292	A05443	268	A00292	A05462	156
A00292	A05709	188	A00292	A05759	278

A00292	A06065	402	A00292	A06450	297
A00292	A07531	171	A00292	A08473	286
A00292	A08714	457	A00292	A09184	297
A00292	A10288	274	A00292	A10376	267
A00292	A10419	241	A00292	A10422	201
A00292	A10766	208	A00292	A10790	205
A00292	A11064	344	A00292	A11123	208
A00292	A11369	445	A00292	A11525	104
A00292	A11693	389	A00292	A11995	252
A00292	A13052	118	A00292	A13339	213
A00292	A13607	259	A00292	A13989	479
A00292	A14053	340	A00292	A15498	177
A00292	A15789	241	A00292	A15913	411
A00292	A15928	201	A00292	A16636	475
A00292	A17090	340	A00293	A00556	121
A00293	A00876	198	A00293	A02902	486
A00293	A02951	208	A00293	A03286	394
A00293	A03752	160	A00293	A04015	283
A00293	A05811	480	A00293	A06466	454
A00293	A07311	268	A00293	A09021	353
A00293	A09577	177	A00293	A10313	344
A00293	A11921	302	A00293	A12328	162
A00293	A13340	372	A00293	A14197	198
A00294	A00305	406	A00294	A00309	491
A00294	A00316	260	A00294	A00324	945
A00294	A00330	234	A00294	A00370	491
A00294	A00388	475	A00294	A00470	439
A00294	A00552	283	A00294	A00597	160
A00294	A00643	491	A00294	A00669	427
A00294	A00671	332	A00294	A00696	238
A00294	A00703	481	A00294	A00747	491
A00294	A00754	477	A00294	A00763	422
A00294	A00786	899	A00294	A00799	329
A00294	A00809	486	A00294	A00853	224
A00294	A00871	473	A00294	A00887	490
A00294	A00914	160	A00294	A00981	360
A00294	A01052	491	A00294	A01070	914
A00294	A01091	291	A00294	A01157	483
A00294	A01168	486	A00294	A01172	491
A00294	A01307	193	A00294	A01378	172
A00294	A01470	419	A00294	A01507	232
A00294	A01524	201	A00294	A01527	939
A00294	A01548	491	A00294	A01554	899
A00294	A01557	251	A00294	A01558	899
A00294	A01560	899	A00294	A01562	159
A00294	A01587	329	A00294	A01588	899

A00294	A01592	899	A00294	A01602	491
A00294	A01615	899	A00294	A01638	899
A00294	A01640	406	A00294	A01660	157
A00294	A01668	408	A00294	A01676	933
A00294	A01688	238	A00294	A01702	396
A00294	A01723	435	A00294	A01741	490
A00294	A01743	401	A00294	A01763	461
A00294	A01790	454	A00294	A01798	180
A00294	A01839	443	A00294	A01844	462
A00294	A01850	484	A00294	A01886	243
A00294	A01983	63	A00294	A02000	435
A00294	A02018	480	A00294	A02021	435
A00294	A02049	76	A00294	A02059	185
A00294	A02121	229	A00294	A02178	419
A00294	A02180	413	A00294	A02183	207
A00294	A02191	63	A00294	A02211	154
A00294	A02219	63	A00294	A02239	286
A00294	A02261	168	A00294	A02329	371
A00294	A02361	363	A00294	A02495	224
A00294	A02502	366	A00294	A02562	263
A00294	A02567	213	A00294	A02607	168
A00294	A02687	480	A00294	A02839	174
A00294	A02842	396	A00294	A02902	279
A00294	A02965	432	A00294	A03026	338
A00294	A03067	246	A00294	A03070	63
A00294	A03080	249	A00294	A03090	441
A00294	A03112	221	A00294	A03213	414
A00294	A03271	515	A00294	A03304	486
A00294	A03373	238	A00294	A03390	154
A00294	A03391	479	A00294	A03401	347
A00294	A03415	151	A00294	A03436	303
A00294	A03451	375	A00294	A03532	174
A00294	A03575	902	A00294	A03725	491
A00294	A03821	160	A00294	A03977	201
A00294	A03990	171	A00294	A04020	314
A00294	A04080	480	A00294	A04104	363
A00294	A04334	324	A00294	A04448	191
A00294	A04449	450	A00294	A04659	201
A00294	A04842	352	A00294	A04927	260
A00294	A04996	154	A00294	A05141	491
A00294	A05185	477	A00294	A05216	467
A00294	A05260	174	A00294	A05365	316
A00294	A05566	200	A00294	A05629	213
A00294	A05797	439	A00294	A05873	207
A00294	A05914	267	A00294	A06078	367
A00294	A06146	191	A00294	A06196	179

A00294	A06201	241	A00294	A06256	168
A00294	A06455	179	A00294	A06463	286
A00294	A06581	484	A00294	A06597	223
A00294	A06609	899	A00294	A06610	491
A00294	A06615	290	A00294	A06642	334
A00294	A07158	399	A00294	A07201	899
A00294	A07209	257	A00294	A07218	419
A00294	A07339	477	A00294	A07803	414
A00294	A08022	486	A00294	A08836	193
A00294	A08839	899	A00294	A08858	899
A00294	A08875	491	A00294	A09044	274
A00294	A09124	303	A00294	A09141	491
A00294	A09235	156	A00294	A09399	160
A00294	A09542	340	A00294	A09561	490
A00294	A09727	491	A00294	A09791	491
A00294	A09826	480	A00294	A09941	154
A00294	A09997	256	A00294	A10114	195
A00294	A10251	207	A00294	A10267	260
A00294	A10283	229	A00294	A10304	263
A00294	A10412	263	A00294	A10754	388
A00294	A10808	156	A00294	A10913	232
A00294	A10934	369	A00294	A11003	356
A00294	A11115	229	A00294	A11286	433
A00294	A11369	899	A00294	A11600	191
A00294	A11650	160	A00294	A12371	188
A00294	A12417	191	A00294	A12513	207
A00294	A12897	486	A00294	A12992	899
A00294	A13027	207	A00294	A13089	168
A00294	A13346	205	A00294	A13388	157
A00294	A13560	160	A00294	A13589	241
A00294	A13655	191	A00294	A13997	369
A00294	A14009	486	A00294	A14016	433
A00294	A14029	899	A00294	A14157	229
A00294	A14200	491	A00294	A14494	328
A00294	A14596	457	A00294	A14636	475
A00294	A14643	439	A00294	A14787	188
A00294	A15292	899	A00294	A15293	899
A00294	A15294	899	A00294	A15661	219
A00294	A15926	188	A00294	A15948	395
A00294	A16157	485	A00294	A16203	179
A00294	A16299	313	A00294	A16300	347
A00294	A16833	168	A00294	A16911	491
A00295	A00318	452	A00295	A00472	479
A00295	A00553	288	A00295	A00636	162
A00295	A00694	475	A00295	A00870	356
A00295	A00882	441	A00295	A00902	306

A00295	A01011	356	A00295	A01161	305
A00295	A01216	429	A00295	A01235	340
A00295	A01276	374	A00295	A01345	170
A00295	A01478	301	A00295	A01543	480
A00295	A01551	373	A00295	A01557	355
A00295	A01566	491	A00295	A01578	488
A00295	A01595	460	A00295	A01607	488
A00295	A01632	490	A00295	A01652	160
A00295	A01746	160	A00295	A01967	195
A00295	A02099	185	A00295	A02167	162
A00295	A02188	210	A00295	A02221	185
A00295	A02502	259	A00295	A02642	172
A00295	A02691	183	A00295	A02979	483
A00295	A03104	452	A00295	A03451	490
A00295	A03471	491	A00295	A03625	350
A00295	A03806	159	A00295	A04006	291
A00295	A04175	480	A00295	A04275	441
A00295	A04305	204	A00295	A04349	183
A00295	A04361	172	A00295	A04715	491
A00295	A04870	263	A00295	A05073	172
A00295	A05300	420	A00295	A05370	241
A00295	A05439	198	A00295	A05558	241
A00295	A05574	249	A00295	A06100	490
A00295	A06234	235	A00295	A06566	301
A00295	A06651	185	A00295	A06701	180
A00295	A06708	335	A00295	A06739	447
A00295	A07132	156	A00295	A07209	439
A00295	A07282	168	A00295	A07815	489
A00295	A07903	432	A00295	A07984	301
A00295	A08172	173	A00295	A08309	345
A00295	A08578	163	A00295	A08658	213
A00295	A08685	156	A00295	A08686	198
A00295	A08708	270	A00295	A08859	159
A00295	A09384	328	A00295	A09511	165
A00295	A09734	324	A00295	A09886	301
A00295	A10445	191	A00295	A10630	198
A00295	A11036	259	A00295	A11914	416
A00295	A11921	236	A00295	A11983	435
A00295	A12853	260	A00295	A13369	430
A00295	A13806	232	A00295	A13824	236
A00295	A14202	252	A00295	A14210	154
A00295	A14330	159	A00295	A14570	340
A00295	A14635	420	A00295	A14733	152
A00295	A14757	205	A00295	A15181	235
A00295	A15314	279	A00295	A15322	246
A00295	A15385	168	A00295	A15578	419

A00295	A15793	201	A00295	A16074	208
A00295	A16081	185	A00295	A16274	484
A00295	A16534	235	A00295	A17009	205
A00296	A00297	694	A00296	A00308	241
A00296	A00323	385	A00296	A00339	676
A00296	A00348	441	A00296	A00367	695
A00296	A00368	694	A00296	A00369	491
A00296	A00373	470	A00296	A00421	695
A00296	A00496	201	A00296	A00531	694
A00296	A00559	491	A00296	A00560	486
A00296	A00646	124	A00296	A00677	306
A00296	A00691	491	A00296	A00819	249
A00296	A00822	491	A00296	A00831	326
A00296	A00883	393	A00296	A00905	480
A00296	A00916	491	A00296	A00932	210
A00296	A01139	63	A00296	A01150	191
A00296	A01151	63	A00296	A01231	263
A00296	A01243	162	A00296	A01265	219
A00296	A01266	200	A00296	A01268	452
A00296	A01354	267	A00296	A01359	63
A00296	A01372	383	A00296	A01558	486
A00296	A01588	335	A00296	A01598	339
A00296	A01612	491	A00296	A01615	369
A00296	A01638	460	A00296	A01649	417
A00296	A01707	323	A00296	A02120	167
A00296	A03298	491	A00296	A03494	646
A00296	A03569	207	A00296	A03740	446
A00296	A03752	355	A00296	A04268	179
A00296	A06466	477	A00296	A06907	183
A00296	A07311	489	A00296	A07834	243
A00296	A08137	491	A00296	A08280	174
A00296	A08357	226	A00296	A08359	180
A00296	A08524	383	A00296	A08528	247
A00296	A08571	491	A00296	A08818	474
A00296	A08885	491	A00296	A09011	210
A00296	A09256	388	A00296	A09502	152
A00296	A09506	160	A00296	A09646	173
A00296	A10250	188	A00296	A10615	479
A00296	A10624	490	A00296	A10853	468
A00296	A10894	171	A00296	A11105	458
A00296	A11139	695	A00296	A11291	176
A00296	A11302	227	A00296	A11372	227
A00296	A11409	170	A00296	A11420	193
A00296	A11920	491	A00296	A11923	461
A00296	A11928	491	A00296	A12238	173
A00296	A12251	154	A00296	A12328	366

A00296	A12416	402	A00296	A12861	353
A00296	A13147	419	A00296	A13310	491
A00296	A13311	491	A00296	A13805	438
A00296	A13989	462	A00296	A14129	446
A00296	A14220	154	A00296	A14257	440
A00296	A14476	491	A00296	A14596	322
A00296	A14899	363	A00296	A15366	236
A00296	A15660	363	A00296	A15711	661
A00296	A15766	299	A00296	A16490	221
A00296	A16491	477	A00296	A16568	353
A00296	A16690	383	A00296	A16866	383
A00296	A16911	196	A00296	A16912	486
A00297	A00339	637	A00297	A00367	504
A00297	A00368	637	A00297	A00373	419
A00297	A00421	554	A00297	A00501	221
A00297	A00531	661	A00297	A00677	162
A00297	A00691	430	A00297	A00856	322
A00297	A00905	475	A00297	A01266	281
A00297	A01372	383	A00297	A01430	168
A00297	A01431	238	A00297	A01478	196
A00297	A01495	163	A00297	A01558	208
A00297	A01615	200	A00297	A01651	174
A00297	A03298	484	A00297	A03494	543
A00297	A07311	165	A00297	A07348	183
A00297	A07834	177	A00297	A08022	307
A00297	A08137	251	A00297	A08524	383
A00297	A08571	491	A00297	A09902	218
A00297	A10250	218	A00297	A10615	154
A00297	A10894	196	A00297	A11105	483
A00297	A11139	588	A00297	A13311	193
A00297	A13510	151	A00297	A13805	438
A00297	A13989	469	A00297	A14476	479
A00297	A15711	438	A00297	A16490	333
A00297	A16690	383	A00297	A16866	383
A00298	A00343	491	A00298	A00417	160
A00298	A00453	187	A00298	A00458	412
A00298	A00532	314	A00298	A00537	196
A00298	A00604	491	A00298	A00653	491
A00298	A00669	366	A00298	A00691	407
A00298	A00783	491	A00298	A00785	491
A00298	A00790	396	A00298	A00953	251
A00298	A01019	208	A00298	A01027	460
A00298	A01042	435	A00298	A01054	446
A00298	A01075	152	A00298	A01087	373
A00298	A01161	156	A00298	A01268	229
A00298	A01399	241	A00298	A01446	374

A00298	A01448	227	A00298	A01457	174
A00298	A01540	338	A00298	A01574	446
A00298	A01576	343	A00298	A01837	255
A00298	A02180	176	A00298	A02607	205
A00298	A03070	491	A00298	A03293	481
A00298	A03298	330	A00298	A03740	477
A00298	A03752	246	A00298	A03776	263
A00298	A04630	259	A00298	A04686	201
A00298	A05387	347	A00298	A06434	182
A00298	A06466	267	A00298	A07086	404
A00298	A07706	417	A00298	A07732	177
A00298	A08461	234	A00298	A08571	219
A00298	A08835	174	A00298	A10703	163
A00298	A10797	419	A00298	A10798	474
A00298	A11244	279	A00298	A11322	296
A00298	A11337	470	A00298	A11338	419
A00298	A11374	424	A00298	A11427	321
A00298	A11612	246	A00298	A12241	491
A00298	A13340	205	A00298	A14461	362
A00298	A14471	188	A00298	A16462	154
A00298	A17128	484	A00298	A17205	63
A00299	A00310	167	A00299	A00312	433
A00299	A00314	473	A00299	A00325	490
A00299	A00328	486	A00299	A00355	565
A00299	A00356	485	A00299	A00357	490
A00299	A00358	483	A00299	A00403	491
A00299	A00405	433	A00299	A00444	241
A00299	A00468	413	A00299	A00521	565
A00299	A00590	486	A00299	A00603	486
A00299	A00605	456	A00299	A00629	491
A00299	A00648	490	A00299	A00651	472
A00299	A00653	457	A00299	A00660	486
A00299	A00701	480	A00299	A00763	491
A00299	A00783	182	A00299	A00787	491
A00299	A00827	230	A00299	A00839	488
A00299	A00870	246	A00299	A00878	473
A00299	A00923	491	A00299	A00965	317
A00299	A00988	270	A00299	A00990	307
A00299	A01000	491	A00299	A01017	307
A00299	A01025	491	A00299	A01034	314
A00299	A01047	566	A00299	A01054	307
A00299	A01056	468	A00299	A01066	200
A00299	A01067	491	A00299	A01095	154
A00299	A01101	490	A00299	A01112	475
A00299	A01127	488	A00299	A01140	171
A00299	A01166	228	A00299	A01184	485

A00299	A01189	491	A00299	A01206	466
A00299	A01210	428	A00299	A01211	159
A00299	A01234	465	A00299	A01249	566
A00299	A01264	200	A00299	A01282	485
A00299	A01291	566	A00299	A01292	218
A00299	A01294	333	A00299	A01306	491
A00299	A01338	552	A00299	A01392	491
A00299	A01429	200	A00299	A01436	162
A00299	A01437	483	A00299	A01457	475
A00299	A01458	491	A00299	A01464	526
A00299	A01468	177	A00299	A01470	198
A00299	A01490	177	A00299	A01496	314
A00299	A01540	240	A00299	A01579	463
A00299	A01617	167	A00299	A01618	200
A00299	A01620	213	A00299	A01649	480
A00299	A01700	182	A00299	A01718	270
A00299	A02114	168	A00299	A02178	184
A00299	A02208	352	A00299	A02216	417
A00299	A02254	491	A00299	A02257	378
A00299	A02284	438	A00299	A02286	154
A00299	A02738	463	A00299	A02978	253
A00299	A03069	486	A00299	A03087	490
A00299	A03099	185	A00299	A03174	208
A00299	A03223	515	A00299	A03460	254
A00299	A03567	491	A00299	A03644	313
A00299	A03645	246	A00299	A03705	245
A00299	A03775	347	A00299	A03968	335
A00299	A04781	196	A00299	A05208	430
A00299	A05224	230	A00299	A05330	369
A00299	A05331	246	A00299	A05338	200
A00299	A05411	469	A00299	A05501	246
A00299	A06252	179	A00299	A06279	332
A00299	A06861	460	A00299	A06909	254
A00299	A06965	491	A00299	A06973	378
A00299	A07104	235	A00299	A07105	241
A00299	A07201	312	A00299	A07243	235
A00299	A07349	193	A00299	A07449	156
A00299	A07451	480	A00299	A07464	213
A00299	A07520	441	A00299	A08011	433
A00299	A08055	240	A00299	A08135	382
A00299	A08197	305	A00299	A08357	372
A00299	A08359	283	A00299	A08398	558
A00299	A08403	388	A00299	A08419	174
A00299	A08452	565	A00299	A08521	322
A00299	A08558	200	A00299	A08559	466
A00299	A08664	474	A00299	A08760	160

A00299	A08773	417	A00299	A08972	167
A00299	A09044	465	A00299	A09322	491
A00299	A09442	208	A00299	A09674	308
A00299	A09702	271	A00299	A09991	172
A00299	A10006	173	A00299	A10034	441
A00299	A10417	329	A00299	A10518	216
A00299	A10564	226	A00299	A10616	314
A00299	A10679	307	A00299	A11322	168
A00299	A11390	429	A00299	A11400	291
A00299	A11438	566	A00299	A11480	183
A00299	A11729	200	A00299	A11945	226
A00299	A11981	251	A00299	A11992	202
A00299	A12168	254	A00299	A12177	281
A00299	A12233	299	A00299	A12661	168
A00299	A12687	389	A00299	A12948	230
A00299	A13155	270	A00299	A13249	358
A00299	A13333	200	A00299	A13362	414
A00299	A13930	165	A00299	A13989	457
A00299	A13992	457	A00299	A14188	303
A00299	A14216	401	A00299	A15218	477
A00299	A15221	400	A00299	A15235	491
A00299	A15418	160	A00299	A15743	465
A00299	A15776	173	A00299	A15932	180
A00299	A15988	288	A00299	A16118	461
A00299	A17193	201	A00300	A00476	208
A00300	A00621	486	A00300	A01605	368
A00300	A01631	432	A00300	A01639	274
A00300	A01640	238	A00300	A01690	191
A00300	A01697	208	A00300	A01719	196
A00300	A01755	268	A00300	A01789	279
A00300	A01908	187	A00300	A02786	307
A00300	A02788	193	A00300	A02878	180
A00300	A03069	195	A00300	A03100	265
A00300	A03861	270	A00300	A04073	223
A00300	A06000	454	A00300	A06701	159
A00300	A10534	433	A00300	A14148	265
A00300	A14827	165	A00301	A00338	329
A00301	A00377	226	A00301	A00438	355
A00301	A00472	283	A00301	A00485	483
A00301	A00576	386	A00301	A00577	205
A00301	A00609	480	A00301	A00650	213
A00301	A00717	152	A00301	A00719	326
A00301	A00802	424	A00301	A00861	151
A00301	A00883	182	A00301	A00939	177
A00301	A01004	202	A00301	A01022	473
A00301	A01026	402	A00301	A01027	182

A00301	A01049	486	A00301	A01091	301
A00301	A01156	382	A00301	A01161	488
A00301	A01211	488	A00301	A01326	245
A00301	A01387	176	A00301	A01481	480
A00301	A01983	452	A00301	A02071	184
A00301	A02147	272	A00301	A02187	246
A00301	A02221	318	A00301	A02222	157
A00301	A02289	243	A00301	A02326	243
A00301	A02341	200	A00301	A02383	229
A00301	A02692	193	A00301	A02706	307
A00301	A02761	174	A00301	A02764	274
A00301	A02795	238	A00301	A02817	188
A00301	A02818	224	A00301	A02838	168
A00301	A03095	291	A00301	A03304	205
A00301	A03417	299	A00301	A03430	323
A00301	A03440	293	A00301	A03945	208
A00301	A03989	277	A00301	A04163	198
A00301	A04275	252	A00301	A04302	272
A00301	A04337	398	A00301	A04449	213
A00301	A04488	299	A00301	A04671	212
A00301	A04773	159	A00301	A04834	232
A00301	A04995	205	A00301	A05020	200
A00301	A05065	174	A00301	A05087	241
A00301	A05106	160	A00301	A05356	180
A00301	A05472	170	A00301	A05493	274
A00301	A05494	172	A00301	A05775	200
A00301	A06277	391	A00301	A06294	379
A00301	A06404	269	A00301	A06756	185
A00301	A06779	285	A00301	A06818	254
A00301	A06877	252	A00301	A06991	252
A00301	A07030	347	A00301	A07106	385
A00301	A07151	183	A00301	A07197	348
A00301	A07366	154	A00301	A07390	241
A00301	A07424	279	A00301	A08093	357
A00301	A08936	413	A00301	A09054	414
A00301	A09416	259	A00301	A09750	168
A00301	A09857	162	A00301	A09971	329
A00301	A10018	154	A00301	A10156	307
A00301	A10547	385	A00301	A10621	168
A00301	A10713	182	A00301	A10731	174
A00301	A10778	434	A00301	A11307	328
A00301	A11313	152	A00301	A11317	252
A00301	A11469	183	A00301	A11553	265
A00301	A11590	388	A00301	A11628	246
A00301	A11897	329	A00301	A12035	196
A00301	A12361	156	A00301	A12371	246

A00301	A12605	323	A00301	A13331	329
A00301	A13874	436	A00301	A14190	160
A00301	A14196	486	A00301	A14272	480
A00301	A14381	274	A00301	A14405	288
A00301	A14524	201	A00301	A14589	400
A00301	A14643	185	A00301	A15052	378
A00301	A15053	347	A00301	A15229	272
A00301	A15282	165	A00301	A15292	374
A00301	A15498	279	A00301	A15596	490
A00301	A15765	190	A00301	A15839	243
A00301	A15867	285	A00301	A16121	217
A00301	A16463	191	A00301	A16905	188
A00302	A00433	477	A00302	A00469	201
A00302	A00626	483	A00302	A00655	174
A00302	A00833	382	A00302	A00932	187
A00302	A09471	445	A00302	A09695	430
A00302	A12042	368	A00302	A12503	480
A00303	A00308	428	A00303	A00326	184
A00303	A00822	205	A00303	A00828	252
A00303	A00958	183	A00303	A01088	154
A00303	A01299	246	A00303	A01598	208
A00303	A02000	196	A00303	A03076	162
A00303	A03414	195	A00303	A03720	195
A00303	A03854	151	A00303	A05822	327
A00303	A06443	430	A00303	A06528	401
A00303	A08859	273	A00303	A11300	274
A00303	A11576	198	A00303	A14196	270
A00303	A14660	224	A00304	A00322	515
A00304	A00335	468	A00304	A00340	485
A00304	A00356	484	A00304	A00366	485
A00304	A00411	285	A00304	A00414	382
A00304	A00467	441	A00304	A00643	228
A00304	A00648	420	A00304	A00651	470
A00304	A00700	369	A00304	A00701	238
A00304	A00713	407	A00304	A00839	489
A00304	A00856	485	A00304	A00911	270
A00304	A00966	491	A00304	A00983	385
A00304	A01056	491	A00304	A01085	171
A00304	A01090	490	A00304	A01112	491
A00304	A01140	491	A00304	A01211	274
A00304	A01212	457	A00304	A01261	396
A00304	A01270	465	A00304	A01288	305
A00304	A01291	491	A00304	A01296	515
A00304	A01306	254	A00304	A01320	485
A00304	A01335	330	A00304	A01411	182
A00304	A01447	243	A00304	A01468	396

A00304	A01527	205	A00304	A01558	302
A00304	A01579	491	A00304	A01586	369
A00304	A01601	592	A00304	A01723	232
A00304	A01787	334	A00304	A01995	491
A00304	A02179	490	A00304	A02494	450
A00304	A02924	278	A00304	A02985	173
A00304	A03048	196	A00304	A03070	466
A00304	A03272	202	A00304	A03304	480
A00304	A03461	441	A00304	A03571	335
A00304	A03981	491	A00304	A04988	486
A00304	A05298	232	A00304	A05418	486
A00304	A05525	177	A00304	A06121	449
A00304	A06144	183	A00304	A06328	302
A00304	A06466	473	A00304	A06534	404
A00304	A06580	232	A00304	A06814	491
A00304	A06973	168	A00304	A07245	168
A00304	A07307	226	A00304	A07311	486
A00304	A07398	480	A00304	A07536	265
A00304	A07658	160	A00304	A07719	297
A00304	A07726	719	A00304	A07797	297
A00304	A08131	170	A00304	A08359	474
A00304	A08369	480	A00304	A08457	154
A00304	A08476	364	A00304	A08835	480
A00304	A08869	369	A00304	A08983	340
A00304	A09246	159	A00304	A09308	372
A00304	A09704	219	A00304	A10532	435
A00304	A10569	219	A00304	A10633	335
A00304	A10711	486	A00304	A10748	472
A00304	A11334	364	A00304	A11400	296
A00304	A11487	411	A00304	A11562	319
A00304	A11688	299	A00304	A11728	465
A00304	A11983	310	A00304	A12084	201
A00304	A12168	407	A00304	A12269	362
A00304	A12270	205	A00304	A12618	491
A00304	A13063	223	A00304	A13340	477
A00304	A13621	297	A00304	A13989	491
A00304	A14055	240	A00304	A14176	383
A00304	A14188	288	A00304	A14205	188
A00304	A14498	270	A00304	A14908	286
A00304	A14937	204	A00304	A15047	297
A00304	A15506	341	A00304	A15551	160
A00304	A15711	190	A00304	A16682	241
A00304	A16793	174	A00304	A16839	430
A00305	A00309	380	A00305	A00851	433
A00305	A01483	422	A00305	A01666	173
A00305	A01741	489	A00305	A01743	301

A00305	A01939	252	A00305	A02212	217
A00305	A02294	291	A00305	A02349	185
A00305	A02352	491	A00305	A02407	170
A00305	A02488	265	A00305	A02491	171
A00305	A02568	160	A00305	A02599	238
A00305	A02699	174	A00305	A02744	213
A00305	A02749	462	A00305	A02763	210
A00305	A02784	470	A00305	A02792	316
A00305	A02811	188	A00305	A02817	154
A00305	A02833	188	A00305	A02836	488
A00305	A02869	243	A00305	A02874	219
A00305	A02875	355	A00305	A03070	215
A00305	A03102	207	A00305	A03112	379
A00305	A03259	180	A00305	A03322	176
A00305	A03417	262	A00305	A03486	174
A00305	A03515	172	A00305	A03609	491
A00305	A03634	313	A00305	A03661	157
A00305	A03923	193	A00305	A03977	223
A00305	A04009	230	A00305	A04049	235
A00305	A04092	157	A00305	A04243	313
A00305	A04256	151	A00305	A04334	335
A00305	A04429	361	A00305	A04444	286
A00305	A04445	210	A00305	A04548	165
A00305	A04736	274	A00305	A04737	491
A00305	A04743	259	A00305	A04770	174
A00305	A04795	278	A00305	A04842	152
A00305	A04941	195	A00305	A04988	182
A00305	A05122	489	A00305	A05141	156
A00305	A05185	196	A00305	A05353	489
A00305	A05398	185	A00305	A05701	119
A00305	A05741	234	A00305	A06059	191
A00305	A06376	340	A00305	A06581	371
A00305	A07128	185	A00305	A07210	180
A00305	A07339	281	A00305	A08070	221
A00305	A08686	180	A00305	A08753	157
A00305	A09011	266	A00305	A09028	312
A00305	A09170	340	A00305	A09265	238
A00305	A09431	259	A00305	A09448	163
A00305	A09504	201	A00305	A09545	266
A00305	A09556	367	A00305	A09559	279
A00305	A09671	167	A00305	A09928	218
A00305	A10156	363	A00305	A11333	373
A00305	A11513	254	A00305	A12373	204
A00305	A13348	195	A00305	A13665	160
A00305	A13755	340	A00305	A13886	219
A00305	A14589	488	A00305	A14639	223

A00305	A14643	208	A00305	A16281	157
A00305	A16295	326	A00305	A16371	163
A00305	A16531	337	A00305	A16877	187
A00305	A17040	435	A00306	A00421	156
A00306	A00496	252	A00306	A00531	156
A00306	A00583	156	A00306	A00646	156
A00306	A00691	156	A00306	A00716	156
A00306	A00736	491	A00306	A00836	251
A00306	A00847	491	A00306	A00867	156
A00306	A00879	156	A00306	A00905	156
A00306	A00957	236	A00306	A01029	156
A00306	A01136	156	A00306	A01151	156
A00306	A01259	263	A00306	A01266	156
A00306	A01268	156	A00306	A01295	156
A00306	A01334	156	A00306	A01391	156
A00306	A01434	156	A00306	A01478	165
A00306	A01576	286	A00306	A02737	185
A00306	A02833	191	A00306	A02860	165
A00306	A03494	156	A00306	A03742	260
A00306	A05029	408	A00306	A06907	266
A00306	A07898	156	A00306	A08201	156
A00306	A08327	156	A00306	A08391	491
A00306	A08431	156	A00306	A08542	156
A00306	A08745	174	A00306	A09311	200
A00306	A09785	340	A00306	A10460	485
A00306	A11291	489	A00306	A11496	156
A00306	A11497	156	A00306	A11727	182
A00306	A12228	475	A00306	A12229	467
A00306	A12492	193	A00306	A12501	490
A00306	A12757	156	A00306	A13928	446
A00306	A14178	156	A00306	A14196	228
A00306	A14199	241	A00306	A14220	193
A00306	A14836	391	A00306	A15291	246
A00306	A15492	162	A00306	A16497	467
A00306	A16699	156	A00306	A17198	173
A00307	A00571	227	A00307	A00740	393
A00307	A00782	319	A00307	A00839	215
A00307	A00955	361	A00307	A01217	346
A00307	A01221	310	A00307	A01335	283
A00307	A01365	313	A00307	A01406	283
A00307	A02227	283	A00307	A06564	385
A00307	A09735	361	A00307	A10338	452
A00307	A14568	373	A00308	A00326	491
A00308	A00348	491	A00308	A00366	491
A00308	A00382	245	A00308	A00436	196
A00308	A00440	393	A00308	A00448	224

A00308	A00453	480	A00308	A00531	351
A00308	A00560	329	A00308	A00589	433
A00308	A00609	402	A00308	A00822	491
A00308	A00831	489	A00308	A00835	335
A00308	A00837	270	A00308	A00848	272
A00308	A00883	491	A00308	A00905	490
A00308	A00916	488	A00308	A00954	218
A00308	A00958	491	A00308	A01026	170
A00308	A01029	477	A00308	A01046	449
A00308	A01052	368	A00308	A01075	489
A00308	A01077	291	A00308	A01080	162
A00308	A01088	486	A00308	A01096	480
A00308	A01097	299	A00308	A01136	378
A00308	A01143	477	A00308	A01194	270
A00308	A01206	307	A00308	A01234	466
A00308	A01243	210	A00308	A01266	188
A00308	A01268	447	A00308	A01274	475
A00308	A01291	473	A00308	A01295	491
A00308	A01299	491	A00308	A01305	491
A00308	A01354	491	A00308	A01378	252
A00308	A01441	491	A00308	A01470	416
A00308	A01507	187	A00308	A01512	157
A00308	A01598	491	A00308	A01612	475
A00308	A01614	174	A00308	A01649	424
A00308	A02064	297	A00308	A02830	486
A00308	A02835	159	A00308	A02902	460
A00308	A02931	484	A00308	A03112	196
A00308	A03304	293	A00308	A03406	440
A00308	A03591	305	A00308	A04076	172
A00308	A04900	408	A00308	A05288	486
A00308	A05295	154	A00308	A05709	160
A00308	A06143	246	A00308	A06466	452
A00308	A07311	434	A00308	A07604	156
A00308	A07625	190	A00308	A07706	201
A00308	A08078	152	A00308	A08118	456
A00308	A08137	488	A00308	A08280	425
A00308	A08571	457	A00308	A09705	180
A00308	A09768	238	A00308	A09857	355
A00308	A10321	305	A00308	A10532	183
A00308	A10624	340	A00308	A11291	355
A00308	A11300	491	A00308	A11324	165
A00308	A11387	491	A00308	A11496	252
A00308	A11497	235	A00308	A11649	355
A00308	A12152	168	A00308	A12189	251
A00308	A12328	238	A00308	A12365	375
A00308	A12373	168	A00308	A12757	227

A00308	A13340	446	A00308	A13376	457
A00308	A13928	460	A00308	A13989	489
A00308	A14006	486	A00308	A14196	223
A00308	A14220	484	A00308	A14257	414
A00308	A14310	162	A00308	A14408	339
A00308	A14596	439	A00308	A14659	230
A00308	A14660	491	A00308	A15296	428
A00308	A15366	307	A00308	A15504	172
A00308	A15520	184	A00308	A15559	418
A00308	A15596	269	A00308	A15747	184
A00308	A15749	182	A00308	A15752	369
A00308	A16324	348	A00308	A16700	168
A00308	A16798	491	A00308	A17005	274
A00308	A17063	157	A00309	A00324	218
A00309	A00370	489	A00309	A00597	249
A00309	A00643	491	A00309	A00671	227
A00309	A00736	462	A00309	A00747	491
A00309	A00879	491	A00309	A00887	185
A00309	A00914	260	A00309	A01029	491
A00309	A01052	281	A00309	A01104	229
A00309	A01114	172	A00309	A01125	420
A00309	A01157	303	A00309	A01209	378
A00309	A01483	255	A00309	A01527	932
A00309	A01553	221	A00309	A01558	899
A00309	A01588	899	A00309	A01602	455
A00309	A01605	274	A00309	A01640	177
A00309	A01666	174	A00309	A01668	414
A00309	A01676	452	A00309	A01702	205
A00309	A01741	491	A00309	A01743	489
A00309	A01790	156	A00309	A01844	249
A00309	A01850	154	A00309	A01886	286
A00309	A01931	160	A00309	A01990	152
A00309	A02018	279	A00309	A02020	486
A00309	A02178	339	A00309	A02180	317
A00309	A02215	424	A00309	A02261	312
A00309	A02278	154	A00309	A02349	191
A00309	A02481	281	A00309	A02491	223
A00309	A02492	425	A00309	A02502	263
A00309	A02510	152	A00309	A02562	488
A00309	A02564	229	A00309	A02567	251
A00309	A02593	162	A00309	A02676	267
A00309	A02754	400	A00309	A02784	368
A00309	A02796	243	A00309	A02816	210
A00309	A02817	460	A00309	A02823	221
A00309	A02836	154	A00309	A02837	343
A00309	A02842	489	A00309	A02868	297

A00309	A02890	221	A00309	A02902	351
A00309	A03026	235	A00309	A03070	160
A00309	A03080	468	A00309	A03095	227
A00309	A03112	412	A00309	A03126	332
A00309	A03188	152	A00309	A03213	358
A00309	A03304	413	A00309	A03391	413
A00309	A03415	462	A00309	A03421	208
A00309	A03451	310	A00309	A03681	319
A00309	A03719	246	A00309	A03725	491
A00309	A03976	361	A00309	A03977	241
A00309	A04080	491	A00309	A04334	465
A00309	A04348	171	A00309	A04420	152
A00309	A04421	199	A00309	A04448	221
A00309	A04453	243	A00309	A04576	184
A00309	A04621	162	A00309	A04638	208
A00309	A04645	208	A00309	A04739	205
A00309	A04744	177	A00309	A04836	162
A00309	A04988	291	A00309	A05141	491
A00309	A05161	165	A00309	A05179	221
A00309	A05185	475	A00309	A05260	224
A00309	A05270	235	A00309	A05365	475
A00309	A05418	491	A00309	A05491	305
A00309	A05525	385	A00309	A05629	485
A00309	A05767	177	A00309	A05868	204
A00309	A06063	385	A00309	A06078	329
A00309	A06146	221	A00309	A06189	330
A00309	A06196	208	A00309	A06201	463
A00309	A06256	199	A00309	A06581	488
A00309	A06586	283	A00309	A06601	200
A00309	A06617	221	A00309	A06683	199
A00309	A06813	340	A00309	A07158	457
A00309	A07245	157	A00309	A07339	407
A00309	A07420	221	A00309	A07935	374
A00309	A08327	187	A00309	A08836	252
A00309	A08875	490	A00309	A09028	182
A00309	A09073	330	A00309	A09081	191
A00309	A09136	184	A00309	A09141	430
A00309	A09147	165	A00309	A09265	356
A00309	A09271	411	A00309	A09431	152
A00309	A09524	165	A00309	A09561	491
A00309	A09791	490	A00309	A09882	162
A00309	A10069	199	A00309	A10267	162
A00309	A10283	257	A00309	A10337	212
A00309	A10393	288	A00309	A10809	162
A00309	A10838	361	A00309	A10913	270
A00309	A10958	288	A00309	A11003	375

A00309	A11044	286	A00309	A11114	221
A00309	A11259	235	A00309	A11369	899
A00309	A11650	191	A00309	A12019	235
A00309	A12897	446	A00309	A13373	238
A00309	A13589	361	A00309	A13667	172
A00309	A13791	375	A00309	A13851	177
A00309	A13877	165	A00309	A13938	177
A00309	A14029	899	A00309	A14157	257
A00309	A14494	413	A00309	A14643	465
A00309	A14650	238	A00309	A14670	171
A00309	A14680	288	A00309	A14787	329
A00309	A15917	167	A00309	A15926	393
A00309	A16041	350	A00309	A16300	486
A00309	A16911	491	A00310	A00312	491
A00310	A00314	481	A00310	A00395	265
A00310	A00449	294	A00310	A00468	374
A00310	A00579	162	A00310	A00600	151
A00310	A00601	278	A00310	A00660	477
A00310	A00752	313	A00310	A00825	380
A00310	A00842	205	A00310	A01005	286
A00310	A01137	200	A00310	A01164	201
A00310	A01212	154	A00310	A01397	281
A00310	A01409	246	A00310	A01462	180
A00310	A01463	191	A00310	A01466	218
A00310	A01476	279	A00310	A01490	416
A00310	A01508	152	A00310	A01511	258
A00310	A01527	899	A00310	A01543	488
A00310	A01590	470	A00310	A01612	236
A00310	A01635	346	A00310	A01726	238
A00310	A01926	163	A00310	A01978	270
A00310	A02008	246	A00310	A02014	272
A00310	A02054	345	A00310	A02074	152
A00310	A02083	474	A00310	A02104	177
A00310	A02114	322	A00310	A02141	152
A00310	A02254	429	A00310	A02257	490
A00310	A02258	369	A00310	A02259	311
A00310	A02284	486	A00310	A02306	340
A00310	A02329	204	A00310	A02330	945
A00310	A02363	162	A00310	A02366	226
A00310	A02468	174	A00310	A02492	278
A00310	A02522	149	A00310	A02574	263
A00310	A02601	65	A00310	A03014	409
A00310	A03019	174	A00310	A03034	198
A00310	A03084	130	A00310	A03087	486
A00310	A03099	451	A00310	A03146	277
A00310	A03148	238	A00310	A03167	335

A00310	A03168	187	A00310	A03174	386
A00310	A03199	389	A00310	A03207	174
A00310	A03281	205	A00310	A03402	176
A00310	A03520	187	A00310	A03961	317
A00310	A04160	218	A00310	A04421	419
A00310	A04471	167	A00310	A04487	217
A00310	A04781	159	A00310	A04990	188
A00310	A05007	159	A00310	A05224	327
A00310	A05330	232	A00310	A05333	210
A00310	A05337	223	A00310	A05356	491
A00310	A05376	188	A00310	A05393	195
A00310	A05424	457	A00310	A05429	311
A00310	A05452	199	A00310	A05462	236
A00310	A05550	188	A00310	A05740	311
A00310	A05772	170	A00310	A05820	246
A00310	A05893	311	A00310	A06037	484
A00310	A06092	208	A00310	A06421	210
A00310	A06473	168	A00310	A06520	200
A00310	A06523	185	A00310	A06607	185
A00310	A06626	199	A00310	A06632	199
A00310	A06668	185	A00310	A06769	462
A00310	A06874	174	A00310	A06949	160
A00310	A06970	185	A00310	A06994	162
A00310	A07031	236	A00310	A07334	218
A00310	A07407	224	A00310	A07486	185
A00310	A07656	481	A00310	A07763	105
A00310	A07795	235	A00310	A07796	156
A00310	A07851	157	A00310	A08473	246
A00310	A08571	188	A00310	A08960	167
A00310	A09185	262	A00310	A09514	226
A00310	A10159	414	A00310	A10286	402
A00310	A10288	235	A00310	A10358	201
A00310	A10428	262	A00310	A10701	479
A00310	A11393	185	A00310	A11783	218
A00310	A13155	235	A00310	A13364	424
A00310	A13666	311	A00310	A13726	240
A00310	A14196	207	A00310	A14443	899
A00310	A14531	162	A00310	A14570	366
A00310	A14821	174	A00310	A14878	205
A00310	A15682	366	A00310	A15684	260
A00310	A15874	193	A00310	A16028	255
A00310	A16375	262	A00310	A17078	352
A00311	A00736	432	A00311	A01590	481
A00311	A01766	168	A00311	A01923	491
A00311	A01924	443	A00311	A01926	322
A00311	A02074	191	A00311	A02077	249

A00311	A02083	486	A00311	A02145	267
A00311	A02305	491	A00311	A02306	338
A00311	A02330	152	A00311	A02405	201
A00311	A02464	213	A00311	A02667	491
A00311	A02749	204	A00311	A02756	265
A00311	A02859	229	A00311	A03402	368
A00311	A03520	229	A00311	A03599	328
A00311	A04002	340	A00311	A04164	337
A00311	A04368	477	A00311	A04437	491
A00311	A04608	167	A00311	A04633	265
A00311	A04746	260	A00311	A04770	236
A00311	A04883	405	A00311	A04902	165
A00311	A05153	246	A00311	A05376	218
A00311	A05390	469	A00311	A05957	422
A00311	A06263	230	A00311	A06607	224
A00311	A06843	308	A00311	A07407	246
A00311	A07468	475	A00311	A07541	241
A00311	A07617	290	A00311	A07958	257
A00311	A09072	202	A00311	A10967	480
A00311	A12793	404	A00311	A14215	208
A00311	A16237	343	A00311	A17108	455
A00312	A00313	446	A00312	A00314	484
A00312	A00316	281	A00312	A00351	179
A00312	A00395	462	A00312	A00449	491
A00312	A00541	241	A00312	A00574	475
A00312	A00600	333	A00312	A00601	470
A00312	A00654	332	A00312	A00660	491
A00312	A00663	369	A00312	A00668	479
A00312	A00696	327	A00312	A00736	230
A00312	A00825	457	A00312	A00832	208
A00312	A00930	491	A00312	A00962	430
A00312	A01008	190	A00312	A01018	435
A00312	A01046	346	A00312	A01137	480
A00312	A01178	356	A00312	A01212	371
A00312	A01227	368	A00312	A01297	307
A00312	A01397	347	A00312	A01409	452
A00312	A01462	171	A00312	A01466	208
A00312	A01476	362	A00312	A01490	259
A00312	A01524	899	A00312	A01565	357
A00312	A01572	407	A00312	A01590	480
A00312	A01591	199	A00312	A01635	212
A00312	A01660	227	A00312	A01722	400
A00312	A01726	485	A00312	A01793	255
A00312	A01850	190	A00312	A01926	196
A00312	A01978	259	A00312	A02053	268
A00312	A02054	491	A00312	A02083	457

A00312	A02091	227	A00312	A02104	168
A00312	A02112	479	A00312	A02114	236
A00312	A02254	491	A00312	A02257	443
A00312	A02258	238	A00312	A02266	303
A00312	A02284	491	A00312	A02285	424
A00312	A02286	266	A00312	A02301	177
A00312	A02306	457	A00312	A02310	314
A00312	A02330	469	A00312	A02492	383
A00312	A02644	188	A00312	A02903	163
A00312	A03054	360	A00312	A03084	405
A00312	A03087	488	A00312	A03099	945
A00312	A03164	434	A00312	A03167	162
A00312	A03171	485	A00312	A03174	294
A00312	A03234	185	A00312	A03520	307
A00312	A03523	204	A00312	A03572	180
A00312	A03591	452	A00312	A04160	340
A00312	A04162	226	A00312	A04194	345
A00312	A04421	217	A00312	A04471	157
A00312	A04883	249	A00312	A05006	177
A00312	A05066	185	A00312	A05176	157
A00312	A05208	406	A00312	A05224	185
A00312	A05288	343	A00312	A05310	156
A00312	A05331	227	A00312	A05333	201
A00312	A05356	372	A00312	A05374	252
A00312	A05416	313	A00312	A05418	405
A00312	A05424	488	A00312	A05430	303
A00312	A05462	490	A00312	A05493	154
A00312	A05533	339	A00312	A05550	176
A00312	A05610	201	A00312	A05696	227
A00312	A05740	303	A00312	A05753	363
A00312	A05754	353	A00312	A05786	190
A00312	A06037	491	A00312	A06086	350
A00312	A06245	195	A00312	A06473	338
A00312	A06607	318	A00312	A06626	481
A00312	A06762	945	A00312	A06769	488
A00312	A06860	154	A00312	A06874	163
A00312	A06914	249	A00312	A07059	313
A00312	A07088	445	A00312	A07239	430
A00312	A07422	207	A00312	A07520	433
A00312	A07656	428	A00312	A07684	224
A00312	A07763	210	A00312	A07771	466
A00312	A07851	297	A00312	A08133	199
A00312	A08135	241	A00312	A08197	252
A00312	A08636	363	A00312	A08707	491
A00312	A08744	303	A00312	A09418	208
A00312	A09659	160	A00312	A09943	154

A00312	A09955	308	A00312	A10679	255
A00312	A11302	165	A00312	A11621	837
A00312	A13032	386	A00312	A13054	400
A00312	A13155	227	A00312	A13362	388
A00312	A13734	326	A00312	A13916	154
A00312	A14252	483	A00312	A14503	227
A00312	A14589	408	A00312	A15298	252
A00312	A15318	235	A00312	A15356	489
A00312	A16027	191	A00312	A16719	190
A00312	A17201	273	A00313	A00395	263
A00313	A00574	205	A00313	A00654	208
A00313	A00660	324	A00313	A00962	191
A00313	A01008	177	A00313	A01018	278
A00313	A01367	272	A00313	A01635	224
A00313	A02083	288	A00313	A02285	450
A00313	A02698	199	A00313	A03087	335
A00313	A03099	270	A00313	A03477	229
A00313	A04880	299	A00313	A05333	408
A00313	A07933	262	A00313	A08138	236
A00313	A10882	406	A00314	A00376	219
A00314	A00468	439	A00314	A00579	333
A00314	A00633	179	A00314	A00660	468
A00314	A00695	247	A00314	A00736	160
A00314	A01137	162	A00314	A01230	258
A00314	A01233	174	A00314	A01397	259
A00314	A01490	452	A00314	A01793	200
A00314	A01832	308	A00314	A01972	243
A00314	A02026	198	A00314	A02053	323
A00314	A02054	167	A00314	A02114	450
A00314	A02148	151	A00314	A02254	456
A00314	A02257	486	A00314	A02259	408
A00314	A02284	451	A00314	A02286	409
A00314	A02290	319	A00314	A02366	369
A00314	A02495	196	A00314	A02522	324
A00314	A02621	257	A00314	A02802	396
A00314	A02852	420	A00314	A02908	184
A00314	A02968	252	A00314	A03084	310
A00314	A03087	486	A00314	A03099	296
A00314	A03167	329	A00314	A03168	265
A00314	A03171	425	A00314	A03174	458
A00314	A03436	154	A00314	A03520	156
A00314	A04162	234	A00314	A04211	226
A00314	A05208	312	A00314	A05224	353
A00314	A05330	294	A00314	A05331	281
A00314	A05338	305	A00314	A05424	462
A00314	A05573	246	A00314	A05760	257

A00314	A06633	297	A00314	A06762	286
A00314	A06769	215	A00314	A06970	156
A00314	A07191	339	A00314	A07264	279
A00314	A07771	238	A00314	A07851	183
A00314	A08190	218	A00314	A08197	313
A00314	A08732	260	A00314	A09124	154
A00314	A09526	179	A00314	A10679	314
A00314	A13980	199	A00314	A16027	213
A00314	A16028	307	A00314	A16741	152
A00315	A00446	63	A00315	A00469	235
A00315	A00674	163	A00315	A00815	480
A00315	A00989	93	A00315	A01009	240
A00315	A01124	226	A00315	A01235	160
A00315	A01257	468	A00315	A01278	299
A00315	A01400	339	A00315	A01477	358
A00315	A02143	168	A00315	A02214	288
A00315	A02236	273	A00315	A02305	241
A00315	A02425	470	A00315	A02429	440
A00315	A02454	177	A00315	A02492	190
A00315	A02703	162	A00315	A03047	329
A00315	A03110	290	A00315	A03155	385
A00315	A03578	219	A00315	A03633	318
A00315	A03750	218	A00315	A03788	168
A00315	A03942	202	A00315	A03961	344
A00315	A04180	318	A00315	A04380	458
A00315	A04647	310	A00315	A04666	176
A00315	A04751	210	A00315	A04813	294
A00315	A05439	168	A00315	A05497	374
A00315	A05517	296	A00315	A05949	276
A00315	A05965	374	A00315	A06997	255
A00315	A07226	227	A00315	A08583	307
A00315	A08875	198	A00315	A09038	230
A00315	A09062	422	A00315	A09239	171
A00315	A10716	405	A00315	A12824	162
A00315	A13997	171	A00315	A14007	174
A00315	A14210	324	A00315	A14443	906
A00315	A14693	438	A00315	A15430	339
A00316	A00348	335	A00316	A00350	210
A00316	A00351	465	A00316	A00378	477
A00316	A00418	447	A00316	A00421	329
A00316	A00517	272	A00316	A00531	433
A00316	A00532	456	A00316	A00552	665
A00316	A00565	485	A00316	A00571	455
A00316	A00655	424	A00316	A00658	210
A00316	A00696	395	A00316	A00719	383
A00316	A00733	480	A00316	A00757	491

A00316	A00805	405	A00316	A00822	473
A00316	A00825	383	A00316	A00843	260
A00316	A00887	484	A00316	A00905	212
A00316	A00981	377	A00316	A01052	491
A00316	A01128	235	A00316	A01136	229
A00316	A01137	180	A00316	A01164	274
A00316	A01173	485	A00316	A01212	490
A00316	A01215	277	A00316	A01353	317
A00316	A01354	480	A00316	A01385	302
A00316	A01406	399	A00316	A01426	383
A00316	A01485	188	A00316	A01492	276
A00316	A01494	396	A00316	A01524	262
A00316	A01527	435	A00316	A01553	302
A00316	A01554	900	A00316	A01558	945
A00316	A01559	486	A00316	A01560	283
A00316	A01572	490	A00316	A01588	940
A00316	A01591	308	A00316	A01602	198
A00316	A01610	489	A00316	A01612	418
A00316	A01615	908	A00316	A01628	446
A00316	A01631	428	A00316	A01639	412
A00316	A01640	430	A00316	A01647	490
A00316	A01656	243	A00316	A01660	491
A00316	A01694	488	A00316	A01708	332
A00316	A01730	430	A00316	A01995	665
A00316	A02074	183	A00316	A02076	152
A00316	A02083	391	A00316	A02110	63
A00316	A02111	491	A00316	A02114	472
A00316	A02116	63	A00316	A02117	664
A00316	A02120	224	A00316	A02306	310
A00316	A02314	184	A00316	A02325	212
A00316	A02360	104	A00316	A02428	63
A00316	A02435	252	A00316	A02436	63
A00316	A02442	193	A00316	A02450	451
A00316	A02470	63	A00316	A02477	63
A00316	A02482	467	A00316	A02644	384
A00316	A02715	358	A00316	A02737	159
A00316	A03056	93	A00316	A03060	184
A00316	A03099	449	A00316	A03104	188
A00316	A03107	369	A00316	A03225	174
A00316	A03292	335	A00316	A03436	441
A00316	A03451	160	A00316	A03468	85
A00316	A03511	462	A00316	A03569	429
A00316	A03611	182	A00316	A03647	151
A00316	A03714	168	A00316	A03737	193
A00316	A03771	177	A00316	A03886	180
A00316	A03968	664	A00316	A03990	491

A00316	A04012	294	A00316	A04092	486
A00316	A04157	489	A00316	A04168	184
A00316	A04215	205	A00316	A04256	219
A00316	A04266	176	A00316	A04297	156
A00316	A04326	168	A00316	A04336	252
A00316	A04349	268	A00316	A04379	217
A00316	A04383	338	A00316	A04387	276
A00316	A04437	193	A00316	A04463	229
A00316	A04650	205	A00316	A04822	308
A00316	A04823	168	A00316	A04997	350
A00316	A05216	480	A00316	A05224	283
A00316	A05288	465	A00316	A05330	174
A00316	A05332	65	A00316	A05337	165
A00316	A05411	438	A00316	A05418	363
A00316	A05435	221	A00316	A05455	162
A00316	A05474	193	A00316	A05751	345
A00316	A05786	156	A00316	A06380	205
A00316	A06405	165	A00316	A06660	236
A00316	A06733	241	A00316	A06919	249
A00316	A07054	210	A00316	A07118	168
A00316	A07119	294	A00316	A07311	490
A00316	A07339	491	A00316	A07474	168
A00316	A07528	221	A00316	A07870	276
A00316	A08280	312	A00316	A08291	184
A00316	A08292	85	A00316	A08334	276
A00316	A08343	278	A00316	A08571	491
A00316	A09004	184	A00316	A09044	165
A00316	A09078	299	A00316	A09110	132
A00316	A09160	63	A00316	A09393	193
A00316	A09543	184	A00316	A09791	397
A00316	A09993	441	A00316	A10296	193
A00316	A10332	369	A00316	A10483	276
A00316	A10514	277	A00316	A10537	276
A00316	A11068	266	A00316	A11277	388
A00316	A11487	491	A00316	A11611	205
A00316	A12383	314	A00316	A12829	162
A00316	A12988	301	A00316	A13108	193
A00316	A13250	899	A00316	A13824	208
A00316	A13997	299	A00316	A14006	491
A00316	A14009	486	A00316	A14055	229
A00316	A14063	176	A00316	A14198	435
A00316	A14444	903	A00316	A14452	267
A00316	A14824	243	A00316	A15226	146
A00316	A15230	63	A00316	A15233	475
A00316	A15241	405	A00316	A15271	416
A00316	A15279	243	A00316	A15325	202

A00316	A15504	238	A00316	A15628	156
A00316	A15726	917	A00316	A15935	302
A00316	A16060	288	A00316	A16746	184
A00316	A16867	283	A00316	A16994	449
A00316	A17168	260	A00317	A04728	311
A00317	A04783	373	A00317	A07746	322
A00318	A00322	463	A00318	A00338	447
A00318	A00472	490	A00318	A00477	312
A00318	A00485	396	A00318	A00531	383
A00318	A00802	477	A00318	A00882	457
A00318	A01036	610	A00318	A01156	306
A00318	A01235	241	A00318	A01458	254
A00318	A01578	475	A00318	A01632	462
A00318	A02178	307	A00318	A02180	324
A00318	A02221	489	A00318	A02303	373
A00318	A02511	221	A00318	A02738	302
A00318	A02749	265	A00318	A02908	425
A00318	A02909	196	A00318	A03014	491
A00318	A03211	291	A00318	A03349	227
A00318	A03358	345	A00318	A03366	202
A00318	A03726	380	A00318	A03984	301
A00318	A04252	352	A00318	A04275	408
A00318	A04337	463	A00318	A04361	321
A00318	A04410	362	A00318	A04520	307
A00318	A04597	252	A00318	A04603	285
A00318	A04715	224	A00318	A04964	177
A00318	A05155	185	A00318	A05356	411
A00318	A05439	335	A00318	A05556	357
A00318	A05603	159	A00318	A05777	157
A00318	A06070	238	A00318	A06277	168
A00318	A06346	424	A00318	A06574	273
A00318	A06623	179	A00318	A06731	195
A00318	A06739	458	A00318	A07054	515
A00318	A07242	343	A00318	A07985	291
A00318	A08093	347	A00318	A08097	351
A00318	A08171	463	A00318	A08172	491
A00318	A08309	491	A00318	A08531	611
A00318	A08681	490	A00318	A08685	491
A00318	A08686	491	A00318	A08753	491
A00318	A08835	480	A00318	A08859	357
A00318	A09046	199	A00318	A10278	163
A00318	A10621	240	A00318	A11191	388
A00318	A11278	491	A00318	A11298	480
A00318	A11315	491	A00318	A11333	452
A00318	A11431	294	A00318	A11454	450
A00318	A11463	391	A00318	A11585	326

A00318	A11594	311	A00318	A12003	446
A00318	A12046	288	A00318	A12684	326
A00318	A12781	311	A00318	A13369	474
A00318	A13987	350	A00318	A14381	205
A00318	A14570	200	A00318	A14633	318
A00318	A15292	416	A00318	A15313	172
A00318	A15413	185	A00318	A15416	477
A00318	A15452	232	A00318	A15457	216
A00318	A15498	489	A00318	A15552	221
A00318	A15578	446	A00318	A15638	491
A00318	A16274	188	A00318	A16281	490
A00319	A01690	182	A00319	A01774	401
A00319	A01781	286	A00319	A01844	375
A00319	A02076	243	A00319	A02470	163
A00319	A02494	160	A00319	A02564	191
A00319	A02737	338	A00319	A02816	217
A00319	A02870	368	A00319	A02904	475
A00319	A03001	247	A00319	A03214	296
A00319	A03271	160	A00319	A04346	486
A00319	A04517	324	A00319	A06398	338
A00319	A06654	307	A00319	A10721	258
A00320	A00348	126	A00320	A00432	283
A00320	A00804	321	A00320	A01274	201
A00320	A01983	152	A00320	A02175	379
A00320	A02507	296	A00320	A02621	226
A00320	A02816	162	A00320	A03906	212
A00320	A04909	281	A00320	A05176	221
A00320	A05256	274	A00320	A05597	266
A00320	A06078	286	A00320	A07226	208
A00320	A08097	162	A00320	A09341	276
A00320	A09812	316	A00320	A10894	201
A00321	A00422	385	A00321	A01622	360
A00321	A03119	418	A00321	A03232	416
A00321	A04376	438	A00321	A04967	452
A00321	A05874	452	A00321	A07694	452
A00321	A08105	441	A00321	A09478	441
A00321	A10223	445	A00322	A00338	491
A00322	A00347	490	A00322	A00352	286
A00322	A00472	491	A00322	A00477	202
A00322	A00482	341	A00322	A00485	462
A00322	A00531	265	A00322	A00620	180
A00322	A00625	477	A00322	A00636	306
A00322	A00638	389	A00322	A00802	213
A00322	A00855	177	A00322	A00856	707
A00322	A00887	447	A00322	A00911	720
A00322	A00989	260	A00322	A01011	401

A00322	A01022	393	A00322	A01026	260
A00322	A01086	485	A00322	A01124	443
A00322	A01161	477	A00322	A01214	631
A00322	A01296	687	A00322	A01311	414
A00322	A01320	824	A00322	A01327	561
A00322	A01454	258	A00322	A01485	491
A00322	A01529	491	A00322	A01530	491
A00322	A01543	394	A00322	A01556	455
A00322	A01557	490	A00322	A01572	899
A00322	A01578	470	A00322	A01582	170
A00322	A01630	221	A00322	A01632	485
A00322	A01689	345	A00322	A01790	245
A00322	A01970	172	A00322	A02048	484
A00322	A02188	491	A00322	A02198	252
A00322	A02221	491	A00322	A02228	491
A00322	A02231	391	A00322	A02261	174
A00322	A02310	157	A00322	A02316	205
A00322	A02320	394	A00322	A02333	296
A00322	A02511	319	A00322	A02523	347
A00322	A02539	210	A00322	A02668	221
A00322	A02672	174	A00322	A02673	177
A00322	A02687	390	A00322	A02738	193
A00322	A02761	483	A00322	A02773	258
A00322	A02839	358	A00322	A03077	221
A00322	A03080	170	A00322	A03104	263
A00322	A03138	180	A00322	A03178	475
A00322	A03259	180	A00322	A03304	321
A00322	A03334	374	A00322	A03374	228
A00322	A03451	427	A00322	A03463	198
A00322	A03477	491	A00322	A03505	294
A00322	A03573	452	A00322	A03721	318
A00322	A03838	210	A00322	A03909	446
A00322	A04070	285	A00322	A04076	224
A00322	A04098	347	A00322	A04193	182
A00322	A04224	307	A00322	A04275	406
A00322	A04305	243	A00322	A04337	485
A00322	A04351	185	A00322	A04358	258
A00322	A04361	491	A00322	A04410	491
A00322	A04411	321	A00322	A04424	466
A00322	A04493	440	A00322	A04520	469
A00322	A04662	154	A00322	A04678	232
A00322	A04840	232	A00322	A04870	297
A00322	A04886	285	A00322	A04928	152
A00322	A04944	176	A00322	A04964	435
A00322	A05148	401	A00322	A05155	351
A00322	A05220	151	A00322	A05294	489

A00322	A05442	408	A00322	A05471	165
A00322	A05516	151	A00322	A05556	401
A00322	A05594	446	A00322	A05603	484
A00322	A05720	347	A00322	A05721	486
A00322	A05722	395	A00322	A05777	483
A00322	A05855	395	A00322	A05896	408
A00322	A05897	263	A00322	A05898	440
A00322	A05979	307	A00322	A06035	323
A00322	A06057	277	A00322	A06141	188
A00322	A06148	411	A00322	A06212	483
A00322	A06307	377	A00322	A06329	235
A00322	A06346	485	A00322	A06396	323
A00322	A06428	486	A00322	A06461	347
A00322	A06462	347	A00322	A06484	177
A00322	A06516	475	A00322	A06532	417
A00322	A06574	361	A00322	A06655	243
A00322	A06710	263	A00322	A06729	215
A00322	A06731	293	A00322	A06739	469
A00322	A06835	323	A00322	A06925	390
A00322	A07190	171	A00322	A07209	414
A00322	A07218	346	A00322	A07282	208
A00322	A07286	294	A00322	A07384	483
A00322	A07407	157	A00322	A07442	408
A00322	A07479	358	A00322	A07483	162
A00322	A07494	417	A00322	A07605	234
A00322	A07641	174	A00322	A07704	260
A00322	A07705	345	A00322	A07828	307
A00322	A07843	193	A00322	A08043	307
A00322	A08162	270	A00322	A08171	418
A00322	A08172	489	A00322	A08236	258
A00322	A08244	277	A00322	A08309	486
A00322	A08310	270	A00322	A08392	307
A00322	A08440	347	A00322	A08681	276
A00322	A08685	486	A00322	A08686	458
A00322	A08835	382	A00322	A08839	899
A00322	A08878	168	A00322	A09380	327
A00322	A09428	193	A00322	A09518	285
A00322	A09668	216	A00322	A10101	417
A00322	A10250	428	A00322	A10278	466
A00322	A10731	241	A00322	A10942	270
A00322	A10954	270	A00322	A11282	163
A00322	A11315	357	A00322	A11321	457
A00322	A11466	277	A00322	A11483	458
A00322	A11573	221	A00322	A12132	285
A00322	A12391	347	A00322	A12551	323
A00322	A12698	417	A00322	A12941	347

A00322	A13369	491	A00322	A14226	899
A00322	A14381	374	A00322	A14411	347
A00322	A14449	443	A00322	A14548	391
A00322	A14586	419	A00322	A14589	318
A00322	A14740	347	A00322	A15232	347
A00322	A15233	180	A00322	A15277	491
A00322	A15282	330	A00322	A15288	183
A00322	A15313	411	A00322	A15336	221
A00322	A15416	263	A00322	A15495	414
A00322	A15498	208	A00322	A15638	491
A00322	A16055	198	A00322	A16281	218
A00322	A17038	154	A00323	A00329	654
A00323	A00348	207	A00323	A00366	160
A00323	A00454	491	A00323	A00653	193
A00323	A00910	480	A00323	A00933	180
A00323	A01042	491	A00323	A01054	362
A00323	A01082	157	A00323	A01083	449
A00323	A01150	436	A00323	A01194	174
A00323	A01232	193	A00323	A01300	422
A00323	A01431	474	A00323	A01540	362
A00323	A01649	163	A00323	A02738	258
A00323	A03446	246	A00323	A03740	654
A00323	A03752	265	A00323	A03809	163
A00323	A05981	160	A00323	A06466	488
A00323	A07311	488	A00323	A07487	159
A00323	A07798	174	A00323	A08054	481
A00323	A08359	276	A00323	A08488	263
A00323	A08571	262	A00323	A09502	361
A00323	A11290	234	A00323	A11296	234
A00323	A11302	489	A00323	A11372	653
A00323	A11476	160	A00323	A11724	362
A00323	A11928	168	A00323	A11942	238
A00323	A12167	550	A00323	A12328	324
A00323	A13340	183	A00323	A14073	472
A00323	A15244	411	A00323	A15275	367
A00323	A15660	179	A00323	A15684	160
A00323	A15699	249	A00323	A15721	249
A00323	A16088	328	A00323	A16633	433
A00324	A00370	255	A00324	A00381	341
A00324	A00470	457	A00324	A00556	160
A00324	A00630	446	A00324	A00669	422
A00324	A00679	490	A00324	A00733	899
A00324	A00747	941	A00324	A00820	428
A00324	A00832	945	A00324	A00887	491
A00324	A00914	938	A00324	A00954	378
A00324	A01002	912	A00324	A01052	491

A00324	A01100	356	A00324	A01172	491
A00324	A01178	491	A00324	A01211	335
A00324	A01244	899	A00324	A01307	463
A00324	A01364	63	A00324	A01415	899
A00324	A01450	486	A00324	A01461	316
A00324	A01478	363	A00324	A01492	193
A00324	A01507	484	A00324	A01512	945
A00324	A01524	994	A00324	A01527	994
A00324	A01548	489	A00324	A01553	174
A00324	A01554	942	A00324	A01558	989
A00324	A01561	252	A00324	A01572	491
A00324	A01574	899	A00324	A01588	989
A00324	A01592	916	A00324	A01593	899
A00324	A01596	912	A00324	A01602	945
A00324	A01605	313	A00324	A01606	491
A00324	A01615	899	A00324	A01634	899
A00324	A01639	921	A00324	A01651	152
A00324	A01665	937	A00324	A01668	435
A00324	A01675	201	A00324	A01676	208
A00324	A01688	491	A00324	A01691	263
A00324	A01694	351	A00324	A01698	467
A00324	A01709	909	A00324	A01724	201
A00324	A01730	301	A00324	A01742	475
A00324	A01749	63	A00324	A01756	328
A00324	A01775	63	A00324	A01776	115
A00324	A01790	274	A00324	A01796	462
A00324	A01797	246	A00324	A01798	227
A00324	A01844	945	A00324	A01850	485
A00324	A01898	188	A00324	A02073	899
A00324	A02086	305	A00324	A02142	899
A00324	A02189	271	A00324	A02241	279
A00324	A02300	899	A00324	A02305	899
A00324	A02343	491	A00324	A02421	154
A00324	A02425	899	A00324	A02452	335
A00324	A02464	899	A00324	A02492	208
A00324	A02668	899	A00324	A02670	177
A00324	A02687	432	A00324	A02699	296
A00324	A02706	202	A00324	A02759	899
A00324	A02789	393	A00324	A02821	323
A00324	A02822	276	A00324	A02828	490
A00324	A02856	213	A00324	A02872	441
A00324	A02873	196	A00324	A02908	910
A00324	A02918	247	A00324	A02941	154
A00324	A02942	899	A00324	A02968	899
A00324	A03071	254	A00324	A03095	385
A00324	A03191	297	A00324	A03201	348

A00324	A03213	290	A00324	A03223	491
A00324	A03224	899	A00324	A03304	479
A00324	A03336	96	A00324	A03451	484
A00324	A03535	899	A00324	A03575	931
A00324	A03612	104	A00324	A03718	901
A00324	A03726	260	A00324	A03739	335
A00324	A03778	488	A00324	A03846	208
A00324	A03854	263	A00324	A03869	163
A00324	A03871	176	A00324	A03949	205
A00324	A03990	395	A00324	A04007	369
A00324	A04008	491	A00324	A04020	413
A00324	A04050	907	A00324	A04077	163
A00324	A04202	343	A00324	A04260	899
A00324	A04449	491	A00324	A04490	899
A00324	A04540	489	A00324	A04673	899
A00324	A05039	154	A00324	A05064	899
A00324	A05216	899	A00324	A05315	200
A00324	A05355	899	A00324	A05384	899
A00324	A05414	993	A00324	A05415	254
A00324	A05436	260	A00324	A05454	903
A00324	A05634	88	A00324	A05701	409
A00324	A05797	402	A00324	A05854	393
A00324	A06050	213	A00324	A06189	286
A00324	A06277	489	A00324	A06610	491
A00324	A06660	455	A00324	A06873	899
A00324	A06925	154	A00324	A07091	899
A00324	A07157	215	A00324	A07339	943
A00324	A07422	913	A00324	A07683	163
A00324	A07834	159	A00324	A08244	154
A00324	A08308	176	A00324	A08615	163
A00324	A08627	179	A00324	A08692	452
A00324	A08839	899	A00324	A08851	474
A00324	A08858	992	A00324	A08878	491
A00324	A09016	249	A00324	A09018	463
A00324	A09054	827	A00324	A09055	249
A00324	A09124	474	A00324	A09149	465
A00324	A09162	485	A00324	A09214	163
A00324	A09423	204	A00324	A09727	443
A00324	A09826	483	A00324	A10051	163
A00324	A10098	899	A00324	A10133	163
A00324	A10174	899	A00324	A10185	899
A00324	A10501	346	A00324	A10580	944
A00324	A10615	899	A00324	A10626	306
A00324	A10637	213	A00324	A10789	899
A00324	A10793	899	A00324	A10808	389
A00324	A10825	176	A00324	A10934	283

A00324	A10994	249	A00324	A11125	899
A00324	A11206	899	A00324	A11283	899
A00324	A11293	440	A00324	A11327	174
A00324	A11369	899	A00324	A11487	899
A00324	A11604	899	A00324	A11983	994
A00324	A12003	310	A00324	A12036	249
A00324	A12047	899	A00324	A12116	213
A00324	A12618	154	A00324	A12820	154
A00324	A13089	154	A00324	A13225	929
A00324	A13387	899	A00324	A13737	899
A00324	A13829	480	A00324	A13874	319
A00324	A13981	989	A00324	A13989	195
A00324	A13990	184	A00324	A13997	491
A00324	A14009	297	A00324	A14016	446
A00324	A14052	208	A00324	A14055	414
A00324	A14063	899	A00324	A14195	899
A00324	A14200	338	A00324	A14211	436
A00324	A14221	899	A00324	A14371	202
A00324	A14443	899	A00324	A14444	899
A00324	A14527	174	A00324	A14636	187
A00324	A14643	460	A00324	A14741	357
A00324	A14887	202	A00324	A14930	899
A00324	A15027	249	A00324	A16072	260
A00324	A16245	176	A00324	A16739	226
A00325	A00355	491	A00325	A00356	63
A00325	A00357	414	A00325	A00358	491
A00325	A00360	152	A00325	A00361	119
A00325	A00402	402	A00325	A00403	491
A00325	A00469	394	A00325	A00521	430
A00325	A00561	160	A00325	A00590	347
A00325	A00629	449	A00325	A00648	266
A00325	A00705	174	A00325	A00787	468
A00325	A00827	463	A00325	A00831	199
A00325	A00839	344	A00325	A00848	455
A00325	A00883	212	A00325	A00923	491
A00325	A01000	491	A00325	A01017	491
A00325	A01025	491	A00325	A01047	486
A00325	A01101	491	A00325	A01120	151
A00325	A01140	469	A00325	A01184	425
A00325	A01206	358	A00325	A01234	380
A00325	A01249	490	A00325	A01291	491
A00325	A01306	491	A00325	A01335	369
A00325	A01338	491	A00325	A01464	221
A00325	A01470	416	A00325	A01579	218
A00325	A01617	207	A00325	A01618	105
A00325	A01649	474	A00325	A01700	235

A00325	A01707	156	A00325	A02068	165
A00325	A02638	157	A00325	A02978	191
A00325	A02988	200	A00325	A03069	475
A00325	A03567	491	A00325	A03740	307
A00325	A03754	338	A00325	A03859	270
A00325	A05385	361	A00325	A06279	93
A00325	A06861	378	A00325	A06965	491
A00325	A07451	468	A00325	A08011	330
A00325	A08055	156	A00325	A08398	324
A00325	A08452	351	A00325	A08558	205
A00325	A08760	200	A00325	A09322	490
A00325	A09674	215	A00325	A09702	321
A00325	A10529	268	A00325	A11438	483
A00325	A11480	491	A00325	A11704	489
A00325	A11733	223	A00325	A11945	419
A00325	A12077	338	A00325	A12221	457
A00325	A12233	207	A00325	A12241	202
A00325	A12509	268	A00325	A13989	439
A00325	A14152	185	A00325	A14188	318
A00325	A14216	430	A00325	A14596	353
A00325	A15139	360	A00325	A15218	417
A00325	A15221	154	A00325	A16118	424
A00325	A16187	156	A00325	A16387	235
A00325	A16631	151	A00325	A17127	329
A00326	A00440	168	A00326	A00643	241
A00326	A00714	208	A00326	A01046	486
A00326	A01088	229	A00326	A01143	230
A00326	A01274	417	A00326	A01299	647
A00326	A01305	483	A00326	A01441	193
A00326	A01550	160	A00326	A01740	193
A00326	A02064	362	A00326	A02830	278
A00326	A02902	651	A00326	A02931	489
A00326	A03591	154	A00326	A05288	483
A00326	A06881	362	A00326	A07311	188
A00326	A08528	199	A00326	A09361	385
A00326	A11300	630	A00326	A11387	601
A00326	A11447	362	A00326	A11621	167
A00326	A11706	219	A00326	A11923	167
A00326	A11932	165	A00326	A12111	228
A00326	A12365	335	A00326	A12373	235
A00326	A12757	188	A00326	A13376	259
A00326	A14660	286	A00326	A15560	291
A00326	A15852	185	A00326	A16330	362
A00326	A16700	252	A00326	A16798	311
A00327	A00328	670	A00327	A00353	486
A00327	A00356	454	A00327	A00357	201

A00327	A00427	317	A00327	A00531	224
A00327	A00648	317	A00327	A00649	255
A00327	A00651	629	A00327	A00701	341
A00327	A00763	481	A00327	A00787	308
A00327	A00861	251	A00327	A00872	293
A00327	A00878	317	A00327	A00914	296
A00327	A00922	477	A00327	A00965	317
A00327	A00966	330	A00327	A00990	667
A00327	A00994	481	A00327	A01053	424
A00327	A01108	221	A00327	A01129	463
A00327	A01160	245	A00327	A01168	469
A00327	A01238	317	A00327	A01240	450
A00327	A01291	490	A00327	A01416	317
A00327	A01425	317	A00327	A01436	317
A00327	A01448	172	A00327	A01464	642
A00327	A01468	317	A00327	A01481	63
A00327	A01496	477	A00327	A01538	190
A00327	A01586	317	A00327	A01618	503
A00327	A01619	263	A00327	A01620	260
A00327	A01711	176	A00327	A01740	176
A00327	A01797	154	A00327	A01822	594
A00327	A02164	479	A00327	A02165	401
A00327	A02282	246	A00327	A02414	380
A00327	A02434	210	A00327	A02527	208
A00327	A02809	258	A00327	A02859	180
A00327	A03093	326	A00327	A03259	319
A00327	A03281	174	A00327	A03413	388
A00327	A03437	187	A00327	A03438	490
A00327	A03490	317	A00327	A03527	255
A00327	A03609	446	A00327	A03644	356
A00327	A03645	290	A00327	A03671	174
A00327	A03754	393	A00327	A03809	212
A00327	A03888	561	A00327	A03981	629
A00327	A04314	196	A00327	A05411	257
A00327	A05443	151	A00327	A05449	659
A00327	A05654	490	A00327	A06763	344
A00327	A06814	168	A00327	A06825	483
A00327	A06955	154	A00327	A07105	394
A00327	A07116	435	A00327	A07159	198
A00327	A07201	489	A00327	A07243	670
A00327	A07360	174	A00327	A07471	185
A00327	A07506	313	A00327	A08011	419
A00327	A08377	317	A00327	A08550	317
A00327	A08662	235	A00327	A08664	642
A00327	A08682	470	A00327	A08700	217
A00327	A08752	385	A00327	A08973	317

A00327	A09428	224	A00327	A10034	391
A00327	A10189	344	A00327	A10417	667
A00327	A10564	272	A00327	A10569	310
A00327	A10924	285	A00327	A11264	252
A00327	A11331	491	A00327	A11926	451
A00327	A11981	491	A00327	A12195	177
A00327	A12661	217	A00327	A12687	466
A00327	A13052	291	A00327	A13435	443
A00327	A13581	217	A00327	A13626	491
A00327	A13752	258	A00327	A13917	196
A00327	A14216	490	A00327	A14606	319
A00327	A14979	364	A00327	A15028	428
A00327	A15624	489	A00327	A15649	409
A00327	A15651	215	A00327	A15652	162
A00327	A15661	235	A00327	A15684	486
A00327	A16088	452	A00327	A16095	481
A00327	A16149	334	A00327	A16279	319
A00328	A00334	260	A00328	A00353	486
A00328	A00356	491	A00328	A00357	196
A00328	A00405	252	A00328	A00427	317
A00328	A00531	428	A00328	A00536	291
A00328	A00544	188	A00328	A00590	486
A00328	A00648	535	A00328	A00649	363
A00328	A00651	629	A00328	A00653	196
A00328	A00700	454	A00328	A00701	491
A00328	A00763	491	A00328	A00785	486
A00328	A00787	491	A00328	A00839	452
A00328	A00857	276	A00328	A00861	339
A00328	A00878	324	A00328	A00922	491
A00328	A00923	481	A00328	A00953	201
A00328	A00965	317	A00328	A00966	532
A00328	A00990	669	A00328	A00994	489
A00328	A01000	355	A00328	A01012	480
A00328	A01037	229	A00328	A01047	477
A00328	A01053	317	A00328	A01085	358
A00328	A01090	184	A00328	A01095	223
A00328	A01108	395	A00328	A01112	313
A00328	A01127	435	A00328	A01129	477
A00328	A01168	480	A00328	A01238	317
A00328	A01240	594	A00328	A01249	195
A00328	A01276	215	A00328	A01291	491
A00328	A01346	190	A00328	A01416	317
A00328	A01425	327	A00328	A01436	317
A00328	A01437	449	A00328	A01446	353
A00328	A01448	385	A00328	A01457	439
A00328	A01464	663	A00328	A01468	391

A00328	A01496	491	A00328	A01586	317
A00328	A01618	593	A00328	A01620	368
A00328	A01711	159	A00328	A01741	177
A00328	A01822	661	A00328	A02164	467
A00328	A02165	168	A00328	A02414	243
A00328	A02527	311	A00328	A02590	279
A00328	A02855	260	A00328	A03048	454
A00328	A03069	208	A00328	A03085	216
A00328	A03093	474	A00328	A03259	212
A00328	A03413	340	A00328	A03437	179
A00328	A03438	490	A00328	A03490	317
A00328	A03567	152	A00328	A03596	278
A00328	A03609	439	A00328	A03614	152
A00328	A03644	454	A00328	A03754	393
A00328	A03809	491	A00328	A03888	667
A00328	A03959	460	A00328	A03981	629
A00328	A04949	311	A00328	A05129	232
A00328	A05411	424	A00328	A05443	436
A00328	A05449	670	A00328	A05654	490
A00328	A06356	272	A00328	A06495	291
A00328	A06763	252	A00328	A06814	430
A00328	A06815	185	A00328	A06825	479
A00328	A07105	389	A00328	A07116	424
A00328	A07159	297	A00328	A07201	491
A00328	A07243	670	A00328	A07360	243
A00328	A07451	461	A00328	A07506	271
A00328	A08011	491	A00328	A08377	317
A00328	A08550	317	A00328	A08559	452
A00328	A08653	217	A00328	A08662	243
A00328	A08664	602	A00328	A08682	485
A00328	A08752	157	A00328	A08773	230
A00328	A08860	291	A00328	A08973	317
A00328	A09044	263	A00328	A09674	430
A00328	A09893	335	A00328	A10034	485
A00328	A10140	167	A00328	A10417	665
A00328	A10532	408	A00328	A10564	425
A00328	A10569	408	A00328	A10572	285
A00328	A10924	433	A00328	A11331	491
A00328	A11420	216	A00328	A11926	466
A00328	A11981	491	A00328	A12028	414
A00328	A12661	463	A00328	A12687	489
A00328	A13340	358	A00328	A13344	232
A00328	A13417	272	A00328	A13626	491
A00328	A13752	272	A00328	A13912	157
A00328	A13917	190	A00328	A13963	382
A00328	A13989	473	A00328	A14216	491

A00328	A14299	402	A00328	A14381	327
A00328	A14410	351	A00328	A14560	63
A00328	A14606	216	A00328	A15028	252
A00328	A15235	223	A00328	A15624	491
A00328	A15649	418	A00328	A15651	291
A00328	A15661	228	A00328	A15684	489
A00328	A15932	411	A00328	A15933	157
A00328	A16088	491	A00328	A16095	489
A00328	A16149	303	A00328	A16234	216
A00328	A16279	216	A00328	A17193	460
A00329	A00345	341	A00329	A00409	182
A00329	A00439	219	A00329	A00454	491
A00329	A00489	183	A00329	A00653	377
A00329	A00698	188	A00329	A00822	198
A00329	A00848	383	A00329	A00910	479
A00329	A00912	411	A00329	A00932	463
A00329	A00933	159	A00329	A00970	162
A00329	A01042	491	A00329	A01054	362
A00329	A01083	486	A00329	A01127	310
A00329	A01150	154	A00329	A01219	187
A00329	A01291	414	A00329	A01300	340
A00329	A01431	472	A00329	A01435	190
A00329	A01540	362	A00329	A01649	257
A00329	A01850	163	A00329	A02065	154
A00329	A02703	279	A00329	A03567	204
A00329	A03740	654	A00329	A03752	450
A00329	A03809	283	A00329	A06466	488
A00329	A07220	174	A00329	A07311	490
A00329	A07798	296	A00329	A08053	414
A00329	A08054	491	A00329	A08137	193
A00329	A08571	180	A00329	A09476	283
A00329	A09768	183	A00329	A10506	198
A00329	A11296	205	A00329	A11302	489
A00329	A11372	653	A00329	A11588	232
A00329	A11724	362	A00329	A12167	537
A00329	A12328	207	A00329	A12988	152
A00329	A12990	168	A00329	A13335	191
A00329	A13340	210	A00329	A13989	199
A00329	A14073	483	A00329	A14596	450
A00329	A15244	461	A00329	A15275	224
A00329	A15376	151	A00329	A15660	477
A00329	A15684	179	A00329	A15699	229
A00329	A16633	240	A00330	A00346	486
A00330	A00359	484	A00330	A00394	488
A00330	A00421	470	A00330	A00469	491
A00330	A00482	319	A00330	A00531	488

A00330	A00546	360	A00330	A00643	469
A00330	A00864	488	A00330	A00937	452
A00330	A00981	245	A00330	A01011	491
A00330	A01143	201	A00330	A01348	485
A00330	A01359	317	A00330	A01454	457
A00330	A01478	486	A00330	A01479	199
A00330	A01527	674	A00330	A01557	389
A00330	A01588	238	A00330	A01595	488
A00330	A01630	416	A00330	A01660	452
A00330	A01687	480	A00330	A01705	179
A00330	A01723	363	A00330	A01790	430
A00330	A01839	477	A00330	A02106	483
A00330	A02318	162	A00330	A02321	221
A00330	A02424	490	A00330	A02463	141
A00330	A02773	226	A00330	A03166	226
A00330	A03496	254	A00330	A03512	430
A00330	A03637	152	A00330	A03832	364
A00330	A04001	195	A00330	A04012	168
A00330	A04033	409	A00330	A04175	180
A00330	A04216	303	A00330	A04236	232
A00330	A04449	463	A00330	A04715	432
A00330	A04790	240	A00330	A04911	156
A00330	A05008	412	A00330	A05009	162
A00330	A05015	334	A00330	A05142	221
A00330	A05181	323	A00330	A05288	234
A00330	A06176	247	A00330	A06277	424
A00330	A06348	321	A00330	A06591	484
A00330	A07004	574	A00330	A07242	326
A00330	A07495	469	A00330	A07947	281
A00330	A08571	457	A00330	A08869	283
A00330	A09488	299	A00330	A09999	162
A00330	A10039	469	A00330	A10175	450
A00330	A10619	465	A00330	A11099	491
A00330	A11160	321	A00330	A11732	188
A00330	A12500	466	A00330	A13020	299
A00330	A13299	256	A00330	A14170	159
A00330	A14266	273	A00330	A14279	491
A00330	A14281	187	A00330	A14633	343
A00330	A14636	379	A00330	A14661	205
A00330	A14689	491	A00330	A15004	205
A00330	A15140	191	A00330	A15385	165
A00330	A16851	171	A00330	A16889	267
A00330	A16911	485	A00330	A17190	854
A00331	A00726	174	A00331	A00747	65
A00331	A00832	217	A00331	A00856	246
A00331	A01131	313	A00331	A01140	241

A00331	A01148	152	A00331	A01205	271
A00331	A01241	328	A00331	A01289	486
A00331	A01507	488	A00331	A01555	243
A00331	A01623	246	A00331	A01646	378
A00331	A02208	196	A00331	A02494	401
A00331	A02703	73	A00331	A02704	475
A00331	A02705	435	A00331	A02816	291
A00331	A02827	263	A00331	A02928	446
A00331	A03587	477	A00331	A04006	469
A00331	A04072	174	A00331	A04787	229
A00331	A04905	205	A00331	A05351	452
A00331	A08091	236	A00331	A09123	193
A00331	A09303	187	A00331	A09423	163
A00331	A10813	173	A00331	A12971	305
A00331	A13971	274	A00331	A14198	411
A00331	A14642	174	A00331	A16718	210
A00332	A00390	312	A00332	A01522	201
A00332	A01634	193	A00332	A03891	326
A00332	A04105	340	A00332	A04117	224
A00332	A04668	157	A00332	A12282	401
A00332	A13701	63	A00333	A00451	310
A00333	A00600	276	A00333	A00601	276
A00333	A00696	428	A00333	A01213	276
A00333	A01438	276	A00333	A01524	899
A00333	A01576	480	A00333	A01691	243
A00333	A03815	217	A00333	A03830	337
A00333	A04018	308	A00333	A04323	263
A00333	A04558	728	A00333	A05234	190
A00333	A05986	252	A00333	A08293	470
A00333	A08356	462	A00333	A08362	474
A00333	A08821	462	A00333	A08831	276
A00333	A08956	276	A00333	A09401	725
A00333	A09743	276	A00333	A09996	276
A00333	A12606	276	A00333	A13605	270
A00333	A13981	899	A00333	A13983	480
A00333	A14212	462	A00333	A14213	462
A00333	A14214	462	A00333	A15417	306
A00333	A15493	345	A00333	A16449	276
A00333	A16702	276	A00334	A00335	490
A00334	A00356	491	A00334	A00404	213
A00334	A00467	486	A00334	A00521	314
A00334	A00561	246	A00334	A00590	288
A00334	A00605	364	A00334	A00648	201
A00334	A00651	469	A00334	A00700	491
A00334	A00787	291	A00334	A00811	271
A00334	A00839	491	A00334	A00851	229

A00334	A00923	310	A00334	A00970	152
A00334	A01000	235	A00334	A01056	372
A00334	A01083	179	A00334	A01090	195
A00334	A01098	200	A00334	A01112	486
A00334	A01127	424	A00334	A01140	491
A00334	A01212	435	A00334	A01216	210
A00334	A01291	491	A00334	A01306	297
A00334	A01335	457	A00334	A01425	238
A00334	A01437	380	A00334	A01457	305
A00334	A01468	491	A00334	A01492	191
A00334	A01496	363	A00334	A01579	491
A00334	A01601	491	A00334	A01605	380
A00334	A01617	208	A00334	A01649	414
A00334	A01707	157	A00334	A01718	452
A00334	A02350	491	A00334	A03272	180
A00334	A03451	380	A00334	A03461	172
A00334	A03479	173	A00334	A03740	183
A00334	A03963	241	A00334	A03981	163
A00334	A03993	283	A00334	A04099	393
A00334	A04101	219	A00334	A04230	151
A00334	A04474	269	A00334	A04827	390
A00334	A05121	162	A00334	A05443	467
A00334	A06317	221	A00334	A06534	474
A00334	A06814	240	A00334	A06861	205
A00334	A06897	200	A00334	A06965	436
A00334	A06973	252	A00334	A07323	404
A00334	A07398	457	A00334	A07451	198
A00334	A07506	305	A00334	A08357	263
A00334	A08358	312	A00334	A08359	481
A00334	A08398	223	A00334	A08408	259
A00334	A08452	273	A00334	A08543	196
A00334	A08547	234	A00334	A08972	208
A00334	A09308	174	A00334	A09620	165
A00334	A09852	368	A00334	A10949	187
A00334	A11390	273	A00334	A11392	171
A00334	A11808	234	A00334	A11857	208
A00334	A13989	491	A00334	A13992	469
A00334	A14176	443	A00334	A14216	257
A00334	A14505	171	A00334	A15221	288
A00334	A16159	401	A00334	A16437	157
A00334	A16839	260	A00335	A00353	154
A00335	A00356	491	A00335	A00414	350
A00335	A00467	467	A00335	A00571	204
A00335	A00605	193	A00335	A00648	389
A00335	A00651	486	A00335	A00700	486
A00335	A00782	165	A00335	A00839	491

A00335	A00848	254	A00335	A00851	218
A00335	A00861	110	A00335	A00870	302
A00335	A00916	63	A00335	A00990	241
A00335	A01031	212	A00335	A01037	296
A00335	A01056	462	A00335	A01090	489
A00335	A01091	899	A00335	A01098	696
A00335	A01112	450	A00335	A01130	204
A00335	A01140	491	A00335	A01196	455
A00335	A01212	182	A00335	A01249	229
A00335	A01291	597	A00335	A01306	340
A00335	A01335	303	A00335	A01425	714
A00335	A01468	470	A00335	A01492	624
A00335	A01496	481	A00335	A01579	491
A00335	A01601	945	A00335	A01740	154
A00335	A02179	247	A00335	A02243	229
A00335	A02265	98	A00335	A02270	249
A00335	A02350	489	A00335	A03083	441
A00335	A03164	224	A00335	A03259	154
A00335	A03272	172	A00335	A03332	401
A00335	A03432	154	A00335	A03445	474
A00335	A03726	152	A00335	A03963	937
A00335	A03981	489	A00335	A04230	418
A00335	A05076	363	A00335	A05385	228
A00335	A05443	438	A00335	A05450	162
A00335	A05501	152	A00335	A05996	156
A00335	A06814	456	A00335	A06889	177
A00335	A06897	291	A00335	A06973	380
A00335	A06987	238	A00335	A07398	452
A00335	A07412	308	A00335	A07422	900
A00335	A07726	461	A00335	A08154	167
A00335	A08273	205	A00335	A08301	353
A00335	A09550	371	A00335	A09722	306
A00335	A09893	296	A00335	A09960	306
A00335	A10336	84	A00335	A10532	249
A00335	A10645	469	A00335	A10748	458
A00335	A11096	350	A00335	A11124	163
A00335	A11190	271	A00335	A12126	207
A00335	A12305	333	A00335	A12404	276
A00335	A12970	281	A00335	A13107	351
A00335	A13989	626	A00335	A14055	274
A00335	A14216	347	A00335	A14471	210
A00335	A15225	168	A00335	A15229	357
A00335	A15268	942	A00335	A15381	441
A00335	A15684	378	A00335	A16107	924
A00335	A16437	279	A00335	A16778	457
A00336	A00454	335	A00336	A00747	288

A00336	A01042	159	A00336	A01178	401
A00336	A01540	329	A00336	A01560	241
A00336	A01850	491	A00336	A01897	219
A00336	A02180	198	A00336	A02562	240
A00336	A02564	156	A00336	A02737	172
A00336	A02837	88	A00336	A02904	193
A00336	A03740	371	A00336	A03785	260
A00336	A04540	224	A00336	A04568	285
A00336	A06703	341	A00336	A07311	230
A00336	A07900	266	A00336	A08345	281
A00336	A08692	389	A00336	A09476	246
A00336	A09568	196	A00336	A09988	358
A00336	A10623	213	A00336	A10728	171
A00336	A12215	322	A00336	A13980	185
A00336	A14073	269	A00337	A00340	602
A00337	A00482	489	A00337	A00562	321
A00337	A00565	486	A00337	A00630	485
A00337	A00643	367	A00337	A00826	523
A00337	A00839	319	A00337	A00939	283
A00337	A00957	474	A00337	A00971	281
A00337	A00980	485	A00337	A01011	639
A00337	A01125	436	A00337	A01291	180
A00337	A01309	515	A00337	A01348	345
A00337	A01553	252	A00337	A01561	194
A00337	A01572	302	A00337	A01630	355
A00337	A01632	472	A00337	A01657	160
A00337	A01660	345	A00337	A01730	327
A00337	A01737	232	A00337	A01747	319
A00337	A01839	475	A00337	A01995	384
A00337	A02110	485	A00337	A02703	379
A00337	A03136	151	A00337	A03188	324
A00337	A03541	163	A00337	A03591	318
A00337	A04175	771	A00337	A04449	541
A00337	A04715	491	A00337	A04790	249
A00337	A05416	347	A00337	A05853	184
A00337	A06021	228	A00337	A06379	340
A00337	A06466	260	A00337	A06603	151
A00337	A06680	201	A00337	A06887	257
A00337	A07163	561	A00337	A07848	218
A00337	A07903	268	A00337	A08858	167
A00337	A09182	302	A00337	A09504	176
A00337	A09511	196	A00337	A09525	323
A00337	A09551	277	A00337	A09607	424
A00337	A09646	489	A00337	A09840	216
A00337	A10051	491	A00337	A10205	488
A00337	A10430	561	A00337	A10722	174

A00337	A11196	208	A00337	A11435	358
A00337	A11487	443	A00337	A11726	307
A00337	A11732	221	A00337	A11919	418
A00337	A11937	309	A00337	A12603	323
A00337	A13587	196	A00337	A13928	491
A00337	A14174	260	A00337	A14281	154
A00337	A14410	317	A00337	A14633	243
A00337	A15555	177	A00337	A15665	598
A00337	A15682	393	A00337	A16072	180
A00337	A16283	216	A00337	A16410	491
A00337	A16847	561	A00337	A17156	188
A00338	A00342	154	A00338	A00377	609
A00338	A00392	200	A00338	A00438	462
A00338	A00472	678	A00338	A00485	491
A00338	A00531	177	A00338	A00532	268
A00338	A00576	162	A00338	A00584	202
A00338	A00600	491	A00338	A00609	441
A00338	A00625	484	A00338	A00663	310
A00338	A00719	446	A00338	A00736	372
A00338	A00743	243	A00338	A00799	312
A00338	A00802	469	A00338	A00821	312
A00338	A00849	483	A00338	A00882	281
A00338	A00911	382	A00338	A00939	215
A00338	A00967	399	A00338	A00981	263
A00338	A00985	165	A00338	A01011	417
A00338	A01022	491	A00338	A01026	386
A00338	A01100	417	A00338	A01102	335
A00338	A01139	353	A00338	A01156	489
A00338	A01161	708	A00338	A01162	416
A00338	A01211	491	A00338	A01235	223
A00338	A01276	483	A00338	A01278	586
A00338	A01326	335	A00338	A01359	307
A00338	A01373	299	A00338	A01431	477
A00338	A01451	491	A00338	A01454	414
A00338	A01461	213	A00338	A01478	170
A00338	A01481	491	A00338	A01483	165
A00338	A01515	364	A00338	A01520	196
A00338	A01524	419	A00338	A01526	491
A00338	A01530	483	A00338	A01543	469
A00338	A01551	489	A00338	A01556	491
A00338	A01557	479	A00338	A01578	491
A00338	A01582	491	A00338	A01585	486
A00338	A01593	565	A00338	A01600	391
A00338	A01627	485	A00338	A01630	317
A00338	A01632	491	A00338	A01648	159
A00338	A01660	401	A00338	A01664	268

A00338	A01676	154	A00338	A01689	272
A00338	A01701	157	A00338	A01723	152
A00338	A01729	152	A00338	A01758	235
A00338	A01763	462	A00338	A01790	174
A00338	A01855	152	A00338	A01931	475
A00338	A01935	221	A00338	A02007	384
A00338	A02021	265	A00338	A02148	491
A00338	A02188	488	A00338	A02198	270
A00338	A02205	249	A00338	A02215	491
A00338	A02220	154	A00338	A02221	475
A00338	A02222	488	A00338	A02224	258
A00338	A02228	475	A00338	A02231	165
A00338	A02333	452	A00338	A02443	429
A00338	A02477	486	A00338	A02485	380
A00338	A02491	232	A00338	A02548	263
A00338	A02691	157	A00338	A02738	429
A00338	A02924	486	A00338	A03050	489
A00338	A03065	241	A00338	A03079	408
A00338	A03080	491	A00338	A03104	486
A00338	A03178	332	A00338	A03186	173
A00338	A03200	257	A00338	A03211	327
A00338	A03233	380	A00338	A03259	288
A00338	A03293	491	A00338	A03334	229
A00338	A03347	389	A00338	A03348	319
A00338	A03349	396	A00338	A03351	491
A00338	A03356	417	A00338	A03358	435
A00338	A03361	363	A00338	A03365	221
A00338	A03369	374	A00338	A03370	408
A00338	A03372	257	A00338	A03374	232
A00338	A03376	490	A00338	A03378	226
A00338	A03379	491	A00338	A03383	210
A00338	A03384	466	A00338	A03388	174
A00338	A03398	168	A00338	A03417	458
A00338	A03440	224	A00338	A03477	226
A00338	A03491	221	A00338	A03499	154
A00338	A03569	329	A00338	A03573	236
A00338	A03650	172	A00338	A03708	488
A00338	A03748	191	A00338	A03794	195
A00338	A03819	154	A00338	A03885	291
A00338	A03910	396	A00338	A03918	266
A00338	A03945	371	A00338	A03984	252
A00338	A04006	234	A00338	A04072	356
A00338	A04150	228	A00338	A04266	154
A00338	A04337	491	A00338	A04361	347
A00338	A04410	490	A00338	A04418	223
A00338	A04424	235	A00338	A04449	418

A00338	A04491	152	A00338	A04493	390
A00338	A04518	213	A00338	A04520	488
A00338	A04532	457	A00338	A04715	447
A00338	A04782	293	A00338	A04790	414
A00338	A04830	160	A00338	A04832	217
A00338	A04833	286	A00338	A04928	313
A00338	A04944	157	A00338	A04964	385
A00338	A05022	380	A00338	A05155	433
A00338	A05184	157	A00338	A05231	221
A00338	A05234	491	A00338	A05294	271
A00338	A05356	629	A00338	A05427	170
A00338	A05439	303	A00338	A05487	372
A00338	A05494	434	A00338	A05577	372
A00338	A05582	184	A00338	A05587	477
A00338	A05603	466	A00338	A05642	200
A00338	A05643	236	A00338	A05644	380
A00338	A05657	389	A00338	A05678	416
A00338	A05720	257	A00338	A05739	466
A00338	A05777	440	A00338	A05812	313
A00338	A05860	172	A00338	A05887	433
A00338	A05891	491	A00338	A05952	388
A00338	A05968	480	A00338	A05969	406
A00338	A05970	456	A00338	A05971	257
A00338	A06035	221	A00338	A06069	358
A00338	A06071	172	A00338	A06092	174
A00338	A06141	180	A00338	A06148	380
A00338	A06193	200	A00338	A06228	172
A00338	A06277	152	A00338	A06321	216
A00338	A06346	481	A00338	A06428	241
A00338	A06448	172	A00338	A06480	278
A00338	A06484	157	A00338	A06517	172
A00338	A06532	221	A00338	A06556	257
A00338	A06574	430	A00338	A06617	436
A00338	A06701	435	A00338	A06708	210
A00338	A06712	184	A00338	A06719	366
A00338	A06726	257	A00338	A06729	491
A00338	A06731	491	A00338	A06739	180
A00338	A06799	200	A00338	A06815	154
A00338	A06837	184	A00338	A06875	258
A00338	A06895	154	A00338	A06907	259
A00338	A06910	154	A00338	A06953	458
A00338	A06954	369	A00338	A07004	191
A00338	A07064	200	A00338	A07065	184
A00338	A07068	221	A00338	A07110	200
A00338	A07209	488	A00338	A07218	489
A00338	A07275	184	A00338	A07286	184

A00338	A07361	154	A00338	A07384	443
A00338	A07391	434	A00338	A07406	200
A00338	A07407	322	A00338	A07442	491
A00338	A07452	154	A00338	A07471	491
A00338	A07472	485	A00338	A07475	485
A00338	A07479	398	A00338	A07541	486
A00338	A07561	466	A00338	A07578	200
A00338	A07601	241	A00338	A07605	449
A00338	A07622	184	A00338	A07635	377
A00338	A07673	188	A00338	A07704	195
A00338	A07754	483	A00338	A07810	221
A00338	A07815	156	A00338	A07843	185
A00338	A07913	411	A00338	A07950	184
A00338	A07958	479	A00338	A07994	257
A00338	A08033	257	A00338	A08034	154
A00338	A08040	267	A00338	A08093	202
A00338	A08097	481	A00338	A08159	162
A00338	A08172	469	A00338	A08202	172
A00338	A08210	210	A00338	A08306	232
A00338	A08309	449	A00338	A08347	257
A00338	A08426	172	A00338	A08441	350
A00338	A08630	221	A00338	A08685	262
A00338	A08686	446	A00338	A08747	172
A00338	A08753	193	A00338	A08783	172
A00338	A08785	486	A00338	A08786	172
A00338	A09112	154	A00338	A09154	257
A00338	A09204	249	A00338	A09246	269
A00338	A09380	291	A00338	A09416	489
A00338	A09507	177	A00338	A09515	252
A00338	A09796	172	A00338	A09890	219
A00338	A10071	184	A00338	A10095	391
A00338	A10096	406	A00338	A10156	411
A00338	A10228	185	A00338	A10340	252
A00338	A10511	271	A00338	A10547	154
A00338	A10563	204	A00338	A10649	221
A00338	A10651	469	A00338	A10667	278
A00338	A10954	154	A00338	A10962	257
A00338	A10983	191	A00338	A11011	257
A00338	A11026	184	A00338	A11135	286
A00338	A11143	221	A00338	A11154	221
A00338	A11164	363	A00338	A11230	257
A00338	A11282	422	A00338	A11311	232
A00338	A11313	347	A00338	A11315	283
A00338	A11317	291	A00338	A11319	326
A00338	A11339	419	A00338	A11377	162
A00338	A11399	221	A00338	A11411	294

A00338	A11414	380	A00338	A11424	252
A00338	A11428	246	A00338	A11433	236
A00338	A11448	221	A00338	A11479	162
A00338	A11513	484	A00338	A11552	177
A00338	A11553	205	A00338	A11590	219
A00338	A11664	257	A00338	A11983	469
A00338	A12022	184	A00338	A12099	154
A00338	A12117	332	A00338	A12430	257
A00338	A12459	294	A00338	A12599	221
A00338	A12641	221	A00338	A12758	257
A00338	A12779	350	A00338	A12786	221
A00338	A12787	291	A00338	A13088	257
A00338	A13140	162	A00338	A13243	221
A00338	A13275	184	A00338	A13280	184
A00338	A13331	172	A00338	A13361	405
A00338	A13369	491	A00338	A13668	257
A00338	A13678	221	A00338	A13824	234
A00338	A13874	210	A00338	A14125	229
A00338	A14204	432	A00338	A14221	160
A00338	A14265	277	A00338	A14272	380
A00338	A14350	172	A00338	A14353	252
A00338	A14377	401	A00338	A14381	491
A00338	A14395	468	A00338	A14399	188
A00338	A14498	424	A00338	A14548	399
A00338	A14589	352	A00338	A14633	338
A00338	A14673	257	A00338	A14690	180
A00338	A14735	299	A00338	A14838	151
A00338	A15232	257	A00338	A15282	490
A00338	A15292	396	A00338	A15295	561
A00338	A15322	333	A00338	A15328	176
A00338	A15398	435	A00338	A15422	407
A00338	A15432	368	A00338	A15477	154
A00338	A15494	200	A00338	A15498	655
A00338	A15638	485	A00338	A15707	154
A00338	A15809	238	A00338	A15917	490
A00338	A15922	279	A00338	A16005	257
A00338	A16121	380	A00338	A16340	221
A00338	A16420	340	A00338	A16480	154
A00338	A16607	257	A00338	A17037	219
A00338	A17078	481	A00339	A00365	193
A00339	A00367	676	A00339	A00368	649
A00339	A00369	491	A00339	A00373	184
A00339	A00421	695	A00339	A00531	694
A00339	A00559	399	A00339	A00646	213
A00339	A00691	413	A00339	A00716	290
A00339	A00905	486	A00339	A00916	460

A00339	A01029	154	A00339	A01136	224
A00339	A01139	351	A00339	A01151	452
A00339	A01231	368	A00339	A01266	229
A00339	A01268	477	A00339	A01351	486
A00339	A01359	486	A00339	A01372	383
A00339	A01612	467	A00339	A03298	486
A00339	A03494	691	A00339	A03569	228
A00339	A03902	234	A00339	A06379	172
A00339	A08137	465	A00339	A08201	224
A00339	A08431	224	A00339	A08524	383
A00339	A08571	491	A00339	A08885	301
A00339	A08928	404	A00339	A08963	322
A00339	A09515	173	A00339	A09538	427
A00339	A09591	307	A00339	A09654	174
A00339	A10250	395	A00339	A10853	460
A00339	A11105	385	A00339	A11139	688
A00339	A11920	383	A00339	A13310	180
A00339	A13800	435	A00339	A13805	544
A00339	A14246	152	A00339	A14304	200
A00339	A14476	234	A00339	A14728	226
A00339	A15297	205	A00339	A15370	188
A00339	A15711	544	A00339	A16490	301
A00339	A16690	383	A00339	A16866	383
A00339	A16912	180	A00340	A00343	324
A00340	A00366	263	A00340	A00367	180
A00340	A00460	324	A00340	A00630	545
A00340	A00643	426	A00340	A00655	249
A00340	A00822	243	A00340	A00826	561
A00340	A00839	389	A00340	A00931	269
A00340	A00932	414	A00340	A00980	695
A00340	A01112	185	A00340	A01125	491
A00340	A01261	404	A00340	A01291	229
A00340	A01309	561	A00340	A01380	324
A00340	A01540	324	A00340	A01553	313
A00340	A01560	319	A00340	A01561	493
A00340	A01579	207	A00340	A01767	152
A00340	A01850	324	A00340	A01905	457
A00340	A01995	702	A00340	A02110	534
A00340	A02111	310	A00340	A02353	324
A00340	A02734	157	A00340	A02738	190
A00340	A03188	247	A00340	A03516	324
A00340	A03591	374	A00340	A03740	324
A00340	A03752	480	A00340	A03758	195
A00340	A03981	393	A00340	A04012	475
A00340	A04092	258	A00340	A04321	283
A00340	A04449	409	A00340	A04465	260

A00340	A04541	240	A00340	A04569	283
A00340	A05086	324	A00340	A05247	160
A00340	A05288	486	A00340	A05416	396
A00340	A06466	642	A00340	A06517	393
A00340	A06606	383	A00340	A07102	324
A00340	A07187	324	A00340	A07298	490
A00340	A07311	569	A00340	A07726	484
A00340	A07803	191	A00340	A07848	286
A00340	A08577	165	A00340	A08860	445
A00340	A08869	441	A00340	A09245	240
A00340	A09496	486	A00340	A09606	201
A00340	A10430	601	A00340	A10744	351
A00340	A11374	324	A00340	A11393	481
A00340	A11487	523	A00340	A11694	324
A00340	A11937	340	A00340	A11983	452
A00340	A13340	490	A00340	A14073	324
A00340	A14198	345	A00340	A14410	313
A00340	A14433	899	A00340	A14443	899
A00340	A14642	324	A00340	A15252	160
A00340	A15277	241	A00340	A15430	152
A00340	A15506	406	A00340	A15546	375
A00340	A15665	291	A00340	A15682	428
A00340	A15728	229	A00340	A15843	324
A00340	A15989	293	A00340	A16580	185
A00340	A16627	327	A00340	A16847	720
A00340	A16879	182	A00340	A16947	199
A00341	A00398	180	A00341	A00748	340
A00341	A00825	193	A00341	A00933	329
A00341	A01189	170	A00341	A06687	339
A00341	A07155	378	A00341	A09256	224
A00341	A13975	238	A00342	A00882	279
A00342	A01332	279	A00342	A01578	162
A00342	A02049	258	A00342	A08097	296
A00342	A11421	433	A00342	A14199	260
A00342	A14202	317	A00342	A14369	357
A00342	A14372	314	A00342	A14374	279
A00342	A14408	322	A00342	A14570	177
A00342	A14893	443	A00342	A15478	296
A00342	A16901	385	A00343	A00352	419
A00343	A00405	174	A00343	A00417	340
A00343	A00453	210	A00343	A00458	443
A00343	A00460	324	A00343	A00502	63
A00343	A00510	339	A00343	A00537	240
A00343	A00604	491	A00343	A00649	313
A00343	A00653	491	A00343	A00669	384
A00343	A00691	452	A00343	A00783	491

A00343	A00785	491	A00343	A00790	452
A00343	A00953	480	A00343	A00974	319
A00343	A01027	440	A00343	A01042	484
A00343	A01054	472	A00343	A01075	200
A00343	A01081	218	A00343	A01086	460
A00343	A01087	350	A00343	A01108	281
A00343	A01150	458	A00343	A01204	121
A00343	A01246	138	A00343	A01265	440
A00343	A01346	130	A00343	A01380	324
A00343	A01399	388	A00343	A01446	457
A00343	A01448	333	A00343	A01540	619
A00343	A01574	213	A00343	A01576	198
A00343	A01850	324	A00343	A02185	162
A00343	A02353	324	A00343	A03070	491
A00343	A03293	474	A00343	A03298	428
A00343	A03516	324	A00343	A03740	627
A00343	A03776	279	A00343	A03795	205
A00343	A03959	152	A00343	A05086	324
A00343	A05387	356	A00343	A05839	404
A00343	A06959	193	A00343	A07102	324
A00343	A07187	324	A00343	A07706	263
A00343	A08371	438	A00343	A08571	380
A00343	A08835	398	A00343	A08885	240
A00343	A09591	259	A00343	A09720	266
A00343	A11296	457	A00343	A11322	477
A00343	A11337	362	A00343	A11338	486
A00343	A11374	621	A00343	A11427	489
A00343	A11612	263	A00343	A11694	324
A00343	A11836	221	A00343	A11930	157
A00343	A12241	486	A00343	A13261	271
A00343	A14073	393	A00343	A14461	201
A00343	A14471	297	A00343	A14642	324
A00343	A14845	276	A00343	A15843	324
A00343	A16371	154	A00343	A17128	483
A00344	A15606	63	A00345	A00454	236
A00345	A01042	243	A00345	A01075	170
A00345	A01083	337	A00345	A03740	219
A00346	A00359	306	A00346	A00469	263
A00346	A00482	207	A00346	A00531	160
A00346	A01003	228	A00346	A01011	327
A00346	A01920	241	A00346	A03166	363
A00346	A03361	159	A00346	A04342	247
A00346	A10619	278	A00346	A11604	173
A00346	A14044	357	A00347	A00418	443
A00347	A00470	605	A00347	A00473	466
A00347	A00565	165	A00347	A00605	296

A00347	A00637	276	A00347	A00688	252
A00347	A00922	249	A00347	A00987	267
A00347	A01046	199	A00347	A01117	472
A00347	A01124	465	A00347	A01172	486
A00347	A01214	224	A00347	A01215	393
A00347	A01244	414	A00347	A01320	468
A00347	A01322	347	A00347	A01325	450
A00347	A01492	245	A00347	A01530	283
A00347	A01548	485	A00347	A01596	741
A00347	A01615	232	A00347	A01775	899
A00347	A01930	184	A00347	A01993	217
A00347	A02104	193	A00347	A02144	217
A00347	A02147	177	A00347	A02221	172
A00347	A02424	347	A00347	A02425	213
A00347	A02487	190	A00347	A02494	63
A00347	A02557	277	A00347	A02673	267
A00347	A02687	427	A00347	A02688	216
A00347	A03044	291	A00347	A03046	238
A00347	A03090	193	A00347	A03115	224
A00347	A03152	171	A00347	A03193	481
A00347	A03213	207	A00347	A03434	489
A00347	A04473	160	A00347	A04911	151
A00347	A05032	191	A00347	A05297	345
A00347	A05690	468	A00347	A05825	205
A00347	A05966	526	A00347	A06039	199
A00347	A06137	407	A00347	A06212	249
A00347	A06423	205	A00347	A06466	605
A00347	A06494	252	A00347	A06610	202
A00347	A06822	277	A00347	A07014	154
A00347	A07062	475	A00347	A07207	270
A00347	A07311	333	A00347	A07706	199
A00347	A07848	219	A00347	A08133	199
A00347	A08209	317	A00347	A08863	276
A00347	A08884	167	A00347	A09195	199
A00347	A09390	152	A00347	A09652	201
A00347	A09727	176	A00347	A09826	435
A00347	A09854	198	A00347	A10039	168
A00347	A10580	180	A00347	A10671	201
A00347	A11250	205	A00347	A11376	276
A00347	A11379	229	A00347	A11467	228
A00347	A11483	221	A00347	A11843	276
A00347	A11851	276	A00347	A11937	199
A00347	A12215	176	A00347	A13272	252
A00347	A13311	160	A00347	A13356	276
A00347	A13726	383	A00347	A14200	152
A00347	A14452	321	A00347	A14888	176

A00347	A14932	191	A00347	A15315	165
A00347	A15347	171	A00347	A15480	276
A00347	A15896	408	A00347	A16038	252
A00347	A16707	263	A00347	A16942	187
A00348	A00351	291	A00348	A00352	301
A00348	A00368	423	A00348	A00371	480
A00348	A00378	428	A00348	A00420	173
A00348	A00421	235	A00348	A00436	477
A00348	A00439	335	A00348	A00454	489
A00348	A00458	491	A00348	A00489	353
A00348	A00496	393	A00348	A00501	283
A00348	A00517	463	A00348	A00527	450
A00348	A00531	491	A00348	A00532	491
A00348	A00537	446	A00348	A00560	384
A00348	A00589	256	A00348	A00605	413
A00348	A00609	483	A00348	A00626	230
A00348	A00640	191	A00348	A00647	317
A00348	A00655	484	A00348	A00697	296
A00348	A00707	438	A00348	A00733	156
A00348	A00741	251	A00348	A00758	329
A00348	A00781	235	A00348	A00793	200
A00348	A00819	463	A00348	A00822	491
A00348	A00831	390	A00348	A00833	461
A00348	A00835	199	A00348	A00839	227
A00348	A00841	257	A00348	A00843	382
A00348	A00848	467	A00348	A00871	474
A00348	A00883	491	A00348	A00905	475
A00348	A00909	229	A00348	A00916	490
A00348	A00922	467	A00348	A00932	374
A00348	A00933	305	A00348	A00935	382
A00348	A00940	218	A00348	A00958	433
A00348	A00970	319	A00348	A01031	350
A00348	A01032	363	A00348	A01033	276
A00348	A01042	177	A00348	A01051	286
A00348	A01052	246	A00348	A01057	212
A00348	A01069	490	A00348	A01075	491
A00348	A01077	481	A00348	A01082	441
A00348	A01083	285	A00348	A01084	414
A00348	A01087	475	A00348	A01091	177
A00348	A01115	472	A00348	A01150	462
A00348	A01194	350	A00348	A01206	477
A00348	A01211	195	A00348	A01215	486
A00348	A01234	486	A00348	A01242	283
A00348	A01243	274	A00348	A01261	479
A00348	A01268	291	A00348	A01291	485
A00348	A01299	382	A00348	A01335	215

A00348	A01353	201	A00348	A01354	730
A00348	A01385	486	A00348	A01439	486
A00348	A01470	488	A00348	A01539	307
A00348	A01540	488	A00348	A01541	730
A00348	A01558	416	A00348	A01560	353
A00348	A01569	480	A00348	A01572	484
A00348	A01577	479	A00348	A01598	491
A00348	A01612	436	A00348	A01613	270
A00348	A01649	491	A00348	A01655	200
A00348	A01707	491	A00348	A01763	470
A00348	A01770	449	A00348	A01781	314
A00348	A01822	609	A00348	A02111	156
A00348	A02219	485	A00348	A02446	201
A00348	A02470	294	A00348	A02715	388
A00348	A02765	216	A00348	A02784	185
A00348	A02797	277	A00348	A02916	154
A00348	A03057	212	A00348	A03261	247
A00348	A03298	171	A00348	A03446	486
A00348	A03560	328	A00348	A03569	286
A00348	A03638	391	A00348	A03654	490
A00348	A03740	491	A00348	A03752	489
A00348	A03817	454	A00348	A03820	491
A00348	A03881	179	A00348	A03977	318
A00348	A03992	215	A00348	A04288	152
A00348	A04561	162	A00348	A04562	386
A00348	A04814	179	A00348	A04944	375
A00348	A05120	219	A00348	A05122	463
A00348	A05928	238	A00348	A06182	85
A00348	A06198	272	A00348	A06234	162
A00348	A06377	216	A00348	A06466	491
A00348	A06907	279	A00348	A07311	550
A00348	A07604	456	A00348	A07706	484
A00348	A07798	418	A00348	A08054	378
A00348	A08118	457	A00348	A08137	491
A00348	A08178	226	A00348	A08280	249
A00348	A08359	372	A00348	A08377	256
A00348	A08543	257	A00348	A08550	313
A00348	A08552	193	A00348	A08561	257
A00348	A08571	491	A00348	A08805	157
A00348	A08996	204	A00348	A09194	417
A00348	A09269	427	A00348	A09609	350
A00348	A09621	650	A00348	A09655	238
A00348	A09768	154	A00348	A09857	333
A00348	A10165	219	A00348	A10321	362
A00348	A10399	477	A00348	A10532	182
A00348	A10624	200	A00348	A10642	447

A00348	A10789	217	A00348	A10853	418
A00348	A10935	151	A00348	A11132	160
A00348	A11286	467	A00348	A11297	374
A00348	A11301	268	A00348	A11302	490
A00348	A11324	724	A00348	A11344	219
A00348	A11372	475	A00348	A11374	160
A00348	A11387	154	A00348	A11472	446
A00348	A11675	409	A00348	A11735	329
A00348	A11833	463	A00348	A11914	263
A00348	A11940	210	A00348	A11954	201
A00348	A12215	235	A00348	A12328	465
A00348	A12506	215	A00348	A12507	288
A00348	A12606	257	A00348	A12658	383
A00348	A12826	238	A00348	A12858	266
A00348	A13116	162	A00348	A13128	460
A00348	A13311	157	A00348	A13335	435
A00348	A13340	462	A00348	A13696	238
A00348	A13708	257	A00348	A13784	195
A00348	A13786	221	A00348	A13787	290
A00348	A13788	188	A00348	A13809	273
A00348	A13870	162	A00348	A13912	272
A00348	A13989	491	A00348	A14006	491
A00348	A14073	485	A00348	A14196	465
A00348	A14214	294	A00348	A14220	458
A00348	A14221	157	A00348	A14257	339
A00348	A14270	254	A00348	A14286	184
A00348	A14351	416	A00348	A14399	195
A00348	A14449	243	A00348	A14467	455
A00348	A14476	236	A00348	A14551	170
A00348	A14566	255	A00348	A14569	283
A00348	A14596	480	A00348	A14659	168
A00348	A14739	170	A00348	A14826	308
A00348	A14921	157	A00348	A15116	283
A00348	A15244	407	A00348	A15261	488
A00348	A15507	467	A00348	A15541	221
A00348	A15653	216	A00348	A15660	430
A00348	A15680	230	A00348	A15684	305
A00348	A15709	303	A00348	A15711	247
A00348	A15734	152	A00348	A15755	179
A00348	A15932	162	A00348	A16060	443
A00348	A16096	216	A00348	A16157	435
A00348	A16243	196	A00348	A16320	313
A00348	A16410	451	A00348	A16565	243
A00348	A16566	205	A00348	A16567	238
A00348	A16611	274	A00348	A16664	154
A00348	A16675	172	A00348	A16764	486

A00348	A16776	314	A00348	A16788	346
A00348	A16829	299	A00348	A16874	191
A00348	A16908	170	A00348	A17019	157
A00348	A17142	266	A00348	A17144	168
A00348	A17230	157	A00349	A00601	238
A00349	A00815	226	A00349	A01125	180
A00349	A01137	363	A00349	A01257	446
A00349	A01547	344	A00349	A02148	227
A00349	A02312	355	A00349	A02425	273
A00349	A02531	355	A00349	A02559	270
A00349	A02673	347	A00349	A02759	324
A00349	A03219	302	A00349	A03661	241
A00349	A03961	296	A00349	A04256	454
A00349	A05533	413	A00349	A06688	229
A00349	A13875	312	A00349	A15233	357
A00350	A00351	361	A00350	A00478	355
A00350	A00491	251	A00350	A00509	390
A00350	A00531	648	A00350	A00556	165
A00350	A00567	269	A00350	A00568	483
A00350	A00573	210	A00350	A00577	193
A00350	A00611	382	A00350	A00616	338
A00350	A00659	185	A00350	A00664	229
A00350	A00841	326	A00350	A00846	185
A00350	A00916	332	A00350	A01137	409
A00350	A01404	324	A00350	A01485	230
A00350	A01492	281	A00350	A01567	446
A00350	A01590	434	A00350	A01604	173
A00350	A01698	249	A00350	A01716	190
A00350	A01899	296	A00350	A01926	378
A00350	A02083	470	A00350	A02111	416
A00350	A02122	201	A00350	A02132	313
A00350	A02138	241	A00350	A02156	483
A00350	A02222	278	A00350	A02251	152
A00350	A02260	218	A00350	A02262	241
A00350	A02268	223	A00350	A02272	235
A00350	A02299	285	A00350	A02306	474
A00350	A02309	335	A00350	A02314	313
A00350	A02317	230	A00350	A02330	112
A00350	A02415	157	A00350	A02454	390
A00350	A02524	302	A00350	A02544	162
A00350	A02564	190	A00350	A02574	290
A00350	A02644	481	A00350	A02698	414
A00350	A02759	176	A00350	A02972	191
A00350	A03019	221	A00350	A03034	276
A00350	A03036	196	A00350	A03086	229
A00350	A03099	486	A00350	A03173	340

A00350	A03238	440	A00350	A03498	351
A00350	A03919	232	A00350	A03952	367
A00350	A03968	238	A00350	A04086	204
A00350	A04256	173	A00350	A04326	302
A00350	A04439	307	A00350	A04975	274
A00350	A04981	249	A00350	A05098	188
A00350	A05157	215	A00350	A05208	468
A00350	A05376	177	A00350	A05653	272
A00350	A05812	156	A00350	A05934	307
A00350	A06067	165	A00350	A06253	335
A00350	A06471	396	A00350	A06503	340
A00350	A06922	321	A00350	A07140	477
A00350	A07311	176	A00350	A07324	176
A00350	A07336	260	A00350	A07342	334
A00350	A07655	351	A00350	A07746	180
A00350	A07878	296	A00350	A08137	322
A00350	A08660	185	A00350	A08688	351
A00350	A10635	156	A00350	A13434	272
A00350	A14209	346	A00350	A14220	462
A00350	A14344	224	A00350	A14430	163
A00350	A14451	188	A00350	A14735	246
A00350	A15718	198	A00350	A16087	157
A00351	A00449	422	A00351	A00472	396
A00351	A00478	469	A00351	A00601	297
A00351	A00659	481	A00351	A00662	665
A00351	A00663	288	A00351	A00784	183
A00351	A00794	263	A00351	A00868	221
A00351	A00981	435	A00351	A01005	481
A00351	A01137	361	A00351	A01404	491
A00351	A01425	63	A00351	A01460	484
A00351	A01461	491	A00351	A01485	347
A00351	A01515	491	A00351	A01573	491
A00351	A01583	205	A00351	A01585	389
A00351	A01635	346	A00351	A01710	286
A00351	A01737	168	A00351	A01741	335
A00351	A01743	168	A00351	A01848	213
A00351	A01923	374	A00351	A01924	200
A00351	A01926	491	A00351	A01929	207
A00351	A01937	208	A00351	A01978	191
A00351	A02008	306	A00351	A02083	329
A00351	A02111	483	A00351	A02156	419
A00351	A02167	190	A00351	A02174	329
A00351	A02222	319	A00351	A02229	156
A00351	A02275	227	A00351	A02287	193
A00351	A02306	297	A00351	A02317	182
A00351	A02330	401	A00351	A02336	196

A00351	A02356	398	A00351	A02405	489
A00351	A02410	341	A00351	A02428	235
A00351	A02442	283	A00351	A02484	182
A00351	A02524	344	A00351	A02574	332
A00351	A02601	213	A00351	A02669	196
A00351	A02738	457	A00351	A02859	416
A00351	A03007	316	A00351	A03034	486
A00351	A03099	485	A00351	A03104	173
A00351	A03134	157	A00351	A03144	198
A00351	A03147	159	A00351	A03238	491
A00351	A03513	165	A00351	A03523	374
A00351	A03545	234	A00351	A03609	165
A00351	A03892	187	A00351	A03919	184
A00351	A03926	177	A00351	A03961	307
A00351	A03968	173	A00351	A04051	160
A00351	A04087	193	A00351	A04092	411
A00351	A04232	168	A00351	A04258	374
A00351	A04318	356	A00351	A04340	268
A00351	A04437	174	A00351	A04487	372
A00351	A04568	162	A00351	A04646	306
A00351	A04679	447	A00351	A04839	460
A00351	A04926	346	A00351	A05112	199
A00351	A05208	184	A00351	A05311	454
A00351	A05330	160	A00351	A05376	491
A00351	A05459	425	A00351	A05471	162
A00351	A05573	330	A00351	A05620	236
A00351	A05629	204	A00351	A05653	226
A00351	A05751	151	A00351	A05829	274
A00351	A05923	179	A00351	A06258	435
A00351	A06574	358	A00351	A06769	291
A00351	A06831	469	A00351	A07141	329
A00351	A07184	184	A00351	A07218	291
A00351	A07342	152	A00351	A07407	156
A00351	A07438	193	A00351	A07704	258
A00351	A07705	343	A00351	A07726	288
A00351	A07753	184	A00351	A08292	168
A00351	A08671	486	A00351	A09036	411
A00351	A09174	213	A00351	A09277	441
A00351	A09426	293	A00351	A09461	177
A00351	A10052	443	A00351	A10078	299
A00351	A10091	406	A00351	A10410	306
A00351	A10548	306	A00351	A10967	321
A00351	A11279	473	A00351	A11310	191
A00351	A11328	198	A00351	A11329	414
A00351	A11343	372	A00351	A11684	200
A00351	A11709	407	A00351	A12069	258

A00351	A12824	313	A00351	A12982	236
A00351	A13871	345	A00351	A13987	389
A00351	A14202	196	A00351	A14247	491
A00351	A14258	375	A00351	A14344	308
A00351	A14430	187	A00351	A14661	462
A00351	A15230	436	A00351	A15293	408
A00351	A15301	335	A00351	A15366	490
A00351	A15430	238	A00351	A15462	452
A00351	A15463	461	A00351	A15498	417
A00351	A15506	352	A00351	A16150	322
A00351	A16337	322	A00351	A16368	260
A00351	A16503	241	A00351	A16751	245
A00352	A00356	171	A00352	A00377	543
A00352	A00378	706	A00352	A00383	135
A00352	A00400	508	A00352	A00421	491
A00352	A00438	491	A00352	A00442	467
A00352	A00446	461	A00352	A00447	172
A00352	A00454	490	A00352	A00457	480
A00352	A00460	362	A00352	A00485	188
A00352	A00494	187	A00352	A00500	419
A00352	A00523	416	A00352	A00531	640
A00352	A00532	489	A00352	A00537	871
A00352	A00538	491	A00352	A00539	467
A00352	A00561	439	A00352	A00565	246
A00352	A00566	156	A00352	A00571	490
A00352	A00598	491	A00352	A00604	491
A00352	A00609	229	A00352	A00638	262
A00352	A00648	475	A00352	A00649	318
A00352	A00653	557	A00352	A00655	428
A00352	A00680	430	A00352	A00687	491
A00352	A00688	235	A00352	A00693	491
A00352	A00694	272	A00352	A00697	636
A00352	A00698	356	A00352	A00741	491
A00352	A00742	486	A00352	A00758	491
A00352	A00769	228	A00352	A00778	330
A00352	A00783	369	A00352	A00785	378
A00352	A00787	443	A00352	A00790	257
A00352	A00805	299	A00352	A00809	588
A00352	A00821	475	A00352	A00839	489
A00352	A00841	557	A00352	A00849	488
A00352	A00856	418	A00352	A00865	227
A00352	A00868	438	A00352	A00871	652
A00352	A00878	490	A00352	A00891	182
A00352	A00909	294	A00352	A00911	574
A00352	A00922	457	A00352	A00935	627
A00352	A00936	168	A00352	A00953	491

A00352	A00975	290	A00352	A00984	362
A00352	A00985	695	A00352	A00987	456
A00352	A00998	249	A00352	A01020	345
A00352	A01022	180	A00352	A01024	694
A00352	A01026	278	A00352	A01027	491
A00352	A01042	452	A00352	A01050	424
A00352	A01075	490	A00352	A01082	477
A00352	A01086	602	A00352	A01100	592
A00352	A01106	173	A00352	A01108	382
A00352	A01135	184	A00352	A01150	659
A00352	A01155	469	A00352	A01156	430
A00352	A01161	745	A00352	A01169	490
A00352	A01192	555	A00352	A01210	491
A00352	A01212	391	A00352	A01214	666
A00352	A01215	227	A00352	A01218	172
A00352	A01226	400	A00352	A01232	597
A00352	A01234	542	A00352	A01261	452
A00352	A01287	457	A00352	A01296	293
A00352	A01307	347	A00352	A01311	561
A00352	A01320	298	A00352	A01321	188
A00352	A01322	160	A00352	A01326	477
A00352	A01327	355	A00352	A01329	369
A00352	A01331	371	A00352	A01336	491
A00352	A01347	235	A00352	A01352	193
A00352	A01365	418	A00352	A01385	491
A00352	A01399	386	A00352	A01406	232
A00352	A01413	491	A00352	A01435	609
A00352	A01436	159	A00352	A01447	491
A00352	A01451	461	A00352	A01483	360
A00352	A01502	251	A00352	A01504	174
A00352	A01507	171	A00352	A01539	190
A00352	A01540	491	A00352	A01542	400
A00352	A01559	204	A00352	A01571	246
A00352	A01573	470	A00352	A01576	457
A00352	A01578	210	A00352	A01582	200
A00352	A01596	491	A00352	A01612	160
A00352	A01626	293	A00352	A01627	249
A00352	A01707	486	A00352	A01711	430
A00352	A01741	263	A00352	A01815	263
A00352	A01824	443	A00352	A01827	491
A00352	A01850	401	A00352	A01937	159
A00352	A01988	267	A00352	A02030	418
A00352	A02040	472	A00352	A02225	182
A00352	A02227	230	A00352	A02607	491
A00352	A02813	193	A00352	A02994	491
A00352	A02996	251	A00352	A02999	152

A00352	A03259	156	A00352	A03328	314
A00352	A03494	479	A00352	A03512	330
A00352	A03651	708	A00352	A03676	470
A00352	A03740	489	A00352	A03752	364
A00352	A03798	355	A00352	A03809	242
A00352	A03930	184	A00352	A03959	475
A00352	A04508	184	A00352	A04668	314
A00352	A04777	251	A00352	A04782	424
A00352	A04838	484	A00352	A05144	210
A00352	A05417	491	A00352	A05449	305
A00352	A05812	660	A00352	A05882	184
A00352	A05891	399	A00352	A06048	221
A00352	A06287	172	A00352	A06379	63
A00352	A06466	460	A00352	A06545	414
A00352	A06546	486	A00352	A06750	367
A00352	A06752	400	A00352	A06754	221
A00352	A07160	488	A00352	A07218	484
A00352	A07394	491	A00352	A07480	183
A00352	A07527	232	A00352	A07670	243
A00352	A07695	486	A00352	A07706	443
A00352	A07767	514	A00352	A07845	221
A00352	A07846	221	A00352	A07882	263
A00352	A07887	281	A00352	A08011	207
A00352	A08014	221	A00352	A08073	146
A00352	A08144	190	A00352	A08222	400
A00352	A08319	446	A00352	A08519	475
A00352	A08524	252	A00352	A08863	206
A00352	A08882	398	A00352	A08926	85
A00352	A09054	485	A00352	A09475	184
A00352	A09526	198	A00352	A09581	364
A00352	A09709	477	A00352	A09902	182
A00352	A10039	152	A00352	A10154	98
A00352	A10250	207	A00352	A10385	221
A00352	A10473	162	A00352	A10487	221
A00352	A10535	439	A00352	A10598	486
A00352	A10853	160	A00352	A11307	296
A00352	A11313	659	A00352	A11452	235
A00352	A11526	458	A00352	A11795	156
A00352	A11833	472	A00352	A11860	232
A00352	A12027	184	A00352	A12215	345
A00352	A12302	485	A00352	A12673	221
A00352	A12703	221	A00352	A12746	184
A00352	A12795	221	A00352	A12824	457
A00352	A13037	221	A00352	A13077	221
A00352	A13116	263	A00352	A13146	238
A00352	A13251	263	A00352	A13273	221

A00352	A13340	458	A00352	A13344	162
A00352	A13398	182	A00352	A13446	184
A00352	A13576	317	A00352	A13653	221
A00352	A13721	221	A00352	A13754	221
A00352	A13809	440	A00352	A13870	324
A00352	A14006	488	A00352	A14073	573
A00352	A14074	179	A00352	A14077	321
A00352	A14080	485	A00352	A14178	424
A00352	A14272	477	A00352	A14394	162
A00352	A14449	530	A00352	A14507	277
A00352	A14562	396	A00352	A14566	488
A00352	A14642	388	A00352	A14655	491
A00352	A14699	658	A00352	A14701	461
A00352	A14845	343	A00352	A15077	221
A00352	A15081	221	A00352	A15161	221
A00352	A15321	309	A00352	A15347	353
A00352	A15498	401	A00352	A15617	419
A00352	A15653	627	A00352	A15666	491
A00352	A15765	486	A00352	A15932	490
A00352	A15933	228	A00352	A16007	491
A00352	A16060	374	A00352	A16157	486
A00352	A16400	228	A00352	A16403	319
A00352	A16430	201	A00352	A16626	400
A00352	A16683	263	A00353	A00411	260
A00353	A00438	490	A00353	A00517	435
A00353	A00552	177	A00353	A00642	251
A00353	A00835	335	A00353	A00849	243
A00353	A00863	196	A00353	A00967	491
A00353	A00993	299	A00353	A01537	432
A00353	A01598	151	A00353	A01632	182
A00353	A01655	152	A00353	A01941	162
A00353	A02121	157	A00353	A02376	382
A00353	A02465	236	A00353	A02481	190
A00353	A03049	433	A00353	A03126	369
A00353	A03265	481	A00353	A03278	314
A00353	A03927	333	A00353	A03981	259
A00353	A04076	240	A00353	A04145	278
A00353	A04945	218	A00353	A05468	246
A00353	A05543	316	A00353	A05654	276
A00353	A06269	243	A00353	A06660	167
A00353	A09911	219	A00353	A11070	268
A00353	A11318	199	A00353	A11331	260
A00353	A11452	156	A00353	A11530	353
A00353	A11566	303	A00353	A11576	213
A00353	A11726	316	A00353	A11986	159
A00353	A12207	286	A00353	A12660	188

A00353	A12740	353	A00353	A13023	157
A00353	A13626	185	A00353	A14006	443
A00353	A14646	159	A00353	A16279	303
A00353	A16410	221	A00354	A00509	475
A00354	A01591	160	A00354	A02268	308
A00354	A05106	340	A00354	A07140	338
A00354	A07815	317	A00354	A07878	350
A00354	A08660	363	A00354	A14430	273
A00355	A00357	430	A00355	A00358	491
A00355	A00403	491	A00355	A00469	313
A00355	A00501	260	A00355	A00521	565
A00355	A00590	477	A00355	A00629	484
A00355	A00648	327	A00355	A00787	401
A00355	A00831	350	A00355	A00839	180
A00355	A00848	156	A00355	A00883	473
A00355	A00916	337	A00355	A00923	491
A00355	A00965	278	A00355	A00970	193
A00355	A00990	200	A00355	A01000	491
A00355	A01017	483	A00355	A01025	491
A00355	A01047	565	A00355	A01066	530
A00355	A01096	213	A00355	A01101	491
A00355	A01120	435	A00355	A01140	452
A00355	A01166	374	A00355	A01184	490
A00355	A01189	395	A00355	A01206	480
A00355	A01234	490	A00355	A01243	168
A00355	A01249	559	A00355	A01263	417
A00355	A01264	461	A00355	A01266	154
A00355	A01282	430	A00355	A01291	566
A00355	A01292	236	A00355	A01306	491
A00355	A01325	249	A00355	A01338	566
A00355	A01425	256	A00355	A01429	421
A00355	A01430	281	A00355	A01437	491
A00355	A01464	547	A00355	A01468	193
A00355	A01470	483	A00355	A01598	171
A00355	A01612	486	A00355	A01614	377
A00355	A01617	319	A00355	A01618	312
A00355	A01649	491	A00355	A01700	311
A00355	A01718	63	A00355	A01997	163
A00355	A02216	221	A00355	A02355	200
A00355	A02738	299	A00355	A02978	562
A00355	A02980	268	A00355	A03069	491
A00355	A03272	159	A00355	A03567	491
A00355	A03731	221	A00355	A03754	485
A00355	A03767	168	A00355	A03859	485
A00355	A04430	259	A00355	A05385	345
A00355	A05501	419	A00355	A06279	79

A00355	A06812	265	A00355	A06861	454
A00355	A06897	273	A00355	A06965	491
A00355	A06973	552	A00355	A07348	91
A00355	A07451	461	A00355	A07533	234
A00355	A08011	288	A00355	A08055	358
A00355	A08137	491	A00355	A08357	456
A00355	A08398	310	A00355	A08419	327
A00355	A08452	565	A00355	A08521	200
A00355	A08547	343	A00355	A08558	518
A00355	A08664	200	A00355	A08972	319
A00355	A09044	205	A00355	A09322	491
A00355	A09674	193	A00355	A09702	545
A00355	A10417	200	A00355	A10439	249
A00355	A10529	457	A00355	A10616	260
A00355	A11316	162	A00355	A11390	491
A00355	A11438	553	A00355	A11480	488
A00355	A11704	491	A00355	A11729	238
A00355	A11945	491	A00355	A12233	490
A00355	A12243	418	A00355	A12270	160
A00355	A12667	157	A00355	A13333	200
A00355	A13709	286	A00355	A13710	294
A00355	A13989	484	A00355	A14188	461
A00355	A14216	338	A00355	A14505	420
A00355	A15218	462	A00355	A15219	408
A00355	A15221	486	A00355	A15235	343
A00355	A15727	427	A00355	A15743	172
A00355	A15776	441	A00355	A15777	469
A00355	A15778	182	A00355	A16118	484
A00355	A16523	259	A00355	A16631	491
A00355	A16682	188	A00355	A16823	249
A00356	A00357	462	A00356	A00358	489
A00356	A00360	443	A00356	A00361	440
A00356	A00378	177	A00356	A00400	165
A00356	A00414	432	A00356	A00467	480
A00356	A00521	486	A00356	A00537	188
A00356	A00561	319	A00356	A00571	420
A00356	A00590	491	A00356	A00603	491
A00356	A00604	395	A00356	A00605	460
A00356	A00629	489	A00356	A00648	490
A00356	A00651	491	A00356	A00653	466
A00356	A00663	262	A00356	A00694	384
A00356	A00697	294	A00356	A00700	491
A00356	A00701	491	A00356	A00713	450
A00356	A00763	491	A00356	A00787	580
A00356	A00806	408	A00356	A00812	452
A00356	A00839	491	A00356	A00841	229

A00356	A00849	165	A00356	A00851	374
A00356	A00870	430	A00356	A00875	274
A00356	A00878	491	A00356	A00900	401
A00356	A00902	224	A00356	A00906	208
A00356	A00923	491	A00356	A00932	424
A00356	A00965	262	A00356	A00970	162
A00356	A00972	479	A00356	A01000	477
A00356	A01025	455	A00356	A01037	469
A00356	A01042	259	A00356	A01047	219
A00356	A01056	693	A00356	A01072	412
A00356	A01090	486	A00356	A01095	475
A00356	A01098	436	A00356	A01101	419
A00356	A01112	491	A00356	A01115	249
A00356	A01121	272	A00356	A01127	694
A00356	A01140	491	A00356	A01178	152
A00356	A01184	419	A00356	A01206	299
A00356	A01212	491	A00356	A01216	473
A00356	A01240	63	A00356	A01249	486
A00356	A01291	988	A00356	A01306	473
A00356	A01335	301	A00356	A01347	290
A00356	A01392	160	A00356	A01425	446
A00356	A01431	310	A00356	A01435	313
A00356	A01437	396	A00356	A01451	229
A00356	A01457	491	A00356	A01458	486
A00356	A01464	180	A00356	A01468	491
A00356	A01492	539	A00356	A01496	489
A00356	A01527	899	A00356	A01540	243
A00356	A01560	454	A00356	A01579	491
A00356	A01586	394	A00356	A01598	157
A00356	A01601	797	A00356	A01605	405
A00356	A01649	424	A00356	A01684	165
A00356	A01718	491	A00356	A02250	281
A00356	A02265	176	A00356	A02494	474
A00356	A02818	247	A00356	A02928	236
A00356	A03069	472	A00356	A03158	281
A00356	A03331	174	A00356	A03332	491
A00356	A03445	439	A00356	A03567	490
A00356	A03652	182	A00356	A03939	432
A00356	A03959	215	A00356	A03981	488
A00356	A04230	273	A00356	A04446	160
A00356	A04754	469	A00356	A04761	491
A00356	A04846	283	A00356	A05044	258
A00356	A05076	328	A00356	A05090	263
A00356	A05411	485	A00356	A05443	491
A00356	A05449	396	A00356	A05996	491
A00356	A06037	409	A00356	A06279	470

A00356	A06351	364	A00356	A06664	191
A00356	A06814	747	A00356	A06861	344
A00356	A06965	488	A00356	A07004	193
A00356	A07140	290	A00356	A07191	160
A00356	A07243	274	A00356	A07311	507
A00356	A07398	778	A00356	A07449	340
A00356	A07451	491	A00356	A07517	219
A00356	A07726	443	A00356	A07766	151
A00356	A08011	491	A00356	A08052	433
A00356	A08055	396	A00356	A08061	419
A00356	A08315	297	A00356	A08321	243
A00356	A08357	377	A00356	A08398	252
A00356	A08452	472	A00356	A08521	235
A00356	A08773	449	A00356	A09044	419
A00356	A09313	230	A00356	A09332	465
A00356	A09581	162	A00356	A09585	294
A00356	A09591	324	A00356	A09702	65
A00356	A09704	171	A00356	A09960	183
A00356	A10234	183	A00356	A10650	183
A00356	A10949	245	A00356	A11190	339
A00356	A11263	223	A00356	A11331	260
A00356	A11390	171	A00356	A11438	210
A00356	A11612	329	A00356	A11940	185
A00356	A11981	296	A00356	A12028	183
A00356	A12109	219	A00356	A12168	374
A00356	A12310	219	A00356	A12643	257
A00356	A12813	152	A00356	A12970	152
A00356	A12990	419	A00356	A13107	205
A00356	A13340	271	A00356	A13470	160
A00356	A13626	338	A00356	A13830	268
A00356	A13989	838	A00356	A13992	491
A00356	A14216	317	A00356	A14386	188
A00356	A14505	368	A00356	A15660	443
A00356	A15684	473	A00356	A15971	285
A00356	A15988	480	A00356	A16025	368
A00356	A16118	391	A00356	A16159	380
A00357	A00358	490	A00357	A00360	293
A00357	A00361	252	A00357	A00362	319
A00357	A00403	241	A00357	A00454	303
A00357	A00467	160	A00357	A00521	489
A00357	A00590	443	A00357	A00595	208
A00357	A00603	441	A00357	A00604	232
A00357	A00605	458	A00357	A00629	491
A00357	A00648	457	A00357	A00651	468
A00357	A00653	344	A00357	A00700	462
A00357	A00701	491	A00357	A00713	452

A00357	A00763	481	A00357	A00783	208
A00357	A00787	469	A00357	A00839	486
A00357	A00870	240	A00357	A00878	170
A00357	A00910	229	A00357	A00923	491
A00357	A00990	483	A00357	A01000	489
A00357	A01020	162	A00357	A01025	486
A00357	A01042	401	A00357	A01047	491
A00357	A01056	417	A00357	A01072	344
A00357	A01081	232	A00357	A01090	291
A00357	A01101	224	A00357	A01112	451
A00357	A01127	491	A00357	A01140	475
A00357	A01150	318	A00357	A01166	330
A00357	A01184	483	A00357	A01189	424
A00357	A01206	439	A00357	A01234	441
A00357	A01240	449	A00357	A01249	491
A00357	A01291	491	A00357	A01304	167
A00357	A01306	489	A00357	A01338	63
A00357	A01431	221	A00357	A01437	246
A00357	A01464	490	A00357	A01468	435
A00357	A01470	281	A00357	A01496	469
A00357	A01579	489	A00357	A01586	167
A00357	A01601	210	A00357	A01609	188
A00357	A01617	480	A00357	A01618	491
A00357	A01619	472	A00357	A01620	458
A00357	A01649	396	A00357	A01718	483
A00357	A01822	279	A00357	A02003	177
A00357	A02068	172	A00357	A03048	316
A00357	A03069	489	A00357	A03437	458
A00357	A03567	491	A00357	A03645	288
A00357	A03705	165	A00357	A03740	322
A00357	A03754	491	A00357	A03888	386
A00357	A03981	303	A00357	A04761	404
A00357	A05044	201	A00357	A05121	167
A00357	A05385	241	A00357	A05464	317
A00357	A05501	334	A00357	A06279	447
A00357	A06351	329	A00357	A06861	480
A00357	A06965	491	A00357	A06973	483
A00357	A07201	490	A00357	A07323	252
A00357	A07398	474	A00357	A07451	491
A00357	A08053	215	A00357	A08055	162
A00357	A08357	274	A00357	A08358	217
A00357	A08398	481	A00357	A08452	491
A00357	A08664	343	A00357	A08835	223
A00357	A09248	198	A00357	A09313	256
A00357	A09322	386	A00357	A09585	301
A00357	A10417	472	A00357	A10518	274

A00357	A10616	195	A00357	A11400	249
A00357	A11438	485	A00357	A11550	163
A00357	A11588	232	A00357	A11612	281
A00357	A11945	151	A00357	A11981	303
A00357	A12237	288	A00357	A12661	465
A00357	A13600	363	A00357	A13989	468
A00357	A13992	486	A00357	A14029	324
A00357	A14505	176	A00357	A15215	165
A00357	A15416	201	A00357	A15418	207
A00357	A15660	285	A00357	A15743	332
A00357	A15988	483	A00357	A16053	328
A00357	A16118	486	A00357	A16149	201
A00357	A17193	246	A00358	A00360	491
A00358	A00361	491	A00358	A00362	462
A00358	A00390	486	A00358	A00403	491
A00358	A00416	345	A00358	A00469	462
A00358	A00521	396	A00358	A00590	443
A00358	A00603	202	A00358	A00629	489
A00358	A00630	491	A00358	A00631	486
A00358	A00705	188	A00358	A00763	348
A00358	A00780	182	A00358	A00787	373
A00358	A00839	348	A00358	A00848	341
A00358	A00905	356	A00358	A00916	391
A00358	A00923	491	A00358	A01000	491
A00358	A01017	485	A00358	A01025	491
A00358	A01047	484	A00358	A01056	319
A00358	A01072	348	A00358	A01098	395
A00358	A01101	491	A00358	A01120	347
A00358	A01127	480	A00358	A01128	180
A00358	A01130	174	A00358	A01140	488
A00358	A01166	347	A00358	A01184	449
A00358	A01206	154	A00358	A01234	457
A00358	A01240	110	A00358	A01249	491
A00358	A01291	491	A00358	A01292	221
A00358	A01306	491	A00358	A01335	172
A00358	A01338	491	A00358	A01425	439
A00358	A01464	486	A00358	A01468	240
A00358	A01470	380	A00358	A01569	262
A00358	A01612	456	A00358	A01617	475
A00358	A01618	480	A00358	A01649	485
A00358	A01700	291	A00358	A01718	451
A00358	A01740	210	A00358	A02118	265
A00358	A02216	201	A00358	A02265	276
A00358	A02738	174	A00358	A02978	286
A00358	A03069	480	A00358	A03083	218
A00358	A03332	491	A00358	A03437	143

A00358	A03567	491	A00358	A03571	229
A00358	A03754	491	A00358	A03913	330
A00358	A03955	373	A00358	A04316	363
A00358	A04694	274	A00358	A04736	238
A00358	A04754	420	A00358	A04761	945
A00358	A04846	174	A00358	A04911	174
A00358	A05044	466	A00358	A05142	238
A00358	A05370	263	A00358	A05385	489
A00358	A05443	440	A00358	A05501	255
A00358	A05996	945	A00358	A06279	475
A00358	A06814	306	A00358	A06861	427
A00358	A06965	491	A00358	A06973	457
A00358	A07449	160	A00358	A07451	74
A00358	A07738	313	A00358	A08011	458
A00358	A08018	283	A00358	A08055	245
A00358	A08137	465	A00358	A08357	193
A00358	A08452	481	A00358	A08664	168
A00358	A08835	234	A00358	A08869	160
A00358	A09322	491	A00358	A09562	171
A00358	A09702	450	A00358	A10154	234
A00358	A10529	234	A00358	A11190	235
A00358	A11390	227	A00358	A11400	475
A00358	A11438	469	A00358	A11480	490
A00358	A11612	491	A00358	A11704	491
A00358	A11729	205	A00358	A12077	313
A00358	A12233	303	A00358	A12651	475
A00358	A12653	489	A00358	A12918	360
A00358	A13241	366	A00358	A13388	151
A00358	A13466	154	A00358	A13989	477
A00358	A13992	396	A00358	A14216	151
A00358	A14737	313	A00358	A15218	438
A00358	A15255	205	A00358	A15776	177
A00358	A15778	165	A00358	A16118	489
A00358	A16523	368	A00358	A16631	435
A00358	A16823	360	A00358	A17127	160
A00359	A00385	452	A00359	A00469	413
A00359	A00472	221	A00359	A00546	205
A00359	A00971	204	A00359	A01725	168
A00359	A02000	227	A00359	A02021	310
A00359	A02049	157	A00359	A02697	344
A00359	A02738	226	A00359	A03080	323
A00359	A03186	163	A00359	A03321	301
A00359	A03390	481	A00359	A03391	296
A00359	A03401	401	A00359	A03733	411
A00359	A03744	363	A00359	A04410	343
A00359	A05603	195	A00359	A06346	322

A00359	A06635	218	A00359	A09748	303
A00359	A09749	260	A00359	A10000	339
A00359	A11293	224	A00360	A00361	491
A00360	A00783	154	A00360	A00923	232
A00360	A01206	432	A00360	A01291	490
A00360	A01470	243	A00360	A01718	455
A00360	A03332	339	A00360	A03955	267
A00360	A04754	486	A00360	A04761	491
A00360	A05044	274	A00360	A05996	428
A00360	A06965	234	A00360	A08018	438
A00360	A08137	371	A00360	A10316	472
A00360	A11612	491	A00360	A11704	208
A00360	A12073	321	A00360	A13989	235
A00360	A15247	488	A00360	A15255	451
A00361	A00362	227	A00361	A00802	165
A00361	A00900	208	A00361	A01092	196
A00361	A01291	491	A00361	A01524	899
A00361	A01718	380	A00361	A02293	259
A00361	A02927	205	A00361	A03332	486
A00361	A03571	190	A00361	A03726	899
A00361	A04147	333	A00361	A04162	165
A00361	A04230	198	A00361	A04754	468
A00361	A04761	491	A00361	A05044	241
A00361	A05443	306	A00361	A05996	488
A00361	A08018	428	A00361	A10316	294
A00361	A11612	491	A00361	A12653	279
A00361	A12897	163	A00361	A13241	480
A00361	A13989	165	A00361	A13992	102
A00361	A15247	467	A00361	A15498	226
A00362	A00563	251	A00362	A00590	302
A00362	A00603	196	A00362	A00701	154
A00362	A00878	215	A00362	A00882	167
A00362	A00900	383	A00362	A01072	157
A00362	A01291	480	A00362	A01740	167
A00362	A02179	162	A00362	A02265	210
A00362	A03913	191	A00362	A04033	462
A00362	A04761	418	A00362	A05044	296
A00362	A05354	346	A00362	A07803	322
A00362	A08835	486	A00362	A09707	419
A00362	A10748	294	A00362	A11322	210
A00362	A11338	218	A00362	A11400	489
A00362	A12918	348	A00362	A14003	303
A00362	A15256	334	A00362	A15416	460
A00363	A00417	313	A00363	A00431	329
A00363	A00942	324	A00363	A01257	182
A00363	A03748	213	A00363	A03910	454

A00363	A04145	366	A00363	A04169	305
A00363	A04337	185	A00363	A07165	163
A00363	A08320	424	A00363	A08544	409
A00363	A09416	258	A00363	A10525	283
A00363	A11084	406	A00363	A13987	196
A00363	A14190	184	A00363	A14210	165
A00363	A14511	291	A00363	A14539	271
A00363	A15292	424	A00363	A15301	238
A00363	A15313	317	A00363	A15352	165
A00363	A15431	337	A00363	A15432	469
A00363	A15435	208	A00363	A15436	388
A00363	A15437	356	A00363	A15453	443
A00363	A15456	417	A00363	A15457	435
A00364	A00421	212	A00364	A00531	199
A00364	A00571	227	A00364	A00653	208
A00364	A01012	281	A00364	A01150	215
A00364	A01496	259	A00364	A08571	160
A00364	A14216	185	A00365	A00369	135
A00365	A00421	491	A00365	A00531	463
A00365	A00559	241	A00365	A00600	345
A00365	A00601	461	A00365	A00619	215
A00365	A00689	254	A00365	A00716	474
A00365	A00905	132	A00365	A00915	218
A00365	A01139	435	A00365	A01151	70
A00365	A01239	429	A00365	A01359	461
A00365	A01380	180	A00365	A01471	234
A00365	A01472	260	A00365	A01486	162
A00365	A01741	200	A00365	A02165	196
A00365	A02802	266	A00365	A03259	195
A00365	A03301	152	A00365	A03467	256
A00365	A03494	484	A00365	A03902	394
A00365	A04345	356	A00365	A07826	485
A00365	A08201	389	A00365	A09258	270
A00365	A09377	329	A00365	A09427	406
A00365	A09451	420	A00365	A09515	419
A00365	A09538	413	A00365	A10228	296
A00365	A10250	332	A00365	A10340	333
A00365	A10563	201	A00365	A10578	266
A00365	A11139	191	A00365	A15920	187
A00365	A17168	272	A00366	A00553	312
A00366	A00626	224	A00366	A00651	369
A00366	A00653	391	A00366	A00822	247
A00366	A00841	440	A00366	A00876	184
A00366	A00883	469	A00366	A00958	469
A00366	A00967	193	A00366	A01096	191
A00366	A01097	413	A00366	A01140	238

A00366	A01150	152	A00366	A01261	180
A00366	A01274	488	A00366	A01291	378
A00366	A01295	467	A00366	A01299	466
A00366	A01305	339	A00366	A01431	485
A00366	A01540	463	A00366	A01598	235
A00366	A01787	202	A00366	A01822	329
A00366	A02692	400	A00366	A02931	236
A00366	A03406	489	A00366	A03591	490
A00366	A03752	486	A00366	A03977	177
A00366	A03981	469	A00366	A04116	232
A00366	A04900	278	A00366	A05288	486
A00366	A05361	215	A00366	A05852	291
A00366	A06328	347	A00366	A06434	160
A00366	A06466	491	A00366	A06518	291
A00366	A06580	440	A00366	A07311	490
A00366	A07726	484	A00366	A08679	401
A00366	A08869	485	A00366	A10282	398
A00366	A10299	384	A00366	A10493	251
A00366	A11316	230	A00366	A11387	182
A00366	A11621	322	A00366	A11733	180
A00366	A12227	251	A00366	A12328	481
A00366	A12365	400	A00366	A12618	157
A00366	A12652	212	A00366	A13340	491
A00366	A13581	165	A00366	A13989	371
A00366	A14216	283	A00366	A14410	286
A00366	A14596	462	A00366	A14609	168
A00366	A14660	207	A00366	A15257	260
A00366	A15309	152	A00366	A15660	378
A00366	A15684	402	A00366	A15763	152
A00366	A15773	205	A00366	A15905	191
A00366	A17127	152	A00367	A00368	665
A00367	A00369	488	A00367	A00373	456
A00367	A00421	694	A00367	A00496	200
A00367	A00531	673	A00367	A00559	435
A00367	A00560	451	A00367	A00677	230
A00367	A00691	473	A00367	A00822	485
A00367	A00883	454	A00367	A00905	435
A00367	A00916	368	A00367	A00945	159
A00367	A01206	263	A00367	A01266	216
A00367	A01372	383	A00367	A01558	475
A00367	A01588	373	A00367	A01598	355
A00367	A01612	402	A00367	A01614	457
A00367	A01615	372	A00367	A01638	455
A00367	A02759	176	A00367	A03298	491
A00367	A03435	321	A00367	A03494	539
A00367	A03567	154	A00367	A03740	238

A00367	A04009	252	A00367	A06134	208
A00367	A06637	279	A00367	A06907	257
A00367	A08137	461	A00367	A08524	383
A00367	A08554	218	A00367	A08571	491
A00367	A08818	324	A00367	A08885	439
A00367	A10250	385	A00367	A10615	463
A00367	A10624	470	A00367	A10853	457
A00367	A11139	692	A00367	A11258	165
A00367	A11920	489	A00367	A11923	168
A00367	A11928	430	A00367	A11965	260
A00367	A12660	235	A00367	A13147	345
A00367	A13310	475	A00367	A13311	270
A00367	A13805	438	A00367	A13989	353
A00367	A14476	491	A00367	A14931	196
A00367	A15711	438	A00367	A16463	180
A00367	A16490	400	A00367	A16678	278
A00367	A16690	383	A00367	A16866	383
A00367	A16912	477	A00368	A00369	469
A00368	A00373	477	A00368	A00421	691
A00368	A00454	218	A00368	A00501	160
A00368	A00531	694	A00368	A00537	382
A00368	A00559	460	A00368	A00691	447
A00368	A00697	205	A00368	A00819	302
A00368	A00822	323	A00368	A00905	441
A00368	A01139	127	A00368	A01359	90
A00368	A01372	383	A00368	A01558	358
A00368	A01560	168	A00368	A01612	417
A00368	A01615	385	A00368	A02607	221
A00368	A03298	486	A00368	A03333	213
A00368	A03494	593	A00368	A03570	260
A00368	A03752	279	A00368	A03981	165
A00368	A04147	188	A00368	A06466	401
A00368	A07311	485	A00368	A08137	436
A00368	A08357	232	A00368	A08524	383
A00368	A08571	491	A00368	A08885	417
A00368	A09256	171	A00368	A10615	234
A00368	A10624	281	A00368	A10853	260
A00368	A11105	270	A00368	A11139	694
A00368	A11379	201	A00368	A11420	168
A00368	A11920	162	A00368	A11928	486
A00368	A12328	151	A00368	A13310	394
A00368	A13311	371	A00368	A13340	195
A00368	A13805	438	A00368	A13978	168
A00368	A14476	491	A00368	A15244	191
A00368	A15711	523	A00368	A16690	383
A00368	A16866	383	A00368	A16912	455

A00369	A00372	162	A00369	A00421	491
A00369	A00458	193	A00369	A00531	491
A00369	A00532	310	A00369	A00559	460
A00369	A00605	399	A00369	A00648	205
A00369	A00677	452	A00369	A00691	491
A00369	A00716	104	A00369	A00822	490
A00369	A00831	470	A00369	A00848	213
A00369	A00883	490	A00369	A00905	491
A00369	A00916	489	A00369	A00947	293
A00369	A01000	281	A00369	A01091	266
A00369	A01112	246	A00369	A01136	432
A00369	A01139	80	A00369	A01150	477
A00369	A01151	274	A00369	A01206	422
A00369	A01234	475	A00369	A01243	353
A00369	A01266	424	A00369	A01268	425
A00369	A01291	340	A00369	A01354	352
A00369	A01359	363	A00369	A01468	328
A00369	A01470	485	A00369	A01471	347
A00369	A01558	491	A00369	A01588	450
A00369	A01612	491	A00369	A01615	485
A00369	A01638	210	A00369	A01649	484
A00369	A03298	491	A00369	A03494	462
A00369	A03566	229	A00369	A03567	213
A00369	A03569	384	A00369	A03902	176
A00369	A04406	224	A00369	A05015	204
A00369	A06466	160	A00369	A06637	291
A00369	A07311	177	A00369	A07451	157
A00369	A07706	218	A00369	A08137	490
A00369	A08357	441	A00369	A08571	491
A00369	A08876	163	A00369	A08885	486
A00369	A09256	182	A00369	A10250	485
A00369	A10615	394	A00369	A10624	491
A00369	A10853	491	A00369	A11105	465
A00369	A11139	486	A00369	A11258	241
A00369	A11831	185	A00369	A11920	394
A00369	A11928	243	A00369	A13310	406
A00369	A13311	208	A00369	A13989	491
A00369	A14476	491	A00369	A14596	243
A00369	A15244	208	A00369	A15559	224
A00369	A15660	274	A00369	A15684	374
A00369	A16685	246	A00369	A16912	279
A00370	A00511	380	A00370	A00567	154
A00370	A00597	490	A00370	A00611	469
A00370	A00642	247	A00370	A00643	475
A00370	A00679	396	A00370	A00684	168
A00370	A00692	163	A00370	A00747	385

A00370	A00888	302	A00370	A01094	326
A00370	A01172	276	A00370	A01246	371
A00370	A01257	163	A00370	A01450	267
A00370	A01527	899	A00370	A01602	479
A00370	A01668	479	A00370	A01716	221
A00370	A02018	479	A00370	A02021	263
A00370	A02049	63	A00370	A02051	177
A00370	A02143	333	A00370	A02148	278
A00370	A02154	270	A00370	A02180	491
A00370	A02190	201	A00370	A02191	447
A00370	A02213	157	A00370	A02219	63
A00370	A02223	212	A00370	A02239	375
A00370	A02371	165	A00370	A02741	265
A00370	A03012	302	A00370	A03025	207
A00370	A03049	162	A00370	A03070	177
A00370	A03186	288	A00370	A03213	334
A00370	A03226	154	A00370	A03304	472
A00370	A03416	190	A00370	A03451	276
A00370	A03459	291	A00370	A03725	413
A00370	A03749	260	A00370	A03963	491
A00370	A04174	276	A00370	A04183	398
A00370	A04185	352	A00370	A04258	236
A00370	A04360	207	A00370	A04395	201
A00370	A04396	259	A00370	A04405	167
A00370	A04413	230	A00370	A04516	162
A00370	A04550	273	A00370	A04556	151
A00370	A04614	218	A00370	A04659	288
A00370	A04677	273	A00370	A04739	165
A00370	A04744	217	A00370	A04870	347
A00370	A04874	486	A00370	A05041	217
A00370	A05135	386	A00370	A05299	262
A00370	A05536	167	A00370	A05666	180
A00370	A05720	319	A00370	A05956	157
A00370	A06130	396	A00370	A07158	470
A00370	A07261	291	A00370	A08238	154
A00370	A08600	273	A00370	A08836	318
A00370	A09371	386	A00370	A09559	217
A00370	A09790	279	A00370	A09791	330
A00370	A09906	230	A00370	A10015	230
A00370	A10387	418	A00370	A10440	168
A00370	A10825	259	A00370	A11517	319
A00370	A12504	319	A00370	A12505	319
A00370	A12750	319	A00370	A13408	319
A00370	A13476	291	A00370	A13716	156
A00370	A13997	180	A00370	A14643	193
A00370	A15989	259	A00370	A16718	151

A00370	A16814	174	A00371	A00382	404
A00371	A00396	240	A00371	A00412	646
A00371	A00421	373	A00371	A00426	227
A00371	A00433	380	A00371	A00444	485
A00371	A00489	404	A00371	A00494	373
A00371	A00517	238	A00371	A00521	341
A00371	A00537	182	A00371	A00640	389
A00371	A00666	271	A00371	A00671	293
A00371	A00689	269	A00371	A00706	638
A00371	A00713	208	A00371	A00740	227
A00371	A00824	447	A00371	A00839	618
A00371	A00843	386	A00371	A00851	213
A00371	A00865	245	A00371	A00875	380
A00371	A00878	407	A00371	A00883	314
A00371	A00942	224	A00371	A00970	486
A00371	A00973	334	A00371	A00980	488
A00371	A00996	241	A00371	A00999	561
A00371	A01016	268	A00371	A01031	358
A00371	A01061	281	A00371	A01068	229
A00371	A01069	669	A00371	A01082	290
A00371	A01106	243	A00371	A01113	607
A00371	A01115	489	A00371	A01127	174
A00371	A01139	190	A00371	A01192	634
A00371	A01194	461	A00371	A01231	221
A00371	A01242	210	A00371	A01268	224
A00371	A01291	472	A00371	A01311	477
A00371	A01317	249	A00371	A01354	173
A00371	A01365	151	A00371	A01374	345
A00371	A01379	187	A00371	A01419	159
A00371	A01420	259	A00371	A01429	246
A00371	A01458	200	A00371	A01508	490
A00371	A01539	446	A00371	A01541	374
A00371	A01548	465	A00371	A01569	491
A00371	A01577	152	A00371	A01606	428
A00371	A01633	228	A00371	A01648	338
A00371	A01680	301	A00371	A01792	210
A00371	A02180	372	A00371	A02219	241
A00371	A02426	232	A00371	A03401	480
A00371	A03446	571	A00371	A03477	168
A00371	A03560	676	A00371	A03738	236
A00371	A03773	221	A00371	A03783	404
A00371	A03802	157	A00371	A03863	430
A00371	A03909	152	A00371	A04124	337
A00371	A04187	345	A00371	A04272	443
A00371	A04394	193	A00371	A04397	221
A00371	A05017	274	A00371	A05022	221

A00371	A05029	334	A00371	A05313	177
A00371	A05449	462	A00371	A05928	277
A00371	A06330	243	A00371	A06466	176
A00371	A06604	165	A00371	A06814	462
A00371	A06878	288	A00371	A07203	243
A00371	A07229	449	A00371	A07247	232
A00371	A07311	183	A00371	A07378	397
A00371	A07677	281	A00371	A07695	286
A00371	A07700	163	A00371	A08061	445
A00371	A08261	329	A00371	A08546	600
A00371	A08858	199	A00371	A08860	340
A00371	A08978	489	A00371	A08991	535
A00371	A09024	163	A00371	A09269	463
A00371	A09591	477	A00371	A09814	162
A00371	A10490	486	A00371	A10532	488
A00371	A10815	258	A00371	A11409	281
A00371	A11468	243	A00371	A11563	277
A00371	A11657	486	A00371	A11705	329
A00371	A11931	485	A00371	A12242	357
A00371	A12350	258	A00371	A12813	213
A00371	A13135	180	A00371	A13311	228
A00371	A13388	227	A00371	A13424	324
A00371	A13529	258	A00371	A13609	215
A00371	A13989	229	A00371	A14178	346
A00371	A14247	154	A00371	A14351	491
A00371	A14674	271	A00371	A14716	157
A00371	A15146	258	A00371	A15152	307
A00371	A15297	241	A00371	A15503	249
A00371	A15696	243	A00371	A15698	290
A00371	A15714	223	A00371	A15953	193
A00371	A16090	384	A00371	A16321	258
A00371	A16400	462	A00371	A16462	213
A00371	A16756	425	A00371	A16759	307
A00371	A16824	176	A00371	A17088	509
A00372	A00421	157	A00372	A00424	436
A00372	A00440	152	A00372	A00445	184
A00372	A00446	406	A00372	A00452	457
A00372	A00508	183	A00372	A00532	238
A00372	A00553	226	A00372	A00570	283
A00372	A00687	302	A00372	A00791	425
A00372	A00822	207	A00372	A00826	208
A00372	A00835	314	A00372	A00867	160
A00372	A00868	438	A00372	A00883	491
A00372	A00913	183	A00372	A00928	373
A00372	A01022	445	A00372	A01057	367
A00372	A01091	491	A00372	A01097	296

A00372	A01193	154	A00372	A01211	384
A00372	A01234	219	A00372	A01243	485
A00372	A01259	363	A00372	A01339	232
A00372	A01451	457	A00372	A01612	159
A00372	A01660	180	A00372	A01975	224
A00372	A02212	490	A00372	A02452	462
A00372	A03065	193	A00372	A03567	419
A00372	A04337	311	A00372	A04881	190
A00372	A04922	330	A00372	A05157	201
A00372	A06122	187	A00372	A07756	213
A00372	A07803	174	A00372	A07956	286
A00372	A08183	167	A00372	A08281	328
A00372	A08517	462	A00372	A08630	182
A00372	A08982	483	A00372	A08989	230
A00372	A08990	345	A00372	A10321	157
A00372	A10870	180	A00372	A11316	205
A00372	A11323	183	A00372	A11678	241
A00372	A12101	395	A00372	A12253	266
A00372	A12292	296	A00372	A12557	303
A00372	A14170	226	A00372	A14196	491
A00372	A14246	213	A00372	A14476	306
A00372	A14931	182	A00372	A15237	489
A00372	A15292	329	A00372	A15297	486
A00372	A15309	391	A00372	A15323	491
A00372	A15324	491	A00372	A15339	489
A00372	A15341	218	A00372	A15353	491
A00372	A15355	168	A00372	A15396	299
A00372	A15419	449	A00372	A15431	489
A00372	A15502	416	A00372	A15537	273
A00372	A15543	377	A00372	A15559	224
A00372	A15564	488	A00372	A15631	352
A00372	A15717	177	A00372	A15747	368
A00372	A15763	223	A00372	A15788	466
A00372	A15871	224	A00372	A16458	291
A00372	A16463	299	A00372	A16861	319
A00372	A17063	484	A00373	A00421	445
A00373	A00531	348	A00373	A00677	382
A00373	A00691	458	A00373	A01266	151
A00373	A01615	353	A00373	A01685	308
A00373	A03298	481	A00373	A03528	224
A00373	A03570	176	A00373	A05015	290
A00373	A08013	406	A00373	A08137	176
A00373	A08554	385	A00373	A08571	491
A00373	A08883	324	A00373	A08885	469
A00373	A09256	230	A00373	A10250	218
A00373	A10623	188	A00373	A11105	307

A00373	A11139	313	A00373	A11168	409
A00373	A11284	152	A00373	A11928	219
A00373	A13310	224	A00373	A13311	327
A00373	A13978	213	A00373	A14476	286
A00373	A14931	249	A00373	A15660	238
A00374	A00397	434	A00374	A00406	491
A00374	A00425	414	A00374	A00442	171
A00374	A00448	708	A00374	A00459	249
A00374	A00463	374	A00374	A00488	706
A00374	A00490	630	A00374	A00497	706
A00374	A00518	402	A00374	A00520	388
A00374	A00530	266	A00374	A00532	398
A00374	A00550	481	A00374	A00551	432
A00374	A00554	707	A00374	A00566	347
A00374	A00575	469	A00374	A00595	313
A00374	A00605	170	A00374	A00651	170
A00374	A00708	435	A00374	A00713	170
A00374	A00749	170	A00374	A00767	170
A00374	A00856	247	A00374	A00858	486
A00374	A00861	170	A00374	A00871	216
A00374	A00872	170	A00374	A00916	549
A00374	A00918	170	A00374	A00929	232
A00374	A00934	549	A00374	A00944	297
A00374	A00978	363	A00374	A00992	271
A00374	A01020	708	A00374	A01028	170
A00374	A01075	490	A00374	A01087	468
A00374	A01088	283	A00374	A01116	491
A00374	A01120	170	A00374	A01129	170
A00374	A01160	170	A00374	A01168	170
A00374	A01184	491	A00374	A01195	167
A00374	A01201	491	A00374	A01206	271
A00374	A01214	385	A00374	A01224	241
A00374	A01226	306	A00374	A01234	491
A00374	A01304	491	A00374	A01351	449
A00374	A01356	170	A00374	A01398	177
A00374	A01425	170	A00374	A01429	218
A00374	A01487	323	A00374	A01492	528
A00374	A01503	160	A00374	A01538	170
A00374	A01576	168	A00374	A01598	170
A00374	A01614	170	A00374	A01700	191
A00374	A01721	257	A00374	A01741	268
A00374	A02265	185	A00374	A02364	202
A00374	A02680	254	A00374	A03112	324
A00374	A03438	170	A00374	A03652	170
A00374	A03734	321	A00374	A03981	170
A00374	A05528	215	A00374	A05845	179

A00374	A06275	486	A00374	A06356	170
A00374	A07223	179	A00374	A07244	278
A00374	A07347	491	A00374	A07378	234
A00374	A07524	380	A00374	A07533	472
A00374	A07584	486	A00374	A07695	317
A00374	A07700	491	A00374	A07706	299
A00374	A07725	380	A00374	A07850	238
A00374	A07924	411	A00374	A08022	314
A00374	A08024	228	A00374	A08144	156
A00374	A08211	325	A00374	A08326	350
A00374	A08430	177	A00374	A08491	670
A00374	A09307	205	A00374	A09619	503
A00374	A09664	256	A00374	A09697	170
A00374	A10616	384	A00374	A13052	170
A00374	A13055	247	A00374	A13989	479
A00374	A14505	266	A00375	A00377	263
A00375	A00446	346	A00375	A00556	157
A00375	A00674	274	A00375	A00751	424
A00375	A00761	188	A00375	A00766	299
A00375	A00781	441	A00375	A00791	443
A00375	A00847	265	A00375	A00868	454
A00375	A00883	491	A00375	A00925	219
A00375	A00926	465	A00375	A00927	386
A00375	A00975	400	A00375	A01058	168
A00375	A01077	345	A00375	A01106	414
A00375	A01340	297	A00375	A01370	391
A00375	A02722	172	A00375	A03570	179
A00375	A03601	157	A00375	A05146	238
A00375	A05570	254	A00375	A06628	179
A00375	A06721	245	A00375	A06968	205
A00375	A08123	382	A00375	A08413	396
A00375	A11735	171	A00375	A12824	473
A00375	A15537	328	A00376	A00453	452
A00376	A00541	411	A00376	A00573	223
A00376	A00574	491	A00376	A00579	269
A00376	A00614	272	A00376	A00628	486
A00376	A00654	490	A00376	A00662	185
A00376	A00666	481	A00376	A00681	491
A00376	A00695	299	A00376	A00738	457
A00376	A00770	451	A00376	A00771	337
A00376	A00773	394	A00376	A00825	224
A00376	A00962	490	A00376	A00995	491
A00376	A01008	480	A00376	A01063	188
A00376	A01068	235	A00376	A01109	449
A00376	A01165	205	A00376	A01174	283
A00376	A01175	255	A00376	A01233	447

A00376	A01256	486	A00376	A01277	180
A00376	A01360	412	A00376	A01367	199
A00376	A01397	177	A00376	A01508	475
A00376	A01635	414	A00376	A01741	213
A00376	A01743	245	A00376	A01972	160
A00376	A02026	241	A00376	A02290	252
A00376	A02366	321	A00376	A02562	171
A00376	A02859	303	A00376	A02992	263
A00376	A03004	357	A00376	A03016	411
A00376	A03167	400	A00376	A03168	477
A00376	A03259	463	A00376	A03323	321
A00376	A03324	348	A00376	A03431	240
A00376	A03477	319	A00376	A03484	196
A00376	A03861	160	A00376	A04211	367
A00376	A04307	156	A00376	A04308	258
A00376	A04660	232	A00376	A04778	232
A00376	A04784	299	A00376	A04791	207
A00376	A04834	230	A00376	A04850	294
A00376	A04995	201	A00376	A05007	467
A00376	A05098	196	A00376	A05102	491
A00376	A05416	335	A00376	A05472	167
A00376	A05515	243	A00376	A05516	207
A00376	A05573	165	A00376	A05758	183
A00376	A05759	413	A00376	A05768	224
A00376	A05772	389	A00376	A05937	267
A00376	A06091	201	A00376	A06358	162
A00376	A06633	418	A00376	A06634	279
A00376	A06689	213	A00376	A06756	396
A00376	A06934	262	A00376	A06970	288
A00376	A06979	218	A00376	A07002	310
A00376	A07122	477	A00376	A07221	262
A00376	A07238	259	A00376	A07267	404
A00376	A07304	340	A00376	A07341	269
A00376	A07413	371	A00376	A07477	162
A00376	A07530	243	A00376	A07553	258
A00376	A07554	277	A00376	A07558	371
A00376	A07590	288	A00376	A07627	321
A00376	A07688	457	A00376	A07736	252
A00376	A07800	475	A00376	A07933	184
A00376	A07973	167	A00376	A08099	475
A00376	A08751	279	A00376	A08817	159
A00376	A09612	348	A00376	A10456	483
A00376	A10614	251	A00376	A10681	367
A00376	A10702	363	A00376	A10712	429
A00376	A10714	270	A00376	A11309	441
A00376	A11325	326	A00376	A13257	190

A00376	A14337	447	A00376	A14637	226
A00376	A14640	395	A00376	A14827	156
A00376	A15497	257	A00376	A15874	486
A00376	A15905	375	A00376	A16026	463
A00376	A16027	490	A00376	A16028	236
A00376	A16033	228	A00376	A16262	456
A00376	A16528	305	A00376	A16529	480
A00376	A16741	180	A00376	A16959	308
A00376	A17040	279	A00377	A00382	157
A00377	A00420	157	A00377	A00421	623
A00377	A00426	323	A00377	A00433	199
A00377	A00438	623	A00377	A00446	840
A00377	A00448	152	A00377	A00485	474
A00377	A00506	391	A00377	A00519	489
A00377	A00525	369	A00377	A00531	541
A00377	A00537	557	A00377	A00540	291
A00377	A00544	361	A00377	A00553	491
A00377	A00564	461	A00377	A00565	472
A00377	A00570	491	A00377	A00571	179
A00377	A00576	490	A00377	A00577	224
A00377	A00600	446	A00377	A00601	401
A00377	A00638	185	A00377	A00643	228
A00377	A00653	481	A00377	A00664	446
A00377	A00674	491	A00377	A00687	488
A00377	A00694	246	A00377	A00697	440
A00377	A00709	382	A00377	A00714	170
A00377	A00724	183	A00377	A00735	474
A00377	A00751	438	A00377	A00766	457
A00377	A00769	489	A00377	A00776	322
A00377	A00781	310	A00377	A00791	490
A00377	A00799	272	A00377	A00802	185
A00377	A00805	286	A00377	A00809	486
A00377	A00813	347	A00377	A00815	263
A00377	A00816	449	A00377	A00821	337
A00377	A00829	446	A00377	A00841	430
A00377	A00849	249	A00377	A00858	207
A00377	A00863	430	A00377	A00865	283
A00377	A00868	561	A00377	A00871	484
A00377	A00878	218	A00377	A00883	553
A00377	A00911	514	A00377	A00915	490
A00377	A00922	374	A00377	A00926	491
A00377	A00927	491	A00377	A00953	362
A00377	A00975	491	A00377	A00983	491
A00377	A00985	272	A00377	A00986	279
A00377	A00987	294	A00377	A01001	151
A00377	A01017	486	A00377	A01020	418

A00377	A01022	238	A00377	A01027	446
A00377	A01058	423	A00377	A01066	362
A00377	A01068	452	A00377	A01080	491
A00377	A01097	357	A00377	A01100	218
A00377	A01101	313	A00377	A01102	446
A00377	A01103	63	A00377	A01106	228
A00377	A01108	467	A00377	A01122	479
A00377	A01125	177	A00377	A01139	475
A00377	A01156	839	A00377	A01161	767
A00377	A01169	491	A00377	A01212	472
A00377	A01214	377	A00377	A01221	440
A00377	A01225	63	A00377	A01232	364
A00377	A01234	390	A00377	A01264	319
A00377	A01267	491	A00377	A01278	491
A00377	A01307	409	A00377	A01322	486
A00377	A01329	218	A00377	A01338	485
A00377	A01339	447	A00377	A01343	431
A00377	A01359	163	A00377	A01370	308
A00377	A01371	221	A00377	A01379	243
A00377	A01385	278	A00377	A01392	188
A00377	A01404	411	A00377	A01415	440
A00377	A01419	388	A00377	A01481	294
A00377	A01485	165	A00377	A01497	119
A00377	A01507	318	A00377	A01524	899
A00377	A01551	491	A00377	A01573	417
A00377	A01578	235	A00377	A01582	490
A00377	A01593	916	A00377	A01606	485
A00377	A01627	608	A00377	A01632	481
A00377	A01639	182	A00377	A01668	221
A00377	A01711	374	A00377	A01741	479
A00377	A01790	263	A00377	A01931	217
A00377	A01953	323	A00377	A01977	299
A00377	A01995	179	A00377	A02020	361
A00377	A02058	257	A00377	A02086	457
A00377	A02148	481	A00377	A02164	486
A00377	A02192	63	A00377	A02203	401
A00377	A02411	389	A00377	A02477	433
A00377	A02549	73	A00377	A02583	63
A00377	A02584	68	A00377	A02594	138
A00377	A02598	79	A00377	A02618	205
A00377	A02709	486	A00377	A02761	162
A00377	A02908	286	A00377	A02978	483
A00377	A03079	378	A00377	A03104	455
A00377	A03259	299	A00377	A03304	391
A00377	A03334	489	A00377	A03454	360
A00377	A03495	63	A00377	A03538	174

A00377	A03573	279	A00377	A03651	254
A00377	A03676	491	A00377	A03713	432
A00377	A03726	941	A00377	A03742	176
A00377	A03750	184	A00377	A03819	446
A00377	A03822	151	A00377	A03851	185
A00377	A03859	443	A00377	A03885	281
A00377	A03902	279	A00377	A03981	200
A00377	A03992	439	A00377	A04033	475
A00377	A04072	236	A00377	A04079	185
A00377	A04256	321	A00377	A04275	378
A00377	A04337	160	A00377	A04423	486
A00377	A04449	557	A00377	A04491	491
A00377	A04666	252	A00377	A04668	258
A00377	A04925	152	A00377	A05095	307
A00377	A05323	378	A00377	A05356	330
A00377	A05442	152	A00377	A05690	162
A00377	A05738	385	A00377	A05812	378
A00377	A06099	260	A00377	A06272	488
A00377	A06277	486	A00377	A06297	174
A00377	A06400	70	A00377	A06454	425
A00377	A06556	212	A00377	A06624	318
A00377	A06631	345	A00377	A06708	213
A00377	A06721	213	A00377	A06825	477
A00377	A06861	291	A00377	A06938	483
A00377	A07014	462	A00377	A07046	249
A00377	A07136	88	A00377	A07157	218
A00377	A07229	273	A00377	A07346	419
A00377	A07360	491	A00377	A07422	391
A00377	A07457	305	A00377	A07545	296
A00377	A07641	185	A00377	A07670	334
A00377	A07675	314	A00377	A07756	329
A00377	A07799	162	A00377	A07822	450
A00377	A08025	212	A00377	A08064	488
A00377	A08123	174	A00377	A08180	174
A00377	A08442	212	A00377	A08558	404
A00377	A08659	212	A00377	A08878	405
A00377	A08927	210	A00377	A09328	491
A00377	A09366	212	A00377	A09672	462
A00377	A09750	218	A00377	A09766	152
A00377	A09910	152	A00377	A10186	361
A00377	A10264	440	A00377	A10278	899
A00377	A10371	486	A00377	A10372	457
A00377	A10540	470	A00377	A10755	240
A00377	A11313	438	A00377	A11602	154
A00377	A11706	188	A00377	A11735	405
A00377	A11737	467	A00377	A11914	424

A00377	A11983	646	A00377	A12453	212
A00377	A12581	152	A00377	A12654	154
A00377	A12658	374	A00377	A12824	491
A00377	A13225	174	A00377	A13298	351
A00377	A13344	152	A00377	A13369	300
A00377	A13397	286	A00377	A13614	491
A00377	A13793	307	A00377	A14077	423
A00377	A14196	906	A00377	A14381	160
A00377	A14449	243	A00377	A14489	288
A00377	A14566	210	A00377	A14622	218
A00377	A14635	159	A00377	A14648	490
A00377	A14655	348	A00377	A14701	446
A00377	A14735	229	A00377	A15185	406
A00377	A15257	313	A00377	A15282	490
A00377	A15295	583	A00377	A15297	366
A00377	A15322	340	A00377	A15379	191
A00377	A15422	431	A00377	A15498	582
A00377	A15558	409	A00377	A15624	491
A00377	A15626	210	A00377	A15649	470
A00377	A15651	491	A00377	A15652	401
A00377	A15661	491	A00377	A15684	491
A00377	A15746	408	A00377	A15900	212
A00377	A15932	489	A00377	A15933	299
A00377	A15937	204	A00377	A16088	266
A00377	A16149	269	A00377	A16157	273
A00377	A16343	198	A00377	A16626	176
A00377	A16627	430	A00377	A16700	488
A00378	A00380	380	A00378	A00383	191
A00378	A00421	454	A00378	A00436	240
A00378	A00441	436	A00378	A00463	529
A00378	A00475	401	A00378	A00479	279
A00378	A00488	307	A00378	A00517	542
A00378	A00531	249	A00378	A00537	560
A00378	A00553	177	A00378	A00566	541
A00378	A00575	537	A00378	A00604	441
A00378	A00622	459	A00378	A00626	371
A00378	A00653	188	A00378	A00665	715
A00378	A00680	479	A00378	A00690	446
A00378	A00697	485	A00378	A00733	541
A00378	A00741	480	A00378	A00744	715
A00378	A00791	177	A00378	A00805	542
A00378	A00814	193	A00378	A00826	335
A00378	A00838	180	A00378	A00858	541
A00378	A00860	513	A00378	A00866	156
A00378	A00871	396	A00378	A00881	528
A00378	A00883	436	A00378	A00935	286

A00378	A00955	157	A00378	A00961	420
A00378	A00978	401	A00378	A00998	327
A00378	A01007	156	A00378	A01019	537
A00378	A01024	480	A00378	A01057	477
A00378	A01077	219	A00378	A01150	424
A00378	A01155	188	A00378	A01161	486
A00378	A01195	535	A00378	A01200	398
A00378	A01210	433	A00378	A01212	477
A00378	A01214	228	A00378	A01238	339
A00378	A01325	620	A00378	A01336	290
A00378	A01347	280	A00378	A01354	212
A00378	A01398	540	A00378	A01401	528
A00378	A01413	486	A00378	A01447	483
A00378	A01451	374	A00378	A01492	156
A00378	A01500	537	A00378	A01503	420
A00378	A01539	489	A00378	A01540	227
A00378	A01571	489	A00378	A01610	393
A00378	A01640	337	A00378	A01720	286
A00378	A01844	911	A00378	A01914	262
A00378	A02030	177	A00378	A02389	536
A00378	A02715	391	A00378	A02994	185
A00378	A03014	491	A00378	A03207	408
A00378	A03494	429	A00378	A03526	457
A00378	A03569	228	A00378	A03863	196
A00378	A03889	390	A00378	A04181	358
A00378	A04648	479	A00378	A04687	156
A00378	A04879	489	A00378	A04892	260
A00378	A05802	334	A00378	A05981	715
A00378	A06129	152	A00378	A06803	457
A00378	A07203	193	A00378	A07347	491
A00378	A07670	168	A00378	A07686	394
A00378	A07725	329	A00378	A07850	156
A00378	A07887	354	A00378	A08137	404
A00378	A08383	178	A00378	A08818	230
A00378	A08860	340	A00378	A09125	218
A00378	A09349	165	A00378	A09502	156
A00378	A09526	238	A00378	A09529	180
A00378	A09595	152	A00378	A09712	311
A00378	A09723	358	A00378	A09806	427
A00378	A10070	152	A00378	A10162	191
A00378	A11033	154	A00378	A11344	156
A00378	A11674	156	A00378	A11735	270
A00378	A11737	273	A00378	A11755	240
A00378	A11804	347	A00378	A12026	218
A00378	A12099	172	A00378	A12507	187
A00378	A13788	479	A00378	A14006	491

A00378	A14188	249	A00378	A14399	474
A00378	A14540	177	A00378	A15297	346
A00378	A15507	168	A00378	A15545	252
A00378	A15666	491	A00378	A15735	204
A00378	A16228	355	A00378	A16400	280
A00378	A16410	221	A00378	A16786	170
A00379	A00395	491	A00379	A00449	491
A00379	A00574	470	A00379	A00628	255
A00379	A00638	383	A00379	A00654	306
A00379	A00825	290	A00379	A00995	446
A00379	A01018	491	A00379	A01222	259
A00379	A01225	486	A00379	A01297	422
A00379	A01409	433	A00379	A01562	414
A00379	A01899	463	A00379	A02192	337
A00379	A02254	259	A00379	A02521	267
A00379	A02708	198	A00379	A02992	210
A00379	A03484	199	A00379	A03513	183
A00379	A03795	489	A00379	A04179	430
A00379	A04228	491	A00379	A05045	412
A00379	A05393	193	A00379	A05757	204
A00379	A05759	223	A00379	A05789	168
A00379	A06439	452	A00379	A06689	210
A00379	A06847	262	A00379	A06855	162
A00379	A06860	267	A00379	A07037	154
A00379	A07059	383	A00379	A07067	310
A00379	A07088	475	A00379	A07239	490
A00379	A07281	462	A00379	A07520	380
A00379	A07674	330	A00379	A07739	446
A00379	A08133	199	A00379	A08155	293
A00379	A08368	369	A00379	A08687	162
A00379	A09879	157	A00379	A10424	172
A00379	A10457	162	A00379	A10458	172
A00379	A10652	440	A00379	A10687	176
A00379	A10696	457	A00379	A10708	168
A00379	A11021	408	A00379	A13065	279
A00379	A14193	152	A00379	A14221	283
A00379	A16763	443	A00379	A17006	417
A00379	A17092	260	A00380	A00459	409
A00380	A00545	162	A00380	A00572	491
A00380	A00761	157	A00380	A00817	462
A00380	A00818	243	A00380	A00819	390
A00380	A00822	488	A00380	A00981	190
A00380	A01169	281	A00380	A01241	160
A00380	A01320	172	A00380	A01330	205
A00380	A01383	489	A00380	A01385	491
A00380	A01510	305	A00380	A01575	279

A00380	A01683	152	A00380	A02479	218
A00380	A03091	199	A00380	A03525	360
A00380	A03526	215	A00380	A04695	302
A00380	A04861	165	A00380	A05120	272
A00380	A07725	307	A00380	A07885	184
A00380	A09056	301	A00380	A09675	368
A00380	A10642	195	A00380	A11710	279
A00380	A14183	185	A00381	A00642	196
A00381	A00643	441	A00381	A00732	263
A00381	A00864	486	A00381	A01051	218
A00381	A01529	306	A00381	A01747	339
A00381	A01897	270	A00381	A01901	124
A00381	A02318	241	A00381	A02392	296
A00381	A02842	235	A00381	A03213	180
A00381	A03636	229	A00381	A03771	473
A00381	A03922	243	A00381	A04133	227
A00381	A04239	399	A00381	A04282	202
A00381	A04305	162	A00381	A04358	296
A00381	A04534	302	A00381	A04543	218
A00381	A05065	162	A00381	A05760	899
A00381	A05779	243	A00381	A05848	307
A00381	A06653	212	A00381	A06705	414
A00381	A06752	241	A00381	A07178	205
A00381	A07201	70	A00381	A07339	899
A00381	A07683	319	A00381	A08221	485
A00381	A08370	265	A00381	A09078	247
A00381	A09123	449	A00381	A09553	899
A00381	A09900	188	A00381	A10161	339
A00381	A10278	205	A00381	A10941	296
A00381	A11648	281	A00381	A11695	238
A00381	A11842	199	A00381	A12136	288
A00381	A12292	307	A00381	A12468	352
A00381	A12867	339	A00381	A12965	339
A00381	A13954	257	A00381	A13956	238
A00381	A13990	368	A00381	A14543	208
A00381	A14588	299	A00381	A14643	321
A00381	A14660	156	A00381	A15840	428
A00381	A16463	469	A00381	A16485	257
A00381	A16732	462	A00382	A00397	491
A00382	A00412	404	A00382	A00421	490
A00382	A00438	302	A00382	A00444	658
A00382	A00452	414	A00382	A00458	418
A00382	A00489	721	A00382	A00496	183
A00382	A00508	310	A00382	A00517	401
A00382	A00532	484	A00382	A00555	356
A00382	A00570	470	A00382	A00640	330

A00382	A00667	386	A00382	A00687	246
A00382	A00694	302	A00382	A00703	413
A00382	A00767	481	A00382	A00822	339
A00382	A00843	256	A00382	A00848	491
A00382	A00867	188	A00382	A00868	176
A00382	A00871	293	A00382	A00883	559
A00382	A00905	467	A00382	A00916	547
A00382	A00928	452	A00382	A00930	259
A00382	A00958	235	A00382	A00970	470
A00382	A00992	456	A00382	A01040	502
A00382	A01069	753	A00382	A01075	484
A00382	A01091	267	A00382	A01115	721
A00382	A01120	193	A00382	A01136	249
A00382	A01150	463	A00382	A01156	243
A00382	A01166	185	A00382	A01194	491
A00382	A01206	461	A00382	A01234	491
A00382	A01243	463	A00382	A01266	188
A00382	A01268	463	A00382	A01279	491
A00382	A01291	477	A00382	A01311	63
A00382	A01351	489	A00382	A01352	466
A00382	A01354	291	A00382	A01413	63
A00382	A01425	188	A00382	A01431	160
A00382	A01442	159	A00382	A01457	228
A00382	A01470	490	A00382	A01492	490
A00382	A01539	845	A00382	A01569	728
A00382	A01577	326	A00382	A01598	491
A00382	A01612	485	A00382	A01614	470
A00382	A01615	491	A00382	A01649	452
A00382	A01770	457	A00382	A01917	179
A00382	A02212	168	A00382	A02535	485
A00382	A02792	63	A00382	A03446	721
A00382	A03476	165	A00382	A03494	424
A00382	A03560	404	A00382	A03783	404
A00382	A03863	502	A00382	A04075	251
A00382	A04212	491	A00382	A04499	162
A00382	A05645	270	A00382	A05712	477
A00382	A05738	346	A00382	A06204	489
A00382	A06295	152	A00382	A06347	491
A00382	A06650	491	A00382	A06698	366
A00382	A06832	154	A00382	A06892	162
A00382	A07247	457	A00382	A07572	235
A00382	A07604	179	A00382	A07706	379
A00382	A08118	234	A00382	A08137	480
A00382	A08183	446	A00382	A08290	258
A00382	A08340	217	A00382	A08546	488
A00382	A08571	479	A00382	A08627	201

A00382	A08707	402	A00382	A08859	402
A00382	A08896	424	A00382	A08989	193
A00382	A08991	404	A00382	A09004	156
A00382	A09197	185	A00382	A09397	221
A00382	A09673	182	A00382	A10006	363
A00382	A10266	185	A00382	A10465	201
A00382	A10532	658	A00382	A10632	270
A00382	A11240	372	A00382	A11300	349
A00382	A11316	272	A00382	A11409	491
A00382	A11476	159	A00382	A11913	425
A00382	A12202	323	A00382	A12323	221
A00382	A12656	200	A00382	A12673	258
A00382	A12757	216	A00382	A12862	154
A00382	A13308	174	A00382	A13534	258
A00382	A13609	486	A00382	A13989	537
A00382	A14006	404	A00382	A14186	488
A00382	A14196	465	A00382	A14200	409
A00382	A14220	451	A00382	A14246	549
A00382	A14546	223	A00382	A14596	491
A00382	A15204	200	A00382	A15205	185
A00382	A15206	185	A00382	A15207	185
A00382	A15210	200	A00382	A15237	193
A00382	A15292	230	A00382	A15297	381
A00382	A15323	427	A00382	A15324	490
A00382	A15334	183	A00382	A15353	491
A00382	A15370	491	A00382	A15374	341
A00382	A15419	265	A00382	A15559	210
A00382	A15564	384	A00382	A15660	243
A00382	A15698	372	A00382	A15717	302
A00382	A15724	475	A00382	A15763	301
A00382	A15867	419	A00382	A16575	179
A00382	A16661	162	A00382	A16664	205
A00382	A16904	340	A00382	A17063	163
A00382	A17089	491	A00383	A00537	152
A00383	A00741	270	A00383	A00998	393
A00383	A01086	263	A00383	A01155	346
A00383	A01161	124	A00383	A01210	271
A00383	A01336	351	A00383	A01413	307
A00383	A01447	285	A00383	A01540	184
A00383	A09709	386	A00384	A00669	154
A00384	A00852	486	A00384	A00906	259
A00384	A01359	191	A00384	A02642	378
A00384	A03157	180	A00384	A13146	276
A00385	A00429	452	A00385	A00437	183
A00385	A00483	240	A00385	A00532	210
A00385	A00537	301	A00385	A00981	259

A00385	A01211	184	A00385	A01373	260
A00385	A01417	196	A00385	A01669	160
A00385	A01897	157	A00385	A01970	235
A00385	A01997	226	A00385	A02010	316
A00385	A02049	291	A00385	A02054	313
A00385	A02158	424	A00385	A02180	488
A00385	A02225	353	A00385	A02621	184
A00385	A02654	160	A00385	A02666	246
A00385	A02815	319	A00385	A02836	177
A00385	A03054	329	A00385	A03070	172
A00385	A03080	357	A00385	A03390	383
A00385	A03401	491	A00385	A03731	278
A00385	A03744	378	A00385	A03748	205
A00385	A03879	219	A00385	A03910	240
A00385	A04361	157	A00385	A04754	299
A00385	A04928	216	A00385	A05617	219
A00385	A06211	283	A00385	A06584	160
A00385	A06828	319	A00385	A07394	184
A00385	A09111	299	A00385	A09131	179
A00385	A09205	219	A00385	A09206	241
A00385	A09230	311	A00385	A09234	319
A00385	A09235	165	A00385	A09363	190
A00385	A09745	162	A00385	A09761	347
A00385	A10018	159	A00385	A11068	165
A00385	A11359	362	A00385	A11394	465
A00385	A11526	210	A00385	A11858	177
A00385	A12037	441	A00385	A12140	294
A00385	A12326	294	A00385	A12644	274
A00385	A12722	196	A00385	A13133	340
A00385	A13942	461	A00385	A14267	483
A00385	A14378	412	A00385	A14611	340
A00385	A15493	409	A00385	A15494	350
A00385	A15911	200	A00385	A16531	152
A00386	A00531	282	A00386	A00568	174
A00386	A00651	283	A00386	A00684	162
A00386	A01064	474	A00386	A01093	271
A00386	A01359	497	A00386	A01425	319
A00386	A01492	162	A00386	A01524	208
A00386	A01557	65	A00386	A01559	440
A00386	A01650	202	A00386	A01660	390
A00386	A02180	112	A00386	A02206	424
A00386	A02219	404	A00386	A02524	182
A00386	A03297	200	A00386	A03401	413
A00386	A03762	268	A00386	A03981	319
A00386	A04316	213	A00386	A04403	347
A00386	A04666	497	A00386	A04668	385

A00386	A05620	291	A00386	A05721	221
A00386	A06597	553	A00386	A06814	383
A00386	A07140	157	A00386	A07184	245
A00386	A08698	159	A00386	A09372	324
A00386	A10353	193	A00386	A10532	199
A00386	A14272	443	A00386	A14299	269
A00386	A14443	165	A00386	A14444	363
A00386	A14661	380	A00386	A14674	497
A00386	A15277	179	A00386	A15420	322
A00386	A15850	313	A00386	A15941	283
A00386	A16738	497	A00387	A00513	391
A00387	A00751	238	A00387	A01459	249
A00387	A02747	367	A00387	A07135	167
A00387	A11284	238	A00387	A12047	212
A00387	A12264	352	A00388	A00411	486
A00388	A00418	899	A00388	A00450	406
A00388	A00671	255	A00388	A00754	491
A00388	A00778	485	A00388	A00809	257
A00388	A00812	491	A00388	A01146	491
A00388	A01149	407	A00388	A01212	350
A00388	A01262	473	A00388	A01374	296
A00388	A01459	490	A00388	A01478	491
A00388	A01479	281	A00388	A01514	589
A00388	A01537	491	A00388	A01576	199
A00388	A01591	172	A00388	A01593	899
A00388	A01633	243	A00388	A01685	249
A00388	A01687	408	A00388	A01689	491
A00388	A01708	463	A00388	A01871	199
A00388	A01900	202	A00388	A01976	479
A00388	A01988	475	A00388	A02215	428
A00388	A02381	491	A00388	A02998	378
A00388	A03637	188	A00388	A04079	234
A00388	A04553	443	A00388	A04693	460
A00388	A05008	454	A00388	A05009	430
A00388	A05124	260	A00388	A05474	174
A00388	A05880	458	A00388	A05892	157
A00388	A06143	334	A00388	A06348	491
A00388	A06572	486	A00388	A06661	327
A00388	A06864	183	A00388	A07313	188
A00388	A07425	486	A00388	A07433	491
A00388	A07495	188	A00388	A07593	491
A00388	A07598	168	A00388	A07961	333
A00388	A08059	201	A00388	A08184	490
A00388	A08185	485	A00388	A08196	483
A00388	A08402	157	A00388	A08463	411
A00388	A08503	481	A00388	A08525	491

A00388	A08549	462	A00388	A08557	160
A00388	A08814	489	A00388	A08839	213
A00388	A08847	441	A00388	A08853	489
A00388	A08854	469	A00388	A08902	265
A00388	A09052	434	A00388	A09256	480
A00388	A09320	238	A00388	A09643	229
A00388	A09660	456	A00388	A09767	160
A00388	A10373	391	A00388	A10607	267
A00388	A10673	422	A00388	A11656	219
A00388	A11884	357	A00388	A12533	393
A00388	A12553	185	A00388	A12595	174
A00388	A12932	420	A00388	A13082	201
A00388	A13591	199	A00388	A13949	330
A00388	A14133	201	A00388	A14209	174
A00388	A14221	395	A00388	A14222	210
A00388	A14227	491	A00388	A14272	447
A00388	A14279	441	A00388	A14285	177
A00388	A14289	434	A00388	A14306	174
A00388	A14307	201	A00388	A14309	465
A00388	A15123	259	A00388	A15367	361
A00388	A15380	332	A00388	A15533	491
A00388	A15910	174	A00388	A15949	217
A00388	A16128	321	A00388	A16131	386
A00388	A16602	201	A00388	A16605	174
A00389	A00411	154	A00389	A00444	230
A00389	A00667	401	A00389	A00687	288
A00389	A00710	409	A00389	A00913	247
A00389	A00915	207	A00389	A01188	419
A00389	A01190	324	A00389	A01194	427
A00389	A01211	396	A00389	A01353	286
A00389	A01411	259	A00389	A01455	412
A00389	A01495	162	A00389	A01504	313
A00389	A01506	163	A00389	A01582	260
A00389	A01826	299	A00389	A01937	301
A00389	A02051	408	A00389	A02487	413
A00389	A02542	305	A00389	A02998	296
A00389	A03029	461	A00389	A03180	457
A00389	A03439	256	A00389	A03446	213
A00389	A03453	490	A00389	A03676	223
A00389	A04055	291	A00389	A04066	412
A00389	A04495	212	A00389	A05025	243
A00389	A05154	230	A00389	A06030	328
A00389	A09042	245	A00389	A09056	193
A00389	A09587	460	A00389	A10532	335
A00389	A11409	411	A00389	A12903	278
A00389	A13659	361	A00389	A14594	263

A00389	A14622	208	A00389	A15469	301
A00389	A15673	157	A00389	A15920	174
A00390	A00416	411	A00390	A00630	488
A00390	A00631	480	A00390	A00925	249
A00390	A01274	223	A00390	A01291	484
A00390	A01522	328	A00390	A01606	491
A00390	A01681	423	A00390	A01724	283
A00390	A01930	228	A00390	A03048	380
A00390	A03336	489	A00390	A03862	199
A00390	A03891	462	A00390	A04880	174
A00390	A05370	450	A00390	A06097	281
A00390	A07220	374	A00390	A08144	177
A00390	A09229	184	A00390	A09303	174
A00390	A09326	215	A00390	A11293	305
A00390	A11514	312	A00390	A12282	208
A00390	A13992	439	A00391	A00454	333
A00391	A00457	177	A00391	A00483	241
A00391	A00544	299	A00391	A00548	241
A00391	A00564	549	A00391	A00571	263
A00391	A00576	299	A00391	A00640	152
A00391	A00653	355	A00391	A00688	241
A00391	A00726	562	A00391	A00810	152
A00391	A00833	299	A00391	A00849	160
A00391	A00974	299	A00391	A00984	520
A00391	A00987	368	A00391	A01001	299
A00391	A01004	241	A00391	A01007	302
A00391	A01102	299	A00391	A01147	385
A00391	A01221	151	A00391	A01311	152
A00391	A01379	529	A00391	A01387	585
A00391	A01417	241	A00391	A01439	299
A00391	A01577	152	A00391	A01627	299
A00391	A01661	241	A00391	A01711	346
A00391	A02416	241	A00391	A02601	299
A00391	A03415	238	A00391	A03697	152
A00391	A03981	190	A00391	A04495	299
A00391	A06287	152	A00391	A07744	152
A00391	A08061	152	A00391	A08098	152
A00391	A08178	152	A00391	A08763	152
A00391	A08861	241	A00391	A08947	299
A00391	A09004	299	A00391	A10842	152
A00391	A14299	241	A00391	A14525	152
A00391	A14981	422	A00391	A15375	152
A00391	A15546	152	A00392	A15852	310
A00393	A00440	312	A00393	A00496	468
A00393	A00560	339	A00393	A00592	311
A00393	A00835	226	A00393	A00940	156

A00393	A00954	156	A00393	A00957	481
A00393	A01075	188	A00393	A01143	491
A00393	A01426	235	A00393	A03136	170
A00393	A04449	364	A00393	A11291	174
A00393	A11648	179	A00393	A13313	232
A00393	A13329	340	A00393	A13587	425
A00393	A13928	319	A00393	A14490	274
A00393	A14527	159	A00393	A15366	490
A00393	A15492	430	A00393	A15773	238
A00393	A15852	327	A00393	A15867	168
A00393	A16566	263	A00393	A16724	259
A00394	A01729	168	A00394	A02450	227
A00394	A03246	279	A00395	A00449	491
A00395	A00453	174	A00395	A00468	457
A00395	A00478	294	A00395	A00541	489
A00395	A00574	491	A00395	A00625	240
A00395	A00628	353	A00395	A00633	229
A00395	A00651	283	A00395	A00654	488
A00395	A00659	294	A00395	A00660	485
A00395	A00662	433	A00395	A00663	281
A00395	A00666	313	A00395	A00681	416
A00395	A00695	327	A00395	A00696	486
A00395	A00738	337	A00395	A00739	334
A00395	A00770	441	A00395	A00825	473
A00395	A00962	485	A00395	A01005	451
A00395	A01008	413	A00395	A01018	491
A00395	A01088	332	A00395	A01174	461
A00395	A01176	213	A00395	A01222	435
A00395	A01223	472	A00395	A01225	491
A00395	A01227	479	A00395	A01230	456
A00395	A01233	223	A00395	A01297	491
A00395	A01344	369	A00395	A01367	208
A00395	A01397	401	A00395	A01409	491
A00395	A01503	154	A00395	A01635	474
A00395	A01643	348	A00395	A01660	183
A00395	A01713	379	A00395	A01714	340
A00395	A01743	216	A00395	A01822	208
A00395	A01829	170	A00395	A01899	486
A00395	A01931	163	A00395	A01932	319
A00395	A01982	179	A00395	A02008	302
A00395	A02011	281	A00395	A02014	323
A00395	A02071	245	A00395	A02083	240
A00395	A02114	188	A00395	A02168	207
A00395	A02192	296	A00395	A02254	326
A00395	A02310	152	A00395	A02316	196
A00395	A02468	238	A00395	A02521	486

A00395	A02545	221	A00395	A02574	226
A00395	A02992	467	A00395	A03004	168
A00395	A03016	369	A00395	A03050	452
A00395	A03087	329	A00395	A03099	291
A00395	A03146	326	A00395	A03148	382
A00395	A03167	393	A00395	A03423	159
A00395	A03484	443	A00395	A03513	491
A00395	A03550	184	A00395	A03795	485
A00395	A03837	362	A00395	A04020	408
A00395	A04115	445	A00395	A04179	489
A00395	A04228	491	A00395	A04366	170
A00395	A04369	213	A00395	A04556	185
A00395	A04674	271	A00395	A04713	343
A00395	A04791	481	A00395	A04834	168
A00395	A04839	246	A00395	A04883	430
A00395	A04927	394	A00395	A05006	350
A00395	A05007	223	A00395	A05014	394
A00395	A05045	480	A00395	A05050	218
A00395	A05138	165	A00395	A05140	213
A00395	A05310	232	A00395	A05337	151
A00395	A05358	171	A00395	A05376	251
A00395	A05393	463	A00395	A05416	308
A00395	A05418	446	A00395	A05424	162
A00395	A05431	163	A00395	A05451	229
A00395	A05463	299	A00395	A05548	193
A00395	A05573	484	A00395	A05753	398
A00395	A05754	391	A00395	A05757	267
A00395	A05774	338	A00395	A05789	347
A00395	A05883	391	A00395	A05937	207
A00395	A06284	380	A00395	A06358	219
A00395	A06385	400	A00395	A06405	489
A00395	A06439	491	A00395	A06607	307
A00395	A06626	246	A00395	A06641	157
A00395	A06847	183	A00395	A06848	236
A00395	A06855	413	A00395	A06860	395
A00395	A06912	302	A00395	A06916	205
A00395	A06970	394	A00395	A06994	340
A00395	A07027	374	A00395	A07037	205
A00395	A07059	491	A00395	A07067	344
A00395	A07088	491	A00395	A07109	475
A00395	A07226	319	A00395	A07238	177
A00395	A07239	491	A00395	A07281	470
A00395	A07322	191	A00395	A07325	249
A00395	A07334	263	A00395	A07407	283
A00395	A07477	322	A00395	A07490	236
A00395	A07520	490	A00395	A07530	183

A00395	A07553	358	A00395	A07590	313
A00395	A07613	425	A00395	A07674	490
A00395	A07684	160	A00395	A07688	243
A00395	A07692	232	A00395	A07711	205
A00395	A07736	274	A00395	A07737	180
A00395	A07739	472	A00395	A07757	363
A00395	A07763	281	A00395	A07779	344
A00395	A07795	279	A00395	A07796	205
A00395	A07800	318	A00395	A07803	497
A00395	A07847	355	A00395	A07856	241
A00395	A07926	279	A00395	A07933	393
A00395	A07951	414	A00395	A08155	411
A00395	A08199	183	A00395	A08238	190
A00395	A08293	319	A00395	A08366	374
A00395	A08368	290	A00395	A08636	290
A00395	A08690	236	A00395	A10424	221
A00395	A10446	293	A00395	A10457	213
A00395	A10458	221	A00395	A10500	344
A00395	A10532	199	A00395	A10635	326
A00395	A10652	469	A00395	A10686	196
A00395	A10687	224	A00395	A10690	411
A00395	A10692	257	A00395	A10696	467
A00395	A10703	157	A00395	A10708	416
A00395	A10845	344	A00395	A11021	486
A00395	A11189	157	A00395	A11606	364
A00395	A12033	271	A00395	A12699	344
A00395	A13065	414	A00395	A14208	263
A00395	A14216	168	A00395	A14549	246
A00395	A15386	497	A00395	A15684	190
A00395	A15874	350	A00395	A15970	177
A00395	A16027	281	A00395	A16103	183
A00395	A16216	383	A00395	A16741	443
A00395	A16744	160	A00395	A17006	400
A00395	A17078	479	A00396	A00430	243
A00396	A00446	299	A00396	A00471	221
A00396	A00498	475	A00396	A00517	240
A00396	A00564	252	A00396	A00568	317
A00396	A00613	258	A00396	A00614	285
A00396	A00664	530	A00396	A00670	491
A00396	A00671	270	A00396	A00684	227
A00396	A00855	338	A00396	A00943	477
A00396	A00968	624	A00396	A01021	491
A00396	A01074	738	A00396	A01105	499
A00396	A01169	474	A00396	A01189	288
A00396	A01205	491	A00396	A01244	355
A00396	A01269	257	A00396	A01360	491

A00396	A01374	246	A00396	A01375	479
A00396	A01409	307	A00396	A01419	228
A00396	A01459	218	A00396	A01483	491
A00396	A01506	229	A00396	A01508	419
A00396	A01524	899	A00396	A01568	307
A00396	A01622	531	A00396	A01647	307
A00396	A01679	317	A00396	A01844	198
A00396	A01978	276	A00396	A02061	170
A00396	A02086	165	A00396	A02774	188
A00396	A03095	491	A00396	A03123	188
A00396	A03259	401	A00396	A03272	204
A00396	A03468	391	A00396	A03501	307
A00396	A03710	317	A00396	A03880	307
A00396	A04307	371	A00396	A04769	154
A00396	A04977	274	A00396	A05262	347
A00396	A05332	619	A00396	A05433	232
A00396	A05609	612	A00396	A06085	201
A00396	A06101	324	A00396	A06111	157
A00396	A06203	210	A00396	A06689	230
A00396	A06718	491	A00396	A06819	491
A00396	A06924	313	A00396	A06970	329
A00396	A06990	489	A00396	A07007	307
A00396	A07222	620	A00396	A07277	246
A00396	A07333	486	A00396	A07571	435
A00396	A07816	307	A00396	A07880	488
A00396	A07931	491	A00396	A07974	618
A00396	A07981	307	A00396	A08097	317
A00396	A08402	182	A00396	A08939	202
A00396	A09054	307	A00396	A09135	217
A00396	A09153	340	A00396	A09316	183
A00396	A09340	352	A00396	A09350	173
A00396	A09667	177	A00396	A10159	293
A00396	A11224	173	A00396	A13819	307
A00396	A13866	307	A00396	A14135	154
A00396	A14239	307	A00396	A14525	488
A00396	A14589	351	A00396	A14803	388
A00396	A14810	168	A00396	A14858	221
A00396	A15372	491	A00396	A15482	191
A00396	A15837	358	A00396	A16748	152
A00396	A16753	475	A00396	A16761	183
A00396	A16769	165	A00396	A16807	307
A00397	A00406	484	A00397	A00421	491
A00397	A00444	205	A00397	A00448	491
A00397	A00452	386	A00397	A00456	469
A00397	A00459	174	A00397	A00463	174
A00397	A00488	480	A00397	A00497	441

A00397	A00503	416	A00397	A00520	165
A00397	A00532	439	A00397	A00550	283
A00397	A00551	504	A00397	A00554	398
A00397	A00555	313	A00397	A00570	461
A00397	A00584	547	A00397	A00589	285
A00397	A00595	552	A00397	A00605	491
A00397	A00646	379	A00397	A00648	416
A00397	A00687	439	A00397	A00690	224
A00397	A00697	369	A00397	A00749	174
A00397	A00763	63	A00397	A00767	751
A00397	A00805	294	A00397	A00813	487
A00397	A00822	441	A00397	A00864	416
A00397	A00878	229	A00397	A00879	279
A00397	A00883	676	A00397	A00905	480
A00397	A00908	691	A00397	A00916	630
A00397	A00918	679	A00397	A00928	489
A00397	A00934	430	A00397	A00945	159
A00397	A00960	549	A00397	A00992	695
A00397	A01020	677	A00397	A01025	241
A00397	A01029	457	A00397	A01040	362
A00397	A01053	63	A00397	A01075	672
A00397	A01088	308	A00397	A01098	63
A00397	A01107	340	A00397	A01116	424
A00397	A01120	252	A00397	A01136	432
A00397	A01149	267	A00397	A01166	483
A00397	A01184	489	A00397	A01193	694
A00397	A01201	491	A00397	A01206	490
A00397	A01224	63	A00397	A01234	491
A00397	A01243	443	A00397	A01266	458
A00397	A01268	491	A00397	A01277	182
A00397	A01279	695	A00397	A01291	260
A00397	A01304	416	A00397	A01309	443
A00397	A01334	174	A00397	A01351	695
A00397	A01352	227	A00397	A01356	227
A00397	A01384	162	A00397	A01386	495
A00397	A01424	550	A00397	A01425	223
A00397	A01439	180	A00397	A01442	296
A00397	A01451	405	A00397	A01459	481
A00397	A01468	377	A00397	A01470	491
A00397	A01488	486	A00397	A01492	552
A00397	A01514	384	A00397	A01539	259
A00397	A01579	366	A00397	A01598	491
A00397	A01612	491	A00397	A01614	488
A00397	A01615	327	A00397	A01649	490
A00397	A01778	266	A00397	A01988	341
A00397	A02164	63	A00397	A02212	165

A00397	A02265	63	A00397	A02456	152
A00397	A02535	491	A00397	A03438	481
A00397	A03494	491	A00397	A03567	205
A00397	A03569	469	A00397	A03597	291
A00397	A04104	383	A00397	A04848	489
A00397	A05230	312	A00397	A05617	362
A00397	A06204	491	A00397	A06275	400
A00397	A06347	491	A00397	A06351	176
A00397	A06698	549	A00397	A06832	152
A00397	A07244	694	A00397	A07347	481
A00397	A07432	671	A00397	A07524	706
A00397	A07537	486	A00397	A07584	413
A00397	A07625	537	A00397	A07700	477
A00397	A07706	205	A00397	A07924	552
A00397	A08137	573	A00397	A08183	490
A00397	A08201	335	A00397	A08267	184
A00397	A08357	312	A00397	A08447	486
A00397	A08807	706	A00397	A08918	168
A00397	A08982	254	A00397	A09004	154
A00397	A09324	312	A00397	A09352	63
A00397	A09664	543	A00397	A10250	322
A00397	A10317	480	A00397	A10465	485
A00397	A10616	398	A00397	A10710	435
A00397	A11316	201	A00397	A11544	174
A00397	A11618	257	A00397	A11831	433
A00397	A11832	510	A00397	A11913	491
A00397	A11965	216	A00397	A12097	223
A00397	A12177	184	A00397	A12202	219
A00397	A12722	621	A00397	A12757	469
A00397	A13068	350	A00397	A13076	330
A00397	A13165	257	A00397	A13357	335
A00397	A13792	388	A00397	A13875	314
A00397	A13989	563	A00397	A14186	491
A00397	A14192	416	A00397	A14200	279
A00397	A14246	152	A00397	A14597	180
A00397	A15297	480	A00397	A15324	467
A00397	A15338	358	A00397	A15353	469
A00397	A15370	738	A00397	A15374	460
A00397	A15724	490	A00397	A15764	484
A00397	A16302	447	A00397	A16544	257
A00397	A16703	433	A00397	A17089	486
A00398	A00748	611	A00398	A00805	258
A00398	A00880	485	A00398	A01252	764
A00398	A01302	772	A00398	A01461	162
A00398	A01514	151	A00398	A01585	152
A00398	A01706	432	A00398	A05216	458

A00398	A05454	283	A00398	A06687	611
A00398	A07399	317	A00398	A08293	193
A00398	A08356	184	A00398	A08555	611
A00398	A08821	353	A00398	A09298	611
A00398	A09609	429	A00398	A10532	238
A00398	A11497	187	A00398	A11735	179
A00398	A12200	480	A00398	A12253	172
A00398	A12508	249	A00398	A12738	180
A00398	A12850	168	A00398	A12858	335
A00398	A13711	303	A00398	A13983	491
A00398	A14167	266	A00398	A14182	263
A00398	A14199	452	A00398	A14247	599
A00398	A14661	283	A00398	A14711	283
A00398	A15315	607	A00398	A15347	666
A00398	A15528	756	A00398	A15555	180
A00398	A15617	513	A00398	A16113	263
A00398	A16364	283	A00399	A00551	438
A00399	A00655	226	A00399	A00708	695
A00399	A00774	170	A00399	A00819	229
A00399	A00911	277	A00399	A00934	438
A00399	A00961	236	A00399	A00988	224
A00399	A01062	456	A00399	A01099	246
A00399	A01120	439	A00399	A01166	401
A00399	A01206	694	A00399	A01295	363
A00399	A01306	182	A00399	A01349	695
A00399	A01429	520	A00399	A01430	456
A00399	A01470	267	A00399	A01598	455
A00399	A02278	263	A00399	A03681	168
A00399	A07348	690	A00399	A07822	249
A00399	A08211	438	A00399	A08461	491
A00399	A09009	198	A00399	A11496	302
A00399	A11497	467	A00399	A12138	695
A00399	A12255	201	A00399	A14214	154
A00399	A14223	152	A00399	A14508	154
A00399	A15366	227	A00399	A15519	190
A00399	A15774	334	A00400	A00421	419
A00400	A00440	491	A00400	A00450	490
A00400	A00471	659	A00400	A00531	176
A00400	A00532	355	A00400	A00537	663
A00400	A00564	199	A00400	A00566	491
A00400	A00571	168	A00400	A00643	491
A00400	A00654	404	A00400	A00655	208
A00400	A00765	357	A00400	A00786	489
A00400	A00799	180	A00400	A00805	160
A00400	A00847	451	A00400	A00858	441
A00400	A00864	283	A00400	A00866	372

A00400	A00868	401	A00400	A00883	658
A00400	A00935	372	A00400	A00954	294
A00400	A00967	334	A00400	A00981	466
A00400	A01091	241	A00400	A01139	277
A00400	A01149	484	A00400	A01151	488
A00400	A01161	307	A00400	A01169	319
A00400	A01203	372	A00400	A01211	263
A00400	A01212	658	A00400	A01213	217
A00400	A01254	236	A00400	A01322	198
A00400	A01329	657	A00400	A01359	414
A00400	A01378	326	A00400	A01451	188
A00400	A01459	154	A00400	A01472	338
A00400	A01507	400	A00400	A01514	283
A00400	A01527	177	A00400	A01558	190
A00400	A01568	261	A00400	A01580	207
A00400	A01591	395	A00400	A01627	372
A00400	A01647	658	A00400	A01682	630
A00400	A01692	226	A00400	A01926	334
A00400	A01988	283	A00400	A01995	488
A00400	A02202	241	A00400	A02481	334
A00400	A03014	283	A00400	A03188	435
A00400	A03304	466	A00400	A03436	156
A00400	A03591	365	A00400	A03819	185
A00400	A03873	334	A00400	A03893	200
A00400	A03939	386	A00400	A03981	243
A00400	A04033	491	A00400	A04232	183
A00400	A04449	636	A00400	A04492	467
A00400	A04747	154	A00400	A04773	417
A00400	A05443	157	A00400	A05458	226
A00400	A05780	165	A00400	A05897	165
A00400	A06100	324	A00400	A06129	461
A00400	A06297	336	A00400	A06588	380
A00400	A06750	603	A00400	A07209	216
A00400	A07232	480	A00400	A07670	426
A00400	A07706	214	A00400	A07726	363
A00400	A07803	416	A00400	A08022	238
A00400	A08376	182	A00400	A08402	374
A00400	A08571	486	A00400	A08850	334
A00400	A08878	332	A00400	A09006	173
A00400	A09256	196	A00400	A09258	433
A00400	A09377	363	A00400	A09451	480
A00400	A09474	240	A00400	A09511	216
A00400	A09515	429	A00400	A09538	334
A00400	A09603	165	A00400	A09996	474
A00400	A10250	600	A00400	A10283	240
A00400	A10340	274	A00400	A10342	374

A00400	A10962	274	A00400	A11011	274
A00400	A11143	240	A00400	A11394	260
A00400	A11492	335	A00400	A11559	334
A00400	A11698	173	A00400	A11983	451
A00400	A12047	243	A00400	A12111	185
A00400	A12284	658	A00400	A12365	429
A00400	A12373	334	A00400	A12681	460
A00400	A12824	372	A00400	A13892	188
A00400	A13917	165	A00400	A13986	193
A00400	A14084	640	A00400	A14210	334
A00400	A14272	460	A00400	A14278	484
A00400	A14371	334	A00400	A14410	327
A00400	A14484	491	A00400	A14633	517
A00400	A14635	488	A00400	A14636	637
A00400	A14827	178	A00400	A15273	154
A00400	A15367	455	A00400	A16294	366
A00400	A16635	486	A00401	A00688	182
A00401	A00765	160	A00401	A00766	247
A00401	A00791	165	A00401	A00868	480
A00401	A00883	423	A00401	A00956	256
A00401	A01117	255	A00401	A01203	414
A00401	A01370	256	A00401	A01371	317
A00401	A03570	377	A00401	A06137	328
A00401	A08022	180	A00401	A14196	185
A00401	A15297	288	A00402	A00667	162
A00402	A00759	162	A00402	A00873	260
A00402	A00969	408	A00402	A01101	481
A00402	A01291	491	A00402	A02120	268
A00402	A03246	176	A00402	A04932	168
A00402	A07802	168	A00402	A08450	271
A00402	A08501	274	A00402	A08505	157
A00402	A08518	168	A00402	A08521	177
A00402	A08543	270	A00402	A08550	299
A00402	A08552	193	A00402	A08560	396
A00402	A08561	327	A00402	A08844	353
A00402	A09009	221	A00402	A09298	156
A00402	A09717	224	A00402	A09828	202
A00402	A10789	205	A00402	A11497	216
A00402	A11616	201	A00402	A11648	174
A00402	A11688	165	A00402	A11851	205
A00402	A11940	263	A00402	A12011	259
A00402	A12255	223	A00402	A12507	260
A00402	A12606	193	A00402	A12658	390
A00402	A12665	291	A00402	A12725	316
A00402	A12858	268	A00402	A12873	281
A00402	A13246	286	A00402	A13342	221

A00402	A13356	157	A00402	A13399	276
A00402	A13781	160	A00402	A13805	240
A00402	A13809	180	A00402	A14239	154
A00402	A14255	168	A00402	A14569	187
A00402	A14903	256	A00402	A15519	212
A00402	A15541	154	A00402	A15653	174
A00402	A16157	303	A00402	A16187	204
A00402	A16261	196	A00402	A16322	160
A00402	A16587	240	A00402	A16611	335
A00402	A16664	294	A00402	A16680	281
A00402	A16838	227	A00402	A16874	371
A00402	A17015	208	A00403	A00521	221
A00403	A00560	321	A00403	A00590	196
A00403	A00629	412	A00403	A00787	252
A00403	A00883	246	A00403	A00923	491
A00403	A01000	491	A00403	A01017	457
A00403	A01025	488	A00403	A01047	475
A00403	A01066	157	A00403	A01096	270
A00403	A01101	491	A00403	A01120	306
A00403	A01140	414	A00403	A01184	404
A00403	A01206	450	A00403	A01234	408
A00403	A01249	417	A00403	A01282	436
A00403	A01291	491	A00403	A01306	491
A00403	A01335	257	A00403	A01338	491
A00403	A01437	441	A00403	A01441	210
A00403	A01470	297	A00403	A01598	232
A00403	A01617	245	A00403	A01649	475
A00403	A01700	151	A00403	A01718	85
A00403	A02978	154	A00403	A03069	461
A00403	A03567	491	A00403	A03571	182
A00403	A03754	285	A00403	A03859	330
A00403	A05385	385	A00403	A05712	190
A00403	A06279	127	A00403	A06861	351
A00403	A06965	486	A00403	A07451	71
A00403	A07604	201	A00403	A08137	224
A00403	A08357	241	A00403	A08664	243
A00403	A09322	491	A00403	A09674	252
A00403	A09702	226	A00403	A11390	180
A00403	A11438	485	A00403	A11480	465
A00403	A11704	488	A00403	A12233	469
A00403	A12270	221	A00403	A13989	179
A00403	A13992	96	A00403	A14188	454
A00403	A14216	163	A00403	A15218	458
A00403	A15221	193	A00403	A15235	312
A00403	A15743	232	A00403	A16118	470
A00403	A16523	308	A00403	A16631	190

A00404	A00435	243	A00404	A00444	173
A00404	A00651	436	A00404	A00700	486
A00404	A00713	196	A00404	A00839	367
A00404	A01085	232	A00404	A01112	254
A00404	A01127	347	A00404	A01166	221
A00404	A01291	329	A00404	A01335	221
A00404	A01411	195	A00404	A01468	412
A00404	A01496	286	A00404	A01538	210
A00404	A01540	196	A00404	A01579	265
A00404	A01649	174	A00404	A03437	307
A00404	A05501	445	A00404	A07323	258
A00404	A09707	256	A00404	A11986	302
A00404	A13989	477	A00404	A15659	188
A00404	A15988	199	A00405	A00427	353
A00405	A00447	165	A00405	A00457	247
A00405	A00502	591	A00405	A00510	477
A00405	A00521	491	A00405	A00529	176
A00405	A00531	221	A00405	A00532	248
A00405	A00537	597	A00405	A00544	425
A00405	A00547	210	A00405	A00590	474
A00405	A00598	486	A00405	A00603	488
A00405	A00604	592	A00405	A00605	347
A00405	A00608	396	A00405	A00648	490
A00405	A00649	591	A00405	A00651	416
A00405	A00653	592	A00405	A00697	279
A00405	A00731	589	A00405	A00750	439
A00405	A00760	490	A00405	A00780	337
A00405	A00783	591	A00405	A00785	591
A00405	A00787	491	A00405	A00790	589
A00405	A00809	294	A00405	A00839	353
A00405	A00849	247	A00405	A00865	347
A00405	A00870	490	A00405	A00878	491
A00405	A00953	592	A00405	A00965	474
A00405	A00974	569	A00405	A00987	210
A00405	A00994	460	A00405	A01022	356
A00405	A01023	157	A00405	A01025	385
A00405	A01027	591	A00405	A01037	299
A00405	A01042	427	A00405	A01054	486
A00405	A01067	480	A00405	A01075	310
A00405	A01081	247	A00405	A01086	590
A00405	A01095	305	A00405	A01101	251
A00405	A01108	592	A00405	A01150	356
A00405	A01161	551	A00405	A01204	578
A00405	A01234	322	A00405	A01246	467
A00405	A01265	352	A00405	A01287	342
A00405	A01291	490	A00405	A01346	591

A00405	A01352	277	A00405	A01382	246
A00405	A01385	228	A00405	A01392	481
A00405	A01399	592	A00405	A01419	302
A00405	A01433	474	A00405	A01443	499
A00405	A01446	491	A00405	A01448	486
A00405	A01458	517	A00405	A01496	299
A00405	A01540	480	A00405	A01573	302
A00405	A01576	199	A00405	A01649	434
A00405	A01711	240	A00405	A02364	428
A00405	A02814	199	A00405	A02994	247
A00405	A03752	218	A00405	A03880	433
A00405	A03959	276	A00405	A03981	162
A00405	A04668	329	A00405	A04895	480
A00405	A05029	156	A00405	A05121	160
A00405	A05155	346	A00405	A05234	428
A00405	A05387	288	A00405	A06959	449
A00405	A07237	457	A00405	A07311	224
A00405	A07350	256	A00405	A07362	329
A00405	A07569	311	A00405	A07597	490
A00405	A07773	263	A00405	A07792	224
A00405	A08011	490	A00405	A08133	199
A00405	A08252	288	A00405	A08398	165
A00405	A08452	246	A00405	A08773	489
A00405	A08835	285	A00405	A08982	217
A00405	A08987	474	A00405	A08990	180
A00405	A09332	219	A00405	A10199	462
A00405	A10535	440	A00405	A11015	183
A00405	A11322	377	A00405	A11338	385
A00405	A11419	481	A00405	A11427	460
A00405	A11677	574	A00405	A13683	183
A00405	A13684	208	A00405	A13963	278
A00405	A13989	263	A00405	A14216	269
A00405	A14272	404	A00405	A14482	356
A00405	A14568	157	A00405	A14655	215
A00405	A14699	247	A00405	A14778	247
A00405	A14825	273	A00405	A14888	411
A00405	A15322	260	A00405	A15345	274
A00405	A15384	428	A00405	A15624	423
A00405	A15651	443	A00405	A15661	384
A00405	A15684	260	A00405	A15932	433
A00405	A15949	274	A00405	A16157	472
A00405	A16587	247	A00405	A16876	183
A00406	A00448	638	A00406	A00459	252
A00406	A00488	633	A00406	A00490	358
A00406	A00497	635	A00406	A00500	168
A00406	A00518	438	A00406	A00520	472

A00406	A00521	177	A00406	A00532	319
A00406	A00550	623	A00406	A00551	433
A00406	A00554	458	A00406	A00570	205
A00406	A00571	185	A00406	A00575	260
A00406	A00584	353	A00406	A00595	457
A00406	A00605	515	A00406	A00648	427
A00406	A00687	262	A00406	A00693	205
A00406	A00734	167	A00406	A00831	334
A00406	A00856	252	A00406	A00871	173
A00406	A00878	246	A00406	A00916	491
A00406	A00923	294	A00406	A00934	490
A00406	A00944	468	A00406	A00960	156
A00406	A00978	363	A00406	A00992	180
A00406	A01000	180	A00406	A01020	638
A00406	A01040	334	A00406	A01047	263
A00406	A01092	243	A00406	A01112	334
A00406	A01116	638	A00406	A01127	232
A00406	A01150	288	A00406	A01166	356
A00406	A01184	491	A00406	A01201	491
A00406	A01206	428	A00406	A01214	227
A00406	A01215	199	A00406	A01224	422
A00406	A01226	82	A00406	A01234	491
A00406	A01291	432	A00406	A01304	638
A00406	A01306	350	A00406	A01351	477
A00406	A01353	374	A00406	A01425	229
A00406	A01468	303	A00406	A01470	491
A00406	A01487	324	A00406	A01488	470
A00406	A01492	491	A00406	A01495	474
A00406	A01503	333	A00406	A01542	162
A00406	A01598	435	A00406	A01612	477
A00406	A01617	176	A00406	A01649	467
A00406	A01721	460	A00406	A01741	274
A00406	A01864	157	A00406	A02265	179
A00406	A02364	205	A00406	A02456	160
A00406	A02484	176	A00406	A03047	216
A00406	A03296	152	A00406	A03438	345
A00406	A03568	185	A00406	A03569	236
A00406	A03736	227	A00406	A04447	191
A00406	A05295	185	A00406	A05665	173
A00406	A05845	457	A00406	A06188	219
A00406	A06275	468	A00406	A06825	167
A00406	A06861	162	A00406	A06957	229
A00406	A06965	168	A00406	A07223	636
A00406	A07244	274	A00406	A07347	491
A00406	A07524	251	A00406	A07533	428
A00406	A07537	171	A00406	A07570	208

A00406	A07584	479	A00406	A07625	395
A00406	A07700	638	A00406	A07725	358
A00406	A07924	461	A00406	A08024	432
A00406	A08078	184	A00406	A08112	278
A00406	A08137	308	A00406	A08211	240
A00406	A08398	283	A00406	A08452	463
A00406	A08491	199	A00406	A08768	179
A00406	A08890	429	A00406	A08952	198
A00406	A09307	446	A00406	A09664	460
A00406	A09702	424	A00406	A10465	196
A00406	A10616	474	A00406	A10923	423
A00406	A11565	162	A00406	A11741	196
A00406	A11831	311	A00406	A11832	486
A00406	A12097	213	A00406	A12419	293
A00406	A13220	243	A00406	A13989	491
A00406	A14151	174	A00406	A14197	202
A00406	A14449	291	A00406	A14482	176
A00406	A14505	268	A00406	A15499	176
A00406	A15764	404	A00406	A16302	294
A00406	A17168	294	A00407	A00697	221
A00407	A01161	338	A00407	A01445	355
A00407	A01473	480	A00407	A01524	937
A00407	A03195	278	A00407	A03763	340
A00407	A03790	466	A00407	A04015	263
A00407	A05617	184	A00407	A08300	429
A00407	A08926	443	A00407	A13555	419
A00407	A13980	208	A00407	A15292	259
A00407	A15317	210	A00408	A00625	491
A00408	A00703	236	A00408	A00786	486
A00408	A00877	432	A00408	A00951	205
A00408	A01024	373	A00408	A01052	286
A00408	A01068	232	A00408	A01216	435
A00408	A01291	241	A00408	A01297	63
A00408	A01345	273	A00408	A01451	159
A00408	A01452	199	A00408	A01454	419
A00408	A01478	199	A00408	A01484	329
A00408	A01505	199	A00408	A01539	314
A00408	A01553	199	A00408	A01561	199
A00408	A01563	422	A00408	A01568	443
A00408	A01588	171	A00408	A01650	186
A00408	A01660	172	A00408	A01686	443
A00408	A02472	462	A00408	A02671	379
A00408	A02778	254	A00408	A03423	340
A00408	A03545	232	A00408	A04054	162
A00408	A04213	154	A00408	A04674	227
A00408	A04710	467	A00408	A05008	305

A00408	A05009	302	A00408	A05250	190
A00408	A05822	393	A00408	A05823	311
A00408	A06021	174	A00408	A06327	311
A00408	A06743	262	A00408	A06888	391
A00408	A06889	491	A00408	A06994	162
A00408	A07073	283	A00408	A07242	491
A00408	A07404	159	A00408	A07483	157
A00408	A07495	475	A00408	A07816	409
A00408	A08133	63	A00408	A08208	441
A00408	A08400	329	A00408	A08507	152
A00408	A08531	379	A00408	A08571	199
A00408	A08573	323	A00408	A09051	227
A00408	A09668	162	A00408	A10291	177
A00408	A10319	238	A00408	A10773	483
A00408	A11043	311	A00408	A11098	281
A00408	A12047	486	A00408	A12693	435
A00408	A13209	311	A00408	A13358	311
A00408	A13949	235	A00408	A14266	296
A00408	A14281	489	A00408	A14313	218
A00408	A14640	182	A00408	A15023	190
A00408	A15369	187	A00408	A15626	391
A00408	A15654	252	A00408	A15941	171
A00408	A16112	182	A00408	A16127	293
A00409	A00499	291	A00409	A00812	196
A00409	A00883	279	A00409	A01097	270
A00409	A01132	232	A00409	A01133	462
A00409	A01172	283	A00409	A01291	356
A00409	A01457	326	A00409	A01614	221
A00409	A01707	303	A00409	A01781	480
A00409	A03612	152	A00409	A03740	344
A00409	A03858	488	A00409	A03981	212
A00409	A04547	223	A00409	A05449	491
A00409	A07311	488	A00409	A08137	447
A00409	A09857	246	A00409	A10532	341
A00409	A11291	272	A00409	A11297	491
A00409	A13023	162	A00409	A14196	441
A00409	A14220	235	A00409	A14454	290
A00409	A14497	372	A00409	A14591	491
A00409	A14596	491	A00409	A14659	491
A00409	A14738	272	A00409	A14843	369
A00409	A15188	491	A00409	A15254	484
A00409	A15507	491	A00409	A15547	491
A00409	A15611	454	A00409	A15612	216
A00409	A15660	335	A00409	A15684	332
A00409	A15722	389	A00409	A15769	491
A00409	A16006	201	A00409	A16291	151

A00409	A16293	491	A00409	A16298	477
A00409	A16580	205	A00409	A16627	491
A00409	A16696	484	A00409	A16822	491
A00409	A16879	479	A00409	A16947	449
A00409	A17031	299	A00410	A00743	352
A00410	A01342	168	A00410	A01515	177
A00410	A02044	361	A00410	A05473	236
A00410	A07815	168	A00410	A08502	191
A00410	A09084	255	A00410	A09277	182
A00410	A10315	305	A00410	A11470	491
A00410	A13322	390	A00410	A13832	445
A00410	A14193	491	A00410	A14204	483
A00410	A14247	193	A00410	A14253	490
A00410	A14788	417	A00410	A15308	288
A00410	A15557	423	A00410	A15680	251
A00410	A15688	380	A00410	A15690	486
A00410	A15948	165	A00410	A16113	221
A00410	A16381	308	A00410	A16534	458
A00411	A00454	346	A00411	A00467	563
A00411	A00494	582	A00411	A00516	238
A00411	A00521	413	A00411	A00565	484
A00411	A00571	346	A00411	A00605	193
A00411	A00624	213	A00411	A00629	363
A00411	A00643	358	A00411	A00648	473
A00411	A00651	489	A00411	A00653	174
A00411	A00669	561	A00411	A00687	201
A00411	A00700	391	A00411	A00742	245
A00411	A00782	386	A00411	A00787	472
A00411	A00809	411	A00411	A00812	445
A00411	A00839	573	A00411	A00875	513
A00411	A00878	451	A00411	A00922	446
A00411	A00935	172	A00411	A00970	63
A00411	A00980	588	A00411	A00994	306
A00411	A01012	345	A00411	A01038	447
A00411	A01072	163	A00411	A01090	439
A00411	A01102	420	A00411	A01112	385
A00411	A01115	177	A00411	A01144	468
A00411	A01177	230	A00411	A01192	286
A00411	A01196	581	A00411	A01218	241
A00411	A01234	356	A00411	A01247	469
A00411	A01261	299	A00411	A01262	491
A00411	A01263	235	A00411	A01270	582
A00411	A01291	472	A00411	A01294	288
A00411	A01306	335	A00411	A01335	355
A00411	A01354	160	A00411	A01358	238
A00411	A01365	173	A00411	A01369	229

A00411	A01385	259	A00411	A01411	586
A00411	A01425	245	A00411	A01436	173
A00411	A01468	483	A00411	A01496	474
A00411	A01537	452	A00411	A01540	205
A00411	A01569	221	A00411	A01579	588
A00411	A01586	246	A00411	A01601	588
A00411	A01649	180	A00411	A01652	241
A00411	A01689	396	A00411	A01730	423
A00411	A01822	388	A00411	A01917	68
A00411	A02568	270	A00411	A02737	222
A00411	A02786	63	A00411	A02816	63
A00411	A03435	402	A00411	A04342	179
A00411	A05501	547	A00411	A05916	198
A00411	A06348	486	A00411	A06466	491
A00411	A06534	250	A00411	A06564	301
A00411	A06597	259	A00411	A06814	588
A00411	A06973	573	A00411	A07047	241
A00411	A07311	469	A00411	A07398	587
A00411	A07399	213	A00411	A07433	449
A00411	A07593	491	A00411	A07686	207
A00411	A08131	486	A00411	A08196	157
A00411	A08369	527	A00411	A08408	241
A00411	A08476	241	A00411	A08533	340
A00411	A08556	202	A00411	A08557	491
A00411	A08847	491	A00411	A08973	272
A00411	A08991	249	A00411	A09308	586
A00411	A09322	241	A00411	A09506	238
A00411	A09620	241	A00411	A11728	577
A00411	A12208	193	A00411	A12215	269
A00411	A12227	241	A00411	A12401	160
A00411	A13024	207	A00411	A13340	488
A00411	A13992	167	A00411	A14172	328
A00411	A14188	162	A00411	A14216	470
A00411	A14260	241	A00411	A14280	174
A00411	A14289	174	A00411	A14729	216
A00411	A14819	369	A00411	A14843	272
A00411	A15174	243	A00411	A15250	207
A00411	A15268	459	A00411	A15381	576
A00411	A15666	385	A00411	A15711	363
A00411	A15932	475	A00411	A16272	434
A00411	A16351	207	A00411	A16682	236
A00412	A00489	429	A00412	A00640	286
A00412	A00647	154	A00412	A00740	363
A00412	A01069	674	A00412	A01242	404
A00412	A01564	399	A00412	A01569	468
A00412	A01577	361	A00412	A02068	260

A00412	A02493	205	A00412	A03325	441
A00412	A03446	438	A00412	A03476	398
A00412	A03560	641	A00412	A03681	363
A00412	A03783	404	A00412	A03863	643
A00412	A07247	165	A00412	A08546	404
A00412	A08763	191	A00412	A08991	404
A00412	A09024	362	A00412	A13135	335
A00412	A13609	246	A00413	A00636	230
A00413	A00743	357	A00413	A00929	346
A00413	A00941	485	A00413	A01036	491
A00413	A01358	329	A00413	A01390	452
A00413	A01588	263	A00413	A01598	268
A00413	A02010	491	A00413	A03032	319
A00413	A03080	213	A00413	A03817	378
A00413	A06941	452	A00413	A07054	461
A00413	A07242	436	A00413	A09056	485
A00413	A09671	162	A00413	A11408	190
A00413	A12047	469	A00413	A12234	228
A00413	A12264	486	A00413	A14196	180
A00413	A14926	288	A00413	A15323	302
A00413	A15684	227	A00413	A15788	534
A00414	A00590	418	A00414	A00648	479
A00414	A00651	491	A00414	A00700	484
A00414	A00701	333	A00414	A00713	344
A00414	A00787	475	A00414	A00839	490
A00414	A00861	177	A00414	A00870	319
A00414	A00965	424	A00414	A00966	490
A00414	A01012	490	A00414	A01056	383
A00414	A01085	451	A00414	A01090	491
A00414	A01112	451	A00414	A01140	399
A00414	A01144	234	A00414	A01160	307
A00414	A01291	486	A00414	A01306	485
A00414	A01335	405	A00414	A01337	210
A00414	A01365	347	A00414	A01407	200
A00414	A01411	243	A00414	A01436	294
A00414	A01449	185	A00414	A01468	477
A00414	A01496	491	A00414	A01579	486
A00414	A01586	241	A00414	A01601	154
A00414	A01822	436	A00414	A02350	369
A00414	A03048	238	A00414	A03461	360
A00414	A03671	357	A00414	A03981	479
A00414	A04230	213	A00414	A05149	296
A00414	A05191	278	A00414	A05501	432
A00414	A06351	452	A00414	A06814	339
A00414	A07047	327	A00414	A07243	215
A00414	A07262	340	A00414	A07307	259

A00414	A07398	409	A00414	A07726	404
A00414	A08055	416	A00414	A08131	191
A00414	A08359	247	A00414	A08398	163
A00414	A08559	172	A00414	A09285	323
A00414	A11263	299	A00414	A11334	314
A00414	A11550	348	A00414	A12404	330
A00414	A12821	334	A00414	A13989	477
A00414	A14025	182	A00414	A14216	490
A00414	A14377	162	A00414	A15500	326
A00414	A16053	363	A00415	A00418	475
A00415	A00534	491	A00415	A00883	213
A00415	A00885	491	A00415	A00886	489
A00415	A00889	491	A00415	A00890	485
A00415	A00891	446	A00415	A00892	491
A00415	A00893	491	A00415	A00894	491
A00415	A00895	491	A00415	A00896	162
A00415	A00897	490	A00415	A00898	491
A00415	A00899	488	A00415	A01212	236
A00415	A01572	168	A00415	A01602	260
A00415	A02024	489	A00415	A02112	202
A00415	A03151	491	A00415	A05347	317
A00415	A06345	317	A00415	A06673	486
A00415	A06992	188	A00415	A07446	139
A00415	A08125	288	A00415	A08435	473
A00415	A08818	218	A00415	A08891	491
A00415	A09008	245	A00415	A10527	162
A00415	A10983	152	A00415	A11443	361
A00415	A11650	227	A00415	A11710	165
A00415	A13740	179	A00415	A14174	491
A00415	A16046	162	A00415	A17186	201
A00416	A00432	279	A00416	A00546	191
A00416	A00625	491	A00416	A00630	488
A00416	A00631	468	A00416	A00732	156
A00416	A00786	414	A00416	A00877	486
A00416	A00971	428	A00416	A01036	232
A00416	A01151	343	A00416	A01366	330
A00416	A01378	255	A00416	A01454	388
A00416	A01539	339	A00416	A01606	491
A00416	A01687	293	A00416	A01742	188
A00416	A03304	486	A00416	A03336	312
A00416	A03832	358	A00416	A04449	474
A00416	A05056	294	A00416	A05142	216
A00416	A06277	210	A00416	A06752	488
A00416	A07242	485	A00416	A07483	490
A00416	A07495	477	A00416	A07665	430
A00416	A07954	456	A00416	A08379	281

A00416	A08536	428	A00416	A08551	190
A00416	A08961	168	A00416	A09300	328
A00416	A09624	486	A00416	A10043	263
A00416	A10421	488	A00416	A10516	323
A00416	A10607	216	A00416	A11061	198
A00416	A11150	160	A00416	A11199	484
A00416	A11281	455	A00416	A11292	422
A00416	A11293	491	A00416	A11516	294
A00416	A11659	243	A00416	A11983	210
A00416	A12365	193	A00416	A12862	235
A00416	A13866	180	A00416	A14167	162
A00416	A14281	483	A00416	A15736	373
A00416	A16647	216	A00417	A00431	722
A00417	A00629	401	A00417	A00718	259
A00417	A00794	601	A00417	A00821	477
A00417	A00942	722	A00417	A01022	491
A00417	A01026	357	A00417	A01035	602
A00417	A01060	272	A00417	A01113	190
A00417	A01211	288	A00417	A01451	602
A00417	A01452	201	A00417	A01453	427
A00417	A01457	491	A00417	A01458	610
A00417	A01459	269	A00417	A01461	596
A00417	A01500	469	A00417	A01503	616
A00417	A01514	269	A00417	A01515	491
A00417	A01522	488	A00417	A01574	491
A00417	A01575	491	A00417	A01576	561
A00417	A01578	433	A00417	A01583	269
A00417	A01585	595	A00417	A01612	163
A00417	A01634	491	A00417	A01646	475
A00417	A01660	489	A00417	A01683	446
A00417	A01706	602	A00417	A01811	460
A00417	A03293	698	A00417	A03569	339
A00417	A03618	367	A00417	A03626	346
A00417	A03748	159	A00417	A03815	177
A00417	A03889	247	A00417	A03891	401
A00417	A03892	480	A00417	A03910	218
A00417	A04145	722	A00417	A04292	602
A00417	A04337	447	A00417	A04682	561
A00417	A04686	489	A00417	A04715	198
A00417	A04835	486	A00417	A04909	371
A00417	A05024	578	A00417	A05453	228
A00417	A05638	269	A00417	A06011	597
A00417	A06168	490	A00417	A06593	232
A00417	A06941	474	A00417	A07203	508
A00417	A07442	321	A00417	A07499	193
A00417	A07558	270	A00417	A07640	485

A00417	A08056	190	A00417	A08137	163
A00417	A08261	260	A00417	A08293	154
A00417	A08400	485	A00417	A08454	156
A00417	A08461	167	A00417	A08484	721
A00417	A08506	721	A00417	A08544	721
A00417	A09053	246	A00417	A09326	269
A00417	A09396	302	A00417	A09546	466
A00417	A09996	486	A00417	A10584	391
A00417	A10655	314	A00417	A10739	162
A00417	A10804	357	A00417	A11086	191
A00417	A11266	238	A00417	A11279	326
A00417	A11303	383	A00417	A11885	423
A00417	A12883	256	A00417	A13027	391
A00417	A13628	402	A00417	A13831	334
A00417	A13874	216	A00417	A13882	183
A00417	A13895	269	A00417	A14056	413
A00417	A14182	269	A00417	A14247	491
A00417	A14272	491	A00417	A14461	491
A00417	A14661	603	A00417	A14711	269
A00417	A14783	269	A00417	A14877	174
A00417	A14914	279	A00417	A14970	297
A00417	A15432	402	A00417	A15552	183
A00417	A16669	432	A00418	A00471	550
A00418	A00517	249	A00418	A00578	213
A00418	A00622	377	A00418	A00742	234
A00418	A00805	486	A00418	A00814	358
A00418	A00856	388	A00418	A00858	321
A00418	A00883	466	A00418	A00889	229
A00418	A00893	180	A00418	A00903	235
A00418	A00954	340	A00418	A01050	143
A00418	A01150	157	A00418	A01164	465
A00418	A01169	443	A00418	A01195	238
A00418	A01212	701	A00418	A01214	433
A00418	A01329	589	A00418	A01354	491
A00418	A01469	179	A00418	A01503	491
A00418	A01535	167	A00418	A01536	474
A00418	A01610	391	A00418	A01741	227
A00418	A01813	428	A00418	A01978	163
A00418	A02030	251	A00418	A02075	266
A00418	A02318	165	A00418	A02321	223
A00418	A02702	198	A00418	A02773	229
A00418	A02803	221	A00418	A03458	154
A00418	A03784	262	A00418	A03809	443
A00418	A03886	360	A00418	A04001	198
A00418	A04012	235	A00418	A04236	235
A00418	A04379	163	A00418	A04911	159

A00418	A05142	223	A00418	A05162	241
A00418	A05216	402	A00418	A05621	162
A00418	A06381	119	A00418	A06453	283
A00418	A07319	160	A00418	A07670	156
A00418	A08022	462	A00418	A08434	223
A00418	A09272	174	A00418	A09597	340
A00418	A09654	93	A00418	A09708	371
A00418	A11369	168	A00418	A11859	185
A00418	A13824	176	A00418	A14006	463
A00418	A14128	698	A00418	A14655	267
A00418	A15297	411	A00418	A15324	375
A00418	A15498	182	A00418	A15559	159
A00418	A16491	193	A00419	A00454	216
A00419	A00653	368	A00419	A00697	79
A00419	A00910	314	A00419	A01042	438
A00419	A01113	344	A00419	A01192	215
A00419	A01562	165	A00419	A01709	245
A00419	A02629	259	A00419	A03740	185
A00419	A07178	260	A00419	A08054	201
A00419	A10504	338	A00419	A10506	157
A00419	A12710	436	A00419	A14364	204
A00419	A15699	401	A00420	A00438	491
A00420	A00446	187	A00420	A00467	229
A00420	A00514	199	A00420	A00658	243
A00420	A00849	195	A00420	A00888	160
A00420	A00915	446	A00420	A00918	191
A00420	A01076	297	A00420	A01137	266
A00420	A01161	313	A00420	A01163	358
A00420	A01164	299	A00420	A01169	341
A00420	A01253	468	A00420	A01289	247
A00420	A01339	232	A00420	A01485	316
A00420	A01576	154	A00420	A01582	491
A00420	A01668	344	A00420	A01950	218
A00420	A01984	277	A00420	A02189	221
A00420	A02192	443	A00420	A02196	366
A00420	A02301	165	A00420	A02304	279
A00420	A02306	400	A00420	A02317	218
A00420	A02326	218	A00420	A02467	160
A00420	A02477	193	A00420	A02586	205
A00420	A02758	191	A00420	A02759	294
A00420	A02889	246	A00420	A03076	373
A00420	A03099	462	A00420	A03110	174
A00420	A03115	224	A00420	A03165	228
A00420	A03310	241	A00420	A03458	218
A00420	A03570	230	A00420	A03820	345
A00420	A03885	489	A00420	A04002	286

A00420	A04162	257	A00420	A04256	291
A00420	A04647	230	A00420	A04915	330
A00420	A04925	330	A00420	A04938	228
A00420	A05020	174	A00420	A05450	182
A00420	A05570	318	A00420	A05620	270
A00420	A05768	201	A00420	A05914	263
A00420	A06032	188	A00420	A06033	330
A00420	A06091	179	A00420	A06147	297
A00420	A06596	224	A00420	A06622	152
A00420	A06623	218	A00420	A06727	218
A00420	A07244	338	A00420	A07440	241
A00420	A07647	277	A00420	A07833	358
A00420	A08001	281	A00420	A08141	366
A00420	A08191	281	A00420	A08289	154
A00420	A08308	319	A00420	A08927	350
A00420	A09531	281	A00420	A10597	255
A00420	A11352	319	A00420	A13432	165
A00420	A14642	372	A00420	A15277	152
A00420	A15295	419	A00420	A15672	254
A00421	A00438	713	A00421	A00446	316
A00421	A00448	272	A00421	A00452	474
A00421	A00454	168	A00421	A00489	388
A00421	A00503	240	A00421	A00517	446
A00421	A00523	369	A00421	A00531	759
A00421	A00532	491	A00421	A00537	491
A00421	A00559	479	A00421	A00570	477
A00421	A00571	321	A00421	A00572	152
A00421	A00576	423	A00421	A00583	156
A00421	A00588	491	A00421	A00595	180
A00421	A00605	466	A00421	A00629	401
A00421	A00646	488	A00421	A00653	484
A00421	A00655	468	A00421	A00677	475
A00421	A00687	467	A00421	A00691	542
A00421	A00693	447	A00421	A00697	486
A00421	A00707	463	A00421	A00709	256
A00421	A00716	542	A00421	A00725	491
A00421	A00733	360	A00421	A00735	160
A00421	A00741	479	A00421	A00747	491
A00421	A00761	285	A00421	A00767	491
A00421	A00775	345	A00421	A00802	252
A00421	A00805	489	A00421	A00817	396
A00421	A00821	185	A00421	A00822	488
A00421	A00829	446	A00421	A00831	423
A00421	A00839	195	A00421	A00849	288
A00421	A00865	433	A00421	A00867	201
A00421	A00868	406	A00421	A00871	404

A00421	A00878	529	A00421	A00879	539
A00421	A00883	491	A00421	A00905	542
A00421	A00908	190	A00421	A00916	491
A00421	A00928	491	A00421	A00936	218
A00421	A00942	229	A00421	A00950	480
A00421	A00967	243	A00421	A00975	162
A00421	A00985	313	A00421	A00992	491
A00421	A00993	414	A00421	A01020	427
A00421	A01024	462	A00421	A01027	391
A00421	A01029	541	A00421	A01050	355
A00421	A01053	174	A00421	A01057	389
A00421	A01067	160	A00421	A01075	489
A00421	A01091	372	A00421	A01093	486
A00421	A01099	489	A00421	A01100	412
A00421	A01135	154	A00421	A01136	542
A00421	A01139	491	A00421	A01150	430
A00421	A01151	541	A00421	A01156	676
A00421	A01161	491	A00421	A01166	477
A00421	A01169	366	A00421	A01173	193
A00421	A01187	396	A00421	A01189	201
A00421	A01193	249	A00421	A01206	401
A00421	A01210	469	A00421	A01212	307
A00421	A01214	486	A00421	A01215	184
A00421	A01231	483	A00421	A01234	618
A00421	A01239	491	A00421	A01243	475
A00421	A01251	281	A00421	A01255	195
A00421	A01266	542	A00421	A01268	542
A00421	A01271	198	A00421	A01278	165
A00421	A01279	491	A00421	A01287	168
A00421	A01291	433	A00421	A01295	156
A00421	A01307	384	A00421	A01329	217
A00421	A01334	516	A00421	A01351	491
A00421	A01352	481	A00421	A01354	241
A00421	A01359	491	A00421	A01369	229
A00421	A01372	383	A00421	A01384	177
A00421	A01385	491	A00421	A01391	336
A00421	A01406	486	A00421	A01424	271
A00421	A01434	156	A00421	A01442	380
A00421	A01451	380	A00421	A01459	457
A00421	A01468	430	A00421	A01470	491
A00421	A01471	316	A00421	A01473	157
A00421	A01474	165	A00421	A01487	151
A00421	A01488	296	A00421	A01492	491
A00421	A01540	337	A00421	A01558	490
A00421	A01569	168	A00421	A01582	454
A00421	A01588	472	A00421	A01598	486

A00421	A01610	288	A00421	A01612	585
A00421	A01614	240	A00421	A01615	491
A00421	A01627	504	A00421	A01628	446
A00421	A01647	473	A00421	A01648	151
A00421	A01649	480	A00421	A01660	473
A00421	A01846	433	A00421	A01847	475
A00421	A01848	154	A00421	A01900	188
A00421	A01941	488	A00421	A01988	345
A00421	A02086	291	A00421	A02212	255
A00421	A02535	281	A00421	A02715	246
A00421	A02802	316	A00421	A02847	283
A00421	A03019	187	A00421	A03298	491
A00421	A03438	491	A00421	A03494	695
A00421	A03567	380	A00421	A03569	491
A00421	A03628	152	A00421	A03629	162
A00421	A03676	268	A00421	A03742	255
A00421	A03902	488	A00421	A04068	170
A00421	A04156	267	A00421	A04337	328
A00421	A04345	340	A00421	A04449	363
A00421	A04664	170	A00421	A04667	185
A00421	A04668	486	A00421	A05015	395
A00421	A05356	314	A00421	A06084	195
A00421	A06204	489	A00421	A06267	252
A00421	A06347	488	A00421	A06631	345
A00421	A06698	441	A00421	A06713	212
A00421	A06819	319	A00421	A06825	352
A00421	A06937	364	A00421	A07106	154
A00421	A07303	212	A00421	A07414	173
A00421	A07432	400	A00421	A07442	173
A00421	A07480	351	A00421	A07706	483
A00421	A07826	129	A00421	A07834	318
A00421	A07898	202	A00421	A08132	138
A00421	A08137	491	A00421	A08183	491
A00421	A08201	540	A00421	A08208	210
A00421	A08212	486	A00421	A08327	453
A00421	A08431	508	A00421	A08447	180
A00421	A08516	180	A00421	A08517	269
A00421	A08524	383	A00421	A08542	156
A00421	A08571	491	A00421	A08745	424
A00421	A08756	425	A00421	A08818	218
A00421	A08885	479	A00421	A08915	174
A00421	A08920	174	A00421	A08928	307
A00421	A08963	435	A00421	A08992	267
A00421	A09256	195	A00421	A09258	483
A00421	A09377	364	A00421	A09427	477
A00421	A09451	491	A00421	A09515	491

A00421	A09529	99	A00421	A09538	491
A00421	A09614	174	A00421	A09686	99
A00421	A09731	152	A00421	A09732	232
A00421	A09733	187	A00421	A09759	152
A00421	A09762	188	A00421	A09992	301
A00421	A10125	152	A00421	A10205	160
A00421	A10228	488	A00421	A10250	491
A00421	A10340	294	A00421	A10342	204
A00421	A10373	283	A00421	A10492	138
A00421	A10553	152	A00421	A10624	369
A00421	A10853	491	A00421	A10956	212
A00421	A10957	152	A00421	A11052	174
A00421	A11099	299	A00421	A11105	337
A00421	A11139	694	A00421	A11258	306
A00421	A11277	474	A00421	A11487	328
A00421	A11492	267	A00421	A11496	156
A00421	A11497	156	A00421	A11869	389
A00421	A11965	154	A00421	A12017	152
A00421	A12082	406	A00421	A12294	456
A00421	A12369	152	A00421	A12416	170
A00421	A12757	479	A00421	A13048	152
A00421	A13067	174	A00421	A13068	307
A00421	A13164	285	A00421	A13310	366
A00421	A13311	180	A00421	A13369	428
A00421	A13510	339	A00421	A13635	369
A00421	A13805	438	A00421	A13928	156
A00421	A13978	152	A00421	A13989	491
A00421	A14006	491	A00421	A14009	201
A00421	A14077	201	A00421	A14080	419
A00421	A14148	218	A00421	A14178	351
A00421	A14186	234	A00421	A14196	291
A00421	A14246	263	A00421	A14255	262
A00421	A14257	193	A00421	A14272	358
A00421	A14304	73	A00421	A14476	489
A00421	A14597	335	A00421	A14614	251
A00421	A14655	491	A00421	A14701	489
A00421	A14728	177	A00421	A14986	259
A00421	A15019	118	A00421	A15143	267
A00421	A15292	274	A00421	A15297	491
A00421	A15322	322	A00421	A15324	348
A00421	A15338	491	A00421	A15353	162
A00421	A15356	170	A00421	A15370	460
A00421	A15374	416	A00421	A15524	283
A00421	A15531	401	A00421	A15539	246
A00421	A15543	199	A00421	A15550	219
A00421	A15559	465	A00421	A15564	271

A00421	A15624	198	A00421	A15660	371
A00421	A15711	438	A00421	A15724	223
A00421	A15932	154	A00421	A15935	395
A00421	A16491	269	A00421	A16568	384
A00421	A16685	196	A00421	A16690	383
A00421	A16699	156	A00421	A16866	383
A00421	A16912	468	A00421	A17063	374
A00421	A17129	267	A00422	A01248	265
A00422	A01269	195	A00422	A01469	171
A00422	A01475	162	A00422	A01622	246
A00422	A01625	151	A00422	A01666	221
A00422	A01930	288	A00422	A02185	241
A00422	A02389	174	A00422	A02621	193
A00422	A02778	162	A00422	A02797	319
A00422	A02868	235	A00422	A03114	291
A00422	A03119	382	A00422	A03149	202
A00422	A03197	184	A00422	A03232	258
A00422	A03300	185	A00422	A03654	188
A00422	A03759	163	A00422	A03820	385
A00422	A03977	269	A00422	A04174	180
A00422	A04376	333	A00422	A04678	296
A00422	A04967	385	A00422	A05370	348
A00422	A05874	385	A00422	A05891	162
A00422	A06129	223	A00422	A06234	322
A00422	A06595	157	A00422	A06622	193
A00422	A06648	160	A00422	A06864	240
A00422	A06974	240	A00422	A07641	246
A00422	A07694	385	A00422	A08000	230
A00422	A08105	347	A00422	A08208	274
A00422	A08408	180	A00422	A09127	249
A00422	A09225	347	A00422	A09478	347
A00422	A09838	313	A00422	A10038	267
A00422	A10223	356	A00422	A11728	227
A00422	A12464	347	A00422	A12633	306
A00422	A13098	333	A00422	A13665	208
A00422	A14485	329	A00422	A14585	183
A00422	A14643	432	A00422	A15928	281
A00422	A15939	374	A00422	A15941	301
A00422	A15942	263	A00422	A16129	268
A00422	A16226	419	A00422	A16613	204
A00422	A16616	188	A00423	A00469	491
A00423	A00571	481	A00423	A00590	383
A00423	A00593	362	A00423	A00603	196
A00423	A00651	243	A00423	A00705	165
A00423	A00758	162	A00423	A00782	485
A00423	A00787	399	A00423	A00809	196

A00423	A00839	491	A00423	A00955	406
A00423	A01017	456	A00423	A01034	452
A00423	A01037	299	A00423	A01095	201
A00423	A01152	340	A00423	A01154	400
A00423	A01247	491	A00423	A01263	491
A00423	A01270	179	A00423	A01291	412
A00423	A01335	491	A00423	A01337	446
A00423	A01365	468	A00423	A01369	474
A00423	A01406	241	A00423	A01416	185
A00423	A01433	313	A00423	A01436	210
A00423	A01449	488	A00423	A01496	243
A00423	A01579	484	A00423	A03493	257
A00423	A05247	180	A00423	A05499	277
A00423	A05501	486	A00423	A05502	157
A00423	A07412	177	A00423	A07464	247
A00423	A07965	369	A00423	A08011	404
A00423	A08358	475	A00423	A08359	391
A00423	A08457	268	A00423	A08462	263
A00423	A08501	490	A00423	A08543	491
A00423	A08556	491	A00423	A08760	195
A00423	A08761	391	A00423	A08773	445
A00423	A08973	269	A00423	A09613	408
A00423	A09622	388	A00423	A09735	357
A00423	A09999	213	A00423	A11440	105
A00423	A11597	446	A00423	A11616	279
A00423	A11728	390	A00423	A11734	307
A00423	A11857	407	A00423	A11992	346
A00423	A12011	208	A00423	A12052	299
A00423	A12821	413	A00423	A13231	230
A00423	A13356	226	A00423	A13710	311
A00423	A14167	217	A00423	A14482	219
A00423	A14819	198	A00423	A15225	303
A00423	A15499	201	A00423	A15500	489
A00423	A15743	187	A00423	A15771	388
A00423	A16682	205	A00423	A16818	285
A00423	A17133	191	A00424	A00435	217
A00424	A00447	217	A00424	A00452	544
A00424	A00515	193	A00424	A00555	217
A00424	A00570	353	A00424	A00629	246
A00424	A00638	154	A00424	A00687	217
A00424	A00721	217	A00424	A00772	217
A00424	A00854	302	A00424	A00867	249
A00424	A00928	565	A00424	A00952	217
A00424	A00972	217	A00424	A00997	217
A00424	A01107	451	A00424	A01159	217
A00424	A01221	217	A00424	A01243	236

A00424	A01255	400	A00424	A01418	217
A00424	A02212	196	A00424	A03556	217
A00424	A03617	217	A00424	A04666	174
A00424	A04881	276	A00424	A05157	285
A00424	A05605	217	A00424	A06369	217
A00424	A06832	217	A00424	A07756	185
A00424	A08183	217	A00424	A08467	247
A00424	A08630	269	A00424	A09673	217
A00424	A14088	217	A00424	A15323	236
A00424	A15324	358	A00424	A15353	429
A00424	A15377	451	A00424	A15419	185
A00424	A15543	570	A00424	A15564	297
A00424	A15724	217	A00424	A16664	217
A00424	A17063	160	A00425	A00443	360
A00425	A00448	491	A00425	A00458	401
A00425	A00463	486	A00425	A00481	185
A00425	A00497	489	A00425	A00532	682
A00425	A00535	491	A00425	A00551	475
A00425	A00566	491	A00425	A00575	491
A00425	A00632	154	A00425	A00691	486
A00425	A00703	176	A00425	A00708	491
A00425	A00760	491	A00425	A00805	263
A00425	A00856	470	A00425	A00858	491
A00425	A00881	489	A00425	A00903	491
A00425	A00916	344	A00425	A00919	491
A00425	A00954	491	A00425	A00964	491
A00425	A01020	489	A00425	A01075	491
A00425	A01079	490	A00425	A01082	491
A00425	A01084	491	A00425	A01087	491
A00425	A01088	489	A00425	A01234	185
A00425	A01295	254	A00425	A01308	229
A00425	A01309	372	A00425	A01349	274
A00425	A01434	202	A00425	A01500	185
A00425	A01503	481	A00425	A01555	191
A00425	A01576	481	A00425	A01741	375
A00425	A02064	263	A00425	A02390	429
A00425	A03496	162	A00425	A03944	443
A00425	A04033	176	A00425	A05288	177
A00425	A06660	221	A00425	A06881	230
A00425	A07700	432	A00425	A07706	491
A00425	A08022	488	A00425	A08118	361
A00425	A08190	193	A00425	A08356	380
A00425	A08362	428	A00425	A08377	329
A00425	A08382	456	A00425	A08439	420
A00425	A08450	463	A00425	A08518	491
A00425	A08527	491	A00425	A08543	430

A00425	A08550	430	A00425	A08552	408
A00425	A08553	491	A00425	A08560	202
A00425	A08561	409	A00425	A08773	286
A00425	A08814	157	A00425	A08976	491
A00425	A08996	491	A00425	A09010	491
A00425	A09609	160	A00425	A09619	393
A00425	A10691	475	A00425	A10789	160
A00425	A10875	246	A00425	A11234	293
A00425	A11301	446	A00425	A11395	491
A00425	A11472	168	A00425	A11496	435
A00425	A11497	443	A00425	A11674	490
A00425	A11914	327	A00425	A11917	313
A00425	A12254	480	A00425	A12433	443
A00425	A12507	218	A00425	A12606	446
A00425	A12658	317	A00425	A12659	165
A00425	A12665	207	A00425	A12725	172
A00425	A12858	438	A00425	A12873	191
A00425	A13309	256	A00425	A13313	319
A00425	A13399	389	A00425	A13708	329
A00425	A13737	350	A00425	A13781	424
A00425	A13784	337	A00425	A13805	223
A00425	A13912	176	A00425	A13989	463
A00425	A14084	378	A00425	A14212	260
A00425	A14214	441	A00425	A14222	218
A00425	A14263	154	A00425	A14358	205
A00425	A14462	373	A00425	A14566	232
A00425	A14569	446	A00425	A14655	455
A00425	A14708	335	A00425	A14921	191
A00425	A15105	391	A00425	A15138	216
A00425	A15189	283	A00425	A15266	154
A00425	A15373	205	A00425	A15520	491
A00425	A15666	165	A00425	A15683	171
A00425	A15733	491	A00425	A15762	152
A00425	A15774	201	A00425	A15945	491
A00425	A15946	491	A00425	A16067	362
A00425	A16109	462	A00425	A16144	429
A00425	A16157	435	A00425	A16212	490
A00425	A16261	163	A00425	A16394	468
A00425	A16552	313	A00425	A16565	216
A00425	A16597	475	A00425	A16611	417
A00425	A16664	405	A00425	A16675	369
A00425	A16680	191	A00425	A16690	313
A00425	A16695	185	A00425	A16699	196
A00425	A16703	187	A00425	A16704	199
A00425	A16707	235	A00425	A16709	221
A00425	A16764	307	A00425	A16774	157

A00425	A16790	221	A00425	A16815	176
A00425	A16824	167	A00425	A16838	420
A00425	A16874	235	A00425	A16944	266
A00425	A17014	157	A00425	A17015	179
A00425	A17043	180	A00426	A00441	157
A00426	A00532	193	A00426	A00565	491
A00426	A00570	406	A00426	A00576	205
A00426	A00638	483	A00426	A00862	474
A00426	A00977	363	A00426	A01027	293
A00426	A01156	406	A00426	A01209	491
A00426	A01243	193	A00426	A01244	491
A00426	A01385	398	A00426	A01393	207
A00426	A02001	187	A00426	A02180	491
A00426	A03327	243	A00426	A03328	486
A00426	A03387	183	A00426	A03764	343
A00426	A04174	307	A00426	A04187	449
A00426	A04396	279	A00426	A04561	246
A00426	A04647	179	A00426	A04663	196
A00426	A05359	185	A00426	A05690	491
A00426	A06423	224	A00426	A06602	191
A00426	A06671	486	A00426	A06982	210
A00426	A07449	154	A00426	A07572	265
A00426	A08450	154	A00426	A08518	191
A00426	A08700	167	A00426	A08884	491
A00426	A09046	174	A00426	A09067	456
A00426	A09269	491	A00426	A09316	488
A00426	A09340	491	A00426	A09423	351
A00426	A10258	240	A00426	A10289	219
A00426	A10808	899	A00426	A11858	491
A00426	A13519	335	A00426	A13912	224
A00426	A14214	229	A00426	A14230	160
A00426	A14272	167	A00426	A14825	205
A00426	A14921	240	A00426	A15288	163
A00426	A15932	443	A00426	A15940	157
A00426	A16055	179	A00426	A16215	174
A00426	A16404	196	A00426	A16410	170
A00426	A16440	490	A00426	A16774	205
A00426	A16815	224	A00427	A00521	335
A00427	A00590	583	A00427	A00603	658
A00427	A00648	628	A00427	A00651	361
A00427	A00763	372	A00427	A00782	202
A00427	A00787	654	A00427	A00827	162
A00427	A00839	290	A00427	A00870	251
A00427	A00878	629	A00427	A00902	157
A00427	A00906	411	A00427	A00923	380
A00427	A00949	474	A00427	A00955	491

A00427	A00965	664	A00427	A00966	317
A00427	A01025	664	A00427	A01037	328
A00427	A01067	383	A00427	A01090	484
A00427	A01154	378	A00427	A01238	559
A00427	A01249	389	A00427	A01263	324
A00427	A01291	472	A00427	A01292	384
A00427	A01306	319	A00427	A01335	478
A00427	A01337	160	A00427	A01392	664
A00427	A01416	626	A00427	A01425	317
A00427	A01433	521	A00427	A01436	658
A00427	A01468	317	A00427	A01586	329
A00427	A01718	383	A00427	A01741	219
A00427	A02895	157	A00427	A03490	317
A00427	A03981	317	A00427	A05411	372
A00427	A05450	185	A00427	A05499	475
A00427	A05735	259	A00427	A06308	254
A00427	A06351	338	A00427	A06388	386
A00427	A06425	259	A00427	A06775	226
A00427	A07248	204	A00427	A07412	329
A00427	A07658	266	A00427	A08011	535
A00427	A08055	372	A00427	A08154	439
A00427	A08377	317	A00427	A08550	372
A00427	A08773	553	A00427	A08973	335
A00427	A09293	238	A00427	A09735	201
A00427	A09852	157	A00427	A09978	210
A00427	A10014	165	A00427	A11453	218
A00427	A13249	372	A00427	A14482	490
A00427	A14568	232	A00427	A15218	234
A00427	A15745	323	A00427	A15988	604
A00427	A17152	246	A00428	A00871	165
A00428	A00956	271	A00428	A01326	260
A00428	A01363	407	A00428	A03570	274
A00428	A08927	355	A00428	A14474	157
A00428	A16398	383	A00429	A00522	423
A00429	A00532	413	A00429	A00546	485
A00429	A00625	483	A00429	A00719	491
A00429	A00786	486	A00429	A00853	480
A00429	A00864	474	A00429	A00877	310
A00429	A00951	171	A00429	A00971	368
A00429	A01036	936	A00429	A01064	475
A00429	A01114	473	A00429	A01454	485
A00429	A01514	201	A00429	A01568	491
A00429	A01595	241	A00429	A01630	167
A00429	A01647	308	A00429	A01687	491
A00429	A01717	256	A00429	A02010	218
A00429	A02219	321	A00429	A02352	205

A00429	A02573	424	A00429	A02635	152
A00429	A02715	210	A00429	A03401	491
A00429	A03496	483	A00429	A03880	485
A00429	A04307	184	A00429	A04845	800
A00429	A04971	154	A00429	A05301	488
A00429	A05967	490	A00429	A06176	185
A00429	A06223	323	A00429	A06548	285
A00429	A06591	491	A00429	A06661	611
A00429	A06715	385	A00429	A07054	491
A00429	A07073	324	A00429	A07083	156
A00429	A07242	491	A00429	A07444	378
A00429	A07495	491	A00429	A07816	380
A00429	A07872	234	A00429	A07873	385
A00429	A07893	276	A00429	A07897	174
A00429	A07934	390	A00429	A07937	355
A00429	A07942	420	A00429	A07953	329
A00429	A07954	491	A00429	A08126	262
A00429	A08192	385	A00429	A08418	270
A00429	A08456	401	A00429	A08531	458
A00429	A08551	274	A00429	A08563	191
A00429	A08910	491	A00429	A09324	317
A00429	A09624	469	A00429	A09668	322
A00429	A09757	686	A00429	A09771	162
A00429	A10651	474	A00429	A10909	272
A00429	A11121	216	A00429	A11196	180
A00429	A11283	332	A00429	A11346	490
A00429	A11385	491	A00429	A11394	483
A00429	A11408	272	A00429	A11473	152
A00429	A11661	285	A00429	A11881	402
A00429	A11902	201	A00429	A11998	285
A00429	A12004	238	A00429	A12047	486
A00429	A12129	350	A00429	A12234	659
A00429	A12537	329	A00429	A12538	932
A00429	A12598	272	A00429	A12654	188
A00429	A12678	935	A00429	A12722	489
A00429	A12834	970	A00429	A13323	212
A00429	A13681	272	A00429	A13953	243
A00429	A14266	491	A00429	A14278	384
A00429	A14279	434	A00429	A14281	485
A00429	A14474	353	A00429	A14484	207
A00429	A14633	490	A00429	A15217	291
A00429	A15273	486	A00429	A15366	474
A00429	A15485	152	A00429	A15685	262
A00429	A15768	246	A00429	A16851	177
A00430	A00444	445	A00430	A00848	319
A00430	A00970	474	A00430	A01410	174

A00430	A01917	200	A00430	A02153	176
A00430	A02352	193	A00430	A02877	462
A00430	A03102	258	A00430	A03294	171
A00430	A03582	269	A00430	A03738	489
A00430	A03747	469	A00430	A03790	234
A00430	A04191	491	A00430	A04192	456
A00430	A04614	218	A00430	A05995	456
A00430	A06289	230	A00430	A07163	427
A00430	A07245	306	A00430	A07421	165
A00430	A08093	305	A00430	A09248	246
A00430	A09271	341	A00430	A10532	491
A00430	A10588	461	A00430	A10589	430
A00430	A10830	369	A00430	A10907	358
A00430	A11240	454	A00430	A11299	168
A00430	A12839	475	A00430	A12996	435
A00430	A13012	152	A00430	A13674	358
A00430	A13870	218	A00430	A14102	335
A00430	A14130	413	A00430	A14131	302
A00430	A14132	489	A00430	A15696	254
A00430	A15698	212	A00430	A15913	162
A00430	A17085	347	A00431	A00492	477
A00431	A00553	321	A00431	A00625	279
A00431	A00786	307	A00431	A00794	402
A00431	A00942	867	A00431	A01035	269
A00431	A01060	269	A00431	A01066	193
A00431	A01252	491	A00431	A01302	378
A00431	A01451	269	A00431	A01458	268
A00431	A01459	269	A00431	A01461	596
A00431	A01514	269	A00431	A01515	484
A00431	A01574	216	A00431	A01575	573
A00431	A01583	269	A00431	A01585	592
A00431	A01634	488	A00431	A01706	625
A00431	A03618	162	A00431	A04145	722
A00431	A04292	603	A00431	A05024	555
A00431	A05638	269	A00431	A06011	596
A00431	A06597	319	A00431	A06890	210
A00431	A07483	419	A00431	A07736	157
A00431	A08056	443	A00431	A08369	571
A00431	A08454	173	A00431	A08461	277
A00431	A08484	721	A00431	A08506	722
A00431	A08544	721	A00431	A08569	419
A00431	A09298	467	A00431	A09326	269
A00431	A11279	603	A00431	A11917	430
A00431	A12011	174	A00431	A12834	236
A00431	A12858	256	A00431	A13708	288
A00431	A13786	240	A00431	A13895	269

A00431	A14167	184	A00431	A14182	269
A00431	A14247	571	A00431	A14272	174
A00431	A14661	603	A00431	A14711	602
A00431	A14783	269	A00431	A14844	477
A00431	A14877	324	A00431	A15347	246
A00431	A15552	200	A00431	A15856	171
A00431	A16669	271	A00431	A16900	188
A00431	A17133	157	A00432	A00503	226
A00432	A00963	294	A00432	A01539	441
A00432	A02477	205	A00432	A02709	180
A00432	A02822	329	A00432	A05556	350
A00432	A05597	310	A00432	A05601	219
A00432	A08097	447	A00432	A08212	156
A00432	A09246	279	A00432	A09341	199
A00432	A11293	238	A00432	A14372	363
A00432	A14648	154	A00433	A00444	154
A00433	A00626	491	A00433	A00654	238
A00433	A00706	334	A00433	A00783	338
A00433	A00833	477	A00433	A00841	198
A00433	A00864	177	A00433	A00875	252
A00433	A00932	319	A00433	A00970	299
A00433	A00973	633	A00433	A01042	330
A00433	A01072	190	A00433	A01098	238
A00433	A01113	638	A00433	A01150	433
A00433	A01192	638	A00433	A01249	251
A00433	A01291	314	A00433	A01425	207
A00433	A01431	218	A00433	A01478	199
A00433	A01496	340	A00433	A01540	205
A00433	A01561	199	A00433	A01569	454
A00433	A01572	199	A00433	A01596	199
A00433	A01598	475	A00433	A01612	199
A00433	A01648	334	A00433	A01660	199
A00433	A01822	168	A00433	A01871	199
A00433	A02110	366	A00433	A02472	395
A00433	A03560	334	A00433	A03676	199
A00433	A03740	385	A00433	A03752	480
A00433	A04446	477	A00433	A05029	346
A00433	A06316	255	A00433	A06466	477
A00433	A06936	255	A00433	A07311	958
A00433	A07378	394	A00433	A07706	152
A00433	A07798	352	A00433	A07959	340
A00433	A08133	199	A00433	A08207	199
A00433	A08261	486	A00433	A08571	199
A00433	A08689	340	A00433	A08991	137
A00433	A09471	162	A00433	A09586	154
A00433	A09591	636	A00433	A09653	163

A00433	A09691	240	A00433	A10490	168
A00433	A12059	255	A00433	A12284	471
A00433	A12473	290	A00433	A12503	477
A00433	A12813	480	A00433	A13340	224
A00433	A13522	196	A00433	A13989	293
A00433	A14178	502	A00433	A14265	366
A00433	A14299	199	A00433	A14443	199
A00433	A14758	199	A00433	A15146	255
A00433	A15244	180	A00433	A16073	199
A00433	A17088	334	A00434	A00600	152
A00434	A01158	390	A00434	A01971	317
A00434	A04351	355	A00434	A06347	188
A00434	A09560	399	A00435	A00447	424
A00435	A00452	217	A00435	A00518	185
A00435	A00555	229	A00435	A00563	391
A00435	A00566	408	A00435	A00570	511
A00435	A00571	358	A00435	A00589	341
A00435	A00594	224	A00435	A00605	475
A00435	A00629	265	A00435	A00655	199
A00435	A00683	235	A00435	A00687	560
A00435	A00689	330	A00435	A00721	502
A00435	A00772	217	A00435	A00822	257
A00435	A00831	383	A00435	A00839	224
A00435	A00843	401	A00435	A00848	277
A00435	A00883	171	A00435	A00928	539
A00435	A00952	323	A00435	A00970	402
A00435	A00972	257	A00435	A00997	217
A00435	A01065	491	A00435	A01107	217
A00435	A01112	213	A00435	A01120	371
A00435	A01159	568	A00435	A01166	408
A00435	A01179	430	A00435	A01201	249
A00435	A01206	213	A00435	A01214	553
A00435	A01215	618	A00435	A01221	504
A00435	A01234	435	A00435	A01243	553
A00435	A01291	188	A00435	A01352	340
A00435	A01356	460	A00435	A01368	262
A00435	A01384	480	A00435	A01412	601
A00435	A01418	564	A00435	A01440	452
A00435	A01468	294	A00435	A01470	480
A00435	A01492	221	A00435	A01649	483
A00435	A01976	151	A00435	A03101	221
A00435	A03438	188	A00435	A03494	162
A00435	A03556	217	A00435	A03567	436
A00435	A03617	217	A00435	A05605	217
A00435	A06017	258	A00435	A06122	208
A00435	A06279	436	A00435	A06369	297

A00435	A06564	154	A00435	A06584	152
A00435	A06832	369	A00435	A07487	296
A00435	A08118	159	A00435	A08183	328
A00435	A08357	489	A00435	A09292	434
A00435	A09327	334	A00435	A09673	217
A00435	A09707	486	A00435	A10156	170
A00435	A11462	367	A00435	A13831	378
A00435	A14088	217	A00435	A14098	303
A00435	A14485	202	A00435	A14701	213
A00435	A15377	472	A00435	A15543	217
A00435	A15724	217	A00435	A16401	260
A00435	A16664	217	A00436	A00517	490
A00436	A00531	480	A00436	A00733	418
A00436	A00781	428	A00436	A00805	489
A00436	A00822	484	A00436	A00835	230
A00436	A00866	486	A00436	A00905	159
A00436	A00916	461	A00436	A01057	491
A00436	A01075	475	A00436	A01077	491
A00436	A01158	224	A00436	A01189	353
A00436	A01354	485	A00436	A01406	188
A00436	A01781	358	A00436	A01956	177
A00436	A02007	241	A00436	A02044	162
A00436	A02225	190	A00436	A02518	174
A00436	A02671	227	A00436	A02715	457
A00436	A03025	273	A00436	A03080	465
A00436	A03495	433	A00436	A04181	260
A00436	A04329	219	A00436	A04477	255
A00436	A05284	154	A00436	A06149	188
A00436	A06591	489	A00436	A06983	363
A00436	A07201	229	A00436	A07290	205
A00436	A07311	162	A00436	A07428	341
A00436	A08137	447	A00436	A08531	481
A00436	A08551	473	A00436	A08911	163
A00436	A09421	480	A00436	A09757	491
A00436	A09790	217	A00436	A09890	246
A00436	A09943	246	A00436	A10006	218
A00436	A11132	347	A00436	A13851	281
A00436	A14006	475	A00436	A14399	469
A00436	A14661	157	A00436	A15545	434
A00436	A16371	165	A00437	A00636	491
A00437	A00722	96	A00437	A00797	191
A00437	A00815	469	A00437	A00883	205
A00437	A00971	174	A00437	A01004	465
A00437	A01032	396	A00437	A01091	159
A00437	A01161	319	A00437	A01235	205
A00437	A01275	310	A00437	A01342	490

A00437	A01417	491	A00437	A01478	174
A00437	A01552	293	A00437	A01578	463
A00437	A01661	474	A00437	A01671	456
A00437	A01956	134	A00437	A01961	273
A00437	A02010	360	A00437	A02157	481
A00437	A02278	481	A00437	A02931	420
A00437	A02951	208	A00437	A02961	154
A00437	A03080	274	A00437	A03223	451
A00437	A03556	204	A00437	A03597	290
A00437	A03625	314	A00437	A03712	485
A00437	A03722	297	A00437	A04037	232
A00437	A04184	324	A00437	A04329	308
A00437	A04615	165	A00437	A05157	151
A00437	A05210	172	A00437	A05539	480
A00437	A06022	294	A00437	A06832	152
A00437	A06886	441	A00437	A06907	430
A00437	A07180	334	A00437	A07209	418
A00437	A07294	441	A00437	A07655	159
A00437	A08111	337	A00437	A08148	345
A00437	A08268	409	A00437	A08296	491
A00437	A08529	337	A00437	A08701	156
A00437	A09202	319	A00437	A09729	279
A00437	A09736	409	A00437	A09738	324
A00437	A09882	165	A00437	A10579	183
A00437	A10603	255	A00437	A10611	425
A00437	A11523	212	A00437	A12038	337
A00437	A12047	489	A00437	A12648	446
A00437	A12897	313	A00437	A13074	165
A00437	A13207	188	A00437	A14179	168
A00437	A14193	462	A00437	A14196	281
A00437	A14375	193	A00437	A15249	271
A00437	A15315	291	A00437	A15323	335
A00437	A15347	488	A00437	A15376	63
A00437	A15556	486	A00437	A15557	247
A00437	A15644	490	A00437	A15683	491
A00437	A15712	210	A00437	A15719	160
A00437	A15788	151	A00437	A16042	171
A00437	A16292	183	A00437	A16495	227
A00437	A16665	201	A00437	A17198	490
A00438	A00446	543	A00438	A00448	452
A00438	A00457	435	A00438	A00472	330
A00438	A00485	391	A00438	A00531	491
A00438	A00532	383	A00438	A00537	490
A00438	A00576	279	A00438	A00597	441
A00438	A00649	394	A00438	A00653	486
A00438	A00680	425	A00438	A00697	351

A00438	A00709	301	A00438	A00724	180
A00438	A00725	258	A00438	A00737	241
A00438	A00821	456	A00438	A00841	317
A00438	A00849	491	A00438	A00868	631
A00438	A00871	486	A00438	A00878	338
A00438	A00883	535	A00438	A00911	382
A00438	A00915	491	A00438	A00926	446
A00438	A00934	416	A00438	A00953	477
A00438	A00967	491	A00438	A00983	333
A00438	A00985	205	A00438	A01020	481
A00438	A01021	388	A00438	A01022	374
A00438	A01027	335	A00438	A01075	290
A00438	A01076	205	A00438	A01086	265
A00438	A01100	319	A00438	A01108	467
A00438	A01122	277	A00438	A01135	361
A00438	A01150	449	A00438	A01156	682
A00438	A01161	491	A00438	A01164	173
A00438	A01169	491	A00438	A01209	306
A00438	A01210	244	A00438	A01211	446
A00438	A01234	491	A00438	A01253	411
A00438	A01271	358	A00438	A01278	346
A00438	A01304	176	A00438	A01320	486
A00438	A01322	267	A00438	A01329	367
A00438	A01369	263	A00438	A01380	333
A00438	A01385	483	A00438	A01413	472
A00438	A01483	404	A00438	A01502	201
A00438	A01573	271	A00438	A01582	491
A00438	A01627	356	A00438	A01640	172
A00438	A01820	249	A00438	A01984	180
A00438	A02477	353	A00438	A02607	462
A00438	A03139	466	A00438	A03676	424
A00438	A03713	345	A00438	A03885	456
A00438	A03945	196	A00438	A04145	430
A00438	A04256	165	A00438	A04337	420
A00438	A04668	268	A00438	A04782	286
A00438	A04915	249	A00438	A04925	372
A00438	A05095	187	A00438	A05337	380
A00438	A05417	227	A00438	A05577	198
A00438	A05843	270	A00438	A05982	286
A00438	A06015	168	A00438	A06147	205
A00438	A06275	151	A00438	A06600	260
A00438	A06615	310	A00438	A07071	270
A00438	A07414	168	A00438	A07433	171
A00438	A07442	404	A00438	A07520	154
A00438	A07697	200	A00438	A07700	219
A00438	A07706	393	A00438	A07894	452

A00438	A08144	165	A00438	A08191	187
A00438	A08945	249	A00438	A09112	205
A00438	A09451	256	A00438	A09512	749
A00438	A09515	152	A00438	A09538	316
A00438	A10150	249	A00438	A10369	283
A00438	A11044	168	A00438	A11123	152
A00438	A11242	224	A00438	A11352	361
A00438	A11830	272	A00438	A12657	187
A00438	A12824	286	A00438	A12900	249
A00438	A13824	177	A00438	A13890	301
A00438	A14080	299	A00438	A14132	224
A00438	A14196	180	A00438	A14272	347
A00438	A14449	483	A00438	A14643	182
A00438	A14655	441	A00438	A14699	199
A00438	A14701	480	A00438	A14894	187
A00438	A15295	490	A00438	A15297	210
A00438	A15498	467	A00438	A15524	165
A00438	A15624	171	A00438	A15645	195
A00438	A15917	413	A00438	A16145	232
A00438	A16647	187	A00438	A16994	427
A00438	A17078	272	A00439	A00454	434
A00439	A00527	251	A00439	A00626	215
A00439	A00698	470	A00439	A00812	185
A00439	A00932	486	A00439	A00933	486
A00439	A01083	311	A00439	A01560	489
A00439	A01612	523	A00439	A02505	152
A00439	A02877	247	A00439	A03740	436
A00439	A03752	694	A00439	A04833	332
A00439	A05453	217	A00439	A06466	694
A00439	A06950	438	A00439	A07311	570
A00439	A07798	695	A00439	A08137	483
A00439	A08519	165	A00439	A09263	341
A00439	A09319	737	A00439	A09504	221
A00439	A09804	171	A00439	A09902	480
A00439	A10506	321	A00439	A10728	470
A00439	A11286	491	A00439	A11332	165
A00439	A12328	473	A00439	A12990	441
A00439	A13149	467	A00439	A13320	480
A00439	A13335	791	A00439	A13340	246
A00439	A14073	441	A00439	A14257	224
A00439	A14571	219	A00439	A14738	380
A00439	A15106	296	A00439	A15244	435
A00439	A15246	357	A00439	A15697	168
A00439	A15741	173	A00439	A16291	180
A00439	A16939	218	A00439	A17142	423
A00440	A00496	257	A00440	A00535	395

A00440	A00560	435	A00440	A00650	369
A00440	A00714	154	A00440	A00822	283
A00440	A00853	296	A00440	A00856	185
A00440	A00883	424	A00440	A00954	489
A00440	A00957	423	A00440	A00981	180
A00440	A01088	241	A00440	A01091	314
A00440	A01097	355	A00440	A01143	475
A00440	A01157	601	A00440	A01493	219
A00440	A01500	351	A00440	A01503	333
A00440	A01524	490	A00440	A01555	432
A00440	A01598	422	A00440	A01723	218
A00440	A01822	346	A00440	A01995	491
A00440	A02052	216	A00440	A02088	254
A00440	A02139	180	A00440	A02174	235
A00440	A02236	144	A00440	A02245	288
A00440	A02356	180	A00440	A02382	176
A00440	A02416	283	A00440	A02452	200
A00440	A02464	435	A00440	A03081	255
A00440	A03109	163	A00440	A03110	462
A00440	A03136	470	A00440	A03183	481
A00440	A03219	205	A00440	A03304	355
A00440	A03719	174	A00440	A03765	278
A00440	A03799	168	A00440	A03919	177
A00440	A03942	408	A00440	A04033	445
A00440	A04076	424	A00440	A04437	168
A00440	A04449	682	A00440	A04492	165
A00440	A04773	477	A00440	A04949	601
A00440	A05182	191	A00440	A05288	456
A00440	A05418	458	A00440	A05706	484
A00440	A06118	334	A00440	A06491	322
A00440	A06554	561	A00440	A06599	534
A00440	A06907	216	A00440	A07004	412
A00440	A07311	184	A00440	A07706	308
A00440	A07803	319	A00440	A08382	202
A00440	A08553	398	A00440	A08996	235
A00440	A09732	401	A00440	A09734	171
A00440	A10018	202	A00440	A10090	380
A00440	A10332	165	A00440	A11395	157
A00440	A11621	270	A00440	A12111	491
A00440	A12365	491	A00440	A12660	344
A00440	A12873	283	A00440	A13587	337
A00440	A13928	263	A00440	A14196	394
A00440	A14202	307	A00440	A14210	434
A00440	A14247	179	A00440	A14371	312
A00440	A14570	235	A00440	A14636	210
A00440	A14647	151	A00440	A15114	306

A00440	A15224	179	A00440	A15257	174
A00440	A15292	281	A00440	A15365	255
A00440	A15366	205	A00440	A15419	260
A00440	A15447	151	A00440	A15492	208
A00440	A15596	378	A00440	A15606	441
A00440	A15700	259	A00440	A15763	348
A00440	A15852	402	A00440	A15870	184
A00440	A15916	179	A00440	A15952	317
A00440	A16323	188	A00440	A16623	162
A00440	A16724	251	A00440	A16978	301
A00441	A00459	180	A00441	A00488	474
A00441	A00501	682	A00441	A00589	539
A00441	A00605	682	A00441	A00690	452
A00441	A00749	210	A00441	A00791	302
A00441	A00861	416	A00441	A00872	210
A00441	A00916	443	A00441	A01028	210
A00441	A01065	557	A00441	A01092	210
A00441	A01098	210	A00441	A01120	568
A00441	A01179	212	A00441	A01206	489
A00441	A01234	212	A00441	A01356	177
A00441	A01379	414	A00441	A01384	168
A00441	A01439	499	A00441	A01470	467
A00441	A01488	297	A00441	A01492	568
A00441	A01538	210	A00441	A01539	473
A00441	A01598	540	A00441	A01612	201
A00441	A01614	390	A00441	A01649	491
A00441	A02265	210	A00441	A02978	210
A00441	A05230	210	A00441	A06340	174
A00441	A07076	479	A00441	A07347	490
A00441	A07725	419	A00441	A08137	344
A00441	A08357	489	A00441	A09292	346
A00441	A09327	193	A00441	A13052	210
A00441	A13875	288	A00441	A13989	561
A00441	A14216	414	A00441	A14816	210
A00441	A14817	205	A00441	A15532	490
A00441	A15735	462	A00441	A15739	198
A00442	A00481	484	A00442	A00500	429
A00442	A00532	490	A00442	A00693	491
A00442	A00769	63	A00442	A00871	491
A00442	A01020	339	A00442	A01024	491
A00442	A01116	159	A00442	A01181	288
A00442	A01184	187	A00442	A01201	240
A00442	A01214	491	A00442	A01226	488
A00442	A01234	486	A00442	A01304	446
A00442	A01382	176	A00442	A06915	251
A00442	A07414	434	A00442	A07467	232

A00442	A07695	491	A00442	A08144	474
A00442	A08269	255	A00442	A08892	434
A00442	A08971	229	A00442	A10208	257
A00442	A12124	340	A00442	A12447	402
A00442	A13219	235	A00442	A13706	357
A00442	A14006	252	A00442	A14449	293
A00442	A14451	323	A00442	A14655	276
A00442	A16320	363	A00442	A17049	260
A00443	A00501	177	A00443	A00760	174
A00443	A00964	382	A00443	A01075	472
A00443	A01079	258	A00443	A01082	121
A00443	A01084	323	A00443	A01087	417
A00443	A03944	347	A00443	A07706	363
A00443	A08118	177	A00443	A08527	213
A00443	A08996	291	A00443	A11674	446
A00443	A15520	395	A00443	A15733	243
A00443	A15852	208	A00444	A00489	658
A00444	A00605	391	A00444	A00651	378
A00444	A00666	218	A00444	A00667	440
A00444	A00839	488	A00444	A00843	484
A00444	A00848	480	A00444	A00970	491
A00444	A01031	170	A00444	A01069	484
A00444	A01074	252	A00444	A01101	201
A00444	A01113	316	A00444	A01115	659
A00444	A01166	340	A00444	A01192	391
A00444	A01194	491	A00444	A01196	236
A00444	A01205	260	A00444	A01218	215
A00444	A01249	177	A00444	A01276	389
A00444	A01291	455	A00444	A01393	447
A00444	A01396	184	A00444	A01411	474
A00444	A01455	424	A00444	A01496	358
A00444	A01539	659	A00444	A01540	344
A00444	A01548	216	A00444	A01557	190
A00444	A01569	491	A00444	A01577	400
A00444	A01579	480	A00444	A01612	177
A00444	A01770	350	A00444	A02040	232
A00444	A02153	299	A00444	A02640	232
A00444	A02661	190	A00444	A02877	475
A00444	A03224	238	A00444	A03439	272
A00444	A03446	658	A00444	A03453	488
A00444	A03476	380	A00444	A03477	375
A00444	A03560	223	A00444	A03572	419
A00444	A03573	215	A00444	A03675	162
A00444	A03721	193	A00444	A03722	193
A00444	A03738	412	A00444	A03740	258
A00444	A03752	243	A00444	A03785	491

A00444	A04021	329	A00444	A04449	391
A00444	A04519	232	A00444	A04563	190
A00444	A04681	162	A00444	A04846	173
A00444	A05060	457	A00444	A05232	311
A00444	A05385	196	A00444	A05442	294
A00444	A05501	313	A00444	A05712	434
A00444	A05845	173	A00444	A05995	152
A00444	A06109	247	A00444	A06365	183
A00444	A06965	154	A00444	A07247	435
A00444	A07323	193	A00444	A07449	157
A00444	A08061	428	A00444	A08390	390
A00444	A08546	563	A00444	A08668	480
A00444	A08676	468	A00444	A08896	441
A00444	A08900	319	A00444	A08972	168
A00444	A08978	255	A00444	A09276	196
A00444	A09586	200	A00444	A09591	219
A00444	A09600	162	A00444	A09691	281
A00444	A10006	433	A00444	A10490	491
A00444	A10532	659	A00444	A11103	434
A00444	A11240	372	A00444	A11409	491
A00444	A11477	180	A00444	A12763	337
A00444	A12839	352	A00444	A13218	223
A00444	A13609	481	A00444	A13674	294
A00444	A13729	168	A00444	A13989	424
A00444	A14073	402	A00444	A14130	424
A00444	A14351	247	A00444	A14394	230
A00444	A14833	271	A00444	A15358	224
A00444	A15470	212	A00444	A15475	272
A00444	A15684	399	A00444	A15698	372
A00444	A15827	200	A00444	A16118	260
A00444	A16756	200	A00444	A16923	441
A00445	A00482	199	A00445	A00491	208
A00445	A00496	227	A00445	A00531	315
A00445	A00552	475	A00445	A00718	460
A00445	A00883	466	A00445	A01091	479
A00445	A01131	491	A00445	A01132	229
A00445	A01259	475	A00445	A01275	656
A00445	A01276	191	A00445	A01280	174
A00445	A01406	234	A00445	A01441	162
A00445	A01572	199	A00445	A02212	201
A00445	A02452	483	A00445	A03057	154
A00445	A03149	274	A00445	A03224	276
A00445	A03721	232	A00445	A04844	310
A00445	A05015	458	A00445	A06082	253
A00445	A06118	224	A00445	A06151	196
A00445	A06488	259	A00445	A06778	159

A00445	A06907	168	A00445	A09044	172
A00445	A09825	258	A00445	A09980	356
A00445	A11039	490	A00445	A11291	491
A00445	A11477	491	A00445	A11631	228
A00445	A11713	477	A00445	A12241	193
A00445	A12518	465	A00445	A12532	318
A00445	A12534	475	A00445	A12944	356
A00445	A12991	422	A00445	A13178	333
A00445	A13348	184	A00445	A14007	434
A00445	A14128	470	A00445	A14196	491
A00445	A14220	489	A00445	A14371	193
A00445	A14689	305	A00445	A14809	199
A00445	A15237	278	A00445	A15297	165
A00445	A15309	418	A00445	A15470	251
A00445	A15471	319	A00445	A15475	305
A00445	A15482	162	A00445	A15525	177
A00445	A15635	205	A00445	A15739	165
A00445	A15916	201	A00445	A17063	154
A00445	A17076	267	A00446	A00450	168
A00446	A00519	240	A00446	A00525	277
A00446	A00531	531	A00446	A00532	440
A00446	A00548	480	A00446	A00553	709
A00446	A00564	313	A00446	A00565	497
A00446	A00570	467	A00446	A00572	216
A00446	A00583	599	A00446	A00597	299
A00446	A00600	481	A00446	A00601	338
A00446	A00607	347	A00446	A00618	251
A00446	A00629	434	A00446	A00652	438
A00446	A00674	545	A00446	A00687	473
A00446	A00691	491	A00446	A00714	501
A00446	A00723	319	A00446	A00728	480
A00446	A00735	303	A00446	A00751	543
A00446	A00761	541	A00446	A00766	544
A00446	A00776	462	A00446	A00781	491
A00446	A00782	228	A00446	A00791	746
A00446	A00801	168	A00446	A00805	443
A00446	A00813	204	A00446	A00815	491
A00446	A00816	443	A00446	A00819	224
A00446	A00846	299	A00446	A00862	483
A00446	A00864	529	A00446	A00868	779
A00446	A00883	810	A00446	A00884	529
A00446	A00903	151	A00446	A00915	465
A00446	A00923	598	A00446	A00926	545
A00446	A00927	537	A00446	A00936	374
A00446	A00956	183	A00446	A00975	545
A00446	A00981	217	A00446	A00982	260

A00446	A00983	406	A00446	A00985	182
A00446	A00989	327	A00446	A01009	391
A00446	A01014	358	A00446	A01017	468
A00446	A01020	472	A00446	A01039	189
A00446	A01058	543	A00446	A01066	409
A00446	A01068	273	A00446	A01072	245
A00446	A01075	446	A00446	A01077	342
A00446	A01080	544	A00446	A01088	557
A00446	A01091	658	A00446	A01097	634
A00446	A01100	584	A00446	A01106	485
A00446	A01122	486	A00446	A01124	99
A00446	A01151	490	A00446	A01156	563
A00446	A01169	491	A00446	A01173	259
A00446	A01180	485	A00446	A01191	436
A00446	A01212	278	A00446	A01213	274
A00446	A01234	241	A00446	A01235	238
A00446	A01241	229	A00446	A01243	486
A00446	A01258	74	A00446	A01264	249
A00446	A01267	673	A00446	A01277	159
A00446	A01278	491	A00446	A01281	483
A00446	A01291	579	A00446	A01329	491
A00446	A01338	483	A00446	A01339	540
A00446	A01358	328	A00446	A01370	483
A00446	A01371	375	A00446	A01372	599
A00446	A01390	486	A00446	A01391	469
A00446	A01419	424	A00446	A01438	213
A00446	A01442	466	A00446	A01451	740
A00446	A01459	489	A00446	A01461	185
A00446	A01470	184	A00446	A01483	299
A00446	A01492	529	A00446	A01507	486
A00446	A01514	491	A00446	A01516	170
A00446	A01546	247	A00446	A01559	199
A00446	A01582	491	A00446	A01588	534
A00446	A01599	159	A00446	A01606	463
A00446	A01622	221	A00446	A01660	259
A00446	A01695	165	A00446	A01720	246
A00446	A01823	326	A00446	A01985	63
A00446	A01988	429	A00446	A02041	188
A00446	A02212	538	A00446	A02252	276
A00446	A02381	634	A00446	A02452	466
A00446	A02978	486	A00446	A03012	452
A00446	A03043	93	A00446	A03328	240
A00446	A03334	291	A00446	A03458	291
A00446	A03494	224	A00446	A03498	199
A00446	A03503	491	A00446	A03567	485
A00446	A03570	486	A00446	A03651	188

A00446	A03676	491	A00446	A03749	441
A00446	A03750	491	A00446	A03859	481
A00446	A03873	262	A00446	A03885	438
A00446	A03948	358	A00446	A04033	486
A00446	A04180	333	A00446	A04421	185
A00446	A04449	283	A00446	A04666	477
A00446	A04687	235	A00446	A04944	218
A00446	A04962	240	A00446	A05095	332
A00446	A05234	240	A00446	A05249	485
A00446	A05276	221	A00446	A05563	238
A00446	A05570	221	A00446	A06214	171
A00446	A06272	488	A00446	A06520	422
A00446	A06624	374	A00446	A06750	480
A00446	A06861	159	A00446	A06907	154
A00446	A06938	455	A00446	A07081	196
A00446	A07096	160	A00446	A07101	406
A00446	A07209	157	A00446	A07226	494
A00446	A07266	313	A00446	A07282	374
A00446	A07355	176	A00446	A07360	185
A00446	A07403	428	A00446	A07457	477
A00446	A07483	154	A00446	A07516	171
A00446	A07575	185	A00446	A07641	193
A00446	A07675	461	A00446	A07697	235
A00446	A07706	488	A00446	A07750	171
A00446	A07756	491	A00446	A07813	332
A00446	A07851	162	A00446	A07853	208
A00446	A07956	470	A00446	A08022	422
A00446	A08025	171	A00446	A08032	407
A00446	A08097	318	A00446	A08133	199
A00446	A08159	185	A00446	A08205	240
A00446	A08213	326	A00446	A08248	597
A00446	A08281	491	A00446	A08356	468
A00446	A08362	348	A00446	A08517	473
A00446	A08524	586	A00446	A08558	445
A00446	A08570	313	A00446	A08834	160
A00446	A08882	479	A00446	A08927	221
A00446	A08960	458	A00446	A08977	439
A00446	A08982	518	A00446	A08989	316
A00446	A08990	395	A00446	A09315	165
A00446	A09319	899	A00446	A09343	176
A00446	A09351	185	A00446	A09627	160
A00446	A09659	202	A00446	A09670	278
A00446	A09672	229	A00446	A09876	208
A00446	A09898	113	A00446	A10156	356
A00446	A10159	488	A00446	A10232	171
A00446	A10372	493	A00446	A11001	385

A00446	A11279	361	A00446	A11365	160
A00446	A11395	278	A00446	A11472	248
A00446	A11544	262	A00446	A11598	271
A00446	A11649	599	A00446	A11706	412
A00446	A11735	537	A00446	A11737	446
A00446	A11914	537	A00446	A12153	171
A00446	A12255	345	A00446	A12282	165
A00446	A12658	528	A00446	A12810	165
A00446	A12824	696	A00446	A13340	162
A00446	A13356	394	A00446	A13447	326
A00446	A13452	171	A00446	A13595	171
A00446	A13618	165	A00446	A13784	187
A00446	A13892	262	A00446	A14007	451
A00446	A14009	199	A00446	A14148	157
A00446	A14167	160	A00446	A14196	870
A00446	A14218	475	A00446	A14246	552
A00446	A14345	134	A00446	A14362	262
A00446	A14566	385	A00446	A14597	288
A00446	A14650	188	A00446	A14655	465
A00446	A14701	489	A00446	A14709	483
A00446	A15136	318	A00446	A15185	299
A00446	A15232	171	A00446	A15237	576
A00446	A15257	524	A00446	A15297	814
A00446	A15321	574	A00446	A15323	539
A00446	A15324	606	A00446	A15330	414
A00446	A15338	599	A00446	A15339	561
A00446	A15353	626	A00446	A15355	356
A00446	A15359	660	A00446	A15363	345
A00446	A15366	306	A00446	A15374	463
A00446	A15419	462	A00446	A15502	190
A00446	A15529	433	A00446	A15535	236
A00446	A15540	179	A00446	A15558	491
A00446	A15559	681	A00446	A15561	195
A00446	A15564	599	A00446	A15624	491
A00446	A15631	360	A00446	A15649	213
A00446	A15651	210	A00446	A15660	162
A00446	A15661	317	A00446	A15684	519
A00446	A15729	633	A00446	A15735	299
A00446	A15738	395	A00446	A15747	486
A00446	A15763	435	A00446	A15788	491
A00446	A15900	171	A00446	A15946	241
A00446	A15947	468	A00446	A16022	168
A00446	A16152	252	A00446	A16215	152
A00446	A16311	350	A00446	A16312	171
A00446	A16463	246	A00446	A16614	262
A00446	A16654	157	A00446	A16690	491

A00446	A16700	262	A00446	A16866	491
A00446	A16874	344	A00446	A16962	241
A00446	A17019	157	A00446	A17063	369
A00446	A17134	457	A00447	A00452	694
A00447	A00481	348	A00447	A00487	378
A00447	A00493	199	A00447	A00524	306
A00447	A00532	262	A00447	A00537	329
A00447	A00540	193	A00447	A00555	695
A00447	A00570	574	A00447	A00571	432
A00447	A00581	236	A00447	A00597	373
A00447	A00603	340	A00447	A00606	273
A00447	A00609	450	A00447	A00629	544
A00447	A00648	274	A00447	A00683	190
A00447	A00687	574	A00447	A00693	159
A00447	A00694	337	A00447	A00717	491
A00447	A00718	410	A00447	A00721	403
A00447	A00727	485	A00447	A00760	338
A00447	A00762	401	A00447	A00772	694
A00447	A00793	347	A00447	A00798	235
A00447	A00822	152	A00447	A00826	440
A00447	A00839	208	A00447	A00841	165
A00447	A00854	329	A00447	A00865	488
A00447	A00878	346	A00447	A00907	424
A00447	A00913	159	A00447	A00928	574
A00447	A00947	204	A00447	A00952	695
A00447	A00965	291	A00447	A00968	307
A00447	A00972	217	A00447	A00977	502
A00447	A00981	411	A00447	A00997	217
A00447	A01016	303	A00447	A01027	435
A00447	A01067	254	A00447	A01074	489
A00447	A01107	574	A00447	A01154	383
A00447	A01159	530	A00447	A01166	412
A00447	A01209	327	A00447	A01211	224
A00447	A01217	399	A00447	A01221	640
A00447	A01238	371	A00447	A01243	573
A00447	A01252	356	A00447	A01280	232
A00447	A01304	290	A00447	A01306	375
A00447	A01352	858	A00447	A01365	581
A00447	A01377	241	A00447	A01399	470
A00447	A01406	454	A00447	A01412	353
A00447	A01416	374	A00447	A01418	275
A00447	A01518	229	A00447	A01547	168
A00447	A02051	224	A00447	A02164	465
A00447	A03035	199	A00447	A03556	217
A00447	A03601	505	A00447	A03617	217
A00447	A03734	165	A00447	A04099	165

A00447	A04668	436	A00447	A05327	308
A00447	A05375	357	A00447	A05501	583
A00447	A05605	217	A00447	A06122	486
A00447	A06204	462	A00447	A06347	461
A00447	A06369	261	A00447	A06472	269
A00447	A06531	338	A00447	A06832	571
A00447	A06861	236	A00447	A07312	235
A00447	A07349	229	A00447	A08183	512
A00447	A08293	367	A00447	A08402	467
A00447	A08403	199	A00447	A08477	176
A00447	A08501	302	A00447	A08554	180
A00447	A08567	462	A00447	A08813	389
A00447	A08984	693	A00447	A09059	416
A00447	A09609	238	A00447	A09621	480
A00447	A09673	217	A00447	A10317	380
A00447	A10373	324	A00447	A10789	218
A00447	A11277	340	A00447	A11675	227
A00447	A11707	157	A00447	A11950	157
A00447	A12255	296	A00447	A12292	278
A00447	A12507	162	A00447	A12658	238
A00447	A12816	484	A00447	A13787	383
A00447	A13788	188	A00447	A13804	352
A00447	A13805	180	A00447	A13905	249
A00447	A13963	435	A00447	A14077	283
A00447	A14088	237	A00447	A14122	157
A00447	A14186	337	A00447	A14220	152
A00447	A14230	156	A00447	A14246	174
A00447	A14270	294	A00447	A14272	489
A00447	A14478	373	A00447	A14482	294
A00447	A14566	329	A00447	A14701	419
A00447	A14835	299	A00447	A14917	319
A00447	A15377	463	A00447	A15543	307
A00447	A15724	278	A00447	A15949	307
A00447	A16117	152	A00447	A16118	193
A00447	A16566	201	A00447	A16567	226
A00447	A16664	217	A00447	A16701	218
A00447	A16774	235	A00447	A16815	252
A00447	A16874	190	A00447	A17152	221
A00448	A00459	215	A00448	A00463	491
A00448	A00488	708	A00448	A00490	704
A00448	A00494	236	A00448	A00497	708
A00448	A00500	436	A00448	A00518	450
A00448	A00520	486	A00448	A00521	160
A00448	A00530	321	A00448	A00532	491
A00448	A00535	393	A00448	A00537	480
A00448	A00550	637	A00448	A00551	491

A00448	A00554	708	A00448	A00566	491
A00448	A00575	491	A00448	A00589	168
A00448	A00595	488	A00448	A00605	638
A00448	A00648	362	A00448	A00691	488
A00448	A00693	468	A00448	A00708	491
A00448	A00760	490	A00448	A00767	491
A00448	A00787	182	A00448	A00805	472
A00448	A00813	419	A00448	A00822	269
A00448	A00824	274	A00448	A00831	627
A00448	A00848	475	A00448	A00856	491
A00448	A00858	491	A00448	A00868	473
A00448	A00870	407	A00448	A00871	302
A00448	A00881	490	A00448	A00883	469
A00448	A00898	251	A00448	A00903	491
A00448	A00916	491	A00448	A00919	480
A00448	A00923	439	A00448	A00926	385
A00448	A00934	491	A00448	A00944	356
A00448	A00954	491	A00448	A00960	162
A00448	A00975	180	A00448	A00992	429
A00448	A01004	515	A00448	A01017	251
A00448	A01020	708	A00448	A01024	185
A00448	A01032	458	A00448	A01040	334
A00448	A01075	491	A00448	A01084	477
A00448	A01087	491	A00448	A01088	489
A00448	A01099	217	A00448	A01108	353
A00448	A01112	372	A00448	A01116	638
A00448	A01147	163	A00448	A01150	477
A00448	A01166	486	A00448	A01179	375
A00448	A01184	491	A00448	A01193	350
A00448	A01194	154	A00448	A01195	372
A00448	A01201	491	A00448	A01206	491
A00448	A01224	286	A00448	A01226	348
A00448	A01234	491	A00448	A01304	638
A00448	A01322	420	A00448	A01349	449
A00448	A01351	491	A00448	A01398	350
A00448	A01401	454	A00448	A01404	249
A00448	A01415	152	A00448	A01425	393
A00448	A01429	252	A00448	A01430	252
A00448	A01431	428	A00448	A01454	462
A00448	A01468	488	A00448	A01470	491
A00448	A01488	486	A00448	A01492	491
A00448	A01495	334	A00448	A01503	364
A00448	A01576	477	A00448	A01598	491
A00448	A01612	477	A00448	A01614	486
A00448	A01649	491	A00448	A01684	451
A00448	A01700	221	A00448	A01741	433

A00448	A01848	229	A00448	A02064	470
A00448	A02265	274	A00448	A03438	213
A00448	A03567	332	A00448	A03569	177
A00448	A03595	358	A00448	A03734	377
A00448	A04139	391	A00448	A04687	234
A00448	A05845	416	A00448	A05986	299
A00448	A06275	491	A00448	A06803	271
A00448	A06881	313	A00448	A07223	636
A00448	A07244	263	A00448	A07347	491
A00448	A07487	259	A00448	A07524	491
A00448	A07533	452	A00448	A07584	490
A00448	A07625	486	A00448	A07697	363
A00448	A07700	638	A00448	A07706	491
A00448	A07725	621	A00448	A07924	491
A00448	A08022	491	A00448	A08024	491
A00448	A08118	265	A00448	A08137	465
A00448	A08211	353	A00448	A08326	475
A00448	A08357	435	A00448	A08382	405
A00448	A08439	490	A00448	A08450	457
A00448	A08491	701	A00448	A08518	367
A00448	A08553	491	A00448	A08890	480
A00448	A09010	452	A00448	A09292	350
A00448	A09307	460	A00448	A09619	708
A00448	A09664	307	A00448	A09665	157
A00448	A09705	174	A00448	A09708	262
A00448	A09917	151	A00448	A10465	317
A00448	A10469	163	A00448	A10616	488
A00448	A10923	265	A00448	A11395	491
A00448	A11565	274	A00448	A11720	182
A00448	A12052	182	A00448	A12108	173
A00448	A12254	488	A00448	A12305	232
A00448	A12419	167	A00448	A12696	232
A00448	A12744	266	A00448	A12824	473
A00448	A13055	151	A00448	A13294	486
A00448	A13989	491	A00448	A14006	278
A00448	A14505	236	A00448	A14566	179
A00448	A14655	450	A00448	A14701	408
A00448	A15215	303	A00448	A15373	218
A00448	A15733	489	A00448	A15762	344
A00448	A15764	486	A00448	A15945	374
A00448	A15946	479	A00448	A16067	457
A00448	A16144	466	A00448	A16212	228
A00448	A16302	485	A00448	A16842	173
A00449	A00453	330	A00449	A00468	490
A00449	A00472	162	A00449	A00478	196
A00449	A00484	172	A00449	A00537	383

A00449	A00541	491	A00449	A00574	491
A00449	A00614	285	A00449	A00628	440
A00449	A00633	436	A00449	A00651	152
A00449	A00654	491	A00449	A00659	174
A00449	A00660	488	A00449	A00661	446
A00449	A00662	488	A00449	A00663	456
A00449	A00666	457	A00449	A00681	486
A00449	A00695	419	A00449	A00696	491
A00449	A00738	335	A00449	A00770	452
A00449	A00773	395	A00449	A00784	337
A00449	A00813	200	A00449	A00825	491
A00449	A00868	227	A00449	A00892	249
A00449	A00962	491	A00449	A00993	457
A00449	A00995	208	A00449	A01005	475
A00449	A01008	490	A00449	A01018	491
A00449	A01046	401	A00449	A01137	170
A00449	A01138	213	A00449	A01174	301
A00449	A01176	272	A00449	A01222	491
A00449	A01223	491	A00449	A01225	447
A00449	A01227	480	A00449	A01230	491
A00449	A01233	449	A00449	A01256	251
A00449	A01297	491	A00449	A01322	345
A00449	A01344	469	A00449	A01397	394
A00449	A01404	385	A00449	A01409	491
A00449	A01461	486	A00449	A01515	324
A00449	A01573	213	A00449	A01585	319
A00449	A01590	224	A00449	A01635	491
A00449	A01643	477	A00449	A01668	167
A00449	A01713	389	A00449	A01722	193
A00449	A01740	921	A00449	A01741	446
A00449	A01743	455	A00449	A01755	438
A00449	A01793	159	A00449	A01883	176
A00449	A01899	423	A00449	A01926	480
A00449	A02008	227	A00449	A02011	337
A00449	A02014	254	A00449	A02020	404
A00449	A02023	196	A00449	A02071	160
A00449	A02083	420	A00449	A02114	385
A00449	A02129	202	A00449	A02215	152
A00449	A02252	154	A00449	A02254	490
A00449	A02284	358	A00449	A02306	246
A00449	A02316	449	A00449	A02377	357
A00449	A02443	346	A00449	A02463	224
A00449	A02468	154	A00449	A02491	218
A00449	A02521	491	A00449	A02524	257
A00449	A02529	216	A00449	A02574	240
A00449	A02691	193	A00449	A02777	491

A00449	A02778	398	A00449	A02779	491
A00449	A02859	177	A00449	A02930	185
A00449	A02992	457	A00449	A03016	199
A00449	A03050	474	A00449	A03056	210
A00449	A03059	278	A00449	A03099	424
A00449	A03146	353	A00449	A03148	218
A00449	A03167	485	A00449	A03168	165
A00449	A03174	154	A00449	A03234	174
A00449	A03259	441	A00449	A03370	374
A00449	A03423	491	A00449	A03442	232
A00449	A03484	488	A00449	A03513	491
A00449	A03545	337	A00449	A03795	469
A00449	A03968	413	A00449	A04095	417
A00449	A04188	221	A00449	A04228	452
A00449	A04332	184	A00449	A04366	228
A00449	A04460	165	A00449	A04556	249
A00449	A04702	358	A00449	A04791	486
A00449	A04839	424	A00449	A04883	491
A00449	A04891	383	A00449	A04927	491
A00449	A05006	273	A00449	A05007	467
A00449	A05014	241	A00449	A05045	491
A00449	A05050	424	A00449	A05051	479
A00449	A05064	252	A00449	A05224	177
A00449	A05297	475	A00449	A05309	165
A00449	A05315	899	A00449	A05330	447
A00449	A05331	378	A00449	A05337	483
A00449	A05358	330	A00449	A05376	270
A00449	A05403	299	A00449	A05416	488
A00449	A05418	489	A00449	A05420	232
A00449	A05424	450	A00449	A05433	232
A00449	A05451	299	A00449	A05462	218
A00449	A05463	465	A00449	A05548	477
A00449	A05549	247	A00449	A05550	485
A00449	A05573	491	A00449	A05610	328
A00449	A05753	358	A00449	A05754	350
A00449	A05757	185	A00449	A05771	196
A00449	A05789	286	A00449	A05883	335
A00449	A05912	369	A00449	A05966	330
A00449	A05986	456	A00449	A06014	196
A00449	A06165	210	A00449	A06245	188
A00449	A06358	232	A00449	A06385	308
A00449	A06402	369	A00449	A06405	491
A00449	A06439	491	A00449	A06466	199
A00449	A06553	247	A00449	A06607	358
A00449	A06626	184	A00449	A06635	176
A00449	A06638	221	A00449	A06639	268

A00449	A06641	462	A00449	A06650	191
A00449	A06682	247	A00449	A06689	457
A00449	A06700	434	A00449	A06733	274
A00449	A06809	268	A00449	A06847	436
A00449	A06855	422	A00449	A06860	491
A00449	A06970	450	A00449	A06979	313
A00449	A06994	369	A00449	A07007	219
A00449	A07027	446	A00449	A07037	417
A00449	A07059	491	A00449	A07078	165
A00449	A07088	491	A00449	A07109	462
A00449	A07122	458	A00449	A07205	163
A00449	A07230	247	A00449	A07238	268
A00449	A07239	475	A00449	A07311	199
A00449	A07354	328	A00449	A07366	407
A00449	A07407	204	A00449	A07408	291
A00449	A07477	466	A00449	A07501	185
A00449	A07503	485	A00449	A07520	491
A00449	A07553	179	A00449	A07558	406
A00449	A07559	167	A00449	A07590	411
A00449	A07619	196	A00449	A07674	491
A00449	A07684	319	A00449	A07688	260
A00449	A07690	488	A00449	A07736	479
A00449	A07757	490	A00449	A07763	306
A00449	A07779	299	A00449	A07800	279
A00449	A07847	281	A00449	A07851	346
A00449	A07856	312	A00449	A07933	472
A00449	A07973	324	A00449	A08114	184
A00449	A08127	174	A00449	A08138	163
A00449	A08155	278	A00449	A08366	308
A00449	A08368	232	A00449	A08372	256
A00449	A08385	418	A00449	A08636	232
A00449	A08785	201	A00449	A09126	188
A00449	A09845	427	A00449	A10420	406
A00449	A10424	157	A00449	A10444	201
A00449	A10446	216	A00449	A10456	473
A00449	A10537	299	A00449	A10625	335
A00449	A10635	486	A00449	A10652	235
A00449	A10686	260	A00449	A10687	380
A00449	A10690	278	A00449	A10693	198
A00449	A10696	380	A00449	A10708	286
A00449	A10715	243	A00449	A10780	307
A00449	A10883	268	A00449	A10926	202
A00449	A11425	311	A00449	A11457	417
A00449	A12033	210	A00449	A12055	188
A00449	A12507	491	A00449	A13155	221
A00449	A13179	247	A00449	A13871	394

A00449	A14247	463	A00449	A14828	335
A00449	A14907	268	A00449	A15390	174
A00449	A15637	180	A00449	A15874	168
A00449	A16027	338	A00449	A16138	162
A00449	A16529	434	A00449	A16741	483
A00449	A16744	319	A00449	A17006	480
A00449	A17046	162	A00449	A17078	486
A00450	A00471	185	A00450	A00522	585
A00450	A00540	224	A00450	A00546	179
A00450	A00562	491	A00450	A00565	577
A00450	A00606	154	A00450	A00625	689
A00450	A00638	491	A00450	A00691	379
A00450	A00786	490	A00450	A00805	404
A00450	A00812	473	A00450	A00814	251
A00450	A00824	226	A00450	A00829	229
A00450	A00862	688	A00450	A00864	375
A00450	A00877	480	A00450	A00941	491
A00450	A00951	168	A00450	A00971	446
A00450	A00989	491	A00450	A01021	490
A00450	A01036	485	A00450	A01091	255
A00450	A01169	193	A00450	A01216	457
A00450	A01242	469	A00450	A01291	241
A00450	A01327	428	A00450	A01345	193
A00450	A01358	490	A00450	A01366	424
A00450	A01402	167	A00450	A01454	229
A00450	A01459	686	A00450	A01472	488
A00450	A01507	491	A00450	A01514	581
A00450	A01541	247	A00450	A01568	491
A00450	A01622	373	A00450	A01647	491
A00450	A01681	443	A00450	A01687	468
A00450	A01988	629	A00450	A02535	462
A00450	A03330	213	A00450	A03528	210
A00450	A03597	393	A00450	A03769	170
A00450	A05008	268	A00450	A05015	319
A00450	A05016	402	A00450	A05301	368
A00450	A05428	462	A00450	A05638	441
A00450	A05872	191	A00450	A05894	346
A00450	A06176	419	A00450	A06819	488
A00450	A06924	205	A00450	A06941	477
A00450	A07054	475	A00450	A07198	168
A00450	A07207	489	A00450	A07242	491
A00450	A07253	401	A00450	A07364	249
A00450	A07495	154	A00450	A07665	193
A00450	A07706	152	A00450	A07802	391
A00450	A07893	439	A00450	A07954	171
A00450	A08934	441	A00450	A08962	260

A00450	A09083	180	A00450	A09650	273
A00450	A10996	324	A00450	A11284	491
A00450	A12047	578	A00450	A12048	154
A00450	A13146	232	A00450	A13949	388
A00450	A14167	154	A00450	A14209	491
A00450	A14222	491	A00450	A14281	480
A00450	A14474	200	A00450	A14484	764
A00450	A14540	230	A00450	A14633	710
A00450	A14827	196	A00450	A15197	152
A00450	A15222	146	A00450	A15232	229
A00450	A15295	303	A00450	A15370	491
A00450	A15380	491	A00450	A15618	358
A00450	A15644	200	A00450	A15665	491
A00450	A16552	491	A00450	A16611	268
A00450	A16980	229	A00451	A00697	63
A00451	A00931	486	A00451	A01021	490
A00451	A01059	204	A00451	A01286	486
A00451	A01307	63	A00451	A01315	489
A00451	A01342	473	A00451	A01546	254
A00451	A01832	439	A00451	A02278	491
A00451	A02931	462	A00451	A03110	179
A00451	A03271	319	A00451	A03314	475
A00451	A03398	152	A00451	A03625	65
A00451	A03684	213	A00451	A03784	200
A00451	A03830	483	A00451	A04015	154
A00451	A04111	288	A00451	A04323	187
A00451	A04668	474	A00451	A04731	193
A00451	A04737	174	A00451	A05310	152
A00451	A05400	347	A00451	A05534	306
A00451	A06650	461	A00451	A06982	483
A00451	A07148	227	A00451	A07173	343
A00451	A07311	899	A00451	A07883	172
A00451	A08502	489	A00451	A09744	132
A00451	A09997	199	A00451	A10899	171
A00451	A13605	195	A00451	A13829	63
A00451	A13994	281	A00451	A14072	470
A00451	A14103	322	A00451	A14193	488
A00451	A14208	469	A00451	A14319	177
A00451	A14502	179	A00451	A15314	221
A00451	A15325	221	A00451	A15557	486
A00451	A15677	210	A00451	A15834	152
A00451	A16911	151	A00452	A00480	371
A00452	A00503	305	A00452	A00508	168
A00452	A00555	695	A00452	A00570	572
A00452	A00629	424	A00452	A00670	414
A00452	A00687	573	A00452	A00689	296

A00452	A00717	490	A00452	A00721	561
A00452	A00727	488	A00452	A00772	693
A00452	A00854	339	A00452	A00865	407
A00452	A00867	419	A00452	A00883	384
A00452	A00928	575	A00452	A00952	694
A00452	A00972	531	A00452	A00997	217
A00452	A01091	428	A00452	A01107	574
A00452	A01159	217	A00452	A01221	471
A00452	A01243	572	A00452	A01255	441
A00452	A01352	694	A00452	A01412	229
A00452	A01418	217	A00452	A01451	255
A00452	A01598	452	A00452	A02212	414
A00452	A02877	273	A00452	A02910	221
A00452	A03065	486	A00452	A03556	217
A00452	A03617	217	A00452	A03887	165
A00452	A04075	213	A00452	A05605	217
A00452	A06122	172	A00452	A06204	472
A00452	A06347	384	A00452	A06369	372
A00452	A06698	162	A00452	A06832	565
A00452	A07756	414	A00452	A07956	414
A00452	A08183	488	A00452	A08467	419
A00452	A08515	321	A00452	A08517	429
A00452	A08982	445	A00452	A08984	679
A00452	A08990	229	A00452	A09627	414
A00452	A09673	470	A00452	A09734	208
A00452	A10317	322	A00452	A11277	296
A00452	A11406	201	A00452	A11584	243
A00452	A13397	159	A00452	A14087	172
A00452	A14088	479	A00452	A14186	446
A00452	A14196	434	A00452	A14246	278
A00452	A14596	185	A00452	A14836	168
A00452	A15237	180	A00452	A15278	293
A00452	A15297	185	A00452	A15323	480
A00452	A15324	489	A00452	A15339	221
A00452	A15353	452	A00452	A15370	485
A00452	A15374	422	A00452	A15377	693
A00452	A15543	574	A00452	A15564	452
A00452	A15677	472	A00452	A15724	518
A00452	A16463	470	A00452	A16495	379
A00452	A16664	217	A00452	A17063	419
A00453	A00458	491	A00453	A00529	241
A00453	A00531	374	A00453	A00532	490
A00453	A00541	452	A00453	A00547	157
A00453	A00574	491	A00453	A00579	199
A00453	A00614	281	A00453	A00628	335
A00453	A00654	491	A00453	A00660	191

A00453	A00666	455	A00453	A00681	491
A00453	A00695	313	A00453	A00731	218
A00453	A00738	481	A00453	A00739	218
A00453	A00762	208	A00453	A00770	452
A00453	A00771	190	A00453	A00773	227
A00453	A00905	187	A00453	A00916	401
A00453	A00919	428	A00453	A00962	490
A00453	A00995	435	A00453	A01008	491
A00453	A01029	213	A00453	A01040	162
A00453	A01065	154	A00453	A01075	491
A00453	A01082	490	A00453	A01084	450
A00453	A01087	490	A00453	A01109	369
A00453	A01150	162	A00453	A01174	190
A00453	A01175	484	A00453	A01223	204
A00453	A01233	401	A00453	A01255	160
A00453	A01256	443	A00453	A01268	195
A00453	A01272	439	A00453	A01295	455
A00453	A01354	205	A00453	A01397	393
A00453	A01442	154	A00453	A01481	188
A00453	A01508	466	A00453	A01597	162
A00453	A01598	385	A00453	A01614	152
A00453	A01635	491	A00453	A01707	299
A00453	A01735	162	A00453	A01787	207
A00453	A01857	299	A00453	A02053	185
A00453	A02290	313	A00453	A02366	262
A00453	A02521	207	A00453	A02992	272
A00453	A03004	157	A00453	A03016	267
A00453	A03167	419	A00453	A03168	434
A00453	A03441	196	A00453	A03477	466
A00453	A03487	174	A00453	A03569	154
A00453	A03861	306	A00453	A04779	491
A00453	A04784	235	A00453	A04791	263
A00453	A04834	425	A00453	A04850	205
A00453	A04995	353	A00453	A05007	488
A00453	A05013	254	A00453	A05102	396
A00453	A05416	440	A00453	A05462	151
A00453	A05472	213	A00453	A05516	358
A00453	A05759	271	A00453	A05774	488
A00453	A05820	281	A00453	A05883	163
A00453	A06088	205	A00453	A06252	179
A00453	A06358	461	A00453	A06359	286
A00453	A06405	291	A00453	A06633	422
A00453	A06700	190	A00453	A06756	232
A00453	A06847	172	A00453	A06912	159
A00453	A06968	302	A00453	A06970	416
A00453	A07059	296	A00453	A07109	366

A00453	A07122	483	A00453	A07238	430
A00453	A07256	210	A00453	A07304	420
A00453	A07376	180	A00453	A07413	479
A00453	A07530	388	A00453	A07553	450
A00453	A07554	474	A00453	A07590	373
A00453	A07690	151	A00453	A07706	488
A00453	A07736	156	A00453	A07800	348
A00453	A07885	299	A00453	A08108	414
A00453	A08113	154	A00453	A08137	467
A00453	A08155	199	A00453	A08248	201
A00453	A08431	151	A00453	A08694	270
A00453	A08751	210	A00453	A08991	180
A00453	A09849	340	A00453	A10020	294
A00453	A10038	179	A00453	A10443	187
A00453	A10444	268	A00453	A10452	238
A00453	A10456	443	A00453	A10475	196
A00453	A10702	279	A00453	A10712	480
A00453	A10714	406	A00453	A10880	311
A00453	A10885	382	A00453	A11132	191
A00453	A11271	162	A00453	A11297	171
A00453	A11309	160	A00453	A11324	195
A00453	A11325	468	A00453	A11381	484
A00453	A11496	291	A00453	A11695	177
A00453	A11703	285	A00453	A11930	483
A00453	A11933	465	A00453	A12850	213
A00453	A13311	187	A00453	A13314	193
A00453	A13527	379	A00453	A13735	156
A00453	A13781	270	A00453	A13804	196
A00453	A13989	399	A00453	A14086	215
A00453	A14272	171	A00453	A14298	391
A00453	A14337	235	A00453	A14566	205
A00453	A14628	199	A00453	A14821	210
A00453	A14903	170	A00453	A14917	159
A00453	A15353	259	A00453	A15371	177
A00453	A15654	201	A00453	A15870	480
A00453	A15874	469	A00453	A15905	469
A00453	A15940	160	A00453	A15941	159
A00453	A15951	191	A00453	A16027	266
A00453	A16028	163	A00453	A16060	213
A00453	A16103	172	A00453	A16215	177
A00453	A16262	422	A00453	A16291	241
A00453	A16346	294	A00453	A16529	205
A00453	A16587	152	A00453	A16646	156
A00453	A16798	491	A00453	A16822	172
A00453	A16896	226	A00453	A16944	187
A00453	A16959	484	A00453	A17005	400

A00453	A17012	159	A00453	A17033	167
A00453	A17036	174	A00453	A17040	185
A00453	A17142	205	A00454	A00489	383
A00454	A00494	252	A00454	A00521	388
A00454	A00532	165	A00454	A00537	491
A00454	A00544	373	A00454	A00564	462
A00454	A00571	462	A00454	A00576	311
A00454	A00604	491	A00454	A00605	724
A00454	A00629	441	A00454	A00640	152
A00454	A00648	446	A00454	A00651	450
A00454	A00653	620	A00454	A00687	414
A00454	A00694	363	A00454	A00697	491
A00454	A00698	447	A00454	A00700	484
A00454	A00701	105	A00454	A00726	445
A00454	A00741	429	A00454	A00762	180
A00454	A00782	281	A00454	A00783	491
A00454	A00785	483	A00454	A00787	180
A00454	A00790	447	A00454	A00808	256
A00454	A00809	491	A00454	A00810	152
A00454	A00811	485	A00454	A00831	333
A00454	A00833	299	A00454	A00839	491
A00454	A00843	185	A00454	A00848	480
A00454	A00878	443	A00454	A00910	695
A00454	A00912	368	A00454	A00922	704
A00454	A00932	445	A00454	A00935	420
A00454	A00970	224	A00454	A00974	299
A00454	A00980	343	A00454	A00984	605
A00454	A00987	299	A00454	A01001	544
A00454	A01027	483	A00454	A01038	283
A00454	A01042	695	A00454	A01052	207
A00454	A01054	412	A00454	A01075	447
A00454	A01082	462	A00454	A01083	491
A00454	A01101	401	A00454	A01102	299
A00454	A01108	279	A00454	A01112	368
A00454	A01115	745	A00454	A01121	213
A00454	A01127	413	A00454	A01150	491
A00454	A01161	455	A00454	A01166	297
A00454	A01192	411	A00454	A01206	457
A00454	A01219	285	A00454	A01234	379
A00454	A01249	157	A00454	A01261	168
A00454	A01265	317	A00454	A01291	491
A00454	A01300	490	A00454	A01311	152
A00454	A01335	452	A00454	A01354	460
A00454	A01369	245	A00454	A01379	620
A00454	A01387	610	A00454	A01411	449
A00454	A01413	160	A00454	A01431	491

A00454	A01435	491	A00454	A01439	299
A00454	A01446	526	A00454	A01468	474
A00454	A01470	483	A00454	A01540	480
A00454	A01569	468	A00454	A01577	152
A00454	A01579	484	A00454	A01586	249
A00454	A01601	457	A00454	A01612	374
A00454	A01627	299	A00454	A01641	188
A00454	A01649	477	A00454	A01707	234
A00454	A01822	188	A00454	A01850	474
A00454	A02350	489	A00454	A02601	603
A00454	A02703	401	A00454	A02791	269
A00454	A02864	401	A00454	A03298	473
A00454	A03446	430	A00454	A03516	249
A00454	A03567	474	A00454	A03697	152
A00454	A03740	491	A00454	A03752	490
A00454	A03809	491	A00454	A03817	279
A00454	A03981	177	A00454	A04495	299
A00454	A05086	693	A00454	A05419	205
A00454	A05501	461	A00454	A06037	257
A00454	A06287	152	A00454	A06466	491
A00454	A06742	188	A00454	A07311	491
A00454	A07323	246	A00454	A07362	201
A00454	A07394	462	A00454	A07398	132
A00454	A07451	204	A00454	A07527	296
A00454	A07686	177	A00454	A07744	152
A00454	A07798	468	A00454	A08053	490
A00454	A08054	491	A00454	A08061	152
A00454	A08098	152	A00454	A08110	272
A00454	A08137	466	A00454	A08178	152
A00454	A08359	457	A00454	A08571	489
A00454	A08763	152	A00454	A08826	362
A00454	A08835	171	A00454	A08947	299
A00454	A09004	299	A00454	A09476	430
A00454	A09592	389	A00454	A09902	196
A00454	A10532	516	A00454	A10842	152
A00454	A11286	301	A00454	A11296	174
A00454	A11302	491	A00454	A11322	348
A00454	A11338	302	A00454	A11344	394
A00454	A11372	491	A00454	A11480	151
A00454	A11787	236	A00454	A11944	245
A00454	A12167	486	A00454	A12328	351
A00454	A12329	360	A00454	A12418	272
A00454	A12569	162	A00454	A13335	458
A00454	A13340	490	A00454	A13360	272
A00454	A13494	188	A00454	A13522	288
A00454	A13865	236	A00454	A13989	488

A00454	A14073	491	A00454	A14216	490
A00454	A14476	162	A00454	A14525	152
A00454	A14596	485	A00454	A14614	234
A00454	A14642	345	A00454	A14937	263
A00454	A15244	468	A00454	A15275	491
A00454	A15375	152	A00454	A15546	152
A00454	A15652	235	A00454	A15660	491
A00454	A15666	481	A00454	A15684	491
A00454	A15694	472	A00454	A15695	362
A00454	A15699	462	A00454	A15730	241
A00454	A15769	340	A00454	A15889	168
A00454	A15931	480	A00454	A15932	160
A00454	A15988	260	A00454	A16088	296
A00454	A16092	375	A00454	A16157	270
A00454	A16633	481	A00454	A17134	288
A00454	A17142	335	A00454	A17188	200
A00455	A00486	249	A00455	A00582	488
A00455	A00664	411	A00455	A00736	394
A00455	A01046	258	A00455	A01185	394
A00455	A01361	338	A00455	A01405	179
A00455	A01419	449	A00455	A01835	247
A00455	A01915	188	A00455	A01926	491
A00455	A02011	204	A00455	A02342	182
A00455	A02397	163	A00455	A02405	252
A00455	A02490	177	A00455	A02491	382
A00455	A02539	430	A00455	A02593	152
A00455	A02623	224	A00455	A02687	154
A00455	A02697	363	A00455	A02713	200
A00455	A02741	165	A00455	A02751	390
A00455	A02756	167	A00455	A02761	353
A00455	A02798	180	A00455	A02837	193
A00455	A02888	163	A00455	A02961	193
A00455	A03034	288	A00455	A03777	224
A00455	A04487	490	A00455	A04548	152
A00455	A04567	162	A00455	A04600	276
A00455	A04793	205	A00455	A04805	324
A00455	A04962	151	A00455	A05256	187
A00455	A05278	430	A00455	A05309	180
A00455	A05559	288	A00455	A05571	332
A00455	A05591	491	A00455	A05665	195
A00455	A05667	288	A00455	A05828	351
A00455	A05900	332	A00455	A07129	449
A00455	A07343	355	A00455	A07933	454
A00455	A08638	306	A00455	A09036	224
A00455	A10091	447	A00455	A10159	486
A00455	A10358	154	A00455	A10414	301

A00455	A10609	491	A00455	A10742	238
A00455	A11498	407	A00455	A12629	355
A00455	A13500	213	A00455	A13647	229
A00455	A14454	484	A00455	A15224	201
A00455	A15228	468	A00455	A16623	185
A00456	A00460	183	A00456	A00595	330
A00456	A00619	218	A00456	A00691	177
A00456	A00749	205	A00456	A00767	321
A00456	A00916	95	A00456	A00918	380
A00456	A00992	409	A00456	A01092	241
A00456	A01098	230	A00456	A01136	382
A00456	A01170	323	A00456	A01278	157
A00456	A01279	224	A00456	A01351	279
A00456	A01538	162	A00456	A02164	79
A00456	A02265	401	A00456	A03028	217
A00456	A03432	252	A00456	A03438	180
A00456	A03628	196	A00456	A03629	321
A00456	A05415	172	A00456	A07244	262
A00456	A07378	428	A00456	A16076	249
A00457	A00460	467	A00457	A00473	408
A00457	A00502	247	A00457	A00506	306
A00457	A00514	137	A00457	A00523	475
A00457	A00525	467	A00457	A00531	404
A00457	A00537	207	A00457	A00561	430
A00457	A00571	474	A00457	A00604	445
A00457	A00649	264	A00457	A00651	427
A00457	A00653	591	A00457	A00731	247
A00457	A00737	154	A00457	A00741	393
A00457	A00783	321	A00457	A00785	286
A00457	A00790	382	A00457	A00796	229
A00457	A00820	360	A00457	A00821	449
A00457	A00822	356	A00457	A00849	592
A00457	A00878	232	A00457	A00902	234
A00457	A00953	445	A00457	A00974	247
A00457	A00984	294	A00457	A00985	190
A00457	A00987	343	A00457	A00998	488
A00457	A01013	402	A00457	A01022	294
A00457	A01027	247	A00457	A01042	401
A00457	A01086	585	A00457	A01108	356
A00457	A01109	265	A00457	A01127	252
A00457	A01150	592	A00457	A01161	592
A00457	A01204	247	A00457	A01210	395
A00457	A01211	353	A00457	A01215	307
A00457	A01245	260	A00457	A01253	168
A00457	A01275	218	A00457	A01287	591
A00457	A01328	163	A00457	A01346	247

A00457	A01353	218	A00457	A01399	287
A00457	A01413	299	A00457	A01431	343
A00457	A01443	247	A00457	A01448	177
A00457	A01468	224	A00457	A01470	159
A00457	A01496	281	A00457	A01502	480
A00457	A01504	427	A00457	A01540	490
A00457	A01543	297	A00457	A01567	353
A00457	A01572	162	A00457	A01582	486
A00457	A01627	160	A00457	A01667	491
A00457	A01668	188	A00457	A01711	456
A00457	A01815	260	A00457	A01819	389
A00457	A01825	469	A00457	A01832	390
A00457	A01833	260	A00457	A01844	418
A00457	A01957	157	A00457	A02138	191
A00457	A02162	210	A00457	A02208	165
A00457	A02994	588	A00457	A03150	210
A00457	A03257	243	A00457	A03320	229
A00457	A03435	404	A00457	A03456	171
A00457	A03520	154	A00457	A03716	235
A00457	A03740	408	A00457	A03790	254
A00457	A03856	274	A00457	A03885	232
A00457	A03959	342	A00457	A04056	347
A00457	A04265	157	A00457	A04304	196
A00457	A04473	302	A00457	A04903	208
A00457	A05041	229	A00457	A05449	172
A00457	A05608	372	A00457	A05703	270
A00457	A05734	171	A00457	A06048	329
A00457	A06463	286	A00457	A06794	329
A00457	A06804	329	A00457	A07102	302
A00457	A07160	243	A00457	A07403	229
A00457	A07446	154	A00457	A07773	345
A00457	A07793	190	A00457	A07990	224
A00457	A08073	270	A00457	A08118	200
A00457	A08191	418	A00457	A08528	457
A00457	A08903	312	A00457	A09321	243
A00457	A09709	160	A00457	A09778	171
A00457	A09941	235	A00457	A10598	405
A00457	A11352	270	A00457	A11677	247
A00457	A12056	329	A00457	A12143	329
A00457	A13287	243	A00457	A13555	306
A00457	A13769	329	A00457	A14073	303
A00457	A14699	591	A00457	A14778	421
A00457	A14816	173	A00457	A15109	385
A00457	A15117	329	A00457	A15624	423
A00457	A15668	177	A00457	A15684	465
A00457	A15699	171	A00457	A15917	441

A00457	A16072	283	A00457	A16076	452
A00457	A16082	162	A00457	A16096	154
A00457	A16299	205	A00457	A16587	247
A00458	A00481	341	A00458	A00491	205
A00458	A00501	379	A00458	A00531	491
A00458	A00532	491	A00458	A00560	338
A00458	A00589	385	A00458	A00604	290
A00458	A00605	457	A00458	A00658	238
A00458	A00760	454	A00458	A00762	425
A00458	A00793	271	A00458	A00822	484
A00458	A00835	423	A00458	A00879	314
A00458	A00883	439	A00458	A00905	477
A00458	A00916	491	A00458	A00919	491
A00458	A00940	363	A00458	A00947	430
A00458	A01029	201	A00458	A01075	491
A00458	A01077	318	A00458	A01082	491
A00458	A01084	491	A00458	A01087	491
A00458	A01150	274	A00458	A01166	430
A00458	A01206	488	A00458	A01234	469
A00458	A01285	361	A00458	A01291	484
A00458	A01354	491	A00458	A01406	480
A00458	A01470	347	A00458	A01488	213
A00458	A01492	241	A00458	A01503	329
A00458	A01540	420	A00458	A01598	491
A00458	A01612	480	A00458	A01614	486
A00458	A01649	477	A00458	A01707	491
A00458	A01731	199	A00458	A03171	480
A00458	A03446	272	A00458	A03569	477
A00458	A03601	412	A00458	A03740	306
A00458	A03752	221	A00458	A03757	299
A00458	A03817	489	A00458	A05230	314
A00458	A05501	279	A00458	A05633	341
A00458	A06466	260	A00458	A06907	485
A00458	A07004	265	A00458	A07414	183
A00458	A07524	296	A00458	A07604	483
A00458	A07706	491	A00458	A07956	171
A00458	A08137	491	A00458	A08214	157
A00458	A08357	344	A00458	A08527	454
A00458	A08571	394	A00458	A09825	355
A00458	A10624	307	A00458	A11109	190
A00458	A11452	307	A00458	A11930	491
A00458	A11948	167	A00458	A12329	204
A00458	A12543	207	A00458	A12746	278
A00458	A13989	491	A00458	A14006	489
A00458	A14220	491	A00458	A14221	185
A00458	A14566	490	A00458	A14594	469

A00458	A14596	265	A00458	A14835	218
A00458	A15141	167	A00458	A15215	294
A00458	A15366	380	A00458	A15463	213
A00458	A15660	210	A00458	A15733	185
A00458	A15773	299	A00458	A16044	243
A00458	A16060	227	A00458	A16328	217
A00458	A16619	278	A00458	A17063	463
A00458	A17142	252	A00459	A00488	443
A00459	A00520	251	A00459	A00550	490
A00459	A00584	174	A00459	A00595	174
A00459	A00690	223	A00459	A00749	174
A00459	A00761	191	A00459	A00767	174
A00459	A00908	174	A00459	A00916	453
A00459	A00918	174	A00459	A00956	313
A00459	A00960	174	A00459	A00992	174
A00459	A01020	224	A00459	A01116	238
A00459	A01184	455	A00459	A01193	174
A00459	A01234	326	A00459	A01279	174
A00459	A01339	185	A00459	A01351	174
A00459	A01365	196	A00459	A01424	174
A00459	A01492	251	A00459	A01539	411
A00459	A02364	408	A00459	A05845	291
A00459	A06660	157	A00459	A06698	174
A00459	A07054	402	A00459	A07223	321
A00459	A07244	174	A00459	A07347	490
A00459	A07432	174	A00459	A07524	174
A00459	A07625	174	A00459	A07661	399
A00459	A07725	480	A00459	A07924	174
A00459	A07961	191	A00459	A11544	174
A00459	A11831	174	A00459	A11832	174
A00459	A14188	191	A00459	A15354	226
A00460	A00531	260	A00460	A00571	221
A00460	A00598	204	A00460	A00604	172
A00460	A00651	198	A00460	A00653	484
A00460	A00697	173	A00460	A00731	159
A00460	A00741	391	A00460	A00745	229
A00460	A00796	294	A00460	A00806	223
A00460	A00821	462	A00460	A00849	210
A00460	A00987	353	A00460	A01013	263
A00460	A01023	278	A00460	A01042	202
A00460	A01150	489	A00460	A01155	152
A00460	A01161	477	A00460	A01192	232
A00460	A01245	204	A00460	A01380	626
A00460	A01393	152	A00460	A01413	472
A00460	A01496	160	A00460	A01540	634
A00460	A01569	191	A00460	A01611	171

A00460	A01648	286	A00460	A01667	355
A00460	A01711	191	A00460	A01819	243
A00460	A01827	177	A00460	A01850	484
A00460	A02059	228	A00460	A02353	324
A00460	A02877	63	A00460	A03398	159
A00460	A03456	241	A00460	A03467	322
A00460	A03516	356	A00460	A03671	187
A00460	A03691	241	A00460	A03740	622
A00460	A03759	489	A00460	A04752	257
A00460	A05086	324	A00460	A05375	185
A00460	A06048	371	A00460	A07081	347
A00460	A07102	324	A00460	A07187	324
A00460	A07258	473	A00460	A07502	371
A00460	A09546	486	A00460	A11080	371
A00460	A11296	223	A00460	A11374	324
A00460	A11694	324	A00460	A14073	630
A00460	A14479	241	A00460	A14642	429
A00460	A14699	484	A00460	A15653	235
A00460	A15843	324	A00460	A15891	235
A00460	A16548	337	A00461	A00515	217
A00461	A00524	188	A00461	A01169	267
A00461	A03770	157	A00461	A04666	369
A00461	A08382	157	A00461	A09531	276
A00461	A10706	184	A00461	A10859	362
A00461	A14356	241	A00462	A00541	477
A00462	A00551	435	A00462	A00574	479
A00462	A00628	465	A00462	A00654	425
A00462	A00663	168	A00462	A00770	208
A00462	A00771	174	A00462	A00773	364
A00462	A00929	241	A00462	A00962	490
A00462	A01005	182	A00462	A01233	491
A00462	A01256	485	A00462	A01347	229
A00462	A01481	249	A00462	A01635	488
A00462	A02082	180	A00462	A02204	305
A00462	A02312	283	A00462	A02432	199
A00462	A02444	319	A00462	A02531	152
A00462	A02673	490	A00462	A03004	151
A00462	A03168	227	A00462	A04307	188
A00462	A05059	262	A00462	A05277	314
A00462	A05472	307	A00462	A05767	401
A00462	A05768	274	A00462	A05771	241
A00462	A06091	246	A00462	A06523	447
A00462	A06632	229	A00462	A06700	285
A00462	A07061	473	A00462	A07122	468
A00462	A07238	154	A00462	A07376	174
A00462	A07413	408	A00462	A07424	201

A00462	A07800	489	A00462	A08127	416
A00462	A08463	200	A00462	A09612	363
A00462	A11309	479	A00462	A15497	446
A00462	A15637	241	A00462	A15874	385
A00462	A16033	420	A00462	A16529	396
A00462	A16739	358	A00462	A16741	405
A00462	A16744	364	A00463	A00475	477
A00463	A00479	486	A00463	A00517	541
A00463	A00566	826	A00463	A00572	398
A00463	A00575	714	A00463	A00622	706
A00463	A00626	423	A00463	A00665	419
A00463	A00708	406	A00463	A00733	532
A00463	A00744	294	A00463	A00805	541
A00463	A00819	446	A00463	A00822	299
A00463	A00856	480	A00463	A00858	853
A00463	A00860	714	A00463	A00866	156
A00463	A00881	533	A00463	A00934	435
A00463	A00978	487	A00463	A01007	648
A00463	A01019	530	A00463	A01020	445
A00463	A01032	198	A00463	A01075	457
A00463	A01116	301	A00463	A01147	286
A00463	A01195	538	A00463	A01200	708
A00463	A01206	348	A00463	A01212	447
A00463	A01234	491	A00463	A01325	294
A00463	A01383	460	A00463	A01385	355
A00463	A01398	538	A00463	A01401	714
A00463	A01500	539	A00463	A01503	416
A00463	A01720	440	A00463	A02872	195
A00463	A03175	182	A00463	A03207	246
A00463	A03306	249	A00463	A03526	466
A00463	A04648	522	A00463	A04687	156
A00463	A05027	427	A00463	A06803	539
A00463	A07203	156	A00463	A07290	196
A00463	A07347	481	A00463	A07670	391
A00463	A07700	470	A00463	A07850	156
A00463	A07887	443	A00463	A08022	486
A00463	A08383	311	A00463	A08818	156
A00463	A09502	156	A00463	A09712	486
A00463	A10642	416	A00463	A11344	156
A00463	A11395	469	A00463	A11674	156
A00463	A13413	279	A00463	A15946	183
A00463	A16228	458	A00464	A00600	232
A00464	A00651	313	A00464	A00742	245
A00464	A00763	470	A00464	A00966	259
A00464	A01011	374	A00464	A01054	450
A00464	A01160	450	A00464	A01168	499

A00464	A01209	227	A00464	A01294	450
A00464	A01361	196	A00464	A01419	177
A00464	A01576	356	A00464	A01722	172
A00464	A02165	515	A00464	A03615	404
A00464	A03652	171	A00464	A03782	450
A00464	A05411	399	A00464	A06520	229
A00464	A06591	414	A00464	A07069	422
A00464	A07447	443	A00464	A07495	337
A00464	A09044	271	A00464	A11487	218
A00464	A11929	185	A00464	A13052	450
A00464	A14086	204	A00464	A14843	431
A00464	A15666	404	A00464	A15701	176
A00464	A16681	404	A00465	A01223	188
A00465	A01225	205	A00465	A01409	480
A00465	A02254	193	A00465	A04095	288
A00465	A04791	235	A00465	A04883	193
A00465	A04927	196	A00465	A05007	193
A00465	A05014	267	A00465	A05140	294
A00465	A05330	246	A00465	A05337	240
A00465	A05425	162	A00465	A05433	353
A00465	A05451	307	A00465	A05550	215
A00465	A05573	193	A00465	A05912	361
A00465	A05966	199	A00465	A06358	190
A00465	A06629	180	A00465	A06641	247
A00465	A06734	286	A00465	A06847	270
A00465	A06994	293	A00465	A07007	395
A00465	A07027	243	A00465	A07059	180
A00465	A07408	302	A00465	A07501	228
A00465	A07507	416	A00465	A07590	183
A00465	A07619	329	A00465	A07674	363
A00465	A07737	267	A00465	A07847	350
A00465	A08135	262	A00465	A10658	281
A00465	A10702	168	A00465	A10706	241
A00465	A14821	302	A00465	A17006	276
A00466	A00687	457	A00466	A01453	221
A00466	A07141	249	A00466	A08933	383
A00466	A13490	417	A00466	A15624	279
A00466	A15920	274	A00466	A15932	313
A00467	A00494	474	A00467	A00539	273
A00467	A00550	249	A00467	A00590	306
A00467	A00603	162	A00467	A00605	346
A00467	A00648	366	A00467	A00651	483
A00467	A00694	205	A00467	A00700	491
A00467	A00705	293	A00467	A00708	232
A00467	A00782	224	A00467	A00787	369
A00467	A00806	419	A00467	A00809	241

A00467	A00839	574	A00467	A00877	251
A00467	A00972	262	A00467	A00980	241
A00467	A01000	196	A00467	A01016	190
A00467	A01056	457	A00467	A01066	216
A00467	A01072	303	A00467	A01090	447
A00467	A01101	165	A00467	A01102	282
A00467	A01112	491	A00467	A01127	486
A00467	A01140	483	A00467	A01166	204
A00467	A01177	232	A00467	A01196	571
A00467	A01212	154	A00467	A01218	241
A00467	A01234	272	A00467	A01238	182
A00467	A01249	358	A00467	A01270	465
A00467	A01288	157	A00467	A01291	491
A00467	A01306	401	A00467	A01335	455
A00467	A01369	217	A00467	A01385	273
A00467	A01411	373	A00467	A01464	176
A00467	A01468	486	A00467	A01470	256
A00467	A01496	479	A00467	A01579	587
A00467	A01586	224	A00467	A01597	187
A00467	A01601	588	A00467	A01605	347
A00467	A01718	419	A00467	A01720	240
A00467	A02350	245	A00467	A02494	174
A00467	A02737	217	A00467	A03445	491
A00467	A03451	162	A00467	A03567	480
A00467	A04099	156	A00467	A04101	196
A00467	A04230	256	A00467	A04827	258
A00467	A04846	190	A00467	A05090	252
A00467	A05443	400	A00467	A05453	157
A00467	A05501	542	A00467	A06534	582
A00467	A06814	578	A00467	A06861	174
A00467	A06965	480	A00467	A06973	570
A00467	A06974	167	A00467	A07047	241
A00467	A07323	425	A00467	A07398	588
A00467	A07451	243	A00467	A08131	434
A00467	A08357	221	A00467	A08359	404
A00467	A08369	484	A00467	A08408	241
A00467	A08476	241	A00467	A08558	176
A00467	A09308	420	A00467	A09322	334
A00467	A09620	410	A00467	A09741	243
A00467	A10599	254	A00467	A10616	157
A00467	A11334	215	A00467	A11438	213
A00467	A11550	358	A00467	A11728	492
A00467	A11857	185	A00467	A12227	307
A00467	A13340	268	A00467	A13989	489
A00467	A13992	491	A00467	A14172	489
A00467	A14908	353	A00467	A15268	587

A00467	A15381	586	A00467	A15551	358
A00467	A15988	317	A00467	A16272	323
A00467	A16306	202	A00467	A16318	283
A00467	A16658	165	A00467	A16682	188
A00467	A17133	174	A00468	A00660	475
A00468	A00681	372	A00468	A00696	439
A00468	A00825	475	A00468	A01018	400
A00468	A01225	277	A00468	A01227	441
A00468	A01297	469	A00468	A01397	278
A00468	A01409	338	A00468	A01490	416
A00468	A01643	311	A00468	A01793	201
A00468	A01832	168	A00468	A01958	263
A00468	A02020	159	A00468	A02114	323
A00468	A02254	484	A00468	A02257	353
A00468	A02284	337	A00468	A02521	198
A00468	A02525	162	A00468	A03087	490
A00468	A03174	369	A00468	A03513	483
A00468	A03968	380	A00468	A04533	219
A00468	A04674	296	A00468	A04927	267
A00468	A05014	210	A00468	A05045	154
A00468	A05098	165	A00468	A05102	152
A00468	A05208	210	A00468	A05224	269
A00468	A05330	460	A00468	A05331	375
A00468	A05337	435	A00468	A05338	163
A00468	A05358	201	A00468	A05424	378
A00468	A05516	174	A00468	A05548	224
A00468	A05550	154	A00468	A05573	480
A00468	A05758	151	A00468	A05820	313
A00468	A05883	205	A00468	A05912	324
A00468	A06405	468	A00468	A06439	395
A00468	A06640	313	A00468	A06641	399
A00468	A06733	362	A00468	A06847	213
A00468	A06860	416	A00468	A06994	240
A00468	A07027	318	A00468	A07037	235
A00468	A07059	405	A00468	A07088	461
A00468	A07238	218	A00468	A07283	267
A00468	A07408	251	A00468	A07501	168
A00468	A07520	490	A00468	A07674	443
A00468	A07684	191	A00468	A07690	472
A00468	A07711	235	A00468	A07723	219
A00468	A07763	313	A00468	A07800	238
A00468	A07844	411	A00468	A07847	162
A00468	A07851	283	A00468	A07856	269
A00468	A07933	474	A00468	A07976	436
A00468	A08114	160	A00468	A08190	328
A00468	A08197	213	A00468	A08199	343

A00468	A08278	157	A00468	A08280	396
A00468	A08366	396	A00468	A08372	341
A00468	A08385	435	A00468	A08663	212
A00468	A08687	241	A00468	A09845	157
A00468	A09846	408	A00468	A09880	241
A00468	A10446	191	A00468	A10456	260
A00468	A10537	361	A00468	A10652	205
A00468	A10679	447	A00468	A10687	254
A00468	A10690	240	A00468	A10691	391
A00468	A10699	413	A00468	A10715	341
A00468	A10778	228	A00468	A10781	351
A00468	A10783	254	A00468	A11235	219
A00468	A11708	296	A00468	A12723	246
A00468	A13341	379	A00468	A14094	369
A00468	A14803	270	A00468	A15874	208
A00468	A16026	177	A00468	A16739	183
A00468	A16744	191	A00468	A16757	339
A00468	A17046	430	A00468	A17092	324
A00469	A00470	210	A00469	A00476	176
A00469	A00505	408	A00469	A00571	456
A00469	A00580	466	A00469	A00593	491
A00469	A00597	232	A00469	A00626	396
A00469	A00661	193	A00469	A00705	486
A00469	A00782	288	A00469	A00839	491
A00469	A00899	154	A00469	A00923	274
A00469	A00955	193	A00469	A01000	348
A00469	A01017	491	A00469	A01037	473
A00469	A01041	490	A00469	A01046	390
A00469	A01101	466	A00469	A01189	328
A00469	A01214	286	A00469	A01263	451
A00469	A01278	475	A00469	A01291	491
A00469	A01306	490	A00469	A01309	318
A00469	A01335	491	A00469	A01337	491
A00469	A01365	327	A00469	A01369	491
A00469	A01422	327	A00469	A01462	330
A00469	A01527	483	A00469	A01540	238
A00469	A01579	491	A00469	A01588	171
A00469	A01595	251	A00469	A01647	180
A00469	A01741	196	A00469	A01839	378
A00469	A01897	398	A00469	A01913	170
A00469	A01992	191	A00469	A02072	324
A00469	A02106	346	A00469	A02143	63
A00469	A02159	243	A00469	A02227	422
A00469	A02262	159	A00469	A02278	335
A00469	A02416	63	A00469	A02418	183
A00469	A02424	268	A00469	A02494	374

A00469	A02495	481	A00469	A02522	167
A00469	A02570	243	A00469	A02584	176
A00469	A02592	361	A00469	A02614	235
A00469	A02695	339	A00469	A02743	238
A00469	A02768	324	A00469	A02796	162
A00469	A02820	171	A00469	A02877	465
A00469	A03047	188	A00469	A03069	154
A00469	A03477	221	A00469	A03512	201
A00469	A03567	385	A00469	A03681	196
A00469	A03839	151	A00469	A03981	263
A00469	A04216	167	A00469	A04218	165
A00469	A04265	151	A00469	A04332	346
A00469	A04399	228	A00469	A04416	463
A00469	A04419	380	A00469	A04470	277
A00469	A04472	221	A00469	A04476	323
A00469	A04511	294	A00469	A04512	263
A00469	A04579	283	A00469	A04677	277
A00469	A04740	179	A00469	A04838	305
A00469	A04970	294	A00469	A05258	198
A00469	A05302	310	A00469	A05335	201
A00469	A05497	263	A00469	A05526	323
A00469	A05623	263	A00469	A06057	243
A00469	A06066	277	A00469	A06742	252
A00469	A06849	263	A00469	A06851	154
A00469	A07047	286	A00469	A07075	252
A00469	A07163	404	A00469	A07262	243
A00469	A07264	333	A00469	A07334	235
A00469	A07412	249	A00469	A07581	357
A00469	A07775	217	A00469	A07880	326
A00469	A07944	207	A00469	A07965	488
A00469	A08131	312	A00469	A08358	491
A00469	A08359	383	A00469	A08462	210
A00469	A08479	217	A00469	A08576	167
A00469	A08981	440	A00469	A09083	183
A00469	A09088	323	A00469	A09285	217
A00469	A09330	327	A00469	A09369	252
A00469	A09389	294	A00469	A09446	243
A00469	A09471	188	A00469	A09475	294
A00469	A09613	380	A00469	A09726	159
A00469	A09808	390	A00469	A09999	427
A00469	A10039	430	A00469	A10106	294
A00469	A10138	277	A00469	A10385	323
A00469	A10525	171	A00469	A11099	474
A00469	A11115	294	A00469	A11135	423
A00469	A11189	255	A00469	A11211	217
A00469	A11316	447	A00469	A11429	332

A00469	A11440	486	A00469	A11480	490
A00469	A11576	332	A00469	A11704	473
A00469	A11729	486	A00469	A11857	279
A00469	A11935	329	A00469	A11945	200
A00469	A12027	294	A00469	A12077	294
A00469	A12079	361	A00469	A12251	151
A00469	A12503	344	A00469	A12821	484
A00469	A13622	401	A00469	A13710	347
A00469	A13734	294	A00469	A14190	177
A00469	A14278	455	A00469	A14498	447
A00469	A14508	455	A00469	A14643	321
A00469	A14701	188	A00469	A14737	294
A00469	A15286	290	A00469	A15500	475
A00469	A15717	229	A00469	A15727	338
A00469	A15748	321	A00469	A15759	221
A00469	A16682	151	A00469	A16718	154
A00469	A16911	191	A00469	A17026	221
A00469	A17107	151	A00469	A17112	191
A00470	A00561	174	A00470	A00637	276
A00470	A00722	899	A00470	A00832	154
A00470	A00887	461	A00470	A00921	899
A00470	A01052	418	A00470	A01149	472
A00470	A01172	491	A00470	A01390	391
A00470	A01450	188	A00470	A01483	278
A00470	A01548	491	A00470	A01562	217
A00470	A01602	477	A00470	A01728	208
A00470	A01844	932	A00470	A01907	177
A00470	A02219	229	A00470	A02470	430
A00470	A02494	467	A00470	A02687	491
A00470	A02859	899	A00470	A02874	899
A00470	A02877	201	A00470	A02908	899
A00470	A02968	899	A00470	A03237	457
A00470	A03271	235	A00470	A03643	198
A00470	A03802	202	A00470	A03958	201
A00470	A04176	174	A00470	A04957	310
A00470	A05216	307	A00470	A05797	439
A00470	A06234	251	A00470	A06610	489
A00470	A08627	486	A00470	A08863	276
A00470	A09169	174	A00470	A09504	162
A00470	A09826	491	A00470	A10174	899
A00470	A11376	293	A00470	A11843	276
A00470	A11851	418	A00470	A11983	899
A00470	A12035	350	A00470	A12266	394
A00470	A12630	212	A00470	A12681	218
A00470	A13356	276	A00470	A13462	265
A00470	A13604	245	A00470	A14002	899

A00470	A14016	484	A00470	A14234	339
A00470	A14496	196	A00470	A14639	185
A00470	A15292	899	A00470	A15312	899
A00470	A15480	276	A00470	A16215	180
A00470	A16718	489	A00470	A16887	377
A00471	A00532	405	A00471	A00566	151
A00471	A00575	168	A00471	A00600	254
A00471	A00622	170	A00471	A00665	312
A00471	A00733	368	A00471	A00765	409
A00471	A00784	229	A00471	A00805	332
A00471	A00858	391	A00471	A00860	170
A00471	A00866	438	A00471	A00883	694
A00471	A00954	460	A00471	A00978	201
A00471	A00999	229	A00471	A01052	328
A00471	A01099	229	A00471	A01114	229
A00471	A01169	408	A00471	A01187	229
A00471	A01195	249	A00471	A01197	229
A00471	A01200	154	A00471	A01203	372
A00471	A01212	910	A00471	A01315	229
A00471	A01319	229	A00471	A01329	853
A00471	A01415	229	A00471	A01438	290
A00471	A01627	438	A00471	A01647	495
A00471	A01680	229	A00471	A01682	677
A00471	A01712	534	A00471	A03149	182
A00471	A03207	277	A00471	A03327	229
A00471	A03408	484	A00471	A03809	408
A00471	A04379	246	A00471	A04648	160
A00471	A05216	366	A00471	A05690	229
A00471	A06032	208	A00471	A06130	229
A00471	A06381	229	A00471	A06750	438
A00471	A06803	168	A00471	A07054	524
A00471	A07242	253	A00471	A07353	229
A00471	A07355	259	A00471	A07495	270
A00471	A07670	682	A00471	A07920	330
A00471	A08495	286	A00471	A09712	243
A00471	A09857	157	A00471	A11068	154
A00471	A12284	616	A00471	A12824	383
A00471	A13892	489	A00471	A13950	260
A00471	A14253	378	A00471	A14266	485
A00471	A14825	274	A00471	A15324	329
A00471	A15502	450	A00471	A15837	229
A00471	A16228	223	A00471	A16626	229
A00471	A16643	229	A00472	A00477	486
A00472	A00485	491	A00472	A00486	461
A00472	A00531	246	A00472	A00532	249
A00472	A00576	168	A00472	A00600	303

A00472	A00625	602	A00472	A00656	274
A00472	A00662	183	A00472	A00663	439
A00472	A00675	327	A00472	A00694	294
A00472	A00719	477	A00472	A00752	154
A00472	A00802	306	A00472	A00849	467
A00472	A00855	246	A00472	A00864	455
A00472	A00882	485	A00472	A00985	229
A00472	A01005	201	A00472	A01011	491
A00472	A01022	490	A00472	A01125	213
A00472	A01156	483	A00472	A01161	588
A00472	A01169	362	A00472	A01211	477
A00472	A01235	221	A00472	A01276	486
A00472	A01278	174	A00472	A01332	468
A00472	A01381	363	A00472	A01431	458
A00472	A01454	486	A00472	A01461	230
A00472	A01515	263	A00472	A01524	407
A00472	A01526	491	A00472	A01529	229
A00472	A01530	479	A00472	A01543	477
A00472	A01551	440	A00472	A01556	491
A00472	A01557	491	A00472	A01566	409
A00472	A01578	491	A00472	A01582	491
A00472	A01585	301	A00472	A01600	429
A00472	A01627	486	A00472	A01630	491
A00472	A01632	491	A00472	A01676	177
A00472	A01689	215	A00472	A01743	483
A00472	A01749	353	A00472	A01931	475
A00472	A01935	199	A00472	A01947	167
A00472	A01956	185	A00472	A01982	491
A00472	A02007	438	A00472	A02010	278
A00472	A02021	314	A00472	A02048	491
A00472	A02049	677	A00472	A02092	173
A00472	A02099	185	A00472	A02146	104
A00472	A02148	485	A00472	A02154	238
A00472	A02167	154	A00472	A02183	489
A00472	A02186	425	A00472	A02188	491
A00472	A02205	452	A00472	A02215	491
A00472	A02221	491	A00472	A02222	455
A00472	A02224	221	A00472	A02228	491
A00472	A02283	306	A00472	A02303	326
A00472	A02320	266	A00472	A02333	485
A00472	A02356	251	A00472	A02402	235
A00472	A02477	490	A00472	A02485	357
A00472	A02511	361	A00472	A02642	165
A00472	A02691	162	A00472	A02697	356
A00472	A02698	401	A00472	A02738	490
A00472	A02756	188	A00472	A02859	488

A00472	A02874	462	A00472	A02909	427
A00472	A02924	334	A00472	A02951	283
A00472	A03034	425	A00472	A03050	484
A00472	A03067	491	A00472	A03080	491
A00472	A03090	213	A00472	A03104	489
A00472	A03168	152	A00472	A03178	341
A00472	A03186	409	A00472	A03211	477
A00472	A03259	489	A00472	A03293	313
A00472	A03297	414	A00472	A03314	291
A00472	A03321	350	A00472	A03344	483
A00472	A03345	486	A00472	A03346	489
A00472	A03347	491	A00472	A03348	474
A00472	A03349	491	A00472	A03350	473
A00472	A03351	491	A00472	A03353	491
A00472	A03356	486	A00472	A03357	422
A00472	A03358	491	A00472	A03359	201
A00472	A03360	270	A00472	A03361	491
A00472	A03363	457	A00472	A03364	177
A00472	A03365	199	A00472	A03366	396
A00472	A03368	486	A00472	A03369	491
A00472	A03370	491	A00472	A03371	491
A00472	A03373	491	A00472	A03374	445
A00472	A03375	371	A00472	A03376	491
A00472	A03378	456	A00472	A03379	491
A00472	A03383	283	A00472	A03384	481
A00472	A03385	355	A00472	A03387	348
A00472	A03388	327	A00472	A03389	199
A00472	A03397	362	A00472	A03402	277
A00472	A03417	411	A00472	A03440	255
A00472	A03496	196	A00472	A03532	490
A00472	A03545	371	A00472	A03569	308
A00472	A03573	207	A00472	A03708	446
A00472	A03711	294	A00472	A03724	324
A00472	A03729	191	A00472	A03909	283
A00472	A03910	446	A00472	A03984	486
A00472	A04033	430	A00472	A04051	286
A00472	A04079	335	A00472	A04202	193
A00472	A04275	480	A00472	A04329	256
A00472	A04337	491	A00472	A04349	257
A00472	A04361	490	A00472	A04385	235
A00472	A04409	286	A00472	A04410	491
A00472	A04449	597	A00472	A04493	303
A00472	A04518	183	A00472	A04520	491
A00472	A04532	398	A00472	A04567	184
A00472	A04603	340	A00472	A04710	303
A00472	A04715	491	A00472	A04764	177

A00472	A04790	488	A00472	A04832	441
A00472	A04833	469	A00472	A04840	207
A00472	A04870	405	A00472	A04928	477
A00472	A04944	302	A00472	A04964	473
A00472	A05015	491	A00472	A05080	199
A00472	A05106	180	A00472	A05117	297
A00472	A05148	160	A00472	A05155	665
A00472	A05157	216	A00472	A05234	399
A00472	A05286	199	A00472	A05294	445
A00472	A05340	306	A00472	A05356	736
A00472	A05381	160	A00472	A05426	160
A00472	A05431	497	A00472	A05439	301
A00472	A05487	274	A00472	A05489	369
A00472	A05494	340	A00472	A05556	379
A00472	A05561	235	A00472	A05587	198
A00472	A05601	488	A00472	A05603	491
A00472	A05612	243	A00472	A05643	485
A00472	A05657	294	A00472	A05716	207
A00472	A05731	470	A00472	A05739	483
A00472	A05777	445	A00472	A05813	177
A00472	A05885	294	A00472	A05886	489
A00472	A05887	490	A00472	A05890	330
A00472	A05891	490	A00472	A05968	223
A00472	A05970	281	A00472	A06069	491
A00472	A06070	314	A00472	A06096	235
A00472	A06145	235	A00472	A06148	279
A00472	A06177	235	A00472	A06322	199
A00472	A06346	491	A00472	A06428	466
A00472	A06483	235	A00472	A06569	187
A00472	A06574	488	A00472	A06617	380
A00472	A06650	249	A00472	A06711	270
A00472	A06726	235	A00472	A06729	491
A00472	A06730	380	A00472	A06731	491
A00472	A06732	330	A00472	A06907	406
A00472	A06920	462	A00472	A06921	235
A00472	A06952	294	A00472	A06953	491
A00472	A06954	439	A00472	A07004	176
A00472	A07044	335	A00472	A07064	177
A00472	A07065	380	A00472	A07066	347
A00472	A07068	199	A00472	A07209	486
A00472	A07218	491	A00472	A07242	205
A00472	A07268	177	A00472	A07286	428
A00472	A07361	491	A00472	A07384	475
A00472	A07391	486	A00472	A07392	235
A00472	A07396	160	A00472	A07442	491
A00472	A07452	191	A00472	A07471	491

A00472	A07472	371	A00472	A07475	414
A00472	A07514	473	A00472	A07541	475
A00472	A07561	373	A00472	A07577	485
A00472	A07578	391	A00472	A07579	235
A00472	A07580	428	A00472	A07601	212
A00472	A07605	491	A00472	A07606	160
A00472	A07620	446	A00472	A07623	160
A00472	A07635	193	A00472	A07676	235
A00472	A07702	199	A00472	A07704	484
A00472	A07705	463	A00472	A07754	481
A00472	A07790	235	A00472	A07810	330
A00472	A07827	199	A00472	A07828	177
A00472	A07843	488	A00472	A07904	441
A00472	A07905	330	A00472	A07906	235
A00472	A07907	310	A00472	A07913	288
A00472	A07927	235	A00472	A07949	235
A00472	A07958	438	A00472	A07982	177
A00472	A07990	235	A00472	A08033	235
A00472	A08034	132	A00472	A08039	281
A00472	A08040	489	A00472	A08041	235
A00472	A08042	391	A00472	A08044	294
A00472	A08059	310	A00472	A08093	491
A00472	A08097	466	A00472	A08115	160
A00472	A08157	260	A00472	A08162	260
A00472	A08171	439	A00472	A08172	490
A00472	A08202	281	A00472	A08203	458
A00472	A08210	179	A00472	A08225	199
A00472	A08229	235	A00472	A08244	270
A00472	A08247	177	A00472	A08270	235
A00472	A08271	235	A00472	A08276	235
A00472	A08288	160	A00472	A08291	270
A00472	A08292	436	A00472	A08298	235
A00472	A08309	490	A00472	A08392	177
A00472	A08404	384	A00472	A08459	330
A00472	A08594	477	A00472	A08630	184
A00472	A08658	324	A00472	A08671	360
A00472	A08681	177	A00472	A08685	481
A00472	A08686	470	A00472	A08693	232
A00472	A08738	360	A00472	A08747	281
A00472	A08753	313	A00472	A08754	199
A00472	A08785	485	A00472	A08835	345
A00472	A08859	443	A00472	A08949	293
A00472	A09139	294	A00472	A09140	199
A00472	A09173	177	A00472	A09204	281
A00472	A09205	283	A00472	A09341	230
A00472	A09380	259	A00472	A09416	436

A00472	A09511	159	A00472	A09525	199
A00472	A09552	260	A00472	A09745	174
A00472	A09807	199	A00472	A09886	199
A00472	A10001	202	A00472	A10095	193
A00472	A10101	199	A00472	A10156	205
A00472	A10450	281	A00472	A10511	119
A00472	A10532	422	A00472	A10561	290
A00472	A10563	230	A00472	A10649	199
A00472	A10651	329	A00472	A10900	199
A00472	A11049	235	A00472	A11154	199
A00472	A11164	475	A00472	A11172	371
A00472	A11240	165	A00472	A11278	350
A00472	A11289	436	A00472	A11299	352
A00472	A11305	428	A00472	A11313	395
A00472	A11317	157	A00472	A11319	475
A00472	A11327	481	A00472	A11340	391
A00472	A11377	270	A00472	A11391	212
A00472	A11411	159	A00472	A11428	319
A00472	A11448	199	A00472	A11449	297
A00472	A11454	180	A00472	A11468	160
A00472	A11513	179	A00472	A11552	388
A00472	A11557	391	A00472	A11573	193
A00472	A11599	199	A00472	A11664	235
A00472	A11671	235	A00472	A11723	199
A00472	A11888	235	A00472	A11963	235
A00472	A11983	484	A00472	A11997	235
A00472	A12031	177	A00472	A12194	235
A00472	A12317	235	A00472	A12365	408
A00472	A12426	310	A00472	A12459	270
A00472	A12480	235	A00472	A12493	177
A00472	A12549	235	A00472	A12572	235
A00472	A12619	330	A00472	A12620	235
A00472	A12642	235	A00472	A12649	177
A00472	A12689	235	A00472	A12787	462
A00472	A12804	235	A00472	A12845	229
A00472	A12876	330	A00472	A12897	221
A00472	A12906	235	A00472	A12928	235
A00472	A12955	199	A00472	A13035	235
A00472	A13110	160	A00472	A13120	199
A00472	A13351	281	A00472	A13361	269
A00472	A13369	678	A00472	A13760	235
A00472	A13853	198	A00472	A13855	305
A00472	A13960	217	A00472	A14202	432
A00472	A14204	477	A00472	A14247	179
A00472	A14279	152	A00472	A14353	477
A00472	A14377	369	A00472	A14381	484

A00472	A14394	416	A00472	A14395	297
A00472	A14523	162	A00472	A14570	446
A00472	A14633	491	A00472	A14689	200
A00472	A14901	281	A00472	A14918	196
A00472	A15178	235	A00472	A15232	235
A00472	A15267	176	A00472	A15277	480
A00472	A15282	400	A00472	A15292	484
A00472	A15296	259	A00472	A15301	201
A00472	A15322	586	A00472	A15333	151
A00472	A15336	474	A00472	A15398	384
A00472	A15399	380	A00472	A15416	215
A00472	A15422	283	A00472	A15432	419
A00472	A15433	172	A00472	A15451	246
A00472	A15452	205	A00472	A15457	360
A00472	A15495	486	A00472	A15498	491
A00472	A15552	290	A00472	A15638	491
A00472	A15665	168	A00472	A15678	294
A00472	A15880	207	A00472	A15911	162
A00472	A15917	491	A00472	A16005	235
A00472	A16117	173	A00472	A16121	200
A00472	A16147	177	A00472	A16274	188
A00472	A16495	223	A00472	A16626	212
A00472	A17078	486	A00472	A17085	462
A00472	A17124	177	A00473	A00476	134
A00473	A00504	210	A00473	A00519	572
A00473	A00524	485	A00473	A00526	573
A00473	A00531	423	A00473	A00556	152
A00473	A00571	302	A00473	A00638	238
A00473	A00658	577	A00473	A00687	295
A00473	A00715	223	A00473	A00747	412
A00473	A00871	578	A00473	A00886	240
A00473	A00918	240	A00473	A00986	559
A00473	A01009	152	A00473	A01111	223
A00473	A01117	176	A00473	A01118	381
A00473	A01124	63	A00473	A01156	391
A00473	A01202	323	A00473	A01208	575
A00473	A01214	439	A00473	A01215	578
A00473	A01228	263	A00473	A01257	156
A00473	A01325	337	A00473	A01328	207
A00473	A01353	578	A00473	A01367	406
A00473	A01431	188	A00473	A01547	154
A00473	A01736	303	A00473	A01797	170
A00473	A01848	212	A00473	A01890	154
A00473	A01975	187	A00473	A02011	82
A00473	A02028	193	A00473	A02041	223
A00473	A02051	223	A00473	A02061	257

A00473	A02063	165	A00473	A02117	252
A00473	A02191	355	A00473	A02214	167
A00473	A02251	223	A00473	A02260	430
A00473	A02268	469	A00473	A02358	174
A00473	A02454	154	A00473	A02470	171
A00473	A02531	174	A00473	A02537	171
A00473	A02539	308	A00473	A02634	157
A00473	A02655	200	A00473	A02672	168
A00473	A02673	490	A00473	A02830	288
A00473	A02958	207	A00473	A03043	151
A00473	A03044	191	A00473	A03045	405
A00473	A03057	479	A00473	A03091	123
A00473	A03154	202	A00473	A03155	276
A00473	A03169	530	A00473	A03193	489
A00473	A03223	290	A00473	A03276	179
A00473	A03792	249	A00473	A03820	291
A00473	A03958	411	A00473	A03972	281
A00473	A03982	281	A00473	A04001	224
A00473	A04447	421	A00473	A04468	235
A00473	A04911	185	A00473	A05058	460
A00473	A05143	200	A00473	A05210	185
A00473	A05483	303	A00473	A05577	255
A00473	A05689	281	A00473	A05748	383
A00473	A06137	157	A00473	A06787	291
A00473	A06822	303	A00473	A06825	260
A00473	A07062	147	A00473	A07140	210
A00473	A07165	205	A00473	A07265	407
A00473	A07588	532	A00473	A07655	172
A00473	A07695	154	A00473	A07799	218
A00473	A08739	176	A00473	A08955	223
A00473	A08971	223	A00473	A09268	291
A00473	A09753	154	A00473	A09814	218
A00473	A09977	170	A00473	A10033	303
A00473	A10362	193	A00473	A10755	223
A00473	A10855	345	A00473	A11202	223
A00473	A11254	355	A00473	A13298	230
A00473	A13870	288	A00473	A13886	191
A00473	A14153	154	A00473	A14216	565
A00473	A14554	420	A00473	A14782	210
A00473	A15641	274	A00473	A15789	369
A00473	A16038	281	A00473	A16118	174
A00474	A00505	268	A00474	A00593	227
A00474	A00599	243	A00474	A00705	199
A00474	A00711	193	A00474	A00839	198
A00474	A00971	251	A00474	A01072	262
A00474	A01121	232	A00474	A01125	409

A00474	A01154	228	A00474	A01159	218
A00474	A01217	469	A00474	A01362	215
A00474	A01408	198	A00474	A01414	249
A00474	A01586	266	A00474	A03391	434
A00474	A03476	279	A00474	A03601	293
A00474	A05804	294	A00474	A06122	258
A00474	A06369	210	A00474	A06436	317
A00474	A06785	268	A00474	A06889	267
A00474	A07934	488	A00474	A09470	235
A00474	A09671	246	A00474	A10987	422
A00474	A11707	252	A00474	A13965	190
A00474	A14479	180	A00474	A15657	208
A00475	A00479	430	A00475	A00517	462
A00475	A00566	408	A00475	A00575	463
A00475	A00622	430	A00475	A00626	398
A00475	A00655	318	A00475	A00733	398
A00475	A00805	438	A00475	A00817	159
A00475	A00819	163	A00475	A00858	463
A00475	A00860	430	A00475	A00881	274
A00475	A00978	391	A00475	A01019	353
A00475	A01195	463	A00475	A01200	454
A00475	A01212	373	A00475	A01320	256
A00475	A01383	247	A00475	A01398	479
A00475	A01401	480	A00475	A01500	283
A00475	A01741	162	A00475	A01888	152
A00475	A02040	229	A00475	A03525	199
A00475	A03526	273	A00475	A03734	185
A00475	A04648	367	A00475	A06803	470
A00475	A07290	303	A00475	A07887	210
A00475	A08938	364	A00475	A09676	424
A00475	A09712	411	A00475	A10642	256
A00475	A10759	358	A00475	A14132	232
A00475	A14801	180	A00475	A15318	188
A00475	A15365	269	A00475	A15649	358
A00475	A15819	263	A00475	A16228	446
A00476	A00478	216	A00476	A00509	271
A00476	A00526	190	A00476	A00645	306
A00476	A00659	477	A00476	A00736	243
A00476	A00752	327	A00476	A01009	455
A00476	A01046	485	A00476	A01165	162
A00476	A01215	63	A00476	A01256	154
A00476	A01301	180	A00476	A01313	168
A00476	A01489	156	A00476	A01553	247
A00476	A01604	311	A00476	A01640	367
A00476	A01743	272	A00476	A01795	347
A00476	A01870	413	A00476	A02031	446

A00476	A02121	159	A00476	A02122	168
A00476	A02138	187	A00476	A02143	163
A00476	A02242	232	A00476	A02251	404
A00476	A02260	185	A00476	A02268	80
A00476	A02272	202	A00476	A02278	380
A00476	A02281	422	A00476	A02289	405
A00476	A02331	200	A00476	A02439	199
A00476	A02454	79	A00476	A02474	332
A00476	A02488	407	A00476	A02493	218
A00476	A02512	226	A00476	A02575	382
A00476	A02579	457	A00476	A02606	196
A00476	A02624	395	A00476	A02651	182
A00476	A02654	396	A00476	A02727	324
A00476	A02737	167	A00476	A02766	202
A00476	A02802	170	A00476	A02831	229
A00476	A02859	201	A00476	A02885	288
A00476	A03033	160	A00476	A03043	302
A00476	A03045	484	A00476	A03144	388
A00476	A03218	180	A00476	A03224	446
A00476	A03239	396	A00476	A03309	265
A00476	A03431	198	A00476	A03486	163
A00476	A03570	204	A00476	A03688	151
A00476	A03690	294	A00476	A03790	168
A00476	A03827	260	A00476	A03839	201
A00476	A03898	332	A00476	A03990	151
A00476	A04073	281	A00476	A04076	241
A00476	A04191	185	A00476	A04211	221
A00476	A04273	281	A00476	A04428	247
A00476	A04567	162	A00476	A04755	229
A00476	A04784	265	A00476	A05597	177
A00476	A05645	191	A00476	A05679	302
A00476	A05738	159	A00476	A06009	185
A00476	A06414	200	A00476	A06415	347
A00476	A06644	157	A00476	A07145	396
A00476	A07655	190	A00476	A08275	355
A00476	A08612	281	A00476	A08651	281
A00476	A08818	157	A00476	A09539	160
A00476	A09826	152	A00476	A10556	216
A00476	A10640	328	A00476	A10847	247
A00476	A11318	201	A00476	A11440	217
A00476	A11651	156	A00476	A11734	491
A00476	A12009	182	A00476	A12251	443
A00476	A13222	163	A00476	A13935	227
A00476	A13957	306	A00476	A14136	281
A00476	A14146	177	A00476	A14199	306
A00476	A14335	168	A00476	A14365	355

A00476	A14366	418	A00476	A14374	427
A00476	A14391	344	A00476	A14419	306
A00476	A14502	485	A00476	A14554	430
A00476	A14599	154	A00476	A15285	466
A00476	A15425	274	A00476	A15516	263
A00476	A15518	353	A00476	A15660	360
A00476	A15770	241	A00476	A15795	401
A00476	A15811	210	A00476	A15827	229
A00476	A15964	174	A00476	A15996	270
A00476	A16014	306	A00476	A16218	461
A00476	A16532	265	A00476	A16939	185
A00477	A00485	319	A00477	A00671	240
A00477	A00675	167	A00477	A00694	170
A00477	A00743	296	A00477	A00882	480
A00477	A01273	308	A00477	A01332	490
A00477	A01526	257	A00477	A01565	490
A00477	A01578	212	A00477	A01931	170
A00477	A02099	388	A00477	A02388	270
A00477	A02494	170	A00477	A02544	177
A00477	A02585	195	A00477	A02586	488
A00477	A02601	157	A00477	A02637	162
A00477	A02818	227	A00477	A02859	251
A00477	A02909	252	A00477	A03010	170
A00477	A03104	452	A00477	A03136	252
A00477	A03228	401	A00477	A03244	263
A00477	A03314	371	A00477	A03349	162
A00477	A03358	177	A00477	A03417	170
A00477	A03806	371	A00477	A03923	229
A00477	A04034	319	A00477	A04072	230
A00477	A04108	337	A00477	A04228	191
A00477	A04252	480	A00477	A04273	414
A00477	A04275	441	A00477	A04449	470
A00477	A04458	348	A00477	A04985	266
A00477	A05174	246	A00477	A05556	407
A00477	A05594	339	A00477	A05601	491
A00477	A05767	488	A00477	A05782	224
A00477	A05865	481	A00477	A06069	201
A00477	A06100	318	A00477	A06298	183
A00477	A06414	246	A00477	A06415	269
A00477	A06530	296	A00477	A07178	221
A00477	A07442	205	A00477	A07903	200
A00477	A08093	490	A00477	A08097	402
A00477	A08171	205	A00477	A08596	221
A00477	A08690	296	A00477	A08708	339
A00477	A08859	345	A00477	A09164	432
A00477	A09354	361	A00477	A09454	290

A00477	A09745	160	A00477	A09926	301
A00477	A10024	484	A00477	A10651	202
A00477	A11299	374	A00477	A11454	446
A00477	A11732	279	A00477	A11888	380
A00477	A11982	485	A00477	A11983	219
A00477	A12689	380	A00477	A12804	380
A00477	A13587	200	A00477	A13945	205
A00477	A14005	157	A00477	A14071	396
A00477	A14202	168	A00477	A14375	291
A00477	A14570	160	A00477	A14611	208
A00477	A14893	245	A00477	A15282	352
A00477	A15292	193	A00477	A15394	170
A00477	A15495	270	A00477	A15642	176
A00478	A00659	491	A00478	A00662	344
A00478	A00688	262	A00478	A01005	469
A00478	A01257	257	A00478	A01297	306
A00478	A01461	474	A00478	A01573	283
A00478	A01635	230	A00478	A01737	455
A00478	A01741	218	A00478	A01846	369
A00478	A01848	271	A00478	A01861	262
A00478	A01864	224	A00478	A01926	491
A00478	A01990	286	A00478	A02008	491
A00478	A02009	311	A00478	A02011	491
A00478	A02012	490	A00478	A02013	358
A00478	A02014	488	A00478	A02015	294
A00478	A02028	273	A00478	A02031	419
A00478	A02092	262	A00478	A02114	474
A00478	A02119	172	A00478	A02156	221
A00478	A02268	238	A00478	A02275	299
A00478	A02405	170	A00478	A02454	235
A00478	A02468	393	A00478	A02524	180
A00478	A02574	489	A00478	A02605	213
A00478	A02646	258	A00478	A02695	468
A00478	A02802	215	A00478	A02804	160
A00478	A02806	465	A00478	A02859	360
A00478	A02951	215	A00478	A02992	267
A00478	A03003	185	A00478	A03007	154
A00478	A03034	491	A00478	A03036	491
A00478	A03045	258	A00478	A03058	378
A00478	A03091	316	A00478	A03099	418
A00478	A03144	491	A00478	A03146	483
A00478	A03147	416	A00478	A03148	480
A00478	A03156	488	A00478	A03157	362
A00478	A03238	445	A00478	A03370	174
A00478	A03513	404	A00478	A03914	182
A00478	A04212	467	A00478	A04286	208

A00478	A04341	358	A00478	A04487	210
A00478	A04520	198	A00478	A04839	232
A00478	A05203	294	A00478	A05246	302
A00478	A05376	460	A00478	A05393	350
A00478	A05459	294	A00478	A05570	210
A00478	A06052	347	A00478	A06053	306
A00478	A06195	457	A00478	A06831	294
A00478	A06970	188	A00478	A07059	286
A00478	A07195	322	A00478	A07342	491
A00478	A07373	366	A00478	A07407	346
A00478	A07477	296	A00478	A07736	480
A00478	A08724	191	A00478	A09267	337
A00478	A09991	428	A00478	A10052	262
A00478	A10091	258	A00478	A10816	281
A00478	A11076	378	A00478	A11279	369
A00478	A12793	165	A00478	A13004	378
A00478	A13153	358	A00478	A14096	180
A00478	A14135	180	A00478	A14146	223
A00478	A14247	465	A00478	A14258	162
A00478	A14344	486	A00478	A15366	313
A00478	A15463	223	A00478	A15482	207
A00478	A15874	252	A00478	A16099	221
A00478	A16337	358	A00478	A16741	183
A00479	A00517	481	A00479	A00566	425
A00479	A00572	466	A00479	A00575	477
A00479	A00622	417	A00479	A00626	385
A00479	A00655	317	A00479	A00805	469
A00479	A00817	245	A00479	A00819	477
A00479	A00822	458	A00479	A00858	481
A00479	A00860	488	A00479	A00881	480
A00479	A00978	428	A00479	A01019	406
A00479	A01147	401	A00479	A01195	485
A00479	A01200	486	A00479	A01325	440
A00479	A01383	483	A00479	A01385	466
A00479	A01401	488	A00479	A03525	281
A00479	A03526	485	A00479	A04548	338
A00479	A04648	413	A00479	A06803	486
A00479	A07290	360	A00479	A09712	489
A00479	A10642	470	A00479	A16228	434
A00480	A00555	414	A00480	A00570	439
A00480	A00687	348	A00480	A00717	399
A00480	A00727	408	A00480	A00772	477
A00480	A00865	213	A00480	A00928	335
A00480	A00952	356	A00480	A01107	291
A00480	A01189	157	A00480	A01221	176
A00480	A01243	157	A00480	A01352	204

A00480	A02877	154	A00480	A07312	299
A00480	A13117	483	A00480	A14482	217
A00480	A14621	219	A00480	A15377	481
A00481	A00495	236	A00481	A00531	235
A00481	A00532	491	A00481	A00571	266
A00481	A00597	223	A00481	A00603	283
A00481	A00606	257	A00481	A00691	472
A00481	A00693	457	A00481	A00760	438
A00481	A00762	584	A00481	A00793	403
A00481	A00826	262	A00481	A00854	249
A00481	A00871	474	A00481	A00916	274
A00481	A00919	457	A00481	A00947	489
A00481	A00965	224	A00481	A00977	324
A00481	A00981	240	A00481	A01024	377
A00481	A01074	373	A00481	A01075	491
A00481	A01082	480	A00481	A01084	460
A00481	A01087	491	A00481	A01088	483
A00481	A01154	246	A00481	A01166	288
A00481	A01214	182	A00481	A01217	270
A00481	A01226	174	A00481	A01234	470
A00481	A01238	286	A00481	A01252	281
A00481	A01304	417	A00481	A01306	256
A00481	A01352	465	A00481	A01354	270
A00481	A01365	478	A00481	A01406	616
A00481	A01416	246	A00481	A01707	385
A00481	A01781	168	A00481	A03601	626
A00481	A03958	218	A00481	A05501	293
A00481	A07290	183	A00481	A07700	200
A00481	A07706	491	A00481	A08144	176
A00481	A08269	213	A00481	A08304	438
A00481	A08527	382	A00481	A08594	451
A00481	A08925	177	A00481	A08971	187
A00481	A09059	165	A00481	A09608	409
A00481	A10538	183	A00481	A11291	165
A00481	A11452	281	A00481	A12124	385
A00481	A13069	221	A00481	A13706	207
A00481	A13732	396	A00481	A13870	217
A00481	A13905	229	A00481	A13989	205
A00481	A14006	477	A00481	A14220	204
A00481	A14451	369	A00481	A14566	490
A00481	A14594	480	A00481	A15667	180
A00481	A15774	159	A00481	A16704	157
A00481	A16750	299	A00481	A17125	205
A00482	A00531	207	A00482	A00560	488
A00482	A00722	373	A00482	A00764	199
A00482	A00765	160	A00482	A00853	196

A00482	A00857	473	A00482	A01003	488
A00482	A01011	672	A00482	A01093	235
A00482	A01136	251	A00482	A01227	199
A00482	A01237	477	A00482	A01280	491
A00482	A01348	486	A00482	A01478	491
A00482	A01572	199	A00482	A01741	475
A00482	A01839	294	A00482	A01997	195
A00482	A02179	159	A00482	A02208	460
A00482	A02837	305	A00482	A03070	491
A00482	A03112	318	A00482	A03575	168
A00482	A03696	157	A00482	A03703	420
A00482	A03704	490	A00482	A03719	162
A00482	A03721	283	A00482	A03727	443
A00482	A03852	305	A00482	A04033	174
A00482	A04067	458	A00482	A04175	462
A00482	A04622	268	A00482	A04715	165
A00482	A04861	423	A00482	A05009	256
A00482	A05220	467	A00482	A05853	483
A00482	A06001	491	A00482	A06082	199
A00482	A06280	491	A00482	A06314	221
A00482	A06488	490	A00482	A06502	489
A00482	A07004	462	A00482	A07229	152
A00482	A07427	135	A00482	A08133	199
A00482	A08842	491	A00482	A08858	279
A00482	A08946	172	A00482	A09226	200
A00482	A09714	184	A00482	A09740	254
A00482	A09741	441	A00482	A09775	263
A00482	A09780	490	A00482	A09825	249
A00482	A10175	272	A00482	A10395	339
A00482	A11162	401	A00482	A11227	162
A00482	A11277	232	A00482	A11283	174
A00482	A11431	159	A00482	A11544	452
A00482	A11560	207	A00482	A11653	232
A00482	A11774	274	A00482	A11952	221
A00482	A12669	221	A00482	A12851	362
A00482	A12884	210	A00482	A12895	396
A00482	A12904	236	A00482	A13207	221
A00482	A13338	335	A00482	A13422	221
A00482	A13466	480	A00482	A13924	165
A00482	A13987	180	A00482	A14017	470
A00482	A14018	484	A00482	A14041	406
A00482	A14049	424	A00482	A14050	200
A00482	A14059	221	A00482	A14118	466
A00482	A14202	293	A00482	A14279	267
A00482	A14436	172	A00482	A14570	490
A00482	A14644	475	A00482	A14809	199

A00482	A15187	179	A00482	A15292	380
A00482	A15419	296	A00482	A15431	473
A00482	A15445	338	A00482	A15461	455
A00482	A15487	411	A00482	A15506	152
A00482	A15800	163	A00482	A15810	184
A00482	A16075	454	A00482	A16687	177
A00482	A16768	312	A00482	A16806	172
A00482	A17196	184	A00483	A00548	241
A00483	A00591	490	A00483	A00688	241
A00483	A00929	419	A00483	A01004	588
A00483	A01183	486	A00483	A01370	218
A00483	A01386	224	A00483	A01417	586
A00483	A01661	588	A00483	A01743	177
A00483	A01970	405	A00483	A01997	460
A00483	A02416	241	A00483	A02859	168
A00483	A02936	212	A00483	A02983	235
A00483	A03006	159	A00483	A03079	268
A00483	A03170	443	A00483	A03259	249
A00483	A03714	286	A00483	A03724	491
A00483	A03731	427	A00483	A03732	488
A00483	A03748	256	A00483	A03887	357
A00483	A03899	491	A00483	A03900	210
A00483	A03945	391	A00483	A04676	216
A00483	A04818	180	A00483	A04928	179
A00483	A05778	362	A00483	A06620	172
A00483	A06828	445	A00483	A06899	229
A00483	A07422	899	A00483	A07442	71
A00483	A07479	160	A00483	A08209	159
A00483	A08639	455	A00483	A08683	486
A00483	A08746	491	A00483	A08861	241
A00483	A08866	484	A00483	A09205	491
A00483	A09206	479	A00483	A09230	281
A00483	A09233	246	A00483	A09254	174
A00483	A11326	168	A00483	A11383	447
A00483	A11422	449	A00483	A12137	291
A00483	A12884	306	A00483	A12897	162
A00483	A12975	281	A00483	A13338	290
A00483	A13826	411	A00483	A13935	454
A00483	A13941	185	A00483	A13987	414
A00483	A14122	455	A00483	A14124	434
A00483	A14299	241	A00483	A14378	235
A00483	A14408	241	A00483	A14439	152
A00483	A14511	252	A00483	A14598	208
A00483	A15113	362	A00483	A15300	945
A00483	A15312	273	A00483	A15318	457
A00483	A15349	241	A00483	A15352	265

A00483	A15391	308	A00483	A15419	205
A00483	A15422	486	A00483	A15459	180
A00483	A15799	191	A00483	A15896	262
A00483	A16117	489	A00483	A16473	260
A00483	A17196	286	A00484	A01175	180
A00484	A01485	432	A00484	A01599	491
A00484	A01958	218	A00484	A02969	255
A00484	A03110	272	A00484	A03858	180
A00484	A03942	188	A00484	A03955	473
A00484	A04423	401	A00484	A05045	238
A00484	A05706	196	A00484	A06439	191
A00484	A06641	385	A00484	A06943	491
A00484	A07173	191	A00484	A08330	243
A00484	A09784	477	A00484	A09787	435
A00484	A09846	285	A00484	A14210	375
A00484	A14371	228	A00484	A14598	286
A00484	A15308	162	A00484	A16285	324
A00485	A00531	157	A00485	A00532	293
A00485	A00576	447	A00485	A00587	416
A00485	A00600	491	A00485	A00601	480
A00485	A00609	484	A00485	A00625	337
A00485	A00663	185	A00485	A00675	475
A00485	A00694	199	A00485	A00719	490
A00485	A00743	363	A00485	A00799	291
A00485	A00802	484	A00485	A00849	395
A00485	A00882	491	A00485	A00911	484
A00485	A00936	338	A00485	A00939	433
A00485	A00981	196	A00485	A00985	156
A00485	A01004	360	A00485	A01022	491
A00485	A01026	208	A00485	A01049	481
A00485	A01100	443	A00485	A01102	196
A00485	A01156	472	A00485	A01161	491
A00485	A01211	491	A00485	A01260	263
A00485	A01276	283	A00485	A01326	393
A00485	A01332	235	A00485	A01343	366
A00485	A01438	205	A00485	A01451	491
A00485	A01458	196	A00485	A01461	452
A00485	A01481	489	A00485	A01483	185
A00485	A01524	224	A00485	A01530	334
A00485	A01551	319	A00485	A01556	489
A00485	A01559	167	A00485	A01578	486
A00485	A01582	480	A00485	A01585	488
A00485	A01627	377	A00485	A01632	483
A00485	A01634	357	A00485	A01660	163
A00485	A01758	159	A00485	A01763	216
A00485	A01855	286	A00485	A01895	154

A00485	A01909	249	A00485	A01910	204
A00485	A01911	152	A00485	A01914	163
A00485	A01931	393	A00485	A01935	285
A00485	A02046	326	A00485	A02148	485
A00485	A02156	160	A00485	A02186	364
A00485	A02188	263	A00485	A02198	188
A00485	A02205	172	A00485	A02215	491
A00485	A02221	404	A00485	A02222	481
A00485	A02253	223	A00485	A02283	393
A00485	A02289	263	A00485	A02320	263
A00485	A02330	196	A00485	A02333	462
A00485	A02338	329	A00485	A02417	185
A00485	A02477	491	A00485	A02485	427
A00485	A02491	235	A00485	A02565	154
A00485	A02633	230	A00485	A02661	157
A00485	A02668	221	A00485	A02831	167
A00485	A02924	226	A00485	A03020	313
A00485	A03050	433	A00485	A03079	273
A00485	A03095	205	A00485	A03104	486
A00485	A03165	368	A00485	A03230	204
A00485	A03293	491	A00485	A03304	362
A00485	A03310	159	A00485	A03349	154
A00485	A03351	416	A00485	A03367	241
A00485	A03373	154	A00485	A03376	196
A00485	A03417	470	A00485	A03440	156
A00485	A03477	420	A00485	A03562	375
A00485	A03608	152	A00485	A03650	364
A00485	A03708	228	A00485	A03729	326
A00485	A03748	286	A00485	A03819	163
A00485	A03866	185	A00485	A03910	488
A00485	A03935	199	A00485	A03945	463
A00485	A04206	154	A00485	A04252	302
A00485	A04275	447	A00485	A04304	308
A00485	A04337	491	A00485	A04361	329
A00485	A04410	483	A00485	A04412	223
A00485	A04424	395	A00485	A04429	294
A00485	A04443	154	A00485	A04449	474
A00485	A04453	151	A00485	A04493	188
A00485	A04518	296	A00485	A04520	475
A00485	A04567	321	A00485	A04576	216
A00485	A04620	163	A00485	A04630	193
A00485	A04668	348	A00485	A04713	168
A00485	A04739	156	A00485	A04987	319
A00485	A05003	266	A00485	A05155	450
A00485	A05189	285	A00485	A05234	491
A00485	A05272	313	A00485	A05291	185

A00485	A05356	491	A00485	A05439	489
A00485	A05469	230	A00485	A05494	171
A00485	A05587	174	A00485	A05601	473
A00485	A05603	172	A00485	A05678	328
A00485	A05777	327	A00485	A05812	268
A00485	A05855	425	A00485	A05891	485
A00485	A06038	279	A00485	A06058	210
A00485	A06092	302	A00485	A06172	199
A00485	A06225	254	A00485	A06277	402
A00485	A06359	238	A00485	A06617	373
A00485	A06729	445	A00485	A06731	475
A00485	A06952	252	A00485	A07054	428
A00485	A07069	462	A00485	A07106	167
A00485	A07209	469	A00485	A07218	311
A00485	A07242	246	A00485	A07273	204
A00485	A07275	252	A00485	A07286	252
A00485	A07292	193	A00485	A07310	266
A00485	A07442	491	A00485	A07452	163
A00485	A07471	488	A00485	A07472	199
A00485	A07475	273	A00485	A07479	374
A00485	A07496	204	A00485	A07505	152
A00485	A07541	351	A00485	A07605	452
A00485	A07636	302	A00485	A07754	480
A00485	A07958	407	A00485	A08097	488
A00485	A08309	454	A00485	A08342	266
A00485	A08451	313	A00485	A08686	451
A00485	A08747	241	A00485	A08785	474
A00485	A08936	351	A00485	A09204	452
A00485	A09235	156	A00485	A09246	474
A00485	A09416	489	A00485	A09454	185
A00485	A09518	241	A00485	A09742	63
A00485	A09745	152	A00485	A09800	313
A00485	A09939	168	A00485	A10024	167
A00485	A10156	472	A00485	A10594	160
A00485	A10622	210	A00485	A10651	380
A00485	A10658	152	A00485	A10790	167
A00485	A10832	266	A00485	A10954	351
A00485	A11230	313	A00485	A11280	456
A00485	A11308	172	A00485	A11311	312
A00485	A11348	235	A00485	A11411	369
A00485	A11428	190	A00485	A11449	172
A00485	A11513	257	A00485	A11523	179
A00485	A11561	172	A00485	A11573	172
A00485	A11977	252	A00485	A11982	190
A00485	A11983	404	A00485	A12212	223
A00485	A12265	364	A00485	A12298	230

A00485	A12614	395	A00485	A12633	193
A00485	A12674	223	A00485	A12721	285
A00485	A12787	473	A00485	A12887	210
A00485	A13361	347	A00485	A13369	491
A00485	A13617	424	A00485	A13874	188
A00485	A13879	160	A00485	A13960	190
A00485	A13987	179	A00485	A14196	187
A00485	A14202	252	A00485	A14209	243
A00485	A14213	534	A00485	A14247	224
A00485	A14272	477	A00485	A14287	210
A00485	A14375	251	A00485	A14381	491
A00485	A14392	195	A00485	A14405	251
A00485	A14498	417	A00485	A14589	316
A00485	A15282	461	A00485	A15292	472
A00485	A15311	84	A00485	A15312	191
A00485	A15313	63	A00485	A15318	176
A00485	A15352	118	A00485	A15422	486
A00485	A15431	63	A00485	A15432	465
A00485	A15433	98	A00485	A15435	68
A00485	A15447	101	A00485	A15451	360
A00485	A15452	63	A00485	A15456	224
A00485	A15457	160	A00485	A15460	127
A00485	A15498	486	A00485	A15809	245
A00485	A15913	232	A00485	A15917	489
A00485	A15922	419	A00485	A16143	167
A00485	A16507	252	A00485	A16732	185
A00485	A16876	216	A00485	A17078	454
A00486	A00820	174	A00486	A01011	486
A00486	A01276	182	A00486	A01431	63
A00486	A01557	430	A00486	A01566	418
A00486	A01572	232	A00486	A01578	344
A00486	A01630	447	A00486	A01632	363
A00486	A01645	333	A00486	A01652	199
A00486	A01661	156	A00486	A01668	274
A00486	A01752	303	A00486	A01769	241
A00486	A01839	394	A00486	A01902	115
A00486	A01903	307	A00486	A01911	210
A00486	A01915	477	A00486	A02188	167
A00486	A02350	438	A00486	A02687	302
A00486	A02691	246	A00486	A02713	193
A00486	A03019	63	A00486	A03259	63
A00486	A03297	369	A00486	A03321	433
A00486	A03388	191	A00486	A03550	199
A00486	A03611	171	A00486	A03648	411
A00486	A03803	180	A00486	A03813	229
A00486	A04139	340	A00486	A04168	283

A00486	A04171	241	A00486	A04410	185
A00486	A04435	266	A00486	A04704	151
A00486	A04715	450	A00486	A04790	190
A00486	A04805	185	A00486	A04832	152
A00486	A04903	257	A00486	A04907	229
A00486	A05075	283	A00486	A05094	270
A00486	A05114	475	A00486	A05422	270
A00486	A05759	165	A00486	A05889	463
A00486	A06106	313	A00486	A06428	296
A00486	A06653	174	A00486	A07384	170
A00486	A07810	328	A00486	A08002	276
A00486	A09953	283	A00486	A11172	291
A00486	A12009	177	A00486	A12734	328
A00486	A13369	383	A00486	A13434	235
A00486	A13829	310	A00486	A14633	185
A00486	A16538	361	A00487	A00555	271
A00487	A00619	154	A00487	A00673	217
A00487	A00687	276	A00487	A00709	293
A00487	A00851	360	A00487	A01061	332
A00487	A01064	268	A00487	A01151	408
A00487	A01217	188	A00487	A01243	484
A00487	A01352	152	A00487	A01362	160
A00487	A02499	285	A00487	A02565	152
A00487	A02626	475	A00487	A03628	260
A00487	A03901	475	A00487	A06273	199
A00487	A06832	341	A00487	A07132	238
A00487	A08576	255	A00487	A09020	228
A00487	A09648	156	A00487	A09977	243
A00487	A11522	241	A00487	A13667	198
A00487	A14148	204	A00487	A14178	215
A00487	A14566	467	A00487	A14594	486
A00487	A14701	417	A00487	A16324	152
A00488	A00490	584	A00488	A00497	706
A00488	A00501	484	A00488	A00518	356
A00488	A00520	202	A00488	A00521	382
A00488	A00532	321	A00488	A00550	638
A00488	A00551	398	A00488	A00554	683
A00488	A00595	411	A00488	A00605	589
A00488	A00648	151	A00488	A00690	489
A00488	A00701	407	A00488	A00708	272
A00488	A00767	428	A00488	A00831	334
A00488	A00916	491	A00488	A00923	399
A00488	A00934	463	A00488	A00944	177
A00488	A01020	707	A00488	A01032	328
A00488	A01034	190	A00488	A01040	424
A00488	A01047	174	A00488	A01075	208

A00488	A01079	198	A00488	A01085	183
A00488	A01112	334	A00488	A01116	635
A00488	A01184	491	A00488	A01201	409
A00488	A01206	483	A00488	A01234	491
A00488	A01291	462	A00488	A01304	637
A00488	A01306	254	A00488	A01470	205
A00488	A01492	485	A00488	A01495	334
A00488	A01539	489	A00488	A01564	182
A00488	A01598	383	A00488	A01649	408
A00488	A03069	218	A00488	A03457	185
A00488	A03734	154	A00488	A06275	344
A00488	A06965	466	A00488	A07223	637
A00488	A07347	491	A00488	A07524	154
A00488	A07533	433	A00488	A07584	380
A00488	A07625	436	A00488	A07700	636
A00488	A07706	168	A00488	A07725	638
A00488	A07924	272	A00488	A08326	234
A00488	A08357	299	A00488	A08382	221
A00488	A08452	457	A00488	A08491	462
A00488	A08521	232	A00488	A08972	187
A00488	A09209	229	A00488	A09322	180
A00488	A09619	512	A00488	A09705	398
A00488	A10616	429	A00488	A13989	485
A00488	A14505	463	A00488	A15525	165
A00488	A15541	303	A00488	A15709	162
A00488	A15735	483	A00488	A15739	474
A00488	A16302	306	A00488	A16394	188
A00489	A00532	322	A00489	A00605	430
A00489	A00629	291	A00489	A00640	307
A00489	A00667	385	A00489	A00705	477
A00489	A00774	288	A00489	A00831	232
A00489	A00843	316	A00489	A00848	491
A00489	A00878	210	A00489	A00883	489
A00489	A00905	263	A00489	A00916	491
A00489	A00970	477	A00489	A01017	491
A00489	A01042	286	A00489	A01069	675
A00489	A01075	172	A00489	A01100	396
A00489	A01115	722	A00489	A01119	302
A00489	A01150	321	A00489	A01166	479
A00489	A01194	491	A00489	A01201	383
A00489	A01206	396	A00489	A01219	378
A00489	A01234	490	A00489	A01243	185
A00489	A01291	481	A00489	A01351	299
A00489	A01354	346	A00489	A01420	394
A00489	A01431	489	A00489	A01470	490
A00489	A01492	321	A00489	A01495	196

A00489	A01539	721	A00489	A01558	329
A00489	A01569	491	A00489	A01577	344
A00489	A01598	490	A00489	A01612	472
A00489	A01614	486	A00489	A01640	168
A00489	A01649	474	A00489	A01707	378
A00489	A01770	398	A00489	A01917	185
A00489	A02602	156	A00489	A02626	323
A00489	A03446	721	A00489	A03560	404
A00489	A03567	416	A00489	A03569	479
A00489	A03740	154	A00489	A03783	404
A00489	A03863	404	A00489	A04021	430
A00489	A05385	165	A00489	A05645	441
A00489	A05712	371	A00489	A06109	218
A00489	A06531	475	A00489	A07004	281
A00489	A07247	435	A00489	A07706	341
A00489	A08137	326	A00489	A08489	391
A00489	A08546	507	A00489	A08896	382
A00489	A08978	215	A00489	A08991	459
A00489	A09318	317	A00489	A10006	366
A00489	A10266	252	A00489	A10465	288
A00489	A10490	457	A00489	A10532	658
A00489	A11240	372	A00489	A11409	491
A00489	A11704	160	A00489	A11843	171
A00489	A13609	485	A00489	A13989	480
A00489	A14152	177	A00489	A14208	224
A00489	A14596	473	A00489	A14597	469
A00489	A14609	313	A00489	A14701	480
A00489	A15297	477	A00489	A15338	154
A00489	A15468	168	A00489	A15474	205
A00489	A15519	210	A00489	A15660	436
A00489	A15684	335	A00489	A15698	372
A00489	A16834	468	A00489	A17067	267
A00490	A00497	506	A00490	A00520	134
A00490	A00554	462	A00490	A00566	339
A00490	A00584	252	A00490	A00767	104
A00490	A00858	383	A00490	A00916	235
A00490	A00934	384	A00490	A00944	395
A00490	A00960	278	A00490	A01020	693
A00490	A01116	347	A00490	A01184	294
A00490	A01201	433	A00490	A01224	170
A00490	A01234	489	A00490	A01351	177
A00490	A06797	254	A00490	A07223	196
A00490	A07533	226	A00490	A07584	337
A00490	A07725	218	A00490	A07924	177
A00490	A08491	462	A00490	A09619	462
A00490	A09664	254	A00490	A14508	224

A00491	A00511	430	A00491	A00531	484
A00491	A00552	327	A00491	A00567	274
A00491	A00718	375	A00491	A00762	159
A00491	A00835	188	A00491	A01075	388
A00491	A01087	196	A00491	A01125	290
A00491	A01198	363	A00491	A01232	63
A00491	A01406	152	A00491	A01598	395
A00491	A01665	152	A00491	A01790	389
A00491	A02021	419	A00491	A02180	344
A00491	A02402	74	A00491	A02470	160
A00491	A02505	159	A00491	A02607	213
A00491	A02671	185	A00491	A02839	246
A00491	A03104	395	A00491	A03181	154
A00491	A03379	190	A00491	A03391	463
A00491	A03625	165	A00491	A03757	386
A00491	A03771	151	A00491	A04770	200
A00491	A04780	263	A00491	A05228	318
A00491	A05439	490	A00491	A05629	256
A00491	A05697	343	A00491	A06038	429
A00491	A06660	221	A00491	A06891	243
A00491	A06907	324	A00491	A07209	378
A00491	A07210	208	A00491	A08555	310
A00491	A08568	185	A00491	A09141	362
A00491	A09272	483	A00491	A09464	210
A00491	A09956	356	A00491	A10461	386
A00491	A11291	420	A00491	A11476	162
A00491	A11670	162	A00491	A12265	418
A00491	A12298	414	A00491	A13299	324
A00491	A14026	152	A00491	A14220	945
A00491	A15366	234	A00491	A15717	196
A00491	A16410	358	A00491	A16892	318
A00492	A00720	151	A00492	A00794	688
A00492	A00942	179	A00492	A01078	688
A00492	A01143	249	A00492	A01270	157
A00492	A01461	483	A00492	A01500	198
A00492	A01522	433	A00492	A01574	215
A00492	A01575	586	A00492	A01585	491
A00492	A01634	344	A00492	A01660	271
A00492	A01671	157	A00492	A02671	469
A00492	A03044	196	A00492	A03452	649
A00492	A03618	688	A00492	A03879	157
A00492	A03891	340	A00492	A03892	491
A00492	A04292	185	A00492	A04897	238
A00492	A06137	162	A00492	A07062	152
A00492	A07736	607	A00492	A08137	156
A00492	A08400	380	A00492	A08461	477

A00492	A09622	154	A00492	A10078	199
A00492	A10655	688	A00492	A11279	461
A00492	A11570	425	A00492	A12011	191
A00492	A12834	252	A00492	A13882	465
A00492	A13894	329	A00492	A13895	420
A00492	A13902	730	A00492	A14115	212
A00492	A14167	201	A00492	A14182	425
A00492	A14247	458	A00492	A14272	281
A00492	A14661	473	A00492	A15315	190
A00492	A15347	154	A00492	A15377	208
A00492	A15647	500	A00492	A15774	154
A00492	A17121	219	A00492	A17133	456
A00493	A00527	447	A00493	A00581	485
A00493	A00609	491	A00493	A00617	435
A00493	A00718	491	A00493	A00778	486
A00493	A00779	340	A00493	A00781	185
A00493	A00797	483	A00493	A00907	489
A00493	A00920	343	A00493	A00948	406
A00493	A01021	263	A00493	A01026	491
A00493	A01100	191	A00493	A01313	486
A00493	A01360	210	A00493	A01365	210
A00493	A01375	173	A00493	A01377	490
A00493	A01386	480	A00493	A02135	230
A00493	A02494	335	A00493	A02704	367
A00493	A02774	196	A00493	A02836	308
A00493	A02878	463	A00493	A03408	170
A00493	A03556	152	A00493	A04145	224
A00493	A05052	291	A00493	A06472	311
A00493	A06616	286	A00493	A07111	195
A00493	A07558	418	A00493	A07621	279
A00493	A07693	479	A00493	A08113	417
A00493	A08161	162	A00493	A08260	374
A00493	A08263	479	A00493	A08289	170
A00493	A08346	491	A00493	A08402	491
A00493	A08694	323	A00493	A08975	461
A00493	A09164	243	A00493	A09648	307
A00493	A12174	353	A00493	A13821	267
A00493	A13873	168	A00493	A14077	473
A00493	A14092	462	A00493	A14191	395
A00493	A14272	491	A00493	A14707	491
A00493	A14840	480	A00493	A15367	382
A00493	A15562	416	A00493	A15877	489
A00493	A16418	163	A00493	A17152	258
A00494	A00507	288	A00494	A00521	285
A00494	A00537	382	A00494	A00539	180
A00494	A00550	217	A00494	A00571	473

A00494	A00593	156	A00494	A00604	433
A00494	A00610	238	A00494	A00623	247
A00494	A00638	224	A00494	A00653	488
A00494	A00667	401	A00494	A00671	270
A00494	A00673	332	A00494	A00687	307
A00494	A00697	333	A00494	A00700	385
A00494	A00739	152	A00494	A00740	577
A00494	A00742	213	A00494	A00779	267
A00494	A00782	355	A00494	A00787	191
A00494	A00788	218	A00494	A00789	157
A00494	A00791	227	A00494	A00793	154
A00494	A00809	241	A00494	A00811	152
A00494	A00824	478	A00494	A00839	574
A00494	A00843	234	A00494	A00856	241
A00494	A00865	252	A00494	A00878	311
A00494	A00886	252	A00494	A00897	228
A00494	A00929	230	A00494	A00953	160
A00494	A00970	329	A00494	A00980	287
A00494	A00991	266	A00494	A00996	247
A00494	A01004	241	A00494	A01016	418
A00494	A01017	317	A00494	A01025	273
A00494	A01027	272	A00494	A01086	234
A00494	A01102	568	A00494	A01115	190
A00494	A01119	247	A00494	A01150	170
A00494	A01154	377	A00494	A01161	154
A00494	A01194	565	A00494	A01196	241
A00494	A01207	65	A00494	A01218	590
A00494	A01221	338	A00494	A01242	414
A00494	A01263	446	A00494	A01270	241
A00494	A01291	353	A00494	A01317	311
A00494	A01337	160	A00494	A01365	423
A00494	A01369	426	A00494	A01374	366
A00494	A01411	592	A00494	A01420	277
A00494	A01421	165	A00494	A01436	234
A00494	A01449	443	A00494	A01455	247
A00494	A01539	313	A00494	A01569	480
A00494	A01579	392	A00494	A01581	247
A00494	A01601	585	A00494	A01747	165
A00494	A01824	433	A00494	A01826	247
A00494	A01841	427	A00494	A02227	202
A00494	A02353	489	A00494	A02718	196
A00494	A02737	217	A00494	A02887	235
A00494	A03453	589	A00494	A03959	180
A00494	A04687	229	A00494	A05499	163
A00494	A05501	321	A00494	A05502	174
A00494	A05702	185	A00494	A06084	213

A00494	A06534	327	A00494	A06814	267
A00494	A06878	232	A00494	A06973	408
A00494	A07047	241	A00494	A07398	539
A00494	A07677	223	A00494	A07695	319
A00494	A07746	268	A00494	A08154	188
A00494	A08277	254	A00494	A08315	177
A00494	A08358	326	A00494	A08359	163
A00494	A08369	249	A00494	A08408	241
A00494	A08476	241	A00494	A08556	249
A00494	A08761	193	A00494	A08762	243
A00494	A09308	562	A00494	A09320	296
A00494	A09322	241	A00494	A09502	221
A00494	A09587	479	A00494	A09620	241
A00494	A09713	369	A00494	A09717	247
A00494	A09735	460	A00494	A10159	228
A00494	A10490	159	A00494	A11540	243
A00494	A11597	230	A00494	A11728	332
A00494	A12227	241	A00494	A13340	224
A00494	A13609	416	A00494	A14074	247
A00494	A14479	396	A00494	A14482	249
A00494	A14566	213	A00494	A15268	241
A00494	A15381	241	A00494	A15696	213
A00494	A15771	241	A00494	A16437	168
A00495	A00606	240	A00495	A00636	665
A00495	A00722	393	A00495	A01074	229
A00495	A01162	488	A00495	A01365	329
A00495	A01473	411	A00495	A02010	456
A00495	A02542	330	A00495	A02993	393
A00495	A03097	224	A00495	A03134	193
A00495	A03686	393	A00495	A03703	480
A00495	A03721	260	A00495	A03728	393
A00495	A04161	488	A00495	A05327	350
A00495	A06786	313	A00495	A06964	451
A00495	A07181	439	A00495	A09362	475
A00495	A12835	438	A00495	A14431	252
A00495	A14470	472	A00495	A17076	157
A00496	A00532	436	A00496	A00560	491
A00496	A00635	351	A00496	A00718	291
A00496	A00835	491	A00496	A00836	168
A00496	A00837	458	A00496	A00883	352
A00496	A00905	440	A00496	A00916	363
A00496	A00933	185	A00496	A00940	475
A00496	A00954	263	A00496	A00957	491
A00496	A01075	489	A00496	A01082	246
A00496	A01091	263	A00496	A01097	262
A00496	A01132	293	A00496	A01143	491

A00496	A01206	375	A00496	A01234	176
A00496	A01259	337	A00496	A01284	157
A00496	A01291	293	A00496	A01426	226
A00496	A01598	481	A00496	A01612	457
A00496	A01638	195	A00496	A01660	488
A00496	A03057	486	A00496	A03112	156
A00496	A03136	486	A00496	A03446	208
A00496	A03557	344	A00496	A03569	458
A00496	A04020	481	A00496	A04050	388
A00496	A04449	364	A00496	A06466	270
A00496	A06491	156	A00496	A06783	157
A00496	A06907	491	A00496	A07311	469
A00496	A07360	213	A00496	A07604	491
A00496	A08137	491	A00496	A08571	477
A00496	A09319	899	A00496	A09591	167
A00496	A10615	168	A00496	A11291	491
A00496	A11323	212	A00496	A11405	486
A00496	A11487	364	A00496	A11767	263
A00496	A11983	199	A00496	A12069	489
A00496	A12138	183	A00496	A12328	199
A00496	A12329	208	A00496	A12365	156
A00496	A12543	481	A00496	A12660	343
A00496	A12943	208	A00496	A13320	174
A00496	A13335	160	A00496	A13587	490
A00496	A13609	221	A00496	A13762	324
A00496	A13928	491	A00496	A13989	353
A00496	A14148	319	A00496	A14196	490
A00496	A14220	491	A00496	A14221	488
A00496	A14257	491	A00496	A14265	279
A00496	A14303	457	A00496	A14476	168
A00496	A14490	279	A00496	A14596	343
A00496	A14659	273	A00496	A14660	260
A00496	A14875	208	A00496	A14931	210
A00496	A15140	168	A00496	A15237	165
A00496	A15291	446	A00496	A15297	208
A00496	A15309	477	A00496	A15366	491
A00496	A15492	491	A00496	A15596	154
A00496	A15614	404	A00496	A15665	433
A00496	A15741	183	A00496	A15749	152
A00496	A15752	485	A00496	A15753	283
A00496	A15754	187	A00496	A15763	445
A00496	A15773	484	A00496	A15851	380
A00496	A15852	341	A00496	A15870	364
A00496	A15878	183	A00496	A16115	218
A00496	A16724	414	A00496	A16768	283
A00496	A16814	160	A00496	A16895	324

A00496	A16897	252	A00496	A16994	383
A00496	A17063	319	A00496	A17126	157
A00496	A17198	223	A00497	A00517	224
A00497	A00520	281	A00497	A00521	278
A00497	A00532	303	A00497	A00550	630
A00497	A00551	491	A00497	A00554	707
A00497	A00562	229	A00497	A00595	460
A00497	A00605	334	A00497	A00691	402
A00497	A00708	177	A00497	A00760	243
A00497	A00787	63	A00497	A00824	224
A00497	A00831	334	A00497	A00858	490
A00497	A00884	288	A00497	A00903	198
A00497	A00916	491	A00497	A00934	439
A00497	A00942	260	A00497	A00954	324
A00497	A00960	162	A00497	A01001	232
A00497	A01020	708	A00497	A01040	334
A00497	A01075	446	A00497	A01079	191
A00497	A01087	369	A00497	A01112	334
A00497	A01116	635	A00497	A01184	489
A00497	A01201	313	A00497	A01206	187
A00497	A01234	491	A00497	A01304	637
A00497	A01351	436	A00497	A01488	311
A00497	A01492	446	A00497	A01495	334
A00497	A01500	316	A00497	A01633	226
A00497	A01649	173	A00497	A01741	249
A00497	A06564	249	A00497	A07111	228
A00497	A07223	423	A00497	A07347	486
A00497	A07533	228	A00497	A07584	375
A00497	A07697	258	A00497	A07700	636
A00497	A07706	270	A00497	A07725	362
A00497	A08022	184	A00497	A08024	400
A00497	A08211	491	A00497	A08491	633
A00497	A08518	213	A00497	A08528	152
A00497	A08553	152	A00497	A08807	221
A00497	A08834	254	A00497	A09010	345
A00497	A09307	319	A00497	A09619	707
A00497	A09664	414	A00497	A10380	151
A00497	A10456	477	A00497	A10616	152
A00497	A11395	451	A00497	A12254	438
A00497	A13294	205	A00497	A13989	484
A00497	A14212	168	A00497	A14655	407
A00497	A14745	245	A00497	A15946	229
A00497	A16109	308	A00497	A16302	167
A00498	A00565	185	A00498	A00568	317
A00498	A00664	620	A00498	A00670	246
A00498	A00684	160	A00498	A00846	489

A00498	A00850	165	A00498	A00855	317
A00498	A00943	366	A00498	A00968	443
A00498	A01021	218	A00498	A01074	618
A00498	A01105	317	A00498	A01189	274
A00498	A01205	491	A00498	A01269	394
A00498	A01360	226	A00498	A01404	257
A00498	A01508	347	A00498	A01574	183
A00498	A01622	350	A00498	A01679	524
A00498	A01801	347	A00498	A01926	185
A00498	A01993	326	A00498	A02061	323
A00498	A02214	419	A00498	A02343	160
A00498	A02364	466	A00498	A02524	179
A00498	A02525	196	A00498	A03044	252
A00498	A03095	401	A00498	A03468	229
A00498	A03710	317	A00498	A03728	267
A00498	A03954	341	A00498	A03962	154
A00498	A05150	219	A00498	A05210	477
A00498	A05332	615	A00498	A05370	205
A00498	A05435	429	A00498	A05609	569
A00498	A05689	329	A00498	A06101	356
A00498	A06134	218	A00498	A06250	329
A00498	A06264	170	A00498	A06629	154
A00498	A06819	296	A00498	A07074	281
A00498	A07222	403	A00498	A07232	323
A00498	A07311	899	A00498	A07333	317
A00498	A07499	266	A00498	A07571	532
A00498	A07654	235	A00498	A07974	430
A00498	A08097	317	A00498	A09276	436
A00498	A09340	317	A00498	A09350	466
A00498	A10394	347	A00498	A10483	378
A00498	A10982	288	A00498	A11271	236
A00498	A11357	262	A00498	A13517	319
A00498	A14590	429	A00498	A14803	390
A00498	A14858	329	A00498	A15560	388
A00498	A15655	477	A00498	A16219	339
A00498	A16597	152	A00498	A16753	314
A00499	A01133	319	A00499	A02878	265
A00499	A05449	317	A00499	A11297	355
A00499	A15188	413	A00499	A15254	438
A00499	A15507	412	A00499	A15547	435
A00499	A16822	321	A00500	A00519	176
A00500	A00532	477	A00500	A00605	385
A00500	A00623	183	A00500	A00648	486
A00500	A00651	373	A00500	A00693	486
A00500	A00700	296	A00500	A00713	110
A00500	A00831	491	A00500	A00843	486

A00500	A00848	491	A00500	A00871	491
A00500	A00902	168	A00500	A00916	470
A00500	A00970	346	A00500	A00992	163
A00500	A01024	469	A00500	A01050	395
A00500	A01069	302	A00500	A01072	390
A00500	A01088	154	A00500	A01090	201
A00500	A01112	489	A00500	A01140	358
A00500	A01150	477	A00500	A01164	204
A00500	A01166	491	A00500	A01201	418
A00500	A01206	475	A00500	A01214	491
A00500	A01215	232	A00500	A01224	313
A00500	A01226	215	A00500	A01234	491
A00500	A01238	340	A00500	A01243	262
A00500	A01291	430	A00500	A01304	490
A00500	A01354	201	A00500	A01384	323
A00500	A01418	182	A00500	A01425	491
A00500	A01468	491	A00500	A01470	491
A00500	A01487	382	A00500	A01492	491
A00500	A01495	245	A00500	A01496	385
A00500	A01506	247	A00500	A01538	218
A00500	A01546	321	A00500	A01569	480
A00500	A01577	258	A00500	A01626	475
A00500	A01649	486	A00500	A01684	481
A00500	A01917	224	A00500	A03438	201
A00500	A03456	180	A00500	A03461	258
A00500	A03494	228	A00500	A03567	412
A00500	A03859	266	A00500	A04178	274
A00500	A04697	486	A00500	A05025	350
A00500	A06025	310	A00500	A07111	241
A00500	A07414	247	A00500	A07481	417
A00500	A07644	168	A00500	A07695	466
A00500	A07723	177	A00500	A08144	243
A00500	A08537	355	A00500	A08892	333
A00500	A09621	368	A00500	A09674	307
A00500	A10238	160	A00500	A10532	465
A00500	A10624	243	A00500	A13989	491
A00500	A14006	484	A00500	A14074	276
A00500	A14216	489	A00500	A14597	263
A00501	A00550	377	A00501	A00589	572
A00501	A00605	682	A00501	A00648	475
A00501	A00687	154	A00501	A00690	469
A00501	A00705	303	A00501	A00708	294
A00501	A00713	340	A00501	A00749	210
A00501	A00822	327	A00501	A00831	165
A00501	A00848	204	A00501	A00872	210
A00501	A00883	380	A00501	A00905	327

A00501	A00916	491	A00501	A00945	419
A00501	A00992	152	A00501	A01028	212
A00501	A01065	552	A00501	A01075	316
A00501	A01092	210	A00501	A01098	210
A00501	A01112	347	A00501	A01115	159
A00501	A01120	572	A00501	A01150	306
A00501	A01166	490	A00501	A01179	489
A00501	A01206	491	A00501	A01234	490
A00501	A01268	177	A00501	A01291	485
A00501	A01349	346	A00501	A01354	288
A00501	A01379	526	A00501	A01384	405
A00501	A01425	380	A00501	A01430	467
A00501	A01439	672	A00501	A01470	491
A00501	A01492	571	A00501	A01538	210
A00501	A01539	462	A00501	A01555	156
A00501	A01588	204	A00501	A01597	424
A00501	A01598	572	A00501	A01612	486
A00501	A01614	572	A00501	A01649	491
A00501	A02120	174	A00501	A02178	255
A00501	A02265	210	A00501	A02978	210
A00501	A05230	528	A00501	A06429	152
A00501	A06897	440	A00501	A07076	438
A00501	A07347	491	A00501	A07348	343
A00501	A07604	185	A00501	A07725	412
A00501	A07759	193	A00501	A08137	491
A00501	A08357	491	A00501	A08359	196
A00501	A08377	184	A00501	A08571	364
A00501	A08814	228	A00501	A08965	303
A00501	A09292	568	A00501	A09707	484
A00501	A11462	491	A00501	A11914	238
A00501	A11942	485	A00501	A11945	462
A00501	A12077	303	A00501	A13052	210
A00501	A13126	438	A00501	A13522	232
A00501	A13875	419	A00501	A13989	572
A00501	A14167	174	A00501	A14216	375
A00501	A14816	210	A00501	A14817	263
A00501	A14921	232	A00501	A15215	486
A00501	A15256	199	A00501	A15338	183
A00501	A15541	483	A00501	A15660	404
A00501	A15684	179	A00501	A15709	480
A00501	A15735	491	A00501	A15739	475
A00501	A16157	154	A00501	A16565	177
A00501	A16597	179	A00501	A16857	193
A00501	A17014	199	A00501	A17063	254
A00501	A17064	273	A00502	A00510	353
A00502	A00521	361	A00502	A00547	269

A00502	A00561	196	A00502	A00598	484
A00502	A00604	590	A00502	A00608	270
A00502	A00610	430	A00502	A00649	590
A00502	A00653	592	A00502	A00731	581
A00502	A00745	283	A00502	A00750	246
A00502	A00780	427	A00502	A00783	357
A00502	A00785	591	A00502	A00790	589
A00502	A00793	373	A00502	A00842	223
A00502	A00849	247	A00502	A00953	591
A00502	A00974	590	A00502	A00987	205
A00502	A01023	202	A00502	A01027	581
A00502	A01037	201	A00502	A01081	385
A00502	A01086	590	A00502	A01108	591
A00502	A01150	247	A00502	A01161	247
A00502	A01204	588	A00502	A01246	305
A00502	A01287	247	A00502	A01346	585
A00502	A01365	329	A00502	A01399	590
A00502	A01443	505	A00502	A01446	463
A00502	A01448	451	A00502	A01458	173
A00502	A01503	184	A00502	A01585	303
A00502	A01711	172	A00502	A01743	228
A00502	A02994	247	A00502	A03489	485
A00502	A03959	247	A00502	A04028	257
A00502	A04895	477	A00502	A05125	296
A00502	A05839	276	A00502	A06356	191
A00502	A06959	366	A00502	A07004	212
A00502	A07237	351	A00502	A07350	296
A00502	A07362	152	A00502	A07569	445
A00502	A07597	218	A00502	A07773	68
A00502	A07792	149	A00502	A07793	183
A00502	A08011	256	A00502	A08052	188
A00502	A08760	363	A00502	A09616	458
A00502	A10199	319	A00502	A10535	405
A00502	A11015	229	A00502	A11677	247
A00502	A13497	296	A00502	A14479	319
A00502	A14699	247	A00502	A14778	247
A00502	A16587	247	A00503	A00540	226
A00503	A00570	210	A00503	A00595	314
A00503	A00687	427	A00503	A00729	613
A00503	A00829	671	A00503	A00992	263
A00503	A00993	159	A00503	A01159	152
A00503	A01539	406	A00503	A02203	226
A00503	A02377	172	A00503	A02500	452
A00503	A03438	324	A00503	A03595	279
A00503	A03597	419	A00503	A03609	324
A00503	A03952	238	A00503	A04449	668

A00503	A05154	174	A00503	A05605	407
A00503	A05646	218	A00503	A06122	195
A00503	A06336	335	A00503	A07439	302
A00503	A08010	293	A00503	A08077	177
A00503	A08340	414	A00503	A08918	414
A00503	A10590	513	A00503	A12304	256
A00503	A12369	335	A00503	A12988	163
A00503	A13397	182	A00503	A14729	318
A00503	A15624	363	A00503	A15932	357
A00504	A00505	332	A00504	A00521	154
A00504	A00540	344	A00504	A00563	299
A00504	A00570	168	A00504	A00687	473
A00504	A00689	317	A00504	A00829	188
A00504	A00878	196	A00504	A00997	174
A00504	A01102	205	A00504	A01159	291
A00504	A01220	332	A00504	A01221	157
A00504	A01238	257	A00504	A01353	160
A00504	A01375	204	A00504	A01392	316
A00504	A01418	213	A00504	A01469	177
A00504	A01594	213	A00504	A01625	162
A00504	A01711	224	A00504	A02166	393
A00504	A02268	243	A00504	A02454	241
A00504	A02518	226	A00504	A02542	258
A00504	A03070	188	A00504	A03958	361
A00504	A04507	413	A00504	A05143	356
A00504	A05199	427	A00504	A06351	263
A00504	A06825	326	A00504	A08452	227
A00504	A09227	352	A00504	A14188	157
A00504	A14482	199	A00505	A00571	304
A00505	A00593	543	A00505	A00653	188
A00505	A00673	188	A00505	A00687	198
A00505	A00705	539	A00505	A00709	188
A00505	A00711	234	A00505	A00824	238
A00505	A00839	188	A00505	A00953	188
A00505	A00972	188	A00505	A01017	558
A00505	A01101	188	A00505	A01214	385
A00505	A01217	275	A00505	A01220	188
A00505	A01335	457	A00505	A01337	472
A00505	A01362	348	A00505	A01369	441
A00505	A01375	154	A00505	A01406	208
A00505	A01408	188	A00505	A01414	188
A00505	A01451	188	A00505	A01579	226
A00505	A01976	188	A00505	A01988	188
A00505	A03901	188	A00505	A04507	396
A00505	A05702	188	A00505	A06785	520
A00505	A07965	511	A00505	A08452	177

A00505	A09227	321	A00505	A11480	188
A00505	A13855	168	A00505	A14813	188
A00505	A15657	188	A00505	A16680	188
A00506	A00525	191	A00506	A00531	272
A00506	A00687	274	A00506	A00694	218
A00506	A00735	457	A00506	A00737	385
A00506	A00821	363	A00506	A00865	216
A00506	A00998	288	A00506	A01013	183
A00506	A01053	213	A00506	A01150	167
A00506	A01161	154	A00506	A01245	372
A00506	A01401	176	A00506	A01403	260
A00506	A01502	243	A00506	A01504	172
A00506	A01711	351	A00506	A01741	157
A00506	A01815	350	A00506	A01819	286
A00506	A01820	364	A00506	A01825	246
A00506	A01832	491	A00506	A01833	462
A00506	A02709	335	A00506	A02994	199
A00506	A03856	260	A00506	A03860	245
A00506	A03931	472	A00506	A04056	416
A00506	A04423	279	A00506	A05418	182
A00506	A06358	196	A00506	A08521	198
A00506	A09321	339	A00506	A15282	312
A00506	A15668	290	A00506	A16149	260
A00507	A00547	383	A00507	A00571	468
A00507	A00610	291	A00507	A00711	281
A00507	A00782	457	A00507	A00788	290
A00507	A00802	188	A00507	A00839	491
A00507	A00991	196	A00507	A01121	417
A00507	A01152	193	A00507	A01153	217
A00507	A01154	462	A00507	A01186	474
A00507	A01247	176	A00507	A01263	358
A00507	A01306	258	A00507	A01335	438
A00507	A01337	416	A00507	A01365	436
A00507	A01369	152	A00507	A01407	208
A00507	A01411	254	A00507	A01449	472
A00507	A01741	240	A00507	A02227	224
A00507	A02814	193	A00507	A05501	407
A00507	A05502	360	A00507	A07248	260
A00507	A07658	313	A00507	A08154	246
A00507	A08358	162	A00507	A09735	462
A00507	A11597	167	A00507	A11616	352
A00507	A11688	460	A00507	A12011	281
A00507	A12821	486	A00507	A13146	183
A00507	A14813	290	A00507	A15215	229
A00507	A15500	235	A00507	A15771	310
A00508	A00727	236	A00508	A00883	458

A00508	A00985	163	A00508	A01173	475
A00508	A01243	188	A00508	A01442	191
A00508	A03149	252	A00508	A03893	174
A00508	A05563	183	A00508	A05944	475
A00508	A07708	475	A00508	A07887	198
A00508	A08840	475	A00508	A08982	281
A00508	A11933	456	A00508	A14196	346
A00508	A15353	430	A00508	A15431	475
A00508	A15543	177	A00508	A15559	151
A00508	A15561	217	A00508	A15564	486
A00508	A15631	449	A00508	A15747	452
A00508	A15775	334	A00509	A00515	173
A00509	A00556	154	A00509	A00568	216
A00509	A00664	294	A00509	A00820	283
A00509	A01404	424	A00509	A01604	245
A00509	A01716	243	A00509	A01737	174
A00509	A01899	466	A00509	A02156	276
A00509	A02209	401	A00509	A02251	208
A00509	A02260	271	A00509	A02262	291
A00509	A02268	290	A00509	A02272	285
A00509	A02524	351	A00509	A02698	469
A00509	A02779	176	A00509	A02904	302
A00509	A03086	279	A00509	A03091	227
A00509	A03167	293	A00509	A03173	371
A00509	A03212	243	A00509	A03821	163
A00509	A04258	374	A00509	A05106	204
A00509	A05157	267	A00509	A05369	441
A00509	A05603	151	A00509	A06471	472
A00509	A06970	344	A00509	A07062	252
A00509	A07140	443	A00509	A07182	346
A00509	A07209	151	A00509	A07324	357
A00509	A07655	490	A00509	A07748	419
A00509	A07815	168	A00509	A07878	486
A00509	A08630	249	A00509	A08660	425
A00509	A08688	489	A00509	A10394	371
A00509	A10466	311	A00509	A10980	396
A00509	A11522	165	A00509	A11885	367
A00509	A12150	396	A00509	A14430	337
A00510	A00598	438	A00510	A00604	373
A00510	A00608	388	A00510	A00649	462
A00510	A00653	490	A00510	A00731	477
A00510	A00745	260	A00510	A00750	420
A00510	A00780	268	A00510	A00783	449
A00510	A00785	491	A00510	A00790	488
A00510	A00802	168	A00510	A00849	160
A00510	A00953	438	A00510	A00974	157

A00510	A01027	480	A00510	A01086	490
A00510	A01094	301	A00510	A01108	409
A00510	A01204	393	A00510	A01246	441
A00510	A01260	377	A00510	A01346	462
A00510	A01399	491	A00510	A01443	286
A00510	A01446	455	A00510	A01448	486
A00510	A01540	246	A00510	A01563	172
A00510	A04627	162	A00510	A04895	484
A00510	A05318	179	A00510	A06320	226
A00510	A06411	238	A00510	A06589	254
A00510	A06959	252	A00510	A07237	236
A00510	A07569	369	A00510	A07597	340
A00510	A08982	477	A00510	A08990	294
A00510	A09616	290	A00510	A10199	398
A00510	A10535	232	A00510	A11015	323
A00510	A11322	154	A00510	A11427	173
A00510	A11474	159	A00510	A13253	385
A00510	A13275	475	A00510	A13286	462
A00510	A13303	440	A00510	A15051	385
A00511	A00567	384	A00511	A00597	384
A00511	A00609	154	A00511	A00611	361
A00511	A00888	296	A00511	A01375	195
A00511	A01716	310	A00511	A02018	313
A00511	A04770	313	A00511	A08655	340
A00511	A10709	266	A00511	A15383	165
A00511	A16529	216	A00512	A00589	301
A00512	A00655	208	A00512	A00822	152
A00512	A00832	207	A00512	A01056	254
A00512	A01140	219	A00512	A01179	288
A00512	A01649	281	A00512	A06814	213
A00512	A06897	299	A00512	A08357	419
A00512	A09292	296	A00512	A09707	324
A00512	A11462	319	A00513	A00862	171
A00513	A00941	363	A00513	A01378	432
A00513	A01459	479	A00513	A01507	491
A00513	A02677	355	A00513	A03465	324
A00513	A03778	301	A00513	A04449	398
A00513	A04818	226	A00513	A06819	165
A00513	A07803	240	A00513	A08282	385
A00513	A08621	473	A00513	A10808	174
A00513	A11345	232	A00513	A12047	486
A00514	A00601	165	A00514	A00728	263
A00514	A00915	369	A00514	A00925	255
A00514	A01150	63	A00514	A01161	252
A00514	A01169	369	A00514	A01253	322
A00514	A01278	182	A00514	A01317	168

A00514	A01329	165	A00514	A01582	317
A00514	A01648	249	A00514	A01667	296
A00514	A03559	371	A00514	A03885	274
A00514	A04666	191	A00514	A05276	313
A00514	A08191	357	A00514	A11352	446
A00514	A13432	269	A00514	A15295	432
A00514	A15498	157	A00515	A00564	160
A00515	A00697	291	A00515	A00759	267
A00515	A00781	180	A00515	A00883	414
A00515	A00926	184	A00515	A01122	156
A00515	A01169	361	A00515	A01309	262
A00515	A01458	207	A00515	A01671	425
A00515	A01809	268	A00515	A02049	346
A00515	A04092	177	A00515	A04223	257
A00515	A04383	255	A00515	A04881	201
A00515	A05100	296	A00515	A05157	213
A00515	A05273	196	A00515	A05276	232
A00515	A05427	202	A00515	A05539	317
A00515	A06488	160	A00515	A06670	283
A00515	A07101	388	A00515	A07311	199
A00515	A07434	193	A00515	A07748	168
A00515	A07878	160	A00515	A08047	372
A00515	A08630	193	A00515	A08660	185
A00515	A08667	229	A00515	A10090	307
A00515	A10104	372	A00515	A10640	227
A00515	A13734	199	A00515	A14170	236
A00515	A14180	340	A00515	A15230	167
A00516	A00583	191	A00516	A00726	210
A00516	A00742	255	A00516	A00766	223
A00516	A00918	219	A00516	A00973	269
A00516	A01061	488	A00516	A01151	490
A00516	A01177	313	A00516	A01192	252
A00516	A01312	224	A00516	A01470	359
A00516	A07146	241	A00516	A07378	326
A00516	A08965	435	A00516	A09394	241
A00516	A14594	208	A00516	A15338	224
A00516	A17088	335	A00517	A00521	228
A00517	A00532	435	A00517	A00562	279
A00517	A00566	542	A00517	A00572	185
A00517	A00575	540	A00517	A00609	446
A00517	A00622	452	A00517	A00626	372
A00517	A00665	400	A00517	A00671	252
A00517	A00689	247	A00517	A00703	291
A00517	A00733	542	A00517	A00743	218
A00517	A00744	500	A00517	A00781	252
A00517	A00791	227	A00517	A00805	824

A00517	A00819	369	A00517	A00822	491
A00517	A00848	312	A00517	A00858	541
A00517	A00860	538	A00517	A00866	509
A00517	A00881	536	A00517	A00883	622
A00517	A00902	255	A00517	A00905	481
A00517	A00910	191	A00517	A00916	490
A00517	A00940	196	A00517	A00978	499
A00517	A00996	236	A00517	A01007	169
A00517	A01019	540	A00517	A01042	405
A00517	A01052	622	A00517	A01057	491
A00517	A01075	291	A00517	A01077	491
A00517	A01087	163	A00517	A01091	234
A00517	A01173	466	A00517	A01195	542
A00517	A01200	509	A00517	A01208	310
A00517	A01212	491	A00517	A01234	229
A00517	A01243	299	A00517	A01325	265
A00517	A01351	435	A00517	A01354	486
A00517	A01374	263	A00517	A01383	423
A00517	A01385	329	A00517	A01390	254
A00517	A01398	530	A00517	A01401	537
A00517	A01414	278	A00517	A01470	193
A00517	A01492	424	A00517	A01500	536
A00517	A01503	466	A00517	A01571	286
A00517	A01572	393	A00517	A01598	452
A00517	A01610	465	A00517	A01612	491
A00517	A01633	333	A00517	A01649	260
A00517	A01655	201	A00517	A01720	452
A00517	A01729	238	A00517	A01770	263
A00517	A01781	469	A00517	A02715	457
A00517	A03207	294	A00517	A03479	477
A00517	A03526	439	A00517	A03569	456
A00517	A03740	374	A00517	A03793	265
A00517	A04181	455	A00517	A04648	498
A00517	A04687	156	A00517	A05017	224
A00517	A05027	193	A00517	A05981	243
A00517	A06803	538	A00517	A06907	358
A00517	A07203	234	A00517	A07290	234
A00517	A07604	311	A00517	A07670	484
A00517	A07850	523	A00517	A07887	539
A00517	A07920	484	A00517	A08137	491
A00517	A08150	187	A00517	A08272	229
A00517	A08383	373	A00517	A08522	223
A00517	A08571	378	A00517	A08818	156
A00517	A08834	308	A00517	A08882	184
A00517	A09432	177	A00517	A09502	156
A00517	A09712	480	A00517	A09943	283

A00517	A10321	451	A00517	A10365	260
A00517	A10642	341	A00517	A11344	156
A00517	A11673	160	A00517	A11674	156
A00517	A11737	176	A00517	A13824	457
A00517	A14006	491	A00517	A14220	481
A00517	A14221	472	A00517	A14257	468
A00517	A14399	488	A00517	A14467	177
A00517	A14596	180	A00517	A14827	282
A00517	A15183	177	A00517	A15297	483
A00517	A15338	168	A00517	A15366	450
A00517	A15367	208	A00517	A15545	475
A00517	A15649	256	A00517	A15752	490
A00517	A16228	452	A00517	A16410	301
A00518	A00520	404	A00518	A00550	430
A00518	A00734	276	A00518	A00767	307
A00518	A00878	326	A00518	A00916	373
A00518	A00934	416	A00518	A00944	439
A00518	A01020	408	A00518	A01116	433
A00518	A01184	483	A00518	A01201	477
A00518	A01224	288	A00518	A01234	489
A00518	A01304	438	A00518	A01374	396
A00518	A01721	243	A00518	A03596	201
A00518	A04152	273	A00518	A04337	223
A00518	A04770	251	A00518	A05845	396
A00518	A06275	157	A00518	A06627	240
A00518	A07223	458	A00518	A07347	393
A00518	A07584	401	A00518	A07700	339
A00518	A08700	283	A00518	A09664	241
A00518	A10616	156	A00518	A11313	198
A00518	A12419	270	A00518	A13993	160
A00518	A14925	191	A00519	A00524	283
A00519	A00526	449	A00519	A00605	491
A00519	A00653	480	A00519	A00658	577
A00519	A00687	223	A00519	A00711	416
A00519	A00715	223	A00519	A00724	480
A00519	A00749	457	A00519	A00769	491
A00519	A00785	177	A00519	A00814	374
A00519	A00824	188	A00519	A00841	491
A00519	A00871	578	A00519	A00909	491
A00519	A00911	270	A00519	A00916	270
A00519	A00922	491	A00519	A00926	258
A00519	A00927	490	A00519	A00983	491
A00519	A00986	578	A00519	A00994	208
A00519	A01027	491	A00519	A01069	486
A00519	A01101	388	A00519	A01111	223
A00519	A01118	555	A00519	A01150	358

A00519	A01202	395	A00519	A01208	578
A00519	A01215	578	A00519	A01228	471
A00519	A01232	491	A00519	A01293	479
A00519	A01311	363	A00519	A01353	578
A00519	A01492	491	A00519	A01518	469
A00519	A01538	407	A00519	A01598	156
A00519	A01707	193	A00519	A01736	232
A00519	A01908	152	A00519	A01988	447
A00519	A02041	250	A00519	A02051	223
A00519	A02072	191	A00519	A02270	232
A00519	A03061	154	A00519	A03169	578
A00519	A03438	491	A00519	A03454	490
A00519	A03600	212	A00519	A03677	406
A00519	A03792	255	A00519	A03952	182
A00519	A03958	419	A00519	A03972	163
A00519	A04447	477	A00519	A05695	260
A00519	A06600	283	A00519	A07180	429
A00519	A07225	249	A00519	A07265	577
A00519	A07360	491	A00519	A07456	375
A00519	A07524	443	A00519	A07588	578
A00519	A07695	118	A00519	A07706	256
A00519	A07744	473	A00519	A07881	491
A00519	A08098	399	A00519	A08228	488
A00519	A08548	221	A00519	A08955	223
A00519	A08971	223	A00519	A09328	255
A00519	A09686	260	A00519	A09910	191
A00519	A10380	485	A00519	A10586	193
A00519	A10755	578	A00519	A11202	223
A00519	A11608	490	A00519	A11635	344
A00519	A11918	152	A00519	A13298	578
A00519	A13645	176	A00519	A14216	223
A00519	A14443	324	A00519	A14482	297
A00519	A14699	491	A00519	A14778	274
A00519	A15624	364	A00519	A15651	205
A00519	A15652	490	A00519	A15661	489
A00519	A15662	205	A00519	A15684	614
A00519	A15746	182	A00519	A15937	434
A00519	A16088	489	A00519	A16802	485
A00520	A00532	234	A00520	A00550	394
A00520	A00595	358	A00520	A00883	221
A00520	A00916	491	A00520	A00934	333
A00520	A00944	165	A00520	A00994	391
A00520	A01020	457	A00520	A01116	469
A00520	A01184	480	A00520	A01201	398
A00520	A01206	386	A00520	A01234	491
A00520	A01304	364	A00520	A01398	335

A00520	A01470	411	A00520	A01492	456
A00520	A01500	261	A00520	A01598	380
A00520	A01612	212	A00520	A01647	254
A00520	A01649	154	A00520	A02064	191
A00520	A03734	236	A00520	A04446	219
A00520	A04908	251	A00520	A05547	199
A00520	A05845	313	A00520	A06275	65
A00520	A06662	156	A00520	A06881	272
A00520	A07223	360	A00520	A07242	193
A00520	A07311	199	A00520	A07347	455
A00520	A07483	314	A00520	A07524	187
A00520	A07584	369	A00520	A07700	486
A00520	A07924	152	A00520	A08024	229
A00520	A08280	329	A00520	A09619	232
A00520	A09664	409	A00520	A09712	435
A00520	A10616	144	A00520	A10923	234
A00520	A11472	428	A00520	A11831	176
A00520	A11832	485	A00520	A12290	286
A00520	A12419	159	A00520	A12849	424
A00520	A13989	458	A00520	A14006	234
A00520	A14655	314	A00520	A14661	177
A00520	A15347	154	A00520	A15764	488
A00520	A16088	329	A00520	A16285	185
A00520	A16302	160	A00520	A16877	152
A00521	A00531	388	A00521	A00532	157
A00521	A00537	475	A00521	A00561	269
A00521	A00571	460	A00521	A00590	491
A00521	A00598	429	A00521	A00603	491
A00521	A00604	457	A00521	A00605	491
A00521	A00629	483	A00521	A00648	491
A00521	A00649	369	A00521	A00651	462
A00521	A00653	491	A00521	A00671	263
A00521	A00687	265	A00521	A00694	172
A00521	A00697	400	A00521	A00701	329
A00521	A00703	251	A00521	A00731	163
A00521	A00763	486	A00521	A00772	232
A00521	A00783	491	A00521	A00785	433
A00521	A00787	491	A00521	A00790	246
A00521	A00824	305	A00521	A00839	491
A00521	A00865	541	A00521	A00870	481
A00521	A00878	491	A00521	A00923	491
A00521	A00942	283	A00521	A00949	167
A00521	A00953	491	A00521	A00965	419
A00521	A00983	163	A00521	A00987	451
A00521	A00990	200	A00521	A00996	271
A00521	A01000	491	A00521	A01001	238

A00521	A01012	269	A00521	A01016	229
A00521	A01017	235	A00521	A01025	490
A00521	A01027	483	A00521	A01034	436
A00521	A01037	475	A00521	A01042	252
A00521	A01047	565	A00521	A01054	490
A00521	A01061	235	A00521	A01066	200
A00521	A01067	488	A00521	A01085	278
A00521	A01101	491	A00521	A01106	305
A00521	A01108	456	A00521	A01127	491
A00521	A01140	369	A00521	A01150	470
A00521	A01161	165	A00521	A01184	491
A00521	A01186	269	A00521	A01189	490
A00521	A01195	234	A00521	A01196	252
A00521	A01206	489	A00521	A01211	162
A00521	A01234	477	A00521	A01246	172
A00521	A01247	399	A00521	A01249	565
A00521	A01263	488	A00521	A01264	200
A00521	A01268	226	A00521	A01291	649
A00521	A01306	491	A00521	A01317	221
A00521	A01335	452	A00521	A01338	559
A00521	A01365	329	A00521	A01374	306
A00521	A01382	408	A00521	A01392	491
A00521	A01399	484	A00521	A01416	168
A00521	A01418	235	A00521	A01429	345
A00521	A01430	443	A00521	A01433	491
A00521	A01436	200	A00521	A01437	491
A00521	A01446	473	A00521	A01448	469
A00521	A01457	473	A00521	A01464	520
A00521	A01468	216	A00521	A01496	480
A00521	A01540	419	A00521	A01579	490
A00521	A01601	191	A00521	A01618	246
A00521	A01633	262	A00521	A01649	490
A00521	A01700	224	A00521	A01822	229
A00521	A02216	443	A00521	A02978	306
A00521	A03044	235	A00521	A03069	491
A00521	A03490	252	A00521	A03567	491
A00521	A03754	374	A00521	A04430	201
A00521	A04507	278	A00521	A04542	215
A00521	A05017	262	A00521	A05121	371
A00521	A05411	322	A00521	A05501	329
A00521	A06861	270	A00521	A06878	224
A00521	A06897	384	A00521	A06965	490
A00521	A06973	497	A00521	A07412	223
A00521	A07451	475	A00521	A07533	425
A00521	A07584	221	A00521	A07597	291
A00521	A07677	224	A00521	A07819	333

A00521	A08011	491	A00521	A08055	290
A00521	A08137	473	A00521	A08301	266
A00521	A08357	484	A00521	A08398	566
A00521	A08403	411	A00521	A08419	358
A00521	A08452	566	A00521	A08488	425
A00521	A08521	565	A00521	A08543	396
A00521	A08547	483	A00521	A08556	422
A00521	A08558	200	A00521	A08664	253
A00521	A08773	490	A00521	A08860	230
A00521	A08987	435	A00521	A09044	281
A00521	A09322	480	A00521	A09332	441
A00521	A09442	165	A00521	A09592	372
A00521	A09615	319	A00521	A09702	562
A00521	A10417	200	A00521	A10518	491
A00521	A10532	343	A00521	A10616	319
A00521	A10719	173	A00521	A11316	168
A00521	A11322	360	A00521	A11338	368
A00521	A11390	167	A00521	A11419	449
A00521	A11427	483	A00521	A11438	544
A00521	A11480	486	A00521	A11540	230
A00521	A11588	419	A00521	A11616	182
A00521	A11729	200	A00521	A11731	347
A00521	A11836	279	A00521	A11945	171
A00521	A12059	258	A00521	A12079	215
A00521	A12080	195	A00521	A12233	350
A00521	A12252	332	A00521	A12433	434
A00521	A12821	346	A00521	A13069	157
A00521	A13333	556	A00521	A13340	238
A00521	A13525	473	A00521	A13709	452
A00521	A13710	223	A00521	A13989	486
A00521	A13992	274	A00521	A14152	243
A00521	A14216	391	A00521	A14482	305
A00521	A14505	480	A00521	A14508	151
A00521	A14674	249	A00521	A14738	307
A00521	A14825	165	A00521	A15500	367
A00521	A15660	364	A00521	A15684	463
A00521	A15714	260	A00521	A15931	443
A00521	A15988	475	A00521	A16118	424
A00521	A16157	173	A00521	A17193	160
A00522	A00542	270	A00522	A00546	491
A00522	A00562	491	A00522	A00625	902
A00522	A00643	276	A00522	A00786	446
A00522	A00812	168	A00522	A00814	291
A00522	A00864	491	A00522	A00877	436
A00522	A00951	383	A00522	A00971	488
A00522	A01036	329	A00522	A01191	488

A00522	A01216	484	A00522	A01290	335
A00522	A01308	466	A00522	A01366	752
A00522	A01454	458	A00522	A01459	475
A00522	A01484	286	A00522	A01514	475
A00522	A01549	479	A00522	A01568	490
A00522	A01591	200	A00522	A01630	63
A00522	A01657	296	A00522	A01687	491
A00522	A01781	266	A00522	A01988	488
A00522	A02535	497	A00522	A02634	191
A00522	A02864	172	A00522	A03407	424
A00522	A04012	156	A00522	A04573	157
A00522	A04583	411	A00522	A04592	257
A00522	A05016	443	A00522	A05094	154
A00522	A05301	195	A00522	A05381	221
A00522	A05899	199	A00522	A06060	350
A00522	A06176	468	A00522	A06930	229
A00522	A07054	484	A00522	A07083	252
A00522	A07187	262	A00522	A07201	173
A00522	A07242	473	A00522	A07495	491
A00522	A07665	489	A00522	A07696	480
A00522	A07709	271	A00522	A07714	288
A00522	A07816	274	A00522	A07934	162
A00522	A07954	470	A00522	A08464	191
A00522	A08531	213	A00522	A09662	344
A00522	A09666	473	A00522	A10784	497
A00522	A11116	446	A00522	A11196	489
A00522	A11281	369	A00522	A11346	452
A00522	A11675	425	A00522	A12047	491
A00522	A12419	202	A00522	A13146	279
A00522	A13239	171	A00522	A13488	165
A00522	A13784	497	A00522	A14209	486
A00522	A14266	407	A00522	A14281	752
A00522	A14464	452	A00522	A14474	709
A00522	A14484	489	A00522	A14633	485
A00522	A14665	308	A00522	A15004	378
A00522	A15039	208	A00522	A15232	288
A00522	A15260	221	A00522	A15272	157
A00522	A15295	218	A00522	A15380	486
A00523	A00525	481	A00523	A00544	228
A00523	A00571	183	A00523	A00649	151
A00523	A00651	254	A00523	A00653	404
A00523	A00741	269	A00523	A00742	299
A00523	A00821	394	A00523	A00829	260
A00523	A00849	467	A00523	A00984	182
A00523	A00985	305	A00523	A00987	227
A00523	A00998	319	A00523	A01013	352

A00523	A01050	204	A00523	A01150	490
A00523	A01152	230	A00523	A01155	241
A00523	A01161	490	A00523	A01168	188
A00523	A01210	446	A00523	A01214	202
A00523	A01278	173	A00523	A01287	491
A00523	A01502	391	A00523	A01504	343
A00523	A01558	899	A00523	A01588	899
A00523	A01618	210	A00523	A01667	374
A00523	A01711	468	A00523	A01741	238
A00523	A01815	372	A00523	A01816	367
A00523	A01819	414	A00523	A01825	281
A00523	A02994	454	A00523	A02997	378
A00523	A03438	305	A00523	A03494	180
A00523	A03856	401	A00523	A03981	306
A00523	A04055	317	A00523	A04268	232
A00523	A05982	267	A00523	A10421	152
A00523	A10598	277	A00523	A14216	191
A00523	A14699	240	A00523	A14778	389
A00523	A15379	356	A00523	A15624	293
A00523	A15684	210	A00524	A00526	223
A00524	A00557	201	A00524	A00609	236
A00524	A00658	575	A00524	A00687	223
A00524	A00715	223	A00524	A00871	530
A00524	A00975	279	A00524	A00986	470
A00524	A01061	193	A00524	A01111	223
A00524	A01118	390	A00524	A01202	223
A00524	A01208	477	A00524	A01214	210
A00524	A01215	576	A00524	A01228	477
A00524	A01353	576	A00524	A01720	224
A00524	A01736	223	A00524	A01995	165
A00524	A02041	446	A00524	A02051	223
A00524	A02452	162	A00524	A02672	202
A00524	A03169	340	A00524	A03954	286
A00524	A03958	329	A00524	A03982	374
A00524	A03984	215	A00524	A04447	223
A00524	A04630	265	A00524	A04908	195
A00524	A07265	223	A00524	A07347	200
A00524	A07588	342	A00524	A08732	260
A00524	A08955	223	A00524	A08971	223
A00524	A10755	223	A00524	A11202	223
A00524	A12988	258	A00524	A13298	223
A00524	A14077	221	A00524	A14216	401
A00524	A15935	159	A00525	A00531	479
A00525	A00544	172	A00525	A00737	257
A00525	A00742	252	A00525	A00821	348
A00525	A00849	430	A00525	A00915	396

A00525	A00998	439	A00525	A01013	385
A00525	A01066	172	A00525	A01150	480
A00525	A01156	162	A00525	A01161	486
A00525	A01210	375	A00525	A01245	236
A00525	A01264	152	A00525	A01287	490
A00525	A01502	229	A00525	A01504	290
A00525	A01582	310	A00525	A01667	102
A00525	A01711	398	A00525	A01731	218
A00525	A01741	196	A00525	A01815	340
A00525	A01819	462	A00525	A01825	232
A00525	A01833	340	A00525	A02817	205
A00525	A02978	435	A00525	A02994	436
A00525	A03570	151	A00525	A03856	247
A00525	A03859	348	A00525	A04056	312
A00525	A05323	274	A00525	A05982	215
A00525	A06546	228	A00525	A07226	313
A00525	A07675	272	A00525	A08024	193
A00525	A08572	283	A00525	A09321	329
A00525	A11617	221	A00525	A14527	160
A00525	A14699	362	A00525	A14778	241
A00525	A15379	307	A00525	A15624	294
A00525	A15668	278	A00526	A00658	223
A00526	A00687	223	A00526	A00715	223
A00526	A00871	494	A00526	A00895	212
A00526	A00986	295	A00526	A01111	223
A00526	A01118	223	A00526	A01202	223
A00526	A01208	304	A00526	A01215	318
A00526	A01228	223	A00526	A01278	191
A00526	A01318	217	A00526	A01353	508
A00526	A01558	899	A00526	A01561	899
A00526	A01563	246	A00526	A01588	899
A00526	A01736	223	A00526	A02041	223
A00526	A02051	223	A00526	A02117	283
A00526	A02260	311	A00526	A02454	205
A00526	A03169	223	A00526	A04447	223
A00526	A04468	355	A00526	A04879	191
A00526	A06750	241	A00526	A07265	223
A00526	A07311	899	A00526	A07588	354
A00526	A08955	223	A00526	A08971	223
A00526	A10755	223	A00526	A11202	223
A00526	A13298	223	A00526	A14216	223
A00526	A14444	899	A00527	A00581	294
A00527	A00609	462	A00527	A00833	224
A00527	A00909	163	A00527	A00932	160
A00527	A00948	241	A00527	A01150	400
A00527	A01354	190	A00527	A01386	395

A00527	A01431	457	A00527	A01569	439
A00527	A01976	63	A00527	A02494	480
A00527	A02704	386	A00527	A03446	281
A00527	A03476	154	A00527	A03740	369
A00527	A03752	170	A00527	A06014	241
A00527	A06466	439	A00527	A07247	210
A00527	A07311	457	A00527	A07923	188
A00527	A08346	379	A00527	A09567	170
A00527	A11302	160	A00527	A11354	288
A00527	A12328	435	A00527	A13149	385
A00527	A13620	210	A00527	A14073	267
A00527	A14596	171	A00527	A14707	375
A00527	A15244	258	A00527	A15562	266
A00527	A17142	302	A00528	A01086	271
A00528	A01367	167	A00528	A01888	156
A00528	A02754	238	A00528	A02936	312
A00528	A03820	162	A00528	A04908	196
A00528	A07164	212	A00528	A07187	273
A00529	A00883	162	A00529	A01097	223
A00529	A08982	258	A00529	A08990	408
A00529	A14196	263	A00529	A15257	299
A00530	A00550	184	A00530	A00575	411
A00530	A00856	490	A00530	A01020	245
A00530	A01079	202	A00530	A01087	418
A00530	A01381	281	A00530	A01393	230
A00530	A01429	159	A00530	A01668	157
A00530	A02064	274	A00530	A03421	391
A00530	A03734	480	A00530	A07158	167
A00530	A07348	202	A00530	A09133	367
A00530	A15945	168	A00531	A00532	491
A00531	A00537	491	A00531	A00540	182
A00531	A00552	450	A00531	A00559	486
A00531	A00560	385	A00531	A00570	485
A00531	A00571	483	A00531	A00576	490
A00531	A00583	156	A00531	A00601	263
A00531	A00604	359	A00531	A00638	160
A00531	A00646	165	A00531	A00648	185
A00531	A00649	238	A00531	A00651	625
A00531	A00653	550	A00531	A00677	241
A00531	A00687	452	A00531	A00691	526
A00531	A00707	424	A00531	A00716	535
A00531	A00719	285	A00531	A00725	480
A00531	A00735	396	A00531	A00741	373
A00531	A00757	488	A00531	A00766	188
A00531	A00769	208	A00531	A00775	160
A00531	A00781	167	A00531	A00783	475

A00531	A00784	328	A00531	A00787	278
A00531	A00790	217	A00531	A00802	467
A00531	A00813	391	A00531	A00822	477
A00531	A00829	202	A00531	A00835	347
A00531	A00839	466	A00531	A00841	751
A00531	A00849	352	A00531	A00856	241
A00531	A00863	182	A00531	A00864	620
A00531	A00867	156	A00531	A00868	491
A00531	A00871	602	A00531	A00878	547
A00531	A00879	184	A00531	A00883	409
A00531	A00905	542	A00531	A00909	549
A00531	A00915	491	A00531	A00916	533
A00531	A00919	337	A00531	A00926	465
A00531	A00950	208	A00531	A00966	219
A00531	A00975	356	A00531	A00983	334
A00531	A00985	483	A00531	A00987	260
A00531	A01011	613	A00531	A01012	486
A00531	A01017	269	A00531	A01024	245
A00531	A01027	483	A00531	A01029	392
A00531	A01038	578	A00531	A01046	352
A00531	A01053	485	A00531	A01054	443
A00531	A01066	223	A00531	A01070	899
A00531	A01075	491	A00531	A01077	490
A00531	A01082	433	A00531	A01084	451
A00531	A01086	281	A00531	A01087	469
A00531	A01093	395	A00531	A01099	386
A00531	A01100	440	A00531	A01112	446
A00531	A01114	263	A00531	A01136	541
A00531	A01139	962	A00531	A01144	340
A00531	A01150	477	A00531	A01151	541
A00531	A01156	572	A00531	A01161	491
A00531	A01168	279	A00531	A01187	419
A00531	A01192	288	A00531	A01206	432
A00531	A01210	281	A00531	A01214	436
A00531	A01215	491	A00531	A01217	367
A00531	A01231	416	A00531	A01232	312
A00531	A01234	584	A00531	A01239	254
A00531	A01264	229	A00531	A01266	514
A00531	A01268	526	A00531	A01275	480
A00531	A01278	488	A00531	A01287	180
A00531	A01291	420	A00531	A01294	380
A00531	A01295	156	A00531	A01296	265
A00531	A01311	651	A00531	A01320	552
A00531	A01322	449	A00531	A01326	180
A00531	A01327	340	A00531	A01334	440
A00531	A01338	480	A00531	A01353	491

A00531	A01354	491	A00531	A01359	962
A00531	A01366	196	A00531	A01372	383
A00531	A01380	409	A00531	A01385	481
A00531	A01391	156	A00531	A01399	236
A00531	A01404	470	A00531	A01406	480
A00531	A01410	488	A00531	A01413	159
A00531	A01425	335	A00531	A01434	156
A00531	A01436	165	A00531	A01446	323
A00531	A01454	475	A00531	A01468	324
A00531	A01470	452	A00531	A01479	479
A00531	A01492	655	A00531	A01496	491
A00531	A01497	277	A00531	A01524	899
A00531	A01527	899	A00531	A01540	491
A00531	A01557	452	A00531	A01558	405
A00531	A01569	375	A00531	A01572	578
A00531	A01579	425	A00531	A01582	491
A00531	A01591	188	A00531	A01595	351
A00531	A01598	491	A00531	A01601	285
A00531	A01612	531	A00531	A01615	271
A00531	A01627	272	A00531	A01630	445
A00531	A01648	363	A00531	A01649	485
A00531	A01655	465	A00531	A01687	270
A00531	A01705	205	A00531	A01707	430
A00531	A01741	429	A00531	A01808	199
A00531	A01826	308	A00531	A01989	265
A00531	A02138	258	A00531	A02164	391
A00531	A02165	307	A00531	A02203	182
A00531	A02219	182	A00531	A02221	162
A00531	A02265	374	A00531	A02275	184
A00531	A02454	217	A00531	A02518	265
A00531	A02671	491	A00531	A02959	396
A00531	A02978	491	A00531	A03007	159
A00531	A03057	450	A00531	A03298	491
A00531	A03313	163	A00531	A03438	491
A00531	A03446	469	A00531	A03494	694
A00531	A03567	154	A00531	A03569	559
A00531	A03628	63	A00531	A03637	246
A00531	A03676	382	A00531	A03740	355
A00531	A03858	272	A00531	A03859	405
A00531	A03902	488	A00531	A03931	245
A00531	A03981	558	A00531	A04337	408
A00531	A04345	223	A00531	A04666	327
A00531	A04856	327	A00531	A04971	385
A00531	A05015	407	A00531	A05095	187
A00531	A06046	154	A00531	A06082	568
A00531	A06267	434	A00531	A06379	344

A00531	A06390	199	A00531	A06591	341
A00531	A06600	205	A00531	A06624	297
A00531	A06713	199	A00531	A06733	230
A00531	A06814	208	A00531	A06825	491
A00531	A07014	196	A00531	A07183	419
A00531	A07442	176	A00531	A07604	313
A00531	A07706	491	A00531	A07810	162
A00531	A07898	156	A00531	A08137	530
A00531	A08192	340	A00531	A08201	156
A00531	A08212	269	A00531	A08280	263
A00531	A08327	156	A00531	A08335	254
A00531	A08431	174	A00531	A08437	274
A00531	A08524	383	A00531	A08542	156
A00531	A08548	226	A00531	A08558	256
A00531	A08571	528	A00531	A08686	330
A00531	A08756	235	A00531	A08773	213
A00531	A08811	199	A00531	A08818	63
A00531	A08859	491	A00531	A08885	168
A00531	A08928	85	A00531	A09044	375
A00531	A09195	501	A00531	A09258	945
A00531	A09319	899	A00531	A09377	433
A00531	A09451	491	A00531	A09491	383
A00531	A09515	490	A00531	A09526	180
A00531	A09538	491	A00531	A09686	88
A00531	A09732	489	A00531	A09733	489
A00531	A09992	455	A00531	A10001	199
A00531	A10174	899	A00531	A10205	252
A00531	A10228	945	A00531	A10250	774
A00531	A10333	199	A00531	A10340	936
A00531	A10527	385	A00531	A10532	534
A00531	A10537	199	A00531	A10561	235
A00531	A10615	477	A00531	A10616	333
A00531	A10624	205	A00531	A10760	321
A00531	A10853	491	A00531	A10887	297
A00531	A10957	140	A00531	A11099	274
A00531	A11139	693	A00531	A11291	485
A00531	A11346	439	A00531	A11415	417
A00531	A11454	380	A00531	A11486	491
A00531	A11492	251	A00531	A11496	156
A00531	A11497	156	A00531	A11928	334
A00531	A11938	201	A00531	A12045	434
A00531	A12046	399	A00531	A12757	156
A00531	A12759	294	A00531	A12824	484
A00531	A12861	193	A00531	A12906	199
A00531	A13052	160	A00531	A13063	265
A00531	A13310	288	A00531	A13311	488

A00531	A13369	260	A00531	A13805	494
A00531	A13928	571	A00531	A13989	530
A00531	A14000	489	A00531	A14006	490
A00531	A14007	344	A00531	A14064	162
A00531	A14073	490	A00531	A14122	281
A00531	A14178	156	A00531	A14209	304
A00531	A14216	582	A00531	A14220	547
A00531	A14257	374	A00531	A14272	274
A00531	A14279	549	A00531	A14449	484
A00531	A14476	485	A00531	A14511	465
A00531	A14566	363	A00531	A14594	254
A00531	A14614	375	A00531	A14622	254
A00531	A14674	327	A00531	A14699	348
A00531	A14755	162	A00531	A14809	199
A00531	A14843	208	A00531	A15232	199
A00531	A15292	356	A00531	A15293	899
A00531	A15294	899	A00531	A15366	317
A00531	A15504	227	A00531	A15624	491
A00531	A15649	270	A00531	A15660	208
A00531	A15665	330	A00531	A15684	518
A00531	A15711	565	A00531	A15773	466
A00531	A15932	490	A00531	A15933	255
A00531	A16088	463	A00531	A16095	396
A00531	A16149	461	A00531	A16328	443
A00531	A16400	208	A00531	A16568	88
A00531	A16690	383	A00531	A16699	156
A00531	A16738	327	A00531	A16866	383
A00531	A16912	435	A00531	A17063	318
A00531	A17213	899	A00532	A00537	484
A00532	A00552	436	A00532	A00553	268
A00532	A00554	425	A00532	A00560	469
A00532	A00565	556	A00532	A00566	445
A00532	A00570	474	A00532	A00571	491
A00532	A00575	481	A00532	A00589	251
A00532	A00603	293	A00532	A00605	490
A00532	A00608	290	A00532	A00629	481
A00532	A00643	172	A00532	A00648	401
A00532	A00653	170	A00532	A00655	491
A00532	A00658	458	A00532	A00677	477
A00532	A00687	484	A00532	A00691	491
A00532	A00693	491	A00532	A00697	379
A00532	A00708	232	A00532	A00718	301
A00532	A00724	334	A00532	A00733	341
A00532	A00734	172	A00532	A00757	212
A00532	A00760	682	A00532	A00762	491
A00532	A00767	489	A00532	A00772	416

A00532	A00774	223	A00532	A00781	174
A00532	A00805	479	A00532	A00809	198
A00532	A00822	491	A00532	A00831	443
A00532	A00835	490	A00532	A00837	247
A00532	A00839	188	A00532	A00841	484
A00532	A00843	191	A00532	A00848	486
A00532	A00856	491	A00532	A00858	490
A00532	A00868	207	A00532	A00871	491
A00532	A00876	65	A00532	A00878	486
A00532	A00879	475	A00532	A00881	416
A00532	A00883	491	A00532	A00905	491
A00532	A00909	296	A00532	A00916	604
A00532	A00919	491	A00532	A00923	479
A00532	A00928	351	A00532	A00934	474
A00532	A00935	213	A00532	A00947	491
A00532	A00954	182	A00532	A00964	396
A00532	A00985	296	A00532	A00992	344
A00532	A00994	293	A00532	A01002	401
A00532	A01020	491	A00532	A01022	234
A00532	A01024	491	A00532	A01027	474
A00532	A01029	362	A00532	A01032	409
A00532	A01036	489	A00532	A01052	258
A00532	A01057	288	A00532	A01069	358
A00532	A01075	695	A00532	A01077	443
A00532	A01079	481	A00532	A01082	491
A00532	A01084	491	A00532	A01087	491
A00532	A01088	491	A00532	A01091	477
A00532	A01100	302	A00532	A01116	374
A00532	A01119	353	A00532	A01136	491
A00532	A01150	486	A00532	A01161	573
A00532	A01166	486	A00532	A01173	340
A00532	A01181	361	A00532	A01184	489
A00532	A01194	352	A00532	A01201	468
A00532	A01206	491	A00532	A01209	486
A00532	A01211	205	A00532	A01212	491
A00532	A01214	491	A00532	A01215	490
A00532	A01221	404	A00532	A01226	491
A00532	A01234	553	A00532	A01243	489
A00532	A01244	270	A00532	A01291	488
A00532	A01304	491	A00532	A01326	458
A00532	A01329	400	A00532	A01338	483
A00532	A01351	235	A00532	A01352	395
A00532	A01353	483	A00532	A01354	491
A00532	A01365	435	A00532	A01384	313
A00532	A01385	626	A00532	A01391	172
A00532	A01399	247	A00532	A01406	491

A00532	A01419	212	A00532	A01433	63
A00532	A01468	491	A00532	A01470	491
A00532	A01471	154	A00532	A01492	491
A00532	A01495	196	A00532	A01500	420
A00532	A01503	432	A00532	A01540	364
A00532	A01541	441	A00532	A01555	305
A00532	A01558	490	A00532	A01568	491
A00532	A01573	212	A00532	A01598	553
A00532	A01612	491	A00532	A01614	489
A00532	A01647	660	A00532	A01649	490
A00532	A01660	184	A00532	A01665	171
A00532	A01689	285	A00532	A01700	271
A00532	A01707	491	A00532	A01763	484
A00532	A01959	424	A00532	A02050	190
A00532	A02064	491	A00532	A02180	358
A00532	A02227	307	A00532	A02364	329
A00532	A02452	400	A00532	A02635	489
A00532	A02671	348	A00532	A02978	462
A00532	A03057	485	A00532	A03328	335
A00532	A03446	388	A00532	A03473	439
A00532	A03494	477	A00532	A03567	489
A00532	A03569	583	A00532	A03601	491
A00532	A03720	472	A00532	A03728	63
A00532	A03734	205	A00532	A03817	479
A00532	A03859	172	A00532	A03880	436
A00532	A03918	388	A00532	A03944	299
A00532	A04447	362	A00532	A04507	163
A00532	A04668	570	A00532	A05155	249
A00532	A05230	210	A00532	A05234	329
A00532	A05301	491	A00532	A05356	173
A00532	A05501	477	A00532	A05633	352
A00532	A06273	296	A00532	A06275	168
A00532	A06277	486	A00532	A06531	416
A00532	A06591	212	A00532	A06662	154
A00532	A06861	308	A00532	A06907	489
A00532	A07054	491	A00532	A07103	163
A00532	A07207	202	A00532	A07225	201
A00532	A07242	547	A00532	A07244	324
A00532	A07347	374	A00532	A07349	443
A00532	A07414	469	A00532	A07474	205
A00532	A07483	586	A00532	A07487	439
A00532	A07495	491	A00532	A07524	281
A00532	A07604	486	A00532	A07695	465
A00532	A07700	483	A00532	A07706	491
A00532	A07726	294	A00532	A07893	485
A00532	A07898	185	A00532	A07954	488

A00532	A08022	491	A00532	A08118	489
A00532	A08137	491	A00532	A08144	344
A00532	A08280	243	A00532	A08281	221
A00532	A08304	465	A00532	A08337	435
A00532	A08356	223	A00532	A08381	221
A00532	A08402	318	A00532	A08439	196
A00532	A08491	375	A00532	A08524	293
A00532	A08527	491	A00532	A08531	160
A00532	A08537	257	A00532	A08567	455
A00532	A08571	679	A00532	A08982	226
A00532	A09059	208	A00532	A09131	207
A00532	A09307	462	A00532	A09339	472
A00532	A09352	63	A00532	A09621	467
A00532	A09717	391	A00532	A09737	156
A00532	A09745	170	A00532	A09761	393
A00532	A09825	490	A00532	A10250	151
A00532	A10538	470	A00532	A10875	162
A00532	A11068	301	A00532	A11277	235
A00532	A11288	276	A00532	A11291	491
A00532	A11346	425	A00532	A11401	446
A00532	A11406	475	A00532	A11452	473
A00532	A11472	329	A00532	A11487	391
A00532	A11532	163	A00532	A11674	435
A00532	A11685	201	A00532	A11699	419
A00532	A11930	481	A00532	A12139	151
A00532	A12254	168	A00532	A12722	358
A00532	A12988	152	A00532	A13340	425
A00532	A13870	486	A00532	A13963	452
A00532	A13989	491	A00532	A14006	491
A00532	A14074	195	A00532	A14080	412
A00532	A14122	330	A00532	A14169	316
A00532	A14178	293	A00532	A14196	488
A00532	A14213	398	A00532	A14220	491
A00532	A14221	236	A00532	A14257	380
A00532	A14266	467	A00532	A14272	490
A00532	A14281	419	A00532	A14303	199
A00532	A14481	401	A00532	A14566	491
A00532	A14570	378	A00532	A14594	491
A00532	A14655	594	A00532	A14661	480
A00532	A14701	180	A00532	A14956	63
A00532	A15041	201	A00532	A15046	201
A00532	A15128	201	A00532	A15292	213
A00532	A15297	489	A00532	A15315	229
A00532	A15322	536	A00532	A15324	461
A00532	A15338	411	A00532	A15345	188
A00532	A15353	479	A00532	A15366	488

A00532	A15384	329	A00532	A15418	174
A00532	A15499	185	A00532	A15501	491
A00532	A15502	491	A00532	A15559	490
A00532	A15660	335	A00532	A15666	417
A00532	A15729	201	A00532	A15733	488
A00532	A15763	154	A00532	A15851	162
A00532	A15932	350	A00532	A15935	412
A00532	A15949	172	A00532	A16060	477
A00532	A16088	234	A00532	A16117	267
A00532	A16144	491	A00532	A16320	485
A00532	A16328	434	A00532	A16366	201
A00532	A16410	193	A00532	A16525	163
A00532	A16690	391	A00532	A17063	491
A00532	A17142	185	A00532	A17168	388
A00532	A17198	168	A00533	A00748	291
A00533	A00801	190	A00533	A00820	303
A00533	A00901	174	A00533	A00905	196
A00533	A00943	183	A00533	A01162	312
A00533	A01451	491	A00533	A01558	452
A00533	A01588	296	A00533	A01615	474
A00533	A01636	477	A00533	A01640	438
A00533	A01665	201	A00533	A01712	486
A00533	A01844	205	A00533	A02531	163
A00533	A03186	266	A00533	A03502	218
A00533	A03821	151	A00533	A03878	229
A00533	A04207	208	A00533	A04751	486
A00533	A04784	238	A00533	A04937	190
A00533	A05216	302	A00533	A05699	238
A00533	A06597	483	A00533	A06819	490
A00533	A06834	160	A00533	A07528	296
A00533	A07834	433	A00533	A08571	480
A00533	A09319	121	A00533	A10894	469
A00533	A13844	234	A00533	A13991	162
A00533	A14006	330	A00533	A14193	162
A00533	A14221	202	A00533	A15315	418
A00533	A15347	297	A00534	A00885	481
A00534	A00886	221	A00534	A00889	491
A00534	A00893	152	A00534	A00895	337
A00534	A00898	491	A00534	A00986	156
A00534	A01303	151	A00534	A01927	193
A00534	A03151	441	A00534	A06673	469
A00534	A06992	283	A00534	A08435	473
A00534	A08891	486	A00534	A14174	491
A00534	A14463	324	A00535	A00560	278
A00535	A00566	475	A00535	A00760	316
A00535	A00805	676	A00535	A00856	481

A00535	A00858	491	A00535	A00883	165
A00535	A00903	688	A00535	A00954	688
A00535	A00964	394	A00535	A01075	185
A00535	A01143	473	A00535	A01185	435
A00535	A01401	152	A00535	A01431	182
A00535	A01451	377	A00535	A01457	230
A00535	A01458	173	A00535	A01483	154
A00535	A01500	826	A00535	A01503	762
A00535	A01572	190	A00535	A01573	366
A00535	A01574	174	A00535	A01598	205
A00535	A02202	152	A00535	A03435	263
A00535	A05424	302	A00535	A07004	402
A00535	A08022	490	A00535	A08382	447
A00535	A08439	187	A00535	A08527	168
A00535	A08553	688	A00535	A08564	670
A00535	A08565	626	A00535	A09010	688
A00535	A10420	249	A00535	A11301	363
A00535	A11395	486	A00535	A11439	201
A00535	A12365	477	A00535	A12660	305
A00535	A13398	191	A00535	A13989	451
A00535	A14085	185	A00535	A14147	283
A00535	A14214	378	A00535	A14462	171
A00535	A15366	221	A00535	A15373	676
A00535	A15626	378	A00535	A15666	307
A00535	A15749	491	A00535	A15852	159
A00535	A15943	457	A00535	A15946	483
A00535	A16109	429	A00535	A16144	400
A00535	A16212	291	A00536	A00651	306
A00536	A00994	193	A00536	A01150	241
A00536	A01585	218	A00536	A01743	208
A00536	A02859	424	A00536	A02983	338
A00536	A03259	408	A00536	A03614	274
A00536	A03794	489	A00536	A03959	185
A00536	A04217	160	A00536	A04232	210
A00536	A05449	747	A00536	A07050	491
A00536	A07585	456	A00536	A08137	165
A00536	A11297	486	A00536	A11331	160
A00536	A11357	368	A00536	A11358	238
A00536	A11981	180	A00536	A12927	162
A00536	A14071	205	A00536	A14216	210
A00536	A15019	308	A00536	A15188	475
A00536	A15498	394	A00536	A15507	680
A00536	A15522	262	A00536	A15547	704
A00536	A15624	229	A00536	A15657	481
A00536	A15676	196	A00536	A15684	423
A00536	A15769	460	A00536	A15773	193

A00536	A15794	491	A00536	A16822	345
A00537	A00538	491	A00537	A00555	252
A00537	A00561	417	A00537	A00564	348
A00537	A00566	183	A00537	A00571	486
A00537	A00572	480	A00537	A00590	470
A00537	A00598	491	A00537	A00600	450
A00537	A00601	491	A00537	A00603	480
A00537	A00604	491	A00537	A00605	486
A00537	A00629	240	A00537	A00648	489
A00537	A00649	366	A00537	A00653	573
A00537	A00687	229	A00537	A00693	407
A00537	A00697	785	A00537	A00698	199
A00537	A00703	224	A00537	A00741	491
A00537	A00742	380	A00537	A00758	491
A00537	A00760	152	A00537	A00766	240
A00537	A00782	329	A00537	A00783	167
A00537	A00787	491	A00537	A00809	730
A00537	A00813	490	A00537	A00821	317
A00537	A00839	491	A00537	A00849	241
A00537	A00865	291	A00537	A00868	491
A00537	A00871	489	A00537	A00878	491
A00537	A00890	348	A00537	A00892	484
A00537	A00911	498	A00537	A00915	460
A00537	A00922	407	A00537	A00926	488
A00537	A00935	653	A00537	A00953	490
A00537	A00965	341	A00537	A00975	446
A00537	A00980	177	A00537	A00985	601
A00537	A00987	390	A00537	A00994	174
A00537	A00998	174	A00537	A01020	475
A00537	A01024	606	A00537	A01025	445
A00537	A01027	481	A00537	A01034	452
A00537	A01037	193	A00537	A01046	345
A00537	A01050	263	A00537	A01067	377
A00537	A01071	157	A00537	A01075	283
A00537	A01082	466	A00537	A01086	447
A00537	A01095	458	A00537	A01099	380
A00537	A01100	369	A00537	A01108	486
A00537	A01139	361	A00537	A01149	226
A00537	A01150	649	A00537	A01152	321
A00537	A01153	226	A00537	A01154	221
A00537	A01155	340	A00537	A01161	708
A00537	A01169	461	A00537	A01192	390
A00537	A01210	491	A00537	A01214	472
A00537	A01220	156	A00537	A01221	198
A00537	A01234	489	A00537	A01247	483
A00537	A01261	345	A00537	A01263	491

A00537	A01287	247	A00537	A01291	517
A00537	A01320	185	A00537	A01322	491
A00537	A01327	254	A00537	A01329	254
A00537	A01335	241	A00537	A01336	489
A00537	A01347	273	A00537	A01352	433
A00537	A01365	472	A00537	A01369	377
A00537	A01379	358	A00537	A01392	475
A00537	A01399	446	A00537	A01404	490
A00537	A01411	430	A00537	A01413	491
A00537	A01415	484	A00537	A01416	283
A00537	A01433	474	A00537	A01435	491
A00537	A01436	424	A00537	A01447	491
A00537	A01449	180	A00537	A01515	401
A00537	A01539	283	A00537	A01540	491
A00537	A01542	163	A00537	A01573	489
A00537	A01578	252	A00537	A01596	483
A00537	A01601	403	A00537	A01707	481
A00537	A01711	168	A00537	A01741	486
A00537	A01815	283	A00537	A01827	489
A00537	A01850	385	A00537	A01926	465
A00537	A01988	170	A00537	A02040	299
A00537	A02086	274	A00537	A02097	193
A00537	A02225	451	A00537	A02227	196
A00537	A02524	398	A00537	A02607	491
A00537	A02978	180	A00537	A02994	488
A00537	A03223	227	A00537	A03259	278
A00537	A03298	223	A00537	A03401	204
A00537	A03490	63	A00537	A03494	475
A00537	A03651	581	A00537	A03676	486
A00537	A03740	480	A00537	A03752	443
A00537	A03770	243	A00537	A03798	382
A00537	A03959	488	A00537	A03981	216
A00537	A04397	273	A00537	A04642	255
A00537	A04668	490	A00537	A04686	333
A00537	A04782	462	A00537	A04838	455
A00537	A05146	167	A00537	A05417	491
A00537	A05419	454	A00537	A05501	299
A00537	A05557	265	A00537	A05812	637
A00537	A06014	469	A00537	A06046	221
A00537	A06158	243	A00537	A06297	317
A00537	A06466	480	A00537	A06478	180
A00537	A06545	449	A00537	A06546	486
A00537	A06733	290	A00537	A06754	238
A00537	A06783	396	A00537	A07360	470
A00537	A07394	491	A00537	A07686	273
A00537	A07695	371	A00537	A07697	489

A00537	A07706	390	A00537	A07767	335
A00537	A07902	338	A00537	A08011	481
A00537	A08131	390	A00537	A08222	441
A00537	A08319	473	A00537	A08519	489
A00537	A08543	422	A00537	A08550	205
A00537	A08556	422	A00537	A08567	311
A00537	A08745	188	A00537	A08773	488
A00537	A08812	290	A00537	A08831	198
A00537	A08967	163	A00537	A08975	562
A00537	A09054	480	A00537	A09581	486
A00537	A09595	238	A00537	A09709	391
A00537	A09735	446	A00537	A10373	468
A00537	A10527	486	A00537	A10535	401
A00537	A10537	238	A00537	A10598	279
A00537	A10853	372	A00537	A11041	201
A00537	A11242	283	A00537	A11307	255
A00537	A11313	671	A00537	A11526	491
A00537	A11597	196	A00537	A11688	172
A00537	A11728	271	A00537	A11833	481
A00537	A11860	260	A00537	A12027	201
A00537	A12140	486	A00537	A12170	391
A00537	A12326	486	A00537	A12480	238
A00537	A12507	444	A00537	A12824	491
A00537	A12997	152	A00537	A13116	294
A00537	A13340	379	A00537	A13398	310
A00537	A13446	201	A00537	A13524	163
A00537	A13809	474	A00537	A13963	396
A00537	A14073	470	A00537	A14077	172
A00537	A14084	603	A00537	A14089	201
A00537	A14167	271	A00537	A14178	409
A00537	A14216	352	A00537	A14230	246
A00537	A14272	361	A00537	A14478	177
A00537	A14479	324	A00537	A14482	470
A00537	A14502	419	A00537	A14507	374
A00537	A14547	283	A00537	A14566	481
A00537	A14568	198	A00537	A14642	201
A00537	A14655	488	A00537	A14701	435
A00537	A14845	449	A00537	A15161	238
A00537	A15500	172	A00537	A15524	224
A00537	A15531	260	A00537	A15617	165
A00537	A15624	450	A00537	A15653	491
A00537	A15661	367	A00537	A15666	491
A00537	A15765	491	A00537	A15771	283
A00537	A15932	441	A00537	A15988	165
A00537	A15992	401	A00537	A16060	243
A00537	A16157	491	A00537	A16162	394

A00537	A16318	193	A00537	A16403	339
A00537	A16430	230	A00537	A16437	249
A00537	A16662	152	A00537	A16676	185
A00537	A16683	283	A00537	A16813	180
A00538	A00604	454	A00538	A00649	274
A00538	A00697	491	A00538	A00741	484
A00538	A00758	460	A00538	A00809	411
A00538	A00935	491	A00538	A00985	374
A00538	A01095	235	A00538	A01153	165
A00538	A01161	491	A00538	A01210	491
A00538	A01336	162	A00538	A01435	451
A00538	A01436	245	A00538	A01447	473
A00538	A01741	475	A00538	A02225	445
A00538	A02333	357	A00538	A02607	491
A00538	A02996	312	A00538	A03401	198
A00538	A05417	454	A00538	A05445	198
A00538	A05812	374	A00538	A06014	404
A00538	A06158	353	A00538	A06424	351
A00538	A06545	274	A00538	A06546	486
A00538	A07196	291	A00538	A07394	489
A00538	A07605	154	A00538	A08319	249
A00538	A08519	165	A00538	A08938	306
A00538	A09581	474	A00538	A09709	232
A00538	A10598	193	A00538	A11101	202
A00538	A11307	180	A00538	A11313	362
A00538	A11526	485	A00538	A11712	338
A00538	A12140	391	A00538	A12326	440
A00538	A12454	371	A00538	A12480	371
A00538	A13809	286	A00538	A15052	245
A00538	A15653	380	A00538	A15666	187
A00538	A15765	308	A00538	A16157	165
A00538	A16430	218	A00539	A00550	246
A00539	A00599	255	A00539	A00667	210
A00539	A00687	489	A00539	A00712	334
A00539	A00829	246	A00539	A00839	486
A00539	A00859	223	A00539	A00876	479
A00539	A00911	249	A00539	A00922	335
A00539	A00930	224	A00539	A00943	205
A00539	A00947	196	A00539	A00994	334
A00539	A01110	334	A00539	A01119	121
A00539	A01154	245	A00539	A01189	180
A00539	A01215	224	A00539	A01385	271
A00539	A01411	283	A00539	A01420	238
A00539	A01720	307	A00539	A01822	441
A00539	A01824	491	A00539	A01831	334
A00539	A02304	152	A00539	A02353	210

A00539	A02602	334	A00539	A03453	183
A00539	A03490	480	A00539	A03601	307
A00539	A03752	179	A00539	A03881	435
A00539	A04495	232	A00539	A04622	152
A00539	A04666	171	A00539	A05017	334
A00539	A05417	227	A00539	A06000	188
A00539	A06273	152	A00539	A06365	307
A00539	A06425	520	A00539	A06466	470
A00539	A06827	506	A00539	A06975	328
A00539	A07206	217	A00539	A07311	400
A00539	A07531	498	A00539	A07581	224
A00539	A08201	334	A00539	A08301	485
A00539	A08408	353	A00539	A08419	302
A00539	A09297	218	A00539	A09587	391
A00539	A11344	201	A00539	A11688	213
A00539	A12328	234	A00539	A12329	238
A00539	A13340	454	A00539	A13375	468
A00539	A14132	294	A00539	A14594	154
A00539	A15624	485	A00539	A15651	334
A00539	A15750	334	A00539	A15917	399
A00539	A15932	638	A00539	A15933	375
A00539	A15937	334	A00539	A15951	313
A00540	A00557	223	A00540	A00563	276
A00540	A00570	472	A00540	A00594	313
A00540	A00687	468	A00540	A00689	294
A00540	A00703	263	A00540	A00709	486
A00540	A00735	307	A00540	A00829	160
A00540	A00854	329	A00540	A00865	165
A00540	A00913	241	A00540	A00972	213
A00540	A01053	277	A00540	A01102	310
A00540	A01154	159	A00540	A01159	435
A00540	A01278	201	A00540	A01323	227
A00540	A01352	157	A00540	A01418	436
A00540	A01598	204	A00540	A01704	334
A00540	A01826	340	A00540	A01976	283
A00540	A02164	474	A00540	A02203	418
A00540	A02252	276	A00540	A02268	215
A00540	A02454	213	A00540	A02812	168
A00540	A02847	154	A00540	A03438	238
A00540	A03901	226	A00540	A03907	416
A00540	A04308	338	A00540	A05646	321
A00540	A06017	338	A00540	A06122	405
A00540	A06369	379	A00540	A06825	383
A00540	A06832	450	A00540	A07309	180
A00540	A07312	259	A00540	A07677	223
A00540	A14594	193	A00540	A15649	156

A00540	A15651	224	A00540	A15918	204
A00540	A15932	414	A00541	A00565	223
A00541	A00573	380	A00541	A00574	491
A00541	A00614	242	A00541	A00628	463
A00541	A00633	228	A00541	A00654	491
A00541	A00662	340	A00541	A00666	489
A00541	A00670	223	A00541	A00681	490
A00541	A00695	452	A00541	A00696	461
A00541	A00738	454	A00541	A00739	486
A00541	A00770	418	A00541	A00771	430
A00541	A00773	384	A00541	A00825	460
A00541	A00962	491	A00541	A00968	223
A00541	A00980	355	A00541	A00995	477
A00541	A01005	210	A00541	A01008	491
A00541	A01018	481	A00541	A01074	223
A00541	A01109	378	A00541	A01174	490
A00541	A01175	171	A00541	A01189	223
A00541	A01222	177	A00541	A01223	427
A00541	A01227	332	A00541	A01230	238
A00541	A01233	491	A00541	A01256	334
A00541	A01297	479	A00541	A01360	411
A00541	A01397	238	A00541	A01409	462
A00541	A01481	283	A00541	A01508	319
A00541	A01511	177	A00541	A01635	491
A00541	A01730	252	A00541	A01741	452
A00541	A01743	413	A00541	A01972	217
A00541	A02026	157	A00541	A02053	195
A00541	A02071	341	A00541	A02082	196
A00541	A02114	185	A00541	A02148	475
A00541	A02254	221	A00541	A02330	160
A00541	A02335	523	A00541	A02432	221
A00541	A02439	171	A00541	A02476	283
A00541	A02789	223	A00541	A02927	441
A00541	A02992	182	A00541	A03004	484
A00541	A03050	162	A00541	A03095	238
A00541	A03115	168	A00541	A03167	461
A00541	A03168	346	A00541	A03186	190
A00541	A03188	154	A00541	A03259	276
A00541	A03477	474	A00541	A03484	357
A00541	A03487	458	A00541	A03513	483
A00541	A03591	201	A00541	A03609	157
A00541	A03719	223	A00541	A03728	223
A00541	A03752	223	A00541	A03804	228
A00541	A03858	173	A00541	A03968	165
A00541	A04087	322	A00541	A04307	491
A00541	A04556	185	A00541	A04570	210

A00541	A04674	270	A00541	A04779	157
A00541	A04791	446	A00541	A04834	301
A00541	A04880	157	A00541	A04937	196
A00541	A04995	472	A00541	A05006	407
A00541	A05007	463	A00541	A05045	347
A00541	A05050	217	A00541	A05088	235
A00541	A05103	235	A00541	A05138	475
A00541	A05277	491	A00541	A05310	395
A00541	A05358	302	A00541	A05416	401
A00541	A05418	258	A00541	A05424	263
A00541	A05451	228	A00541	A05463	429
A00541	A05471	474	A00541	A05472	491
A00541	A05517	174	A00541	A05548	324
A00541	A05550	249	A00541	A05555	279
A00541	A05573	473	A00541	A05660	256
A00541	A05713	343	A00541	A05753	288
A00541	A05754	391	A00541	A05759	372
A00541	A05767	469	A00541	A05768	490
A00541	A05771	375	A00541	A05774	337
A00541	A05789	217	A00541	A05792	318
A00541	A06018	318	A00541	A06091	445
A00541	A06282	249	A00541	A06358	218
A00541	A06359	417	A00541	A06360	240
A00541	A06397	249	A00541	A06405	375
A00541	A06420	288	A00541	A06439	457
A00541	A06523	484	A00541	A06629	380
A00541	A06632	469	A00541	A06633	160
A00541	A06635	353	A00541	A06640	281
A00541	A06641	290	A00541	A06689	577
A00541	A06692	352	A00541	A06700	486
A00541	A06716	343	A00541	A06819	223
A00541	A06847	182	A00541	A06860	394
A00541	A06912	491	A00541	A06916	491
A00541	A06970	443	A00541	A06979	286
A00541	A06981	185	A00541	A07027	152
A00541	A07059	308	A00541	A07061	491
A00541	A07077	343	A00541	A07080	226
A00541	A07088	324	A00541	A07109	491
A00541	A07122	481	A00541	A07222	223
A00541	A07238	491	A00541	A07239	435
A00541	A07256	221	A00541	A07267	443
A00541	A07287	318	A00541	A07304	157
A00541	A07322	191	A00541	A07333	223
A00541	A07376	191	A00541	A07381	418
A00541	A07424	466	A00541	A07477	450
A00541	A07520	446	A00541	A07530	182

A00541	A07553	356	A00541	A07558	375
A00541	A07590	470	A00541	A07710	213
A00541	A07718	208	A00541	A07736	452
A00541	A07800	249	A00541	A07848	489
A00541	A07894	162	A00541	A07933	443
A00541	A08023	475	A00541	A08083	195
A00541	A08127	481	A00541	A08155	208
A00541	A08379	417	A00541	A08474	343
A00541	A08667	177	A00541	A08751	221
A00541	A08905	154	A00541	A09211	160
A00541	A09879	205	A00541	A10020	301
A00541	A10326	343	A00541	A10420	451
A00541	A10443	329	A00541	A10444	279
A00541	A10456	473	A00541	A10475	205
A00541	A10550	208	A00541	A10656	355
A00541	A10658	195	A00541	A10659	235
A00541	A10677	332	A00541	A10681	182
A00541	A10694	461	A00541	A10703	288
A00541	A10715	180	A00541	A10883	318
A00541	A10904	279	A00541	A11309	277
A00541	A13471	343	A00541	A13731	318
A00541	A13822	223	A00541	A14525	223
A00541	A14652	151	A00541	A14803	396
A00541	A14804	223	A00541	A14827	152
A00541	A14828	263	A00541	A15372	223
A00541	A15497	174	A00541	A15874	491
A00541	A15905	446	A00541	A15970	310
A00541	A16026	276	A00541	A16027	480
A00541	A16219	223	A00541	A16355	419
A00541	A16419	322	A00541	A16529	307
A00541	A16531	223	A00541	A16739	488
A00541	A16741	491	A00541	A16744	490
A00541	A16959	398	A00541	A17078	167
A00542	A00545	450	A00542	A00625	485
A00542	A00786	491	A00542	A01191	218
A00542	A01282	343	A00542	A01345	477
A00542	A01366	441	A00542	A01454	491
A00542	A01478	281	A00542	A01484	160
A00542	A01568	488	A00542	A01645	165
A00542	A01681	281	A00542	A01687	485
A00542	A03321	172	A00542	A04845	193
A00542	A04903	184	A00542	A05075	326
A00542	A05944	380	A00542	A06100	217
A00542	A06162	168	A00542	A06661	391
A00542	A07054	475	A00542	A07242	491
A00542	A07495	438	A00542	A07709	488

A00542	A07816	400	A00542	A07934	269
A00542	A08094	241	A00542	A08418	151
A00542	A08986	254	A00542	A09342	245
A00542	A09666	425	A00542	A09774	283
A00542	A11150	259	A00542	A11473	241
A00542	A11513	163	A00542	A11684	263
A00542	A11986	210	A00542	A12047	232
A00542	A12324	350	A00542	A12722	491
A00542	A14281	219	A00542	A14484	176
A00542	A14783	157	A00542	A16261	322
A00543	A01042	168	A00543	A01192	232
A00543	A13255	400	A00543	A13992	265
A00543	A14073	174	A00544	A00564	391
A00544	A00569	429	A00544	A00576	576
A00544	A00581	171	A00544	A00604	306
A00544	A00640	152	A00544	A00649	207
A00544	A00653	577	A00544	A00726	299
A00544	A00758	232	A00544	A00810	152
A00544	A00833	299	A00544	A00915	311
A00544	A00953	306	A00544	A00974	299
A00544	A00984	299	A00544	A00985	234
A00544	A00987	619	A00544	A00994	374
A00544	A01001	595	A00544	A01038	208
A00544	A01102	567	A00544	A01108	358
A00544	A01156	156	A00544	A01311	152
A00544	A01379	453	A00544	A01387	299
A00544	A01439	299	A00544	A01446	404
A00544	A01448	391	A00544	A01577	152
A00544	A01627	468	A00544	A01644	414
A00544	A02601	299	A00544	A03676	411
A00544	A03677	187	A00544	A03697	152
A00544	A03981	271	A00544	A04495	299
A00544	A04497	266	A00544	A04931	205
A00544	A05323	473	A00544	A05570	351
A00544	A06046	238	A00544	A06173	367
A00544	A06287	152	A00544	A07360	241
A00544	A07531	373	A00544	A07744	152
A00544	A07773	156	A00544	A08061	152
A00544	A08098	152	A00544	A08178	152
A00544	A08763	152	A00544	A08947	299
A00544	A09004	299	A00544	A10199	286
A00544	A10842	152	A00544	A14216	249
A00544	A14525	152	A00544	A14527	154
A00544	A15375	152	A00544	A15379	168
A00544	A15546	152	A00544	A15624	399
A00544	A15651	461	A00544	A15661	441

A00544	A15684	229	A00544	A15932	151
A00544	A15992	167	A00545	A00691	160
A00545	A00786	491	A00545	A01282	243
A00545	A01345	491	A00545	A01454	486
A00545	A01479	224	A00545	A02846	162
A00545	A02895	184	A00545	A05568	160
A00545	A05894	490	A00545	A06100	216
A00545	A07242	299	A00545	A07709	417
A00545	A08986	374	A00545	A09662	374
A00545	A09666	157	A00545	A11283	221
A00545	A11986	340	A00545	A12099	319
A00545	A12225	319	A00545	A12226	340
A00545	A12722	416	A00545	A14661	308
A00545	A15533	184	A00546	A00562	491
A00546	A00625	491	A00546	A00643	477
A00546	A00652	486	A00546	A00668	213
A00546	A00732	420	A00546	A00733	321
A00546	A00786	491	A00546	A00864	490
A00546	A00877	491	A00546	A00951	491
A00546	A00971	491	A00546	A00981	290
A00546	A01003	257	A00546	A01011	408
A00546	A01036	489	A00546	A01216	491
A00546	A01286	382	A00546	A01290	491
A00546	A01345	200	A00546	A01348	491
A00546	A01366	491	A00546	A01451	477
A00546	A01454	491	A00546	A01459	396
A00546	A01478	319	A00546	A01479	438
A00546	A01514	491	A00546	A01568	491
A00546	A01580	281	A00546	A01687	491
A00546	A01698	160	A00546	A01723	462
A00546	A01790	462	A00546	A01912	290
A00546	A02020	450	A00546	A02698	196
A00546	A03018	74	A00546	A03304	170
A00546	A03451	466	A00546	A03478	267
A00546	A03512	340	A00546	A03819	215
A00546	A03880	343	A00546	A03922	217
A00546	A04034	190	A00546	A04111	177
A00546	A04449	449	A00546	A04533	246
A00546	A04715	491	A00546	A04790	457
A00546	A04845	152	A00546	A04905	393
A00546	A05301	344	A00546	A05438	490
A00546	A05918	473	A00546	A06021	277
A00546	A06277	491	A00546	A06591	480
A00546	A06752	490	A00546	A06778	490
A00546	A07004	488	A00546	A07054	481
A00546	A07083	167	A00546	A07231	152

A00546	A07242	491	A00546	A07444	183
A00546	A07483	466	A00546	A07495	491
A00546	A07665	486	A00546	A07696	491
A00546	A07709	491	A00546	A07732	466
A00546	A07816	485	A00546	A07893	347
A00546	A07897	344	A00546	A07903	160
A00546	A07934	491	A00546	A07944	160
A00546	A08409	474	A00546	A08464	190
A00546	A08507	251	A00546	A08531	443
A00546	A08536	238	A00546	A08551	457
A00546	A08878	490	A00546	A08917	170
A00546	A09311	491	A00546	A09666	347
A00546	A09671	451	A00546	A09859	208
A00546	A09995	491	A00546	A10264	208
A00546	A10315	235	A00546	A10358	165
A00546	A10540	177	A00546	A11034	288
A00546	A11057	375	A00546	A11155	256
A00546	A11160	252	A00546	A11161	208
A00546	A11183	183	A00546	A11187	446
A00546	A11201	447	A00546	A11209	488
A00546	A11281	406	A00546	A11292	489
A00546	A11346	483	A00546	A11576	332
A00546	A11689	323	A00546	A11725	223
A00546	A11727	288	A00546	A11983	490
A00546	A12024	208	A00546	A12047	491
A00546	A12333	288	A00546	A12511	288
A00546	A12584	375	A00546	A13479	235
A00546	A13513	424	A00546	A13917	274
A00546	A13942	152	A00546	A14266	491
A00546	A14270	179	A00546	A14278	235
A00546	A14281	491	A00546	A14358	190
A00546	A14474	305	A00546	A14484	451
A00546	A14633	489	A00546	A14661	491
A00546	A15004	290	A00546	A15010	183
A00546	A15187	407	A00546	A15282	489
A00546	A15367	398	A00546	A15626	480
A00546	A16423	199	A00546	A16611	176
A00546	A16626	243	A00546	A16664	187
A00546	A16824	152	A00547	A00571	451
A00547	A00603	104	A00547	A00608	245
A00547	A00648	76	A00547	A00653	388
A00547	A00698	196	A00547	A00750	296
A00547	A00782	260	A00547	A00839	395
A00547	A00878	420	A00547	A00949	265
A00547	A00955	199	A00547	A01121	386
A00547	A01153	436	A00547	A01154	371

A00547	A01186	382	A00547	A01306	229
A00547	A01335	469	A00547	A01337	271
A00547	A01365	347	A00547	A01408	240
A00547	A01449	247	A00547	A03490	255
A00547	A03493	259	A00547	A03770	440
A00547	A05499	274	A00547	A05501	439
A00547	A08358	218	A00547	A09735	330
A00547	A11616	274	A00547	A12011	323
A00547	A12821	263	A00547	A14479	224
A00547	A14482	160	A00547	A14568	351
A00547	A14813	330	A00547	A15500	299
A00547	A15771	230	A00548	A00688	241
A00548	A00751	483	A00548	A00761	249
A00548	A00766	429	A00548	A00769	170
A00548	A00781	172	A00548	A00791	479
A00548	A00816	151	A00548	A00841	190
A00548	A00868	488	A00548	A00883	491
A00548	A00884	481	A00548	A00909	272
A00548	A00922	168	A00548	A00926	491
A00548	A00975	446	A00548	A00983	386
A00548	A01004	241	A00548	A01077	429
A00548	A01106	152	A00548	A01122	386
A00548	A01169	486	A00548	A01232	165
A00548	A01339	467	A00548	A01340	240
A00548	A01370	481	A00548	A01417	241
A00548	A01458	227	A00548	A01613	441
A00548	A01661	241	A00548	A02049	154
A00548	A02416	241	A00548	A05258	159
A00548	A05276	226	A00548	A06411	335
A00548	A06750	409	A00548	A07101	297
A00548	A07226	272	A00548	A07360	188
A00548	A07457	156	A00548	A07642	165
A00548	A07708	484	A00548	A07756	463
A00548	A08490	338	A00548	A08861	241
A00548	A09242	245	A00548	A09331	355
A00548	A10576	210	A00548	A12255	191
A00548	A12824	490	A00548	A12863	240
A00548	A14299	241	A00548	A14699	296
A00548	A15297	485	A00548	A15323	168
A00548	A15498	468	A00548	A15662	224
A00548	A15937	460	A00548	A16671	286
A00549	A00966	379	A00549	A02350	215
A00549	A02737	267	A00549	A02825	327
A00549	A03720	296	A00549	A05838	313
A00549	A08867	360	A00550	A00554	461
A00550	A00555	232	A00550	A00605	385

A00550	A00690	481	A00550	A00742	246
A00550	A00831	392	A00550	A00911	224
A00550	A00916	490	A00550	A00930	232
A00550	A00934	473	A00550	A00944	210
A00550	A01020	636	A00550	A01040	370
A00550	A01079	243	A00550	A01112	334
A00550	A01116	631	A00550	A01177	228
A00550	A01184	491	A00550	A01201	435
A00550	A01206	345	A00550	A01234	491
A00550	A01304	633	A00550	A01351	152
A00550	A01385	283	A00550	A01470	412
A00550	A01492	465	A00550	A01495	334
A00550	A01503	174	A00550	A01539	486
A00550	A01598	281	A00550	A06340	165
A00550	A07223	637	A00550	A07347	491
A00550	A07533	276	A00550	A07584	457
A00550	A07661	368	A00550	A07700	635
A00550	A07706	190	A00550	A07725	636
A00550	A07850	160	A00550	A08491	433
A00550	A09664	174	A00550	A09705	447
A00550	A12419	205	A00550	A13989	469
A00550	A14936	180	A00550	A15354	154
A00550	A15735	469	A00550	A15739	399
A00551	A00554	490	A00551	A00589	156
A00551	A00708	676	A00551	A00760	170
A00551	A00856	362	A00551	A00858	422
A00551	A00903	152	A00551	A00916	632
A00551	A00934	694	A00551	A00954	473
A00551	A01020	747	A00551	A01075	402
A00551	A01184	483	A00551	A01206	687
A00551	A01213	462	A00551	A01226	172
A00551	A01234	491	A00551	A01304	477
A00551	A01349	438	A00551	A01429	514
A00551	A01434	283	A00551	A01500	446
A00551	A01503	170	A00551	A01598	341
A00551	A02049	375	A00551	A02925	311
A00551	A04033	266	A00551	A06273	180
A00551	A06881	221	A00551	A07347	424
A00551	A07348	438	A00551	A07524	652
A00551	A07584	199	A00551	A07700	274
A00551	A07706	204	A00551	A08022	240
A00551	A08024	146	A00551	A08211	695
A00551	A08481	245	A00551	A08491	165
A00551	A08553	286	A00551	A08807	601
A00551	A09010	335	A00551	A09619	491
A00551	A09664	174	A00551	A09880	384

A00551	A10456	475	A00551	A11291	160
A00551	A11395	179	A00551	A12138	438
A00551	A13989	603	A00551	A14505	185
A00551	A14635	384	A00551	A14655	489
A00551	A14826	364	A00551	A15580	296
A00551	A15733	162	A00551	A15764	160
A00551	A15946	152	A00551	A16209	180
A00552	A00571	190	A00552	A00573	347
A00552	A00588	486	A00552	A00669	307
A00552	A00680	445	A00552	A00696	491
A00552	A00718	329	A00552	A00719	383
A00552	A00725	210	A00552	A00757	480
A00552	A00825	383	A00552	A00887	256
A00552	A00981	425	A00552	A01052	491
A00552	A01064	335	A00552	A01070	350
A00552	A01075	281	A00552	A01093	483
A00552	A01099	411	A00552	A01173	183
A00552	A01235	176	A00552	A01406	343
A00552	A01426	383	A00552	A01468	402
A00552	A01524	188	A00552	A01527	213
A00552	A01558	484	A00552	A01559	480
A00552	A01572	488	A00552	A01660	443
A00552	A01707	262	A00552	A01730	466
A00552	A01790	230	A00552	A01995	664
A00552	A02117	384	A00552	A02152	173
A00552	A02156	187	A00552	A02482	480
A00552	A02715	199	A00552	A03292	480
A00552	A03436	486	A00552	A03611	273
A00552	A03749	271	A00552	A03968	383
A00552	A03990	491	A00552	A04012	165
A00552	A04157	170	A00552	A04663	395
A00552	A04668	347	A00552	A05150	256
A00552	A05288	183	A00552	A05315	273
A00552	A05560	172	A00552	A06010	274
A00552	A06197	177	A00552	A06660	427
A00552	A07011	179	A00552	A07311	430
A00552	A07339	491	A00552	A07538	201
A00552	A08210	177	A00552	A08212	262
A00552	A08221	296	A00552	A08355	204
A00552	A08516	274	A00552	A08571	414
A00552	A09078	485	A00552	A09543	246
A00552	A09598	263	A00552	A09791	383
A00552	A09837	201	A00552	A10072	348
A00552	A10514	184	A00552	A11291	489
A00552	A11324	172	A00552	A11487	451
A00552	A11624	324	A00552	A13250	900

A00552	A13824	224	A00552	A14006	488
A00552	A14009	180	A00552	A14147	200
A00552	A14198	213	A00552	A14220	491
A00552	A14399	270	A00552	A15293	187
A00552	A15294	183	A00552	A15504	477
A00552	A15505	167	A00552	A15850	230
A00552	A15935	152	A00552	A16410	227
A00552	A16994	159	A00552	A17136	245
A00553	A00652	458	A00553	A00674	160
A00553	A00714	481	A00553	A00744	418
A00553	A00751	719	A00553	A00761	160
A00553	A00766	160	A00553	A00786	278
A00553	A00791	537	A00553	A00809	463
A00553	A00838	205	A00553	A00868	665
A00553	A00883	545	A00553	A00884	160
A00553	A00926	533	A00553	A00927	533
A00553	A00936	330	A00553	A00941	347
A00553	A00942	247	A00553	A00956	251
A00553	A00957	271	A00553	A00975	160
A00553	A01039	184	A00553	A01058	714
A00553	A01077	160	A00553	A01080	545
A00553	A01097	746	A00553	A01100	479
A00553	A01169	177	A00553	A01252	215
A00553	A01261	477	A00553	A01263	319
A00553	A01267	861	A00553	A01302	272
A00553	A01325	489	A00553	A01326	575
A00553	A01339	160	A00553	A01348	335
A00553	A01358	479	A00553	A01385	379
A00553	A01451	542	A00553	A01571	191
A00553	A01578	313	A00553	A01660	199
A00553	A01787	185	A00553	A02131	156
A00553	A02212	160	A00553	A02389	462
A00553	A02635	335	A00553	A03014	424
A00553	A03129	168	A00553	A03665	515
A00553	A04750	196	A00553	A05439	199
A00553	A05591	191	A00553	A06554	462
A00553	A06599	428	A00553	A06739	152
A00553	A06907	352	A00553	A06997	162
A00553	A07096	160	A00553	A07209	200
A00553	A07226	160	A00553	A07360	363
A00553	A07483	171	A00553	A07848	178
A00553	A08262	184	A00553	A08461	267
A00553	A08506	474	A00553	A08517	443
A00553	A08536	458	A00553	A08550	368
A00553	A08551	477	A00553	A08569	460
A00553	A08834	160	A00553	A09062	156

A00553	A09239	202	A00553	A09627	160
A00553	A09672	369	A00553	A09723	160
A00553	A10159	247	A00553	A10315	321
A00553	A10372	160	A00553	A10383	215
A00553	A10532	216	A00553	A11365	160
A00553	A11497	162	A00553	A11598	435
A00553	A11648	219	A00553	A11649	463
A00553	A11735	722	A00553	A11737	721
A00553	A11914	722	A00553	A11917	446
A00553	A12253	160	A00553	A12255	414
A00553	A12325	174	A00553	A12658	722
A00553	A12824	545	A00553	A12834	177
A00553	A12858	157	A00553	A12873	177
A00553	A13313	427	A00553	A13587	246
A00553	A13915	184	A00553	A14007	160
A00553	A14084	195	A00553	A14196	562
A00553	A14202	268	A00553	A14212	174
A00553	A14286	218	A00553	A14570	462
A00553	A14650	402	A00553	A14661	393
A00553	A15189	269	A00553	A15237	419
A00553	A15257	544	A00553	A15297	577
A00553	A15309	162	A00553	A15431	213
A00553	A15558	477	A00553	A15559	627
A00553	A15651	154	A00553	A15666	427
A00553	A15680	294	A00553	A15700	172
A00553	A16157	374	A00553	A16611	190
A00553	A16720	177	A00553	A16860	473
A00553	A16874	226	A00553	A16901	355
A00553	A17044	205	A00553	A17133	319
A00554	A00575	473	A00554	A00595	295
A00554	A00605	286	A00554	A00651	170
A00554	A00691	276	A00554	A00708	226
A00554	A00713	170	A00554	A00749	170
A00554	A00767	207	A00554	A00772	182
A00554	A00858	160	A00554	A00861	170
A00554	A00872	170	A00554	A00916	546
A00554	A00918	170	A00554	A00923	251
A00554	A00934	549	A00554	A00970	159
A00554	A00992	170	A00554	A01020	708
A00554	A01028	170	A00554	A01075	398
A00554	A01088	243	A00554	A01116	485
A00554	A01120	170	A00554	A01129	170
A00554	A01160	170	A00554	A01168	170
A00554	A01184	491	A00554	A01201	357
A00554	A01206	210	A00554	A01226	63
A00554	A01234	491	A00554	A01304	477

A00554	A01351	488	A00554	A01356	170
A00554	A01425	170	A00554	A01429	353
A00554	A01487	267	A00554	A01492	189
A00554	A01538	170	A00554	A01555	259
A00554	A01576	424	A00554	A01598	170
A00554	A01614	253	A00554	A02265	170
A00554	A03112	172	A00554	A03438	170
A00554	A03652	170	A00554	A03981	170
A00554	A04638	307	A00554	A06356	170
A00554	A06974	193	A00554	A07223	329
A00554	A07244	170	A00554	A07347	439
A00554	A07524	170	A00554	A07533	358
A00554	A07625	274	A00554	A07700	486
A00554	A07706	234	A00554	A07924	170
A00554	A08024	472	A00554	A08211	488
A00554	A08491	651	A00554	A08528	180
A00554	A08674	162	A00554	A09307	475
A00554	A09619	687	A00554	A09664	467
A00554	A09697	170	A00554	A11831	213
A00554	A13052	170	A00554	A13989	244
A00554	A15129	363	A00554	A16994	157
A00555	A00570	573	A00555	A00590	201
A00555	A00629	494	A00555	A00687	572
A00555	A00717	491	A00555	A00721	557
A00555	A00727	491	A00555	A00772	695
A00555	A00809	265	A00555	A00865	491
A00555	A00928	574	A00555	A00952	695
A00555	A00972	512	A00555	A00997	217
A00555	A01068	238	A00555	A01101	201
A00555	A01107	572	A00555	A01159	247
A00555	A01181	208	A00555	A01189	462
A00555	A01221	495	A00555	A01243	570
A00555	A01313	268	A00555	A01314	236
A00555	A01352	695	A00555	A01385	249
A00555	A01418	319	A00555	A01440	205
A00555	A01442	427	A00555	A01459	246
A00555	A01741	243	A00555	A01809	218
A00555	A01977	173	A00555	A01988	219
A00555	A02164	486	A00555	A03460	255
A00555	A03556	217	A00555	A03617	217
A00555	A05605	217	A00555	A06122	462
A00555	A06204	414	A00555	A06347	433
A00555	A06369	480	A00555	A06832	561
A00555	A07083	171	A00555	A07312	483
A00555	A08183	395	A00555	A08272	202
A00555	A08450	157	A00555	A08558	154

A00555	A08627	394	A00555	A08984	694
A00555	A09345	247	A00555	A09485	170
A00555	A09673	217	A00555	A09713	196
A00555	A10317	404	A00555	A10373	201
A00555	A11277	458	A00555	A11933	328
A00555	A12052	271	A00555	A12140	223
A00555	A12252	151	A00555	A13117	461
A00555	A13231	196	A00555	A13356	180
A00555	A13788	162	A00555	A14084	245
A00555	A14087	266	A00555	A14088	492
A00555	A14167	184	A00555	A14186	475
A00555	A14246	452	A00555	A14358	255
A00555	A14462	302	A00555	A14482	374
A00555	A14701	443	A00555	A14819	254
A00555	A15270	215	A00555	A15323	374
A00555	A15324	457	A00555	A15370	473
A00555	A15373	182	A00555	A15374	486
A00555	A15377	684	A00555	A15543	217
A00555	A15677	159	A00555	A15724	323
A00555	A16463	241	A00555	A16611	213
A00555	A16664	235	A00555	A16682	171
A00555	A16824	218	A00555	A17133	290
A00556	A00557	458	A00556	A00558	491
A00556	A00568	473	A00556	A00640	63
A00556	A00647	176	A00556	A00658	196
A00556	A00659	162	A00556	A00736	481
A00556	A00747	152	A00556	A00847	311
A00556	A00914	463	A00556	A01064	441
A00556	A01137	157	A00556	A01188	172
A00556	A01257	283	A00556	A01404	223
A00556	A01450	378	A00556	A01462	159
A00556	A01554	159	A00556	A01563	360
A00556	A01602	435	A00556	A01626	475
A00556	A01650	488	A00556	A01668	485
A00556	A01676	313	A00556	A01694	395
A00556	A01704	484	A00556	A01741	477
A00556	A01743	326	A00556	A01770	462
A00556	A01812	172	A00556	A01844	273
A00556	A01926	159	A00556	A01951	205
A00556	A01959	473	A00556	A02018	288
A00556	A02083	201	A00556	A02086	363
A00556	A02111	200	A00556	A02117	174
A00556	A02152	210	A00556	A02223	165
A00556	A02226	191	A00556	A02304	176
A00556	A02306	159	A00556	A02564	168
A00556	A02668	168	A00556	A02698	162

A00556	A02722	408	A00556	A02811	201
A00556	A02834	314	A00556	A02874	479
A00556	A02877	63	A00556	A02895	146
A00556	A02896	490	A00556	A02910	63
A00556	A02930	488	A00556	A02939	277
A00556	A02944	371	A00556	A02963	391
A00556	A02967	353	A00556	A02972	177
A00556	A02976	217	A00556	A02995	151
A00556	A03019	258	A00556	A03043	191
A00556	A03076	341	A00556	A03099	232
A00556	A03118	241	A00556	A03150	162
A00556	A03212	279	A00556	A03223	491
A00556	A03224	262	A00556	A03275	281
A00556	A03288	408	A00556	A03321	167
A00556	A03451	156	A00556	A03523	319
A00556	A03568	243	A00556	A03576	172
A00556	A03604	323	A00556	A03839	235
A00556	A03893	363	A00556	A03914	252
A00556	A03952	247	A00556	A04010	395
A00556	A04061	303	A00556	A04092	162
A00556	A04164	196	A00556	A04721	172
A00556	A04779	168	A00556	A04873	177
A00556	A04879	191	A00556	A05112	491
A00556	A05176	241	A00556	A05315	207
A00556	A05349	238	A00556	A05369	241
A00556	A05380	168	A00556	A05417	263
A00556	A05476	215	A00556	A05508	475
A00556	A05709	251	A00556	A05721	200
A00556	A05767	310	A00556	A05878	208
A00556	A05879	201	A00556	A06286	226
A00556	A06581	218	A00556	A06610	160
A00556	A06623	154	A00556	A06642	486
A00556	A06644	179	A00556	A06949	201
A00556	A07123	489	A00556	A07140	404
A00556	A07141	279	A00556	A07170	301
A00556	A07190	151	A00556	A07218	429
A00556	A07552	172	A00556	A07723	246
A00556	A08065	294	A00556	A08279	152
A00556	A08572	172	A00556	A08673	227
A00556	A08805	183	A00556	A08851	391
A00556	A09033	343	A00556	A09087	241
A00556	A09540	488	A00556	A09932	165
A00556	A09977	157	A00556	A10022	196
A00556	A10081	168	A00556	A10174	377
A00556	A10267	168	A00556	A10296	215
A00556	A10353	162	A00556	A10394	241

A00556	A10601	63	A00556	A10625	486
A00556	A10737	215	A00556	A10866	270
A00556	A10942	196	A00556	A12972	190
A00556	A13632	215	A00556	A13829	485
A00556	A14430	368	A00556	A14957	162
A00556	A15099	247	A00556	A16751	172
A00556	A16829	332	A00557	A00697	63
A00557	A00736	490	A00557	A00847	180
A00557	A00939	144	A00557	A01307	475
A00557	A01524	919	A00557	A01527	370
A00557	A01848	254	A00557	A01870	163
A00557	A02578	235	A00557	A02668	177
A00557	A02753	184	A00557	A02847	317
A00557	A02852	251	A00557	A02883	132
A00557	A02889	251	A00557	A02896	179
A00557	A02916	95	A00557	A02920	240
A00557	A02922	270	A00557	A02931	102
A00557	A02944	486	A00557	A02951	190
A00557	A02953	318	A00557	A02961	488
A00557	A02962	409	A00557	A02971	265
A00557	A02972	491	A00557	A02976	491
A00557	A03223	404	A00557	A03279	226
A00557	A03288	272	A00557	A03982	316
A00557	A03998	417	A00557	A04050	440
A00557	A04051	491	A00557	A04052	246
A00557	A04110	182	A00557	A04117	202
A00557	A04278	290	A00557	A04608	243
A00557	A04609	172	A00557	A04611	446
A00557	A04612	413	A00557	A04613	380
A00557	A04614	180	A00557	A04616	486
A00557	A04618	236	A00557	A04659	601
A00557	A04693	305	A00557	A05169	271
A00557	A05242	439	A00557	A05273	190
A00557	A05349	358	A00557	A05539	205
A00557	A05747	369	A00557	A05760	159
A00557	A06149	201	A00557	A06312	256
A00557	A06333	256	A00557	A06385	274
A00557	A06589	218	A00557	A06627	176
A00557	A06631	312	A00557	A06644	184
A00557	A06735	199	A00557	A07135	201
A00557	A07149	167	A00557	A07157	88
A00557	A07167	162	A00557	A07168	296
A00557	A07955	347	A00557	A08654	369
A00557	A10178	347	A00557	A10573	347
A00557	A10601	77	A00557	A10930	324
A00557	A13131	262	A00557	A14199	260

A00557	A14201	328	A00557	A14208	458
A00557	A14443	989	A00557	A14502	182
A00557	A14707	217	A00557	A15613	190
A00557	A15639	446	A00557	A15674	347
A00557	A15960	393	A00557	A15974	184
A00557	A15996	291	A00557	A16216	208
A00557	A16485	372	A00558	A00647	445
A00558	A00820	475	A00558	A01280	369
A00558	A01404	488	A00558	A01450	360
A00558	A01602	417	A00558	A01650	491
A00558	A01660	213	A00558	A01668	369
A00558	A01704	205	A00558	A01712	163
A00558	A01830	168	A00558	A01903	414
A00558	A01921	190	A00558	A02018	350
A00558	A02049	458	A00558	A02138	204
A00558	A02149	157	A00558	A02162	216
A00558	A02208	353	A00558	A02268	226
A00558	A02698	308	A00558	A02722	246
A00558	A02896	340	A00558	A02930	246
A00558	A03047	201	A00558	A03212	245
A00558	A03223	485	A00558	A03247	160
A00558	A03742	419	A00558	A04608	160
A00558	A05016	277	A00558	A05112	434
A00558	A05467	160	A00558	A05476	265
A00558	A05709	177	A00558	A06608	475
A00558	A06727	162	A00558	A07016	190
A00558	A07123	480	A00558	A07170	221
A00558	A07218	291	A00558	A07341	193
A00558	A08673	276	A00558	A09033	171
A00558	A09380	171	A00558	A09540	196
A00558	A10986	228	A00558	A11576	168
A00558	A11931	228	A00558	A12472	163
A00558	A12962	306	A00558	A13054	154
A00558	A13597	212	A00558	A13829	455
A00559	A00691	245	A00559	A00905	408
A00559	A01054	152	A00559	A01085	174
A00559	A01139	456	A00559	A01206	157
A00559	A01231	193	A00559	A01266	286
A00559	A01268	446	A00559	A01359	258
A00559	A01558	216	A00559	A01588	207
A00559	A01612	419	A00559	A01707	184
A00559	A02165	190	A00559	A03298	486
A00559	A03902	278	A00559	A07604	168
A00559	A08137	259	A00559	A08571	491
A00559	A08818	247	A00559	A09665	343
A00559	A09686	339	A00559	A10250	324

A00559	A10624	241	A00559	A10853	180
A00559	A11105	408	A00559	A11139	396
A00559	A11338	191	A00559	A11920	341
A00559	A11928	480	A00559	A12251	286
A00559	A13310	468	A00559	A13311	479
A00559	A14476	483	A00559	A14728	271
A00559	A14899	347	A00559	A15711	252
A00559	A15769	151	A00559	A16491	316
A00559	A16912	343	A00560	A00655	488
A00560	A00781	462	A00560	A00812	193
A00560	A00822	491	A00560	A00835	491
A00560	A00836	176	A00560	A00837	413
A00560	A00883	435	A00560	A00905	436
A00560	A00916	432	A00560	A00923	241
A00560	A00940	488	A00560	A00954	451
A00560	A00957	491	A00560	A00958	358
A00560	A01052	217	A00560	A01075	486
A00560	A01077	335	A00560	A01082	271
A00560	A01087	213	A00560	A01091	157
A00560	A01143	491	A00560	A01194	245
A00560	A01206	473	A00560	A01237	486
A00560	A01266	260	A00560	A01268	201
A00560	A01280	491	A00560	A01284	165
A00560	A01291	207	A00560	A01378	402
A00560	A01406	200	A00560	A01552	366
A00560	A01560	177	A00560	A01598	489
A00560	A01612	446	A00560	A01614	152
A00560	A01647	223	A00560	A01660	470
A00560	A01995	154	A00560	A01997	400
A00560	A02452	290	A00560	A02592	165
A00560	A03057	447	A00560	A03070	484
A00560	A03390	157	A00560	A03435	474
A00560	A03446	154	A00560	A03569	445
A00560	A04020	301	A00560	A04050	152
A00560	A04449	296	A00560	A05029	162
A00560	A06466	472	A00560	A06907	491
A00560	A07311	486	A00560	A07427	168
A00560	A07604	491	A00560	A07616	162
A00560	A07785	485	A00560	A08137	491
A00560	A08300	157	A00560	A08571	490
A00560	A09591	177	A00560	A09737	243
A00560	A11227	195	A00560	A11291	491
A00560	A11323	223	A00560	A11324	337
A00560	A11405	462	A00560	A11422	369
A00560	A11920	267	A00560	A11928	180
A00560	A11952	461	A00560	A12020	180

A00560	A12069	418	A00560	A12138	199
A00560	A12329	467	A00560	A12365	172
A00560	A12416	180	A00560	A12458	232
A00560	A12543	489	A00560	A12660	468
A00560	A13287	224	A00560	A13310	187
A00560	A13340	355	A00560	A13466	488
A00560	A13889	348	A00560	A13928	491
A00560	A13989	229	A00560	A14017	297
A00560	A14073	193	A00560	A14147	263
A00560	A14148	160	A00560	A14196	475
A00560	A14220	491	A00560	A14221	491
A00560	A14257	491	A00560	A14265	296
A00560	A14303	277	A00560	A14451	212
A00560	A14476	268	A00560	A14497	187
A00560	A14596	368	A00560	A14738	324
A00560	A14875	217	A00560	A14931	428
A00560	A15116	251	A00560	A15291	461
A00560	A15309	402	A00560	A15366	491
A00560	A15376	201	A00560	A15431	198
A00560	A15492	491	A00560	A15503	357
A00560	A15535	238	A00560	A15660	399
A00560	A15684	356	A00560	A15695	286
A00560	A15741	296	A00560	A15749	252
A00560	A15752	486	A00560	A15760	168
A00560	A15773	483	A00560	A15804	170
A00560	A15851	249	A00560	A15852	221
A00560	A16218	249	A00560	A16301	241
A00560	A16328	310	A00560	A16724	272
A00560	A16768	210	A00560	A17013	151
A00560	A17063	188	A00560	A17134	208
A00561	A00571	434	A00561	A00590	168
A00561	A00605	358	A00561	A00651	419
A00561	A00653	697	A00561	A00687	227
A00561	A00694	462	A00561	A00700	270
A00561	A00705	236	A00561	A00718	489
A00561	A00724	157	A00561	A00736	458
A00561	A00769	314	A00561	A00783	313
A00561	A00790	477	A00561	A00804	176
A00561	A00821	461	A00561	A00839	489
A00561	A00841	486	A00561	A00849	480
A00561	A00865	491	A00561	A00887	263
A00561	A00909	408	A00561	A00922	491
A00561	A00953	344	A00561	A00983	483
A00561	A00987	377	A00561	A01000	367
A00561	A01006	326	A00561	A01013	319
A00561	A01027	489	A00561	A01062	177

A00561	A01090	266	A00561	A01112	319
A00561	A01127	245	A00561	A01136	200
A00561	A01140	283	A00561	A01150	791
A00561	A01161	655	A00561	A01172	440
A00561	A01206	283	A00561	A01212	267
A00561	A01215	173	A00561	A01221	475
A00561	A01232	307	A00561	A01244	484
A00561	A01287	480	A00561	A01291	486
A00561	A01335	238	A00561	A01346	185
A00561	A01360	485	A00561	A01399	369
A00561	A01403	475	A00561	A01431	491
A00561	A01439	477	A00561	A01446	274
A00561	A01468	307	A00561	A01496	408
A00561	A01527	933	A00561	A01548	302
A00561	A01567	234	A00561	A01579	480
A00561	A01615	899	A00561	A01626	435
A00561	A01647	485	A00561	A01649	306
A00561	A01668	477	A00561	A01699	311
A00561	A01705	80	A00561	A01711	465
A00561	A01751	369	A00561	A01775	177
A00561	A01782	390	A00561	A01787	491
A00561	A01793	63	A00561	A01796	491
A00561	A01798	462	A00561	A01807	176
A00561	A01893	63	A00561	A01894	491
A00561	A01895	281	A00561	A01909	63
A00561	A01914	490	A00561	A02278	423
A00561	A02350	372	A00561	A02407	422
A00561	A02630	219	A00561	A02637	247
A00561	A02687	491	A00561	A02854	165
A00561	A02917	162	A00561	A02985	399
A00561	A02988	491	A00561	A03435	154
A00561	A03456	276	A00561	A03611	156
A00561	A03643	156	A00561	A03713	443
A00561	A03872	363	A00561	A03875	310
A00561	A04053	323	A00561	A04213	154
A00561	A04302	160	A00561	A04304	432
A00561	A04371	204	A00561	A04452	246
A00561	A04623	204	A00561	A04878	259
A00561	A04879	428	A00561	A04933	457
A00561	A05094	190	A00561	A05258	355
A00561	A05285	173	A00561	A05443	183
A00561	A05579	340	A00561	A05752	310
A00561	A05797	224	A00561	A05866	407
A00561	A05940	363	A00561	A06025	279
A00561	A06095	156	A00561	A06607	470
A00561	A06637	265	A00561	A06664	366

A00561	A06926	377	A00561	A06970	185
A00561	A07160	489	A00561	A07200	302
A00561	A08275	310	A00561	A08359	408
A00561	A08389	322	A00561	A08408	257
A00561	A08452	302	A00561	A08547	156
A00561	A08627	462	A00561	A08684	184
A00561	A09223	310	A00561	A09262	180
A00561	A09366	310	A00561	A09696	180
A00561	A09826	489	A00561	A09901	204
A00561	A09964	204	A00561	A10146	193
A00561	A10386	302	A00561	A10616	167
A00561	A11372	190	A00561	A11411	271
A00561	A11606	193	A00561	A12259	218
A00561	A13333	170	A00561	A13340	152
A00561	A13866	259	A00561	A13989	377
A00561	A14016	485	A00561	A14216	347
A00561	A14699	425	A00561	A14839	210
A00561	A15232	310	A00561	A15379	420
A00561	A15660	263	A00561	A15684	457
A00561	A16281	416	A00561	A16397	160
A00561	A16438	446	A00561	A17132	154
A00562	A00565	208	A00562	A00625	491
A00562	A00638	384	A00562	A00703	269
A00562	A00709	234	A00562	A00719	294
A00562	A00733	457	A00562	A00786	490
A00562	A00862	433	A00562	A00864	463
A00562	A00877	299	A00562	A00881	243
A00562	A00951	446	A00562	A00971	491
A00562	A01011	323	A00562	A01036	490
A00562	A01064	279	A00562	A01208	279
A00562	A01216	491	A00562	A01244	266
A00562	A01290	428	A00562	A01358	430
A00562	A01366	484	A00562	A01454	490
A00562	A01459	491	A00562	A01484	229
A00562	A01507	465	A00562	A01514	491
A00562	A01568	491	A00562	A01633	227
A00562	A01647	485	A00562	A01657	215
A00562	A01681	294	A00562	A01687	485
A00562	A01931	201	A00562	A01988	490
A00562	A02154	241	A00562	A02836	213
A00562	A03042	171	A00562	A03044	263
A00562	A03111	252	A00562	A03116	272
A00562	A03117	238	A00562	A03321	401
A00562	A03518	271	A00562	A03529	208
A00562	A03769	278	A00562	A04081	394
A00562	A04715	221	A00562	A04790	156

A00562	A04877	184	A00562	A05015	260
A00562	A05094	279	A00562	A06176	491
A00562	A06237	226	A00562	A06277	475
A00562	A06379	216	A00562	A06502	297
A00562	A06564	286	A00562	A06591	173
A00562	A06680	491	A00562	A06752	420
A00562	A06819	443	A00562	A06966	333
A00562	A07054	396	A00562	A07083	486
A00562	A07111	228	A00562	A07242	491
A00562	A07404	491	A00562	A07483	188
A00562	A07495	491	A00562	A07665	407
A00562	A07893	458	A00562	A07944	179
A00562	A07954	191	A00562	A08492	302
A00562	A08675	302	A00562	A08834	278
A00562	A08917	486	A00562	A09256	485
A00562	A09311	229	A00562	A09760	154
A00562	A10174	899	A00562	A10250	467
A00562	A10909	238	A00562	A11023	446
A00562	A11281	272	A00562	A11346	260
A00562	A12047	491	A00562	A12313	327
A00562	A12561	272	A00562	A13744	302
A00562	A13784	384	A00562	A13938	195
A00562	A14209	246	A00562	A14266	404
A00562	A14281	491	A00562	A14406	361
A00562	A14407	238	A00562	A14474	491
A00562	A14484	468	A00562	A14633	385
A00562	A15281	160	A00562	A15380	480
A00562	A15396	281	A00562	A16004	303
A00562	A16048	486	A00562	A16423	346
A00562	A16649	385	A00563	A00570	481
A00563	A00629	172	A00563	A00648	228
A00563	A00687	473	A00563	A00689	363
A00563	A00709	335	A00563	A00854	279
A00563	A00865	484	A00563	A00878	236
A00563	A00972	463	A00563	A01016	255
A00563	A01068	235	A00563	A01102	235
A00563	A01154	213	A00563	A01161	452
A00563	A01168	152	A00563	A01189	424
A00563	A01220	229	A00563	A01221	472
A00563	A01243	424	A00563	A01352	210
A00563	A01393	205	A00563	A01412	317
A00563	A01416	151	A00563	A01418	344
A00563	A01582	152	A00563	A01702	307
A00563	A01976	488	A00563	A01977	391
A00563	A02542	167	A00563	A03272	252
A00563	A03322	245	A00563	A03460	425

A00563	A03652	469	A00563	A03901	294
A00563	A06017	291	A00563	A06351	172
A00563	A06369	199	A00563	A06832	467
A00563	A07449	266	A00563	A09485	389
A00563	A11277	162	A00563	A11288	299
A00563	A13874	154	A00563	A13992	249
A00563	A14335	249	A00563	A14566	91
A00563	A14594	259	A00563	A16401	294
A00563	A17150	329	A00564	A00569	382
A00564	A00576	350	A00564	A00577	236
A00564	A00640	152	A00564	A00653	421
A00564	A00726	299	A00564	A00810	152
A00564	A00833	299	A00564	A00849	283
A00564	A00915	196	A00564	A00974	299
A00564	A00975	221	A00564	A00984	524
A00564	A00987	381	A00564	A01001	402
A00564	A01102	500	A00564	A01169	221
A00564	A01311	152	A00564	A01379	299
A00564	A01387	559	A00564	A01439	299
A00564	A01577	152	A00564	A01627	383
A00564	A01722	388	A00564	A02601	299
A00564	A02616	474	A00564	A03697	152
A00564	A04495	299	A00564	A06272	221
A00564	A06287	152	A00564	A06742	461
A00564	A07744	152	A00564	A08061	152
A00564	A08098	152	A00564	A08178	152
A00564	A08763	152	A00564	A08947	299
A00564	A09004	299	A00564	A09548	318
A00564	A09602	391	A00564	A10321	215
A00564	A10699	199	A00564	A10842	152
A00564	A11242	430	A00564	A14525	152
A00564	A15375	152	A00564	A15546	152
A00564	A15917	252	A00565	A00567	462
A00565	A00570	486	A00565	A00571	490
A00565	A00597	358	A00565	A00614	223
A00565	A00625	153	A00565	A00629	446
A00565	A00638	491	A00565	A00643	531
A00565	A00648	465	A00565	A00653	255
A00565	A00670	502	A00565	A00805	489
A00565	A00856	279	A00565	A00862	642
A00565	A00868	230	A00565	A00871	185
A00565	A00878	302	A00565	A00883	479
A00565	A00888	677	A00565	A00928	255
A00565	A00941	213	A00565	A00968	223
A00565	A00977	430	A00565	A00985	452
A00565	A01013	254	A00565	A01017	378

A00565	A01021	485	A00565	A01052	375
A00565	A01074	472	A00565	A01100	506
A00565	A01122	238	A00565	A01149	434
A00565	A01169	458	A00565	A01172	265
A00565	A01189	302	A00565	A01209	703
A00565	A01214	200	A00565	A01221	454
A00565	A01244	491	A00565	A01324	196
A00565	A01325	388	A00565	A01365	311
A00565	A01373	369	A00565	A01385	185
A00565	A01406	316	A00565	A01409	279
A00565	A01452	200	A00565	A01459	516
A00565	A01483	378	A00565	A01507	491
A00565	A01514	382	A00565	A01516	272
A00565	A01559	515	A00565	A01622	464
A00565	A01647	491	A00565	A01660	491
A00565	A01730	491	A00565	A01796	475
A00565	A01976	186	A00565	A01988	420
A00565	A02032	165	A00565	A02051	355
A00565	A02158	221	A00565	A02180	491
A00565	A02219	475	A00565	A02335	223
A00565	A02452	303	A00565	A02568	223
A00565	A02687	157	A00565	A02789	223
A00565	A02876	229	A00565	A03050	221
A00565	A03297	63	A00565	A03321	317
A00565	A03327	475	A00565	A03328	762
A00565	A03401	469	A00565	A03651	254
A00565	A03652	465	A00565	A03719	223
A00565	A03728	694	A00565	A03752	223
A00565	A03764	361	A00565	A04079	319
A00565	A04174	481	A00565	A04178	265
A00565	A04187	490	A00565	A04316	171
A00565	A04405	262	A00565	A05359	154
A00565	A05378	165	A00565	A05427	254
A00565	A05621	160	A00565	A05690	491
A00565	A06016	165	A00565	A06017	154
A00565	A06085	664	A00565	A06122	172
A00565	A06264	485	A00565	A06351	172
A00565	A06423	310	A00565	A06493	255
A00565	A06671	484	A00565	A06689	270
A00565	A06819	563	A00565	A06924	438
A00565	A06982	443	A00565	A07054	348
A00565	A07222	223	A00565	A07249	352
A00565	A07261	187	A00565	A07333	502
A00565	A07339	519	A00565	A07349	286
A00565	A07572	490	A00565	A07756	196
A00565	A07793	174	A00565	A07869	204

A00565	A08259	235	A00565	A08463	384
A00565	A08524	506	A00565	A08863	229
A00565	A08884	491	A00565	A08939	438
A00565	A09067	434	A00565	A09269	491
A00565	A09276	165	A00565	A09316	491
A00565	A09340	491	A00565	A09442	182
A00565	A09607	221	A00565	A09659	182
A00565	A09761	491	A00565	A09826	180
A00565	A09873	491	A00565	A10076	201
A00565	A10258	430	A00565	A10289	445
A00565	A10314	458	A00565	A10421	188
A00565	A10508	281	A00565	A10575	438
A00565	A10870	162	A00565	A10978	224
A00565	A11107	165	A00565	A11165	267
A00565	A11487	217	A00565	A11828	326
A00565	A11858	491	A00565	A11983	215
A00565	A12047	525	A00565	A12249	165
A00565	A12824	227	A00565	A12865	224
A00565	A12875	385	A00565	A12912	196
A00565	A13050	334	A00565	A13287	468
A00565	A13519	224	A00565	A13771	187
A00565	A13822	229	A00565	A13986	484
A00565	A14025	283	A00565	A14074	901
A00565	A14230	477	A00565	A14481	277
A00565	A14525	223	A00565	A14540	163
A00565	A14665	191	A00565	A14804	223
A00565	A14827	277	A00565	A14976	255
A00565	A15292	344	A00565	A15297	191
A00565	A15367	438	A00565	A15372	293
A00565	A15557	173	A00565	A15818	160
A00565	A16035	201	A00565	A16219	223
A00565	A16223	165	A00565	A16401	258
A00565	A16404	452	A00565	A16410	491
A00565	A16440	491	A00565	A16531	223
A00565	A16572	224	A00565	A16690	378
A00565	A16769	196	A00565	A17059	162
A00566	A00572	484	A00566	A00575	715
A00566	A00605	452	A00566	A00622	712
A00566	A00626	518	A00566	A00655	485
A00566	A00665	377	A00566	A00708	470
A00566	A00733	542	A00566	A00744	236
A00566	A00760	246	A00566	A00805	667
A00566	A00831	450	A00566	A00856	491
A00566	A00858	917	A00566	A00860	714
A00566	A00866	156	A00566	A00868	180
A00566	A00881	542	A00566	A00883	420

A00566	A00903	465	A00566	A00923	188
A00566	A00934	486	A00566	A00954	597
A00566	A00978	523	A00566	A01007	713
A00566	A01019	541	A00566	A01020	489
A00566	A01024	271	A00566	A01032	443
A00566	A01075	489	A00566	A01087	419
A00566	A01116	439	A00566	A01147	395
A00566	A01184	484	A00566	A01195	534
A00566	A01200	714	A00566	A01206	491
A00566	A01212	472	A00566	A01214	252
A00566	A01215	408	A00566	A01234	490
A00566	A01325	450	A00566	A01349	434
A00566	A01383	296	A00566	A01398	541
A00566	A01401	715	A00566	A01412	408
A00566	A01429	226	A00566	A01468	488
A00566	A01492	202	A00566	A01500	617
A00566	A01503	787	A00566	A01555	386
A00566	A01576	486	A00566	A01598	152
A00566	A01720	299	A00566	A01761	346
A00566	A02855	408	A00566	A03191	347
A00566	A03306	271	A00566	A04106	190
A00566	A04548	151	A00566	A04648	539
A00566	A04687	156	A00566	A05027	152
A00566	A06062	227	A00566	A06543	428
A00566	A06793	246	A00566	A06803	536
A00566	A06972	152	A00566	A07348	460
A00566	A07520	346	A00566	A07670	503
A00566	A07700	340	A00566	A07706	401
A00566	A07850	436	A00566	A07887	533
A00566	A07920	343	A00566	A08022	491
A00566	A08383	445	A00566	A08495	279
A00566	A08553	485	A00566	A08818	156
A00566	A09010	480	A00566	A09502	156
A00566	A09708	205	A00566	A09712	480
A00566	A10684	188	A00566	A11344	156
A00566	A11395	486	A00566	A11674	156
A00566	A11804	419	A00566	A13078	152
A00566	A13620	308	A00566	A13831	160
A00566	A13980	308	A00566	A14006	292
A00566	A14359	267	A00566	A14655	455
A00566	A14899	314	A00566	A15034	328
A00566	A15315	663	A00566	A15347	556
A00566	A15373	435	A00566	A15733	210
A00566	A15945	493	A00566	A15946	329
A00566	A16067	260	A00566	A16144	326
A00566	A16212	466	A00566	A16228	497

A00567	A00568	314	A00567	A00597	449
A00567	A00611	475	A00567	A00718	221
A00567	A00743	218	A00567	A00888	326
A00567	A01198	255	A00567	A01716	340
A00567	A02018	216	A00567	A02306	210
A00567	A03498	366	A00567	A03961	157
A00567	A06253	351	A00567	A06629	296
A00567	A08377	219	A00567	A09442	286
A00567	A10477	226	A00568	A00597	187
A00568	A00611	404	A00568	A00616	210
A00568	A00664	317	A00568	A00820	182
A00568	A00846	152	A00568	A00850	196
A00568	A00855	438	A00568	A00914	441
A00568	A01074	317	A00568	A01105	438
A00568	A01137	435	A00568	A01343	213
A00568	A01404	469	A00568	A01590	227
A00568	A01602	185	A00568	A01622	438
A00568	A01679	317	A00568	A01937	399
A00568	A02083	137	A00568	A02111	180
A00568	A02156	260	A00568	A02219	154
A00568	A02251	195	A00568	A02260	260
A00568	A02262	279	A00568	A02275	196
A00568	A02524	340	A00568	A02698	382
A00568	A02772	281	A00568	A03086	461
A00568	A03091	213	A00568	A03099	238
A00568	A03173	363	A00568	A03238	168
A00568	A03498	380	A00568	A03584	204
A00568	A03710	438	A00568	A03919	272
A00568	A04163	355	A00568	A05143	286
A00568	A05208	272	A00568	A05332	317
A00568	A05459	318	A00568	A05609	317
A00568	A05679	291	A00568	A06253	367
A00568	A06446	374	A00568	A06471	321
A00568	A06607	226	A00568	A07019	235
A00568	A07140	438	A00568	A07222	317
A00568	A07333	438	A00568	A07559	218
A00568	A07571	317	A00568	A07655	380
A00568	A07803	162	A00568	A07974	317
A00568	A08097	438	A00568	A08660	355
A00568	A08688	263	A00568	A08724	223
A00568	A08850	156	A00568	A09135	255
A00568	A09236	390	A00568	A09340	438
A00568	A09429	441	A00568	A14128	162
A00568	A14438	279	A00568	A16099	249
A00569	A00576	401	A00569	A00577	293
A00569	A00653	188	A00569	A00984	408

A00569	A01001	460	A00569	A01102	303
A00569	A01379	221	A00569	A01387	267
A00569	A01627	238	A00569	A01650	457
A00569	A02814	251	A00569	A03676	156
A00569	A05323	422	A00569	A10321	274
A00569	A15498	174	A00570	A00571	279
A00570	A00572	163	A00570	A00599	210
A00570	A00605	305	A00570	A00606	296
A00570	A00629	688	A00570	A00648	191
A00570	A00653	235	A00570	A00658	267
A00570	A00687	575	A00570	A00689	218
A00570	A00693	79	A00570	A00703	223
A00570	A00709	480	A00570	A00717	424
A00570	A00718	154	A00570	A00721	568
A00570	A00727	488	A00570	A00772	561
A00570	A00829	477	A00570	A00839	226
A00570	A00848	434	A00570	A00854	276
A00570	A00865	688	A00570	A00868	474
A00570	A00871	156	A00570	A00878	458
A00570	A00883	491	A00570	A00915	170
A00570	A00916	391	A00570	A00923	346
A00570	A00928	688	A00570	A00952	688
A00570	A00972	450	A00570	A00975	268
A00570	A00983	312	A00570	A00997	283
A00570	A01025	183	A00570	A01040	223
A00570	A01053	484	A00570	A01065	451
A00570	A01075	363	A00570	A01091	429
A00570	A01101	425	A00570	A01102	343
A00570	A01107	556	A00570	A01120	221
A00570	A01150	432	A00570	A01159	688
A00570	A01166	443	A00570	A01169	219
A00570	A01184	217	A00570	A01201	156
A00570	A01206	383	A00570	A01221	688
A00570	A01234	491	A00570	A01243	575
A00570	A01244	240	A00570	A01251	358
A00570	A01278	378	A00570	A01291	490
A00570	A01352	491	A00570	A01356	446
A00570	A01368	163	A00570	A01384	414
A00570	A01412	588	A00570	A01418	574
A00570	A01448	152	A00570	A01451	460
A00570	A01458	208	A00570	A01459	321
A00570	A01468	396	A00570	A01470	486
A00570	A01492	396	A00570	A01569	227
A00570	A01598	490	A00570	A01649	396
A00570	A01702	389	A00570	A01741	394
A00570	A01846	276	A00570	A01847	457

A00570	A01941	219	A00570	A01976	417
A00570	A02024	188	A00570	A02064	330
A00570	A02164	491	A00570	A02195	187
A00570	A02203	417	A00570	A02212	435
A00570	A02227	152	A00570	A02268	346
A00570	A02454	344	A00570	A03019	352
A00570	A03438	485	A00570	A03494	274
A00570	A03556	227	A00570	A03567	491
A00570	A03597	374	A00570	A03617	217
A00570	A03788	259	A00570	A03797	199
A00570	A04099	234	A00570	A04100	160
A00570	A04932	157	A00570	A05095	208
A00570	A05323	157	A00570	A05605	217
A00570	A05646	357	A00570	A05690	286
A00570	A06017	291	A00570	A06049	204
A00570	A06122	486	A00570	A06204	201
A00570	A06369	675	A00570	A06436	394
A00570	A06602	428	A00570	A06664	152
A00570	A06825	491	A00570	A06832	688
A00570	A07309	419	A00570	A07312	490
A00570	A07473	172	A00570	A07583	475
A00570	A07706	182	A00570	A08183	519
A00570	A08596	338	A00570	A08982	424
A00570	A08984	205	A00570	A08989	152
A00570	A09289	246	A00570	A09317	195
A00570	A09327	107	A00570	A09673	217
A00570	A10402	204	A00570	A10549	278
A00570	A10662	163	A00570	A11277	490
A00570	A11411	152	A00570	A11532	288
A00570	A11842	232	A00570	A11933	188
A00570	A12252	347	A00570	A13989	477
A00570	A14087	230	A00570	A14088	264
A00570	A14188	491	A00570	A14196	187
A00570	A14216	224	A00570	A14482	332
A00570	A14594	157	A00570	A14596	675
A00570	A14597	491	A00570	A14701	491
A00570	A15297	213	A00570	A15353	368
A00570	A15364	208	A00570	A15377	488
A00570	A15543	546	A00570	A15550	350
A00570	A15564	167	A00570	A15624	480
A00570	A15649	484	A00570	A15660	472
A00570	A15684	450	A00570	A15724	217
A00570	A15932	491	A00570	A16118	215
A00570	A16401	162	A00570	A16664	217
A00570	A16810	204	A00571	A00590	221
A00571	A00593	373	A00571	A00597	263

A00571	A00603	621	A00571	A00604	283
A00571	A00605	490	A00571	A00606	236
A00571	A00608	152	A00571	A00610	157
A00571	A00629	581	A00571	A00648	603
A00571	A00651	491	A00571	A00653	710
A00571	A00658	491	A00571	A00673	188
A00571	A00687	557	A00571	A00693	196
A00571	A00694	307	A00571	A00697	467
A00571	A00700	490	A00571	A00705	556
A00571	A00709	188	A00571	A00711	558
A00571	A00718	258	A00571	A00740	450
A00571	A00741	299	A00571	A00750	268
A00571	A00762	299	A00571	A00763	210
A00571	A00781	159	A00571	A00782	491
A00571	A00785	184	A00571	A00787	466
A00571	A00788	469	A00571	A00789	204
A00571	A00790	277	A00571	A00793	565
A00571	A00805	235	A00571	A00821	285
A00571	A00826	371	A00571	A00831	411
A00571	A00835	167	A00571	A00839	693
A00571	A00843	249	A00571	A00848	408
A00571	A00849	477	A00571	A00854	317
A00571	A00856	279	A00571	A00865	485
A00571	A00870	312	A00571	A00871	486
A00571	A00878	491	A00571	A00883	466
A00571	A00902	486	A00571	A00922	428
A00571	A00923	430	A00571	A00953	549
A00571	A00955	491	A00571	A00965	584
A00571	A00968	249	A00571	A00970	424
A00571	A00972	202	A00571	A00974	260
A00571	A00977	334	A00571	A00981	365
A00571	A00983	491	A00571	A00984	450
A00571	A00987	688	A00571	A00990	481
A00571	A00991	489	A00571	A00994	380
A00571	A01000	470	A00571	A01004	475
A00571	A01012	401	A00571	A01016	358
A00571	A01017	560	A00571	A01023	308
A00571	A01025	491	A00571	A01052	207
A00571	A01056	154	A00571	A01065	221
A00571	A01072	450	A00571	A01074	388
A00571	A01075	419	A00571	A01086	721
A00571	A01090	375	A00571	A01091	168
A00571	A01098	571	A00571	A01101	325
A00571	A01108	414	A00571	A01112	452
A00571	A01115	383	A00571	A01120	481
A00571	A01121	489	A00571	A01127	411

A00571	A01136	324	A00571	A01140	460
A00571	A01150	491	A00571	A01152	475
A00571	A01153	485	A00571	A01154	657
A00571	A01160	204	A00571	A01161	491
A00571	A01166	612	A00571	A01169	480
A00571	A01186	449	A00571	A01196	461
A00571	A01206	490	A00571	A01209	428
A00571	A01211	432	A00571	A01214	311
A00571	A01215	490	A00571	A01217	704
A00571	A01218	446	A00571	A01220	560
A00571	A01221	491	A00571	A01230	339
A00571	A01234	491	A00571	A01238	577
A00571	A01243	485	A00571	A01247	491
A00571	A01249	296	A00571	A01252	243
A00571	A01263	491	A00571	A01287	484
A00571	A01291	582	A00571	A01306	684
A00571	A01325	279	A00571	A01335	491
A00571	A01337	484	A00571	A01352	402
A00571	A01353	491	A00571	A01362	517
A00571	A01365	682	A00571	A01369	480
A00571	A01380	159	A00571	A01384	439
A00571	A01392	388	A00571	A01406	700
A00571	A01407	489	A00571	A01408	560
A00571	A01411	488	A00571	A01412	592
A00571	A01413	241	A00571	A01414	210
A00571	A01416	632	A00571	A01418	380
A00571	A01425	478	A00571	A01436	412
A00571	A01439	184	A00571	A01449	491
A00571	A01451	188	A00571	A01464	491
A00571	A01468	491	A00571	A01470	491
A00571	A01487	255	A00571	A01492	484
A00571	A01495	457	A00571	A01496	491
A00571	A01539	167	A00571	A01540	477
A00571	A01569	488	A00571	A01579	491
A00571	A01582	454	A00571	A01586	486
A00571	A01597	347	A00571	A01601	580
A00571	A01611	488	A00571	A01612	441
A00571	A01613	486	A00571	A01618	435
A00571	A01647	460	A00571	A01649	491
A00571	A01652	238	A00571	A01711	375
A00571	A01822	461	A00571	A01825	419
A00571	A01976	188	A00571	A01988	188
A00571	A01993	361	A00571	A01998	152
A00571	A01999	238	A00571	A02051	281
A00571	A02068	159	A00571	A02117	274
A00571	A02211	339	A00571	A02214	207

A00571	A02216	157	A00571	A02225	191
A00571	A02227	491	A00571	A02364	152
A00571	A02452	438	A00571	A02924	256
A00571	A02978	272	A00571	A03095	491
A00571	A03187	352	A00571	A03401	235
A00571	A03438	232	A00571	A03453	486
A00571	A03461	254	A00571	A03472	357
A00571	A03490	246	A00571	A03493	490
A00571	A03567	580	A00571	A03568	488
A00571	A03600	98	A00571	A03601	640
A00571	A03652	394	A00571	A03671	434
A00571	A03676	235	A00571	A03705	428
A00571	A03720	255	A00571	A03859	483
A00571	A03901	482	A00571	A03981	490
A00571	A03990	204	A00571	A04072	224
A00571	A04230	213	A00571	A04563	160
A00571	A04688	474	A00571	A04689	247
A00571	A04844	445	A00571	A05025	162
A00571	A05198	160	A00571	A05199	226
A00571	A05210	235	A00571	A05258	337
A00571	A05318	440	A00571	A05327	234
A00571	A05375	591	A00571	A05450	378
A00571	A05499	489	A00571	A05501	595
A00571	A05502	405	A00571	A05510	283
A00571	A05512	491	A00571	A05557	409
A00571	A05702	188	A00571	A05804	386
A00571	A06025	188	A00571	A06105	352
A00571	A06114	227	A00571	A06115	322
A00571	A06279	419	A00571	A06308	177
A00571	A06351	457	A00571	A06388	205
A00571	A06564	491	A00571	A06589	480
A00571	A06785	304	A00571	A06861	499
A00571	A06965	364	A00571	A06973	157
A00571	A07028	167	A00571	A07075	270
A00571	A07160	293	A00571	A07201	200
A00571	A07248	201	A00571	A07305	152
A00571	A07323	434	A00571	A07349	486
A00571	A07447	188	A00571	A07473	174
A00571	A07581	477	A00571	A07706	463
A00571	A07730	173	A00571	A07773	401
A00571	A07965	199	A00571	A08011	168
A00571	A08128	227	A00571	A08154	489
A00571	A08301	266	A00571	A08335	63
A00571	A08357	232	A00571	A08358	489
A00571	A08359	458	A00571	A08408	312
A00571	A08419	467	A00571	A08501	486

A00571	A08543	474	A00571	A08556	491
A00571	A08559	424	A00571	A08760	124
A00571	A08761	456	A00571	A08900	154
A00571	A08901	485	A00571	A08929	266
A00571	A08979	230	A00571	A09232	319
A00571	A09285	326	A00571	A09289	270
A00571	A09322	228	A00571	A09520	491
A00571	A09530	227	A00571	A09563	227
A00571	A09613	152	A00571	A09622	177
A00571	A09664	364	A00571	A09726	329
A00571	A09735	491	A00571	A09852	414
A00571	A10117	385	A00571	A10137	227
A00571	A10338	227	A00571	A10339	152
A00571	A10480	227	A00571	A10498	195
A00571	A10586	154	A00571	A10607	326
A00571	A10909	152	A00571	A11277	469
A00571	A11364	205	A00571	A11380	210
A00571	A11383	323	A00571	A11480	188
A00571	A11593	277	A00571	A11597	167
A00571	A11616	423	A00571	A11780	167
A00571	A11882	455	A00571	A12011	323
A00571	A12059	167	A00571	A12160	259
A00571	A12477	296	A00571	A12523	167
A00571	A12598	152	A00571	A12821	408
A00571	A12923	160	A00571	A12924	195
A00571	A12966	270	A00571	A13144	322
A00571	A13497	188	A00571	A13527	215
A00571	A13583	188	A00571	A13769	227
A00571	A13782	152	A00571	A13905	249
A00571	A13989	491	A00571	A13992	167
A00571	A14006	390	A00571	A14026	232
A00571	A14073	436	A00571	A14216	418
A00571	A14443	171	A00571	A14449	276
A00571	A14478	468	A00571	A14479	491
A00571	A14482	488	A00571	A14508	361
A00571	A14566	313	A00571	A14568	491
A00571	A14594	190	A00571	A14648	205
A00571	A14699	480	A00571	A14813	356
A00571	A15499	374	A00571	A15500	491
A00571	A15624	440	A00571	A15657	276
A00571	A15659	424	A00571	A15660	467
A00571	A15684	491	A00571	A15751	188
A00571	A15771	489	A00571	A15920	174
A00571	A15932	177	A00571	A15988	340
A00571	A16035	308	A00571	A16053	167
A00571	A16118	338	A00571	A16324	350

A00571	A16410	240	A00571	A16680	188
A00571	A17063	338	A00571	A17152	463
A00572	A00575	221	A00572	A00600	475
A00572	A00622	205	A00572	A00655	467
A00572	A00694	475	A00572	A00805	364
A00572	A00817	491	A00572	A00818	474
A00572	A00819	491	A00572	A00822	491
A00572	A00858	462	A00572	A00860	420
A00572	A00863	156	A00572	A00881	291
A00572	A01156	163	A00572	A01169	417
A00572	A01195	419	A00572	A01200	411
A00572	A01325	438	A00572	A01383	491
A00572	A01385	491	A00572	A01401	430
A00572	A01474	449	A00572	A01612	227
A00572	A03525	490	A00572	A03526	474
A00572	A04141	380	A00572	A04265	159
A00572	A04570	190	A00572	A04648	193
A00572	A05915	302	A00572	A06803	419
A00572	A07004	467	A00572	A07290	279
A00572	A07885	201	A00572	A08591	361
A00572	A09093	361	A00572	A09672	187
A00572	A09675	302	A00572	A09712	452
A00572	A10642	486	A00572	A12296	441
A00572	A15295	219	A00572	A15660	450
A00572	A15876	187	A00572	A16964	243
A00573	A00574	423	A00573	A00633	168
A00573	A00669	187	A00573	A00696	328
A00573	A00930	334	A00573	A01008	303
A00573	A01175	210	A00573	A01227	185
A00573	A01233	266	A00573	A01297	157
A00573	A01360	435	A00573	A01409	262
A00573	A01572	450	A00573	A01625	462
A00573	A01635	414	A00573	A01726	334
A00573	A02290	347	A00573	A02301	152
A00573	A02311	165	A00573	A02316	232
A00573	A02525	291	A00573	A02758	488
A00573	A03016	308	A00573	A03027	187
A00573	A03164	369	A00573	A03167	369
A00573	A03234	313	A00573	A03484	160
A00573	A03513	469	A00573	A03917	215
A00573	A04229	391	A00573	A04231	447
A00573	A04556	213	A00573	A04781	234
A00573	A04791	469	A00573	A04832	165
A00573	A04927	358	A00573	A05007	441
A00573	A05034	334	A00573	A05050	245
A00573	A05103	262	A00573	A05140	238

A00573	A05289	334	A00573	A05337	177
A00573	A05358	330	A00573	A05416	396
A00573	A05448	224	A00573	A05451	255
A00573	A05452	271	A00573	A05463	193
A00573	A05472	357	A00573	A05548	221
A00573	A05615	234	A00573	A05841	348
A00573	A05883	334	A00573	A06315	267
A00573	A06385	393	A00573	A06598	334
A00573	A06629	491	A00573	A06640	177
A00573	A06641	319	A00573	A06689	238
A00573	A06734	356	A00573	A06914	352
A00573	A06970	418	A00573	A06994	236
A00573	A07002	281	A00573	A07238	215
A00573	A07256	457	A00573	A07264	301
A00573	A07384	184	A00573	A07477	408
A00573	A07508	337	A00573	A07520	188
A00573	A07690	188	A00573	A07711	232
A00573	A07728	334	A00573	A07736	311
A00573	A07763	177	A00573	A07973	443
A00573	A08135	201	A00573	A08199	210
A00573	A08707	334	A00573	A09182	334
A00573	A10141	302	A00573	A10694	223
A00573	A11189	319	A00573	A12705	236
A00573	A14252	334	A00573	A15356	359
A00573	A15498	195	A00573	A16741	229
A00574	A00614	490	A00574	A00628	491
A00574	A00633	469	A00574	A00654	757
A00574	A00660	463	A00574	A00662	490
A00574	A00663	480	A00574	A00666	491
A00574	A00681	491	A00574	A00695	486
A00574	A00696	469	A00574	A00738	491
A00574	A00739	484	A00574	A00770	491
A00574	A00771	488	A00574	A00773	491
A00574	A00825	473	A00574	A00843	333
A00574	A00962	491	A00574	A00970	334
A00574	A00995	491	A00574	A01005	479
A00574	A01008	491	A00574	A01018	491
A00574	A01046	475	A00574	A01063	405
A00574	A01115	313	A00574	A01165	299
A00574	A01174	490	A00574	A01175	485
A00574	A01225	160	A00574	A01229	462
A00574	A01230	429	A00574	A01233	491
A00574	A01242	319	A00574	A01256	491
A00574	A01272	474	A00574	A01277	267
A00574	A01297	489	A00574	A01347	491
A00574	A01351	190	A00574	A01360	462

A00574	A01367	371	A00574	A01397	396
A00574	A01409	490	A00574	A01481	490
A00574	A01496	324	A00574	A01508	479
A00574	A01511	243	A00574	A01548	360
A00574	A01569	369	A00574	A01576	160
A00574	A01635	491	A00574	A01713	441
A00574	A01735	340	A00574	A01741	456
A00574	A01743	433	A00574	A01747	393
A00574	A01841	412	A00574	A01855	380
A00574	A01857	401	A00574	A01899	152
A00574	A01926	419	A00574	A01937	274
A00574	A01972	441	A00574	A02097	199
A00574	A02148	224	A00574	A02156	391
A00574	A02168	278	A00574	A02252	160
A00574	A02254	443	A00574	A02316	361
A00574	A02330	252	A00574	A02353	782
A00574	A02432	384	A00574	A02521	463
A00574	A02695	470	A00574	A02718	724
A00574	A02847	466	A00574	A02859	396
A00574	A02922	229	A00574	A02992	489
A00574	A03003	401	A00574	A03004	230
A00574	A03005	306	A00574	A03008	270
A00574	A03016	486	A00574	A03050	475
A00574	A03059	371	A00574	A03167	486
A00574	A03168	463	A00574	A03259	366
A00574	A03311	302	A00574	A03370	281
A00574	A03429	344	A00574	A03441	465
A00574	A03442	235	A00574	A03443	238
A00574	A03477	791	A00574	A03484	288
A00574	A03501	312	A00574	A03508	199
A00574	A03513	474	A00574	A03545	205
A00574	A03586	238	A00574	A03783	267
A00574	A03858	182	A00574	A04211	432
A00574	A04229	430	A00574	A04369	229
A00574	A04674	213	A00574	A04701	173
A00574	A04778	396	A00574	A04779	486
A00574	A04791	374	A00574	A04834	479
A00574	A04839	303	A00574	A04850	238
A00574	A04880	160	A00574	A04883	243
A00574	A04927	332	A00574	A04936	301
A00574	A04937	361	A00574	A04995	486
A00574	A04998	319	A00574	A05006	465
A00574	A05007	491	A00574	A05020	790
A00574	A05045	274	A00574	A05088	177
A00574	A05098	185	A00574	A05102	486
A00574	A05310	484	A00574	A05311	173

A00574	A05337	207	A00574	A05376	172
A00574	A05390	205	A00574	A05393	174
A00574	A05416	491	A00574	A05418	223
A00574	A05459	177	A00574	A05472	445
A00574	A05516	469	A00574	A05532	224
A00574	A05573	327	A00574	A05600	258
A00574	A05659	347	A00574	A05713	301
A00574	A05754	224	A00574	A05755	301
A00574	A05757	293	A00574	A05759	380
A00574	A05771	199	A00574	A05772	157
A00574	A05774	447	A00574	A05789	157
A00574	A05820	235	A00574	A05974	152
A00574	A06086	451	A00574	A06088	288
A00574	A06089	213	A00574	A06091	296
A00574	A06252	477	A00574	A06301	251
A00574	A06358	491	A00574	A06359	791
A00574	A06360	314	A00574	A06386	235
A00574	A06405	462	A00574	A06439	460
A00574	A06473	491	A00574	A06474	182
A00574	A06523	173	A00574	A06555	251
A00574	A06592	348	A00574	A06597	170
A00574	A06633	324	A00574	A06641	319
A00574	A06689	489	A00574	A06692	297
A00574	A06699	154	A00574	A06700	486
A00574	A06756	491	A00574	A06831	177
A00574	A06912	232	A00574	A06914	160
A00574	A06934	364	A00574	A06970	451
A00574	A06979	477	A00574	A07002	199
A00574	A07059	371	A00574	A07080	479
A00574	A07088	477	A00574	A07109	486
A00574	A07122	491	A00574	A07238	477
A00574	A07256	480	A00574	A07267	489
A00574	A07271	224	A00574	A07304	450
A00574	A07376	260	A00574	A07381	218
A00574	A07385	352	A00574	A07413	491
A00574	A07459	337	A00574	A07477	490
A00574	A07503	152	A00574	A07507	187
A00574	A07520	430	A00574	A07530	457
A00574	A07553	466	A00574	A07554	291
A00574	A07558	481	A00574	A07590	489
A00574	A07627	414	A00574	A07674	491
A00574	A07688	491	A00574	A07721	372
A00574	A07736	489	A00574	A07737	245
A00574	A07746	424	A00574	A07757	296
A00574	A07800	491	A00574	A07844	188
A00574	A07847	180	A00574	A07848	420

A00574	A07933	490	A00574	A07973	490
A00574	A07976	218	A00574	A08099	456
A00574	A08108	306	A00574	A08135	338
A00574	A08252	276	A00574	A08318	157
A00574	A08687	152	A00574	A08751	294
A00574	A08817	378	A00574	A09043	160
A00574	A09612	386	A00574	A09711	251
A00574	A09837	168	A00574	A09876	389
A00574	A10027	301	A00574	A10121	199
A00574	A10147	301	A00574	A10229	301
A00574	A10420	329	A00574	A10424	160
A00574	A10427	294	A00574	A10437	168
A00574	A10443	267	A00574	A10444	446
A00574	A10452	405	A00574	A10455	177
A00574	A10456	491	A00574	A10532	402
A00574	A10656	467	A00574	A10659	177
A00574	A10677	481	A00574	A10681	347
A00574	A10686	358	A00574	A10693	202
A00574	A10694	265	A00574	A10702	483
A00574	A10703	449	A00574	A10708	157
A00574	A10710	188	A00574	A10712	419
A00574	A10714	468	A00574	A10763	301
A00574	A10885	238	A00574	A11309	491
A00574	A11325	361	A00574	A11381	480
A00574	A11431	466	A00574	A12055	324
A00574	A13326	160	A00574	A13330	210
A00574	A13362	302	A00574	A13609	367
A00574	A13677	301	A00574	A13735	229
A00574	A13871	162	A00574	A13915	193
A00574	A14196	263	A00574	A14803	335
A00574	A14827	185	A00574	A14856	446
A00574	A15282	196	A00574	A15497	465
A00574	A15637	290	A00574	A15874	490
A00574	A15905	454	A00574	A16002	411
A00574	A16026	475	A00574	A16027	490
A00574	A16033	484	A00574	A16048	277
A00574	A16262	480	A00574	A16286	423
A00574	A16309	270	A00574	A16344	334
A00574	A16355	302	A00574	A16419	258
A00574	A16528	193	A00574	A16529	491
A00574	A16636	157	A00574	A16741	467
A00574	A16836	213	A00574	A16959	490
A00574	A17006	258	A00574	A17007	219
A00574	A17078	489	A00574	A17194	251
A00575	A00605	489	A00575	A00622	699
A00575	A00626	383	A00575	A00665	433

A00575	A00708	491	A00575	A00733	538
A00575	A00744	253	A00575	A00760	294
A00575	A00805	541	A00575	A00819	279
A00575	A00831	188	A00575	A00856	488
A00575	A00858	715	A00575	A00860	713
A00575	A00866	156	A00575	A00881	541
A00575	A00903	176	A00575	A00934	445
A00575	A00954	290	A00575	A00978	528
A00575	A01007	629	A00575	A01019	538
A00575	A01020	491	A00575	A01032	456
A00575	A01075	474	A00575	A01087	414
A00575	A01116	491	A00575	A01184	447
A00575	A01195	534	A00575	A01200	712
A00575	A01206	445	A00575	A01212	450
A00575	A01234	486	A00575	A01325	406
A00575	A01383	385	A00575	A01385	156
A00575	A01398	541	A00575	A01401	714
A00575	A01429	208	A00575	A01468	489
A00575	A01500	541	A00575	A01503	479
A00575	A01555	316	A00575	A01576	473
A00575	A01720	466	A00575	A01741	152
A00575	A03068	170	A00575	A03207	213
A00575	A03526	407	A00575	A03629	205
A00575	A04068	213	A00575	A04648	534
A00575	A04687	156	A00575	A05027	274
A00575	A05419	268	A00575	A06803	540
A00575	A07223	172	A00575	A07290	162
A00575	A07670	508	A00575	A07700	451
A00575	A07850	398	A00575	A07887	487
A00575	A08022	486	A00575	A08024	323
A00575	A08199	173	A00575	A08362	281
A00575	A08382	278	A00575	A08383	271
A00575	A08430	305	A00575	A08518	195
A00575	A08553	452	A00575	A08818	156
A00575	A08952	191	A00575	A09502	156
A00575	A09712	480	A00575	A10642	328
A00575	A11344	156	A00575	A11674	156
A00575	A13413	249	A00575	A15733	391
A00575	A15945	310	A00575	A15946	204
A00575	A16144	380	A00575	A16228	505
A00576	A00577	491	A00576	A00609	430
A00576	A00636	477	A00576	A00640	152
A00576	A00653	476	A00576	A00694	419
A00576	A00719	162	A00576	A00726	299
A00576	A00737	196	A00576	A00751	173
A00576	A00799	477	A00576	A00802	489

A00576	A00810	152	A00576	A00821	168
A00576	A00822	286	A00576	A00833	299
A00576	A00841	283	A00576	A00849	160
A00576	A00863	329	A00576	A00868	205
A00576	A00871	200	A00576	A00904	176
A00576	A00911	472	A00576	A00939	480
A00576	A00974	299	A00576	A00984	458
A00576	A00985	491	A00576	A00987	365
A00576	A01001	580	A00576	A01004	281
A00576	A01027	491	A00576	A01067	157
A00576	A01075	486	A00576	A01077	223
A00576	A01100	467	A00576	A01102	484
A00576	A01139	301	A00576	A01156	491
A00576	A01161	301	A00576	A01276	190
A00576	A01311	152	A00576	A01313	167
A00576	A01326	490	A00576	A01329	236
A00576	A01352	278	A00576	A01379	320
A00576	A01385	491	A00576	A01387	299
A00576	A01413	278	A00576	A01439	299
A00576	A01551	212	A00576	A01577	152
A00576	A01582	272	A00576	A01585	491
A00576	A01599	268	A00576	A01627	618
A00576	A01632	470	A00576	A01728	151
A00576	A01738	154	A00576	A01741	375
A00576	A01758	160	A00576	A01787	257
A00576	A01819	215	A00576	A01826	221
A00576	A01983	167	A00576	A02040	176
A00576	A02324	385	A00576	A02477	270
A00576	A02491	218	A00576	A02601	299
A00576	A02620	238	A00576	A02691	254
A00576	A03241	162	A00576	A03259	152
A00576	A03304	491	A00576	A03473	363
A00576	A03562	218	A00576	A03598	241
A00576	A03697	152	A00576	A03902	191
A00576	A03945	162	A00576	A03955	177
A00576	A03959	389	A00576	A03992	210
A00576	A04124	185	A00576	A04275	441
A00576	A04337	485	A00576	A04495	299
A00576	A04668	167	A00576	A04947	229
A00576	A04987	247	A00576	A05087	196
A00576	A05323	465	A00576	A05601	185
A00576	A06225	188	A00576	A06287	152
A00576	A06293	205	A00576	A06328	251
A00576	A06580	306	A00576	A06686	162
A00576	A06868	152	A00576	A07197	174
A00576	A07237	176	A00576	A07442	477

A00576	A07483	310	A00576	A07744	152
A00576	A07894	183	A00576	A08061	152
A00576	A08098	152	A00576	A08178	152
A00576	A08517	180	A00576	A08567	173
A00576	A08763	152	A00576	A08947	299
A00576	A09004	299	A00576	A09351	182
A00576	A09939	232	A00576	A10321	484
A00576	A10532	246	A00576	A10742	238
A00576	A10842	152	A00576	A11348	174
A00576	A11558	216	A00576	A11565	185
A00576	A11714	238	A00576	A12789	288
A00576	A12824	152	A00576	A13124	193
A00576	A13369	160	A00576	A13885	198
A00576	A14112	218	A00576	A14202	159
A00576	A14272	485	A00576	A14405	335
A00576	A14525	152	A00576	A14589	205
A00576	A14648	185	A00576	A14838	389
A00576	A15375	152	A00576	A15422	461
A00576	A15546	152	A00576	A15598	167
A00576	A15809	160	A00576	A15824	167
A00576	A15858	167	A00576	A15917	152
A00576	A15932	491	A00576	A15933	390
A00576	A15949	154	A00576	A16064	243
A00576	A16613	490	A00576	A16750	306
A00577	A00636	446	A00577	A00694	347
A00577	A00799	279	A00577	A00802	217
A00577	A00863	446	A00577	A00985	160
A00577	A01027	363	A00577	A01156	391
A00577	A01326	182	A00577	A01385	159
A00577	A01434	208	A00577	A01599	246
A00577	A01627	338	A00577	A01741	219
A00577	A02745	224	A00577	A03241	269
A00577	A03304	458	A00577	A03959	156
A00577	A04275	188	A00577	A05812	229
A00577	A10321	368	A00577	A13124	188
A00577	A14405	218	A00577	A14838	154
A00577	A15422	427	A00577	A15434	202
A00577	A15498	314	A00577	A15596	167
A00577	A15932	427	A00577	A15933	452
A00577	A16613	490	A00578	A00829	218
A00578	A02830	363	A00578	A03115	283
A00578	A03763	389	A00578	A05309	208
A00578	A07483	212	A00579	A00628	177
A00579	A00695	294	A00579	A01256	299
A00579	A01397	302	A00579	A01508	191
A00579	A01695	449	A00579	A01696	475

A00579	A01748	299	A00579	A02026	249
A00579	A02114	205	A00579	A02290	353
A00579	A02366	451	A00579	A03167	247
A00579	A03168	308	A00579	A03661	218
A00579	A04211	480	A00579	A05363	332
A00579	A05466	414	A00579	A06236	414
A00579	A06633	335	A00579	A06970	208
A00579	A07770	424	A00579	A09985	368
A00579	A11309	262	A00579	A13343	424
A00579	A14176	294	A00579	A16027	263
A00579	A16028	344	A00579	A16966	424
A00580	A01041	473	A00580	A01309	405
A00580	A01687	152	A00580	A02061	294
A00580	A02499	267	A00580	A07054	195
A00580	A07483	246	A00580	A07944	466
A00580	A08352	288	A00580	A08535	321
A00580	A08536	409	A00580	A08550	179
A00580	A08552	311	A00580	A08561	201
A00580	A11456	296	A00580	A11472	235
A00580	A11675	262	A00580	A11706	326
A00580	A12506	290	A00580	A12507	152
A00580	A12508	274	A00580	A12658	270
A00580	A12858	235	A00580	A13116	314
A00580	A13785	363	A00580	A13786	293
A00580	A13787	374	A00580	A13969	252
A00580	A14286	326	A00580	A14569	281
A00580	A14838	202	A00580	A15189	249
A00580	A15855	163	A00580	A16157	184
A00580	A16552	167	A00580	A16664	258
A00580	A16776	340	A00580	A17144	318
A00581	A00597	318	A00581	A00609	486
A00581	A00617	288	A00581	A00642	335
A00581	A00718	466	A00581	A00778	486
A00581	A00826	419	A00581	A00840	241
A00581	A00888	201	A00581	A00907	215
A00581	A00947	224	A00581	A01002	310
A00581	A01016	245	A00581	A01026	466
A00581	A01074	232	A00581	A01166	401
A00581	A01209	256	A00581	A01217	232
A00581	A01306	319	A00581	A01313	229
A00581	A01352	235	A00581	A01365	286
A00581	A01377	395	A00581	A01386	423
A00581	A01406	227	A00581	A01416	229
A00581	A01689	390	A00581	A02572	375
A00581	A02925	179	A00581	A03601	260
A00581	A05337	232	A00581	A06046	241

A00581	A07693	491	A00581	A08113	246
A00581	A08263	420	A00581	A08346	491
A00581	A08402	491	A00581	A08502	163
A00581	A09164	268	A00581	A09648	348
A00581	A11184	156	A00581	A14077	486
A00581	A14209	168	A00581	A14272	473
A00581	A14288	313	A00581	A14707	251
A00581	A15452	274	A00581	A15877	414
A00581	A16611	240	A00581	A16692	235
A00582	A00587	191	A00582	A00600	439
A00582	A00765	465	A00582	A00813	272
A00582	A00883	281	A00582	A01097	401
A00582	A01143	439	A00582	A01185	491
A00582	A01361	491	A00582	A01419	491
A00582	A01438	491	A00582	A01926	294
A00582	A01994	202	A00582	A03822	200
A00582	A04449	457	A00582	A04962	477
A00582	A06907	208	A00582	A07697	412
A00582	A07835	154	A00582	A10159	491
A00582	A11487	190	A00582	A12183	363
A00582	A13587	201	A00582	A13647	456
A00582	A13928	388	A00582	A14192	162
A00582	A14193	425	A00582	A14253	466
A00582	A14454	491	A00582	A15224	447
A00582	A15237	297	A00582	A15297	168
A00582	A15710	330	A00582	A16623	479
A00582	A16709	198	A00582	A16724	200
A00582	A16802	451	A00583	A00646	238
A00583	A00691	533	A00583	A00716	156
A00583	A00867	156	A00583	A00879	553
A00583	A00905	305	A00583	A00916	157
A00583	A00981	217	A00583	A01024	156
A00583	A01029	743	A00583	A01061	429
A00583	A01088	213	A00583	A01100	154
A00583	A01136	159	A00583	A01151	541
A00583	A01213	432	A00583	A01266	160
A00583	A01268	530	A00583	A01295	172
A00583	A01334	156	A00583	A01372	599
A00583	A01390	217	A00583	A01391	265
A00583	A01434	279	A00583	A01576	252
A00583	A01588	458	A00583	A01612	481
A00583	A03494	156	A00583	A03569	260
A00583	A03873	262	A00583	A07004	424
A00583	A07898	156	A00583	A07956	262
A00583	A08137	457	A00583	A08201	156
A00583	A08248	262	A00583	A08327	531

A00583	A08431	156	A00583	A08524	491
A00583	A08542	156	A00583	A08571	350
A00583	A08977	362	A00583	A08982	262
A00583	A10208	369	A00583	A11324	386
A00583	A11496	156	A00583	A11497	156
A00583	A11544	262	A00583	A11649	262
A00583	A12757	156	A00583	A13783	185
A00583	A13892	262	A00583	A13928	156
A00583	A14178	156	A00583	A14257	229
A00583	A14362	262	A00583	A14454	162
A00583	A15321	217	A00583	A15338	535
A00583	A15478	263	A00583	A15544	223
A00583	A15559	405	A00583	A15561	446
A00583	A15564	400	A00583	A15729	207
A00583	A16465	168	A00583	A16614	262
A00583	A16690	345	A00583	A16699	156
A00583	A16831	238	A00583	A16866	229
A00584	A00595	551	A00584	A00749	174
A00584	A00767	457	A00584	A00908	174
A00584	A00916	314	A00584	A00918	450
A00584	A00960	548	A00584	A00992	174
A00584	A01131	185	A00584	A01193	174
A00584	A01234	276	A00584	A01279	210
A00584	A01351	538	A00584	A01424	494
A00584	A01492	174	A00584	A01542	218
A00584	A01879	183	A00584	A04715	157
A00584	A06698	174	A00584	A06797	249
A00584	A07244	233	A00584	A07432	174
A00584	A07524	174	A00584	A07533	348
A00584	A07537	286	A00584	A07584	332
A00584	A07625	174	A00584	A07924	174
A00584	A09664	249	A00584	A09707	205
A00584	A11462	332	A00584	A11544	174
A00584	A11831	174	A00584	A11832	174
A00584	A12097	318	A00584	A13792	361
A00585	A14594	63	A00586	A00588	491
A00586	A00725	491	A00586	A00759	293
A00586	A00761	283	A00586	A00775	430
A00586	A00784	489	A00586	A00814	294
A00586	A00950	470	A00586	A01014	255
A00586	A01064	491	A00586	A01093	491
A00586	A01099	491	A00586	A01114	491
A00586	A01187	491	A00586	A01197	480
A00586	A01212	196	A00586	A01322	344
A00586	A01331	212	A00586	A01341	187
A00586	A01415	475	A00586	A01686	449

A00586	A02180	407	A00586	A06242	369
A00586	A06864	440	A00586	A06982	217
A00586	A07319	288	A00586	A07346	429
A00586	A07534	152	A00586	A07603	303
A00586	A07996	195	A00586	A08117	249
A00586	A08141	232	A00586	A08208	398
A00586	A08212	490	A00586	A08516	408
A00586	A08526	455	A00586	A08769	210
A00586	A09598	454	A00586	A11236	329
A00586	A11724	247	A00586	A13913	488
A00586	A13949	480	A00586	A14079	196
A00586	A14138	216	A00586	A14282	212
A00586	A16127	208	A00586	A16226	414
A00586	A16437	201	A00586	A16613	196
A00586	A16616	183	A00586	A16626	483
A00587	A00588	156	A00587	A00600	491
A00587	A00601	491	A00587	A00609	173
A00587	A00660	246	A00587	A00697	191
A00587	A00743	362	A00587	A00765	491
A00587	A00813	491	A00587	A00939	468
A00587	A01161	489	A00587	A01213	288
A00587	A01298	255	A00587	A01343	449
A00587	A01419	604	A00587	A01438	719
A00587	A01451	417	A00587	A01522	193
A00587	A01542	213	A00587	A01576	567
A00587	A01595	251	A00587	A02574	358
A00587	A02611	252	A00587	A02768	163
A00587	A03034	480	A00587	A03104	408
A00587	A03523	401	A00587	A03524	484
A00587	A04275	312	A00587	A04454	252
A00587	A05234	389	A00587	A05356	177
A00587	A05678	179	A00587	A06092	491
A00587	A06520	383	A00587	A07069	467
A00587	A07107	167	A00587	A07276	422
A00587	A07697	491	A00587	A07756	201
A00587	A08097	372	A00587	A08251	266
A00587	A08293	418	A00587	A08345	319
A00587	A08356	477	A00587	A08362	358
A00587	A08831	443	A00587	A08956	171
A00587	A09323	447	A00587	A09461	204
A00587	A09659	369	A00587	A10619	160
A00587	A10987	407	A00587	A11559	152
A00587	A11591	210	A00587	A14212	434
A00587	A14253	229	A00587	A15224	212
A00587	A15295	160	A00587	A15314	352
A00587	A15336	366	A00587	A15612	366

A00587	A15917	167	A00587	A16623	196
A00588	A00725	491	A00588	A00759	468
A00588	A00761	488	A00588	A00775	491
A00588	A00778	484	A00588	A00784	491
A00588	A00814	491	A00588	A00824	241
A00588	A00868	165	A00588	A00911	491
A00588	A00950	491	A00588	A00989	385
A00588	A00993	151	A00588	A01004	480
A00588	A01021	491	A00588	A01059	424
A00588	A01064	491	A00588	A01093	491
A00588	A01099	491	A00588	A01106	246
A00588	A01114	491	A00588	A01124	249
A00588	A01151	213	A00588	A01187	491
A00588	A01189	486	A00588	A01197	462
A00588	A01212	419	A00588	A01296	484
A00588	A01320	419	A00588	A01321	254
A00588	A01322	491	A00588	A01325	443
A00588	A01331	486	A00588	A01341	491
A00588	A01375	307	A00588	A01388	491
A00588	A01404	425	A00588	A01415	491
A00588	A01468	472	A00588	A01536	491
A00588	A01559	491	A00588	A01589	491
A00588	A01594	491	A00588	A01616	491
A00588	A01625	489	A00588	A01659	491
A00588	A01680	474	A00588	A01686	491
A00588	A01690	360	A00588	A01741	463
A00588	A02058	404	A00588	A02180	486
A00588	A02216	215	A00588	A02731	479
A00588	A02806	491	A00588	A02847	489
A00588	A02887	475	A00588	A03057	489
A00588	A03301	468	A00588	A04667	204
A00588	A04668	491	A00588	A05218	398
A00588	A05334	491	A00588	A06242	491
A00588	A06297	457	A00588	A06606	271
A00588	A06851	398	A00588	A06864	491
A00588	A06924	183	A00588	A06982	491
A00588	A07079	249	A00588	A07319	491
A00588	A07332	172	A00588	A07346	491
A00588	A07466	362	A00588	A07480	491
A00588	A07534	491	A00588	A07603	491
A00588	A07647	469	A00588	A07902	449
A00588	A07996	491	A00588	A08000	475
A00588	A08029	424	A00588	A08030	491
A00588	A08117	486	A00588	A08141	477
A00588	A08208	491	A00588	A08212	491
A00588	A08402	217	A00588	A08516	491

A00588	A08526	491	A00588	A08669	491
A00588	A08694	358	A00588	A08769	491
A00588	A08820	489	A00588	A08912	240
A00588	A08919	369	A00588	A08964	390
A00588	A08995	491	A00588	A09000	491
A00588	A09001	469	A00588	A09135	190
A00588	A09288	489	A00588	A09301	162
A00588	A09434	162	A00588	A09598	491
A00588	A09653	260	A00588	A09654	180
A00588	A09706	474	A00588	A09762	491
A00588	A09765	294	A00588	A10083	195
A00588	A10250	232	A00588	A10373	491
A00588	A10565	219	A00588	A11236	491
A00588	A11540	486	A00588	A11703	490
A00588	A11829	490	A00588	A11880	466
A00588	A11916	491	A00588	A12064	183
A00588	A12190	294	A00588	A12556	491
A00588	A12625	489	A00588	A12749	419
A00588	A12760	219	A00588	A13103	294
A00588	A13332	285	A00588	A13486	183
A00588	A13913	490	A00588	A13949	491
A00588	A14076	491	A00588	A14079	491
A00588	A14094	491	A00588	A14223	490
A00588	A14230	491	A00588	A14273	488
A00588	A14282	491	A00588	A14297	332
A00588	A14460	491	A00588	A14477	357
A00588	A14609	174	A00588	A14674	474
A00588	A14694	491	A00588	A14706	490
A00588	A15367	434	A00588	A15369	491
A00588	A15371	477	A00588	A15524	456
A00588	A15531	218	A00588	A15539	323
A00588	A15654	458	A00588	A15714	313
A00588	A15837	491	A00588	A15876	165
A00588	A15939	443	A00588	A15941	491
A00588	A16073	288	A00588	A16127	491
A00588	A16128	491	A00588	A16129	491
A00588	A16132	193	A00588	A16134	465
A00588	A16226	491	A00588	A16294	488
A00588	A16299	455	A00588	A16378	489
A00588	A16606	491	A00588	A16613	491
A00588	A16616	489	A00588	A16626	491
A00588	A16646	469	A00588	A16754	486
A00588	A16760	208	A00588	A16762	491
A00589	A00605	572	A00589	A00609	193
A00589	A00648	297	A00589	A00749	210
A00589	A00832	413	A00589	A00839	235

A00589	A00866	176	A00589	A00870	252
A00589	A00872	502	A00589	A00876	172
A00589	A00883	217	A00589	A00916	488
A00589	A00945	449	A00589	A01028	502
A00589	A01056	479	A00589	A01065	714
A00589	A01072	172	A00589	A01075	489
A00589	A01077	341	A00589	A01087	172
A00589	A01092	210	A00589	A01098	210
A00589	A01120	714	A00589	A01140	424
A00589	A01150	438	A00589	A01166	490
A00589	A01179	491	A00589	A01206	491
A00589	A01234	488	A00589	A01291	461
A00589	A01356	534	A00589	A01379	249
A00589	A01384	461	A00589	A01425	409
A00589	A01429	185	A00589	A01430	452
A00589	A01439	563	A00589	A01440	475
A00589	A01457	191	A00589	A01468	185
A00589	A01470	488	A00589	A01492	570
A00589	A01495	219	A00589	A01538	502
A00589	A01597	235	A00589	A01598	730
A00589	A01614	729	A00589	A01649	491
A00589	A01684	191	A00589	A02164	475
A00589	A02265	475	A00589	A02978	502
A00589	A05230	570	A00589	A05501	190
A00589	A06584	461	A00589	A06814	177
A00589	A06825	443	A00589	A06897	462
A00589	A07076	210	A00589	A07228	368
A00589	A07700	151	A00589	A07706	191
A00589	A07759	255	A00589	A08022	168
A00589	A08137	362	A00589	A08357	491
A00589	A08359	167	A00589	A08559	249
A00589	A09292	568	A00589	A09352	299
A00589	A09622	191	A00589	A09707	488
A00589	A10156	152	A00589	A10321	162
A00589	A10518	152	A00589	A11462	488
A00589	A11942	353	A00589	A13052	475
A00589	A13126	413	A00589	A13527	213
A00589	A13989	572	A00589	A14006	324
A00589	A14216	255	A00589	A14816	210
A00589	A15215	491	A00589	A15235	154
A00589	A15309	229	A00589	A15764	165
A00590	A00603	659	A00590	A00605	473
A00590	A00629	462	A00590	A00648	491
A00590	A00651	429	A00590	A00653	486
A00590	A00697	177	A00590	A00700	277
A00590	A00701	491	A00590	A00706	363

A00590	A00713	475	A00590	A00763	657
A00590	A00787	665	A00590	A00809	324
A00590	A00839	491	A00590	A00870	443
A00590	A00878	491	A00590	A00923	491
A00590	A00935	416	A00590	A00953	297
A00590	A00955	561	A00590	A00965	665
A00590	A00966	488	A00590	A00980	216
A00590	A00985	481	A00590	A00988	347
A00590	A00994	152	A00590	A01000	491
A00590	A01012	469	A00590	A01020	226
A00590	A01025	665	A00590	A01034	491
A00590	A01037	491	A00590	A01047	468
A00590	A01056	486	A00590	A01067	663
A00590	A01068	227	A00590	A01072	452
A00590	A01085	491	A00590	A01090	490
A00590	A01091	229	A00590	A01095	491
A00590	A01100	297	A00590	A01101	477
A00590	A01112	391	A00590	A01127	491
A00590	A01140	486	A00590	A01154	246
A00590	A01184	479	A00590	A01186	386
A00590	A01206	489	A00590	A01238	436
A00590	A01247	484	A00590	A01249	652
A00590	A01263	491	A00590	A01291	667
A00590	A01306	468	A00590	A01335	597
A00590	A01338	433	A00590	A01365	474
A00590	A01392	664	A00590	A01416	489
A00590	A01433	665	A00590	A01436	664
A00590	A01437	491	A00590	A01446	165
A00590	A01448	180	A00590	A01457	486
A00590	A01458	358	A00590	A01464	436
A00590	A01468	458	A00590	A01496	424
A00590	A01579	458	A00590	A01586	251
A00590	A01596	198	A00590	A01649	276
A00590	A01718	383	A00590	A01741	395
A00590	A01787	191	A00590	A02270	162
A00590	A02874	182	A00590	A02966	210
A00590	A03048	297	A00590	A03069	440
A00590	A03490	374	A00590	A03567	474
A00590	A03676	185	A00590	A04507	265
A00590	A04760	200	A00590	A05323	257
A00590	A05411	410	A00590	A05443	382
A00590	A05501	340	A00590	A06351	472
A00590	A06805	218	A00590	A06812	326
A00590	A06814	462	A00590	A06861	159
A00590	A06897	235	A00590	A06965	488
A00590	A07201	496	A00590	A07360	333

A00590	A07412	204	A00590	A07451	488
A00590	A07584	198	A00590	A08011	665
A00590	A08055	658	A00590	A08357	386
A00590	A08359	405	A00590	A08377	456
A00590	A08398	419	A00590	A08452	485
A00590	A08457	151	A00590	A08501	173
A00590	A08543	467	A00590	A08550	658
A00590	A08556	455	A00590	A08559	350
A00590	A08773	659	A00590	A08972	235
A00590	A08973	491	A00590	A09322	324
A00590	A09332	488	A00590	A09376	229
A00590	A09735	454	A00590	A09788	398
A00590	A09852	152	A00590	A11254	291
A00590	A11334	475	A00590	A11390	445
A00590	A11438	404	A00590	A11480	399
A00590	A11518	218	A00590	A11728	252
A00590	A11945	335	A00590	A11992	486
A00590	A12052	218	A00590	A12433	491
A00590	A12525	200	A00590	A12918	176
A00590	A13055	319	A00590	A13249	650
A00590	A13340	273	A00590	A13525	411
A00590	A13626	367	A00590	A13989	491
A00590	A13992	477	A00590	A14167	255
A00590	A14216	491	A00590	A14447	357
A00590	A14449	462	A00590	A14479	417
A00590	A14482	488	A00590	A14505	185
A00590	A15029	339	A00590	A15218	252
A00590	A15221	400	A00590	A15235	445
A00590	A15500	383	A00590	A15666	389
A00590	A15715	185	A00590	A15771	343
A00590	A15932	303	A00590	A15988	658
A00590	A16053	245	A00590	A16157	281
A00590	A16159	208	A00590	A16631	396
A00590	A16793	274	A00590	A16805	193
A00591	A01004	468	A00591	A01183	358
A00591	A01661	477	A00591	A01870	177
A00591	A02240	249	A00591	A02416	180
A00591	A02458	246	A00591	A03079	461
A00591	A03724	479	A00591	A03732	270
A00591	A03899	429	A00591	A03945	299
A00591	A04337	290	A00591	A05439	204
A00591	A06415	290	A00591	A07152	221
A00591	A08097	216	A00591	A09204	270
A00591	A09205	485	A00591	A09307	257
A00591	A09734	357	A00591	A09743	249
A00591	A09745	257	A00591	A13987	301

A00591	A14391	201	A00591	A14611	446
A00591	A15313	154	A00591	A15352	266
A00591	A15422	198	A00591	A15431	176
A00591	A15433	293	A00591	A15461	243
A00591	A15886	369	A00591	A16117	445
A00592	A11917	469	A00592	A13313	443
A00592	A15665	235	A00593	A00653	276
A00593	A00673	188	A00593	A00687	188
A00593	A00705	557	A00593	A00709	188
A00593	A00711	205	A00593	A00782	368
A00593	A00839	556	A00593	A00953	188
A00593	A00972	188	A00593	A01017	559
A00593	A01090	303	A00593	A01101	188
A00593	A01112	396	A00593	A01206	299
A00593	A01217	239	A00593	A01220	188
A00593	A01306	462	A00593	A01335	489
A00593	A01337	484	A00593	A01362	425
A00593	A01365	179	A00593	A01369	474
A00593	A01406	188	A00593	A01408	188
A00593	A01414	188	A00593	A01451	188
A00593	A01579	486	A00593	A01976	188
A00593	A01988	188	A00593	A02925	200
A00593	A03567	235	A00593	A03901	188
A00593	A05501	179	A00593	A05702	188
A00593	A06564	328	A00593	A06785	531
A00593	A07047	204	A00593	A07581	406
A00593	A07925	394	A00593	A07965	538
A00593	A08131	461	A00593	A08154	246
A00593	A08358	162	A00593	A08359	450
A00593	A09726	286	A00593	A11338	182
A00593	A11480	188	A00593	A12821	474
A00593	A13734	159	A00593	A14479	170
A00593	A14508	407	A00593	A14813	188
A00593	A15500	434	A00593	A15657	188
A00593	A15771	177	A00593	A16035	414
A00593	A16680	188	A00594	A00658	154
A00594	A00687	272	A00594	A00689	281
A00594	A00846	224	A00594	A00854	318
A00594	A00972	196	A00594	A01065	212
A00594	A01098	226	A00594	A01221	238
A00594	A01234	340	A00594	A01418	301
A00594	A01468	190	A00594	A01535	170
A00594	A01536	396	A00594	A03051	228
A00594	A03227	260	A00594	A03567	201
A00594	A05275	322	A00594	A06551	364
A00594	A06832	208	A00594	A07583	424

A00594	A11493	213	A00594	A13594	216
A00594	A14597	165	A00594	A14701	404
A00594	A16128	167	A00595	A00603	221
A00595	A00605	475	A00595	A00648	489
A00595	A00651	190	A00595	A00713	531
A00595	A00749	174	A00595	A00767	552
A00595	A00774	205	A00595	A00861	170
A00595	A00872	170	A00595	A00878	402
A00595	A00905	259	A00595	A00908	174
A00595	A00916	634	A00595	A00918	546
A00595	A00934	192	A00595	A00960	551
A00595	A00965	347	A00595	A00992	628
A00595	A01020	488	A00595	A01025	278
A00595	A01028	170	A00595	A01115	216
A00595	A01116	207	A00595	A01120	170
A00595	A01129	170	A00595	A01160	170
A00595	A01166	185	A00595	A01168	170
A00595	A01184	307	A00595	A01193	391
A00595	A01201	402	A00595	A01206	470
A00595	A01234	491	A00595	A01249	402
A00595	A01279	543	A00595	A01291	445
A00595	A01294	232	A00595	A01304	196
A00595	A01351	552	A00595	A01356	170
A00595	A01424	547	A00595	A01425	170
A00595	A01439	201	A00595	A01451	339
A00595	A01457	168	A00595	A01468	294
A00595	A01488	417	A00595	A01492	534
A00595	A01538	170	A00595	A01542	168
A00595	A01583	312	A00595	A01584	173
A00595	A01598	489	A00595	A01612	400
A00595	A01614	340	A00595	A01649	460
A00595	A01715	283	A00595	A01741	443
A00595	A02265	170	A00595	A03438	209
A00595	A03494	191	A00595	A03569	371
A00595	A03626	462	A00595	A03652	170
A00595	A03890	156	A00595	A03981	170
A00595	A04622	167	A00595	A04893	358
A00595	A05201	188	A00595	A05318	182
A00595	A05531	318	A00595	A06275	294
A00595	A06356	170	A00595	A06659	451
A00595	A06675	404	A00595	A06698	413
A00595	A06797	227	A00595	A06860	195
A00595	A06965	205	A00595	A07244	608
A00595	A07347	328	A00595	A07432	250
A00595	A07524	545	A00595	A07533	188
A00595	A07537	369	A00595	A07584	462

A00595	A07625	486	A00595	A07700	441
A00595	A07908	299	A00595	A07924	541
A00595	A08055	322	A00595	A08137	326
A00595	A08211	201	A00595	A08357	293
A00595	A09050	267	A00595	A09664	430
A00595	A09697	170	A00595	A10465	171
A00595	A10616	357	A00595	A11544	174
A00595	A11831	448	A00595	A11832	235
A00595	A12097	439	A00595	A12821	198
A00595	A13052	170	A00595	A13792	472
A00595	A13989	546	A00595	A13992	249
A00595	A14693	307	A00595	A14953	267
A00595	A15215	326	A00595	A15764	213
A00595	A15988	384	A00595	A16302	445
A00596	A00600	467	A00596	A00601	401
A00596	A00765	366	A00596	A00813	277
A00596	A01073	310	A00596	A01213	427
A00596	A01298	435	A00596	A01438	188
A00596	A01576	477	A00596	A03195	294
A00596	A03432	297	A00596	A06092	241
A00596	A06195	229	A00596	A06769	243
A00596	A07107	301	A00596	A07276	346
A00596	A07380	374	A00596	A07697	468
A00596	A07814	374	A00596	A08293	241
A00596	A08356	215	A00596	A08362	407
A00596	A08831	271	A00596	A11991	380
A00596	A14212	168	A00596	A14213	215
A00596	A15345	151	A00596	A16109	257
A00596	A16831	278	A00597	A00609	270
A00597	A00611	396	A00597	A00643	398
A00597	A00667	221	A00597	A00694	302
A00597	A00736	252	A00597	A00747	358
A00597	A00762	340	A00597	A00791	561
A00597	A00793	251	A00597	A00813	402
A00597	A00826	367	A00597	A00850	177
A00597	A00868	408	A00597	A00883	378
A00597	A00887	352	A00597	A00888	490
A00597	A00965	224	A00597	A00977	345
A00597	A00981	288	A00597	A01074	397
A00597	A01154	260	A00597	A01166	358
A00597	A01209	268	A00597	A01217	290
A00597	A01306	281	A00597	A01308	358
A00597	A01352	396	A00597	A01365	329
A00597	A01367	154	A00597	A01386	380
A00597	A01406	299	A00597	A01416	243
A00597	A01461	227	A00597	A01527	297

A00597	A01537	450	A00597	A01576	160
A00597	A01716	477	A00597	A01727	299
A00597	A01739	219	A00597	A01741	340
A00597	A01766	241	A00597	A01784	170
A00597	A01993	216	A00597	A02001	430
A00597	A02006	213	A00597	A02105	356
A00597	A02180	467	A00597	A02202	230
A00597	A02219	168	A00597	A02220	232
A00597	A02411	428	A00597	A02471	185
A00597	A02663	188	A00597	A02728	322
A00597	A02744	224	A00597	A02841	163
A00597	A02877	265	A00597	A03057	479
A00597	A03070	183	A00597	A03535	226
A00597	A03598	188	A00597	A03601	402
A00597	A03728	183	A00597	A03834	180
A00597	A03872	154	A00597	A04001	188
A00597	A04002	196	A00597	A04264	270
A00597	A04509	205	A00597	A04555	193
A00597	A04623	327	A00597	A04647	159
A00597	A04743	196	A00597	A04874	152
A00597	A05009	484	A00597	A05327	229
A00597	A05375	271	A00597	A05539	278
A00597	A05619	177	A00597	A06085	241
A00597	A06130	398	A00597	A06264	411
A00597	A06606	491	A00597	A06607	293
A00597	A06621	299	A00597	A06849	262
A00597	A07013	322	A00597	A07242	154
A00597	A07339	276	A00597	A07519	215
A00597	A07768	454	A00597	A09025	322
A00597	A09370	177	A00597	A09548	205
A00597	A09620	223	A00597	A09840	163
A00597	A10781	159	A00597	A11475	399
A00597	A11759	193	A00597	A11792	167
A00597	A11998	274	A00597	A12824	497
A00597	A14173	247	A00597	A14180	440
A00597	A14247	179	A00597	A14713	205
A00597	A15141	185	A00597	A15372	219
A00597	A15575	229	A00597	A15837	405
A00597	A16298	260	A00598	A00604	491
A00598	A00608	435	A00598	A00610	224
A00598	A00649	489	A00598	A00653	491
A00598	A00697	491	A00598	A00731	414
A00598	A00737	202	A00598	A00741	490
A00598	A00745	207	A00598	A00758	483
A00598	A00780	346	A00598	A00783	479
A00598	A00785	490	A00598	A00790	486

A00598	A00809	484	A00598	A00935	491
A00598	A00953	491	A00598	A00974	452
A00598	A00987	162	A00598	A01025	160
A00598	A01027	489	A00598	A01042	229
A00598	A01081	224	A00598	A01086	491
A00598	A01090	423	A00598	A01108	491
A00598	A01161	491	A00598	A01204	483
A00598	A01210	470	A00598	A01246	418
A00598	A01336	414	A00598	A01346	488
A00598	A01399	490	A00598	A01435	491
A00598	A01443	321	A00598	A01446	350
A00598	A01447	486	A00598	A01448	340
A00598	A01540	485	A00598	A01711	160
A00598	A01741	361	A00598	A01827	286
A00598	A02040	182	A00598	A02607	477
A00598	A03713	338	A00598	A03740	174
A00598	A04895	485	A00598	A05417	339
A00598	A05473	154	A00598	A05801	293
A00598	A05839	201	A00598	A06746	310
A00598	A06959	405	A00598	A07086	346
A00598	A07115	436	A00598	A07237	436
A00598	A07350	335	A00598	A07362	208
A00598	A07394	488	A00598	A07412	180
A00598	A07569	401	A00598	A07597	154
A00598	A08055	374	A00598	A08222	235
A00598	A08315	162	A00598	A08653	204
A00598	A08835	294	A00598	A08987	276
A00598	A09616	364	A00598	A09709	208
A00598	A09720	270	A00598	A10199	364
A00598	A10535	491	A00598	A10553	322
A00598	A11015	281	A00598	A11322	218
A00598	A11374	240	A00598	A11419	411
A00598	A11427	341	A00598	A11677	190
A00598	A13809	258	A00598	A14566	188
A00598	A14845	372	A00598	A15653	486
A00598	A15666	490	A00598	A15765	488
A00598	A15933	160	A00598	A16403	270
A00599	A00687	411	A00599	A01159	223
A00599	A01461	469	A00599	A01574	454
A00599	A01585	198	A00599	A01824	249
A00599	A01831	337	A00599	A02108	179
A00599	A02170	260	A00599	A02364	210
A00599	A03228	241	A00599	A03881	356
A00599	A04589	193	A00599	A05446	227
A00599	A06122	263	A00599	A06369	216
A00599	A06425	321	A00599	A06436	321

A00599	A10590	285	A00599	A14156	311
A00599	A14527	329	A00599	A15750	262
A00599	A15951	308	A00600	A00601	607
A00600	A00691	452	A00600	A00697	257
A00600	A00765	491	A00600	A00813	491
A00600	A00862	361	A00600	A00868	384
A00600	A00911	327	A00600	A00939	491
A00600	A00973	74	A00600	A01022	302
A00600	A01073	474	A00600	A01156	294
A00600	A01161	491	A00600	A01185	375
A00600	A01211	252	A00600	A01213	606
A00600	A01215	177	A00600	A01241	456
A00600	A01298	450	A00600	A01329	475
A00600	A01343	329	A00600	A01360	160
A00600	A01361	207	A00600	A01385	358
A00600	A01419	473	A00600	A01438	607
A00600	A01451	480	A00600	A01519	151
A00600	A01520	477	A00600	A01522	452
A00600	A01524	207	A00600	A01527	423
A00600	A01542	177	A00600	A01572	207
A00600	A01573	302	A00600	A01576	491
A00600	A01582	160	A00600	A01695	319
A00600	A01860	174	A00600	A01908	427
A00600	A02144	160	A00600	A02148	193
A00600	A02257	63	A00600	A02278	160
A00600	A02284	473	A00600	A02333	216
A00600	A02443	173	A00600	A02499	484
A00600	A02574	310	A00600	A02601	63
A00600	A02619	162	A00600	A02664	168
A00600	A02719	157	A00600	A02935	195
A00600	A02965	450	A00600	A03009	179
A00600	A03034	491	A00600	A03036	179
A00600	A03104	477	A00600	A03195	396
A00600	A03205	490	A00600	A03293	378
A00600	A03335	329	A00600	A03417	218
A00600	A03523	489	A00600	A03524	486
A00600	A03625	246	A00600	A03628	173
A00600	A03686	259	A00600	A04012	277
A00600	A04195	185	A00600	A04275	307
A00600	A04276	218	A00600	A04337	374
A00600	A04731	413	A00600	A04990	162
A00600	A05234	469	A00600	A05405	168
A00600	A05407	152	A00600	A05602	258
A00600	A05633	230	A00600	A06037	218
A00600	A06042	258	A00600	A06092	491
A00600	A06195	479	A00600	A06480	185

A00600	A06520	491	A00600	A06701	263
A00600	A06708	168	A00600	A06769	252
A00600	A06846	193	A00600	A07069	490
A00600	A07106	461	A00600	A07107	170
A00600	A07209	286	A00600	A07276	480
A00600	A07310	238	A00600	A07380	312
A00600	A07389	193	A00600	A07447	258
A00600	A07605	436	A00600	A07697	491
A00600	A07826	488	A00600	A07940	168
A00600	A08091	472	A00600	A08097	290
A00600	A08235	360	A00600	A08243	291
A00600	A08251	382	A00600	A08293	602
A00600	A08356	561	A00600	A08362	527
A00600	A08426	210	A00600	A08473	223
A00600	A08608	452	A00600	A08758	162
A00600	A08821	515	A00600	A08831	595
A00600	A08956	605	A00600	A09287	291
A00600	A09291	152	A00600	A09315	457
A00600	A09323	467	A00600	A09335	193
A00600	A09514	201	A00600	A09560	201
A00600	A09659	446	A00600	A09743	276
A00600	A09878	297	A00600	A09996	276
A00600	A10156	229	A00600	A10159	386
A00600	A10214	285	A00600	A10226	159
A00600	A10288	210	A00600	A10437	154
A00600	A10846	210	A00600	A10987	263
A00600	A11101	486	A00600	A11377	333
A00600	A11862	213	A00600	A11929	283
A00600	A11991	324	A00600	A12260	223
A00600	A12464	351	A00600	A12523	238
A00600	A12606	276	A00600	A12633	296
A00600	A12931	382	A00600	A13043	258
A00600	A13088	291	A00600	A13301	291
A00600	A13361	435	A00600	A13362	154
A00600	A13824	460	A00600	A13983	441
A00600	A14196	418	A00600	A14208	463
A00600	A14212	276	A00600	A14213	320
A00600	A14214	276	A00600	A14253	252
A00600	A14362	168	A00600	A14381	484
A00600	A14594	170	A00600	A14635	165
A00600	A14648	151	A00600	A14936	172
A00600	A14990	152	A00600	A15295	488
A00600	A15314	422	A00600	A15323	520
A00600	A15386	383	A00600	A15498	457
A00600	A15596	750	A00600	A16005	291
A00600	A16024	201	A00600	A16316	393

A00600	A16363	221	A00600	A16449	306
A00600	A16459	210	A00600	A16702	276
A00600	A16719	173	A00601	A00653	252
A00601	A00674	238	A00601	A00697	288
A00601	A00761	198	A00601	A00765	486
A00601	A00766	477	A00601	A00787	283
A00601	A00813	491	A00601	A00868	491
A00601	A00883	288	A00601	A00889	218
A00601	A00890	435	A00601	A00892	435
A00601	A00915	432	A00601	A00926	491
A00601	A00939	488	A00601	A00949	243
A00601	A00975	467	A00601	A01020	168
A00601	A01073	154	A00601	A01095	177
A00601	A01099	413	A00601	A01108	255
A00601	A01137	299	A00601	A01161	486
A00601	A01162	247	A00601	A01169	208
A00601	A01212	224	A00601	A01213	350
A00601	A01215	157	A00601	A01222	185
A00601	A01234	270	A00601	A01298	230
A00601	A01322	469	A00601	A01370	183
A00601	A01404	483	A00601	A01415	208
A00601	A01438	606	A00601	A01451	288
A00601	A01515	458	A00601	A01522	258
A00601	A01573	154	A00601	A01576	488
A00601	A01677	263	A00601	A01796	198
A00601	A01914	383	A00601	A01926	419
A00601	A02054	435	A00601	A02108	176
A00601	A02112	472	A00601	A02148	281
A00601	A02254	112	A00601	A02257	227
A00601	A02284	205	A00601	A02499	428
A00601	A02574	234	A00601	A02611	323
A00601	A02802	157	A00601	A02965	224
A00601	A02977	269	A00601	A03034	490
A00601	A03084	184	A00601	A03086	316
A00601	A03087	235	A00601	A03104	176
A00601	A03164	205	A00601	A03195	358
A00601	A03225	162	A00601	A03238	191
A00601	A03408	196	A00601	A03415	162
A00601	A03498	176	A00601	A03520	290
A00601	A03523	466	A00601	A03524	394
A00601	A03593	307	A00601	A03594	224
A00601	A03672	252	A00601	A03676	177
A00601	A04031	216	A00601	A04194	458
A00601	A04288	152	A00601	A04402	263
A00601	A04421	389	A00601	A04731	157
A00601	A04870	165	A00601	A04990	366

A00601	A05208	319	A00601	A05234	366
A00601	A05466	323	A00601	A05533	340
A00601	A05539	152	A00601	A06037	208
A00601	A06042	323	A00601	A06046	168
A00601	A06092	491	A00601	A06195	483
A00601	A06298	246	A00601	A06520	491
A00601	A06769	368	A00601	A07027	160
A00601	A07069	417	A00601	A07237	162
A00601	A07276	411	A00601	A07419	243
A00601	A07435	228	A00601	A07447	323
A00601	A07697	491	A00601	A07756	272
A00601	A07814	257	A00601	A07826	485
A00601	A07940	246	A00601	A07961	199
A00601	A08091	416	A00601	A08199	188
A00601	A08235	346	A00601	A08293	405
A00601	A08356	276	A00601	A08362	276
A00601	A08572	291	A00601	A08821	276
A00601	A08831	601	A00601	A08956	602
A00601	A09315	443	A00601	A09323	193
A00601	A09335	270	A00601	A09743	276
A00601	A09821	230	A00601	A09996	276
A00601	A10214	151	A00601	A10226	238
A00601	A10527	223	A00601	A10986	162
A00601	A11559	256	A00601	A11663	243
A00601	A12606	276	A00601	A12824	491
A00601	A12931	366	A00601	A13362	235
A00601	A13368	235	A00601	A13387	217
A00601	A13666	347	A00601	A13983	154
A00601	A14196	377	A00601	A14208	424
A00601	A14212	276	A00601	A14213	276
A00601	A14214	276	A00601	A14253	188
A00601	A14332	208	A00601	A14381	302
A00601	A14605	152	A00601	A15295	351
A00601	A15314	235	A00601	A15498	488
A00601	A16392	196	A00601	A16449	276
A00601	A16702	276	A00602	A03223	154
A00602	A15524	193	A00603	A00604	472
A00603	A00605	486	A00603	A00629	586
A00603	A00648	796	A00603	A00651	461
A00603	A00653	490	A00603	A00687	291
A00603	A00697	422	A00603	A00700	443
A00603	A00701	490	A00603	A00718	234
A00603	A00762	258	A00603	A00763	658
A00603	A00782	545	A00603	A00787	775
A00603	A00793	247	A00603	A00826	294
A00603	A00839	491	A00603	A00854	241

A00603	A00870	486	A00603	A00878	785
A00603	A00902	279	A00603	A00906	430
A00603	A00923	441	A00603	A00935	171
A00603	A00949	638	A00603	A00953	240
A00603	A00965	597	A00603	A00966	466
A00603	A00977	269	A00603	A00981	286
A00603	A00985	265	A00603	A01000	469
A00603	A01012	260	A00603	A01025	491
A00603	A01031	337	A00603	A01034	643
A00603	A01037	491	A00603	A01038	151
A00603	A01047	312	A00603	A01056	490
A00603	A01067	475	A00603	A01072	486
A00603	A01074	352	A00603	A01085	486
A00603	A01090	491	A00603	A01095	474
A00603	A01111	188	A00603	A01112	385
A00603	A01115	339	A00603	A01127	490
A00603	A01140	489	A00603	A01144	160
A00603	A01152	434	A00603	A01153	249
A00603	A01154	357	A00603	A01166	579
A00603	A01184	299	A00603	A01186	243
A00603	A01189	319	A00603	A01206	338
A00603	A01209	249	A00603	A01212	204
A00603	A01217	306	A00603	A01220	454
A00603	A01221	394	A00603	A01234	333
A00603	A01238	729	A00603	A01247	419
A00603	A01249	486	A00603	A01252	265
A00603	A01263	484	A00603	A01291	667
A00603	A01292	293	A00603	A01306	340
A00603	A01335	446	A00603	A01352	319
A00603	A01365	683	A00603	A01392	658
A00603	A01406	386	A00603	A01412	349
A00603	A01416	722	A00603	A01425	408
A00603	A01433	658	A00603	A01436	490
A00603	A01437	335	A00603	A01457	314
A00603	A01458	452	A00603	A01464	291
A00603	A01468	488	A00603	A01496	384
A00603	A01518	436	A00603	A01553	899
A00603	A01569	374	A00603	A01579	475
A00603	A01586	362	A00603	A01628	899
A00603	A01649	390	A00603	A01718	470
A00603	A01741	402	A00603	A02227	420
A00603	A02859	240	A00603	A03069	301
A00603	A03112	171	A00603	A03275	157
A00603	A03490	629	A00603	A03567	490
A00603	A03570	172	A00603	A03601	290
A00603	A03652	456	A00603	A03676	247

A00603	A03770	160	A00603	A03981	156
A00603	A04622	196	A00603	A05375	272
A00603	A05411	470	A00603	A05419	486
A00603	A05443	213	A00603	A05501	483
A00603	A05502	173	A00603	A06279	238
A00603	A06351	643	A00603	A06814	317
A00603	A06861	261	A00603	A06965	489
A00603	A07157	378	A00603	A07243	159
A00603	A07360	279	A00603	A07412	305
A00603	A07449	427	A00603	A07451	333
A00603	A07464	157	A00603	A08011	659
A00603	A08052	379	A00603	A08055	654
A00603	A08088	191	A00603	A08154	294
A00603	A08452	446	A00603	A08543	277
A00603	A08550	639	A00603	A08556	270
A00603	A08752	168	A00603	A08760	223
A00603	A08773	659	A00603	A08958	243
A00603	A08973	484	A00603	A09308	188
A00603	A09332	638	A00603	A09697	188
A00603	A09735	469	A00603	A09978	322
A00603	A10732	173	A00603	A10924	184
A00603	A11288	165	A00603	A11438	205
A00603	A11518	216	A00603	A11815	343
A00603	A11992	369	A00603	A12433	461
A00603	A12525	490	A00603	A12923	260
A00603	A13024	205	A00603	A13249	643
A00603	A13549	216	A00603	A13905	221
A00603	A13989	491	A00603	A13992	452
A00603	A14216	288	A00603	A14449	395
A00603	A14479	257	A00603	A14482	643
A00603	A14573	228	A00603	A14729	216
A00603	A15500	380	A00603	A15660	446
A00603	A15684	474	A00603	A15771	340
A00603	A15931	324	A00603	A15932	182
A00603	A15988	659	A00603	A16118	411
A00603	A16306	255	A00603	A16351	205
A00603	A16805	191	A00603	A16885	243
A00604	A00605	477	A00604	A00608	306
A00604	A00610	265	A00604	A00629	340
A00604	A00648	157	A00604	A00649	591
A00604	A00653	765	A00604	A00663	265
A00604	A00687	379	A00604	A00691	273
A00604	A00697	837	A00604	A00731	584
A00604	A00741	491	A00604	A00745	121
A00604	A00750	307	A00604	A00758	474
A00604	A00780	408	A00604	A00782	273

A00604	A00783	592	A00604	A00785	592
A00604	A00787	483	A00604	A00790	591
A00604	A00796	191	A00604	A00809	491
A00604	A00839	489	A00604	A00842	324
A00604	A00849	247	A00604	A00870	485
A00604	A00871	184	A00604	A00878	462
A00604	A00910	276	A00604	A00935	491
A00604	A00953	592	A00604	A00974	590
A00604	A00985	202	A00604	A00987	307
A00604	A01012	235	A00604	A01023	171
A00604	A01027	591	A00604	A01037	462
A00604	A01042	491	A00604	A01054	483
A00604	A01081	470	A00604	A01086	592
A00604	A01087	160	A00604	A01108	592
A00604	A01150	588	A00604	A01155	241
A00604	A01161	652	A00604	A01186	152
A00604	A01192	681	A00604	A01204	583
A00604	A01210	664	A00604	A01234	441
A00604	A01246	260	A00604	A01247	303
A00604	A01249	236	A00604	A01263	430
A00604	A01265	474	A00604	A01270	358
A00604	A01287	426	A00604	A01291	489
A00604	A01336	425	A00604	A01346	590
A00604	A01369	460	A00604	A01382	165
A00604	A01392	303	A00604	A01399	591
A00604	A01411	485	A00604	A01413	490
A00604	A01433	441	A00604	A01435	491
A00604	A01443	247	A00604	A01446	491
A00604	A01447	490	A00604	A01448	485
A00604	A01449	224	A00604	A01471	160
A00604	A01539	286	A00604	A01540	491
A00604	A01579	257	A00604	A01586	430
A00604	A01601	603	A00604	A01741	441
A00604	A01822	168	A00604	A01824	285
A00604	A01827	473	A00604	A01850	180
A00604	A02040	213	A00604	A02300	251
A00604	A02607	486	A00604	A02814	411
A00604	A02961	380	A00604	A02994	309
A00604	A03070	483	A00604	A03259	296
A00604	A03298	363	A00604	A03494	200
A00604	A03567	313	A00604	A03676	265
A00604	A03740	491	A00604	A03752	294
A00604	A03776	277	A00604	A03860	152
A00604	A03959	544	A00604	A04030	213
A00604	A04895	435	A00604	A05387	299
A00604	A05417	472	A00604	A05501	385

A00604	A05839	236	A00604	A06466	307
A00604	A06814	337	A00604	A06959	466
A00604	A06973	247	A00604	A07237	213
A00604	A07350	269	A00604	A07362	246
A00604	A07394	489	A00604	A07398	335
A00604	A07569	393	A00604	A07597	279
A00604	A07767	341	A00604	A07773	355
A00604	A07792	119	A00604	A07961	199
A00604	A08011	473	A00604	A08052	445
A00604	A08423	249	A00604	A08519	259
A00604	A08556	286	A00604	A08760	232
A00604	A08773	480	A00604	A08835	480
A00604	A08987	462	A00604	A09054	235
A00604	A09285	185	A00604	A09449	356
A00604	A09595	185	A00604	A09616	286
A00604	A09709	246	A00604	A09720	185
A00604	A10199	424	A00604	A10535	422
A00604	A10798	157	A00604	A11015	196
A00604	A11296	159	A00604	A11302	348
A00604	A11322	486	A00604	A11331	157
A00604	A11337	236	A00604	A11338	486
A00604	A11374	486	A00604	A11419	485
A00604	A11427	489	A00604	A11677	458
A00604	A11724	337	A00604	A11728	430
A00604	A12241	335	A00604	A12433	314
A00604	A12746	269	A00604	A13261	191
A00604	A13340	329	A00604	A13992	402
A00604	A14073	463	A00604	A14482	217
A00604	A14507	154	A00604	A14566	429
A00604	A14699	426	A00604	A14778	362
A00604	A14825	157	A00604	A14845	272
A00604	A15624	428	A00604	A15652	249
A00604	A15653	491	A00604	A15661	474
A00604	A15666	489	A00604	A15684	224
A00604	A15765	352	A00604	A16118	333
A00604	A16157	458	A00604	A16418	310
A00604	A16587	247	A00604	A17128	436
A00605	A00629	491	A00605	A00648	491
A00605	A00651	491	A00605	A00653	491
A00605	A00655	488	A00605	A00687	283
A00605	A00694	465	A00605	A00697	230
A00605	A00700	491	A00605	A00701	196
A00605	A00705	481	A00605	A00708	435
A00605	A00711	424	A00605	A00713	491
A00605	A00749	225	A00605	A00763	488
A00605	A00769	491	A00605	A00782	452

A00605	A00783	461	A00605	A00787	486
A00605	A00805	446	A00605	A00809	490
A00605	A00822	364	A00605	A00831	638
A00605	A00839	491	A00605	A00841	374
A00605	A00843	466	A00605	A00848	491
A00605	A00849	176	A00605	A00856	491
A00605	A00858	490	A00605	A00870	396
A00605	A00871	491	A00605	A00872	210
A00605	A00878	491	A00605	A00881	386
A00605	A00883	483	A00605	A00905	486
A00605	A00909	479	A00605	A00916	491
A00605	A00922	623	A00605	A00923	491
A00605	A00926	252	A00605	A00934	491
A00605	A00945	491	A00605	A00953	152
A00605	A00965	428	A00605	A00970	489
A00605	A00983	486	A00605	A00986	439
A00605	A00992	165	A00605	A00994	491
A00605	A01000	347	A00605	A01001	247
A00605	A01010	165	A00605	A01012	488
A00605	A01017	481	A00605	A01020	638
A00605	A01025	491	A00605	A01027	491
A00605	A01028	550	A00605	A01032	491
A00605	A01034	439	A00605	A01040	636
A00605	A01042	480	A00605	A01047	263
A00605	A01054	213	A00605	A01056	488
A00605	A01065	572	A00605	A01068	379
A00605	A01069	491	A00605	A01072	462
A00605	A01075	491	A00605	A01082	173
A00605	A01085	470	A00605	A01086	171
A00605	A01087	436	A00605	A01088	457
A00605	A01090	460	A00605	A01092	265
A00605	A01098	536	A00605	A01101	458
A00605	A01112	638	A00605	A01115	693
A00605	A01116	457	A00605	A01119	447
A00605	A01120	572	A00605	A01121	207
A00605	A01127	486	A00605	A01136	355
A00605	A01140	490	A00605	A01144	201
A00605	A01150	491	A00605	A01166	491
A00605	A01179	491	A00605	A01184	486
A00605	A01192	477	A00605	A01194	445
A00605	A01196	470	A00605	A01201	347
A00605	A01206	491	A00605	A01215	152
A00605	A01232	490	A00605	A01234	582
A00605	A01238	374	A00605	A01242	479
A00605	A01243	452	A00605	A01249	491
A00605	A01263	281	A00605	A01291	541

A00605	A01292	195	A00605	A01293	469
A00605	A01304	614	A00605	A01306	491
A00605	A01335	491	A00605	A01338	160
A00605	A01349	483	A00605	A01351	484
A00605	A01353	434	A00605	A01354	435
A00605	A01356	351	A00605	A01365	232
A00605	A01379	682	A00605	A01384	491
A00605	A01392	481	A00605	A01398	163
A00605	A01418	374	A00605	A01425	491
A00605	A01429	491	A00605	A01430	480
A00605	A01431	491	A00605	A01433	457
A00605	A01436	230	A00605	A01437	221
A00605	A01439	682	A00605	A01440	439
A00605	A01454	157	A00605	A01457	483
A00605	A01458	486	A00605	A01464	232
A00605	A01468	491	A00605	A01470	491
A00605	A01488	486	A00605	A01492	572
A00605	A01495	606	A00605	A01496	491
A00605	A01500	270	A00605	A01503	167
A00605	A01506	394	A00605	A01538	386
A00605	A01539	463	A00605	A01540	446
A00605	A01569	491	A00605	A01579	491
A00605	A01586	409	A00605	A01597	491
A00605	A01598	572	A00605	A01601	623
A00605	A01612	491	A00605	A01614	572
A00605	A01640	162	A00605	A01649	491
A00605	A01684	469	A00605	A01707	373
A00605	A01787	188	A00605	A01822	283
A00605	A02216	151	A00605	A02265	210
A00605	A02350	182	A00605	A02353	270
A00605	A02978	485	A00605	A03035	316
A00605	A03069	306	A00605	A03296	443
A00605	A03298	479	A00605	A03304	371
A00605	A03438	481	A00605	A03446	452
A00605	A03452	485	A00605	A03454	232
A00605	A03567	491	A00605	A03569	404
A00605	A03676	224	A00605	A03734	490
A00605	A03740	485	A00605	A03859	473
A00605	A03863	151	A00605	A03959	394
A00605	A04507	185	A00605	A04846	440
A00605	A05230	567	A00605	A05370	160
A00605	A05501	491	A00605	A05845	230
A00605	A06275	458	A00605	A06279	457
A00605	A06351	445	A00605	A06466	374
A00605	A06543	368	A00605	A06584	411
A00605	A06661	180	A00605	A06825	234

A00605	A06826	216	A00605	A06861	486
A00605	A06897	479	A00605	A06965	490
A00605	A06973	477	A00605	A07047	243
A00605	A07076	682	A00605	A07203	229
A00605	A07223	334	A00605	A07306	163
A00605	A07323	491	A00605	A07347	484
A00605	A07348	486	A00605	A07360	485
A00605	A07378	157	A00605	A07398	451
A00605	A07451	462	A00605	A07481	182
A00605	A07487	489	A00605	A07524	485
A00605	A07581	160	A00605	A07677	414
A00605	A07700	569	A00605	A07706	484
A00605	A07725	334	A00605	A07744	425
A00605	A07759	486	A00605	A07881	491
A00605	A07924	468	A00605	A08011	260
A00605	A08022	490	A00605	A08131	484
A00605	A08137	491	A00605	A08228	461
A00605	A08258	224	A00605	A08357	724
A00605	A08359	489	A00605	A08377	480
A00605	A08398	385	A00605	A08408	240
A00605	A08452	483	A00605	A08491	434
A00605	A08521	167	A00605	A08548	277
A00605	A08571	488	A00605	A08679	180
A00605	A08756	170	A00605	A08835	216
A00605	A08952	486	A00605	A08973	408
A00605	A09292	572	A00605	A09293	151
A00605	A09307	152	A00605	A09322	215
A00605	A09327	489	A00605	A09328	201
A00605	A09655	152	A00605	A09707	491
A00605	A09735	163	A00605	A09736	159
A00605	A10466	472	A00605	A10518	445
A00605	A10532	569	A00605	A10616	457
A00605	A10624	157	A00605	A10755	485
A00605	A10853	443	A00605	A11297	420
A00605	A11312	245	A00605	A11322	176
A00605	A11427	221	A00605	A11438	378
A00605	A11462	491	A00605	A11562	358
A00605	A11608	157	A00605	A11942	489
A00605	A11957	170	A00605	A12138	177
A00605	A12329	458	A00605	A12821	185
A00605	A13052	210	A00605	A13066	361
A00605	A13126	491	A00605	A13164	163
A00605	A13207	205	A00605	A13298	491
A00605	A13340	480	A00605	A13699	163
A00605	A13764	224	A00605	A13875	484
A00605	A13989	624	A00605	A13992	488

A00605	A14073	430	A00605	A14172	339
A00605	A14176	176	A00605	A14216	491
A00605	A14479	163	A00605	A14482	414
A00605	A14566	390	A00605	A14596	428
A00605	A14597	475	A00605	A14699	352
A00605	A14778	347	A00605	A14816	370
A00605	A14817	182	A00605	A15204	163
A00605	A15208	185	A00605	A15210	163
A00605	A15215	491	A00605	A15256	408
A00605	A15297	311	A00605	A15338	419
A00605	A15416	441	A00605	A15500	447
A00605	A15624	162	A00605	A15652	467
A00605	A15659	201	A00605	A15660	491
A00605	A15661	324	A00605	A15684	491
A00605	A15746	162	A00605	A15918	208
A00605	A15931	491	A00605	A15937	199
A00605	A15988	454	A00605	A16088	491
A00605	A16096	353	A00605	A16118	332
A00605	A16218	236	A00605	A16272	345
A00605	A17140	165	A00606	A00652	311
A00606	A00687	428	A00606	A00689	241
A00606	A00712	297	A00606	A00762	223
A00606	A00854	281	A00606	A00878	232
A00606	A00965	238	A00606	A00977	232
A00606	A01004	163	A00606	A01016	234
A00606	A01018	218	A00606	A01074	306
A00606	A01102	118	A00606	A01154	258
A00606	A01220	226	A00606	A01238	286
A00606	A01306	274	A00606	A01352	281
A00606	A01356	229	A00606	A01365	307
A00606	A01406	265	A00606	A01416	283
A00606	A01709	154	A00606	A01824	236
A00606	A02051	228	A00606	A02164	151
A00606	A03601	274	A00606	A03918	182
A00606	A05987	160	A00606	A06369	201
A00606	A06832	162	A00606	A07458	380
A00606	A08910	218	A00606	A08974	389
A00606	A10065	251	A00606	A14395	196
A00606	A14456	236	A00606	A14484	182
A00606	A14546	246	A00606	A15291	408
A00606	A15367	218	A00606	A15614	495
A00606	A15932	188	A00607	A00848	154
A00607	A00923	171	A00607	A00926	201
A00607	A01372	475	A00607	A03503	340
A00607	A08524	63	A00607	A08839	291
A00607	A12288	427	A00607	A14303	257

A00607	A15495	238	A00608	A00610	385
A00608	A00653	477	A00608	A00731	321
A00608	A00745	257	A00608	A00750	486
A00608	A00780	265	A00608	A00785	451
A00608	A00790	303	A00608	A00953	369
A00608	A00974	154	A00608	A01027	343
A00608	A01081	272	A00608	A01086	439
A00608	A01108	358	A00608	A01121	199
A00608	A01186	188	A00608	A01204	391
A00608	A01246	193	A00608	A01346	205
A00608	A01382	405	A00608	A01399	452
A00608	A01446	173	A00608	A03776	205
A00608	A04895	385	A00608	A05839	251
A00608	A07237	358	A00608	A07325	183
A00608	A07362	258	A00608	A07569	243
A00608	A08521	177	A00608	A08987	196
A00608	A09616	286	A00608	A11338	157
A00608	A11419	316	A00608	A11427	302
A00608	A16144	457	A00608	A16935	286
A00609	A00617	468	A00609	A00647	302
A00609	A00718	783	A00609	A00753	373
A00609	A00778	722	A00609	A00779	491
A00609	A00781	419	A00609	A00797	480
A00609	A00807	240	A00609	A00822	490
A00609	A00826	312	A00609	A00840	483
A00609	A00883	490	A00609	A00907	491
A00609	A00920	638	A00609	A00948	414
A00609	A00975	321	A00609	A00977	235
A00609	A01021	483	A00609	A01022	485
A00609	A01026	722	A00609	A01074	285
A00609	A01075	441	A00609	A01077	270
A00609	A01087	419	A00609	A01091	470
A00609	A01096	160	A00609	A01161	490
A00609	A01166	293	A00609	A01171	285
A00609	A01211	484	A00609	A01280	249
A00609	A01306	232	A00609	A01313	490
A00609	A01326	457	A00609	A01329	288
A00609	A01343	291	A00609	A01354	488
A00609	A01360	350	A00609	A01365	313
A00609	A01375	411	A00609	A01377	721
A00609	A01386	491	A00609	A01428	171
A00609	A01451	470	A00609	A01571	159
A00609	A01573	185	A00609	A01598	266
A00609	A01686	229	A00609	A01716	383
A00609	A01900	202	A00609	A02389	154
A00609	A02452	286	A00609	A02477	168

A00609	A02494	353	A00609	A02629	230
A00609	A02704	463	A00609	A02774	217
A00609	A02852	163	A00609	A02878	430
A00609	A03057	423	A00609	A03181	157
A00609	A03237	174	A00609	A03293	314
A00609	A03417	163	A00609	A03597	270
A00609	A03601	273	A00609	A04145	337
A00609	A04263	157	A00609	A04275	201
A00609	A04337	483	A00609	A04463	374
A00609	A04668	373	A00609	A04737	205
A00609	A05052	348	A00609	A05234	364
A00609	A05353	152	A00609	A05525	330
A00609	A05539	173	A00609	A05556	193
A00609	A06312	168	A00609	A06390	313
A00609	A06472	369	A00609	A07069	208
A00609	A07106	165	A00609	A07245	190
A00609	A07391	182	A00609	A07442	216
A00609	A07558	385	A00609	A07605	270
A00609	A07621	319	A00609	A07693	488
A00609	A07736	205	A00609	A07976	435
A00609	A08113	586	A00609	A08137	423
A00609	A08263	484	A00609	A08346	483
A00609	A08400	199	A00609	A08402	491
A00609	A08656	230	A00609	A08694	240
A00609	A08975	358	A00609	A09135	436
A00609	A09136	216	A00609	A09271	240
A00609	A09648	717	A00609	A09857	260
A00609	A10156	435	A00609	A10289	190
A00609	A10321	491	A00609	A10420	224
A00609	A10424	177	A00609	A10664	188
A00609	A11297	235	A00609	A11324	469
A00609	A11965	165	A00609	A12863	152
A00609	A13785	458	A00609	A13873	475
A00609	A13905	321	A00609	A13963	205
A00609	A14077	491	A00609	A14092	491
A00609	A14191	490	A00609	A14196	490
A00609	A14220	450	A00609	A14272	722
A00609	A14288	333	A00609	A14381	316
A00609	A14707	458	A00609	A14840	327
A00609	A15256	174	A00609	A15367	491
A00609	A15383	480	A00609	A15419	347
A00609	A15431	195	A00609	A15559	243
A00609	A15596	249	A00609	A15752	160
A00609	A15763	204	A00609	A15877	489
A00609	A16037	252	A00609	A16406	160
A00610	A00649	329	A00610	A00653	440

A00610	A00731	396	A00610	A00780	461
A00610	A00785	356	A00610	A00790	326
A00610	A00842	445	A00610	A00953	266
A00610	A00974	434	A00610	A01023	440
A00610	A01027	193	A00610	A01081	430
A00610	A01086	457	A00610	A01108	391
A00610	A01184	269	A00610	A01204	391
A00610	A01246	294	A00610	A01346	196
A00610	A01399	168	A00610	A01446	172
A00610	A01448	341	A00610	A02814	273
A00610	A04895	386	A00610	A05839	424
A00610	A07237	324	A00610	A07362	340
A00610	A07569	330	A00610	A09616	361
A00610	A10799	283	A00610	A16591	396
A00611	A01716	322	A00611	A02018	326
A00611	A02180	154	A00611	A02191	317
A00611	A03070	196	A00611	A03498	428
A00611	A04874	432	A00611	A06253	420
A00611	A06607	207	A00611	A07803	296
A00611	A14128	296	A00612	A04065	411
A00613	A00968	310	A00613	A01074	235
A00613	A01365	246	A00613	A01647	477
A00613	A02548	338	A00613	A03880	324
A00613	A05016	221	A00613	A05301	156
A00613	A05563	246	A00613	A05998	871
A00613	A07981	375	A00613	A08050	812
A00613	A08069	825	A00613	A08297	63
A00613	A08351	258	A00613	A08411	104
A00613	A09054	208	A00613	A10377	740
A00613	A10378	929	A00613	A11202	358
A00613	A13415	430	A00613	A13920	433
A00614	A00628	458	A00614	A00654	491
A00614	A00660	154	A00614	A00666	470
A00614	A00670	223	A00614	A00681	279
A00614	A00695	170	A00614	A00738	469
A00614	A00739	391	A00614	A00770	477
A00614	A00773	232	A00614	A00962	489
A00614	A00968	224	A00614	A00995	224
A00614	A01005	422	A00614	A01008	474
A00614	A01018	213	A00614	A01063	191
A00614	A01074	223	A00614	A01109	352
A00614	A01174	424	A00614	A01175	259
A00614	A01189	223	A00614	A01233	215
A00614	A01256	414	A00614	A01272	333
A00614	A01360	314	A00614	A01408	151
A00614	A01481	419	A00614	A01508	483

A00614	A01511	243	A00614	A01635	456
A00614	A01794	221	A00614	A01857	469
A00614	A01866	252	A00614	A01867	210
A00614	A01995	221	A00614	A02138	262
A00614	A02154	241	A00614	A02227	251
A00614	A02319	329	A00614	A02335	223
A00614	A02353	313	A00614	A02363	438
A00614	A02541	238	A00614	A02546	291
A00614	A02574	171	A00614	A02593	330
A00614	A02642	154	A00614	A02718	238
A00614	A02789	223	A00614	A03003	438
A00614	A03004	234	A00614	A03005	291
A00614	A03016	213	A00614	A03224	191
A00614	A03243	152	A00614	A03264	245
A00614	A03441	483	A00614	A03443	424
A00614	A03468	232	A00614	A03477	484
A00614	A03500	477	A00614	A03501	414
A00614	A03719	223	A00614	A03728	223
A00614	A03752	223	A00614	A04372	312
A00614	A04620	270	A00614	A04779	346
A00614	A04834	452	A00614	A04995	408
A00614	A05007	302	A00614	A05013	318
A00614	A05020	201	A00614	A05102	188
A00614	A05262	483	A00614	A05416	260
A00614	A05463	232	A00614	A05472	305
A00614	A05516	210	A00614	A05550	191
A00614	A05573	168	A00614	A05772	279
A00614	A05774	270	A00614	A05859	288
A00614	A06101	230	A00614	A06211	279
A00614	A06252	374	A00614	A06309	183
A00614	A06358	165	A00614	A06558	338
A00614	A06689	223	A00614	A06700	154
A00614	A06756	458	A00614	A06819	420
A00614	A06979	490	A00614	A07080	388
A00614	A07109	450	A00614	A07122	457
A00614	A07222	223	A00614	A07267	333
A00614	A07333	223	A00614	A07413	374
A00614	A07710	313	A00614	A07746	198
A00614	A07800	434	A00614	A07931	167
A00614	A08193	489	A00614	A09711	347
A00614	A10701	168	A00614	A11309	262
A00614	A13822	223	A00614	A13906	270
A00614	A14525	278	A00614	A14685	318
A00614	A14804	223	A00614	A15372	223
A00614	A15497	262	A00614	A15637	462
A00614	A15874	255	A00614	A15905	157

A00614	A16026	413	A00614	A16028	425
A00614	A16219	223	A00614	A16419	254
A00614	A16531	223	A00614	A16753	184
A00614	A17155	256	A00615	A00981	227
A00615	A01143	411	A00615	A03141	177
A00615	A15274	335	A00615	A15321	469
A00615	A15944	311	A00615	A16724	224
A00616	A00736	294	A00616	A00752	327
A00616	A00799	388	A00616	A00815	407
A00616	A00900	243	A00616	A01137	491
A00616	A01138	216	A00616	A01161	172
A00616	A01163	179	A00616	A01257	490
A00616	A01277	224	A00616	A01425	63
A00616	A01477	251	A00616	A01485	238
A00616	A01527	899	A00616	A01590	488
A00616	A01604	167	A00616	A01605	940
A00616	A01626	179	A00616	A01639	899
A00616	A01654	180	A00616	A01668	345
A00616	A01722	168	A00616	A01888	251
A00616	A01908	252	A00616	A01926	63
A00616	A01983	491	A00616	A02021	154
A00616	A02074	196	A00616	A02083	408
A00616	A02107	201	A00616	A02111	408
A00616	A02132	258	A00616	A02136	385
A00616	A02138	208	A00616	A02142	475
A00616	A02143	119	A00616	A02148	460
A00616	A02176	162	A00616	A02213	179
A00616	A02237	204	A00616	A02240	180
A00616	A02245	472	A00616	A02246	278
A00616	A02298	462	A00616	A02305	385
A00616	A02306	485	A00616	A02309	486
A00616	A02313	452	A00616	A02326	467
A00616	A02333	174	A00616	A02346	227
A00616	A02367	334	A00616	A02393	291
A00616	A02396	334	A00616	A02397	257
A00616	A02405	462	A00616	A02412	160
A00616	A02419	234	A00616	A02423	466
A00616	A02425	457	A00616	A02434	159
A00616	A02444	190	A00616	A02451	430
A00616	A02461	399	A00616	A02464	465
A00616	A02465	469	A00616	A02468	218
A00616	A02490	480	A00616	A02491	416
A00616	A02501	216	A00616	A02508	190
A00616	A02587	204	A00616	A02608	183
A00616	A02618	162	A00616	A02620	204
A00616	A02624	278	A00616	A02644	486

A00616	A02667	456	A00616	A02668	466
A00616	A02670	307	A00616	A02756	462
A00616	A02759	452	A00616	A02764	198
A00616	A02772	477	A00616	A02785	364
A00616	A02803	432	A00616	A02807	213
A00616	A02854	210	A00616	A02917	208
A00616	A02935	271	A00616	A02945	285
A00616	A02952	171	A00616	A03049	191
A00616	A03053	154	A00616	A03056	238
A00616	A03062	195	A00616	A03063	251
A00616	A03099	441	A00616	A03110	347
A00616	A03129	223	A00616	A03165	173
A00616	A03184	174	A00616	A03186	475
A00616	A03219	87	A00616	A03238	430
A00616	A03239	185	A00616	A03259	207
A00616	A03271	395	A00616	A03304	208
A00616	A03310	321	A00616	A03314	490
A00616	A03315	159	A00616	A03436	163
A00616	A03470	288	A00616	A03540	218
A00616	A03584	99	A00616	A03610	347
A00616	A03633	188	A00616	A03639	945
A00616	A03771	296	A00616	A03788	212
A00616	A03800	339	A00616	A03817	439
A00616	A03818	435	A00616	A03915	238
A00616	A03919	429	A00616	A03942	163
A00616	A03961	358	A00616	A03975	183
A00616	A04040	170	A00616	A04086	265
A00616	A04092	474	A00616	A04101	174
A00616	A04163	456	A00616	A04202	218
A00616	A04248	218	A00616	A04254	278
A00616	A04255	390	A00616	A04256	226
A00616	A04276	157	A00616	A04423	173
A00616	A04437	156	A00616	A04457	278
A00616	A04472	238	A00616	A04482	351
A00616	A04484	227	A00616	A04504	243
A00616	A04516	183	A00616	A04536	299
A00616	A04578	210	A00616	A04679	483
A00616	A04682	413	A00616	A04734	201
A00616	A04746	154	A00616	A04748	238
A00616	A04813	157	A00616	A04974	232
A00616	A04976	340	A00616	A05078	210
A00616	A05111	232	A00616	A05117	201
A00616	A05143	183	A00616	A05208	297
A00616	A05288	193	A00616	A05311	449
A00616	A05459	351	A00616	A05533	384
A00616	A05653	207	A00616	A05679	152

A00616	A05869	174	A00616	A05923	160
A00616	A05934	251	A00616	A05944	367
A00616	A05985	490	A00616	A06054	335
A00616	A06055	329	A00616	A06263	246
A00616	A06404	193	A00616	A06588	252
A00616	A06747	224	A00616	A06818	174
A00616	A06831	223	A00616	A07140	188
A00616	A07143	491	A00616	A07173	168
A00616	A07209	241	A00616	A07422	899
A00616	A08264	278	A00616	A08714	235
A00616	A08728	267	A00616	A08751	207
A00616	A08817	373	A00616	A09074	223
A00616	A09217	278	A00616	A09218	465
A00616	A09480	333	A00616	A09513	258
A00616	A09559	238	A00616	A09797	160
A00616	A10018	364	A00616	A10044	267
A00616	A10045	267	A00616	A10046	390
A00616	A10357	195	A00616	A10650	278
A00616	A10665	207	A00616	A10806	267
A00616	A11014	170	A00616	A11064	204
A00616	A11123	198	A00616	A11485	210
A00616	A11556	188	A00616	A11950	255
A00616	A12412	210	A00616	A13391	179
A00616	A13872	243	A00616	A13978	903
A00616	A14003	162	A00616	A14125	469
A00616	A14194	408	A00616	A14198	390
A00616	A14210	172	A00616	A14370	152
A00616	A14443	924	A00616	A14554	180
A00616	A14604	170	A00616	A15230	323
A00616	A15290	316	A00616	A15466	238
A00616	A15498	443	A00616	A15674	159
A00616	A15966	238	A00616	A16636	469
A00617	A00718	451	A00617	A00778	215
A00617	A00779	151	A00617	A00907	452
A00617	A01026	424	A00617	A01035	221
A00617	A01091	291	A00617	A01211	202
A00617	A01313	480	A00617	A01377	466
A00617	A03055	363	A00617	A08113	458
A00617	A08263	456	A00617	A08402	338
A00617	A14092	324	A00617	A14272	333
A00617	A14840	379	A00617	A15339	204
A00617	A15498	228	A00617	A15788	196
A00617	A15877	452	A00618	A14502	63
A00619	A00697	179	A00619	A00734	252
A00619	A00918	457	A00619	A00969	210
A00619	A01020	172	A00619	A01061	358

A00619	A01066	307	A00619	A01082	204
A00619	A01136	454	A00619	A01151	201
A00619	A01234	190	A00619	A01264	286
A00619	A01338	329	A00619	A01384	210
A00619	A01412	165	A00619	A01471	201
A00619	A02050	317	A00619	A02499	312
A00619	A02978	279	A00619	A03009	486
A00619	A03289	201	A00619	A03494	200
A00619	A03567	205	A00619	A03776	210
A00619	A03859	168	A00619	A04398	170
A00619	A04861	168	A00619	A05301	247
A00619	A06861	418	A00619	A07226	313
A00619	A07378	252	A00619	A08558	274
A00619	A09529	247	A00619	A11248	386
A00619	A14566	477	A00619	A14594	399
A00619	A16664	168	A00620	A01042	474
A00620	A01461	286	A00620	A01673	173
A00620	A03326	160	A00620	A03876	276
A00620	A06290	414	A00620	A06593	301
A00620	A07989	184	A00620	A09375	273
A00620	A09583	306	A00620	A10578	201
A00620	A11285	165	A00620	A11742	296
A00620	A14247	465	A00620	A14661	263
A00620	A14710	383	A00620	A14799	65
A00620	A15770	515	A00621	A00624	343
A00621	A00652	101	A00621	A01373	235
A00621	A01507	63	A00621	A01553	480
A00621	A01562	414	A00621	A01610	210
A00621	A01628	475	A00621	A01631	491
A00621	A01639	486	A00621	A01640	430
A00621	A01660	265	A00621	A01692	179
A00621	A01708	396	A00621	A01709	74
A00621	A01908	432	A00621	A03419	180
A00621	A03444	218	A00621	A03959	190
A00621	A04699	168	A00621	A05009	218
A00621	A05953	265	A00621	A06000	455
A00621	A06254	188	A00621	A06289	93
A00621	A06423	281	A00621	A06466	63
A00621	A06701	477	A00621	A06821	212
A00621	A07422	479	A00621	A08571	383
A00621	A09162	281	A00621	A09643	144
A00621	A09767	213	A00621	A10014	303
A00621	A10392	357	A00621	A10514	149
A00621	A10534	434	A00621	A10623	259
A00621	A10844	224	A00621	A11027	247
A00621	A11258	160	A00621	A11379	438

A00621	A12383	371	A00621	A14008	367
A00621	A14009	217	A00621	A14073	288
A00621	A14747	371	A00621	A16782	327
A00622	A00626	428	A00622	A00655	156
A00622	A00665	347	A00622	A00733	171
A00622	A00744	156	A00622	A00805	508
A00622	A00831	457	A00622	A00856	221
A00622	A00858	713	A00622	A00860	689
A00622	A00866	156	A00622	A00881	483
A00622	A00978	521	A00622	A01007	564
A00622	A01019	511	A00622	A01116	490
A00622	A01195	484	A00622	A01200	704
A00622	A01212	418	A00622	A01318	157
A00622	A01323	293	A00622	A01383	205
A00622	A01398	197	A00622	A01401	683
A00622	A01500	293	A00622	A01720	380
A00622	A01901	170	A00622	A03057	247
A00622	A03207	308	A00622	A03329	165
A00622	A03525	154	A00622	A03526	232
A00622	A04648	515	A00622	A04687	156
A00622	A05027	171	A00622	A05386	208
A00622	A05419	167	A00622	A06803	517
A00622	A07290	267	A00622	A07348	173
A00622	A07670	376	A00622	A07850	156
A00622	A07887	445	A00622	A08383	266
A00622	A08398	170	A00622	A08818	156
A00622	A09195	436	A00622	A09502	156
A00622	A09597	317	A00622	A09676	308
A00622	A09712	462	A00622	A10642	213
A00622	A11344	156	A00622	A11674	156
A00622	A14153	249	A00622	A16228	490
A00623	A00667	430	A00623	A00709	307
A00623	A00740	247	A00623	A00791	388
A00623	A00824	247	A00623	A00854	254
A00623	A00861	516	A00623	A00996	247
A00623	A01012	63	A00623	A01040	363
A00623	A01069	190	A00623	A01102	247
A00623	A01119	247	A00623	A01159	177
A00623	A01194	247	A00623	A01218	340
A00623	A01228	435	A00623	A01313	219
A00623	A01411	247	A00623	A01420	708
A00623	A01455	462	A00623	A01492	462
A00623	A01539	457	A00623	A01581	247
A00623	A01598	707	A00623	A01649	495
A00623	A01826	247	A00623	A02705	162
A00623	A03453	247	A00623	A03863	263

A00623	A07347	195	A00623	A07725	165
A00623	A09717	247	A00623	A10624	294
A00623	A12862	322	A00623	A14074	247
A00623	A17035	200	A00624	A00747	98
A00624	A00933	271	A00624	A01104	259
A00624	A01262	184	A00624	A01571	185
A00624	A01631	368	A00624	A01646	210
A00624	A01730	299	A00624	A01877	299
A00624	A01908	162	A00624	A02026	205
A00624	A02500	378	A00624	A02786	481
A00624	A02787	193	A00624	A02816	201
A00624	A02860	279	A00624	A02874	322
A00624	A02877	212	A00624	A02878	157
A00624	A02895	263	A00624	A02983	156
A00624	A03100	366	A00624	A03157	157
A00624	A03171	163	A00624	A03419	388
A00624	A03740	254	A00624	A03767	302
A00624	A03936	305	A00624	A05473	276
A00624	A05910	369	A00624	A06000	344
A00624	A06466	180	A00624	A06622	262
A00624	A06654	296	A00624	A07163	121
A00624	A07798	215	A00624	A08527	180
A00624	A08738	151	A00624	A08885	170
A00624	A09464	401	A00624	A11286	151
A00624	A11296	205	A00624	A11694	241
A00624	A11908	348	A00624	A14073	191
A00624	A14738	188	A00625	A00638	488
A00625	A00643	346	A00625	A00681	270
A00625	A00732	436	A00625	A00786	491
A00625	A00812	347	A00625	A00814	366
A00625	A00827	234	A00625	A00862	489
A00625	A00864	491	A00625	A00877	491
A00625	A00941	489	A00625	A00951	491
A00625	A00971	491	A00625	A00979	324
A00625	A01002	182	A00625	A01011	484
A00625	A01017	486	A00625	A01036	491
A00625	A01088	213	A00625	A01101	491
A00625	A01161	447	A00625	A01169	381
A00625	A01216	645	A00625	A01217	435
A00625	A01275	475	A00625	A01276	267
A00625	A01282	423	A00625	A01290	491
A00625	A01345	491	A00625	A01348	171
A00625	A01358	491	A00625	A01366	752
A00625	A01373	327	A00625	A01451	491
A00625	A01453	490	A00625	A01454	538
A00625	A01459	628	A00625	A01484	417

A00625	A01507	491	A00625	A01514	626
A00625	A01524	899	A00625	A01543	299
A00625	A01549	449	A00625	A01557	460
A00625	A01568	491	A00625	A01578	597
A00625	A01580	491	A00625	A01584	334
A00625	A01595	195	A00625	A01600	408
A00625	A01630	480	A00625	A01632	486
A00625	A01647	490	A00625	A01657	363
A00625	A01668	319	A00625	A01681	486
A00625	A01687	491	A00625	A01694	480
A00625	A01706	450	A00625	A01768	205
A00625	A01811	167	A00625	A01988	590
A00625	A02116	490	A00625	A02120	252
A00625	A02188	377	A00625	A02228	283
A00625	A02303	157	A00625	A02535	383
A00625	A02698	491	A00625	A02786	252
A00625	A03197	311	A00625	A03407	486
A00625	A03434	204	A00625	A03518	491
A00625	A03597	177	A00625	A03742	296
A00625	A03769	272	A00625	A03817	196
A00625	A03837	240	A00625	A03880	477
A00625	A03893	383	A00625	A04115	283
A00625	A04139	337	A00625	A04256	234
A00625	A04292	361	A00625	A04410	477
A00625	A04449	152	A00625	A04520	489
A00625	A04710	385	A00625	A04715	488
A00625	A04790	443	A00625	A04944	279
A00625	A05016	326	A00625	A05150	177
A00625	A05155	240	A00625	A05243	177
A00625	A05301	485	A00625	A05431	383
A00625	A05638	334	A00625	A05894	490
A00625	A05987	213	A00625	A06100	241
A00625	A06176	491	A00625	A06177	229
A00625	A06213	174	A00625	A06277	423
A00625	A06661	205	A00625	A06752	488
A00625	A06819	491	A00625	A06889	180
A00625	A06941	396	A00625	A07054	491
A00625	A07073	408	A00625	A07083	486
A00625	A07180	213	A00625	A07227	491
A00625	A07242	545	A00625	A07253	458
A00625	A07282	465	A00625	A07364	245
A00625	A07404	472	A00625	A07444	272
A00625	A07483	491	A00625	A07495	491
A00625	A07665	491	A00625	A07696	491
A00625	A07709	491	A00625	A07732	424
A00625	A07802	363	A00625	A07803	383

A00625	A07816	491	A00625	A07893	491
A00625	A07897	170	A00625	A07934	480
A00625	A07954	491	A00625	A07965	389
A00625	A08056	491	A00625	A08094	286
A00625	A08293	204	A00625	A08402	240
A00625	A08484	215	A00625	A08531	491
A00625	A08536	483	A00625	A08551	475
A00625	A08569	488	A00625	A08594	350
A00625	A08917	486	A00625	A08934	491
A00625	A08961	177	A00625	A08962	414
A00625	A09112	283	A00625	A09256	400
A00625	A09283	160	A00625	A09298	391
A00625	A09311	274	A00625	A09527	174
A00625	A09567	238	A00625	A09594	174
A00625	A09624	463	A00625	A09666	491
A00625	A09727	204	A00625	A10008	441
A00625	A10100	174	A00625	A10250	281
A00625	A10345	241	A00625	A10369	171
A00625	A10375	266	A00625	A10657	383
A00625	A11022	307	A00625	A11023	491
A00625	A11149	491	A00625	A11150	477
A00625	A11152	174	A00625	A11180	425
A00625	A11185	238	A00625	A11281	491
A00625	A11284	488	A00625	A11301	265
A00625	A11346	491	A00625	A11547	443
A00625	A11675	269	A00625	A11684	170
A00625	A11690	160	A00625	A11716	446
A00625	A11863	238	A00625	A11902	488
A00625	A11915	228	A00625	A12047	550
A00625	A12051	419	A00625	A12083	254
A00625	A12091	322	A00625	A12161	322
A00625	A12313	263	A00625	A12337	417
A00625	A12365	283	A00625	A12397	272
A00625	A12511	174	A00625	A12722	436
A00625	A12843	174	A00625	A12897	383
A00625	A12911	174	A00625	A13146	490
A00625	A13239	152	A00625	A13369	594
A00625	A13489	174	A00625	A13784	671
A00625	A13831	374	A00625	A13866	384
A00625	A13894	483	A00625	A13895	311
A00625	A13927	364	A00625	A13936	414
A00625	A13949	162	A00625	A14163	477
A00625	A14208	162	A00625	A14209	491
A00625	A14222	484	A00625	A14266	491
A00625	A14281	752	A00625	A14464	317
A00625	A14471	386	A00625	A14474	645

A00625	A14484	491	A00625	A14621	490
A00625	A14633	491	A00625	A14661	491
A00625	A14665	195	A00625	A14711	399
A00625	A14744	174	A00625	A14783	353
A00625	A14854	174	A00625	A15004	232
A00625	A15035	486	A00625	A15122	174
A00625	A15232	174	A00625	A15277	152
A00625	A15367	406	A00625	A15380	491
A00625	A15386	383	A00625	A15626	456
A00625	A15693	174	A00625	A15895	174
A00625	A15930	458	A00625	A16048	491
A00625	A16216	259	A00625	A16423	368
A00625	A16680	439	A00625	A16727	117
A00625	A16728	238	A00625	A17211	216
A00626	A00655	480	A00626	A00665	152
A00626	A00733	255	A00626	A00744	196
A00626	A00805	188	A00626	A00833	491
A00626	A00858	513	A00626	A00860	428
A00626	A00866	156	A00626	A00881	381
A00626	A00932	436	A00626	A00978	451
A00626	A01007	156	A00626	A01019	405
A00626	A01083	182	A00626	A01195	385
A00626	A01200	467	A00626	A01300	207
A00626	A01398	389	A00626	A01401	490
A00626	A01500	156	A00626	A01572	218
A00626	A02178	254	A00626	A02671	396
A00626	A03207	252	A00626	A03526	165
A00626	A03740	313	A00626	A03752	621
A00626	A03959	167	A00626	A04648	421
A00626	A04687	156	A00626	A05154	157
A00626	A06466	613	A00626	A06803	427
A00626	A07290	202	A00626	A07311	620
A00626	A07670	200	A00626	A07798	763
A00626	A07850	156	A00626	A07887	296
A00626	A08280	226	A00626	A08383	156
A00626	A08818	156	A00626	A09471	249
A00626	A09502	156	A00626	A09712	414
A00626	A10875	193	A00626	A11344	156
A00626	A11674	156	A00626	A11731	207
A00626	A12042	475	A00626	A12328	483
A00626	A12503	490	A00626	A12990	162
A00626	A13320	369	A00626	A13335	290
A00626	A13340	486	A00626	A14073	402
A00626	A14216	234	A00626	A14220	257
A00626	A14738	180	A00626	A14845	156
A00626	A14848	226	A00626	A15244	486

A00626	A15546	472	A00626	A15734	310
A00626	A15741	154	A00626	A16228	497
A00626	A16291	293	A00627	A01069	157
A00627	A01114	251	A00627	A01189	165
A00627	A01218	411	A00627	A01411	245
A00627	A01824	332	A00627	A02188	263
A00627	A04174	308	A00627	A05596	374
A00628	A00654	491	A00628	A00662	269
A00628	A00663	406	A00628	A00666	469
A00628	A00681	491	A00628	A00738	457
A00628	A00770	486	A00628	A00771	491
A00628	A00773	486	A00628	A00825	402
A00628	A00962	491	A00628	A00995	486
A00628	A01004	163	A00628	A01005	432
A00628	A01008	481	A00628	A01018	374
A00628	A01052	199	A00628	A01063	210
A00628	A01165	490	A00628	A01174	263
A00628	A01186	191	A00628	A01223	278
A00628	A01233	491	A00628	A01256	491
A00628	A01272	429	A00628	A01297	188
A00628	A01347	480	A00628	A01409	281
A00628	A01481	491	A00628	A01508	457
A00628	A01552	168	A00628	A01558	199
A00628	A01635	488	A00628	A01713	180
A00628	A01741	228	A00628	A01748	327
A00628	A02047	185	A00628	A02330	213
A00628	A02545	167	A00628	A02584	174
A00628	A02655	152	A00628	A02718	199
A00628	A02847	424	A00628	A02859	451
A00628	A02992	477	A00628	A03003	378
A00628	A03016	339	A00628	A03167	291
A00628	A03401	199	A00628	A03487	152
A00628	A03899	154	A00628	A04211	491
A00628	A04266	232	A00628	A04778	268
A00628	A04839	193	A00628	A04937	165
A00628	A05007	358	A00628	A05102	205
A00628	A05363	249	A00628	A05393	219
A00628	A05416	396	A00628	A05617	167
A00628	A05715	274	A00628	A05738	174
A00628	A05759	343	A00628	A05783	249
A00628	A05859	208	A00628	A05961	162
A00628	A06039	208	A00628	A06086	491
A00628	A06358	176	A00628	A06405	260
A00628	A06592	491	A00628	A06689	418
A00628	A06700	160	A00628	A06756	486
A00628	A06773	380	A00628	A06979	249

A00628	A07088	179	A00628	A07109	260
A00628	A07122	489	A00628	A07234	379
A00628	A07238	416	A00628	A07267	473
A00628	A07413	486	A00628	A07459	232
A00628	A07477	356	A00628	A07495	199
A00628	A07520	286	A00628	A07525	439
A00628	A07553	465	A00628	A07590	451
A00628	A07627	241	A00628	A07659	486
A00628	A07660	457	A00628	A07674	288
A00628	A07688	489	A00628	A07753	473
A00628	A07770	322	A00628	A07800	491
A00628	A07848	430	A00628	A07933	215
A00628	A07970	241	A00628	A07973	182
A00628	A07976	339	A00628	A08099	491
A00628	A08133	211	A00628	A08155	391
A00628	A08193	485	A00628	A08205	473
A00628	A08318	185	A00628	A08332	293
A00628	A08687	179	A00628	A08751	188
A00628	A09043	188	A00628	A09612	440
A00628	A09879	174	A00628	A09985	185
A00628	A10151	191	A00628	A10370	241
A00628	A10420	245	A00628	A10424	188
A00628	A10443	165	A00628	A10444	340
A00628	A10452	210	A00628	A10455	204
A00628	A10456	474	A00628	A10550	177
A00628	A10656	218	A00628	A10673	246
A00628	A10677	188	A00628	A10681	449
A00628	A10693	238	A00628	A10694	162
A00628	A10702	330	A00628	A10712	358
A00628	A10714	458	A00628	A10904	249
A00628	A11309	491	A00628	A11325	425
A00628	A11381	434	A00628	A13343	322
A00628	A13677	322	A00628	A13694	208
A00628	A13695	191	A00628	A13768	288
A00628	A14209	185	A00628	A14221	468
A00628	A14628	177	A00628	A14856	238
A00628	A14862	188	A00628	A15190	171
A00628	A15497	491	A00628	A15637	390
A00628	A15752	445	A00628	A15874	488
A00628	A15974	247	A00628	A16002	162
A00628	A16027	491	A00628	A16028	273
A00628	A16033	443	A00628	A16262	360
A00628	A16263	171	A00628	A16286	344
A00628	A16368	467	A00628	A16419	288
A00628	A16488	167	A00628	A16529	491
A00628	A16741	345	A00628	A16767	165

A00628	A16959	491	A00628	A17006	157
A00628	A17040	339	A00628	A17078	432
A00629	A00648	489	A00629	A00651	480
A00629	A00653	480	A00629	A00683	407
A00629	A00687	574	A00629	A00689	419
A00629	A00697	462	A00629	A00700	477
A00629	A00701	489	A00629	A00705	168
A00629	A00709	157	A00629	A00721	242
A00629	A00727	346	A00629	A00763	491
A00629	A00772	233	A00629	A00780	270
A00629	A00782	386	A00629	A00787	491
A00629	A00790	176	A00629	A00831	208
A00629	A00839	491	A00629	A00848	473
A00629	A00854	262	A00629	A00865	682
A00629	A00868	356	A00629	A00870	310
A00629	A00878	490	A00629	A00883	491
A00629	A00906	234	A00629	A00916	383
A00629	A00923	491	A00629	A00928	687
A00629	A00952	668	A00629	A00955	160
A00629	A00965	240	A00629	A00970	260
A00629	A00972	528	A00629	A00990	474
A00629	A00997	217	A00629	A01000	491
A00629	A01017	485	A00629	A01022	475
A00629	A01025	491	A00629	A01034	238
A00629	A01035	452	A00629	A01037	235
A00629	A01042	307	A00629	A01047	486
A00629	A01056	236	A00629	A01072	201
A00629	A01085	281	A00629	A01086	344
A00629	A01090	321	A00629	A01091	201
A00629	A01098	574	A00629	A01101	682
A00629	A01107	511	A00629	A01108	213
A00629	A01112	439	A00629	A01115	338
A00629	A01127	491	A00629	A01140	488
A00629	A01150	461	A00629	A01151	294
A00629	A01159	597	A00629	A01166	491
A00629	A01169	293	A00629	A01182	308
A00629	A01184	452	A00629	A01186	433
A00629	A01189	327	A00629	A01196	328
A00629	A01206	491	A00629	A01217	334
A00629	A01221	687	A00629	A01234	636
A00629	A01238	63	A00629	A01240	274
A00629	A01243	575	A00629	A01249	491
A00629	A01291	648	A00629	A01306	581
A00629	A01335	467	A00629	A01338	418
A00629	A01352	486	A00629	A01365	535
A00629	A01379	191	A00629	A01384	317

A00629	A01392	477	A00629	A01393	259
A00629	A01406	285	A00629	A01412	526
A00629	A01418	554	A00629	A01425	305
A00629	A01436	204	A00629	A01437	179
A00629	A01451	490	A00629	A01457	307
A00629	A01458	489	A00629	A01464	486
A00629	A01468	491	A00629	A01470	491
A00629	A01492	440	A00629	A01495	389
A00629	A01496	469	A00629	A01539	182
A00629	A01569	480	A00629	A01579	491
A00629	A01597	202	A00629	A01598	232
A00629	A01601	511	A00629	A01617	256
A00629	A01618	362	A00629	A01649	491
A00629	A01718	472	A00629	A01917	174
A00629	A02003	313	A00629	A02195	174
A00629	A02212	483	A00629	A02221	223
A00629	A02227	232	A00629	A02452	382
A00629	A02978	363	A00629	A03069	414
A00629	A03304	187	A00629	A03451	438
A00629	A03494	183	A00629	A03556	221
A00629	A03567	582	A00629	A03617	217
A00629	A03705	479	A00629	A03740	430
A00629	A03754	414	A00629	A03859	435
A00629	A04292	473	A00629	A04846	262
A00629	A04922	232	A00629	A05025	205
A00629	A05199	351	A00629	A05409	246
A00629	A05449	345	A00629	A05501	491
A00629	A05605	217	A00629	A06011	154
A00629	A06064	191	A00629	A06122	472
A00629	A06275	326	A00629	A06279	466
A00629	A06369	591	A00629	A06435	157
A00629	A06436	168	A00629	A06664	272
A00629	A06832	677	A00629	A06843	174
A00629	A06861	582	A00629	A06909	217
A00629	A06965	491	A00629	A06973	468
A00629	A07203	443	A00629	A07323	324
A00629	A07349	326	A00629	A07398	254
A00629	A07430	308	A00629	A07449	490
A00629	A07451	485	A00629	A07581	251
A00629	A07706	274	A00629	A08011	406
A00629	A08055	188	A00629	A08131	328
A00629	A08183	217	A00629	A08343	187
A00629	A08357	477	A00629	A08369	193
A00629	A08398	210	A00629	A08452	358
A00629	A08578	268	A00629	A08773	417
A00629	A08972	353	A00629	A08982	337

A00629	A09289	234	A00629	A09314	396
A00629	A09322	578	A00629	A09673	217
A00629	A10503	234	A00629	A10518	163
A00629	A10616	435	A00629	A11277	489
A00629	A11338	223	A00629	A11427	241
A00629	A11438	485	A00629	A11480	228
A00629	A11704	151	A00629	A12053	260
A00629	A12363	174	A00629	A12480	308
A00629	A12605	467	A00629	A12824	219
A00629	A12889	486	A00629	A12918	191
A00629	A13249	425	A00629	A13272	234
A00629	A13441	360	A00629	A13989	491
A00629	A13992	490	A00629	A14088	217
A00629	A14115	154	A00629	A14140	308
A00629	A14169	326	A00629	A14196	205
A00629	A14272	215	A00629	A14482	454
A00629	A14566	364	A00629	A14594	394
A00629	A14596	614	A00629	A14597	447
A00629	A14701	361	A00629	A14875	208
A00629	A14938	308	A00629	A15235	168
A00629	A15324	201	A00629	A15377	217
A00629	A15465	208	A00629	A15498	337
A00629	A15500	322	A00629	A15543	375
A00629	A15660	491	A00629	A15684	486
A00629	A15724	217	A00629	A15743	238
A00629	A15772	202	A00629	A15776	263
A00629	A15971	217	A00629	A15988	441
A00629	A16118	491	A00629	A16463	190
A00629	A16664	217	A00629	A16686	168
A00630	A00631	470	A00630	A00643	322
A00630	A00679	183	A00630	A00820	335
A00630	A00826	485	A00630	A00839	283
A00630	A00875	452	A00630	A00887	436
A00630	A00980	452	A00630	A00981	347
A00630	A01052	469	A00630	A01125	399
A00630	A01291	164	A00630	A01299	156
A00630	A01309	485	A00630	A01461	154
A00630	A01487	213	A00630	A01507	193
A00630	A01512	407	A00630	A01551	374
A00630	A01553	228	A00630	A01561	528
A00630	A01574	174	A00630	A01585	152
A00630	A01606	682	A00630	A01683	346
A00630	A01688	441	A00630	A01698	165
A00630	A01724	424	A00630	A01730	163
A00630	A01740	205	A00630	A01920	594
A00630	A01995	343	A00630	A02110	452

A00630	A02703	208	A00630	A02715	229
A00630	A02766	306	A00630	A02822	174
A00630	A02873	191	A00630	A03041	167
A00630	A03321	229	A00630	A03336	235
A00630	A03451	245	A00630	A03591	274
A00630	A03611	362	A00630	A03892	219
A00630	A04020	441	A00630	A04316	266
A00630	A04449	447	A00630	A05176	324
A00630	A05414	469	A00630	A05416	297
A00630	A05418	288	A00630	A05438	180
A00630	A06143	281	A00630	A06277	440
A00630	A06466	227	A00630	A07220	257
A00630	A07311	359	A00630	A07848	188
A00630	A08815	270	A00630	A08878	480
A00630	A09162	443	A00630	A09303	152
A00630	A09562	168	A00630	A10247	321
A00630	A10430	534	A00630	A10655	452
A00630	A11061	218	A00630	A11158	245
A00630	A11293	274	A00630	A11487	408
A00630	A11793	337	A00630	A11937	480
A00630	A11983	603	A00630	A12062	168
A00630	A13213	218	A00630	A13392	205
A00630	A13902	452	A00630	A14002	306
A00630	A14206	170	A00630	A14211	230
A00630	A15336	391	A00630	A15506	254
A00630	A15665	201	A00630	A15682	329
A00630	A16478	168	A00630	A16847	534
A00630	A16926	311	A00631	A01366	327
A00631	A01512	199	A00631	A01606	486
A00631	A01724	191	A00631	A02846	228
A00631	A03336	432	A00631	A07746	152
A00631	A11293	208	A00632	A00765	172
A00632	A01041	288	A00632	A01309	353
A00632	A01338	162	A00632	A01568	283
A00632	A01647	182	A00632	A01683	172
A00632	A02278	151	A00632	A03271	196
A00632	A03496	291	A00632	A03880	200
A00632	A05301	165	A00632	A06039	177
A00632	A07483	286	A00632	A08255	278
A00632	A08362	154	A00632	A08501	267
A00632	A08502	281	A00632	A08503	208
A00632	A08543	187	A00632	A08550	207
A00632	A08552	337	A00632	A08556	208
A00632	A08561	229	A00632	A08814	254
A00632	A08845	238	A00632	A08996	306
A00632	A09333	258	A00632	A09622	329

A00632	A11301	190	A00632	A11376	281
A00632	A11430	218	A00632	A11472	260
A00632	A11497	223	A00632	A11597	266
A00632	A11675	286	A00632	A11820	351
A00632	A11917	154	A00632	A11940	355
A00632	A12254	267	A00632	A12433	221
A00632	A12501	305	A00632	A12507	180
A00632	A12606	338	A00632	A12725	219
A00632	A12825	324	A00632	A13309	407
A00632	A13313	174	A00632	A13399	268
A00632	A13706	277	A00632	A13708	356
A00632	A13737	241	A00632	A13781	313
A00632	A13784	205	A00632	A13804	366
A00632	A13912	380	A00632	A14084	205
A00632	A14214	200	A00632	A14222	157
A00632	A14288	344	A00632	A14706	224
A00632	A14708	265	A00632	A14903	351
A00632	A15105	345	A00632	A15189	274
A00632	A15245	416	A00632	A15262	259
A00632	A15357	185	A00632	A15402	160
A00632	A15680	218	A00632	A15683	219
A00632	A16157	210	A00632	A16552	327
A00632	A16597	395	A00632	A16611	362
A00632	A16675	374	A00632	A16680	272
A00632	A16703	323	A00632	A16709	340
A00632	A16764	288	A00632	A16776	356
A00632	A16829	348	A00632	A16866	296
A00632	A16905	313	A00632	A16913	371
A00632	A17018	352	A00632	A17030	356
A00632	A17190	347	A00633	A00654	462
A00633	A00660	235	A00633	A00662	411
A00633	A00870	217	A00633	A01005	386
A00633	A01008	154	A00633	A01018	255
A00633	A01207	210	A00633	A01229	271
A00633	A01297	232	A00633	A01409	324
A00633	A01490	210	A00633	A01635	372
A00633	A01739	154	A00633	A01832	259
A00633	A01972	469	A00633	A02023	277
A00633	A03050	439	A00633	A03174	240
A00633	A03442	307	A00633	A03729	168
A00633	A04791	165	A00633	A05050	240
A00633	A05337	172	A00633	A05358	193
A00633	A05390	388	A00633	A05416	457
A00633	A05451	249	A00633	A05532	401
A00633	A05548	216	A00633	A05549	319
A00633	A05610	277	A00633	A06439	321

A00633	A06641	180	A00633	A06855	234
A00633	A06994	230	A00633	A07376	213
A00633	A07477	247	A00633	A07590	339
A00633	A07674	286	A00633	A07736	200
A00633	A10686	218	A00633	A11519	319
A00633	A11767	205	A00633	A12121	290
A00633	A14081	208	A00633	A15811	212
A00633	A17078	205	A00634	A01299	246
A00634	A01907	195	A00634	A03397	313
A00634	A03591	347	A00634	A03636	291
A00634	A03964	297	A00634	A05288	477
A00634	A09494	345	A00634	A09555	377
A00634	A10506	168	A00634	A11190	238
A00634	A12047	391	A00634	A15854	195
A00635	A00736	347	A00635	A01143	168
A00635	A01668	302	A00635	A01743	162
A00635	A02005	350	A00635	A02010	480
A00635	A02983	302	A00635	A03701	334
A00635	A11951	486	A00635	A12959	469
A00635	A12960	366	A00635	A12976	486
A00635	A12983	466	A00635	A13826	254
A00635	A14178	201	A00635	A14295	334
A00635	A14490	226	A00635	A15318	340
A00635	A15346	180	A00635	A15366	344
A00635	A15376	177	A00635	A15851	252
A00635	A15852	185	A00636	A00722	670
A00636	A00921	439	A00636	A00981	168
A00636	A01036	326	A00636	A01125	305
A00636	A01162	432	A00636	A01248	254
A00636	A01417	479	A00636	A01473	393
A00636	A01578	210	A00636	A01712	479
A00636	A01723	168	A00636	A01956	486
A00636	A01961	348	A00636	A01963	361
A00636	A01967	391	A00636	A02000	273
A00636	A02007	474	A00636	A02010	491
A00636	A02157	348	A00636	A02466	213
A00636	A02993	393	A00636	A03025	481
A00636	A03080	399	A00636	A03134	416
A00636	A03135	319	A00636	A03137	319
A00636	A03141	180	A00636	A03344	256
A00636	A03345	258	A00636	A03384	249
A00636	A03686	393	A00636	A03692	313
A00636	A03694	215	A00636	A03701	377
A00636	A03703	490	A00636	A03712	276
A00636	A03721	486	A00636	A03722	490
A00636	A03728	393	A00636	A03894	469

A00636	A04076	165	A00636	A04333	167
A00636	A04340	414	A00636	A04477	283
A00636	A04932	159	A00636	A05243	224
A00636	A05313	188	A00636	A05361	290
A00636	A05887	364	A00636	A06771	171
A00636	A06862	330	A00636	A06964	420
A00636	A07181	344	A00636	A07242	446
A00636	A07413	154	A00636	A07954	163
A00636	A08111	380	A00636	A08161	176
A00636	A08268	361	A00636	A09235	162
A00636	A09253	182	A00636	A09421	260
A00636	A09804	188	A00636	A09824	378
A00636	A09890	276	A00636	A09900	335
A00636	A10579	259	A00636	A11601	269
A00636	A12016	330	A00636	A12648	348
A00636	A12835	393	A00636	A13410	296
A00636	A14470	484	A00636	A14789	247
A00636	A15277	313	A00636	A15627	319
A00636	A17076	317	A00637	A00652	334
A00637	A00853	617	A00637	A00886	162
A00637	A01093	308	A00637	A01149	456
A00637	A01595	383	A00637	A01705	195
A00637	A02311	152	A00637	A03237	347
A00637	A03336	266	A00637	A03435	243
A00637	A04268	232	A00637	A05004	188
A00637	A05958	475	A00637	A07054	379
A00637	A07232	334	A00637	A07378	160
A00637	A07732	334	A00637	A08863	276
A00637	A09078	334	A00637	A10001	201
A00637	A11376	276	A00637	A11843	276
A00637	A11851	276	A00637	A12251	195
A00637	A12252	174	A00637	A13356	276
A00637	A14009	334	A00637	A14288	334
A00637	A15480	276	A00637	A16417	168
A00637	A16635	334	A00638	A00652	240
A00638	A00791	398	A00638	A00805	171
A00638	A00856	455	A00638	A00862	489
A00638	A00883	281	A00638	A00911	557
A00638	A00985	469	A00638	A01086	352
A00638	A01099	488	A00638	A01124	371
A00638	A01149	491	A00638	A01151	241
A00638	A01156	305	A00638	A01209	491
A00638	A01214	436	A00638	A01244	624
A00638	A01296	675	A00638	A01311	270
A00638	A01317	358	A00638	A01318	272
A00638	A01319	471	A00638	A01320	653

A00638	A01322	491	A00638	A01323	477
A00638	A01325	486	A00638	A01327	708
A00638	A01328	305	A00638	A01331	182
A00638	A01333	412	A00638	A01358	705
A00638	A01415	624	A00638	A01459	490
A00638	A01507	258	A00638	A01514	491
A00638	A01524	313	A00638	A01580	170
A00638	A01596	385	A00638	A01647	491
A00638	A01680	182	A00638	A01828	707
A00638	A01928	267	A00638	A01988	473
A00638	A02212	345	A00638	A02228	348
A00638	A02319	193	A00638	A02473	252
A00638	A02504	221	A00638	A02523	302
A00638	A02539	374	A00638	A02541	416
A00638	A03216	183	A00638	A03217	113
A00638	A03237	422	A00638	A03264	204
A00638	A03304	174	A00638	A03327	210
A00638	A03328	623	A00638	A03929	307
A00638	A04140	491	A00638	A04359	193
A00638	A04372	168	A00638	A04498	205
A00638	A04668	385	A00638	A04772	174
A00638	A04776	152	A00638	A04922	177
A00638	A05556	188	A00638	A05690	491
A00638	A05707	276	A00638	A06002	276
A00638	A06318	396	A00638	A06493	317
A00638	A06517	193	A00638	A06558	205
A00638	A06572	159	A00638	A06588	236
A00638	A06602	307	A00638	A06819	172
A00638	A06941	213	A00638	A06982	479
A00638	A07205	463	A00638	A07386	241
A00638	A07433	406	A00638	A07564	221
A00638	A07572	162	A00638	A07687	307
A00638	A07695	238	A00638	A07833	266
A00638	A08195	276	A00638	A08196	363
A00638	A08212	337	A00638	A08252	259
A00638	A08352	422	A00638	A08516	252
A00638	A08552	307	A00638	A08884	489
A00638	A09304	602	A00638	A09316	218
A00638	A09340	196	A00638	A09342	483
A00638	A09680	193	A00638	A10250	290
A00638	A10257	276	A00638	A10373	484
A00638	A10456	327	A00638	A10761	183
A00638	A10839	234	A00638	A11283	307
A00638	A11332	307	A00638	A11467	168
A00638	A11676	218	A00638	A11828	579
A00638	A11834	344	A00638	A11840	307

A00638	A11854	491	A00638	A11858	198
A00638	A12013	501	A00638	A12047	623
A00638	A12824	168	A00638	A13949	491
A00638	A14209	395	A00638	A14221	162
A00638	A14230	281	A00638	A14272	363
A00638	A14449	369	A00638	A14477	491
A00638	A14674	491	A00638	A14685	174
A00638	A14703	463	A00638	A14909	193
A00638	A14976	317	A00638	A15233	196
A00638	A15380	472	A00638	A15556	462
A00638	A15557	483	A00638	A15644	443
A00638	A16440	345	A00638	A16738	447
A00638	A16763	552	A00638	A17172	348
A00639	A02671	162	A00639	A11277	301
A00640	A00647	420	A00640	A00653	152
A00640	A00689	236	A00640	A00711	224
A00640	A00726	152	A00640	A00740	256
A00640	A00810	610	A00640	A00833	152
A00640	A00843	396	A00640	A00848	491
A00640	A00970	491	A00640	A00974	152
A00640	A00980	63	A00640	A00984	152
A00640	A00987	152	A00640	A01001	152
A00640	A01069	490	A00640	A01102	152
A00640	A01115	400	A00640	A01189	180
A00640	A01211	202	A00640	A01215	465
A00640	A01311	730	A00640	A01353	224
A00640	A01354	489	A00640	A01379	152
A00640	A01387	152	A00640	A01439	152
A00640	A01506	439	A00640	A01539	419
A00640	A01541	168	A00640	A01548	272
A00640	A01569	488	A00640	A01577	629
A00640	A01588	899	A00640	A01627	152
A00640	A01684	391	A00640	A01747	199
A00640	A01770	489	A00640	A01917	411
A00640	A02051	228	A00640	A02601	152
A00640	A02719	364	A00640	A02877	63
A00640	A02892	291	A00640	A03282	157
A00640	A03325	405	A00640	A03446	335
A00640	A03448	313	A00640	A03476	458
A00640	A03669	251	A00640	A03697	502
A00640	A03738	475	A00640	A04013	408
A00640	A04495	152	A00640	A04537	236
A00640	A04697	195	A00640	A04957	198
A00640	A05417	411	A00640	A05508	202
A00640	A05712	267	A00640	A05760	899
A00640	A06178	313	A00640	A06287	360

A00640	A06365	208	A00640	A06543	462
A00640	A06677	423	A00640	A07247	449
A00640	A07744	545	A00640	A08061	317
A00640	A08098	389	A00640	A08178	343
A00640	A08763	317	A00640	A08947	152
A00640	A09004	152	A00640	A09033	202
A00640	A09395	184	A00640	A09423	154
A00640	A09603	271	A00640	A09819	353
A00640	A10238	399	A00640	A10532	491
A00640	A10574	177	A00640	A10842	152
A00640	A11403	385	A00640	A12839	152
A00640	A13465	291	A00640	A13609	276
A00640	A14525	514	A00640	A14728	177
A00640	A15375	317	A00640	A15546	502
A00640	A16026	160	A00641	A00696	366
A00641	A00697	63	A00641	A00782	491
A00641	A00788	485	A00641	A00789	488
A00641	A00799	151	A00641	A00839	479
A00641	A00939	491	A00641	A01154	369
A00641	A01164	393	A00641	A01247	193
A00641	A01254	151	A00641	A01286	443
A00641	A01307	491	A00641	A01428	84
A00641	A01602	363	A00641	A01604	217
A00641	A02425	296	A00641	A02604	213
A00641	A02611	76	A00641	A02651	130
A00641	A02659	346	A00641	A02681	305
A00641	A02803	469	A00641	A02847	249
A00641	A02851	377	A00641	A02853	364
A00641	A02887	490	A00641	A02917	82
A00641	A02920	162	A00641	A02925	353
A00641	A02931	489	A00641	A02935	124
A00641	A02944	435	A00641	A02945	427
A00641	A02950	486	A00641	A02953	110
A00641	A02961	408	A00641	A02962	129
A00641	A02969	112	A00641	A02972	107
A00641	A02975	82	A00641	A03019	68
A00641	A03195	99	A00641	A03271	491
A00641	A03275	358	A00641	A03282	112
A00641	A03524	79	A00641	A03565	480
A00641	A03625	65	A00641	A03626	413
A00641	A03686	63	A00641	A03830	393
A00641	A03834	358	A00641	A03835	324
A00641	A03838	171	A00641	A03984	441
A00641	A03985	243	A00641	A04005	204
A00641	A04050	491	A00641	A04085	191
A00641	A04251	274	A00641	A04494	174

A00641	A04657	369	A00641	A04685	302
A00641	A04862	249	A00641	A05037	333
A00641	A05211	449	A00641	A05212	322
A00641	A05213	249	A00641	A05220	435
A00641	A05349	367	A00641	A05508	154
A00641	A05633	366	A00641	A05902	185
A00641	A06111	322	A00641	A06289	63
A00641	A06819	338	A00641	A07141	185
A00641	A07148	151	A00641	A07157	262
A00641	A07161	377	A00641	A07166	151
A00641	A07168	226	A00641	A07173	243
A00641	A07188	213	A00641	A07218	477
A00641	A07602	256	A00641	A08277	490
A00641	A08335	179	A00641	A08672	249
A00641	A08761	243	A00641	A08762	385
A00641	A08840	63	A00641	A09395	414
A00641	A10060	274	A00641	A10214	235
A00641	A10609	299	A00641	A10739	157
A00641	A11085	274	A00641	A11129	302
A00641	A11604	151	A00641	A11688	396
A00641	A11906	179	A00641	A12364	293
A00641	A12792	383	A00641	A12824	899
A00641	A13199	200	A00641	A13302	196
A00641	A13400	241	A00641	A13824	152
A00641	A13829	445	A00641	A13846	201
A00641	A14208	491	A00641	A14430	202
A00641	A14431	68	A00641	A14647	246
A00641	A14830	457	A00641	A15314	488
A00641	A15347	187	A00641	A15361	352
A00641	A15386	104	A00641	A15425	190
A00641	A15500	454	A00641	A15505	160
A00641	A15578	63	A00641	A15613	256
A00641	A15639	172	A00641	A15771	420
A00641	A15780	440	A00641	A15781	293
A00641	A15782	260	A00641	A15976	174
A00641	A16225	151	A00641	A16377	188
A00641	A16583	388	A00641	A16594	328
A00641	A16971	260	A00642	A00643	491
A00642	A00864	154	A00642	A00875	283
A00642	A00931	185	A00642	A01546	363
A00642	A03049	486	A00642	A03213	201
A00642	A06269	425	A00642	A07158	263
A00642	A07568	457	A00642	A09556	273
A00642	A09624	344	A00642	A10440	351
A00642	A11827	420	A00642	A12246	246
A00642	A13726	180	A00642	A13785	246

A00642	A14289	152	A00642	A14720	480
A00642	A15498	172	A00642	A16764	230
A00643	A00669	420	A00643	A00671	491
A00643	A00679	252	A00643	A00747	467
A00643	A00799	265	A00643	A00815	366
A00643	A00826	374	A00643	A00839	171
A00643	A00852	438	A00643	A00864	475
A00643	A00875	404	A00643	A00883	469
A00643	A00947	258	A00643	A00980	322
A00643	A00981	480	A00643	A01022	299
A00643	A01075	248	A00643	A01091	443
A00643	A01112	433	A00643	A01125	449
A00643	A01148	400	A00643	A01161	281
A00643	A01211	430	A00643	A01213	427
A00643	A01216	358	A00643	A01235	462
A00643	A01299	460	A00643	A01305	362
A00643	A01309	358	A00643	A01310	484
A00643	A01378	375	A00643	A01405	332
A00643	A01409	322	A00643	A01452	236
A00643	A01474	241	A00643	A01507	465
A00643	A01514	165	A00643	A01546	489
A00643	A01561	159	A00643	A01602	484
A00643	A01606	224	A00643	A01660	449
A00643	A01698	367	A00643	A01723	491
A00643	A01747	436	A00643	A01763	480
A00643	A01781	477	A00643	A01790	491
A00643	A01887	168	A00643	A01931	475
A00643	A01995	440	A00643	A02018	226
A00643	A02020	398	A00643	A02110	322
A00643	A02143	247	A00643	A02502	384
A00643	A02698	168	A00643	A02709	188
A00643	A02839	452	A00643	A02902	460
A00643	A03014	267	A00643	A03049	424
A00643	A03050	269	A00643	A03104	473
A00643	A03188	477	A00643	A03213	665
A00643	A03304	486	A00643	A03434	424
A00643	A03435	244	A00643	A03436	174
A00643	A03451	227	A00643	A03591	165
A00643	A03723	228	A00643	A03739	469
A00643	A03893	484	A00643	A03961	283
A00643	A04033	491	A00643	A04139	234
A00643	A04449	571	A00643	A04638	174
A00643	A04759	483	A00643	A04873	368
A00643	A05158	436	A00643	A05416	182
A00643	A05418	490	A00643	A05810	439
A00643	A05920	156	A00643	A06190	196

A00643	A06196	174	A00643	A06277	486
A00643	A06588	488	A00643	A06705	307
A00643	A07209	491	A00643	A07229	265
A00643	A07242	445	A00643	A07339	463
A00643	A07568	490	A00643	A07693	232
A00643	A07726	352	A00643	A07803	491
A00643	A08205	358	A00643	A08875	463
A00643	A08878	469	A00643	A09072	326
A00643	A09078	187	A00643	A09449	259
A00643	A09624	466	A00643	A09791	461
A00643	A10114	188	A00643	A10203	224
A00643	A10264	174	A00643	A10430	420
A00643	A10439	356	A00643	A10440	447
A00643	A10449	281	A00643	A10761	163
A00643	A11020	174	A00643	A11104	162
A00643	A11300	210	A00643	A11335	168
A00643	A11433	240	A00643	A11487	283
A00643	A11560	159	A00643	A11827	224
A00643	A11983	491	A00643	A12246	343
A00643	A12365	484	A00643	A12513	334
A00643	A12897	486	A00643	A13299	174
A00643	A13388	151	A00643	A13784	426
A00643	A14055	361	A00643	A14062	420
A00643	A14063	420	A00643	A14196	396
A00643	A14198	260	A00643	A14279	201
A00643	A14292	269	A00643	A14410	486
A00643	A14511	193	A00643	A14540	195
A00643	A14635	491	A00643	A14636	491
A00643	A14643	491	A00643	A14650	265
A00643	A14653	227	A00643	A14714	339
A00643	A14720	451	A00643	A14943	277
A00643	A15282	486	A00643	A15292	386
A00643	A15296	480	A00643	A15318	199
A00643	A15349	310	A00643	A15606	260
A00643	A15665	228	A00643	A15682	205
A00643	A15879	163	A00643	A16764	424
A00643	A16847	430	A00644	A05449	63
A00645	A00888	362	A00645	A01021	162
A00645	A01725	293	A00645	A02289	390
A00645	A06607	171	A00645	A07800	241
A00645	A08104	63	A00645	A14502	371
A00646	A00691	156	A00646	A00716	156
A00646	A00867	156	A00646	A00879	431
A00646	A00905	522	A00646	A00916	191
A00646	A01029	521	A00646	A01058	196
A00646	A01136	156	A00646	A01151	519

A00646	A01231	219	A00646	A01266	538
A00646	A01268	541	A00646	A01295	156
A00646	A01334	156	A00646	A01391	156
A00646	A01434	156	A00646	A01612	477
A00646	A02487	246	A00646	A03494	261
A00646	A03569	296	A00646	A07898	156
A00646	A08137	458	A00646	A08201	156
A00646	A08327	156	A00646	A08431	156
A00646	A08542	156	A00646	A08571	377
A00646	A11496	156	A00646	A11497	156
A00646	A12082	408	A00646	A12189	372
A00646	A12202	329	A00646	A12757	515
A00646	A13510	313	A00646	A13928	156
A00646	A14178	451	A00646	A14255	229
A00646	A14257	243	A00646	A14284	302
A00646	A14728	466	A00646	A14882	308
A00646	A15629	408	A00646	A16699	156
A00647	A00655	198	A00647	A00662	180
A00647	A00663	152	A00647	A00736	319
A00647	A00764	195	A00647	A00822	438
A00647	A00853	170	A00647	A01354	439
A00647	A01385	281	A00647	A01564	188
A00647	A01923	470	A00647	A02059	195
A00647	A02180	447	A00647	A02187	196
A00647	A02215	324	A00647	A02545	486
A00647	A03565	394	A00647	A03725	491
A00647	A03839	196	A00647	A04661	154
A00647	A04916	302	A00647	A05112	213
A00647	A05135	313	A00647	A05153	491
A00647	A05299	184	A00647	A05315	227
A00647	A05356	318	A00647	A05926	477
A00647	A06162	238	A00647	A06180	353
A00647	A06288	240	A00647	A07016	221
A00647	A07123	157	A00647	A07452	241
A00647	A07468	488	A00647	A07469	439
A00647	A07617	347	A00647	A07728	218
A00647	A07958	424	A00647	A08063	302
A00647	A08494	221	A00647	A08785	335
A00647	A10321	262	A00647	A10967	491
A00647	A10986	174	A00647	A11324	330
A00647	A11471	157	A00647	A12588	353
A00647	A12793	485	A00647	A13879	238
A00647	A15503	190	A00647	A15850	271
A00647	A17108	383	A00648	A00651	813
A00648	A00653	491	A00648	A00658	227
A00648	A00670	159	A00648	A00687	340

A00648	A00697	313	A00648	A00700	491
A00648	A00701	489	A00648	A00705	443
A00648	A00713	491	A00648	A00763	485
A00648	A00782	475	A00648	A00787	740
A00648	A00790	294	A00648	A00809	414
A00648	A00812	384	A00648	A00826	243
A00648	A00831	491	A00648	A00839	603
A00648	A00843	272	A00648	A00848	355
A00648	A00854	240	A00648	A00861	218
A00648	A00870	491	A00648	A00871	451
A00648	A00878	838	A00648	A00902	477
A00648	A00906	353	A00648	A00916	491
A00648	A00922	326	A00648	A00923	490
A00648	A00935	379	A00648	A00949	643
A00648	A00953	296	A00648	A00955	201
A00648	A00965	727	A00648	A00966	628
A00648	A00970	281	A00648	A00985	434
A00648	A00986	414	A00648	A00988	177
A00648	A00994	477	A00648	A01000	474
A00648	A01012	657	A00648	A01016	317
A00648	A01020	329	A00648	A01025	491
A00648	A01031	193	A00648	A01034	640
A00648	A01037	491	A00648	A01042	170
A00648	A01047	391	A00648	A01054	195
A00648	A01056	491	A00648	A01067	480
A00648	A01072	491	A00648	A01085	491
A00648	A01090	491	A00648	A01095	489
A00648	A01098	174	A00648	A01101	350
A00648	A01112	491	A00648	A01115	467
A00648	A01120	486	A00648	A01127	491
A00648	A01140	491	A00648	A01144	534
A00648	A01150	474	A00648	A01153	274
A00648	A01154	599	A00648	A01161	413
A00648	A01166	491	A00648	A01179	324
A00648	A01184	396	A00648	A01186	180
A00648	A01189	396	A00648	A01192	177
A00648	A01196	409	A00648	A01201	229
A00648	A01206	491	A00648	A01211	419
A00648	A01217	258	A00648	A01220	483
A00648	A01221	463	A00648	A01234	491
A00648	A01238	724	A00648	A01243	450
A00648	A01249	480	A00648	A01263	408
A00648	A01268	470	A00648	A01291	697
A00648	A01292	249	A00648	A01294	385
A00648	A01304	195	A00648	A01306	580
A00648	A01335	491	A00648	A01365	491

A00648	A01392	491	A00648	A01406	586
A00648	A01416	736	A00648	A01425	629
A00648	A01431	388	A00648	A01433	491
A00648	A01436	628	A00648	A01437	452
A00648	A01439	152	A00648	A01457	491
A00648	A01458	157	A00648	A01464	159
A00648	A01468	629	A00648	A01470	491
A00648	A01488	291	A00648	A01492	561
A00648	A01496	491	A00648	A01538	486
A00648	A01539	213	A00648	A01540	241
A00648	A01569	358	A00648	A01579	491
A00648	A01586	626	A00648	A01597	486
A00648	A01598	489	A00648	A01601	538
A00648	A01612	414	A00648	A01614	490
A00648	A01628	899	A00648	A01649	491
A00648	A01652	190	A00648	A01707	409
A00648	A01718	296	A00648	A01722	198
A00648	A01741	213	A00648	A01822	412
A00648	A02051	261	A00648	A02227	288
A00648	A03069	396	A00648	A03290	177
A00648	A03296	151	A00648	A03461	483
A00648	A03490	633	A00648	A03567	488
A00648	A03652	271	A00648	A03770	460
A00648	A03963	350	A00648	A03981	604
A00648	A04846	188	A00648	A05267	262
A00648	A05443	301	A00648	A05501	489
A00648	A05654	205	A00648	A06102	205
A00648	A06275	445	A00648	A06279	353
A00648	A06340	468	A00648	A06351	643
A00648	A06466	313	A00648	A06517	177
A00648	A06543	272	A00648	A06690	160
A00648	A06812	374	A00648	A06814	424
A00648	A06861	291	A00648	A06965	483
A00648	A07157	417	A00648	A07312	152
A00648	A07323	472	A00648	A07360	396
A00648	A07394	71	A00648	A07398	208
A00648	A07412	424	A00648	A07447	227
A00648	A07449	350	A00648	A07451	491
A00648	A07536	210	A00648	A07584	210
A00648	A07597	462	A00648	A07726	484
A00648	A07924	346	A00648	A08011	738
A00648	A08052	171	A00648	A08055	486
A00648	A08137	371	A00648	A08301	334
A00648	A08357	491	A00648	A08359	483
A00648	A08377	627	A00648	A08398	408
A00648	A08452	433	A00648	A08533	196

A00648	A08543	167	A00648	A08550	620
A00648	A08773	738	A00648	A08966	190
A00648	A08973	808	A00648	A08983	168
A00648	A09148	177	A00648	A09293	308
A00648	A09332	632	A00648	A09352	399
A00648	A09530	262	A00648	A09620	219
A00648	A09697	441	A00648	A09735	462
A00648	A09811	227	A00648	A09978	480
A00648	A10532	433	A00648	A10616	455
A00648	A10910	96	A00648	A10924	277
A00648	A11254	402	A00648	A11263	332
A00648	A11334	425	A00648	A11390	268
A00648	A11392	316	A00648	A11453	491
A00648	A11462	452	A00648	A11518	177
A00648	A11535	154	A00648	A11815	643
A00648	A11945	187	A00648	A11992	369
A00648	A12052	177	A00648	A12168	291
A00648	A12252	157	A00648	A12433	419
A00648	A12881	489	A00648	A12923	383
A00648	A13024	168	A00648	A13126	466
A00648	A13171	205	A00648	A13249	419
A00648	A13340	467	A00648	A13549	177
A00648	A13989	649	A00648	A13992	328
A00648	A14006	486	A00648	A14172	574
A00648	A14188	299	A00648	A14216	812
A00648	A14447	323	A00648	A14449	489
A00648	A14479	405	A00648	A14482	643
A00648	A14508	160	A00648	A14530	193
A00648	A14551	160	A00648	A14729	177
A00648	A14816	205	A00648	A14875	152
A00648	A14889	177	A00648	A15215	210
A00648	A15500	347	A00648	A15660	481
A00648	A15684	570	A00648	A15756	168
A00648	A15917	168	A00648	A15931	319
A00648	A15932	439	A00648	A15988	491
A00648	A16029	262	A00648	A16053	205
A00648	A16157	296	A00648	A16306	308
A00648	A16351	168	A00648	A16462	324
A00648	A16467	154	A00648	A16793	210
A00648	A16858	168	A00648	A16886	162
A00648	A17095	152	A00649	A00651	419
A00649	A00653	592	A00649	A00662	635
A00649	A00687	321	A00649	A00694	188
A00649	A00697	162	A00649	A00731	516
A00649	A00741	174	A00649	A00780	323
A00649	A00783	589	A00649	A00785	592

A00649	A00787	151	A00649	A00790	588
A00649	A00849	301	A00649	A00863	177
A00649	A00953	592	A00649	A00974	590
A00649	A00985	465	A00649	A00987	271
A00649	A00994	380	A00649	A01027	590
A00649	A01081	198	A00649	A01086	591
A00649	A01100	319	A00649	A01108	592
A00649	A01150	467	A00649	A01161	581
A00649	A01204	591	A00649	A01234	241
A00649	A01246	340	A00649	A01271	352
A00649	A01287	384	A00649	A01346	592
A00649	A01399	590	A00649	A01403	165
A00649	A01433	191	A00649	A01443	427
A00649	A01446	473	A00649	A01448	462
A00649	A01540	419	A00649	A01560	252
A00649	A01622	443	A00649	A01668	355
A00649	A01711	436	A00649	A01741	454
A00649	A01743	445	A00649	A02814	313
A00649	A02994	247	A00649	A03259	223
A00649	A03959	298	A00649	A03981	313
A00649	A03990	633	A00649	A04771	413
A00649	A04895	485	A00649	A05054	271
A00649	A05323	196	A00649	A05449	380
A00649	A05839	488	A00649	A05840	344
A00649	A05982	260	A00649	A06258	647
A00649	A06825	171	A00649	A06959	489
A00649	A07157	230	A00649	A07164	278
A00649	A07237	489	A00649	A07311	281
A00649	A07339	485	A00649	A07350	319
A00649	A07362	316	A00649	A07412	154
A00649	A07442	294	A00649	A07551	257
A00649	A07569	430	A00649	A07597	352
A00649	A07599	414	A00649	A07623	290
A00649	A07773	193	A00649	A07792	490
A00649	A08653	177	A00649	A08760	171
A00649	A08773	180	A00649	A09616	345
A00649	A09696	356	A00649	A09720	246
A00649	A09978	168	A00649	A10199	447
A00649	A10535	465	A00649	A11015	257
A00649	A11427	210	A00649	A11453	174
A00649	A11628	180	A00649	A11677	247
A00649	A11992	218	A00649	A12600	344
A00649	A13678	414	A00649	A14216	307
A00649	A14699	345	A00649	A14778	247
A00649	A15233	174	A00649	A15293	249
A00649	A15498	468	A00649	A15624	462

A00649	A15651	165	A00649	A15684	396
A00649	A16088	177	A00649	A16095	358
A00649	A16587	247	A00650	A00926	208
A00650	A01488	163	A00650	A02284	218
A00650	A02675	168	A00650	A03570	205
A00650	A03948	152	A00650	A04773	229
A00650	A05209	479	A00650	A05577	260
A00650	A05796	255	A00650	A06279	193
A00650	A07143	245	A00650	A07961	344
A00650	A10234	377	A00650	A11435	483
A00650	A12111	191	A00650	A14374	167
A00650	A14596	491	A00650	A14659	424
A00650	A15507	201	A00650	A15612	208
A00650	A15722	279	A00650	A16230	269
A00650	A16627	235	A00651	A00653	491
A00651	A00687	208	A00651	A00694	316
A00651	A00697	428	A00651	A00700	491
A00651	A00701	491	A00651	A00702	104
A00651	A00713	519	A00651	A00749	170
A00651	A00756	477	A00651	A00763	491
A00651	A00767	170	A00651	A00769	279
A00651	A00782	488	A00651	A00785	285
A00651	A00787	585	A00651	A00806	174
A00651	A00831	210	A00651	A00839	570
A00651	A00849	490	A00651	A00861	550
A00651	A00870	406	A00651	A00872	500
A00651	A00878	629	A00651	A00902	414
A00651	A00916	242	A00651	A00918	170
A00651	A00919	428	A00651	A00922	490
A00651	A00923	461	A00651	A00934	170
A00651	A00945	98	A00651	A00965	317
A00651	A00966	688	A00651	A00970	389
A00651	A00974	173	A00651	A00980	230
A00651	A00983	630	A00651	A00986	329
A00651	A00987	477	A00651	A00990	491
A00651	A00992	170	A00651	A00994	491
A00651	A01012	766	A00651	A01017	407
A00651	A01025	475	A00651	A01028	170
A00651	A01038	491	A00651	A01042	475
A00651	A01047	408	A00651	A01053	457
A00651	A01054	439	A00651	A01056	484
A00651	A01072	486	A00651	A01085	477
A00651	A01086	243	A00651	A01090	688
A00651	A01092	65	A00651	A01098	420
A00651	A01101	386	A00651	A01108	474
A00651	A01112	826	A00651	A01115	488

A00651	A01120	170	A00651	A01127	491
A00651	A01129	549	A00651	A01139	436
A00651	A01140	491	A00651	A01144	607
A00651	A01150	491	A00651	A01160	548
A00651	A01161	477	A00651	A01166	490
A00651	A01168	549	A00651	A01192	296
A00651	A01196	479	A00651	A01206	480
A00651	A01211	380	A00651	A01234	489
A00651	A01238	376	A00651	A01240	404
A00651	A01247	160	A00651	A01249	481
A00651	A01251	115	A00651	A01263	484
A00651	A01275	486	A00651	A01287	480
A00651	A01288	167	A00651	A01291	566
A00651	A01294	267	A00651	A01306	491
A00651	A01335	491	A00651	A01351	170
A00651	A01356	170	A00651	A01359	428
A00651	A01365	467	A00651	A01380	391
A00651	A01392	213	A00651	A01393	232
A00651	A01414	230	A00651	A01416	317
A00651	A01425	712	A00651	A01431	462
A00651	A01436	579	A00651	A01437	335
A00651	A01446	165	A00651	A01448	310
A00651	A01457	490	A00651	A01464	489
A00651	A01468	629	A00651	A01470	486
A00651	A01492	321	A00651	A01496	688
A00651	A01527	899	A00651	A01538	384
A00651	A01539	432	A00651	A01540	491
A00651	A01546	146	A00651	A01569	488
A00651	A01579	491	A00651	A01586	628
A00651	A01597	400	A00651	A01598	170
A00651	A01601	673	A00651	A01605	73
A00651	A01614	471	A00651	A01618	378
A00651	A01649	488	A00651	A01707	174
A00651	A01711	479	A00651	A01787	452
A00651	A01822	558	A00651	A01989	188
A00651	A01995	218	A00651	A02064	491
A00651	A02151	63	A00651	A02164	490
A00651	A02165	490	A00651	A02179	205
A00651	A02236	160	A00651	A02248	82
A00651	A02249	63	A00651	A02265	255
A00651	A02350	474	A00651	A02414	481
A00651	A02494	420	A00651	A02527	460
A00651	A02590	491	A00651	A02605	63
A00651	A02814	208	A00651	A03048	374
A00651	A03069	63	A00651	A03083	167
A00651	A03093	176	A00651	A03112	162

A00651	A03437	377	A00651	A03438	550
A00651	A03461	405	A00651	A03490	338
A00651	A03502	82	A00651	A03567	486
A00651	A03571	182	A00651	A03601	219
A00651	A03602	154	A00651	A03609	486
A00651	A03644	490	A00651	A03645	430
A00651	A03652	528	A00651	A03671	445
A00651	A03676	465	A00651	A03677	313
A00651	A03740	382	A00651	A03752	540
A00651	A03809	484	A00651	A03858	366
A00651	A03888	446	A00651	A03906	156
A00651	A03981	758	A00651	A04197	218
A00651	A04230	380	A00651	A04309	156
A00651	A04666	428	A00651	A04689	236
A00651	A04697	217	A00651	A04764	159
A00651	A04846	369	A00651	A05086	299
A00651	A05153	208	A00651	A05191	417
A00651	A05255	252	A00651	A05411	455
A00651	A05418	224	A00651	A05443	256
A00651	A05449	491	A00651	A05477	286
A00651	A05501	491	A00651	A05557	450
A00651	A05566	486	A00651	A05654	344
A00651	A06084	480	A00651	A06286	366
A00651	A06351	491	A00651	A06356	533
A00651	A06466	490	A00651	A06495	350
A00651	A06525	180	A00651	A06564	377
A00651	A06682	159	A00651	A06807	475
A00651	A06814	636	A00651	A06815	435
A00651	A06825	491	A00651	A06901	291
A00651	A06955	408	A00651	A06965	484
A00651	A06973	469	A00651	A07047	159
A00651	A07105	219	A00651	A07116	416
A00651	A07138	132	A00651	A07201	490
A00651	A07243	491	A00651	A07244	170
A00651	A07251	85	A00651	A07258	177
A00651	A07307	490	A00651	A07311	576
A00651	A07323	469	A00651	A07360	230
A00651	A07398	408	A00651	A07399	208
A00651	A07412	174	A00651	A07447	180
A00651	A07449	427	A00651	A07451	488
A00651	A07506	491	A00651	A07524	364
A00651	A07581	288	A00651	A07726	480
A00651	A07773	245	A00651	A07874	180
A00651	A07921	218	A00651	A07924	170
A00651	A08011	684	A00651	A08052	165
A00651	A08072	218	A00651	A08131	385

A00651	A08211	170	A00651	A08301	246
A00651	A08321	452	A00651	A08357	371
A00651	A08358	260	A00651	A08359	491
A00651	A08377	628	A00651	A08408	388
A00651	A08457	259	A00651	A08476	230
A00651	A08543	430	A00651	A08546	221
A00651	A08550	425	A00651	A08559	457
A00651	A08653	488	A00651	A08664	451
A00651	A08682	491	A00651	A08752	199
A00651	A08768	126	A00651	A08773	558
A00651	A08914	313	A00651	A08970	180
A00651	A08973	596	A00651	A09044	470
A00651	A09618	218	A00651	A09656	485
A00651	A09657	205	A00651	A09697	170
A00651	A09852	466	A00651	A10001	441
A00651	A10034	466	A00651	A10417	489
A00651	A10532	722	A00651	A10564	443
A00651	A10569	260	A00651	A10814	159
A00651	A10901	384	A00651	A10928	159
A00651	A10983	63	A00651	A11263	170
A00651	A11264	433	A00651	A11331	491
A00651	A11334	327	A00651	A11344	345
A00651	A11355	291	A00651	A11359	180
A00651	A11392	152	A00651	A11420	358
A00651	A11438	267	A00651	A11440	174
A00651	A11618	218	A00651	A11619	218
A00651	A11697	156	A00651	A11926	407
A00651	A11945	185	A00651	A11981	491
A00651	A11983	195	A00651	A12259	236
A00651	A12270	416	A00651	A12329	303
A00651	A12404	228	A00651	A12589	159
A00651	A12687	488	A00651	A13052	548
A00651	A13063	589	A00651	A13126	135
A00651	A13340	491	A00651	A13344	159
A00651	A13425	213	A00651	A13581	488
A00651	A13626	491	A00651	A13712	218
A00651	A13752	436	A00651	A13989	643
A00651	A13992	396	A00651	A14073	597
A00651	A14090	91	A00651	A14172	372
A00651	A14176	238	A00651	A14188	227
A00651	A14216	855	A00651	A14272	366
A00651	A14479	215	A00651	A14482	185
A00651	A14508	154	A00651	A14606	366
A00651	A14674	428	A00651	A14738	247
A00651	A14754	180	A00651	A14816	327
A00651	A14817	173	A00651	A14937	257

A00651	A14959	218	A00651	A15063	313
A00651	A15075	218	A00651	A15076	218
A00651	A15225	162	A00651	A15235	249
A00651	A15244	356	A00651	A15268	157
A00651	A15323	191	A00651	A15381	188
A00651	A15500	490	A00651	A15614	151
A00651	A15624	491	A00651	A15649	440
A00651	A15651	190	A00651	A15659	390
A00651	A15660	491	A00651	A15661	263
A00651	A15684	621	A00651	A15707	236
A00651	A15732	346	A00651	A15743	190
A00651	A15773	232	A00651	A15917	219
A00651	A15918	249	A00651	A15931	483
A00651	A15933	218	A00651	A15988	355
A00651	A16088	491	A00651	A16095	490
A00651	A16118	417	A00651	A16149	394
A00651	A16272	377	A00651	A16274	210
A00651	A16575	335	A00651	A16633	156
A00651	A16686	318	A00651	A16738	428
A00651	A16778	193	A00651	A16790	188
A00651	A17035	462	A00651	A17134	319
A00651	A17193	269	A00652	A00847	201
A00652	A00852	337	A00652	A00853	339
A00652	A00868	174	A00652	A00876	191
A00652	A00882	286	A00652	A01045	475
A00652	A01149	638	A00652	A01178	188
A00652	A01209	157	A00652	A01244	247
A00652	A01451	232	A00652	A01478	395
A00652	A01522	396	A00652	A01528	260
A00652	A01550	396	A00652	A01562	221
A00652	A01566	435	A00652	A01575	314
A00652	A01578	327	A00652	A01595	190
A00652	A01660	491	A00652	A01671	157
A00652	A01692	491	A00652	A01708	63
A00652	A02325	470	A00652	A02544	396
A00652	A02703	213	A00652	A02786	465
A00652	A02911	183	A00652	A02912	249
A00652	A03104	213	A00652	A03237	477
A00652	A03488	375	A00652	A03517	212
A00652	A03974	457	A00652	A04275	339
A00652	A04342	151	A00652	A04449	156
A00652	A04715	485	A00652	A04897	156
A00652	A04985	396	A00652	A05453	243
A00652	A05556	260	A00652	A05601	380
A00652	A05986	191	A00652	A06100	386
A00652	A06466	243	A00652	A06530	324

A00652	A07232	447	A00652	A07422	324
A00652	A07709	176	A00652	A07732	334
A00652	A07848	274	A00652	A08097	489
A00652	A08352	172	A00652	A08461	433
A00652	A08532	363	A00652	A08859	255
A00652	A08899	463	A00652	A09078	334
A00652	A09402	241	A00652	A09434	460
A00652	A09736	200	A00652	A09842	162
A00652	A09857	243	A00652	A09995	377
A00652	A10603	198	A00652	A11187	229
A00652	A11687	160	A00652	A11901	274
A00652	A12199	241	A00652	A12282	363
A00652	A13949	341	A00652	A13977	380
A00652	A14009	334	A00652	A14016	288
A00652	A14193	574	A00652	A14253	416
A00652	A14288	334	A00652	A14395	596
A00652	A14570	398	A00652	A14648	321
A00652	A14661	440	A00652	A15297	362
A00652	A15322	373	A00652	A15366	372
A00652	A15483	219	A00652	A15557	372
A00652	A15632	241	A00652	A15680	486
A00652	A16022	420	A00652	A16635	637
A00652	A16979	152	A00653	A00673	188
A00653	A00677	154	A00653	A00687	559
A00653	A00691	391	A00653	A00693	238
A00653	A00697	547	A00653	A00698	460
A00653	A00700	417	A00653	A00705	188
A00653	A00707	294	A00653	A00709	188
A00653	A00711	659	A00653	A00718	477
A00653	A00724	615	A00653	A00726	313
A00653	A00731	590	A00653	A00736	291
A00653	A00741	491	A00653	A00745	477
A00653	A00750	484	A00653	A00758	484
A00653	A00763	363	A00653	A00769	665
A00653	A00780	484	A00653	A00782	457
A00653	A00783	592	A00653	A00785	592
A00653	A00787	491	A00653	A00790	592
A00653	A00795	247	A00653	A00796	380
A00653	A00802	177	A00653	A00805	418
A00653	A00809	486	A00653	A00810	155
A00653	A00811	435	A00653	A00821	391
A00653	A00824	622	A00653	A00833	436
A00653	A00839	560	A00653	A00841	615
A00653	A00849	591	A00653	A00856	316
A00653	A00865	486	A00653	A00868	563
A00653	A00870	385	A00653	A00871	491

A00653	A00876	258	A00653	A00878	491
A00653	A00892	281	A00653	A00909	615
A00653	A00910	479	A00653	A00911	469
A00653	A00922	665	A00653	A00926	362
A00653	A00934	213	A00653	A00935	486
A00653	A00953	592	A00653	A00965	462
A00653	A00970	423	A00653	A00972	211
A00653	A00973	267	A00653	A00974	620
A00653	A00983	659	A00653	A00984	613
A00653	A00985	483	A00653	A00987	702
A00653	A00990	417	A00653	A00994	490
A00653	A01001	611	A00653	A01006	575
A00653	A01012	485	A00653	A01013	296
A00653	A01017	556	A00653	A01020	256
A00653	A01023	361	A00653	A01025	489
A00653	A01027	665	A00653	A01037	227
A00653	A01042	491	A00653	A01047	278
A00653	A01054	491	A00653	A01055	180
A00653	A01056	183	A00653	A01069	202
A00653	A01072	157	A00653	A01075	438
A00653	A01081	440	A00653	A01082	357
A00653	A01083	219	A00653	A01086	716
A00653	A01087	249	A00653	A01088	215
A00653	A01090	393	A00653	A01094	174
A00653	A01095	172	A00653	A01100	484
A00653	A01101	558	A00653	A01102	564
A00653	A01108	592	A00653	A01115	461
A00653	A01116	307	A00653	A01121	251
A00653	A01127	467	A00653	A01135	241
A00653	A01140	270	A00653	A01150	797
A00653	A01154	462	A00653	A01155	213
A00653	A01156	310	A00653	A01161	707
A00653	A01169	469	A00653	A01192	663
A00653	A01204	591	A00653	A01206	329
A00653	A01210	489	A00653	A01215	324
A00653	A01217	421	A00653	A01218	475
A00653	A01219	165	A00653	A01220	446
A00653	A01221	491	A00653	A01232	665
A00653	A01234	562	A00653	A01242	490
A00653	A01244	411	A00653	A01246	484
A00653	A01249	353	A00653	A01260	398
A00653	A01265	491	A00653	A01287	658
A00653	A01289	201	A00653	A01291	532
A00653	A01293	320	A00653	A01300	193
A00653	A01306	180	A00653	A01311	539
A00653	A01326	316	A00653	A01331	395

A00653	A01335	485	A00653	A01336	297
A00653	A01346	592	A00653	A01352	187
A00653	A01360	441	A00653	A01362	374
A00653	A01365	490	A00653	A01369	465
A00653	A01379	618	A00653	A01382	486
A00653	A01385	455	A00653	A01387	563
A00653	A01392	361	A00653	A01399	659
A00653	A01403	333	A00653	A01406	518
A00653	A01407	221	A00653	A01408	227
A00653	A01411	485	A00653	A01412	406
A00653	A01413	491	A00653	A01414	188
A00653	A01416	347	A00653	A01418	334
A00653	A01431	654	A00653	A01433	399
A00653	A01435	488	A00653	A01436	411
A00653	A01437	213	A00653	A01439	619
A00653	A01443	577	A00653	A01446	491
A00653	A01447	394	A00653	A01448	491
A00653	A01451	188	A00653	A01457	489
A00653	A01468	475	A00653	A01470	419
A00653	A01496	491	A00653	A01539	491
A00653	A01540	491	A00653	A01548	297
A00653	A01569	491	A00653	A01577	420
A00653	A01579	489	A00653	A01582	378
A00653	A01613	489	A00653	A01627	299
A00653	A01647	159	A00653	A01649	490
A00653	A01711	491	A00653	A01741	420
A00653	A01747	450	A00653	A01822	489
A00653	A01824	276	A00653	A01827	268
A00653	A01837	416	A00653	A01841	455
A00653	A01850	400	A00653	A01894	291
A00653	A01976	188	A00653	A01988	188
A00653	A02040	162	A00653	A02165	278
A00653	A02227	373	A00653	A02350	324
A00653	A02353	491	A00653	A02601	299
A00653	A02607	475	A00653	A02718	417
A00653	A02994	498	A00653	A03070	462
A00653	A03298	467	A00653	A03421	405
A00653	A03453	489	A00653	A03454	385
A00653	A03494	485	A00653	A03516	266
A00653	A03567	341	A00653	A03651	210
A00653	A03676	477	A00653	A03697	152
A00653	A03740	491	A00653	A03752	491
A00653	A03776	472	A00653	A03809	664
A00653	A03901	282	A00653	A03959	590
A00653	A03981	488	A00653	A04495	299
A00653	A04642	380	A00653	A04771	446

A00653	A04782	337	A00653	A04895	491
A00653	A05054	434	A00653	A05086	489
A00653	A05227	664	A00653	A05258	480
A00653	A05323	227	A00653	A05387	208
A00653	A05417	440	A00653	A05449	486
A00653	A05501	460	A00653	A05542	208
A00653	A05562	224	A00653	A05702	517
A00653	A05839	457	A00653	A05840	208
A00653	A05859	389	A00653	A06000	238
A00653	A06049	193	A00653	A06084	188
A00653	A06287	403	A00653	A06356	165
A00653	A06466	491	A00653	A06543	372
A00653	A06637	486	A00653	A06750	443
A00653	A06785	188	A00653	A06794	208
A00653	A06959	483	A00653	A06965	307
A00653	A07046	168	A00653	A07160	489
A00653	A07208	485	A00653	A07237	490
A00653	A07311	204	A00653	A07331	215
A00653	A07350	171	A00653	A07360	423
A00653	A07362	485	A00653	A07394	281
A00653	A07442	306	A00653	A07502	208
A00653	A07523	74	A00653	A07527	215
A00653	A07551	385	A00653	A07569	475
A00653	A07597	486	A00653	A07599	305
A00653	A07686	335	A00653	A07706	156
A00653	A07731	160	A00653	A07744	538
A00653	A07746	330	A00653	A07773	491
A00653	A07792	474	A00653	A07799	157
A00653	A07819	207	A00653	A07881	627
A00653	A07911	291	A00653	A07965	188
A00653	A08011	463	A00653	A08053	201
A00653	A08054	313	A00653	A08055	263
A00653	A08061	152	A00653	A08098	524
A00653	A08178	234	A00653	A08228	658
A00653	A08319	319	A00653	A08357	385
A00653	A08358	173	A00653	A08371	310
A00653	A08398	484	A00653	A08452	491
A00653	A08488	446	A00653	A08521	490
A00653	A08547	241	A00653	A08548	383
A00653	A08571	200	A00653	A08763	152
A00653	A08773	414	A00653	A08835	698
A00653	A08843	71	A00653	A08922	208
A00653	A08947	299	A00653	A08968	281
A00653	A08987	491	A00653	A09004	299
A00653	A09285	199	A00653	A09297	184
A00653	A09298	238	A00653	A09303	258

A00653	A09332	217	A00653	A09476	358
A00653	A09587	411	A00653	A09616	394
A00653	A09655	205	A00653	A09702	375
A00653	A09717	427	A00653	A09720	419
A00653	A09735	474	A00653	A10199	394
A00653	A10487	208	A00653	A10518	344
A00653	A10527	399	A00653	A10532	364
A00653	A10535	477	A00653	A10797	401
A00653	A10798	490	A00653	A10842	152
A00653	A10853	430	A00653	A10935	302
A00653	A11015	102	A00653	A11296	490
A00653	A11302	468	A00653	A11316	252
A00653	A11322	491	A00653	A11337	406
A00653	A11338	491	A00653	A11372	435
A00653	A11374	491	A00653	A11380	185
A00653	A11419	491	A00653	A11427	491
A00653	A11480	539	A00653	A11521	201
A00653	A11677	591	A00653	A11724	395
A00653	A11823	171	A00653	A11860	475
A00653	A11974	413	A00653	A11975	454
A00653	A12056	208	A00653	A12059	281
A00653	A12241	457	A00653	A12400	171
A00653	A12418	208	A00653	A12824	333
A00653	A13069	249	A00653	A13116	157
A00653	A13261	207	A00653	A13328	343
A00653	A13340	491	A00653	A13497	171
A00653	A13525	375	A00653	A13707	224
A00653	A13754	208	A00653	A13769	208
A00653	A13787	207	A00653	A13989	456
A00653	A13992	212	A00653	A14073	491
A00653	A14077	180	A00653	A14080	446
A00653	A14152	369	A00653	A14216	491
A00653	A14272	286	A00653	A14479	429
A00653	A14482	488	A00653	A14516	226
A00653	A14525	152	A00653	A14566	458
A00653	A14569	156	A00653	A14594	238
A00653	A14642	443	A00653	A14655	454
A00653	A14699	665	A00653	A14701	369
A00653	A14738	316	A00653	A14778	662
A00653	A14813	188	A00653	A14819	281
A00653	A14825	430	A00653	A14845	489
A00653	A14888	468	A00653	A15081	208
A00653	A15121	302	A00653	A15133	391
A00653	A15219	227	A00653	A15375	152
A00653	A15379	183	A00653	A15416	424
A00653	A15498	257	A00653	A15499	198

A00653	A15500	398	A00653	A15546	152
A00653	A15624	491	A00653	A15651	367
A00653	A15652	201	A00653	A15653	475
A00653	A15657	246	A00653	A15660	491
A00653	A15661	308	A00653	A15662	478
A00653	A15666	394	A00653	A15684	491
A00653	A15699	310	A00653	A15715	207
A00653	A15746	454	A00653	A15765	184
A00653	A15917	240	A00653	A15931	439
A00653	A15932	457	A00653	A15933	363
A00653	A15937	479	A00653	A15988	286
A00653	A15992	180	A00653	A16088	473
A00653	A16118	238	A00653	A16272	436
A00653	A16371	276	A00653	A16437	329
A00653	A16587	247	A00653	A16627	205
A00653	A16680	188	A00653	A16683	346
A00653	A16793	332	A00653	A16840	171
A00653	A16886	332	A00653	A17128	491
A00654	A00660	224	A00654	A00662	480
A00654	A00663	213	A00654	A00666	491
A00654	A00681	491	A00654	A00695	488
A00654	A00696	398	A00654	A00738	491
A00654	A00739	479	A00654	A00770	491
A00654	A00773	472	A00654	A00825	321
A00654	A00853	174	A00654	A00962	491
A00654	A00995	486	A00654	A01005	467
A00654	A01008	491	A00654	A01018	488
A00654	A01046	344	A00654	A01063	357
A00654	A01174	480	A00654	A01175	486
A00654	A01222	247	A00654	A01223	240
A00654	A01227	238	A00654	A01229	348
A00654	A01230	345	A00654	A01233	491
A00654	A01256	490	A00654	A01272	367
A00654	A01297	488	A00654	A01347	343
A00654	A01397	163	A00654	A01409	485
A00654	A01452	207	A00654	A01478	199
A00654	A01481	472	A00654	A01508	481
A00654	A01511	246	A00654	A01572	199
A00654	A01596	199	A00654	A01635	491
A00654	A01642	160	A00654	A01714	152
A00654	A01730	199	A00654	A01741	388
A00654	A01743	335	A00654	A01857	343
A00654	A01972	419	A00654	A02156	217
A00654	A02254	329	A00654	A02268	157
A00654	A02319	226	A00654	A02353	646
A00654	A02363	465	A00654	A02432	329

A00654	A02521	414	A00654	A02546	174
A00654	A02593	187	A00654	A02695	390
A00654	A02718	560	A00654	A02719	281
A00654	A02859	401	A00654	A02992	477
A00654	A03003	401	A00654	A03004	396
A00654	A03005	174	A00654	A03008	272
A00654	A03016	380	A00654	A03050	274
A00654	A03059	283	A00654	A03167	486
A00654	A03168	405	A00654	A03259	179
A00654	A03429	346	A00654	A03430	217
A00654	A03441	466	A00654	A03477	756
A00654	A03484	366	A00654	A03500	188
A00654	A03508	201	A00654	A03513	168
A00654	A03545	286	A00654	A04211	212
A00654	A04372	201	A00654	A04660	399
A00654	A04674	346	A00654	A04702	238
A00654	A04779	479	A00654	A04784	188
A00654	A04791	377	A00654	A04834	474
A00654	A04850	241	A00654	A04880	165
A00654	A04891	272	A00654	A04927	252
A00654	A04995	483	A00654	A05006	358
A00654	A05007	491	A00654	A05013	208
A00654	A05020	756	A00654	A05041	195
A00654	A05045	277	A00654	A05051	435
A00654	A05098	188	A00654	A05102	452
A00654	A05310	479	A00654	A05311	174
A00654	A05416	480	A00654	A05459	179
A00654	A05472	249	A00654	A05515	249
A00654	A05516	488	A00654	A05532	226
A00654	A05573	468	A00654	A05600	260
A00654	A05659	271	A00654	A05699	322
A00654	A05714	226	A00654	A05759	485
A00654	A05772	380	A00654	A05774	473
A00654	A05783	353	A00654	A06063	402
A00654	A06086	348	A00654	A06088	425
A00654	A06089	346	A00654	A06091	443
A00654	A06098	208	A00654	A06195	199
A00654	A06211	160	A00654	A06252	480
A00654	A06358	491	A00654	A06359	756
A00654	A06402	373	A00654	A06405	435
A00654	A06439	469	A00654	A06473	486
A00654	A06523	174	A00654	A06555	252
A00654	A06558	238	A00654	A06562	208
A00654	A06597	279	A00654	A06630	229
A00654	A06633	224	A00654	A06638	226
A00654	A06689	441	A00654	A06692	299

A00654	A06699	157	A00654	A06700	454
A00654	A06756	486	A00654	A06831	179
A00654	A06847	252	A00654	A06934	274
A00654	A06970	306	A00654	A06979	319
A00654	A07002	333	A00654	A07027	425
A00654	A07080	389	A00654	A07088	329
A00654	A07109	475	A00654	A07122	491
A00654	A07238	346	A00654	A07239	163
A00654	A07256	480	A00654	A07267	414
A00654	A07271	226	A00654	A07288	174
A00654	A07304	412	A00654	A07322	413
A00654	A07336	152	A00654	A07339	384
A00654	A07376	263	A00654	A07381	223
A00654	A07413	491	A00654	A07477	477
A00654	A07503	286	A00654	A07520	369
A00654	A07530	252	A00654	A07553	274
A00654	A07554	160	A00654	A07590	477
A00654	A07627	414	A00654	A07674	449
A00654	A07688	432	A00654	A07736	455
A00654	A07737	457	A00654	A07739	201
A00654	A07757	417	A00654	A07800	488
A00654	A07933	356	A00654	A08099	243
A00654	A08133	299	A00654	A08135	241
A00654	A08280	199	A00654	A08596	213
A00654	A08751	383	A00654	A09195	199
A00654	A09425	272	A00654	A09632	402
A00654	A09711	252	A00654	A09917	195
A00654	A09924	424	A00654	A10020	252
A00654	A10027	302	A00654	A10147	302
A00654	A10456	486	A00654	A10550	283
A00654	A10582	238	A00654	A10656	185
A00654	A10659	393	A00654	A10686	267
A00654	A10702	207	A00654	A10703	218
A00654	A11309	423	A00654	A11346	263
A00654	A11381	172	A00654	A11431	490
A00654	A11432	208	A00654	A11611	363
A00654	A12055	195	A00654	A12990	195
A00654	A13481	302	A00654	A13677	302
A00654	A14196	180	A00654	A14299	199
A00654	A14313	208	A00654	A14443	207
A00654	A14678	184	A00654	A14685	208
A00654	A14827	317	A00654	A14857	329
A00654	A14862	163	A00654	A15135	160
A00654	A15190	278	A00654	A15497	443
A00654	A15637	441	A00654	A15874	334
A00654	A15892	184	A00654	A15905	483

A00654	A16026	461	A00654	A16027	424
A00654	A16028	340	A00654	A16033	489
A00654	A16262	167	A00654	A16335	245
A00654	A16344	337	A00654	A16346	252
A00654	A16507	238	A00654	A16529	462
A00654	A16741	297	A00654	A16959	469
A00654	A17078	481	A00655	A00757	385
A00655	A00805	479	A00655	A00817	463
A00655	A00818	353	A00655	A00819	491
A00655	A00822	795	A00655	A00831	236
A00655	A00833	235	A00655	A00856	491
A00655	A00858	443	A00655	A00860	156
A00655	A00905	480	A00655	A00916	452
A00655	A00918	294	A00655	A00934	483
A00655	A00940	199	A00655	A00978	199
A00655	A01032	491	A00655	A01033	477
A00655	A01075	491	A00655	A01082	271
A00655	A01087	480	A00655	A01088	447
A00655	A01136	466	A00655	A01206	477
A00655	A01234	466	A00655	A01325	374
A00655	A01354	486	A00655	A01378	277
A00655	A01384	232	A00655	A01385	836
A00655	A01401	358	A00655	A01429	281
A00655	A01468	258	A00655	A01470	488
A00655	A01474	352	A00655	A01492	532
A00655	A01506	258	A00655	A01553	226
A00655	A01558	547	A00655	A01588	797
A00655	A01598	350	A00655	A01612	219
A00655	A01628	377	A00655	A01649	221
A00655	A01995	441	A00655	A02050	188
A00655	A02869	414	A00655	A02980	414
A00655	A03014	270	A00655	A03399	393
A00655	A03473	273	A00655	A03734	479
A00655	A04790	185	A00655	A05357	193
A00655	A06543	230	A00655	A06545	170
A00655	A06735	414	A00655	A06803	154
A00655	A06907	364	A00655	A07004	477
A00655	A07244	240	A00655	A07290	235
A00655	A07311	481	A00655	A07339	234
A00655	A07487	243	A00655	A07706	236
A00655	A07835	480	A00655	A08022	424
A00655	A08118	490	A00655	A08137	474
A00655	A08193	254	A00655	A08357	399
A00655	A08571	490	A00655	A09004	452
A00655	A09007	318	A00655	A09279	204
A00655	A09327	299	A00655	A09471	160

A00655	A09568	314	A00655	A09644	152
A00655	A09707	489	A00655	A09712	252
A00655	A10070	188	A00655	A10642	263
A00655	A11324	313	A00655	A11487	455
A00655	A11820	417	A00655	A12660	257
A00655	A13889	257	A00655	A13928	329
A00655	A13989	474	A00655	A14006	362
A00655	A14220	472	A00655	A14566	240
A00655	A14931	486	A00655	A15256	427
A00655	A15291	154	A00655	A15492	191
A00655	A16228	227	A00655	A16994	182
A00656	A01157	172	A00656	A01526	216
A00656	A01527	899	A00656	A01556	313
A00656	A01741	308	A00656	A01877	193
A00656	A01947	168	A00656	A01982	394
A00656	A02021	205	A00656	A02036	467
A00656	A02037	257	A00656	A02048	491
A00656	A02049	491	A00656	A02087	283
A00656	A02088	165	A00656	A02146	395
A00656	A02156	491	A00656	A02180	152
A00656	A02186	374	A00656	A02190	271
A00656	A02215	489	A00656	A02226	235
A00656	A02238	185	A00656	A02309	188
A00656	A02390	240	A00656	A02505	267
A00656	A02776	191	A00656	A02859	305
A00656	A02951	177	A00656	A02977	168
A00656	A03074	396	A00656	A03080	490
A00656	A03095	477	A00656	A03195	163
A00656	A03218	198	A00656	A03312	257
A00656	A03353	154	A00656	A03356	185
A00656	A03363	245	A00656	A03367	408
A00656	A03370	490	A00656	A03378	356
A00656	A03379	462	A00656	A03390	355
A00656	A03392	294	A00656	A03396	446
A00656	A03397	243	A00656	A03401	463
A00656	A03402	419	A00656	A03403	364
A00656	A03408	329	A00656	A03541	195
A00656	A03729	396	A00656	A03733	210
A00656	A03744	469	A00656	A03921	208
A00656	A03989	430	A00656	A04051	475
A00656	A04089	254	A00656	A04278	420
A00656	A04293	241	A00656	A04407	254
A00656	A04433	254	A00656	A04488	274
A00656	A04608	479	A00656	A04614	171
A00656	A04639	180	A00656	A04775	271
A00656	A04830	335	A00656	A04922	196

A00656	A04923	329	A00656	A05136	294
A00656	A05377	322	A00656	A05427	286
A00656	A05460	318	A00656	A05731	229
A00656	A05901	288	A00656	A06088	251
A00656	A06387	247	A00656	A06838	271
A00656	A07187	167	A00656	A07218	490
A00656	A09131	276	A00656	A12674	294
A00656	A13661	267	A00656	A13683	288
A00656	A13734	154	A00656	A14444	326
A00656	A15809	183	A00656	A16595	396
A00657	A01400	485	A00657	A01551	419
A00657	A01618	183	A00657	A01637	191
A00657	A01651	176	A00657	A01668	469
A00657	A01675	201	A00657	A01705	434
A00657	A01725	458	A00657	A01728	268
A00657	A01729	316	A00657	A01731	245
A00657	A01732	490	A00657	A01737	177
A00657	A01739	241	A00657	A01743	491
A00657	A01744	384	A00657	A01745	469
A00657	A01751	399	A00657	A01754	263
A00657	A01757	260	A00657	A01766	184
A00657	A01798	193	A00657	A01872	310
A00657	A01873	357	A00657	A01883	363
A00657	A01893	305	A00657	A01909	200
A00657	A02300	167	A00657	A02305	221
A00657	A02335	159	A00657	A02338	257
A00657	A02362	254	A00657	A02687	191
A00657	A02691	488	A00657	A02700	299
A00657	A02744	174	A00657	A02810	299
A00657	A02811	263	A00657	A02857	202
A00657	A02858	408	A00657	A02893	347
A00657	A02906	301	A00657	A02977	241
A00657	A03268	317	A00657	A03585	303
A00657	A03845	288	A00657	A03849	382
A00657	A03964	288	A00657	A04005	328
A00657	A04105	177	A00657	A04122	288
A00657	A04139	371	A00657	A04709	252
A00657	A04797	382	A00657	A04960	185
A00657	A05390	258	A00657	A05911	411
A00657	A06298	224	A00657	A06635	308
A00657	A07200	443	A00657	A08617	341
A00657	A08741	235	A00657	A08806	207
A00657	A08844	257	A00657	A09275	363
A00657	A11400	196	A00657	A12122	441
A00657	A13632	357	A00657	A14320	372
A00657	A14389	328	A00657	A14405	333

A00657	A14440	182	A00657	A15795	205
A00657	A16636	171	A00658	A00687	273
A00658	A00715	418	A00658	A00757	374
A00658	A00871	578	A00658	A00878	412
A00658	A00911	332	A00658	A00986	577
A00658	A01052	176	A00658	A01065	259
A00658	A01111	223	A00658	A01118	451
A00658	A01126	385	A00658	A01143	259
A00658	A01150	168	A00658	A01202	223
A00658	A01208	574	A00658	A01211	162
A00658	A01214	445	A00658	A01215	578
A00658	A01228	435	A00658	A01234	481
A00658	A01287	218	A00658	A01311	724
A00658	A01353	578	A00658	A01406	490
A00658	A01558	157	A00658	A01647	401
A00658	A01707	394	A00658	A01736	225
A00658	A01790	210	A00658	A01995	252
A00658	A02041	446	A00658	A02051	339
A00658	A02092	191	A00658	A02180	283
A00658	A02270	219	A00658	A02274	272
A00658	A02385	180	A00658	A02452	424
A00658	A02454	236	A00658	A02565	200
A00658	A02695	601	A00658	A03169	542
A00658	A03279	172	A00658	A03568	179
A00658	A03569	268	A00658	A03601	380
A00658	A03792	363	A00658	A03895	219
A00658	A03952	396	A00658	A03958	473
A00658	A03972	272	A00658	A03982	272
A00658	A04447	471	A00658	A04477	213
A00658	A04833	389	A00658	A05058	312
A00658	A05216	718	A00658	A05411	196
A00658	A05467	167	A00658	A06907	193
A00658	A07265	393	A00658	A07447	312
A00658	A07588	499	A00658	A07644	172
A00658	A07706	230	A00658	A08925	157
A00658	A08942	353	A00658	A08955	223
A00658	A08971	223	A00658	A09281	177
A00658	A09641	281	A00658	A09756	174
A00658	A09977	154	A00658	A10006	174
A00658	A10755	223	A00658	A10871	219
A00658	A11202	223	A00658	A11254	216
A00658	A11277	152	A00658	A11451	263
A00658	A12661	170	A00658	A13132	312
A00658	A13298	327	A00658	A14006	243
A00658	A14196	288	A00658	A14216	538
A00658	A15875	191	A00658	A16465	154

A00658	A17063	157	A00659	A00662	165
A00659	A00688	256	A00659	A00794	434
A00659	A00813	443	A00659	A01005	438
A00659	A01046	430	A00659	A01073	157
A00659	A01235	63	A00659	A01396	208
A00659	A01438	462	A00659	A01461	389
A00659	A01515	184	A00659	A01573	424
A00659	A01575	163	A00659	A01583	212
A00659	A01585	467	A00659	A01726	221
A00659	A01737	489	A00659	A01741	377
A00659	A01750	159	A00659	A01845	221
A00659	A01846	460	A00659	A01847	407
A00659	A01848	414	A00659	A01856	252
A00659	A01864	168	A00659	A01867	152
A00659	A01878	185	A00659	A01926	491
A00659	A01941	212	A00659	A01990	236
A00659	A02008	491	A00659	A02009	435
A00659	A02011	491	A00659	A02012	491
A00659	A02013	324	A00659	A02014	490
A00659	A02015	456	A00659	A02028	93
A00659	A02031	396	A00659	A02034	339
A00659	A02093	229	A00659	A02114	491
A00659	A02147	218	A00659	A02152	221
A00659	A02262	198	A00659	A02268	345
A00659	A02275	433	A00659	A02287	198
A00659	A02336	200	A00659	A02371	324
A00659	A02377	213	A00659	A02405	238
A00659	A02454	263	A00659	A02468	249
A00659	A02574	490	A00659	A02606	185
A00659	A02611	324	A00659	A02635	201
A00659	A02649	334	A00659	A02695	456
A00659	A02802	466	A00659	A02806	489
A00659	A02816	165	A00659	A02859	420
A00659	A02992	416	A00659	A03016	156
A00659	A03034	491	A00659	A03035	191
A00659	A03036	491	A00659	A03043	157
A00659	A03045	335	A00659	A03058	347
A00659	A03091	408	A00659	A03099	367
A00659	A03107	268	A00659	A03144	491
A00659	A03146	491	A00659	A03147	382
A00659	A03148	486	A00659	A03156	491
A00659	A03157	283	A00659	A03196	265
A00659	A03224	357	A00659	A03238	443
A00659	A03247	185	A00659	A03251	347
A00659	A03254	278	A00659	A03259	302
A00659	A03275	162	A00659	A03278	172

A00659	A03279	188	A00659	A03305	152
A00659	A03513	472	A00659	A03638	205
A00659	A03950	200	A00659	A03957	347
A00659	A04010	310	A00659	A04051	252
A00659	A04076	227	A00659	A04212	472
A00659	A04229	224	A00659	A04286	151
A00659	A04318	358	A00659	A04341	324
A00659	A04349	327	A00659	A04400	286
A00659	A04450	241	A00659	A04487	286
A00659	A04506	296	A00659	A04615	183
A00659	A04839	157	A00659	A04881	159
A00659	A04886	286	A00659	A04956	249
A00659	A05145	241	A00659	A05203	368
A00659	A05330	297	A00659	A05376	412
A00659	A05393	180	A00659	A05437	278
A00659	A05459	245	A00659	A05463	174
A00659	A05467	185	A00659	A05506	210
A00659	A05570	286	A00659	A05597	165
A00659	A05682	177	A00659	A06053	378
A00659	A06195	491	A00659	A06414	188
A00659	A06642	163	A00659	A06643	286
A00659	A06650	241	A00659	A06798	210
A00659	A06827	198	A00659	A06831	245
A00659	A07006	278	A00659	A07059	321
A00659	A07130	288	A00659	A07168	382
A00659	A07175	191	A00659	A07184	188
A00659	A07191	362	A00659	A07342	491
A00659	A07373	458	A00659	A07407	379
A00659	A07477	395	A00659	A07659	205
A00659	A07736	484	A00659	A08093	274
A00659	A08250	218	A00659	A08667	185
A00659	A09036	213	A00659	A09110	229
A00659	A09267	296	A00659	A09277	301
A00659	A09411	229	A00659	A09991	485
A00659	A10091	204	A00659	A10214	152
A00659	A10226	364	A00659	A10816	356
A00659	A11076	347	A00659	A12927	291
A00659	A13826	339	A00659	A13832	227
A00659	A13871	252	A00659	A14146	165
A00659	A14247	357	A00659	A14344	490
A00659	A14419	296	A00659	A15308	162
A00659	A15346	215	A00659	A15366	176
A00659	A15411	299	A00659	A15462	358
A00659	A15498	213	A00659	A15518	345
A00659	A15874	179	A00659	A16008	218
A00659	A16037	296	A00659	A16063	347

A00659	A16337	324	A00659	A16636	258
A00660	A00681	294	A00660	A00696	334
A00660	A00825	458	A00660	A01018	484
A00660	A01176	157	A00660	A01225	324
A00660	A01297	467	A00660	A01397	472
A00660	A01409	475	A00660	A01463	311
A00660	A01466	324	A00660	A01490	434
A00660	A01511	252	A00660	A01635	333
A00660	A01726	281	A00660	A01832	229
A00660	A01972	176	A00660	A02020	323
A00660	A02054	388	A00660	A02114	445
A00660	A02115	366	A00660	A02254	491
A00660	A02257	457	A00660	A02284	480
A00660	A02330	263	A00660	A02363	281
A00660	A02532	273	A00660	A02719	202
A00660	A03054	204	A00660	A03087	484
A00660	A03099	213	A00660	A03164	159
A00660	A03174	465	A00660	A03484	210
A00660	A03498	246	A00660	A03513	356
A00660	A03520	379	A00660	A03968	267
A00660	A04781	279	A00660	A04791	221
A00660	A04927	183	A00660	A05007	179
A00660	A05102	198	A00660	A05208	374
A00660	A05224	408	A00660	A05277	273
A00660	A05330	234	A00660	A05331	481
A00660	A05337	227	A00660	A05338	465
A00660	A05416	274	A00660	A05418	362
A00660	A05424	490	A00660	A05462	238
A00660	A05463	241	A00660	A05550	201
A00660	A05573	180	A00660	A05610	414
A00660	A05614	345	A00660	A05988	490
A00660	A06086	195	A00660	A06405	296
A00660	A06439	154	A00660	A06473	185
A00660	A06629	167	A00660	A06632	311
A00660	A06668	299	A00660	A06700	165
A00660	A06769	279	A00660	A07007	168
A00660	A07027	230	A00660	A07059	430
A00660	A07080	182	A00660	A07088	477
A00660	A07520	350	A00660	A07674	441
A00660	A07684	362	A00660	A07690	238
A00660	A07711	390	A00660	A07763	226
A00660	A07771	303	A00660	A07779	384
A00660	A07847	446	A00660	A07851	467
A00660	A07933	377	A00660	A08083	269
A00660	A08114	223	A00660	A08135	429
A00660	A08190	449	A00660	A08197	258

A00660	A08279	399	A00660	A08280	305
A00660	A08372	323	A00660	A08385	351
A00660	A08707	269	A00660	A09846	372
A00660	A10422	391	A00660	A10446	236
A00660	A10456	389	A00660	A10679	433
A00660	A10687	294	A00660	A10691	417
A00660	A10698	428	A00660	A10700	311
A00660	A10715	375	A00660	A10781	266
A00660	A12723	301	A00660	A13155	337
A00660	A13362	401	A00660	A14803	185
A00660	A15356	333	A00660	A15628	311
A00660	A16288	348	A00661	A00696	283
A00661	A00825	176	A00661	A01022	468
A00661	A01161	157	A00661	A01174	187
A00661	A01225	213	A00661	A01397	213
A00661	A01409	288	A00661	A01462	469
A00661	A01635	358	A00661	A01878	271
A00661	A02075	180	A00661	A02187	274
A00661	A02215	201	A00661	A02329	358
A00661	A02359	357	A00661	A02450	270
A00661	A02495	449	A00661	A02669	283
A00661	A02854	205	A00661	A02928	428
A00661	A03028	326	A00661	A03234	296
A00661	A03304	257	A00661	A03819	330
A00661	A03968	291	A00661	A04325	375
A00661	A04458	366	A00661	A04589	172
A00661	A04710	185	A00661	A04910	366
A00661	A05330	479	A00661	A05331	465
A00661	A05659	217	A00661	A05963	393
A00661	A05964	466	A00661	A06405	185
A00661	A06934	293	A00661	A07007	190
A00661	A07408	307	A00661	A07690	258
A00661	A07847	356	A00661	A08199	277
A00661	A09880	474	A00661	A10687	311
A00661	A14371	205	A00661	A14386	283
A00661	A14498	351	A00661	A14589	337
A00661	A14665	489	A00662	A00663	491
A00662	A00681	399	A00662	A00738	423
A00662	A00794	489	A00662	A00882	259
A00662	A00962	486	A00662	A00981	212
A00662	A01005	491	A00662	A01008	430
A00662	A01018	321	A00662	A01035	491
A00662	A01138	281	A00662	A01229	154
A00662	A01233	324	A00662	A01256	294
A00662	A01297	165	A00662	A01397	151
A00662	A01409	430	A00662	A01461	485

A00662	A01560	305	A00662	A01575	489
A00662	A01583	435	A00662	A01585	475
A00662	A01590	409	A00662	A01669	433
A00662	A01705	436	A00662	A01713	491
A00662	A01726	486	A00662	A01741	191
A00662	A01743	405	A00662	A01926	477
A00662	A01928	452	A00662	A01934	276
A00662	A01947	172	A00662	A01972	454
A00662	A01978	486	A00662	A01995	212
A00662	A02012	191	A00662	A02022	283
A00662	A02077	205	A00662	A02168	472
A00662	A02215	491	A00662	A02319	217
A00662	A02402	364	A00662	A02413	168
A00662	A02521	156	A00662	A02530	160
A00662	A02538	409	A00662	A02541	430
A00662	A02542	91	A00662	A02545	371
A00662	A02546	297	A00662	A02574	234
A00662	A02575	180	A00662	A02601	484
A00662	A02785	265	A00662	A02859	484
A00662	A02992	491	A00662	A03027	208
A00662	A03034	310	A00662	A03050	491
A00662	A03167	302	A00662	A03238	180
A00662	A03402	299	A00662	A03487	243
A00662	A03513	485	A00662	A03523	184
A00662	A03545	475	A00662	A03618	273
A00662	A03858	170	A00662	A03892	432
A00662	A03990	583	A00662	A04023	190
A00662	A04214	229	A00662	A04369	486
A00662	A04372	191	A00662	A04674	207
A00662	A04839	491	A00662	A04883	377
A00662	A05009	236	A00662	A05146	168
A00662	A05153	483	A00662	A05356	491
A00662	A05390	417	A00662	A05393	162
A00662	A05407	157	A00662	A05416	288
A00662	A05487	473	A00662	A05573	230
A00662	A05775	406	A00662	A05776	481
A00662	A05858	243	A00662	A05926	480
A00662	A05945	347	A00662	A06009	218
A00662	A06013	356	A00662	A06019	191
A00662	A06086	255	A00662	A06211	151
A00662	A06258	802	A00662	A06379	420
A00662	A06397	185	A00662	A06527	484
A00662	A06558	229	A00662	A06607	480
A00662	A06659	400	A00662	A06709	229
A00662	A06875	307	A00662	A06908	208
A00662	A06979	205	A00662	A07122	379

A00662	A07292	168	A00662	A07300	440
A00662	A07311	180	A00662	A07330	185
A00662	A07339	420	A00662	A07389	199
A00662	A07407	480	A00662	A07413	205
A00662	A07452	489	A00662	A07468	327
A00662	A07471	413	A00662	A07472	347
A00662	A07475	473	A00662	A07477	472
A00662	A07491	335	A00662	A07530	240
A00662	A07541	491	A00662	A07542	491
A00662	A07543	491	A00662	A07544	401
A00662	A07561	483	A00662	A07563	217
A00662	A07590	215	A00662	A07610	480
A00662	A07630	373	A00662	A07631	217
A00662	A07633	185	A00662	A07634	491
A00662	A07635	416	A00662	A07688	152
A00662	A07736	491	A00662	A07754	391
A00662	A07800	210	A00662	A07847	171
A00662	A07912	179	A00662	A07958	481
A00662	A07973	417	A00662	A08063	229
A00662	A08404	486	A00662	A08693	260
A00662	A08785	481	A00662	A08798	263
A00662	A09837	160	A00662	A09932	360
A00662	A10056	419	A00662	A10655	319
A00662	A10967	490	A00662	A11370	263
A00662	A11450	168	A00662	A12793	465
A00662	A13327	185	A00662	A13602	296
A00662	A13871	491	A00662	A13879	224
A00662	A14247	470	A00662	A14444	235
A00662	A14685	199	A00662	A15293	380
A00662	A15462	318	A00662	A15506	778
A00662	A15608	477	A00662	A15992	210
A00662	A16877	204	A00662	A17078	491
A00662	A17108	372	A00663	A00666	232
A00663	A00688	63	A00663	A00738	183
A00663	A00962	480	A00663	A01005	491
A00663	A01018	152	A00663	A01035	491
A00663	A01161	491	A00663	A01166	207
A00663	A01233	156	A00663	A01256	477
A00663	A01409	196	A00663	A01461	475
A00663	A01493	180	A00663	A01556	363
A00663	A01585	334	A00663	A01705	366
A00663	A01713	491	A00663	A01722	375
A00663	A01726	170	A00663	A01741	191
A00663	A01743	385	A00663	A01926	483
A00663	A01928	322	A00663	A01947	208
A00663	A01972	430	A00663	A01978	489

A00663	A02022	177	A00663	A02075	241
A00663	A02078	172	A00663	A02092	160
A00663	A02107	174	A00663	A02156	165
A00663	A02168	468	A00663	A02170	224
A00663	A02215	491	A00663	A02317	361
A00663	A02402	321	A00663	A02530	188
A00663	A02538	360	A00663	A02542	121
A00663	A02545	154	A00663	A02601	391
A00663	A02785	260	A00663	A02859	477
A00663	A02992	491	A00663	A03027	245
A00663	A03050	491	A00663	A03056	187
A00663	A03080	356	A00663	A03099	218
A00663	A03167	191	A00663	A03168	196
A00663	A03238	226	A00663	A03293	159
A00663	A03370	371	A00663	A03402	424
A00663	A03523	154	A00663	A03545	245
A00663	A03858	302	A00663	A03917	279
A00663	A04369	462	A00663	A04633	412
A00663	A04839	491	A00663	A04883	171
A00663	A04975	185	A00663	A05153	428
A00663	A05237	255	A00663	A05356	489
A00663	A05389	167	A00663	A05390	433
A00663	A05393	360	A00663	A05407	185
A00663	A05487	452	A00663	A05687	269
A00663	A05775	316	A00663	A05776	469
A00663	A05801	234	A00663	A05858	269
A00663	A05926	317	A00663	A06009	252
A00663	A06092	221	A00663	A06148	162
A00663	A06527	486	A00663	A06607	470
A00663	A06874	180	A00663	A06908	341
A00663	A07088	170	A00663	A07122	274
A00663	A07134	269	A00663	A07209	337
A00663	A07218	319	A00663	A07300	449
A00663	A07330	213	A00663	A07407	454
A00663	A07419	196	A00663	A07452	483
A00663	A07471	251	A00663	A07472	201
A00663	A07475	174	A00663	A07477	165
A00663	A07541	491	A00663	A07542	486
A00663	A07543	480	A00663	A07544	347
A00663	A07561	462	A00663	A07605	172
A00663	A07610	447	A00663	A07630	177
A00663	A07632	168	A00663	A07633	213
A00663	A07634	488	A00663	A07635	330
A00663	A07721	249	A00663	A07736	475
A00663	A07800	472	A00663	A07890	316
A00663	A07958	485	A00663	A07970	234

A00663	A07973	172	A00663	A08079	174
A00663	A08089	160	A00663	A08210	163
A00663	A08404	338	A00663	A08693	299
A00663	A08785	246	A00663	A09174	154
A00663	A09932	224	A00663	A10052	380
A00663	A10056	469	A00663	A10967	480
A00663	A11309	216	A00663	A11329	172
A00663	A11370	286	A00663	A12793	350
A00663	A13327	213	A00663	A13602	316
A00663	A13871	491	A00663	A13992	223
A00663	A14247	477	A00663	A15992	152
A00663	A16118	363	A00663	A17078	491
A00664	A00846	474	A00664	A00855	317
A00664	A01074	724	A00664	A01105	317
A00664	A01137	268	A00664	A01549	228
A00664	A01622	317	A00664	A01679	317
A00664	A01699	180	A00664	A01716	177
A00664	A01779	188	A00664	A01899	281
A00664	A01926	63	A00664	A01978	355
A00664	A02168	369	A00664	A02170	184
A00664	A02513	221	A00664	A02524	156
A00664	A02530	267	A00664	A02538	406
A00664	A02539	485	A00664	A02541	213
A00664	A02542	229	A00664	A02572	202
A00664	A02616	221	A00664	A02761	151
A00664	A02832	193	A00664	A03086	217
A00664	A03155	183	A00664	A03710	317
A00664	A04487	185	A00664	A05060	227
A00664	A05150	196	A00664	A05154	165
A00664	A05157	201	A00664	A05208	219
A00664	A05222	151	A00664	A05313	160
A00664	A05315	259	A00664	A05332	317
A00664	A05407	263	A00664	A05435	457
A00664	A05609	317	A00664	A05691	195
A00664	A05775	457	A00664	A06454	491
A00664	A06471	278	A00664	A06650	217
A00664	A07222	317	A00664	A07333	317
A00664	A07571	317	A00664	A07655	340
A00664	A07796	243	A00664	A07974	317
A00664	A08097	317	A00664	A08688	210
A00664	A09340	317	A00664	A10109	260
A00664	A10394	332	A00664	A10483	366
A00664	A10693	188	A00664	A11841	217
A00664	A13195	283	A00664	A13614	488
A00664	A14195	228	A00664	A14986	483
A00664	A15097	213	A00664	A16219	184

A00665	A00680	180	A00665	A00733	291
A00665	A00744	714	A00665	A00766	163
A00665	A00805	329	A00665	A00858	475
A00665	A00860	449	A00665	A00881	330
A00665	A00961	179	A00665	A00978	249
A00665	A01007	232	A00665	A01019	440
A00665	A01074	165	A00665	A01195	305
A00665	A01200	202	A00665	A01212	333
A00665	A01325	486	A00665	A01335	380
A00665	A01336	218	A00665	A01401	319
A00665	A01442	168	A00665	A01447	458
A00665	A01564	277	A00665	A01571	484
A00665	A01720	385	A00665	A02389	552
A00665	A02872	296	A00665	A03014	484
A00665	A03207	314	A00665	A03759	191
A00665	A04100	296	A00665	A04648	210
A00665	A05027	180	A00665	A05981	713
A00665	A06803	216	A00665	A07347	335
A00665	A07670	183	A00665	A07695	229
A00665	A07708	171	A00665	A07887	307
A00665	A09432	326	A00665	A09712	286
A00665	A09806	473	A00665	A11735	385
A00665	A11737	170	A00665	A13788	484
A00665	A15666	477	A00665	A16228	268
A00666	A00676	162	A00666	A00681	491
A00666	A00695	458	A00666	A00738	470
A00666	A00739	306	A00666	A00770	491
A00666	A00771	228	A00666	A00773	358
A00666	A00962	491	A00666	A00968	358
A00666	A00995	489	A00666	A01005	173
A00666	A01008	491	A00666	A01011	358
A00666	A01018	268	A00666	A01063	490
A00666	A01115	224	A00666	A01165	245
A00666	A01174	454	A00666	A01175	384
A00666	A01189	344	A00666	A01222	377
A00666	A01225	210	A00666	A01233	491
A00666	A01256	479	A00666	A01272	467
A00666	A01277	213	A00666	A01297	212
A00666	A01347	294	A00666	A01360	466
A00666	A01397	210	A00666	A01409	163
A00666	A01481	238	A00666	A01483	347
A00666	A01508	491	A00666	A01511	154
A00666	A01548	489	A00666	A01569	481
A00666	A01630	162	A00666	A01635	491
A00666	A01725	160	A00666	A01741	373
A00666	A01747	419	A00666	A01841	374

A00666	A01855	473	A00666	A01857	374
A00666	A01859	221	A00666	A01870	238
A00666	A01895	434	A00666	A02020	174
A00666	A02167	162	A00666	A02240	401
A00666	A02254	193	A00666	A02290	168
A00666	A02353	480	A00666	A02363	187
A00666	A02541	371	A00666	A02718	477
A00666	A02719	224	A00666	A02826	193
A00666	A02834	271	A00666	A02940	441
A00666	A02992	335	A00666	A03002	173
A00666	A03005	210	A00666	A03008	301
A00666	A03059	187	A00666	A03167	463
A00666	A03168	323	A00666	A03184	167
A00666	A03259	154	A00666	A03311	408
A00666	A03430	251	A00666	A03431	154
A00666	A03441	361	A00666	A03468	274
A00666	A03477	491	A00666	A03484	335
A00666	A03501	409	A00666	A03586	441
A00666	A03861	457	A00666	A03863	441
A00666	A04095	314	A00666	A04175	187
A00666	A04211	165	A00666	A04274	224
A00666	A04372	235	A00666	A04465	235
A00666	A04517	152	A00666	A04602	329
A00666	A04660	420	A00666	A04674	372
A00666	A04779	198	A00666	A04784	352
A00666	A04791	346	A00666	A04834	368
A00666	A04850	490	A00666	A04880	224
A00666	A04937	390	A00666	A04995	340
A00666	A05006	451	A00666	A05007	481
A00666	A05020	434	A00666	A05026	330
A00666	A05050	196	A00666	A05051	283
A00666	A05088	213	A00666	A05102	490
A00666	A05155	446	A00666	A05310	483
A00666	A05330	263	A00666	A05348	157
A00666	A05416	408	A00666	A05472	417
A00666	A05515	291	A00666	A05516	469
A00666	A05600	385	A00666	A05659	385
A00666	A05673	313	A00666	A05679	241
A00666	A05699	224	A00666	A05757	238
A00666	A05759	353	A00666	A05768	269
A00666	A05771	235	A00666	A05772	441
A00666	A05774	436	A00666	A05789	196
A00666	A05937	184	A00666	A06024	441
A00666	A06088	193	A00666	A06089	251
A00666	A06091	401	A00666	A06098	243
A00666	A06165	251	A00666	A06252	167

A00666	A06344	167	A00666	A06358	488
A00666	A06359	486	A00666	A06360	416
A00666	A06389	182	A00666	A06415	274
A00666	A06517	260	A00666	A06555	283
A00666	A06592	385	A00666	A06630	364
A00666	A06633	445	A00666	A06635	243
A00666	A06641	265	A00666	A06692	407
A00666	A06700	278	A00666	A06756	386
A00666	A06773	171	A00666	A06819	432
A00666	A06934	313	A00666	A06970	152
A00666	A07002	361	A00666	A07080	417
A00666	A07109	274	A00666	A07122	491
A00666	A07134	283	A00666	A07185	465
A00666	A07222	212	A00666	A07238	330
A00666	A07239	313	A00666	A07246	329
A00666	A07267	335	A00666	A07288	340
A00666	A07341	187	A00666	A07413	477
A00666	A07477	293	A00666	A07530	293
A00666	A07554	196	A00666	A07558	172
A00666	A07627	251	A00666	A07688	384
A00666	A07739	235	A00666	A07746	434
A00666	A07757	241	A00666	A07780	270
A00666	A07800	481	A00666	A07931	190
A00666	A07973	196	A00666	A08099	257
A00666	A08223	455	A00666	A08265	441
A00666	A08616	329	A00666	A08751	405
A00666	A08817	185	A00666	A08847	191
A00666	A08978	400	A00666	A09024	204
A00666	A09169	157	A00666	A09381	151
A00666	A09632	424	A00666	A10027	329
A00666	A10240	235	A00666	A10370	372
A00666	A10420	177	A00666	A10444	252
A00666	A10456	156	A00666	A10488	378
A00666	A10550	319	A00666	A10614	167
A00666	A10659	213	A00666	A10738	301
A00666	A11304	252	A00666	A11402	435
A00666	A13033	283	A00666	A13478	329
A00666	A13746	329	A00666	A13821	193
A00666	A14125	185	A00666	A14313	243
A00666	A14337	352	A00666	A14366	249
A00666	A14372	157	A00666	A14419	270
A00666	A14525	419	A00666	A14613	218
A00666	A14827	485	A00666	A14858	260
A00666	A15079	329	A00666	A15372	418
A00666	A15637	485	A00666	A15874	477
A00666	A15905	489	A00666	A16026	475

A00666	A16027	490	A00666	A16028	474
A00666	A16283	438	A00666	A16284	398
A00666	A16328	184	A00666	A16346	394
A00666	A16529	185	A00666	A16741	445
A00666	A17078	286	A00667	A00706	154
A00667	A00709	480	A00667	A00710	395
A00667	A00740	376	A00667	A00762	223
A00667	A00774	321	A00667	A00789	188
A00667	A00824	508	A00667	A00843	363
A00667	A00848	356	A00667	A00913	216
A00667	A00965	221	A00667	A00969	405
A00667	A00970	310	A00667	A00982	205
A00667	A00996	320	A00667	A01016	254
A00667	A01017	154	A00667	A01102	356
A00667	A01115	486	A00667	A01119	583
A00667	A01136	241	A00667	A01190	301
A00667	A01192	165	A00667	A01194	592
A00667	A01218	447	A00667	A01411	589
A00667	A01420	299	A00667	A01455	512
A00667	A01539	467	A00667	A01569	430
A00667	A01581	247	A00667	A01608	296
A00667	A01824	205	A00667	A01826	247
A00667	A02705	163	A00667	A03051	151
A00667	A03180	312	A00667	A03446	401
A00667	A03453	247	A00667	A03601	228
A00667	A03785	202	A00667	A03885	204
A00667	A04932	163	A00667	A06531	303
A00667	A07196	307	A00667	A08447	191
A00667	A08550	188	A00667	A08896	273
A00667	A09073	193	A00667	A09587	230
A00667	A09717	374	A00667	A10490	439
A00667	A11372	260	A00667	A11409	491
A00667	A11614	200	A00667	A11704	170
A00667	A12433	208	A00667	A12508	177
A00667	A12607	328	A00667	A12665	154
A00667	A12825	213	A00667	A13310	190
A00667	A13609	279	A00667	A13788	243
A00667	A14074	247	A00667	A14358	322
A00667	A14375	162	A00667	A14462	165
A00667	A14622	174	A00667	A15660	157
A00667	A15804	279	A00667	A16187	200
A00667	A16597	160	A00667	A16664	157
A00667	A16682	252	A00667	A16807	184
A00667	A16824	294	A00668	A00864	489
A00668	A01908	170	A00668	A02839	221
A00668	A03570	227	A00668	A04282	404

A00668	A06715	223	A00668	A07073	219
A00668	A08196	367	A00668	A09263	224
A00668	A09624	196	A00668	A11475	247
A00668	A11675	246	A00668	A14084	160
A00668	A14266	286	A00669	A00719	184
A00669	A00838	243	A00669	A00875	534
A00669	A00887	489	A00669	A00997	491
A00669	A01052	491	A00669	A01172	486
A00669	A01524	475	A00669	A01527	944
A00669	A01548	469	A00669	A01558	899
A00669	A01560	942	A00669	A01561	899
A00669	A01572	324	A00669	A01588	901
A00669	A01591	157	A00669	A01592	177
A00669	A01602	899	A00669	A01615	899
A00669	A01645	172	A00669	A01650	188
A00669	A01688	489	A00669	A01694	391
A00669	A01839	491	A00669	A02178	151
A00669	A02436	369	A00669	A02452	446
A00669	A02517	252	A00669	A02586	202
A00669	A02640	461	A00669	A02749	446
A00669	A02752	205	A00669	A02792	245
A00669	A03070	185	A00669	A03226	160
A00669	A03435	462	A00669	A03451	369
A00669	A03477	286	A00669	A03479	179
A00669	A03611	490	A00669	A03741	156
A00669	A03854	491	A00669	A03878	205
A00669	A03949	232	A00669	A03990	454
A00669	A04062	485	A00669	A04442	202
A00669	A04468	201	A00669	A04494	310
A00669	A04790	407	A00669	A04807	396
A00669	A04879	265	A00669	A05082	491
A00669	A05120	163	A00669	A05415	430
A00669	A05701	491	A00669	A05797	252
A00669	A06118	173	A00669	A06268	408
A00669	A06379	450	A00669	A06597	319
A00669	A06610	455	A00669	A06660	424
A00669	A06897	363	A00669	A07011	332
A00669	A07091	319	A00669	A07158	185
A00669	A07206	167	A00669	A07311	924
A00669	A07375	443	A00669	A07834	235
A00669	A08571	241	A00669	A08851	198
A00669	A09092	160	A00669	A09124	491
A00669	A09566	179	A00669	A09727	252
A00669	A09826	168	A00669	A10006	152
A00669	A10111	228	A00669	A10189	294
A00669	A10386	187	A00669	A10551	305

A00669	A11348	457	A00669	A11369	216
A00669	A12383	299	A00669	A12886	152
A00669	A13824	899	A00669	A14188	899
A00669	A14198	915	A00669	A14577	286
A00669	A15071	323	A00669	A15545	398
A00669	A15893	157	A00669	A16719	216
A00669	A17064	263	A00670	A00850	179
A00670	A00854	151	A00670	A00883	490
A00670	A00943	460	A00670	A00968	577
A00670	A01074	475	A00670	A01091	480
A00670	A01105	210	A00670	A01189	568
A00670	A01205	457	A00670	A01243	368
A00670	A01269	302	A00670	A01360	486
A00670	A01385	232	A00670	A01483	422
A00670	A01508	486	A00670	A01622	391
A00670	A02212	610	A00670	A02335	223
A00670	A02601	165	A00670	A02789	223
A00670	A03242	249	A00670	A03501	404
A00670	A03567	347	A00670	A03597	240
A00670	A03719	223	A00670	A03728	223
A00670	A03752	223	A00670	A04734	184
A00670	A05332	390	A00670	A06290	252
A00670	A06689	223	A00670	A06819	577
A00670	A07107	226	A00670	A07222	247
A00670	A07232	180	A00670	A07333	709
A00670	A07413	160	A00670	A07756	450
A00670	A07931	177	A00670	A07956	414
A00670	A07974	249	A00670	A08289	198
A00670	A08517	414	A00670	A08960	184
A00670	A09276	391	A00670	A09317	201
A00670	A09627	414	A00670	A12924	485
A00670	A13822	343	A00670	A14196	488
A00670	A14525	562	A00670	A14803	294
A00670	A14804	223	A00670	A14858	252
A00670	A15323	475	A00670	A15324	486
A00670	A15339	281	A00670	A15353	281
A00670	A15372	354	A00670	A15564	335
A00670	A16219	223	A00670	A16463	438
A00670	A16531	524	A00670	A16753	201
A00670	A16761	216	A00670	A16769	441
A00671	A00679	452	A00671	A00703	234
A00671	A00747	474	A00671	A00759	240
A00671	A00791	288	A00671	A00856	263
A00671	A00942	302	A00671	A01068	283
A00671	A01124	196	A00671	A01148	378
A00671	A01202	263	A00671	A01291	268

A00671	A01374	353	A00671	A01405	187
A00671	A01418	224	A00671	A01474	193
A00671	A01507	440	A00671	A01602	491
A00671	A01633	303	A00671	A01665	157
A00671	A01724	353	A00671	A01790	260
A00671	A01917	240	A00671	A01931	308
A00671	A02121	218	A00671	A02253	353
A00671	A02344	263	A00671	A02902	486
A00671	A03021	218	A00671	A03104	171
A00671	A03213	491	A00671	A03273	104
A00671	A03304	458	A00671	A03465	475
A00671	A03663	212	A00671	A03738	266
A00671	A03843	262	A00671	A03954	270
A00671	A04020	245	A00671	A04033	201
A00671	A04449	172	A00671	A04638	243
A00671	A04687	263	A00671	A05017	226
A00671	A05112	238	A00671	A05158	389
A00671	A05418	335	A00671	A05454	321
A00671	A06011	226	A00671	A06200	411
A00671	A06813	151	A00671	A07203	234
A00671	A07209	290	A00671	A07572	223
A00671	A07677	262	A00671	A07695	293
A00671	A07803	232	A00671	A08022	313
A00671	A08538	384	A00671	A08621	151
A00671	A08734	255	A00671	A08836	130
A00671	A08860	210	A00671	A09042	389
A00671	A09072	281	A00671	A09171	286
A00671	A09320	266	A00671	A09487	483
A00671	A09566	302	A00671	A09624	63
A00671	A09789	367	A00671	A09874	154
A00671	A10159	223	A00671	A10255	384
A00671	A10440	163	A00671	A11403	218
A00671	A11543	154	A00671	A11983	262
A00671	A12246	188	A00671	A12513	269
A00671	A12897	279	A00671	A14211	191
A00671	A14410	154	A00671	A14635	191
A00671	A14643	296	A00671	A14660	171
A00671	A14711	165	A00671	A14928	290
A00671	A16304	177	A00671	A16610	154
A00672	A00996	229	A00672	A14247	63
A00672	A14504	63	A00673	A00687	363
A00673	A00705	344	A00673	A00709	188
A00673	A00711	234	A00673	A00727	252
A00673	A00740	257	A00673	A00751	235
A00673	A00839	188	A00673	A00953	188
A00673	A00972	188	A00673	A00988	241

A00673	A01017	188	A00673	A01101	315
A00673	A01207	146	A00673	A01217	275
A00673	A01218	430	A00673	A01220	188
A00673	A01362	188	A00673	A01369	226
A00673	A01406	188	A00673	A01408	188
A00673	A01411	303	A00673	A01414	188
A00673	A01451	188	A00673	A01579	226
A00673	A01841	201	A00673	A01976	249
A00673	A01988	188	A00673	A03453	202
A00673	A03901	313	A00673	A04687	329
A00673	A05127	185	A00673	A05702	188
A00673	A06785	188	A00673	A07746	162
A00673	A07965	188	A00673	A09587	296
A00673	A11480	188	A00673	A14566	257
A00673	A14594	307	A00673	A14813	188
A00673	A15657	188	A00673	A16680	188
A00674	A00697	396	A00674	A00709	272
A00674	A00714	160	A00674	A00735	173
A00674	A00736	369	A00674	A00751	175
A00674	A00759	394	A00674	A00761	486
A00674	A00766	539	A00674	A00781	486
A00674	A00791	543	A00674	A00805	279
A00674	A00816	351	A00674	A00868	544
A00674	A00883	544	A00674	A00884	407
A00674	A00926	544	A00674	A00927	272
A00674	A00975	544	A00674	A01014	335
A00674	A01039	160	A00674	A01058	160
A00674	A01077	511	A00674	A01080	160
A00674	A01106	255	A00674	A01122	461
A00674	A01169	484	A00674	A01197	168
A00674	A01212	321	A00674	A01267	160
A00674	A01278	160	A00674	A01329	238
A00674	A01339	518	A00674	A01340	217
A00674	A01370	419	A00674	A01826	223
A00674	A01985	177	A00674	A02126	265
A00674	A02212	286	A00674	A02254	249
A00674	A03043	168	A00674	A03061	288
A00674	A03271	198	A00674	A03498	324
A00674	A03676	429	A00674	A03885	152
A00674	A03902	191	A00674	A04579	204
A00674	A04793	207	A00674	A05377	173
A00674	A05383	170	A00674	A05570	299
A00674	A06032	170	A00674	A06750	452
A00674	A06773	213	A00674	A06938	380
A00674	A07096	160	A00674	A07226	212
A00674	A07266	312	A00674	A07389	281

A00674	A07403	385	A00674	A07756	468
A00674	A07822	271	A00674	A07986	276
A00674	A08834	160	A00674	A08882	451
A00674	A09627	160	A00674	A09740	212
A00674	A10372	338	A00674	A11365	160
A00674	A11724	196	A00674	A11735	160
A00674	A11737	160	A00674	A11914	160
A00674	A12658	160	A00674	A12824	545
A00674	A12887	270	A00674	A14007	160
A00674	A14138	162	A00674	A15257	160
A00674	A15498	491	A00674	A15624	486
A00675	A00882	491	A00675	A01332	468
A00675	A01461	393	A00675	A01575	343
A00675	A01723	187	A00675	A01916	241
A00675	A01983	255	A00675	A03104	196
A00675	A03753	463	A00675	A04337	191
A00675	A04848	177	A00675	A05356	356
A00675	A05601	446	A00675	A06611	291
A00675	A07754	163	A00675	A08093	204
A00675	A09469	428	A00675	A09742	322
A00675	A11279	256	A00675	A11552	265
A00675	A12540	351	A00675	A13313	165
A00675	A13369	399	A00675	A13945	474
A00675	A13987	205	A00675	A14052	228
A00675	A14202	353	A00675	A14247	480
A00675	A14381	297	A00675	A14570	445
A00675	A14661	151	A00675	A15366	293
A00675	A15922	171	A00675	A16132	386
A00676	A00914	199	A00676	A01364	294
A00676	A01402	302	A00676	A01556	171
A00676	A01841	357	A00676	A01859	290
A00676	A02011	241	A00676	A02192	319
A00676	A02353	462	A00676	A02481	165
A00676	A02506	265	A00676	A02718	488
A00676	A02951	205	A00676	A02984	230
A00676	A03126	278	A00676	A03832	157
[truncated: 480,281 more chars]
